# Transcriptome analyses reveal the synergistic effects of feeding and eyestalk ablation on ovarian maturation in black tiger shrimp

**DOI:** 10.1038/s41598-020-60192-2

**Published:** 2020-02-24

**Authors:** Kanchana Sittikankaew, Wirulda Pootakham, Chutima Sonthirod, Duangjai Sangsrakru, Thippawan Yoocha, Jutatip Khudet, Intawat Nookaew, Umaporn Uawisetwathana, Wanilada Rungrassamee, Nitsara Karoonuthaisiri

**Affiliations:** 1 National Center for Genetic Engineering and Biotechnology (BIOTEC), National Science and Technology Development Agency, Pathum Thani, 12120 Thailand; 2 National Omics Center, National Center for Genetic Engineering and Biotechnology (BIOTEC), National Science and Technology Development Agency, Pathum Thani, Thailand; 3 Department Biomedical Informatics, University of Arkansas for Medical Sciences, Little Rock, AR 72205 USA

**Keywords:** Gene regulatory networks, Transcriptomics

## Abstract

Unilateral eyestalk ablation in the female black tiger shrimp *Penaeus monodon* is commonly employed to induce ovarian maturation. However, the importance of complementing this practice with the provision of live feed supplement (such as polychaetes) has not been emphasized in previous studies. Indeed, it has been less emphasized that female broodstock must be fed with live feeds such as polychaetes for this practice to be effective. While the effects of eyestalk ablation have been thoroughly studied in various aspects, the synergistic effects of feeding with live feeds and the ablation have never been elucidated at a transcriptome-wide level. With recent advances in the next-generation sequencing platforms, it is now possible to investigate the effects of eyestalk ablation and live feeds at the transcriptomic levels. This study employed both short-read Illumina RNA sequencing and long-read Pacific Biosciences (PacBio) isoform sequencing (Iso-seq) to generate the first high-quality ovarian reference transcriptome in *P*. *monodon*. This novel assembly allowed us to dissect the effects of feeds and eyestalk ablation and reveal their synergistic effects at the transcriptomic level through the regulation of important genes involved in fatty acid regulation, energy production, and hormone-mediated oocyte maturation pathways. The synergistic effects between the polychaete feeding and the eyestalk ablation in the process of ovarian maturation in black tiger shrimp suggest that without having proper nutrients from the polychaetes, female broodstock might not be ready to develop its ovary. However, even with proper nutrients, the eyestalk ablation is still necessary to perhaps manipulate the female endocrine of the black tiger shrimp. These findings shed the light on molecular mechanisms and key molecular pathways that lead to successful ovarian maturation.

## Introduction

The black tiger shrimp (*Penaeus monodon*) is the second most widely cultured shrimp species in the world (FAO 2016). Albeit high demand worldwide, the shrimp production level remains stagnant mainly due to the outbreak of diseases and lack of selective breeding programs. The future success of the shrimp industry will depend upon increasing supplies of healthy, high-quality seed for stocking ponds and decreasing reliance on wild broodstock, which might potentially be a disease carrier and have seasonal variation^1^. To overcome these challenges, it is vital to be able to enhance reproductive maturation of captive broodstock.

Ovarian maturation is a well-orchestrated and complex molecular process that requires the regulation of large sets of genes to ensure proper oocyte development for embryogenesis. In penaeid shrimp, the process can be categorized into four stages: (I) previtellogenesis, (II) vitellogenesis, (III) early cortical rod, and (IV) late cortical rod^2^. Numerous factors and methods have been investigated to improve reproductive maturation in female black tiger shrimp. Dietary choices have been shown to be important for the maturation. Although nutritional requirements for shrimp reproduction are poorly defined, live feeds such as polychaetes, squid, and mussels are generally used to prepare broodstock before mating because they are nutritionally rich in proteins, lipids, polyunsaturated fatty acids (PUFA)^3,4^, and sex steroid hormones, such as progesterone and its derivatives^5^. Dietary PUFAs have been reported to benefit ovarian development in several crustacean species, such as the Pacific white shrimp *Litopenaeus vannamei*^6^, the Chinese mitten crab *Eriocheir sinensis*^7^, the mud crab *Scylla serrata*^8^ and *P*. *monodon*^9^. However, in *P*. *monodon*, the maturation diet alone could not induce female maturation to the desirable degree, and unilateral eyestalk ablation is still necessary^10^.

Eyestalk ablation is generally practiced to induce ovarian maturation in shrimp farming^11,12^. This technique has been reported to enhance the ovarian maturation by eliminating gonad-inhibiting hormone (GIH)/vitellogenesis-inhibiting hormone (VIH) secreted from the X-organ sinus gland complex within eyestalks^13^. From this knowledge, GIH-specific double-stranded RNA was used experimentally to silence GIH expression, which in turn increased the expression level of vitellogenin gene, ovarian maturation and spawning in both domesticated and wild female broodstock *P*. *monodon* shrimp^14^. However, the brooders used in the study were also fed with polychaetes and squids. Besides the GIH removal hypothesis, eyestalk removal has been linked to reduced light intensity and thereby inducing ovarian maturation in the banana prawn *P*. *merguiensis*^15^. The exact mechanism by which the eyestalk ablation induces ovarian maturation remains inconclusive.

Previously, we employed cDNA microarray to investigate the molecular and physiological effects of unilateral eyestalk ablation leading to ovarian maturation in polychaete-fed female *P*. *monodon* and discovered the up-regulation of genes in several crucial pathways, namely the gonadotropin-releasing hormone (GnRH) signaling pathway, calcium signaling pathway, and progesterone-mediated oocyte maturation pathway^16^. However, it was not clear whether the reported changes in transcript accumulation of genes in those pathways were affected by the eyestalk ablation, the polychaete diet, or both. Moreover, with the limitation of cDNA microarray technique, transcripts excluded from the probes on the microarray were not examined.

In this study, we employed the next-generation sequencing to dissect the effects of eyestalk ablation and feeds on gene expression in domesticated *P*. *monodon* female broodstock by comparing changes in transcript accumulation between two groups of broodstock fed with either pellet or polychaetes before and after eyestalk ablation.

## Results

### Effects of feeds and eyestalk ablation on ovarian maturation

**Figure 1:**
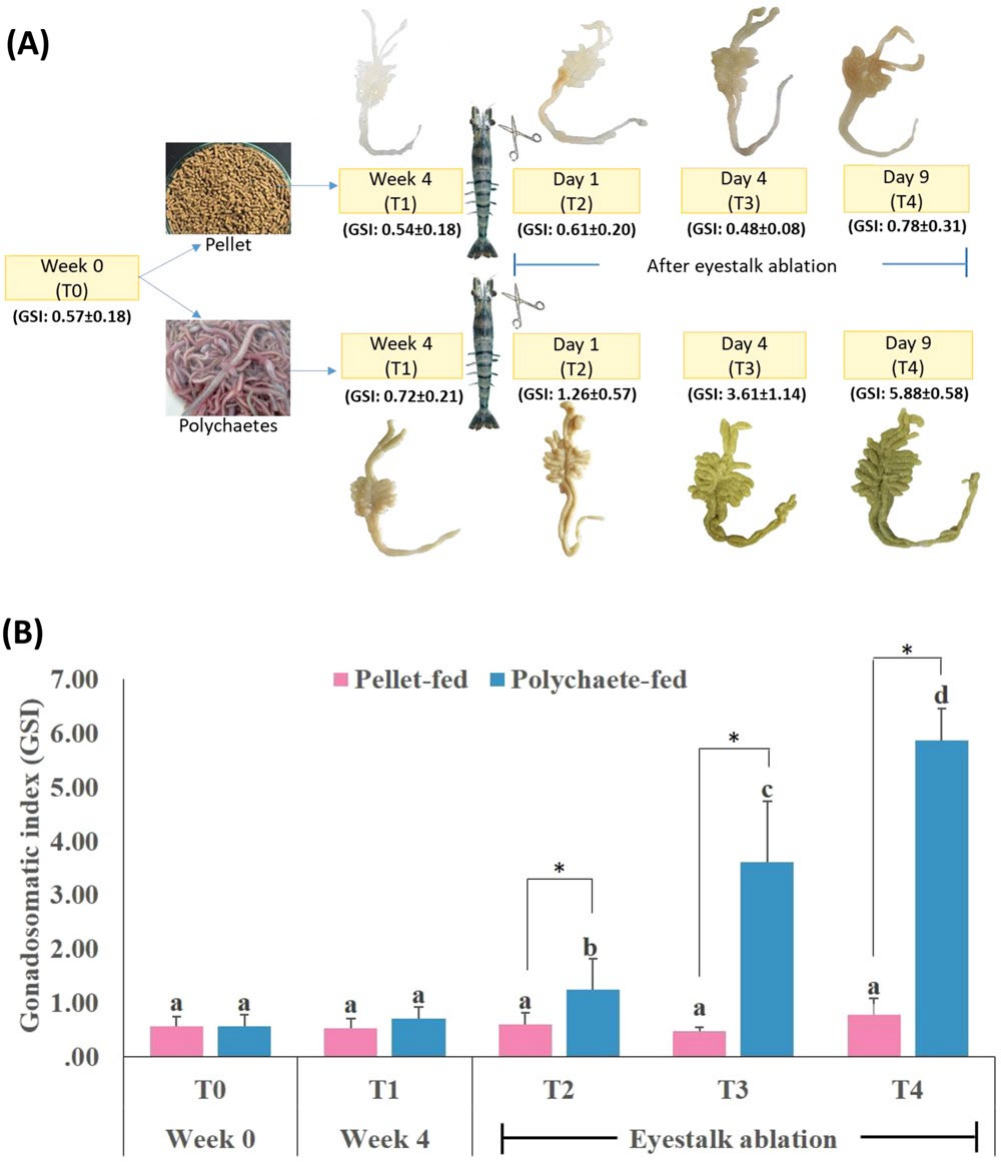
Ovarian maturation after unilateral eyestalk ablation in the shrimp fed with pellet or polychaetes. (**A**) Representatives of ovaries from different time points. (**B**) Gonadosomatic Index (GSI) of the shrimp at different time points. Error bars represent standard derivation. Different letters indicate significant differences among time points of the same feed group (p < 0.05, ANOVA *Duncan test*). Asterisks indicate significant differences between the two feed groups at the same time point (p < 0.05, *t-test)*.

To examine the effects of feeding and eyestalk ablation on ovarian maturation of domesticated female black tiger shrimp broodstock, shrimps were fed with either broodstock pellets or live polychaetes for four weeks before an eyestalk ablation was performed (Fig. 1). Both feeds could not induce ovarian maturation over the 4-week feeding period as evidenced by the pale white color of ovaries (Fig. 1A) and the insignificant changes in gonadosomatic index (GSI), a known indicator of ovarian maturation in the female black tiger shrimp (0.57 ± 0.18% to 0.54 ± 0.18% for the pellet-fed group and 0.57 ± 0.18% to 0.72 ± 0.21% for the polychaete-fed group; Fig. 1B, Supplementary Table S1).

After the eyestalk ablation, ovaries of the two groups matured differently. The ovaries of the pellet-fed group did not develop further than previtellogenic stage (stage I, see Materials and Methods for definition of ovarian stages) as they remained white-yellow in color and small in sizes with the GSIs of 0.61 ± 0.20%, 0.48 ± 0.08%, and 0.78 ± 0.31% for Day 1, 4, and 9 post ablation, respectively. On the other hand, the polychaete-fed shrimp were able to reach the reproductively mature stage after the eyestalk ablation as their ovaries continually became larger and eventually turned greenish by Day 9 post ablation. Their GSIs started from 0.72 ± 0.21% and significantly increased to 1.26 ± 0.57%, 3.61 ± 1.14%, and 5.88 ± 0.58% after eyestalk ablation at Day 1, 4, and 9, respectively (Fig. 1B, Supplementary Table S1).

### *P. monodon* ovarian transcriptome assembly and annotation

To generate the reference ovarian transcriptome sequences for black tiger shrimp, we applied both short-read Illumina sequencing and long-read Pacific Biosciences (PacBio) isoform sequencing (Iso-seq). The depth coverage of Illumina sequencing allowed us to capture the sequences of rare transcripts (Supplementary Table S2) while the long PacBio reads enabled the identification of full-length transcripts (Supplementary Table S3). After processing raw read data from the PacBio RS II, we obtained a total of 546,473 reads of insert (ROIs) totaling 18.82 Gb from 8 SMRT cells (Table 1; Supplementary Table S3). Of the 546,473 ROIs, 26,609 were high-quality, full-length non-chimeric transcripts identified based on the presence of 5′ and 3′ cDNA primers and a polyA tail signal preceding the 3′-primer. The sizes of the ROIs ranged from 10 to 15,000 with an average read length of 2,087 and an N50 of 2,454 nucleotides. Short-read RNA sequencing was also carried out on the Illumina NovaSeq^TM^ 6000 and yielded 1,235,809,734 raw reads, totaling 187.92 Gb. After a *de novo* transcriptome assembly using Trinity v2.2.0^17^, we obtained 398,088 assembled transcripts ranging from 201 to 13,211 nucleotides. Of which, 204,819 sequences had a length of 300 nucleotides or longer (Table 1). To enhance the coverage of the reference transcriptome assembly, we combined high-quality, full-length transcripts from PacBio Iso-seq experiment with Trinity-assembled transcripts longer than 300 nucleotides. After clustering these sequences at 95% identity, we obtained a final set of 33,277 non-re dundant transcripts with an average contig length of 917 nucleotides and an N50 length of 1,728 nucleotides (Table 1). We investigated the coverage of the benchmarking universal single-copy orthologs (BUSCO)^18^ to assess the completeness of the gene space in the assembly. Even though we only examined transcripts deriving from ovary tissues of the black tiger shrimp, 92.7% of the assembly was identified as ‘complete’ (i.e., the length of the recovered genes are within two standard deviations of the BUSCO group mean length^18^), suggesting that the assembly is relatively complete with respect to the gene space. Gene ontology (GO) annotations describing biological processes, molecular functions, and cellular components were retrieved for 15,480 sequences (46.52% of the assembly; Supplementary Fig. S1). The most prevalent GO term associated with biological processes were oxidation-reduction process (239) followed by protein phosphorylation (157), translation (147), proteolysis (146), regulation of transcription (139), membrane transport (111) and signal transduction (103; Supplementary Fig. S1, Table S4). Among genes annotated to various molecular functions, the largest category was ATP binding (597), followed by metal ion binding (286) and nucleic acid binding (271), another ten subcategories with 100 or more terms (Supplementary Fig. S1, Table S4).

**Table 1:** Assembly statistics for *P*. *monodon* transcriptome.

**PacBio Iso-seq:**	
Number of PacBio raw reads	546,473
Number of bases sequenced on PacBio (Gb)	18.82
Number of high-quality PacBio Iso-seq reads	26,609
**Illumina RNA-seq:**	
Number of Illumina raw reads	1,235,809,734
Number of bases sequenced on Illumina (Gb)	187.92
Number of Illumina Trinity-assembled contigs> 300 nt	204,819
**Transcriptome assembly:**	
Number of non-redundant contigs (PacBio + Ilumina)	33,277
Mean GC content	46.90
Mean contig length (nt)	917.65
Median contig length (nt)	459
Contig N90 (nt)	1,329
Contig N50 (nt)	1,728
Contig N20 (nt)	2,862
Maximum contig length (nt)	13,077
Total assembled bases	30,539,792
**Transcriptome annotation:**	
% of contigs annotated	46.52
% core KOGs	30.01
**BUSCO:**	
% complete	92.7
% partial	4.7
% missing	2.6

### Differential gene expression profiles

#### Feed effects

**Figure 2:**
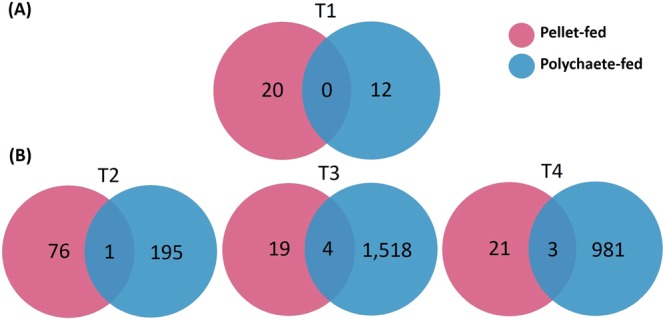
Venn diagram of DEGs of the pellet-fed shrimp and the polychaete-fed shrimp (**A**) after the 4-week feeding trial and before eyestalk ablation (T1) and (**B**) after eyestalk ablation at Day 1, 4, and 9 (T2, T3, and T4, respectively).

After the 4-week feeding trial, only a small portion of total transcripts was differentially expressed in both feed groups, and each type of diet regulated different sets of genes (Fig. 2A). For the pellet-fed group, 20 transcripts were differentially expressed, whereas 12 transcripts were for the polychaete-fed group. The pellet-fed group significantly induced mitochondrial assembly of ribosomal protein, furin-like protease, replication protein and transcription factor, all of which appeared to be involved in regular cell maintenance. On the other hand, the polychaete-fed group differentially induced genes related to oogenesis such as *vitellogenin*, *collagen alpha-1V chain-like*, and *heme-binding protein 2-like*, suggesting that the polychaete-fed shrimp were able to prepare for ovarian maturation process after obtaining nutrients necessary for reproduction provided by polychaetes.

#### Eyestalk ablation effects

**Figure 3:**
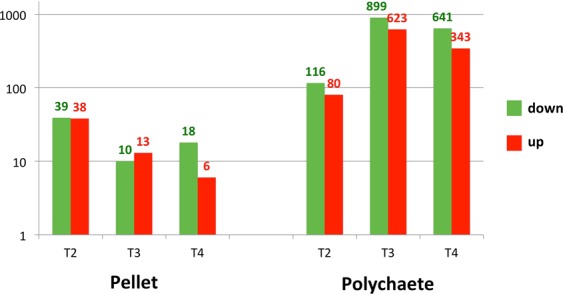
Numbers of DEGs after eyestalk ablation of the pellet-fed shrimp and the polychaete-fed shrimp at Day 1, 4, and 9 (T2, T3, and T4, respectively). Red and green bars indicate up- and downregulated DEGs. A base-10 log scale is used for the Y axis.

After the eyestalk ablation, the two feed groups exhibited almost entirely different expression profiles, and the two diets regulated different sets of genes (Supplementary Fig. S2). The number of differentially expressed genes (DEGs) was higher for the polychaete-fed group than the pellet-fed group throughout the ablation time course (Fig. 3A). While the pellet-fed group differentially expressed most number of genes (77 transcripts) at Day 1 after the ablation, the polychaete-fed group differentially expressed most number of genes (1,522 transcripts) at Day 4 (Fig. 3), coinciding with the time when the most noticeable change in ovarian physiology occurred (Fig. 1A).

**Figure 4:**
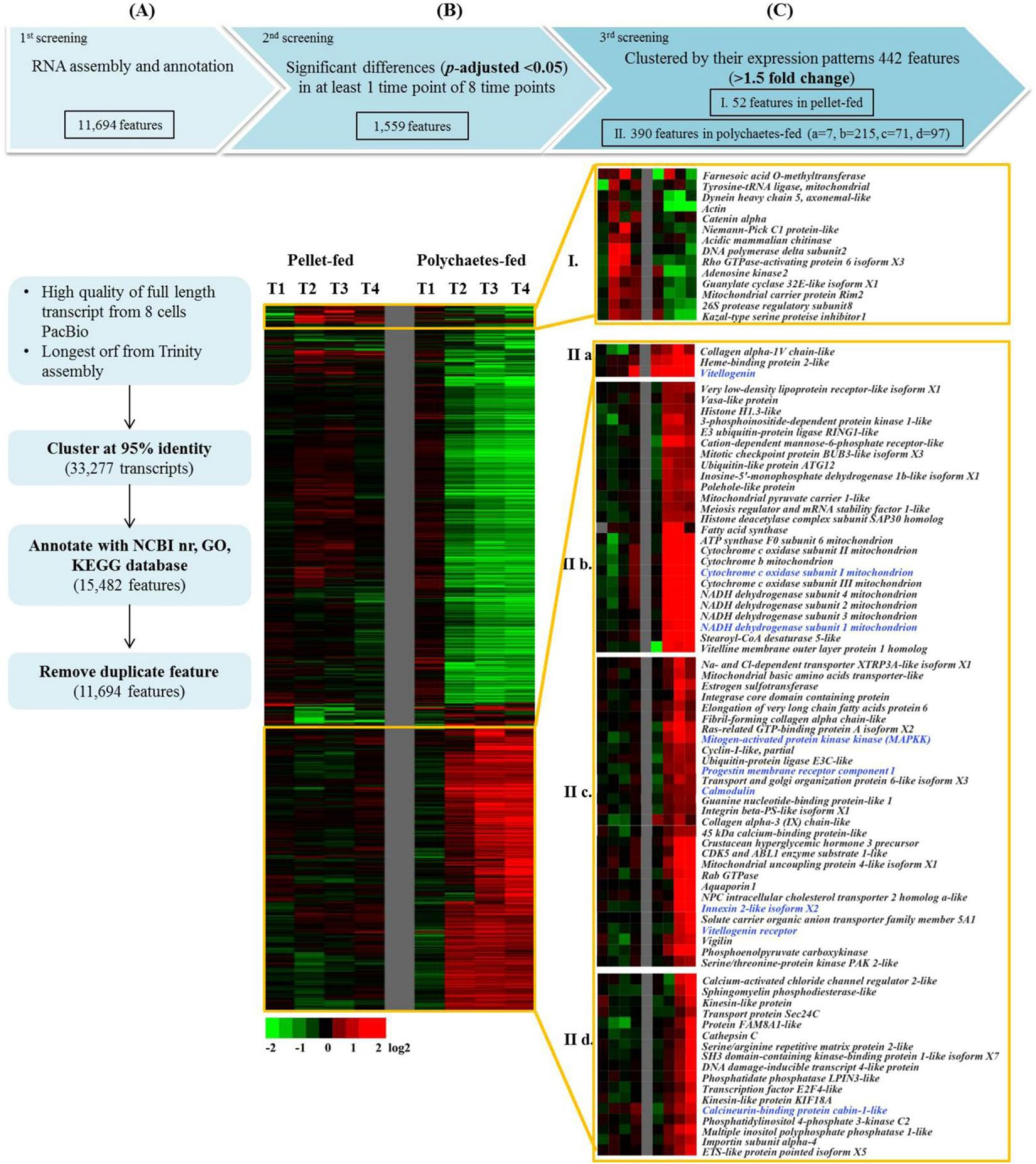
Gene expression analysis by RNA sequencing. Transcript levels in ovaries after a 4-week feeding trial (T1) were compared to before the trial (T0). Transcript levels in ovaries after eyestalk ablation at Day 1, 4 and 9 (T2, T3, and T4, respectively) were compared to those from non-ablated broodstock (T1). (**A**) A total of non-redundant and annotated 11,694 features were obtained from RNA sequencing. (**B**) Hierarchical clustering analysis of the 1,559 features with significant differences (p-adjusted < 0.05) in at least 1 time point of 8 time points. Transcript levels in ovaries after eyestalk ablation at Day 1, 4 and 9 (T2, T3, and T4, respectively) were compared to those from non-ablated broodstock (T1). (**C**) Genes that exhibited changes in their expression level > 1.5 folds (442 features) were clustered into Cluster I–II exhibiting various differentially expressed patterns as effects of diets and eyestalk ablation: Upregulation in pellet-fed group (I), Upregulation in polychaete-fed group (II) with cluster IIa contains genes with upregulation before (T1) and after (T2-T4) the ablation, cluster IIb contains genes with upregulation after the ablation (T2, Day 1) until T4 (Day 9), cluster IIc contains genes with upregulation at T3 (Day 4) after the ablation, and cluster IId contains genes with upregulation at T4 (Day 9) after the ablation. Transcripts in blue letters were further characterized by quantitative real-time PCR (Supplementary Fig. S4).

To examine transcriptomic responses after eyestalk ablation, DEGs at each time point were identified. From 11,694 non-redundant annotated transcripts, 1,559 features that were significantly up- or downregulated (p-adjusted <0.05) in at least one time point were clustered according to their expression patterns (Fig. 4B). We subsequently clustered them based on their expression patterns and selected groups of genes that were affected by feed and eyestalk ablation. A total of 442 features exhibited changes in their expression level >1.5 folds (Supplementary Table S5), and selected features with known functions were plotted in groups according to their similar expression patterns (Fig. 4C).

The eyestalk ablation affected almost totally different sets of genes between the two feed groups with negligible number of co-expressed genes in both time courses (Fig. 2B). For the pellet-fed group, the early response at Day 1 seemed to induce genes involved in stress recovery such as cytoskeletal and cell repair (*actin*, *catenin alpha*, *acidic mammalian chitinase*), homeostasis maintenance (*mitochondrial carrier Rim2*), and protein repair (*adenosine kinase2*, *tyrosine-tRNA ligase*, *mitochondrial*; Fig. 4). At Day 4 after the ablation, the pellet-fed group differentially induced farnesoic acid O-methyltransferase (*FAMeT*), *Niemann-Pick C1 protein-like*, and *rho GTPase-activating protein 6 isoform X*3, while the well-known ovarian maturation marker gene, *vitellogenin* (Fig. 4, Cluster IIa), was only differentially induced at Day 9 after the eyestalk ablation.

For the polychaete-fed group, a large number of transcripts were differentially expressed after the eyestalk ablation, which can be categorized according to their expression patterns (Fig. 4). The first pattern includes transcripts that were upregulated after four weeks of polychaete feeding and their levels of expression continually increased after the eyestalk ablation (Fig. 4, Cluster IIa). These genes are *vitellogenin*, *collagen alpha-1V chain-like*, and *heme-binding protein 2-like*. The second group of genes were upregulated early (Day 1), and most of them remained upregulated until the end of the time-course experiment after the ablation (Fig. 4, Cluster IIb). This group includes fatty acid regulatory genes (*fatty acid synthase*, *very low density lipoprotein receptor-like isoform X1*, *stearoyl-CoA desaturase 5-like*, *cation-dependent mannose-6-phosphate receptor-like*, *mitochondrial pyruvate carrier1-like*), mitosis and meiosis regulation (*mitotic checkpoint protein BUB3-like isoform X3*, *meiosis regulator and mRNA stability factor 1-like*), purine regulatory genes *(inosine-5*′*-monophosphate dehydrogenase 1b-like isoform X1 and 3-phosphoinositide-dependent protein kinase 1-like*), protein degradation/modification via ubiquitination (*E3 ubiquitin-protein ligase RING1-like*, *ubiquitin-like protein ATG12*, *histone H1*.*3-like*, *histone deacetylase complex subunit SAP30 homolog*, energy production genes (*NADH dehydrogenase subunit 1-4*, *cytochrome b mitochondrion*, *cytochrome c oxidase subunit I*, *II*, *III mitochondrion*, *ATP synthase)*, and oogenesis genes (*polehole-like protein*, *vasa-like protein* and *vitelline membrane outer layer protein 1 homolog*; Fig. 4: Cluster IIb).

At Day 4 after the ablation, several genes belonging to a hormone-mediated ovarian maturation and calcium signaling pathways such as *progestin membrane receptor component 1* (*PGMRC1)*, *estrogen sulfotransferase*, *crustacean hyperglycemic hormone 3 precursor*, *calmodulin*, *guanine nucleotide-binding protein-like 1* (Gq/11) and *45* *kDa calcium binding protein-like* were differentially induced. In addition, transport proteins (*transport and Golgi organization protein 6-like isoform X3*), fatty acid regulatory genes (*elongation of very long chain fatty acids protein 6*), collagen gene and its receptor gene (*collagen alpha-3(IX) chain-like*, and *integrin beta-PS-like isoform X1)*, and cellular development (*mitogen-activated protein kinase kinase (MAPKK)*) were induced at Day 4 (Fig. 4: Cluster IIc). For Day 4 to Day 9 after the ablation, vitellogenesis genes (*vitellogenin receptor* and *NPC intracellular cholesterol transporter 2 homolog a-like*), cellular development genes (*innexin 2-like isoform X2*, *rab GTPase* and *CDK5 and ABL1 enzyme substrate 1-like*), osmotic regulatory gene (*aquaporin 1*), reproduction-related gene (*vigilin*) and gluconeogenesis gene (*phosphoenolpyruvate carboxykinase*) were induced (Fig. 4: Cluster IIc). For the later time point at Day 9, *cathepsin C*, *kinesin-like protein KIF18A*, *calcineurin-binding protein cabin-1-like*, *DNA damage-inducible transcript 4-like protein*, *multiple inositol polyphosphate phosphatase 1-like*, *transport protein Sec*. *24* *C* and *calcium-activated chloride channel-regulator 2-like* were induced (Fig. 4: Cluster IId).

**Figure 5:**
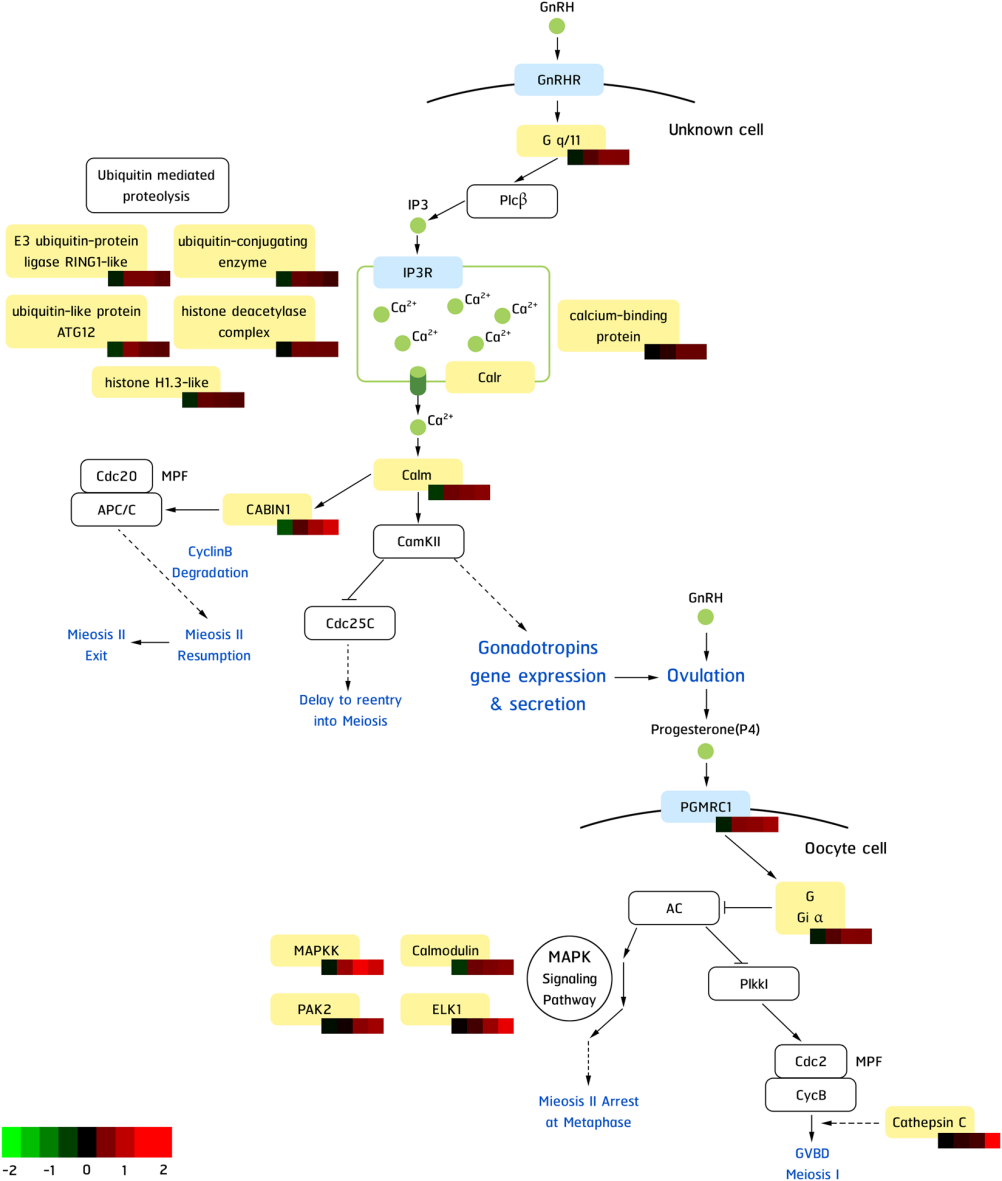
Differential expressed genes in the hormone-mediated ovarian maturation pathways in the polychaete-fed group. Light blue box, yellow box and white box represent receptor genes, genes were found in this study, and genes known to be in this pathway, respectively. The heatmap bars under each gene in the pathway reflect expression change from RNA-seq result after a 4-week feeding trial (T1) compared to before the trial (T0), after eyestalk ablation at Day 1, 4 and 9 (T2, T3, and T4, respectively) compared to those from non-ablated broodstock (T1). GnRH: Gonadotropin releasing hormone, GnRHR: GnRH receptor, G q/11: guanine nucleotide-binding protein G(q) subunit alpha, Plcβ: phospholipase C beta, IP3: inositol-1,4,5-triphosphate, IP3R: IP3 receptor, Calr: calreticulin, Calm: calmodulin, CABIN1: calcineurin-binding protein cabin-1-like, Cdc20: cell division cycle 20, APC/C: Anaphase-promoting complex, CamKII: calmodulin kinase II, Cdc25C: cell division cycle 25C, PGMRC1:progestin membrane receptor component 1, G Gi α: guanine nucleotide-binding protein G(i) subunit alpha, AC: adenylate cyclase, Plkkl: Polo-like kinase I, Cdc2: cell division cycle 2, CycB: cyclinB, MPF: maturation-promoting factor, MAPKK: mitogen-activated protein kinase kinase, PAK2: serine/threonine-protein kinase PAK 2-like, ELK1: ETS-like protein pointed isoform X5.

From these 442 differentially expressed genes, pathway analysis was performed to identify enriched pathways and the most enriched pathway was found to be very general “metabolic pathway” (Supplementary Fig. S3). However, the second most enriched pathway namely the gonadotropin-releasing hormone (GnRH)/MAPK signaling pathway presented relevant biological functions to the reproductive maturation. Therefore, expression levels of these transcripts in this pathway were further investigated and potentially found to be involved in the shrimp reproductive maturation (Fig. 5).

### Validation of gene expression by quantitative real-time PCR (qPCR) analysis

To confirm the expression patterns of selected transcripts with differential expression levels in the RNA-seq result, real-time qPCR was performed for nine transcripts with significant expression levels in polychaete-fed group, especially after the eyestalk ablation (Supplementary Fig. S4). All examined transcripts were differentially expressed between pellet-fed and polychaete-fed groups after the 4-week feeding trial. As observed in the RNA-seq result, *vitellogenin* was expressed highly in polychaete-fed group after the 4-week trial. After the eyestalk ablation, all of these transcripts were expressed higher in the polychaete-fed group than the pellet-fed group, with *cytochrome c oxidase* and *vitellogenin* being the most obvious transcripts.

## Discussion

Poor ovarian maturation in shrimp domestication hampers further growth and viability of the industry. While several methods such as boosting with live feeds and unilateral eyestalk ablation have been used to induce the maturation, the molecular effects of these methods have not been investigated in detail. This study employed transcriptomic analyses to elucidate the effects of live feed diet and eyestalk ablation on ovarian maturation and reveal important genes and pathways involved. The availability of high-quality ovarian transcriptome assembly is crucial for understanding the underlying transcriptional regulations triggered by the interactions between diet and eyestalk ablation. Previously, we employed 454 pyrosequencing technology to obtain transcriptomic information on reproduction of the *P*. *monodon* ovary and testis, and found several novel sets of transcripts^19^. However, the transcriptomic information obtained was of short-read sequences due to the limitation of the sequencing technology employed at that time. In this work, we reported the first high-quality reference ovarian transcriptome in *P*. *monodon* assembled from both Illumina short-read sequences and PacBio long-read sequences, the latter of which enabled us to capture full-length transcripts. Our assembly has a much higher proportion of annotated sequences (46.52%) compared to the number reported in a previous study (17.83%)^20^. Future expression studies and genome sequencing project in black tiger shrimp will greatly benefit from the annotated transcriptome assembly reported here.

Ovarian maturation in penaied shrimp requires coordination of large sets of genes for dramatic physiological changes into four stages: (I) previtellogenesis, (II) vitellogenesis, (III) early cortical rod, and (IV) late cortical rod^2^. This study shows that without eyestalk ablation, the *P*. *monodon* broodstock was unable to develop pass the previtellogenesis stage regardless of the feed given. However, eyestalk ablation was able to induce ovarian maturation only in the group fed with polychaetes, indicating that there was a synergy between polychaetes and ablation effects. The significant changes in GSI and physiology of the polychaete-fed ovaries after the ablation suggested that feeding with polychaetes helped prepare the broodstock before the eyestalk ablation.

### Feed effects

Nutritional requirements for shrimp reproduction are poorly defined. Generally, live feeds such as polychaetes are used to prepare broodstock before mating, but their nutritional value can vary with species, season of collection and life stage^21^. Their use is also labor-intensive, expensive and affects water quality^22^. The replacement of live feeds with dry feeds for shrimp reproduction will not be possible without understanding the molecular effects of how these live feeds prepare broodstock during ovarian maturation.

From the transcriptomic data, diets showed strong influences on gene regulation. The two examined types of feed regulated totally different sets of genes throughout the experiment as evidenced by an almost complete absence of co-expressed DEGs in both feed groups, suggesting that the pellet and polychaete utilized different molecular pathways. For the pellet-fed group, genes with altered expression levels belong to regular cell maintenance, and there was no significant alteration of expression levels for genes related to reproduction. Oppositely, the polychaete-fed group differentially expressed a large number of important genes whose functions are relevant to the preparation for ovarian maturation.

Polychaetes are high in polyunsaturated fatty acids (PUFAs), which have been reported to enhance ovarian maturation in many crustacean species such as the Pacific white shrimp *Litopenaeus vannamei*, the Chinese mitten crab *Eriocheir sinensis*, the mud crab *Scylla serrata* and *P*. *monodon*^7–9,23^. This study reveals the benefits of polychaete at the molecular level, which are highlighted by a strong induction of an oogenesis gene marker, *vitellogenin*, just after four weeks of polychaete feeding and prior to the eyestalk ablation (Fig. 4: Cluster IIa). Vitellogenin, also known as apolipophorin, is a precursor for the synthesis of a major egg yolk protein vitellin through vitellogenesis process^24^. This protein provides nutrition required for crustacean embryo development^25^. Hepatopancreas and ovary are common sites for yolk biosynthesis in several penaeid shrimps such as *Litopenaeus vannamei*^26^, *Penaeus semisulcatus*^27^ and *Penaeus monodon*^28^. While the level of vitellogenin transcripts was significantly induced in the polychaete-fed group within the first 24 hours after the removal of the eyestalk, the same induction was not observed until Day 9 for the pellet-fed group (Fig. 4: Cluster IIa). Coordination of reproduction and yolk biosynthesis is achieved via the endocrine system, which was partly located in the eyestalk^29,30^. While it is not surprising that the *vitellogenin* transcript was induced upon removing GIH through eyestalk ablation, it is worth noting that its expression level was induced even before the ablation in the polychaete-fed group, providing the evidence to support that ovarian maturation not only depends on the release of hormone, but also on the readiness primed by specific nutrition obtained from the polychaetes. Moreover, the fact that the *vitellogenin transcript* was upregulated at Day 9 after the ablation in the pellet-fed group but did not result in ovarian maturation also suggests that the completion of the maturation process requires various factors to work together.

In addition to the induction of *vitellogenin*, polychaete feeding differentially induced *heme-binding protein* and *collagen alpha-1* even before the eyestalk ablation. While both of these genes have not previously been studied in *P*. *monodon*, they have been linked to reproduction in other organisms. *Heme-binding protein* encodes a specific yolk protein in the insect *Rhodnius prolixus*, and its uptake in the ovary was modulated by eicosanoids^31^. Eicosanoids, hormone-like mediators of reproduction, have been reported to be involved in female reproduction of numerous crustaceans such as *Daphnia magna*^32^. Eicosanoids or prostanoids play crucial roles in regulation of female reproductive maturation^33,34^. Injection of some PUFA derivatives such as prostaglandin F(2alpha) and prostaglandin E(2) significantly increased an ovarian index and oocyte diameter in fresh water crabs^35^. PUFAs in polychaetes are the precursors of eicosanoids; thus, it is not surprising that *heme-binding protein* was induced after feeding polychaetes for four weeks. Similarly, *collagen alpha-1* was induced before eyestalk ablation in the polychaete-fed group. Although the roles of collagen for ovarian maturation has not been studied in the penaied shrimp, collagens are a major component of the extracellular matrix, which regulates follicle maturation and a process of explosive follicle growth called folliculogenesis in several model organisms^36^. Various types of collagens were present throughout the surface epithelium, follicular compartments, stroma, and granulosa cells of mouse ovary^37^.

### Synergistic effects from diets and eyestalk ablation

The most interesting DEGs were those induced in the polychaete-fed group after the eyestalk ablation but not in the pellet-fed group. We hypothesized that those transcripts were up- or downregulated as a result of synergistic effects from polychaete feeding and eyestalk ablation. While most of those genes were involved in fatty acid regulation, energy production, and hormone-mediated oocyte maturation pathways, there were several genes whose functions have previously been linked to various biological processes worth examining in detail.

#### Fatty acid regulation genes

Given polychaete’s richness in PUFAs, it is not surprising to find several genes involved in fatty acid regulatory networks differentially specifically induced in the polychaete-fed group, especially after the eyestalk ablation. Previously, genes in the fatty acid regulation pathways have been shown to be highly expressed in mature ovaries of *P*. *monodon* fed with polychaetes after eyestalk ablation^38^. Fatty acids and dietary lipids are important for vitellogenesis as yolk proteins are composed mainly of lipoglycoproteins^39^. The importance of fatty-acid rich feed to the ovarian development in crustaceans has previously been demonstrated. In *L*. *vannamei*, the higher fatty acid content in feed led to a higher vitellogenin concentration in hemolymph^40^, whereas dietary lipids influenced gene expression of fatty acid regulatory pathways including fatty acid beta-oxidation, metabolism, synthesis and transport in the Chinese mitten crab *E*. *sinensis* and the Pacific white shrimp *L*. *vannamei*^41,42^.

#### Energy production genes

The induction of energy-related genes such as *ATP synthase*, *cytochrome*, *NADH dehydrogenase*, *phosphoenolpyruvate carboxykinase*, *glucose receptor* seems to be a result of synergistic effect from the polychaete feeding and the eyestalk ablation (Fig. 4: Cluster IIb). The importance of energy to reproduction has been evidenced by the fact that a large amount of lipids stored in the hepatopancreas are used for energy generation and directly transferred to the ovary during ovarian development^43^.The lipid stored in eggs will be mobilized for energy production during the course of embryogenesis^44^.

#### Hormone-mediated oocyte maturation genes

Another interesting group of genes that were upregulated specifically in the polychaete-fed group after the eyestalk ablation includes genes encoding gonadotrophin releasing hormone (GnRH) signaling, calcium signaling and progesterone pathways such as *calmodulin*, *45* *kDa calcium-binding protein-like*, *mitogen-activated protein kinase kinase (MAPKK)*, *progestin membrane receptor component 1 (PGMRC1)*, and *progesterone-induced-blocking factor* (Fig. 4: Cluster IIc, Supplementary Table S5). Moreover, *cathepsin C* was found to be highly induced at the later time point after the eyestalk ablation in the polychaete-fed group, which coincides with the final stage of oocyte maturation indicated by the GSI and ovarian morphology. Cathepsin C, known as dipeptidyl peptidase I (DDPI) or dipeptidyl transferase, is a lysosomal cysteine protease that is involved in the final stages of oocyte maturation in kuruma prawn (*Marsupenaeus japanicus*)^45^. It plays physiological roles in intracellular protein degradation and has been postulated to facilitate cortical rod expulsion at the exposure of seawater. Our expression data agree well with the literature, suggesting that *cathepsin C* plays a vital role in the final stage of the *P*. *monodon* oocyte maturation. The induction of these genes confirms our finding from previous studies using cDNA microarray to decipher affected pathways after eyestalk ablation^16^ and 454 pyroseqeuncing to reveal pathways and genes related to reproduction of *P*. *monodon*^19^. The expression patterns of these gene were validated using real-time qPCR. Most of the results from the qPCR agreed with those from the RNA-seq.

#### Ubiquitin, protein degradation or modification genes

Genes involved in ubiquitin-mediated proteolysis, protein degradation or modification (importin subunit alpha-4, E3 ubiquitin-protein ligase RING1-like, ubiquitin-like protein ATG12, histone H1.3-like, histone deacetylase complex subunit SAP30 homolog) were up-regulated after eyestalk ablation in the polychaete-fed group. Protein ubiquitination plays a role in protein refolding and is involved in the control of a multitude of processes such as cell differentiation, the cell cycle, responses to stress, chromatin modification in the context of DNA repair, gene silencing, as well as, gametogenesis in various organisms^46^. For histone genes, it is not surprising to see their involvement during the ovarian maturation in this study. In *Caenorhabditis elegans*, a histone deacetylase, HDA-1 forms a protein complex with nuclear autoantigenic sperm protein (NASP) and a zinc finger transcription factor, TRA-4 to promote female development^47^, while NASP has been linked to female reproductive maturation in P. monodon^48^.

#### Other genes

First, crustacean hyperglycemic hormone (CHH) is a neuropeptide that is synthesized, stored and released by eyestalk structure in crustaceans^49^. From our result, *crustacean hyperglycemic hormone 3 (CHH3) precursor* was up-regulated after eyestalk ablation in the polychaete-fed group. When we performed the sequence alignment (BLASTX) of our CHH3 precursor to the Genbank nr database, the best hit returned was CHH3 precursor from *L*. *vannamei*. When we aligned our CHH3 precursor with previously published Pem-CHH1, Pem-CHH2 and Pem-CHH3 sequences of *P*. *monodon*^50^, the percent identity obtained was very low (<20%) and the aligned portions were also small. Based on these results, it is unlikely that our CHH3 precursor is the same as Pem-CHH3 (or Pem-CHH1 and Pem-CHH2). It is probably a new member of the CHH family that has not been reported.

It is a member of a structurally related peptide family, which includes molt-inhibiting hormone (MIH), gonad-inhibiting hormone (GIH) and mandibular organ-inhibiting hormone (MO-IH)^50^. Reported to be involved in several physiological processes such as edysteroid production, lipid metabolism, ovarian physiology and osmoregulation, CHH’s main function is to regulate sugar levels in the hemolymph and glycogen metabolism. Given that the upregulation of this gene was observed after the eyestalk ablation only in the polychaete-fed group (Fig. 4: Cluster IIc), we hypothesize that this gene helps boost the sugar level in ovary tissues to provide the energy necessary for the ovarian maturation.

*Polehole* exhibiting similar expression patterns within the synergistic gene group may play important roles for ovarian maturation. In *Drosophila*, *Polehole* located on the oocyte surface mediates the assembly of the eggshell participating in the Torso RTK signaling pathway that specifies the terminal regions of the embryo and crosslinks vitelline membrane product^51^. In *P*. *monodon*, a transcript that significantly matched the polehole precursor was characterized and found to be expressed specially in ovary, highly expressed after eyestalk-ablation and localized in the ooplasm of oocyetes, suggesting its important function in oocyte development and maturation^52^. However, its detailed molecular mechanism needs to be further explored.

## Conclusion

Our RNA-seq results strongly suggest that there are synergistic effects between the polychaete feeding and the eyestalk ablation in the process of ovarian maturation in black tiger shrimp. Without having proper nutrients from the polychaetes, female broodstock might not be ready to develop its ovary; however, even with the proper nutrients, the eyestalk ablation is still necessary to perhaps manipulate the female endocrine of the black tiger shrimp. The synergy seems to be the induction of important groups of genes, namely energy production genes, endocrinological genes, fatty acid regulatory genes, and oogenesis genes. These findings shed to light on molecular mechanisms and key molecular pathways that lead to successful ovarian maturation.

## Material and Methods

### Experimental design, sample collection, and RNA extraction

The shrimp culture conditions were followed a previously published work^16^. Female domesticated *P*. *monodon* broodstock (9-month-old) and live sand polychaetes *Perinereis nuntia* (6-month-old) were cultured at the Shrimp Genetic Improvement Center (SGIC, Surat Thani, Thailand). Brooders were maintained at a biosecure station containing seawater pumped from the Gulf of Thailand at a salinity of 30 ppt with water temperature at around 27 °C, and acclimated in tanks with 2 m in diameter for 2 weeks. The brooders were separated into two groups (n = 46 each): pellet-fed (Pe) and polychaete-fed (Po) groups. Pellet (Pe) and polychaete (Po) groups were fed five times/day (8:00, 11:00, 14:00, 16:30, and 22:00 h). The amount of feed/shrimp weight/day was 2% broodstock pellets (Charoen Pokphand Foods PCL) for Pe group and 7% polychaetes for Po group.

Ovaries of the brooders were collected at the beginning of the feeding trial (T0), 4 weeks after feeding trial before unilateral eyestalk ablation (T1), and 1, 4, and 9 days after unilateral eyestalk ablation (T2, T3 and T4, respectively; Fig. 1A). All ovary samples were quickly frozen in liquid nitrogen and stored at −80 °C until use. The gonadosomatic index (GSI) of each shrimp was calculated as a percentage of ovary weight to body weight, classifying different ovarian developmental stages as the following: previtellogenic (stage I, GSI < 2), vitellogenic (stage 2, GSI = 2–4), early cortical rod stage (stage 3, GSI =<4–6) and mature stage (stage 4, GSI > 6)^53^. Differences among GSI from time points of the same feed group were considered significant Duncan; ANOVA (95% confidence).

RNA extraction was performed according a published protocol^54^. Briefly, frozen ovary tissues were pulverized in liquid nitrogen for RNA extraction using TRI-REAGENT (Molecular Research Center, USA). Contaminating genomic DNA was removed by treatment with DNase I at 0.15 U/µg total RNA at 37 °C for 30 min. The quality and quantity of the RNA were assessed using gel electrophoresis and a NanoDrop (ND-8000) spectrophotometer before subsequent experiments.

### Transcriptome sequencing

To perform Iso-seq sequencing, we followed the protocol reported in Pootakham *et al*.^55^. Equal amounts of RNA samples from all treatments were pooled (Supplementary Table S1 indicates the samples that were used for Iso-seq), and poly(A) mRNAs were enriched using the Dynabeads mRNA Direct Purification Kit (Thermo Fisher Scientific, Waltham, USA). The RNA integrity was assessed using the Fragment Analyzer (Agilent Technologies, Santa Clara, USA) prior to the construction of the Iso-seq library. We followed the Iso-seq protocol from Pacific Biosciences, using the SMARTer PCR cDNA Synthesis Kit (Clontech, Mountain View, USA) to construct the library and the BluePippin Size Selection System (Sage Science, Beverly, USA) to partition the library into 1–2 kb, 2–3 kb and 3–6 kb ranges. The sequencing was performed on a PacBio RS II instrument using P4-C6 polymerase and chemistry with 360 min movie times (Pacific Biosciences, Menlo Park, USA).

To obtain short-read RNA sequences, Illumina sequencing was performed at Macrogen (South Korea). Each library was constructed from 1 μg total RNA from the ovaries of 2–4 individuals per treatment (Supplementary Table S1 indicates the samples that were used for RNAseq) using the TruSeq stranded Library Preparation kit (Illumina, San Diego, USA) and sequenced using Illumina NovaSeq™ 6000 instrument with 150 bp pair end read. Sequence data were deposited in the NCBI GenBank under the BioProject ID PRJNA542029.

### Transcriptome assembly and annotation

Transcriptome assembly based on the Iso-seq data was performed according to the method reported in Pootakham *et al*.^55^. The PacBio SMRT Analysis Package (version 2.3; default parameters) was used to process raw reads from the PacBio RS II into error-corrected reads of insert. Adapter sequences, poly-A tails, artificial concatemers and 3′ truncated transcript sequences were removed using the Iso-seq protocol with default parameters. An additional round of error correction was carried out on the full-length non-chimeric transcripts using the PacBio ICE software without the Quiver step^56^. If the consensus accuracy was ≥ 0.99 (default parameter; https://github.com/ben-lerch/IsoSeq-3.0/blob/master/README.md), the polished isoforms were classified as “high-quality.”

Illumina short reads were quality-filtered and adapter-trimmed using TrimGalore (https://github.com/FelixKrueger/TrimGalore). FastQC v0.11.8 (http://www.bioinformatics.babraham.ac.uk/projects/fastqc/) was used to check data quality before and after trimming. After the removal of low-quality reads, an Illumina-based de novo transcriptome assembly was performed using Trinity v2.2.0 using the default parameter setting^17^. In order to obtain a complete ovarian transcriptome, we combined high-quality, full-length transcripts from the Iso-seq experiment with Trinity-assembled transcripts. To remove redundant sequences and obtain reference transcripts from the combined set of transcripts, we clustered these transcript sequences at 95% identity using UCLUST^57^. This reference transcriptome assembly was assigned function using BLASTX against custom NCBI non-redundant eukaryote protein database with an E-value cutoff of 1 × 10^−10^. BLASTX results were imported into Blast2Go Pro (version 5.2.4) for mapping and retrieving gene ontology (GO) terms associated with predicted sequences. The software also assigned the enzyme commission (EC) number and Kyoto encyclopedia of genes and genomes (KEGG) pathway annotations to the query sequences. The completeness of the transcriptome was evaluated using the coverage of the benchmarking universal single-copy orthologs (BUSCO)^18^.

### Mapping and differential gene expression analysis

The pair-end Illumina reads were aligned to the reference transcriptome using Bowtie 2 version 2.2.9^58^. The total mapped reads in each bam file were counted by HTSeq package through the HThtseq-count union function [14].

The R package DESeq. 2 [15] provides a test for differential expression using negative binomial generalized linear models, will operate to identify the differently expressed genes between shrimp fed polychaetes or pellet at different time points after eyestalk ablation. After read count normalization, log2 fold changes between shrimp fed polychaetes or pellet after eyestalk ablation will be calculated. Significant differently expressed genes will be identified with Bonferroni adjusted p-value of <0.05. Genes displayed in Fig. 4 were selected based on their expression profiles. Our ovarian transcriptome assembly contained a total of 11,694 non-redundant features. We first selected genes that were significantly (p-value < 0.05) up- or down-regulated during at least one time point (1,559 features). We subsequently clustered them based on their expression patterns and selected groups of genes that were affected by feed effect and eyestalk ablation using Cluster 3.0 software^59^. A total of 442 features exhibited changes in their expression level > 1.5 folds (Supplementary Table S5), and selected features with known functions were plotted in groups according to their similar expression patterns based on the results obtained from Cluster 3.0 software. Treeview version 1.6 was used to visualize gene expression data in Fig. 4^59^. KEGG Automatic Annotation Server (KAAS) program was used to associate nucleotide sequences from 442 features to KEGG (Kyoto Encyclopaedia of Genes and Genomes) Orthology database (http://www.genome.jp/kegg/kaas/). Subsequently, the KEGGREST in R package was used to map KEGG Orthology (KO) to the pathways (Tenenbaum D (2019). KEGGREST: Client-side REST access to KEGG. R package version 1.26.1).

### Quantitative real-time PCR (qPCR) validation of selected genes

Among differentially expressed genes, nine genes were selected for qPCR validation. Primers were designed using Primer Premier Program (Table 2). We followed the previously published protocol^16^. Briefly, 15 μg of total RNA were treated with RQ1-RNase-free DNase (Promega, USA) to eliminate contamination of genomic DNA. The first strand cDNA were synthesized from 1.5 µg of DNase-treated total RNA using an ImProm- II^TM^ Reverse Transcription System Kit (Promega, USA) according to manufacturer’s instruction. Concentration and quality of the first strand cDNA were examined by Nanodrop UV (OD_260_/OD_280_). The cDNA of individual ovaries of black tiger shrimp from different time points were used as template. Each reaction was performed in a total volume of 20 µL containing 2x iQ™SYBR® Green Supermix (Bio-RAD, USA), 0.2 µg of cDNA template, 0.2 µM of primer pair. Cycling parameters were 95 °C for 2 min 30 s, followed by 40 cycles of 95 °C for 30 s, 58 °C for 20 s and 72 °C for 30 s. To ensure specificity of PCR products, melting curve analysis was performed from 55 °C to 95 °C with continuous fluorescence reading every 0.5 °C increment. The housekeeping gene *Elongation factor 1 alpha* (*EF-1α*) was used as an internal control^60^. All qPCRs were performed in three biological replications (*n* = *3*).

**Table 2:** Primers used in quantitative real-time PCR (qPCR) analysis.

Gene	ID	Primer sequence (5′-3′)	Size	R^2^	Efficiency
*NADH dehydrogenase I*	*NADH*	F: TAC TCT GTA GTT CGT GGA TGT GA R: CGT GAA AAG AGC AAT GAA AGG TGG	191	0.99	99.3
*Cytochrome C oxidase*	*COX*	F: CGA AAG AGG AGT GGG AAC TGG AT R: TGA TGA GAC CCC TGC TAA ATG TAA	118	0.99	100.6
*Innexin2*	*Inx2*	F: GGC ATC GCC AAC CCC AAC ATC CA R: ACC AGC ATC TTT ACC TTA CCA CC	164	0.99	99.5
*Calmodulin*	*Calm*	F: GCA CCA TCA CCA CCA AGG AAC TG R: TTA CCG TCA GCG TCC ACC TCG TT	105	1.00	97.5
*Calcineurin-binding protein cabin-1-like*	*CABIN1*	F: TTG ACA ACT CTG GCT CAC TAT CCG R: ACA CTG AAC TGT GAC ATG CCT TGG	157	1.00	98.0
*Vitellogenin*	*VTG*	F: ATT CGG AAC GTG CAT TTG CTG CA R: GTT CTC AAG CAT TGT GAC AGG ATT	188	0.99	98.9
*Vitellogenin receptor* *Progestin membrane receptor component 1*	*VTGR* *PGMRC1*	F: GAGTTCACCTGCAGCAACAA R: ACAGTCCTCGTCTTCGCCTA F: TCC TGG GCT CAC TAC TCA AA R: CCC ATG CCA TCA TAC TGC TT	243 199	0.99 0.99	95.2 97.5
*Mitogen-activated protein kinase kinase*	*MAPKK*	F: CAT TGT CTT GCA CAC CCC AAC R: TGG CAA TAT TTG AGG TGC TG	215	0.99	95.8
*Elongation factor 1-alpha*	*EF1a*	F: TTC CGA CTC CAA GAA CGA CC R: GAG CAG TGT GGC AAT CAA GC	122	0.99	98.0

For construction of the standard curve for each transcript, a plasmid containing the transcript was constructed by cloning the PCR product of the transcript into a pGEM-T easy vector (Promega, USA). The resulting vector was transformed into *E*. *coli* JM109. The plasmid was extracted and used as the template for construction of the standard curve by 10-fold serial dilutions (10^3^–10^8^ copy numbers).

Expression levels between feed groups were statistically tested by t-test (p < 0.05), and those among different time points post ablation of the same feed group were statistically tested by ANOVA followed by Duncan method (p < 0.05).

## Supplementary information

Supplementary Information.
