# Author Correction: Transcriptome analyses reveal the synergistic effects of feeding and eyestalk ablation on ovarian maturation in black tiger shrimp

**DOI:** 10.1038/s41598-020-62221-6

**Published:** 2020-03-20

**Authors:** Kanchana Sittikankaew, Wirulda Pootakham, Chutima Sonthirod, Duangjai Sangsrakru, Thippawan Yoocha, Jutatip Khudet, Intawat Nookaew, Umaporn Uawisetwathana, Wanilada Rungrassamee, Nitsara Karoonuthaisiri

**Affiliations:** 1 National Center for Genetic Engineering and Biotechnology (BIOTEC), National Science and Technology Development Agency, Pathum Thani, 12120 Thailand; 2 National Omics Center, National Center for Genetic Engineering and Biotechnology (BIOTEC), National Science and Technology Development Agency, Pathum Thani, Thailand; 3 Department Biomedical Informatics, University of Arkansas for Medical Sciences, Little Rock, AR 72205 USA

Correction to: *Scientific Reports* 10.1038/s41598-020-60192-2, published online 24 February 2020

The original version of this Article contained a typographical error in the spelling of the author Intawat Nookaew, which was incorrectly given as Intawat Nookeaw. This has now been corrected in the PDF and HTML versions of the Article, and in the accompanying Supplementary Information file.

