# Supplementary material for: Transcriptome analyses reveal the synergistic effects of feeding and eyestalk ablation on ovarian maturation in black tiger shrimp
Source: Sci Rep. 2020 Feb 24;10:3239. doi: 10.1038/s41598-020-60192-2 (PMC7040003; doi:10.1038/s41598-020-60192-2)
Supplement: Supplementary file 1 — Supplementary Information. [file 41598_2020_60192_MOESM1_ESM.pdf]

# Title Page for Supplementary Information

## Transcriptome analyses reveal the synergistic effects of feeding and eyestalk ablation on ovarian maturation in black tiger shrimp

Kanchana Sittikankaew[a,1], Wirulda Pootakham[a,2], Chutima Sonthirod[2], Duangjai Sangsrakru[2], Thippawan Yoocha[2], Jutatip Khudet[1], Intawat Nookaew[3], Umaporn Uawisetwathana[1], Wanilada Rungrassamee[1], Nitsara Karoonuthaisiri[1]*

*[1]National Center for Genetic Engineering and Biotechnology (BIOTEC), National Science and Technology Development Agency, Pathum Thani 12120, Thailand*

*[2]National Omics Center, National Center for Genetic Engineering and Biotechnology (BIOTEC), National Science and Technology Development Agency, Pathum Thani, Thailand*

*[3]College of Medicine, Department Biomedical Informatics, University of Arkansas for Medical Sciences, Little Rock, AR 72205, USA*

[a]Contributing equally

*Corresponding author

Tel.: +66-2-5646700 ext 3255 Fax: +66-2-5646707

Email: nitsara@alumni.stanford.edu

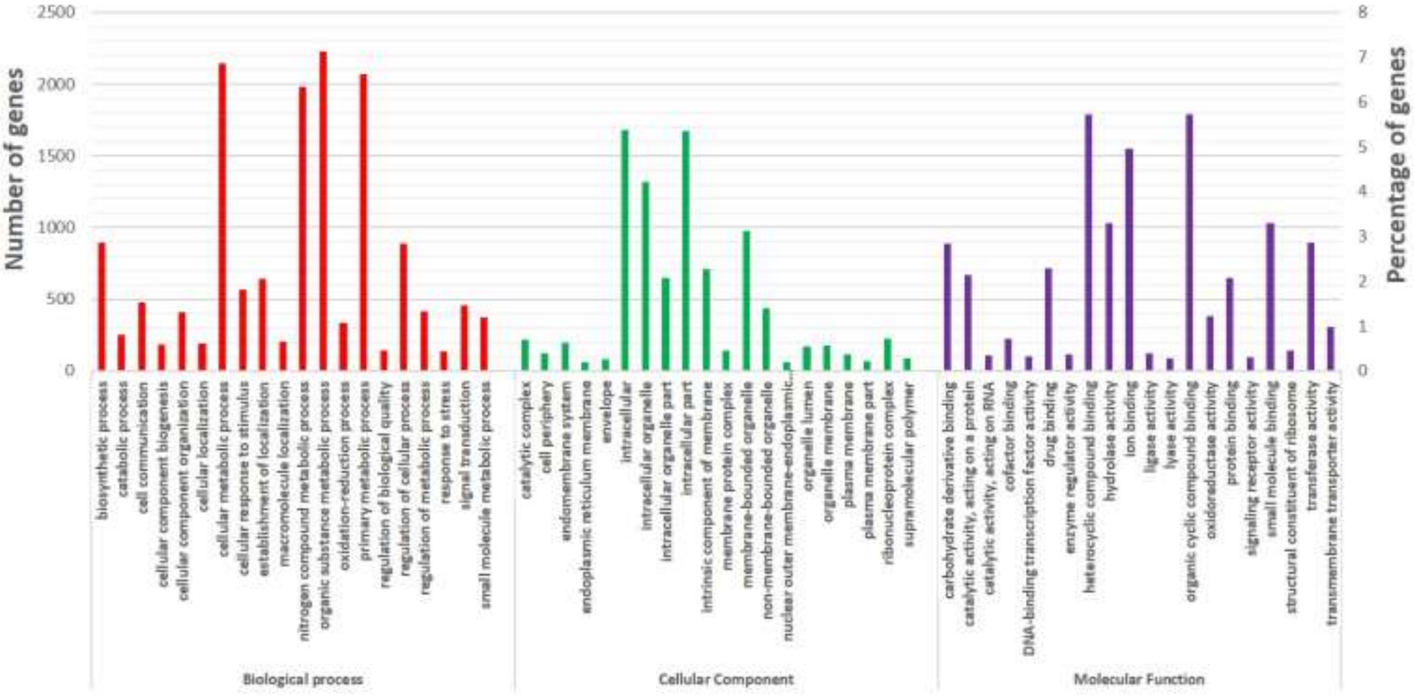


**Supplementary Figure S1. Gene Ontology (GO) annotation of black tiger shrimp genes in the ovarian transcriptome assembly. Results are summarized in three main categories: cellular components, molecular functions and biological processes.**

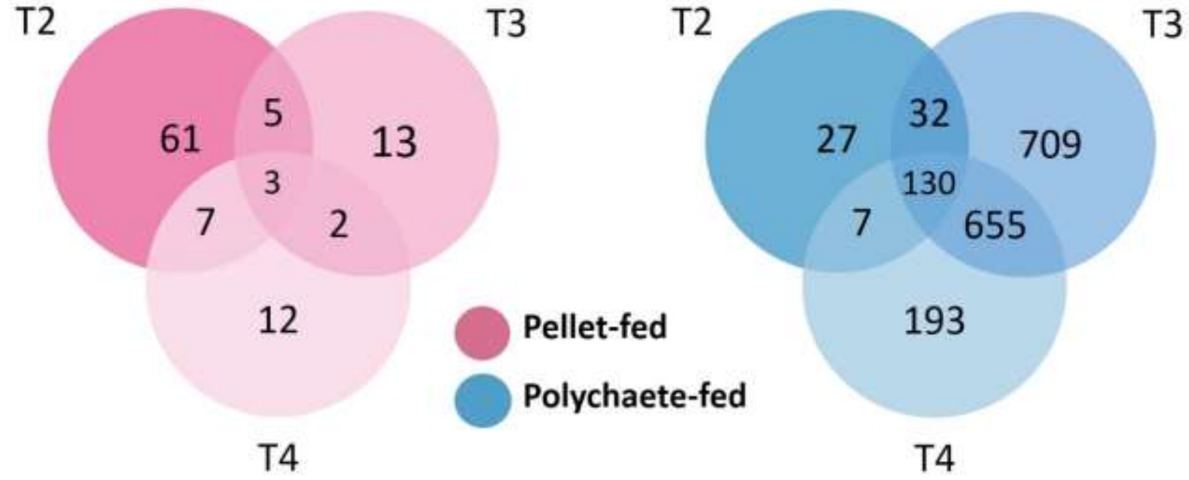


**Supplementary Figure S2. Venn diagram of DEGs of the pellet-fed shrimp and the polychaete-fed shrimp after eyestalk ablation.**

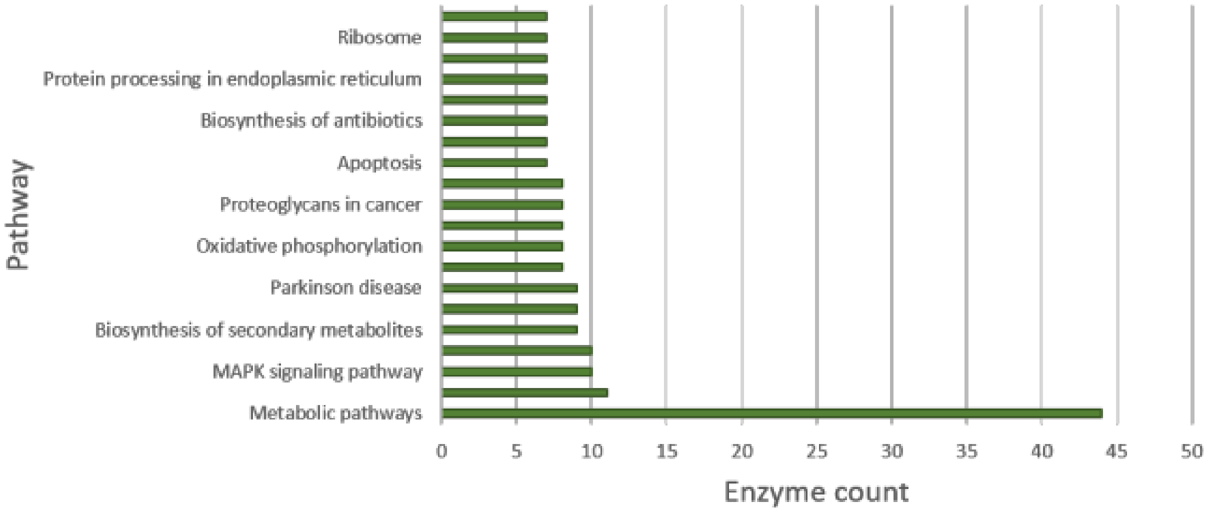


**Supplementary Figure S3. Pathway enrichment analysis. KEGG Automatic Annotation Server (KAAS) program was used to associate nucleotide sequences from 442 features to KEGG (Kyoto Encyclopaedia of Genes and Genomes) Orthology database. KO assignments were subsequently mapped to the pathways.**

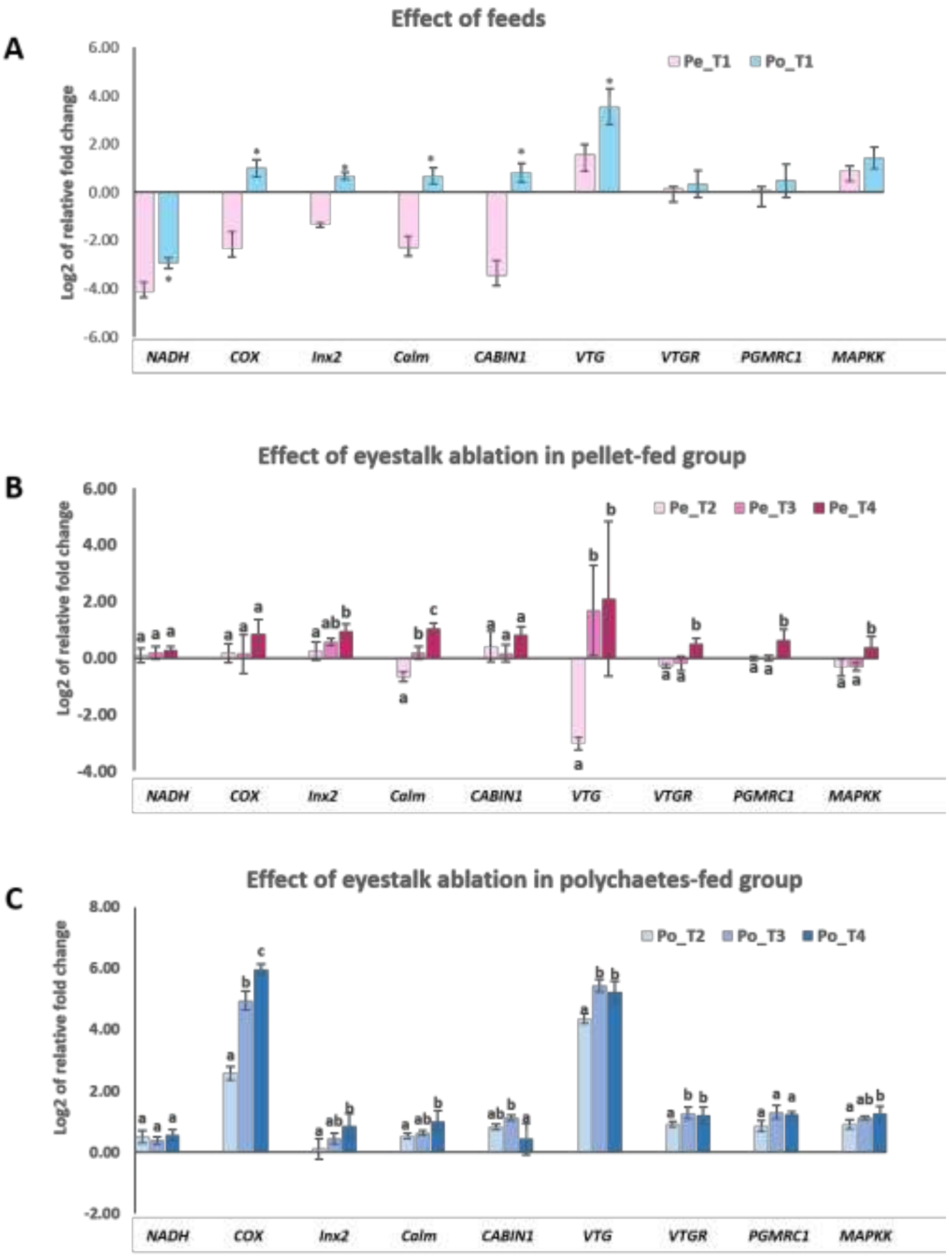


Supplementary Figure S4. Quantitative real-time PCR (qPCR) analysis. The relative expression levels of the selected nine transcripts in ovaries (A) after the 4-week feeding trial (T1) and after eyestalk ablation at Days 1, 4, and 9 (T2, T3, and T4, respectively; n = 3 each) in (B) pellet-fed group and (C) polychaete-fed group were measured using RT-qPCR to confirm RNA sequencing results. Asterisks indicate significant differences between the two feed groups at the same time point ($p<0.05$, t-test). Different letters above each bar indicate significant difference ($P<0.05$; Duncan test).

**Supplementary Table S1**. A list of samples used in the experiment with the GSI. Samples used for Illumina RNAseq experiment are highlighted in blue. Asterisks indicate samples used for PacBio Iso-seq experiment.

| | | Pellet-fed group | | | | Polychaete-fed group | | | |
|---|---|---|---|---|---|---|---|---|---|
| | T0 (N=14) | T1 (N=10) | T2 (N=10) | T3 (N=10) | T4 (N=10) | T1 (N=10) | T2 (N=7) | T3 (N=7) | T4 (N=3) |
| | 0.32 | 0.29 | 0.44 | 0.36 | 1.21 | 0.78* | 1.02 | 4.82 | 6.28* |
| | 0.74 | 0.50 | 0.93 | 0.52 | 0.42 | 0.74 | 2.51 | 2.56 | 6.15 |
| | 0.37 | 0.77 | 0.60 | 0.49 | 0.54 | 0.80 | 1.07* | 4.90 | 5.21 |
| | 0.89 | 0.44 | 0.31 | 0.47 | 1.30 | 0.48 | 0.85 | 4.42* | |
| | 0.56 | 0.55* | 0.79 | 0.53* | 0.43 | 0.61 | 0.92 | 2.55 | |
| | 0.73 | 0.47 | 0.90 | 0.39 | 0.58 | 0.47 | 1.28 | 2.29 | |
| | 0.42 | 0.92 | 0.63* | 0.46 | 0.79* | 0.66 | 1.15 | 3.73 | |
| | 0.56 | 0.46 | 0.54 | 0.60 | 0.73 | 0.70 | | | |
| | 0.92 | 0.48 | 0.54 | 0.55 | 0.76 | 0.72 | | | |
| | 0.52* | 0.53 | 0.45 | 0.39 | 1.03 | 1.21 | | | |
| | 0.52 | | | | | | | | |
| | 0.53 | | | | | | | | |
| | 0.56* | | | | | | | | |
| | 0.38 | | | | | | | | |
| AVERAGE | 0.57 | 0.54 | 0.61 | 0.48 | 0.78 | 0.72 | 1.26 | 3.61 | 5.88 |
| STDV | 0.18 | 0.18 | 0.20 | 0.08 | 0.31 | 0.21 | 0.57 | 1.14 | 0.58 |

**Supplementary Table S2**. Summary of the statistics on Illumina sequence data.
Pe represents pellet-fed group. Po represents polychaete-fed group.

| Sample | Total bases | Read count | Q20 (%) | Clean read count | Mapping read (%) |
|---|---|---|---|---|---|
| Pe_T0 | 8,523,313,082 | 56,445,782 | 98.41 | 56,177,800 | 95.84 |
| Pe_T0 | 7,905,624,026 | 52,355,126 | 98.23 | 52,034,970 | 95.35 |
| Pe_T1 | 8,496,045,200 | 56,265,200 | 98.49 | 55,924,104 | 95.67 |
| Pe_T1 | 9,946,635,156 | 65,871,756 | 98.24 | 65,537,020 | 94.98 |
| Pe_T2 | 7,623,150,742 | 50,484,442 | 98.18 | 50,102,776 | 94.97 |
| Pe_T2 | 8,290,180,558 | 54,901,858 | 98.08 | 54,476,192 | 95.52 |
| Pe_T3 | 6,454,357,590 | 42,744,090 | 97.69 | 41,924,396 | 96.16 |
| Pe_T3 | 8,054,805,684 | 53,343,084 | 98.38 | 53,050,638 | 95.62 |
| Pe_T4 | 9,290,801,990 | 61,528,490 | 98.34 | 61,179,018 | 95.16 |
| Pe_T4 | 8,783,586,950 | 58,169,450 | 98.25 | 57,839,808 | 95.52 |
| Po_T0 | 10,298,164,666 | 68,199,766 | 98.39 | 67,892,656 | 96.68 |
| Po_T0 | 7,799,762,154 | 51,654,054 | 98.21 | 51,276,902 | 95.29 |
| Po_T1 | 6,632,188,478 | 43,921,778 | 97.98 | 43,516,636 | 95.21 |
| Po_T1 | 8,114,662,990 | 53,739,490 | 98.41 | 53,424,898 | 95.25 |
| Po_T2 | 8,710,044,816 | 57,682,416 | 98.41 | 57,311,742 | 95.28 |
| Po_T2 | 8,790,015,624 | 58,212,024 | 98.42 | 57,803,204 | 96.09 |
| Po_T3 | 9,187,214,782 | 60,842,482 | 98.48 | 60,409,642 | 96.2 |
| Po_T3 | 8,407,414,542 | 55,678,242 | 98.4 | 55,294,100 | 96.12 |
| Po_T3 | 9,164,506,194 | 60,692,094 | 98.47 | 60,249,850 | 96.41 |
| Po_T3 | 8,868,687,832 | 58,733,032 | 98.37 | 58,321,262 | 96.09 |
| Po_T4 | 9,146,371,396 | 60,571,996 | 98.43 | 60,099,450 | 95.86 |
| Po_T4 | 9,434,934,510 | 62,483,010 | 98.34 | 61,962,670 | 95.88 |

**Supplementary Table S3.** Summary of the statistics on PacBio Iso-seq sequence data.

| Total bases | Subread count | CCS read count | Number of full-length high quality |
|---|---|---|---|
| 2,694,522,732 | 1,517,489 | 78,546 | 17,428 |
| 2,390,558,693 | 1,569,016 | 75,991 | 18,108 |
| 2,431,784,447 | 1,642,271 | 74,711 | 17,698 |
| 2,237,846,965 | 935,857 | 63,155 | 13,341 |
| 2,482,025,965 | 1,045,998 | 66,494 | 15,454 |
| 2,312,630,450 | 1,094,140 | 63,865 | 15,947 |
| 2,209,565,319 | 1,028,365 | 63,838 | 16,373 |
| 2,066,566,476 | 856,067 | 59,873 | 12,648 |

**Supplementary Table S4.** Lists of transcript annotations and GO assignments.

| Sequence ID | Function | GO | Enzyme Codes |
|---|---|---|---|
| TRINITY_DN31081_c0_g1_i1_len_13211_186744.p1_type_5prime_partial_len_4359 | XP_018024055.1 dynein heavy chain, cytoplasmic-like | F:GO:0003777; F:GO:0005524; P:GO:0007018; C:GO:0030286 | EC:3.6.1.15 |
| TRINITY_DN30754_c0_g1_i1_len_9018_186754.p1_type_internal_len_3006 | XP_023721689.1E3 SUMO-protein ligase RanBP2 isoform X2 | | |
| TRINITY_DN30266_c0_g1_i2_len_11252_186747.p1_type_complete_len_2864 | EZA58779.1E3 ubiquitin-protein ligase hyd | F:GO:0003723; F:GO:0004842; F:GO:0008270; P:GO:0016567; F:GO:0016740; F:GO:0016874; F:GO:0043130 | |
| TRINITY_DN26131_c0_g1_i1_len_9419_227464.p1_type_complete_len_2717 | ATU31746.1Talin-2-like protein | C:GO:0001726; F:GO:0005200; C:GO:0005856; C:GO:0005925; P:GO:0007016; P:GO:0007155; F:GO:0051015 | |
| TRINITY_DN23255_c0_g1_i1_len_10585_153350.p1_type_complete_len_2706 | XP_023719028.1protein PRRC2C-like isoform X2 | | |
| TRINITY_DN31221_c0_g1_i1_len_8911_186755.p1_type_5prime_partial_len_2662 | XP_023720925.1eIF-2-alpha kinase activator GCN1 | F:GO:0005488; P:GO:0032268; P:GO:0043549 | |
| TRINITY_DN30071_c0_g1_i2_len_8246_186763.p1_type_complete_len_2633 | XP_013417435.1serine/threonine-protein kinase ATR-like isoform X2 | F:GO:0000166; F:GO:0004674; F:GO:0005524; P:GO:0006468; F:GO:0016301; P:GO:0016310; F:GO:0016740 | |
| TRINITY_DN31226_c0_g1_i6_len_7893_186771.p1_type_3prime_partial_len_2574 | XP_022240236.1zinc finger protein 423-like isoform X2 | | |
| TRINITY_DN26234_c0_g1_i1_len_7690_227490.p1_type_5prime_partial_len_2507 | XP_022251192.1serine-protein kinase ATM-like | | |
| TRINITY_DN31095_c0_g1_i1_len_7906_186769.p1_type_5prime_partial_len_2492 | XP_015115877.1 transformation/transcription domain-associated protein | | |
| TRINITY_DN30346_c0_g1_i3_len_7173_186800.p1_type_complete_len_1832 | XP_022195576.1transcriptional regulator ATRX homolog | F:GO:0005524 | |

<table>
<tr><td>TRINITY_DN26173_c1_g1_i1_len_6342_227569.p1_type_5prime_partial_len_1831</td><td>XP_018324759.1Ca(2+)/calmodulin-responsive adenylate cyclase isoform X6</td><td>F:GO:0000166; F:GO:0004016; C:GO:0005886; P:GO:0006171; C:GO:0016021; P:GO:0035556</td><td>EC:4.6.1.1</td></tr>
<tr><td>TRINITY_DN25996_c0_g1_i1_len_5677_227659.p1_type_complete_len_1821</td><td>---NA---</td><td></td><td></td></tr>
<tr><td>TRINITY_DN30572_c0_g1_i1_len_7013_186815.p1_type_5prime_partial_len_1819</td><td>XP_021933811.1tyrosine-protein phosphatase Lar isoform X4</td><td></td><td></td></tr>
<tr><td>TRINITY_DN26255_c0_g1_i4_len_6027_227608.p1_type_5prime_partial_len_1814</td><td>PNF19985.1hypothetical protein B7P43_G08658</td><td>C:GO:0005634</td><td></td></tr>
<tr><td>TRINITY_DN31162_c0_g1_i1_len_5825_186956.p1_type_complete_len_1813</td><td>XP_013773545.1dedicator of cytokinesis protein 7-like isoform X1</td><td></td><td></td></tr>
<tr><td>TRINITY_DN31029_c0_g1_i1_len_6701_186834.p1_type_5prime_partial_len_1808</td><td>XP_023708386.1leucine-rich repeat serine/threonine-protein kinase 1</td><td></td><td></td></tr>
<tr><td>TRINITY_DN25764_c0_g1_i1_len_5656_227662.p1_type_complete_len_1807</td><td>XP_023705642.1dynamin-binding protein-like isoform X2</td><td></td><td></td></tr>
<tr><td>TRINITY_DN30491_c0_g1_i2_len_6140_186916.p1_type_complete_len_1804</td><td>XP_013775973.1gamma-tubulin complex component 6-like isoform X1</td><td></td><td></td></tr>
<tr><td>TRINITY_DN26257_c0_g1_i3_len_6052_227605.p1_type_5prime_partial_len_1795</td><td>XP_021917974.1regulator of G-protein signaling loco isoform X3</td><td></td><td></td></tr>
<tr><td>1273|_|c751_f10p17_3058_isoform_c751_full_length_coverage_10_non_full_length_coverage_17_isoform_length_3058_25189</td><td>XP_018024135.1 sodium- and chloride-dependent glycine transporter 1-like</td><td></td><td></td></tr>
<tr><td>TRINITY_DN31151_c0_g1_i1_len_6440_186868.p1_type_5prime_partial_len_1019</td><td>XP_022193060.1multiple C2 and transmembrane domain-containing protein 1 isoform X1</td><td>C:GO:0016021</td><td></td></tr>
<tr><td>TRINITY_DN29449_c0_g1_i1_len_4098_187531.p1_type_complete_len_1019</td><td>XP_022913358.1immunoglobulin domain and leucine-rich repeat-containing protein 2</td><td></td><td></td></tr>
<tr><td>1278|_|c19764_f2p10_3056_isoform_c19764_full_length_coverage_2_non_full_length_coverage_10_isoform_length_3056_28971</td><td>XP_023319773.1histone-lysine N-methyltransferase 2D-like</td><td></td><td></td></tr>
</table>

<table>
<tr><td>1279|_|c2324_f4p25_3056_isoform_c2324_full_length_coverage_4_non_full_length_coverage_25_isoform_length_3056_25647</td><td>XP_026286490.1rab proteins geranylgeranyltransferase component A 2</td><td></td><td></td></tr>
<tr><td>1281|_|c19801_f2p8_3055_isoform_c19801_full_length_coverage_2_non_full_length_coverage_8_isoform_length_3055_28990</td><td>XP_006784301.1 ARF GTPase-activating protein GIT2-like isoform X5</td><td></td><td></td></tr>
<tr><td>1282|_|c2437_f2p5_3054_isoform_c2437_full_length_coverage_2_non_full_length_coverage_5_isoform_length_3054_25652</td><td>XP_008200143.1 ornithine decarboxylase 1</td><td>F:GO:0004586; C:GO:0005737; P:GO:0033387</td><td>EC:4.1.1.17</td></tr>
<tr><td>TRINITY_DN23929_c0_g1_i1_len_3091_122071.p1_type_5prime_partial_len_1018</td><td>XP_019644407.1 MMS19 nucleotide excision repair protein homolog</td><td>P:GO:0097428</td><td></td></tr>
<tr><td>1285|_|c1454_f3p3_3053_isoform_c1454_full_length_coverage_3_non_full_length_coverage_3_isoform_length_3053_27788</td><td>XP_011349355.1 N-alpha-acetyltransferase 30 isoform X2</td><td></td><td></td></tr>
<tr><td>1287|_|c2101_f3p4_3052_isoform_c2101_full_length_coverage_3_non_full_length_coverage_4_isoform_length_3052_28220</td><td>XP_026289728.1exocyst complex component 6 isoform X2</td><td></td><td></td></tr>
<tr><td>TRINITY_DN20792_c1_g1_i1_len_775_5051.p1_type_5prime_partial_len_237</td><td>ADF58427.1histone H3</td><td>F:GO:0005488; C:GO:0005694</td><td></td></tr>
<tr><td>TRINITY_DN15706_c0_g1_i1_len_934_33870.p1_type_5prime_partial_len_237</td><td>XP_019539967.1 coiled-coil domain-containing protein 25</td><td></td><td></td></tr>
<tr><td>TRINITY_DN8171_c0_g1_i1_len_838_162035.p1_type_5prime_partial_len_237</td><td>---NA---</td><td></td><td></td></tr>
<tr><td>TRINITY_DN4431_c0_g1_i1_len_1014_95513.p2_type_complete_len_237</td><td>---NA---</td><td></td><td></td></tr>
<tr><td>TRINITY_DN32143_c0_g1_i1_len_878_127396.p1_type_5prime_partial_len_237</td><td>XP_018578511.2tyrosine-protein phosphatase 10D</td><td>F:GO:0004725; C:GO:0016021; P:GO:0035335</td><td>EC:3.1.3.16; EC:3.1.3.48</td></tr>
<tr><td>TRINITY_DN31270_c0_g1_i2_len_1140_195381.p1_type_complete_len_237</td><td>---NA---</td><td></td><td></td></tr>
<tr><td>TRINITY_DN10868_c0_g1_i1_len_714_238999.p1_type_internal_len_238</td><td>---NA---</td><td></td><td></td></tr>
<tr><td>TRINITY_DN9768_c0_g1_i1_len_715_238968.p1_type_internal_len_238</td><td>---NA---</td><td></td><td></td></tr>
</table>

| | | | |
|---|---|---|---|
| TRINITY_DN19693_c0_g1_i1_len_714_128852.p1_type_internal_len_238 | ---NA--- | | |
| TRINITY_DN18583_c0_g1_i1_len_714_238992.p1_type_internal_len_238 | ---NA--- | | |
| TRINITY_DN10839_c0_g1_i1_len_845_237493.p1_type_5prime_partial_len_237 | ---NA--- | | |
| 11905\|_\|c52157_f3p2_709_isoform_c52157_full_length_coverage_3_non_full_length_coverage_2_isoform_length_709_6520 | XP_018021730.1 prefoldin subunit 1-like | P:GO:0006457; C:GO:0016272; F:GO:0051082 | |
| TRINITY_DN23275_c0_g1_i1_len_2877_122195.p2_type_complete_len_236 | XP_015212255.1 tigger transposable element-derived protein 1-like | | |
| TRINITY_DN18986_c0_g1_i1_len_791_238050.p1_type_5prime_partial_len_236 | XP_025104775.1tRNA 2'-phosphotransferase 1-like isoform X1 | | |
| TRINITY_DN25485_c0_g1_i1_len_2780_229312.p2_type_complete_len_236 | XP_022916486.1putative inorganic phosphate cotransporter | | |
| TRINITY_DN23466_c0_g1_i1_len_1838_191523.p1_type_complete_len_236 | XP_018011110.1 desumoylating isopeptidase 2-like | | |
| TRINITY_DN22532_c0_g1_i1_len_3915_153718.p2_type_complete_len_236 | XP_023334201.1transmembrane protein 186-like | C:GO:0016020; C:GO:0016021 | |
| TRINITY_DN17669_c0_g1_i1_len_2044_230982.p1_type_complete_len_236 | AIT97136.1innexin 4 | C:GO:0005886; C:GO:0005921; P:GO:0006811; C:GO:0016021 | |
| TRINITY_DN20137_c0_g1_i1_len_1043_298572.p1_type_5prime_partial_len_236 | AXR98459.1galactose-specific lectin nattectin-like protein | F:GO:0030246 | |
| TRINITY_DN15686_c0_g1_i1_len_710_199839.p1_type_internal_len_237 | XP_018011235.1 mucin-22-like isoform X3 | | |
| TRINITY_DN8082_c0_g1_i1_len_712_239020.p1_type_internal_len_237 | XP_014681730.1 major facilitator superfamily domain-containing protein 10-like | C:GO:0016020 | |
| TRINITY_DN6100_c0_g1_i1_len_841_237538.p1_type_5prime_partial_len_236 | ---NA--- | | |
| TRINITY_DN12017_c0_g1_i1_len_711_239034.p1_type_internal_len_237 | ---NA--- | | |

| | | | |
|---|---|---|---|
| TRINITY_DN19621_c0_g1_i1_len_708_239073.p1_type_internal_len_237 | KFM65941.1N-acetyllactosaminide beta-1,3-N-acetylglucosaminyltransferase | F:GO:0016740; F:GO:0016757 | |
| TRINITY_DN16270_c0_g1_i1_len_736_300736.p1_type_5prime_partial_len_236 | XP_023349348.1uncharacterized protein LOC111718083 | | |
| TRINITY_DN24330_c0_g1_i4_len_988_236214.p1_type_3prime_partial_len_237 | ---NA--- | | |
| TRINITY_DN14966_c0_g1_i2_len_861_237328.p1_type_3prime_partial_len_237 | ---NA--- | | |
| TRINITY_DN23459_c1_g1_i2_len_1459_233227.p1_type_5prime_partial_len_236 | ---NA--- | | |
| TRINITY_DN25582_c0_g1_i2_len_710_199833.p1_type_internal_len_237 | KRT83353.1phosphotransferase | F:GO:0016740 | |
| TRINITY_DN36756_c0_g1_i1_len_709_239063.p1_type_internal_len_237 | ---NA--- | | |
| TRINITY_DN33374_c0_g1_i1_len_866_197858.p1_type_5prime_partial_len_236 | ---NA--- | | |
| TRINITY_DN22583_c0_g1_i1_len_1222_94389.p1_type_5prime_partial_len_236 | XP_018012155.1PREDICTED: uncharacterized protein LOC108669355 | | |
| TRINITY_DN10426_c0_g1_i2_len_711_5626.p1_type_internal_len_237 | ---NA--- | | |
| TRINITY_DN35200_c0_g1_i1_len_712_199815.p1_type_internal_len_237 | ---NA--- | | |
| TRINITY_DN23915_c0_g1_i1_len_837_198183.p1_type_3prime_partial_len_237 | ---NA--- | | |
| TRINITY_DN31326_c0_g1_i1_len_711_65535.p1_type_internal_len_237 | ---NA--- | | |
| TRINITY_DN24505_c0_g1_i1_len_1304_194235.p1_type_5prime_partial_len_236 | AYN72711.1armadillo repeat containing protein 6 | | |
| TRINITY_DN24657_c0_g1_i1_len_838_198166.p1_type_5prime_partial_len_236 | OXA64012.1Tyrosine-protein phosphatase yvh1 | P:GO:0000209; F:GO:0061630 | |
| TRINITY_DN17217_c0_g1_i4_len_1765_156795.p2_type_5prime_partial_len_236 | ESN89823.1hypothetical protein HELRODRAFT_194786 | P:GO:0006486; F:GO:0016758 | |

| | | | |
|---|---|---|---|
| TRINITY_DN146_c0_g1_i1_len_756_238455.p1_type_3prime_partial_len_237 | ---NA--- | | |
| TRINITY_DN26755_c0_g1_i2_len_2100_190550.p3_type_complete_len_235 | ---NA--- | | |
| TRINITY_DN15951_c0_g1_i1_len_756_199181.p1_type_3prime_partial_len_236 | XP_018026417.1 spermatogenesis-associated protein 20-like | F:GO:0003824 | |
| TRINITY_DN21608_c0_g1_i1_len_753_199240.p1_type_5prime_partial_len_235 | AKG54423.1interferon regulatory factor | F:GO:0044212 | |
| TRINITY_DN20090_c1_g1_i1_len_707_239093.p1_type_internal_len_236 | XP_018013305.1 solute carrier family 13 member 5-like | P:GO:0006810; C:GO:0016020 | |
| TRINITY_DN18742_c0_g1_i1_len_871_197796.p1_type_3prime_partial_len_236 | PNF31518.1E3 ubiquitin-protein ligase KCMF1 | F:GO:0003676; F:GO:0008270; P:GO:0016567; F:GO:0016874; F:GO:0061630 | |
| TRINITY_DN286_c0_g1_i1_len_871_197792.p1_type_5prime_partial_len_235 | XP_018022669.1 RNA-binding protein cabeza-like isoform X3 | | |
| TRINITY_DN24441_c0_g1_i2_len_1347_61279.p1_type_complete_len_235 | XP_020783417.1peroxisomal membrane protein 11C | C:GO:0005779; C:GO:0016020; C:GO:0016021; P:GO:0016559 | |
| TRINITY_DN13175_c0_g1_i1_len_707_239083.p1_type_internal_len_236 | PIK35125.1hypothetical protein BSL78_28051 | F:GO:0003676; F:GO:0003700; P:GO:0006355; F:GO:0008270; P:GO:0015074 | |
| TRINITY_DN5067_c0_g1_i1_len_707_239087.p1_type_internal_len_236 | XP_023704757.11-phosphatidylinositol 4,5-bisphosphate phosphodiesterase epsilon-1-like isoform X2 | F:GO:0005085; P:GO:0007264; P:GO:0035556 | |
| TRINITY_DN28568_c0_g1_i1_len_2455_189564.p2_type_complete_len_235 | XP_018026073.1 regulator of chromosome condensation-like | | |
| TRINITY_DN33850_c0_g1_i1_len_708_199862.p1_type_internal_len_236 | XP_021913638.1PAX-interacting protein 1-like isoform X2 | | |
| TRINITY_DN6609_c0_g1_i1_len_1040_196191.p1_type_5prime_partial_len_235 | XP_018008674.1 palmitoyltransferase ZDHHC22-like | C:GO:0016020; C:GO:0016021; F:GO:0016740; F:GO:0016746; F:GO:0019706 | |
| TRINITY_DN6071_c0_g1_i1_len_818_271596.p1_type_5prime_partial_len_235 | ---NA--- | | |

| | | | |
|---|---|---|---|
| TRINITY_DN40724_c0_g1_i1_len_709_199859.p1_type_internal_len_236 | ---NA--- | | |
| TRINITY_DN2101_c0_g1_i1_len_709_199856.p1_type_internal_len_236 | ---NA--- | | |
| TRINITY_DN14244_c0_g1_i1_len_707_128953.p1_type_internal_len_236 | ANA78437.1dopamine beta-hydroxylase | F:GO:0005507; C:GO:0016021; F:GO:0016715; P:GO:0055114 | EC:1.14.17 |
| TRINITY_DN1197_c0_g1_i1_len_931_236695.p1_type_3prime_partial_len_236 | XP_014669173.1 serine/threonine-protein kinase unc-51-like | F:GO:0004674; F:GO:0005524; P:GO:0006468 | EC:2.7.11 |
| TRINITY_DN32937_c0_g1_i1_len_708_239070.p1_type_internal_len_236 | XP_023717764.1S phase cyclin A-associated protein in the endoplasmic reticulum | | |
| TRINITY_DN22247_c0_g1_i1_len_705_199913.p1_type_internal_len_236 | ---NA--- | | |
| TRINITY_DN20345_c0_g1_i1_len_878_63931.p1_type_5prime_partial_len_235 | ---NA--- | | |
| TRINITY_DN16279_c0_g1_i1_len_705_199906.p1_type_internal_len_236 | XP_014353888.1 E3 ubiquitin-protein ligase SHPRH-like | | |
| TRINITY_DN19664_c0_g1_i1_len_707_199873.p1_type_internal_len_236 | ---NA--- | | |
| TRINITY_DN19358_c0_g1_i1_len_1261_234292.p1_type_5prime_partial_len_235 | KDR22217.1Calcium and integrin-binding protein 1 | F:GO:0005509; P:GO:0007229 | |
| TRINITY_DN1655_c0_g1_i1_len_957_196931.p1_type_5prime_partial_len_235 | XP_018021156.1 netrin receptor UNC5C-like | F:GO:0005042; P:GO:0007165; C:GO:0016020; C:GO:0016021; P:GO:0038007 | |
| TRINITY_DN9913_c0_g1_i1_len_708_239082.p1_type_internal_len_236 | XP_015110986.1 kinesin-like protein unc-104 isoform X5 | | |
| TRINITY_DN19398_c0_g1_i2_len_2214_553.p1_type_5prime_partial_len_235 | XP_018009727.1 ubiquitin-conjugating enzyme E2 variant 2-like | | |
| TRINITY_DN7723_c0_g1_i1_len_707_5666.p1_type_internal_len_236 | XP_018010562.1 vacuolar protein sorting-associated protein 13D-like | | |
| TRINITY_DN24772_c0_g1_i2_len_1445_233294.p1_type_5prime_partial_len_235 | ---NA--- | | |

| | | | |
|---|---|---|---|
| TRINITY_DN35149_c0_g1_i1_len_785_198790.p1_type_5prime_partial_len_234 | XP_026280478.1male-specific lethal 1-like 1 isoform X2 | P:GO:0043984; C:GO:0072487 | |
| TRINITY_DN26431_c0_g1_i1_len_916_197325.p1_type_complete_len_234 | KZS21212.1putative CDP-diacylglycerol--inositol 3-phosphatidyltransferase | P:GO:0008654; C:GO:0016020; C:GO:0016021; F:GO:0016740; F:GO:0016780 | |
| TRINITY_DN24749_c0_g1_i3_len_789_198745.p1_type_3prime_partial_len_235 | ---NA--- | | |
| TRINITY_DN9898_c0_g1_i2_len_986_270291.p1_type_complete_len_234 | ---NA--- | | |
| TRINITY_DN4158_c0_g1_i1_len_706_239109.p1_type_internal_len_235 | ---NA--- | | |
| TRINITY_DN9608_c0_g1_i1_len_704_65617.p1_type_internal_len_235 | ---NA--- | | |
| TRINITY_DN17033_c0_g1_i1_len_704_65616.p1_type_internal_len_235 | ---NA--- | | |
| TRINITY_DN25131_c0_g1_i2_len_2032_231025.p1_type_5prime_partial_len_234 | ---NA--- | | |
| TRINITY_DN20927_c1_g2_i2_len_949_196994.p1_type_complete_len_234 | PRD20449.1zinc finger protein | F:GO:0003676 | |
| TRINITY_DN12412_c0_g1_i1_len_703_199941.p1_type_3prime_partial_len_235 | XP_018021333.1PREDICTED: putative uncharacterized protein DDB_G0282133 | | |
| TRINITY_DN13887_c0_g1_i1_len_939_236621.p1_type_complete_len_234 | XP_018025298.1 synaptophysin-like | C:GO:0008021; C:GO:0016020; C:GO:0016021 | |
| TRINITY_DN16016_c0_g1_i1_len_865_197862.p1_type_3prime_partial_len_235 | XP_018013305.1 solute carrier family 13 member 5-like | P:GO:0006810; C:GO:0016020 | |
| TRINITY_DN33216_c0_g1_i1_len_725_199641.p1_type_5prime_partial_len_234 | ---NA--- | | |
| TRINITY_DN14336_c0_g1_i1_len_1627_232423.p1_type_complete_len_234 | EEN56807.1hypothetical protein BRAFLDRAFT_95082 | C:GO:0000139; F:GO:0003924; F:GO:0005525; C:GO:0005789; P:GO:0006886; P:GO:0006888; P:GO:0032482 | EC:3.6.1.15 |
| TRINITY_DN18397_c0_g1_i4_len_704_5700.p1_type_internal_len_235 | XP_018011797.1 E3 ubiquitin-protein ligase HUWE1-like | | |

| | | | |
|---|---|---|---|
| TRINITY_DN19724_c0_g1_i2_len_733_199540.p1_type_5prime_partial_len_234 | ---NA--- | | |
| TRINITY_DN10279_c0_g1_i1_len_704_35682.p1_type_internal_len_235 | ---NA--- | | |
| TRINITY_DN6930_c1_g1_i1_len_702_199957.p1_type_internal_len_235 | ---NA--- | | |
| TRINITY_DN27550_c0_g1_i1_len_704_301077.p1_type_internal_len_235 | XP_018018253.1 xaa-Pro aminopeptidase 2-like | F:GO:0004177; P:GO:0006508; F:GO:0046872 | |
| TRINITY_DN15883_c0_g1_i1_len_702_129001.p1_type_internal_len_235 | AXR98489.1putative multiple epidermal growth factor-like domains protein 10 | | |
| TRINITY_DN10889_c0_g1_i1_len_704_199917.p1_type_internal_len_235 | ---NA--- | | |
| TRINITY_DN15784_c0_g2_i1_len_705_128961.p1_type_internal_len_235 | ---NA--- | | |
| TRINITY_DN10944_c0_g1_i1_len_910_63699.p1_type_3prime_partial_len_235 | XP_018027633.1 carbohydrate sulfotransferase 1-like | F:GO:0001517; P:GO:0006044; P:GO:0006790; F:GO:0008146; F:GO:0016740 | |
| TRINITY_DN11310_c0_g1_i1_len_905_161453.p1_type_complete_len_234 | AVD68963.1obstructor E2 | C:GO:0005576; P:GO:0006030; F:GO:0008061 | |
| TRINITY_DN21010_c0_g1_i1_len_1732_156917.p1_type_complete_len_234 | ---NA--- | | |
| TRINITY_DN23076_c0_g1_i1_len_1693_60068.p2_type_5prime_partial_len_234 | XP_018011541.1 solute carrier family 52, riboflavin transporter, member 3-B-like | C:GO:0005887; F:GO:0032217; P:GO:0032218 | |
| TRINITY_DN4553_c0_g1_i2_len_790_34867.p2_type_5prime_partial_len_234 | XP_023706910.1neurotrophin 1-like isoform X2 | | |
| TRINITY_DN23137_c0_g1_i1_len_1765_191791.p2_type_complete_len_234 | APO14259.1juvenile hormone esterase-like carboxylesterase 1 | F:GO:0016787 | |
| TRINITY_DN11088_c0_g1_i1_len_1171_195161.p1_type_5prime_partial_len_234 | XP_018026157.1 zinc finger FYVE domain-containing protein 21-like | F:GO:0046872 | |
| TRINITY_DN276_c0_g1_i1_len_704_199933.p1_type_internal_len_235 | ---NA--- | | |
| TRINITY_DN30043_c0_g1_i1_len_1879_191357.p1_type_complete_len_234 | ---NA--- | | |

| | | | |
|---|---|---|---|
| TRINITY_DN22856_c0_g1_i5_len_957_63375.p1_type_5prime_partial_len_234 | KZS21553.1Protein FAM195A | | |
| TRINITY_DN28910_c0_g1_i1_len_3990_187593.p1_type_5prime_partial_len_1017 | AKM12664.1Janus kinase | F:GO:0004715; F:GO:0005524; C:GO:0005856; C:GO:0016020; P:GO:0018108; P:GO:0035556 | EC:2.7.10; EC:2.7.10.2 |
| TRINITY_DN24277_c0_g1_i1_len_3976_228198.p1_type_5prime_partial_len_1017 | KDR16567.1General vesicular transport factor p115 | C:GO:0005737; P:GO:0006810; C:GO:0016020 | |
| TRINITY_DN29638_c0_g1_i3_len_3173_188290.p1_type_5prime_partial_len_1016 | XP_012252457.1multidrug resistance-associated protein 1 isoform X5 | | |
| 1292\|_\|c19823_f2p9_3045_isoform_c19823_full_length_coverage_2_non_full_length_coverage_9_isoform_length_3045_28998 | XP_018020304.1 nuclear pore complex protein Nup155-like isoform X3 | | |
| 1293\|_\|c13633_f3p14_3044_isoform_c13633_full_length_coverage_3_non_full_length_coverage_14_isoform_length_3044_26215 | KDR23609.1Periodic tryptophan protein 2-like protein | | |
| 1294\|_\|c2311_f2p3_3043_isoform_c2311_full_length_coverage_2_non_full_length_coverage_3_isoform_length_3043_25645 | ---NA--- | | |
| 1295\|_\|c12173_f2p7_3042_isoform_c12173_full_length_coverage_2_non_full_length_coverage_7_isoform_length_3042_26159 | ---NA--- | | |
| 1296\|_\|c8131_f2p24_3042_isoform_c8131_full_length_coverage_2_non_full_length_coverage_24_isoform_length_3042_28493 | XP_026288288.1transcriptional repressor p66-alpha isoform X2 | | |
| TRINITY_DN30199_c0_g1_i2_len_3141_188324.p1_type_5prime_partial_len_1013 | ---NA--- | | |
| 1300\|_\|c1829_f5p18_3037_isoform_c1829_full_length_coverage_5_non_full_length_coverage_18_isoform_length_3037_25519 | XP_013780205.1LON peptidase N-terminal domain and RING finger protein 3-like | | |
| TRINITY_DN8227_c0_g1_i1_len_705_239118.p1_type_internal_len_235 | AMO44056.1Na+/K+/2Cl- cotransporter protein | F:GO:0015377; C:GO:0016021; P:GO:1902476 | |
| TRINITY_DN9506_c0_g1_i1_len_704_239138.p1_type_internal_len_235 | PSN32447.1hypothetical protein C0J52_17592, partial | F:GO:0005488 | |

| | | | |
|---|---|---|---|
| TRINITY_DN8416_c0_g1_i1_len_703_239155.p1_type_internal_len_235 | PNF16969.1Regulatory-associated protein of mTOR | P:GO:0031929; C:GO:0031931 | |
| TRINITY_DN21183_c0_g1_i1_len_2049_155976.p2_type_5prime_partial_len_234 | KZS01138.14-coumarate--CoA ligase 3 | F:GO:0003824; C:GO:0016020 | |
| TRINITY_DN29419_c0_g1_i1_len_703_239162.p1_type_internal_len_235 | PNF38744.1putative ATP-dependent RNA helicase YTHDC2 | F:GO:0004386; F:GO:0005524 | EC:3.6.1.15 |
| TRINITY_DN21424_c0_g1_i1_len_820_198377.p1_type_5prime_partial_len_234 | XP_023707647.1transmembrane protein 62-like isoform X2 | C:GO:0016020 | |
| TRINITY_DN14483_c0_g1_i1_len_844_198090.p1_type_5prime_partial_len_234 | ---NA--- | | |
| TRINITY_DN17290_c0_g1_i1_len_1335_93894.p1_type_5prime_partial_len_234 | XP_018014429.1 mitochondrial import inner membrane translocase subunit Tim23-like | C:GO:0016021 | |
| TRINITY_DN10361_c0_g1_i2_len_878_237150.p1_type_3prime_partial_len_234 | PNF31516.1E3 ubiquitin-protein ligase KCMF1 | F:GO:0003676; F:GO:0008270; P:GO:0016567; F:GO:0016874; F:GO:0061630 | |
| TRINITY_DN18014_c0_g1_i2_len_861_299679.p1_type_complete_len_233 | ---NA--- | | |
| TRINITY_DN38760_c0_g1_i1_len_702_199951.p1_type_internal_len_234 | ---NA--- | | |
| TRINITY_DN39580_c0_g1_i1_len_701_199984.p1_type_internal_len_234 | XP_018014164.1 bestrophin-3-like | | |
| TRINITY_DN15222_c0_g1_i1_len_830_198267.p2_type_complete_len_233 | AVD68963.1obstructor E2 | C:GO:0005576; P:GO:0006030; F:GO:0008061 | |
| TRINITY_DN34961_c0_g1_i1_len_701_199967.p1_type_internal_len_234 | XP_013794564.2extracellular matrix protein FRAS1-like | | |
| TRINITY_DN19903_c0_g1_i1_len_699_200000.p1_type_internal_len_234 | ---NA--- | | |
| TRINITY_DN19516_c0_g1_i1_len_871_4347.p1_type_complete_len_233 | ---NA--- | | |
| TRINITY_DN179_c0_g1_i1_len_708_163436.p1_type_5prime_partial_len_233 | AGB51123.1NO-sensitive soluble guanylyl cyclase beta 1 | F:GO:0000166; F:GO:0004383; P:GO:0006182; F:GO:0020037; P:GO:0035556 | EC:4.6.1.2 |

| | | | |
|---|---|---|---|
| TRINITY_DN26467_c0_g1_i1_len_702_163518.p1_type_internal_len_234 | XP_018018254.1 xaa-Pro aminopeptidase 1-like | F:GO:0016787 | |
| TRINITY_DN2575_c0_g1_i1_len_701_163529.p1_type_internal_len_234 | XP_010768590.1 zinc finger FYVE domain-containing protein 1-like | F:GO:0008270 | |
| TRINITY_DN14575_c0_g1_i1_len_758_5181.p1_type_5prime_partial_len_233 | P80363.1RecName: Full=Crustacean calcium-binding protein 23; Short=CCBP-23 | F:GO:0005509 | |
| TRINITY_DN17596_c0_g1_i2_len_1094_235416.p1_type_complete_len_233 | ---NA--- | | |
| TRINITY_DN13588_c0_g1_i1_len_703_239154.p1_type_internal_len_234 | ---NA--- | | |
| TRINITY_DN1888_c0_g1_i1_len_700_239201.p1_type_internal_len_234 | XP_014099915.1 tau-tubulin kinase 2-like | F:GO:0004672; F:GO:0005524; P:GO:0006468 | |
| TRINITY_DN14217_c0_g1_i2_len_1133_235156.p1_type_5prime_partial_len_233 | XP_018027035.1 synaptogyrin-1-like | | |
| TRINITY_DN21995_c0_g1_i1_len_1526_157698.p1_type_5prime_partial_len_233 | ---NA--- | | |
| TRINITY_DN8968_c0_g1_i1_len_701_65664.p1_type_internal_len_234 | ---NA--- | | |
| TRINITY_DN8158_c0_g1_i1_len_701_65662.p1_type_internal_len_234 | CDH61181.1retrotransposon ty3-gypsy subclass | F:GO:0003676; F:GO:0004190; P:GO:0006508; F:GO:0008270; P:GO:0015074 | |
| TRINITY_DN15258_c0_g1_i1_len_703_199938.p1_type_internal_len_234 | XP_013173636.1PREDICTED: uncharacterized protein LOC106122258 | F:GO:0003676; F:GO:0008270 | |
| TRINITY_DN6243_c0_g1_i1_len_701_239182.p1_type_internal_len_234 | XP_022920241.1endoplasmic reticulum-Golgi intermediate compartment protein 2 | C:GO:0016020 | |
| TRINITY_DN18072_c0_g1_i3_len_702_272778.p1_type_internal_len_234 | ---NA--- | | |
| TRINITY_DN33773_c0_g1_i1_len_699_200025.p1_type_internal_len_234 | XP_023727721.1transcription factor cwo isoform X5 | F:GO:0003677; C:GO:0005634; P:GO:0006355; F:GO:0046983 | |
| TRINITY_DN7978_c0_g1_i1_len_701_129015.p1_type_internal_len_234 | XP_014288825.1protein O-linked-mannose beta-1,2-N-acetylglucosaminyltransferase 1 | | |

| | | | |
|---|---|---|---|
| TRINITY_DN37807_c0_g1_i1_len_702_239174.p1_type_internal_len_234 | PNF24359.1Ubiquitin carboxyl-terminal hydrolase 32 | F:GO:0004843; F:GO:0005509; P:GO:0006511; P:GO:0016579 | EC:3.4.19.12 |
| TRINITY_DN6293_c0_g1_i1_len_702_239172.p1_type_internal_len_234 | ---NA--- | | |
| TRINITY_DN27973_c0_g1_i2_len_2555_189321.p2_type_complete_len_233 | ---NA--- | | |
| TRINITY_DN14590_c0_g1_i1_len_821_237724.p1_type_3prime_partial_len_234 | XP_018011394.1 protein wings apart-like | | |
| TRINITY_DN17682_c0_g1_i1_len_778_238215.p1_type_5prime_partial_len_233 | XP_018010841.1PREDICTED: uncharacterized protein LOC108668182 | | |
| TRINITY_DN26052_c0_g1_i2_len_701_239176.p1_type_internal_len_234 | XP_018006612.1 protein bric-a-brac 1-like isoform X4 | | |
| TRINITY_DN29087_c0_g1_i1_len_702_239166.p1_type_internal_len_234 | ---NA--- | | |
| TRINITY_DN33296_c0_g1_i1_len_767_199027.p1_type_5prime_partial_len_233 | ---NA--- | | |
| TRINITY_DN19527_c0_g1_i1_len_884_34189.p1_type_complete_len_232 | ---NA--- | | |
| TRINITY_DN21190_c0_g1_i5_len_696_5788.p1_type_internal_len_233 | XP_013787776.1Fanconi anemia core complex-associated protein 24-like isoform X1 | | |
| TRINITY_DN20508_c0_g2_i1_len_698_239224.p1_type_internal_len_233 | ---NA--- | | |
| TRINITY_DN17226_c0_g1_i1_len_703_163506.p1_type_5prime_partial_len_232 | ---NA--- | | |
| TRINITY_DN21042_c1_g1_i1_len_1389_233563.p1_type_5prime_partial_len_232 | ---NA--- | | |
| TRINITY_DN23699_c0_g1_i1_len_697_301151.p1_type_internal_len_233 | XP_018010484.1 ATP-dependent helicase brm-like | F:GO:0004386; F:GO:0005524; C:GO:0016514; F:GO:0016887; F:GO:0042393; P:GO:0043044 | EC:3.6.1.3; EC:3.6.1.15 |
| TRINITY_DN18648_c0_g1_i1_len_1358_233734.p1_type_5prime_partial_len_232 | ---NA--- | | |

| | | | |
|---|---|---|---|
| TRINITY_DN18455_c0_g1_i1_len_698_200042.p1_type_internal_len_233 | XP_018021126.1 inositol hexakisphosphate and diphosphoinositol-pentakisphosphate kinase-like | F:GO:0000829; F:GO:0000832; F:GO:0005524; C:GO:0005829; P:GO:0016310; F:GO:0033857; F:GO:0052723; F:GO:0052724; F:GO:0102092 | EC:2.7.4.24; EC:2.7.4.21 |
| TRINITY_DN22712_c0_g1_i2_len_1884_156449.p1_type_complete_len_232 | ---NA--- | | |
| TRINITY_DN7040_c0_g1_i1_len_700_239199.p1_type_internal_len_233 | XP_018008188.1 putative ATP-dependent RNA helicase TDRD12 isoform X1 | | |
| TRINITY_DN16361_c0_g1_i1_len_1282_32194.p1_type_5prime_partial_len_232 | ---NA--- | | |
| TRINITY_DN15622_c0_g1_i1_len_698_129053.p1_type_internal_len_233 | XP_018026663.1 organic cation transporter protein-like | | |
| TRINITY_DN12435_c0_g1_i2_len_816_198417.p1_type_5prime_partial_len_232 | ---NA--- | | |
| TRINITY_DN15434_c1_g1_i1_len_696_200068.p1_type_internal_len_233 | XP_018011574.1 hemocytin-like | C:GO:0005576; P:GO:0006030; F:GO:0008061 | |
| TRINITY_DN8857_c0_g1_i2_len_724_97799.p1_type_5prime_partial_len_232 | XP_008555612.1 protein Malvolio isoform X1 | | |
| TRINITY_DN8949_c0_g1_i1_len_703_272767.p1_type_5prime_partial_len_232 | ---NA--- | | |
| TRINITY_DN19001_c0_g1_i1_len_1588_60386.p2_type_5prime_partial_len_232 | ---NA--- | | |
| TRINITY_DN16171_c0_g1_i1_len_698_5768.p1_type_internal_len_233 | XP_018028072.1PREDICTED: uncharacterized protein LOC108683280 | | |
| TRINITY_DN14157_c0_g1_i1_len_697_163592.p1_type_internal_len_233 | XP_022252134.1probable ATP-dependent RNA helicase DDX4 | F:GO:0003676; F:GO:0003677; P:GO:0006139; F:GO:0008270; P:GO:0032968 | |
| TRINITY_DN20832_c0_g1_i2_len_1612_157376.p2_type_complete_len_232 | XP_015127606.1 max-like protein X | P:GO:0006355; F:GO:0008134; F:GO:0046983 | |
| TRINITY_DN19733_c0_g1_i2_len_699_5750.p1_type_internal_len_233 | ---NA--- | | |

| | | | |
|---|---|---|---|
| TRINITY_DN20242_c0_g1_i1_len_700_199995.p1_type_internal_len_233 | ---NA--- | | |
| TRINITY_DN16185_c0_g1_i1_len_1604_157402.p1_type_complete_len_232 | XP_018024607.1 ufm1-specific protease 1-like | F:GO:0008233 | |
| TRINITY_DN16989_c0_g1_i2_len_698_5760.p1_type_internal_len_233 | XP_021944442.1angiotensin-converting enzyme-like | | |
| TRINITY_DN14126_c0_g1_i1_len_783_97197.p1_type_5prime_partial_len_232 | XP_018009779.1PREDICTED: uncharacterized protein LOC108667285 | | |
| TRINITY_DN10600_c0_g1_i1_len_698_5761.p1_type_internal_len_233 | XP_013788241.1nucleoredoxin-like | C:GO:0005623; P:GO:0045454 | |
| TRINITY_DN26362_c0_g1_i1_len_5498_227682.p2_type_complete_len_232 | XP_018011191.1 copper-transporting ATPase 2-like | | |
| TRINITY_DN20991_c0_g1_i1_len_713_239007.p1_type_5prime_partial_len_232 | ---NA--- | | |
| TRINITY_DN12952_c0_g1_i2_len_722_163249.p1_type_5prime_partial_len_232 | ---NA--- | | |
| TRINITY_DN21810_c0_g1_i1_len_698_65701.p1_type_internal_len_233 | OMJ08900.1Retrovirus-related Pol polyprotein from transposon | F:GO:0003676; P:GO:0015074 | |
| TRINITY_DN9058_c0_g1_i1_len_698_239234.p1_type_internal_len_233 | XP_018021013.1PREDICTED: uncharacterized protein LOC108677311 | | |
| TRINITY_DN20110_c0_g1_i1_len_699_239216.p1_type_internal_len_233 | ---NA--- | | |
| TRINITY_DN8939_c0_g1_i1_len_699_200013.p1_type_internal_len_233 | AEV89780.1ATP-synthase subunit beta | F:GO:0005524; P:GO:0015986; C:GO:0045261; F:GO:0046933; P:GO:0099132 | EC:3.6.1.3; EC:3.6.1.15 |
| TRINITY_DN11627_c0_g1_i1_len_788_271875.p1_type_5prime_partial_len_232 | AFP92127.1plasmolipin isoform 2 | C:GO:0016021 | |
| TRINITY_DN10476_c0_g1_i2_len_699_200021.p1_type_internal_len_233 | ---NA--- | | |
| TRINITY_DN38733_c0_g1_i1_len_699_200019.p1_type_internal_len_233 | XP_019919370.1 von Willebrand factor A domain-containing protein 8 | F:GO:0005524; F:GO:0016887 | |
| TRINITY_DN3055_c0_g1_i1_len_698_239244.p1_type_internal_len_233 | XP_013070857.1 huntingtin-like | C:GO:0005634; C:GO:0005737 | |

| | | | |
|---|---|---|---|
| TRINITY_DN2573_c0_g1_i1_len_696_239263.p1_type_internal_len_233 | XP_018023688.1 probable ubiquitin carboxyl-terminal hydrolase FAF-X | | |
| TRINITY_DN9984_c0_g1_i1_len_697_200049.p1_type_internal_len_233 | ---NA--- | | |
| TRINITY_DN23948_c0_g1_i1_len_873_197772.p1_type_5prime_partial_len_232 | KFO95444.1WASH complex subunit CCDC53 | C:GO:0071203 | |
| TRINITY_DN17402_c0_g1_i1_len_826_127817.p1_type_5prime_partial_len_232 | XP_018914228.1 probable ribosome biogenesis protein RLP24 | C:GO:0005840 | |
| TRINITY_DN31121_c0_g1_i1_len_835_237595.p1_type_5prime_partial_len_232 | AAC05909.1potassium channel | F:GO:0005249; C:GO:0008076; P:GO:0034765; P:GO:0051260; P:GO:0071805 | |
| TRINITY_DN14670_c0_g1_i2_len_700_239205.p1_type_internal_len_233 | ---NA--- | | |
| TRINITY_DN36755_c0_g1_i1_len_699_239220.p1_type_internal_len_233 | ---NA--- | | |
| TRINITY_DN19753_c0_g1_i1_len_867_197835.p1_type_3prime_partial_len_233 | PNF19977.1ATP-dependent Clp protease ATP-binding subunit clpX-like, mitochondrial | F:GO:0005524; P:GO:0006457; P:GO:0006508; F:GO:0008233; F:GO:0051082 | |
| TRINITY_DN15006_c0_g1_i1_len_899_197510.p1_type_complete_len_232 | ---NA--- | | |
| TRINITY_DN9292_c0_g1_i1_len_697_239254.p1_type_internal_len_233 | XP_018014670.1 transmembrane channel-like protein 7 | | |
| TRINITY_DN25930_c0_g1_i1_len_3329_228617.p2_type_complete_len_232 | XP_018015375.1 polypeptide N-acetylgalactosaminyltransferase 13-like | | |
| TRINITY_DN28355_c0_g1_i2_len_2639_189153.p2_type_complete_len_231 | AHI85755.1cytoplasmic dynein intermediate chain | C:GO:0005868; P:GO:0007018 | |
| TRINITY_DN31436_c0_g1_i1_len_696_200077.p1_type_internal_len_232 | OWF35857.1JmjC domain-containing protein 4 | | |
| TRINITY_DN17149_c0_g1_i1_len_736_199490.p1_type_5prime_partial_len_231 | ACO11311.1Kynurenine/alpha-aminoadipate aminotransferase mitochondrial precursor | F:GO:0016212; P:GO:1901605 | EC:2.6.1.7 |
| TRINITY_DN10578_c0_g1_i2_len_695_200089.p1_type_internal_len_232 | XP_018011489.1 heparan sulfate glucosamine 3-O-sulfotransferase 1-like | | |

| | | | |
|---|---|---|---|
| TRINITY_DN1188_c0_g1_i1_len_696_200064.p1_type_internal_len_232 | ---NA--- | | |
| TRINITY_DN12932_c0_g1_i1_len_697_200054.p1_type_internal_len_232 | ---NA--- | | |
| TRINITY_DN28705_c1_g1_i1_len_697_200046.p1_type_internal_len_232 | ---NA--- | | |
| TRINITY_DN14287_c0_g1_i1_len_693_239324.p1_type_internal_len_232 | ---NA--- | | |
| TRINITY_DN9644_c0_g1_i1_len_695_200094.p1_type_internal_len_232 | XP_020785056.1multidrug resistance-associated protein 7 | F:GO:0005524; C:GO:0016021; F:GO:0042626; P:GO:0055085 | EC:3.6.1.3; EC:3.6.1.15 |
| TRINITY_DN197_c0_g1_i1_len_694_200103.p1_type_internal_len_232 | XP_018012218.1PREDICTED: uncharacterized protein LOC108669405 | | |
| TRINITY_DN39100_c0_g1_i1_len_694_200101.p1_type_internal_len_232 | XP_019868756.1 S-adenosylmethionine decarboxylase proenzyme isoform X2 | | |
| TRINITY_DN12991_c0_g1_i3_len_759_162831.p1_type_5prime_partial_len_231 | XP_018020182.1 CKLF-like MARVEL transmembrane domain-containing protein 4 | C:GO:0016020 | |
| TRINITY_DN22418_c0_g1_i2_len_3107_228879.p1_type_5prime_partial_len_1012 | XP_018017445.1 ATP-dependent RNA helicase DHX36-like | F:GO:0097159; F:GO:1901363 | |
| 1302\|_\|c1848_f2p6_3036_isoform_c1848_full_length_coverage_2_non_full_length_coverage_6_isoform_length_3036_25534 | XP_018023942.1 pre-mRNA-splicing factor ATP-dependent RNA helicase PRP16-like | | |
| 1303\|_\|c1791_f3p2_3035_isoform_c1791_full_length_coverage_3_non_full_length_coverage_2_isoform_length_3035_25488 | AXR98496.1protein lap4-like isoform X2 | | |
| 1304\|_\|c17875_f2p17_3035_isoform_c17875_full_length_coverage_2_non_full_length_coverage_17_isoform_length_3035_22532 | PSN45205.1DDB1- and CUL4-associated factor 7 | | |
| 1305\|_\|c18361_f5p64_3035_isoform_c18361_full_length_coverage_5_non_full_length_coverage_64_isoform_length_3035_26383 | AQZ36563.1serine/threonine protein phosphatase 1 | C:GO:0000164; F:GO:0004722; P:GO:0006470; F:GO:0046872 | EC:3.1.3.16 |

<table>
<tr><td>1306|_|c7733_f2p16_3034_isoform_c7733_full_length_coverage_2_non_full_length_coverage_16_isoform_length_3034_28484</td><td>XP_018016368.1PREDICTED: uncharacterized protein LOC108673097</td><td></td><td></td></tr>
<tr><td>TRINITY_DN26973_c0_g1_i1_len_3035_188480.p1_type_internal_len_1012</td><td>XP_020294735.1ATP-dependent helicase brm-like</td><td></td><td></td></tr>
<tr><td>1308|_|c954_f9p26_3032_isoform_c954_full_length_coverage_9_non_full_length_coverage_26_isoform_length_3032_28413</td><td>XP_023711124.1metastasis-associated protein MTA3 isoform X3</td><td></td><td></td></tr>
<tr><td>1309|_|c2485_f3p2_3031_isoform_c2485_full_length_coverage_3_non_full_length_coverage_2_isoform_length_3031_20977</td><td>ATA58412.1mitochondrial ECHA</td><td>F:GO:0003857; F:GO:0004300; P:GO:0006635; C:GO:0016507</td><td>EC:1.1.1.35; EC:4.2.1.17</td></tr>
<tr><td>1310|_|c1775_f4p39_3031_isoform_c1775_full_length_coverage_4_non_full_length_coverage_39_isoform_length_3031_27967</td><td>XP_018018815.1 importin subunit beta-1-like</td><td>C:GO:0005623; P:GO:0006606; F:GO:0008536</td><td></td></tr>
<tr><td>TRINITY_DN24125_c0_g1_i1_len_969_196819.p1_type_complete_len_231</td><td>XP_018024921.1 enhancer of split mbeta protein-like</td><td>F:GO:0000977; P:GO:0001756; F:GO:0003714; C:GO:0005634; P:GO:0006355; P:GO:0007219; F:GO:0008134; F:GO:0046983; P:GO:0050767</td><td></td></tr>
<tr><td>TRINITY_DN18509_c0_g1_i1_len_696_239267.p1_type_internal_len_232</td><td>KDR24269.1putative phosphorylase b kinase regulatory subunit alpha</td><td></td><td></td></tr>
<tr><td>TRINITY_DN15513_c0_g1_i1_len_1609_232500.p1_type_complete_len_231</td><td>XP_023221379.1equilibrative nucleoside transporter 4-like</td><td></td><td></td></tr>
<tr><td>TRINITY_DN17543_c0_g1_i2_len_859_34367.p1_type_3prime_partial_len_232</td><td>XP_023342824.1probable methyltransferase BMT2 homolog isoform X1</td><td></td><td></td></tr>
<tr><td>TRINITY_DN21492_c0_g1_i3_len_2279_295357.p2_type_3prime_partial_len_232</td><td>---NA---</td><td></td><td></td></tr>
<tr><td>TRINITY_DN6780_c0_g1_i1_len_825_198317.p1_type_3prime_partial_len_232</td><td>XP_026678252.1nuclear factor interleukin-3-regulated protein</td><td>F:GO:0003700; P:GO:0006355</td><td></td></tr>
<tr><td>TRINITY_DN31655_c0_g1_i1_len_729_199594.p1_type_3prime_partial_len_232</td><td>AAM21213.1beta-1,3-glucan binding protein</td><td>F:GO:0001872; F:GO:0004553; C:GO:0005576; P:GO:0005975; P:GO:0045087</td><td></td></tr>
</table>

| | | | |
|---|---|---|---|
| TRINITY_DN7324_c0_g1_i1_len_737_199475.p1_type_5prime_partial_len_231 | XP_018021718.1 MAGUK p55 subfamily member 7-like | | |
| TRINITY_DN9610_c0_g1_i2_len_963_63335.p1_type_5prime_partial_len_231 | XP_008191489.1 protein NDRG3 isoform X2 | | |
| TRINITY_DN18315_c0_g2_i1_len_958_196918.p1_type_5prime_partial_len_231 | XP_018013672.1 MOB kinase activator-like 3 isoform X1 | | |
| TRINITY_DN13794_c0_g1_i1_len_694_301179.p1_type_internal_len_232 | XP_018024607.1 ufm1-specific protease 1-like | | |
| TRINITY_DN19366_c0_g1_i2_len_2085_674.p2_type_5prime_partial_len_231 | KXJ14131.1Adenylosuccinate lyase | | |
| TRINITY_DN20094_c0_g1_i1_len_1400_193649.p1_type_complete_len_231 | XP_023720012.1E3 ubiquitin-protein ligase ZNRF1 isoform X2 | | |
| TRINITY_DN14395_c0_g1_i1_len_693_272885.p1_type_internal_len_232 | ---NA--- | | |
| TRINITY_DN16956_c0_g1_i1_len_695_239288.p1_type_internal_len_232 | ---NA--- | | |
| TRINITY_DN15210_c0_g1_i1_len_695_239282.p1_type_internal_len_232 | ---NA--- | | |
| TRINITY_DN15106_c0_g1_i1_len_948_33779.p1_type_5prime_partial_len_230 | ---NA--- | | |
| TRINITY_DN20484_c0_g1_i1_len_700_199986.p1_type_5prime_partial_len_230 | XP_018011853.1PREDICTED: uncharacterized protein LOC108669081 | F:GO:0008289 | |
| TRINITY_DN12968_c0_g1_i1_len_827_162144.p1_type_complete_len_230 | ---NA--- | | |
| TRINITY_DN21921_c0_g2_i1_len_896_299439.p1_type_5prime_partial_len_230 | ---NA--- | | |
| TRINITY_DN4692_c0_g1_i2_len_762_199098.p1_type_3prime_partial_len_231 | XP_018017489.1 BTB/POZ domain-containing protein 7-like | | |
| TRINITY_DN16005_c0_g1_i1_len_936_96026.p1_type_complete_len_230 | XP_023727912.1probable 28S ribosomal protein S26, mitochondrial | C:GO:0005763; C:GO:0005840 | |
| TRINITY_DN24895_c0_g1_i1_len_692_65777.p1_type_internal_len_231 | XP_018013899.1 translocation protein SEC62-like | | |

| | | | |
|---|---|---|---|
| TRINITY_DN24557_c0_g1_i1_len_817_34656.p1_type_3prime_partial_len_231 | DAA34418.1TPA_inf: BTB/POZ and Kelch domain-containing protein | | |
| TRINITY_DN8282_c0_g1_i1_len_691_5838.p1_type_internal_len_231 | ---NA--- | | |
| TRINITY_DN22371_c0_g1_i1_len_1110_195625.p1_type_5prime_partial_len_230 | XP_018012068.1 charged multivesicular body protein 1b-2-like | | |
| TRINITY_DN15636_c1_g1_i1_len_740_272357.p1_type_complete_len_230 | ---NA--- | | |
| TRINITY_DN35615_c0_g1_i1_len_693_200107.p1_type_internal_len_231 | XP_012214850.1 phosphatidylinositol 4-kinase alpha isoform X2 | P:GO:0016310; F:GO:0016740 | |
| TRINITY_DN16057_c0_g1_i1_len_690_239372.p1_type_internal_len_231 | ---NA--- | | |
| TRINITY_DN27839_c4_g1_i1_len_691_200139.p1_type_internal_len_231 | ---NA--- | | |
| TRINITY_DN30158_c0_g1_i1_len_3086_188398.p1_type_5prime_partial_len_230 | XP_018014740.1 transport and Golgi organization protein 11-like | | |
| TRINITY_DN15822_c0_g1_i1_len_692_200115.p1_type_3prime_partial_len_231 | ---NA--- | | |
| TRINITY_DN15411_c0_g1_i1_len_845_198086.p1_type_5prime_partial_len_230 | XP_021925982.1alpha-sarcoglycan isoform X1 | C:GO:0016012; C:GO:0016020; C:GO:0016021 | |
| TRINITY_DN14208_c0_g1_i1_len_694_98152.p1_type_internal_len_231 | KZS19268.1Disrupted in renal carcinoma protein 2 | C:GO:0016020; C:GO:0016021; C:GO:0043231; P:GO:0055085 | |
| TRINITY_DN9339_c0_g1_i1_len_694_98154.p1_type_3prime_partial_len_231 | XP_018022851.1 transmembrane protein 65-like | | |
| TRINITY_DN16748_c0_g1_i1_len_694_200098.p1_type_internal_len_231 | ---NA--- | | |
| TRINITY_DN13785_c0_g1_i3_len_691_35834.p1_type_internal_len_231 | OQR74152.1cuticular protein-like | C:GO:0005576; P:GO:0006030; F:GO:0008061 | |
| TRINITY_DN20151_c0_g1_i1_len_690_239363.p1_type_internal_len_231 | XP_021933594.1probable E3 ubiquitin-protein ligase HERC1 isoform X2 | F:GO:0004842; P:GO:0016567 | |
| TRINITY_DN26105_c0_g1_i7_len_1314_233963.p1_type_complete_len_230 | ---NA--- | | |

| | | | |
|---|---|---|---|
| TRINITY_DN15621_c0_g1_i1_len_803_237917.p1_type_3prime_partial_len_231 | ---NA--- | | |
| TRINITY_DN6898_c0_g1_i1_len_691_239348.p1_type_internal_len_231 | XP_018027560.1 exocyst complex component 2-like | P:GO:0016192 | |
| TRINITY_DN18363_c0_g1_i1_len_871_197797.p1_type_5prime_partial_len_230 | XP_022194901.1carbohydrate sulfotransferase 11-like isoform X3 | C:GO:0000139; C:GO:0005794; P:GO:0005975; F:GO:0008146; C:GO:0016021; P:GO:0016051; F:GO:0016740 | |
| TRINITY_DN34867_c0_g1_i1_len_691_239355.p1_type_internal_len_231 | ---NA--- | | |
| TRINITY_DN41024_c0_g1_i1_len_692_200119.p1_type_internal_len_231 | OXA61697.1Transmembrane protein 62 | C:GO:0016020; C:GO:0016021; F:GO:0016787 | |
| TRINITY_DN13297_c0_g1_i1_len_693_239314.p1_type_internal_len_231 | PNF42530.1hypothetical protein B7P43_G08113, partial | | |
| TRINITY_DN11077_c0_g1_i1_len_691_200146.p1_type_internal_len_231 | XP_018012231.1 PH and SEC7 domain-containing protein 3-like isoform X3 | | |
| 12152\|_\|c58211_f4p3_688_isoform_c58211_full_length_coverage_4_non_full_length_coverage_3_isoform_length_688_6879 | AGM32197.1RNA binding domain containing protein | F:GO:0003723 | |
| TRINITY_DN25243_c0_g1_i1_len_1669_192214.p1_type_5prime_partial_len_229 | XP_017892772.1charged multivesicular body protein 6 | P:GO:0007034 | |
| TRINITY_DN11004_c0_g1_i1_len_962_236409.p1_type_5prime_partial_len_229 | XP_018020402.1 transcription factor AP-2-epsilon-like isoform X2 | F:GO:0003700; C:GO:0005634; P:GO:0006355 | |
| TRINITY_DN16675_c0_g1_i2_len_689_239382.p1_type_internal_len_230 | ---NA--- | | |
| TRINITY_DN2008_c0_g1_i1_len_690_35840.p1_type_internal_len_230 | XP_018010562.1 vacuolar protein sorting-associated protein 13D-like | | |
| TRINITY_DN15309_c0_g1_i1_len_688_129144.p1_type_internal_len_230 | XP_018011694.1 laminin subunit alpha-1-like | | |
| TRINITY_DN3831_c0_g1_i1_len_841_237534.p1_type_5prime_partial_len_229 | XP_022250474.1deleted in autism-related protein 1 homolog isoform X3 | | |
| TRINITY_DN13463_c0_g1_i1_len_690_239369.p1_type_5prime_partial_len_229 | OWF51807.1UPF0505 protein C16orf62-like | | |

| | | | |
|---|---|---|---|
| TRINITY_DN20903_c0_g1_i1_len_835_198202.p1_type_5prime_partial_len_229 | XP_013403254.1tail-anchored protein insertion receptor WRB | C:GO:0016020; C:GO:0016021; P:GO:0071816 | |
| TRINITY_DN17732_c0_g1_i1_len_919_161341.p1_type_complete_len_229 | XP_018010355.1 pyroglutamyl-peptidase 1-like | C:GO:0005829; P:GO:0006508; F:GO:0008233; F:GO:0016920 | |
| TRINITY_DN23606_c0_g1_i3_len_2042_123043.p2_type_5prime_partial_len_229 | ---NA--- | | |
| TRINITY_DN18700_c0_g1_i1_len_2594_229617.p1_type_5prime_partial_len_229 | XP_018021907.1 V-type proton ATPase 16 kDa proteolipid subunit | C:GO:0005774; F:GO:0015078; P:GO:0015991; C:GO:0016021; C:GO:0033179 | |
| TRINITY_DN18260_c0_g1_i1_len_852_237414.p1_type_3prime_partial_len_230 | ---NA--- | | |
| TRINITY_DN16533_c0_g1_i1_len_689_65807.p1_type_internal_len_230 | ---NA--- | | |
| TRINITY_DN16064_c0_g1_i2_len_691_65779.p2_type_internal_len_230 | ACL79884.1putative articulin p60 | | |
| TRINITY_DN23370_c0_g1_i2_len_879_197709.p1_type_5prime_partial_len_229 | XP_023210660.1sodium/glucose cotransporter 4-like isoform X3 | | |
| TRINITY_DN23930_c0_g1_i1_len_1421_233396.p1_type_complete_len_229 | XP_018021506.1 REM2- and Rab-like small GTPase 1 | F:GO:0003924; F:GO:0005525 | |
| TRINITY_DN372_c0_g1_i1_len_848_237449.p1_type_5prime_partial_len_229 | XP_021955035.1ecdysone-induced protein 74EF-like | F:GO:0003700; C:GO:0005634; P:GO:0006355; F:GO:0043565 | |
| TRINITY_DN25523_c0_g1_i1_len_702_199960.p1_type_5prime_partial_len_229 | ---NA--- | | |
| TRINITY_DN4913_c0_g1_i1_len_727_199623.p1_type_3prime_partial_len_230 | ---NA--- | | |
| TRINITY_DN34504_c0_g1_i1_len_688_239392.p1_type_internal_len_230 | ---NA--- | | |
| TRINITY_DN29162_c0_g1_i4_len_2237_190143.p3_type_complete_len_229 | AWF70751.1Wnt9a protein | F:GO:0005102; C:GO:0005576; P:GO:0007275; P:GO:0016055 | |
| TRINITY_DN13192_c0_g1_i1_len_689_239384.p1_type_internal_len_230 | ---NA--- | | |
| TRINITY_DN14544_c0_g1_i1_len_688_239400.p1_type_internal_len_230 | XP_018017231.1 dmX-like protein 2 isoform X2 | | |

| | | | |
|---|---|---|---|
| TRINITY_DN7747_c0_g1_i1_len_688_239397.p1_type_internal_len_230 | AYN63914.1amine oxidase | | |
| TRINITY_DN31621_c0_g1_i1_len_692_163650.p1_type_complete_len_229 | ALL27072.1vascular endothelial growth factor 2 | F:GO:0008083; P:GO:0010469; C:GO:0016020 | |
| TRINITY_DN3871_c0_g1_i1_len_690_200150.p1_type_3prime_partial_len_230 | XP_022252404.1probable phospholipid-transporting ATPase VA isoform X2 | F:GO:0000287; F:GO:0004012; F:GO:0005524; C:GO:0016021; P:GO:0045332 | EC:3.6.1.3; EC:3.6.3.1; EC:3.6.1.15 |
| TRINITY_DN30215_c0_g1_i1_len_689_163688.p1_type_internal_len_230 | XP_010834622.1 zinc finger protein 239-like | F:GO:0003676 | |
| TRINITY_DN39819_c0_g1_i1_len_687_200195.p1_type_internal_len_230 | XP_018020970.1 synaptojanin-1-like isoform X2 | | |
| TRINITY_DN12845_c0_g1_i1_len_690_200154.p1_type_internal_len_230 | ---NA--- | | |
| TRINITY_DN9063_c0_g1_i1_len_691_98182.p1_type_internal_len_230 | ---NA--- | | |
| TRINITY_DN22078_c0_g1_i1_len_690_200157.p1_type_internal_len_230 | XP_021449982.1probable thiopurine S-methyltransferase | F:GO:0016740 | |
| TRINITY_DN7542_c0_g1_i1_len_791_128112.p1_type_5prime_partial_len_229 | XP_021353595.1maleylacetoacetate isomerase-like | F:GO:0003824; C:GO:0005737; P:GO:0009072 | |
| TRINITY_DN19018_c0_g1_i1_len_688_200175.p1_type_internal_len_230 | XP_018330991.1A-kinase anchor protein 1, mitochondrial isoform X2 | F:GO:0003676; F:GO:0003723; C:GO:0005739; P:GO:0010738; F:GO:0016301; P:GO:0016310; F:GO:0034237 | |
| TRINITY_DN12423_c0_g1_i1_len_688_200193.p1_type_internal_len_230 | ---NA--- | | |
| TRINITY_DN12939_c0_g1_i1_len_1105_94965.p1_type_5prime_partial_len_229 | XP_020300550.1eukaryotic translation initiation factor 4E-like isoform X2 | F:GO:0003743; C:GO:0005737; P:GO:0006413 | |
| TRINITY_DN33917_c0_g1_i1_len_688_163708.p1_type_internal_len_230 | ---NA--- | | |
| TRINITY_DN13965_c0_g1_i1_len_722_238874.p1_type_5prime_partial_len_229 | XP_003746523.1 calcipressin-2 | F:GO:0003676; P:GO:0019722 | |
| TRINITY_DN19166_c0_g1_i1_len_1115_269547.p1_type_5prime_partial_len_229 | ---NA--- | | |

| | | | |
|---|---|---|---|
| TRINITY_DN21696_c0_g1_i2_len_2189_91840.p2_type_5prime_partial_len_229 | AWM95782.1type 1 phosphodiesterase | F:GO:0004114; P:GO:0007165; F:GO:0046872 | EC:3.1.4.17 |
| TRINITY_DN28705_c2_g1_i1_len_3605_187844.p2_type_complete_len_229 | ---NA--- | | |
| TRINITY_DN12873_c0_g1_i1_len_868_271170.p1_type_5prime_partial_len_229 | XP_018010761.1 synaptobrevin homolog YKT6-like | C:GO:0016021; P:GO:0016192 | |
| TRINITY_DN20954_c1_g1_i2_len_879_197717.p1_type_complete_len_228 | XP_018025858.1 ribulose-phosphate 3-epimerase-like | | |
| TRINITY_DN16120_c0_g1_i1_len_686_129169.p1_type_internal_len_229 | ---NA--- | | |
| TRINITY_DN18288_c0_g1_i1_len_949_299097.p1_type_complete_len_228 | ASS34530.1peroxiredoxin 3 | C:GO:0005623; P:GO:0045454; F:GO:0051920; P:GO:0055114; P:GO:0098869 | EC:1.11.1.15 |
| TRINITY_DN14334_c0_g1_i1_len_688_5870.p1_type_internal_len_229 | EEB20500.1ubiquitously transcribed X chromosome tetratricopeptide repeat protein, putative | P:GO:0010468; F:GO:0046872; P:GO:0071557; F:GO:0071558 | |
| TRINITY_DN28658_c0_g1_i1_len_684_239444.p1_type_internal_len_229 | XP_018023301.1 formin-like protein CG32138 | C:GO:0005884; F:GO:0008017; P:GO:0045010 | |
| TRINITY_DN12942_c0_g1_i1_len_685_5900.p1_type_internal_len_229 | ---NA--- | | |
| TRINITY_DN15349_c0_g1_i1_len_909_236868.p1_type_5prime_partial_len_228 | ---NA--- | | |
| TRINITY_DN21331_c0_g1_i3_len_998_196564.p1_type_5prime_partial_len_228 | AAD41899.1fushi tarazu-factor 1 | F:GO:0003700; F:GO:0003707; C:GO:0005634; P:GO:0006355; F:GO:0008270; P:GO:0043401; F:GO:0043565 | |
| TRINITY_DN14873_c0_g1_i1_len_686_200225.p1_type_internal_len_229 | XP_018015385.1 serine/arginine repetitive matrix protein 2-like | | |
| TRINITY_DN33527_c0_g1_i1_len_686_200219.p1_type_internal_len_229 | XP_018007608.1 protein PTHB1-like isoform X2 | C:GO:0034464 | |
| TRINITY_DN21114_c0_g1_i1_len_735_199518.p1_type_3prime_partial_len_229 | XP_018020565.1 calpain-7-like | | |

| | | | |
|---|---|---|---|
| TRINITY_DN4701_c0_g1_i1_len_684_200246.p1_type_internal_len_229 | XP_018012344.1 dystonin-like isoform X2 | F:GO:0005509; F:GO:0008017 | |
| TRINITY_DN19876_c0_g1_i1_len_3280_154074.p1_type_complete_len_1010 | XP_023230845.1integrator complex subunit 7-like | | |
| TRINITY_DN22966_c1_g1_i1_len_3520_153896.p1_type_complete_len_1010 | XP_015230520.1 zinc finger protein OZF-like isoform X2 | | |
| TRINITY_DN29489_c0_g1_i3_len_3224_188216.p1_type_3prime_partial_len_1011 | XP_021695061.1uncharacterized protein LOC110674855 | | |
| 1314\|_\|c1233_f6p16_3028_isoform_c1233_full_length_coverage_6_non_full_length_coverage_16_isoform_length_3028_25841 | AYJ76527.1cell division cycle 42 protein | F:GO:0003924; F:GO:0005525; P:GO:0007264; P:GO:0051301 | EC:3.6.1.15 |
| TRINITY_DN23055_c0_g1_i3_len_3238_228728.p1_type_3prime_partial_len_1010 | XP_018018909.1PREDICTED: uncharacterized protein LOC108675412 | | |
| TRINITY_DN29132_c0_g1_i1_len_3625_187831.p1_type_5prime_partial_len_1009 | KMQ93617.1protein furry | P:GO:0000902 | |
| 1317\|_\|c2054_f2p14_3027_isoform_c2054_full_length_coverage_2_non_full_length_coverage_14_isoform_length_3027_28187 | XP_014480905.1 E3 ubiquitin-protein ligase hyd isoform X4 | | |
| TRINITY_DN21483_c0_g1_i1_len_3878_90908.p1_type_5prime_partial_len_1009 | XP_018024748.1 N-alpha-acetyltransferase 25, NatB auxiliary subunit-like | | |
| 1319\|_\|c5569_f2p5_3026_isoform_c5569_full_length_coverage_2_non_full_length_coverage_5_isoform_length_3026_28400 | XP_026076677.1uncharacterized protein LOC113054993 isoform X2 | | |
| TRINITY_DN25474_c0_g1_i1_len_3074_228911.p1_type_3prime_partial_len_1009 | XP_018007992.1 rootletin-like | | |
| TRINITY_DN6092_c0_g1_i1_len_684_163758.p1_type_internal_len_229 | XP_021695049.1uncharacterized protein LOC110674850 | | |
| TRINITY_DN3285_c0_g1_i1_len_686_98243.p1_type_internal_len_229 | XP_015524156.1 protein lunapark-B isoform X2 | | |
| TRINITY_DN3016_c0_g1_i1_len_686_200214.p1_type_internal_len_229 | AFP33455.1kinesin-like protein KIF3A | F:GO:0003777; F:GO:0005524; C:GO:0005874; P:GO:0007018; F:GO:0008017 | EC:3.6.1.15 |

| | | | |
|---|---|---|---|
| TRINITY_DN275_c0_g1_i1_len_687_200211.p1_type_internal_len_229 | ---NA--- | | |
| TRINITY_DN33390_c0_g1_i1_len_688_200180.p1_type_internal_len_229 | KDR08642.1Bromodomain and WD repeat-containing protein 2 | | |
| TRINITY_DN17958_c0_g1_i1_len_807_198515.p1_type_5prime_partial_len_228 | XP_015438616.1 type II inositol 1,4,5-trisphosphate 5-phosphatase | P:GO:0009987; F:GO:0016787 | |
| TRINITY_DN11092_c0_g1_i1_len_686_65845.p1_type_internal_len_229 | ---NA--- | | |
| TRINITY_DN20207_c0_g1_i1_len_2071_190652.p1_type_5prime_partial_len_228 | ---NA--- | | |
| TRINITY_DN31173_c0_g1_i1_len_808_198506.p1_type_3prime_partial_len_229 | ---NA--- | | |
| TRINITY_DN16416_c0_g1_i2_len_814_237800.p2_type_5prime_partial_len_228 | ---NA--- | | |
| TRINITY_DN27721_c0_g1_i1_len_855_161893.p1_type_5prime_partial_len_228 | ---NA--- | | |
| TRINITY_DN13790_c0_g1_i1_len_685_239428.p1_type_internal_len_229 | ---NA--- | | |
| TRINITY_DN19012_c0_g1_i1_len_688_239387.p1_type_internal_len_229 | ---NA--- | | |
| TRINITY_DN24373_c0_g1_i1_len_1003_196527.p1_type_5prime_partial_len_227 | ---NA--- | | |
| TRINITY_DN15865_c0_g1_i1_len_683_272993.p1_type_internal_len_228 | ---NA--- | | |
| TRINITY_DN14455_c0_g1_i1_len_683_35924.p1_type_internal_len_228 | ---NA--- | | |
| TRINITY_DN17161_c0_g1_i1_len_995_95629.p1_type_complete_len_227 | XP_018026696.1 diphosphoinositol polyphosphate phosphohydrolase 1-like | | |
| TRINITY_DN12706_c0_g1_i1_len_1015_235992.p1_type_complete_len_227 | ---NA--- | | |
| TRINITY_DN16616_c0_g1_i1_len_1446_60898.p1_type_complete_len_227 | XP_016427331.1 putative lipoyltransferase 2, mitochondrial | | |

| | | | |
|---|---|---|---|
| TRINITY_DN20002_c0_g2_i2_len_683_272989.p1_type_internal_len_228 | XP_026280890.1oocyte zinc finger protein XlCOF6-like isoform X4 | | |
| TRINITY_DN30014_c0_g1_i1_len_3443_187994.p1_type_5prime_partial_len_227 | XP_018018040.1 SPRY domain-containing protein 7-like | | |
| TRINITY_DN6980_c0_g1_i1_len_683_200262.p1_type_internal_len_228 | PSN46505.1Arf-GAP with coiled-coil | F:GO:0005096; P:GO:0043547; F:GO:0046872 | |
| TRINITY_DN33383_c0_g1_i1_len_683_200260.p1_type_internal_len_228 | XP_018012609.1 glucosylceramidase-like | | |
| TRINITY_DN15330_c0_g1_i1_len_683_35920.p1_type_internal_len_228 | ---NA--- | | |
| TRINITY_DN31511_c0_g1_i1_len_682_200283.p1_type_internal_len_228 | PNF39819.1hypothetical protein B7P43_G03176 | F:GO:0005089; P:GO:0035023 | |
| TRINITY_DN31879_c0_g1_i1_len_683_200265.p1_type_internal_len_228 | KFM69677.1DNA repair protein REV1 | P:GO:0006281 | |
| TRINITY_DN33747_c0_g1_i1_len_684_200257.p1_type_internal_len_228 | APC26118.1metabotropic glutamate receptor type 7 | F:GO:0004930; C:GO:0005886; P:GO:0007186; C:GO:0016021 | |
| TRINITY_DN20700_c0_g1_i1_len_684_200253.p1_type_internal_len_228 | ---NA--- | | |
| TRINITY_DN8008_c0_g1_i1_len_683_200272.p1_type_internal_len_228 | AOO86752.1A-type potassium channel Shaker | F:GO:0005249; C:GO:0008076; P:GO:0034765; P:GO:0051260; P:GO:0071805 | |
| TRINITY_DN17495_c0_g1_i1_len_685_200236.p1_type_internal_len_228 | XP_008334194.1centrosomal protein of 78 kDa | | |
| TRINITY_DN13309_c0_g1_i1_len_685_200234.p1_type_internal_len_228 | ASW35072.1rho GTPase-activating protein 7 isoform X2 like | | |
| TRINITY_DN37410_c0_g1_i1_len_683_200259.p1_type_internal_len_228 | ADM45311.1caspase | F:GO:0004197; P:GO:0006508; F:GO:0008234 | |
| TRINITY_DN1132_c0_g1_i1_len_683_200263.p1_type_internal_len_228 | XP_021935740.1cadherin-23 isoform X4 | | |
| TRINITY_DN9428_c0_g1_i1_len_3751_228315.p2_type_complete_len_227 | ---NA--- | | |
| TRINITY_DN14631_c0_g1_i1_len_683_200258.p1_type_internal_len_228 | ---NA--- | | |

| | | | |
|---|---|---|---|
| TRINITY_DN24491_c0_g1_i1_len_771_238284.p1_type_5prime_partial_len_227 | XP_022186156.1gastrula zinc finger protein XlCGF57.1-like isoform X1 | | |
| TRINITY_DN9123_c0_g1_i1_len_847_161965.p2_type_5prime_partial_len_227 | ---NA--- | | |
| TRINITY_DN24375_c0_g1_i1_len_856_197977.p1_type_5prime_partial_len_227 | XP_018009784.1PREDICTED: uncharacterized protein LOC108667286 | | |
| TRINITY_DN17494_c0_g1_i1_len_685_239430.p1_type_internal_len_228 | XP_018028226.1 protein ecdysoneless homolog | | |
| TRINITY_DN25130_c1_g1_i2_len_733_199541.p1_type_5prime_partial_len_227 | ---NA--- | | |
| TRINITY_DN17648_c0_g2_i2_len_682_239468.p1_type_internal_len_228 | AFP33455.1kinesin-like protein KIF3A | F:GO:0003777; F:GO:0005524; C:GO:0005874; P:GO:0007018; F:GO:0008017 | EC:3.6.1.15 |
| TRINITY_DN14694_c0_g1_i3_len_682_239470.p1_type_internal_len_228 | ---NA--- | | |
| TRINITY_DN365_c0_g1_i1_len_716_163332.p1_type_5prime_partial_len_227 | XP_018016091.1 PQ-loop repeat-containing protein 3-like | | |
| TRINITY_DN30448_c0_g1_i1_len_683_239457.p1_type_internal_len_228 | ---NA--- | | |
| TRINITY_DN22928_c0_g1_i2_len_1486_233089.p2_type_3prime_partial_len_228 | ---NA--- | | |
| TRINITY_DN17221_c0_g1_i1_len_1797_156691.p1_type_5prime_partial_len_227 | ADD38097.1Mitotic spindle assembly checkpoint protein MAD2A | P:GO:0007094 | |
| TRINITY_DN6558_c0_g1_i1_len_755_199205.p1_type_3prime_partial_len_228 | XP_018025653.1 protein ELYS-like | | |
| TRINITY_DN5176_c0_g1_i1_len_684_163751.p1_type_internal_len_228 | XP_018022654.1 putative fatty acyl-CoA reductase CG5065 isoform X2 | P:GO:0006629; C:GO:0016020; C:GO:0016021; F:GO:0016491; P:GO:0055114; F:GO:0080019; F:GO:0102965 | |
| TRINITY_DN16679_c0_g1_i1_len_792_198716.p1_type_5prime_partial_len_227 | XP_019762789.1 nocturnin isoform X1 | | |
| TRINITY_DN112_c0_g1_i1_len_684_129189.p1_type_internal_len_228 | ---NA--- | | |

| | | | |
|---|---|---|---|
| TRINITY_DN26355_c0_g1_i3_len_2402_189689.p2_type_complete_len_226 | ---NA--- | | |
| TRINITY_DN33340_c0_g1_i1_len_680_200303.p1_type_internal_len_227 | XP_023222053.1membrane-associated guanylate kinase, WW and PDZ domain-containing protein 2-like isoform X4 | | |
| TRINITY_DN17184_c0_g1_i1_len_1327_93926.p1_type_complete_len_226 | XP_018012221.1 protein enhancer of sevenless 2B | P:GO:0007265 | |
| TRINITY_DN13001_c0_g1_i1_len_737_300731.p1_type_5prime_partial_len_226 | XP_026273170.139S ribosomal protein L21, mitochondrial | C:GO:0005840 | |
| TRINITY_DN19233_c0_g1_i1_len_972_298948.p1_type_complete_len_226 | XP_015269543.1 oocyte zinc finger protein XlCOF6-like | | |
| TRINITY_DN23541_c0_g1_i5_len_1182_159441.p1_type_5prime_partial_len_226 | AEL23121.1EF-Tu d2 protein | F:GO:0003676; F:GO:0008270 | |
| TRINITY_DN14437_c0_g1_i1_len_694_239300.p1_type_3prime_partial_len_227 | XP_025104863.1 UDP-galactose transporter senju-like | | |
| TRINITY_DN13892_c0_g1_i1_len_1292_297465.p1_type_complete_len_226 | XP_023723632.128S ribosomal protein S11, mitochondrial isoform X1 | | |
| TRINITY_DN27979_c0_g1_i3_len_1497_193123.p1_type_5prime_partial_len_226 | XP_023166198.1uncharacterized protein LOC111596281 isoform X2 | | |
| TRINITY_DN16404_c0_g1_i1_len_765_300460.p1_type_5prime_partial_len_226 | ANH58178.1delta class glutathione S-transferase | C:GO:0005739; F:GO:0016740 | |
| TRINITY_DN11616_c0_g1_i2_len_684_129198.p1_type_5prime_partial_len_226 | KZS12189.1Uncharacterized protein APZ42_022991 | | |
| TRINITY_DN6497_c0_g1_i1_len_681_200293.p1_type_internal_len_227 | AUI80370.1cryptochrome 1 | | |
| TRINITY_DN2107_c0_g1_i1_len_678_200344.p1_type_internal_len_227 | ---NA--- | | |
| TRINITY_DN4504_c0_g1_i1_len_679_200330.p1_type_internal_len_227 | XP_018020814.1 UDP-N-acetylhexosamine pyrophosphorylase-like | F:GO:0003977; P:GO:0006048 | EC:2.7.7.23 |
| TRINITY_DN19123_c0_g1_i2_len_681_129227.p1_type_internal_len_227 | XP_019753388.1 SWI/SNF-related matrix-associated actin-dependent regulator of chromatin subfamily E member 1 | F:GO:0003677; C:GO:0005634; P:GO:0006508; F:GO:0008234; C:GO:0016514; P:GO:0043044 | |

| | | | |
|---|---|---|---|
| TRINITY_DN30921_c4_g3_i1_len_1385_193736.p1_type_complete_len_226 | AGC24180.1inhibition of apoptosis protein | | |
| TRINITY_DN34981_c0_g1_i1_len_678_239528.p1_type_internal_len_227 | XP_023237597.1fat-like cadherin-related tumor suppressor homolog isoform X1 | C:GO:0016020 | |
| TRINITY_DN19335_c0_g1_i3_len_1084_33060.p1_type_complete_len_226 | ---NA--- | | |
| TRINITY_DN31189_c0_g1_i1_len_6657_186842.p4_type_5prime_partial_len_226 | ---NA--- | | |
| TRINITY_DN20913_c0_g1_i1_len_679_200326.p1_type_internal_len_227 | XP_018019174.1PREDICTED: uncharacterized protein LOC108675658 | | |
| TRINITY_DN24225_c0_g1_i1_len_801_198600.p1_type_5prime_partial_len_226 | ---NA--- | | |
| TRINITY_DN35463_c0_g1_i1_len_750_199275.p1_type_3prime_partial_len_227 | ---NA--- | | |
| TRINITY_DN39183_c0_g1_i1_len_680_200311.p1_type_3prime_partial_len_227 | ---NA--- | | |
| TRINITY_DN16069_c0_g1_i2_len_984_236253.p1_type_5prime_partial_len_226 | XP_002739066.1 isochorismatase domain-containing protein 2, mitochondrial-like | | |
| TRINITY_DN23374_c0_g1_i4_len_1834_231628.p2_type_5prime_partial_len_226 | XP_018019213.1 glutamate receptor ionotropic, kainate 5-like | | |
| TRINITY_DN9608_c0_g1_i1_len_682_239469.p1_type_internal_len_227 | ---NA--- | | |
| TRINITY_DN15975_c0_g1_i1_len_741_199414.p1_type_5prime_partial_len_226 | KDR13603.1Male-specific lethal 2-like protein | P:GO:0016567; F:GO:0061630; C:GO:0072487 | |
| TRINITY_DN11968_c0_g1_i1_len_1032_235859.p1_type_5prime_partial_len_226 | AID61753.1fructose 1,6-biphosphate-aldolase A | F:GO:0004332; P:GO:0006096 | EC:4.1.2.13 |
| TRINITY_DN13621_c0_g1_i1_len_908_96213.p1_type_5prime_partial_len_226 | ---NA--- | | |
| TRINITY_DN22134_c0_g1_i2_len_989_196647.p1_type_complete_len_226 | PNF43004.1Translin | F:GO:0003676 | |
| TRINITY_DN34715_c0_g1_i1_len_680_239498.p1_type_internal_len_227 | KFM63814.1Myelin regulatory factor | F:GO:0003677; F:GO:0003700; P:GO:0006355; C:GO:0016021 | |

| | | | |
|---|---|---|---|
| TRINITY_DN19617_c0_g1_i4_len_1291_268638.p1_type_5prime_partial_len_226 | ---NA--- | | |
| TRINITY_DN15841_c0_g1_i1_len_680_239499.p1_type_internal_len_227 | ---NA--- | | |
| TRINITY_DN17815_c0_g1_i1_len_894_197557.p1_type_5prime_partial_len_226 | XP_018027616.1 1-acyl-sn-glycerol-3-phosphate acyltransferase alpha-like | | |
| TRINITY_DN23437_c1_g1_i3_len_1137_159712.p2_type_complete_len_226 | XP_018009219.1 putative glycine-rich cell wall structural protein 1 | F:GO:0030246 | |
| TRINITY_DN8824_c0_g1_i1_len_676_239544.p1_type_internal_len_226 | XP_015431024.1 ectopic P granules protein 5 homolog | | |
| TRINITY_DN11228_c0_g1_i1_len_676_239549.p1_type_internal_len_226 | XP_012257980.1codanin-1 isoform X3 | | |
| TRINITY_DN1625_c0_g1_i1_len_784_198804.p1_type_5prime_partial_len_225 | XP_019636261.1 TBC1 domain family member 2B-like isoform X1 | | |
| TRINITY_DN16487_c0_g1_i4_len_677_273059.p1_type_internal_len_226 | EDO36870.1predicted protein | | |
| TRINITY_DN25962_c0_g1_i2_len_1191_195027.p1_type_5prime_partial_len_225 | ---NA--- | | |
| TRINITY_DN26101_c0_g1_i2_len_2231_190159.p2_type_complete_len_225 | ---NA--- | | |
| TRINITY_DN19821_c0_g1_i1_len_776_198912.p1_type_3prime_partial_len_226 | XP_018010863.1 FGGY carbohydrate kinase domain-containing protein-like | | |
| TRINITY_DN25235_c1_g3_i2_len_676_239548.p1_type_internal_len_226 | ABB73282.1reverse transcriptase | F:GO:0003964; P:GO:0006278 | EC:2.7.7.49 |
| TRINITY_DN7761_c0_g1_i1_len_679_239516.p1_type_internal_len_226 | XP_018027655.1 cyclin-G-associated kinase-like | F:GO:0004672; F:GO:0005524; P:GO:0006468 | |
| TRINITY_DN17732_c0_g1_i1_len_678_65945.p1_type_internal_len_226 | XP_018024833.1PREDICTED: uncharacterized protein LOC108680513 | | |
| TRINITY_DN26200_c0_g1_i1_len_675_239565.p1_type_internal_len_226 | XP_018019219.1 thyroid adenoma-associated protein homolog | | |
| TRINITY_DN19965_c0_g1_i1_len_677_65962.p1_type_internal_len_226 | ---NA--- | | |

| | | | |
|---|---|---|---|
| TRINITY_DN10165_c0_g1_i1_len_676_65966.p1_type_internal_len_226 | KZS16849.1Sn1-specific diacylglycerol lipase beta | P:GO:0006629; F:GO:0016787 | |
| TRINITY_DN20474_c0_g1_i1_len_676_65970.p1_type_internal_len_226 | ---NA--- | | |
| TRINITY_DN23537_c0_g1_i5_len_1027_196333.p1_type_complete_len_225 | ACD13594.1ubiquitin-conjugating enzyme E2 | F:GO:0005524; F:GO:0016740 | |
| TRINITY_DN30117_c0_g1_i1_len_3688_187779.p1_type_complete_len_1008 | XP_021915515.1sorting nexin-25 isoform X2 | C:GO:0016020; C:GO:0016021; P:GO:0030512; F:GO:0035091 | |
| 1323\|_\|c1472_f3p7_3022_isoform_c1472_full_length_coverage_3_non_full_length_coverage_7_isoform_length_3022_27800 | ---NA--- | | |
| TRINITY_DN29429_c0_g1_i2_len_3708_187768.p1_type_5prime_partial_len_1007 | XP_024938616.1uncharacterized protein LOC107265616 isoform X4 | C:GO:0016020; C:GO:0016021 | |
| TRINITY_DN26320_c0_g1_i1_len_4754_187215.p1_type_5prime_partial_len_1007 | XP_023714733.1vinculin isoform X4 | F:GO:0005198; P:GO:0007155; C:GO:0015629; F:GO:0051015 | |
| TRINITY_DN30277_c1_g1_i1_len_6785_186831.p1_type_complete_len_1007 | XP_021921786.1ecotropic viral integration site 5 ortholog isoform X3 | | |
| TRINITY_DN23025_c0_g1_i1_len_3611_153848.p1_type_5prime_partial_len_1006 | XP_021926837.1zinc finger protein 567-like | F:GO:0003676 | |
| TRINITY_DN28416_c0_g1_i1_len_3238_188202.p1_type_5prime_partial_len_1006 | XP_018026721.1 serine-rich adhesin for platelets-like | F:GO:0003677; F:GO:0004842; C:GO:0005634; P:GO:0006281; P:GO:0006974; F:GO:0008270; P:GO:0016567 | |
| 1330\|_\|c871_f10p33_3017_isoform_c871_full_length_coverage_10_non_full_length_coverage_33_isoform_length_3017_27649 | XP_018021754.1 replication protein A 70 kDa DNA-binding subunit-like isoform X1 | F:GO:0003676; P:GO:0006259 | |
| 1331\|_\|c2228_f2p7_3015_isoform_c2228_full_length_coverage_2_non_full_length_coverage_7_isoform_length_3015_28267 | ---NA--- | | |
| TRINITY_DN30362_c0_g1_i2_len_3545_187891.p1_type_5prime_partial_len_1005 | ---NA--- | | |
| TRINITY_DN26677_c0_g1_i1_len_679_239517.p1_type_internal_len_226 | XP_023718620.1post-GPI attachment to proteins factor 6-like | C:GO:0016020; C:GO:0016021 | |

| | | | |
|---|---|---|---|
| TRINITY_DN10734_c0_g1_i1_len_675_239556.p1_type_internal_len_226 | KDR23187.1D-glucuronyl C5-epimerase | P:GO:0015012; F:GO:0047464 | |
| TRINITY_DN29182_c0_g1_i1_len_677_239538.p1_type_internal_len_226 | XP_018011576.1PREDICTED: uncharacterized protein LOC108668835 | | |
| TRINITY_DN12790_c0_g1_i2_len_1098_195720.p1_type_3prime_partial_len_226 | ---NA--- | | |
| TRINITY_DN20822_c0_g1_i1_len_1042_196165.p1_type_complete_len_225 | KDR07158.1Phospholipid scramblase 2 | | |
| TRINITY_DN20217_c0_g1_i1_len_677_129269.p1_type_internal_len_226 | XP_018019054.1 alpha-L-fucosidase-like | | |
| TRINITY_DN16254_c0_g1_i2_len_999_33502.p1_type_5prime_partial_len_225 | ACD13590.1dynactin subunit 5 | | |
| TRINITY_DN11619_c0_g1_i1_len_1007_63081.p1_type_5prime_partial_len_225 | AQW41372.1peroxiredoxin | C:GO:0005623; P:GO:0045454; F:GO:0051920; P:GO:0055114; P:GO:0098869 | EC:1.11.1.15 |
| TRINITY_DN2207_c0_g1_i1_len_679_200324.p1_type_internal_len_226 | ---NA--- | | |
| TRINITY_DN10800_c0_g1_i1_len_739_199459.p1_type_3prime_partial_len_226 | ---NA--- | | |
| TRINITY_DN18826_c0_g1_i2_len_964_196865.p1_type_3prime_partial_len_226 | ---NA--- | | |
| TRINITY_DN4032_c0_g1_i1_len_709_128921.p1_type_5prime_partial_len_225 | XP_022084530.1zinc finger matrin-type protein 2-like | | |
| TRINITY_DN21076_c0_g1_i1_len_969_236368.p1_type_complete_len_225 | XP_018006370.1 oxidoreductase HTATIP2-like | C:GO:0005737; P:GO:0051170 | |
| TRINITY_DN32831_c0_g1_i1_len_800_64596.p1_type_5prime_partial_len_225 | ---NA--- | | |
| TRINITY_DN7131_c0_g1_i1_len_935_236662.p1_type_complete_len_225 | ---NA--- | | |
| TRINITY_DN4413_c0_g1_i1_len_676_200380.p1_type_internal_len_226 | ---NA--- | | |
| TRINITY_DN1677_c0_g1_i1_len_677_200364.p1_type_internal_len_226 | XP_018018204.1 ubiquitin carboxyl-terminal hydrolase 36-like | | |

| | | | |
|---|---|---|---|
| TRINITY_DN16523_c0_g1_i1_len_677_200356.p1_type_internal_len_226 | XP_011330349.1 myotubularin-related protein 3 isoform X2 | P:GO:0016311; F:GO:0016791 | |
| TRINITY_DN40816_c0_g1_i1_len_677_200346.p1_type_internal_len_226 | XP_018014662.1 teneurin-m-like | | |
| TRINITY_DN17189_c0_g1_i1_len_678_200334.p1_type_internal_len_226 | ---NA--- | | |
| TRINITY_DN6930_c0_g1_i1_len_679_200328.p1_type_internal_len_226 | ---NA--- | | |
| TRINITY_DN24267_c0_g1_i1_len_678_301364.p1_type_internal_len_226 | ---NA--- | | |
| TRINITY_DN21018_c1_g1_i1_len_934_161217.p1_type_complete_len_224 | XP_020610519.1UDP-N-acetylglucosamine transferase subunit ALG14 homolog isoform X1 | P:GO:0006488; F:GO:0016740 | |
| TRINITY_DN30887_c0_g1_i1_len_674_239578.p1_type_internal_len_225 | AJO70197.1sodium-calcium exchanger 3 | F:GO:0005432; P:GO:0006816; P:GO:0007154; C:GO:0016021; P:GO:0035725 | |
| TRINITY_DN22993_c0_g1_i1_len_2051_123029.p2_type_complete_len_224 | KPM09896.1carboxypeptidase-like protein 1 | F:GO:0004180; P:GO:0006508; F:GO:0016787 | |
| TRINITY_DN20226_c0_g1_i3_len_1246_194624.p1_type_5prime_partial_len_224 | ---NA--- | | |
| TRINITY_DN7091_c0_g1_i1_len_674_239590.p1_type_internal_len_225 | ---NA--- | | |
| TRINITY_DN11192_c0_g1_i1_len_673_239594.p1_type_internal_len_225 | ---NA--- | | |
| TRINITY_DN41476_c0_g1_i1_len_674_200394.p1_type_internal_len_225 | ---NA--- | | |
| TRINITY_DN15434_c0_g1_i1_len_675_200384.p1_type_internal_len_225 | XP_018011574.1 hemocytin-like | | |
| TRINITY_DN14471_c0_g1_i1_len_776_34992.p1_type_5prime_partial_len_224 | XP_012274745.1protein tyrosine phosphatase type IVA 1 | | |
| TRINITY_DN28631_c0_g1_i1_len_674_200412.p1_type_internal_len_225 | KXJ05854.1RNA-directed DNA polymerase from mobile element jockey | F:GO:0003964; P:GO:0006278 | |

| | | | |
|---|---|---|---|
| TRINITY_DN134_c0_g1_i1_len_674_200405.p1_type_internal_len_225 | XP_018018427.1 rho GTPase-activating protein 25-like | F:GO:0005096; P:GO:0007264; F:GO:0017160; P:GO:0043547 | |
| TRINITY_DN31126_c0_g1_i1_len_674_239574.p2_type_internal_len_225 | ---NA--- | | |
| TRINITY_DN35422_c0_g1_i1_len_675_239571.p1_type_internal_len_225 | ---NA--- | | |
| TRINITY_DN22454_c0_g1_i2_len_1769_59872.p2_type_5prime_partial_len_224 | AWM95782.1type 1 phosphodiesterase | F:GO:0004114; P:GO:0007165; F:GO:0046872 | EC:3.1.4.17 |
| TRINITY_DN37009_c0_g1_i1_len_843_198105.p1_type_5prime_partial_len_224 | ---NA--- | | |
| TRINITY_DN21897_c0_g1_i1_len_3172_91077.p1_type_complete_len_224 | ---NA--- | | |
| TRINITY_DN18820_c0_g1_i1_len_780_128206.p1_type_5prime_partial_len_224 | ---NA--- | | |
| TRINITY_DN7463_c0_g1_i1_len_674_273090.p1_type_internal_len_225 | XP_016922044.1 histone deacetylase 4-like | C:GO:0000118; F:GO:0032041; F:GO:0046872; P:GO:0070932 | EC:3.5.1.98 |
| TRINITY_DN20941_c0_g1_i1_len_775_198917.p1_type_5prime_partial_len_224 | XP_018015532.1 ras-specific guanine nucleotide-releasing factor RalGPS1-like | | |
| TRINITY_DN11372_c0_g1_i1_len_832_198242.p1_type_3prime_partial_len_225 | AMJ52357.1cullin 4 | P:GO:0006511; C:GO:0031461; F:GO:0031625 | |
| TRINITY_DN14160_c0_g1_i2_len_673_273112.p1_type_internal_len_225 | XP_022248104.1alpha-N-acetylglucosaminidase-like, partial | | |
| TRINITY_DN16035_c0_g1_i1_len_1404_31776.p1_type_5prime_partial_len_224 | ACY66440.1putative myosin regulatory light chain 2 smooth muscle | F:GO:0005509 | |
| TRINITY_DN15114_c0_g1_i3_len_676_163867.p1_type_internal_len_225 | XP_023323798.1protein msta-like | F:GO:0046872 | |
| TRINITY_DN24009_c0_g1_i1_len_1684_192156.p1_type_5prime_partial_len_223 | XP_021918711.1matrix metalloproteinase-2-like isoform X4 | | |
| TRINITY_DN18158_c0_g1_i1_len_695_301175.p1_type_5prime_partial_len_223 | AKC35142.1vacuolar-H-ATPase H | C:GO:0000221; P:GO:0015991; F:GO:0046961 | EC:3.6.1.3; EC:3.6.1.15 |
| TRINITY_DN6078_c0_g1_i1_len_682_65892.p1_type_5prime_partial_len_223 | XP_019869459.1 galactosylgalactosylxylosylprotein 3-beta-glucuronosyltransferase P | C:GO:0005794; C:GO:0016020; F:GO:0016740 | |

| | | | |
|---|---|---|---|
| TRINITY_DN13404_c0_g1_i1_len_678_5971.p1_type_5prime_partial_len_223 | KDR16019.1Autophagy-related protein 101 | P:GO:0006914 | |
| TRINITY_DN13305_c0_g1_i1_len_669_66061.p1_type_internal_len_224 | ALH22586.1fibrinogen-related protein isoform 1 | | |
| TRINITY_DN20854_c0_g1_i3_len_1465_93450.p2_type_5prime_partial_len_223 | ---NA--- | | |
| TRINITY_DN12874_c0_g1_i1_len_672_98406.p1_type_internal_len_224 | XP_023718751.1zinc finger Ran-binding domain-containing protein 2 | F:GO:0003723; C:GO:0005634; F:GO:0046872 | |
| TRINITY_DN15136_c0_g1_i1_len_920_197297.p1_type_5prime_partial_len_223 | XP_018014427.1 oxysterol-binding protein-related protein 9-like isoform X4 | | |
| TRINITY_DN19710_c0_g1_i1_len_671_301440.p1_type_internal_len_224 | ---NA--- | | |
| TRINITY_DN31142_c0_g1_i1_len_3684_187782.p1_type_complete_len_223 | ---NA--- | | |
| TRINITY_DN31344_c0_g1_i1_len_671_239631.p1_type_internal_len_224 | ---NA--- | | |
| TRINITY_DN13742_c0_g1_i1_len_670_239635.p1_type_internal_len_224 | ---NA--- | | |
| TRINITY_DN22136_c0_g2_i1_len_1101_125936.p1_type_5prime_partial_len_223 | EEC06908.1flavonol reductase/cinnamoyl-CoA reductase, putative | F:GO:0016491 | |
| TRINITY_DN36572_c0_g1_i1_len_670_239638.p1_type_internal_len_224 | BAL49600.1NADPH oxidase | | |
| TRINITY_DN11916_c0_g1_i1_len_672_239619.p1_type_internal_len_224 | EEB12539.1Rho GTPase regulating protein, putative | P:GO:0007165 | |
| TRINITY_DN3019_c0_g1_i1_len_694_98151.p1_type_5prime_partial_len_223 | ---NA--- | | |
| TRINITY_DN27961_c3_g1_i1_len_713_199801.p1_type_5prime_partial_len_223 | ---NA--- | | |
| TRINITY_DN14947_c0_g1_i2_len_1129_235189.p1_type_complete_len_223 | ---NA--- | | |
| TRINITY_DN19036_c0_g1_i1_len_1503_233008.p1_type_5prime_partial_len_223 | ANR94954.1Ras protein | F:GO:0003924; F:GO:0005525; P:GO:0007165; C:GO:0016020 | EC:3.6.1.15 |

| | | | |
|---|---|---|---|
| TRINITY_DN8988_c0_g1_i1_len_670_239639.p1_type_internal_len_224 | XP_018022944.1 carboxypeptidase D-like | | |
| TRINITY_DN14807_c0_g1_i2_len_990_270268.p2_type_5prime_partial_len_223 | ---NA--- | | |
| TRINITY_DN13355_c0_g1_i1_len_669_239657.p1_type_internal_len_224 | XP_012150676.1 histone deacetylase 6 isoform X4 | F:GO:0003676; F:GO:0008270; F:GO:0016787 | |
| TRINITY_DN23616_c0_g1_i1_len_1444_193401.p2_type_complete_len_223 | ---NA--- | | |
| TRINITY_DN20035_c0_g1_i2_len_1382_193752.p1_type_complete_len_223 | XP_026461519.1carnitine O-palmitoyltransferase 1, liver isoform-like isoform X2 | | |
| TRINITY_DN8851_c0_g1_i1_len_950_270539.p1_type_5prime_partial_len_223 | ---NA--- | | |
| TRINITY_DN10585_c0_g1_i1_len_743_163001.p1_type_5prime_partial_len_223 | XP_018007540.1 innexin inx2-like | | |
| TRINITY_DN9467_c0_g1_i1_len_670_163930.p1_type_internal_len_224 | ---NA--- | | |
| TRINITY_DN31291_c0_g1_i1_len_670_200459.p1_type_internal_len_224 | ---NA--- | | |
| TRINITY_DN13420_c0_g1_i1_len_671_200451.p1_type_internal_len_224 | ---NA--- | | |
| TRINITY_DN40985_c0_g1_i1_len_671_200441.p1_type_internal_len_224 | KDR19041.1SID1 transmembrane family member 1 | C:GO:0016020; C:GO:0016021; P:GO:0033227; F:GO:0051033 | |
| TRINITY_DN14708_c0_g1_i2_len_807_162350.p1_type_complete_len_223 | ---NA--- | | |
| TRINITY_DN22514_c0_g1_i1_len_935_197133.p1_type_5prime_partial_len_223 | ---NA--- | | |
| TRINITY_DN28052_c0_g1_i1_len_1600_192563.p1_type_complete_len_223 | ---NA--- | | |
| TRINITY_DN22546_c0_g1_i1_len_997_236136.p1_type_complete_len_223 | ---NA--- | | |
| TRINITY_DN16975_c0_g1_i1_len_1015_235989.p1_type_complete_len_223 | ---NA--- | | |

| | | | |
|---|---|---|---|
| TRINITY_DN23834_c0_g1_i1_len_2546_122416.p3_type_3prime_partial_len_224 | ---NA--- | | |
| TRINITY_DN20938_c0_g1_i1_len_1327_233895.p1_type_5prime_partial_len_223 | XP_018020277.1 protein Mpv17-like isoform X1 | C:GO:0016020; C:GO:0016021 | |
| TRINITY_DN12004_c0_g1_i1_len_857_64087.p1_type_complete_len_222 | XP_018011515.1 thioredoxin domain-containing protein 9-like | C:GO:0005623; P:GO:0045454 | |
| TRINITY_DN10358_c0_g1_i1_len_773_198961.p1_type_complete_len_222 | XP_018016167.1 charged multivesicular body protein 3-like | | |
| TRINITY_DN21384_c0_g1_i1_len_2054_230952.p1_type_5prime_partial_len_222 | ---NA--- | | |
| TRINITY_DN17280_c0_g1_i3_len_791_97107.p1_type_5prime_partial_len_222 | ---NA--- | | |
| TRINITY_DN165_c0_g1_i1_len_755_199206.p1_type_5prime_partial_len_222 | ---NA--- | | |
| TRINITY_DN1714_c0_g1_i1_len_667_98464.p1_type_internal_len_223 | XP_013420234.1hemicentin-1 | F:GO:0005509 | |
| TRINITY_DN6880_c0_g1_i1_len_666_163979.p1_type_internal_len_223 | EDO32819.1predicted protein | | |
| TRINITY_DN8983_c0_g1_i1_len_820_162214.p1_type_complete_len_222 | ---NA--- | | |
| TRINITY_DN22761_c0_g1_i1_len_956_236478.p1_type_complete_len_222 | XP_018013589.1 flavin reductase (NADPH)-like | | |
| TRINITY_DN17968_c0_g1_i1_len_987_236215.p1_type_complete_len_222 | XP_015918228.1transmembrane protein 98 isoform X1 | | |
| TRINITY_DN23884_c0_g1_i1_len_811_198482.p1_type_5prime_partial_len_222 | ---NA--- | | |
| TRINITY_DN30341_c0_g1_i1_len_853_237403.p1_type_5prime_partial_len_222 | XP_018354758.1 vacuolar protein sorting-associated protein 16 homolog isoform X2 | | |
| TRINITY_DN19246_c0_g1_i1_len_1046_196141.p1_type_complete_len_222 | AJD14729.1glutathione s-transferase | F:GO:0004364; C:GO:0005739; F:GO:0016740 | |
| TRINITY_DN6328_c0_g1_i1_len_669_239665.p1_type_internal_len_223 | ACU30155.1hypoxia inducible factor 1 beta | F:GO:0003700; C:GO:0005634; C:GO:0005667; C:GO:0005737; P:GO:0006355; F:GO:0046983 | |

| | | | |
|---|---|---|---|
| TRINITY_DN5635_c0_g1_i1_len_710_272694.p1_type_5prime_partial_len_222 | XP_014612466.1 protein LSM12 homolog | | |
| TRINITY_DN3650_c0_g1_i1_len_668_239668.p1_type_internal_len_223 | ---NA--- | | |
| TRINITY_DN9889_c0_g1_i1_len_1323_194121.p1_type_complete_len_222 | XP_013775655.1DNA-directed RNA polymerase III subunit RPC8-like | F:GO:0001056; C:GO:0005666; P:GO:0006384 | EC:2.7.7.6 |
| TRINITY_DN16954_c0_g1_i3_len_1073_33113.p1_type_complete_len_222 | ---NA--- | | |
| 1333\|_\|c11962_f2p9_3015_isoform_c11962_full_length_coverage_2_non_full_length_coverage_9_isoform_length_3015_26129 | ---NA--- | | |
| TRINITY_DN30909_c0_g1_i1_len_3377_188052.p1_type_complete_len_1005 | ---NA--- | | |
| 1335\|_\|c1102_f9p30_3014_isoform_c1102_full_length_coverage_9_non_full_length_coverage_30_isoform_length_3014_25264 | XP_018009272.1 vacuolar protein sorting-associated protein 53 homolog isoform X1 | | |
| 1338\|_\|c1673_f2p6_3012_isoform_c1673_full_length_coverage_2_non_full_length_coverage_6_isoform_length_3012_26324 | XP_023324004.1protein asteroid-like | | |
| 1339\|_\|c14257_f2p34_3012_isoform_c14257_full_length_coverage_2_non_full_length_coverage_34_isoform_length_3012_26233 | XP_014221375.1hypoxia up-regulated protein 1 | F:GO:0000166 | |
| 1340\|_\|c1369_f3p6_3010_isoform_c1369_full_length_coverage_3_non_full_length_coverage_6_isoform_length_3010_25912 | XP_013086899.1PREDICTED: LOW QUALITY PROTEIN: uncharacterized protein LOC106071357 | F:GO:0003677 | |
| TRINITY_DN20891_c0_g1_i1_len_3300_154058.p1_type_complete_len_1003 | XP_023701579.1ribosomal protein S6 kinase alpha-5-like isoform X1 | | |
| 1342\|_\|c1291_f10p18_3009_isoform_c1291_full_length_coverage_10_non_full_length_coverage_18_isoform_length_3009_27732 | ---NA--- | | |
| TRINITY_DN28583_c0_g1_i1_len_3163_188299.p1_type_complete_len_1003 | KYB26661.1Angiogenic factor with G patch and FHA domains 1-like Protein | F:GO:0003676 | |

| | | | |
|---|---|---|---|
| TRINITY_DN24631_c0_g1_i1_len_3596_187853.p1_type_5prime_partial_len_1002 | XP_006614264.1 receptor-type tyrosine-protein phosphatase alpha-like isoform X2 | F:GO:0004721; P:GO:0006470; F:GO:0016740 | EC:3.1.3.16 |
| TRINITY_DN20648_c0_g1_i4_len_1138_2877.p1_type_complete_len_222 | XP_018016270.1PREDICTED: uncharacterized protein LOC108673015 | | |
| TRINITY_DN2957_c0_g1_i1_len_686_200216.p1_type_5prime_partial_len_222 | DAA13314.1TPA: hypothetical protein BOS_25275 | C:GO:0016020; C:GO:0016021 | |
| TRINITY_DN17943_c0_g1_i1_len_666_273199.p1_type_internal_len_223 | ---NA--- | | |
| TRINITY_DN23353_c0_g1_i1_len_667_273195.p1_type_internal_len_223 | XP_020296662.1zinc finger protein 2 homolog | F:GO:0003676 | |
| TRINITY_DN14606_c0_g1_i1_len_669_239658.p1_type_internal_len_223 | XP_015904214.1inositol-trisphosphate 3-kinase homolog isoform X3 | | |
| TRINITY_DN28628_c0_g1_i1_len_669_239662.p1_type_internal_len_223 | XP_021930705.1F-box only protein 33 | C:GO:0019005; P:GO:0031146 | |
| TRINITY_DN24007_c0_g1_i2_len_1635_232382.p2_type_5prime_partial_len_222 | EFN79339.1Phospholipase A2 isozyme PA4 | F:GO:0004623; C:GO:0005576; P:GO:0006644; P:GO:0050482 | |
| TRINITY_DN12418_c0_g1_i1_len_847_161966.p1_type_5prime_partial_len_222 | KZS14344.1Dr1-associated corepressor | F:GO:0003712; C:GO:0005634; F:GO:0046982; P:GO:1903506 | |
| TRINITY_DN9131_c0_g1_i2_len_896_270964.p2_type_5prime_partial_len_222 | AHI59453.1farnesoic acid O-methyltransferase long form | F:GO:0008168; P:GO:0032259 | |
| TRINITY_DN23907_c0_g1_i3_len_921_63620.p1_type_5prime_partial_len_222 | XP_022258887.1transmembrane protein 8B-like | | |
| TRINITY_DN5211_c0_g1_i1_len_689_200167.p1_type_5prime_partial_len_222 | XP_018008374.1 myotubularin-related protein 2-like isoform X2 | F:GO:0004725; P:GO:0035335 | EC:3.1.3.16; EC:3.1.3.48 |
| TRINITY_DN12850_c0_g2_i1_len_668_239679.p1_type_internal_len_223 | ---NA--- | | |
| TRINITY_DN16708_c0_g1_i1_len_667_200512.p1_type_internal_len_223 | XP_018013573.1 protein msta-like | | |
| TRINITY_DN12785_c0_g1_i1_len_668_200500.p2_type_internal_len_223 | AAD40313.1chitinase 1 | F:GO:0004553; C:GO:0005576; P:GO:0005975; P:GO:0006030; F:GO:0008061 | |
| TRINITY_DN21313_c0_g1_i1_len_806_198529.p1_type_3prime_partial_len_223 | XP_013395886.1SET and MYND domain-containing protein 4-like | | |

| | | | |
|---|---|---|---|
| TRINITY_DN30791_c0_g1_i1_len_667_239694.p1_type_internal_len_223 | ---NA--- | | |
| TRINITY_DN13755_c0_g1_i1_len_669_200487.p1_type_internal_len_223 | XP_023244479.1tyrosine-protein kinase CSK-like | | |
| TRINITY_DN12666_c0_g1_i2_len_667_301493.p1_type_internal_len_223 | XP_018019004.1 oxysterol-binding protein-related protein 11-like | | |
| TRINITY_DN19570_c0_g1_i1_len_670_200465.p1_type_internal_len_223 | PIK53202.1hypothetical protein BSL78_09912 | F:GO:0003676; F:GO:0003700; P:GO:0006355; P:GO:0015074 | |
| TRINITY_DN17174_c0_g1_i1_len_668_200492.p1_type_internal_len_223 | ---NA--- | | |
| TRINITY_DN31453_c0_g1_i1_len_669_6077.p1_type_internal_len_223 | ---NA--- | | |
| TRINITY_DN31380_c0_g1_i1_len_714_199784.p1_type_3prime_partial_len_223 | NP_919347.1polycomb complex protein BMI-1-A | F:GO:0005488; P:GO:0006325; P:GO:0021549; C:GO:0031519; P:GO:0035701; P:GO:0045814; P:GO:0045892; P:GO:0060215 | |
| TRINITY_DN4499_c0_g1_i1_len_669_200490.p1_type_internal_len_223 | XP_018013869.1PREDICTED: uncharacterized protein LOC108670892, partial | | |
| TRINITY_DN2471_c0_g1_i1_len_663_239747.p1_type_internal_len_222 | PNF37943.1Echinoderm microtubule-associated protein-like 1 | | |
| TRINITY_DN16300_c0_g1_i1_len_663_239745.p1_type_internal_len_222 | ---NA--- | | |
| TRINITY_DN17654_c0_g1_i1_len_665_239730.p1_type_internal_len_222 | ---NA--- | | |
| TRINITY_DN10923_c0_g1_i1_len_667_239685.p1_type_internal_len_222 | XP_021944555.1echinoderm microtubule-associated protein-like 2 | | |
| TRINITY_DN8304_c0_g1_i1_len_665_200537.p1_type_internal_len_222 | KZS20145.1Protein SZT2 | C:GO:0005777 | |
| TRINITY_DN21423_c0_g1_i1_len_1466_93446.p1_type_complete_len_221 | XP_018008984.1 vacuolar protein sorting-associated protein 28 homolog | C:GO:0000813; P:GO:0043328 | |
| TRINITY_DN6419_c0_g1_i1_len_665_200541.p1_type_internal_len_222 | ---NA--- | | |

| | | | |
|---|---|---|---|
| TRINITY_DN37293_c0_g1_i1_len_664_200554.p1_type_internal_len_222 | XP_023715449.1zinc finger and SCAN domain-containing protein 2-like isoform X2 | F:GO:0003676 | |
| TRINITY_DN39461_c0_g1_i1_len_664_200555.p1_type_internal_len_222 | ---NA--- | | |
| TRINITY_DN9153_c0_g1_i1_len_664_200559.p1_type_internal_len_222 | ---NA--- | | |
| TRINITY_DN20428_c0_g1_i1_len_2420_265563.p2_type_complete_len_221 | XP_018008378.1 cysteine--tRNA ligase, cytoplasmic-like | F:GO:0000166; F:GO:0004812; F:GO:0004817; F:GO:0005524; P:GO:0006418; P:GO:0006423; F:GO:0016874 | |
| TRINITY_DN18863_c0_g1_i1_len_663_200563.p1_type_internal_len_222 | ---NA--- | | |
| TRINITY_DN18857_c0_g1_i1_len_1309_158708.p1_type_5prime_partial_len_221 | XP_002120798.1ADP-ribosylation factor-like protein 2 | F:GO:0005525 | |
| TRINITY_DN10828_c0_g1_i1_len_715_238972.p1_type_complete_len_221 | ---NA--- | | |
| TRINITY_DN4532_c0_g1_i1_len_824_237701.p1_type_5prime_partial_len_221 | ---NA--- | | |
| TRINITY_DN21755_c0_g1_i1_len_1507_157779.p1_type_complete_len_221 | XP_013419337.1esterase OVCA2-like | | |
| TRINITY_DN17962_c0_g1_i2_len_792_238042.p1_type_complete_len_221 | XP_018025829.1 osteoclast-stimulating factor 1-like | | |
| TRINITY_DN10416_c0_g1_i1_len_748_128479.p1_type_complete_len_221 | XP_014668416.1 phosphatidylinositol-glycan biosynthesis class X protein-like | | |
| TRINITY_DN12499_c0_g1_i1_len_1234_94336.p2_type_5prime_partial_len_221 | XP_019555351.1 4-hydroxyphenylpyruvate dioxygenase-like | F:GO:0003868; P:GO:0009072; F:GO:0046872; P:GO:0055114 | EC:1.13.11; EC:1.13.11.27 |
| TRINITY_DN24852_c6_g1_i2_len_759_65005.p1_type_5prime_partial_len_221 | XP_024417670.1cancer-related nucleoside-triphosphatase | P:GO:0016311; F:GO:0098519 | |
| TRINITY_DN16030_c0_g1_i1_len_998_236133.p1_type_complete_len_221 | ---NA--- | | |
| TRINITY_DN23815_c0_g1_i1_len_955_196942.p1_type_3prime_partial_len_222 | ---NA--- | | |

| | | | |
|---|---|---|---|
| TRINITY_DN22106_c0_g1_i1_len_950_196990.p1_type_complete_len_221 | XP_022194911.1esterase OVCA2-like isoform X1 | | |
| TRINITY_DN16863_c0_g1_i2_len_664_36137.p1_type_internal_len_222 | ---NA--- | | |
| TRINITY_DN18578_c0_g1_i1_len_685_272978.p1_type_5prime_partial_len_221 | ---NA--- | | |
| TRINITY_DN13348_c0_g1_i2_len_664_98517.p1_type_internal_len_222 | XP_018016499.1 cytosolic 5'-nucleotidase 3-like isoform X1 | | |
| TRINITY_DN12505_c0_g1_i1_len_666_163974.p1_type_internal_len_222 | ---NA--- | | |
| TRINITY_DN25368_c0_g1_i1_len_4179_228098.p1_type_5prime_partial_len_221 | XP_018023522.1PREDICTED: uncharacterized protein LOC108679414 | C:GO:0016020; C:GO:0016021 | |
| TRINITY_DN20663_c0_g1_i1_len_1200_194966.p1_type_5prime_partial_len_221 | ---NA--- | | |
| TRINITY_DN21423_c0_g1_i1_len_1207_194904.p1_type_5prime_partial_len_221 | ---NA--- | | |
| TRINITY_DN2912_c0_g1_i1_len_804_198545.p1_type_complete_len_221 | ---NA--- | | |
| TRINITY_DN13734_c0_g1_i2_len_665_66112.p1_type_internal_len_222 | ---NA--- | | |
| TRINITY_DN12264_c0_g1_i2_len_665_66122.p1_type_internal_len_222 | ---NA--- | | |
| TRINITY_DN21732_c0_g1_i1_len_1270_234241.p1_type_complete_len_220 | ---NA--- | | |
| TRINITY_DN4155_c0_g1_i1_len_768_199012.p1_type_5prime_partial_len_220 | XP_018023449.1PREDICTED: uncharacterized protein LOC108679354 isoform X2 | | |
| TRINITY_DN25725_c0_g1_i1_len_661_164050.p1_type_internal_len_221 | XP_011669293.1 ras GTPase-activating-like protein IQGAP1 isoform X1 | | |
| TRINITY_DN23799_c0_g1_i2_len_1034_196256.p1_type_3prime_partial_len_221 | ---NA--- | | |
| TRINITY_DN26290_c0_g1_i2_len_2017_190842.p1_type_complete_len_220 | XP_018023619.1 myotubularin-related protein DDB_G0290005-like isoform X4 | | |

| | | | |
|---|---|---|---|
| TRINITY_DN27991_c0_g1_i1_len_662_98545.p1_type_internal_len_221 | KFM57926.1A disintegrin and metalloproteinase with thrombospondin motifs 16 | F:GO:0004222; P:GO:0006508; P:GO:0007229; F:GO:0008237 | |
| TRINITY_DN22103_c0_g1_i1_len_808_127985.p1_type_complete_len_220 | ---NA--- | | |
| TRINITY_DN3319_c0_g1_i1_len_810_162325.p1_type_5prime_partial_len_220 | ---NA--- | | |
| TRINITY_DN10755_c0_g1_i1_len_660_239781.p1_type_internal_len_221 | AYD78165.1nuclear respiratory factor-1/DNA-binding protein Ewg isoform | F:GO:0003677; F:GO:0003700; P:GO:0006357 | |
| TRINITY_DN6420_c0_g1_i1_len_661_239765.p1_type_internal_len_221 | ---NA--- | | |
| TRINITY_DN2818_c0_g1_i1_len_661_239763.p1_type_internal_len_221 | PIK51903.1hypothetical protein BSL78_11202 | F:GO:0003676; F:GO:0003700; P:GO:0006355; P:GO:0015074 | |
| TRINITY_DN5813_c0_g1_i2_len_662_239757.p1_type_internal_len_221 | ---NA--- | | |
| TRINITY_DN9451_c0_g1_i1_len_663_239749.p1_type_internal_len_221 | ---NA--- | | |
| TRINITY_DN16453_c0_g1_i2_len_662_129465.p1_type_internal_len_221 | ---NA--- | | |
| TRINITY_DN17092_c0_g1_i1_len_970_33654.p1_type_5prime_partial_len_220 | AHF21000.1C-type lectin | F:GO:0030246 | |
| TRINITY_DN7080_c0_g1_i1_len_704_35680.p1_type_3prime_partial_len_221 | ---NA--- | | |
| TRINITY_DN25738_c0_g1_i1_len_1283_194372.p1_type_complete_len_220 | XP_026272850.1ras-related protein Rab-43 | | |
| TRINITY_DN16327_c0_g1_i2_len_727_35411.p1_type_5prime_partial_len_220 | XP_017047122.1 39S ribosomal protein L11, mitochondrial | F:GO:0003735; C:GO:0005840; P:GO:0006412 | |
| TRINITY_DN1326_c0_g1_i1_len_663_200570.p1_type_internal_len_221 | KRT83033.1hypothetical protein AMK59_4612, partial | F:GO:0003700; P:GO:0006355 | |
| TRINITY_DN1915_c0_g1_i1_len_663_200577.p1_type_internal_len_221 | XP_014064265.1 smoothened homolog | F:GO:0004888; P:GO:0007166; P:GO:0007224; P:GO:0007275; C:GO:0016020; C:GO:0016021 | |

| | | | |
|---|---|---|---|
| TRINITY_DN37699_c0_g1_i1_len_662_200578.p1_type_internal_len_221 | XP_023239997.1host cell factor 1-like | C:GO:0048188; C:GO:0071339 | |
| TRINITY_DN21594_c0_g1_i2_len_798_198619.p1_type_complete_len_220 | XP_011702073.1 glutathione S-transferase-like | | |
| TRINITY_DN6630_c0_g1_i1_len_662_200584.p1_type_internal_len_221 | ERL96173.1hypothetical protein D910_01274, partial | | |
| TRINITY_DN31417_c0_g1_i1_len_660_200628.p1_type_internal_len_221 | XP_016836517.1 alpha-aminoadipic semialdehyde synthase, mitochondrial isoform X4 | | |
| TRINITY_DN11260_c0_g1_i1_len_660_200629.p1_type_internal_len_221 | XP_020296133.1F-box only protein 33-like | C:GO:0019005; P:GO:0031146 | |
| TRINITY_DN12061_c0_g1_i1_len_815_271621.p1_type_5prime_partial_len_220 | XP_018022505.1 protein lifeguard 1-like | C:GO:0016020 | |
| TRINITY_DN22205_c0_g1_i1_len_660_200630.p1_type_internal_len_221 | AXC43914.1chitin binding-like protein | | |
| TRINITY_DN5806_c0_g1_i1_len_815_271617.p1_type_3prime_partial_len_221 | ---NA--- | | |
| TRINITY_DN186_c0_g1_i2_len_671_129327.p1_type_5prime_partial_len_220 | ---NA--- | | |
| TRINITY_DN9047_c0_g1_i1_len_663_200564.p1_type_internal_len_221 | ---NA--- | | |
| TRINITY_DN22426_c0_g1_i1_len_1669_123781.p1_type_5prime_partial_len_220 | ASS34533.1peroxiredoxin 5 isoform 1 | C:GO:0005623; F:GO:0016491; P:GO:0045454; P:GO:0055114 | |
| TRINITY_DN16199_c0_g1_i1_len_919_161335.p2_type_5prime_partial_len_220 | ---NA--- | | |
| TRINITY_DN19284_c0_g1_i1_len_704_199929.p1_type_5prime_partial_len_220 | ---NA--- | | |
| TRINITY_DN19343_c0_g1_i1_len_1317_158658.p2_type_complete_len_219 | ---NA--- | | |
| TRINITY_DN16778_c0_g1_i1_len_889_237057.p1_type_5prime_partial_len_219 | XP_018015438.1  multidrug resistance-associated protein 1-like | | |
| TRINITY_DN21781_c0_g1_i1_len_660_301590.p1_type_internal_len_220 | ---NA--- | | |

| | | | |
|---|---|---|---|
| TRINITY_DN25130_c1_g1_i2_len_733_199541.p2_type_complete_len_219 | ---NA--- | | |
| TRINITY_DN16988_c0_g1_i1_len_663_200576.p1_type_5prime_partial_len_219 | ---NA--- | | |
| TRINITY_DN20334_c0_g1_i2_len_1400_93676.p2_type_complete_len_219 | PNF22491.1Transmembrane protein 189 | C:GO:0016021 | |
| TRINITY_DN10804_c0_g1_i1_len_1026_270057.p2_type_5prime_partial_len_219 | BAF64529.1peptidylglycine alpha-hydroxylating monooxygenase | F:GO:0005507; P:GO:0006518; C:GO:0016020; F:GO:0016715; P:GO:0055114 | EC:1.14.17 |
| TRINITY_DN21044_c0_g1_i1_len_664_129438.p1_type_5prime_partial_len_219 | XP_013792075.1MDS1 and EVI1 complex locus protein EVI1-A-like | | |
| TRINITY_DN23767_c0_g1_i1_len_663_200571.p1_type_5prime_partial_len_219 | XP_018010487.1 mediator of RNA polymerase II transcription subunit 20-like isoform X2 | C:GO:0005634 | |
| TRINITY_DN8627_c0_g1_i1_len_1194_2660.p1_type_complete_len_219 | ADF87491.1ferritin 2 | F:GO:0004322; C:GO:0005623; P:GO:0006826; P:GO:0006879; F:GO:0008199; F:GO:0016491; F:GO:0046872; P:GO:0055114 | |
| TRINITY_DN17783_c0_g1_i1_len_909_197392.p1_type_3prime_partial_len_220 | XP_023225726.1endonuclease/exonuclease/phosphatase family domain-containing protein 1-like isoform X2 | | |
| TRINITY_DN22801_c0_g1_i1_len_4065_228147.p1_type_5prime_partial_len_1002 | XP_018007859.1 importin-13-like isoform X2 | | |
| TRINITY_DN22809_c0_g1_i1_len_3104_188369.p1_type_5prime_partial_len_1002 | XP_021926859.1kinase suppressor of Ras 2 isoform X2 | F:GO:0005488; P:GO:0009987; F:GO:0016301 | |
| TRINITY_DN24843_c0_g1_i1_len_3163_228807.p1_type_complete_len_1002 | XP_018022556.1 golgin subfamily A member 4-like | P:GO:0043001 | |
| TRINITY_DN30793_c0_g1_i3_len_3009_188532.p1_type_internal_len_1003 | XP_018014576.1 pre-mRNA-splicing factor CWC22 homolog isoform X1 | F:GO:0003723 | |
| 1351\|_\|c19687_f3p16_3004_isoform_c19687_full_length_coverage_3_non_full_length_coverage_16_isoform_length_3004_28953 | XP_018008577.1 DNA excision repair protein haywire-like | F:GO:0003677; F:GO:0004003; F:GO:0005524; P:GO:0006289; P:GO:0006367; P:GO:0032508 | EC:3.6.1.3; EC:3.6.1.15 |

| | | | |
|---|---|---|---|
| TRINITY_DN20902_c3_g2_i2_len_3752_264585.p1_type_complete_len_1001 | ---NA--- | | |
| 1353\|_\|c1149_f8p10_3003_isoform_c1149_full_length_coverage_8_non_full_length_coverage_10_isoform_length_3003_27685 | XP_013780205.1LON peptidase N-terminal domain and RING finger protein 3-like | | |
| TRINITY_DN29558_c0_g1_i2_len_3043_188465.p1_type_5prime_partial_len_1001 | ---NA--- | | |
| 1355\|_\|c1406_f4p7_3002_isoform_c1406_full_length_coverage_4_non_full_length_coverage_7_isoform_length_3002_27766 | XP_018019011.1 torsin-1A-like isoform X2 | F:GO:0000166; C:GO:0005783 | |
| 1356\|_\|c2024_f2p1_3001_isoform_c2024_full_length_coverage_2_non_full_length_coverage_1_isoform_length_3001_28159 | XP_018006606.1 mucin-5AC-like | F:GO:0003676; F:GO:0003723 | |
| TRINITY_DN11326_c0_g1_i1_len_661_200611.p1_type_internal_len_220 | XP_018026491.1 cell adhesion molecule 4-like | | |
| TRINITY_DN7348_c0_g1_i1_len_776_272001.p1_type_complete_len_219 | XP_018015432.1 hypoxanthine-guanine phosphoribosyltransferase-like | | |
| TRINITY_DN32253_c0_g1_i1_len_660_200621.p1_type_internal_len_220 | XP_018021461.1 Niemann-Pick C1 protein-like | C:GO:0016020 | |
| TRINITY_DN6692_c0_g1_i1_len_660_200619.p1_type_internal_len_220 | ---NA--- | | |
| TRINITY_DN2868_c0_g1_i1_len_660_200618.p1_type_internal_len_220 | ---NA--- | | |
| TRINITY_DN18759_c0_g1_i1_len_1812_30726.p2_type_complete_len_219 | APO14259.1juvenile hormone esterase-like carboxylesterase 1 | F:GO:0016787 | |
| TRINITY_DN21807_c0_g1_i1_len_661_200610.p1_type_internal_len_220 | ---NA--- | | |
| TRINITY_DN378_c0_g1_i1_len_661_239769.p1_type_internal_len_220 | ---NA--- | | |
| TRINITY_DN27455_c0_g1_i1_len_660_239791.p1_type_internal_len_220 | ---NA--- | | |
| TRINITY_DN33702_c0_g1_i1_len_659_239802.p1_type_internal_len_220 | KDR24089.1Vacuolar protein sorting-associated protein 16-like protein | | |

| | | | |
|---|---|---|---|
| TRINITY_DN19078_c0_g1_i1_len_1212_234624.p1_type_complete_len_219 | ODN00847.1Transmembrane protein | C:GO:0016020; C:GO:0016021 | |
| TRINITY_DN6660_c0_g1_i1_len_657_129529.p1_type_internal_len_220 | ---NA--- | | |
| TRINITY_DN7268_c0_g1_i1_len_750_272253.p1_type_5prime_partial_len_219 | ---NA--- | | |
| TRINITY_DN183_c0_g1_i2_len_658_129517.p1_type_internal_len_220 | ---NA--- | | |
| TRINITY_DN18023_c0_g1_i4_len_674_163879.p1_type_5prime_partial_len_219 | ---NA--- | | |
| TRINITY_DN16429_c1_g1_i1_len_844_198098.p1_type_5prime_partial_len_219 | XP_018580067.1ADP-ribosylation factor-like protein 6-interacting protein 4 | | |
| TRINITY_DN19748_c0_g1_i1_len_796_64635.p1_type_complete_len_219 | ASK05861.1chitin-binding protein | F:GO:0042302 | |
| TRINITY_DN20665_c0_g1_i1_len_3476_97.p2_type_5prime_partial_len_219 | XP_018019486.1 myosin-IB-like | | |
| TRINITY_DN15555_c0_g1_i2_len_1443_267962.p1_type_complete_len_219 | XP_018025634.1 kynurenine formamidase-like | F:GO:0004061; P:GO:0019441 | |
| TRINITY_DN9078_c0_g1_i1_len_657_200678.p1_type_internal_len_220 | ---NA--- | | |
| TRINITY_DN33464_c0_g1_i1_len_659_200638.p2_type_internal_len_220 | XP_018014662.1 teneurin-m-like | | |
| TRINITY_DN23667_c0_g1_i1_len_2072_230888.p2_type_5prime_partial_len_219 | ---NA--- | | |
| TRINITY_DN8208_c0_g1_i1_len_659_200634.p1_type_internal_len_220 | ---NA--- | | |
| TRINITY_DN28780_c6_g1_i1_len_1481_193194.p1_type_5prime_partial_len_219 | XP_006798084.1 FAD-linked sulfhydryl oxidase ALR-like isoform X1 | | |
| TRINITY_DN16052_c0_g1_i2_len_804_198550.p1_type_3prime_partial_len_220 | ---NA--- | | |
| TRINITY_DN29068_c1_g1_i1_len_1959_191051.p1_type_5prime_partial_len_219 | ---NA--- | | |

| | | | |
|---|---|---|---|
| TRINITY_DN14476_c0_g1_i1_len_658_200658.p1_type_internal_len_220 | ---NA--- | | |
| TRINITY_DN26061_c1_g1_i8_len_1823_231662.p2_type_5prime_partial_len_218 | XP_026292635.1myc box-dependent-interacting protein 1-like | C:GO:0005737 | |
| TRINITY_DN40962_c0_g1_i1_len_655_200728.p1_type_internal_len_219 | KZS07936.1Multiple epidermal growth factor domains 8-like protein | F:GO:0005509; C:GO:0016020; C:GO:0016021 | |
| TRINITY_DN24386_c0_g1_i2_len_1115_235272.p1_type_complete_len_218 | XP_023713009.1biogenesis of lysosome-related organelles complex 1 subunit 4 isoform X2 | | |
| TRINITY_DN15722_c0_g1_i2_len_656_273329.p1_type_internal_len_219 | XP_018016943.1 decapping and exoribonuclease protein-like | | |
| TRINITY_DN15109_c0_g1_i1_len_657_239821.p1_type_internal_len_219 | ---NA--- | | |
| TRINITY_DN22525_c0_g1_i2_len_866_127505.p1_type_complete_len_218 | ---NA--- | | |
| TRINITY_DN329_c0_g1_i1_len_656_239845.p1_type_internal_len_219 | XP_018023968.1 spermatogenesis-defective protein 39 homolog | C:GO:0005737; P:GO:0006886 | |
| TRINITY_DN14543_c0_g1_i2_len_656_239850.p1_type_internal_len_219 | ---NA--- | | |
| TRINITY_DN34826_c0_g1_i1_len_656_239856.p1_type_internal_len_219 | BBC20718.1integrin alpha V | P:GO:0007155; P:GO:0007229; C:GO:0008305 | |
| TRINITY_DN14514_c0_g1_i1_len_655_239866.p1_type_internal_len_219 | XP_018007750.1 neogenin-like | C:GO:0016020 | |
| TRINITY_DN5595_c0_g1_i1_len_655_239871.p1_type_internal_len_219 | ---NA--- | | |
| TRINITY_DN16613_c0_g1_i1_len_1465_157957.p2_type_5prime_partial_len_218 | ABP97431.1alpha 2 macroglobulin | F:GO:0004866; C:GO:0005615; P:GO:0010951 | |
| TRINITY_DN20387_c0_g3_i1_len_919_33950.p1_type_3prime_partial_len_219 | ---NA--- | | |
| TRINITY_DN14111_c0_g1_i2_len_849_161948.p1_type_5prime_partial_len_218 | XP_018027683.1 alpha-tocopherol transfer protein-like isoform X4 | | |
| TRINITY_DN23408_c0_g1_i2_len_1037_196220.p1_type_complete_len_218 | XP_022111465.1embryonic polyadenylate-binding protein B-like isoform X2 | F:GO:0003723 | |

| | | | |
|---|---|---|---|
| TRINITY_DN14270_c0_g1_i1_len_697_163578.p1_type_5prime_partial_len_218 | AYD41576.1angiopoietin-related protein 2-like protein | | |
| TRINITY_DN16557_c0_g1_i1_len_781_238166.p1_type_5prime_partial_len_218 | XP_018023502.1 polycystin-2-like | | |
| TRINITY_DN27287_c0_g1_i2_len_927_197212.p1_type_3prime_partial_len_219 | ---NA--- | | |
| TRINITY_DN19154_c0_g1_i1_len_697_239253.p1_type_5prime_partial_len_218 | XP_018023774.1 probable glucosamine 6-phosphate N-acetyltransferase | F:GO:0016746 | |
| TRINITY_DN20532_c0_g1_i1_len_900_197489.p1_type_complete_len_218 | ---NA--- | | |
| TRINITY_DN11006_c0_g1_i1_len_657_200685.p1_type_internal_len_219 | ---NA--- | | |
| TRINITY_DN31971_c0_g1_i1_len_657_200675.p1_type_internal_len_219 | ---NA--- | | |
| TRINITY_DN16912_c0_g1_i3_len_656_164124.p1_type_internal_len_219 | ---NA--- | | |
| TRINITY_DN34324_c0_g1_i1_len_658_200654.p1_type_internal_len_219 | ---NA--- | | |
| TRINITY_DN10662_c0_g1_i2_len_656_200692.p1_type_internal_len_219 | XP_023710357.1protein SON-like isoform X2 | F:GO:0003723; P:GO:0043484; P:GO:0051726 | |
| TRINITY_DN18645_c0_g1_i1_len_1104_235342.p1_type_3prime_partial_len_219 | KXJ10292.1putative uncharacterized transposon-derived protein F52C9.6 | | |
| TRINITY_DN29947_c0_g1_i1_len_655_200723.p1_type_internal_len_219 | ---NA--- | | |
| TRINITY_DN4408_c0_g1_i1_len_655_200720.p1_type_internal_len_219 | ODN06541.1putative phospholipid-transporting ATPase VD | F:GO:0000166; F:GO:0000287; F:GO:0004012; F:GO:0005524; P:GO:0015914; C:GO:0016020; C:GO:0016021; P:GO:0045332 | |
| TRINITY_DN30520_c0_g1_i1_len_714_238995.p1_type_3prime_partial_len_219 | ---NA--- | | |
| TRINITY_DN33232_c0_g1_i1_len_655_200719.p2_type_internal_len_219 | ---NA--- | | |

| | | | |
|---|---|---|---|
| TRINITY_DN35041_c0_g1_i1_len_656_200707.p1_type_internal_len_219 | ---NA--- | | |
| TRINITY_DN2019_c0_g1_i1_len_656_200703.p1_type_internal_len_219 | ---NA--- | | |
| TRINITY_DN7425_c0_g1_i1_len_656_200691.p1_type_internal_len_219 | KDR00542.1E3 ubiquitin-protein ligase HUWE1 | F:GO:0016874 | |
| TRINITY_DN15783_c0_g1_i1_len_912_270833.p1_type_complete_len_217 | XP_018022460.1 protein MIS12 homolog | | |
| TRINITY_DN20176_c0_g1_i2_len_1943_231289.p2_type_complete_len_217 | AEL23015.1protein spaetzle | | |
| TRINITY_DN19795_c0_g1_i1_len_843_127671.p1_type_complete_len_217 | XP_018006726.1PREDICTED: uncharacterized protein LOC108664612 isoform X2 | | |
| TRINITY_DN15114_c0_g1_i1_len_1825_231655.p1_type_5prime_partial_len_217 | ---NA--- | | |
| TRINITY_DN17427_c0_g1_i1_len_1076_3188.p1_type_5prime_partial_len_217 | ---NA--- | | |
| TRINITY_DN24494_c0_g1_i3_len_2381_189745.p2_type_5prime_partial_len_217 | ---NA--- | | |
| TRINITY_DN12206_c0_g1_i1_len_875_271118.p1_type_complete_len_217 | PSN38759.1CDP-diacylglycerol--inositol 3-phosphatidyltransferase | | |
| TRINITY_DN26361_c2_g1_i6_len_783_238152.p1_type_5prime_partial_len_217 | ---NA--- | | |
| TRINITY_DN15628_c0_g1_i1_len_717_199743.p1_type_5prime_partial_len_217 | XP_018017486.1 Niemann-Pick C1 protein-like | C:GO:0016020 | |
| TRINITY_DN17112_c0_g1_i1_len_751_238517.p1_type_5prime_partial_len_217 | ---NA--- | | |
| TRINITY_DN17848_c0_g1_i1_len_1069_195942.p1_type_complete_len_217 | XP_017886933.1MOB kinase activator-like 1 | | |
| TRINITY_DN6469_c0_g1_i1_len_1132_195445.p2_type_5prime_partial_len_217 | ---NA--- | | |
| TRINITY_DN16303_c0_g1_i1_len_682_129214.p1_type_5prime_partial_len_217 | ---NA--- | | |

| | | | |
|---|---|---|---|
| TRINITY_DN7405_c0_g1_i1_len_1236_194706.p1_type_5prime_partial_len_217 | ---NA--- | | |
| TRINITY_DN10009_c0_g1_i1_len_652_200767.p3_type_internal_len_218 | ---NA--- | | |
| TRINITY_DN8791_c0_g1_i1_len_652_66305.p2_type_internal_len_218 | ---NA--- | | |
| TRINITY_DN16409_c0_g1_i1_len_651_301677.p1_type_internal_len_218 | ---NA--- | | |
| TRINITY_DN31823_c0_g1_i1_len_652_200776.p1_type_internal_len_218 | XP_018011143.1 triple functional domain protein-like | F:GO:0005089; P:GO:0035023 | |
| TRINITY_DN9622_c0_g1_i1_len_655_66260.p1_type_internal_len_218 | XP_023332368.1DNA topoisomerase 2-binding protein 1-A-like | | |
| TRINITY_DN13314_c0_g1_i1_len_652_200780.p1_type_internal_len_218 | PNF19231.1hypothetical protein B7P43_G08208 | F:GO:0004842; P:GO:0016567; F:GO:0046872 | |
| TRINITY_DN14858_c0_g1_i1_len_651_200788.p1_type_internal_len_218 | XP_013794025.1myotubularin-related protein 2-like | | |
| TRINITY_DN11708_c0_g1_i1_len_654_66268.p1_type_internal_len_218 | ---NA--- | | |
| TRINITY_DN22400_c0_g1_i2_len_979_160843.p2_type_complete_len_217 | KZS04058.1Glutathione S-transferase omega-2 | F:GO:0003824 | |
| TRINITY_DN29583_c0_g1_i1_len_652_164173.p1_type_internal_len_218 | ---NA--- | | |
| TRINITY_DN40919_c0_g1_i1_len_652_200773.p1_type_3prime_partial_len_218 | XP_023239808.1PHD finger protein 12-like | F:GO:0046872 | |
| TRINITY_DN4599_c0_g1_i1_len_653_164149.p1_type_internal_len_218 | XP_015763302.1PREDICTED: LOW QUALITY PROTEIN: uncharacterized protein LOC107342331 | F:GO:0005488 | |
| TRINITY_DN37106_c0_g1_i1_len_863_197890.p1_type_3prime_partial_len_218 | XP_018026450.1PREDICTED: uncharacterized protein LOC108681885 | | |
| TRINITY_DN4723_c0_g1_i1_len_653_200756.p2_type_internal_len_218 | ABP97431.1alpha 2 macroglobulin | F:GO:0004866; C:GO:0005615; P:GO:0010951 | |
| TRINITY_DN16283_c0_g1_i2_len_900_299418.p1_type_5prime_partial_len_217 | AEL23047.1dystroglycan, partial | F:GO:0005509; P:GO:0007016; C:GO:0016010; C:GO:0016021 | |

| | | | |
|---|---|---|---|
| TRINITY_DN38986_c0_g1_i1_len_655_200725.p1_type_internal_len_218 | XP_019639352.1 inactive hydroxysteroid dehydrogenase-like protein 1 | F:GO:0016491; P:GO:0055114 | |
| TRINITY_DN4546_c0_g1_i2_len_653_239894.p1_type_3prime_partial_len_218 | XP_021942949.1homeodomain-interacting protein kinase 2 isoform X2 | F:GO:0003677; F:GO:0004672; F:GO:0005524; P:GO:0006468; F:GO:0016301; P:GO:0016310 | |
| TRINITY_DN20936_c0_g1_i1_len_654_200741.p1_type_internal_len_218 | XP_002733395.1 arylsulfatase I-like | | |
| TRINITY_DN26390_c0_g1_i1_len_787_198767.p1_type_5prime_partial_len_217 | ---NA--- | | |
| TRINITY_DN17602_c0_g1_i1_len_653_200745.p1_type_internal_len_218 | ---NA--- | | |
| TRINITY_DN10281_c0_g1_i1_len_653_200743.p1_type_internal_len_218 | XP_026281862.1uncharacterized protein LOC113208834 isoform X2 | P:GO:0006355 | |
| TRINITY_DN12475_c0_g1_i1_len_654_200731.p1_type_internal_len_218 | XP_021919183.1proton-coupled folate transporter-like isoform X2 | | |
| TRINITY_DN39406_c0_g1_i1_len_654_200740.p1_type_internal_len_218 | ---NA--- | | |
| TRINITY_DN24829_c0_g3_i1_len_651_239931.p1_type_internal_len_218 | ---NA--- | | |
| TRINITY_DN38886_c0_g1_i1_len_653_200755.p1_type_internal_len_218 | XP_018011656.1 laminin subunit alpha-2-like | P:GO:0007155 | |
| TRINITY_DN6879_c0_g1_i1_len_652_239925.p1_type_internal_len_218 | PVD34174.1hypothetical protein C0Q70_05437 | F:GO:0003824; C:GO:0016020; C:GO:0016021 | |
| 1357\|_\|c10145_f2p1_3001_isoform_c10145_full_length_coverage_2_non_full_length_coverage_1_isoform_length_3001_21922 | ---NA--- | | |
| TRINITY_DN21133_c0_g1_i1_len_3441_90993.p1_type_complete_len_1000 | AGZ84437.1cold shock protein | F:GO:0003676 | |
| TRINITY_DN19625_c0_g1_i3_len_3398_29353.p1_type_complete_len_1000 | XP_018014158.1PREDICTED: uncharacterized protein LOC108671173 | | |
| TRINITY_DN19008_c0_g1_i1_len_3218_58031.p1_type_complete_len_1000 | KXJ22893.1tRNA 2-thiocytidine biosynthesis protein TtcA | | |

| | | | |
|---|---|---|---|
| TRINITY_DN25264_c0_g1_i2_len_4612_227905.p1_type_5prime_partial_len_1000 | AXR98481.1large proline-rich protein BAG6 | | |
| 1363\|_\|c1848_f2p1_3000_isoform_c1848_full_length_coverage_2_non_full_length_coverage_1_isoform_length_3000_28025 | XP_019636418.1 cell division control protein 6 homolog | P:GO:0009987 | |
| TRINITY_DN21307_c0_g1_i1_len_3369_57979.p1_type_complete_len_999 | XP_018022264.1 tubulin polyglutamylase TTLL4-like | P:GO:0006464 | |
| 1365\|_\|c10445_f2p41_2996_isoform_c10445_full_length_coverage_2_non_full_length_coverage_41_isoform_length_2996_26066 | ---NA--- | | |
| 1366\|_\|c1143_f4p5_2996_isoform_c1143_full_length_coverage_4_non_full_length_coverage_5_isoform_length_2996_25815 | XP_015514919.1 pre-rRNA-processing protein TSR1 homolog | | |
| 1368\|_\|c1657_f2p8_2994_isoform_c1657_full_length_coverage_2_non_full_length_coverage_8_isoform_length_2994_27880 | ---NA--- | | |
| TRINITY_DN476_c0_g1_i1_len_655_273335.p1_type_internal_len_218 | XP_018019118.1PREDICTED: uncharacterized protein LOC108675608 | | |
| TRINITY_DN20555_c0_g1_i1_len_653_200764.p1_type_internal_len_218 | ---NA--- | | |
| TRINITY_DN9041_c0_g1_i1_len_653_273376.p1_type_internal_len_218 | XP_018024334.1 cell division cycle and apoptosis regulator protein 1-like | | |
| TRINITY_DN15236_c0_g1_i1_len_737_5368.p1_type_5prime_partial_len_217 | ---NA--- | | |
| TRINITY_DN18208_c0_g1_i1_len_655_200717.p1_type_internal_len_218 | XP_013399086.1guanine deaminase-like isoform X2 | F:GO:0016810 | |
| TRINITY_DN20227_c0_g1_i3_len_2019_266260.p2_type_3prime_partial_len_218 | ---NA--- | | |
| TRINITY_DN32043_c0_g1_i1_len_653_200760.p1_type_internal_len_218 | XP_018017090.1 GPI mannosyltransferase 4-like | C:GO:0016020 | |
| TRINITY_DN23535_c0_g1_i1_len_3197_228773.p1_type_5prime_partial_len_216 | ADK38544.1progestin membrane receptor component 1 | | |

| | | | |
|---|---|---|---|
| TRINITY_DN18827_c0_g1_i2_len_2386_155206.p1_type_complete_len_216 | ---NA--- | | |
| TRINITY_DN26558_c0_g1_i6_len_648_200842.p1_type_internal_len_217 | EDO36870.1predicted protein | | |
| TRINITY_DN17789_c0_g1_i1_len_649_200829.p1_type_internal_len_217 | ---NA--- | | |
| TRINITY_DN16984_c0_g1_i1_len_832_4603.p1_type_5prime_partial_len_216 | XP_022249506.1protein prune homolog 2-like isoform X1 | F:GO:0004309; C:GO:0005737; P:GO:0006798 | EC:3.6.1.11 |
| TRINITY_DN35230_c0_g1_i1_len_650_200812.p1_type_internal_len_217 | XP_023660370.1hemicentin-1 isoform X3 | | |
| TRINITY_DN11760_c0_g1_i1_len_650_200811.p1_type_internal_len_217 | ---NA--- | | |
| TRINITY_DN33194_c0_g1_i1_len_650_200808.p1_type_internal_len_217 | XP_017876911.1putative tyrosine-protein kinase Wsck | F:GO:0004672; F:GO:0005524; P:GO:0006468; C:GO:0016020; C:GO:0016021 | |
| TRINITY_DN25046_c0_g1_i2_len_2096_230802.p2_type_5prime_partial_len_216 | ---NA--- | | |
| TRINITY_DN11811_c0_g1_i1_len_652_200779.p1_type_internal_len_217 | ---NA--- | | |
| TRINITY_DN23137_c0_g1_i1_len_1765_191791.p3_type_complete_len_216 | APO14259.1juvenile hormone esterase-like carboxylesterase 1 | F:GO:0016787 | |
| TRINITY_DN23494_c0_g1_i3_len_1650_123846.p2_type_5prime_partial_len_216 | XP_018011541.1 solute carrier family 52, riboflavin transporter, member 3-B-like | C:GO:0005887; F:GO:0032217; P:GO:0032218 | |
| TRINITY_DN19263_c0_g1_i1_len_1463_267881.p1_type_5prime_partial_len_216 | XP_008483537.1splicing factor U2af 38 kDa subunit | P:GO:0000398; F:GO:0003723; F:GO:0046872; C:GO:0089701 | |
| TRINITY_DN18481_c1_g1_i1_len_1643_192338.p1_type_5prime_partial_len_216 | XP_023223218.1partner of Y14 and mago-like | | |
| TRINITY_DN23995_c0_g1_i2_len_1250_194587.p1_type_complete_len_216 | XP_023664209.1E3 ubiquitin-protein ligase RNF185 | | |
| TRINITY_DN1848_c0_g1_i1_len_759_199132.p1_type_5prime_partial_len_216 | XP_014661604.1 zinc finger MYND domain-containing protein 19-like | F:GO:0046872 | |

| | | | |
|---|---|---|---|
| TRINITY_DN10386_c0_g1_i2_len_651_301686.p1_type_internal_len_217 | AVN99021.1map kinase-interacting serine/threonine | F:GO:0004683; F:GO:0005516; F:GO:0005524; C:GO:0005634; C:GO:0005737; F:GO:0009931; P:GO:0018105; P:GO:0035556; P:GO:0046777 | EC:2.7.11; EC:2.7.11.17 |
| TRINITY_DN27074_c0_g1_i1_len_648_273445.p1_type_internal_len_217 | XP_019770016.1 homeodomain-interacting protein kinase 2-like | | |
| TRINITY_DN34724_c0_g1_i1_len_649_239977.p1_type_internal_len_217 | XP_018370350.1 lysosomal acid phosphatase-like isoform X2 | | |
| TRINITY_DN2418_c0_g1_i1_len_649_239976.p1_type_internal_len_217 | XP_018021126.1 inositol hexakisphosphate and diphosphoinositol-pentakisphosphate kinase-like | F:GO:0000829; F:GO:0000832; F:GO:0005524; C:GO:0005829; P:GO:0016310; F:GO:0033857; F:GO:0052723; F:GO:0052724; F:GO:0102092 | EC:2.7.4.24; EC:2.7.4.21 |
| TRINITY_DN11242_c0_g1_i1_len_649_239967.p1_type_internal_len_217 | XP_018011797.1 E3 ubiquitin-protein ligase HUWE1-like | | |
| TRINITY_DN33133_c0_g1_i1_len_650_239965.p1_type_internal_len_217 | XP_018012955.1 inhibin beta chain-like | | |
| TRINITY_DN2095_c0_g1_i2_len_650_239952.p1_type_internal_len_217 | ---NA--- | | |
| TRINITY_DN17293_c0_g1_i1_len_650_239950.p1_type_internal_len_217 | ---NA--- | | |
| TRINITY_DN7325_c0_g1_i1_len_649_129615.p1_type_internal_len_217 | ---NA--- | | |
| TRINITY_DN21961_c0_g1_i1_len_1499_157816.p2_type_5prime_partial_len_216 | ---NA--- | | |
| TRINITY_DN28221_c0_g1_i1_len_2076_190635.p1_type_5prime_partial_len_216 | XP_019634804.1 aprataxin-like | F:GO:0003676; F:GO:0003677; F:GO:0003824; P:GO:0006281; F:GO:0033699; P:GO:0090305 | |
| TRINITY_DN15742_c0_g1_i4_len_650_164210.p1_type_internal_len_217 | ---NA--- | | |
| TRINITY_DN3002_c0_g1_i1_len_755_97496.p1_type_3prime_partial_len_217 | ---NA--- | | |

| | | | |
|---|---|---|---|
| TRINITY_DN13526_c0_g1_i1_len_687_98236.p1_type_5prime_partial_len_216 | XP_018007781.1 probable palmitoyltransferase ZDHHC24 | | |
| TRINITY_DN25546_c0_g1_i1_len_686_301282.p1_type_3prime_partial_len_217 | AEC22817.1hypothetical protein | | |
| TRINITY_DN14450_c0_g1_i2_len_774_272025.p1_type_3prime_partial_len_217 | XP_018016678.1 choline transporter-like protein 2 | C:GO:0016020; C:GO:0016021 | |
| TRINITY_DN24643_c0_g1_i2_len_2990_58176.p3_type_complete_len_216 | ARS88259.1ATP binding cassette transporter 3 | F:GO:0005524; C:GO:0016021; F:GO:0016887 | EC:3.6.1.3; EC:3.6.1.15 |
| TRINITY_DN19544_c0_g1_i1_len_701_239183.p1_type_5prime_partial_len_216 | ---NA--- | | |
| TRINITY_DN19223_c0_g1_i1_len_997_298832.p1_type_complete_len_215 | XP_004612925.1 B9 domain-containing protein 1 | P:GO:0001701; P:GO:0001944; F:GO:0005515; C:GO:0005813; P:GO:0007224; F:GO:0008158; C:GO:0016020; P:GO:0032880; C:GO:0036038; C:GO:0036064; P:GO:0042733; P:GO:0043010; P:GO:0060271; P:GO:0060563 | |
| TRINITY_DN14834_c0_g1_i1_len_775_238246.p1_type_5prime_partial_len_215 | ---NA--- | | |
| TRINITY_DN14406_c0_g1_i1_len_748_199312.p1_type_5prime_partial_len_215 | XP_021954102.1methyltransferase-like protein 27 | | |
| TRINITY_DN3159_c0_g1_i1_len_645_66398.p1_type_internal_len_216 | XP_015835602.1 sodium- and chloride-dependent glycine transporter 2 isoform X2 | | |
| TRINITY_DN21102_c0_g1_i2_len_854_198003.p1_type_5prime_partial_len_215 | ---NA--- | | |
| TRINITY_DN5614_c0_g1_i1_len_647_164258.p1_type_internal_len_216 | ---NA--- | | |
| TRINITY_DN22375_c1_g1_i1_len_1264_234273.p2_type_5prime_partial_len_215 | PHZ15642.1TPR-like protein | C:GO:0005634; P:GO:0006396; P:GO:0006397 | |
| TRINITY_DN19704_c0_g1_i1_len_830_198264.p1_type_5prime_partial_len_215 | ---NA--- | | |

| | | | |
|---|---|---|---|
| TRINITY_DN31581_c0_g1_i1_len_731_163136.p1_type_5prime_partial_len_215 | KZS20093.1putative Beta-hexosaminidase fdl | F:GO:0016787 | |
| TRINITY_DN17444_c0_g1_i1_len_752_65076.p1_type_5prime_partial_len_215 | PNF22034.1KxDL motif-containing protein CG10681 | | |
| TRINITY_DN21181_c0_g1_i2_len_809_198497.p1_type_5prime_partial_len_215 | ---NA--- | | |
| TRINITY_DN24463_c0_g1_i1_len_1952_191080.p1_type_5prime_partial_len_215 | ---NA--- | | |
| TRINITY_DN23317_c0_g1_i1_len_1822_191589.p1_type_complete_len_215 | AID07491.1innexin 4 | C:GO:0005886; C:GO:0005921; P:GO:0006811; C:GO:0016021 | |
| TRINITY_DN10957_c0_g1_i2_len_664_301532.p1_type_3prime_partial_len_216 | KDR21087.1S-phase kinase-associated protein 2 | F:GO:0016301; P:GO:0016310 | |
| TRINITY_DN21485_c0_g1_i1_len_699_200004.p1_type_5prime_partial_len_215 | ---NA--- | | |
| TRINITY_DN34741_c0_g1_i1_len_646_240033.p1_type_internal_len_216 | XP_013408243.1ectopic P granules protein 5 homolog isoform X1 | P:GO:0006914 | |
| TRINITY_DN16527_c0_g1_i1_len_646_240028.p1_type_internal_len_216 | ---NA--- | | |
| TRINITY_DN36989_c0_g1_i1_len_649_239966.p1_type_internal_len_216 | XP_018026075.1 protein ROP-like | P:GO:0006904 | |
| TRINITY_DN3429_c0_g1_i1_len_648_239990.p1_type_internal_len_216 | ---NA--- | | |
| TRINITY_DN36742_c0_g1_i1_len_647_240006.p1_type_internal_len_216 | XP_023712281.1protein TANC2 isoform X1 | | |
| TRINITY_DN3645_c0_g1_i1_len_647_240016.p1_type_internal_len_216 | XP_018015345.1PREDICTED: uncharacterized protein LOC108672220, partial | | |
| TRINITY_DN8157_c0_g1_i1_len_690_200151.p1_type_5prime_partial_len_215 | XP_005953354.2 mitochondrial sodium/hydrogen exchanger 9B2-like | | |
| TRINITY_DN7758_c0_g1_i1_len_648_200851.p1_type_internal_len_216 | ODM95035.1Tyrosine-protein phosphatase non-receptor type 12 | F:GO:0004725; P:GO:0035335 | EC:3.1.3.16; EC:3.1.3.48 |
| TRINITY_DN21087_c0_g1_i1_len_648_200854.p1_type_internal_len_216 | ---NA--- | | |

| | | | |
|---|---|---|---|
| TRINITY_DN11945_c0_g1_i1_len_648_200858.p1_type_internal_len_216 | XP_018011388.1 ral GTPase-activating protein subunit alpha-1-like | F:GO:0005096; C:GO:0005634; P:GO:0043547; P:GO:0051056 | |
| TRINITY_DN8625_c0_g1_i1_len_647_200861.p1_type_internal_len_216 | XP_023211622.1WD repeat and FYVE domain-containing protein 3-like | F:GO:0046872 | |
| TRINITY_DN26535_c0_g1_i3_len_647_200863.p1_type_internal_len_216 | ---NA--- | | |
| TRINITY_DN13389_c0_g1_i2_len_646_301751.p1_type_internal_len_216 | XP_018025051.1 sulfotransferase 1A3-like | F:GO:0008146; F:GO:0016740 | |
| TRINITY_DN1469_c0_g1_i1_len_647_301738.p1_type_internal_len_216 | ---NA--- | | |
| TRINITY_DN14727_c0_g1_i1_len_649_129620.p1_type_internal_len_216 | ---NA--- | | |
| TRINITY_DN8698_c0_g1_i1_len_648_129633.p1_type_internal_len_216 | ---NA--- | | |
| TRINITY_DN14481_c0_g1_i1_len_647_129650.p1_type_internal_len_216 | ---NA--- | | |
| TRINITY_DN1003_c0_g1_i1_len_649_200825.p1_type_internal_len_216 | XP_018013850.1 pecanex-like protein 4 | | |
| TRINITY_DN32148_c0_g1_i1_len_647_200869.p1_type_internal_len_216 | KDR17018.1Centrosomal protein of 97 kDa | | |
| TRINITY_DN39439_c0_g1_i1_len_647_200874.p1_type_internal_len_216 | XP_014781139.1 protein O-linked-mannose beta-1,2-N-acetylglucosaminyltransferase 1-like | P:GO:0006486; F:GO:0008375 | |
| TRINITY_DN16401_c0_g1_i1_len_647_200877.p1_type_internal_len_216 | ---NA--- | | |
| TRINITY_DN16501_c0_g1_i1_len_646_200878.p1_type_internal_len_216 | XP_018026573.1 facilitated trehalose transporter Tret1-like | | |
| TRINITY_DN39020_c0_g1_i1_len_646_200885.p1_type_internal_len_216 | ---NA--- | | |
| TRINITY_DN11027_c0_g1_i1_len_647_200865.p1_type_internal_len_216 | XP_018016004.1PREDICTED: uncharacterized protein LOC108672784 | F:GO:0005509; F:GO:0030234; P:GO:0050790 | |
| TRINITY_DN18169_c0_g1_i1_len_711_65541.p1_type_5prime_partial_len_214 | ---NA--- | | |

| | | | |
|---|---|---|---|
| TRINITY_DN13202_c0_g1_i1_len_702_65644.p1_type_5prime_partial_len_214 | ---NA--- | | |
| TRINITY_DN10949_c0_g1_i1_len_768_300425.p1_type_complete_len_214 | XP_018024167.1 vesicle-trafficking protein SEC22b-like isoform X3 | | |
| TRINITY_DN12328_c0_g1_i1_len_776_300349.p1_type_complete_len_214 | XP_015189995.1 ras-related protein Rab-2 | F:GO:0003924; F:GO:0005525 | EC:3.6.1.15 |
| TRINITY_DN21248_c0_g2_i1_len_644_200907.p1_type_internal_len_215 | XP_018027163.1 UDP-glucuronosyltransferase 2B14-like | | |
| TRINITY_DN41218_c0_g1_i1_len_644_200910.p1_type_internal_len_215 | XP_018006820.1 protein diaphanous-like | P:GO:0016043 | |
| TRINITY_DN31596_c0_g1_i1_len_643_200925.p1_type_internal_len_215 | PIK62099.1hypothetical protein BSL78_00982 | F:GO:0003676; F:GO:0003700; F:GO:0004190; P:GO:0006355; P:GO:0006508 | |
| TRINITY_DN18036_c0_g1_i1_len_3257_264793.p2_type_complete_len_214 | XP_018020340.1 integrator complex subunit 7-like | P:GO:0016180; C:GO:0032039 | |
| TRINITY_DN19838_c0_g1_i3_len_1952_156254.p3_type_5prime_partial_len_214 | ---NA--- | | |
| TRINITY_DN33982_c0_g1_i1_len_646_200882.p1_type_internal_len_215 | XP_018015017.1 follistatin-like | | |
| TRINITY_DN12096_c0_g1_i1_len_645_200904.p1_type_internal_len_215 | XP_018025794.1 voltage-dependent calcium channel subunit alpha-2/delta-3-like | | |
| TRINITY_DN6242_c0_g1_i1_len_644_200905.p1_type_internal_len_215 | ---NA--- | | |
| TRINITY_DN6384_c0_g1_i1_len_644_200906.p1_type_internal_len_215 | XP_018017230.1 dmX-like protein 2 isoform X1 | | |
| TRINITY_DN18920_c0_g1_i1_len_643_6385.p1_type_5prime_partial_len_214 | ---NA--- | | |
| TRINITY_DN1178_c0_g1_i1_len_644_6370.p1_type_internal_len_215 | ---NA--- | | |
| TRINITY_DN22802_c0_g1_i1_len_789_198743.p1_type_5prime_partial_len_214 | XP_003746523.1 calcipressin-2 | F:GO:0003676; P:GO:0019722 | |

| | | | |
|---|---|---|---|
| TRINITY_DN1431_c0_g1_i1_len_644_66424.p1_type_internal_len_215 | ---NA--- | | |
| TRINITY_DN6684_c0_g1_i1_len_645_66404.p1_type_internal_len_215 | ---NA--- | | |
| TRINITY_DN1113_c0_g1_i1_len_644_273502.p1_type_internal_len_215 | KDR14641.1hypothetical protein L798_10748 | C:GO:0016020; C:GO:0016021 | |
| TRINITY_DN2409_c0_g1_i2_len_897_236983.p2_type_5prime_partial_len_214 | ---NA--- | | |
| TRINITY_DN26399_c0_g1_i1_len_3146_228822.p1_type_5prime_partial_len_998 | KRY40733.1Transposon Ty3-G Gag-Pol polyprotein | F:GO:0003676; P:GO:0015074 | |
| TRINITY_DN29627_c0_g1_i2_len_3446_187990.p1_type_5prime_partial_len_998 | ODM99513.1Rho guanine nucleotide exchange factor 33 | F:GO:0005089; P:GO:0035023 | |
| 1373\|_\|c15655_f2p5_2993_isoform_c15655_full_length_coverage_2_non_full_length_coverage_5_isoform_length_2993_26273 | ---NA--- | | |
| 1374\|_\|c9846_f2p9_2992_isoform_c9846_full_length_coverage_2_non_full_length_coverage_9_isoform_length_2992_25996 | AJO69983.1organellar Rab protein | F:GO:0003924; F:GO:0005525 | EC:3.6.1.15 |
| TRINITY_DN26867_c0_g1_i1_len_4119_187522.p1_type_complete_len_997 | XP_017932143.1DNA ligase 3 isoform X1 | | |
| 1377\|_\|c4589_f2p24_2989_isoform_c4589_full_length_coverage_2_non_full_length_coverage_24_isoform_length_2989_28348 | ---NA--- | | |
| 1378\|_\|c7360_f2p27_2989_isoform_c7360_full_length_coverage_2_non_full_length_coverage_27_isoform_length_2989_28469 | XP_018014282.1 serine/threonine-protein phosphatase 2A catalytic subunit beta isoform | F:GO:0004721; P:GO:0006470 | EC:3.1.3.16 |
| 1379\|_\|c4908_f2p0_2986_isoform_c4908_full_length_coverage_2_non_full_length_coverage_0_isoform_length_2986_12425 | XP_018322472.1probable cleavage and polyadenylation specificity factor subunit 2 | P:GO:0016070 | |
| TRINITY_DN23092_c0_g1_i1_len_3176_228797.p1_type_complete_len_995 | XP_018014589.1 band 4.1-like protein 4B isoform X2 | C:GO:0005856; F:GO:0008092 | |

| | | | |
|---|---|---|---|
| TRINITY_DN29102_c0_g1_i1_len_3297_188139.p1_type_5prime_partial_len_995 | XP_018006687.1PREDICTED: uncharacterized protein LOC108664581 isoform X4 | | |
| TRINITY_DN4580_c0_g1_i1_len_646_301761.p1_type_internal_len_215 | ---NA--- | | |
| TRINITY_DN784_c0_g1_i1_len_645_301767.p1_type_internal_len_215 | ---NA--- | | |
| TRINITY_DN19718_c0_g1_i4_len_794_64649.p1_type_5prime_partial_len_214 | XP_019490632.1 S-adenosylmethionine synthase-like | F:GO:0000166; F:GO:0016740; F:GO:0043167; P:GO:0044237 | |
| TRINITY_DN15405_c0_g1_i1_len_756_300542.p1_type_5prime_partial_len_214 | XP_018025672.1 kynurenine 3-monooxygenase-like | F:GO:0004502; C:GO:0005741; P:GO:0006569; F:GO:0016174; P:GO:0019674; P:GO:0019805; P:GO:0034354; P:GO:0043420; P:GO:0055114; P:GO:0070189; F:GO:0071949 | EC:1.6.3.1; EC:1.14.13.9; EC:1.14.13 |
| TRINITY_DN20679_c0_g1_i1_len_736_5382.p1_type_5prime_partial_len_214 | XP_018027472.1 28S ribosomal protein S18c, mitochondrial-like | | |
| TRINITY_DN18251_c0_g1_i3_len_1755_30857.p2_type_3prime_partial_len_215 | RMY52170.1hypothetical protein D0864_14304 | F:GO:0004122; P:GO:0006535; P:GO:0019343 | EC:4.2.1.22 |
| TRINITY_DN27482_c0_g1_i1_len_645_164288.p1_type_internal_len_215 | XP_023295729.1D-aspartate oxidase | F:GO:0003884; P:GO:0046416; F:GO:0048037 | EC:1.4.3.3 |
| TRINITY_DN17824_c0_g1_i1_len_705_128963.p1_type_5prime_partial_len_214 | XP_018009236.1 frataxin, mitochondrial-like | F:GO:0004322; C:GO:0005739; F:GO:0008199; P:GO:0016226; P:GO:0055114 | EC:1.16.3.1; EC:1.16.3 |
| TRINITY_DN13864_c0_g1_i1_len_645_98762.p1_type_internal_len_215 | ---NA--- | | |
| TRINITY_DN7463_c0_g1_i1_len_840_237543.p1_type_5prime_partial_len_214 | ---NA--- | | |
| TRINITY_DN31025_c0_g1_i1_len_646_240027.p1_type_internal_len_215 | XP_018010802.1 disks large homolog 5-like | | |
| TRINITY_DN4788_c0_g1_i1_len_688_239402.p1_type_3prime_partial_len_215 | AJR22372.1serine proteinase inhibitor-2 | C:GO:0005615 | |

| | | | |
|---|---|---|---|
| TRINITY_DN17115_c0_g1_i2_len_722_199686.p1_type_5prime_partial_len_214 | ---NA--- | | |
| TRINITY_DN22539_c0_g1_i5_len_643_240065.p1_type_internal_len_215 | XP_019861243.1 serine/threonine-protein phosphatase 6 regulatory ankyrin repeat subunit A-like | | |
| TRINITY_DN15299_c0_g1_i1_len_645_240038.p1_type_internal_len_215 | ---NA--- | | |
| TRINITY_DN11402_c0_g1_i1_len_864_237294.p1_type_5prime_partial_len_214 | ---NA--- | | |
| TRINITY_DN8941_c0_g1_i1_len_644_240046.p1_type_internal_len_215 | ---NA--- | | |
| TRINITY_DN17044_c0_g1_i1_len_1176_94605.p1_type_complete_len_213 | XP_017095699.1 28S ribosomal protein S18b, mitochondrial | | |
| TRINITY_DN28093_c0_g1_i1_len_3416_188020.p2_type_5prime_partial_len_213 | ---NA--- | | |
| TRINITY_DN23748_c0_g1_i1_len_821_198360.p1_type_5prime_partial_len_213 | XP_023341639.1UPF0676 protein C1494.01-like isoform X2 | F:GO:0016491 | |
| TRINITY_DN26489_c0_g1_i1_len_639_240131.p1_type_internal_len_214 | OWF40905.1Autophagy-related protein 16 | | |
| TRINITY_DN21218_c0_g1_i1_len_774_64842.p1_type_complete_len_213 | ---NA--- | | |
| TRINITY_DN23456_c0_g1_i1_len_2742_154641.p2_type_complete_len_213 | ---NA--- | | |
| TRINITY_DN18566_c0_g1_i1_len_1156_159600.p1_type_5prime_partial_len_213 | XP_018012939.1 mitochondrial import receptor subunit TOM20 homolog | C:GO:0005741; P:GO:0006886; C:GO:0044425 | |
| TRINITY_DN15961_c0_g1_i1_len_908_299367.p1_type_5prime_partial_len_213 | XP_018027347.1 RWD domain-containing protein 4-like | | |
| TRINITY_DN15238_c0_g1_i1_len_642_129725.p1_type_internal_len_214 | ---NA--- | | |
| TRINITY_DN8314_c0_g1_i1_len_641_6413.p1_type_internal_len_214 | ---NA--- | | |
| TRINITY_DN26349_c0_g1_i1_len_641_164356.p1_type_internal_len_214 | KFM69607.1hypothetical protein X975_03903, partial | C:GO:0005576; P:GO:0006030; F:GO:0008061 | |

| | | | |
|---|---|---|---|
| TRINITY_DN10505_c0_g1_i1_len_859_197944.p1_type_5prime_partial_len_213 | EFA04011.1Putative tyrosine-protein kinase Wsck-like Protein | F:GO:0004714; C:GO:0005887; P:GO:0007169; P:GO:0008284; P:GO:0016310; C:GO:0043235 | EC:2.7.10; EC:2.7.10.1; EC:2.7.11 |
| TRINITY_DN12480_c0_g1_i1_len_684_239435.p1_type_5prime_partial_len_213 | XP_018010599.1 neprilysin-like isoform X4 | | |
| TRINITY_DN8569_c0_g1_i1_len_964_196861.p1_type_5prime_partial_len_213 | XP_013412118.1peroxisomal targeting signal 1 receptor isoform X3 | | |
| TRINITY_DN8033_c0_g1_i1_len_742_35276.p1_type_5prime_partial_len_213 | XP_018024271.1PREDICTED: uncharacterized protein LOC108680026 isoform X1 | | |
| TRINITY_DN40667_c0_g1_i1_len_919_197302.p1_type_5prime_partial_len_213 | ---NA--- | | |
| TRINITY_DN21679_c0_g1_i1_len_842_96701.p1_type_3prime_partial_len_214 | ---NA--- | | |
| TRINITY_DN10890_c0_g1_i1_len_641_273546.p1_type_internal_len_214 | ---NA--- | | |
| TRINITY_DN9394_c0_g1_i2_len_639_240115.p1_type_internal_len_214 | XP_018008266.1PREDICTED: uncharacterized protein LOC108665966 | | |
| TRINITY_DN27304_c0_g1_i1_len_641_240087.p1_type_internal_len_214 | XP_022253234.1 probable cation-transporting ATPase 13A3 | | |
| TRINITY_DN34279_c0_g1_i1_len_663_164014.p1_type_5prime_partial_len_213 | CAA47046.1preprochymotrypsin 1 | F:GO:0004252; C:GO:0005615; P:GO:0006508; P:GO:0030574 | EC:3.4.21 |
| TRINITY_DN12688_c0_g1_i1_len_642_200938.p1_type_internal_len_214 | ---NA--- | | |
| TRINITY_DN18941_c0_g1_i1_len_640_200988.p2_type_internal_len_214 | XP_023228602.1jerky protein homolog-like | | |
| TRINITY_DN18360_c0_g1_i1_len_641_200982.p1_type_internal_len_214 | ---NA--- | | |
| TRINITY_DN29674_c0_g1_i3_len_1398_193662.p2_type_3prime_partial_len_214 | XP_018025187.1 intraflagellar transport protein 57 homolog | | |
| TRINITY_DN20546_c0_g1_i1_len_640_201003.p1_type_internal_len_214 | ---NA--- | | |

| | | | |
|---|---|---|---|
| TRINITY_DN12673_c0_g1_i1_len_801_300126.p1_type_complete_len_213 | ---NA--- | | |
| TRINITY_DN17999_c0_g1_i1_len_649_200827.p1_type_5prime_partial_len_213 | EEB14790.1ubiquitin-conjugating enzyme E2-230k, putative | F:GO:0016874 | |
| TRINITY_DN12496_c0_g1_i1_len_641_200985.p1_type_internal_len_214 | XP_021001228.1zinc finger protein 271-like isoform X2 | F:GO:0003676 | |
| TRINITY_DN920_c0_g1_i1_len_641_200984.p1_type_internal_len_214 | ---NA--- | | |
| TRINITY_DN5634_c0_g1_i1_len_642_200933.p1_type_internal_len_214 | ---NA--- | | |
| TRINITY_DN16083_c0_g1_i1_len_641_200960.p1_type_internal_len_214 | XP_022303542.1uncharacterized protein LOC111111072 | | |
| TRINITY_DN6833_c0_g1_i1_len_673_200425.p1_type_5prime_partial_len_213 | XP_018025500.1PREDICTED: uncharacterized protein LOC108681045 | | |
| TRINITY_DN37592_c0_g1_i1_len_641_200962.p1_type_internal_len_214 | ---NA--- | | |
| TRINITY_DN24417_c0_g1_i2_len_641_200974.p1_type_internal_len_214 | XP_018018917.1PREDICTED: uncharacterized protein LOC108675418 | | |
| TRINITY_DN8444_c0_g1_i1_len_641_200967.p1_type_internal_len_214 | XP_018024017.1 coronin-7-like | C:GO:0005794 | |
| TRINITY_DN14578_c0_g1_i1_len_641_200969.p1_type_internal_len_214 | ---NA--- | | |
| TRINITY_DN15967_c0_g1_i1_len_641_200970.p1_type_internal_len_214 | ---NA--- | | |
| TRINITY_DN1860_c0_g1_i1_len_641_200972.p1_type_internal_len_214 | XP_022238379.1serine/threonine-protein phosphatase 4 regulatory subunit 4-like isoform X3 | | |
| TRINITY_DN18039_c0_g1_i1_len_642_200940.p1_type_internal_len_214 | XP_018020023.1 spectrin beta chain, non-erythrocytic 1-like | | |
| TRINITY_DN19402_c0_g1_i1_len_642_200942.p1_type_internal_len_214 | ---NA--- | | |
| TRINITY_DN15868_c0_g1_i1_len_641_200978.p1_type_internal_len_214 | PSN32562.1Ankyrin-3 | P:GO:0007165 | |

| | | | |
|---|---|---|---|
| TRINITY_DN39115_c0_g1_i1_len_642_200949.p1_type_internal_len_214 | XP_018027486.1 connectin-like | | |
| TRINITY_DN17132_c0_g1_i1_len_642_200954.p1_type_internal_len_214 | XP_018010522.1PREDICTED: uncharacterized protein LOC108667928 | | |
| TRINITY_DN23535_c0_g1_i1_len_1702_192076.p2_type_complete_len_212 | ---NA--- | | |
| TRINITY_DN18609_c0_g1_i1_len_1230_94353.p1_type_complete_len_212 | AFN54651.1Rab GTPase | F:GO:0003924; F:GO:0005525 | EC:3.6.1.15 |
| TRINITY_DN7006_c0_g1_i1_len_697_65713.p1_type_5prime_partial_len_212 | AKR76217.1cysteine dioxygenase | | |
| TRINITY_DN10095_c0_g1_i1_len_638_36453.p1_type_internal_len_213 | XP_008468060.1lactosylceramide 4-alpha-galactosyltransferase-like isoform X2 | C:GO:0016020; C:GO:0016021; F:GO:0016740; F:GO:0016757 | |
| TRINITY_DN24470_c0_g1_i3_len_1450_233266.p1_type_complete_len_212 | XP_022255913.1type-1 angiotensin II receptor-associated protein-like | | |
| TRINITY_DN24751_c0_g1_i4_len_1121_195517.p1_type_3prime_partial_len_213 | KDR20326.1Sodium- and chloride-dependent glycine transporter 2 | F:GO:0005328; P:GO:0006836; C:GO:0016021; P:GO:0055085 | |
| TRINITY_DN19275_c0_g1_i2_len_637_201051.p1_type_internal_len_213 | XP_023705971.1methyltransferase-like protein 22 isoform X2 | P:GO:0006479; F:GO:0008276 | |
| TRINITY_DN33588_c0_g1_i1_len_638_201045.p1_type_internal_len_213 | ---NA--- | | |
| TRINITY_DN35450_c0_g1_i1_len_638_201037.p2_type_internal_len_213 | ---NA--- | | |
| TRINITY_DN9841_c0_g1_i1_len_637_201071.p1_type_internal_len_213 | XP_018011143.1 triple functional domain protein-like | | |
| TRINITY_DN17183_c0_g1_i1_len_637_201067.p1_type_internal_len_213 | ADO00267.1multidrug-resistance associated protein 2 | F:GO:0000166; P:GO:0006810; C:GO:0016021; F:GO:0016887; F:GO:0022857 | EC:3.6.1.3; EC:3.6.1.15 |
| TRINITY_DN3147_c0_g1_i1_len_637_201061.p1_type_internal_len_213 | XP_021945682.1transmembrane protein 62-like isoform X2 | | |
| TRINITY_DN40982_c0_g1_i1_len_640_201000.p1_type_internal_len_213 | EEB13658.1transcriptional enhancer factor TEF-1, putative | F:GO:0003677; F:GO:0003700; C:GO:0005634; P:GO:0006355; P:GO:0035329 | |

| | | | |
|---|---|---|---|
| TRINITY_DN843_c0_g1_i1_len_876_197742.p1_type_complete_len_212 | XP_008555893.1 nicotinamide riboside kinase 2 | | |
| TRINITY_DN16180_c0_g1_i2_len_1174_269199.p1_type_3prime_partial_len_213 | XP_023721003.1nucleic acid dioxygenase ALKBH1 isoform X2 | F:GO:0051213; P:GO:0055114 | |
| TRINITY_DN39269_c0_g1_i1_len_638_201032.p1_type_internal_len_213 | ---NA--- | | |
| TRINITY_DN20755_c0_g1_i2_len_638_201027.p3_type_internal_len_213 | ---NA--- | | |
| TRINITY_DN21028_c0_g1_i1_len_639_201022.p1_type_internal_len_213 | ADM88556.1fatty acid synthase | F:GO:0004312; P:GO:0009058; F:GO:0016491; F:GO:0016788; F:GO:0031177; P:GO:0055114 | EC:2.3.1.85 |
| TRINITY_DN10178_c0_g1_i1_len_640_201002.p1_type_internal_len_213 | ---NA--- | | |
| TRINITY_DN15588_c0_g1_i1_len_1470_157931.p1_type_5prime_partial_len_212 | ---NA--- | | |
| TRINITY_DN14717_c0_g1_i1_len_799_237961.p1_type_5prime_partial_len_212 | ---NA--- | | |
| TRINITY_DN28061_c0_g1_i1_len_1354_2099.p2_type_5prime_partial_len_212 | ---NA--- | | |
| TRINITY_DN12187_c0_g1_i1_len_1210_297767.p1_type_5prime_partial_len_212 | KZS20766.1Ras-related protein Rab-8A | F:GO:0003924; F:GO:0005525; C:GO:0005768; C:GO:0005886; P:GO:0006886; P:GO:0006904; C:GO:0008021; P:GO:0009306; P:GO:0017157; C:GO:0030140; P:GO:0032482; P:GO:0032869; P:GO:0048210; P:GO:0072659 | EC:3.6.1.15 |
| TRINITY_DN11452_c0_g1_i1_len_636_273613.p1_type_internal_len_213 | ---NA--- | | |
| TRINITY_DN31342_c0_g1_i1_len_636_240177.p1_type_internal_len_213 | XP_012218039.1 centrosomal protein of 97 kDa | | |
| TRINITY_DN17749_c0_g1_i1_len_731_238745.p1_type_5prime_partial_len_212 | XP_021925805.1mitochondrial import inner membrane translocase subunit Tim29 | C:GO:0042721 | |

| | | | |
|---|---|---|---|
| TRINITY_DN23103_c0_g3_i1_len_639_164388.p1_type_internal_len_213 | XP_018026450.1PREDICTED: uncharacterized protein LOC108681885 | | |
| TRINITY_DN9031_c0_g1_i1_len_639_164383.p1_type_internal_len_213 | ---NA--- | | |
| TRINITY_DN8874_c0_g1_i1_len_839_237558.p1_type_5prime_partial_len_212 | ---NA--- | | |
| TRINITY_DN35576_c0_g1_i1_len_637_240171.p1_type_internal_len_213 | XP_018023301.1 formin-like protein CG32138 | F:GO:0003779; F:GO:0017048; P:GO:0030036 | |
| TRINITY_DN8452_c0_g1_i1_len_637_240154.p1_type_internal_len_213 | ---NA--- | | |
| TRINITY_DN2550_c0_g1_i1_len_638_240149.p1_type_internal_len_213 | ---NA--- | | |
| TRINITY_DN15360_c0_g1_i1_len_640_240109.p1_type_internal_len_213 | XP_023708641.1ras-associated and pleckstrin homology domains-containing protein 1-like isoform X4 | | |
| TRINITY_DN7048_c0_g1_i1_len_638_240139.p1_type_internal_len_213 | PNF30223.1hypothetical protein B7P43_G02541 | P:GO:0007165; F:GO:0046872 | |
| TRINITY_DN23267_c0_g1_i1_len_746_199349.p1_type_5prime_partial_len_212 | XP_018024624.1 type-2 ice-structuring protein-like | | |
| TRINITY_DN22121_c0_g1_i1_len_733_199535.p1_type_5prime_partial_len_212 | XP_018025823.1 3-oxo-5-alpha-steroid 4-dehydrogenase 1-like | | |
| TRINITY_DN20348_c0_g1_i2_len_1573_192693.p1_type_complete_len_212 | XP_018024833.1PREDICTED: uncharacterized protein LOC108680513 | | |
| TRINITY_DN1639_c0_g1_i1_len_686_129172.p1_type_5prime_partial_len_212 | ---NA--- | | |
| TRINITY_DN14453_c0_g1_i1_len_800_162418.p1_type_5prime_partial_len_212 | KDR14769.139S ribosomal protein L32, mitochondrial | F:GO:0003735; C:GO:0005840; P:GO:0006412; C:GO:0015934 | |
| 1382\|_\|c1722_f2p2_2983_isoform_c1722_full_length_coverage_2_non_full_length_coverage_2_isoform_length_2983_27927 | ---NA--- | | |
| TRINITY_DN21101_c0_g1_i1_len_3632_153838.p1_type_complete_len_993 | XP_018023645.1 dual 3',5'-cyclic-AMP and -GMP phosphodiesterase 11-like isoform X2 | | |

<table>
<tr><td></td><td></td><td></td><td></td></tr>
<tr><td>TRINITY_DN29158_c0_g1_i1_len_3470_187966.p1_type_complete_len_993</td><td>XP_018008518.1 26S proteasome non-ATPase regulatory subunit 1-like isoform X1</td><td>C:GO:0016020; C:GO:0022624; P:GO:0065007</td><td></td></tr>
<tr><td>TRINITY_DN26059_c0_g1_i1_len_3662_228359.p1_type_complete_len_993</td><td>EDO32713.1predicted protein</td><td>F:GO:0003676; C:GO:0005737; C:GO:0036064; P:GO:0060271</td><td></td></tr>
<tr><td>1389|_|c2028_f2p7_2977_isoform_c2028_full_length_coverage_2_non_full_length_coverage_7_isoform_length_2977_26158</td><td>XP_018012308.1 putative ATP-dependent RNA helicase DHX33</td><td></td><td></td></tr>
<tr><td>1390|_|c19670_f3p18_2976_isoform_c19670_full_length_coverage_3_non_full_length_coverage_18_isoform_length_2976_28950</td><td>ANZ80593.1ubiquitin-activating enzyme E1</td><td>F:GO:0004839; F:GO:0005524; P:GO:0016567</td><td>EC:6.2.1.45</td></tr>
<tr><td>TRINITY_DN26690_c0_g1_i1_len_3489_187943.p1_type_5prime_partial_len_992</td><td>XP_018014386.1 serine/threonine-protein kinase PRP4 homolog</td><td>F:GO:0004672; F:GO:0005524; P:GO:0006468</td><td></td></tr>
<tr><td>1392|_|c26222_f4p46_2972_isoform_c26222_full_length_coverage_4_non_full_length_coverage_46_isoform_length_2972_13955</td><td>ANJ04737.1glycoprotein 94</td><td>F:GO:0005524; P:GO:0006457; F:GO:0051082</td><td></td></tr>
<tr><td>1393|_|c13411_f2p0_2971_isoform_c13411_full_length_coverage_2_non_full_length_coverage_0_isoform_length_2971_28620</td><td>XP_013096880.1PREDICTED: uncharacterized protein C1orf112 homolog</td><td></td><td></td></tr>
<tr><td>1394|_|c36204_f2p1_2971_isoform_c36204_full_length_coverage_2_non_full_length_coverage_1_isoform_length_2971_18916</td><td>ACA09717.1ubiquitin-conjugating enyzme E2r</td><td>F:GO:0005524; F:GO:0016740</td><td></td></tr>
<tr><td>TRINITY_DN22100_c0_g1_i1_len_797_162446.p1_type_3prime_partial_len_213</td><td>XP_020850955.1zinc finger protein 420-like</td><td></td><td></td></tr>
<tr><td>TRINITY_DN489_c0_g1_i1_len_768_199013.p1_type_3prime_partial_len_213</td><td>ADM15669.1myosin Va</td><td>F:GO:0003774; F:GO:0005524; C:GO:0016459; F:GO:0051015</td><td>EC:3.6.1.15</td></tr>
<tr><td>TRINITY_DN12415_c0_g1_i1_len_673_200421.p1_type_3prime_partial_len_213</td><td>XP_013780127.1laminin subunit gamma-1-like isoform X1</td><td></td><td></td></tr>
<tr><td>TRINITY_DN15703_c0_g1_i1_len_1760_92631.p1_type_5prime_partial_len_211</td><td>---NA---</td><td></td><td></td></tr>
<tr><td>TRINITY_DN20970_c0_g1_i1_len_1602_60348.p2_type_complete_len_211</td><td>XP_018009509.1 actin-like protein 6A</td><td></td><td></td></tr>
</table>

| | | | |
|---|---|---|---|
| TRINITY_DN9170_c0_g1_i1_len_636_240178.p1_type_internal_len_212 | ---NA--- | | |
| TRINITY_DN32921_c0_g1_i1_len_637_240152.p1_type_internal_len_212 | ---NA--- | | |
| TRINITY_DN19238_c0_g1_i1_len_636_240186.p1_type_internal_len_212 | PNF24257.1WD repeat-containing protein 7 | | |
| TRINITY_DN20544_c0_g1_i2_len_3072_154257.p2_type_complete_len_211 | ---NA--- | | |
| TRINITY_DN35547_c0_g1_i1_len_637_240163.p1_type_internal_len_212 | CCD78543.1KRAB-A domain-containing protein | F:GO:0003676; P:GO:0015074 | |
| TRINITY_DN35891_c0_g1_i1_len_635_201118.p1_type_internal_len_212 | ---NA--- | | |
| TRINITY_DN35398_c0_g1_i1_len_752_199249.p1_type_3prime_partial_len_212 | EEB11111.1bric-A-brac, putative | F:GO:0003677; C:GO:0005634 | |
| TRINITY_DN23383_c0_g1_i2_len_635_201109.p1_type_internal_len_212 | ---NA--- | | |
| TRINITY_DN12264_c0_g1_i1_len_657_200689.p1_type_5prime_partial_len_211 | XP_023231797.1protein pygopus-like isoform X6 | | |
| TRINITY_DN15053_c0_g1_i3_len_683_5919.p1_type_5prime_partial_len_211 | ---NA--- | | |
| TRINITY_DN30677_c0_g1_i1_len_4599_187272.p2_type_complete_len_211 | ---NA--- | | |
| TRINITY_DN1746_c0_g1_i1_len_634_240213.p1_type_internal_len_212 | PNF15335.1D-serine dehydratase | | |
| TRINITY_DN1173_c0_g1_i1_len_635_240197.p1_type_internal_len_212 | ---NA--- | | |
| TRINITY_DN2890_c0_g1_i1_len_635_240192.p1_type_internal_len_212 | ---NA--- | | |
| TRINITY_DN16412_c0_g1_i2_len_636_164442.p1_type_internal_len_212 | XP_022202941.1acetyl-CoA acetyltransferase, cytosolic | F:GO:0016747 | |
| TRINITY_DN688_c0_g1_i1_len_635_273636.p1_type_internal_len_212 | XP_014281279.1tyrosine-protein kinase PR2 isoform X4 | | |

| | | | |
|---|---|---|---|
| TRINITY_DN352_c0_g1_i1_len_633_240229.p1_type_internal_len_212 | XP_020712165.1ectopic P granules protein 5 homolog isoform X2 | | |
| TRINITY_DN26953_c0_g1_i1_len_633_240227.p1_type_internal_len_212 | XP_023220667.1pantothenate kinase 3-like | | |
| TRINITY_DN15648_c0_g1_i1_len_633_240226.p1_type_internal_len_212 | EEB16407.1tuberin, putative | P:GO:0065007 | |
| TRINITY_DN16595_c0_g1_i1_len_690_200158.p1_type_5prime_partial_len_211 | XP_023214802.1autophagy-related protein 16-like | | |
| TRINITY_DN18802_c0_g1_i1_len_927_299254.p1_type_complete_len_211 | XP_018018424.1 apolipoprotein D-like | P:GO:0000302; C:GO:0005737; P:GO:0006629; F:GO:0031409 | |
| TRINITY_DN25221_c0_g1_i2_len_1087_195798.p1_type_complete_len_211 | XP_023718153.1uridine diphosphate glucose pyrophosphatase-like isoform X1 | P:GO:0006753; F:GO:0008768; P:GO:0019693 | EC:3.6.1.45 |
| TRINITY_DN4290_c0_g1_i1_len_784_128171.p1_type_5prime_partial_len_211 | XP_018024594.1 ADP-ribosylation factor-like protein 8B-A | F:GO:0005525 | |
| TRINITY_DN7813_c0_g1_i1_len_636_98880.p1_type_internal_len_212 | XP_011433886.1 methyltransferase-like protein 9 | | |
| TRINITY_DN15733_c0_g1_i1_len_1035_196250.p2_type_complete_len_211 | ---NA--- | | |
| TRINITY_DN20984_c0_g1_i1_len_1316_124939.p1_type_complete_len_211 | AFN54651.1Rab GTPase | F:GO:0003924; F:GO:0005525 | EC:3.6.1.15 |
| TRINITY_DN9208_c0_g1_i1_len_635_201120.p1_type_internal_len_212 | EEB13922.1zinc metalloproteinase, putative | F:GO:0016787 | |
| TRINITY_DN15905_c0_g1_i1_len_634_98918.p1_type_internal_len_212 | ATU31747.1Calcium-activated chloride channel regulator 2 | | |
| TRINITY_DN19024_c0_g1_i1_len_633_201142.p1_type_internal_len_212 | ---NA--- | | |
| TRINITY_DN26283_c0_g1_i1_len_7227_227505.p3_type_5prime_partial_len_211 | ---NA--- | | |
| TRINITY_DN19241_c0_g1_i2_len_634_301896.p1_type_internal_len_212 | ---NA--- | | |
| TRINITY_DN22209_c0_g1_i1_len_634_201127.p1_type_internal_len_212 | XP_026733301.1phosphoinositide 3-kinase adapter protein 1 isoform X3 | | |

| | | | |
|---|---|---|---|
| TRINITY_DN19254_c0_g1_i1_len_1950_156258.p1_type_5prime_partial_len_211 | XP_006811109.1 lethal(3)malignant brain tumor-like protein 3-like | | |
| TRINITY_DN12001_c0_g1_i1_len_637_201064.p1_type_internal_len_212 | ---NA--- | | |
| TRINITY_DN26390_c0_g1_i3_len_2982_229008.p2_type_5prime_partial_len_211 | PIK35201.1Retrovirus-related Pol polyprotein from transposon | | |
| TRINITY_DN17541_c0_g1_i1_len_799_198618.p1_type_5prime_partial_len_211 | ---NA--- | | |
| TRINITY_DN6909_c0_g1_i1_len_636_201090.p1_type_internal_len_212 | XP_018008507.1 NFX1-type zinc finger-containing protein 1-like | | |
| TRINITY_DN22190_c0_g1_i1_len_1051_196105.p1_type_complete_len_211 | XP_022124940.1ras-related protein Rab-31 | | |
| TRINITY_DN14275_c0_g1_i1_len_1015_196430.p1_type_5prime_partial_len_211 | XP_017759668.1 discoidin domain-containing receptor 2-like | | |
| TRINITY_DN11604_c0_g1_i1_len_637_6466.p1_type_internal_len_212 | ---NA--- | | |
| TRINITY_DN25517_c0_g1_i1_len_635_66551.p1_type_internal_len_212 | XP_023678238.1canalicular multispecific organic anion transporter 1 | F:GO:0000166; P:GO:0006810; C:GO:0016021; F:GO:0016887; F:GO:0022857 | EC:3.6.1.3; EC:3.6.1.15 |
| TRINITY_DN24283_c0_g1_i1_len_2193_190267.p1_type_5prime_partial_len_211 | ---NA--- | | |
| TRINITY_DN7036_c0_g1_i1_len_636_201086.p1_type_internal_len_212 | ---NA--- | | |
| TRINITY_DN12441_c0_g1_i2_len_641_66468.p1_type_5prime_partial_len_211 | ---NA--- | | |
| TRINITY_DN8988_c0_g1_i1_len_636_201081.p1_type_internal_len_212 | ---NA--- | | |
| TRINITY_DN10726_c0_g1_i1_len_636_201078.p1_type_internal_len_212 | ---NA--- | | |
| TRINITY_DN13377_c0_g1_i1_len_636_201089.p1_type_internal_len_212 | ACU31810.1alpha2 macroglobulin isoform 2 | F:GO:0004866; C:GO:0005615; P:GO:0010951 | |
| TRINITY_DN26355_c0_g2_i2_len_636_201076.p1_type_internal_len_212 | ---NA--- | | |

| | | | |
|---|---|---|---|
| TRINITY_DN27220_c2_g1_i4_len_1636_192377.p2_type_3prime_partial_len_212 | ---NA--- | | |
| TRINITY_DN14478_c0_g1_i1_len_636_129808.p1_type_internal_len_212 | ---NA--- | | |
| TRINITY_DN36804_c0_g1_i1_len_866_237270.p1_type_complete_len_210 | ---NA--- | | |
| TRINITY_DN22365_c0_g1_i3_len_1182_195085.p2_type_5prime_partial_len_210 | ---NA--- | | |
| TRINITY_DN12467_c0_g1_i2_len_633_201139.p1_type_internal_len_211 | KYB26340.1Protein CBFA2T3-like Protein | P:GO:0006355; F:GO:0140110 | |
| TRINITY_DN16589_c0_g1_i1_len_966_160943.p1_type_complete_len_210 | XP_026280262.1coiled-coil domain-containing protein 124 | C:GO:0005829; C:GO:0005886 | |
| TRINITY_DN26993_c0_g1_i1_len_633_129848.p1_type_internal_len_211 | ODM93437.1BMP-binding endothelial regulator protein | | |
| TRINITY_DN2843_c0_g1_i1_len_632_98953.p1_type_internal_len_211 | ---NA--- | | |
| TRINITY_DN25596_c0_g1_i1_len_3051_228942.p2_type_5prime_partial_len_210 | ---NA--- | | |
| TRINITY_DN12593_c0_g1_i1_len_632_201169.p1_type_internal_len_211 | AEB00643.1glucose transporter 1 | F:GO:0005215; P:GO:0006810; C:GO:0016020 | |
| TRINITY_DN3488_c0_g1_i1_len_631_201174.p1_type_internal_len_211 | ---NA--- | | |
| TRINITY_DN2800_c0_g1_i1_len_743_128526.p1_type_complete_len_210 | XP_013774780.1UPF0454 protein C12orf49 homolog | C:GO:0016020; C:GO:0016021 | |
| TRINITY_DN8385_c0_g1_i1_len_631_201180.p1_type_internal_len_211 | XP_018012344.1 dystonin-like isoform X2 | | |
| TRINITY_DN26555_c0_g1_i1_len_653_239908.p1_type_5prime_partial_len_210 | XP_018007216.1 exostosin-3-like | C:GO:0016021; F:GO:0016757 | |
| TRINITY_DN15199_c0_g1_i1_len_713_163366.p1_type_3prime_partial_len_211 | ---NA--- | | |
| TRINITY_DN29880_c0_g1_i1_len_2314_189914.p2_type_3prime_partial_len_211 | ---NA--- | | |

| | | | |
|---|---|---|---|
| TRINITY_DN36939_c0_g1_i1_len_633_201147.p1_type_internal_len_211 | XP_018009194.1 tankyrase-1-like | | |
| TRINITY_DN6798_c0_g1_i1_len_633_201151.p1_type_internal_len_211 | XP_018021621.1PREDICTED: uncharacterized protein CG10915-like isoform X2 | | |
| TRINITY_DN17641_c0_g1_i1_len_632_201157.p1_type_internal_len_211 | ---NA--- | | |
| TRINITY_DN19000_c0_g1_i1_len_630_164532.p1_type_internal_len_211 | ---NA--- | | |
| TRINITY_DN11911_c0_g1_i1_len_633_240234.p1_type_internal_len_211 | ---NA--- | | |
| TRINITY_DN19510_c0_g1_i3_len_632_164506.p4_type_internal_len_211 | EDO36870.1predicted protein | F:GO:0016301; P:GO:0016310 | |
| TRINITY_DN15422_c0_g1_i1_len_633_36508.p1_type_internal_len_211 | ---NA--- | | |
| TRINITY_DN19301_c0_g1_i1_len_1565_267496.p1_type_complete_len_210 | XP_014245100.1TM2 domain-containing protein CG10795 | | |
| TRINITY_DN34156_c0_g1_i1_len_633_164488.p1_type_internal_len_211 | ---NA--- | | |
| TRINITY_DN21453_c0_g1_i7_len_2390_91594.p2_type_complete_len_210 | XP_024127829.1adenylosuccinate lyase | F:GO:0003824 | |
| TRINITY_DN17455_c0_g2_i1_len_631_240272.p1_type_internal_len_211 | ---NA--- | | |
| TRINITY_DN8346_c0_g1_i1_len_631_240269.p1_type_internal_len_211 | XP_013413082.1E3 ubiquitin-protein ligase UBR4 | F:GO:0008270; F:GO:0016874 | |
| TRINITY_DN29588_c0_g1_i1_len_631_240277.p1_type_internal_len_211 | ---NA--- | | |
| TRINITY_DN11479_c0_g1_i1_len_790_198736.p1_type_5prime_partial_len_210 | ---NA--- | | |
| TRINITY_DN7814_c0_g1_i1_len_630_240288.p1_type_internal_len_211 | ---NA--- | | |
| TRINITY_DN1000_c0_g1_i1_len_632_240258.p1_type_3prime_partial_len_211 | ---NA--- | | |

| | | | |
|---|---|---|---|
| TRINITY_DN30606_c0_g1_i1_len_632_240254.p1_type_internal_len_211 | XP_018012981.1 alpha-mannosidase 2x-like | | |
| TRINITY_DN19727_c0_g1_i3_len_804_128012.p1_type_3prime_partial_len_211 | ---NA--- | | |
| TRINITY_DN8880_c0_g1_i1_len_691_239345.p1_type_5prime_partial_len_209 | ---NA--- | | |
| TRINITY_DN18910_c0_g2_i1_len_1153_269315.p1_type_complete_len_209 | BAC45226.1synaptosome-associated protein of 25 kDa | F:GO:0005249; F:GO:0017075; P:GO:0071805 | |
| TRINITY_DN17537_c0_g1_i2_len_887_237076.p1_type_complete_len_209 | XP_018010284.1 ninjurin-1-like | C:GO:0016020 | |
| TRINITY_DN12734_c0_g1_i1_len_1209_159285.p1_type_5prime_partial_len_209 | XP_018027966.1 probable RNA-binding protein 18 | | |
| TRINITY_DN17923_c0_g1_i1_len_869_197811.p1_type_complete_len_209 | BAN20755.1pmp22 peroxisomal membrane protein, putative | C:GO:0016020 | |
| TRINITY_DN19372_c0_g1_i4_len_1162_269263.p1_type_complete_len_209 | AUZ97323.1vitelline membrane outer layer 1-like protein | | |
| TRINITY_DN28723_c0_g1_i1_len_916_236814.p1_type_5prime_partial_len_209 | XP_018016724.1 protein FAM69A-like | | |
| TRINITY_DN20452_c0_g1_i1_len_1055_235681.p1_type_complete_len_209 | ---NA--- | | |
| TRINITY_DN9984_c0_g1_i1_len_656_98603.p1_type_5prime_partial_len_209 | XP_021001841.1dimethylglycine dehydrogenase, mitochondrial-like | F:GO:0016491; P:GO:0055114 | |
| TRINITY_DN30705_c0_g1_i1_len_629_273719.p1_type_internal_len_210 | XP_011690427.1 ATP synthase subunit beta, mitochondrial-like | F:GO:0005524; P:GO:0015986; C:GO:0045261; F:GO:0046933; P:GO:0099132 | EC:3.6.1.3; EC:3.6.1.15 |
| TRINITY_DN11374_c0_g1_i3_len_630_164525.p1_type_internal_len_210 | ---NA--- | | |
| TRINITY_DN5030_c0_g1_i1_len_629_164540.p1_type_internal_len_210 | XP_018025998.1PREDICTED: uncharacterized protein LOC108681475 | | |
| TRINITY_DN6768_c0_g1_i1_len_796_198666.p1_type_5prime_partial_len_209 | ---NA--- | | |
| TRINITY_DN26265_c0_g1_i1_len_5789_227638.p1_type_5prime_partial_len_1791 | XP_018009184.1 serine-rich adhesin for platelets-like | | |

| | | | |
|---|---|---|---|
| TRINITY_DN22809_c0_g1_i1_len_5601_227667.p1_type_complete_len_1787 | XP_018024019.1 DNA-directed RNA polymerase I subunit RPA1-like | | |
| TRINITY_DN26242_c0_g1_i3_len_6772_227528.p1_type_5prime_partial_len_1785 | XP_012260103.1bromodomain adjacent to zinc finger domain protein 2B isoform X4 | | |
| TRINITY_DN28803_c0_g1_i1_len_6267_186896.p1_type_complete_len_1772 | XP_018022773.1 laminin subunit beta-1-like isoform X5 | | |
| TRINITY_DN26240_c0_g1_i2_len_5498_227683.p1_type_5prime_partial_len_1760 | XP_013379529.1Fanconi anemia group M protein | F:GO:0003677; F:GO:0004518; F:GO:0005524; P:GO:0006281; F:GO:0016787; P:GO:0032508; F:GO:0043140; P:GO:0090305 | |
| TRINITY_DN21616_c0_g1_i1_len_5887_227630.p1_type_complete_len_1750 | ---NA--- | | |
| TRINITY_DN23981_c0_g1_i1_len_5496_227684.p1_type_complete_len_1748 | XP_005151694.1 E3 ubiquitin-protein ligase listerin | | |
| TRINITY_DN29941_c0_g1_i1_len_6427_186871.p1_type_5prime_partial_len_1746 | ALK82309.1acetyl-CoA carboxylase | F:GO:0003989; F:GO:0005524; P:GO:0006633; F:GO:0046872 | EC:6.4.1.2 |
| TRINITY_DN26199_c0_g1_i1_len_5922_227624.p1_type_5prime_partial_len_1743 | PRD20660.1zinc finger protein | F:GO:0003676 | |
| TRINITY_DN23784_c0_g1_i1_len_5226_227741.p1_type_internal_len_1743 | XP_022195576.1transcriptional regulator ATRX homolog | | |
| 1395\|_\|c5504_f3p4_2970_isoform_c5504_full_length_coverage_3_non_full_length_coverage_4_isoform_length_2970_21531 | XP_013387202.1DNA mismatch repair protein Msh2 | | |
| 1397\|_\|c1256_f4p15_2969_isoform_c1256_full_length_coverage_4_non_full_length_coverage_15_isoform_length_2969_27711 | XP_006811353.1 serine/threonine-protein kinase Nek4-like | F:GO:0000166; F:GO:0004674; C:GO:0005737; P:GO:0023014; P:GO:0031098; P:GO:0032147 | EC:2.7.11 |
| 1398\|_\|c7822_f2p0_2969_isoform_c7822_full_length_coverage_2_non_full_length_coverage_0_isoform_length_2969_25871 | ---NA--- | | |
| 1399\|_\|c1222_f4p20_2967_isoform_c1222_full_length_coverage_4_non_full_length_coverage_20_isoform_length_2967_25365 | XP_018019322.1 protein CIP2A homolog isoform X1 | | |

| | | | |
|---|---|---|---|
| 1400\|_\|c19735_f2p11_2967_isoform_c19735_full_length_coverage_2_non_full_length_coverage_11_isoform_length_2967_28961 | XP_015370117.1 26S proteasome non-ATPase regulatory subunit 11 | | |
| 1401\|_\|c9996_f3p4_2966_isoform_c9996_full_length_coverage_3_non_full_length_coverage_4_isoform_length_2966_17321 | XP_018010073.1 alkaline phosphatase, tissue-nonspecific isozyme-like | | |
| 1402\|_\|c1675_f2p7_2966_isoform_c1675_full_length_coverage_2_non_full_length_coverage_7_isoform_length_2966_26020 | XP_018014331.1 nuclear RNA export factor 1-like | | |
| 1403\|_\|c2663_f2p1_2964_isoform_c2663_full_length_coverage_2_non_full_length_coverage_1_isoform_length_2964_16597 | ---NA--- | | |
| 1404\|_\|c18583_f2p0_2964_isoform_c18583_full_length_coverage_2_non_full_length_coverage_0_isoform_length_2964_22745 | XP_018014148.1 patched domain-containing protein 3-like isoform X2 | | |
| TRINITY_DN25751_c0_g1_i1_len_3041_228948.p1_type_3prime_partial_len_988 | XP_019761451.1 spermatogenesis-associated protein 5-like | F:GO:0000166; F:GO:0005524; C:GO:0005737; F:GO:0016887 | |
| TRINITY_DN35152_c0_g1_i1_len_629_66641.p1_type_internal_len_210 | ---NA--- | | |
| TRINITY_DN21173_c0_g1_i2_len_820_127868.p1_type_5prime_partial_len_209 | XP_018006990.1 epidermal retinol dehydrogenase 2-like | | |
| TRINITY_DN15271_c0_g1_i2_len_756_65043.p1_type_5prime_partial_len_209 | PSN44104.1Cleft lip and palate transmembrane protein 1 | C:GO:0016021 | |
| TRINITY_DN9079_c0_g1_i1_len_1071_95155.p1_type_5prime_partial_len_209 | ---NA--- | | |
| TRINITY_DN15109_c0_g2_i1_len_714_199787.p1_type_5prime_partial_len_209 | ---NA--- | | |
| TRINITY_DN7613_c0_g1_i1_len_629_240300.p1_type_internal_len_210 | ---NA--- | | |
| TRINITY_DN10891_c0_g1_i2_len_627_240339.p1_type_internal_len_210 | ---NA--- | | |
| TRINITY_DN4143_c0_g1_i1_len_627_240334.p1_type_internal_len_210 | XP_018014477.1 exostosin-1-like | | |

| | | | |
|---|---|---|---|
| TRINITY_DN35499_c0_g1_i1_len_628_240326.p1_type_internal_len_210 | XP_014186903.1 lysosomal alpha-mannosidase-like | F:GO:0004559; P:GO:0006013; F:GO:0030246 | EC:3.2.1.24 |
| TRINITY_DN28851_c0_g1_i1_len_629_240314.p1_type_internal_len_210 | XP_013399853.1codanin-1-like isoform X1 | | |
| TRINITY_DN3636_c0_g1_i1_len_630_240284.p1_type_internal_len_210 | XP_023229090.1germ cell-less protein-like 1 | | |
| TRINITY_DN357_c0_g1_i1_len_630_201213.p1_type_internal_len_210 | ---NA--- | | |
| TRINITY_DN8357_c0_g1_i1_len_629_201233.p1_type_internal_len_210 | ---NA--- | | |
| TRINITY_DN31206_c1_g1_i4_len_629_201216.p1_type_internal_len_210 | AEL23121.1EF-Tu d2 protein | F:GO:0003676; F:GO:0008270 | |
| TRINITY_DN19638_c0_g1_i1_len_833_64293.p1_type_5prime_partial_len_209 | XP_018026732.1 serine/threonine-protein phosphatase 2A regulatory subunit B'' subunit beta-like | F:GO:0005509 | |
| TRINITY_DN3212_c0_g1_i1_len_628_201243.p1_type_internal_len_210 | ---NA--- | | |
| TRINITY_DN16728_c0_g1_i1_len_630_201204.p1_type_internal_len_210 | ---NA--- | | |
| TRINITY_DN8229_c0_g1_i1_len_629_201221.p1_type_internal_len_210 | ---NA--- | | |
| TRINITY_DN18324_c0_g1_i1_len_629_201227.p1_type_internal_len_210 | KRT85747.1protein kinase | F:GO:0004672; F:GO:0005524; P:GO:0006468 | |
| TRINITY_DN39701_c0_g1_i1_len_629_201230.p1_type_internal_len_210 | XP_020853745.1thiamin pyrophosphokinase 1 isoform X2 | | |
| TRINITY_DN216_c0_g1_i1_len_629_201232.p1_type_internal_len_210 | XP_018017038.1PREDICTED: uncharacterized protein LOC108673689 isoform X1 | | |
| TRINITY_DN18438_c0_g1_i1_len_852_4477.p1_type_5prime_partial_len_209 | XP_018023529.1PREDICTED: uncharacterized protein LOC108679422 isoform X1 | | |
| TRINITY_DN8184_c0_g1_i1_len_630_201197.p1_type_internal_len_210 | XP_018027154.1PREDICTED: uncharacterized protein LOC108682497 | | |

| | | | |
|---|---|---|---|
| TRINITY_DN33155_c0_g1_i1_len_630_201196.p1_type_internal_len_210 | XP_018024208.1 protein O-mannosyl-transferase 2-like | | |
| TRINITY_DN18848_c0_g1_i1_len_629_201214.p1_type_internal_len_210 | XP_011705685.1 tubulin beta chain-like | F:GO:0003924; F:GO:0005200; F:GO:0005525; C:GO:0005874; P:GO:0007010; P:GO:0007017 | EC:3.6.1.15 |
| TRINITY_DN3399_c0_g1_i1_len_625_201312.p1_type_internal_len_209 | XP_018011709.1 zinc finger protein 239-like isoform X2 | | |
| TRINITY_DN2005_c0_g1_i1_len_625_201314.p1_type_internal_len_209 | ---NA--- | | |
| TRINITY_DN1893_c0_g1_i1_len_1013_196446.p1_type_5prime_partial_len_208 | ---NA--- | | |
| TRINITY_DN20616_c0_g1_i1_len_624_240392.p1_type_internal_len_209 | XP_013780200.1lutropin-choriogonadotropic hormone receptor-like | F:GO:0004715; C:GO:0005840; C:GO:0005887; P:GO:0007189; P:GO:0007190; F:GO:0008528; P:GO:0009755; F:GO:0016500; P:GO:0018108 | EC:2.7.10; EC:2.7.10.2 |
| TRINITY_DN10066_c1_g1_i1_len_625_240380.p1_type_internal_len_209 | ---NA--- | | |
| TRINITY_DN4782_c0_g1_i1_len_625_240376.p1_type_internal_len_209 | ---NA--- | | |
| TRINITY_DN31239_c0_g1_i3_len_4823_187187.p2_type_5prime_partial_len_208 | ---NA--- | | |
| TRINITY_DN40759_c0_g1_i1_len_774_198943.p1_type_5prime_partial_len_208 | ---NA--- | | |
| TRINITY_DN14215_c0_g1_i1_len_627_201254.p1_type_internal_len_209 | ---NA--- | | |
| TRINITY_DN2727_c0_g1_i1_len_626_273767.p1_type_internal_len_209 | XP_018021136.1 protocadherin Fat 2-like | | |
| TRINITY_DN3792_c0_g1_i1_len_783_198831.p1_type_3prime_partial_len_209 | XP_018014403.1 ras-related and estrogen-regulated growth inhibitor-like protein | F:GO:0000166; F:GO:0003924; F:GO:0005525; P:GO:0007165; C:GO:0016020 | |
| TRINITY_DN7457_c0_g1_i1_len_626_201275.p1_type_internal_len_209 | ---NA--- | | |

| | | | |
|---|---|---|---|
| TRINITY_DN3216_c0_g1_i1_len_627_201258.p1_type_internal_len_209 | ---NA--- | | |
| TRINITY_DN16379_c0_g1_i1_len_1515_232959.p1_type_5prime_partial_len_208 | XP_018020913.1 ORM1-like protein 2 | | |
| TRINITY_DN26219_c1_g1_i1_len_626_201284.p1_type_internal_len_209 | PNF25402.1hypothetical protein B7P43_G09169, partial | F:GO:0005509 | |
| TRINITY_DN14877_c0_g1_i1_len_626_201285.p1_type_internal_len_209 | ---NA--- | | |
| TRINITY_DN2042_c0_g1_i1_len_626_201287.p1_type_internal_len_209 | XP_014776999.1 transmembrane 9 superfamily member 1-like | C:GO:0016021 | |
| TRINITY_DN24584_c1_g2_i3_len_625_201292.p1_type_internal_len_209 | ---NA--- | | |
| TRINITY_DN4432_c0_g1_i1_len_625_201293.p1_type_internal_len_209 | ACH46176.1putative tubulin beta 2 | F:GO:0003924; F:GO:0005200; F:GO:0005525; C:GO:0005737; C:GO:0005874; P:GO:0007010; P:GO:0007017 | EC:3.6.1.15 |
| TRINITY_DN40941_c0_g1_i1_len_626_201281.p1_type_internal_len_209 | ACU30154.1hypoxia inducible factor 1 alpha | F:GO:0003677; C:GO:0005634; P:GO:0006355; F:GO:0046983 | |
| TRINITY_DN24491_c4_g2_i2_len_974_236328.p1_type_5prime_partial_len_208 | XP_018024864.1 longitudinals lacking protein, isoforms A/B/D/L-like isoform X1 | | |
| TRINITY_DN21588_c0_g2_i1_len_976_236308.p1_type_5prime_partial_len_208 | BAM35674.1putative reverse transcriptase | F:GO:0003964; P:GO:0006278 | |
| TRINITY_DN17766_c0_g1_i1_len_1407_124606.p2_type_5prime_partial_len_208 | ALO61081.1C-jun-amino-terminal kinase-interacting protein 4 | F:GO:0005078; F:GO:0016301; P:GO:0043410 | |
| TRINITY_DN127_c0_g1_i1_len_625_66709.p1_type_internal_len_209 | XP_023707647.1transmembrane protein 62-like isoform X2 | C:GO:0016020 | |
| TRINITY_DN18249_c0_g1_i1_len_626_66694.p1_type_internal_len_209 | ARB15844.1crustin A | C:GO:0005576; P:GO:0010466; F:GO:0030414 | |
| TRINITY_DN16721_c0_g1_i1_len_1210_194876.p2_type_5prime_partial_len_208 | XP_018011670.1 UDP-glucuronosyltransferase 2B33-like | | |
| TRINITY_DN8339_c0_g1_i1_len_1168_159523.p2_type_5prime_partial_len_208 | XP_020715039.1uncharacterized protein LOC101458455 isoform X2 | | |

| | | | |
|---|---|---|---|
| TRINITY_DN17132_c0_g1_i1_len_625_129982.p1_type_internal_len_209 | XP_018027075.1 transient receptor potential cation channel subfamily A member 1-like | | |
| TRINITY_DN21176_c0_g1_i1_len_805_237904.p1_type_5prime_partial_len_208 | ---NA--- | | |
| TRINITY_DN19710_c0_g1_i2_len_1127_195471.p1_type_5prime_partial_len_208 | XP_023726339.1ubiquitin-conjugating enzyme E2 W isoform X1 | | |
| TRINITY_DN27030_c0_g1_i1_len_628_129924.p1_type_internal_len_209 | XP_019627113.1 vacuolar protein sorting-associated protein 33B-like | P:GO:0006904; P:GO:0016192 | |
| TRINITY_DN23501_c0_g1_i1_len_805_198537.p1_type_5prime_partial_len_208 | ---NA--- | | |
| TRINITY_DN24259_c0_g1_i6_len_2920_229106.p2_type_3prime_partial_len_209 | ---NA--- | | |
| TRINITY_DN6658_c0_g1_i1_len_808_198511.p2_type_3prime_partial_len_209 | EAW65951.1HECT domain containing 1, isoform CRA_b | | |
| TRINITY_DN5068_c0_g1_i1_len_751_238510.p1_type_complete_len_208 | XP_023714415.1ubiquitin-conjugating enzyme E2 S | F:GO:0005524; F:GO:0016740 | |
| TRINITY_DN25994_c0_g1_i1_len_3552_228434.p2_type_complete_len_208 | ---NA--- | | |
| TRINITY_DN9826_c0_g2_i1_len_626_129953.p1_type_internal_len_209 | EEB13922.1zinc metalloproteinase, putative | F:GO:0016787 | |
| TRINITY_DN15283_c0_g1_i1_len_626_99037.p1_type_5prime_partial_len_208 | XP_025104058.1iron-sulfur cluster assembly 2 homolog, mitochondrial-like | F:GO:0005198; F:GO:0051536; P:GO:0097428 | |
| TRINITY_DN13173_c0_g1_i1_len_1175_234864.p1_type_complete_len_207 | ---NA--- | | |
| TRINITY_DN9730_c0_g1_i1_len_922_33934.p1_type_complete_len_207 | AWU67136.1putative phospholipase A2 | F:GO:0004623; F:GO:0005509; C:GO:0005576; P:GO:0006644; P:GO:0016042; P:GO:0050482; F:GO:0102567; F:GO:0102568 | EC:3.1.1.1; EC:3.1.1.4 |
| TRINITY_DN6736_c0_g1_i1_len_749_238538.p1_type_5prime_partial_len_207 | XP_018022778.1 mucin-17-like isoform X2 | | |
| TRINITY_DN19988_c0_g1_i2_len_1060_196023.p1_type_complete_len_207 | XP_018019276.1 ras-related protein ORAB-1-like | F:GO:0003924; F:GO:0005525 | EC:3.6.1.15 |

| | | | |
|---|---|---|---|
| TRINITY_DN2136_c0_g1_i1_len_624_129992.p1_type_internal_len_208 | XP_015126838.1 homeobox protein B-H1-like | F:GO:0003677; C:GO:0005634; P:GO:0006355; F:GO:0043565 | |
| TRINITY_DN17328_c0_g1_i1_len_1180_269166.p1_type_complete_len_207 | ACR43472.1hypothetical ferritin light-chain subunit | C:GO:0005623; P:GO:0006826; P:GO:0006879; F:GO:0008199 | |
| TRINITY_DN16550_c0_g1_i1_len_734_238721.p1_type_5prime_partial_len_207 | AJR21491.1RNA helicase | F:GO:0003723; F:GO:0032575; P:GO:0035194 | EC:3.6.1.3; EC:3.6.1.15 |
| TRINITY_DN19278_c0_g1_i1_len_1171_234889.p1_type_5prime_partial_len_207 | XP_005154610.1 ADP-ribosylation factor-like protein 3 isoform X2 | | |
| TRINITY_DN2790_c0_g1_i1_len_1010_270140.p1_type_5prime_partial_len_207 | XP_023214647.1RNA polymerase II elongation factor ELL-like | P:GO:0006368; C:GO:0008023 | |
| TRINITY_DN22084_c0_g1_i1_len_623_201350.p1_type_internal_len_208 | ACL79884.1putative articulin p60 | | |
| TRINITY_DN20894_c0_g1_i1_len_903_127221.p1_type_3prime_partial_len_208 | XP_013793110.1probable iron/ascorbate oxidoreductase DDB_G0283291 | F:GO:0016491; F:GO:0046872; F:GO:0051213; P:GO:0055114 | |
| TRINITY_DN9454_c0_g1_i1_len_622_201357.p1_type_internal_len_208 | ---NA--- | | |
| TRINITY_DN14201_c0_g1_i2_len_624_240388.p1_type_internal_len_208 | XP_018016546.1 centrosomal protein of 135 kDa-like | | |
| TRINITY_DN9962_c0_g1_i1_len_686_163734.p1_type_5prime_partial_len_207 | ---NA--- | | |
| TRINITY_DN40502_c0_g1_i1_len_623_201343.p1_type_internal_len_208 | ---NA--- | | |
| TRINITY_DN14724_c0_g1_i2_len_623_164619.p1_type_internal_len_208 | ---NA--- | | |
| TRINITY_DN5956_c0_g1_i1_len_621_201375.p1_type_internal_len_208 | ---NA--- | | |
| TRINITY_DN490_c0_g1_i1_len_1530_192941.p1_type_5prime_partial_len_207 | XP_018007909.1 prefoldin subunit 3-like | | |
| TRINITY_DN10188_c0_g1_i1_len_624_240387.p2_type_internal_len_208 | ---NA--- | | |
| TRINITY_DN42006_c0_g1_i1_len_622_201360.p1_type_internal_len_208 | ---NA--- | | |

| | | | |
|---|---|---|---|
| TRINITY_DN8025_c0_g1_i1_len_630_164528.p1_type_5prime_partial_len_207 | ---NA--- | | |
| TRINITY_DN27436_c0_g1_i3_len_1560_192757.p1_type_5prime_partial_len_207 | ---NA--- | | |
| TRINITY_DN18800_c0_g1_i2_len_1341_268401.p1_type_5prime_partial_len_207 | ---NA--- | | |
| TRINITY_DN2916_c0_g1_i1_len_623_66735.p1_type_internal_len_208 | ---NA--- | | |
| TRINITY_DN2076_c0_g1_i1_len_621_240434.p1_type_internal_len_208 | XP_002741655.1 rapamycin-insensitive companion of mTOR-like | P:GO:0031929; C:GO:0031932 | |
| TRINITY_DN4964_c0_g1_i1_len_624_201326.p2_type_internal_len_208 | XP_018022458.1 kinesin-like protein KIF17 | F:GO:0005524; C:GO:0005871; C:GO:0005874; C:GO:0005929; F:GO:0008017; F:GO:0008574; P:GO:0030030; C:GO:0032839; P:GO:0098971 | EC:3.6.1.3; EC:3.6.1.15; EC:3.6.4.4 |
| TRINITY_DN12060_c0_g1_i1_len_624_201318.p1_type_internal_len_208 | XP_003793868.1tripartite motif-containing protein 45 isoform X1 | C:GO:0005654; C:GO:0005829; F:GO:0008270; C:GO:0045171; F:GO:0046872; P:GO:0060348 | |
| TRINITY_DN11060_c0_g1_i1_len_625_201311.p1_type_internal_len_208 | XP_018021226.1 organic cation transporter protein-like | | |
| TRINITY_DN4865_c0_g1_i1_len_623_201340.p1_type_internal_len_208 | XP_018025653.1 protein ELYS-like | C:GO:0016020; C:GO:0016021 | |
| TRINITY_DN26183_c0_g1_i1_len_784_128172.p1_type_complete_len_207 | XP_018016654.1 facilitated trehalose transporter Tret1-like | | |
| TRINITY_DN26022_c0_g1_i1_len_783_198832.p1_type_5prime_partial_len_207 | XP_018011556.1 optic atrophy 3 protein homolog | | |
| TRINITY_DN17462_c0_g1_i2_len_768_199014.p1_type_5prime_partial_len_207 | PNF42938.1hypothetical protein B7P43_G11838 | | |
| TRINITY_DN30745_c0_g1_i1_len_622_240417.p1_type_internal_len_208 | ---NA--- | | |
| TRINITY_DN19550_c0_g1_i6_len_1069_160180.p1_type_5prime_partial_len_207 | ---NA--- | | |

| | | | |
|---|---|---|---|
| TRINITY_DN16718_c0_g1_i1_len_699_239219.p1_type_5prime_partial_len_207 | ---NA--- | | |
| TRINITY_DN14592_c0_g1_i1_len_673_200417.p1_type_3prime_partial_len_208 | XP_018022661.1 probable serine incorporator | | |
| TRINITY_DN10205_c0_g1_i1_len_684_200248.p1_type_5prime_partial_len_207 | ---NA--- | | |
| TRINITY_DN25843_c0_g1_i1_len_3801_228289.p1_type_5prime_partial_len_987 | XP_013776931.1isoleucine--tRNA ligase, mitochondrial-like | | |
| 1408\|_\|c10577_f2p2_2960_isoform_c10577_full_length_coverage_2_non_full_length_coverage_2_isoform_length_2960_26089 | XP_018020384.1 probable DNA-3-methyladenine glycosylase | F:GO:0003677; F:GO:0003824; F:GO:0003905; P:GO:0006284 | |
| 1409\|_\|c13528_f2p3_2958_isoform_c13528_full_length_coverage_2_non_full_length_coverage_3_isoform_length_2958_26213 | XP_018024939.1 alpha-mannosidase 2C1-like | | |
| 1410\|_\|c2798_f3p4_2958_isoform_c2798_full_length_coverage_3_non_full_length_coverage_4_isoform_length_2958_21246 | ---NA--- | | |
| 1411\|_\|c2010_f2p4_2957_isoform_c2010_full_length_coverage_2_non_full_length_coverage_4_isoform_length_2957_28147 | ---NA--- | | |
| 1412\|_\|c1972_f3p9_2957_isoform_c1972_full_length_coverage_3_non_full_length_coverage_9_isoform_length_2957_25626 | XP_021942542.1ribonuclease Z, mitochondrial-like | P:GO:0008033 | |
| TRINITY_DN20176_c0_g1_i3_len_3411_188028.p1_type_5prime_partial_len_985 | XP_018023526.1 protein O-GlcNAcase-like isoform X1 | | |
| 1414\|_\|c9138_f2p7_2955_isoform_c9138_full_length_coverage_2_non_full_length_coverage_7_isoform_length_2955_28521 | EDO37511.1predicted protein | | |
| TRINITY_DN29114_c0_g1_i4_len_4645_187251.p1_type_5prime_partial_len_985 | XP_006609710.1 protein outspread-like | | |
| TRINITY_DN23313_c0_g1_i1_len_2982_229013.p1_type_5prime_partial_len_985 | XP_006816958.1 trafficking protein particle complex subunit 8-like | | |

| | | | |
|---|---|---|---|
| TRINITY_DN23380_c0_g1_i2_len_863_127526.p1_type_complete_len_206 | ---NA--- | | |
| TRINITY_DN12281_c0_g1_i1_len_620_240452.p1_type_internal_len_207 | XP_021962584.1nose resistant to fluoxetine protein 6-like | C:GO:0016020; C:GO:0016021; F:GO:0016747 | |
| TRINITY_DN5201_c0_g1_i1_len_621_201381.p1_type_internal_len_207 | ---NA--- | | |
| TRINITY_DN12007_c0_g1_i1_len_619_201395.p1_type_internal_len_207 | XP_018006872.1 neither inactivation nor afterpotential protein C-like isoform X2 | F:GO:0000166; F:GO:0003774; F:GO:0003779; F:GO:0004672; F:GO:0005524; P:GO:0006468; C:GO:0016459 | |
| TRINITY_DN14249_c0_g1_i1_len_622_201370.p1_type_internal_len_207 | ---NA--- | | |
| TRINITY_DN19671_c0_g1_i1_len_619_201403.p1_type_internal_len_207 | ---NA--- | | |
| TRINITY_DN35185_c0_g1_i1_len_618_201417.p1_type_internal_len_207 | AAC61691.1inositol 1,4,5-trisphosphate receptor | F:GO:0005220; C:GO:0005783; C:GO:0016021; P:GO:0048016; P:GO:0070588; F:GO:0070679 | |
| TRINITY_DN9919_c0_g1_i1_len_618_201428.p1_type_internal_len_207 | AGH68885.1kazal type protease inhibitor | P:GO:0006508; F:GO:0008233 | |
| TRINITY_DN19555_c0_g1_i1_len_618_201430.p1_type_internal_len_207 | ---NA--- | | |
| TRINITY_DN17854_c0_g1_i1_len_735_238703.p1_type_complete_len_206 | ---NA--- | | |
| TRINITY_DN3366_c0_g1_i1_len_667_66088.p1_type_3prime_partial_len_207 | ---NA--- | | |
| TRINITY_DN2322_c0_g1_i1_len_620_240451.p1_type_internal_len_207 | XP_018016351.1 trimethyllysine dioxygenase, mitochondrial-like isoform X2 | F:GO:0005506; F:GO:0016491; P:GO:0045329; F:GO:0050353; F:GO:0051213; P:GO:0055114 | |
| TRINITY_DN25703_c0_g1_i1_len_877_161706.p1_type_complete_len_206 | EFX79524.1hypothetical protein DAPPUDRAFT_304428 | C:GO:0005761 | |
| TRINITY_DN15051_c0_g1_i1_len_862_161827.p1_type_complete_len_206 | KMQ83242.1transposable element tc3 transposase | F:GO:0003677; P:GO:0006313; P:GO:0015074 | |

| | | | |
|---|---|---|---|
| TRINITY_DN18192_c0_g1_i1_len_632_201162.p1_type_5prime_partial_len_206 | XP_788467.2 COMM domain-containing protein 3 | P:GO:0006814 | |
| TRINITY_DN31388_c0_g1_i1_len_998_160686.p1_type_5prime_partial_len_206 | XP_018016378.1 transmembrane protein 256 homolog isoform X1 | | |
| TRINITY_DN19606_c0_g1_i1_len_767_199032.p1_type_3prime_partial_len_207 | EFA07756.1Chloride channel protein 2-like Protein | F:GO:0005247; F:GO:0005515; C:GO:0005764; C:GO:0005770; C:GO:0016020; P:GO:0050975; P:GO:1902476; P:GO:1905146 | |
| TRINITY_DN22530_c0_g1_i1_len_1593_192597.p2_type_complete_len_206 | ---NA--- | | |
| TRINITY_DN30224_c0_g1_i1_len_1622_192447.p2_type_5prime_partial_len_206 | ---NA--- | | |
| TRINITY_DN25680_c0_g1_i1_len_620_130060.p1_type_internal_len_207 | XP_018018829.1PREDICTED: uncharacterized protein LOC108675338 | | |
| TRINITY_DN25522_c0_g1_i1_len_621_99111.p1_type_internal_len_207 | ---NA--- | | |
| TRINITY_DN16926_c0_g1_i1_len_634_273643.p1_type_3prime_partial_len_207 | ---NA--- | | |
| TRINITY_DN18553_c0_g1_i1_len_947_197024.p1_type_5prime_partial_len_206 | KZS14475.1putative Peripheral-type benzodiazepine receptor-associated protein | P:GO:0007274 | |
| TRINITY_DN19830_c0_g1_i1_len_667_129391.p1_type_5prime_partial_len_206 | ---NA--- | | |
| TRINITY_DN12941_c0_g1_i1_len_619_273868.p1_type_internal_len_207 | ---NA--- | | |
| TRINITY_DN6926_c0_g1_i2_len_620_273860.p1_type_internal_len_207 | XP_015609249.1syntaxin-6 isoform X2 | F:GO:0005484; C:GO:0005623; P:GO:0006886; C:GO:0016021; P:GO:0048193; P:GO:0061025 | |
| TRINITY_DN18100_c0_g1_i1_len_3048_29463.p2_type_complete_len_206 | OTF82139.1myocyte-specific enhancer factor 2C-like protein | F:GO:0000977; C:GO:0005634; P:GO:0045944; F:GO:0046983 | |
| TRINITY_DN21608_c0_g1_i1_len_667_129395.p1_type_complete_len_206 | ---NA--- | | |

| | | | |
|---|---|---|---|
| TRINITY_DN19511_c0_g1_i1_len_619_130081.p1_type_internal_len_207 | ---NA--- | | |
| TRINITY_DN19278_c0_g1_i1_len_1171_234889.p2_type_5prime_partial_len_206 | ---NA--- | | |
| TRINITY_DN12559_c0_g1_i1_len_741_163013.p1_type_5prime_partial_len_206 | KYM78704.1AP-3 complex subunit sigma-2 | P:GO:0006886; P:GO:0016192; C:GO:0030117 | |
| TRINITY_DN2594_c0_g1_i1_len_787_34892.p2_type_5prime_partial_len_206 | ---NA--- | | |
| TRINITY_DN16285_c0_g1_i2_len_730_163146.p1_type_5prime_partial_len_206 | ---NA--- | | |
| TRINITY_DN27391_c0_g1_i4_len_2370_189767.p2_type_complete_len_205 | XP_018011080.1 WD repeat and SOCS box-containing protein 1-like | P:GO:0035556 | |
| TRINITY_DN25163_c0_g1_i1_len_1787_191711.p1_type_complete_len_205 | XP_018023391.1 ADP-ribosylation factor-like | | |
| TRINITY_DN23930_c0_g1_i1_len_615_302159.p1_type_internal_len_206 | ---NA--- | | |
| TRINITY_DN20977_c0_g1_i3_len_1154_195277.p1_type_complete_len_205 | XP_018014250.1 PXMP2/4 family protein 4-like | | |
| TRINITY_DN16345_c0_g1_i2_len_892_270996.p1_type_complete_len_205 | XP_023216423.1meiotic nuclear division protein 1 homolog isoform X2 | C:GO:0005634 | |
| TRINITY_DN19404_c0_g1_i3_len_1186_125488.p1_type_complete_len_205 | ---NA--- | | |
| TRINITY_DN23284_c0_g1_i1_len_1194_195004.p1_type_complete_len_205 | XP_018015243.1 ecto-NOX disulfide-thiol exchanger 2-like | P:GO:0007624; C:GO:0009897; F:GO:0016491; P:GO:0055114 | |
| TRINITY_DN25627_c0_g1_i1_len_3129_228841.p1_type_complete_len_205 | ---NA--- | | |
| TRINITY_DN20853_c0_g1_i1_len_784_238125.p1_type_complete_len_205 | ---NA--- | | |
| TRINITY_DN8934_c0_g1_i2_len_1573_267479.p1_type_5prime_partial_len_205 | XP_018014038.1 lipoma HMGIC fusion partner-like 2 protein | | |
| TRINITY_DN9592_c0_g1_i1_len_1026_95440.p1_type_5prime_partial_len_205 | ---NA--- | | |

| | | | |
|---|---|---|---|
| TRINITY_DN29899_c0_g1_i1_len_615_164737.p1_type_internal_len_206 | ---NA--- | | |
| TRINITY_DN24809_c0_g1_i1_len_922_197267.p1_type_3prime_partial_len_206 | ---NA--- | | |
| TRINITY_DN9034_c0_g1_i1_len_757_238447.p1_type_3prime_partial_len_206 | ---NA--- | | |
| TRINITY_DN13757_c0_g1_i2_len_616_164726.p1_type_internal_len_206 | ---NA--- | | |
| TRINITY_DN23057_c0_g2_i1_len_707_239098.p1_type_3prime_partial_len_206 | ---NA--- | | |
| TRINITY_DN4945_c0_g1_i1_len_654_239886.p1_type_complete_len_205 | PSN33675.1putative E3 ubiquitin-protein ligase HERC1 | F:GO:0004842; P:GO:0016567; F:GO:0016874 | |
| TRINITY_DN27873_c0_g1_i1_len_618_240484.p1_type_internal_len_206 | ---NA--- | | |
| TRINITY_DN7795_c0_g1_i1_len_617_240502.p1_type_internal_len_206 | ALC78853.1adenosine monophosphate deaminase 2 | | |
| TRINITY_DN32434_c0_g1_i1_len_617_240516.p1_type_internal_len_206 | XP_024215570.1cadherin EGF LAG seven-pass G-type receptor 2 | C:GO:0016020 | |
| TRINITY_DN30690_c0_g1_i1_len_616_240529.p1_type_internal_len_206 | ASO76388.1sarcoplasmic/endoplasmic Calcium-transporting ATPase type reticulum | F:GO:0005388; F:GO:0005524; C:GO:0016021; C:GO:0033017; P:GO:0070588; P:GO:0099132 | EC:3.6.1.3; EC:3.6.3.8; EC:3.6.1.15 |
| TRINITY_DN22150_c0_g1_i1_len_670_200464.p1_type_3prime_partial_len_206 | ---NA--- | | |
| TRINITY_DN31271_c0_g1_i1_len_618_66812.p1_type_internal_len_206 | XP_013411493.1neuroblastoma-amplified sequence-like | | |
| TRINITY_DN34398_c0_g1_i1_len_671_66045.p1_type_5prime_partial_len_205 | XP_015602232.1FGGY carbohydrate kinase domain-containing protein | P:GO:0008152; F:GO:0016772 | |
| TRINITY_DN10598_c0_g1_i1_len_654_6260.p1_type_5prime_partial_len_205 | XP_018017427.1 putative cysteine proteinase CG12163 isoform X3 | | |
| TRINITY_DN13260_c0_g1_i3_len_619_201398.p1_type_internal_len_206 | XP_023708009.1MAGUK p55 subfamily member 5 isoform X2 | C:GO:0001917; C:GO:0002142; C:GO:0005886; P:GO:0007605; C:GO:0032391; C:GO:0032426; P:GO:0042052; P:GO:0050953 | |

| | | | |
|---|---|---|---|
| TRINITY_DN4763_c0_g1_i1_len_618_201425.p1_type_internal_len_206 | ---NA--- | | |
| TRINITY_DN12130_c0_g1_i1_len_617_201446.p1_type_internal_len_206 | ---NA--- | | |
| TRINITY_DN9613_c0_g1_i1_len_615_201466.p1_type_internal_len_206 | KDR21557.1Sorting nexin-17 | C:GO:0005623; P:GO:0006886; F:GO:0035091 | |
| TRINITY_DN35703_c0_g1_i1_len_615_201472.p1_type_internal_len_206 | XP_018028153.1 prostaglandin reductase 1-like | P:GO:0055114 | |
| TRINITY_DN16832_c0_g1_i1_len_749_199287.p1_type_5prime_partial_len_205 | XP_018006918.1 homer protein homolog 2-like | | |
| TRINITY_DN10092_c0_g1_i1_len_2226_265828.p1_type_5prime_partial_len_205 | ---NA--- | | |
| TRINITY_DN25080_c0_g1_i1_len_778_198877.p1_type_3prime_partial_len_206 | ---NA--- | | |
| TRINITY_DN18703_c0_g1_i1_len_789_198740.p1_type_5prime_partial_len_205 | ---NA--- | | |
| TRINITY_DN29435_c0_g1_i1_len_5407_187032.p2_type_complete_len_205 | ---NA--- | | |
| TRINITY_DN1821_c0_g1_i1_len_1023_235927.p1_type_5prime_partial_len_205 | ADV33394.1MAPK kinase 1 interacting protein 1 | F:GO:0016301; P:GO:0016310; P:GO:0032006 | |
| TRINITY_DN30808_c0_g1_i1_len_616_273910.p1_type_internal_len_206 | XP_018022122.1 neuropathy target esterase-like | | |
| TRINITY_DN11331_c0_g1_i1_len_616_273907.p1_type_internal_len_206 | XP_018016206.1PREDICTED: uncharacterized protein LOC108672944 | | |
| TRINITY_DN16655_c0_g1_i1_len_934_236667.p1_type_5prime_partial_len_205 | ---NA--- | | |
| TRINITY_DN23853_c0_g1_i1_len_617_273897.p1_type_internal_len_206 | ---NA--- | | |
| TRINITY_DN20622_c0_g1_i3_len_725_272531.p1_type_complete_len_205 | ---NA--- | | |
| TRINITY_DN26289_c0_g1_i1_len_2393_189712.p2_type_complete_len_204 | XP_023214804.1uncharacterized protein LOC111617745 | C:GO:0005741; P:GO:0035694 | |

| | | | |
|---|---|---|---|
| TRINITY_DN17875_c0_g1_i3_len_1718_296251.p1_type_complete_len_204 | ANR94955.1Ral protein | F:GO:0003924; F:GO:0005525; P:GO:0007165; C:GO:0016020 | EC:3.6.1.15 |
| TRINITY_DN16950_c0_g1_i1_len_750_65101.p1_type_5prime_partial_len_204 | XP_018011556.1 optic atrophy 3 protein homolog | | |
| TRINITY_DN21164_c0_g1_i1_len_2145_230660.p2_type_complete_len_204 | ---NA--- | | |
| TRINITY_DN11881_c0_g1_i1_len_663_6156.p1_type_complete_len_204 | XP_018026892.1 DNA replication complex GINS protein PSF3-like | C:GO:0005634; P:GO:0006260 | |
| TRINITY_DN17161_c0_g1_i1_len_995_95629.p2_type_complete_len_204 | XP_018026696.1 diphosphoinositol polyphosphate phosphohydrolase 1-like | F:GO:0016787 | |
| TRINITY_DN17589_c0_g1_i1_len_770_300410.p1_type_5prime_partial_len_204 | ---NA--- | | |
| TRINITY_DN19511_c0_g2_i4_len_1024_95450.p1_type_5prime_partial_len_204 | XP_022085821.1probable ATP-dependent DNA helicase HFM1 | F:GO:0004386 | |
| TRINITY_DN35028_c0_g1_i1_len_613_201511.p1_type_internal_len_205 | ---NA--- | | |
| TRINITY_DN22751_c0_g1_i1_len_613_201520.p1_type_internal_len_205 | ---NA--- | | |
| TRINITY_DN9828_c0_g1_i1_len_612_201527.p1_type_internal_len_205 | KZS08136.1Equilibrative nucleoside transporter 1 | F:GO:0005337; C:GO:0016021; P:GO:1901642 | |
| TRINITY_DN10314_c0_g1_i1_len_1159_94704.p2_type_complete_len_204 | XP_018018358.1PREDICTED: uncharacterized protein LOC108674884 | | |
| TRINITY_DN16097_c0_g1_i1_len_615_201469.p1_type_internal_len_205 | ---NA--- | | |
| TRINITY_DN3930_c0_g1_i1_len_615_201474.p1_type_internal_len_205 | KYM95161.1E3 ubiquitin-protein ligase UBR4 | F:GO:0008270; F:GO:0016874 | |
| TRINITY_DN34507_c0_g1_i1_len_614_201493.p1_type_internal_len_205 | ---NA--- | | |
| TRINITY_DN18798_c0_g1_i1_len_1404_233478.p1_type_5prime_partial_len_204 | XP_014663631.1 dynactin subunit 3-like | | |
| TRINITY_DN20459_c0_g1_i1_len_1638_157284.p2_type_complete_len_204 | XP_018006987.1 sialin-like isoform X3 | | |

| | | | |
|---|---|---|---|
| TRINITY_DN14947_c0_g1_i2_len_639_301838.p1_type_5prime_partial_len_204 | CDF31996.1glutathione S-transferase kappa | P:GO:0008152; P:GO:0009987; F:GO:0016491; F:GO:0016740; C:GO:0043231; C:GO:0044444 | |
| TRINITY_DN14472_c0_g1_i1_len_616_240522.p1_type_internal_len_205 | XP_002927050.1 multidrug resistance-associated protein 6 | F:GO:0000166; F:GO:0005524; C:GO:0016020; C:GO:0016021; F:GO:0016887; F:GO:0022857; F:GO:0042626; P:GO:0055085 | |
| TRINITY_DN14314_c0_g1_i1_len_616_240520.p1_type_internal_len_205 | ---NA--- | | |
| TRINITY_DN26530_c0_g1_i2_len_675_200388.p1_type_5prime_partial_len_204 | ACQ66004.1crustin Pm1 antimicrobial peptide | C:GO:0005576; P:GO:0010466; F:GO:0030414 | |
| TRINITY_DN9090_c0_g1_i1_len_612_240591.p1_type_internal_len_205 | ---NA--- | | |
| TRINITY_DN27402_c0_g1_i1_len_613_240578.p1_type_internal_len_205 | XP_018011388.1 ral GTPase-activating protein subunit alpha-1-like | | |
| TRINITY_DN30822_c0_g1_i1_len_613_240576.p1_type_internal_len_205 | ---NA--- | | |
| TRINITY_DN6845_c0_g1_i1_len_615_240536.p1_type_internal_len_205 | ---NA--- | | |
| TRINITY_DN18525_c0_g1_i1_len_1078_195870.p2_type_complete_len_204 | XP_003403369.1uncharacterized protein LOC100646249 isoform X1 | | |
| 1419\|_\|c12041_f2p7_2953_isoform_c12041_full_length_coverage_2_non_full_length_coverage_7_isoform_length_2953_22102 | ABI30653.1eukaryotic translation initiation factor 5A | F:GO:0003743; F:GO:0003746; P:GO:0006413; P:GO:0006452; F:GO:0043022; P:GO:0045901; P:GO:0045905 | |
| TRINITY_DN23563_c0_g1_i1_len_3765_153782.p1_type_complete_len_984 | PNF20745.1Phosphatidylinositol 3-kinase catalytic subunit type 3 | | |
| 1423\|_\|c1733_f2p10_2950_isoform_c1733_full_length_coverage_2_non_full_length_coverage_10_isoform_length_2950_27935 | XP_016842782.1 egalitarian isoform X2 | | |
| 1426\|_\|c1909_f2p32_2946_isoform_c1909_full_length_coverage_2_non_full_length_coverage_32_isoform_length_2946_28067 | ACO83357.1heat shock protein 90 | F:GO:0005524; P:GO:0006457; F:GO:0051082 | |

<table>
<tr><th></th><th></th><th></th><th></th></tr>
<tr><td>1427|_|c1237_f5p6_2946_isoform_c1237_full_length_coverage_5_non_full_length_coverage_6_isoform_length_2946_27702</td><td>XP_018025697.1 tubulin-specific chaperone E-like</td><td></td><td></td></tr>
<tr><td>TRINITY_DN22215_c0_g1_i1_len_3359_116.p1_type_5prime_partial_len_982</td><td>XP_018015896.1 protein HIRA-like</td><td>C:GO:0005634; P:GO:0006325; P:GO:0006351; P:GO:0006355</td><td></td></tr>
<tr><td>1429|_|c1545_f3p7_2945_isoform_c1545_full_length_coverage_3_non_full_length_coverage_7_isoform_length_2945_27835</td><td>XP_018025271.1 DNA replication factor Cdt1-like</td><td></td><td></td></tr>
<tr><td>TRINITY_DN26643_c0_g1_i1_len_3383_188046.p1_type_5prime_partial_len_981</td><td>XP_018021058.1 protein polybromo-1-like</td><td></td><td></td></tr>
<tr><td>TRINITY_DN19712_c0_g1_i1_len_3290_228670.p1_type_5prime_partial_len_981</td><td>---NA---</td><td></td><td></td></tr>
<tr><td>1432|_|c4226_f2p1_2943_isoform_c4226_full_length_coverage_2_non_full_length_coverage_1_isoform_length_2943_12361</td><td>XP_022196760.1protein GPR107</td><td></td><td></td></tr>
<tr><td>TRINITY_DN21949_c0_g1_i5_len_1002_126532.p1_type_5prime_partial_len_204</td><td>---NA---</td><td></td><td></td></tr>
<tr><td>TRINITY_DN11317_c0_g1_i3_len_616_6727.p1_type_internal_len_205</td><td>ASR74812.1leucine-rich repeat-containing protein</td><td>P:GO:0051130</td><td></td></tr>
<tr><td>TRINITY_DN13765_c0_g1_i2_len_1051_235718.p1_type_complete_len_204</td><td>---NA---</td><td></td><td></td></tr>
<tr><td>TRINITY_DN27921_c0_g1_i1_len_614_66865.p1_type_internal_len_205</td><td>XP_020711179.1beta-galactosidase-1-like protein 2</td><td></td><td></td></tr>
<tr><td>TRINITY_DN28308_c0_g1_i1_len_616_164711.p1_type_internal_len_205</td><td>XP_018011069.1 exportin-T-like</td><td></td><td></td></tr>
<tr><td>TRINITY_DN31530_c0_g1_i1_len_616_164712.p1_type_internal_len_205</td><td>XP_021918471.1WD repeat domain phosphoinositide-interacting protein 4-like isoform X2</td><td>P:GO:0006914</td><td></td></tr>
<tr><td>TRINITY_DN13106_c0_g1_i1_len_613_164777.p1_type_internal_len_205</td><td>---NA---</td><td></td><td></td></tr>
<tr><td>TRINITY_DN33779_c0_g1_i1_len_675_163873.p1_type_5prime_partial_len_204</td><td>AIS92906.1myeloid differentiation factor 88</td><td>P:GO:0002755; P:GO:0043123; F:GO:0070976</td><td></td></tr>
</table>

| | | | |
|---|---|---|---|
| TRINITY_DN14904_c0_g1_i1_len_612_99242.p1_type_internal_len_205 | EEB19050.1papilin, putative | F:GO:0004867; C:GO:0005576; P:GO:0006508; F:GO:0008233; P:GO:0010466; P:GO:0010951; F:GO:0016787; F:GO:0030414 | |
| TRINITY_DN33325_c0_g1_i1_len_612_164788.p1_type_internal_len_205 | XP_019770016.1 homeodomain-interacting protein kinase 2-like | F:GO:0004672; F:GO:0005524; P:GO:0006468 | |
| TRINITY_DN16179_c0_g1_i2_len_612_164799.p1_type_internal_len_205 | ---NA--- | | |
| TRINITY_DN7999_c0_g1_i1_len_816_271610.p1_type_5prime_partial_len_204 | XP_018013858.1 alkylglycerol monooxygenase-like | F:GO:0005506; P:GO:0008610; C:GO:0016020; C:GO:0016021; F:GO:0016491; P:GO:0055114 | |
| TRINITY_DN1299_c0_g1_i1_len_616_99188.p1_type_internal_len_205 | XP_011505440.1PREDICTED: uncharacterized protein LOC105368183 isoform X3 | | |
| TRINITY_DN6644_c0_g1_i1_len_614_130150.p1_type_internal_len_205 | XP_021930126.1adenylate cyclase type 8 | F:GO:0004016; F:GO:0005524; C:GO:0005886; P:GO:0006171; C:GO:0016021; P:GO:0035556; F:GO:0046872 | EC:4.6.1.1 |
| TRINITY_DN15723_c0_g1_i2_len_614_130152.p1_type_internal_len_205 | ACR48141.1SpAN-like protein | | |
| TRINITY_DN23659_c0_g1_i1_len_635_201117.p1_type_5prime_partial_len_203 | XP_022654938.1uncharacterized protein LOC111247798 | | |
| TRINITY_DN2596_c0_g1_i1_len_1082_33069.p1_type_complete_len_203 | ASW35104.1rho GDP-dissociation inhibitor 2-like protein | F:GO:0005094; C:GO:0005829; P:GO:0007266; C:GO:0016020; F:GO:0048365; P:GO:0050790 | |
| TRINITY_DN14647_c0_g1_i1_len_618_36698.p1_type_5prime_partial_len_203 | ---NA--- | | |
| TRINITY_DN18495_c0_g1_i1_len_2332_29940.p2_type_complete_len_203 | XP_019880024.1PREDICTED: uncharacterized protein LOC109608022, partial | | |
| TRINITY_DN21175_c0_g1_i2_len_1410_233452.p1_type_complete_len_203 | EEC08369.1CDK2- associated dual specificity phosphatase, putative | F:GO:0004722; F:GO:0004725; C:GO:0005737; P:GO:0006470; P:GO:0007050 | EC:3.1.3.16; EC:3.1.3.48 |

| | | | |
|---|---|---|---|
| TRINITY_DN19601_c0_g1_i1_len_1103_94983.p1_type_complete_len_203 | ---NA--- | | |
| TRINITY_DN23111_c1_g6_i1_len_902_161482.p1_type_complete_len_203 | XP_018008385.1PREDICTED: uncharacterized protein LOC108666082 isoform X2 | | |
| TRINITY_DN29443_c0_g1_i1_len_610_164829.p1_type_internal_len_204 | XP_018019672.1 toll-like receptor Tollo | P:GO:0007165; C:GO:0016021 | |
| TRINITY_DN5839_c0_g1_i1_len_816_162245.p1_type_complete_len_203 | ESO87686.1hypothetical protein LOTGIDRAFT_166268 | | |
| TRINITY_DN33301_c0_g1_i1_len_748_238556.p1_type_5prime_partial_len_203 | ---NA--- | | |
| TRINITY_DN8514_c0_g1_i1_len_612_6797.p1_type_internal_len_204 | XP_018014859.1 tyrosine-protein kinase Src64B-like isoform X3 | | |
| TRINITY_DN22978_c0_g1_i1_len_1328_194092.p1_type_complete_len_203 | XP_013780078.1protein TEX261-like isoform X1 | | |
| TRINITY_DN8760_c0_g1_i1_len_612_36777.p1_type_internal_len_204 | ---NA--- | | |
| TRINITY_DN462_c0_g1_i1_len_612_240596.p1_type_internal_len_204 | ---NA--- | | |
| TRINITY_DN20660_c0_g1_i1_len_1352_193936.p1_type_3prime_partial_len_204 | ---NA--- | | |
| TRINITY_DN20760_c0_g1_i1_len_612_240600.p1_type_internal_len_204 | XP_018020252.1 RNA-directed DNA polymerase from mobile element jockey-like | F:GO:0003676; F:GO:0003964; F:GO:0004523; P:GO:0006278; C:GO:0016020; C:GO:0016021; P:GO:0090502 | |
| TRINITY_DN19428_c1_g1_i2_len_689_239379.p1_type_5prime_partial_len_203 | ---NA--- | | |
| TRINITY_DN8711_c0_g1_i3_len_638_240135.p1_type_5prime_partial_len_203 | ---NA--- | | |
| TRINITY_DN12893_c0_g1_i1_len_612_99244.p1_type_internal_len_204 | ---NA--- | | |
| TRINITY_DN2321_c0_g1_i1_len_609_240647.p1_type_internal_len_204 | XP_018017292.1 amino acid transporter ANTL1-like | C:GO:0016020; C:GO:0016021 | |

| | | | |
|---|---|---|---|
| TRINITY_DN5826_c0_g1_i1_len_667_200505.p1_type_5prime_partial_len_203 | ---NA--- | | |
| TRINITY_DN14312_c0_g1_i1_len_657_98585.p1_type_5prime_partial_len_203 | ---NA--- | | |
| TRINITY_DN38990_c0_g1_i1_len_619_201401.p1_type_5prime_partial_len_203 | XP_018026620.1 titin-like isoform X3 | | |
| TRINITY_DN26866_c0_g1_i1_len_1056_235679.p1_type_5prime_partial_len_203 | ---NA--- | | |
| TRINITY_DN21641_c0_g1_i1_len_852_198022.p1_type_5prime_partial_len_203 | ---NA--- | | |
| TRINITY_DN32245_c0_g1_i1_len_610_201563.p1_type_internal_len_204 | XP_018907123.1 brefeldin A-inhibited guanine nucleotide-exchange protein 3 | | |
| TRINITY_DN14299_c0_g1_i2_len_609_274005.p1_type_internal_len_204 | ---NA--- | | |
| TRINITY_DN1789_c0_g1_i1_len_611_201546.p1_type_internal_len_204 | XP_022244928.1E3 ubiquitin-protein ligase UBR4-like isoform X3 | | |
| TRINITY_DN9532_c0_g1_i1_len_611_201543.p1_type_internal_len_204 | XP_005090794.1 extracellular tyrosine-protein kinase PKDCC-like | | |
| TRINITY_DN32357_c0_g1_i1_len_795_97073.p1_type_complete_len_203 | ---NA--- | | |
| TRINITY_DN21663_c0_g1_i1_len_611_201555.p1_type_internal_len_204 | ABN58714.1pol-like protein | F:GO:0003676; F:GO:0004523; P:GO:0090502 | |
| TRINITY_DN10618_c0_g1_i1_len_611_201550.p1_type_internal_len_204 | AET79205.1sterile-alpha and armadillo motif containing protein | P:GO:0009967; P:GO:0034128; F:GO:0035591; P:GO:0048678 | |
| TRINITY_DN30896_c0_g1_i1_len_612_66889.p1_type_internal_len_204 | ---NA--- | | |
| TRINITY_DN41075_c0_g1_i1_len_610_201566.p1_type_internal_len_204 | ---NA--- | | |
| TRINITY_DN8437_c0_g1_i1_len_890_237045.p1_type_3prime_partial_len_204 | ---NA--- | | |
| TRINITY_DN9661_c0_g1_i1_len_862_197896.p1_type_5prime_partial_len_203 | ---NA--- | | |

| | | | |
|---|---|---|---|
| TRINITY_DN20149_c0_g1_i1_len_611_66912.p1_type_internal_len_204 | ---NA--- | | |
| TRINITY_DN2270_c0_g1_i1_len_611_66908.p1_type_internal_len_204 | XP_023722290.1zinc finger and BTB domain-containing protein 17-like | F:GO:0003676 | |
| TRINITY_DN20512_c0_g1_i1_len_1258_194531.p2_type_5prime_partial_len_203 | ---NA--- | | |
| TRINITY_DN13341_c0_g1_i2_len_610_201574.p1_type_internal_len_203 | XP_018025022.1 brefeldin A-inhibited guanine nucleotide-exchange protein 1-like isoform X2 | | |
| TRINITY_DN19041_c0_g1_i2_len_609_201576.p1_type_internal_len_203 | ---NA--- | | |
| TRINITY_DN23018_c0_g1_i1_len_609_201578.p1_type_internal_len_203 | XP_018009917.1 tubulin alpha-8 chain-like | F:GO:0003924; F:GO:0005200; F:GO:0005525; C:GO:0005737; C:GO:0005874; P:GO:0007010; P:GO:0007017 | EC:3.6.1.15 |
| TRINITY_DN312_c0_g1_i1_len_609_201583.p1_type_internal_len_203 | XP_013784407.1mitogen-activated protein kinase kinase kinase 4-like | F:GO:0004672; F:GO:0005524; P:GO:0006468 | |
| TRINITY_DN22598_c0_g1_i2_len_609_201589.p1_type_internal_len_203 | ---NA--- | | |
| TRINITY_DN15570_c0_g1_i1_len_608_201603.p1_type_internal_len_203 | ODM93437.1BMP-binding endothelial regulator protein | | |
| TRINITY_DN4063_c0_g1_i1_len_607_201619.p1_type_internal_len_203 | XP_018561161.1putative inorganic phosphate cotransporter | | |
| TRINITY_DN8452_c0_g1_i1_len_607_201622.p1_type_internal_len_203 | ---NA--- | | |
| TRINITY_DN5332_c0_g1_i1_len_607_201631.p1_type_internal_len_203 | XP_018024837.1PREDICTED: uncharacterized protein LOC108680514 | | |
| TRINITY_DN8462_c0_g1_i1_len_607_201639.p1_type_internal_len_203 | XP_023341720.1tyrosine-protein phosphatase non-receptor type 21-like | C:GO:0005856 | |
| TRINITY_DN10457_c0_g1_i1_len_606_201647.p1_type_internal_len_203 | XP_014679533.1PREDICTED: uncharacterized protein LOC106819405 | | |
| TRINITY_DN5727_c0_g1_i2_len_873_197778.p1_type_5prime_partial_len_202 | XP_018027257.1 UPF0047 protein YjbQ-like | | |

| | | | |
|---|---|---|---|
| TRINITY_DN17949_c0_g1_i2_len_967_196837.p1_type_complete_len_202 | XP_002278671.2 zinc finger protein VAR3, chloroplastic | F:GO:0046872 | |
| TRINITY_DN2735_c0_g1_i1_len_616_201448.p1_type_5prime_partial_len_202 | XP_018011693.1PREDICTED: uncharacterized protein LOC108668940 | | |
| TRINITY_DN24151_c0_g1_i1_len_811_198484.p1_type_5prime_partial_len_202 | ---NA--- | | |
| TRINITY_DN19815_c0_g1_i1_len_610_201572.p1_type_internal_len_203 | XP_018011453.1 nicastrin-like | C:GO:0016020; C:GO:0016021; P:GO:0016485 | |
| TRINITY_DN11112_c0_g1_i1_len_1079_235527.p1_type_5prime_partial_len_202 | AJF94636.1cytochrome P450 CYP315a1 | F:GO:0004497; F:GO:0005506; F:GO:0016705; F:GO:0020037; P:GO:0055114 | |
| TRINITY_DN8783_c0_g1_i1_len_607_274046.p1_type_internal_len_203 | ---NA--- | | |
| TRINITY_DN15151_c0_g1_i2_len_609_274011.p1_type_internal_len_203 | XP_018014812.1 organic solute transporter alpha-like protein isoform X1 | | |
| TRINITY_DN16839_c0_g1_i1_len_1185_234799.p2_type_complete_len_202 | ---NA--- | | |
| TRINITY_DN2812_c0_g1_i1_len_610_66922.p1_type_internal_len_203 | ---NA--- | | |
| TRINITY_DN22170_c0_g1_i2_len_1016_63025.p2_type_complete_len_202 | ---NA--- | | |
| TRINITY_DN18095_c0_g1_i1_len_610_66923.p1_type_internal_len_203 | ---NA--- | | |
| TRINITY_DN28836_c0_g1_i1_len_609_66945.p1_type_internal_len_203 | XP_018009420.1PREDICTED: uncharacterized protein LOC108666956 | | |
| TRINITY_DN27215_c0_g1_i1_len_608_66966.p1_type_internal_len_203 | KFM59406.1Proton-coupled amino acid transporter 3 | C:GO:0016020; C:GO:0016021 | |
| TRINITY_DN13133_c0_g1_i1_len_609_130227.p1_type_internal_len_203 | XP_018006872.1 neither inactivation nor afterpotential protein C-like isoform X2 | | |
| TRINITY_DN21048_c0_g1_i4_len_1261_2416.p2_type_5prime_partial_len_202 | ---NA--- | | |
| TRINITY_DN17456_c0_g1_i1_len_1221_234562.p1_type_5prime_partial_len_202 | XP_018008498.1 broad-complex core protein-like isoform X1 | | |

| | | | |
|---|---|---|---|
| TRINITY_DN30729_c0_g2_i1_len_3337_188090.p2_type_complete_len_202 | PIK61335.1hypothetical protein BSL78_01755 | | |
| TRINITY_DN19969_c0_g1_i1_len_1458_93477.p1_type_complete_len_202 | XP_018024567.1 chaoptin-like | | |
| TRINITY_DN32982_c0_g1_i1_len_628_129915.p1_type_5prime_partial_len_202 | XP_018025998.1PREDICTED: uncharacterized protein LOC108681475 | | |
| TRINITY_DN21266_c0_g1_i3_len_948_63444.p1_type_complete_len_202 | ---NA--- | | |
| TRINITY_DN15421_c0_g1_i1_len_607_130266.p1_type_internal_len_203 | ---NA--- | | |
| TRINITY_DN8943_c0_g1_i1_len_607_130265.p1_type_internal_len_203 | XP_018575591.1double-stranded RNA-binding protein Staufen homolog 2 | | |
| TRINITY_DN32611_c0_g1_i1_len_610_164823.p1_type_internal_len_203 | XP_018617949.1 ELMO domain-containing protein 3 | F:GO:0005096; P:GO:0043547 | |
| TRINITY_DN11369_c0_g1_i1_len_675_163868.p1_type_5prime_partial_len_202 | ---NA--- | | |
| TRINITY_DN14568_c0_g1_i1_len_634_66566.p1_type_5prime_partial_len_202 | AEB54648.1ribosomal protein S19 | F:GO:0003735; C:GO:0005840; P:GO:0006412 | |
| TRINITY_DN15215_c0_g1_i1_len_669_98444.p1_type_5prime_partial_len_202 | KDR13501.1putative 28S ribosomal protein S6, mitochondrial | F:GO:0003735; C:GO:0005840; P:GO:0006412; F:GO:0019843 | |
| TRINITY_DN12830_c0_g1_i1_len_960_63357.p1_type_5prime_partial_len_202 | XP_013779827.1protein FAM43A-like | | |
| TRINITY_DN16538_c0_g1_i1_len_1031_33332.p1_type_5prime_partial_len_202 | XP_018019425.1 NEDD8-conjugating enzyme UBE2F-like | | |
| TRINITY_DN161_c0_g1_i1_len_609_240646.p1_type_internal_len_203 | ---NA--- | | |
| TRINITY_DN35125_c0_g1_i1_len_608_240660.p1_type_internal_len_203 | ---NA--- | | |
| TRINITY_DN17097_c0_g1_i1_len_607_240680.p1_type_internal_len_203 | ---NA--- | | |
| TRINITY_DN24216_c0_g2_i1_len_608_240663.p1_type_internal_len_203 | AXC43907.1histone acetyltransferase KAT2A | F:GO:0004402; C:GO:0005634; P:GO:0006355; P:GO:0016573 | EC:2.3.1.5; EC:2.3.1.48 |

| | | | |
|---|---|---|---|
| TRINITY_DN30861_c0_g1_i1_len_653_239913.p1_type_3prime_partial_len_203 | XP_019770667.1 probable helicase with zinc finger domain isoform X1 | F:GO:0005488 | |
| TRINITY_DN10996_c0_g1_i1_len_745_238582.p1_type_3prime_partial_len_203 | ---NA--- | | |
| 1434\|_\|c5971_f3p11_2942_isoform_c5971_full_length_coverage_3_non_full_length_coverage_11_isoform_length_2942_17010 | XP_018006803.1 longitudinals lacking protein, isoforms H/M/V-like isoform X39 | F:GO:0003676 | |
| 1435\|_\|c4485_f2p0_2942_isoform_c4485_full_length_coverage_2_non_full_length_coverage_0_isoform_length_2942_16859 | KZS05039.1putative 7SK snRNA methylphosphate capping enzyme | F:GO:0008171; F:GO:0008173; F:GO:0017069; P:GO:0032259; P:GO:0040031 | |
| 1436\|_\|c3076_f2p0_2940_isoform_c3076_full_length_coverage_2_non_full_length_coverage_0_isoform_length_2940_21314 | XP_018015361.1 reticulon-4-interacting protein 1 homolog, mitochondrial-like isoform X1 | C:GO:0005739; C:GO:0016020; C:GO:0016021; F:GO:0016491; P:GO:0055114 | |
| TRINITY_DN25304_c0_g1_i1_len_3202_228770.p1_type_complete_len_980 | KDR09660.1FAST kinase domain-containing protein 1 | F:GO:0004672; P:GO:0006468; F:GO:0016301; P:GO:0016310 | |
| 1438\|_\|c5629_f2p7_2939_isoform_c5629_full_length_coverage_2_non_full_length_coverage_7_isoform_length_2939_28404 | ---NA--- | | |
| 1439\|_\|c18259_f2p0_2939_isoform_c18259_full_length_coverage_2_non_full_length_coverage_0_isoform_length_2939_18065 | XP_014677193.1 probable ATP-dependent RNA helicase DDX11 isoform X1 | | |
| TRINITY_DN28574_c0_g1_i1_len_3203_188254.p1_type_complete_len_979 | XP_018011069.1 exportin-T-like | | |
| TRINITY_DN21841_c0_g1_i2_len_3005_294883.p1_type_5prime_partial_len_979 | XP_018025251.1 inositol-trisphosphate 3-kinase A-like isoform X3 | | |
| TRINITY_DN20374_c0_g1_i1_len_3606_228399.p1_type_5prime_partial_len_979 | ---NA--- | | |
| 1444\|_\|c2518_f4p3_2936_isoform_c2518_full_length_coverage_4_non_full_length_coverage_3_isoform_length_2936_11934 | XP_018904199.1 non-specific lipid-transfer protein-like | F:GO:0016747 | |
| TRINITY_DN22631_c0_g1_i1_len_1908_156382.p1_type_complete_len_202 | AGM32933.1Xaa-pro dipeptidase-like protein | C:GO:0005829; P:GO:0015031; C:GO:0030904; P:GO:0042147 | |

| | | | |
|---|---|---|---|
| TRINITY_DN13071_c0_g1_i4_len_792_97097.p1_type_5prime_partial_len_201 | PNF23577.1putative glutathione-specific gamma-glutamylcyclotransferase 2 | F:GO:0016829 | |
| TRINITY_DN14849_c0_g1_i1_len_917_96142.p1_type_complete_len_201 | XP_020289058.139S ribosomal protein L40, mitochondrial | C:GO:0005840 | |
| TRINITY_DN16497_c0_g1_i1_len_1243_159077.p1_type_complete_len_201 | ACR43472.1hypothetical ferritin light-chain subunit | C:GO:0005623; P:GO:0006826; P:GO:0006879; F:GO:0008199 | |
| TRINITY_DN22514_c0_g1_i1_len_1311_158698.p1_type_complete_len_201 | ---NA--- | | |
| TRINITY_DN25077_c0_g2_i1_len_604_201694.p1_type_internal_len_202 | ---NA--- | | |
| TRINITY_DN21191_c0_g1_i2_len_751_238512.p1_type_5prime_partial_len_201 | XP_011456774.1 adenine phosphoribosyltransferase | | |
| TRINITY_DN31727_c0_g1_i1_len_603_201704.p1_type_internal_len_202 | AVI10170.1inhibitor of nuclear factor kappa-B kinase epsilon isoform 2 | F:GO:0004672; F:GO:0005524; P:GO:0006468 | |
| TRINITY_DN8970_c0_g1_i1_len_603_201707.p1_type_internal_len_202 | XP_018012496.1 solute carrier family 35 member E2-like | | |
| TRINITY_DN5017_c0_g1_i1_len_603_201708.p1_type_internal_len_202 | XP_001190749.2 serine/threonine-protein phosphatase 6 regulatory ankyrin repeat subunit A-like | C:GO:0016020; C:GO:0016021 | |
| TRINITY_DN31220_c0_g1_i1_len_722_65421.p1_type_5prime_partial_len_201 | XP_020380742.1protein groucho-1-like | C:GO:0005634; P:GO:0006355 | |
| TRINITY_DN19307_c0_g1_i1_len_1119_94901.p1_type_complete_len_201 | ---NA--- | | |
| TRINITY_DN27291_c0_g2_i1_len_689_200163.p1_type_5prime_partial_len_201 | ---NA--- | | |
| TRINITY_DN19382_c0_g1_i7_len_912_161400.p1_type_complete_len_201 | ---NA--- | | |
| TRINITY_DN17581_c0_g1_i2_len_665_6130.p1_type_3prime_partial_len_202 | XP_018007569.1 DNA mismatch repair protein Msh6-like | | |
| TRINITY_DN21606_c0_g1_i1_len_747_199324.p1_type_3prime_partial_len_202 | XP_015244622.1 THAP domain-containing protein 1 A-like | | |
| TRINITY_DN32800_c0_g1_i1_len_605_240723.p1_type_internal_len_202 | XP_014486462.1 multidrug resistance-associated protein 1 isoform X4 | | |

| | | | |
|---|---|---|---|
| TRINITY_DN26795_c0_g1_i1_len_605_240716.p1_type_internal_len_202 | XP_018020584.1 epidermal growth factor receptor-like | | |
| TRINITY_DN5132_c0_g1_i1_len_607_201637.p1_type_internal_len_202 | XP_012286777.1Niemann-Pick C1 protein isoform X3 | | |
| TRINITY_DN1233_c0_g1_i1_len_606_201641.p1_type_internal_len_202 | PNF18160.1Tetratricopeptide repeat protein 21B | | |
| TRINITY_DN6993_c0_g1_i1_len_606_201643.p1_type_3prime_partial_len_202 | ---NA--- | | |
| TRINITY_DN16363_c0_g1_i2_len_605_201653.p1_type_internal_len_202 | ---NA--- | | |
| TRINITY_DN19162_c0_g2_i1_len_605_201656.p1_type_internal_len_202 | ---NA--- | | |
| TRINITY_DN5730_c0_g1_i1_len_848_237458.p1_type_3prime_partial_len_202 | ---NA--- | | |
| TRINITY_DN19153_c0_g1_i1_len_605_201660.p1_type_internal_len_202 | ASA69501.1penlectin 5-1 | | |
| TRINITY_DN997_c0_g1_i1_len_605_201664.p1_type_internal_len_202 | ---NA--- | | |
| TRINITY_DN10921_c0_g1_i1_len_605_201665.p1_type_internal_len_202 | XP_018008089.1 huntingtin-interacting protein 1-like | F:GO:0005515; C:GO:0016020 | |
| TRINITY_DN18795_c0_g1_i1_len_605_201666.p1_type_internal_len_202 | PSN48270.1Kinesin-like protein unc-104 | F:GO:0003777; F:GO:0005524; P:GO:0007018; F:GO:0008017 | EC:3.6.1.15 |
| TRINITY_DN23354_c0_g1_i1_len_664_200557.p1_type_5prime_partial_len_201 | ---NA--- | | |
| TRINITY_DN6632_c0_g1_i1_len_613_66878.p1_type_5prime_partial_len_201 | ---NA--- | | |
| TRINITY_DN9024_c0_g1_i1_len_607_201618.p1_type_internal_len_202 | ---NA--- | | |
| TRINITY_DN24041_c0_g1_i1_len_1953_59455.p2_type_complete_len_201 | ---NA--- | | |
| TRINITY_DN10760_c0_g1_i2_len_607_201621.p1_type_internal_len_202 | XP_017850063.1PREDICTED: uncharacterized protein LOC108605008 | | |

| | | | |
|---|---|---|---|
| TRINITY_DN16051_c0_g1_i1_len_607_201626.p1_type_internal_len_202 | XP_018018437.1PREDICTED: uncharacterized protein LOC108674963 | | |
| TRINITY_DN5089_c0_g1_i1_len_603_240753.p1_type_internal_len_202 | XP_012230516.1 zinc finger homeobox protein 4 isoform X4 | | |
| TRINITY_DN32973_c0_g1_i1_len_604_240740.p1_type_internal_len_202 | XP_018327238.1uncharacterized protein LOC108738347 | | |
| TRINITY_DN14513_c0_g1_i1_len_611_302221.p1_type_5prime_partial_len_201 | ---NA--- | | |
| TRINITY_DN7131_c0_g1_i1_len_935_236662.p2_type_3prime_partial_len_202 | XP_018009600.1 protein unzipped-like | | |
| TRINITY_DN28235_c1_g1_i1_len_9077_186753.p3_type_complete_len_201 | XP_018023890.1 myosin heavy chain, non-muscle-like isoform X3 | | |
| TRINITY_DN21201_c0_g1_i1_len_641_129730.p1_type_5prime_partial_len_201 | ADO00931.1calnexin | F:GO:0005509; C:GO:0005783; P:GO:0006457; C:GO:0016021; F:GO:0051082 | |
| TRINITY_DN19086_c0_g1_i1_len_660_129498.p2_type_5prime_partial_len_201 | XP_018023343.1 methyltransferase-like protein 24 | | |
| TRINITY_DN5278_c0_g1_i1_len_607_274041.p1_type_internal_len_202 | XP_023716661.1LIM domain kinase 1 isoform X2 | F:GO:0016301; P:GO:0016310; F:GO:0043167 | |
| TRINITY_DN4610_c0_g1_i2_len_880_271087.p1_type_complete_len_201 | ---NA--- | | |
| TRINITY_DN15336_c0_g1_i1_len_1125_195486.p1_type_3prime_partial_len_202 | ANW81830.1calcium-calmodulin dependent protein kinase I | F:GO:0004683; F:GO:0005516; F:GO:0005524; P:GO:0006468; P:GO:0022404 | EC:2.7.11; EC:2.7.11.17 |
| TRINITY_DN19162_c0_g1_i1_len_632_129858.p1_type_5prime_partial_len_201 | XP_018010841.1PREDICTED: uncharacterized protein LOC108668182 | | |
| TRINITY_DN7800_c0_g1_i1_len_603_164945.p1_type_internal_len_202 | ---NA--- | | |
| TRINITY_DN16860_c0_g1_i1_len_884_197668.p2_type_5prime_partial_len_201 | ---NA--- | | |
| TRINITY_DN21820_c0_g1_i6_len_1270_194449.p1_type_5prime_partial_len_201 | ---NA--- | | |

| | | | |
|---|---|---|---|
| TRINITY_DN2259_c0_g1_i1_len_606_164889.p1_type_internal_len_202 | XP_025405541.1galactosylgalactosylxylosylprotein 3-beta-glucuronosyltransferase P | C:GO:0000139; F:GO:0015018; P:GO:0050650 | EC:2.4.1.17; EC:2.4.1.135 |
| TRINITY_DN23531_c0_g2_i1_len_604_164932.p1_type_internal_len_202 | XP_018006610.1 protein abrupt-like isoform X2 | | |
| TRINITY_DN12296_c0_g1_i1_len_609_130241.p1_type_5prime_partial_len_201 | XP_018018238.1 neither inactivation nor afterpotential protein C-like | F:GO:0000166; F:GO:0003774; F:GO:0003779; F:GO:0004672; F:GO:0005524; P:GO:0006468; C:GO:0016459 | |
| TRINITY_DN22879_c0_g1_i1_len_2975_229022.p2_type_complete_len_200 | XP_023211817.1exonuclease 1-like | F:GO:0003677; C:GO:0005634; P:GO:0006281; F:GO:0035312; P:GO:0090305 | EC:3.1.11; EC:3.1.15 |
| TRINITY_DN10358_c0_g1_i1_len_601_240793.p1_type_internal_len_201 | ---NA--- | | |
| TRINITY_DN8737_c0_g1_i1_len_601_240784.p1_type_internal_len_201 | ---NA--- | | |
| TRINITY_DN26501_c0_g1_i1_len_601_240780.p1_type_internal_len_201 | XP_018018437.1PREDICTED: uncharacterized protein LOC108674963 | | |
| TRINITY_DN10107_c0_g1_i1_len_604_240736.p1_type_internal_len_201 | ---NA--- | | |
| TRINITY_DN17068_c0_g1_i1_len_914_270821.p1_type_complete_len_200 | AYN64309.1vascular endothelial growth factor 4 | | |
| TRINITY_DN14331_c0_g1_i1_len_602_240757.p2_type_5prime_partial_len_200 | ---NA--- | | |
| TRINITY_DN36836_c0_g1_i1_len_602_240754.p1_type_internal_len_201 | AEK99382.1lipoprotein receptor | F:GO:0004888; F:GO:0004930; P:GO:0007166; P:GO:0007186; C:GO:0016020; C:GO:0016021 | |
| TRINITY_DN23969_c0_g1_i5_len_741_128537.p1_type_complete_len_200 | ---NA--- | | |
| TRINITY_DN12507_c0_g1_i1_len_704_239143.p1_type_complete_len_200 | ---NA--- | | |
| TRINITY_DN20636_c0_g1_i1_len_631_240263.p1_type_5prime_partial_len_200 | ASY04977.1crustacyanin C3 | F:GO:0031409 | |

| | | | |
|---|---|---|---|
| TRINITY_DN17383_c0_g1_i2_len_694_239307.p1_type_5prime_partial_len_200 | XP_020917664.1histone H1-delta-like | C:GO:0000786; F:GO:0003690; F:GO:0005515; C:GO:0005634; P:GO:0006334; P:GO:0016584; P:GO:0030261; F:GO:0031492; P:GO:0031936; P:GO:0045910 | |
| TRINITY_DN10447_c0_g1_i1_len_604_240727.p1_type_internal_len_201 | ---NA--- | | |
| TRINITY_DN16729_c0_g1_i1_len_601_201757.p1_type_internal_len_201 | XP_026468325.1cysteine-rich secretory protein 2-like | | |
| TRINITY_DN29694_c0_g1_i1_len_603_99363.p1_type_internal_len_201 | ---NA--- | | |
| TRINITY_DN19045_c0_g1_i1_len_604_201696.p1_type_internal_len_201 | XP_018026663.1 organic cation transporter protein-like | | |
| TRINITY_DN41799_c0_g1_i1_len_603_201703.p1_type_internal_len_201 | ---NA--- | | |
| TRINITY_DN17507_c0_g1_i2_len_603_201709.p1_type_internal_len_201 | OWR50992.1vav like protein | F:GO:0005089; P:GO:0035023; P:GO:0035556; F:GO:0046872 | |
| TRINITY_DN7255_c0_g1_i1_len_603_201717.p1_type_internal_len_201 | XP_018011656.1 laminin subunit alpha-2-like | | |
| TRINITY_DN14928_c0_g1_i1_len_602_201727.p1_type_internal_len_201 | XP_018018358.1PREDICTED: uncharacterized protein LOC108674884 | | |
| TRINITY_DN7792_c0_g1_i2_len_602_201741.p1_type_internal_len_201 | XP_018015891.1 glutathione synthetase-like | | |
| TRINITY_DN18220_c0_g1_i2_len_602_130338.p1_type_internal_len_201 | ---NA--- | | |
| TRINITY_DN17109_c0_g1_i1_len_600_130387.p1_type_internal_len_201 | ---NA--- | | |
| TRINITY_DN11528_c0_g1_i1_len_600_130384.p1_type_internal_len_201 | ALL27850.1caspase 2 | F:GO:0004197; P:GO:0006508; F:GO:0008234 | |
| TRINITY_DN26471_c0_g1_i1_len_600_6988.p1_type_internal_len_201 | ---NA--- | | |
| TRINITY_DN33022_c0_g1_i1_len_687_129157.p1_type_5prime_partial_len_200 | ---NA--- | | |

| | | | |
|---|---|---|---|
| TRINITY_DN27260_c0_g1_i1_len_602_6952.p1_type_internal_len_201 | ---NA--- | | |
| TRINITY_DN19113_c0_g1_i1_len_602_6950.p1_type_internal_len_201 | ---NA--- | | |
| TRINITY_DN30168_c0_g1_i1_len_602_6947.p1_type_internal_len_201 | ---NA--- | | |
| TRINITY_DN13792_c0_g1_i1_len_603_274099.p1_type_internal_len_201 | ---NA--- | | |
| TRINITY_DN1848_c0_g1_i1_len_857_237360.p1_type_5prime_partial_len_200 | EZA54462.1Ras-like GTP-binding protein RhoL | F:GO:0000166 | |
| TRINITY_DN23957_c0_g1_i1_len_1257_194539.p1_type_complete_len_200 | ABI98680.1ubiquitin-conjugating enzyme H1 | F:GO:0005524; F:GO:0016740 | |
| TRINITY_DN26070_c0_g1_i3_len_815_237792.p1_type_complete_len_200 | ---NA--- | | |
| TRINITY_DN22393_c0_g1_i2_len_917_197311.p1_type_complete_len_200 | ---NA--- | | |
| TRINITY_DN31052_c0_g1_i1_len_676_163858.p1_type_5prime_partial_len_200 | XP_014664686.1 dual specificity protein phosphatase 16-like | P:GO:0006470; F:GO:0008138; P:GO:0016311 | |
| TRINITY_DN9926_c0_g1_i1_len_602_164970.p1_type_internal_len_201 | XP_026284686.1zinc finger MYND domain-containing protein 11 isoform X3 | | |
| TRINITY_DN2166_c0_g1_i1_len_732_163122.p1_type_5prime_partial_len_200 | ---NA--- | | |
| TRINITY_DN34179_c0_g1_i1_len_603_164949.p1_type_internal_len_201 | ---NA--- | | |
| TRINITY_DN30035_c0_g1_i1_len_998_196569.p1_type_5prime_partial_len_200 | ESO98647.1hypothetical protein LOTGIDRAFT_180523 | F:GO:0003676 | |
| TRINITY_DN16178_c0_g1_i1_len_1001_196546.p1_type_5prime_partial_len_200 | ---NA--- | | |
| TRINITY_DN22082_c0_g1_i1_len_1007_196500.p1_type_5prime_partial_len_200 | XP_023937899.1zinc finger protein 436-like isoform X2 | | |
| TRINITY_DN29608_c0_g1_i1_len_602_67068.p1_type_internal_len_201 | ---NA--- | | |

| | | | |
|---|---|---|---|
| TRINITY_DN19782_c0_g1_i1_len_601_67073.p1_type_internal_len_201 | BAM35674.1putative reverse transcriptase | F:GO:0003964; P:GO:0006278 | |
| TRINITY_DN21985_c0_g1_i2_len_726_65367.p1_type_5prime_partial_len_199 | ---NA--- | | |
| TRINITY_DN35496_c0_g1_i1_len_599_201810.p1_type_internal_len_200 | ---NA--- | | |
| TRINITY_DN25084_c2_g3_i3_len_1627_60270.p2_type_complete_len_199 | XP_018006492.1 sialin-like | | |
| TRINITY_DN15650_c0_g1_i1_len_599_201794.p1_type_3prime_partial_len_200 | ---NA--- | | |
| TRINITY_DN16439_c0_g1_i1_len_597_201832.p1_type_internal_len_200 | XP_013394272.1stomatin-like protein 1 isoform X3 | C:GO:0016020; C:GO:0016021 | |
| TRINITY_DN9832_c0_g1_i1_len_597_201841.p1_type_internal_len_200 | ---NA--- | | |
| TRINITY_DN29147_c1_g1_i2_len_3221_188223.p1_type_5prime_partial_len_978 | ASW35094.1fat-like cadherin-related tumor suppressor like-protein | F:GO:0005509; C:GO:0016021 | |
| TRINITY_DN25342_c1_g1_i2_len_3521_228452.p1_type_complete_len_978 | XP_020823602.1serine/threonine-protein phosphatase 6 regulatory subunit 3 isoform X7 | | |
| TRINITY_DN29527_c0_g1_i1_len_3791_187711.p1_type_complete_len_977 | XP_026284522.1protein SMG8 isoform X1 | | |
| TRINITY_DN19855_c0_g1_i2_len_3501_121941.p1_type_complete_len_977 | PNF39756.1E3 ubiquitin-protein ligase Su(dx) | P:GO:0006511; P:GO:0016567; F:GO:0016874; F:GO:0061630 | |
| TRINITY_DN24926_c0_g1_i1_len_5239_227738.p1_type_complete_len_977 | XP_023714629.1MLX-interacting protein isoform X1 | F:GO:0046983 | |
| TRINITY_DN30512_c1_g1_i4_len_4020_187573.p1_type_5prime_partial_len_977 | XP_015117200.1 sushi, von Willebrand factor type A, EGF and pentraxin domain-containing protein 1 | | |
| 1451\|_\|c18343_f6p5_2931_isoform_c18343_full_length_coverage_6_non_full_length_coverage_5_isoform_length_2931_28802 | XP_023716139.1BUB3-interacting and GLEBS motif-containing protein ZNF207 isoform X2 | F:GO:0003677 | |

| | | | |
|---|---|---|---|
| TRINITY_DN24334_c0_g1_i2_len_3217_228754.p1_type_5prime_partial_len_977 | XP_002934435.1 GPI ethanolamine phosphate transferase 1 | F:GO:0003824; C:GO:0005789; P:GO:0006506; C:GO:0016020; C:GO:0016021; F:GO:0016740; F:GO:0051377 | |
| 1453\|_\|c18387_f10p14_2930_isoform_c18387_full_length_coverage_10_non_full_length_coverage_14_isoform_length_2930_26391 | XP_018025485.1 ribonucleoside-diphosphate reductase large subunit-like | F:GO:0004748; F:GO:0005524; C:GO:0005971; P:GO:0006260; P:GO:0009263; P:GO:0055114 | EC:1.17.4; EC:1.17.4.1 |
| 1454\|_\|c2541_f3p1_2929_isoform_c2541_full_length_coverage_3_non_full_length_coverage_1_isoform_length_2929_11954 | KYN06648.1Leucine-rich repeat-containing protein 28 | | |
| TRINITY_DN5831_c0_g1_i1_len_598_201827.p1_type_internal_len_200 | ---NA--- | | |
| TRINITY_DN18825_c0_g1_i1_len_598_201826.p1_type_internal_len_200 | XP_018019402.1 protein slit-like | F:GO:0005509; C:GO:0005576 | |
| TRINITY_DN15811_c0_g1_i1_len_685_98254.p1_type_5prime_partial_len_199 | XP_018021886.1 degenerin-like protein asic-1 | | |
| TRINITY_DN5042_c0_g1_i1_len_598_201820.p1_type_internal_len_200 | ---NA--- | | |
| TRINITY_DN16375_c0_g1_i3_len_919_127108.p1_type_complete_len_199 | XP_026465565.1cytochrome c oxidase assembly protein COX16 homolog, mitochondrial | C:GO:0016020 | |
| TRINITY_DN2584_c0_g1_i1_len_598_165028.p1_type_internal_len_200 | ---NA--- | | |
| TRINITY_DN24597_c0_g1_i2_len_1280_234178.p1_type_complete_len_199 | XP_014614869.1 phosphomevalonate kinase | | |
| TRINITY_DN15749_c0_g1_i2_len_598_165021.p3_type_internal_len_200 | ---NA--- | | |
| TRINITY_DN18329_c0_g1_i3_len_1315_2225.p1_type_3prime_partial_len_200 | ---NA--- | | |
| TRINITY_DN25047_c0_g1_i2_len_898_197517.p1_type_3prime_partial_len_200 | KOF95963.1hypothetical protein OCBIM_22036769mg | F:GO:0003676; F:GO:0046872 | |

| | | | |
|---|---|---|---|
| TRINITY_DN10819_c0_g1_i1_len_601_67077.p1_type_internal_len_200 | ---NA--- | | |
| TRINITY_DN16894_c0_g1_i2_len_599_99437.p1_type_internal_len_200 | XP_018025051.1 sulfotransferase 1A3-like | | |
| TRINITY_DN35603_c0_g1_i1_len_761_199104.p1_type_3prime_partial_len_200 | ---NA--- | | |
| TRINITY_DN20373_c0_g1_i1_len_600_36954.p1_type_internal_len_200 | PRD25409.1zinc finger protein | F:GO:0003676 | |
| TRINITY_DN13912_c0_g1_i1_len_597_99472.p1_type_internal_len_200 | XP_018025235.1 major facilitator superfamily domain-containing protein 4A-like | | |
| TRINITY_DN18616_c0_g1_i1_len_798_198636.p1_type_5prime_partial_len_199 | ---NA--- | | |
| TRINITY_DN2571_c0_g1_i1_len_798_198628.p1_type_5prime_partial_len_199 | ---NA--- | | |
| TRINITY_DN19366_c0_g1_i4_len_1810_266760.p2_type_complete_len_199 | KYB25197.16-phosphofructo-2-kinase/fructose-2,6-bisphosphatase 1-like Protein | F:GO:0003873; F:GO:0004331; F:GO:0005524; C:GO:0005829; P:GO:0006000; P:GO:0006003; P:GO:0016311; P:GO:0046835 | EC:3.1.3.23; EC:3.1.3.46; EC:2.7.1.105 |
| TRINITY_DN10898_c0_g1_i2_len_765_199053.p1_type_3prime_partial_len_200 | XP_022203152.1 spectrin beta chain-like | | |
| TRINITY_DN12514_c0_g1_i1_len_768_199006.p1_type_3prime_partial_len_200 | XP_018013591.1 alpha-(1,6)-fucosyltransferase-like | F:GO:0016740 | |
| TRINITY_DN2582_c0_g1_i1_len_600_201773.p1_type_internal_len_200 | XP_018010986.1PREDICTED: uncharacterized protein LOC108668307 | | |
| TRINITY_DN18278_c0_g1_i4_len_1103_269614.p1_type_5prime_partial_len_199 | ---NA--- | | |
| TRINITY_DN8974_c0_g1_i1_len_599_67121.p1_type_internal_len_200 | XP_017492262.1 putative glucosylceramidase 3 | P:GO:0006665; F:GO:0016798 | |
| TRINITY_DN14955_c0_g1_i1_len_780_162606.p1_type_5prime_partial_len_199 | ---NA--- | | |
| TRINITY_DN9614_c0_g1_i1_len_599_67119.p1_type_internal_len_200 | XP_022241171.1arylsulfatase B-like | F:GO:0008484 | |

| | | | |
|---|---|---|---|
| TRINITY_DN8778_c0_g1_i1_len_682_239472.p1_type_3prime_partial_len_200 | ---NA--- | | |
| TRINITY_DN6111_c1_g1_i1_len_597_240857.p1_type_internal_len_200 | XP_018017205.1 carboxypeptidase E-like | F:GO:0004181; P:GO:0006508; F:GO:0008270 | EC:3.4.17 |
| TRINITY_DN8296_c0_g1_i1_len_597_240849.p1_type_internal_len_200 | ---NA--- | | |
| TRINITY_DN33832_c0_g1_i1_len_598_240841.p1_type_internal_len_200 | ---NA--- | | |
| TRINITY_DN26986_c0_g1_i1_len_599_240818.p1_type_internal_len_200 | ---NA--- | | |
| TRINITY_DN8358_c0_g1_i1_len_599_240817.p1_type_internal_len_200 | ---NA--- | | |
| TRINITY_DN12010_c0_g1_i1_len_600_240808.p1_type_internal_len_200 | XP_018027727.1PREDICTED: uncharacterized protein LOC108682971 | | |
| TRINITY_DN341_c0_g1_i1_len_600_240807.p1_type_internal_len_200 | XP_011439399.1 protein tilB homolog | P:GO:0006996; C:GO:0044464 | |
| TRINITY_DN28018_c0_g1_i1_len_600_240799.p1_type_internal_len_200 | XP_015192409.1 E3 ubiquitin-protein ligase UBR4 | | |
| TRINITY_DN19082_c0_g1_i1_len_681_129228.p1_type_5prime_partial_len_199 | XP_022198815.1zinc finger protein OZF-like | | |
| TRINITY_DN14264_c0_g1_i1_len_599_130400.p1_type_internal_len_200 | ---NA--- | | |
| TRINITY_DN31349_c0_g1_i1_len_689_200162.p1_type_5prime_partial_len_199 | ---NA--- | | |
| TRINITY_DN34121_c0_g1_i1_len_740_199432.p2_type_5prime_partial_len_199 | XP_020711066.1ets DNA-binding protein pokkuri | | |
| TRINITY_DN13826_c0_g1_i1_len_1640_232368.p2_type_complete_len_199 | XP_018026323.1 FIT family protein CG10671-like | C:GO:0005789; C:GO:0016020; C:GO:0016021; P:GO:0019915 | |
| TRINITY_DN20946_c0_g1_i1_len_1063_126153.p2_type_5prime_partial_len_198 | ---NA--- | | |
| TRINITY_DN17170_c0_g1_i1_len_1245_268864.p1_type_complete_len_198 | XP_025855863.15-formyltetrahydrofolate cyclo-ligase isoform X1 | F:GO:0000166; C:GO:0005739; P:GO:0009396; F:GO:0030272; P:GO:0035999; F:GO:0043167 | EC:6.3.3.2 |

| | | | |
|---|---|---|---|
| TRINITY_DN18066_c0_g1_i1_len_595_130448.p2_type_internal_len_199 | ---NA--- | | |
| TRINITY_DN37716_c0_g1_i1_len_594_201889.p1_type_internal_len_199 | XP_018025310.1 sodium/myo-inositol cotransporter 2-like | F:GO:0005412; C:GO:0005886; C:GO:0016021; P:GO:0055085 | |
| TRINITY_DN9947_c0_g1_i1_len_595_201882.p1_type_internal_len_199 | XP_015520525.1 myotubularin-related protein 13-like | | |
| TRINITY_DN19112_c0_g1_i1_len_595_201877.p1_type_internal_len_199 | KZS05703.1Uncharacterized protein APZ42_030953 | | |
| TRINITY_DN35469_c0_g1_i1_len_596_201853.p1_type_internal_len_199 | ---NA--- | | |
| TRINITY_DN7110_c0_g1_i1_len_596_201845.p1_type_internal_len_199 | ---NA--- | | |
| TRINITY_DN25764_c0_g1_i1_len_597_201842.p1_type_internal_len_199 | ---NA--- | | |
| TRINITY_DN24163_c0_g1_i1_len_752_162914.p1_type_3prime_partial_len_199 | PIK34189.1hypothetical protein BSL78_28988 | | |
| TRINITY_DN2806_c0_g1_i1_len_596_165062.p1_type_internal_len_199 | ---NA--- | | |
| TRINITY_DN18994_c0_g1_i1_len_595_302436.p1_type_internal_len_199 | ---NA--- | | |
| TRINITY_DN6466_c0_g1_i1_len_595_165079.p1_type_internal_len_199 | XP_023333559.1SET and MYND domain-containing protein 4-like | | |
| TRINITY_DN10373_c0_g1_i1_len_597_130420.p1_type_internal_len_199 | ---NA--- | | |
| TRINITY_DN37286_c0_g1_i1_len_597_201839.p1_type_internal_len_199 | XP_018012108.1 serine/arginine repetitive matrix protein 2-like | | |
| TRINITY_DN17199_c0_g1_i1_len_597_201835.p1_type_internal_len_199 | ---NA--- | | |
| TRINITY_DN11868_c0_g1_i1_len_598_201829.p1_type_internal_len_199 | AHH32890.1integrin beta 1 | P:GO:0007155; P:GO:0007229; C:GO:0016021 | |
| TRINITY_DN40787_c0_g1_i1_len_598_201814.p1_type_internal_len_199 | AKL71620.1juvenile hormone epoxide hydrolase | C:GO:0005789; P:GO:0019439; F:GO:0033961 | EC:3.3.2.9 |

| | | | |
|---|---|---|---|
| TRINITY_DN10768_c0_g1_i1_len_687_200208.p1_type_3prime_partial_len_199 | ---NA--- | | |
| TRINITY_DN16561_c0_g1_i1_len_836_162051.p1_type_5prime_partial_len_198 | AGG19160.1programmed cell death protein 5 | F:GO:0003677 | |
| TRINITY_DN22469_c0_g1_i2_len_688_200190.p1_type_3prime_partial_len_199 | ---NA--- | | |
| TRINITY_DN21951_c1_g1_i1_len_1689_157084.p1_type_5prime_partial_len_198 | XP_023712122.1protein phosphatase inhibitor 2-like | P:GO:0043666 | |
| TRINITY_DN21703_c0_g1_i1_len_3258_188180.p2_type_complete_len_198 | ---NA--- | | |
| TRINITY_DN26148_c1_g1_i6_len_2884_229163.p3_type_3prime_partial_len_199 | ---NA--- | | |
| TRINITY_DN14180_c0_g1_i1_len_642_200935.p1_type_5prime_partial_len_198 | XP_018009879.1PREDICTED: uncharacterized protein LOC108667371 | | |
| TRINITY_DN4119_c0_g1_i1_len_597_240859.p1_type_internal_len_199 | ---NA--- | | |
| TRINITY_DN10693_c0_g1_i1_len_597_240864.p1_type_internal_len_199 | XP_019758876.1 fibroblast growth factor receptor homolog 1-like isoform X2 | F:GO:0004714; F:GO:0005488; P:GO:0007169; P:GO:0007399; P:GO:0009653; P:GO:0009888; P:GO:0010033; C:GO:0016020; C:GO:0044464; P:GO:0048513 | EC:2.7.10; EC:2.7.10.1; EC:2.7.11 |
| TRINITY_DN28970_c0_g1_i1_len_596_240877.p1_type_internal_len_199 | EEB10952.1mixed-lineage leukemia protein, mll, putative | | |
| TRINITY_DN2591_c0_g1_i1_len_595_240891.p1_type_internal_len_199 | XP_025087688.1tetratricopeptide repeat protein 17-like isoform X4 | | |
| TRINITY_DN31128_c0_g1_i1_len_594_240907.p1_type_internal_len_199 | XP_008510089.1 host cell factor 1-like | C:GO:0048188; C:GO:0071339 | |
| TRINITY_DN10763_c0_g1_i1_len_594_240908.p1_type_internal_len_199 | ---NA--- | | |
| TRINITY_DN6642_c0_g1_i1_len_594_240914.p1_type_internal_len_199 | ---NA--- | | |
| TRINITY_DN24486_c0_g1_i1_len_2673_229482.p2_type_3prime_partial_len_199 | ---NA--- | | |

| | | | |
|---|---|---|---|
| TRINITY_DN1157_c0_g1_i1_len_597_240850.p1_type_internal_len_199 | ---NA--- | | |
| TRINITY_DN15706_c0_g1_i1_len_754_128409.p1_type_5prime_partial_len_198 | XP_024884231.1tetratricopeptide repeat protein 7B-like | | |
| TRINITY_DN4118_c0_g1_i1_len_594_99530.p1_type_3prime_partial_len_199 | AHH29321.1protein inhibitor of activated STAT | F:GO:0008270 | |
| TRINITY_DN19931_c0_g1_i2_len_596_99486.p1_type_internal_len_199 | ---NA--- | | |
| TRINITY_DN17465_c0_g2_i1_len_596_99482.p1_type_internal_len_199 | CAA57964.1putative serine proteinase inhibitor | C:GO:0005615 | |
| TRINITY_DN15912_c0_g1_i1_len_596_67154.p1_type_internal_len_199 | ---NA--- | | |
| TRINITY_DN10281_c0_g1_i1_len_600_99413.p1_type_5prime_partial_len_198 | ---NA--- | | |
| TRINITY_DN2812_c0_g1_i1_len_638_201033.p1_type_5prime_partial_len_198 | AWU67223.1putative chitinase | F:GO:0004568; C:GO:0005576; P:GO:0006030; F:GO:0008061; P:GO:0008152; F:GO:0016787; F:GO:0016798 | |
| TRINITY_DN14956_c0_g1_i1_len_744_199364.p1_type_5prime_partial_len_198 | XP_017892893.1sodium- and chloride-dependent GABA transporter ine | F:GO:0005328; C:GO:0005886; P:GO:0006836; P:GO:0009987; C:GO:0016021; P:GO:0032501; P:GO:0055085 | |
| TRINITY_DN25767_c0_g1_i1_len_2141_190426.p4_type_complete_len_198 | ---NA--- | | |
| TRINITY_DN1201_c0_g1_i1_len_596_7044.p1_type_internal_len_199 | AKK75090.1DNA (cytosine-5-)-methyltransferase 1 | F:GO:0005488; F:GO:0008168; P:GO:0032259 | |
| TRINITY_DN21668_c0_g1_i3_len_1970_123184.p2_type_complete_len_197 | ---NA--- | | |
| TRINITY_DN17108_c0_g1_i2_len_900_4159.p1_type_complete_len_197 | AKB96201.1heat shock protein | | |
| TRINITY_DN17078_c0_g1_i1_len_595_130456.p1_type_internal_len_198 | ---NA--- | | |

| | | | |
|---|---|---|---|
| TRINITY_DN32019_c0_g1_i1_len_655_98616.p1_type_5prime_partial_len_197 | ---NA--- | | |
| TRINITY_DN15848_c0_g1_i4_len_657_98591.p1_type_5prime_partial_len_197 | ---NA--- | | |
| TRINITY_DN19573_c0_g1_i7_len_592_165143.p1_type_internal_len_198 | ---NA--- | | |
| TRINITY_DN17520_c0_g1_i1_len_700_35740.p1_type_3prime_partial_len_198 | XP_003397024.1tetratricopeptide repeat protein 1 | | |
| TRINITY_DN15813_c0_g1_i1_len_592_37079.p1_type_internal_len_198 | ---NA--- | | |
| TRINITY_DN16481_c0_g1_i1_len_591_37095.p1_type_internal_len_198 | ---NA--- | | |
| TRINITY_DN31012_c0_g1_i2_len_3817_187691.p2_type_complete_len_197 | ---NA--- | | |
| TRINITY_DN25656_c0_g1_i1_len_595_165072.p1_type_internal_len_198 | ---NA--- | | |
| TRINITY_DN4355_c0_g1_i1_len_595_165066.p1_type_internal_len_198 | ---NA--- | | |
| TRINITY_DN2344_c0_g1_i1_len_606_240687.p1_type_5prime_partial_len_197 | XP_026468325.1cysteine-rich secretory protein 2-like | | |
| TRINITY_DN7187_c0_g1_i1_len_592_201949.p1_type_internal_len_198 | XP_012270807.1uncharacterized protein LOC105694570 | | |
| TRINITY_DN21685_c0_g1_i1_len_2752_91295.p2_type_5prime_partial_len_197 | ---NA--- | | |
| TRINITY_DN142_c0_g1_i1_len_592_201946.p1_type_internal_len_198 | XP_788180.2 GPI mannosyltransferase 3 | F:GO:0000030; C:GO:0005789; P:GO:0006506 | |
| TRINITY_DN32668_c0_g1_i1_len_593_201936.p1_type_internal_len_198 | PNF22200.1SPRY domain-containing protein 3 | | |
| 1455\|_\|c2668_f2p1_2929_isoform_c2668_full_length_coverage_2_non_full_length_coverage_1_isoform_length_2929_16602 | XP_020613238.1TATA-binding protein-associated factor 172-like | F:GO:0005524; C:GO:0005654; P:GO:0035562 | |

| | | | |
|---|---|---|---|
| 1458\|_\|c1889_f2p6_2926_isoform_c1889_full_length_coverage_2_non_full_length_coverage_6_isoform_length_2926_25565 | ---NA--- | | |
| 1459\|_\|c15322_f3p2_2923_isoform_c15322_full_length_coverage_3_non_full_length_coverage_2_isoform_length_2923_22315 | XP_013388894.1WD repeat-containing protein 43-like | | |
| 1460\|_\|c18056_f2p0_2923_isoform_c18056_full_length_coverage_2_non_full_length_coverage_0_isoform_length_2923_22662 | AYD41601.1lysosome-associated membrane glycoprotein 1-like protein | C:GO:0016020 | |
| 1461\|_\|c12886_f3p0_2923_isoform_c12886_full_length_coverage_3_non_full_length_coverage_0_isoform_length_2923_17551 | ODM95807.1Reticulocalbin-2 | F:GO:0005509 | |
| 1462\|_\|c1847_f2p2_2923_isoform_c1847_full_length_coverage_2_non_full_length_coverage_2_isoform_length_2923_25533 | ---NA--- | | |
| 1466\|_\|c1755_f7p4_2922_isoform_c1755_full_length_coverage_7_non_full_length_coverage_4_isoform_length_2922_15978 | XP_018013989.1 protein LSM14 homolog A-like isoform X1 | | |
| 1468\|_\|c15598_f2p12_2921_isoform_c15598_full_length_coverage_2_non_full_length_coverage_12_isoform_length_2921_26272 | ---NA--- | | |
| TRINITY_DN20924_c0_g1_i1_len_3062_228927.p1_type_complete_len_973 | XP_025831741.1uncharacterized protein LOC108739706 isoform X2 | | |
| 1470\|_\|c1836_f2p3_2919_isoform_c1836_full_length_coverage_2_non_full_length_coverage_3_isoform_length_2919_25524 | ---NA--- | | |
| TRINITY_DN15631_c0_g1_i1_len_593_201932.p1_type_internal_len_198 | ---NA--- | | |
| TRINITY_DN39196_c0_g1_i1_len_593_201915.p1_type_internal_len_198 | ---NA--- | | |
| TRINITY_DN17318_c0_g1_i1_len_593_201904.p1_type_internal_len_198 | XP_025027445.1kelch domain-containing protein 10 isoform X2 | | |

| | | | |
|---|---|---|---|
| TRINITY_DN15044_c0_g1_i1_len_594_201899.p1_type_internal_len_198 | ---NA--- | | |
| TRINITY_DN13759_c1_g1_i1_len_595_201883.p1_type_internal_len_198 | XP_018024578.1 G-protein coupled receptor moody-like | | |
| TRINITY_DN17050_c0_g1_i1_len_1360_193901.p2_type_5prime_partial_len_197 | ACP19561.1clip domain serine proteinase 2 | F:GO:0004252; P:GO:0006508 | EC:3.4.21 |
| TRINITY_DN20497_c0_g1_i1_len_674_200414.p1_type_3prime_partial_len_198 | XP_018025707.1 intraflagellar transport protein 43 homolog | C:GO:0030991; C:GO:0031514 | |
| TRINITY_DN8643_c0_g1_i1_len_1013_95518.p1_type_5prime_partial_len_197 | ---NA--- | | |
| TRINITY_DN8797_c0_g1_i1_len_630_240287.p1_type_complete_len_197 | ---NA--- | | |
| TRINITY_DN27408_c0_g1_i2_len_630_201194.p1_type_5prime_partial_len_197 | AAZ29608.1C-type lectin | F:GO:0030246 | |
| TRINITY_DN17485_c0_g1_i2_len_1577_267462.p2_type_complete_len_197 | EFX82153.1hypothetical protein DAPPUDRAFT_223834 | F:GO:0008146 | |
| TRINITY_DN7481_c0_g1_i1_len_595_67183.p2_type_internal_len_198 | ---NA--- | | |
| TRINITY_DN5719_c0_g1_i1_len_629_201226.p1_type_5prime_partial_len_197 | ---NA--- | | |
| TRINITY_DN7850_c0_g1_i1_len_594_240904.p1_type_internal_len_198 | ---NA--- | | |
| TRINITY_DN7635_c0_g1_i1_len_593_240925.p1_type_internal_len_198 | AGK89909.1nicotinic acetylcholine receptor subunit alpha 10 | F:GO:0004888; F:GO:0005216; F:GO:0005230; P:GO:0006811; C:GO:0016020; C:GO:0016021; P:GO:0034220 | |
| TRINITY_DN11853_c0_g1_i1_len_800_198607.p1_type_5prime_partial_len_197 | XP_018010575.1 elongation of very long chain fatty acids protein 6-like isoform X1 | P:GO:0006629; F:GO:0016747 | |
| TRINITY_DN24070_c0_g1_i2_len_800_198610.p1_type_complete_len_197 | XP_018018850.1 proclotting enzyme-like isoform X1 | F:GO:0008233 | |
| TRINITY_DN1143_c0_g1_i1_len_592_240956.p1_type_internal_len_198 | ---NA--- | | |

| | | | |
|---|---|---|---|
| TRINITY_DN12452_c0_g1_i1_len_595_201872.p1_type_internal_len_198 | XP_018025797.1 zinc finger protein Noc-like | | |
| TRINITY_DN31455_c0_g1_i1_len_595_201865.p1_type_internal_len_198 | ---NA--- | | |
| TRINITY_DN7956_c0_g1_i1_len_595_240902.p1_type_internal_len_198 | XP_018935543.1 DNA polymerase subunit gamma-1-like | | |
| TRINITY_DN22789_c0_g1_i2_len_2383_230042.p1_type_complete_len_197 | ---NA--- | | |
| TRINITY_DN12540_c0_g1_i1_len_694_239305.p1_type_5prime_partial_len_197 | XP_021947891.12-oxo-4-hydroxy-4-carboxy-5-ureidoimidazoline decarboxylase-like | P:GO:0019428 | |
| TRINITY_DN26325_c0_g1_i1_len_1499_193111.p1_type_complete_len_197 | ---NA--- | | |
| TRINITY_DN12221_c0_g1_i1_len_802_162398.p1_type_complete_len_197 | XP_018015360.1 protein RER1-like isoform X2 | | |
| TRINITY_DN33997_c0_g1_i1_len_641_200964.p1_type_5prime_partial_len_197 | ---NA--- | | |
| TRINITY_DN15267_c0_g1_i1_len_700_199985.p1_type_5prime_partial_len_197 | XP_018025443.1PREDICTED: uncharacterized protein LOC108681017 | | |
| TRINITY_DN1189_c0_g1_i1_len_592_67237.p1_type_internal_len_198 | PNF22200.1SPRY domain-containing protein 3 | | |
| TRINITY_DN22631_c0_g1_i1_len_592_67220.p1_type_internal_len_198 | XP_018012650.1 fibrillin-2-like | | |
| TRINITY_DN16109_c0_g1_i1_len_674_239588.p1_type_5prime_partial_len_197 | EDS32952.139S ribosomal protein L17, mitochondrial | F:GO:0003735; C:GO:0005840; P:GO:0006412 | |
| TRINITY_DN523_c0_g1_i1_len_904_161459.p1_type_complete_len_196 | XP_018011734.1 protein scylla-like | | |
| TRINITY_DN18777_c0_g1_i1_len_4456_264439.p2_type_complete_len_196 | XP_008469384.1alpha-actinin, sarcomeric-like | F:GO:0003779 | |
| TRINITY_DN21608_c0_g1_i1_len_1158_234983.p1_type_complete_len_196 | OXA64732.1FMN-dependent NADPH-azoreductase | C:GO:0005829; F:GO:0010181; F:GO:0052873 | EC:1.5.1.38; EC:1.5.1.39 |
| TRINITY_DN20068_c0_g1_i1_len_1045_235765.p1_type_3prime_partial_len_197 | ---NA--- | | |

| | | | |
|---|---|---|---|
| TRINITY_DN9046_c0_g1_i1_len_644_200911.p1_type_5prime_partial_len_196 | ---NA--- | | |
| TRINITY_DN17673_c0_g1_i1_len_1015_235987.p1_type_5prime_partial_len_196 | XP_007885978.1 dnaJ homolog subfamily C member 24 isoform X1 | | |
| TRINITY_DN246_c0_g1_i1_len_589_7166.p1_type_internal_len_197 | PNF17330.1Ubiquitin carboxyl-terminal hydrolase 7 | P:GO:0006508; F:GO:0016787; P:GO:0044267 | |
| TRINITY_DN20063_c0_g1_i2_len_1434_1858.p2_type_5prime_partial_len_196 | XP_018899852.1 protein cereblon homolog isoform X2 | | |
| TRINITY_DN10479_c0_g1_i1_len_675_239559.p1_type_complete_len_196 | XP_018017769.1 anaphase-promoting complex subunit 10-like | | |
| TRINITY_DN16992_c0_g1_i1_len_1171_159501.p1_type_complete_len_196 | XP_018010123.1 1-phosphatidylinositol 4,5-bisphosphate phosphodiesterase eta-2-like | F:GO:0004435; F:GO:0005509; P:GO:0006629; P:GO:0007165; F:GO:0008081; P:GO:0016042; F:GO:0016787; P:GO:0035556 | |
| TRINITY_DN20558_c0_g1_i2_len_1782_92570.p1_type_complete_len_196 | BAE78496.1farnesoic acid O-methyltransferase | F:GO:0008168; F:GO:0016740; P:GO:0032259 | |
| TRINITY_DN21917_c0_g1_i2_len_696_129063.p1_type_5prime_partial_len_196 | KDR20307.1Histidine triad nucleotide-binding protein 3 | F:GO:0003824 | |
| TRINITY_DN960_c0_g1_i1_len_639_240117.p1_type_5prime_partial_len_196 | XP_018016206.1PREDICTED: uncharacterized protein LOC108672944 | | |
| TRINITY_DN22613_c0_g1_i1_len_1618_1419.p2_type_5prime_partial_len_196 | XP_026468871.1sodium- and chloride-dependent glycine transporter 1 | | |
| TRINITY_DN22017_c0_g2_i2_len_699_239213.p1_type_5prime_partial_len_196 | ABR27687.1cuticle proprotein proCP5.2 | F:GO:0042302 | |
| TRINITY_DN12425_c0_g1_i1_len_624_302045.p1_type_3prime_partial_len_197 | XP_018020560.1 vitelline membrane outer layer protein 1 homolog | | |
| TRINITY_DN28653_c0_g1_i1_len_590_240997.p1_type_internal_len_197 | ---NA--- | | |
| TRINITY_DN30849_c0_g1_i1_len_590_240983.p3_type_internal_len_197 | XP_026293568.1unconventional myosin-XV-like isoform X3 | | |
| TRINITY_DN25241_c0_g1_i4_len_3539_228443.p3_type_5prime_partial_len_196 | AAT77811.1calpain B | F:GO:0004198; F:GO:0005509; P:GO:0006508 | EC:3.4.22 |

| | | | |
|---|---|---|---|
| TRINITY_DN39429_c0_g1_i1_len_661_200612.p1_type_5prime_partial_len_196 | XP_022241520.1protein dopey-1-like isoform X3 | | |
| TRINITY_DN41643_c0_g1_i1_len_661_200609.p1_type_5prime_partial_len_196 | XP_023225186.1MKRN2 opposite strand protein-like | | |
| TRINITY_DN35411_c0_g1_i1_len_588_241018.p1_type_internal_len_197 | ---NA--- | | |
| TRINITY_DN21899_c0_g1_i1_len_680_129235.p1_type_5prime_partial_len_196 | XP_022912209.1CDGSH iron-sulfur domain-containing protein 3, mitochondrial-like | C:GO:0043231; F:GO:0051537 | |
| TRINITY_DN22126_c0_g1_i1_len_590_274281.p1_type_internal_len_197 | ---NA--- | | |
| TRINITY_DN21065_c0_g1_i1_len_589_274298.p1_type_3prime_partial_len_197 | XP_018021226.1 organic cation transporter protein-like | | |
| TRINITY_DN8347_c0_g1_i1_len_592_37082.p1_type_internal_len_197 | XP_019635929.1 titin-like isoform X2 | F:GO:0008270; F:GO:0016491; P:GO:0055114 | |
| TRINITY_DN24635_c0_g1_i1_len_591_165155.p1_type_internal_len_197 | ---NA--- | | |
| TRINITY_DN8232_c0_g1_i1_len_590_165172.p1_type_internal_len_197 | ---NA--- | | |
| TRINITY_DN10096_c0_g1_i2_len_590_201987.p1_type_internal_len_197 | ---NA--- | | |
| TRINITY_DN38786_c0_g1_i1_len_590_201981.p1_type_3prime_partial_len_197 | PNF17269.1Small G protein signaling modulator 3-like protein | | |
| TRINITY_DN2517_c0_g1_i1_len_590_201973.p1_type_internal_len_197 | XP_023712632.1calcium-dependent secretion activator isoform X8 | P:GO:0016079; C:GO:0098793; P:GO:1990504 | |
| TRINITY_DN19936_c0_g1_i1_len_591_201971.p1_type_3prime_partial_len_197 | XP_006111734.1kynurenine/alpha-aminoadipate aminotransferase, mitochondrial-like | F:GO:0008483; P:GO:1901605 | |
| TRINITY_DN23198_c0_g1_i1_len_591_201963.p1_type_internal_len_197 | ---NA--- | | |
| TRINITY_DN9845_c0_g1_i1_len_591_201958.p1_type_internal_len_197 | XP_018027255.1PREDICTED: uncharacterized protein LOC108682574 | | |
| TRINITY_DN23630_c0_g1_i1_len_592_201939.p1_type_internal_len_197 | ---NA--- | | |

| | | | |
|---|---|---|---|
| TRINITY_DN13382_c0_g1_i1_len_851_96639.p1_type_5prime_partial_len_196 | ---NA--- | | |
| TRINITY_DN19468_c0_g1_i6_len_1039_95355.p1_type_complete_len_196 | ---NA--- | | |
| TRINITY_DN16122_c0_g1_i1_len_588_202029.p1_type_internal_len_197 | PSN47595.1Exonuclease 3'-5' domain-containing protein 2 | F:GO:0003676; F:GO:0004527; C:GO:0005737; P:GO:0006139; F:GO:0008408; P:GO:0090305 | |
| TRINITY_DN10411_c0_g1_i1_len_588_202021.p1_type_internal_len_197 | KXJ11352.1Retrovirus-related Pol polyprotein from transposon 17.6 | F:GO:0003700; F:GO:0004190; P:GO:0006355; P:GO:0006508 | |
| TRINITY_DN37304_c0_g1_i1_len_589_202014.p1_type_internal_len_197 | ---NA--- | | |
| TRINITY_DN19197_c0_g1_i1_len_590_201997.p1_type_internal_len_197 | KMQ93742.1otoferlin-like protein | F:GO:0005509; C:GO:0016021; P:GO:0016079; C:GO:0098793 | |
| TRINITY_DN38904_c0_g1_i1_len_590_201996.p1_type_internal_len_197 | XP_018027971.1 dnaJ homolog subfamily C member 13-like | | |
| TRINITY_DN30859_c1_g3_i1_len_590_201994.p1_type_internal_len_197 | XP_018014073.1 abnormal spindle-like microcephaly-associated protein homolog | | |
| TRINITY_DN29466_c0_g1_i1_len_591_67250.p1_type_internal_len_197 | ---NA--- | | |
| TRINITY_DN33837_c0_g1_i1_len_589_67282.p1_type_internal_len_197 | ---NA--- | | |
| TRINITY_DN17330_c0_g1_i1_len_589_130536.p1_type_internal_len_197 | ABW77320.1clottable protein 2 | F:GO:0005319; C:GO:0005576; P:GO:0006869; P:GO:0042381 | |
| TRINITY_DN19475_c0_g1_i1_len_766_199040.p1_type_complete_len_196 | ---NA--- | | |
| TRINITY_DN30803_c0_g1_i3_len_1632_192396.p2_type_3prime_partial_len_197 | PNX92003.1retrotransposon-related protein | F:GO:0003676; P:GO:0015074 | |
| TRINITY_DN4692_c0_g1_i1_len_592_99553.p1_type_internal_len_197 | XP_018007971.1 long chain acyl-CoA synthetase 9, chloroplastic-like | | |
| TRINITY_DN12215_c0_g1_i1_len_588_130567.p1_type_internal_len_197 | XP_018017827.1PREDICTED: uncharacterized protein LOC108674399 isoform X2 | | |

| | | | |
|---|---|---|---|
| TRINITY_DN16293_c0_g1_i2_len_590_302503.p2_type_internal_len_197 | ---NA--- | | |
| TRINITY_DN9037_c0_g1_i2_len_938_197110.p1_type_complete_len_196 | XP_018007528.1PREDICTED: uncharacterized protein LOC108665301 | | |
| TRINITY_DN29737_c0_g1_i1_len_589_165196.p1_type_internal_len_197 | ---NA--- | | |
| TRINITY_DN14034_c0_g1_i1_len_592_130485.p1_type_internal_len_197 | ---NA--- | | |
| TRINITY_DN22421_c0_g2_i2_len_897_236981.p1_type_3prime_partial_len_197 | ---NA--- | | |
| TRINITY_DN31139_c3_g1_i1_len_1888_191319.p2_type_complete_len_195 | ---NA--- | | |
| TRINITY_DN24577_c0_g1_i1_len_3035_228954.p4_type_complete_len_195 | PSN37597.1Myb protein | F:GO:0003677; C:GO:0005634 | |
| TRINITY_DN10976_c0_g1_i1_len_1056_269895.p1_type_complete_len_195 | ---NA--- | | |
| TRINITY_DN9233_c0_g1_i1_len_586_67335.p1_type_internal_len_196 | ---NA--- | | |
| TRINITY_DN19550_c0_g1_i1_len_761_199113.p1_type_5prime_partial_len_195 | ---NA--- | | |
| TRINITY_DN10690_c0_g1_i1_len_590_201983.p1_type_5prime_partial_len_195 | ---NA--- | | |
| TRINITY_DN20216_c0_g1_i1_len_589_201998.p1_type_internal_len_196 | XP_018025573.1 tropomyosin isoform X9 | | |
| TRINITY_DN27887_c1_g1_i1_len_589_202020.p1_type_internal_len_196 | XP_018022161.1 histone-lysine N-methyltransferase, H3 lysine-79 specific-like isoform X2 | | |
| TRINITY_DN19137_c0_g1_i4_len_1578_267461.p1_type_5prime_partial_len_195 | XP_023717621.1von Hippel-Lindau disease tumor suppressor | | |
| TRINITY_DN19211_c0_g1_i1_len_588_202030.p1_type_internal_len_196 | ---NA--- | | |
| TRINITY_DN10704_c0_g1_i1_len_588_202037.p1_type_internal_len_196 | XP_018009149.1PREDICTED: uncharacterized protein LOC108666741 | | |

| | | | |
|---|---|---|---|
| TRINITY_DN16754_c1_g1_i1_len_588_202038.p1_type_internal_len_196 | XP_018019672.1 toll-like receptor Tollo | P:GO:0007165; C:GO:0016021 | |
| TRINITY_DN10408_c0_g1_i1_len_587_202041.p1_type_internal_len_196 | ---NA--- | | |
| TRINITY_DN2295_c0_g1_i1_len_587_202042.p1_type_internal_len_196 | XP_018024855.1 serine/threonine-protein kinase PKH3-like | F:GO:0004672; F:GO:0005524; P:GO:0006468 | |
| TRINITY_DN24053_c0_g1_i1_len_587_202043.p1_type_internal_len_196 | ---NA--- | | |
| 1471\|_\|c1995_f2p25_2918_isoform_c1995_full_length_coverage_2_non_full_length_coverage_25_isoform_length_2918_26131 | XP_018015790.1 heterogeneous nuclear ribonucleoprotein U-like protein 1 | | |
| 1472\|_\|c22925_f3p2_2918_isoform_c22925_full_length_coverage_3_non_full_length_coverage_2_isoform_length_2918_22893 | XP_018006990.1 epidermal retinol dehydrogenase 2-like | | |
| 1473\|_\|c34426_f3p4_2917_isoform_c34426_full_length_coverage_3_non_full_length_coverage_4_isoform_length_2917_18751 | XP_018023506.1 DNA replication licensing factor MCM3-like | F:GO:0003677; F:GO:0004386; F:GO:0005524; C:GO:0005634; P:GO:0006270; C:GO:0042555 | EC:3.6.1.15 |
| 1474\|_\|c9532_f3p5_2917_isoform_c9532_full_length_coverage_3_non_full_length_coverage_5_isoform_length_2917_12815 | XP_018015966.1 eukaryotic translation initiation factor 5B-like isoform X1 | | |
| TRINITY_DN22743_c0_g1_i1_len_4405_227982.p1_type_complete_len_972 | XP_013787373.2tripartite motif-containing protein 2-like | | |
| 1476\|_\|c1919_f2p2_2916_isoform_c1919_full_length_coverage_2_non_full_length_coverage_2_isoform_length_2916_28075 | XP_013413006.1protein NETWORKED 2D | | |
| TRINITY_DN18730_c0_g1_i1_len_3161_29422.p1_type_5prime_partial_len_972 | PNF16671.1putative vacuolar protein sorting-associated protein 18 | C:GO:0005623; P:GO:0006886; P:GO:0016192 | |
| TRINITY_DN22350_c1_g1_i1_len_2918_188665.p1_type_internal_len_973 | ATU31752.1basement membrane-specific heparan sulfate proteoglycan core protein | F:GO:0005509 | |
| 1479\|_\|c47022_f2p4_2915_isoform_c47022_full_length_coverage_2_non_full_length_coverage_4_isoform_length_2915_19393 | XP_018006990.1 epidermal retinol dehydrogenase 2-like | | |

| | | | |
|---|---|---|---|
| 1480\|_\|c4780_f2p1_2914_isoform_c4780_full_length_coverage_2_non_full_length_coverage_1_isoform_length_2914_16890 | XP_018007849.1 CD82 antigen-like | C:GO:0016020; C:GO:0016021 | |
| TRINITY_DN35320_c0_g1_i1_len_587_202044.p1_type_internal_len_196 | ---NA--- | | |
| TRINITY_DN13630_c0_g1_i1_len_587_202046.p1_type_internal_len_196 | ---NA--- | | |
| TRINITY_DN17449_c0_g1_i1_len_720_199711.p1_type_3prime_partial_len_196 | XP_019615458.1 ras-related protein Rab-24-like isoform X1 | F:GO:0003924; F:GO:0005525 | |
| TRINITY_DN19514_c0_g1_i1_len_845_237492.p1_type_3prime_partial_len_196 | XP_018015532.1 ras-specific guanine nucleotide-releasing factor RalGPS1-like | F:GO:0005085; P:GO:0007264 | |
| TRINITY_DN19583_c0_g1_i1_len_1117_159859.p1_type_3prime_partial_len_196 | XP_014781812.1 zinc finger protein 239-like | F:GO:0003677 | |
| TRINITY_DN5454_c0_g1_i1_len_587_241032.p1_type_internal_len_196 | ---NA--- | | |
| TRINITY_DN17939_c0_g1_i1_len_879_161687.p1_type_5prime_partial_len_195 | XP_019965835.1 DNA polymerase epsilon subunit 3 | C:GO:0005634; F:GO:0046982 | |
| TRINITY_DN23177_c0_g1_i1_len_744_199368.p1_type_3prime_partial_len_196 | XP_018016654.1 facilitated trehalose transporter Tret1-like | | |
| TRINITY_DN10685_c0_g1_i1_len_587_241036.p1_type_internal_len_196 | ---NA--- | | |
| TRINITY_DN11176_c0_g1_i1_len_587_241039.p1_type_internal_len_196 | GBC13192.1gag-pol fusion protein | | |
| TRINITY_DN34821_c0_g1_i1_len_586_241065.p1_type_internal_len_196 | EEZ97753.1synaptotagmin IV | C:GO:0016020 | |
| TRINITY_DN8983_c0_g1_i1_len_585_241084.p1_type_internal_len_196 | ---NA--- | | |
| TRINITY_DN12959_c0_g1_i1_len_585_241090.p1_type_internal_len_196 | XP_021363719.1zinc finger protein 239-like | | |
| TRINITY_DN17860_c0_g1_i1_len_1036_235817.p2_type_complete_len_195 | ---NA--- | | |
| TRINITY_DN23124_c0_g1_i3_len_1001_196541.p1_type_5prime_partial_len_195 | XP_019965835.1 DNA polymerase epsilon subunit 3 | C:GO:0005634; F:GO:0046982 | |

| | | | |
|---|---|---|---|
| TRINITY_DN13503_c0_g1_i2_len_586_165252.p1_type_internal_len_196 | XP_015509869.1PREDICTED: uncharacterized protein LOC107217026 | | |
| TRINITY_DN8032_c0_g1_i1_len_643_129708.p1_type_5prime_partial_len_195 | AIU40991.1insulin-like peptide 1 | | |
| TRINITY_DN16780_c0_g1_i1_len_587_202054.p1_type_internal_len_196 | ---NA--- | | |
| TRINITY_DN20061_c0_g1_i1_len_586_202058.p1_type_internal_len_196 | ---NA--- | | |
| TRINITY_DN2541_c0_g1_i1_len_586_202066.p1_type_internal_len_196 | ---NA--- | | |
| TRINITY_DN32160_c0_g1_i1_len_586_202074.p1_type_internal_len_196 | ---NA--- | | |
| TRINITY_DN15966_c0_g1_i1_len_585_202089.p1_type_internal_len_196 | AJF94637.1cytochrome P450 epoxidase-like protein | F:GO:0004497; F:GO:0005506; F:GO:0016705; F:GO:0020037; P:GO:0055114 | |
| TRINITY_DN8976_c0_g1_i1_len_585_202093.p1_type_internal_len_196 | ---NA--- | | |
| TRINITY_DN11188_c0_g1_i1_len_585_202097.p1_type_internal_len_196 | ---NA--- | | |
| TRINITY_DN41263_c0_g1_i1_len_840_198127.p1_type_5prime_partial_len_195 | ---NA--- | | |
| TRINITY_DN4601_c0_g1_i1_len_614_164766.p1_type_5prime_partial_len_195 | XP_018023008.1 tripeptidyl-peptidase 2-like isoform X2 | F:GO:0004252; P:GO:0006508; F:GO:0008240 | EC:3.4.21; EC:3.4.14 |
| TRINITY_DN16019_c0_g1_i1_len_698_301139.p1_type_5prime_partial_len_195 | ---NA--- | | |
| TRINITY_DN19364_c0_g1_i3_len_653_36255.p1_type_3prime_partial_len_196 | ---NA--- | | |
| TRINITY_DN21869_c0_g1_i2_len_584_302599.p2_type_internal_len_195 | ---NA--- | | |
| TRINITY_DN14924_c0_g1_i1_len_582_130674.p1_type_internal_len_195 | XP_023321830.1acyl-CoA synthetase family member 2, mitochondrial-like isoform X2 | | |

| | | | |
|---|---|---|---|
| TRINITY_DN20386_c0_g1_i1_len_583_130640.p1_type_internal_len_195 | XP_018018358.1PREDICTED: uncharacterized protein LOC108674884 | | |
| TRINITY_DN155_c0_g1_i1_len_583_241114.p1_type_internal_len_195 | ---NA--- | | |
| TRINITY_DN27074_c0_g1_i1_len_634_129836.p1_type_5prime_partial_len_194 | ---NA--- | | |
| TRINITY_DN18478_c0_g1_i1_len_584_241105.p1_type_internal_len_195 | KZS17443.1Solute carrier family 25 member 44 | C:GO:0016021; F:GO:0022857; P:GO:0055085 | |
| TRINITY_DN3836_c0_g1_i1_len_584_241103.p1_type_internal_len_195 | ---NA--- | | |
| TRINITY_DN6227_c0_g1_i1_len_585_241082.p1_type_internal_len_195 | XP_018026741.1 zinc finger protein 391-like | F:GO:0003676 | |
| TRINITY_DN19854_c0_g1_i1_len_1464_31564.p1_type_complete_len_194 | ---NA--- | | |
| TRINITY_DN8326_c0_g1_i1_len_586_241059.p1_type_internal_len_195 | AXF35707.1CWO | F:GO:0003677; C:GO:0005634; P:GO:0006355; F:GO:0046983 | |
| TRINITY_DN18184_c2_g1_i2_len_623_240408.p1_type_5prime_partial_len_194 | ---NA--- | | |
| TRINITY_DN10224_c0_g1_i1_len_586_37163.p1_type_internal_len_195 | ---NA--- | | |
| TRINITY_DN27752_c0_g1_i1_len_1170_195172.p1_type_complete_len_194 | ---NA--- | | |
| TRINITY_DN24062_c0_g1_i1_len_1174_195146.p1_type_5prime_partial_len_194 | XP_018018110.1 centrosomal protein of 19 kDa-like | | |
| TRINITY_DN14227_c0_g1_i1_len_1182_159438.p1_type_complete_len_194 | ---NA--- | | |
| TRINITY_DN14517_c0_g1_i1_len_674_6013.p1_type_5prime_partial_len_194 | XP_023719892.1succinate dehydrogenase assembly factor 2, mitochondrial-like | C:GO:0005739; P:GO:0044237 | |
| TRINITY_DN11596_c0_g1_i1_len_582_241134.p1_type_internal_len_195 | ---NA--- | | |
| TRINITY_DN37368_c0_g1_i1_len_813_198453.p1_type_5prime_partial_len_194 | ---NA--- | | |

| | | | |
|---|---|---|---|
| TRINITY_DN16833_c0_g1_i3_len_969_33660.p1_type_5prime_partial_len_194 | ---NA--- | | |
| TRINITY_DN27623_c0_g1_i6_len_1621_192452.p2_type_5prime_partial_len_194 | KFM56750.1Pre-mRNA-splicing factor 38B | | |
| TRINITY_DN7640_c0_g1_i1_len_583_202137.p1_type_internal_len_195 | ---NA--- | | |
| TRINITY_DN643_c0_g1_i1_len_583_202140.p1_type_internal_len_195 | XP_023246842.1dedicator of cytokinesis protein 3 | | |
| TRINITY_DN22153_c0_g1_i2_len_991_196636.p1_type_5prime_partial_len_194 | XP_017323656.1 riboflavin kinase | F:GO:0016301; P:GO:0044237 | |
| TRINITY_DN25421_c0_g1_i1_len_1956_191060.p2_type_complete_len_194 | ---NA--- | | |
| TRINITY_DN22139_c0_g1_i1_len_677_200362.p1_type_complete_len_194 | ANJ03341.1vascular endothelial growth factor 3 | | |
| TRINITY_DN20395_c0_g1_i1_len_640_66484.p1_type_5prime_partial_len_194 | ---NA--- | | |
| TRINITY_DN35168_c0_g1_i1_len_586_202060.p1_type_internal_len_195 | XP_018024595.1 polycomb protein Sfmbt-like isoform X1 | C:GO:0005634; P:GO:0006355; C:GO:0016021 | |
| TRINITY_DN20589_c0_g1_i1_len_585_202086.p1_type_internal_len_195 | XP_018017531.1PREDICTED: uncharacterized protein LOC108674134 | | |
| TRINITY_DN35364_c0_g1_i1_len_585_202091.p1_type_internal_len_195 | ---NA--- | | |
| TRINITY_DN12995_c0_g1_i1_len_585_202102.p1_type_internal_len_195 | ---NA--- | | |
| TRINITY_DN31930_c0_g1_i1_len_878_197721.p1_type_3prime_partial_len_195 | XP_018025288.1 DNA damage-regulated autophagy modulator protein 2-like | C:GO:0016020 | |
| TRINITY_DN28431_c0_g3_i1_len_585_202105.p1_type_internal_len_195 | ---NA--- | | |
| TRINITY_DN12508_c0_g1_i2_len_584_202108.p1_type_internal_len_195 | ---NA--- | | |
| TRINITY_DN33577_c0_g1_i1_len_584_202111.p1_type_internal_len_195 | ---NA--- | | |

| | | | |
|---|---|---|---|
| TRINITY_DN33215_c0_g1_i1_len_584_202116.p1_type_internal_len_195 | ---NA--- | | |
| TRINITY_DN10248_c0_g1_i1_len_583_202134.p1_type_internal_len_195 | ESO89220.1hypothetical protein LOTGIDRAFT_58934, partial | | |
| TRINITY_DN19757_c0_g1_i1_len_688_301258.p1_type_5prime_partial_len_194 | PNF21573.1NudC domain-containing protein 2 | | |
| TRINITY_DN25990_c1_g1_i1_len_698_200034.p1_type_5prime_partial_len_194 | XP_022200155.1gastrula zinc finger protein XlCGF57.1-like | | |
| TRINITY_DN21271_c0_g1_i1_len_1279_194400.p1_type_5prime_partial_len_194 | ---NA--- | | |
| TRINITY_DN11551_c0_g1_i1_len_584_165297.p1_type_internal_len_195 | XP_015600282.1uncharacterized protein LOC107270104 | | |
| TRINITY_DN17068_c0_g1_i1_len_914_270821.p2_type_complete_len_194 | AYN64309.1vascular endothelial growth factor 4 | | |
| TRINITY_DN13188_c0_g1_i1_len_1508_193055.p2_type_5prime_partial_len_194 | ---NA--- | | |
| TRINITY_DN31869_c0_g1_i1_len_644_164306.p1_type_5prime_partial_len_194 | XP_012215449.1 BTB/POZ domain-containing protein 6-B-like | | |
| TRINITY_DN26006_c0_g1_i2_len_730_199583.p1_type_3prime_partial_len_195 | XP_022097496.1piggyBac transposable element-derived protein 4-like | | |
| TRINITY_DN18216_c0_g1_i1_len_1096_269656.p2_type_5prime_partial_len_194 | AYH52219.1putative tyrosylprotein sulfotransferase | P:GO:0006478; F:GO:0008476 | EC:2.8.2.20 |
| TRINITY_DN11819_c0_g1_i1_len_781_238176.p1_type_3prime_partial_len_195 | ---NA--- | | |
| TRINITY_DN30805_c0_g1_i10_len_2516_189413.p2_type_5prime_partial_len_194 | ---NA--- | | |
| TRINITY_DN32416_c0_g1_i1_len_581_202189.p1_type_internal_len_194 | ---NA--- | | |
| TRINITY_DN1161_c0_g1_i1_len_581_202180.p1_type_internal_len_194 | XP_018017475.1PREDICTED: uncharacterized protein LOC108674083 isoform X2 | F:GO:0008236 | |
| TRINITY_DN13538_c0_g1_i1_len_703_301085.p1_type_complete_len_193 | ---NA--- | | |

| | | | |
|---|---|---|---|
| TRINITY_DN31794_c0_g1_i1_len_583_202143.p1_type_internal_len_194 | ---NA--- | | |
| TRINITY_DN1000_c0_g1_i1_len_580_202220.p2_type_internal_len_194 | ---NA--- | | |
| TRINITY_DN19479_c0_g1_i4_len_580_202219.p1_type_internal_len_194 | ---NA--- | | |
| TRINITY_DN37085_c0_g1_i1_len_580_202214.p1_type_internal_len_194 | XP_017300339.1cysteine and histidine-rich protein 1 homolog | F:GO:0046872 | |
| TRINITY_DN17212_c0_g1_i1_len_580_202208.p1_type_internal_len_194 | ---NA--- | | |
| TRINITY_DN33477_c0_g1_i1_len_580_202202.p1_type_internal_len_194 | ---NA--- | | |
| TRINITY_DN37520_c0_g1_i1_len_580_202195.p1_type_internal_len_194 | PNF17268.1Small G protein signaling modulator 3-like protein | | |
| TRINITY_DN31583_c0_g1_i1_len_581_202192.p2_type_internal_len_194 | XP_023314615.1probable Ras GTPase-activating protein isoform X8 | | |
| TRINITY_DN22649_c0_g1_i1_len_3326_57995.p2_type_complete_len_193 | ---NA--- | | |
| TRINITY_DN18103_c0_g1_i1_len_1990_231150.p1_type_5prime_partial_len_193 | KDR15023.1Low-density lipoprotein receptor-related protein | C:GO:0016020; C:GO:0016021 | |
| TRINITY_DN18553_c0_g1_i1_len_947_197024.p2_type_5prime_partial_len_193 | ---NA--- | | |
| TRINITY_DN11964_c0_g1_i1_len_586_202062.p1_type_5prime_partial_len_193 | PNF41472.1Serine/threonine/tyrosine-interacting protein | P:GO:0006470; F:GO:0008138 | EC:3.1.3.16 |
| TRINITY_DN28893_c0_g1_i2_len_1917_191197.p2_type_complete_len_193 | ---NA--- | | |
| TRINITY_DN31257_c0_g1_i10_len_3028_188494.p1_type_complete_len_193 | GBC18203.1retrotransposable element | F:GO:0003676; P:GO:0015074 | |
| TRINITY_DN28751_c0_g1_i1_len_3949_187616.p3_type_complete_len_193 | ---NA--- | | |
| TRINITY_DN13881_c0_g1_i1_len_694_35799.p1_type_5prime_partial_len_193 | CDI59403.1Histone 1 | C:GO:0000786; F:GO:0003677; C:GO:0005634; P:GO:0006334 | |

| | | | |
|---|---|---|---|
| TRINITY_DN2841_c0_g1_i1_len_581_99741.p1_type_internal_len_194 | ---NA--- | | |
| TRINITY_DN18952_c0_g1_i1_len_1054_196074.p1_type_complete_len_193 | AEL23143.1tripartite motif-containing 32 | | |
| TRINITY_DN22788_c0_g1_i2_len_1321_124925.p1_type_complete_len_193 | XP_003703439.1 RNA polymerase II subunit A C-terminal domain phosphatase SSU72 | F:GO:0004721; C:GO:0005634; P:GO:0006397; P:GO:0006470 | EC:3.1.3.16 |
| TRINITY_DN18615_c0_g1_i1_len_1276_158905.p2_type_5prime_partial_len_193 | XP_024218177.1multidrug resistance-associated protein 1-like | F:GO:0005524; C:GO:0016021; F:GO:0042626; P:GO:0055085 | EC:3.6.1.3; EC:3.6.1.15 |
| TRINITY_DN32999_c0_g1_i1_len_579_241189.p1_type_internal_len_194 | ---NA--- | | |
| TRINITY_DN5149_c0_g1_i1_len_579_241180.p1_type_internal_len_194 | ---NA--- | | |
| TRINITY_DN26117_c0_g1_i3_len_4708_227869.p1_type_complete_len_971 | ASW35093.1sarcolemmal membrane-associated protein-like isoform X2 | P:GO:0006936; C:GO:0016020 | |
| 1482\|_\|c1691_f2p8_2911_isoform_c1691_full_length_coverage_2_non_full_length_coverage_8_isoform_length_2911_27904 | PNF29480.1Exocyst complex component 5 | C:GO:0005737; P:GO:0009987; P:GO:0016192 | |
| 1483\|_\|c2431_f2p6_2911_isoform_c2431_full_length_coverage_2_non_full_length_coverage_6_isoform_length_2911_16405 | ACA62227.1Mi-2, partial | | |
| TRINITY_DN30004_c0_g1_i1_len_3962_187605.p1_type_5prime_partial_len_970 | XP_013415223.1receptor-type tyrosine-protein phosphatase kappa-like | F:GO:0004721; F:GO:0004725; P:GO:0006470; P:GO:0016311; F:GO:0016787; F:GO:0016791; P:GO:0035335 | |
| TRINITY_DN31005_c0_g1_i2_len_4299_187420.p1_type_5prime_partial_len_970 | PNF25133.1Protein brunelleschi | | |
| TRINITY_DN22108_c0_g1_i2_len_2993_91156.p1_type_3prime_partial_len_971 | XP_023020073.1protein KRI1 homolog | | |
| 1488\|_\|c2029_f2p14_2910_isoform_c2029_full_length_coverage_2_non_full_length_coverage_14_isoform_length_2910_28164 | ---NA--- | | |

| | | | |
|---|---|---|---|
| TRINITY_DN30696_c0_g1_i1_len_2912_188675.p1_type_internal_len_971 | XP_023727333.1thyroid receptor-interacting protein 11-like isoform X1 | | |
| 1491\|_\|c10879_f2p3_2908_isoform_c10879_full_length_coverage_2_non_full_length_coverage_3_isoform_length_2908_28562 | AXR98498.1exocyst complex component 6B-like protein | C:GO:0000145; P:GO:0006904 | |
| 1492\|_\|c5199_f2p3_2908_isoform_c5199_full_length_coverage_2_non_full_length_coverage_3_isoform_length_2908_25752 | APA05127.1polephole-like protein | | |
| TRINITY_DN10590_c0_g1_i2_len_786_198784.p1_type_3prime_partial_len_194 | XP_018016206.1PREDICTED: uncharacterized protein LOC108672944 | | |
| TRINITY_DN13506_c0_g1_i1_len_581_302643.p1_type_internal_len_194 | ---NA--- | | |
| TRINITY_DN28101_c0_g1_i1_len_955_3859.p2_type_5prime_partial_len_193 | ---NA--- | | |
| TRINITY_DN30721_c0_g1_i1_len_636_240175.p1_type_3prime_partial_len_194 | XP_018018028.1  CTP synthase-like | | |
| TRINITY_DN1785_c0_g1_i1_len_581_241143.p1_type_internal_len_194 | XP_018020023.1 spectrin beta chain, non-erythrocytic 1-like | | |
| TRINITY_DN32668_c0_g1_i1_len_582_241138.p1_type_internal_len_194 | XP_021925320.1uncharacterized protein LOC110832537 isoform X4 | | |
| TRINITY_DN12425_c0_g1_i1_len_582_241128.p1_type_internal_len_194 | ---NA--- | | |
| TRINITY_DN21265_c0_g1_i3_len_885_237091.p1_type_5prime_partial_len_193 | EEB15276.1mitochondrial glutamate carrier, putative | | |
| TRINITY_DN11331_c0_g1_i1_len_583_241111.p1_type_internal_len_194 | XP_017772188.1 transmembrane protein 62-like isoform X2 | | |
| TRINITY_DN18191_c0_g1_i1_len_853_237394.p1_type_3prime_partial_len_194 | ---NA--- | | |
| TRINITY_DN20674_c1_g1_i1_len_687_163722.p1_type_complete_len_193 | ---NA--- | | |
| TRINITY_DN8962_c0_g1_i1_len_580_67410.p1_type_internal_len_194 | XP_013777626.1PH-interacting protein-like | | |

| | | | |
|---|---|---|---|
| TRINITY_DN39754_c0_g1_i1_len_645_200901.p1_type_3prime_partial_len_194 | ---NA--- | | |
| TRINITY_DN27455_c0_g1_i1_len_2654_189127.p2_type_complete_len_193 | ---NA--- | | |
| TRINITY_DN23770_c0_g1_i1_len_1128_195468.p1_type_5prime_partial_len_193 | XP_018017911.1 dynactin subunit 6-like | C:GO:0005869 | |
| TRINITY_DN14697_c0_g1_i1_len_664_66138.p1_type_5prime_partial_len_193 | ---NA--- | | |
| TRINITY_DN16061_c0_g1_i1_len_849_198042.p1_type_3prime_partial_len_194 | XP_022189367.1MPN domain-containing protein CG4751 | | |
| TRINITY_DN5581_c0_g1_i1_len_580_274457.p1_type_internal_len_194 | ---NA--- | | |
| TRINITY_DN20534_c0_g1_i1_len_855_127579.p1_type_5prime_partial_len_193 | ---NA--- | | |
| TRINITY_DN10862_c0_g1_i1_len_618_130086.p1_type_5prime_partial_len_193 | XP_018026771.1 drebrin-like protein A isoform X2 | | |
| TRINITY_DN12258_c0_g1_i1_len_771_97325.p1_type_5prime_partial_len_193 | ---NA--- | | |
| TRINITY_DN20201_c0_g1_i2_len_823_64381.p3_type_5prime_partial_len_192 | ---NA--- | | |
| TRINITY_DN39540_c0_g1_i1_len_580_202203.p1_type_internal_len_193 | ---NA--- | | |
| TRINITY_DN2594_c0_g1_i1_len_578_302705.p1_type_internal_len_193 | PIK47160.1hypothetical protein BSL78_15968 | F:GO:0003676; F:GO:0003700; P:GO:0006355; F:GO:0008270 | |
| TRINITY_DN312_c0_g1_i1_len_577_241216.p1_type_internal_len_193 | ---NA--- | | |
| TRINITY_DN20996_c0_g1_i1_len_578_202244.p1_type_internal_len_193 | XP_018019976.1 kynureninase-like | P:GO:0006568; P:GO:0009435; F:GO:0016787; P:GO:0019439; P:GO:0042537; P:GO:1901361; P:GO:1901606 | |

| | | | |
|---|---|---|---|
| TRINITY_DN8578_c0_g1_i1_len_580_241167.p1_type_internal_len_193 | KXZ75916.11-phosphatidylinositol 4,5-bisphosphate phosphodiesterase gamma-1-like Protein | F:GO:0004435; F:GO:0005515; C:GO:0005886; P:GO:0009395; P:GO:0010634; P:GO:0032959; P:GO:0048015; P:GO:0051209 | EC:3.1.4.3; EC:3.1.4.11 |
| TRINITY_DN494_c0_g1_i1_len_580_241158.p1_type_internal_len_193 | ---NA--- | | |
| TRINITY_DN16038_c1_g1_i1_len_578_202251.p1_type_internal_len_193 | XP_018021508.1 sialic acid synthase-like | F:GO:0003824; P:GO:0016051 | |
| TRINITY_DN20997_c0_g1_i1_len_4597_227911.p1_type_complete_len_192 | XP_018018887.1 neurocalcin homolog isoform X3 | F:GO:0005509 | |
| TRINITY_DN13784_c0_g1_i1_len_634_273653.p1_type_5prime_partial_len_192 | XP_018022319.1 myotubularin-related protein 6-like isoform X2 | F:GO:0004438; F:GO:0004725; P:GO:0010506; P:GO:0035335; P:GO:0046856; F:GO:0046872; F:GO:0052629 | EC:3.1.3.16; EC:3.1.3.48; EC:3.1.3.64 |
| TRINITY_DN9457_c0_g1_i1_len_1348_158494.p1_type_complete_len_192 | ---NA--- | | |
| TRINITY_DN32651_c0_g1_i1_len_579_202236.p1_type_internal_len_193 | KDR15614.1Membrane-associated guanylate kinase, WW and PDZ domain-containing protein 2 | F:GO:0016301; P:GO:0016310 | |
| TRINITY_DN22540_c0_g1_i1_len_2158_155687.p1_type_5prime_partial_len_192 | XP_023725807.1transmembrane protein 18 isoform X1 | C:GO:0016020 | |
| TRINITY_DN19700_c0_g1_i1_len_659_301600.p1_type_5prime_partial_len_192 | XP_021954899.1trans-1,2-dihydrobenzene-1,2-diol dehydrogenase-like | F:GO:0016491; P:GO:0055114 | |
| TRINITY_DN1102_c0_g1_i1_len_595_201864.p1_type_5prime_partial_len_192 | ---NA--- | | |
| TRINITY_DN13165_c0_g1_i1_len_578_67453.p1_type_internal_len_193 | ---NA--- | | |
| TRINITY_DN25101_c0_g1_i2_len_577_67459.p1_type_internal_len_193 | ---NA--- | | |
| TRINITY_DN26405_c0_g1_i1_len_579_67422.p1_type_internal_len_193 | ---NA--- | | |
| TRINITY_DN8164_c0_g1_i1_len_579_67425.p1_type_internal_len_193 | ---NA--- | | |

| | | | |
|---|---|---|---|
| TRINITY_DN21969_c0_g2_i3_len_591_67252.p1_type_5prime_partial_len_192 | AKS26011.1crustacyanin A | F:GO:0031409 | |
| TRINITY_DN1750_c0_g1_i2_len_690_272933.p1_type_complete_len_192 | XP_022256987.1methylated-DNA--protein-cysteine methyltransferase-like | | |
| TRINITY_DN16739_c0_g2_i1_len_648_36317.p1_type_3prime_partial_len_193 | ---NA--- | | |
| TRINITY_DN1842_c0_g1_i1_len_578_130734.p1_type_internal_len_193 | XP_014672487.1 hexosaminidase D-like isoform X3 | | |
| TRINITY_DN15530_c0_g1_i1_len_578_130719.p1_type_internal_len_193 | XP_018012803.1 UNC93-like protein MFSD11 isoform X1 | | |
| TRINITY_DN14980_c1_g1_i1_len_692_200122.p1_type_3prime_partial_len_193 | ---NA--- | | |
| TRINITY_DN26026_c0_g1_i1_len_674_65997.p1_type_5prime_partial_len_192 | XP_018014360.1 MFS-type transporter SLC18B1-like | | |
| TRINITY_DN22082_c0_g2_i12_len_1541_93218.p2_type_5prime_partial_len_192 | ---NA--- | | |
| TRINITY_DN24233_c0_g1_i9_len_3132_58085.p1_type_complete_len_192 | ---NA--- | | |
| TRINITY_DN15004_c0_g1_i9_len_1634_267294.p1_type_complete_len_192 | ---NA--- | | |
| TRINITY_DN25490_c0_g2_i1_len_3001_228987.p2_type_complete_len_192 | ---NA--- | | |
| TRINITY_DN22406_c0_g1_i1_len_1313_233968.p1_type_5prime_partial_len_192 | XP_018025719.1 V-type proton ATPase subunit e-like | P:GO:0006811; C:GO:0044425; P:GO:0055085 | |
| TRINITY_DN23891_c0_g1_i1_len_1313_233966.p2_type_complete_len_192 | ---NA--- | | |
| TRINITY_DN17642_c0_g1_i1_len_642_200946.p1_type_5prime_partial_len_192 | ---NA--- | | |
| TRINITY_DN19744_c0_g1_i5_len_578_99795.p1_type_3prime_partial_len_193 | ---NA--- | | |
| TRINITY_DN16546_c0_g1_i2_len_578_99784.p1_type_internal_len_193 | ---NA--- | | |

| | | | |
|---|---|---|---|
| TRINITY_DN32505_c0_g1_i1_len_579_99769.p1_type_internal_len_193 | ---NA--- | | |
| TRINITY_DN11007_c0_g1_i1_len_576_274535.p1_type_5prime_partial_len_192 | APF30038.1ionotropic glutamate receptor NMDA 2A | F:GO:0004970; C:GO:0016021; C:GO:0030054; P:GO:0034220; P:GO:0035235; C:GO:0045211 | |
| TRINITY_DN15049_c0_g1_i1_len_666_163973.p1_type_5prime_partial_len_192 | ---NA--- | | |
| TRINITY_DN34279_c0_g1_i1_len_663_164014.p2_type_5prime_partial_len_192 | CAA47046.1preprochymotrypsin 1 | F:GO:0004252; C:GO:0005615; P:GO:0006508; P:GO:0030574 | EC:3.4.21 |
| TRINITY_DN4227_c0_g1_i2_len_867_237261.p1_type_complete_len_192 | ODN03122.1ADP-ribosylation factor-like protein 6 | F:GO:0005525 | |
| TRINITY_DN14893_c0_g1_i1_len_577_274523.p1_type_internal_len_193 | ABW82154.1crustin-like protein | C:GO:0005576; P:GO:0010466; F:GO:0030414 | |
| TRINITY_DN12945_c0_g1_i1_len_878_237152.p1_type_5prime_partial_len_192 | BAI70368.1suppressor of cytokine signaling-2 like protein | P:GO:0050794 | |
| TRINITY_DN9123_c0_g1_i1_len_674_239586.p1_type_5prime_partial_len_192 | ---NA--- | | |
| TRINITY_DN8075_c0_g1_i1_len_577_165408.p1_type_internal_len_193 | ---NA--- | | |
| TRINITY_DN12913_c0_g1_i1_len_778_198885.p1_type_5prime_partial_len_192 | ---NA--- | | |
| TRINITY_DN11656_c0_g1_i2_len_905_236912.p1_type_complete_len_192 | KYN39175.1Ras GTPase-activating protein 3 | P:GO:0065007 | |
| TRINITY_DN31111_c0_g1_i1_len_578_274510.p1_type_internal_len_193 | XP_022095908.1piggyBac transposable element-derived protein 4-like | | |
| TRINITY_DN1269_c0_g1_i1_len_588_165211.p1_type_3prime_partial_len_193 | XP_018007893.1PREDICTED: uncharacterized protein LOC108665627 | | |
| TRINITY_DN16682_c0_g1_i1_len_577_202277.p1_type_internal_len_193 | ---NA--- | | |
| TRINITY_DN41345_c0_g1_i1_len_576_202297.p1_type_internal_len_193 | XP_018026486.1 two pore calcium channel protein 1-like | F:GO:0005245; F:GO:0005509; C:GO:0016021; P:GO:0070588 | |
| TRINITY_DN29152_c0_g2_i1_len_576_202301.p1_type_internal_len_193 | CAA47046.1preprochymotrypsin 1 | F:GO:0004252; C:GO:0005615; P:GO:0006508; P:GO:0030574 | EC:3.4.21 |

| | | | |
|---|---|---|---|
| TRINITY_DN10549_c0_g1_i1_len_576_202292.p1_type_internal_len_193 | PIK54140.1hypothetical protein BSL78_08981 | F:GO:0003676; P:GO:0015074 | |
| TRINITY_DN30046_c0_g1_i2_len_579_7322.p1_type_internal_len_193 | ADO00931.1calnexin | F:GO:0005509; C:GO:0005783; P:GO:0006457; C:GO:0016021; F:GO:0051082 | |
| TRINITY_DN23361_c0_g2_i2_len_3434_153963.p3_type_complete_len_192 | ARS88259.1ATP binding cassette transporter 3 | F:GO:0005524; C:GO:0016021; F:GO:0016887 | EC:3.6.1.3; EC:3.6.1.15 |
| TRINITY_DN8592_c0_g1_i2_len_578_202265.p1_type_internal_len_193 | KDR12013.1LIM domain kinase 1 | F:GO:0004672; F:GO:0005524; P:GO:0006468; F:GO:0046872 | |
| TRINITY_DN20279_c0_g1_i5_len_726_97783.p1_type_5prime_partial_len_192 | ---NA--- | | |
| TRINITY_DN22332_c0_g1_i1_len_1089_195769.p1_type_5prime_partial_len_192 | ANZ80585.1ubiquitin-conjugating enzyme E2 H | F:GO:0005524; F:GO:0016740 | |
| TRINITY_DN22257_c0_g1_i1_len_1000_196549.p1_type_5prime_partial_len_192 | XP_015177247.1 protein Dr1 | | |
| TRINITY_DN9644_c0_g1_i1_len_876_34243.p1_type_5prime_partial_len_191 | XP_021923912.1uncharacterized protein LOC110831806 | C:GO:0044425 | |
| TRINITY_DN4148_c0_g1_i1_len_990_236186.p1_type_5prime_partial_len_191 | ---NA--- | | |
| TRINITY_DN25269_c0_g1_i2_len_1150_195303.p3_type_5prime_partial_len_191 | ---NA--- | | |
| TRINITY_DN34736_c0_g1_i1_len_574_165457.p1_type_internal_len_192 | EZA54196.1Huntingtin-interacting protein | F:GO:0003779; F:GO:0005543; P:GO:0006897; F:GO:0030276 | |
| TRINITY_DN31801_c0_g1_i1_len_604_201685.p1_type_5prime_partial_len_191 | XP_018007911.1 SH2B adapter protein 1-like | P:GO:0050794 | |
| TRINITY_DN11833_c0_g1_i1_len_575_37346.p1_type_internal_len_192 | ---NA--- | | |
| TRINITY_DN17776_c0_g1_i1_len_1382_193760.p1_type_5prime_partial_len_191 | ---NA--- | | |
| TRINITY_DN18894_c0_g1_i1_len_575_130791.p1_type_internal_len_192 | AET34915.1heat shock protein 21 | | |
| TRINITY_DN24945_c0_g2_i1_len_1649_232333.p1_type_5prime_partial_len_191 | ---NA--- | | |

| | | | |
|---|---|---|---|
| TRINITY_DN10190_c0_g1_i1_len_708_5651.p1_type_complete_len_191 | AEP84099.1acyl-CoA-binding protein | F:GO:0000062 | |
| TRINITY_DN20150_c0_g1_i1_len_1070_235592.p2_type_5prime_partial_len_191 | ---NA--- | | |
| TRINITY_DN21118_c0_g1_i3_len_1286_194347.p1_type_complete_len_191 | ---NA--- | | |
| TRINITY_DN24204_c0_g1_i1_len_899_197513.p1_type_3prime_partial_len_192 | ---NA--- | | |
| TRINITY_DN22545_c0_g1_i2_len_915_4073.p2_type_complete_len_191 | ---NA--- | | |
| TRINITY_DN18822_c0_g1_i1_len_3495_228483.p2_type_complete_len_191 | ---NA--- | | |
| TRINITY_DN20215_c0_g1_i1_len_573_274583.p1_type_internal_len_192 | XP_026479771.1 papilin-like | F:GO:0004867; P:GO:0006508; F:GO:0008233; P:GO:0010951 | |
| TRINITY_DN4657_c0_g1_i1_len_573_241314.p1_type_internal_len_192 | ---NA--- | | |
| TRINITY_DN32300_c0_g1_i1_len_573_241313.p1_type_internal_len_192 | AET79205.1sterile-alpha and armadillo motif containing protein | P:GO:0009967; P:GO:0034128; F:GO:0035591; P:GO:0048678 | |
| TRINITY_DN6331_c0_g1_i1_len_574_241304.p1_type_internal_len_192 | XP_015589803.1GATOR complex protein NPRL3 isoform X2 | | |
| TRINITY_DN8879_c0_g1_i1_len_574_241302.p1_type_internal_len_192 | XP_018017546.1 NFX1-type zinc finger-containing protein 1-like | | |
| TRINITY_DN1849_c0_g1_i1_len_574_241291.p1_type_internal_len_192 | XP_018015738.1 calcium-transporting ATPase type 2C member 1-like | | |
| TRINITY_DN24313_c0_g1_i1_len_3094_228888.p1_type_complete_len_969 | AGU41814.1IRAK1 protein | F:GO:0004672; F:GO:0005524; P:GO:0006468; P:GO:0007165 | |
| TRINITY_DN23334_c0_g1_i1_len_5816_153390.p1_type_complete_len_969 | KDR15146.1Importin-11 | C:GO:0005634; P:GO:0006886; F:GO:0008536 | |
| TRINITY_DN29045_c0_g1_i2_len_3207_188244.p1_type_complete_len_969 | XP_017784184.1 myb-related protein B isoform X1 | F:GO:0003677 | |
| 1498\|_\|c3199_f2p4_2905_isoform_c3199_full_length_coverage_2_non_full_length_coverage_4_isoform_length_2905_28309 | XP_023714865.1transcription elongation regulator 1 isoform X4 | | |

| | | | |
|---|---|---|---|
| 1499\|_\|c1894_f2p11_2905_isoform_c1894_full_length_coverage_2_non_full_length_coverage_11_isoform_length_2905_28055 | ---NA--- | | |
| TRINITY_DN26268_c0_g1_i1_len_4528_227929.p1_type_complete_len_968 | XP_006993041.1 rab3 GTPase-activating protein catalytic subunit isoform X2 | C:GO:0005737; P:GO:0010628; C:GO:0043229; P:GO:0043547; P:GO:0044087; P:GO:0048172; P:GO:0048513; P:GO:0051128; P:GO:0060079; P:GO:0060322; P:GO:0061646; P:GO:1903233 | |
| TRINITY_DN29929_c0_g1_i3_len_3652_187804.p1_type_complete_len_968 | XP_018017949.1 SWI/SNF-related matrix-associated actin-dependent regulator of chromatin subfamily A containing DEAD/H box 1 homolog | | |
| 1502\|_\|c19908_f2p3_2904_isoform_c19908_full_length_coverage_2_non_full_length_coverage_3_isoform_length_2904_22798 | XP_022530100.1histidine-rich glycoprotein-like | | |
| 1503\|_\|c1596_f8p3_2904_isoform_c1596_full_length_coverage_8_non_full_length_coverage_3_isoform_length_2904_15912 | AVN99059.1adenylyl cyclase-associated protein 1 | P:GO:0000902; F:GO:0003779; P:GO:0007010 | |
| 1504\|_\|c33953_f24p19_2903_isoform_c33953_full_length_coverage_24_non_full_length_coverage_19_isoform_length_2903_18648 | AEK49107.1Tudor staphylococcal nuclease | F:GO:0004521; C:GO:0005737; C:GO:0016442; P:GO:0031047; P:GO:0090502 | |
| TRINITY_DN8826_c0_g1_i1_len_575_241279.p1_type_internal_len_192 | XP_018009598.1 mitoguardin-like | | |
| TRINITY_DN13221_c0_g1_i1_len_576_241243.p1_type_internal_len_192 | XP_014243442.1autophagy-related protein 13 isoform X1 | P:GO:0006914 | |
| TRINITY_DN36420_c0_g1_i1_len_576_241246.p1_type_internal_len_192 | ---NA--- | | |
| TRINITY_DN26461_c0_g1_i1_len_576_241248.p1_type_internal_len_192 | XP_020629880.1centrosomal protein of 135 kDa-like isoform X2 | | |
| TRINITY_DN34860_c0_g1_i1_len_576_241255.p1_type_internal_len_192 | XP_022253591.1sodium- and chloride-dependent glycine transporter 1-like | P:GO:0006810; F:GO:0015293; C:GO:0016020 | |

| | | | |
|---|---|---|---|
| TRINITY_DN11763_c0_g1_i2_len_575_241260.p1_type_internal_len_192 | XP_004930658.1RING finger protein 17 | | |
| TRINITY_DN19213_c0_g1_i3_len_3709_228341.p2_type_complete_len_191 | ---NA--- | | |
| TRINITY_DN2183_c0_g1_i1_len_575_241271.p1_type_internal_len_192 | ---NA--- | | |
| TRINITY_DN14104_c0_g1_i1_len_576_274528.p1_type_internal_len_192 | ---NA--- | | |
| TRINITY_DN20026_c0_g1_i1_len_609_201588.p1_type_5prime_partial_len_191 | ---NA--- | | |
| TRINITY_DN16503_c0_g1_i3_len_607_201625.p1_type_3prime_partial_len_192 | ---NA--- | | |
| TRINITY_DN16156_c0_g1_i1_len_772_300390.p1_type_5prime_partial_len_191 | XP_019969096.1 CAD protein-like | | |
| TRINITY_DN21734_c0_g2_i1_len_802_162400.p1_type_3prime_partial_len_192 | ---NA--- | | |
| TRINITY_DN4046_c0_g1_i1_len_768_238333.p1_type_5prime_partial_len_191 | ---NA--- | | |
| TRINITY_DN8397_c0_g1_i1_len_575_67511.p1_type_internal_len_192 | ---NA--- | | |
| TRINITY_DN8310_c0_g1_i1_len_575_202312.p1_type_internal_len_192 | ---NA--- | | |
| TRINITY_DN37034_c0_g1_i1_len_575_202315.p1_type_internal_len_192 | ---NA--- | | |
| TRINITY_DN31686_c0_g1_i1_len_574_202322.p1_type_internal_len_192 | EEB10952.1mixed-lineage leukemia protein, mll, putative | | |
| TRINITY_DN12709_c0_g1_i1_len_576_202304.p1_type_internal_len_192 | ---NA--- | | |
| TRINITY_DN15207_c0_g1_i1_len_577_67467.p1_type_internal_len_192 | XP_023723460.1E3 ubiquitin-protein ligase SIAH1-like | | |
| TRINITY_DN11362_c0_g1_i1_len_575_202310.p1_type_internal_len_192 | EEB11185.1hspc200, putative | | |

| | | | |
|---|---|---|---|
| TRINITY_DN6545_c0_g1_i1_len_577_202271.p1_type_internal_len_192 | XP_021921947.1protein sidekick | C:GO:0016020 | |
| TRINITY_DN18262_c0_g1_i3_len_628_6575.p1_type_5prime_partial_len_191 | XP_018027678.1 gem-associated protein 6-like | | |
| TRINITY_DN19822_c0_g1_i1_len_1912_30537.p1_type_complete_len_191 | ---NA--- | | |
| TRINITY_DN4060_c0_g1_i1_len_600_99428.p1_type_5prime_partial_len_191 | XP_022248041.1voltage-gated hydrogen channel 1-like | | |
| TRINITY_DN22883_c0_g1_i1_len_627_201256.p1_type_5prime_partial_len_191 | EEC19864.1purple acid phosphatase, putative | F:GO:0016787 | |
| TRINITY_DN32476_c0_g1_i1_len_576_202291.p1_type_internal_len_192 | XP_018015557.1 palmitoyltransferase Hip14-like | C:GO:0016020; F:GO:0016409 | |
| TRINITY_DN10406_c0_g1_i1_len_628_201239.p1_type_5prime_partial_len_191 | ---NA--- | | |
| TRINITY_DN18233_c0_g1_i1_len_815_198436.p1_type_5prime_partial_len_191 | KDR06880.1NADH dehydrogenase [ubiquinone] 1 beta subcomplex subunit 8, mitochondrial | F:GO:0003954; C:GO:0016020 | EC:1.6.99.3 |
| TRINITY_DN4688_c0_g1_i1_len_574_202334.p1_type_internal_len_192 | XP_026741703.1dynein heavy chain 12, axonemal | | |
| TRINITY_DN18923_c0_g1_i3_len_576_7390.p1_type_internal_len_192 | ---NA--- | | |
| TRINITY_DN18509_c0_g1_i2_len_1995_266324.p2_type_3prime_partial_len_192 | ---NA--- | | |
| TRINITY_DN16924_c0_g1_i2_len_576_7388.p1_type_internal_len_192 | ---NA--- | | |
| TRINITY_DN13700_c0_g1_i1_len_574_202328.p1_type_internal_len_192 | KZS13329.1Ankyrin repeat and ibr domain containing | F:GO:0016740 | |
| TRINITY_DN14819_c0_g1_i1_len_1998_231126.p2_type_complete_len_191 | ---NA--- | | |
| TRINITY_DN23264_c0_g1_i1_len_1159_62180.p2_type_5prime_partial_len_190 | ---NA--- | | |
| TRINITY_DN17444_c0_g1_i1_len_654_239880.p1_type_5prime_partial_len_190 | ---NA--- | | |

| | | | |
|---|---|---|---|
| TRINITY_DN16596_c0_g1_i1_len_829_198269.p1_type_complete_len_190 | XP_014801789.1 ubiquitin-conjugating enzyme E2 T | F:GO:0005524; F:GO:0016740 | |
| TRINITY_DN8463_c0_g1_i1_len_723_238856.p1_type_5prime_partial_len_190 | XP_022335692.1peptidyl-prolyl cis-trans isomerase NIMA-interacting 4-like | | |
| TRINITY_DN19858_c2_g1_i2_len_740_272356.p1_type_complete_len_190 | XP_019927081.1 protein C8orf37 homolog isoform X2 | | |
| TRINITY_DN13180_c0_g1_i1_len_828_162134.p1_type_complete_len_190 | XP_018025243.1 intraflagellar transport protein 27 homolog | F:GO:0003924; F:GO:0005525; C:GO:0031514 | |
| TRINITY_DN30891_c0_g1_i2_len_6340_186883.p3_type_complete_len_190 | ---NA--- | | |
| TRINITY_DN20661_c0_g1_i4_len_1006_95565.p1_type_complete_len_190 | XP_023242464.1COMM domain-containing protein 4-like isoform X2 | | |
| TRINITY_DN13202_c0_g1_i1_len_638_201042.p1_type_3prime_partial_len_191 | XP_018008089.1 huntingtin-interacting protein 1-like | | |
| TRINITY_DN16706_c0_g1_i1_len_1192_159386.p1_type_complete_len_190 | EAT46923.1AAEL001926-PA | F:GO:0003735; C:GO:0005739 | |
| TRINITY_DN27324_c0_g1_i2_len_3273_188167.p3_type_complete_len_190 | ---NA--- | | |
| TRINITY_DN20565_c0_g1_i1_len_1239_297652.p2_type_5prime_partial_len_190 | XP_018018035.1  uncharacterized oxidoreductase YjmC-like | F:GO:0016491; P:GO:0055114 | |
| TRINITY_DN6432_c0_g1_i1_len_572_37386.p1_type_internal_len_191 | XP_017795072.1 piggyBac transposable element-derived protein 4-like | | |
| TRINITY_DN29919_c0_g1_i1_len_1519_192994.p2_type_complete_len_190 | XP_018012625.1 acyl-coenzyme A thioesterase 9, mitochondrial-like | C:GO:0005739; P:GO:0006637; F:GO:0047617 | EC:3.1.2.20 |
| TRINITY_DN18834_c0_g1_i1_len_1069_160176.p1_type_5prime_partial_len_190 | ---NA--- | | |
| TRINITY_DN13199_c0_g1_i3_len_1029_160431.p1_type_complete_len_190 | XP_018011408.1 mpv17-like protein 2 | C:GO:0016021 | |
| TRINITY_DN22746_c0_g1_i1_len_573_7433.p1_type_internal_len_191 | XP_022190031.1protein mothers against dpp-like isoform X2 | F:GO:0003677; F:GO:0003700; C:GO:0005634; C:GO:0005667; C:GO:0005737; P:GO:0006355; P:GO:0007179 | |

| | | | |
|---|---|---|---|
| TRINITY_DN8127_c0_g1_i1_len_1103_159961.p2_type_complete_len_190 | ---NA--- | | |
| TRINITY_DN29117_c0_g1_i7_len_2889_188719.p2_type_3prime_partial_len_191 | ---NA--- | | |
| TRINITY_DN7465_c0_g1_i2_len_571_7479.p1_type_internal_len_191 | BAO96441.1prophenoloxidase beta | F:GO:0016491; F:GO:0046872; P:GO:0055114 | |
| TRINITY_DN26128_c0_g1_i1_len_5770_227642.p2_type_5prime_partial_len_190 | ---NA--- | | |
| TRINITY_DN30251_c0_g1_i2_len_1255_194553.p1_type_5prime_partial_len_190 | ---NA--- | | |
| TRINITY_DN18281_c0_g1_i3_len_1146_235067.p1_type_5prime_partial_len_190 | XP_018026292.1 sodium- and chloride-dependent glycine transporter 2-like | P:GO:0006810; F:GO:0015293; C:GO:0016020 | |
| TRINITY_DN21468_c0_g1_i3_len_676_163856.p1_type_5prime_partial_len_190 | ---NA--- | | |
| TRINITY_DN30440_c0_g1_i1_len_572_67578.p1_type_internal_len_191 | ---NA--- | | |
| TRINITY_DN24243_c0_g1_i1_len_620_130056.p1_type_5prime_partial_len_190 | KFM82434.1DNA-directed RNA polymerase II subunit RPB7 | P:GO:0000291; C:GO:0000932; F:GO:0003697; F:GO:0003727; F:GO:0003899; C:GO:0005665; P:GO:0006367; F:GO:0031369; P:GO:0045948; P:GO:0060213 | EC:2.7.7.6 |
| TRINITY_DN21423_c2_g1_i1_len_571_67607.p1_type_internal_len_191 | PRD22282.1zinc finger protein | F:GO:0003676 | |
| TRINITY_DN7275_c0_g1_i1_len_572_202363.p1_type_internal_len_191 | XP_018013703.1 ras-GEF domain-containing family member 1B-like isoform X4 | | |
| TRINITY_DN14464_c0_g1_i1_len_855_4443.p1_type_5prime_partial_len_190 | ACY66494.3serine protease | F:GO:0004252; P:GO:0006508; F:GO:0008233 | |
| TRINITY_DN27714_c0_g1_i1_len_572_202364.p1_type_internal_len_191 | XP_010778768.1 low-density lipoprotein receptor-related protein 6-like | | |
| TRINITY_DN11706_c0_g1_i1_len_573_202359.p1_type_internal_len_191 | ---NA--- | | |

| | | | |
|---|---|---|---|
| TRINITY_DN12198_c0_g1_i1_len_570_202407.p1_type_3prime_partial_len_191 | XP_018015382.1 major facilitator superfamily domain-containing protein 8-like | | |
| TRINITY_DN15656_c0_g1_i1_len_574_202332.p1_type_internal_len_191 | ---NA--- | | |
| TRINITY_DN34973_c0_g1_i1_len_573_202360.p1_type_internal_len_191 | XP_021936280.1rab GTPase-activating protein 1-like | F:GO:0005096; P:GO:0043547 | |
| TRINITY_DN23274_c0_g1_i4_len_571_130864.p1_type_internal_len_191 | ---NA--- | | |
| TRINITY_DN11640_c0_g1_i1_len_570_241368.p1_type_internal_len_191 | ---NA--- | | |
| TRINITY_DN3300_c0_g1_i1_len_570_241369.p1_type_internal_len_191 | ---NA--- | | |
| TRINITY_DN19918_c0_g1_i2_len_637_6462.p1_type_5prime_partial_len_190 | ---NA--- | | |
| TRINITY_DN32893_c0_g1_i1_len_573_241317.p1_type_internal_len_191 | XP_018015723.1 copine-8-like | | |
| TRINITY_DN11481_c0_g1_i1_len_573_241324.p1_type_internal_len_191 | AXR98466.1fibrillin 2 | F:GO:0005201; F:GO:0005509; C:GO:0062023 | |
| TRINITY_DN1800_c0_g1_i1_len_572_241327.p1_type_internal_len_191 | ---NA--- | | |
| TRINITY_DN36973_c0_g1_i1_len_572_241329.p1_type_internal_len_191 | ---NA--- | | |
| TRINITY_DN32326_c0_g1_i1_len_572_241340.p1_type_internal_len_191 | ---NA--- | | |
| TRINITY_DN31242_c0_g1_i1_len_572_241344.p1_type_internal_len_191 | ---NA--- | | |
| TRINITY_DN15490_c0_g1_i1_len_574_202338.p1_type_internal_len_191 | ---NA--- | | |
| TRINITY_DN1253_c0_g1_i1_len_572_202373.p1_type_internal_len_191 | XP_018022814.1 nucleosome-remodeling factor subunit NURF301-like | | |
| TRINITY_DN18953_c0_g1_i1_len_582_130663.p1_type_5prime_partial_len_190 | XP_018010107.1PREDICTED: uncharacterized protein LOC108667576 | | |

| | | | |
|---|---|---|---|
| TRINITY_DN3099_c0_g1_i1_len_572_202369.p1_type_internal_len_191 | XP_013384016.1trafficking protein particle complex subunit 8-like | | |
| TRINITY_DN32465_c0_g1_i1_len_571_202391.p1_type_internal_len_191 | ---NA--- | | |
| TRINITY_DN33636_c0_g1_i1_len_572_202376.p1_type_internal_len_191 | ---NA--- | | |
| TRINITY_DN1039_c0_g1_i1_len_571_202396.p1_type_internal_len_191 | EEB11444.1protocadherin fat 2 precursor, putative | F:GO:0004022; F:GO:0005509; C:GO:0005886; P:GO:0007156; C:GO:0016021; P:GO:0055114 | EC:1.1.1.1 |
| TRINITY_DN10958_c0_g1_i1_len_707_199885.p1_type_5prime_partial_len_190 | XP_011450492.1 extracellular tyrosine-protein kinase PKDCC | F:GO:0016301; P:GO:0016310 | |
| TRINITY_DN21017_c0_g1_i2_len_1683_232201.p2_type_complete_len_189 | XP_022237468.1serine/threonine-protein phosphatase 6 regulatory ankyrin repeat subunit A-like | | |
| TRINITY_DN16590_c0_g1_i2_len_1973_156183.p1_type_complete_len_189 | XP_021916611.1peptide methionine sulfoxide reductase isoform X3 | | |
| TRINITY_DN41637_c0_g1_i1_len_773_198945.p1_type_5prime_partial_len_189 | ---NA--- | | |
| TRINITY_DN22915_c0_g1_i1_len_944_236572.p1_type_complete_len_189 | XP_014230124.1haloacid dehalogenase-like hydrolase domain-containing protein 2 | F:GO:0016787 | |
| TRINITY_DN23995_c0_g1_i2_len_1250_194587.p2_type_complete_len_189 | XP_023664209.1E3 ubiquitin-protein ligase RNF185 | | |
| TRINITY_DN4644_c0_g1_i1_len_567_202477.p1_type_internal_len_190 | XP_018027075.1 transient receptor potential cation channel subfamily A member 1-like | | |
| TRINITY_DN15223_c0_g2_i1_len_962_270453.p1_type_5prime_partial_len_189 | ---NA--- | | |
| TRINITY_DN3088_c0_g1_i2_len_670_273148.p1_type_5prime_partial_len_189 | ---NA--- | | |
| TRINITY_DN13426_c0_g1_i1_len_736_300741.p1_type_5prime_partial_len_189 | ---NA--- | | |

| | | | |
|---|---|---|---|
| TRINITY_DN9326_c0_g1_i1_len_567_202483.p1_type_internal_len_190 | ---NA--- | | |
| TRINITY_DN25837_c0_g1_i1_len_1520_192992.p1_type_3prime_partial_len_190 | ---NA--- | | |
| TRINITY_DN19600_c0_g1_i1_len_1783_30793.p2_type_complete_len_189 | XP_019873205.1 6-phosphofructo-2-kinase/fructose-2,6-bisphosphatase-like | | |
| TRINITY_DN41481_c0_g1_i1_len_882_197688.p1_type_5prime_partial_len_189 | XP_015830398.1 zinc finger protein 239-like | | |
| 1505\|_\|c1763_f3p3_2902_isoform_c1763_full_length_coverage_3_non_full_length_coverage_3_isoform_length_2902_25466 | XP_018023639.1 gastrula zinc finger protein xFG20-1-like | F:GO:0003676 | |
| TRINITY_DN20090_c0_g1_i2_len_3182_264837.p1_type_5prime_partial_len_967 | KDR11626.1Oxidation resistance protein 1 | | |
| TRINITY_DN31040_c0_g1_i4_len_4572_187278.p1_type_complete_len_967 | XP_013781538.1protein prune homolog 2-like isoform X2 | | |
| 1508\|_\|c18022_f2p1_2901_isoform_c18022_full_length_coverage_2_non_full_length_coverage_1_isoform_length_2901_13515 | ---NA--- | | |
| TRINITY_DN20466_c0_g2_i3_len_2902_265043.p1_type_internal_len_968 | XP_015179281.1 serine/threonine-protein kinase 11-interacting protein isoform X2 | | |
| TRINITY_DN23286_c0_g1_i4_len_3970_153699.p1_type_complete_len_967 | XP_022082906.1uncharacterized protein LOC110975082 | | |
| TRINITY_DN22661_c0_g1_i1_len_3590_187857.p1_type_5prime_partial_len_967 | ---NA--- | | |
| 1512\|_\|c1981_f2p2_2900_isoform_c1981_full_length_coverage_2_non_full_length_coverage_2_isoform_length_2900_25635 | ---NA--- | | |
| 1513\|_\|c4464_f2p37_2900_isoform_c4464_full_length_coverage_2_non_full_length_coverage_37_isoform_length_2900_12385 | XP_018024310.1 profilin-like | F:GO:0003779; C:GO:0005856 | |
| TRINITY_DN23391_c0_g1_i1_len_3338_154021.p1_type_complete_len_966 | PNF18469.1Polyphosphoinositide phosphatase | F:GO:0042578 | |

| | | | |
|---|---|---|---|
| TRINITY_DN15648_c0_g1_i1_len_567_202481.p1_type_internal_len_190 | XP_022204292.1disheveled-associated activator of morphogenesis 1 | F:GO:0005515; P:GO:0016043 | |
| TRINITY_DN13693_c0_g1_i1_len_570_130877.p1_type_internal_len_190 | XP_013779827.1protein FAM43A-like | | |
| TRINITY_DN11082_c0_g1_i1_len_762_199092.p1_type_3prime_partial_len_190 | XP_023341459.1heme-binding protein 1-like | | |
| TRINITY_DN3602_c0_g1_i1_len_594_67197.p1_type_5prime_partial_len_189 | XP_022920322.1protein NDRG3 isoform X6 | | |
| TRINITY_DN21530_c0_g1_i1_len_681_200299.p1_type_5prime_partial_len_189 | XP_019643620.1 ryncolin-1-like isoform X3 | | |
| TRINITY_DN30111_c1_g3_i1_len_569_202437.p1_type_internal_len_190 | ---NA--- | | |
| TRINITY_DN2072_c0_g1_i1_len_569_202445.p1_type_internal_len_190 | AGL39312.1lipoxygenase | F:GO:0004096; F:GO:0016702; F:GO:0020037; F:GO:0046872; P:GO:0055114; P:GO:0098869 | EC:1.13.11; EC:1.11.1.7; EC:1.11.1.6 |
| TRINITY_DN40624_c0_g1_i1_len_1058_196039.p1_type_5prime_partial_len_189 | XP_018023324.1 branched-chain-amino-acid aminotransferase, cytosolic-like | F:GO:0004084; C:GO:0005739; P:GO:0009098; P:GO:0009099 | EC:2.6.1.42 |
| TRINITY_DN10106_c0_g1_i1_len_567_202498.p1_type_internal_len_190 | ---NA--- | | |
| TRINITY_DN39127_c0_g1_i1_len_567_202496.p1_type_internal_len_190 | OWF53267.1WD repeat, SAM and U-box domain-containing protein 1 | F:GO:0004842; P:GO:0016567 | |
| TRINITY_DN40682_c0_g1_i1_len_568_202467.p1_type_5prime_partial_len_189 | XP_018027076.1 otoferlin-like | F:GO:0005509; C:GO:0016020; C:GO:0016021; P:GO:0016079; C:GO:0045202; C:GO:0098793 | |
| TRINITY_DN34950_c0_g1_i1_len_568_202470.p1_type_internal_len_190 | ---NA--- | | |
| TRINITY_DN4894_c0_g1_i1_len_571_302824.p1_type_internal_len_190 | XP_025116052.1probable leucine--tRNA ligase, mitochondrial | F:GO:0000166; F:GO:0004812; P:GO:0006418 | |
| TRINITY_DN18113_c0_g1_i1_len_571_302814.p1_type_internal_len_190 | XP_018024921.1 enhancer of split mbeta protein-like | F:GO:0000977; P:GO:0001756; F:GO:0003714; C:GO:0005634; P:GO:0006355; P:GO:0007219; F:GO:0008134; F:GO:0046983; P:GO:0050767 | |

| | | | |
|---|---|---|---|
| TRINITY_DN33990_c0_g1_i1_len_567_202499.p1_type_internal_len_190 | ---NA--- | | |
| TRINITY_DN39720_c0_g1_i1_len_568_202459.p1_type_internal_len_190 | KDR12697.1Gustatory receptor trehalose 1 | P:GO:0007165; C:GO:0016020 | |
| TRINITY_DN35471_c0_g1_i1_len_570_241386.p1_type_internal_len_190 | ---NA--- | | |
| TRINITY_DN36468_c0_g1_i1_len_569_241399.p1_type_internal_len_190 | AUC64090.1phosphatidylinositol transfer protein | F:GO:0005548; P:GO:0015914; F:GO:0046872 | |
| TRINITY_DN31446_c0_g1_i1_len_569_241404.p1_type_internal_len_190 | ABP97431.1alpha 2 macroglobulin | F:GO:0004866; C:GO:0005615; P:GO:0010951 | |
| TRINITY_DN8722_c0_g1_i1_len_569_202427.p1_type_internal_len_190 | XP_014616577.1 GAS2-like protein 3 | | |
| TRINITY_DN34372_c0_g1_i1_len_569_241406.p1_type_internal_len_190 | XP_026678252.1nuclear factor interleukin-3-regulated protein | F:GO:0003700; P:GO:0006355 | |
| TRINITY_DN3396_c0_g1_i1_len_568_241413.p1_type_internal_len_190 | XP_022243744.1proteasome activator complex subunit 4-like | C:GO:0005634; C:GO:0005829; P:GO:0010499; F:GO:0016504; F:GO:0070577; F:GO:0070628 | |
| TRINITY_DN8621_c0_g1_i2_len_568_241420.p1_type_internal_len_190 | ---NA--- | | |
| TRINITY_DN2554_c0_g1_i1_len_568_241421.p1_type_internal_len_190 | ---NA--- | | |
| TRINITY_DN20924_c1_g1_i1_len_568_241427.p1_type_internal_len_190 | ---NA--- | | |
| TRINITY_DN1893_c0_g1_i1_len_1013_196446.p2_type_complete_len_189 | XP_018009477.1PREDICTED: uncharacterized protein LOC108667010 | | |
| TRINITY_DN2538_c0_g1_i1_len_571_241348.p1_type_internal_len_190 | ---NA--- | | |
| TRINITY_DN14997_c0_g1_i1_len_571_241352.p1_type_internal_len_190 | KOF92090.1hypothetical protein OCBIM_22007284mg | | |
| TRINITY_DN2991_c0_g1_i1_len_568_274667.p2_type_internal_len_190 | EDV32185.2uncharacterized protein Dana_GF14169 | F:GO:0008146; P:GO:0008152 | |
| TRINITY_DN8391_c0_g1_i1_len_568_274665.p1_type_internal_len_190 | ---NA--- | | |

| | | | |
|---|---|---|---|
| TRINITY_DN9480_c0_g1_i1_len_990_160745.p1_type_5prime_partial_len_189 | ---NA--- | | |
| TRINITY_DN19159_c0_g1_i1_len_612_130166.p1_type_5prime_partial_len_189 | ---NA--- | | |
| TRINITY_DN10066_c0_g1_i1_len_649_239972.p1_type_5prime_partial_len_189 | ---NA--- | | |
| TRINITY_DN34_c0_g1_i1_len_568_241429.p1_type_internal_len_190 | XP_022200368.1uncharacterized protein LOC111057243 isoform X2 | | |
| TRINITY_DN19700_c0_g1_i1_len_612_240603.p1_type_5prime_partial_len_189 | ADM15669.1myosin Va | F:GO:0003774; F:GO:0005524; C:GO:0016459; F:GO:0051015 | EC:3.6.1.15 |
| TRINITY_DN22631_c0_g1_i1_len_571_37404.p1_type_internal_len_190 | XP_022099413.1piggyBac transposable element-derived protein 4-like | | |
| TRINITY_DN25369_c0_g1_i3_len_3290_228671.p3_type_complete_len_189 | ---NA--- | | |
| TRINITY_DN39332_c0_g1_i1_len_568_202472.p1_type_internal_len_190 | BAL49600.1NADPH oxidase | | |
| TRINITY_DN21539_c3_g1_i1_len_970_63303.p1_type_complete_len_188 | ---NA--- | | |
| TRINITY_DN11151_c0_g1_i1_len_565_202549.p1_type_internal_len_189 | ---NA--- | | |
| TRINITY_DN15914_c0_g1_i2_len_1169_269228.p1_type_3prime_partial_len_189 | ---NA--- | | |
| TRINITY_DN17051_c0_g1_i1_len_565_241484.p1_type_internal_len_189 | XP_015765435.1PREDICTED: LOW QUALITY PROTEIN: uncharacterized protein LOC107344296, partial | | |
| TRINITY_DN26539_c0_g1_i1_len_564_241500.p1_type_internal_len_189 | ---NA--- | | |
| TRINITY_DN14064_c0_g1_i1_len_731_272466.p1_type_complete_len_188 | XP_018014250.1 PXMP2/4 family protein 4-like | | |
| TRINITY_DN5304_c0_g1_i1_len_564_241502.p1_type_internal_len_189 | ---NA--- | | |
| TRINITY_DN14471_c0_g1_i1_len_564_241504.p1_type_internal_len_189 | ---NA--- | | |

| | | | |
|---|---|---|---|
| TRINITY_DN18682_c0_g1_i1_len_787_4948.p2_type_complete_len_188 | ---NA--- | | |
| TRINITY_DN31369_c0_g1_i1_len_564_241508.p1_type_internal_len_189 | XP_018022061.1 mucin-5AC-like | | |
| TRINITY_DN27168_c0_g1_i1_len_614_240551.p1_type_5prime_partial_len_188 | XP_018011765.1PREDICTED: uncharacterized protein LOC108668992 | P:GO:0016567 | |
| TRINITY_DN7396_c0_g1_i1_len_722_199682.p1_type_5prime_partial_len_188 | ---NA--- | | |
| TRINITY_DN13942_c0_g1_i1_len_899_63772.p1_type_complete_len_188 | ---NA--- | | |
| TRINITY_DN31290_c0_g1_i1_len_566_130952.p2_type_internal_len_189 | ---NA--- | | |
| TRINITY_DN2535_c0_g1_i1_len_567_202490.p1_type_internal_len_189 | ---NA--- | | |
| TRINITY_DN41895_c0_g1_i1_len_566_202511.p1_type_internal_len_189 | ---NA--- | | |
| TRINITY_DN12139_c0_g1_i1_len_568_202476.p1_type_3prime_partial_len_189 | KZS12485.1Sorting nexin-5 | P:GO:0015031; F:GO:0035091 | |
| TRINITY_DN22967_c0_g1_i1_len_725_65378.p1_type_5prime_partial_len_188 | ---NA--- | | |
| TRINITY_DN1207_c0_g1_i1_len_564_274742.p1_type_internal_len_189 | ---NA--- | | |
| TRINITY_DN33370_c0_g1_i1_len_566_202521.p1_type_internal_len_189 | KZS09955.1Low-density lipoprotein receptor-related protein 6 | F:GO:0005509; C:GO:0016021; F:GO:0017147; F:GO:0042813; P:GO:0060070 | |
| TRINITY_DN2516_c0_g1_i1_len_852_198015.p1_type_complete_len_188 | AHA93923.1Ras | F:GO:0003924; F:GO:0005525; P:GO:0007165; C:GO:0016020 | EC:3.6.1.15 |
| TRINITY_DN820_c0_g1_i1_len_620_273848.p1_type_5prime_partial_len_188 | XP_018006337.1PREDICTED: uncharacterized protein LOC108664281 | | |
| TRINITY_DN9965_c1_g1_i1_len_565_202533.p1_type_5prime_partial_len_188 | XP_021369901.1monoglyceride lipase-like isoform X2 | | |
| TRINITY_DN25152_c0_g1_i1_len_1108_195637.p1_type_complete_len_188 | ---NA--- | | |

| | | | |
|---|---|---|---|
| TRINITY_DN21888_c0_g1_i2_len_776_198908.p1_type_complete_len_188 | ARB15844.1crustin A | C:GO:0005576; P:GO:0010466; F:GO:0030414 | |
| TRINITY_DN3787_c0_g1_i1_len_565_202531.p1_type_internal_len_189 | XP_022820189.1FGGY carbohydrate kinase domain-containing protein | P:GO:0005975; F:GO:0016773 | |
| TRINITY_DN2429_c0_g1_i1_len_565_202523.p1_type_internal_len_189 | ---NA--- | | |
| TRINITY_DN7713_c0_g1_i1_len_567_241437.p2_type_internal_len_189 | ---NA--- | | |
| TRINITY_DN16444_c0_g1_i2_len_567_241440.p1_type_internal_len_189 | ---NA--- | | |
| TRINITY_DN11204_c0_g1_i1_len_567_202488.p2_type_internal_len_189 | XP_018024684.1 protein unc-13 homolog A-like | P:GO:0007268; F:GO:0019992 | |
| TRINITY_DN24355_c0_g1_i3_len_1041_235794.p1_type_complete_len_188 | XP_018020183.1 CKLF-like MARVEL transmembrane domain-containing protein 4 | | |
| TRINITY_DN4118_c0_g1_i1_len_567_241450.p1_type_internal_len_189 | ---NA--- | | |
| TRINITY_DN35652_c0_g1_i1_len_565_241476.p1_type_internal_len_189 | ---NA--- | | |
| TRINITY_DN36387_c0_g1_i1_len_565_241481.p1_type_internal_len_189 | XP_018013792.1 dedicator of cytokinesis protein 1-like | F:GO:0005085; P:GO:0007264 | |
| TRINITY_DN34363_c0_g1_i1_len_566_67681.p1_type_internal_len_189 | XP_018012888.1 hyaluronidase-like | F:GO:0003824; F:GO:0004415; P:GO:0005975; P:GO:0006952; P:GO:0008152; F:GO:0016787; F:GO:0016798 | |
| TRINITY_DN21984_c0_g1_i1_len_914_197346.p1_type_5prime_partial_len_188 | ---NA--- | | |
| TRINITY_DN10187_c0_g1_i1_len_566_202520.p1_type_internal_len_189 | ---NA--- | | |

| | | | |
|---|---|---|---|
| TRINITY_DN37012_c0_g1_i1_len_568_202458.p1_type_5prime_partial_len_188 | EFX86063.1phospholipase-like protein A2, group | F:GO:0004623; F:GO:0005509; C:GO:0005576; P:GO:0006644; P:GO:0016042; F:GO:0016787; P:GO:0050482; F:GO:0102567; F:GO:0102568 | |
| TRINITY_DN17941_c0_g1_i1_len_609_130229.p1_type_5prime_partial_len_188 | XP_018012601.1 coiled-coil domain-containing protein 12-like | | |
| TRINITY_DN19706_c0_g1_i1_len_645_164298.p1_type_5prime_partial_len_188 | ---NA--- | | |
| TRINITY_DN4540_c0_g1_i1_len_564_202564.p2_type_internal_len_189 | BBC28099.1xanthine dehydrogenase | F:GO:0004854; F:GO:0004855; F:GO:0005506; F:GO:0009055; P:GO:0022900; F:GO:0043546; F:GO:0051537; F:GO:0071949 | EC:1.17.3; EC:1.17.1; EC:1.17.1.4; EC:1.17.3.2 |
| TRINITY_DN17316_c0_g1_i2_len_566_7556.p1_type_internal_len_189 | ---NA--- | | |
| TRINITY_DN6859_c0_g1_i1_len_686_163731.p1_type_5prime_partial_len_188 | ---NA--- | | |
| TRINITY_DN6166_c0_g1_i1_len_854_237389.p1_type_5prime_partial_len_188 | XP_023166196.1carbohydrate sulfotransferase 4 isoform X1 | | |
| TRINITY_DN11812_c0_g1_i1_len_568_202471.p1_type_internal_len_189 | ---NA--- | | |
| TRINITY_DN13841_c0_g1_i1_len_601_201753.p1_type_3prime_partial_len_189 | CAX71994.1Tubulin beta-2C chain | F:GO:0003924; F:GO:0005200; F:GO:0005525; C:GO:0005874; P:GO:0007010; P:GO:0007017 | EC:3.6.1.15 |
| TRINITY_DN15068_c0_g1_i1_len_566_100009.p1_type_internal_len_189 | ---NA--- | | |
| TRINITY_DN17414_c0_g1_i1_len_567_302886.p2_type_internal_len_189 | ---NA--- | | |
| TRINITY_DN25739_c0_g1_i1_len_877_63939.p1_type_5prime_partial_len_188 | ANZ80587.1ubiquitin-conjugating enzyme E2 G1 | F:GO:0005524; F:GO:0016740 | |
| TRINITY_DN15216_c0_g1_i1_len_822_198346.p1_type_5prime_partial_len_188 | ---NA--- | | |

| | | | |
|---|---|---|---|
| TRINITY_DN21280_c0_g1_i1_len_568_202473.p1_type_internal_len_189 | XP_018018394.1 zinc finger C4H2 domain-containing protein-like | | |
| TRINITY_DN6690_c0_g1_i1_len_703_239157.p1_type_complete_len_188 | ---NA--- | | |
| TRINITY_DN17334_c0_g1_i1_len_717_238938.p1_type_5prime_partial_len_188 | ---NA--- | | |
| TRINITY_DN24045_c0_g1_i3_len_4163_121841.p3_type_complete_len_188 | XP_022257900.1mothers against decapentaplegic homolog 4-like | | |
| TRINITY_DN23763_c0_g1_i1_len_564_202560.p1_type_internal_len_189 | ---NA--- | | |
| TRINITY_DN28953_c0_g1_i3_len_2071_190654.p1_type_complete_len_188 | ---NA--- | | |
| TRINITY_DN1430_c0_g1_i1_len_563_241518.p1_type_internal_len_188 | XP_018008806.1 integrin alpha-PS1-like | | |
| TRINITY_DN10263_c0_g1_i1_len_562_241533.p1_type_internal_len_188 | KRT83353.1phosphotransferase | F:GO:0016740 | |
| TRINITY_DN18392_c0_g1_i4_len_562_241531.p2_type_internal_len_188 | ---NA--- | | |
| TRINITY_DN23027_c0_g1_i1_len_747_65121.p1_type_complete_len_187 | ---NA--- | | |
| TRINITY_DN35040_c0_g1_i1_len_565_241477.p1_type_internal_len_188 | XP_018027333.1 low-density lipoprotein receptor-related protein 2-like | F:GO:0005509; C:GO:0016021 | |
| TRINITY_DN12119_c0_g1_i1_len_1207_125381.p1_type_complete_len_187 | KOC70521.1Transmembrane and coiled-coil domains protein 1 | F:GO:0005262; C:GO:0030176; P:GO:0032469; P:GO:0070588 | |
| TRINITY_DN30373_c0_g1_i2_len_5622_186989.p1_type_5prime_partial_len_1734 | XP_021940082.1integrin alpha-PS1-like isoform X1 | C:GO:0016020 | |
| TRINITY_DN25650_c0_g1_i1_len_5835_227633.p1_type_5prime_partial_len_1730 | XP_026278316.1phosphatidylinositol 4-phosphate 3-kinase C2 domain-containing subunit beta isoform X1 | | |
| TRINITY_DN30988_c0_g1_i2_len_5525_187011.p1_type_5prime_partial_len_1727 | XP_019923619.1 intersectin-1 isoform X5 | | |
| TRINITY_DN24921_c0_g1_i1_len_5552_227676.p1_type_complete_len_1723 | XP_018015061.1 arginine-glutamic acid dipeptide repeats protein-like isoform X2 | | |

| | | | |
|---|---|---|---|
| TRINITY_DN31152_c1_g1_i3_len_6812_186829.p1_type_5prime_partial_len_1717 | XP_018015533.1PREDICTED: uncharacterized protein LOC108672400 | | |
| TRINITY_DN30613_c0_g1_i1_len_5340_187048.p1_type_5prime_partial_len_1717 | XP_018021438.1 anaphase-promoting complex subunit 1-like isoform X2 | | |
| TRINITY_DN31161_c0_g1_i1_len_6345_186880.p1_type_complete_len_1711 | ---NA--- | | |
| TRINITY_DN31187_c0_g1_i1_len_6337_186884.p1_type_5prime_partial_len_1710 | XP_021927755.1CD109 antigen-like isoform X1 | F:GO:0004866; C:GO:0005576; C:GO:0005615; P:GO:0010951 | |
| TRINITY_DN26364_c0_g1_i1_len_5720_227650.p1_type_complete_len_1708 | ADB65770.2drosha | F:GO:0003723; F:GO:0004525; P:GO:0006396; P:GO:0016075; P:GO:0090502 | EC:3.1.30; EC:3.1.26; EC:3.1.26.3 |
| TRINITY_DN25095_c0_g1_i2_len_5110_57714.p1_type_5prime_partial_len_1701 | PNF42354.1MON2-like protein | P:GO:0015031 | |
| TRINITY_DN23823_c0_g1_i2_len_3156_228813.p1_type_3prime_partial_len_967 | XP_012550293.1tight junction protein ZO-1 isoform X12 | | |
| TRINITY_DN29362_c0_g1_i8_len_5306_187055.p1_type_complete_len_966 | XP_021925419.1rho GTPase-activating protein 26 isoform X3 | | |
| TRINITY_DN25179_c0_g1_i1_len_3492_228486.p1_type_5prime_partial_len_966 | ALM23458.1HMG-CoA reductase membrane form | F:GO:0004420; C:GO:0005789; P:GO:0008299; P:GO:0015936; C:GO:0016021; F:GO:0050661; P:GO:0055114 | EC:1.1.1.34 |
| TRINITY_DN29973_c0_g1_i2_len_4331_187404.p1_type_5prime_partial_len_966 | XP_018015763.1PREDICTED: uncharacterized protein LOC108672575 isoform X3 | | |
| TRINITY_DN30838_c0_g1_i2_len_3328_188099.p1_type_5prime_partial_len_966 | XP_018008188.1 putative ATP-dependent RNA helicase TDRD12 isoform X1 | | |
| 1520\|_\|c2238_f2p1_2895_isoform_c2238_full_length_coverage_2_non_full_length_coverage_1_isoform_length_2895_16254 | XP_018017182.1PREDICTED: uncharacterized protein LOC108673820 | P:GO:0034773; F:GO:0042799 | EC:2.1.1.43 |
| 1521\|_\|c1861_f2p4_2895_isoform_c1861_full_length_coverage_2_non_full_length_coverage_4_isoform_length_2895_28032 | ---NA--- | | |

| | | | |
|---|---|---|---|
| TRINITY_DN19520_c0_g1_i1_len_3256_264794.p1_type_5prime_partial_len_965 | XP_018013942.1 transient receptor potential cation channel subfamily A member 1-like | | |
| TRINITY_DN21962_c0_g1_i1_len_3251_188186.p1_type_5prime_partial_len_965 | XP_021913244.1pecanex-like protein 1 | C:GO:0016021 | |
| 1525\|_\|c18147_f2p9_2894_isoform_c18147_full_length_coverage_2_non_full_length_coverage_9_isoform_length_2894_13609 | ---NA--- | | |
| TRINITY_DN19766_c0_g1_i3_len_1228_159166.p2_type_3prime_partial_len_188 | ---NA--- | | |
| TRINITY_DN31303_c0_g1_i1_len_563_241510.p1_type_internal_len_188 | XP_021940399.1protein furry | P:GO:0000902 | |
| TRINITY_DN20254_c0_g1_i2_len_564_241505.p1_type_internal_len_188 | ---NA--- | | |
| TRINITY_DN14486_c0_g1_i1_len_565_202522.p1_type_internal_len_188 | ---NA--- | | |
| TRINITY_DN37100_c0_g1_i1_len_562_241542.p1_type_internal_len_188 | XP_018024015.1 neurobeachin-like | | |
| TRINITY_DN24333_c0_g1_i1_len_1105_235339.p1_type_complete_len_187 | PRD36862.1Protein LZIC | F:GO:0008013 | |
| TRINITY_DN10081_c0_g1_i1_len_564_130987.p1_type_internal_len_188 | ---NA--- | | |
| TRINITY_DN4870_c0_g1_i1_len_585_241086.p1_type_3prime_partial_len_188 | ---NA--- | | |
| TRINITY_DN7871_c0_g1_i1_len_565_165624.p1_type_internal_len_188 | ---NA--- | | |
| TRINITY_DN26425_c0_g1_i1_len_2071_190657.p3_type_complete_len_187 | ---NA--- | | |
| TRINITY_DN31973_c0_g1_i1_len_564_165648.p1_type_internal_len_188 | XP_018015665.1 MOXD1 homolog 2-like | F:GO:0004500; F:GO:0005507; C:GO:0005615; P:GO:0006589; C:GO:0030667; P:GO:0042420; P:GO:0042421; P:GO:0055114 | EC:1.14.17.1; EC:1.14.17 |

| | | | |
|---|---|---|---|
| TRINITY_DN20873_c0_g1_i1_len_720_65443.p1_type_5prime_partial_len_187 | ---NA--- | | |
| TRINITY_DN35191_c0_g1_i1_len_611_240617.p1_type_5prime_partial_len_187 | ---NA--- | | |
| TRINITY_DN33366_c0_g1_i1_len_576_241235.p1_type_5prime_partial_len_187 | ---NA--- | | |
| TRINITY_DN5147_c0_g1_i1_len_563_100065.p1_type_internal_len_188 | ---NA--- | | |
| TRINITY_DN21980_c0_g1_i1_len_687_200212.p1_type_5prime_partial_len_187 | ---NA--- | | |
| TRINITY_DN8111_c0_g1_i1_len_821_64399.p1_type_5prime_partial_len_187 | XP_018027075.1 transient receptor potential cation channel subfamily A member 1-like | | |
| TRINITY_DN14584_c0_g1_i1_len_1856_59648.p1_type_5prime_partial_len_187 | ADY38993.1nuclear progesterone receptor | | |
| TRINITY_DN14482_c1_g1_i1_len_562_202612.p1_type_internal_len_188 | XP_018010802.1 disks large homolog 5-like | | |
| TRINITY_DN18252_c0_g1_i1_len_563_67753.p2_type_internal_len_188 | KUG02240.1Ovoinhibitor | | |
| TRINITY_DN16984_c0_g1_i1_len_594_99525.p1_type_5prime_partial_len_187 | ---NA--- | | |
| TRINITY_DN19707_c0_g1_i2_len_869_197814.p1_type_3prime_partial_len_188 | ---NA--- | | |
| TRINITY_DN6487_c0_g1_i1_len_564_202572.p1_type_internal_len_188 | ---NA--- | | |
| TRINITY_DN17821_c0_g1_i1_len_1402_193640.p3_type_3prime_partial_len_188 | ---NA--- | | |
| TRINITY_DN15173_c0_g1_i1_len_726_163197.p1_type_3prime_partial_len_188 | ---NA--- | | |
| TRINITY_DN13992_c0_g1_i1_len_703_98039.p1_type_5prime_partial_len_187 | KZS14332.1 Wnt | F:GO:0005102; C:GO:0005576; P:GO:0007275; P:GO:0016055 | |
| TRINITY_DN17653_c0_g1_i1_len_611_130197.p1_type_5prime_partial_len_187 | AET87131.1myosin light chain | F:GO:0005509 | |

| | | | |
|---|---|---|---|
| TRINITY_DN30105_c0_g1_i1_len_3043_188464.p2_type_5prime_partial_len_187 | ---NA--- | | |
| TRINITY_DN11569_c0_g1_i1_len_712_163386.p1_type_5prime_partial_len_187 | ---NA--- | | |
| TRINITY_DN21484_c0_g1_i1_len_981_196717.p1_type_complete_len_187 | XP_018016961.1 phosphatidylinositol N-acetylglucosaminyltransferase subunit H-like | | |
| TRINITY_DN30847_c0_g1_i2_len_1588_192616.p2_type_5prime_partial_len_187 | ---NA--- | | |
| TRINITY_DN15455_c0_g1_i1_len_682_200286.p1_type_complete_len_187 | ---NA--- | | |
| TRINITY_DN11758_c0_g1_i1_len_564_7592.p1_type_internal_len_188 | ---NA--- | | |
| TRINITY_DN9283_c0_g1_i1_len_595_7057.p1_type_5prime_partial_len_186 | XP_026274316.1uncharacterized protein LOC113203717 | | |
| TRINITY_DN14777_c0_g1_i1_len_558_165776.p1_type_internal_len_187 | ---NA--- | | |
| TRINITY_DN20941_c0_g1_i2_len_1024_126396.p2_type_complete_len_186 | XP_012914858.1 COMM domain-containing protein 4 isoform X4 | | |
| TRINITY_DN17425_c0_g1_i1_len_765_300451.p1_type_complete_len_186 | KDR11482.1Dihydrofolate reductase | P:GO:0046394; P:GO:1901566 | |
| TRINITY_DN24197_c0_g1_i1_len_776_198904.p1_type_5prime_partial_len_186 | ---NA--- | | |
| TRINITY_DN16355_c0_g1_i2_len_755_272201.p1_type_3prime_partial_len_187 | ---NA--- | | |
| TRINITY_DN1480_c0_g1_i1_len_559_165752.p1_type_internal_len_187 | KXZ75916.11-phosphatidylinositol 4,5-bisphosphate phosphodiesterase gamma-1-like Protein | P:GO:0007165; F:GO:0008081; P:GO:0016042 | |
| TRINITY_DN15245_c0_g1_i2_len_560_37553.p1_type_internal_len_187 | XP_018015382.1 major facilitator superfamily domain-containing protein 8-like | | |
| TRINITY_DN16850_c0_g1_i1_len_835_198198.p1_type_complete_len_186 | XP_023235830.1tetratricopeptide repeat protein 36 homolog | | |

| | | | |
|---|---|---|---|
| TRINITY_DN21125_c0_g1_i5_len_2700_91332.p2_type_complete_len_186 | ---NA--- | | |
| TRINITY_DN39875_c0_g1_i1_len_560_202662.p1_type_internal_len_187 | ACO12292.1Cell adhesion molecule 3 precursor | C:GO:0016020; C:GO:0016021 | |
| TRINITY_DN15853_c0_g1_i1_len_1120_235242.p2_type_3prime_partial_len_187 | ---NA--- | | |
| TRINITY_DN17402_c0_g1_i3_len_2381_265615.p1_type_complete_len_186 | ---NA--- | | |
| TRINITY_DN20901_c0_g1_i1_len_565_202534.p1_type_5prime_partial_len_186 | XP_018027576.1 3-mercaptopyruvate sulfurtransferase-like | | |
| TRINITY_DN16440_c0_g1_i1_len_559_241584.p1_type_internal_len_187 | XP_018023545.1 oxysterol-binding protein-related protein 6-like | P:GO:0006869 | |
| TRINITY_DN6902_c0_g1_i1_len_560_241573.p1_type_internal_len_187 | XP_018020814.1 UDP-N-acetylhexosamine pyrophosphorylase-like | F:GO:0070569 | |
| TRINITY_DN21243_c0_g1_i1_len_826_64352.p1_type_3prime_partial_len_187 | ---NA--- | | |
| TRINITY_DN18330_c0_g1_i1_len_560_202661.p1_type_internal_len_187 | AFJ11395.1microsomal prostaglandin E synthase | C:GO:0016021 | |
| TRINITY_DN3231_c0_g1_i1_len_560_202667.p1_type_internal_len_187 | ---NA--- | | |
| TRINITY_DN21081_c0_g1_i2_len_565_241479.p1_type_5prime_partial_len_186 | ---NA--- | | |
| TRINITY_DN9667_c0_g1_i1_len_559_274838.p1_type_internal_len_187 | XP_018024766.1 sorting nexin-18-like | | |
| TRINITY_DN25209_c0_g1_i1_len_559_274834.p1_type_internal_len_187 | ---NA--- | | |
| TRINITY_DN22247_c0_g1_i2_len_566_241465.p1_type_5prime_partial_len_186 | CAA39402.1ribulose bisphosphate carboxylase /oxygenase small subunit | F:GO:0004497; C:GO:0009507; P:GO:0009853; F:GO:0016984; P:GO:0019253; P:GO:0055114 | EC:4.1.1.39 |
| TRINITY_DN24486_c0_g1_i1_len_2221_58909.p2_type_complete_len_186 | XP_018022215.1 solute carrier family 28 member 3-like | | |

| | | | |
|---|---|---|---|
| TRINITY_DN3504_c0_g1_i1_len_562_274783.p1_type_internal_len_187 | XP_015835712.1 pyrazinamidase/nicotinamidase isoform X2 | | |
| TRINITY_DN31239_c0_g1_i3_len_4823_187187.p3_type_complete_len_186 | ---NA--- | | |
| TRINITY_DN25080_c0_g1_i1_len_778_198877.p2_type_complete_len_186 | ---NA--- | | |
| TRINITY_DN12010_c0_g1_i1_len_560_7653.p1_type_internal_len_187 | XP_022198579.1BLOC-1-related complex subunit 5 | P:GO:0032418 | |
| TRINITY_DN11014_c0_g1_i1_len_559_131072.p1_type_internal_len_187 | ---NA--- | | |
| TRINITY_DN33564_c0_g1_i1_len_560_131057.p1_type_internal_len_187 | XP_018011797.1 E3 ubiquitin-protein ligase HUWE1-like | | |
| TRINITY_DN9552_c0_g1_i1_len_562_202609.p1_type_internal_len_187 | ---NA--- | | |
| TRINITY_DN21265_c0_g1_i3_len_885_237091.p2_type_5prime_partial_len_186 | ---NA--- | | |
| TRINITY_DN16437_c0_g1_i1_len_559_67850.p1_type_internal_len_187 | ---NA--- | | |
| TRINITY_DN14374_c0_g1_i2_len_560_67817.p1_type_internal_len_187 | XP_018028125.1 retinol dehydrogenase 12-like | | |
| TRINITY_DN13038_c0_g1_i1_len_664_239742.p1_type_3prime_partial_len_187 | XP_018025949.1 methylenetetrahydrofolate reductase-like | F:GO:0004489; C:GO:0005829; P:GO:0009086; P:GO:0035999; P:GO:0055114; F:GO:0071949 | EC:1.5.1.20 |
| TRINITY_DN41724_c0_g1_i1_len_587_202045.p1_type_5prime_partial_len_186 | ---NA--- | | |
| TRINITY_DN28248_c1_g1_i1_len_757_199159.p2_type_5prime_partial_len_186 | ---NA--- | | |
| TRINITY_DN37522_c0_g1_i1_len_558_202701.p1_type_internal_len_187 | XP_013783216.1prickle-like protein 3 | F:GO:0046872 | |
| TRINITY_DN21949_c0_g1_i1_len_1802_191658.p2_type_3prime_partial_len_187 | KDR09499.1Cysteine sulfinic acid decarboxylase | F:GO:0016831; P:GO:0019752; F:GO:0030170 | |

| | | | |
|---|---|---|---|
| TRINITY_DN16867_c0_g1_i1_len_559_202670.p1_type_internal_len_187 | XP_018019219.1 thyroid adenoma-associated protein homolog | | |
| TRINITY_DN21407_c0_g1_i1_len_1371_193835.p2_type_5prime_partial_len_186 | ---NA--- | | |
| TRINITY_DN26835_c0_g1_i1_len_1542_192883.p1_type_5prime_partial_len_186 | ---NA--- | | |
| TRINITY_DN39920_c0_g1_i1_len_560_202657.p1_type_internal_len_187 | XP_026760164.1semaphorin-5A-like, partial | | |
| TRINITY_DN6514_c0_g1_i2_len_560_202656.p1_type_internal_len_187 | ---NA--- | | |
| TRINITY_DN11280_c0_g1_i2_len_559_37570.p1_type_internal_len_187 | ---NA--- | | |
| TRINITY_DN15809_c0_g1_i1_len_715_199763.p1_type_5prime_partial_len_186 | AUC64079.1LWS opsin 2 | F:GO:0004930; P:GO:0007186; P:GO:0007601; P:GO:0007602; F:GO:0009881; C:GO:0016021; P:GO:0018298 | |
| TRINITY_DN8187_c0_g1_i1_len_560_202653.p1_type_internal_len_187 | ---NA--- | | |
| TRINITY_DN12288_c0_g1_i2_len_556_303074.p1_type_internal_len_186 | ---NA--- | | |
| TRINITY_DN25222_c0_g1_i1_len_1011_236032.p2_type_complete_len_185 | XP_018008984.1 vacuolar protein sorting-associated protein 28 homolog | C:GO:0000813; P:GO:0043328 | |
| TRINITY_DN289_c0_g1_i1_len_558_202702.p1_type_internal_len_186 | XP_018028172.1 sushi, von Willebrand factor type A, EGF and pentraxin domain-containing protein 1-like | F:GO:0004930; F:GO:0005509; P:GO:0007186; C:GO:0016020 | |
| TRINITY_DN40669_c0_g1_i1_len_556_202749.p1_type_internal_len_186 | ---NA--- | | |
| TRINITY_DN2074_c0_g1_i1_len_725_272530.p1_type_5prime_partial_len_185 | XP_022187221.1gastrula zinc finger protein XlCGF57.1-like | | |
| TRINITY_DN263_c0_g1_i1_len_716_199755.p1_type_5prime_partial_len_185 | XP_018027697.1PREDICTED: uncharacterized protein LOC108682943 isoform X2 | | |

| | | | |
|---|---|---|---|
| TRINITY_DN18100_c0_g1_i1_len_3048_29463.p3_type_5prime_partial_len_185 | ---NA--- | | |
| TRINITY_DN5321_c0_g1_i1_len_556_202748.p1_type_internal_len_186 | ---NA--- | | |
| TRINITY_DN20859_c0_g1_i2_len_557_303048.p1_type_internal_len_186 | ---NA--- | | |
| TRINITY_DN18058_c0_g1_i1_len_889_127317.p1_type_complete_len_185 | AGN98124.1heat shock protein | | |
| TRINITY_DN29878_c0_g1_i1_len_558_100148.p1_type_internal_len_186 | ---NA--- | | |
| TRINITY_DN8824_c0_g1_i1_len_557_202706.p2_type_internal_len_186 | ---NA--- | | |
| TRINITY_DN28847_c0_g1_i1_len_2624_189186.p1_type_5prime_partial_len_185 | AGM32777.1eukaryotic protein of unknown function (DUF872) | C:GO:0016020; C:GO:0016021 | |
| TRINITY_DN24714_c0_g1_i1_len_3193_58047.p4_type_complete_len_185 | ---NA--- | | |
| TRINITY_DN5260_c0_g1_i1_len_556_165820.p1_type_internal_len_186 | XP_004579512.1 GPI ethanolamine phosphate transferase 1 | | |
| TRINITY_DN8292_c1_g1_i1_len_556_165804.p1_type_internal_len_186 | ODN01722.1Alpha-aminoadipic semialdehyde synthase, mitochondrial | F:GO:0016491; P:GO:0055114 | |
| TRINITY_DN3999_c0_g1_i1_len_556_241659.p1_type_internal_len_186 | XP_022115485.1glutamate synthase [NADH], amyloplastic-like | P:GO:0006537; F:GO:0015930; P:GO:0055114 | |
| TRINITY_DN28191_c0_g1_i1_len_557_241639.p1_type_internal_len_186 | XP_021927530.1exportin-6 isoform X4 | C:GO:0005634; P:GO:0006611; F:GO:0008536; F:GO:0008565 | |
| TRINITY_DN13809_c0_g1_i1_len_557_202710.p1_type_internal_len_186 | ---NA--- | | |
| TRINITY_DN13097_c0_g1_i1_len_645_200900.p1_type_3prime_partial_len_186 | ---NA--- | | |
| 1526\|_\|c2407_f2p3_2894_isoform_c2407_full_length_coverage_2_non_full_length_coverage_3_isoform_length_2894_16386 | XP_018017337.1 RNA-binding protein 39-like | | |

| | | | |
|---|---|---|---|
| 1527\|_\|c1945_f3p21_2893_isoform_c1945_full_length_coverage_3_non_full_length_coverage_21_isoform_length_2893_28096 | APM86328.1serine/threonine-protein phosphatase PP2A regulatory subunit B isoform | C:GO:0000159; F:GO:0019888; P:GO:0043666 | |
| TRINITY_DN20812_c1_g1_i1_len_3440_103.p1_type_5prime_partial_len_964 | XP_018016377.1 transcription initiation factor IIA subunit 1-like | | |
| TRINITY_DN26182_c0_g1_i1_len_4362_227999.p1_type_5prime_partial_len_964 | XP_018022904.1 putative glutamate synthase [NADPH] | P:GO:0006537; F:GO:0015930; F:GO:0016639; F:GO:0051536; P:GO:0055114 | |
| TRINITY_DN25694_c0_g1_i3_len_2993_228995.p1_type_5prime_partial_len_964 | ---NA--- | | |
| TRINITY_DN29009_c0_g1_i1_len_4041_187566.p1_type_5prime_partial_len_964 | XP_021365837.1E3 ubiquitin-protein ligase MARCH6-like | | |
| TRINITY_DN15197_c0_g1_i1_len_3485_228492.p1_type_complete_len_964 | XP_023708903.1calsyntenin-1 isoform X1 | C:GO:0016020 | |
| TRINITY_DN29829_c0_g1_i1_len_3035_188481.p1_type_5prime_partial_len_964 | XP_026778711.1protein broad-minded | | |
| 1534\|_\|c18114_f2p3_2892_isoform_c18114_full_length_coverage_2_non_full_length_coverage_3_isoform_length_2892_17961 | XP_021940064.1cystathionine beta-synthase isoform X2 | | |
| 1535\|_\|c2621_f2p1_2891_isoform_c2621_full_length_coverage_2_non_full_length_coverage_1_isoform_length_2891_16563 | XP_021924902.1protein disulfide-isomerase TMX3 isoform X1 | | |
| TRINITY_DN6933_c0_g1_i1_len_559_241588.p1_type_internal_len_186 | XP_018012231.1 PH and SEC7 domain-containing protein 3-like isoform X3 | | |
| TRINITY_DN4462_c0_g1_i1_len_559_241583.p1_type_internal_len_186 | XP_018014164.1 bestrophin-3-like | | |
| TRINITY_DN5223_c0_g1_i1_len_714_5590.p1_type_5prime_partial_len_185 | XP_018007696.1 U4/U6.U5 small nuclear ribonucleoprotein 27 kDa protein-like isoform X2 | P:GO:0008380 | |
| TRINITY_DN4859_c0_g1_i1_len_639_201010.p1_type_5prime_partial_len_185 | ---NA--- | | |
| TRINITY_DN12052_c0_g1_i1_len_599_201803.p1_type_3prime_partial_len_186 | XP_006608131.1 membrane-associated protein Hem-like | | |

| | | | |
|---|---|---|---|
| TRINITY_DN36873_c0_g1_i1_len_559_202669.p1_type_internal_len_186 | XP_017781454.1 nucleosome-remodeling factor subunit NURF301 isoform X1 | | |
| TRINITY_DN29558_c0_g1_i1_len_665_129429.p1_type_complete_len_185 | ---NA--- | | |
| TRINITY_DN23234_c0_g1_i6_len_754_199221.p1_type_5prime_partial_len_185 | KRY96916.1hypothetical protein T11_4388 | | |
| TRINITY_DN8360_c0_g1_i1_len_556_131149.p1_type_internal_len_186 | KDR09499.1Cysteine sulfinic acid decarboxylase | F:GO:0016829 | |
| TRINITY_DN22971_c0_g1_i1_len_559_202674.p1_type_internal_len_186 | ---NA--- | | |
| TRINITY_DN18261_c0_g1_i2_len_555_131166.p1_type_internal_len_186 | ---NA--- | | |
| TRINITY_DN19504_c0_g1_i2_len_763_64969.p1_type_5prime_partial_len_185 | ---NA--- | | |
| TRINITY_DN14621_c0_g1_i1_len_556_274874.p1_type_internal_len_186 | ---NA--- | | |
| TRINITY_DN18266_c0_g1_i1_len_1099_195707.p2_type_complete_len_185 | ---NA--- | | |
| TRINITY_DN12328_c0_g1_i1_len_556_274876.p1_type_internal_len_186 | ---NA--- | | |
| TRINITY_DN19778_c0_g1_i1_len_794_198694.p2_type_complete_len_185 | ---NA--- | | |
| TRINITY_DN32529_c0_g1_i1_len_557_202716.p1_type_internal_len_186 | ---NA--- | | |
| TRINITY_DN6833_c0_g1_i1_len_673_200425.p2_type_5prime_partial_len_185 | XP_018025500.1PREDICTED: uncharacterized protein LOC108681045 | | |
| TRINITY_DN24141_c3_g1_i3_len_572_67571.p1_type_3prime_partial_len_186 | XP_021346951.1uncharacterized protein LOC110446237 | | |
| TRINITY_DN8643_c0_g1_i1_len_558_202691.p1_type_internal_len_186 | XP_018019102.1 zinc finger protein squeeze-like | | |
| TRINITY_DN2524_c0_g1_i1_len_559_202675.p1_type_internal_len_186 | XP_018026562.1PREDICTED: uncharacterized protein LOC108681986, partial | P:GO:0010466; C:GO:0016020; F:GO:0030414 | |

| | | | |
|---|---|---|---|
| TRINITY_DN29149_c0_g1_i1_len_558_67865.p1_type_internal_len_186 | ---NA--- | | |
| TRINITY_DN13153_c0_g1_i4_len_555_274903.p1_type_internal_len_186 | ---NA--- | | |
| TRINITY_DN12509_c0_g1_i1_len_576_37321.p1_type_5prime_partial_len_185 | ---NA--- | | |
| TRINITY_DN13305_c0_g1_i2_len_558_202693.p1_type_internal_len_186 | ---NA--- | | |
| TRINITY_DN7539_c0_g1_i1_len_627_201253.p1_type_3prime_partial_len_186 | BBC20717.1integrin alpha ps | P:GO:0007155; P:GO:0007229; C:GO:0008305 | |
| TRINITY_DN14131_c0_g1_i2_len_553_202812.p1_type_5prime_partial_len_184 | ---NA--- | | |
| TRINITY_DN1612_c0_g1_i1_len_554_202790.p1_type_internal_len_185 | ---NA--- | | |
| TRINITY_DN18984_c0_g1_i2_len_903_236932.p1_type_complete_len_184 | XP_018022038.1PREDICTED: uncharacterized protein LOC108678190 | | |
| TRINITY_DN14504_c0_g1_i1_len_596_201858.p1_type_5prime_partial_len_184 | XP_018025310.1 sodium/myo-inositol cotransporter 2-like | C:GO:0016020 | |
| TRINITY_DN4742_c0_g1_i1_len_554_165869.p1_type_internal_len_185 | XP_015924419.1WD repeat-containing protein 7 | | |
| TRINITY_DN24237_c0_g1_i1_len_553_165876.p1_type_internal_len_185 | XP_018007338.1 transmembrane protein 104-like | C:GO:0016020 | |
| TRINITY_DN19081_c0_g1_i1_len_766_97386.p1_type_5prime_partial_len_184 | ---NA--- | | |
| TRINITY_DN2676_c0_g1_i1_len_555_37646.p1_type_internal_len_185 | PNF29771.1hypothetical protein B7P43_G10700 | F:GO:0003700; C:GO:0005634; P:GO:0006355; F:GO:0043565 | |
| TRINITY_DN14405_c0_g1_i1_len_566_100013.p1_type_5prime_partial_len_184 | XP_018018829.1PREDICTED: uncharacterized protein LOC108675338 | | |
| TRINITY_DN19796_c0_g1_i1_len_2675_265258.p2_type_3prime_partial_len_185 | XP_022257900.1mothers against decapentaplegic homolog 4-like | | |
| TRINITY_DN15588_c0_g1_i1_len_553_37682.p1_type_internal_len_185 | ---NA--- | | |

| | | | |
|---|---|---|---|
| TRINITY_DN18404_c0_g1_i2_len_1109_32957.p1_type_complete_len_184 | ---NA--- | | |
| TRINITY_DN21158_c0_g1_i1_len_924_4020.p2_type_5prime_partial_len_184 | XP_018011163.1 haloalkane dehalogenase-like | F:GO:0003824; C:GO:0016020; C:GO:0016021 | |
| TRINITY_DN23881_c0_g1_i5_len_1470_233167.p1_type_complete_len_184 | XP_022248476.1potassium channel subfamily K member 1-like | P:GO:0006811; C:GO:0016020 | |
| TRINITY_DN20014_c3_g2_i1_len_657_273310.p1_type_5prime_partial_len_184 | ---NA--- | | |
| TRINITY_DN11519_c0_g1_i1_len_555_202756.p1_type_internal_len_185 | XP_018014604.1 probable phosphorylase b kinase regulatory subunit alpha | F:GO:0005516; C:GO:0005886; P:GO:0005977; F:GO:0016301; P:GO:0016310 | |
| TRINITY_DN17503_c0_g1_i1_len_803_162389.p1_type_complete_len_184 | XP_018022120.1 cuticle protein 19-like | F:GO:0042302 | |
| TRINITY_DN23076_c0_g1_i1_len_3358_154010.p3_type_complete_len_184 | XP_018017762.1 transmembrane and TPR repeat-containing protein CG4050-like | | |
| TRINITY_DN13001_c0_g1_i1_len_555_202772.p1_type_internal_len_185 | XP_018011388.1 ral GTPase-activating protein subunit alpha-1-like | | |
| TRINITY_DN33561_c0_g1_i1_len_593_201931.p1_type_3prime_partial_len_185 | XP_012138980.1 uridine phosphorylase 1 isoform X2 | | |
| TRINITY_DN18479_c0_g1_i1_len_1636_192373.p2_type_5prime_partial_len_184 | ---NA--- | | |
| TRINITY_DN15438_c0_g1_i1_len_777_5028.p1_type_complete_len_184 | XP_019490632.1 S-adenosylmethionine synthase-like | | |
| TRINITY_DN11502_c0_g1_i1_len_574_67525.p1_type_3prime_partial_len_185 | ---NA--- | | |
| TRINITY_DN15513_c0_g1_i1_len_1609_232500.p2_type_complete_len_184 | XP_013781798.1equilibrative nucleoside transporter 4-like | | |
| TRINITY_DN24304_c0_g1_i1_len_555_100200.p1_type_internal_len_185 | ---NA--- | | |
| TRINITY_DN31330_c0_g1_i1_len_554_100207.p1_type_internal_len_185 | APG53788.1transient receptor potential cation channel subfamily M-like protein | F:GO:0005216; C:GO:0016021; F:GO:0016740; P:GO:0034220; P:GO:0051262 | |

| | | | |
|---|---|---|---|
| TRINITY_DN5164_c0_g1_i1_len_552_67974.p1_type_3prime_partial_len_185 | KZS07185.1Zinc finger ZFAT-like protein | F:GO:0000979; P:GO:0010468; C:GO:0035097 | |
| TRINITY_DN24449_c0_g1_i1_len_761_199120.p1_type_3prime_partial_len_185 | XP_013982909.1 gastrula zinc finger protein XlCGF7.1-like | F:GO:0003676 | |
| TRINITY_DN20968_c0_g1_i1_len_554_100227.p1_type_internal_len_185 | XP_023218077.1uncharacterized protein LOC111620395 | | |
| TRINITY_DN7869_c0_g1_i1_len_554_202783.p1_type_internal_len_185 | XP_018013614.1PREDICTED: uncharacterized protein LOC108670650 | | |
| TRINITY_DN36093_c0_g1_i1_len_636_201088.p1_type_5prime_partial_len_184 | ---NA--- | | |
| TRINITY_DN27894_c0_g1_i1_len_552_100257.p1_type_internal_len_185 | KRT84734.1C2 domain containing protein | C:GO:0016020 | |
| TRINITY_DN3385_c0_g1_i1_len_554_274920.p1_type_internal_len_185 | ---NA--- | | |
| TRINITY_DN23291_c0_g1_i1_len_631_66617.p1_type_5prime_partial_len_184 | ---NA--- | | |
| TRINITY_DN3937_c0_g1_i1_len_554_202780.p1_type_internal_len_185 | ---NA--- | | |
| TRINITY_DN23072_c0_g1_i2_len_1115_125868.p2_type_5prime_partial_len_184 | KFM81080.1Adducin-related protein | | |
| TRINITY_DN16491_c0_g1_i1_len_1319_158651.p2_type_5prime_partial_len_184 | XP_012256557.1histone-lysine N-methyltransferase setd3 | | |
| TRINITY_DN8288_c0_g1_i1_len_554_241677.p1_type_internal_len_185 | ---NA--- | | |
| TRINITY_DN14552_c1_g1_i1_len_554_241685.p1_type_internal_len_185 | ---NA--- | | |
| TRINITY_DN2603_c0_g1_i1_len_553_202798.p1_type_internal_len_185 | ---NA--- | | |
| TRINITY_DN33202_c0_g1_i1_len_554_241687.p1_type_internal_len_185 | XP_023244325.1WD repeat-containing protein 11-like isoform X2 | | |
| TRINITY_DN2730_c0_g1_i1_len_554_241689.p1_type_internal_len_185 | ---NA--- | | |

| | | | |
|---|---|---|---|
| TRINITY_DN9253_c0_g1_i1_len_554_202794.p1_type_internal_len_185 | AFP33455.1kinesin-like protein KIF3A | F:GO:0003777; F:GO:0005524; C:GO:0005874; P:GO:0007018; F:GO:0008017 | EC:3.6.1.15 |
| TRINITY_DN6834_c0_g1_i1_len_553_241718.p4_type_internal_len_185 | ---NA--- | | |
| TRINITY_DN11133_c0_g1_i1_len_955_236483.p1_type_5prime_partial_len_184 | XP_013413416.1WD repeat, SAM and U-box domain-containing protein 1 isoform X3 | F:GO:0004842; P:GO:0016567 | |
| TRINITY_DN18092_c1_g1_i1_len_552_241728.p1_type_internal_len_185 | ---NA--- | | |
| TRINITY_DN8170_c0_g1_i1_len_555_274894.p1_type_internal_len_185 | ---NA--- | | |
| TRINITY_DN21904_c0_g2_i6_len_761_300499.p1_type_5prime_partial_len_184 | ---NA--- | | |
| TRINITY_DN6462_c0_g1_i1_len_555_241662.p1_type_internal_len_185 | XP_018025267.1 facilitated trehalose transporter Tret1-2 homolog isoform X2 | | |
| TRINITY_DN10766_c0_g1_i1_len_555_241663.p1_type_3prime_partial_len_185 | ---NA--- | | |
| TRINITY_DN32339_c0_g1_i1_len_555_241674.p1_type_internal_len_185 | ---NA--- | | |
| TRINITY_DN12939_c0_g1_i1_len_1504_296771.p1_type_complete_len_183 | ---NA--- | | |
| TRINITY_DN34606_c0_g1_i1_len_663_239750.p1_type_3prime_partial_len_184 | XP_023713435.1zinc finger with UFM1-specific peptidase domain protein-like isoform X2 | F:GO:0003676 | |
| TRINITY_DN21010_c0_g1_i1_len_549_202918.p1_type_internal_len_184 | AHN53443.1reverse transcriptase | F:GO:0003676; F:GO:0003700; F:GO:0003964; P:GO:0006278; P:GO:0006355; F:GO:0008270; P:GO:0015074 | |
| TRINITY_DN18130_c0_g1_i1_len_851_237417.p1_type_complete_len_183 | KDR21734.1NEDD8-conjugating enzyme Ubc12 | F:GO:0005524; F:GO:0016740 | |
| TRINITY_DN12097_c0_g1_i1_len_551_303163.p1_type_internal_len_184 | XP_018009179.1 zinc finger protein 598-like | F:GO:0003676 | |

| | | | |
|---|---|---|---|
| TRINITY_DN23080_c0_g1_i2_len_774_64838.p1_type_5prime_partial_len_183 | XP_022235403.1tyrosine-protein kinase transmembrane receptor Ror2-like | F:GO:0004714; F:GO:0005524; C:GO:0016021; P:GO:0018108 | EC:2.7.10; EC:2.7.10.1; EC:2.7.11 |
| TRINITY_DN19840_c0_g1_i2_len_865_237289.p1_type_complete_len_183 | ---NA--- | | |
| TRINITY_DN18493_c0_g1_i1_len_552_241727.p1_type_internal_len_184 | ---NA--- | | |
| TRINITY_DN14625_c0_g1_i2_len_551_303161.p1_type_internal_len_184 | ---NA--- | | |
| TRINITY_DN33819_c0_g1_i1_len_550_202892.p2_type_internal_len_184 | ---NA--- | | |
| TRINITY_DN33968_c0_g1_i1_len_552_241724.p1_type_internal_len_184 | PNF38815.1Laminin subunit gamma-1 | | |
| TRINITY_DN14894_c0_g1_i1_len_1120_94896.p1_type_5prime_partial_len_183 | XP_014665956.1 microtubule-associated proteins 1A/1B light chain 3C-like | C:GO:0005737; P:GO:0006914 | |
| TRINITY_DN18109_c0_g1_i1_len_847_237461.p1_type_5prime_partial_len_183 | XP_018021730.1 prefoldin subunit 1-like | P:GO:0006457; C:GO:0016272; F:GO:0051082 | |
| TRINITY_DN14821_c0_g1_i1_len_843_96692.p1_type_5prime_partial_len_183 | EEC08808.1secreted salivary gland peptide, putative | | |
| TRINITY_DN23078_c0_g1_i1_len_551_100274.p1_type_internal_len_184 | ---NA--- | | |
| TRINITY_DN29568_c0_g1_i1_len_2481_189497.p1_type_complete_len_183 | ---NA--- | | |
| TRINITY_DN3476_c0_g1_i1_len_601_67079.p1_type_5prime_partial_len_183 | XP_018013038.1 actin-related protein 2/3 complex subunit 5-like | C:GO:0005737; C:GO:0005885; P:GO:0016477; P:GO:0034314; F:GO:0051015 | |
| TRINITY_DN21124_c0_g1_i1_len_1582_157473.p2_type_3prime_partial_len_184 | ---NA--- | | |
| TRINITY_DN15415_c0_g1_i1_len_677_200360.p1_type_5prime_partial_len_183 | XP_022237073.1organic cation transporter protein-like isoform X2 | C:GO:0016020; C:GO:0016021; P:GO:0055085 | |
| TRINITY_DN21552_c0_g1_i1_len_1548_232781.p1_type_5prime_partial_len_183 | ---NA--- | | |
| TRINITY_DN440_c0_g1_i1_len_550_202888.p1_type_internal_len_184 | ---NA--- | | |

<table>
<tr><th></th><th></th><th></th><th></th></tr>
<tr><td>TRINITY_DN39056_c0_g1_i1_len_551_202854.p1_type_internal_len_184</td><td>OWF43009.1Histone-lysine N-methyltransferase 2C</td><td>F:GO:0005488; C:GO:0005634; F:GO:0008168; P:GO:0032259</td><td></td></tr>
<tr><td>TRINITY_DN13234_c0_g1_i1_len_612_201538.p1_type_5prime_partial_len_183</td><td>---NA---</td><td></td><td></td></tr>
<tr><td>1536|_|c2574_f2p9_2890_isoform_c2574_full_length_coverage_2_non_full_length_coverage_9_isoform_length_2890_16524</td><td>---NA---</td><td></td><td></td></tr>
<tr><td>1537|_|c10556_f2p1_2889_isoform_c10556_full_length_coverage_2_non_full_length_coverage_1_isoform_length_2889_12901</td><td>XP_018021561.1 transcription factor TFIIIB component B'' homolog</td><td>C:GO:0000126; F:GO:0000995; F:GO:0003677; P:GO:0006383</td><td></td></tr>
<tr><td>TRINITY_DN23731_c0_g1_i2_len_2985_188561.p1_type_5prime_partial_len_963</td><td>ACV32155.1argonaute-like protein</td><td>F:GO:0003676; P:GO:0031047</td><td></td></tr>
<tr><td>TRINITY_DN26548_c0_g1_i3_len_2891_188714.p1_type_internal_len_964</td><td>XP_018023470.1 intracellular protein transport protein USO1-like isoform X3</td><td></td><td></td></tr>
<tr><td>TRINITY_DN23642_c0_g2_i1_len_3087_154237.p1_type_5prime_partial_len_963</td><td>KRY40733.1Transposon Ty3-G Gag-Pol polyprotein</td><td>F:GO:0003676; P:GO:0015074</td><td></td></tr>
<tr><td>1541|_|c18228_f2p1_2888_isoform_c18228_full_length_coverage_2_non_full_length_coverage_1_isoform_length_2888_18047</td><td>XP_025829260.1F-box/WD repeat-containing protein 5 isoform X1</td><td></td><td></td></tr>
<tr><td>1543|_|c1976_f3p2_2887_isoform_c1976_full_length_coverage_3_non_full_length_coverage_2_isoform_length_2887_20631</td><td>EFX76039.1hypothetical protein DAPPUDRAFT_322760</td><td>F:GO:0005515; P:GO:0010605; C:GO:0030687; C:GO:0030689; P:GO:0031324; C:GO:0031981; P:GO:0051172; P:GO:0080090</td><td></td></tr>
<tr><td>1544|_|c1835_f2p4_2887_isoform_c1835_full_length_coverage_2_non_full_length_coverage_4_isoform_length_2887_28015</td><td>XP_018026376.1PREDICTED: uncharacterized protein LOC108681812</td><td></td><td></td></tr>
<tr><td>TRINITY_DN23851_c0_g1_i3_len_2888_122184.p1_type_internal_len_963</td><td>XP_015179280.1 serine/threonine-protein kinase 11-interacting protein isoform X1</td><td></td><td></td></tr>
<tr><td>1546|_|c2162_f5p1_2886_isoform_c2162_full_length_coverage_5_non_full_length_coverage_1_isoform_length_2886_20747</td><td>---NA---</td><td></td><td></td></tr>
<tr><td>TRINITY_DN5662_c0_g1_i1_len_551_241753.p1_type_internal_len_184</td><td>---NA---</td><td></td><td></td></tr>
</table>

| | | | |
|---|---|---|---|
| TRINITY_DN13495_c0_g1_i1_len_552_241735.p1_type_internal_len_184 | ---NA--- | | |
| TRINITY_DN30654_c0_g1_i1_len_2044_59256.p1_type_complete_len_183 | XP_018027527.1 ras-related protein Rap-2c-like | | |
| TRINITY_DN11134_c0_g1_i2_len_549_202919.p1_type_internal_len_184 | ARE31873.1extracellular signal-regulated kinases | P:GO:0000165; F:GO:0004707; F:GO:0005524; C:GO:0005634; C:GO:0005737; P:GO:0010468 | EC:2.7.11; EC:2.7.11.24 |
| TRINITY_DN2835_c0_g1_i1_len_551_241745.p1_type_internal_len_184 | XP_018017231.1 dmX-like protein 2 isoform X2 | | |
| TRINITY_DN464_c0_g1_i2_len_583_130644.p1_type_5prime_partial_len_183 | ACP40176.1crustin Pm5 | C:GO:0005576; P:GO:0010466; F:GO:0030414 | |
| TRINITY_DN30468_c0_g1_i1_len_550_241776.p1_type_internal_len_184 | ---NA--- | | |
| TRINITY_DN16990_c0_g1_i2_len_2032_30338.p3_type_5prime_partial_len_183 | XP_018012883.1 dopamine beta-hydroxylase-like | | |
| TRINITY_DN37227_c0_g1_i1_len_549_241785.p1_type_internal_len_184 | XP_018025653.1 protein ELYS-like | | |
| TRINITY_DN10078_c0_g1_i1_len_550_241764.p1_type_internal_len_184 | XP_023714711.1sodium/hydrogen exchanger 1-like isoform X4 | P:GO:0006812; F:GO:0015297; C:GO:0016020; P:GO:0055085 | |
| TRINITY_DN5686_c0_g1_i2_len_550_202887.p1_type_internal_len_184 | XP_018024578.1 G-protein coupled receptor moody-like | | |
| TRINITY_DN28463_c0_g1_i1_len_550_241773.p1_type_internal_len_184 | ---NA--- | | |
| TRINITY_DN4133_c0_g1_i2_len_551_7795.p1_type_internal_len_184 | XP_018025456.1 rho GTPase-activating protein 190-like isoform X3 | | |
| TRINITY_DN25988_c1_g1_i1_len_673_6028.p1_type_5prime_partial_len_183 | ---NA--- | | |
| TRINITY_DN17334_c0_g1_i1_len_551_202842.p2_type_internal_len_184 | AKC96436.1prophenoloxidase activating factor 1 | F:GO:0004252; P:GO:0006508 | EC:3.4.21 |
| TRINITY_DN19390_c0_g1_i1_len_551_202853.p1_type_internal_len_184 | ODN03469.1Ectonucleotide pyrophosphatase/phosphodiesterase family member 5 | F:GO:0003824; C:GO:0016020; C:GO:0016021 | |

| | | | |
|---|---|---|---|
| TRINITY_DN19066_c0_g1_i1_len_552_202831.p1_type_internal_len_184 | ---NA--- | | |
| TRINITY_DN11195_c0_g1_i1_len_551_202850.p1_type_internal_len_184 | XP_018023746.1 probable magnesium transporter NIPA1 | | |
| TRINITY_DN40888_c0_g1_i1_len_549_202923.p2_type_internal_len_184 | XP_018020584.1 epidermal growth factor receptor-like | F:GO:0000166; F:GO:0004713; P:GO:0006468 | EC:2.7.10 |
| TRINITY_DN20693_c1_g1_i2_len_767_97375.p1_type_5prime_partial_len_183 | PRD19726.1zinc finger protein | F:GO:0003676 | |
| TRINITY_DN36911_c0_g1_i1_len_557_202721.p1_type_complete_len_183 | EEB16521.1Palmitoyltransferase ZDHHC3, putative | C:GO:0016021; F:GO:0019706 | EC:2.3.1.225 |
| TRINITY_DN19770_c0_g1_i1_len_773_300379.p1_type_5prime_partial_len_183 | XP_018009093.1 single-stranded DNA-binding protein, mitochondrial-like isoform X2 | | |
| TRINITY_DN5878_c0_g1_i1_len_551_131246.p1_type_internal_len_184 | ---NA--- | | |
| TRINITY_DN25508_c0_g1_i2_len_1091_195760.p1_type_complete_len_183 | ABR27687.1cuticle proprotein proCP5.2 | F:GO:0042302 | |
| TRINITY_DN11075_c0_g1_i1_len_594_274236.p1_type_5prime_partial_len_183 | XP_023339865.1carbohydrate sulfotransferase 11-like | C:GO:0000139; C:GO:0005794; P:GO:0005975; F:GO:0008146; C:GO:0016020; C:GO:0016021; P:GO:0016051; F:GO:0016740 | |
| TRINITY_DN16027_c0_g1_i1_len_551_67989.p1_type_internal_len_184 | XP_023712122.1protein phosphatase inhibitor 2-like | P:GO:0043666 | |
| TRINITY_DN15738_c0_g1_i1_len_551_202844.p1_type_internal_len_184 | ---NA--- | | |
| TRINITY_DN22243_c0_g1_i1_len_2329_230173.p2_type_5prime_partial_len_183 | XP_018023456.1 bifunctional 3'-phosphoadenosine 5'-phosphosulfate synthase-like isoform X2 | | |
| TRINITY_DN38209_c0_g1_i1_len_552_202827.p1_type_internal_len_184 | ---NA--- | | |
| TRINITY_DN26976_c0_g1_i1_len_596_274205.p1_type_3prime_partial_len_184 | ---NA--- | | |

| | | | |
|---|---|---|---|
| TRINITY_DN21421_c0_g1_i3_len_2072_92023.p2_type_complete_len_183 | XP_018015635.1 src substrate protein p85-like | | |
| TRINITY_DN20003_c0_g1_i1_len_894_127284.p1_type_5prime_partial_len_183 | XP_011253390.1apoptosis regulatory protein Siva | F:GO:0008270 | |
| TRINITY_DN20031_c0_g1_i2_len_1012_236021.p1_type_5prime_partial_len_183 | ---NA--- | | |
| TRINITY_DN17877_c0_g1_i1_len_691_129102.p1_type_5prime_partial_len_183 | XP_001604863.1 RNA-binding protein 8A | F:GO:0003729; C:GO:0005737; P:GO:0006397; P:GO:0008380; C:GO:0016607; P:GO:0051028 | |
| TRINITY_DN16932_c0_g1_i2_len_547_131320.p1_type_internal_len_183 | ---NA--- | | |
| TRINITY_DN29787_c1_g1_i1_len_2670_189085.p2_type_complete_len_182 | ---NA--- | | |
| TRINITY_DN11280_c0_g1_i1_len_871_299621.p1_type_5prime_partial_len_182 | XP_018009638.1 peptidyl-prolyl cis-trans isomerase FKBP2-like | P:GO:0000413; F:GO:0003755 | EC:5.2.1.8 |
| TRINITY_DN20938_c0_g1_i2_len_893_197570.p1_type_complete_len_182 | ---NA--- | | |
| TRINITY_DN20883_c0_g1_i1_len_1262_234285.p1_type_complete_len_182 | XP_018014878.1 mpv17-like protein | | |
| TRINITY_DN19810_c0_g1_i1_len_549_165973.p1_type_5prime_partial_len_182 | AFJ11395.1microsomal prostaglandin E synthase | C:GO:0016021 | |
| TRINITY_DN895_c0_g1_i1_len_602_130334.p1_type_5prime_partial_len_182 | XP_018012074.1PREDICTED: uncharacterized protein LOC108669280 | | |
| TRINITY_DN27336_c0_g1_i1_len_1457_193324.p2_type_complete_len_182 | XP_016911621.1 type I inositol 3,4-bisphosphate 4-phosphatase isoform X4 | | |
| TRINITY_DN505_c0_g1_i1_len_548_131315.p1_type_internal_len_183 | XP_018007635.1 SEC14-like protein 5 | | |
| TRINITY_DN16821_c0_g1_i1_len_717_199738.p1_type_5prime_partial_len_182 | ---NA--- | | |
| TRINITY_DN22296_c1_g2_i2_len_546_203005.p1_type_internal_len_183 | ---NA--- | | |
| TRINITY_DN15844_c0_g1_i2_len_547_275039.p2_type_internal_len_183 | ---NA--- | | |

| | | | |
|---|---|---|---|
| TRINITY_DN11439_c0_g1_i1_len_793_128085.p1_type_complete_len_182 | ---NA--- | | |
| TRINITY_DN15099_c1_g1_i1_len_550_274986.p1_type_5prime_partial_len_182 | ---NA--- | | |
| TRINITY_DN17332_c0_g1_i2_len_548_166006.p1_type_internal_len_183 | ---NA--- | | |
| TRINITY_DN23958_c0_g1_i1_len_1916_59530.p2_type_5prime_partial_len_182 | ---NA--- | | |
| TRINITY_DN919_c0_g1_i1_len_549_131280.p1_type_internal_len_183 | KFM78083.1RNA-binding protein fusilli | F:GO:0003676; F:GO:0003723 | |
| TRINITY_DN9001_c0_g1_i1_len_800_237950.p1_type_complete_len_182 | ---NA--- | | |
| TRINITY_DN25066_c0_g1_i2_len_1162_234946.p1_type_complete_len_182 | ---NA--- | | |
| TRINITY_DN33165_c0_g1_i1_len_547_166011.p1_type_internal_len_183 | XP_018011105.1PREDICTED: uncharacterized protein LOC108668402 | | |
| TRINITY_DN18219_c0_g1_i2_len_864_197875.p1_type_5prime_partial_len_182 | ---NA--- | | |
| TRINITY_DN25524_c0_g1_i2_len_2922_229101.p2_type_5prime_partial_len_182 | ---NA--- | | |
| TRINITY_DN27784_c0_g1_i1_len_550_241765.p2_type_internal_len_183 | XP_021926038.11-phosphatidylinositol 4,5-bisphosphate phosphodiesterase epsilon-1-like | | |
| TRINITY_DN14748_c0_g2_i1_len_548_202937.p1_type_internal_len_183 | ---NA--- | | |
| TRINITY_DN17914_c0_g1_i1_len_836_198196.p1_type_5prime_partial_len_182 | ---NA--- | | |
| TRINITY_DN7767_c0_g1_i1_len_546_241853.p1_type_internal_len_183 | PSN58422.1hypothetical protein C0J52_02807 | F:GO:0008013; P:GO:0016055; P:GO:0030178 | |
| TRINITY_DN7881_c0_g1_i1_len_547_241832.p1_type_internal_len_183 | XP_018023549.1 polycystic kidney disease protein 1-like 2 | | |
| TRINITY_DN19021_c0_g1_i2_len_548_241824.p2_type_internal_len_183 | ---NA--- | | |

| | | | |
|---|---|---|---|
| TRINITY_DN31223_c0_g1_i1_len_548_241819.p1_type_internal_len_183 | ---NA--- | | |
| TRINITY_DN3046_c0_g1_i1_len_548_241807.p1_type_internal_len_183 | PRD23668.1zinc finger protein | F:GO:0003676 | |
| TRINITY_DN13341_c0_g1_i1_len_549_241805.p1_type_internal_len_183 | ---NA--- | | |
| TRINITY_DN32525_c0_g1_i1_len_549_241787.p1_type_internal_len_183 | ---NA--- | | |
| TRINITY_DN18247_c0_g1_i1_len_1531_31368.p2_type_3prime_partial_len_183 | XP_022237468.1serine/threonine-protein phosphatase 6 regulatory ankyrin repeat subunit A-like | | |
| TRINITY_DN26135_c0_g1_i1_len_562_100078.p1_type_5prime_partial_len_182 | XP_018014348.1PREDICTED: uncharacterized protein LOC108671312 isoform X4 | | |
| TRINITY_DN1291_c0_g1_i1_len_547_202986.p1_type_internal_len_183 | ---NA--- | | |
| TRINITY_DN21095_c0_g1_i1_len_1033_196274.p2_type_5prime_partial_len_182 | XP_019490632.1 S-adenosylmethionine synthase-like | F:GO:0004478; F:GO:0005524; P:GO:0006556 | EC:2.5.1.6 |
| TRINITY_DN37407_c0_g1_i1_len_547_202988.p1_type_internal_len_183 | XP_018013701.1 ras-GEF domain-containing family member 1B-like isoform X2 | | |
| TRINITY_DN22008_c0_g1_i1_len_1087_3138.p2_type_5prime_partial_len_182 | XP_018026034.1 chromodomain-helicase-DNA-binding protein 7-like isoform X3 | | |
| TRINITY_DN10629_c0_g1_i1_len_549_68027.p1_type_internal_len_183 | ---NA--- | | |
| TRINITY_DN15561_c0_g1_i2_len_548_68036.p1_type_internal_len_183 | XP_023702364.1cytoplasmic polyadenylation element-binding protein 2 isoform X1 | | |
| TRINITY_DN28733_c0_g1_i1_len_713_199804.p1_type_5prime_partial_len_182 | ---NA--- | | |
| TRINITY_DN11483_c0_g1_i1_len_548_202953.p1_type_internal_len_183 | XP_018016965.1 latrophilin Cirl-like isoform X1 | | |

| | | | |
|---|---|---|---|
| TRINITY_DN13540_c0_g1_i1_len_641_200963.p1_type_complete_len_182 | ---NA--- | | |
| TRINITY_DN20033_c0_g1_i1_len_549_202902.p1_type_internal_len_183 | ---NA--- | | |
| TRINITY_DN20526_c0_g1_i2_len_550_68008.p1_type_internal_len_183 | ---NA--- | | |
| TRINITY_DN31969_c0_g1_i1_len_548_202961.p1_type_internal_len_183 | ---NA--- | | |
| TRINITY_DN17330_c0_g1_i2_len_860_34358.p1_type_5prime_partial_len_182 | XP_018025498.1 loricrin-like | | |
| TRINITY_DN5774_c0_g1_i1_len_549_202908.p1_type_internal_len_183 | ---NA--- | | |
| TRINITY_DN17808_c0_g1_i1_len_658_200657.p1_type_5prime_partial_len_182 | ---NA--- | | |
| TRINITY_DN4646_c0_g1_i1_len_548_202958.p1_type_internal_len_183 | XP_018327238.1uncharacterized protein LOC108738347 | | |
| TRINITY_DN24059_c0_g1_i1_len_550_37734.p1_type_internal_len_183 | PNF31516.1E3 ubiquitin-protein ligase KCMF1 | F:GO:0003676; F:GO:0008270; P:GO:0016567; F:GO:0016874; F:GO:0061630 | |
| TRINITY_DN6289_c0_g1_i1_len_784_198821.p1_type_5prime_partial_len_182 | XP_020021071.1DNA polymerase subunit gamma-1-like | F:GO:0003677; F:GO:0003887; C:GO:0005760; P:GO:0006260; P:GO:0071897 | EC:2.7.7.7 |
| TRINITY_DN15520_c0_g1_i2_len_693_35808.p1_type_3prime_partial_len_183 | ---NA--- | | |
| TRINITY_DN13812_c0_g1_i1_len_548_100326.p1_type_internal_len_183 | ---NA--- | | |
| TRINITY_DN26833_c0_g1_i1_len_550_7819.p1_type_internal_len_183 | AWM95782.1type 1 phosphodiesterase | F:GO:0004114; P:GO:0007165; F:GO:0046872 | EC:3.1.4.17 |
| TRINITY_DN11664_c0_g1_i2_len_546_202995.p1_type_internal_len_183 | ---NA--- | | |
| TRINITY_DN165_c0_g1_i1_len_550_7820.p1_type_internal_len_183 | XP_026685291.1ubiquitin carboxyl-terminal hydrolase 43 | P:GO:0006511; P:GO:0016579; F:GO:0036459 | EC:3.4.19.12 |

| | | | |
|---|---|---|---|
| TRINITY_DN190_c0_g1_i1_len_739_199455.p2_type_5prime_partial_len_182 | ---NA--- | | |
| TRINITY_DN4635_c0_g1_i1_len_549_202920.p1_type_internal_len_183 | KFM69607.1hypothetical protein X975_03903, partial | C:GO:0005576; P:GO:0006030; F:GO:0008061 | |
| TRINITY_DN18937_c0_g2_i1_len_1052_33232.p1_type_complete_len_181 | XP_022287851.1protein-lysine methyltransferase METTL21D-like | | |
| TRINITY_DN21480_c0_g1_i1_len_1186_195058.p1_type_5prime_partial_len_181 | XP_018020851.1PREDICTED: uncharacterized protein LOC108677188 | | |
| TRINITY_DN18408_c0_g1_i1_len_1118_235261.p1_type_complete_len_181 | KXS13428.1riboflavin biosynthesis protein RibD | F:GO:0008703; P:GO:0009231; P:GO:0055114 | |
| TRINITY_DN8823_c0_g1_i1_len_546_203003.p1_type_internal_len_182 | ---NA--- | | |
| TRINITY_DN20519_c0_g1_i3_len_928_96074.p1_type_complete_len_181 | ---NA--- | | |
| TRINITY_DN30977_c0_g1_i1_len_572_241338.p1_type_5prime_partial_len_181 | XP_018007454.1 gastrula zinc finger protein XlCGF49.1-like | F:GO:0003676; C:GO:0005634 | |
| TRINITY_DN19681_c0_g1_i2_len_665_200550.p1_type_5prime_partial_len_181 | AAZ29608.1C-type lectin | F:GO:0030246 | |
| 1550\|_\|c6739_f2p11_2885_isoform_c6739_full_length_coverage_2_non_full_length_coverage_11_isoform_length_2885_12574 | XP_018011620.1 eukaryotic translation initiation factor 3 subunit B-like | P:GO:0001732; F:GO:0003743; C:GO:0005852; C:GO:0016282; F:GO:0031369; C:GO:0033290 | |
| 1551\|_\|c9545_f3p1_2885_isoform_c9545_full_length_coverage_3_non_full_length_coverage_1_isoform_length_2885_21877 | XP_010368879.1 zinc finger protein 107-like | | |
| 1552\|_\|c18008_f2p1_2885_isoform_c18008_full_length_coverage_2_non_full_length_coverage_1_isoform_length_2885_13506 | XP_012282676.1GATOR complex protein DEPDC5 isoform X4 | | |
| TRINITY_DN24913_c0_g1_i1_len_6486_186859.p1_type_complete_len_961 | XP_018015462.1 F-box only protein 11-like | C:GO:0000151; F:GO:0008270; P:GO:0016567 | |
| 1554\|_\|c2104_f3p0_2883_isoform_c2104_full_length_coverage_3_non_full_length_coverage_0_isoform_length_2883_20708 | ---NA--- | | |

| | | | |
|---|---|---|---|
| TRINITY_DN20280_c0_g1_i2_len_3194_264826.p1_type_complete_len_961 | XP_015905651.1zinc finger RNA-binding protein | | |
| 1556\|_\|c17860_f2p0_2882_isoform_c17860_full_length_coverage_2_non_full_length_coverage_0_isoform_length_2882_22520 | XP_018006544.1 glycine N-methyltransferase-like | | |
| 1557\|_\|c1859_f6p4_2882_isoform_c1859_full_length_coverage_6_non_full_length_coverage_4_isoform_length_2882_16037 | XP_011499134.1 protein Gawky isoform X4 | | |
| 1560\|_\|c7270_f3p6_2880_isoform_c7270_full_length_coverage_3_non_full_length_coverage_6_isoform_length_2880_17122 | XP_015115200.1 ATPase family AAA domain-containing protein 3 | | |
| TRINITY_DN20217_c0_g1_i1_len_4124_228121.p1_type_complete_len_960 | XP_018017154.1 rho GTPase-activating protein 44-like | | |
| TRINITY_DN21185_c2_g1_i1_len_545_203016.p1_type_internal_len_182 | ---NA--- | | |
| TRINITY_DN26025_c0_g1_i1_len_545_131361.p1_type_internal_len_182 | XP_018011143.1 triple functional domain protein-like | | |
| TRINITY_DN11793_c0_g1_i1_len_546_100355.p1_type_internal_len_182 | ---NA--- | | |
| TRINITY_DN20225_c0_g1_i1_len_545_7925.p1_type_internal_len_182 | AAK48894.1CUB-serine protease | F:GO:0008233 | |
| TRINITY_DN30274_c0_g1_i1_len_546_131347.p1_type_internal_len_182 | XP_023717165.1partitioning defective 3 homolog isoform X4 | | |
| TRINITY_DN6697_c0_g1_i1_len_543_203069.p1_type_internal_len_182 | ---NA--- | | |
| TRINITY_DN30415_c0_g1_i1_len_547_241849.p1_type_3prime_partial_len_182 | XP_014773032.1 E3 ubiquitin-protein ligase HUWE1-like | C:GO:0016021 | |
| TRINITY_DN15289_c0_g1_i1_len_721_300884.p1_type_complete_len_181 | XP_022099952.160S ribosome subunit biogenesis protein NIP7 homolog | F:GO:0003723; C:GO:0005730; C:GO:0030687; P:GO:0042255; P:GO:0042273 | |
| TRINITY_DN17403_c1_g1_i1_len_545_37807.p1_type_internal_len_182 | ---NA--- | | |

| | | | |
|---|---|---|---|
| TRINITY_DN28662_c0_g1_i1_len_544_131391.p1_type_internal_len_182 | ---NA--- | | |
| TRINITY_DN13124_c1_g1_i1_len_547_241837.p1_type_internal_len_182 | XP_018022137.1 protein bark beetle-like | | |
| TRINITY_DN27003_c0_g1_i1_len_546_275047.p1_type_internal_len_182 | XP_018009195.1PREDICTED: uncharacterized protein LOC108666782 | | |
| TRINITY_DN21124_c0_g1_i1_len_639_201020.p1_type_5prime_partial_len_181 | KFM64238.1RNA-directed DNA polymerase from mobile element jockey | F:GO:0003676; F:GO:0003964; F:GO:0004523; P:GO:0006278; F:GO:0008270; P:GO:0090502 | |
| TRINITY_DN30666_c0_g1_i1_len_4045_187563.p2_type_complete_len_181 | XP_013860411.1 zinc finger protein OZF-like | F:GO:0003676 | |
| TRINITY_DN22119_c1_g1_i1_len_2801_245.p2_type_complete_len_181 | XP_022257900.1mothers against decapentaplegic homolog 4-like | | |
| TRINITY_DN21911_c0_g2_i1_len_749_300626.p1_type_3prime_partial_len_182 | ---NA--- | | |
| TRINITY_DN5627_c0_g1_i1_len_547_202980.p1_type_internal_len_182 | ---NA--- | | |
| TRINITY_DN20976_c0_g1_i1_len_546_37792.p1_type_internal_len_182 | ---NA--- | | |
| TRINITY_DN32124_c0_g1_i1_len_546_203002.p1_type_internal_len_182 | ---NA--- | | |
| TRINITY_DN35240_c0_g1_i1_len_543_241939.p1_type_internal_len_182 | XP_018009678.1 putative tyrosine-protein kinase Wsck | P:GO:0009987 | |
| TRINITY_DN112_c0_g1_i1_len_544_203035.p1_type_internal_len_182 | ---NA--- | | |
| TRINITY_DN10478_c0_g1_i1_len_544_275092.p1_type_internal_len_182 | ALO17560.1thyroglobulin-like protein | | |
| TRINITY_DN29439_c0_g1_i1_len_543_275099.p1_type_internal_len_182 | XP_025095664.1uncharacterized protein LOC112564805 | | |

| | | | |
|---|---|---|---|
| TRINITY_DN37364_c0_g1_i1_len_544_203034.p1_type_internal_len_182 | XP_016410529.1 DNA-dependent protein kinase catalytic subunit-like | P:GO:0000723; F:GO:0004674; C:GO:0005634; P:GO:0006302; P:GO:0008630; P:GO:0030183; P:GO:0033153; P:GO:0071707; F:GO:0097159; F:GO:1901363 | EC:2.7.11 |
| TRINITY_DN17534_c0_g1_i4_len_545_303293.p2_type_internal_len_182 | XP_018018358.1PREDICTED: uncharacterized protein LOC108674884 | | |
| TRINITY_DN4404_c0_g1_i1_len_545_241896.p1_type_internal_len_182 | ---NA--- | | |
| TRINITY_DN18568_c0_g1_i3_len_544_241904.p1_type_internal_len_182 | ---NA--- | | |
| TRINITY_DN12498_c0_g1_i1_len_545_166058.p1_type_internal_len_182 | XP_018913136.1 E3 ubiquitin-protein ligase MIB2-like | | |
| TRINITY_DN29428_c0_g1_i2_len_1548_192842.p1_type_complete_len_181 | ---NA--- | | |
| TRINITY_DN23136_c0_g1_i2_len_1992_123146.p2_type_5prime_partial_len_181 | XP_026276963.1DDB1- and CUL4-associated factor 12 | | |
| TRINITY_DN34550_c0_g1_i1_len_545_241892.p1_type_internal_len_182 | ---NA--- | | |
| TRINITY_DN5209_c0_g1_i1_len_564_202565.p1_type_5prime_partial_len_181 | ---NA--- | | |
| TRINITY_DN16172_c0_g1_i1_len_543_203063.p1_type_internal_len_182 | ---NA--- | | |
| TRINITY_DN26209_c0_g1_i1_len_562_131032.p1_type_5prime_partial_len_181 | ---NA--- | | |
| TRINITY_DN22041_c0_g1_i1_len_544_100389.p1_type_internal_len_182 | ---NA--- | | |
| TRINITY_DN22150_c1_g1_i1_len_545_68100.p1_type_internal_len_182 | ---NA--- | | |
| TRINITY_DN18162_c0_g1_i1_len_616_201449.p1_type_5prime_partial_len_181 | ---NA--- | | |
| TRINITY_DN13698_c0_g1_i2_len_665_273207.p1_type_5prime_partial_len_181 | ---NA--- | | |

| | | | |
|---|---|---|---|
| TRINITY_DN9602_c0_g1_i1_len_546_202991.p1_type_internal_len_182 | AEX08659.1lipopolysaccharide and beta-1,3-glucan binding protein | F:GO:0001872; P:GO:0002376 | |
| TRINITY_DN15630_c0_g1_i1_len_545_68092.p1_type_internal_len_182 | ---NA--- | | |
| TRINITY_DN7906_c0_g1_i1_len_591_201967.p1_type_5prime_partial_len_181 | XP_018027063.1 pleckstrin homology domain-containing family A member 3-like | | |
| TRINITY_DN38745_c0_g1_i1_len_544_203054.p1_type_internal_len_182 | XP_018011510.1 regulator of G-protein signaling 2-like | | |
| TRINITY_DN13557_c0_g1_i1_len_544_203060.p1_type_internal_len_182 | ---NA--- | | |
| TRINITY_DN12449_c0_g1_i1_len_544_203061.p1_type_internal_len_182 | ---NA--- | | |
| TRINITY_DN24956_c0_g1_i1_len_1021_196382.p1_type_5prime_partial_len_181 | ---NA--- | | |
| TRINITY_DN33923_c0_g1_i1_len_920_197296.p1_type_5prime_partial_len_181 | XP_018016621.1 beta-galactosidase-1-like protein 2 | F:GO:0016798 | |
| TRINITY_DN12346_c0_g1_i1_len_547_131323.p1_type_internal_len_182 | XP_018006493.1 sialin-like | | |
| TRINITY_DN18576_c0_g1_i1_len_1331_233872.p1_type_complete_len_181 | ---NA--- | | |
| TRINITY_DN6838_c0_g1_i1_len_607_274040.p1_type_3prime_partial_len_181 | ---NA--- | | |
| TRINITY_DN4377_c0_g1_i1_len_1580_232642.p1_type_complete_len_180 | XP_018014248.1 ADP-ribosylation factor-like protein 5B | F:GO:0005525; C:GO:0005802; P:GO:0006886; P:GO:0016192; P:GO:1903292 | |
| TRINITY_DN33922_c0_g1_i1_len_541_241977.p1_type_internal_len_181 | XP_018914875.1PREDICTED: uncharacterized protein LOC109042526 isoform X3 | P:GO:0007275; P:GO:0050794 | |
| TRINITY_DN31156_c0_g1_i1_len_541_241986.p1_type_internal_len_181 | KFM72382.1Nuclear receptor subfamily 2 group C member 2 | P:GO:0050794 | |
| TRINITY_DN22485_c0_g1_i2_len_1347_124816.p2_type_5prime_partial_len_180 | XP_018012091.1 protein FAM50A-like isoform X2 | C:GO:0005634 | |

| | | | |
|---|---|---|---|
| TRINITY_DN10969_c1_g1_i1_len_632_301925.p1_type_5prime_partial_len_180 | AYD41591.1mannose receptor | F:GO:0030246 | |
| TRINITY_DN17808_c0_g1_i1_len_658_200657.p2_type_5prime_partial_len_180 | ---NA--- | | |
| TRINITY_DN11457_c1_g1_i1_len_542_37876.p1_type_internal_len_181 | ACR48148.1mannose receptor | F:GO:0030246 | |
| TRINITY_DN1315_c0_g1_i2_len_542_100416.p1_type_internal_len_181 | ---NA--- | | |
| TRINITY_DN11585_c0_g1_i1_len_705_98023.p1_type_complete_len_180 | XP_018010255.1PREDICTED: uncharacterized protein C1orf50 homolog | | |
| TRINITY_DN31793_c0_g1_i1_len_544_241911.p1_type_internal_len_181 | PNF41555.1JmjC domain-containing protein 4 | | |
| TRINITY_DN18313_c0_g1_i1_len_773_5069.p1_type_5prime_partial_len_180 | AEB40303.1mitochondrial ATP synthase delta subunit precursor | P:GO:0015986; C:GO:0045261; F:GO:0046933; P:GO:0099132 | EC:3.6.1.3; EC:3.6.1.15 |
| TRINITY_DN14528_c0_g1_i1_len_544_241914.p1_type_internal_len_181 | ---NA--- | | |
| TRINITY_DN37340_c0_g1_i1_len_544_241924.p1_type_internal_len_181 | ---NA--- | | |
| TRINITY_DN11322_c0_g1_i1_len_862_197903.p1_type_5prime_partial_len_180 | KDR18851.1CDC42 small effector protein-like protein | F:GO:0017048; P:GO:0035023 | |
| TRINITY_DN21288_c0_g1_i1_len_675_200391.p1_type_5prime_partial_len_180 | XP_015912076.1zinc finger protein 271-like isoform X1 | | |
| TRINITY_DN34177_c0_g1_i1_len_617_130099.p1_type_5prime_partial_len_180 | XP_026424028.1histone H3.2-like | F:GO:0005488; C:GO:0005694 | |
| TRINITY_DN15895_c0_g1_i1_len_1014_236004.p1_type_5prime_partial_len_180 | PNF15314.1Mitochondrial inner membrane protease subunit 1 | C:GO:0005739; P:GO:0006508; F:GO:0008233; C:GO:0016020 | |
| TRINITY_DN4918_c0_g1_i1_len_542_241962.p1_type_internal_len_181 | KZS21355.1Laminin subunit gamma-3 | | |
| TRINITY_DN30653_c0_g1_i1_len_542_241971.p1_type_internal_len_181 | XP_018021226.1 organic cation transporter protein-like | | |
| TRINITY_DN1802_c0_g1_i1_len_542_241973.p1_type_internal_len_181 | ---NA--- | | |

| | | | |
|---|---|---|---|
| TRINITY_DN13492_c0_g1_i1_len_543_241931.p1_type_internal_len_181 | XP_018013606.1 WD repeat-containing protein 19-like | C:GO:0005929; P:GO:0035721 | |
| TRINITY_DN36784_c0_g1_i1_len_543_241942.p1_type_internal_len_181 | ---NA--- | | |
| TRINITY_DN12240_c0_g1_i2_len_816_198424.p1_type_complete_len_180 | ---NA--- | | |
| TRINITY_DN9419_c0_g1_i1_len_599_201793.p1_type_5prime_partial_len_180 | ---NA--- | | |
| TRINITY_DN21771_c0_g1_i1_len_542_203110.p1_type_internal_len_181 | PIK37648.1hypothetical protein BSL78_25519 | | |
| TRINITY_DN7006_c0_g1_i1_len_578_202253.p1_type_3prime_partial_len_181 | ---NA--- | | |
| TRINITY_DN968_c0_g1_i1_len_542_203112.p1_type_internal_len_181 | ---NA--- | | |
| TRINITY_DN37460_c0_g1_i1_len_542_203111.p1_type_internal_len_181 | ---NA--- | | |
| TRINITY_DN20870_c0_g1_i1_len_619_201408.p1_type_3prime_partial_len_181 | ---NA--- | | |
| TRINITY_DN10575_c0_g1_i1_len_542_203104.p1_type_internal_len_181 | XP_018011507.1 SLIT-ROBO Rho GTPase-activating protein 1-like | F:GO:0005096; P:GO:0007165; P:GO:0043547 | |
| TRINITY_DN4154_c0_g1_i1_len_543_203079.p1_type_internal_len_181 | ---NA--- | | |
| TRINITY_DN22734_c0_g1_i2_len_1291_194308.p1_type_3prime_partial_len_181 | ---NA--- | | |
| TRINITY_DN18205_c0_g1_i2_len_787_97146.p1_type_3prime_partial_len_181 | XP_018019761.1 flocculation protein FLO11-like | | |
| TRINITY_DN22584_c0_g2_i1_len_629_129908.p1_type_3prime_partial_len_181 | XP_018022019.1 calcium-binding mitochondrial carrier protein SCaMC-1-like isoform X3 | | |
| TRINITY_DN17073_c0_g1_i1_len_756_65036.p1_type_complete_len_180 | ---NA--- | | |
| TRINITY_DN5559_c0_g1_i1_len_542_203113.p1_type_internal_len_181 | ---NA--- | | |

| | | | |
|---|---|---|---|
| TRINITY_DN17425_c0_g1_i1_len_827_198288.p1_type_5prime_partial_len_180 | ---NA--- | | |
| TRINITY_DN17785_c0_g1_i1_len_543_131425.p1_type_internal_len_181 | ---NA--- | | |
| TRINITY_DN10934_c0_g1_i1_len_542_203117.p2_type_5prime_partial_len_180 | ---NA--- | | |
| TRINITY_DN28161_c1_g1_i1_len_1700_192086.p1_type_complete_len_180 | XP_018026877.1 calcineurin subunit B type 2 isoform X4 | F:GO:0005509 | |
| TRINITY_DN5222_c0_g1_i1_len_592_37093.p1_type_complete_len_180 | XP_026272850.1ras-related protein Rab-43 | F:GO:0003924; F:GO:0005525 | |
| TRINITY_DN31386_c0_g1_i1_len_755_199194.p1_type_5prime_partial_len_180 | ---NA--- | | |
| TRINITY_DN10614_c0_g1_i1_len_634_240204.p1_type_3prime_partial_len_181 | EEH38796.2hypothetical protein PAAG_08523 | | |
| TRINITY_DN12439_c0_g1_i1_len_738_199469.p1_type_5prime_partial_len_180 | ---NA--- | | |
| TRINITY_DN9341_c0_g1_i1_len_542_303356.p1_type_internal_len_181 | ---NA--- | | |
| TRINITY_DN24545_c0_g1_i1_len_541_166133.p1_type_internal_len_181 | ---NA--- | | |
| TRINITY_DN34714_c0_g1_i1_len_570_241389.p1_type_3prime_partial_len_181 | XP_022249682.1protein NipSnap homolog 3B-like isoform X2 | | |
| TRINITY_DN15663_c0_g1_i2_len_541_166138.p1_type_internal_len_181 | ---NA--- | | |
| TRINITY_DN5312_c0_g1_i2_len_542_166107.p1_type_internal_len_181 | ---NA--- | | |
| TRINITY_DN16181_c0_g1_i1_len_542_166109.p1_type_internal_len_181 | ---NA--- | | |
| TRINITY_DN11706_c0_g1_i1_len_542_7983.p1_type_internal_len_181 | ---NA--- | | |
| 1562\|_\|c19794_f2p9_2880_isoform_c19794_full_length_coverage_2_non_full_length_coverage_9_isoform_length_2880_28985 | ---NA--- | | |

| | | | |
|---|---|---|---|
| TRINITY_DN28898_c0_g1_i1_len_3082_188403.p1_type_5prime_partial_len_960 | ---NA--- | | |
| 1564\|_\|c2677_f3p2_2879_isoform_c2677_full_length_coverage_3_non_full_length_coverage_2_isoform_length_2879_16608 | XP_018018739.1 DNA polymerase kappa-like | F:GO:0003684; F:GO:0003887; P:GO:0006281; P:GO:0071897 | |
| 1565\|_\|c13535_f2p4_2879_isoform_c13535_full_length_coverage_2_non_full_length_coverage_4_isoform_length_2879_26214 | ESO86545.1hypothetical protein LOTGIDRAFT_128909, partial | C:GO:0016020 | |
| 1566\|_\|c6654_f3p0_2878_isoform_c6654_full_length_coverage_3_non_full_length_coverage_0_isoform_length_2878_21621 | XP_018016946.1 protein FAM114A2-like | | |
| TRINITY_DN23927_c0_g1_i6_len_2966_122138.p1_type_5prime_partial_len_959 | ---NA--- | | |
| TRINITY_DN28078_c0_g1_i1_len_3880_187652.p1_type_complete_len_959 | KDR10287.1GPI inositol-deacylase | C:GO:0005783; C:GO:0005789; P:GO:0015031; C:GO:0016020; C:GO:0016021; F:GO:0016787; F:GO:0016788 | |
| TRINITY_DN29066_c0_g1_i2_len_4331_187403.p1_type_complete_len_959 | XP_018009558.1 serine/arginine repetitive matrix protein 2-like isoform X1 | C:GO:0005634; P:GO:0006355 | |
| 1570\|_\|c18184_f2p2_2877_isoform_c18184_full_length_coverage_2_non_full_length_coverage_2_isoform_length_2877_18016 | ---NA--- | | |
| 1571\|_\|c18133_f2p5_2876_isoform_c18133_full_length_coverage_2_non_full_length_coverage_5_isoform_length_2876_13598 | ---NA--- | | |
| TRINITY_DN911_c0_g1_i1_len_541_100447.p1_type_internal_len_181 | ACZ63470.1c-type lysozyme | F:GO:0003796 | EC:3.2.1.17 |
| TRINITY_DN28391_c0_g1_i1_len_2539_189369.p3_type_complete_len_180 | ---NA--- | | |
| TRINITY_DN14836_c0_g1_i1_len_1319_93950.p1_type_5prime_partial_len_180 | XP_018027633.1 carbohydrate sulfotransferase 1-like | F:GO:0001517; P:GO:0006044; P:GO:0006790; F:GO:0016740 | |
| TRINITY_DN8209_c0_g1_i1_len_986_236233.p1_type_5prime_partial_len_180 | AHH29321.1protein inhibitor of activated STAT | F:GO:0008270 | |

| | | | |
|---|---|---|---|
| TRINITY_DN17160_c0_g1_i1_len_540_303374.p1_type_internal_len_181 | XP_026302223.1UPF0235 protein C15orf40 homolog | | |
| TRINITY_DN10415_c0_g1_i1_len_537_203228.p1_type_internal_len_180 | PSN56738.1Rho-associated protein kinase 1 | F:GO:0004674; F:GO:0005524; P:GO:0006468; F:GO:0017048 | EC:2.7.11 |
| TRINITY_DN10744_c0_g1_i1_len_851_96634.p2_type_5prime_partial_len_179 | ---NA--- | | |
| TRINITY_DN34314_c0_g1_i1_len_724_65396.p1_type_complete_len_179 | XP_014300796.1 density-regulated protein homolog | | |
| TRINITY_DN34242_c0_g1_i1_len_539_203184.p1_type_internal_len_180 | ---NA--- | | |
| TRINITY_DN27479_c0_g1_i1_len_1068_195948.p1_type_complete_len_179 | ---NA--- | | |
| TRINITY_DN19704_c0_g1_i2_len_950_126872.p1_type_complete_len_179 | XP_026289154.1EEF1A lysine methyltransferase 2 | | |
| TRINITY_DN33597_c0_g1_i1_len_540_203158.p1_type_internal_len_180 | XP_026278399.1bifunctional heparan sulfate N-deacetylase/N-sulfotransferase | F:GO:0015016; F:GO:0016787 | EC:2.8.2.8 |
| TRINITY_DN17408_c0_g1_i1_len_546_131350.p1_type_5prime_partial_len_179 | ---NA--- | | |
| TRINITY_DN15341_c0_g1_i1_len_812_34693.p1_type_complete_len_179 | OWA53049.1putative Translocator protein | | |
| TRINITY_DN24090_c0_g3_i5_len_541_131465.p1_type_internal_len_180 | ---NA--- | | |
| TRINITY_DN24900_c0_g1_i1_len_827_198291.p2_type_complete_len_179 | ASK05861.1chitin-binding protein | F:GO:0042302 | |
| TRINITY_DN14268_c0_g1_i1_len_637_98879.p1_type_5prime_partial_len_179 | XP_018018916.1 glycerol-3-phosphate dehydrogenase [NAD(+)], cytoplasmic-like isoform X1 | F:GO:0004367; P:GO:0005975; C:GO:0009331; F:GO:0042803; P:GO:0046168; F:GO:0051287; P:GO:0055114 | EC:1.1.1.8; EC:1.1.1.94 |
| TRINITY_DN18259_c0_g1_i1_len_1554_192798.p2_type_complete_len_179 | ---NA--- | | |
| TRINITY_DN31713_c0_g1_i1_len_643_240072.p2_type_5prime_partial_len_179 | ---NA--- | | |

| | | | |
|---|---|---|---|
| TRINITY_DN25344_c0_g1_i1_len_2803_229272.p2_type_complete_len_179 | XP_018014697.1 serine/threonine-protein kinase PAK 3-like | | |
| TRINITY_DN17185_c0_g1_i1_len_1121_195518.p1_type_5prime_partial_len_179 | OXA64159.1Glycine cleavage system H protein, mitochondrial | C:GO:0005739; C:GO:0005960; P:GO:0019464 | |
| TRINITY_DN12354_c0_g1_i1_len_557_202712.p1_type_5prime_partial_len_179 | XP_014358796.1 ankyrin repeat and SAM domain-containing protein 1A-like | | |
| TRINITY_DN3201_c0_g1_i1_len_539_203170.p1_type_internal_len_180 | XP_013791847.1CUGBP Elav-like family member 2 isoform X2 | | |
| TRINITY_DN17110_c0_g1_i1_len_538_203192.p1_type_internal_len_180 | ---NA--- | | |
| TRINITY_DN30273_c0_g1_i1_len_540_68184.p1_type_internal_len_180 | ---NA--- | | |
| TRINITY_DN9367_c0_g1_i2_len_541_68175.p1_type_internal_len_180 | ---NA--- | | |
| TRINITY_DN18413_c0_g1_i3_len_541_68171.p1_type_internal_len_180 | ---NA--- | | |
| TRINITY_DN29411_c0_g1_i1_len_540_37929.p1_type_internal_len_180 | XP_018027086.1 semaphorin-1A-like | | |
| TRINITY_DN17448_c0_g1_i1_len_637_66532.p1_type_5prime_partial_len_179 | ACZ63470.1c-type lysozyme | F:GO:0003796 | EC:3.2.1.17 |
| TRINITY_DN21086_c0_g1_i1_len_635_66562.p1_type_5prime_partial_len_179 | ---NA--- | | |
| TRINITY_DN24528_c0_g1_i1_len_540_8034.p1_type_internal_len_180 | ---NA--- | | |
| TRINITY_DN9072_c0_g1_i1_len_862_237311.p1_type_complete_len_179 | EEB14421.1Centrin-1, putative | F:GO:0005509; F:GO:0032440; P:GO:0055114 | EC:1.3.1.74 |
| TRINITY_DN7367_c0_g1_i1_len_626_164576.p1_type_complete_len_179 | ---NA--- | | |
| TRINITY_DN37565_c0_g1_i1_len_540_203161.p1_type_internal_len_180 | ---NA--- | | |
| TRINITY_DN15016_c0_g1_i1_len_635_164454.p1_type_5prime_partial_len_179 | PFX13290.1Retrovirus-related Pol polyprotein | | |

| | | | |
|---|---|---|---|
| TRINITY_DN18975_c0_g1_i1_len_713_239010.p1_type_5prime_partial_len_179 | XP_015518156.1 UPF0235 protein C15orf40 homolog | | |
| TRINITY_DN33547_c0_g1_i1_len_624_201327.p1_type_5prime_partial_len_179 | ---NA--- | | |
| TRINITY_DN5391_c0_g1_i2_len_761_272145.p1_type_5prime_partial_len_179 | ---NA--- | | |
| TRINITY_DN36033_c0_g1_i1_len_539_203176.p1_type_internal_len_180 | XP_006614227.1 protein split ends-like | F:GO:0003723 | |
| TRINITY_DN29111_c0_g1_i1_len_541_241983.p1_type_internal_len_180 | AHH32890.1integrin beta 1 | P:GO:0007155; P:GO:0007229; C:GO:0016021 | |
| TRINITY_DN19677_c0_g1_i2_len_540_242008.p1_type_internal_len_180 | ---NA--- | | |
| TRINITY_DN37151_c0_g1_i1_len_540_242000.p2_type_internal_len_180 | ---NA--- | | |
| TRINITY_DN16388_c0_g1_i1_len_540_241998.p1_type_internal_len_180 | ---NA--- | | |
| TRINITY_DN3710_c0_g1_i1_len_540_241996.p1_type_internal_len_180 | XP_018012134.1 S1 RNA-binding domain-containing protein 1-like isoform X1 | | |
| TRINITY_DN21763_c0_g2_i1_len_1479_296833.p2_type_5prime_partial_len_179 | XP_023196747.1zinc finger protein OZF-like isoform X1 | | |
| TRINITY_DN29331_c0_g1_i1_len_540_275157.p1_type_internal_len_180 | ---NA--- | | |
| TRINITY_DN31767_c0_g1_i1_len_565_202544.p1_type_5prime_partial_len_179 | ---NA--- | | |
| TRINITY_DN26449_c0_g1_i1_len_537_242077.p1_type_internal_len_180 | XP_012219579.1 ankyrin repeat domain-containing protein 13C isoform X3 | | |
| TRINITY_DN16485_c1_g1_i1_len_540_166178.p1_type_internal_len_180 | ---NA--- | | |
| TRINITY_DN32710_c0_g1_i1_len_684_239440.p1_type_5prime_partial_len_179 | ---NA--- | | |
| TRINITY_DN35778_c0_g1_i1_len_538_203207.p1_type_internal_len_180 | ---NA--- | | |

| | | | |
|---|---|---|---|
| TRINITY_DN30805_c0_g1_i1_len_538_242041.p1_type_internal_len_180 | XP_022292572.1uncharacterized protein LOC111103527 | | |
| TRINITY_DN15746_c0_g1_i1_len_539_242029.p1_type_internal_len_180 | ---NA--- | | |
| TRINITY_DN7410_c0_g1_i1_len_539_242027.p2_type_internal_len_180 | XP_018015017.1 follistatin-like | | |
| TRINITY_DN15746_c0_g1_i3_len_833_64288.p1_type_5prime_partial_len_179 | ---NA--- | | |
| TRINITY_DN29089_c0_g1_i1_len_785_238120.p1_type_5prime_partial_len_179 | XP_018007157.1 protein lethal(2)essential for life-like | | |
| TRINITY_DN18673_c0_g1_i1_len_799_97045.p1_type_5prime_partial_len_179 | ---NA--- | | |
| TRINITY_DN1454_c0_g1_i1_len_540_166166.p1_type_internal_len_180 | ---NA--- | | |
| TRINITY_DN17475_c0_g1_i2_len_579_274490.p1_type_5prime_partial_len_179 | ---NA--- | | |
| TRINITY_DN26361_c1_g1_i1_len_1116_235267.p1_type_5prime_partial_len_179 | ---NA--- | | |
| TRINITY_DN7580_c0_g1_i1_len_541_100431.p1_type_internal_len_180 | ---NA--- | | |
| TRINITY_DN4436_c0_g1_i1_len_573_202358.p1_type_3prime_partial_len_180 | XP_015918744.1sex peptide receptor-like | | |
| TRINITY_DN18502_c0_g1_i1_len_602_99383.p1_type_3prime_partial_len_180 | XP_023234825.1ADP-ribosylation factor GTPase-activating protein 1-like isoform X3 | | |
| TRINITY_DN12210_c0_g1_i1_len_538_275196.p1_type_internal_len_180 | XP_018006378.1 glyoxylate/hydroxypyruvate reductase A-like isoform X1 | F:GO:0051287; P:GO:0055114 | |
| TRINITY_DN21477_c0_g1_i1_len_2515_58572.p2_type_complete_len_178 | KPI99065.1Mitochondrial inner membrane protease subunit 2 | P:GO:0006465; P:GO:0006627; F:GO:0008236; C:GO:0042720 | |
| TRINITY_DN32967_c0_g1_i1_len_537_242067.p1_type_internal_len_179 | PNF23587.1hypothetical protein B7P43_G04956 | C:GO:0016020; C:GO:0016021 | |

| | | | |
|---|---|---|---|
| TRINITY_DN13638_c0_g1_i1_len_741_238636.p1_type_3prime_partial_len_179 | XP_018014706.1 histone acetyltransferase KAT5-like isoform X2 | | |
| TRINITY_DN2081_c0_g1_i1_len_536_203258.p2_type_internal_len_179 | ---NA--- | | |
| TRINITY_DN10863_c0_g2_i1_len_537_242071.p1_type_internal_len_179 | XP_018010166.1 cuticle protein 7-like | F:GO:0042302 | |
| TRINITY_DN2056_c0_g1_i1_len_561_241544.p1_type_3prime_partial_len_179 | KZS19959.1Tyrosine-protein phosphatase non-receptor type 14 | F:GO:0004725; C:GO:0005856; P:GO:0035335 | EC:3.1.3.16; EC:3.1.3.48 |
| TRINITY_DN28725_c0_g1_i1_len_536_242105.p1_type_internal_len_179 | ---NA--- | | |
| TRINITY_DN29223_c0_g1_i1_len_536_242106.p1_type_internal_len_179 | XP_018011143.1 triple functional domain protein-like | F:GO:0005089; P:GO:0035023 | |
| TRINITY_DN17499_c0_g1_i1_len_638_240140.p1_type_complete_len_178 | XP_023334090.1mpv17-like protein | | |
| TRINITY_DN35319_c0_g1_i1_len_536_242107.p1_type_internal_len_179 | XP_018020695.1 zinc finger protein 813-like | | |
| TRINITY_DN19563_c0_g1_i3_len_536_242112.p1_type_internal_len_179 | ---NA--- | | |
| TRINITY_DN31033_c0_g1_i1_len_536_242114.p1_type_internal_len_179 | ---NA--- | | |
| TRINITY_DN4474_c0_g1_i1_len_536_275256.p1_type_internal_len_179 | XP_018018978.1PREDICTED: uncharacterized protein LOC108675465 | | |
| TRINITY_DN1540_c0_g1_i1_len_536_203262.p1_type_internal_len_179 | XP_021914140.1sushi, von Willebrand factor type A, EGF and pentraxin domain-containing protein 1 isoform X3 | | |
| TRINITY_DN4562_c0_g1_i1_len_922_299279.p1_type_complete_len_178 | ---NA--- | | |
| TRINITY_DN6127_c0_g1_i1_len_537_242089.p1_type_internal_len_179 | ---NA--- | | |
| TRINITY_DN28483_c0_g1_i4_len_805_198535.p1_type_complete_len_178 | ---NA--- | | |
| TRINITY_DN10057_c0_g1_i1_len_536_242095.p1_type_internal_len_179 | XP_018012955.1 inhibin beta chain-like | | |

| | | | |
|---|---|---|---|
| TRINITY_DN16901_c0_g1_i2_len_783_162578.p1_type_complete_len_178 | XP_018019761.1 flocculation protein FLO11-like | | |
| TRINITY_DN23343_c0_g2_i1_len_536_242103.p1_type_internal_len_179 | ---NA--- | | |
| TRINITY_DN37036_c0_g1_i1_len_536_203251.p1_type_internal_len_179 | PSN56526.1MAGUK p55 subfamily member 7 | | |
| TRINITY_DN31479_c0_g1_i1_len_536_203247.p1_type_internal_len_179 | XP_018018055.1 serine-rich adhesin for platelets-like | F:GO:0003677; C:GO:0005634 | |
| TRINITY_DN19288_c0_g1_i1_len_731_238748.p1_type_5prime_partial_len_178 | PFX13703.1Transposon Ty3-I Gag-Pol polyprotein | F:GO:0003676; F:GO:0008270; P:GO:0015074; C:GO:0016020; C:GO:0016021 | |
| TRINITY_DN6838_c0_g1_i1_len_594_201895.p1_type_3prime_partial_len_179 | XP_008192104.1 UHRF1-binding protein 1-like isoform X2 | | |
| TRINITY_DN24883_c0_g1_i1_len_594_201885.p1_type_5prime_partial_len_178 | ---NA--- | | |
| TRINITY_DN21310_c0_g1_i1_len_612_66888.p1_type_5prime_partial_len_178 | XP_018022120.1 cuticle protein 19-like | F:GO:0042302 | |
| TRINITY_DN13798_c0_g1_i1_len_536_203253.p1_type_internal_len_179 | ---NA--- | | |
| TRINITY_DN20237_c0_g1_i1_len_2316_480.p1_type_3prime_partial_len_179 | XP_018006663.1 prefoldin subunit 2-like | C:GO:0005737; P:GO:0006457; C:GO:0016272; F:GO:0044183; F:GO:0051082 | |
| TRINITY_DN32520_c0_g1_i1_len_536_203241.p1_type_internal_len_179 | ---NA--- | | |
| TRINITY_DN3543_c0_g1_i1_len_538_242055.p1_type_internal_len_179 | XP_018577003.1sushi, von Willebrand factor type A, EGF and pentraxin domain-containing protein 1-like | F:GO:0005509; C:GO:0016021 | |
| TRINITY_DN26232_c0_g1_i4_len_563_202580.p1_type_3prime_partial_len_179 | XP_018027576.1 3-mercaptopyruvate sulfurtransferase-like | | |
| TRINITY_DN18939_c0_g1_i2_len_646_240035.p1_type_complete_len_178 | ---NA--- | | |
| TRINITY_DN347_c0_g1_i1_len_537_203211.p1_type_internal_len_179 | XP_012943573.1 DNA-dependent protein kinase catalytic subunit-like | | |

| | | | |
|---|---|---|---|
| TRINITY_DN23860_c0_g1_i1_len_856_237367.p1_type_3prime_partial_len_179 | XP_018025251.1 inositol-trisphosphate 3-kinase A-like isoform X3 | | |
| TRINITY_DN38734_c0_g1_i1_len_537_203214.p1_type_internal_len_179 | XP_011452872.1 synaptotagmin-15 isoform X4 | C:GO:0016020 | |
| TRINITY_DN3988_c0_g1_i1_len_537_275218.p1_type_internal_len_179 | XP_020555803.2oocyte zinc finger protein XlCOF22-like | C:GO:0005634; P:GO:0050789 | |
| 1572\|_\|c5165_f3p2_2876_isoform_c5165_full_length_coverage_3_non_full_length_coverage_2_isoform_length_2876_12448 | ASC55678.1putative trypsin serine protease | F:GO:0004252; P:GO:0006508; F:GO:0008233 | |
| 1573\|_\|c1637_f2p3_2875_isoform_c1637_full_length_coverage_2_non_full_length_coverage_3_isoform_length_2875_25994 | AHX84170.1target of rapamycin | F:GO:0004674; F:GO:0005524; P:GO:0006468; F:GO:0044877 | EC:2.7.11 |
| 1574\|_\|c11370_f2p1_2874_isoform_c11370_full_length_coverage_2_non_full_length_coverage_1_isoform_length_2874_22051 | ---NA--- | | |
| 1575\|_\|c1365_f12p4_2874_isoform_c1365_full_length_coverage_12_non_full_length_coverage_4_isoform_length_2874_15843 | XP_013777764.2zinc transporter 2-like | P:GO:0006812; F:GO:0008324; C:GO:0016020; C:GO:0016021; P:GO:0055085; P:GO:0098655 | |
| TRINITY_DN26356_c1_g1_i1_len_3258_228701.p1_type_5prime_partial_len_958 | AVN99023.1sortilin-related receptor-like | C:GO:0016020 | |
| 1577\|_\|c17891_f2p2_2873_isoform_c17891_full_length_coverage_2_non_full_length_coverage_2_isoform_length_2873_22544 | ADK63101.1thrombospondin protein | F:GO:0005509; C:GO:0005576; P:GO:0006030; P:GO:0007155; F:GO:0008061 | |
| 1581\|_\|c2035_f2p0_2872_isoform_c2035_full_length_coverage_2_non_full_length_coverage_0_isoform_length_2872_28170 | ---NA--- | | |
| TRINITY_DN22658_c0_g1_i1_len_3679_228353.p1_type_5prime_partial_len_957 | KZS05039.1putative 7SK snRNA methylphosphate capping enzyme | F:GO:0008168; P:GO:0032259 | |
| 1585\|_\|c9421_f3p0_2871_isoform_c9421_full_length_coverage_3_non_full_length_coverage_0_isoform_length_2871_25973 | ---NA--- | | |

| | | | |
|---|---|---|---|
| 1586\|_\|c2835_f4p3_2871_isoform_c2835_full_length_coverage_4_non_full_length_coverage_3_isoform_length_2871_16713 | XP_019877060.1 protein CASC3 | | |
| TRINITY_DN10657_c0_g1_i1_len_663_98526.p1_type_5prime_partial_len_178 | ---NA--- | | |
| TRINITY_DN222_c0_g1_i1_len_537_203230.p1_type_internal_len_179 | ---NA--- | | |
| TRINITY_DN19353_c0_g1_i1_len_1724_156947.p1_type_5prime_partial_len_178 | ---NA--- | | |
| TRINITY_DN24014_c0_g1_i4_len_1070_195935.p1_type_complete_len_178 | ---NA--- | | |
| TRINITY_DN1764_c0_g1_i1_len_534_203330.p1_type_internal_len_179 | XP_018025307.1 mucin-17-like | C:GO:0016021 | |
| TRINITY_DN5679_c0_g1_i1_len_535_203286.p1_type_internal_len_179 | AAV48573.15-HT1 receptor | F:GO:0004930; P:GO:0007186; C:GO:0016021 | |
| TRINITY_DN16995_c0_g1_i1_len_588_165233.p1_type_5prime_partial_len_178 | ---NA--- | | |
| TRINITY_DN7778_c0_g1_i1_len_535_203284.p1_type_internal_len_179 | ---NA--- | | |
| TRINITY_DN21288_c1_g1_i3_len_951_236509.p1_type_5prime_partial_len_178 | ---NA--- | | |
| TRINITY_DN31521_c0_g1_i1_len_535_203288.p1_type_internal_len_179 | XP_023326158.1cartilage oligomeric matrix protein-like | | |
| TRINITY_DN7730_c0_g1_i1_len_538_131519.p1_type_internal_len_179 | CDF32001.1serine protease | F:GO:0004252; P:GO:0006508 | EC:3.4.21 |
| TRINITY_DN13919_c0_g1_i1_len_538_131514.p2_type_internal_len_179 | XP_018015976.1 zinc finger protein 236-like isoform X4 | | |
| TRINITY_DN17196_c0_g1_i1_len_602_201728.p1_type_5prime_partial_len_178 | ---NA--- | | |
| TRINITY_DN29980_c0_g1_i1_len_535_131575.p1_type_internal_len_179 | XP_018019102.1 zinc finger protein squeeze-like | | |
| TRINITY_DN18313_c0_g1_i1_len_537_131548.p1_type_internal_len_179 | ---NA--- | | |

| | | | |
|---|---|---|---|
| TRINITY_DN5817_c0_g1_i1_len_538_203202.p1_type_internal_len_179 | ---NA--- | | |
| TRINITY_DN16633_c0_g1_i1_len_537_131530.p1_type_internal_len_179 | ---NA--- | | |
| TRINITY_DN5273_c0_g1_i1_len_534_203307.p1_type_internal_len_179 | XP_021928026.1uncharacterized protein LOC110833839 isoform X1 | | |
| TRINITY_DN17242_c0_g1_i1_len_535_203299.p1_type_internal_len_179 | XP_018017090.1 GPI mannosyltransferase 4-like | C:GO:0005783; C:GO:0005789; C:GO:0016020; C:GO:0016021; F:GO:0016757 | |
| TRINITY_DN23269_c0_g1_i1_len_1189_61991.p1_type_complete_len_178 | GBC47303.1retrotransposable element | | |
| TRINITY_DN37306_c0_g1_i1_len_710_239037.p1_type_5prime_partial_len_178 | ---NA--- | | |
| TRINITY_DN14654_c0_g1_i1_len_536_203266.p1_type_5prime_partial_len_178 | ---NA--- | | |
| TRINITY_DN28853_c0_g1_i1_len_537_166226.p1_type_internal_len_179 | ---NA--- | | |
| TRINITY_DN21034_c0_g1_i1_len_654_66275.p1_type_5prime_partial_len_178 | ---NA--- | | |
| TRINITY_DN39416_c0_g1_i1_len_537_203220.p1_type_internal_len_179 | ---NA--- | | |
| TRINITY_DN11762_c0_g1_i1_len_536_203268.p1_type_internal_len_179 | AXR98466.1fibrillin 2 | F:GO:0005201; F:GO:0005509; C:GO:0062023 | |
| TRINITY_DN14704_c0_g1_i2_len_636_164435.p1_type_5prime_partial_len_178 | XP_018016706.1 serine-rich adhesin for platelets-like | P:GO:0006796; F:GO:0016740 | |
| TRINITY_DN23885_c0_g1_i1_len_536_166251.p1_type_5prime_partial_len_178 | XP_018011578.1 UDP-glucuronosyltransferase 2B14-like | | |
| TRINITY_DN20137_c0_g1_i1_len_658_200656.p1_type_5prime_partial_len_178 | ---NA--- | | |
| TRINITY_DN16013_c0_g1_i2_len_817_64431.p1_type_5prime_partial_len_178 | ---NA--- | | |
| TRINITY_DN26215_c0_g1_i1_len_4758_227855.p2_type_5prime_partial_len_178 | ---NA--- | | |

| | | | |
|---|---|---|---|
| TRINITY_DN8596_c0_g1_i1_len_537_203226.p1_type_3prime_partial_len_179 | XP_008553148.1 sodium- and chloride-dependent glycine transporter 1-like | | |
| TRINITY_DN10815_c0_g1_i1_len_903_197454.p1_type_5prime_partial_len_178 | PNF25980.1hypothetical protein B7P43_G06365 | P:GO:0006355 | |
| TRINITY_DN22861_c0_g1_i2_len_536_68273.p2_type_internal_len_179 | ---NA--- | | |
| TRINITY_DN14723_c0_g1_i1_len_536_8107.p1_type_internal_len_179 | XP_018020703.1 ATP-dependent DNA helicase Q1-like | | |
| TRINITY_DN21358_c0_g1_i1_len_535_68298.p1_type_internal_len_179 | ---NA--- | | |
| TRINITY_DN13788_c0_g1_i3_len_721_272575.p1_type_5prime_partial_len_178 | XP_008555613.1 protein Malvolio isoform X2 | | |
| TRINITY_DN31321_c0_g1_i1_len_537_203221.p1_type_internal_len_179 | ---NA--- | | |
| TRINITY_DN24817_c0_g1_i4_len_2813_58310.p2_type_3prime_partial_len_179 | ---NA--- | | |
| TRINITY_DN6664_c0_g1_i1_len_720_128774.p1_type_3prime_partial_len_179 | XP_018022654.1 putative fatty acyl-CoA reductase CG5065 isoform X2 | | |
| TRINITY_DN17150_c0_g1_i1_len_1801_231743.p2_type_5prime_partial_len_178 | ---NA--- | | |
| TRINITY_DN39421_c0_g1_i1_len_728_199610.p1_type_5prime_partial_len_178 | XP_021962643.1glutathione synthetase-like isoform X3 | | |
| TRINITY_DN14400_c0_g1_i1_len_707_128950.p1_type_complete_len_178 | ---NA--- | | |
| TRINITY_DN22256_c0_g1_i1_len_553_100239.p1_type_5prime_partial_len_178 | ---NA--- | | |
| TRINITY_DN22673_c0_g1_i1_len_1479_193206.p2_type_complete_len_178 | XP_013197529.1 splicing factor U2af 38 kDa subunit | P:GO:0000398; F:GO:0003723; F:GO:0046872; C:GO:0089701 | |
| TRINITY_DN20147_c0_g1_i2_len_940_95995.p1_type_complete_len_178 | AUB13319.1C-type lectin containing low-density lipoprotein receptor domain | F:GO:0030246 | |
| TRINITY_DN6051_c0_g1_i1_len_534_242147.p1_type_internal_len_178 | ---NA--- | | |

| | | | |
|---|---|---|---|
| TRINITY_DN1658_c0_g1_i1_len_532_242207.p1_type_internal_len_178 | XP_022203406.1protocadherin Fat 4-like | C:GO:0016020 | |
| TRINITY_DN3001_c0_g1_i1_len_532_242213.p1_type_internal_len_178 | XP_018010512.1 putative neutral sphingomyelinase | C:GO:0016020 | |
| TRINITY_DN32771_c0_g1_i1_len_534_242142.p1_type_internal_len_178 | ---NA--- | | |
| TRINITY_DN387_c0_g1_i1_len_532_242204.p1_type_internal_len_178 | ---NA--- | | |
| TRINITY_DN1275_c0_g1_i1_len_532_242199.p1_type_internal_len_178 | XP_018013858.1 alkylglycerol monooxygenase-like | F:GO:0004497; F:GO:0005506; P:GO:0008610; C:GO:0016020; C:GO:0016021; F:GO:0016491; P:GO:0055114 | |
| TRINITY_DN6063_c0_g1_i1_len_532_242193.p1_type_internal_len_178 | ---NA--- | | |
| TRINITY_DN25077_c0_g1_i13_len_533_242177.p1_type_internal_len_178 | ACL98140.1ubiquitin C variant | | |
| TRINITY_DN2857_c0_g1_i1_len_533_242175.p1_type_internal_len_178 | AUM86164.15'-3' exoribonuclease 1 | F:GO:0003824; P:GO:0090304 | |
| TRINITY_DN12754_c0_g1_i1_len_534_131600.p1_type_internal_len_178 | ASA69502.1penlectin 5-2 | | |
| TRINITY_DN12729_c0_g1_i2_len_531_38075.p1_type_internal_len_178 | ---NA--- | | |
| TRINITY_DN8471_c0_g1_i1_len_733_5405.p1_type_5prime_partial_len_177 | XP_018008851.1 collagen alpha-1(V) chain-like isoform X3 | | |
| TRINITY_DN29883_c0_g1_i1_len_872_127451.p1_type_complete_len_177 | XP_018023849.1PREDICTED: uncharacterized protein LOC108679670 | | |
| TRINITY_DN39312_c0_g1_i1_len_670_200472.p1_type_3prime_partial_len_178 | ---NA--- | | |
| TRINITY_DN4097_c0_g1_i1_len_534_203336.p2_type_internal_len_178 | ---NA--- | | |
| TRINITY_DN19344_c0_g1_i1_len_1355_233745.p3_type_complete_len_177 | ---NA--- | | |

| | | | |
|---|---|---|---|
| TRINITY_DN23114_c0_g1_i2_len_1628_232421.p2_type_5prime_partial_len_177 | ---NA--- | | |
| TRINITY_DN22824_c0_g1_i1_len_2793_58323.p2_type_complete_len_177 | ---NA--- | | |
| TRINITY_DN3594_c0_g1_i1_len_534_203329.p1_type_internal_len_178 | XP_018012552.1 ionotropic receptor 21a-like | | |
| TRINITY_DN9199_c0_g1_i1_len_534_203322.p1_type_internal_len_178 | KZS07542.1Carbohydrate sulfotransferase 5 | F:GO:0001517; P:GO:0006044; P:GO:0006790; F:GO:0016740 | |
| TRINITY_DN13361_c0_g1_i1_len_534_203320.p1_type_internal_len_178 | XP_007895573.1 geranylgeranyl transferase type-1 subunit beta | F:GO:0003824 | |
| TRINITY_DN9093_c0_g1_i1_len_533_68345.p1_type_internal_len_178 | XP_018016582.1 zinc finger and BTB domain-containing protein 44-like isoform X2 | | |
| TRINITY_DN24576_c0_g1_i7_len_533_68346.p1_type_internal_len_178 | XP_010293819.1 polyubiquitin-B isoform X15 | | |
| TRINITY_DN12479_c0_g1_i2_len_532_68365.p1_type_internal_len_178 | XP_018015354.1PREDICTED: uncharacterized protein LOC108672228 | | |
| TRINITY_DN19714_c1_g1_i1_len_743_97604.p2_type_5prime_partial_len_177 | ---NA--- | | |
| TRINITY_DN38796_c0_g1_i1_len_534_203339.p1_type_internal_len_178 | XP_018027727.1PREDICTED: uncharacterized protein LOC108682971 | | |
| TRINITY_DN7982_c0_g1_i1_len_535_203275.p1_type_internal_len_178 | XP_018023216.1PREDICTED: uncharacterized protein LOC108679145 | | |
| TRINITY_DN18763_c0_g1_i1_len_608_201605.p1_type_5prime_partial_len_177 | BAO96441.1prophenoloxidase beta | F:GO:0016491; F:GO:0046872; P:GO:0055114 | |
| TRINITY_DN30056_c0_g1_i1_len_2980_188571.p1_type_3prime_partial_len_178 | ---NA--- | | |
| TRINITY_DN13699_c0_g1_i1_len_535_203290.p1_type_internal_len_178 | ---NA--- | | |
| TRINITY_DN21589_c0_g1_i1_len_2541_91445.p4_type_5prime_partial_len_177 | ARS88258.1ATP binding cassette transporter 6 | F:GO:0005524; C:GO:0016021; F:GO:0016887; P:GO:0055085 | EC:3.6.1.3; EC:3.6.1.15 |
| TRINITY_DN29182_c0_g1_i1_len_589_302524.p1_type_5prime_partial_len_177 | XP_017753094.1 protein pinocchio | | |

| | | | |
|---|---|---|---|
| TRINITY_DN22947_c0_g1_i2_len_722_128759.p1_type_5prime_partial_len_177 | ---NA--- | | |
| TRINITY_DN36_c0_g1_i1_len_584_7236.p1_type_5prime_partial_len_177 | ---NA--- | | |
| TRINITY_DN14428_c0_g1_i1_len_534_100576.p1_type_internal_len_178 | XP_023325705.1zinc metalloproteinase nas-4-like | | |
| TRINITY_DN15555_c0_g1_i1_len_673_163893.p1_type_5prime_partial_len_177 | XP_008551618.1 glyoxylate/hydroxypyruvate reductase A-like | | |
| TRINITY_DN24812_c0_g1_i1_len_1947_191102.p1_type_5prime_partial_len_177 | XP_018017286.1 mitochondrial pyruvate carrier 1-like | C:GO:0005743; P:GO:0006850 | |
| TRINITY_DN9962_c0_g1_i1_len_686_163734.p2_type_5prime_partial_len_177 | ---NA--- | | |
| TRINITY_DN24153_c0_g1_i2_len_814_198451.p1_type_5prime_partial_len_177 | ---NA--- | | |
| TRINITY_DN13160_c0_g1_i1_len_533_275326.p1_type_internal_len_178 | ---NA--- | | |
| TRINITY_DN18977_c0_g1_i1_len_743_199377.p1_type_complete_len_177 | XP_018019395.1 transmembrane protein 222-like | C:GO:0016021 | |
| TRINITY_DN15736_c0_g1_i1_len_775_272014.p1_type_5prime_partial_len_177 | XP_013161685.1 mitochondrial import inner membrane translocase subunit Tim8 | C:GO:0005758; F:GO:0046872; P:GO:0072321 | |
| TRINITY_DN27549_c0_g1_i1_len_533_166324.p1_type_internal_len_178 | PNF22538.1hypothetical protein B7P43_G13953 | F:GO:0003677; F:GO:0003700; P:GO:0006355 | |
| TRINITY_DN7991_c0_g1_i1_len_533_166311.p1_type_internal_len_178 | ---NA--- | | |
| TRINITY_DN29345_c0_g1_i1_len_531_275351.p1_type_internal_len_178 | ---NA--- | | |
| TRINITY_DN9213_c0_g1_i2_len_532_131645.p1_type_internal_len_178 | ---NA--- | | |
| TRINITY_DN5399_c0_g1_i1_len_675_239567.p1_type_5prime_partial_len_177 | ---NA--- | | |
| TRINITY_DN9527_c0_g1_i1_len_535_8120.p1_type_internal_len_178 | ---NA--- | | |

| | | | |
|---|---|---|---|
| TRINITY_DN27199_c0_g1_i1_len_651_239939.p1_type_5prime_partial_len_177 | XP_018017336.1 major facilitator superfamily domain-containing protein 8-like | | |
| TRINITY_DN33321_c0_g1_i1_len_593_240934.p1_type_5prime_partial_len_177 | XP_018023492.1 lateral signaling target protein 2 homolog | | |
| TRINITY_DN32591_c0_g1_i1_len_596_240881.p1_type_5prime_partial_len_177 | ---NA--- | | |
| TRINITY_DN18669_c0_g1_i1_len_832_162091.p1_type_5prime_partial_len_177 | ---NA--- | | |
| TRINITY_DN15146_c0_g1_i1_len_533_8158.p1_type_internal_len_178 | XP_018011640.1 purine nucleoside phosphorylase-like | F:GO:0004731; C:GO:0005737; P:GO:0009116 | EC:2.4.2.1 |
| TRINITY_DN22365_c1_g1_i2_len_3142_122048.p2_type_5prime_partial_len_177 | ---NA--- | | |
| 1588\|_\|c46935_f2p2_2870_isoform_c46935_full_length_coverage_2_non_full_length_coverage_2_isoform_length_2870_19337 | XP_018014495.1 TBC1 domain family member 23-like | C:GO:0005829; P:GO:0042147 | |
| 1589\|_\|c36225_f2p0_2870_isoform_c36225_full_length_coverage_2_non_full_length_coverage_0_isoform_length_2870_18925 | ---NA--- | | |
| 1590\|_\|c2708_f3p0_2869_isoform_c2708_full_length_coverage_3_non_full_length_coverage_0_isoform_length_2869_21168 | XP_023707059.1scaffold attachment factor B2 isoform X3 | F:GO:0003723 | |
| TRINITY_DN20187_c0_g1_i1_len_3853_228261.p1_type_5prime_partial_len_956 | XP_018026392.1 ankyrin repeat domain-containing protein 17-like isoform X1 | | |
| TRINITY_DN20713_c0_g1_i1_len_4580_227916.p1_type_complete_len_956 | XP_023210904.1inactive tyrosine-protein kinase transmembrane receptor ROR1-like isoform X1 | | |
| 1595\|_\|c27050_f2p2_2868_isoform_c27050_full_length_coverage_2_non_full_length_coverage_2_isoform_length_2868_18415 | KDR24355.1Retinol dehydrogenase 11 | | |
| 1596\|_\|c17979_f2p1_2868_isoform_c17979_full_length_coverage_2_non_full_length_coverage_1_isoform_length_2868_22605 | XP_018027708.1PREDICTED: uncharacterized protein LOC108682952 | | |

| | | | |
|---|---|---|---|
| 1597\|_\|c2782_f2p1_2868_isoform_c2782_full_length_coverage_2_non_full_length_coverage_1_isoform_length_2868_12156 | ---NA--- | | |
| TRINITY_DN24053_c0_g1_i2_len_3064_228922.p1_type_complete_len_956 | XP_023718092.1probable ATP-dependent RNA helicase DDX20 | F:GO:0097159; F:GO:1901363 | |
| 1600\|_\|c2756_f5p2_2867_isoform_c2756_full_length_coverage_5_non_full_length_coverage_2_isoform_length_2867_21209 | XP_019626500.1  DNA polymerase lambda-like | F:GO:0003677; F:GO:0003887; C:GO:0005634; P:GO:0006281; F:GO:0016779; F:GO:0034061; F:GO:0046872; P:GO:0071897 | |
| TRINITY_DN13216_c0_g1_i1_len_532_203369.p1_type_internal_len_178 | ---NA--- | | |
| TRINITY_DN15817_c0_g1_i1_len_533_203352.p1_type_internal_len_178 | XP_018011840.1 long-chain-fatty-acid--CoA ligase 5-like | P:GO:0001676; F:GO:0004467; F:GO:0102391 | EC:6.2.1.3 |
| TRINITY_DN21207_c0_g1_i3_len_691_200133.p1_type_3prime_partial_len_178 | BAH85843.1pigment dispersing hormone receptor | F:GO:0004930; P:GO:0007166; P:GO:0007186; C:GO:0016021 | |
| TRINITY_DN16654_c0_g1_i3_len_616_130116.p1_type_3prime_partial_len_178 | XP_018013412.1 solute carrier family 12 member 6-like | | |
| TRINITY_DN11378_c0_g1_i1_len_533_68334.p1_type_internal_len_178 | ---NA--- | | |
| TRINITY_DN2443_c0_g1_i1_len_532_203371.p1_type_internal_len_178 | ---NA--- | | |
| TRINITY_DN21744_c0_g1_i1_len_533_203358.p1_type_internal_len_178 | ---NA--- | | |
| TRINITY_DN18815_c0_g1_i2_len_2097_30224.p2_type_complete_len_176 | ---NA--- | | |
| TRINITY_DN21971_c0_g1_i4_len_1456_1788.p2_type_complete_len_176 | ---NA--- | | |
| TRINITY_DN16943_c0_g1_i3_len_531_38077.p1_type_internal_len_177 | ---NA--- | | |
| TRINITY_DN20056_c0_g1_i2_len_1305_297422.p2_type_5prime_partial_len_176 | XP_018006981.1 5'-nucleotidase-like | F:GO:0000166; P:GO:0009166; F:GO:0016787 | |
| TRINITY_DN16548_c0_g1_i1_len_533_100588.p1_type_5prime_partial_len_176 | ---NA--- | | |

| | | | |
|---|---|---|---|
| TRINITY_DN17440_c0_g1_i1_len_688_200181.p1_type_complete_len_176 | XP_014676379.1 28S ribosomal protein S23, mitochondrial-like isoform X1 | F:GO:0003735; C:GO:0005840; P:GO:0006412 | |
| TRINITY_DN6207_c0_g1_i1_len_529_166408.p1_type_internal_len_177 | XP_018011840.1 long-chain-fatty-acid--CoA ligase 5-like | P:GO:0001676; F:GO:0004467; F:GO:0102391 | EC:6.2.1.3 |
| TRINITY_DN26890_c0_g1_i1_len_916_197321.p1_type_complete_len_176 | XP_010785632.1 EKC/KEOPS complex subunit TPRKB | C:GO:0005634 | |
| TRINITY_DN16267_c0_g1_i1_len_573_165472.p1_type_5prime_partial_len_176 | ---NA--- | | |
| TRINITY_DN8582_c0_g1_i1_len_531_203382.p1_type_internal_len_177 | ---NA--- | | |
| TRINITY_DN21958_c0_g1_i1_len_662_129467.p1_type_complete_len_176 | ---NA--- | | |
| TRINITY_DN19947_c0_g1_i3_len_796_162452.p1_type_complete_len_176 | ---NA--- | | |
| TRINITY_DN22541_c0_g1_i1_len_1320_233934.p1_type_complete_len_176 | XP_018007688.1 transmembrane protein 53-like | | |
| TRINITY_DN20960_c0_g1_i1_len_692_98172.p2_type_complete_len_176 | ---NA--- | | |
| TRINITY_DN26022_c0_g1_i2_len_4180_228097.p3_type_3prime_partial_len_177 | ---NA--- | | |
| TRINITY_DN18125_c0_g1_i1_len_2471_265495.p2_type_complete_len_176 | XP_018026458.1 mitochondrial inner membrane protease subunit 2-like isoform X1 | P:GO:0006465; P:GO:0006627; F:GO:0008236; C:GO:0042720 | |
| TRINITY_DN30979_c0_g1_i1_len_530_166381.p1_type_internal_len_177 | XP_015114293.1 sphingomyelin phosphodiesterase-like isoform X2 | | |
| TRINITY_DN17973_c0_g1_i1_len_550_241766.p1_type_5prime_partial_len_176 | ---NA--- | | |
| TRINITY_DN11911_c0_g1_i1_len_651_200793.p1_type_3prime_partial_len_177 | XP_018013798.1 LIM domain only protein 3-like | | |
| TRINITY_DN19527_c0_g1_i1_len_884_34189.p2_type_5prime_partial_len_176 | ---NA--- | | |

| | | | |
|---|---|---|---|
| TRINITY_DN14516_c0_g1_i1_len_530_203423.p1_type_internal_len_177 | XP_018015944.1PREDICTED: uncharacterized protein LOC108672734 isoform X1 | | |
| TRINITY_DN12132_c0_g1_i1_len_529_242272.p1_type_internal_len_177 | XP_009559937.1 kinesin-like protein KIF27 | F:GO:0005488 | |
| TRINITY_DN15468_c0_g1_i1_len_679_98319.p1_type_5prime_partial_len_176 | ---NA--- | | |
| TRINITY_DN35773_c0_g1_i1_len_529_242287.p1_type_5prime_partial_len_176 | XP_018028171.1 semaphorin-1A-like | C:GO:0016020 | |
| TRINITY_DN10727_c0_g1_i1_len_529_242278.p1_type_internal_len_177 | XP_019641686.1 smoothened homolog | | |
| TRINITY_DN21199_c0_g1_i1_len_824_64364.p1_type_3prime_partial_len_177 | ---NA--- | | |
| TRINITY_DN23811_c0_g1_i2_len_3883_57843.p2_type_3prime_partial_len_177 | ATU31753.1protein singed-like | P:GO:0007015; F:GO:0030674; F:GO:0051015 | |
| TRINITY_DN2382_c0_g1_i1_len_530_242248.p1_type_internal_len_177 | ---NA--- | | |
| TRINITY_DN13363_c0_g1_i2_len_530_242251.p1_type_internal_len_177 | AUC64084.1G-protein-coupled receptor kinase 2 | F:GO:0004703; F:GO:0005524; P:GO:0006468; P:GO:0007165 | EC:2.7.11; EC:2.7.11.16 |
| TRINITY_DN19787_c0_g1_i1_len_530_242254.p1_type_internal_len_177 | ---NA--- | | |
| TRINITY_DN11266_c0_g1_i1_len_720_163280.p1_type_3prime_partial_len_177 | ---NA--- | | |
| TRINITY_DN28818_c0_g1_i1_len_531_242219.p1_type_internal_len_177 | ---NA--- | | |
| TRINITY_DN36757_c0_g1_i1_len_531_242227.p1_type_internal_len_177 | ---NA--- | | |
| TRINITY_DN10759_c0_g1_i1_len_530_242243.p1_type_internal_len_177 | XP_018018093.1 kinesin-like protein KIF13A isoform X1 | | |
| TRINITY_DN218_c0_g1_i1_len_530_242247.p1_type_internal_len_177 | XP_023705145.1dnaJ homolog subfamily C member 13 isoform X2 | | |
| TRINITY_DN16453_c0_g1_i1_len_604_164925.p1_type_5prime_partial_len_176 | ---NA--- | | |

| | | | |
|---|---|---|---|
| TRINITY_DN6900_c0_g1_i1_len_529_203441.p1_type_3prime_partial_len_177 | ---NA--- | | |
| TRINITY_DN31004_c0_g1_i1_len_668_239669.p1_type_3prime_partial_len_177 | XP_023243977.1protein phosphatase 1H-like | F:GO:0004721 | EC:3.1.3.16 |
| TRINITY_DN19680_c0_g1_i2_len_1437_60941.p2_type_3prime_partial_len_177 | XP_023992359.1gastrula zinc finger protein XlCGF71.1-like | F:GO:0003676 | |
| TRINITY_DN17403_c0_g1_i1_len_537_100523.p1_type_5prime_partial_len_176 | ---NA--- | | |
| TRINITY_DN2229_c0_g1_i1_len_672_129324.p1_type_5prime_partial_len_176 | XP_018903040.1 short coiled-coil protein B | | |
| TRINITY_DN17711_c0_g1_i1_len_591_302489.p1_type_5prime_partial_len_176 | ---NA--- | | |
| TRINITY_DN28311_c0_g1_i1_len_550_100285.p1_type_5prime_partial_len_176 | CDI59403.1Histone 1 | C:GO:0000786; F:GO:0003690; F:GO:0005515; C:GO:0005634; P:GO:0006334; P:GO:0016584; P:GO:0030261; F:GO:0031492; P:GO:0031936; P:GO:0045910 | |
| TRINITY_DN41969_c0_g1_i1_len_531_203389.p1_type_internal_len_177 | XP_022200755.1calcium-dependent secretion activator isoform X5 | | |
| TRINITY_DN31547_c0_g1_i1_len_530_203420.p1_type_internal_len_177 | XP_018022043.1 tyrosine-protein kinase Btk29A-like isoform X3 | | |
| TRINITY_DN16202_c0_g1_i1_len_529_203428.p1_type_internal_len_177 | GBC44980.1retrotransposable element | | |
| TRINITY_DN10265_c0_g1_i1_len_528_242299.p1_type_internal_len_177 | ---NA--- | | |
| TRINITY_DN30910_c0_g1_i1_len_634_301895.p1_type_3prime_partial_len_177 | ---NA--- | | |
| TRINITY_DN35343_c0_g1_i1_len_528_68455.p1_type_internal_len_177 | KFM81393.1Flap endonuclease GEN-like protein | F:GO:0003677; F:GO:0003824; F:GO:0004518; F:GO:0004519; P:GO:0006281; P:GO:0090305 | |
| TRINITY_DN13007_c0_g1_i1_len_528_68462.p1_type_internal_len_177 | AOD27418.1Notch | F:GO:0005509; P:GO:0007219; P:GO:0007275; C:GO:0016021; P:GO:0030154; P:GO:0050793 | |

| | | | |
|---|---|---|---|
| TRINITY_DN13087_c0_g1_i1_len_530_203416.p1_type_internal_len_177 | XP_018022973.1PREDICTED: uncharacterized protein LOC108678985 | | |
| TRINITY_DN21855_c0_g1_i2_len_1012_63053.p2_type_5prime_partial_len_176 | XP_019617654.1 cytosolic Fe-S cluster assembly factor NUBP2-like | C:GO:0005829; P:GO:0016226; F:GO:0051536 | |
| TRINITY_DN11847_c0_g1_i1_len_615_201477.p1_type_5prime_partial_len_176 | ---NA--- | | |
| TRINITY_DN8906_c0_g1_i1_len_531_275362.p1_type_internal_len_177 | ---NA--- | | |
| TRINITY_DN5408_c0_g1_i1_len_530_275375.p1_type_internal_len_177 | ---NA--- | | |
| TRINITY_DN17892_c0_g1_i2_len_530_68400.p2_type_internal_len_177 | ---NA--- | | |
| TRINITY_DN2255_c0_g1_i1_len_591_201961.p1_type_5prime_partial_len_176 | ---NA--- | | |
| TRINITY_DN29122_c0_g1_i1_len_530_68415.p1_type_internal_len_177 | KRX70196.1Retrovirus-related Pol polyprotein from transposon 17.6 | | |
| TRINITY_DN1659_c0_g1_i1_len_530_203414.p1_type_internal_len_177 | ---NA--- | | |
| TRINITY_DN16342_c0_g1_i2_len_592_67236.p1_type_5prime_partial_len_176 | ---NA--- | | |
| TRINITY_DN20095_c0_g1_i2_len_755_300551.p1_type_complete_len_176 | ALO17555.1Neuroparsin | | |
| TRINITY_DN20835_c0_g1_i2_len_1011_126477.p1_type_5prime_partial_len_176 | XP_023301147.1pyrimidodiazepine synthase | F:GO:0003824 | |
| TRINITY_DN21417_c0_g1_i1_len_1730_232025.p2_type_3prime_partial_len_177 | ---NA--- | | |
| TRINITY_DN12910_c0_g1_i2_len_530_38103.p1_type_internal_len_177 | BAH85843.1pigment dispersing hormone receptor | F:GO:0004930; P:GO:0007166; P:GO:0007186; C:GO:0016021 | |
| TRINITY_DN6718_c0_g1_i1_len_528_275418.p1_type_internal_len_177 | XP_018012650.1 fibrillin-2-like | F:GO:0005201; F:GO:0005509; C:GO:0062023 | |
| TRINITY_DN17983_c0_g1_i3_len_686_65848.p1_type_5prime_partial_len_176 | ---NA--- | | |

| | | | |
|---|---|---|---|
| TRINITY_DN14538_c0_g1_i1_len_681_65901.p1_type_5prime_partial_len_176 | XP_022099413.1piggyBac transposable element-derived protein 4-like | | |
| TRINITY_DN34493_c0_g1_i1_len_528_242316.p1_type_internal_len_176 | ---NA--- | | |
| TRINITY_DN37135_c0_g1_i1_len_525_242378.p1_type_internal_len_176 | XP_009807864.1 TATA element modulatory factor-like | | |
| TRINITY_DN8668_c0_g1_i1_len_527_242335.p1_type_internal_len_176 | XP_022253719.1uncharacterized protein LOC111088305 | | |
| TRINITY_DN10973_c0_g1_i1_len_526_203494.p1_type_internal_len_176 | XP_018013595.1 chloride channel protein 2-like isoform X4 | | |
| TRINITY_DN7520_c0_g1_i1_len_528_242301.p1_type_internal_len_176 | XP_008470299.1unconventional myosin-IXa-like | F:GO:0000166; P:GO:0007165 | |
| TRINITY_DN319_c0_g1_i1_len_529_242286.p1_type_internal_len_176 | XP_018025826.1 DNA-dependent protein kinase catalytic subunit-like | | |
| TRINITY_DN30908_c0_g1_i1_len_526_242353.p1_type_internal_len_176 | ---NA--- | | |
| TRINITY_DN1247_c0_g1_i1_len_527_242346.p2_type_internal_len_176 | ACU30154.1hypoxia inducible factor 1 alpha | F:GO:0003677; C:GO:0005634; P:GO:0006355; F:GO:0046983 | |
| TRINITY_DN21190_c0_g1_i1_len_906_161443.p1_type_5prime_partial_len_175 | ---NA--- | | |
| TRINITY_DN10510_c0_g1_i1_len_527_242350.p1_type_internal_len_176 | ---NA--- | | |
| TRINITY_DN30947_c0_g1_i2_len_3319_188115.p2_type_complete_len_175 | ACD74843.1trehalose-6-phosphate synthase | F:GO:0003825; P:GO:0005992 | EC:2.4.1.15 |
| TRINITY_DN34351_c0_g1_i1_len_527_242340.p1_type_internal_len_176 | ---NA--- | | |
| TRINITY_DN35741_c0_g1_i1_len_529_242274.p1_type_internal_len_176 | XP_018024206.1 protein O-mannosyl-transferase 2-like | | |
| TRINITY_DN29291_c0_g1_i1_len_664_301541.p1_type_5prime_partial_len_175 | ---NA--- | | |
| TRINITY_DN12842_c0_g1_i1_len_526_242358.p1_type_internal_len_176 | ---NA--- | | |

| | | | |
|---|---|---|---|
| TRINITY_DN17768_c0_g1_i2_len_609_240642.p1_type_complete_len_175 | KXJ10292.1putative uncharacterized transposon-derived protein F52C9.6 | | |
| TRINITY_DN3843_c0_g1_i1_len_525_100758.p1_type_internal_len_176 | ---NA--- | | |
| TRINITY_DN17238_c0_g1_i2_len_525_100759.p1_type_internal_len_176 | XP_018020252.1 RNA-directed DNA polymerase from mobile element jockey-like | F:GO:0003676; F:GO:0003964; F:GO:0004523; P:GO:0006278; P:GO:0090502 | |
| TRINITY_DN333_c0_g1_i1_len_526_303642.p1_type_internal_len_176 | ---NA--- | | |
| TRINITY_DN11818_c0_g1_i1_len_526_203497.p1_type_internal_len_176 | CBN80961.1Retrovirus polyprotein | F:GO:0003676; P:GO:0015074 | |
| TRINITY_DN4297_c0_g1_i1_len_1164_159551.p2_type_complete_len_175 | XP_018008334.1 suppressor protein SRP40-like | | |
| TRINITY_DN32510_c0_g1_i1_len_531_203377.p1_type_5prime_partial_len_175 | ---NA--- | | |
| TRINITY_DN26330_c0_g2_i4_len_4298_228027.p2_type_5prime_partial_len_175 | XP_018019530.1 epidermal growth factor receptor kinase substrate 8-like protein 2 | F:GO:0016301; P:GO:0016310 | |
| TRINITY_DN10304_c0_g1_i1_len_527_38161.p1_type_internal_len_176 | ---NA--- | | |
| TRINITY_DN25582_c1_g1_i10_len_2690_229447.p2_type_complete_len_175 | ---NA--- | | |
| TRINITY_DN31657_c0_g1_i1_len_529_203439.p1_type_internal_len_176 | ---NA--- | | |
| 1601\|_\|c15563_f3p2_2867_isoform_c15563_full_length_coverage_3_non_full_length_coverage_2_isoform_length_2867_13276 | ---NA--- | | |
| 1602\|_\|c1496_f3p4_2867_isoform_c1496_full_length_coverage_3_non_full_length_coverage_4_isoform_length_2867_27814 | AMP81927.1Wee1 protein | P:GO:0000278; F:GO:0000287; F:GO:0004715; F:GO:0005524; C:GO:0005634; P:GO:0018108 | EC:2.7.10; EC:2.7.10.2 |
| 1603\|_\|c1900_f2p21_2866_isoform_c1900_full_length_coverage_2_non_full_length_coverage_21_isoform_length_2866_28060 | ASW35071.1Kinesin heavy chain | F:GO:0003777; F:GO:0005524; C:GO:0005874; P:GO:0007018; F:GO:0008017 | EC:3.6.1.15 |

<table>
<tr><td>TRINITY_DN24823_c0_g1_i1_len_2912_58231.p1_type_5prime_partial_len_955</td><td>XP_018007217.1 zinc finger protein 91-like</td><td>F:GO:0003676</td><td></td></tr>
<tr><td>TRINITY_DN16680_c0_g1_i1_len_3025_154306.p1_type_complete_len_955</td><td>XP_018020630.1 integrator complex subunit 5-like</td><td>C:GO:0032039</td><td></td></tr>
<tr><td>1608|_|c2007_f2p6_2865_isoform_c2007_full_length_coverage_2_non_full_length_coverage_6_isoform_length_2865_28144</td><td>AAY56092.114-3-3-like protein</td><td>F:GO:0019904</td><td></td></tr>
<tr><td>TRINITY_DN22091_c0_g1_i1_len_2890_229157.p1_type_5prime_partial_len_955</td><td>XP_023719153.1protein wings apart-like</td><td></td><td></td></tr>
<tr><td>1610|_|c3603_f2p2_2864_isoform_c3603_full_length_coverage_2_non_full_length_coverage_2_isoform_length_2864_12313</td><td>XP_023229109.1translocating chain-associated membrane protein 1-like</td><td></td><td></td></tr>
<tr><td>1611|_|c36249_f2p1_2863_isoform_c36249_full_length_coverage_2_non_full_length_coverage_1_isoform_length_2863_18935</td><td>AYD41592.1sequestosome-1, partial</td><td>F:GO:0008270; F:GO:0046872</td><td></td></tr>
<tr><td>1612|_|c47569_f2p1_2863_isoform_c47569_full_length_coverage_2_non_full_length_coverage_1_isoform_length_2863_24227</td><td>XP_015590384.1nucleolar complex protein 3 homolog isoform X1</td><td></td><td></td></tr>
<tr><td>TRINITY_DN5709_c0_g1_i1_len_649_239968.p1_type_3prime_partial_len_176</td><td>---NA---</td><td></td><td></td></tr>
<tr><td>TRINITY_DN21284_c0_g1_i1_len_1133_62324.p2_type_5prime_partial_len_175</td><td>XP_018016717.1 high-affinity choline transporter 1-like</td><td>P:GO:0006810; F:GO:0022857</td><td></td></tr>
<tr><td>TRINITY_DN22380_c0_g1_i1_len_830_127780.p1_type_complete_len_175</td><td>XP_018025524.1 programmed cell death protein 6-like isoform X2</td><td></td><td></td></tr>
<tr><td>TRINITY_DN15887_c0_g1_i1_len_525_38194.p1_type_internal_len_176</td><td>---NA---</td><td></td><td></td></tr>
<tr><td>TRINITY_DN343_c0_g1_i1_len_526_131771.p2_type_internal_len_176</td><td>---NA---</td><td></td><td></td></tr>
<tr><td>TRINITY_DN17912_c0_g1_i1_len_545_241893.p1_type_5prime_partial_len_175</td><td>AEH05998.1C type lectin containing domain protein</td><td>F:GO:0030246</td><td></td></tr>
<tr><td>TRINITY_DN20379_c0_g1_i1_len_1009_196476.p1_type_complete_len_175</td><td>---NA---</td><td></td><td></td></tr>
</table>

| | | | |
|---|---|---|---|
| TRINITY_DN27372_c0_g1_i1_len_880_197703.p1_type_complete_len_175 | XP_015511813.1 vesicle transport protein SFT2B | | |
| TRINITY_DN10315_c0_g1_i1_len_527_166473.p2_type_internal_len_176 | ---NA--- | | |
| TRINITY_DN20706_c1_g1_i1_len_527_166471.p4_type_internal_len_176 | ---NA--- | | |
| TRINITY_DN10834_c0_g1_i1_len_527_166454.p1_type_internal_len_176 | AJW67343.1glutathione S-transferase | F:GO:0004364 | EC:2.5.1.18 |
| TRINITY_DN21408_c0_g1_i2_len_792_198713.p1_type_5prime_partial_len_175 | XP_018026113.1PREDICTED: uncharacterized protein LOC108681578 | | |
| TRINITY_DN37150_c0_g1_i1_len_946_197032.p1_type_5prime_partial_len_175 | ---NA--- | | |
| TRINITY_DN16419_c0_g1_i2_len_958_196916.p1_type_complete_len_175 | XP_018022083.1 YY1-associated factor 2-like | P:GO:0045893; F:GO:0046872 | |
| TRINITY_DN36_c0_g1_i1_len_527_100696.p1_type_internal_len_176 | ---NA--- | | |
| TRINITY_DN25500_c0_g1_i1_len_527_100694.p1_type_internal_len_176 | ---NA--- | | |
| TRINITY_DN12005_c0_g1_i1_len_945_270591.p1_type_complete_len_175 | XP_018026977.1 putative nuclease HARBI1 isoform X1 | | |
| TRINITY_DN30331_c0_g1_i2_len_527_100706.p3_type_internal_len_176 | ---NA--- | | |
| TRINITY_DN3364_c0_g1_i1_len_542_275121.p1_type_5prime_partial_len_175 | ---NA--- | | |
| TRINITY_DN19099_c0_g1_i1_len_529_203434.p1_type_internal_len_176 | XP_021482657.1collagen alpha-5(IV) chain-like | F:GO:0005201; C:GO:0005581 | |
| TRINITY_DN15806_c0_g1_i2_len_941_270616.p1_type_5prime_partial_len_175 | XP_018024481.1 putative glycerol kinase 5 isoform X2 | P:GO:0008152; F:GO:0016772 | |
| TRINITY_DN21185_c0_g1_i1_len_526_275463.p1_type_internal_len_176 | XP_018026625.1 titin-like isoform X8 | | |
| TRINITY_DN21040_c0_g1_i1_len_525_275486.p1_type_internal_len_176 | KDR09113.1Metal-response element-binding transcription factor 2 | F:GO:0046872 | |

| | | | |
|---|---|---|---|
| TRINITY_DN30321_c0_g1_i1_len_3487_187947.p3_type_complete_len_175 | ---NA--- | | |
| TRINITY_DN13257_c0_g1_i1_len_529_275389.p1_type_internal_len_176 | ---NA--- | | |
| TRINITY_DN25885_c0_g1_i1_len_528_275407.p1_type_internal_len_176 | ---NA--- | | |
| TRINITY_DN41523_c0_g1_i1_len_527_203488.p1_type_internal_len_176 | ---NA--- | | |
| TRINITY_DN20372_c0_g1_i1_len_577_302726.p1_type_5prime_partial_len_175 | XP_013784372.1microsomal glutathione S-transferase 3-like | F:GO:0004364; C:GO:0016021 | EC:2.5.1.18 |
| TRINITY_DN20701_c0_g1_i6_len_526_8299.p1_type_internal_len_176 | ---NA--- | | |
| TRINITY_DN8061_c0_g1_i1_len_528_203452.p1_type_internal_len_176 | ---NA--- | | |
| TRINITY_DN10872_c0_g1_i1_len_822_237720.p1_type_3prime_partial_len_176 | ---NA--- | | |
| TRINITY_DN27708_c0_g1_i1_len_534_38009.p1_type_5prime_partial_len_175 | ---NA--- | | |
| TRINITY_DN28118_c0_g1_i1_len_525_8324.p1_type_internal_len_176 | ---NA--- | | |
| TRINITY_DN20343_c0_g1_i2_len_733_199537.p1_type_3prime_partial_len_175 | ---NA--- | | |
| TRINITY_DN24390_c0_g1_i2_len_1256_61673.p2_type_complete_len_174 | KOC59699.1tRNA-specific adenosine deaminase 2 | F:GO:0003824 | |
| TRINITY_DN20412_c0_g1_i1_len_937_161199.p1_type_complete_len_174 | XP_018009825.1PREDICTED: uncharacterized protein LOC108667321 | | |
| TRINITY_DN6128_c0_g1_i1_len_755_238472.p1_type_complete_len_174 | AFK11134.1adenylate kinase isoenzyme 6-like protein | F:GO:0004017; F:GO:0005524; C:GO:0005634; P:GO:0016310; F:GO:0016887; P:GO:0046940 | EC:2.7.4.4; EC:2.7.4.3; EC:3.6.1.3; EC:3.6.1.15 |
| TRINITY_DN35851_c0_g1_i1_len_525_203521.p1_type_internal_len_175 | ---NA--- | | |
| TRINITY_DN13390_c0_g1_i1_len_778_271984.p2_type_complete_len_174 | XP_023305132.160S ribosomal protein L23a | F:GO:0003735; C:GO:0005840; P:GO:0006412 | |

| | | | |
|---|---|---|---|
| TRINITY_DN8684_c0_g1_i1_len_524_275505.p1_type_internal_len_175 | ---NA--- | | |
| TRINITY_DN3080_c0_g1_i1_len_523_275522.p1_type_internal_len_175 | XP_018898940.1 homeodomain-interacting protein kinase 2 isoform X3 | | |
| TRINITY_DN18440_c0_g1_i1_len_761_238399.p1_type_complete_len_174 | XP_023706027.1transmembrane protein 11 homolog, mitochondrial | P:GO:0007005; C:GO:0031305 | |
| TRINITY_DN2265_c0_g1_i1_len_671_6052.p1_type_complete_len_174 | XP_018011564.1 protein FAM136A-like isoform X2 | | |
| TRINITY_DN16976_c0_g1_i1_len_592_240955.p1_type_5prime_partial_len_174 | XP_018027697.1PREDICTED: uncharacterized protein LOC108682943 isoform X2 | | |
| TRINITY_DN4250_c0_g1_i2_len_627_66672.p1_type_3prime_partial_len_175 | ---NA--- | | |
| TRINITY_DN14845_c0_g1_i1_len_526_8295.p1_type_internal_len_175 | ---NA--- | | |
| TRINITY_DN32407_c0_g1_i1_len_523_203578.p1_type_internal_len_175 | ---NA--- | | |
| TRINITY_DN7994_c0_g1_i1_len_525_203513.p1_type_internal_len_175 | ---NA--- | | |
| TRINITY_DN12643_c0_g1_i1_len_523_203576.p1_type_internal_len_175 | KOX81238.1Inositol hexakisphosphate and diphosphoinositol-pentakisphosphate kinase | F:GO:0000829; F:GO:0000832; F:GO:0005524; C:GO:0005829; P:GO:0016310; F:GO:0033857; F:GO:0052723; F:GO:0052724; F:GO:0102092 | EC:2.7.4.24; EC:2.7.4.21 |
| TRINITY_DN11243_c0_g1_i2_len_654_200730.p1_type_5prime_partial_len_174 | ---NA--- | | |
| TRINITY_DN18719_c0_g1_i1_len_624_201324.p1_type_5prime_partial_len_174 | XP_018010800.1 ADP-ribosylation factor-like protein 4C | | |
| TRINITY_DN17390_c0_g1_i1_len_522_131874.p1_type_internal_len_175 | XP_018022120.1 cuticle protein 19-like | F:GO:0042302 | |
| TRINITY_DN11136_c0_g1_i1_len_525_203514.p1_type_internal_len_175 | ---NA--- | | |

| | | | |
|---|---|---|---|
| TRINITY_DN7178_c0_g1_i1_len_524_131831.p2_type_internal_len_175 | ---NA--- | | |
| TRINITY_DN5463_c0_g1_i1_len_524_203539.p1_type_internal_len_175 | XP_018012021.1 macrophage mannose receptor 1-like | C:GO:0016020; C:GO:0016021 | |
| TRINITY_DN8158_c0_g1_i1_len_524_203558.p1_type_internal_len_175 | XP_023342619.1E3 ubiquitin-protein ligase HERC2-like | F:GO:0016740; F:GO:0046872 | |
| TRINITY_DN39555_c0_g1_i1_len_525_203525.p1_type_internal_len_175 | ---NA--- | | |
| TRINITY_DN5074_c0_g1_i1_len_525_203530.p1_type_internal_len_175 | EFX68457.1ATP-binding cassette, sub-family C, member 4 | F:GO:0005524; C:GO:0016021; F:GO:0042626; P:GO:0055085 | EC:3.6.1.3; EC:3.6.1.15 |
| TRINITY_DN14320_c0_g1_i1_len_524_203557.p1_type_internal_len_175 | ---NA--- | | |
| TRINITY_DN4964_c0_g1_i1_len_523_131859.p1_type_internal_len_175 | XP_018027895.1 protein Skeletor, isoforms B/C-like | | |
| TRINITY_DN6113_c0_g1_i1_len_526_166477.p1_type_internal_len_175 | XP_018009184.1 serine-rich adhesin for platelets-like | P:GO:0009987 | |
| TRINITY_DN31633_c0_g1_i1_len_526_100729.p1_type_internal_len_175 | ---NA--- | | |
| TRINITY_DN24644_c0_g1_i1_len_523_166557.p1_type_internal_len_175 | XP_026277752.1T-cell activation inhibitor, mitochondrial-like isoform X2 | | |
| TRINITY_DN16225_c0_g1_i1_len_804_198552.p1_type_5prime_partial_len_174 | XP_018027613.1 apolipoprotein D-like | P:GO:0006869; P:GO:0007568; F:GO:0008289 | |
| TRINITY_DN13786_c0_g1_i1_len_710_163408.p1_type_5prime_partial_len_174 | XP_026285795.1H/ACA ribonucleoprotein complex subunit 2-like protein | | |
| TRINITY_DN7623_c0_g1_i1_len_523_303717.p1_type_internal_len_175 | ---NA--- | | |
| TRINITY_DN11170_c0_g1_i1_len_523_203564.p1_type_internal_len_175 | XP_019640906.1 exportin-6-like | C:GO:0005634; P:GO:0006611; P:GO:0006886; F:GO:0008536; F:GO:0008565 | |
| TRINITY_DN14297_c0_g1_i2_len_1775_231845.p2_type_3prime_partial_len_175 | ---NA--- | | |
| TRINITY_DN27618_c0_g1_i1_len_524_242429.p1_type_internal_len_175 | ---NA--- | | |

| | | | |
|---|---|---|---|
| TRINITY_DN18208_c0_g1_i1_len_1231_159151.p1_type_3prime_partial_len_175 | ---NA--- | | |
| TRINITY_DN17302_c0_g1_i1_len_774_5057.p1_type_complete_len_174 | ---NA--- | | |
| TRINITY_DN14614_c0_g1_i2_len_2246_230387.p2_type_5prime_partial_len_174 | ---NA--- | | |
| TRINITY_DN26848_c0_g1_i1_len_523_242435.p1_type_internal_len_175 | KDR20478.1hypothetical protein L798_04777 | F:GO:0003677; C:GO:0005634 | |
| TRINITY_DN6284_c0_g1_i1_len_523_242449.p1_type_internal_len_175 | XP_011647373.1solute carrier family 35 member F5 | C:GO:0016020 | |
| TRINITY_DN22356_c0_g1_i4_len_523_242450.p1_type_internal_len_175 | ---NA--- | | |
| TRINITY_DN12701_c0_g1_i1_len_523_242455.p1_type_internal_len_175 | ---NA--- | | |
| TRINITY_DN30646_c0_g1_i1_len_522_242461.p1_type_internal_len_175 | XP_018024684.1 protein unc-13 homolog A-like | | |
| TRINITY_DN18804_c0_g1_i2_len_525_242385.p1_type_internal_len_175 | ---NA--- | | |
| TRINITY_DN17959_c0_g1_i1_len_659_66204.p1_type_complete_len_174 | ---NA--- | | |
| TRINITY_DN18418_c0_g1_i2_len_600_201777.p1_type_5prime_partial_len_174 | ---NA--- | | |
| TRINITY_DN28545_c0_g1_i1_len_3364_188064.p3_type_5prime_partial_len_174 | ---NA--- | | |
| TRINITY_DN7513_c0_g1_i1_len_870_63982.p2_type_3prime_partial_len_175 | KDQ71485.1Activator of basal transcription 1 | F:GO:0003676 | |
| TRINITY_DN9620_c0_g1_i1_len_524_100777.p1_type_internal_len_175 | AYD41579.1laccase-10-like protein | F:GO:0005507; F:GO:0052716; P:GO:0055114 | EC:1.10.3; EC:1.10.3.2 |
| TRINITY_DN21970_c0_g1_i2_len_574_130813.p1_type_5prime_partial_len_174 | ---NA--- | | |
| TRINITY_DN16676_c0_g1_i1_len_556_303060.p1_type_5prime_partial_len_174 | ---NA--- | | |

| | | | |
|---|---|---|---|
| TRINITY_DN12993_c0_g1_i1_len_755_300557.p2_type_5prime_partial_len_174 | XP_018014710.1 2-amino-3-carboxymuconate-6-semialdehyde decarboxylase-like isoform X3 | | |
| TRINITY_DN15234_c0_g1_i1_len_524_68555.p1_type_internal_len_175 | ---NA--- | | |
| TRINITY_DN22122_c0_g1_i1_len_661_200613.p1_type_5prime_partial_len_174 | ACH46006.1putative tubulin beta chain variant 1 | F:GO:0005200; F:GO:0005525; C:GO:0005874; P:GO:0007010; P:GO:0007017 | |
| TRINITY_DN5928_c0_g1_i1_len_525_203510.p1_type_internal_len_175 | ---NA--- | | |
| TRINITY_DN10343_c0_g1_i1_len_635_301883.p1_type_5prime_partial_len_174 | ---NA--- | | |
| TRINITY_DN20092_c0_g2_i2_len_525_68527.p1_type_internal_len_175 | PRD19284.1zinc finger protein | F:GO:0003676 | |
| TRINITY_DN25041_c0_g1_i4_len_2229_190164.p2_type_complete_len_174 | ---NA--- | | |
| TRINITY_DN19990_c0_g1_i1_len_1364_233700.p1_type_5prime_partial_len_174 | ---NA--- | | |
| TRINITY_DN27001_c0_g1_i1_len_522_100802.p1_type_internal_len_175 | XP_021562631.1elongation factor 1-alpha 1-like | F:GO:0003746; P:GO:0006414 | |
| TRINITY_DN21091_c0_g1_i4_len_524_100770.p1_type_internal_len_175 | ---NA--- | | |
| TRINITY_DN19986_c0_g2_i1_len_1090_235447.p1_type_complete_len_173 | ---NA--- | | |
| TRINITY_DN16719_c0_g1_i1_len_520_242514.p1_type_internal_len_174 | ---NA--- | | |
| TRINITY_DN24601_c0_g1_i1_len_519_131937.p1_type_internal_len_174 | XP_021944442.1angiotensin-converting enzyme-like | F:GO:0004180; C:GO:0005615; C:GO:0005886; P:GO:0006508; F:GO:0008237; F:GO:0008241; C:GO:0016021; F:GO:0046872 | EC:3.4.15 |
| TRINITY_DN33354_c0_g1_i1_len_521_242504.p1_type_internal_len_174 | XP_023223356.1tetratricopeptide repeat protein 17-like | | |

| | | | |
|---|---|---|---|
| TRINITY_DN3157_c0_g1_i1_len_521_242499.p1_type_internal_len_174 | ---NA--- | | |
| 1613\|_\|c15849_f2p5_2863_isoform_c15849_full_length_coverage_2_non_full_length_coverage_5_isoform_length_2863_28691 | KDR14048.1hypothetical protein L798_11961 | | |
| TRINITY_DN27013_c0_g1_i1_len_6450_186865.p1_type_complete_len_954 | XP_018013473.1 AP-2 complex subunit alpha-like | | |
| TRINITY_DN19971_c0_g1_i1_len_3527_29323.p1_type_complete_len_954 | XP_023709502.1protein SMG5 isoform X1 | | |
| 1616\|_\|c15581_f7p0_2862_isoform_c15581_full_length_coverage_7_non_full_length_coverage_0_isoform_length_2862_13285 | XP_018009822.1 G patch domain-containing protein 1-like | F:GO:0003676; F:GO:0003723; P:GO:0006397 | |
| TRINITY_DN24701_c0_g1_i1_len_3093_228889.p1_type_complete_len_954 | KZS09396.1WD repeat-containing protein 3 | C:GO:0032040; C:GO:0034388 | |
| 1618\|_\|c2452_f3p3_2862_isoform_c2452_full_length_coverage_3_non_full_length_coverage_3_isoform_length_2862_20948 | XP_026279934.1neurofilament heavy polypeptide-like | | |
| 1620\|_\|c10509_f4p7_2860_isoform_c10509_full_length_coverage_4_non_full_length_coverage_7_isoform_length_2860_17372 | XP_018017998.1 phospholipase A-2-activating protein-like | F:GO:0043130; P:GO:0043161 | |
| 1622\|_\|c1615_f9p6_2860_isoform_c1615_full_length_coverage_9_non_full_length_coverage_6_isoform_length_2860_20477 | XP_013416524.1nuclear pore complex protein Nup88-like | P:GO:0006611 | |
| 1623\|_\|c4379_f2p1_2859_isoform_c4379_full_length_coverage_2_non_full_length_coverage_1_isoform_length_2859_16851 | XP_018010262.1PREDICTED: uncharacterized protein LOC108667718 | | |
| 1624\|_\|c2100_f6p2_2859_isoform_c2100_full_length_coverage_6_non_full_length_coverage_2_isoform_length_2859_11658 | XP_021929488.1uncharacterized protein LOC110834541 isoform X3 | | |
| TRINITY_DN15230_c0_g1_i1_len_521_242498.p1_type_internal_len_174 | XP_014284054.1protein big brother isoform X2 | F:GO:0003713; C:GO:0005634; P:GO:1903508 | |
| TRINITY_DN15274_c0_g1_i1_len_520_242528.p1_type_internal_len_174 | ---NA--- | | |

| | | | |
|---|---|---|---|
| TRINITY_DN37635_c0_g1_i1_len_521_203618.p1_type_internal_len_174 | ---NA--- | | |
| TRINITY_DN28378_c0_g1_i1_len_556_131148.p1_type_complete_len_173 | XP_018013456.1 biogenesis of lysosome-related organelles complex 1 subunit 2-like | C:GO:0000930; P:GO:0016197; C:GO:0031083; P:GO:0032418; F:GO:0043015; C:GO:0099078 | |
| TRINITY_DN10288_c0_g1_i1_len_520_242529.p1_type_internal_len_174 | AFJ11398.1thromboxane A synthase | F:GO:0005506; C:GO:0016021; F:GO:0016705; F:GO:0020037; P:GO:0055114 | |
| TRINITY_DN27801_c0_g1_i1_len_519_242551.p1_type_internal_len_174 | ---NA--- | | |
| TRINITY_DN14224_c0_g1_i1_len_519_131940.p1_type_internal_len_174 | ---NA--- | | |
| TRINITY_DN20036_c0_g1_i1_len_631_201177.p1_type_5prime_partial_len_173 | XP_022908410.1transmembrane protein 242 | C:GO:0016020 | |
| TRINITY_DN8257_c0_g1_i1_len_521_203617.p1_type_internal_len_174 | PSN40609.1Mitogen-activated protein kinase kinase kinase 9 | F:GO:0004672; F:GO:0005524; P:GO:0006468; F:GO:0016301; P:GO:0016310 | |
| TRINITY_DN13585_c0_g1_i2_len_606_6887.p1_type_5prime_partial_len_173 | XP_018012435.1 neuroglian-like isoform X1 | C:GO:0016021 | |
| TRINITY_DN21694_c0_g1_i1_len_1169_195177.p1_type_5prime_partial_len_173 | PNF16666.1Septin-4 | F:GO:0005525 | |
| TRINITY_DN14651_c0_g1_i1_len_735_199511.p1_type_complete_len_173 | ---NA--- | | |
| TRINITY_DN6773_c0_g1_i1_len_523_38213.p1_type_internal_len_174 | ---NA--- | | |
| TRINITY_DN18832_c0_g1_i1_len_798_198635.p1_type_complete_len_173 | KDR09959.1Regulator of G-protein signaling 3 | F:GO:0005089; P:GO:0035023 | |
| TRINITY_DN37696_c0_g1_i1_len_521_242480.p1_type_internal_len_174 | XP_019633507.1 angiotensin-converting enzyme 2-like isoform X1 | C:GO:0005615; C:GO:0005886; F:GO:0008238 | |
| TRINITY_DN9717_c0_g1_i1_len_521_242481.p1_type_internal_len_174 | ---NA--- | | |

| | | | |
|---|---|---|---|
| TRINITY_DN10854_c0_g1_i1_len_521_242485.p1_type_internal_len_174 | ARE31873.1extracellular signal-regulated kinases | P:GO:0000165; F:GO:0004707; F:GO:0005524; C:GO:0005634; C:GO:0005737; P:GO:0010468 | EC:2.7.11; EC:2.7.11.24 |
| TRINITY_DN15807_c0_g1_i2_len_520_131914.p1_type_3prime_partial_len_174 | ---NA--- | | |
| TRINITY_DN3248_c0_g1_i1_len_522_131867.p1_type_internal_len_174 | ACY66381.1alcohol dehydrogenase | P:GO:0055114 | |
| TRINITY_DN30469_c0_g1_i1_len_522_242467.p1_type_internal_len_174 | ---NA--- | | |
| TRINITY_DN21492_c0_g1_i3_len_2279_295357.p3_type_complete_len_173 | XP_025085541.1 FAD synthase-like | F:GO:0016491 | |
| TRINITY_DN13019_c0_g1_i2_len_520_131916.p1_type_internal_len_174 | ---NA--- | | |
| TRINITY_DN11494_c0_g1_i1_len_520_275604.p1_type_internal_len_174 | XP_018021626.1 kielin/chordin-like protein | | |
| TRINITY_DN15208_c0_g1_i1_len_522_203586.p1_type_internal_len_174 | XP_018019714.1 organic cation transporter protein-like | | |
| TRINITY_DN18002_c0_g1_i3_len_618_130093.p1_type_3prime_partial_len_174 | ---NA--- | | |
| TRINITY_DN21307_c0_g1_i1_len_521_38269.p1_type_internal_len_174 | XP_014664686.1 dual specificity protein phosphatase 16-like | | |
| TRINITY_DN28606_c0_g1_i1_len_521_275581.p2_type_internal_len_174 | AKL71620.1juvenile hormone epoxide hydrolase | C:GO:0005789; P:GO:0019439; F:GO:0033961 | EC:3.3.2.9 |
| TRINITY_DN17490_c0_g1_i2_len_522_203593.p1_type_internal_len_174 | XP_018327685.1palmitoyltransferase ZDHHC6 | C:GO:0016020; F:GO:0016746 | |
| TRINITY_DN11266_c0_g1_i1_len_720_163280.p2_type_3prime_partial_len_174 | ---NA--- | | |
| TRINITY_DN19849_c0_g1_i1_len_1345_61290.p2_type_complete_len_173 | XP_026470972.1transmembrane protein 53-A-like | | |
| TRINITY_DN10565_c0_g1_i1_len_521_100827.p1_type_internal_len_174 | XP_023708719.1neuropathy target esterase sws isoform X1 | | |
| TRINITY_DN34217_c0_g1_i1_len_520_203653.p1_type_internal_len_174 | ---NA--- | | |

| | | | |
|---|---|---|---|
| TRINITY_DN25582_c1_g1_i1_len_603_201701.p1_type_5prime_partial_len_173 | KZC14936.1putative oxidoreductase dhs-27 | | |
| TRINITY_DN15872_c0_g1_i1_len_521_275570.p2_type_internal_len_174 | ---NA--- | | |
| TRINITY_DN21873_c0_g1_i1_len_971_196797.p2_type_complete_len_173 | ---NA--- | | |
| TRINITY_DN17827_c0_g1_i1_len_640_200993.p1_type_5prime_partial_len_173 | ---NA--- | | |
| TRINITY_DN6868_c0_g1_i1_len_576_67495.p1_type_5prime_partial_len_173 | XP_003915560.140S ribosomal protein S16 | F:GO:0003735; C:GO:0005840; P:GO:0006412 | |
| TRINITY_DN26768_c0_g1_i1_len_1126_195480.p2_type_complete_len_173 | XP_020774830.1testin isoform X2 | | |
| TRINITY_DN22789_c0_g1_i2_len_2383_230042.p2_type_complete_len_173 | ---NA--- | | |
| TRINITY_DN9703_c0_g1_i2_len_520_68627.p1_type_internal_len_174 | ---NA--- | | |
| TRINITY_DN1734_c0_g1_i1_len_521_68606.p1_type_internal_len_174 | XP_023716120.1A-kinase anchor protein 10, mitochondrial isoform X2 | F:GO:0051018 | |
| TRINITY_DN11828_c0_g1_i2_len_522_8374.p1_type_internal_len_174 | XP_021929365.1eukaryotic translation initiation factor 4B2 | F:GO:0008408; F:GO:0008409; P:GO:0090503 | |
| TRINITY_DN14281_c0_g1_i1_len_644_66423.p1_type_5prime_partial_len_173 | ---NA--- | | |
| TRINITY_DN35711_c0_g1_i1_len_522_203603.p1_type_internal_len_174 | BBC20718.1integrin alpha V | P:GO:0007155; P:GO:0007229; C:GO:0008305 | |
| TRINITY_DN6186_c0_g1_i1_len_521_8385.p1_type_internal_len_174 | AUC64082.1arrestin 2 | P:GO:0007165 | |
| TRINITY_DN19523_c0_g1_i5_len_1035_298613.p1_type_5prime_partial_len_173 | ---NA--- | | |
| TRINITY_DN21145_c0_g1_i1_len_520_8402.p1_type_internal_len_174 | AYE92700.1diphthamide biosynthesis protein 7 | | |
| TRINITY_DN7881_c0_g1_i1_len_523_166551.p1_type_internal_len_174 | AFP33455.1kinesin-like protein KIF3A | F:GO:0003777; F:GO:0005524; C:GO:0005874; P:GO:0007018; F:GO:0008017 | EC:3.6.1.15 |

| | | | |
|---|---|---|---|
| TRINITY_DN22429_c0_g1_i1_len_3850_153740.p3_type_5prime_partial_len_173 | XP_018008304.1PREDICTED: uncharacterized protein LOC108666010 | | |
| TRINITY_DN11198_c0_g1_i1_len_521_166607.p1_type_internal_len_174 | XP_008553085.1 multidrug resistance-associated protein 1 isoform X1 | F:GO:0000166; P:GO:0006810; C:GO:0016021; F:GO:0016887; F:GO:0022857 | EC:3.6.1.3; EC:3.6.1.15 |
| TRINITY_DN12586_c0_g1_i1_len_520_203649.p1_type_internal_len_174 | ---NA--- | | |
| TRINITY_DN40789_c0_g1_i1_len_519_203674.p1_type_internal_len_174 | XP_018015665.1 MOXD1 homolog 2-like | F:GO:0004500; F:GO:0005507; C:GO:0005615; P:GO:0006589; C:GO:0030667; P:GO:0042420; P:GO:0042421 | EC:1.14.17.1; EC:1.14.17 |
| TRINITY_DN7898_c0_g1_i1_len_520_303778.p2_type_internal_len_174 | ---NA--- | | |
| TRINITY_DN34257_c0_g1_i1_len_520_203646.p1_type_internal_len_174 | ---NA--- | | |
| TRINITY_DN30238_c0_g1_i1_len_521_166597.p1_type_internal_len_174 | ---NA--- | | |
| TRINITY_DN40957_c0_g1_i1_len_522_203600.p1_type_internal_len_174 | XP_018021226.1 organic cation transporter protein-like | | |
| TRINITY_DN33843_c0_g1_i1_len_584_241095.p1_type_3prime_partial_len_174 | ---NA--- | | |
| TRINITY_DN21951_c0_g1_i3_len_1874_92393.p4_type_5prime_partial_len_173 | AAT77811.1calpain B | F:GO:0004198; F:GO:0005509; P:GO:0006508 | EC:3.4.22 |
| TRINITY_DN24605_c0_g1_i1_len_519_38308.p1_type_internal_len_174 | ---NA--- | | |
| TRINITY_DN15730_c0_g1_i1_len_630_301963.p1_type_5prime_partial_len_173 | ---NA--- | | |
| TRINITY_DN24979_c0_g1_i1_len_861_127537.p2_type_5prime_partial_len_173 | AFP92127.1plasmolipin isoform 2 | C:GO:0016021 | |
| TRINITY_DN16636_c0_g1_i1_len_824_34605.p2_type_5prime_partial_len_173 | XP_016055510.1 D-beta-hydroxybutyrate dehydrogenase, mitochondrial | F:GO:0016491; P:GO:0055114 | |
| TRINITY_DN13667_c0_g1_i1_len_677_98357.p1_type_complete_len_172 | ACY66625.1eukaryotic translation elongation factor 1 epsilon 1 | C:GO:0016020 | |

| | | | |
|---|---|---|---|
| TRINITY_DN7186_c0_g1_i1_len_519_203669.p1_type_internal_len_173 | XP_026272089.1filamin-A isoform X2 | F:GO:0003779 | |
| TRINITY_DN16440_c0_g1_i1_len_643_200927.p1_type_3prime_partial_len_173 | XP_018016422.1 anoctamin-1-like | | |
| TRINITY_DN20007_c0_g1_i1_len_567_130934.p1_type_5prime_partial_len_172 | ---NA--- | | |
| TRINITY_DN27805_c0_g1_i1_len_518_166675.p1_type_internal_len_173 | ASR74812.1leucine-rich repeat-containing protein | C:GO:0030027; P:GO:0030335; P:GO:0051638; P:GO:1902745; P:GO:2000813 | |
| TRINITY_DN16497_c0_g1_i1_len_519_203663.p1_type_internal_len_173 | ---NA--- | | |
| TRINITY_DN468_c0_g1_i1_len_706_163460.p1_type_5prime_partial_len_172 | XP_018018244.1 enhancer of split m7 protein-like | | |
| TRINITY_DN39287_c0_g1_i1_len_516_203763.p1_type_internal_len_173 | XP_018011984.1 delta-like protein B | F:GO:0005509; P:GO:0007219; P:GO:0007275; C:GO:0016021 | |
| TRINITY_DN31661_c0_g1_i1_len_519_68655.p1_type_internal_len_173 | ---NA--- | | |
| TRINITY_DN19212_c0_g2_i1_len_598_302387.p1_type_5prime_partial_len_172 | ---NA--- | | |
| TRINITY_DN25265_c0_g1_i1_len_520_275593.p1_type_internal_len_173 | ---NA--- | | |
| TRINITY_DN17411_c0_g1_i1_len_4119_153648.p1_type_5prime_partial_len_172 | ---NA--- | | |
| TRINITY_DN24651_c0_g2_i1_len_518_68667.p1_type_internal_len_173 | ---NA--- | | |
| TRINITY_DN15344_c0_g1_i2_len_794_128080.p1_type_5prime_partial_len_172 | XP_018013129.1 UDP-glucuronosyltransferase 2B14-like | | |
| TRINITY_DN18191_c0_g1_i1_len_1435_296990.p2_type_3prime_partial_len_173 | ACY66488.1replication factor C 2 (40kD) isoform 2 | F:GO:0003677; F:GO:0005524; P:GO:0006260 | |
| TRINITY_DN31762_c0_g1_i1_len_529_203429.p1_type_5prime_partial_len_172 | ---NA--- | | |
| TRINITY_DN27501_c0_g1_i3_len_3062_188434.p2_type_3prime_partial_len_173 | XP_018027372.1 AP2-associated protein kinase 1-like | | |

| | | | |
|---|---|---|---|
| TRINITY_DN34099_c0_g1_i1_len_517_203750.p1_type_internal_len_173 | ---NA--- | | |
| TRINITY_DN39943_c0_g1_i1_len_518_203712.p1_type_internal_len_173 | XP_015836484.1PREDICTED: uncharacterized protein LOC655172 isoform X7 | | |
| TRINITY_DN11466_c0_g1_i1_len_518_203695.p1_type_internal_len_173 | ---NA--- | | |
| TRINITY_DN10655_c0_g1_i1_len_529_303591.p1_type_5prime_partial_len_172 | PSN52188.1Calmodulin-like protein 4 | | |
| TRINITY_DN18819_c0_g1_i1_len_692_200130.p1_type_5prime_partial_len_172 | ---NA--- | | |
| TRINITY_DN19646_c0_g1_i1_len_933_236681.p1_type_5prime_partial_len_172 | ---NA--- | | |
| TRINITY_DN24623_c0_g1_i1_len_517_68687.p1_type_internal_len_173 | ---NA--- | | |
| TRINITY_DN37564_c0_g1_i1_len_808_198513.p1_type_5prime_partial_len_172 | ---NA--- | | |
| TRINITY_DN1097_c0_g1_i1_len_518_203701.p1_type_internal_len_173 | XP_018023417.1PREDICTED: uncharacterized protein LOC108679326 | | |
| TRINITY_DN19035_c0_g1_i1_len_611_36800.p1_type_5prime_partial_len_172 | ---NA--- | | |
| TRINITY_DN4767_c0_g1_i1_len_728_199609.p1_type_3prime_partial_len_173 | ---NA--- | | |
| TRINITY_DN20361_c0_g2_i1_len_518_38341.p1_type_internal_len_173 | XP_023321830.1acyl-CoA synthetase family member 2, mitochondrial-like isoform X2 | | |
| TRINITY_DN9790_c0_g1_i1_len_517_203741.p1_type_internal_len_173 | ---NA--- | | |
| TRINITY_DN2832_c0_g1_i1_len_519_203680.p1_type_internal_len_173 | KZS13511.1Filamin-B | F:GO:0003779 | |
| TRINITY_DN14797_c0_g1_i1_len_552_67978.p1_type_5prime_partial_len_172 | KZC11080.1Proclotting enzyme | F:GO:0008233 | |

| | | | |
|---|---|---|---|
| TRINITY_DN8404_c0_g1_i1_len_520_203637.p1_type_internal_len_173 | ---NA--- | | |
| TRINITY_DN15498_c0_g1_i1_len_783_198825.p1_type_5prime_partial_len_172 | ---NA--- | | |
| TRINITY_DN38977_c0_g1_i1_len_518_203703.p1_type_3prime_partial_len_173 | XP_019879931.1 collagen and calcium-binding EGF domain-containing protein 1-like | F:GO:0005509; C:GO:0005581 | |
| TRINITY_DN40670_c0_g1_i1_len_518_203704.p1_type_internal_len_173 | XP_019642477.1 proteasome activator complex subunit 4-like isoform X7 | C:GO:0005634; C:GO:0005829; P:GO:0010499; F:GO:0016504; P:GO:0035093; F:GO:0070577; F:GO:0070628; C:GO:1990111 | |
| TRINITY_DN31042_c0_g1_i1_len_517_100923.p1_type_internal_len_173 | PIK55799.1hypothetical protein BSL78_07287 | F:GO:0003676; P:GO:0015074; C:GO:0016021 | |
| TRINITY_DN28880_c0_g1_i1_len_517_242607.p1_type_internal_len_173 | ASW35087.1CBP80/20-dependent translation initiation factor isoform X1 | F:GO:0003743; P:GO:0006413 | |
| 1625\|_\|c3100_f2p0_2859_isoform_c3100_full_length_coverage_2_non_full_length_coverage_0_isoform_length_2859_12267 | ---NA--- | | |
| 1626\|_\|c11716_f2p6_2859_isoform_c11716_full_length_coverage_2_non_full_length_coverage_6_isoform_length_2859_28577 | ---NA--- | | |
| 1627\|_\|c5185_f2p4_2858_isoform_c5185_full_length_coverage_2_non_full_length_coverage_4_isoform_length_2858_16935 | ARJ31756.1casein kinase II subunit alpha | F:GO:0004674; F:GO:0005524; P:GO:0006468 | EC:2.7.11 |
| 1630\|_\|c4836_f2p10_2856_isoform_c4836_full_length_coverage_2_non_full_length_coverage_10_isoform_length_2856_28368 | XP_018010833.1 alanine--tRNA ligase, cytoplasmic-like | F:GO:0003746; P:GO:0006412; P:GO:0006414 | |
| TRINITY_DN19919_c0_g1_i2_len_3178_29411.p1_type_complete_len_952 | XP_016043304.1 TRPM8 channel-associated factor 2-like isoform X2 | | |
| 1632\|_\|c18090_f2p0_2855_isoform_c18090_full_length_coverage_2_non_full_length_coverage_0_isoform_length_2855_17943 | AVN99055.1SRSF protein kinase 3-like | F:GO:0004672; F:GO:0005524; P:GO:0006468 | |

| | | | |
|---|---|---|---|
| 1633\|_\|c12478_f3p59_2855_isoform_c12478_full_length_coverage_3_non_full_length_coverage_59_isoform_length_2855_26192 | XP_022089587.1trifunctional enzyme subunit alpha, mitochondrial-like | | |
| 1635\|_\|c1952_f2p3_2854_isoform_c1952_full_length_coverage_2_non_full_length_coverage_3_isoform_length_2854_28103 | AAH99236.1Vars protein | | |
| TRINITY_DN27900_c0_g1_i1_len_3018_188512.p1_type_5prime_partial_len_951 | XP_015518116.1 poly(A) RNA polymerase gld-2 homolog A-like isoform X1 | | |
| TRINITY_DN30939_c0_g1_i2_len_3826_187683.p1_type_complete_len_951 | ---NA--- | | |
| TRINITY_DN35534_c0_g1_i1_len_518_242582.p1_type_internal_len_173 | XP_018012118.1 kinesin-like protein KIF21B | | |
| TRINITY_DN903_c0_g1_i1_len_517_242590.p1_type_internal_len_173 | ---NA--- | | |
| TRINITY_DN5286_c0_g1_i1_len_518_166691.p1_type_internal_len_173 | ---NA--- | | |
| TRINITY_DN619_c0_g1_i1_len_517_242596.p1_type_internal_len_173 | ---NA--- | | |
| TRINITY_DN11959_c0_g1_i1_len_544_68112.p1_type_5prime_partial_len_172 | XP_013784100.160S ribosomal protein L12-like | P:GO:0000027; F:GO:0003735; P:GO:0006412; C:GO:0022625; F:GO:0070180 | |
| TRINITY_DN124_c0_g1_i1_len_518_275638.p1_type_internal_len_173 | XP_022308172.1epidermal retinol dehydrogenase 2-like isoform X1 | | |
| TRINITY_DN17660_c0_g2_i2_len_567_165583.p1_type_complete_len_172 | XP_023229438.1bifunctional methylenetetrahydrofolate dehydrogenase/cyclohydrolase, mitochondrial-like | F:GO:0003824; P:GO:0046653 | |
| TRINITY_DN33181_c0_g1_i1_len_520_242531.p1_type_internal_len_173 | ---NA--- | | |
| TRINITY_DN26812_c0_g1_i1_len_519_242536.p1_type_internal_len_173 | ---NA--- | | |
| TRINITY_DN2319_c0_g1_i1_len_519_242542.p1_type_internal_len_173 | ---NA--- | | |

| | | | |
|---|---|---|---|
| TRINITY_DN21005_c0_g2_i1_len_871_161753.p1_type_5prime_partial_len_172 | XP_017061487.1PREDICTED: uncharacterized protein LOC108101587 | | |
| TRINITY_DN17516_c0_g1_i1_len_586_202064.p1_type_5prime_partial_len_172 | ---NA--- | | |
| TRINITY_DN29275_c0_g1_i1_len_935_33862.p1_type_5prime_partial_len_172 | ADE60733.1myosin essential light chain | F:GO:0005509 | |
| TRINITY_DN12893_c0_g1_i1_len_518_242567.p1_type_internal_len_173 | ---NA--- | | |
| TRINITY_DN27924_c0_g1_i1_len_518_242579.p1_type_internal_len_173 | XP_018019402.1 protein slit-like | | |
| TRINITY_DN31114_c0_g1_i1_len_519_242546.p1_type_internal_len_173 | XP_023720222.1tubulin alpha-1 chain-like | F:GO:0003924; F:GO:0005200; F:GO:0005525; C:GO:0005737; C:GO:0005874; P:GO:0007010; P:GO:0007017 | EC:3.6.1.15 |
| TRINITY_DN27924_c0_g1_i1_len_3587_187859.p2_type_3prime_partial_len_173 | XP_023340375.1trifunctional enzyme subunit alpha, mitochondrial-like | P:GO:0008152; F:GO:0016491 | |
| TRINITY_DN2600_c0_g1_i1_len_519_242554.p1_type_internal_len_173 | ---NA--- | | |
| TRINITY_DN10856_c0_g1_i1_len_763_199085.p1_type_3prime_partial_len_173 | ---NA--- | | |
| TRINITY_DN2790_c0_g1_i1_len_516_166728.p1_type_internal_len_172 | XP_018011840.1 long-chain-fatty-acid--CoA ligase 5-like | | |
| TRINITY_DN31498_c0_g1_i1_len_516_166721.p1_type_internal_len_172 | ---NA--- | | |
| TRINITY_DN17115_c0_g1_i2_len_722_199686.p2_type_5prime_partial_len_171 | ---NA--- | | |
| TRINITY_DN24158_c0_g1_i3_len_2492_229800.p2_type_complete_len_171 | KZS19268.1Disrupted in renal carcinoma protein 2 | C:GO:0016020; C:GO:0016021; P:GO:0055085 | |
| TRINITY_DN16007_c0_g1_i1_len_593_201920.p1_type_5prime_partial_len_171 | ---NA--- | | |
| TRINITY_DN5256_c0_g1_i1_len_514_166758.p1_type_internal_len_172 | ---NA--- | | |

| | | | |
|---|---|---|---|
| TRINITY_DN351_c0_g1_i1_len_515_203793.p1_type_internal_len_172 | ---NA--- | | |
| TRINITY_DN2179_c0_g1_i2_len_514_275723.p1_type_internal_len_172 | AOD27418.1Notch | F:GO:0005509; P:GO:0007219; P:GO:0007275; C:GO:0016021; P:GO:0030154; P:GO:0050793 | |
| TRINITY_DN8428_c0_g1_i1_len_671_6046.p1_type_5prime_partial_len_171 | XP_018013762.1PREDICTED: uncharacterized protein LOC108670779 | | |
| TRINITY_DN37218_c0_g1_i1_len_516_203769.p1_type_internal_len_172 | XP_013784583.1 protein zyg-11 homolog B-like | | |
| TRINITY_DN21418_c0_g1_i1_len_516_275680.p1_type_internal_len_172 | XP_018011110.1 desumoylating isopeptidase 2-like | | |
| TRINITY_DN23656_c0_g1_i1_len_545_166056.p1_type_5prime_partial_len_171 | ABN04203.1ribosomal protein L26 | F:GO:0003735; P:GO:0006412; C:GO:0015934 | |
| TRINITY_DN31500_c0_g1_i1_len_514_68749.p1_type_internal_len_172 | ---NA--- | | |
| TRINITY_DN23275_c0_g1_i3_len_2449_155092.p1_type_complete_len_171 | ---NA--- | | |
| TRINITY_DN28503_c0_g1_i1_len_943_236589.p1_type_3prime_partial_len_172 | ---NA--- | | |
| TRINITY_DN207_c0_g1_i1_len_1452_267926.p1_type_complete_len_171 | XP_013781109.1vitamin K epoxide reductase complex subunit 1-like protein 1 | C:GO:0016020; C:GO:0016021 | |
| TRINITY_DN9050_c0_g1_i2_len_516_303843.p1_type_internal_len_172 | XP_012149363.1 ribosome biogenesis protein BMS1 homolog | P:GO:0000462; F:GO:0003924; F:GO:0005525; C:GO:0005634; C:GO:0030686; F:GO:0034511 | EC:3.6.1.15 |
| TRINITY_DN22444_c0_g2_i1_len_513_203854.p1_type_internal_len_172 | ---NA--- | | |
| TRINITY_DN6626_c0_g1_i1_len_516_203775.p1_type_internal_len_172 | ---NA--- | | |
| TRINITY_DN3901_c0_g1_i1_len_515_303851.p1_type_internal_len_172 | XP_018015375.1 polypeptide N-acetylgalactosaminyltransferase 13-like | | |

| | | | |
|---|---|---|---|
| TRINITY_DN17246_c0_g1_i1_len_515_303862.p1_type_internal_len_172 | AGI04378.1chloroplast ribulose-1,5-bisphosphate carboxylase/oxygenase activase precursor protein | F:GO:0005524 | |
| TRINITY_DN28104_c0_g1_i1_len_899_161509.p1_type_5prime_partial_len_171 | ---NA--- | | |
| TRINITY_DN4591_c0_g1_i1_len_516_203776.p1_type_internal_len_172 | XP_018009875.1 protein amalgam-like isoform X1 | | |
| TRINITY_DN15116_c0_g1_i3_len_513_203842.p1_type_internal_len_172 | ---NA--- | | |
| TRINITY_DN25497_c0_g1_i1_len_676_65975.p1_type_5prime_partial_len_171 | ---NA--- | | |
| TRINITY_DN16583_c0_g1_i1_len_616_130115.p1_type_3prime_partial_len_172 | ---NA--- | | |
| TRINITY_DN18513_c0_g1_i1_len_520_203651.p1_type_5prime_partial_len_171 | BBC20717.1integrin alpha ps | C:GO:0016020 | |
| TRINITY_DN14181_c0_g1_i2_len_572_241343.p1_type_5prime_partial_len_171 | ---NA--- | | |
| TRINITY_DN15180_c0_g1_i1_len_571_202381.p1_type_5prime_partial_len_171 | XP_015510122.1 protein pigeon | | |
| TRINITY_DN16894_c0_g1_i1_len_726_5482.p1_type_3prime_partial_len_172 | ---NA--- | | |
| TRINITY_DN7461_c0_g1_i1_len_1578_232650.p2_type_5prime_partial_len_171 | BAO01183.1sterile-alpha and armadillo motif containing protein | P:GO:0009967; P:GO:0034128; F:GO:0035591; P:GO:0048678 | |
| TRINITY_DN18188_c0_g1_i2_len_513_100979.p1_type_internal_len_172 | ---NA--- | | |
| TRINITY_DN27408_c0_g1_i1_len_633_240224.p1_type_3prime_partial_len_172 | ---NA--- | | |
| TRINITY_DN13163_c0_g1_i1_len_755_199201.p1_type_5prime_partial_len_171 | XP_023225893.1zinc finger MYM-type protein 3-like isoform X1 | F:GO:0008270 | |
| TRINITY_DN16892_c0_g1_i1_len_516_203773.p1_type_internal_len_172 | PSN44715.1hypothetical protein C0J52_16645 | | |
| TRINITY_DN35045_c0_g1_i1_len_859_197943.p1_type_5prime_partial_len_171 | XP_018015167.1  protein SCAI-like | | |

| | | | |
|---|---|---|---|
| TRINITY_DN17615_c0_g1_i1_len_685_200241.p1_type_5prime_partial_len_171 | ---NA--- | | |
| TRINITY_DN27188_c0_g1_i1_len_515_8517.p1_type_internal_len_172 | ---NA--- | | |
| TRINITY_DN26326_c1_g1_i1_len_2525_189397.p3_type_3prime_partial_len_172 | ---NA--- | | |
| TRINITY_DN13832_c0_g1_i4_len_730_163147.p1_type_3prime_partial_len_172 | XP_018025688.1PREDICTED: uncharacterized protein LOC108681194 | | |
| TRINITY_DN12512_c0_g1_i1_len_529_203425.p1_type_5prime_partial_len_171 | ---NA--- | | |
| TRINITY_DN24194_c0_g1_i1_len_691_163673.p1_type_3prime_partial_len_172 | ---NA--- | | |
| TRINITY_DN26868_c0_g1_i1_len_1499_193110.p1_type_5prime_partial_len_171 | ---NA--- | | |
| TRINITY_DN23108_c0_g1_i2_len_674_163882.p1_type_5prime_partial_len_171 | XP_002935845.1 THAP domain-containing protein 2 | F:GO:0003676 | |
| TRINITY_DN16963_c1_g1_i1_len_516_68711.p1_type_internal_len_172 | ---NA--- | | |
| TRINITY_DN30629_c0_g1_i5_len_652_200778.p1_type_5prime_partial_len_171 | ---NA--- | | |
| TRINITY_DN30639_c0_g1_i1_len_515_242641.p1_type_internal_len_172 | ---NA--- | | |
| TRINITY_DN197_c0_g1_i1_len_515_242647.p1_type_internal_len_172 | XP_026464401.1dmX-like protein 2 | | |
| TRINITY_DN4566_c0_g1_i1_len_514_242653.p1_type_internal_len_172 | ---NA--- | | |
| TRINITY_DN10695_c1_g1_i1_len_514_242667.p1_type_internal_len_172 | XP_021916657.1low-density lipoprotein receptor-related protein 1 | F:GO:0005509 | |
| TRINITY_DN3102_c0_g1_i1_len_970_196811.p1_type_5prime_partial_len_171 | ADZ96217.1JHE-like carboxylesterase 1 | F:GO:0016787 | |
| TRINITY_DN8321_c0_g1_i1_len_516_242625.p1_type_internal_len_172 | ---NA--- | | |

| | | | |
|---|---|---|---|
| TRINITY_DN16491_c0_g1_i1_len_516_242632.p1_type_internal_len_172 | ---NA--- | | |
| TRINITY_DN11426_c0_g1_i1_len_514_203821.p1_type_internal_len_172 | ACU31810.1alpha2 macroglobulin isoform 2 | F:GO:0004866; C:GO:0005615; P:GO:0010951; C:GO:0016020 | |
| TRINITY_DN13495_c0_g1_i1_len_1312_61439.p1_type_5prime_partial_len_171 | ---NA--- | | |
| TRINITY_DN15290_c0_g1_i1_len_515_203779.p1_type_internal_len_172 | ---NA--- | | |
| TRINITY_DN33703_c0_g1_i1_len_511_166813.p1_type_internal_len_171 | ---NA--- | | |
| TRINITY_DN30174_c0_g1_i1_len_510_166850.p1_type_internal_len_171 | ---NA--- | | |
| TRINITY_DN25072_c0_g1_i1_len_510_166852.p1_type_internal_len_171 | ---NA--- | | |
| TRINITY_DN5218_c0_g1_i1_len_545_37815.p1_type_complete_len_170 | ETN60623.1mitochondrial 28S ribosomal protein S28 | C:GO:0005840 | |
| TRINITY_DN14429_c0_g1_i1_len_645_200898.p1_type_5prime_partial_len_170 | ---NA--- | | |
| TRINITY_DN29975_c0_g1_i5_len_3443_187995.p2_type_5prime_partial_len_170 | ---NA--- | | |
| TRINITY_DN19354_c0_g1_i1_len_514_166772.p1_type_internal_len_171 | ---NA--- | | |
| TRINITY_DN12834_c0_g1_i1_len_513_166786.p1_type_internal_len_171 | ---NA--- | | |
| TRINITY_DN11268_c0_g1_i1_len_758_199152.p1_type_5prime_partial_len_170 | XP_018006337.1PREDICTED: uncharacterized protein LOC108664281 | | |
| TRINITY_DN30548_c0_g1_i1_len_668_239681.p1_type_5prime_partial_len_170 | ---NA--- | | |
| TRINITY_DN4176_c0_g1_i1_len_536_8118.p1_type_5prime_partial_len_170 | XP_018015948.1 ribonuclease H2 subunit C-like | | |
| TRINITY_DN4224_c0_g1_i1_len_514_303883.p1_type_internal_len_171 | XP_018024682.1 microtubule-associated serine/threonine-protein kinase 2-like isoform X1 | F:GO:0000287; F:GO:0004674; F:GO:0005524; P:GO:0006468 | EC:2.7.11 |

| | | | |
|---|---|---|---|
| TRINITY_DN3522_c0_g1_i1_len_532_242201.p1_type_5prime_partial_len_170 | XP_022201848.1tensin-2-like | | |
| TRINITY_DN32978_c0_g1_i1_len_896_63798.p2_type_complete_len_170 | KZS21527.1 Collagen alpha chain | F:GO:0005201; C:GO:0005581 | |
| TRINITY_DN35043_c0_g1_i1_len_513_242701.p1_type_internal_len_171 | XP_018022858.1 homeobox protein unplugged-like | | |
| TRINITY_DN26902_c0_g1_i1_len_512_242705.p1_type_internal_len_171 | XP_018015343.1PREDICTED: uncharacterized protein LOC108672219 | | |
| TRINITY_DN12336_c0_g1_i1_len_512_242711.p1_type_internal_len_171 | XP_015599087.1kelch domain-containing protein 10 isoform X1 | | |
| TRINITY_DN37711_c0_g1_i1_len_512_242719.p1_type_internal_len_171 | XP_018023341.1PREDICTED: uncharacterized protein LOC108679245 | | |
| TRINITY_DN30648_c0_g1_i1_len_801_162413.p1_type_complete_len_170 | XP_018027697.1PREDICTED: uncharacterized protein LOC108682943 isoform X2 | | |
| TRINITY_DN27182_c0_g1_i1_len_511_242737.p1_type_internal_len_171 | PSN54467.1Protein zer-1 | C:GO:0031462 | |
| TRINITY_DN14824_c0_g1_i1_len_510_242780.p1_type_internal_len_171 | XP_018023417.1PREDICTED: uncharacterized protein LOC108679326 | | |
| TRINITY_DN14440_c0_g1_i1_len_878_161700.p1_type_complete_len_170 | XP_018027471.1 ADP-ribosylation factor-like protein 16 | | |
| TRINITY_DN16989_c0_g1_i1_len_1181_32619.p1_type_complete_len_170 | PNF43051.1BolA-like protein | | |
| TRINITY_DN12638_c0_g1_i1_len_546_303256.p1_type_5prime_partial_len_170 | XP_002735558.1 40S ribosomal protein S15-like | F:GO:0003723; F:GO:0003735; P:GO:0006412; C:GO:0015935 | |
| TRINITY_DN20472_c0_g1_i2_len_544_7936.p1_type_3prime_partial_len_171 | ---NA--- | | |
| TRINITY_DN25722_c0_g1_i1_len_5392_227714.p1_type_5prime_partial_len_1692 | XP_023715406.1serine/threonine-protein kinase SMG1 | | |
| TRINITY_DN23990_c0_g1_i1_len_5154_121775.p1_type_5prime_partial_len_1692 | KPJ04966.1Zinc finger protein 407 | F:GO:0003676; F:GO:0008270; F:GO:0046872 | |
| TRINITY_DN29098_c0_g1_i1_len_5585_186996.p1_type_complete_len_1690 | KDR21355.1hypothetical protein L798_03131 | | |

<table>
<tr><td>TRINITY_DN26971_c0_g1_i5_len_10135_186749.p1_type_complete_len_1690</td><td>XP_018913770.1 protein Gawky isoform X3</td><td></td><td></td></tr>
<tr><td>TRINITY_DN27813_c1_g1_i5_len_7557_186781.p1_type_complete_len_1688</td><td>---NA---</td><td></td><td></td></tr>
<tr><td>TRINITY_DN22903_c0_g1_i1_len_5227_57710.p1_type_complete_len_1686</td><td>XP_018018657.1 bifunctional glutamate/proline--tRNA ligase-like</td><td></td><td></td></tr>
<tr><td>TRINITY_DN14934_c0_g1_i2_len_5608_227665.p1_type_complete_len_1685</td><td>AJM87267.1clathrin heavy chain</td><td>F:GO:0005198; P:GO:0006886; P:GO:0016192; C:GO:0030130; C:GO:0030132; F:GO:0032051; P:GO:0048268; C:GO:0071439</td><td></td></tr>
<tr><td>180|_|c1715_f2p50_5035_isoform_c1715_full_length_coverage_2_non_full_length_coverage_50_isoform_length_5035_26039</td><td>KDR21087.1S-phase kinase-associated protein 2</td><td>F:GO:0016301; P:GO:0016310</td><td></td></tr>
<tr><td>TRINITY_DN30134_c0_g1_i1_len_6889_186826.p1_type_complete_len_1671</td><td>XP_018017708.1 girdin-like</td><td>P:GO:0016055</td><td></td></tr>
<tr><td>TRINITY_DN31021_c0_g1_i1_len_6317_186887.p1_type_5prime_partial_len_1668</td><td>XP_021923315.1puratrophin-1-like isoform X5</td><td>F:GO:0005089; P:GO:0035023</td><td></td></tr>
<tr><td>TRINITY_DN20994_c0_g1_i1_len_3018_176.p1_type_5prime_partial_len_951</td><td>XP_021917985.1 SWI/SNF complex subunit SMARCC2</td><td>C:GO:0070603; C:GO:0097458</td><td></td></tr>
<tr><td>1639|_|c2517_f2p0_2853_isoform_c2517_full_length_coverage_2_non_full_length_coverage_0_isoform_length_2853_21007</td><td>AYE56134.1timeless protein</td><td></td><td></td></tr>
<tr><td>TRINITY_DN22076_c0_g1_i3_len_2952_154386.p1_type_5prime_partial_len_951</td><td>XP_018902814.1 partitioning defective 3 homolog isoform X1</td><td></td><td></td></tr>
<tr><td>1642|_|c10762_f3p8_2853_isoform_c10762_full_length_coverage_3_non_full_length_coverage_8_isoform_length_2853_17398</td><td>XP_014669212.1 ancient ubiquitous protein 1-like</td><td>C:GO:0016020; C:GO:0016021</td><td></td></tr>
<tr><td>1643|_|c2312_f7p5_2853_isoform_c2312_full_length_coverage_7_non_full_length_coverage_5_isoform_length_2853_16316</td><td>XP_018013732.1 dolichyl-diphosphooligosaccharide--protein glycosyltransferase subunit STT3A-like</td><td></td><td></td></tr>
<tr><td>1644|_|c2961_f2p0_2853_isoform_c2961_full_length_coverage_2_non_full_length_coverage_0_isoform_length_2853_12256</td><td>KZS19794.1Ubiquitin carboxyl-terminal hydrolase 35</td><td>P:GO:0006511; P:GO:0016579; F:GO:0016787; F:GO:0036459</td><td></td></tr>
</table>

| | | | |
|---|---|---|---|
| 1647\|_\|c11677_f4p1_2852_isoform_c11677_full_length_coverage_4_non_full_length_coverage_1_isoform_length_2852_17466 | PNF39299.1NEDD8-activating enzyme E1 regulatory subunit | F:GO:0008641 | |
| 1648\|_\|c12242_f4p1_2852_isoform_c12242_full_length_coverage_4_non_full_length_coverage_1_isoform_length_2852_17497 | XP_023714842.1histone H2A deubiquitinase MYSM1-like isoform X2 | F:GO:0003677; F:GO:0004843; P:GO:0035522 | |
| 1649\|_\|c2215_f3p6_2851_isoform_c2215_full_length_coverage_3_non_full_length_coverage_6_isoform_length_2851_16242 | ---NA--- | | |
| TRINITY_DN14275_c0_g1_i1_len_3104_228883.p1_type_5prime_partial_len_950 | XP_018014499.1 phospholipase D gamma 3-like | | |
| TRINITY_DN19693_c0_g1_i1_len_607_240668.p1_type_5prime_partial_len_170 | ---NA--- | | |
| TRINITY_DN13951_c0_g1_i1_len_510_203910.p1_type_internal_len_171 | ---NA--- | | |
| TRINITY_DN27597_c0_g2_i1_len_511_203905.p1_type_internal_len_171 | ---NA--- | | |
| TRINITY_DN19127_c0_g1_i1_len_685_98256.p2_type_5prime_partial_len_170 | ---NA--- | | |
| TRINITY_DN37312_c0_g1_i1_len_512_203876.p1_type_internal_len_171 | ---NA--- | | |
| TRINITY_DN36860_c0_g1_i1_len_512_203859.p1_type_internal_len_171 | XP_026282093.1C2 domain-containing protein 5 isoform X1 | | |
| TRINITY_DN37914_c0_g1_i1_len_513_203851.p1_type_internal_len_171 | ---NA--- | | |
| TRINITY_DN20024_c0_g1_i1_len_837_198179.p1_type_complete_len_170 | XP_003706862.1PREDICTED: uncharacterized protein C16orf52 homolog A | C:GO:0016021 | |
| TRINITY_DN25949_c0_g1_i2_len_2281_230296.p2_type_complete_len_170 | ---NA--- | | |
| TRINITY_DN31495_c0_g1_i1_len_511_203890.p1_type_internal_len_171 | ---NA--- | | |

| | | | |
|---|---|---|---|
| TRINITY_DN41163_c0_g1_i1_len_511_203889.p1_type_internal_len_171 | PNF30240.1Serine/threonine-protein kinase tricorner | F:GO:0004674; F:GO:0005524; P:GO:0018105; P:GO:0035556 | EC:2.7.11 |
| TRINITY_DN4636_c0_g1_i1_len_512_203877.p1_type_internal_len_171 | XP_018006612.1 protein bric-a-brac 1-like isoform X4 | | |
| TRINITY_DN26632_c0_g1_i3_len_1147_195325.p1_type_3prime_partial_len_171 | XP_022806369.1piggyBac transposable element-derived protein 4-like | F:GO:0003676; F:GO:0003677 | |
| TRINITY_DN32472_c0_g1_i1_len_513_68780.p1_type_internal_len_171 | XP_018578343.1protein O-mannosyl-transferase 2-like | F:GO:0000030; P:GO:0006493; C:GO:0016020; C:GO:0016021; F:GO:0016740; F:GO:0016757; P:GO:0097502 | |
| TRINITY_DN6531_c0_g1_i1_len_511_275792.p1_type_internal_len_171 | KDQ71515.1Protein O-mannosyl-transferase 2 | F:GO:0000030; P:GO:0006493; C:GO:0016020; C:GO:0016021; F:GO:0016740; P:GO:0097502 | |
| TRINITY_DN11037_c0_g1_i2_len_512_275761.p1_type_internal_len_171 | ---NA--- | | |
| TRINITY_DN4370_c0_g1_i1_len_513_275752.p1_type_internal_len_171 | ---NA--- | | |
| TRINITY_DN12987_c0_g1_i1_len_510_68846.p1_type_internal_len_171 | KDR09959.1Regulator of G-protein signaling 3 | F:GO:0005089; P:GO:0035023 | |
| TRINITY_DN4601_c0_g1_i1_len_512_68816.p1_type_internal_len_171 | ---NA--- | | |
| TRINITY_DN28615_c0_g1_i1_len_512_68797.p2_type_internal_len_171 | XP_015375442.1 putative polypeptide N-acetylgalactosaminyltransferase 9 | C:GO:0016021 | |
| TRINITY_DN3742_c0_g1_i1_len_693_163643.p1_type_5prime_partial_len_170 | XP_018015338.1 zinc finger protein ubi-d4 B-like | F:GO:0005488 | |
| TRINITY_DN41009_c0_g1_i1_len_551_202863.p1_type_5prime_partial_len_170 | XP_018009674.1PREDICTED: uncharacterized protein LOC108667192 | | |
| TRINITY_DN7784_c0_g1_i1_len_512_8575.p1_type_internal_len_171 | ---NA--- | | |
| TRINITY_DN20148_c0_g1_i9_len_1264_268765.p1_type_complete_len_170 | ---NA--- | | |
| TRINITY_DN3184_c0_g1_i1_len_521_8396.p1_type_5prime_partial_len_170 | ACR43472.1hypothetical ferritin light-chain subunit | C:GO:0005623; P:GO:0006826; P:GO:0006879; F:GO:0008199 | |

| | | | |
|---|---|---|---|
| TRINITY_DN9742_c0_g1_i1_len_1126_195479.p1_type_5prime_partial_len_170 | ---NA--- | | |
| TRINITY_DN19684_c0_g1_i1_len_999_95607.p1_type_5prime_partial_len_170 | ---NA--- | | |
| TRINITY_DN5158_c0_g1_i1_len_510_8634.p1_type_internal_len_171 | ---NA--- | | |
| TRINITY_DN20228_c4_g1_i2_len_1333_32005.p1_type_5prime_partial_len_170 | ---NA--- | | |
| TRINITY_DN512_c0_g1_i1_len_514_100968.p1_type_internal_len_171 | XP_018027333.1 low-density lipoprotein receptor-related protein 2-like | | |
| TRINITY_DN2846_c0_g1_i1_len_868_197828.p1_type_5prime_partial_len_170 | ---NA--- | | |
| TRINITY_DN29924_c0_g1_i1_len_511_38477.p1_type_internal_len_171 | ---NA--- | | |
| TRINITY_DN16176_c0_g1_i3_len_543_131417.p1_type_5prime_partial_len_170 | ---NA--- | | |
| TRINITY_DN9701_c0_g1_i1_len_514_100967.p1_type_internal_len_171 | ---NA--- | | |
| TRINITY_DN3082_c0_g1_i2_len_512_101009.p1_type_internal_len_171 | ---NA--- | | |
| TRINITY_DN8362_c0_g1_i2_len_512_38464.p1_type_internal_len_171 | ---NA--- | | |
| TRINITY_DN23328_c0_g1_i1_len_510_101047.p1_type_internal_len_171 | ---NA--- | | |
| TRINITY_DN26803_c0_g1_i1_len_511_132140.p1_type_internal_len_171 | AYJ71540.1cuticle protein-8 | F:GO:0042302 | |
| TRINITY_DN15547_c0_g1_i1_len_512_132111.p1_type_internal_len_171 | ---NA--- | | |
| TRINITY_DN21323_c0_g1_i1_len_514_38427.p1_type_internal_len_171 | XP_018023891.1 myosin heavy chain, non-muscle-like isoform X4 | | |
| TRINITY_DN11758_c0_g1_i1_len_512_132116.p1_type_internal_len_171 | ---NA--- | | |

| | | | |
|---|---|---|---|
| TRINITY_DN4658_c0_g1_i1_len_519_203655.p1_type_5prime_partial_len_170 | ---NA--- | | |
| TRINITY_DN24122_c0_g1_i1_len_510_132150.p1_type_internal_len_171 | XP_018008513.1 bone morphogenetic protein 10-like | | |
| TRINITY_DN14916_c0_g1_i1_len_509_203964.p1_type_internal_len_170 | ---NA--- | | |
| TRINITY_DN9922_c0_g1_i1_len_508_203994.p1_type_internal_len_170 | ---NA--- | | |
| TRINITY_DN15726_c0_g1_i1_len_508_166879.p1_type_internal_len_170 | ---NA--- | | |
| TRINITY_DN20530_c0_g1_i1_len_2558_265394.p2_type_complete_len_169 | XP_018022215.1 solute carrier family 28 member 3-like | | |
| TRINITY_DN22269_c0_g1_i1_len_1518_157729.p1_type_5prime_partial_len_169 | ABY77968.1tetraspanin-like protein CD9 | C:GO:0016021 | |
| TRINITY_DN20049_c0_g1_i1_len_732_65293.p1_type_5prime_partial_len_169 | ---NA--- | | |
| TRINITY_DN9592_c0_g1_i1_len_1026_95440.p2_type_complete_len_169 | ---NA--- | | |
| TRINITY_DN94_c0_g1_i1_len_679_163836.p1_type_5prime_partial_len_169 | XP_025089132.1dCTP pyrophosphatase 1-like | C:GO:0005737; P:GO:0009143; F:GO:0046872; F:GO:0047429 | EC:3.6.1.19 |
| TRINITY_DN8796_c0_g1_i1_len_698_98111.p2_type_5prime_partial_len_169 | ---NA--- | | |
| TRINITY_DN32008_c0_g1_i1_len_510_166844.p1_type_internal_len_170 | XP_018012887.1PREDICTED: uncharacterized protein LOC108669952 | | |
| TRINITY_DN39442_c0_g1_i1_len_508_204000.p1_type_internal_len_170 | XP_023710203.1arf-GAP domain and FG repeat-containing protein 1 isoform X3 | | |
| TRINITY_DN17055_c0_g1_i3_len_542_166120.p1_type_3prime_partial_len_170 | ---NA--- | | |
| TRINITY_DN4613_c0_g1_i1_len_547_241851.p1_type_5prime_partial_len_169 | XP_018024578.1 G-protein coupled receptor moody-like | | |
| TRINITY_DN4970_c0_g1_i1_len_509_166869.p1_type_internal_len_170 | ---NA--- | | |

| | | | |
|---|---|---|---|
| TRINITY_DN13428_c0_g1_i1_len_1249_194593.p2_type_5prime_partial_len_169 | ---NA--- | | |
| TRINITY_DN4005_c0_g1_i1_len_618_201427.p1_type_5prime_partial_len_169 | ---NA--- | | |
| TRINITY_DN11835_c0_g1_i1_len_507_275874.p1_type_internal_len_170 | ---NA--- | | |
| TRINITY_DN4762_c0_g1_i1_len_509_275828.p1_type_internal_len_170 | XP_006819547.1 NHL repeat-containing protein 2-like | C:GO:0005623; P:GO:0045454 | |
| TRINITY_DN12951_c0_g1_i1_len_508_203980.p1_type_internal_len_170 | ---NA--- | | |
| TRINITY_DN6280_c0_g1_i1_len_509_275824.p1_type_internal_len_170 | ---NA--- | | |
| TRINITY_DN5307_c0_g1_i1_len_509_275819.p1_type_internal_len_170 | XP_023718739.1ankyrin repeat and BTB/POZ domain-containing protein 1-like | | |
| TRINITY_DN19863_c0_g3_i1_len_2048_266188.p1_type_complete_len_169 | XP_023313230.1UPF0389 protein CG9231 isoform X1 | | |
| TRINITY_DN5986_c0_g1_i1_len_508_132193.p1_type_internal_len_170 | XP_018008507.1 NFX1-type zinc finger-containing protein 1-like | F:GO:0003700; C:GO:0005634; P:GO:0006355; F:GO:0008270 | |
| TRINITY_DN19394_c0_g1_i2_len_854_299734.p1_type_5prime_partial_len_169 | ---NA--- | | |
| TRINITY_DN15549_c0_g1_i1_len_510_203921.p1_type_internal_len_170 | XP_023561927.1X-linked retinitis pigmentosa GTPase regulator isoform X2 | | |
| TRINITY_DN362_c0_g1_i1_len_509_203969.p1_type_internal_len_170 | PSN32749.1F-box/LRR-repeat protein 20 | | |
| TRINITY_DN19616_c0_g1_i5_len_675_98372.p2_type_5prime_partial_len_169 | ---NA--- | | |
| TRINITY_DN26104_c0_g1_i1_len_1336_194029.p1_type_5prime_partial_len_169 | KYQ49024.1DNA-binding protein D-ETS-4 | F:GO:0003700; C:GO:0005634; P:GO:0006355; F:GO:0043565 | |
| TRINITY_DN11027_c0_g1_i1_len_509_132167.p1_type_internal_len_170 | ---NA--- | | |
| TRINITY_DN5080_c0_g1_i1_len_508_203971.p1_type_internal_len_170 | ---NA--- | | |

| | | | |
|---|---|---|---|
| TRINITY_DN23190_c0_g1_i1_len_507_275887.p1_type_internal_len_170 | ---NA--- | | |
| TRINITY_DN16550_c0_g1_i1_len_2501_29777.p2_type_complete_len_169 | XP_018021638.1 poly(rC)-binding protein 3-like isoform X4 | | |
| TRINITY_DN14348_c0_g1_i1_len_508_203972.p1_type_internal_len_170 | ---NA--- | | |
| TRINITY_DN26297_c0_g1_i2_len_1158_195249.p1_type_complete_len_169 | XP_018020172.1PREDICTED: uncharacterized protein LOC108676584 | | |
| TRINITY_DN6719_c0_g1_i1_len_661_98561.p1_type_5prime_partial_len_169 | ACN38704.1heat shock protein 70 | F:GO:0005524 | |
| TRINITY_DN34179_c0_g1_i1_len_510_203929.p1_type_internal_len_170 | XP_018023968.1 spermatogenesis-defective protein 39 homolog | C:GO:0005737; P:GO:0006886 | |
| TRINITY_DN3271_c0_g1_i1_len_510_242769.p1_type_internal_len_170 | ---NA--- | | |
| TRINITY_DN5238_c0_g1_i1_len_510_242782.p1_type_internal_len_170 | ---NA--- | | |
| TRINITY_DN34429_c0_g1_i1_len_510_242787.p1_type_internal_len_170 | ---NA--- | | |
| TRINITY_DN15186_c0_g1_i1_len_507_204023.p1_type_internal_len_170 | ---NA--- | | |
| TRINITY_DN33261_c0_g1_i1_len_511_242739.p1_type_internal_len_170 | ---NA--- | | |
| TRINITY_DN1506_c0_g1_i1_len_754_199222.p1_type_5prime_partial_len_169 | XP_023704526.1leucine-rich repeat-containing protein 20 | | |
| TRINITY_DN20742_c0_g1_i1_len_633_129843.p2_type_5prime_partial_len_169 | XP_011442459.1PREDICTED: uncharacterized protein LOC105338874 | | |
| TRINITY_DN1771_c0_g1_i1_len_603_164947.p2_type_5prime_partial_len_169 | ---NA--- | | |
| TRINITY_DN1500_c0_g1_i1_len_507_101103.p2_type_internal_len_170 | ---NA--- | | |
| TRINITY_DN14726_c0_g1_i2_len_507_101111.p1_type_internal_len_170 | ---NA--- | | |

| | | | |
|---|---|---|---|
| TRINITY_DN6648_c0_g1_i1_len_615_201465.p1_type_5prime_partial_len_169 | XP_018019402.1 protein slit-like | F:GO:0005509; C:GO:0005576; C:GO:0016021 | |
| TRINITY_DN22185_c0_g1_i1_len_1019_235963.p1_type_5prime_partial_len_169 | ---NA--- | | |
| TRINITY_DN35799_c0_g1_i1_len_509_203944.p1_type_internal_len_170 | XP_023212744.1cytochrome P450 3A8-like | | |
| TRINITY_DN9137_c0_g1_i1_len_577_274516.p1_type_5prime_partial_len_169 | ---NA--- | | |
| TRINITY_DN24063_c0_g1_i1_len_1959_59445.p2_type_5prime_partial_len_169 | XP_017944984.1 oocyte zinc finger protein XlCOF7.1-like | | |
| TRINITY_DN10121_c0_g1_i1_len_569_302854.p1_type_5prime_partial_len_169 | ---NA--- | | |
| TRINITY_DN9766_c0_g1_i1_len_509_68886.p1_type_internal_len_170 | ---NA--- | | |
| TRINITY_DN18056_c0_g1_i1_len_510_203933.p1_type_internal_len_170 | XP_002741199.1 kelch domain-containing protein 10-like | | |
| TRINITY_DN13546_c0_g1_i1_len_944_236578.p1_type_3prime_partial_len_170 | ---NA--- | | |
| TRINITY_DN8760_c0_g1_i1_len_509_68868.p1_type_internal_len_170 | ---NA--- | | |
| TRINITY_DN8612_c0_g1_i1_len_510_101039.p1_type_internal_len_170 | ---NA--- | | |
| TRINITY_DN26340_c0_g1_i1_len_3014_188521.p1_type_5prime_partial_len_950 | XP_013387202.1DNA mismatch repair protein Msh2 | | |
| 1653\|_\|c3313_f2p1_2850_isoform_c3313_full_length_coverage_2_non_full_length_coverage_1_isoform_length_2850_21334 | ---NA--- | | |
| TRINITY_DN21833_c0_g1_i6_len_3858_62.p1_type_5prime_partial_len_949 | XP_026288790.1ribosome-binding protein 1 isoform X2 | | |
| TRINITY_DN31030_c0_g1_i2_len_4529_187305.p1_type_5prime_partial_len_949 | XP_015608172.1thioredoxin domain-containing protein 11 isoform X2 | | |

| | | | |
|---|---|---|---|
| 1659\|_\|c18063_f2p6_2847_isoform_c18063_full_length_coverage_2_non_full_length_coverage_6_isoform_length_2847_22666 | XP_018011098.1 kinesin light chain-like isoform X4 | | |
| TRINITY_DN30099_c0_g1_i1_len_4180_187490.p1_type_5prime_partial_len_949 | XP_018012401.1 centrosomal protein of 290 kDa-like isoform X14 | | |
| 1661\|_\|c11746_f2p25_2846_isoform_c11746_full_length_coverage_2_non_full_length_coverage_25_isoform_length_2846_28580 | AXR98470.126S proteasome non-ATPase regulatory subunit 2-like protein | C:GO:0008540; F:GO:0030234; P:GO:0042176; P:GO:0050790 | |
| 1662\|_\|c7314_f2p4_2846_isoform_c7314_full_length_coverage_2_non_full_length_coverage_4_isoform_length_2846_17127 | ---NA--- | | |
| 1664\|_\|c15424_f24p18_2845_isoform_c15424_full_length_coverage_24_non_full_length_coverage_18_isoform_length_2845_13235 | EFA09884.1pre-rRNA processing protein FTSJ3-like Protein | C:GO:0005634; F:GO:0008649; P:GO:0031167 | |
| 1665\|_\|c2779_f3p0_2844_isoform_c2779_full_length_coverage_3_non_full_length_coverage_0_isoform_length_2844_16693 | XP_011305520.1 probable RNA-binding protein 19 | F:GO:0003676; F:GO:0003723 | |
| TRINITY_DN2801_c0_g1_i1_len_702_272780.p2_type_5prime_partial_len_169 | ---NA--- | | |
| TRINITY_DN11433_c0_g1_i1_len_507_242852.p1_type_internal_len_170 | ---NA--- | | |
| TRINITY_DN11425_c0_g1_i2_len_509_68884.p1_type_internal_len_170 | ---NA--- | | |
| TRINITY_DN17684_c0_g1_i1_len_510_8619.p1_type_internal_len_170 | PIK54499.1Retrovirus-related Pol polyprotein from transposon | | |
| TRINITY_DN39595_c0_g1_i1_len_507_204005.p1_type_internal_len_170 | ---NA--- | | |
| TRINITY_DN7543_c0_g1_i1_len_508_166896.p1_type_internal_len_170 | ---NA--- | | |
| TRINITY_DN14578_c0_g1_i1_len_508_68904.p1_type_internal_len_170 | ---NA--- | | |

| | | | |
|---|---|---|---|
| TRINITY_DN22719_c0_g1_i3_len_2049_230963.p2_type_5prime_partial_len_169 | ---NA--- | | |
| TRINITY_DN11630_c0_g1_i1_len_509_242817.p1_type_internal_len_170 | ---NA--- | | |
| TRINITY_DN2189_c0_g1_i1_len_509_242816.p1_type_internal_len_170 | ---NA--- | | |
| TRINITY_DN10465_c0_g1_i1_len_509_203961.p1_type_internal_len_170 | ---NA--- | | |
| TRINITY_DN34385_c0_g1_i1_len_509_242802.p1_type_internal_len_170 | ---NA--- | | |
| TRINITY_DN1703_c0_g1_i1_len_509_242801.p1_type_internal_len_170 | ---NA--- | | |
| TRINITY_DN19523_c0_g1_i1_len_510_68838.p1_type_internal_len_170 | ---NA--- | | |
| TRINITY_DN1875_c0_g1_i1_len_644_129698.p1_type_5prime_partial_len_169 | AOD27420.1transcription factor CSL | F:GO:0000978; F:GO:0001228; C:GO:0005634; P:GO:0045944 | |
| TRINITY_DN5569_c0_g1_i2_len_510_68859.p1_type_internal_len_170 | XP_012541555.1 kinesin-like protein KIF26B | | |
| TRINITY_DN10373_c0_g1_i1_len_508_242830.p1_type_internal_len_170 | ---NA--- | | |
| TRINITY_DN4232_c0_g1_i1_len_508_242826.p1_type_internal_len_170 | XP_018020275.1 ATP-binding cassette sub-family D member 1-like | F:GO:0000166; F:GO:0005524; C:GO:0005778; P:GO:0006635; C:GO:0016021; F:GO:0016887; F:GO:0042626; P:GO:0055085 | |
| TRINITY_DN11694_c0_g1_i1_len_505_204067.p1_type_internal_len_169 | EPB72015.1zinc finger, C2H2 type | F:GO:0003676 | |
| TRINITY_DN15990_c0_g1_i1_len_506_204046.p1_type_internal_len_169 | XP_023723982.1CRAL-TRIO domain-containing protein C3H8.02 isoform X1 | | |
| TRINITY_DN26397_c0_g1_i1_len_639_66497.p1_type_complete_len_168 | XP_019544797.1 dipeptidase 1-like | C:GO:0016020; F:GO:0070011 | |
| TRINITY_DN1305_c0_g1_i1_len_507_38552.p1_type_internal_len_169 | XP_022244078.1protein tilB homolog | | |

| | | | |
|---|---|---|---|
| TRINITY_DN2424_c0_g1_i2_len_506_204047.p1_type_internal_len_169 | ---NA--- | | |
| TRINITY_DN3048_c0_g1_i1_len_506_204039.p1_type_internal_len_169 | ---NA--- | | |
| TRINITY_DN15414_c0_g1_i1_len_506_204043.p1_type_internal_len_169 | ---NA--- | | |
| TRINITY_DN16550_c0_g1_i1_len_2501_29777.p3_type_complete_len_168 | XP_018021637.1 poly(rC)-binding protein 3-like isoform X3 | | |
| TRINITY_DN2997_c0_g1_i1_len_505_204068.p2_type_internal_len_169 | ADG22162.1chitinase 1 precursor | F:GO:0004553; C:GO:0005576; P:GO:0005975; P:GO:0006030; F:GO:0008061 | |
| TRINITY_DN33210_c0_g1_i1_len_516_131995.p1_type_5prime_partial_len_168 | ---NA--- | | |
| TRINITY_DN41412_c0_g1_i1_len_504_204106.p1_type_internal_len_169 | ---NA--- | | |
| TRINITY_DN4482_c0_g1_i1_len_505_166969.p1_type_internal_len_169 | XP_017775552.1 serine/threonine-protein kinase tousled-like 2 isoform X19 | | |
| TRINITY_DN2167_c0_g1_i1_len_505_204091.p1_type_internal_len_169 | XP_018022748.1 mannosylglucosyl-3-phosphoglycerate phosphatase-like isoform X2 | F:GO:0000166; P:GO:0009166; F:GO:0016787 | |
| TRINITY_DN38565_c0_g1_i1_len_506_204052.p1_type_internal_len_169 | KZS14597.1cAMP and cAMP-inhibited cGMP 3',5'-cyclic phosphodiesterase 10A | F:GO:0004114; P:GO:0007165; F:GO:0046872 | EC:3.1.4.17 |
| TRINITY_DN5104_c0_g1_i1_len_504_204093.p1_type_internal_len_169 | XP_019559734.1 histone deacetylase 6 isoform X2 | F:GO:0016787 | |
| TRINITY_DN12930_c0_g1_i2_len_755_65051.p1_type_complete_len_168 | XP_018023601.1 glutaredoxin domain-containing cysteine-rich protein CG31559-like | C:GO:0005623; F:GO:0009055; F:GO:0015035; P:GO:0022900; P:GO:0045454 | |
| TRINITY_DN4978_c0_g1_i1_len_504_204117.p1_type_internal_len_169 | ---NA--- | | |
| TRINITY_DN15924_c0_g1_i1_len_507_8679.p1_type_internal_len_169 | ACK44247.1vitelline membrane outer layer 1-like protein | | |
| TRINITY_DN4282_c0_g1_i1_len_506_8695.p1_type_internal_len_169 | XP_018022538.1PREDICTED: uncharacterized protein LOC108678590 | | |

| | | | |
|---|---|---|---|
| TRINITY_DN4983_c0_g1_i1_len_506_8692.p2_type_internal_len_169 | ---NA--- | | |
| TRINITY_DN8153_c0_g1_i2_len_505_101170.p1_type_internal_len_169 | ---NA--- | | |
| TRINITY_DN35409_c0_g1_i1_len_507_68934.p1_type_internal_len_169 | ---NA--- | | |
| TRINITY_DN10753_c0_g1_i1_len_879_197707.p1_type_5prime_partial_len_168 | ---NA--- | | |
| TRINITY_DN6033_c0_g1_i1_len_506_68944.p1_type_internal_len_169 | XP_018027289.1 sushi, von Willebrand factor type A, EGF and pentraxin domain-containing protein 1-like | | |
| TRINITY_DN2142_c0_g1_i1_len_592_37084.p1_type_5prime_partial_len_168 | XP_018028131.1PREDICTED: uncharacterized protein LOC108683335 | C:GO:0016021 | |
| TRINITY_DN7947_c0_g1_i2_len_612_164792.p2_type_5prime_partial_len_168 | ---NA--- | | |
| TRINITY_DN21781_c0_g1_i1_len_1625_232431.p2_type_3prime_partial_len_169 | XP_018025051.1 sulfotransferase 1A3-like | | |
| TRINITY_DN11542_c0_g1_i1_len_507_101102.p1_type_internal_len_169 | XP_018020778.1 xylose isomerase-like | | |
| TRINITY_DN26163_c0_g1_i1_len_1798_231757.p2_type_3prime_partial_len_169 | ---NA--- | | |
| TRINITY_DN14196_c0_g1_i1_len_505_101156.p1_type_internal_len_169 | ---NA--- | | |
| TRINITY_DN14934_c0_g1_i1_len_506_275902.p1_type_internal_len_169 | ---NA--- | | |
| TRINITY_DN15134_c0_g1_i1_len_506_275894.p2_type_internal_len_169 | ---NA--- | | |
| TRINITY_DN29230_c0_g1_i1_len_542_68141.p1_type_5prime_partial_len_168 | XP_018010589.1 UNC93-like protein isoform X2 | | |
| TRINITY_DN13920_c0_g1_i2_len_505_275922.p1_type_3prime_partial_len_169 | ---NA--- | | |
| TRINITY_DN26784_c0_g1_i1_len_2964_188600.p3_type_complete_len_168 | XP_023931624.1zinc finger CCCH domain-containing protein 14-like | F:GO:0008143; P:GO:0043488; P:GO:1900364 | |

| | | | |
|---|---|---|---|
| TRINITY_DN3675_c0_g1_i1_len_504_69008.p1_type_internal_len_169 | ---NA--- | | |
| TRINITY_DN28667_c0_g1_i1_len_552_165905.p1_type_5prime_partial_len_168 | XP_015928354.1protein quiver | C:GO:0016020; P:GO:0050789 | |
| TRINITY_DN15204_c0_g1_i1_len_849_237447.p1_type_5prime_partial_len_168 | ---NA--- | | |
| TRINITY_DN25253_c0_g1_i1_len_504_101214.p1_type_internal_len_169 | AYD41580.1laccase-1-like protein | F:GO:0005507; F:GO:0052716; P:GO:0055114 | EC:1.10.3; EC:1.10.3.2 |
| TRINITY_DN28261_c0_g1_i8_len_601_201759.p1_type_3prime_partial_len_169 | ---NA--- | | |
| TRINITY_DN12211_c0_g1_i1_len_568_241430.p1_type_5prime_partial_len_168 | ---NA--- | | |
| TRINITY_DN15850_c0_g1_i2_len_542_241965.p1_type_5prime_partial_len_168 | XP_018026357.1 disrupted in renal carcinoma protein 2 homolog | | |
| TRINITY_DN25779_c0_g1_i1_len_506_242884.p1_type_internal_len_169 | ANQ44674.1heat shock protein 70 kDa | F:GO:0005524 | |
| TRINITY_DN33974_c0_g1_i1_len_514_203825.p1_type_5prime_partial_len_168 | AFJ11391.1cytosolic phospholipase A2 | F:GO:0004623; C:GO:0005737; P:GO:0009395; F:GO:0046872; F:GO:0102567; F:GO:0102568 | EC:3.1.1.1; EC:3.1.1.4 |
| TRINITY_DN23362_c0_g1_i1_len_1593_123987.p3_type_5prime_partial_len_168 | ---NA--- | | |
| TRINITY_DN30655_c0_g1_i1_len_505_242892.p1_type_internal_len_169 | ---NA--- | | |
| TRINITY_DN40898_c0_g1_i1_len_507_204003.p1_type_internal_len_169 | ---NA--- | | |
| TRINITY_DN17864_c0_g1_i1_len_1780_30801.p2_type_5prime_partial_len_168 | ---NA--- | | |
| TRINITY_DN18135_c0_g1_i1_len_910_127172.p1_type_3prime_partial_len_169 | BAM35674.1putative reverse transcriptase | F:GO:0003964; P:GO:0006278 | EC:2.7.7.49 |
| TRINITY_DN6924_c0_g1_i1_len_506_38578.p1_type_internal_len_169 | ---NA--- | | |
| TRINITY_DN17221_c0_g1_i1_len_640_200994.p1_type_5prime_partial_len_168 | XP_023702800.139S ribosomal protein L41, mitochondrial isoform X1 | C:GO:0005840 | |

| | | | |
|---|---|---|---|
| TRINITY_DN11016_c0_g1_i1_len_505_242894.p1_type_3prime_partial_len_169 | XP_018016351.1 trimethyllysine dioxygenase, mitochondrial-like isoform X2 | | |
| TRINITY_DN43_c0_g1_i2_len_507_204015.p1_type_internal_len_169 | ---NA--- | | |
| TRINITY_DN21317_c0_g1_i2_len_1569_1509.p2_type_5prime_partial_len_168 | ---NA--- | | |
| TRINITY_DN7022_c0_g1_i1_len_505_242898.p1_type_internal_len_169 | ---NA--- | | |
| TRINITY_DN37533_c0_g1_i1_len_507_204017.p1_type_internal_len_169 | XP_018014826.1 myotubularin-related protein 10-A-like | | |
| TRINITY_DN35916_c0_g1_i1_len_508_203981.p1_type_internal_len_169 | XP_018012435.1 neuroglian-like isoform X1 | C:GO:0016021 | |
| TRINITY_DN9211_c0_g1_i1_len_508_242827.p1_type_internal_len_169 | XP_018015167.1  protein SCAI-like | F:GO:0003714; C:GO:0005634; P:GO:0006351; P:GO:1903507 | |
| TRINITY_DN809_c0_g1_i1_len_508_242828.p1_type_internal_len_169 | KDR19806.1Alpha- and gamma-adaptin-binding protein p34 | | |
| TRINITY_DN9339_c0_g1_i1_len_727_272513.p1_type_3prime_partial_len_169 | XP_015509869.1PREDICTED: uncharacterized protein LOC107217026 | | |
| TRINITY_DN30079_c0_g1_i1_len_506_304024.p1_type_internal_len_169 | ---NA--- | | |
| TRINITY_DN89_c0_g1_i1_len_507_242853.p1_type_internal_len_169 | XP_023222053.1membrane-associated guanylate kinase, WW and PDZ domain-containing protein 2-like isoform X4 | | |
| TRINITY_DN34979_c0_g1_i1_len_507_242847.p1_type_internal_len_169 | XP_015925985.1protein crumbs | F:GO:0005509; C:GO:0016020; C:GO:0016021 | |
| TRINITY_DN12413_c0_g1_i1_len_507_242848.p1_type_internal_len_169 | XP_022910395.1nucleolar MIF4G domain-containing protein 1 homolog isoform X2 | F:GO:0003723 | |
| TRINITY_DN25101_c1_g1_i1_len_962_63339.p1_type_5prime_partial_len_168 | ---NA--- | | |
| TRINITY_DN12920_c0_g1_i1_len_558_67853.p2_type_5prime_partial_len_168 | PSN52188.1Calmodulin-like protein 4 | | |

| | | | |
|---|---|---|---|
| TRINITY_DN37709_c0_g1_i1_len_504_242942.p1_type_internal_len_169 | XP_018023324.1 branched-chain-amino-acid aminotransferase, cytosolic-like | C:GO:0005739; P:GO:0009098; P:GO:0009099; C:GO:0016021; F:GO:0052654; F:GO:0052655; F:GO:0052656 | EC:2.6.1.42 |
| TRINITY_DN31454_c0_g1_i1_len_504_242945.p1_type_internal_len_169 | XP_018025051.1 sulfotransferase 1A3-like | | |
| TRINITY_DN13109_c0_g1_i1_len_578_302691.p1_type_5prime_partial_len_168 | XP_020634102.1heme-binding protein 2 | | |
| TRINITY_DN2283_c0_g1_i1_len_683_200267.p1_type_5prime_partial_len_168 | XP_024080441.1methionine-R-sulfoxide reductase B1 isoform X1 | | |
| TRINITY_DN1147_c0_g1_i1_len_503_242955.p1_type_internal_len_168 | ---NA--- | | |
| TRINITY_DN13824_c0_g1_i1_len_502_69067.p1_type_internal_len_168 | XP_018024405.1PREDICTED: uncharacterized protein LOC108680144 | | |
| TRINITY_DN25080_c0_g2_i1_len_985_63208.p1_type_5prime_partial_len_167 | ---NA--- | | |
| TRINITY_DN19885_c0_g1_i3_len_602_6962.p1_type_complete_len_167 | ---NA--- | | |
| TRINITY_DN21556_c0_g1_i1_len_1099_235383.p1_type_complete_len_167 | XP_018010905.1 protein cornichon homolog 4-like isoform X2 | | |
| TRINITY_DN11345_c0_g1_i1_len_503_204158.p1_type_internal_len_168 | ---NA--- | | |
| TRINITY_DN21332_c0_g1_i4_len_846_4509.p1_type_5prime_partial_len_167 | ---NA--- | | |
| TRINITY_DN14750_c0_g1_i1_len_966_33676.p1_type_complete_len_167 | XP_021183077.128S ribosomal protein S17, mitochondrial | | |
| TRINITY_DN33380_c0_g1_i1_len_503_204164.p1_type_internal_len_168 | ---NA--- | | |
| TRINITY_DN21585_c0_g1_i1_len_623_273812.p1_type_complete_len_167 | XP_019549802.1 ubiquitin-fold modifier-conjugating enzyme 1 | | |
| TRINITY_DN15048_c0_g1_i1_len_501_204201.p1_type_internal_len_168 | XP_020299673.1ileal sodium/bile acid cotransporter-like | | |

<table>
<tr><td></td><td></td><td></td><td></td></tr>
<tr><td>TRINITY_DN40803_c0_g1_i1_len_503_204167.p1_type_internal_len_168</td><td>XP_018007770.1 non-lysosomal glucosylceramidase-like</td><td>F:GO:0003824</td><td></td></tr>
<tr><td>TRINITY_DN30495_c0_g1_i1_len_3219_188226.p1_type_5prime_partial_len_948</td><td>XP_021932598.1zinc finger and SCAN domain-containing protein 2-like</td><td>F:GO:0003676</td><td></td></tr>
<tr><td>TRINITY_DN24684_c0_g1_i2_len_3055_228937.p1_type_5prime_partial_len_948</td><td>KRT83081.1zinc finger protein</td><td>F:GO:0046872</td><td></td></tr>
<tr><td>1668|_|c1403_f3p19_2843_isoform_c1403_full_length_coverage_3_non_full_length_coverage_19_isoform_length_2843_27764</td><td>ABW79798.1vitellogenin receptor</td><td>F:GO:0005509; C:GO:0016021</td><td></td></tr>
<tr><td>1669|_|c18235_f2p2_2843_isoform_c18235_full_length_coverage_2_non_full_length_coverage_2_isoform_length_2843_13668</td><td>XP_021002187.1 arginine N-methyltransferase 9</td><td>P:GO:0006479; F:GO:0008168; F:GO:0016740; P:GO:0032259</td><td></td></tr>
<tr><td>1671|_|c1816_f3p20_2842_isoform_c1816_full_length_coverage_3_non_full_length_coverage_20_isoform_length_2842_25508</td><td>---NA---</td><td></td><td></td></tr>
<tr><td>1672|_|c18116_f2p0_2842_isoform_c18116_full_length_coverage_2_non_full_length_coverage_0_isoform_length_2842_13586</td><td>XP_018025752.1 replication factor C subunit 1-like</td><td>F:GO:0003689; F:GO:0005524; C:GO:0005634; C:GO:0005663; P:GO:0006260; P:GO:0006281</td><td>EC:3.6.1.3; EC:3.6.1.15</td></tr>
<tr><td>1673|_|c7608_f4p4_2842_isoform_c7608_full_length_coverage_4_non_full_length_coverage_4_isoform_length_2842_12647</td><td>XP_014673274.1 protein PAT1 homolog 1-like</td><td>P:GO:0000290</td><td></td></tr>
<tr><td>TRINITY_DN20476_c0_g1_i1_len_3251_228713.p1_type_5prime_partial_len_947</td><td>XP_018017432.1 dentin sialophosphoprotein-like</td><td></td><td></td></tr>
<tr><td>1676|_|c4429_f2p1_2841_isoform_c4429_full_length_coverage_2_non_full_length_coverage_1_isoform_length_2841_21429</td><td>---NA---</td><td></td><td></td></tr>
<tr><td>TRINITY_DN21694_c0_g1_i1_len_2989_91158.p1_type_3prime_partial_len_948</td><td>XP_018009822.1 G patch domain-containing protein 1-like</td><td>F:GO:0003676; F:GO:0003723; P:GO:0006397</td><td></td></tr>
<tr><td>TRINITY_DN30332_c0_g1_i1_len_502_242975.p1_type_internal_len_168</td><td>---NA---</td><td></td><td></td></tr>
<tr><td>TRINITY_DN2805_c0_g1_i1_len_541_8003.p1_type_5prime_partial_len_167</td><td>---NA---</td><td></td><td></td></tr>
</table>

| | | | |
|---|---|---|---|
| TRINITY_DN4700_c0_g1_i1_len_501_204236.p1_type_internal_len_168 | ---NA--- | | |
| TRINITY_DN20362_c0_g1_i1_len_3166_154161.p3_type_complete_len_167 | ---NA--- | | |
| TRINITY_DN35067_c0_g1_i1_len_503_204143.p1_type_internal_len_168 | ---NA--- | | |
| TRINITY_DN3862_c0_g1_i1_len_503_69049.p1_type_internal_len_168 | ---NA--- | | |
| TRINITY_DN9977_c0_g1_i2_len_503_69028.p1_type_internal_len_168 | XP_018024684.1 protein unc-13 homolog A-like | | |
| TRINITY_DN10678_c0_g1_i1_len_503_204144.p1_type_internal_len_168 | ---NA--- | | |
| TRINITY_DN2666_c0_g1_i1_len_503_204145.p1_type_internal_len_168 | ---NA--- | | |
| TRINITY_DN35293_c0_g1_i1_len_503_204142.p1_type_internal_len_168 | ---NA--- | | |
| TRINITY_DN2959_c0_g1_i1_len_503_204152.p1_type_internal_len_168 | ---NA--- | | |
| TRINITY_DN308_c0_g1_i1_len_508_242834.p1_type_5prime_partial_len_167 | ---NA--- | | |
| TRINITY_DN2596_c0_g1_i1_len_502_69050.p1_type_internal_len_168 | XP_018009933.1 pantetheinase-like | | |
| TRINITY_DN28317_c0_g1_i1_len_585_99663.p1_type_5prime_partial_len_167 | KDR10793.1Mimitin, mitochondrial | F:GO:0008137; F:GO:0009055; C:GO:0016020; P:GO:0022900; P:GO:0055114 | |
| TRINITY_DN12265_c0_g1_i1_len_884_271056.p1_type_complete_len_167 | ---NA--- | | |
| TRINITY_DN14547_c1_g1_i1_len_1735_232008.p2_type_5prime_partial_len_167 | XP_021000263.1rab GTPase-binding effector protein 1 | | |
| TRINITY_DN6796_c0_g1_i1_len_501_243010.p1_type_internal_len_168 | XP_018023446.1  protein brunelleschi-like | | |
| TRINITY_DN6421_c0_g1_i1_len_504_242917.p1_type_internal_len_168 | KFM73690.1A-kinase anchor protein 10, mitochondrial | C:GO:0044464 | |

| | | | |
|---|---|---|---|
| TRINITY_DN20176_c0_g1_i2_len_1943_231289.p3_type_5prime_partial_len_167 | ---NA--- | | |
| TRINITY_DN12536_c0_g1_i1_len_647_200867.p1_type_5prime_partial_len_167 | XP_018006404.1 dysbindin-like isoform X2 | | |
| TRINITY_DN21813_c0_g1_i2_len_1300_297439.p2_type_5prime_partial_len_167 | XP_023014164.1regucalcin-like isoform X2 | | |
| TRINITY_DN18199_c0_g1_i2_len_502_204183.p1_type_internal_len_168 | ---NA--- | | |
| TRINITY_DN33157_c0_g1_i1_len_502_204185.p1_type_internal_len_168 | ---NA--- | | |
| TRINITY_DN28882_c0_g1_i1_len_504_242924.p1_type_internal_len_168 | ---NA--- | | |
| TRINITY_DN8130_c0_g1_i1_len_503_132291.p1_type_internal_len_168 | ---NA--- | | |
| TRINITY_DN32643_c0_g1_i1_len_504_242931.p1_type_internal_len_168 | XP_018012343.1 dystonin-like isoform X1 | | |
| TRINITY_DN18608_c0_g1_i3_len_579_302673.p1_type_5prime_partial_len_167 | BAO96441.1prophenoloxidase beta | F:GO:0016491; F:GO:0046872; P:GO:0055114 | |
| TRINITY_DN9300_c0_g1_i1_len_504_275951.p1_type_internal_len_168 | ---NA--- | | |
| TRINITY_DN22545_c0_g1_i1_len_668_200496.p1_type_5prime_partial_len_167 | ---NA--- | | |
| TRINITY_DN19647_c0_g1_i1_len_702_163523.p2_type_5prime_partial_len_167 | PNF20654.1BLOC-1-related complex subunit 5 | P:GO:0032418 | |
| TRINITY_DN4744_c0_g1_i1_len_502_204191.p1_type_internal_len_168 | ---NA--- | | |
| TRINITY_DN10874_c0_g1_i1_len_502_242983.p1_type_internal_len_168 | ---NA--- | | |
| TRINITY_DN23858_c0_g1_i1_len_503_275968.p1_type_internal_len_168 | XP_021375341.1uncharacterized protein LOC110464448 | | |
| TRINITY_DN21010_c1_g1_i1_len_1586_1478.p1_type_complete_len_167 | XP_018561311.1UPF0389 protein CG9231 isoform X2 | | |

| | | | |
|---|---|---|---|
| TRINITY_DN13516_c0_g1_i1_len_501_204225.p1_type_internal_len_168 | XP_018025785.1 long-chain-fatty-acid--CoA ligase 4-like isoform X3 | | |
| TRINITY_DN8459_c0_g1_i1_len_503_275985.p1_type_internal_len_168 | ---NA--- | | |
| TRINITY_DN18406_c0_g1_i1_len_573_130823.p2_type_3prime_partial_len_168 | ---NA--- | | |
| TRINITY_DN20685_c0_g3_i1_len_610_273995.p1_type_5prime_partial_len_167 | ---NA--- | | |
| TRINITY_DN29668_c0_g1_i1_len_505_242895.p1_type_internal_len_168 | XP_023246157.1baculoviral IAP repeat-containing protein 6 | F:GO:0004842; P:GO:0006915; P:GO:0016567; P:GO:0032465 | |
| TRINITY_DN20673_c0_g1_i1_len_507_132214.p1_type_5prime_partial_len_167 | ---NA--- | | |
| TRINITY_DN16482_c0_g2_i1_len_549_303213.p1_type_5prime_partial_len_167 | XP_018015382.1 major facilitator superfamily domain-containing protein 8-like | | |
| TRINITY_DN27971_c0_g1_i1_len_504_166978.p1_type_3prime_partial_len_168 | CRK93649.1CLUMA_CG007178, isoform A | C:GO:0016020; C:GO:0016021; P:GO:0055085 | |
| TRINITY_DN28013_c0_g1_i1_len_505_166967.p1_type_internal_len_168 | BBC28099.1xanthine dehydrogenase | F:GO:0004854; F:GO:0004855; F:GO:0005506; F:GO:0009055; P:GO:0022900; F:GO:0051537; F:GO:0071949 | EC:1.17.3; EC:1.17.1; EC:1.17.1.4; EC:1.17.3.2 |
| TRINITY_DN22605_c0_g1_i3_len_501_8797.p1_type_internal_len_168 | ---NA--- | | |
| TRINITY_DN8724_c0_g1_i1_len_501_167042.p1_type_internal_len_168 | XP_006607284.1 nucleolar protein 10-like | | |
| TRINITY_DN13139_c0_g1_i4_len_975_270363.p1_type_complete_len_167 | KDR21679.1PCTP-like protein | F:GO:0008289 | |
| TRINITY_DN12824_c0_g1_i1_len_812_162302.p1_type_5prime_partial_len_167 | XP_018895661.1 transcription factor che-1-like | | |
| TRINITY_DN905_c0_g1_i1_len_529_68424.p1_type_5prime_partial_len_167 | XP_018007403.1 ets DNA-binding protein pokkuri-like | F:GO:0003677; P:GO:0045892 | |
| TRINITY_DN19870_c0_g1_i1_len_584_7237.p1_type_5prime_partial_len_167 | ---NA--- | | |

| | | | |
|---|---|---|---|
| TRINITY_DN30967_c0_g1_i1_len_505_166952.p2_type_internal_len_168 | ---NA--- | | |
| TRINITY_DN26404_c0_g1_i1_len_1201_125417.p1_type_5prime_partial_len_167 | ---NA--- | | |
| TRINITY_DN1375_c0_g1_i1_len_502_304125.p1_type_internal_len_168 | ---NA--- | | |
| TRINITY_DN24683_c0_g1_i1_len_502_8761.p1_type_internal_len_168 | ---NA--- | | |
| TRINITY_DN10479_c0_g1_i1_len_523_38233.p1_type_5prime_partial_len_167 | ---NA--- | | |
| TRINITY_DN26207_c0_g1_i1_len_2076_190633.p2_type_3prime_partial_len_168 | ---NA--- | | |
| TRINITY_DN10348_c0_g1_i1_len_501_304131.p1_type_internal_len_168 | KXJ07491.1Retrovirus-related Pol polyprotein from transposon 412 | | |
| TRINITY_DN11068_c0_g1_i1_len_503_304103.p1_type_internal_len_168 | ---NA--- | | |
| TRINITY_DN17864_c0_g1_i1_len_653_301658.p1_type_5prime_partial_len_167 | XP_018013407.1 14 kDa phosphohistidine phosphatase-like | | |
| TRINITY_DN4322_c0_g1_i1_len_503_38618.p1_type_internal_len_168 | ---NA--- | | |
| TRINITY_DN4995_c0_g1_i1_len_722_238863.p1_type_5prime_partial_len_167 | XP_018007338.1 transmembrane protein 104-like | | |
| TRINITY_DN17151_c0_g1_i1_len_504_204113.p1_type_5prime_partial_len_167 | ---NA--- | | |
| TRINITY_DN41061_c0_g1_i1_len_503_204132.p1_type_internal_len_168 | ---NA--- | | |
| TRINITY_DN18357_c0_g1_i1_len_631_201191.p1_type_5prime_partial_len_167 | ---NA--- | | |
| TRINITY_DN22682_c0_g1_i1_len_504_101181.p1_type_internal_len_168 | XP_018012596.1PREDICTED: uncharacterized protein LOC108669703 | | |
| TRINITY_DN58_c0_g1_i1_len_505_101160.p1_type_complete_len_167 | ---NA--- | | |

| | | | |
|---|---|---|---|
| TRINITY_DN24540_c0_g1_i1_len_503_204127.p1_type_internal_len_168 | ---NA--- | | |
| TRINITY_DN28426_c0_g1_i1_len_1418_193552.p2_type_3prime_partial_len_168 | XP_023992359.1gastrula zinc finger protein XlCGF71.1-like | F:GO:0003676 | |
| TRINITY_DN13202_c0_g1_i1_len_1180_234827.p1_type_5prime_partial_len_167 | ---NA--- | | |
| TRINITY_DN27299_c0_g1_i1_len_527_303635.p1_type_5prime_partial_len_167 | ---NA--- | | |
| TRINITY_DN26496_c0_g1_i1_len_1619_192467.p2_type_5prime_partial_len_167 | ---NA--- | | |
| TRINITY_DN2404_c0_g1_i1_len_796_198667.p1_type_3prime_partial_len_167 | PNF22199.1SPRY domain-containing protein 3 | | |
| TRINITY_DN16517_c0_g1_i1_len_977_298916.p1_type_complete_len_166 | ---NA--- | | |
| TRINITY_DN9962_c0_g1_i1_len_499_276071.p1_type_internal_len_167 | XP_018006912.1 SOSS complex subunit B1-B-like | | |
| TRINITY_DN5055_c0_g1_i1_len_518_38324.p1_type_3prime_partial_len_167 | XP_026498446.1UPF0565 protein C2orf69 homolog | | |
| TRINITY_DN21148_c0_g1_i1_len_499_204284.p1_type_internal_len_167 | AID61753.1fructose 1,6-biphosphate-aldolase A | F:GO:0004332; P:GO:0006096 | EC:4.1.2.13 |
| TRINITY_DN10920_c0_g1_i1_len_500_204268.p1_type_internal_len_167 | ---NA--- | | |
| TRINITY_DN16618_c0_g1_i1_len_680_200316.p1_type_3prime_partial_len_167 | AFU61121.1serine/threonine-specific protein kinase AKT | F:GO:0004674; F:GO:0005524; P:GO:0006468; P:GO:0043491 | EC:2.7.11 |
| TRINITY_DN15484_c0_g1_i1_len_1043_196159.p1_type_complete_len_166 | XP_018026869.1PREDICTED: uncharacterized protein LOC108682249 | C:GO:0016020; C:GO:0016021 | |
| TRINITY_DN33369_c0_g1_i1_len_500_204244.p1_type_internal_len_167 | EHH17292.1Zinc finger protein Kr19 | F:GO:0003676; P:GO:0006355; F:GO:0046872 | |
| TRINITY_DN3736_c0_g1_i2_len_501_204204.p1_type_internal_len_167 | XP_018013248.1 spartin-like | | |
| TRINITY_DN20533_c1_g1_i1_len_500_204272.p1_type_internal_len_167 | ---NA--- | | |

| | | | |
|---|---|---|---|
| TRINITY_DN21225_c0_g1_i1_len_618_240492.p1_type_3prime_partial_len_167 | KZC04011.1Xylulose kinase | P:GO:0005975; F:GO:0016301; P:GO:0016310; F:GO:0016773 | |
| TRINITY_DN10606_c0_g1_i1_len_687_200207.p1_type_5prime_partial_len_166 | ---NA--- | | |
| TRINITY_DN11206_c0_g1_i1_len_500_204262.p1_type_internal_len_167 | AXR98493.1headcase protein-like protein | | |
| TRINITY_DN31331_c0_g1_i1_len_500_132361.p1_type_internal_len_167 | XP_018013858.1 alkylglycerol monooxygenase-like | P:GO:0008152; C:GO:0016020; F:GO:0016491 | |
| TRINITY_DN20893_c0_g1_i4_len_500_204252.p1_type_internal_len_167 | ---NA--- | | |
| TRINITY_DN26457_c1_g1_i3_len_500_204251.p1_type_internal_len_167 | XP_023227362.1uncharacterized protein LOC111627913, partial | | |
| TRINITY_DN4845_c0_g1_i1_len_888_63850.p1_type_complete_len_166 | XP_022904808.1magnesium-dependent phosphatase 1-like | | |
| TRINITY_DN15521_c0_g1_i1_len_784_34915.p1_type_complete_len_166 | AMQ13577.1single VWC domain protein 1 | | |
| TRINITY_DN1060_c0_g1_i1_len_500_204249.p1_type_internal_len_167 | ---NA--- | | |
| TRINITY_DN15895_c0_g1_i1_len_499_8849.p1_type_internal_len_167 | ---NA--- | | |
| TRINITY_DN21167_c0_g1_i1_len_1595_192592.p1_type_complete_len_166 | ---NA--- | | |
| TRINITY_DN7633_c0_g1_i1_len_500_276052.p1_type_internal_len_167 | XP_018018401.1 inactive peptidyl-prolyl cis-trans isomerase FKBP6-like | | |
| TRINITY_DN18731_c0_g1_i1_len_957_196920.p1_type_5prime_partial_len_166 | XP_015123059.1 mitochondrial ornithine transporter 1 | | |
| TRINITY_DN38472_c0_g1_i1_len_498_204316.p1_type_internal_len_167 | ---NA--- | | |
| TRINITY_DN23832_c0_g1_i1_len_953_196958.p1_type_5prime_partial_len_166 | XP_023710595.1tumor suppressor candidate 2-like isoform X1 | | |
| TRINITY_DN8100_c0_g1_i1_len_499_204306.p1_type_internal_len_167 | ---NA--- | | |

| | | | |
|---|---|---|---|
| TRINITY_DN33951_c0_g1_i1_len_498_204312.p1_type_internal_len_167 | ---NA--- | | |
| TRINITY_DN3404_c0_g1_i1_len_501_204231.p1_type_internal_len_167 | XP_018022988.1 transducin-like enhancer protein 4 isoform X4 | | |
| TRINITY_DN2879_c0_g1_i2_len_499_276055.p1_type_internal_len_167 | ---NA--- | | |
| TRINITY_DN24060_c0_g1_i1_len_3432_188007.p1_type_5prime_partial_len_947 | XP_023240695.1uncharacterized protein LOC111639138 | | |
| 1681\|_\|c19167_f2p1_2840_isoform_c19167_full_length_coverage_2_non_full_length_coverage_1_isoform_length_2840_22763 | ---NA--- | | |
| TRINITY_DN21807_c0_g1_i1_len_3092_228892.p1_type_complete_len_946 | PSN50736.1E3 ubiquitin-protein ligase SMURF1 | P:GO:0006511; P:GO:0016567; F:GO:0016874; F:GO:0061630 | |
| TRINITY_DN30487_c0_g1_i1_len_3420_188017.p1_type_complete_len_946 | XP_018010654.1 poly(ADP-ribose) glycohydrolase-like | | |
| TRINITY_DN23928_c0_g1_i1_len_3042_188466.p1_type_5prime_partial_len_946 | XP_018334802.1patronin-like isoform X2 | F:GO:0005516; C:GO:0005874; F:GO:0008017 | |
| 1685\|_\|c47862_f57p16_2838_isoform_c47862_full_length_coverage_57_non_full_length_coverage_16_isoform_length_2838_19706 | AGZ89665.1eukaryotic initiation factor 4A | F:GO:0003743; F:GO:0004004; F:GO:0005524; P:GO:0006413 | EC:3.6.1.3; EC:3.6.1.15 |
| 1686\|_\|c2855_f3p0_2838_isoform_c2855_full_length_coverage_3_non_full_length_coverage_0_isoform_length_2838_12218 | KYB25088.1Putative serine/threonine-protein kinase haspin homolog-like Protein | F:GO:0016301; P:GO:0016310 | |
| 1687\|_\|c2184_f7p9_2838_isoform_c2184_full_length_coverage_7_non_full_length_coverage_9_isoform_length_2838_20763 | BAJ53744.1nucleotide excision repair protein | F:GO:0003684; C:GO:0005634; P:GO:0006289; P:GO:0043161 | |
| 1688\|_\|c1858_f5p4_2837_isoform_c1858_full_length_coverage_5_non_full_length_coverage_4_isoform_length_2837_11519 | ---NA--- | | |
| 1690\|_\|c2793_f3p1_2837_isoform_c2793_full_length_coverage_3_non_full_length_coverage_1_isoform_length_2837_12166 | PSN41296.1Leucine-zipper-like transcriptional regulator 1 | | |

| | | | |
|---|---|---|---|
| TRINITY_DN34626_c0_g1_i1_len_566_165604.p1_type_5prime_partial_len_166 | ---NA--- | | |
| TRINITY_DN8965_c0_g1_i1_len_611_240623.p1_type_5prime_partial_len_166 | ---NA--- | | |
| TRINITY_DN12396_c0_g1_i1_len_520_203652.p1_type_5prime_partial_len_166 | ---NA--- | | |
| TRINITY_DN34964_c0_g1_i1_len_499_204293.p1_type_internal_len_167 | ---NA--- | | |
| TRINITY_DN12000_c0_g1_i2_len_501_204239.p1_type_internal_len_167 | ---NA--- | | |
| TRINITY_DN36083_c0_g1_i1_len_499_204299.p1_type_internal_len_167 | ---NA--- | | |
| TRINITY_DN18468_c0_g1_i2_len_500_8803.p1_type_internal_len_167 | XP_022247295.1glutamine--fructose-6-phosphate aminotransferase [isomerizing] 2-like isoform X1 | | |
| TRINITY_DN31389_c0_g1_i1_len_578_202249.p1_type_5prime_partial_len_166 | PNF22086.1hypothetical protein B7P43_G08894 | | |
| TRINITY_DN2702_c0_g1_i1_len_613_164778.p1_type_5prime_partial_len_166 | ---NA--- | | |
| TRINITY_DN1763_c0_g1_i1_len_499_204290.p1_type_internal_len_167 | XP_024214437.1homeodomain-interacting protein kinase 2-like | F:GO:0004674; F:GO:0005524; P:GO:0006468 | EC:2.7.11 |
| TRINITY_DN17222_c0_g1_i1_len_507_242843.p1_type_5prime_partial_len_166 | ---NA--- | | |
| TRINITY_DN4420_c0_g1_i1_len_498_101334.p2_type_internal_len_167 | ASA69502.1penlectin 5-2 | | |
| TRINITY_DN6671_c0_g1_i1_len_499_167085.p1_type_internal_len_167 | XP_018021606.1 arylsulfatase I-like | | |
| TRINITY_DN19496_c0_g1_i5_len_1001_270193.p1_type_5prime_partial_len_166 | ---NA--- | | |
| TRINITY_DN28234_c0_g1_i2_len_1456_193332.p1_type_5prime_partial_len_166 | ---NA--- | | |
| TRINITY_DN31605_c0_g1_i1_len_502_204171.p1_type_internal_len_167 | PSN35490.1G-protein coupled receptor moody | P:GO:0007165; C:GO:0016020 | |

| | | | |
|---|---|---|---|
| TRINITY_DN16127_c0_g1_i1_len_499_243072.p2_type_internal_len_167 | ---NA--- | | |
| TRINITY_DN31295_c0_g1_i1_len_502_204175.p1_type_internal_len_167 | ---NA--- | | |
| TRINITY_DN21960_c0_g1_i1_len_506_275893.p1_type_5prime_partial_len_166 | BAU19444.1serine racemase | P:GO:0006520; F:GO:0030170 | |
| TRINITY_DN16930_c0_g1_i1_len_499_243063.p1_type_internal_len_167 | ---NA--- | | |
| TRINITY_DN31568_c0_g1_i1_len_499_243056.p1_type_internal_len_167 | XP_018011684.1 zinc finger protein 540-like | | |
| TRINITY_DN30228_c0_g1_i1_len_499_243053.p1_type_internal_len_167 | ---NA--- | | |
| TRINITY_DN22869_c0_g1_i1_len_1261_158978.p1_type_5prime_partial_len_166 | ---NA--- | | |
| TRINITY_DN1803_c0_g1_i1_len_498_243087.p1_type_internal_len_167 | ---NA--- | | |
| TRINITY_DN18402_c0_g1_i4_len_1262_61643.p2_type_complete_len_166 | ---NA--- | | |
| TRINITY_DN19235_c0_g1_i2_len_947_33784.p3_type_5prime_partial_len_166 | XP_021914576.1papilin isoform X2 | | |
| TRINITY_DN12148_c0_g1_i1_len_549_68020.p1_type_5prime_partial_len_166 | XP_018022103.1 trafficking protein particle complex subunit 6B-like | P:GO:0043087; P:GO:0048193 | |
| TRINITY_DN17180_c0_g1_i1_len_1168_159526.p1_type_complete_len_166 | ---NA--- | | |
| TRINITY_DN12498_c0_g1_i1_len_619_273866.p2_type_5prime_partial_len_166 | ---NA--- | | |
| TRINITY_DN27137_c0_g1_i1_len_500_101295.p1_type_internal_len_167 | ---NA--- | | |
| TRINITY_DN24951_c0_g1_i1_len_1717_192008.p3_type_5prime_partial_len_166 | ---NA--- | | |
| TRINITY_DN1906_c0_g1_i1_len_548_241806.p1_type_3prime_partial_len_167 | XP_023216198.1BRCA2-interacting transcriptional repressor EMSY-like isoform X1 | | |

| | | | |
|---|---|---|---|
| TRINITY_DN20531_c0_g1_i1_len_500_38707.p1_type_internal_len_167 | ---NA--- | | |
| TRINITY_DN15738_c0_g1_i3_len_704_128980.p1_type_5prime_partial_len_166 | XP_018017161.1 MIP18 family protein FAM96A-like | P:GO:0106035 | |
| TRINITY_DN10611_c0_g1_i1_len_998_270211.p2_type_5prime_partial_len_166 | ---NA--- | | |
| TRINITY_DN3792_c0_g1_i2_len_502_242999.p1_type_internal_len_167 | ---NA--- | | |
| TRINITY_DN15988_c0_g1_i1_len_502_204197.p1_type_internal_len_167 | PNF33522.1hypothetical protein B7P43_G17638, partial | | |
| TRINITY_DN12786_c0_g1_i1_len_502_242986.p1_type_internal_len_167 | XP_019634649.1 von Willebrand factor A domain-containing protein 8-like | F:GO:0005524; F:GO:0016887 | EC:3.6.1.3; EC:3.6.1.15 |
| TRINITY_DN8934_c0_g1_i1_len_501_243013.p1_type_internal_len_167 | ---NA--- | | |
| TRINITY_DN14844_c0_g1_i1_len_502_204187.p1_type_internal_len_167 | XP_018018286.1 glycerol-3-phosphate acyltransferase 1, mitochondrial-like | | |
| TRINITY_DN5704_c0_g1_i1_len_502_69065.p1_type_internal_len_167 | ---NA--- | | |
| TRINITY_DN1670_c0_g1_i1_len_501_132345.p1_type_internal_len_167 | ---NA--- | | |
| TRINITY_DN15469_c0_g1_i1_len_838_271416.p1_type_5prime_partial_len_166 | ---NA--- | | |
| TRINITY_DN15684_c0_g1_i1_len_500_304155.p1_type_internal_len_167 | ABW77320.1clottable protein 2 | F:GO:0005319; C:GO:0005576; P:GO:0006869; P:GO:0042381 | |
| TRINITY_DN30054_c0_g1_i1_len_500_69109.p1_type_internal_len_167 | ---NA--- | | |
| TRINITY_DN13739_c0_g1_i1_len_500_243037.p1_type_internal_len_167 | ---NA--- | | |
| TRINITY_DN34110_c0_g1_i1_len_502_204181.p1_type_internal_len_167 | XP_021920652.1tyrosine-protein phosphatase 10D isoform X1 | P:GO:0016311; F:GO:0016787 | |
| TRINITY_DN32468_c0_g1_i1_len_500_243031.p1_type_internal_len_167 | AYH52220.1putative peptidyl-alpha-hydroxyglycine-alpha-amidating lyase I | | |

| | | | |
|---|---|---|---|
| TRINITY_DN11720_c0_g1_i1_len_500_243029.p1_type_internal_len_167 | AEI88049.1beta chain spectrin-like protein | F:GO:0003779; F:GO:0005200; F:GO:0005543; C:GO:0008091; P:GO:0051693 | |
| TRINITY_DN18311_c0_g1_i1_len_500_243026.p1_type_internal_len_167 | ---NA--- | | |
| TRINITY_DN210_c0_g1_i1_len_500_243045.p2_type_internal_len_167 | ATU31747.1Calcium-activated chloride channel regulator 2 | C:GO:0016020; C:GO:0016021 | |
| TRINITY_DN22460_c0_g1_i1_len_2353_189812.p1_type_3prime_partial_len_167 | ---NA--- | | |
| TRINITY_DN32444_c0_g1_i1_len_500_243044.p1_type_internal_len_167 | AUM86164.15'-3' exoribonuclease 1 | P:GO:0000956; F:GO:0003676; P:GO:0006388; F:GO:0008409; C:GO:0016021; F:GO:0016772; P:GO:0090305 | |
| TRINITY_DN9345_c0_g1_i1_len_519_100867.p1_type_5prime_partial_len_166 | ---NA--- | | |
| TRINITY_DN23830_c0_g1_i1_len_1566_192729.p1_type_5prime_partial_len_166 | XP_018006785.1 longitudinals lacking protein, isoforms H/M/V-like isoform X23 | | |
| TRINITY_DN2213_c0_g1_i1_len_684_239441.p1_type_5prime_partial_len_165 | ABW82154.1crustin-like protein | C:GO:0005576; P:GO:0010466; F:GO:0030414 | |
| TRINITY_DN14837_c0_g1_i1_len_1268_94177.p2_type_complete_len_165 | PNF34937.1DNA-directed RNA polymerase III subunit RPC6 | F:GO:0003899; C:GO:0005666; P:GO:0006383 | EC:2.7.7.6 |
| TRINITY_DN368_c0_g1_i1_len_497_304253.p1_type_internal_len_166 | ---NA--- | | |
| TRINITY_DN2299_c0_g1_i1_len_497_132429.p1_type_internal_len_166 | ---NA--- | | |
| TRINITY_DN16705_c0_g1_i1_len_498_304223.p1_type_internal_len_166 | ---NA--- | | |
| TRINITY_DN11273_c0_g1_i2_len_496_38776.p1_type_3prime_partial_len_166 | XP_022236795.1polycystic kidney disease protein 1-like 3 | | |
| TRINITY_DN21829_c0_g1_i1_len_2831_238.p3_type_5prime_partial_len_165 | PSN31605.1Zinc finger RNA-binding protein | | |
| TRINITY_DN24596_c1_g1_i2_len_2174_59000.p2_type_complete_len_165 | XP_014088307.1 carbohydrate sulfotransferase 4 isoform X1 | F:GO:0008146; C:GO:0016021 | |

| | | | |
|---|---|---|---|
| TRINITY_DN13909_c0_g1_i1_len_497_304236.p1_type_internal_len_166 | ---NA--- | | |
| TRINITY_DN23900_c0_g1_i1_len_3001_228988.p2_type_complete_len_165 | AJR21491.1RNA helicase | F:GO:0003723; F:GO:0004386; F:GO:0032575; P:GO:0035194 | |
| TRINITY_DN7105_c0_g1_i1_len_497_101371.p1_type_internal_len_166 | ---NA--- | | |
| TRINITY_DN23292_c0_g1_i3_len_1572_124047.p2_type_5prime_partial_len_165 | ---NA--- | | |
| TRINITY_DN8475_c0_g1_i1_len_616_201454.p1_type_3prime_partial_len_166 | KDR08391.1General transcription factor 3C polypeptide 1 | | |
| TRINITY_DN7123_c0_g1_i1_len_497_243101.p1_type_internal_len_166 | XP_018027531.1 leucine-rich repeats and immunoglobulin-like domains protein 1 | | |
| TRINITY_DN17139_c0_g1_i1_len_497_243109.p1_type_internal_len_166 | ---NA--- | | |
| TRINITY_DN4464_c0_g1_i1_len_621_240443.p1_type_5prime_partial_len_165 | XP_018016847.1 lysophosphatidylcholine acyltransferase-like | C:GO:0016020; C:GO:0016021; F:GO:0016746 | |
| TRINITY_DN12998_c0_g1_i1_len_497_243113.p1_type_internal_len_166 | ---NA--- | | |
| TRINITY_DN20362_c0_g1_i1_len_1294_2299.p2_type_3prime_partial_len_166 | XP_023014164.1regucalcin-like isoform X2 | | |
| TRINITY_DN22684_c1_g1_i1_len_1159_195241.p1_type_5prime_partial_len_165 | ---NA--- | | |
| TRINITY_DN19912_c0_g2_i1_len_805_198541.p1_type_5prime_partial_len_165 | ---NA--- | | |
| TRINITY_DN18474_c1_g1_i1_len_673_163894.p1_type_complete_len_165 | ---NA--- | | |
| TRINITY_DN10652_c0_g1_i2_len_830_64318.p1_type_5prime_partial_len_165 | ---NA--- | | |
| TRINITY_DN10653_c0_g1_i1_len_542_203105.p1_type_3prime_partial_len_166 | ---NA--- | | |
| TRINITY_DN30930_c0_g1_i1_len_495_276172.p1_type_internal_len_166 | ---NA--- | | |

| | | | |
|---|---|---|---|
| TRINITY_DN13371_c0_g2_i1_len_497_69193.p1_type_internal_len_166 | ---NA--- | | |
| TRINITY_DN14966_c0_g1_i2_len_861_237328.p2_type_3prime_partial_len_166 | ---NA--- | | |
| TRINITY_DN27851_c0_g1_i1_len_495_101417.p1_type_internal_len_166 | ALC78850.15'-nucleotidase domain-containing protein 3 | F:GO:0046872 | |
| TRINITY_DN37141_c0_g1_i1_len_568_202463.p1_type_3prime_partial_len_166 | ---NA--- | | |
| TRINITY_DN8724_c0_g1_i1_len_498_132407.p1_type_internal_len_166 | XP_018018978.1PREDICTED: uncharacterized protein LOC108675465 | | |
| TRINITY_DN17742_c0_g1_i1_len_1088_95065.p2_type_5prime_partial_len_165 | ---NA--- | | |
| TRINITY_DN33779_c0_g1_i1_len_497_132422.p2_type_internal_len_166 | ---NA--- | | |
| TRINITY_DN19373_c0_g1_i2_len_1045_298560.p2_type_5prime_partial_len_165 | AUZ97323.1vitelline membrane outer layer 1-like protein | | |
| TRINITY_DN12035_c0_g1_i1_len_495_38798.p1_type_internal_len_166 | XP_023239873.1pre-mRNA-splicing factor SYF2-like isoform X2 | | |
| TRINITY_DN3187_c0_g1_i1_len_496_243144.p1_type_internal_len_166 | ---NA--- | | |
| TRINITY_DN14865_c0_g1_i1_len_496_243148.p1_type_internal_len_166 | ---NA--- | | |
| TRINITY_DN10194_c0_g1_i1_len_496_243134.p1_type_internal_len_166 | PNF39381.1Tyrosine-protein phosphatase 10D | P:GO:0006470; C:GO:0016020; F:GO:0016791 | |
| TRINITY_DN37277_c0_g1_i1_len_495_243158.p1_type_internal_len_166 | XP_022181038.1sushi, von Willebrand factor type A, EGF and pentraxin domain-containing protein 1 isoform X4 | | |
| TRINITY_DN28552_c0_g1_i1_len_495_243173.p1_type_internal_len_166 | ---NA--- | | |
| TRINITY_DN9198_c0_g1_i1_len_715_35538.p1_type_complete_len_165 | KDR14903.1CCR4-NOT transcription complex subunit 4 | F:GO:0003723; F:GO:0004842; C:GO:0016021; P:GO:0016567; C:GO:0030014; F:GO:0046872 | |

| | | | |
|---|---|---|---|
| TRINITY_DN19086_c0_g1_i3_len_496_167163.p1_type_internal_len_166 | XP_022094844.1gastrula zinc finger protein XlCGF26.1-like isoform X1 | F:GO:0003676 | |
| TRINITY_DN7844_c0_g1_i2_len_495_132487.p1_type_internal_len_166 | ---NA--- | | |
| TRINITY_DN14376_c0_g1_i1_len_499_204305.p1_type_internal_len_166 | ---NA--- | | |
| TRINITY_DN19234_c0_g1_i1_len_539_131504.p1_type_3prime_partial_len_166 | ---NA--- | | |
| TRINITY_DN29566_c0_g1_i1_len_551_241757.p1_type_5prime_partial_len_165 | KRT83794.1hypothetical protein AMK59_4081, partial | | |
| TRINITY_DN9259_c0_g1_i1_len_542_275126.p1_type_5prime_partial_len_165 | ---NA--- | | |
| 1692\|_\|c2437_f4p2_2836_isoform_c2437_full_length_coverage_4_non_full_length_coverage_2_isoform_length_2836_20934 | XP_021914845.1nuclear cap-binding protein subunit 3-like isoform X1 | F:GO:0000340; F:GO:0003729 | |
| 1693\|_\|c3556_f2p2_2836_isoform_c3556_full_length_coverage_2_non_full_length_coverage_2_isoform_length_2836_12307 | XP_018020719.1 phosphatidylinositol 3-kinase regulatory subunit alpha-like | C:GO:0005942; P:GO:0007165; F:GO:0016301; P:GO:0016310; F:GO:0035014; P:GO:0035556; P:GO:0043551; F:GO:0046872 | |
| 1694\|_\|c2092_f3p0_2834_isoform_c2092_full_length_coverage_3_non_full_length_coverage_0_isoform_length_2834_16183 | XP_024936270.1nucleolar protein 14 homolog isoform X2 | | |
| 1695\|_\|c13124_f2p0_2834_isoform_c13124_full_length_coverage_2_non_full_length_coverage_0_isoform_length_2834_13116 | ASW35075.1UDP-N-acetylglucosamine--peptide N-acetylglucosaminyltransferase 110 kDa subunit-like isoform X1 | P:GO:0006493; F:GO:0016757 | |
| 1696\|_\|c2931_f2p2_2833_isoform_c2931_full_length_coverage_2_non_full_length_coverage_2_isoform_length_2833_21297 | ---NA--- | | |
| 1697\|_\|c2483_f2p1_2833_isoform_c2483_full_length_coverage_2_non_full_length_coverage_1_isoform_length_2833_20975 | XP_011500472.1 cyclin-dependent kinase 12 | | |

| | | | |
|---|---|---|---|
| 1698\|_\|c2068_f2p32_2832_isoform_c2068_full_length_coverage_2_non_full_length_coverage_32_isoform_length_2832_26188 | ACH72070.1cyclin B | C:GO:0005634 | |
| TRINITY_DN21663_c0_g1_i1_len_3596_228411.p1_type_5prime_partial_len_944 | XP_020707733.1axin isoform X1 | | |
| TRINITY_DN26204_c1_g1_i1_len_3116_228859.p1_type_5prime_partial_len_944 | XP_016908248.1 protein unc-13 homolog D isoform X2 | | |
| 1702\|_\|c12012_f4p0_2832_isoform_c12012_full_length_coverage_4_non_full_length_coverage_0_isoform_length_2832_22098 | XP_018016182.1 protein DGCR14-like | | |
| TRINITY_DN17733_c0_g1_i1_len_497_204349.p1_type_internal_len_166 | ---NA--- | | |
| TRINITY_DN8561_c0_g1_i1_len_499_276061.p1_type_internal_len_166 | ---NA--- | | |
| TRINITY_DN8189_c0_g1_i1_len_498_204337.p1_type_internal_len_166 | ---NA--- | | |
| TRINITY_DN13853_c0_g1_i1_len_498_204331.p1_type_internal_len_166 | ---NA--- | | |
| TRINITY_DN19285_c0_g1_i1_len_1909_231382.p2_type_5prime_partial_len_165 | ---NA--- | | |
| TRINITY_DN12811_c0_g1_i1_len_498_204329.p1_type_internal_len_166 | ---NA--- | | |
| TRINITY_DN39014_c0_g1_i1_len_498_204327.p1_type_internal_len_166 | ---NA--- | | |
| TRINITY_DN13580_c0_g1_i1_len_498_204338.p1_type_internal_len_166 | AEB00643.1glucose transporter 1 | P:GO:0008643; C:GO:0016021; F:GO:0022857; P:GO:0055085 | |
| TRINITY_DN6672_c0_g1_i1_len_506_166926.p1_type_5prime_partial_len_165 | ---NA--- | | |
| TRINITY_DN32592_c0_g1_i1_len_497_204362.p1_type_internal_len_166 | ---NA--- | | |
| TRINITY_DN34359_c0_g1_i1_len_497_204366.p1_type_internal_len_166 | ---NA--- | | |

| | | | |
|---|---|---|---|
| TRINITY_DN40712_c0_g1_i1_len_496_204374.p1_type_internal_len_166 | KDR22057.1DNA mismatch repair protein Mlh3 | P:GO:0006259; F:GO:0097159; F:GO:1901363 | |
| TRINITY_DN13966_c0_g1_i1_len_496_204377.p1_type_internal_len_166 | AOR07228.1potassium voltage-gated channel subfamily H member 2 | F:GO:0005249; C:GO:0016021; P:GO:0071805 | |
| TRINITY_DN614_c0_g1_i1_len_496_204388.p1_type_internal_len_166 | ---NA--- | | |
| TRINITY_DN41395_c0_g1_i1_len_497_204351.p1_type_internal_len_166 | ---NA--- | | |
| TRINITY_DN29196_c0_g1_i1_len_646_273471.p1_type_5prime_partial_len_164 | XP_018026825.1 neutral ceramidase-like | | |
| TRINITY_DN13647_c0_g1_i1_len_493_243216.p1_type_internal_len_165 | XP_014272753.1methyltransferase-like protein 22 | P:GO:0006479; F:GO:0008276 | |
| TRINITY_DN6995_c0_g1_i1_len_549_37748.p1_type_5prime_partial_len_164 | ---NA--- | | |
| TRINITY_DN40668_c0_g1_i1_len_494_204423.p1_type_internal_len_165 | XP_018026562.1PREDICTED: uncharacterized protein LOC108681986, partial | F:GO:0004857; P:GO:0043086 | |
| TRINITY_DN40723_c0_g1_i1_len_494_204448.p1_type_internal_len_165 | ---NA--- | | |
| TRINITY_DN17487_c0_g1_i1_len_495_204400.p1_type_internal_len_165 | XP_015272158.1 oocyte zinc finger protein XlCOF6-like | | |
| TRINITY_DN22571_c0_g1_i1_len_1147_62241.p1_type_complete_len_164 | ---NA--- | | |
| TRINITY_DN24074_c0_g1_i2_len_1558_60483.p2_type_complete_len_164 | XP_013787287.2L-serine dehydratase/L-threonine deaminase-like | P:GO:0006520; F:GO:0030170 | |
| TRINITY_DN10579_c0_g1_i1_len_493_243196.p1_type_internal_len_165 | ---NA--- | | |
| TRINITY_DN9993_c0_g1_i1_len_556_131146.p1_type_5prime_partial_len_164 | AFU60551.1immune deficiency-like protein | P:GO:0007165 | |
| TRINITY_DN14125_c0_g1_i1_len_493_243215.p1_type_internal_len_165 | ---NA--- | | |
| TRINITY_DN23420_c0_g1_i1_len_1202_194945.p1_type_complete_len_164 | ---NA--- | | |

| | | | |
|---|---|---|---|
| TRINITY_DN12179_c0_g1_i1_len_807_198517.p1_type_complete_len_164 | ---NA--- | | |
| TRINITY_DN40071_c0_g1_i1_len_494_204424.p2_type_internal_len_165 | XP_018024858.1 monocarboxylate transporter 6-like | | |
| TRINITY_DN22254_c0_g1_i1_len_4176_228101.p4_type_5prime_partial_len_164 | ---NA--- | | |
| TRINITY_DN35753_c0_g1_i1_len_775_198923.p1_type_3prime_partial_len_165 | XP_018011324.1 PHD finger protein 12-like | F:GO:0046872 | |
| TRINITY_DN2951_c0_g1_i1_len_1185_234803.p1_type_3prime_partial_len_165 | ---NA--- | | |
| TRINITY_DN10674_c0_g1_i2_len_532_100599.p1_type_5prime_partial_len_164 | XP_018010684.1 uridine-cytidine kinase 2-like | F:GO:0004849; F:GO:0005524; P:GO:0016310; P:GO:0044206; P:GO:0044211 | EC:2.7.1.48 |
| TRINITY_DN1323_c0_g1_i1_len_494_276180.p4_type_internal_len_165 | ---NA--- | | |
| TRINITY_DN4574_c0_g1_i1_len_494_204439.p1_type_internal_len_165 | AEK99382.1lipoprotein receptor | F:GO:0004888; F:GO:0004930; P:GO:0007166; P:GO:0007186; C:GO:0016020; C:GO:0016021 | |
| TRINITY_DN28370_c0_g1_i1_len_3521_187908.p4_type_complete_len_164 | XP_018006626.1PREDICTED: uncharacterized protein LOC108664535 | | |
| TRINITY_DN41054_c0_g1_i1_len_494_204443.p1_type_internal_len_165 | XP_024944369.1potassium voltage-gated channel subfamily H member 6 isoform X10 | | |
| TRINITY_DN34158_c0_g1_i1_len_494_204445.p1_type_internal_len_165 | AEI88130.1hypothetical protein, partial | P:GO:0006928; P:GO:0006935; P:GO:0007166; P:GO:0009653; P:GO:0048731 | |
| TRINITY_DN14061_c0_g1_i1_len_620_302109.p1_type_5prime_partial_len_164 | ---NA--- | | |
| TRINITY_DN28778_c0_g1_i1_len_493_276209.p1_type_internal_len_165 | ---NA--- | | |
| TRINITY_DN32893_c0_g1_i1_len_494_204433.p1_type_internal_len_165 | XP_018009678.1 putative tyrosine-protein kinase Wsck | | |

| | | | |
|---|---|---|---|
| TRINITY_DN26409_c0_g1_i1_len_566_100005.p1_type_5prime_partial_len_164 | XP_013116345.1 39S ribosomal protein L27, mitochondrial | F:GO:0003735; C:GO:0005840; P:GO:0006412 | |
| TRINITY_DN33910_c0_g1_i1_len_494_204429.p1_type_internal_len_165 | ---NA--- | | |
| TRINITY_DN35127_c0_g1_i1_len_494_243186.p1_type_internal_len_165 | XP_018007770.1 non-lysosomal glucosylceramidase-like | F:GO:0004553; P:GO:0006629 | |
| TRINITY_DN9742_c0_g1_i1_len_494_243187.p1_type_internal_len_165 | ---NA--- | | |
| TRINITY_DN928_c0_g1_i1_len_493_38844.p1_type_internal_len_165 | ---NA--- | | |
| TRINITY_DN9990_c0_g1_i1_len_495_243153.p1_type_internal_len_165 | XP_023244479.1tyrosine-protein kinase CSK-like | F:GO:0004715; F:GO:0005524; P:GO:0018108 | EC:2.7.10; EC:2.7.10.2 |
| TRINITY_DN23920_c0_g1_i1_len_4090_187537.p2_type_complete_len_164 | ---NA--- | | |
| TRINITY_DN14586_c0_g1_i1_len_493_69296.p1_type_internal_len_165 | ---NA--- | | |
| TRINITY_DN13290_c0_g1_i2_len_492_204497.p1_type_internal_len_165 | XP_018015167.1  protein SCAI-like | F:GO:0003714; P:GO:0006351; P:GO:1903507 | |
| TRINITY_DN21837_c0_g1_i2_len_545_68090.p1_type_5prime_partial_len_164 | ---NA--- | | |
| TRINITY_DN700_c0_g1_i1_len_499_167083.p1_type_5prime_partial_len_164 | ---NA--- | | |
| TRINITY_DN13426_c0_g1_i1_len_515_203806.p1_type_5prime_partial_len_164 | ---NA--- | | |
| TRINITY_DN16709_c0_g1_i1_len_495_38795.p1_type_internal_len_165 | XP_018015635.1 src substrate protein p85-like | | |
| TRINITY_DN6092_c0_g1_i1_len_982_236264.p1_type_5prime_partial_len_164 | ANZ80586.1ubiquitin/ribosomal S30 fusion protein | F:GO:0003735; C:GO:0005840; P:GO:0006412 | |
| TRINITY_DN11614_c0_g1_i1_len_492_304336.p1_type_internal_len_165 | ---NA--- | | |
| TRINITY_DN19314_c0_g1_i1_len_577_202281.p1_type_5prime_partial_len_164 | EDO41139.1predicted protein | F:GO:0003824; P:GO:0009058 | |

| | | | |
|---|---|---|---|
| TRINITY_DN12234_c0_g1_i1_len_493_69303.p2_type_internal_len_165 | ---NA--- | | |
| TRINITY_DN17825_c0_g1_i2_len_578_241190.p1_type_3prime_partial_len_165 | ---NA--- | | |
| TRINITY_DN21427_c0_g1_i1_len_1438_233317.p2_type_complete_len_164 | ---NA--- | | |
| TRINITY_DN41481_c0_g1_i1_len_882_197688.p2_type_complete_len_164 | ---NA--- | | |
| TRINITY_DN31008_c0_g1_i1_len_519_275626.p1_type_5prime_partial_len_164 | ---NA--- | | |
| TRINITY_DN10873_c0_g1_i1_len_495_69225.p2_type_internal_len_165 | ---NA--- | | |
| TRINITY_DN12977_c0_g1_i1_len_534_166289.p1_type_5prime_partial_len_164 | XP_012062247.1 mitochondrial intermediate peptidase-like | P:GO:0006508; F:GO:0008237 | |
| TRINITY_DN13591_c0_g1_i1_len_503_304091.p1_type_5prime_partial_len_164 | EKC26303.1inscuteable-like protein | P:GO:0008356 | |
| TRINITY_DN22590_c0_g1_i1_len_717_199740.p1_type_5prime_partial_len_164 | ---NA--- | | |
| TRINITY_DN32088_c0_g1_i1_len_506_132221.p1_type_5prime_partial_len_164 | ATD13076.1rhodanese 2-like protein | | |
| TRINITY_DN10743_c0_g1_i1_len_493_204469.p1_type_internal_len_165 | ---NA--- | | |
| TRINITY_DN39669_c0_g1_i1_len_493_204457.p1_type_internal_len_165 | XP_018027895.1 protein Skeletor, isoforms B/C-like | | |
| TRINITY_DN31351_c0_g1_i1_len_671_200446.p1_type_5prime_partial_len_164 | ---NA--- | | |
| TRINITY_DN22049_c0_g1_i6_len_812_4756.p2_type_5prime_partial_len_164 | ---NA--- | | |
| TRINITY_DN21066_c0_g1_i2_len_493_204479.p1_type_internal_len_165 | ---NA--- | | |
| TRINITY_DN10878_c0_g1_i1_len_493_204474.p1_type_internal_len_165 | XP_018014348.1PREDICTED: uncharacterized protein LOC108671312 isoform X4 | | |

| | | | |
|---|---|---|---|
| TRINITY_DN26975_c0_g2_i2_len_602_201737.p1_type_5prime_partial_len_164 | AAQ24529.1crustacean hyperglycemic hormone 3 precursor | F:GO:0005184; C:GO:0005576; P:GO:0010469 | |
| TRINITY_DN14329_c0_g1_i2_len_494_38830.p1_type_internal_len_165 | ---NA--- | | |
| TRINITY_DN29567_c0_g1_i1_len_494_167189.p1_type_internal_len_165 | XP_020484418.1 ecto-NOX disulfide-thiol exchanger 1-like | | |
| TRINITY_DN29147_c0_g1_i1_len_494_167196.p1_type_internal_len_165 | ---NA--- | | |
| TRINITY_DN18722_c0_g1_i1_len_750_300608.p2_type_5prime_partial_len_164 | ---NA--- | | |
| TRINITY_DN37235_c0_g1_i1_len_492_204496.p1_type_internal_len_165 | ---NA--- | | |
| TRINITY_DN5106_c0_g1_i1_len_494_69253.p2_type_internal_len_165 | EEB11185.1hspc200, putative | | |
| TRINITY_DN23114_c0_g1_i5_len_1029_196310.p1_type_complete_len_164 | ---NA--- | | |
| TRINITY_DN4553_c0_g1_i1_len_1140_235113.p1_type_complete_len_164 | ---NA--- | | |
| TRINITY_DN21427_c0_g1_i1_len_1548_192841.p1_type_5prime_partial_len_164 | XP_018017141.1 negative elongation factor A-like | C:GO:0032021; P:GO:0034244 | |
| TRINITY_DN33804_c0_g1_i1_len_688_200188.p1_type_5prime_partial_len_164 | XP_018018330.1 kelch-like protein diablo | | |
| TRINITY_DN20899_c0_g1_i1_len_860_197925.p1_type_3prime_partial_len_165 | ---NA--- | | |
| TRINITY_DN14580_c0_g1_i1_len_492_101484.p1_type_internal_len_165 | ---NA--- | | |
| TRINITY_DN26895_c0_g1_i1_len_2319_189897.p2_type_complete_len_164 | ---NA--- | | |
| TRINITY_DN26976_c0_g1_i1_len_596_274205.p2_type_5prime_partial_len_164 | ---NA--- | | |
| TRINITY_DN16514_c0_g1_i3_len_630_301951.p1_type_5prime_partial_len_164 | XP_013417218.15-hydroxyisourate hydrolase-like isoform X2 | F:GO:0016787 | |

| | | | |
|---|---|---|---|
| TRINITY_DN32140_c0_g1_i1_len_570_202418.p1_type_5prime_partial_len_164 | XP_018910871.1PREDICTED: uncharacterized protein LOC109039710 | C:GO:0016020 | |
| TRINITY_DN18625_c0_g1_i3_len_564_202575.p1_type_3prime_partial_len_165 | XP_018025503.1 cytochrome P450 9e2-like isoform X3 | | |
| TRINITY_DN32931_c0_g1_i1_len_495_132495.p1_type_internal_len_165 | XP_023716963.1uncharacterized protein LOC111869568 | | |
| TRINITY_DN788_c0_g1_i1_len_595_37026.p1_type_5prime_partial_len_164 | XP_018016613.1PREDICTED: uncharacterized protein LOC108673319 | | |
| TRINITY_DN26957_c0_g1_i3_len_1201_194959.p1_type_complete_len_164 | KDR12416.1Vacuolar protein sorting-associated protein 13D | | |
| TRINITY_DN22928_c0_g1_i1_len_1598_192574.p3_type_3prime_partial_len_165 | CDQ05004.1BMA-BEN-1, isoform c | F:GO:0005200; F:GO:0005525; C:GO:0005874; P:GO:0007010; P:GO:0007017 | |
| TRINITY_DN11029_c0_g1_i1_len_495_276168.p1_type_internal_len_165 | XP_018010684.1 uridine-cytidine kinase 2-like | F:GO:0005524; C:GO:0005829; P:GO:0006238; P:GO:0016310; F:GO:0019206; P:GO:0044206 | |
| TRINITY_DN7233_c0_g1_i2_len_495_276154.p1_type_internal_len_165 | XP_003962935.1 protein-S-isoprenylcysteine O-methyltransferase | C:GO:0016020; F:GO:0016740 | |
| TRINITY_DN25419_c0_g1_i1_len_977_196757.p1_type_complete_len_164 | ---NA--- | | |
| TRINITY_DN17716_c0_g1_i1_len_656_66239.p1_type_complete_len_163 | XP_791682.2 thioredoxin domain-containing protein 12 | | |
| TRINITY_DN9537_c0_g1_i1_len_489_167323.p1_type_internal_len_164 | ---NA--- | | |
| 1703\|_\|c6244_f2p1_2831_isoform_c6244_full_length_coverage_2_non_full_length_coverage_1_isoform_length_2831_17037 | AJW83244.1dynamin superfamily protein | F:GO:0003924; F:GO:0005525 | EC:3.6.1.15 |
| 1704\|_\|c7568_f3p0_2831_isoform_c7568_full_length_coverage_3_non_full_length_coverage_0_isoform_length_2831_21702 | XP_026053667.1methylcrotonoyl-CoA carboxylase subunit alpha, mitochondrial | F:GO:0000166; F:GO:0043167 | |
| 1705\|_\|c2193_f9p4_2831_isoform_c2193_full_length_coverage_9_non_full_length_coverage_4_isoform_length_2831_20769 | ---NA--- | | |

| | | | |
|---|---|---|---|
| 1706\|_\|c12985_f2p0_2829_isoform_c12985_full_length_coverage_2_non_full_length_coverage_0_isoform_length_2829_13104 | XP_005099009.1 conserved oligomeric Golgi complex subunit 5-like | | |
| 1709\|_\|c6180_f2p6_2828_isoform_c6180_full_length_coverage_2_non_full_length_coverage_6_isoform_length_2828_28427 | ---NA--- | | |
| 1710\|_\|c1761_f2p33_2827_isoform_c1761_full_length_coverage_2_non_full_length_coverage_33_isoform_length_2827_25465 | ---NA--- | | |
| 1711\|_\|c28360_f2p1_2827_isoform_c28360_full_length_coverage_2_non_full_length_coverage_1_isoform_length_2827_23078 | XP_018023395.1 zinc finger A20 and AN1 domain-containing stress-associated protein 9-like | | |
| 1712\|_\|c9231_f2p0_2827_isoform_c9231_full_length_coverage_2_non_full_length_coverage_0_isoform_length_2827_12785 | XP_021913454.1oxysterol-binding protein-related protein 1-like | | |
| 1713\|_\|c10120_f2p4_2826_isoform_c10120_full_length_coverage_2_non_full_length_coverage_4_isoform_length_2826_28547 | ---NA--- | | |
| TRINITY_DN16144_c0_g1_i1_len_3489_95.p1_type_5prime_partial_len_942 | ---NA--- | | |
| TRINITY_DN32446_c0_g1_i1_len_490_204544.p1_type_internal_len_164 | ---NA--- | | |
| TRINITY_DN13326_c0_g1_i1_len_490_69391.p3_type_internal_len_164 | ---NA--- | | |
| TRINITY_DN13501_c0_g1_i2_len_493_304331.p1_type_internal_len_164 | ---NA--- | | |
| TRINITY_DN7796_c0_g1_i1_len_489_167336.p1_type_internal_len_164 | XP_018016351.1 trimethyllysine dioxygenase, mitochondrial-like isoform X2 | | |
| TRINITY_DN23104_c0_g1_i1_len_2163_59012.p1_type_complete_len_163 | XP_014661459.1 peptidyl-prolyl cis-trans isomerase FKBP1A-like | P:GO:0000413; F:GO:0003755; C:GO:0005737; P:GO:0061077 | EC:5.2.1.8 |
| TRINITY_DN14863_c0_g1_i1_len_776_5045.p1_type_5prime_partial_len_163 | ---NA--- | | |

| | | | |
|---|---|---|---|
| TRINITY_DN25784_c0_g1_i1_len_492_69329.p1_type_internal_len_164 | ---NA--- | | |
| TRINITY_DN5472_c0_g1_i1_len_490_167316.p1_type_internal_len_164 | ---NA--- | | |
| TRINITY_DN14642_c0_g1_i2_len_791_34850.p1_type_complete_len_163 | ---NA--- | | |
| TRINITY_DN14870_c0_g1_i1_len_493_101462.p1_type_internal_len_164 | ---NA--- | | |
| TRINITY_DN18623_c0_g1_i1_len_590_201982.p1_type_5prime_partial_len_163 | XP_018024103.1 organic solute transporter subunit alpha-like | | |
| TRINITY_DN14541_c0_g1_i2_len_490_69385.p1_type_internal_len_164 | ---NA--- | | |
| TRINITY_DN8930_c0_g1_i1_len_490_204568.p1_type_internal_len_164 | ---NA--- | | |
| TRINITY_DN37578_c0_g1_i1_len_507_204025.p1_type_5prime_partial_len_163 | ACI96113.1A-type potassium channel | F:GO:0005249; C:GO:0008076; P:GO:0034765; P:GO:0051260; P:GO:0071805 | |
| TRINITY_DN33173_c0_g1_i1_len_490_204545.p1_type_internal_len_164 | AUI80370.1cryptochrome 1 | | |
| TRINITY_DN18037_c0_g1_i1_len_768_162748.p1_type_5prime_partial_len_163 | AEL79850.1thioesterase superfamily member 2 | F:GO:0047617 | EC:3.1.2.20 |
| TRINITY_DN16262_c0_g1_i1_len_1208_234645.p1_type_complete_len_163 | XP_018324751.1protein charybde | C:GO:0005737; P:GO:0009968 | |
| TRINITY_DN22044_c0_g1_i1_len_5448_227699.p1_type_complete_len_163 | ---NA--- | | |
| TRINITY_DN16127_c0_g1_i1_len_911_96186.p2_type_complete_len_163 | XP_018009825.1PREDICTED: uncharacterized protein LOC108667321 | | |
| TRINITY_DN15839_c0_g1_i1_len_792_162500.p1_type_complete_len_163 | ---NA--- | | |
| TRINITY_DN19111_c0_g1_i2_len_3864_153736.p2_type_5prime_partial_len_163 | ---NA--- | | |
| TRINITY_DN20796_c2_g1_i10_len_1748_266927.p2_type_5prime_partial_len_163 | ---NA--- | | |

| | | | |
|---|---|---|---|
| TRINITY_DN27485_c0_g1_i1_len_492_69330.p1_type_internal_len_164 | ---NA--- | | |
| TRINITY_DN22135_c17_g6_i1_len_493_101469.p1_type_internal_len_164 | AGI56293.2thrombospondin II | F:GO:0005509; C:GO:0005576; P:GO:0006030; P:GO:0007155; F:GO:0008061 | |
| TRINITY_DN274_c0_g1_i1_len_762_128343.p1_type_5prime_partial_len_163 | AFM75821.1myosin light chain 2 | F:GO:0005509 | |
| TRINITY_DN19728_c0_g1_i1_len_1595_267412.p2_type_complete_len_163 | ---NA--- | | |
| TRINITY_DN2548_c0_g1_i1_len_579_241176.p1_type_3prime_partial_len_164 | EZA47934.1Inactive ubiquitin carboxyl-terminal hydrolase | P:GO:0016579; F:GO:0036459 | EC:3.4.19.12 |
| TRINITY_DN20612_c0_g1_i1_len_491_204523.p2_type_internal_len_164 | XP_013778566.2otoancorin-like | | |
| TRINITY_DN20408_c1_g1_i11_len_810_34712.p1_type_3prime_partial_len_164 | XP_012678471.1PREDICTED: uncharacterized protein LOC105896272 | | |
| TRINITY_DN1498_c0_g1_i1_len_740_199436.p1_type_5prime_partial_len_163 | ---NA--- | | |
| TRINITY_DN23298_c0_g1_i1_len_858_197954.p1_type_complete_len_163 | ---NA--- | | |
| TRINITY_DN13679_c0_g1_i3_len_536_242111.p1_type_5prime_partial_len_163 | ---NA--- | | |
| TRINITY_DN33373_c0_g1_i1_len_491_204526.p1_type_internal_len_164 | XP_023337716.1apolipoprotein D-like | | |
| TRINITY_DN18254_c0_g1_i3_len_492_9017.p1_type_internal_len_164 | EZA47852.1hypothetical protein X777_15171 | | |
| TRINITY_DN42076_c0_g1_i1_len_491_204529.p1_type_internal_len_164 | ---NA--- | | |
| TRINITY_DN18114_c0_g1_i1_len_986_196675.p1_type_complete_len_163 | ---NA--- | | |
| TRINITY_DN16150_c0_g1_i1_len_804_198562.p1_type_5prime_partial_len_163 | ---NA--- | | |
| TRINITY_DN16081_c0_g1_i2_len_2163_91873.p2_type_complete_len_163 | ---NA--- | | |

| | | | |
|---|---|---|---|
| TRINITY_DN31402_c0_g1_i1_len_540_303385.p1_type_5prime_partial_len_163 | ---NA--- | | |
| TRINITY_DN27308_c0_g1_i1_len_1371_193833.p1_type_5prime_partial_len_163 | XP_014678597.1 zinc finger protein 271-like isoform X2 | | |
| TRINITY_DN25128_c0_g1_i1_len_764_199068.p1_type_5prime_partial_len_163 | XP_023327479.1probable NADH dehydrogenase [ubiquinone] 1 alpha subcomplex subunit 12 | C:GO:0005739; C:GO:0016020; F:GO:0016491; P:GO:0055114 | |
| TRINITY_DN39929_c0_g1_i1_len_515_203786.p1_type_5prime_partial_len_163 | KRY99450.1hypothetical protein T11_12262, partial | | |
| TRINITY_DN6875_c0_g1_i1_len_493_243199.p1_type_internal_len_164 | XP_018010735.1 multiple epidermal growth factor-like domains protein 8 | F:GO:0005509; C:GO:0016020; C:GO:0016021 | |
| TRINITY_DN21622_c0_g1_i3_len_490_204569.p1_type_internal_len_164 | ---NA--- | | |
| TRINITY_DN29284_c0_g1_i1_len_493_243212.p1_type_internal_len_164 | XP_016769052.2WD repeat-containing protein 7 isoform X14 | | |
| TRINITY_DN35294_c0_g1_i1_len_911_197375.p1_type_5prime_partial_len_163 | XP_014249066.1F-box/LRR-repeat protein 6 | P:GO:0016567; C:GO:0019005; P:GO:0031146; F:GO:0061630 | |
| TRINITY_DN23792_c0_g1_i1_len_492_204509.p1_type_internal_len_164 | ---NA--- | | |
| TRINITY_DN41471_c0_g1_i1_len_558_202692.p1_type_5prime_partial_len_163 | OAJ41130.1FK506-binding protein 2B | P:GO:0000413; F:GO:0003755; F:GO:0016853 | |
| TRINITY_DN24076_c0_g1_i8_len_2508_229762.p2_type_complete_len_163 | ---NA--- | | |
| TRINITY_DN14805_c0_g1_i1_len_492_276235.p1_type_internal_len_164 | ---NA--- | | |
| TRINITY_DN39323_c0_g1_i1_len_490_204560.p1_type_internal_len_164 | ---NA--- | | |
| TRINITY_DN28742_c0_g1_i1_len_492_243259.p1_type_internal_len_164 | XP_022251497.1DNA replication ATP-dependent helicase/nuclease DNA2-like isoform X3 | | |
| TRINITY_DN7109_c0_g1_i1_len_490_204552.p1_type_internal_len_164 | ---NA--- | | |

| | | | |
|---|---|---|---|
| TRINITY_DN12730_c0_g1_i1_len_489_204604.p1_type_internal_len_164 | BAU19444.1serine racemase | P:GO:0006520; F:GO:0030170 | |
| TRINITY_DN8433_c0_g1_i2_len_492_243244.p1_type_internal_len_164 | ---NA--- | | |
| TRINITY_DN2897_c0_g1_i1_len_492_243256.p1_type_internal_len_164 | ---NA--- | | |
| TRINITY_DN10608_c0_g1_i2_len_641_66457.p1_type_5prime_partial_len_163 | ---NA--- | | |
| TRINITY_DN1651_c0_g1_i1_len_490_204556.p1_type_internal_len_164 | ---NA--- | | |
| TRINITY_DN13833_c0_g1_i1_len_572_99905.p1_type_3prime_partial_len_164 | ---NA--- | | |
| TRINITY_DN1695_c0_g1_i1_len_547_68067.p1_type_3prime_partial_len_164 | XP_018017072.1 synaptic vesicle glycoprotein 2B-like | C:GO:0016021; F:GO:0022857; P:GO:0055085 | |
| TRINITY_DN16058_c0_g1_i1_len_648_239981.p1_type_5prime_partial_len_163 | ---NA--- | | |
| TRINITY_DN15309_c0_g1_i1_len_492_9003.p1_type_3prime_partial_len_164 | ---NA--- | | |
| TRINITY_DN19281_c0_g1_i1_len_1640_92943.p2_type_complete_len_163 | EKC39792.1Short-chain specific acyl-CoA dehydrogenase, mitochondrial | F:GO:0003995; F:GO:0050660; P:GO:0055114 | EC:1.3.99.3 |
| TRINITY_DN15284_c0_g1_i3_len_490_304391.p1_type_internal_len_164 | ---NA--- | | |
| TRINITY_DN4695_c0_g1_i1_len_491_204514.p1_type_internal_len_164 | XP_016950805.1 60S ribosomal protein L34 | F:GO:0003735; C:GO:0005840; P:GO:0006412 | |
| TRINITY_DN10756_c0_g1_i1_len_632_240251.p1_type_3prime_partial_len_164 | XP_018020177.1 syndetin-like | | |
| TRINITY_DN13360_c0_g1_i1_len_489_101562.p1_type_internal_len_164 | ---NA--- | | |
| TRINITY_DN6471_c0_g1_i1_len_489_276302.p1_type_internal_len_164 | ---NA--- | | |
| TRINITY_DN36793_c0_g1_i1_len_492_204486.p1_type_internal_len_164 | ---NA--- | | |

| | | | |
|---|---|---|---|
| TRINITY_DN9996_c0_g1_i1_len_492_204489.p1_type_internal_len_164 | XP_023222078.1N-acetylated-alpha-linked acidic dipeptidase 2-like | C:GO:0016020; C:GO:0016021 | |
| TRINITY_DN33101_c0_g1_i1_len_490_204547.p1_type_internal_len_164 | ---NA--- | | |
| TRINITY_DN13958_c0_g1_i1_len_491_204517.p2_type_internal_len_164 | ---NA--- | | |
| TRINITY_DN8027_c0_g1_i1_len_491_132586.p1_type_internal_len_164 | XP_022238379.1serine/threonine-protein phosphatase 4 regulatory subunit 4-like isoform X3 | | |
| TRINITY_DN9184_c0_g1_i1_len_627_302010.p1_type_3prime_partial_len_164 | ---NA--- | | |
| TRINITY_DN13777_c0_g1_i1_len_970_270396.p1_type_5prime_partial_len_163 | ---NA--- | | |
| TRINITY_DN17987_c0_g1_i2_len_651_36286.p2_type_5prime_partial_len_163 | ---NA--- | | |
| TRINITY_DN9111_c0_g1_i1_len_490_243302.p1_type_internal_len_164 | ---NA--- | | |
| TRINITY_DN23396_c0_g2_i1_len_2670_229489.p2_type_5prime_partial_len_163 | ---NA--- | | |
| TRINITY_DN36568_c0_g1_i1_len_491_243274.p1_type_internal_len_164 | XP_018016939.1 neurexin-4-like isoform X2 | | |
| TRINITY_DN21192_c0_g1_i1_len_1262_125161.p1_type_complete_len_163 | XP_026686678.1EH domain-binding protein 1-like isoform X1 | | |
| TRINITY_DN963_c0_g1_i1_len_491_243278.p1_type_internal_len_164 | ---NA--- | | |
| TRINITY_DN4792_c0_g1_i1_len_491_243285.p1_type_internal_len_164 | XP_018017374.1 fat-like cadherin-related tumor suppressor homolog | | |
| TRINITY_DN41343_c0_g1_i1_len_489_204575.p1_type_internal_len_164 | KFM72382.1Nuclear receptor subfamily 2 group C member 2 | F:GO:0003677; F:GO:0046872; P:GO:0050794 | |
| TRINITY_DN35009_c0_g1_i1_len_644_200916.p1_type_3prime_partial_len_164 | XP_018012983.1 cilia- and flagella-associated protein 57-like | | |
| TRINITY_DN27928_c0_g1_i1_len_490_243309.p1_type_internal_len_164 | AAC64660.1pacifastin heavy chain precursor | C:GO:0005576 | |

| | | | |
|---|---|---|---|
| TRINITY_DN23513_c0_g2_i2_len_2209_230482.p1_type_complete_len_162 | ---NA--- | | |
| TRINITY_DN16636_c0_g1_i1_len_1015_126459.p2_type_complete_len_162 | KDR12721.1Folliculin-interacting protein 2 | | |
| TRINITY_DN15532_c0_g1_i1_len_676_200375.p1_type_complete_len_162 | XP_002741445.1 D-tyrosyl-tRNA(Tyr) deacylase 1-like | F:GO:0000049; C:GO:0005737; F:GO:0051500; P:GO:0106074 | EC:3.1.1.96; EC:3.1.1.1 |
| TRINITY_DN17000_c0_g1_i1_len_489_69406.p1_type_internal_len_163 | ---NA--- | | |
| TRINITY_DN17138_c0_g1_i1_len_1117_125855.p2_type_5prime_partial_len_162 | XP_013091507.1 L-galactose dehydrogenase-like | F:GO:0016491; P:GO:0055114 | |
| TRINITY_DN16162_c0_g1_i1_len_711_199825.p1_type_5prime_partial_len_162 | ---NA--- | | |
| TRINITY_DN7906_c0_g1_i1_len_591_201967.p2_type_5prime_partial_len_162 | XP_018027063.1 pleckstrin homology domain-containing family A member 3-like | | |
| TRINITY_DN30685_c0_g1_i1_len_595_240896.p1_type_3prime_partial_len_163 | XP_015922477.1WD repeat-containing protein 48-like isoform X2 | | |
| TRINITY_DN1659_c0_g1_i1_len_956_299045.p3_type_5prime_partial_len_162 | XP_026282602.1adapter molecule Crk | | |
| TRINITY_DN33609_c0_g1_i1_len_676_200372.p1_type_5prime_partial_len_162 | ---NA--- | | |
| TRINITY_DN26636_c0_g1_i1_len_539_242036.p1_type_3prime_partial_len_163 | XP_023712511.1uncharacterized protein LOC111867156 isoform X2 | | |
| TRINITY_DN3037_c0_g1_i1_len_489_204608.p1_type_internal_len_163 | XP_013413082.1E3 ubiquitin-protein ligase UBR4 | F:GO:0008270; F:GO:0016874 | |
| TRINITY_DN13682_c0_g1_i1_len_490_304380.p1_type_internal_len_163 | EUB62980.1Tubulin beta-4 chain | F:GO:0005200; F:GO:0005525; C:GO:0005874; P:GO:0007010; P:GO:0007017 | |
| TRINITY_DN24453_c0_g1_i1_len_551_67994.p1_type_5prime_partial_len_162 | XP_023235940.1protein archease-like | P:GO:0006388; C:GO:0072669 | |
| TRINITY_DN9175_c0_g1_i1_len_489_204592.p1_type_internal_len_163 | ---NA--- | | |

| | | | |
|---|---|---|---|
| TRINITY_DN21616_c0_g1_i1_len_2828_58297.p1_type_internal_len_943 | XP_023719107.1Down syndrome cell adhesion molecule-like protein Dscam2 | C:GO:0016020; C:GO:0016021 | |
| 1716\|_\|c2537_f2p2_2824_isoform_c2537_full_length_coverage_2_non_full_length_coverage_2_isoform_length_2824_21023 | XP_018015523.1 protein FAM98B-like isoform X2 | | |
| TRINITY_DN26198_c0_g1_i1_len_4663_227890.p1_type_5prime_partial_len_941 | XP_023230853.1sorting nexin-14-like | | |
| TRINITY_DN20273_c0_g1_i5_len_3043_29464.p1_type_complete_len_941 | XP_018024960.1 U2 snRNP-associated SURP motif-containing protein-like | | |
| TRINITY_DN30733_c1_g1_i2_len_5580_186998.p1_type_complete_len_941 | KDR17529.1WD repeat-containing protein 22 | | |
| 1720\|_\|c1816_f9p14_2822_isoform_c1816_full_length_coverage_9_non_full_length_coverage_14_isoform_length_2822_16018 | ---NA--- | | |
| 1721\|_\|c4387_f2p5_2821_isoform_c4387_full_length_coverage_2_non_full_length_coverage_5_isoform_length_2821_12374 | AKK75090.1DNA (cytosine-5-)-methyltransferase 1 | F:GO:0003677; F:GO:0003682; F:GO:0003886; C:GO:0005634; F:GO:0008270; P:GO:0090116 | EC:2.1.1.37 |
| 1722\|_\|c24114_f2p3_2821_isoform_c24114_full_length_coverage_2_non_full_length_coverage_3_isoform_length_2821_22927 | ABV65906.1sodium/potassium-transporting ATPase subunit alpha | F:GO:0005524; F:GO:0008556; C:GO:0016021; F:GO:0046872; P:GO:0071805; P:GO:0099132 | EC:3.6.1.3; EC:3.6.3.12; EC:3.6.1.15 |
| 1724\|_\|c7889_f2p4_2821_isoform_c7889_full_length_coverage_2_non_full_length_coverage_4_isoform_length_2821_12671 | ---NA--- | | |
| 1725\|_\|c17892_f2p1_2820_isoform_c17892_full_length_coverage_2_non_full_length_coverage_1_isoform_length_2820_22545 | XP_018028095.1 protein FAM63A-like | C:GO:0005829; F:GO:0016807; P:GO:0071108; F:GO:1990380 | EC:3.4.18 |
| TRINITY_DN15268_c0_g1_i1_len_487_204659.p1_type_internal_len_163 | XP_023218239.1neogenin-like isoform X2 | | |
| TRINITY_DN17696_c0_g1_i3_len_599_240824.p1_type_5prime_partial_len_162 | ---NA--- | | |
| TRINITY_DN3397_c0_g1_i1_len_746_238574.p1_type_complete_len_162 | ---NA--- | | |

| | | | |
|---|---|---|---|
| TRINITY_DN17532_c0_g1_i1_len_489_69393.p1_type_internal_len_163 | ---NA--- | | |
| TRINITY_DN23821_c0_g1_i1_len_490_243298.p1_type_internal_len_163 | ---NA--- | | |
| TRINITY_DN416_c0_g1_i1_len_490_243299.p1_type_internal_len_163 | XP_018006463.1PREDICTED: LOW QUALITY PROTEIN: uncharacterized protein LOC108664398 | C:GO:0016020 | |
| TRINITY_DN25409_c0_g1_i1_len_2925_229096.p2_type_5prime_partial_len_162 | ---NA--- | | |
| TRINITY_DN34920_c0_g1_i1_len_488_243352.p1_type_internal_len_163 | ---NA--- | | |
| TRINITY_DN2619_c0_g1_i1_len_488_243353.p1_type_internal_len_163 | XP_018016349.1 protein KIAA0100-like | | |
| TRINITY_DN7770_c0_g1_i1_len_488_243356.p1_type_internal_len_163 | ---NA--- | | |
| TRINITY_DN35582_c0_g1_i1_len_486_204682.p1_type_internal_len_163 | ---NA--- | | |
| TRINITY_DN7180_c0_g1_i1_len_488_204620.p1_type_internal_len_163 | ---NA--- | | |
| TRINITY_DN18583_c0_g1_i1_len_1049_160314.p1_type_5prime_partial_len_162 | XP_022238495.1transcription factor VBP-like | F:GO:0003700; P:GO:0006357 | |
| TRINITY_DN38927_c0_g1_i1_len_490_204550.p1_type_internal_len_163 | ---NA--- | | |
| TRINITY_DN7749_c0_g1_i1_len_490_243316.p1_type_internal_len_163 | AAC61691.1inositol 1,4,5-trisphosphate receptor | F:GO:0005220; C:GO:0005783; C:GO:0016021; P:GO:0048016; P:GO:0070588; F:GO:0070679 | |
| TRINITY_DN13127_c0_g1_i1_len_488_243343.p1_type_internal_len_163 | ---NA--- | | |
| TRINITY_DN12748_c0_g1_i1_len_632_201154.p1_type_5prime_partial_len_162 | ---NA--- | | |
| TRINITY_DN37575_c0_g1_i1_len_490_204564.p1_type_internal_len_163 | ---NA--- | | |

| | | | |
|---|---|---|---|
| TRINITY_DN25726_c1_g1_i1_len_503_242961.p1_type_5prime_partial_len_162 | XP_018121571.1 zinc finger protein 513-like | F:GO:0003676 | |
| TRINITY_DN2668_c0_g1_i1_len_490_204561.p1_type_internal_len_163 | ---NA--- | | |
| TRINITY_DN14352_c0_g1_i1_len_744_199373.p1_type_complete_len_162 | PSN47434.1Mitochondrial fission process protein 1 | | |
| TRINITY_DN20976_c0_g1_i2_len_2683_229461.p3_type_5prime_partial_len_162 | ---NA--- | | |
| TRINITY_DN30854_c0_g1_i1_len_3756_187733.p2_type_complete_len_162 | XP_018026166.1 probable phospholipid-transporting ATPase IIA | | |
| TRINITY_DN16271_c0_g1_i1_len_490_204558.p1_type_internal_len_163 | ---NA--- | | |
| TRINITY_DN21665_c0_g1_i1_len_1600_93050.p1_type_complete_len_162 | ---NA--- | | |
| TRINITY_DN30688_c0_g1_i1_len_4553_187287.p3_type_5prime_partial_len_162 | ---NA--- | | |
| TRINITY_DN19912_c0_g1_i1_len_487_69464.p1_type_internal_len_163 | ---NA--- | | |
| TRINITY_DN23284_c0_g1_i1_len_1194_195004.p2_type_5prime_partial_len_162 | XP_022904308.1ecto-NOX disulfide-thiol exchanger 2 isoform X2 | F:GO:0003723; P:GO:0007624; C:GO:0009897; F:GO:0016491; P:GO:0055114 | |
| TRINITY_DN1840_c0_g1_i2_len_864_161808.p1_type_5prime_partial_len_162 | BBC28099.1xanthine dehydrogenase | F:GO:0004854; F:GO:0004855; F:GO:0005506; F:GO:0009055; P:GO:0022900; F:GO:0051537; F:GO:0071949 | EC:1.17.3; EC:1.17.1; EC:1.17.1.4; EC:1.17.3.2 |
| TRINITY_DN17171_c0_g1_i1_len_3649_57903.p2_type_complete_len_162 | ---NA--- | | |
| TRINITY_DN37138_c0_g1_i1_len_488_204623.p1_type_internal_len_163 | ---NA--- | | |
| TRINITY_DN15012_c0_g1_i4_len_489_69422.p1_type_internal_len_163 | ---NA--- | | |
| TRINITY_DN814_c0_g1_i1_len_535_203289.p1_type_5prime_partial_len_162 | ---NA--- | | |

| | | | |
|---|---|---|---|
| TRINITY_DN19629_c0_g1_i1_len_1173_269206.p1_type_3prime_partial_len_163 | ---NA--- | | |
| TRINITY_DN20787_c0_g1_i3_len_912_299335.p1_type_5prime_partial_len_162 | ---NA--- | | |
| TRINITY_DN20576_c0_g1_i1_len_1044_196154.p1_type_complete_len_162 | ---NA--- | | |
| TRINITY_DN18527_c0_g1_i1_len_489_204578.p1_type_internal_len_163 | ---NA--- | | |
| TRINITY_DN12733_c1_g1_i1_len_487_167382.p1_type_internal_len_163 | XP_018024684.1 protein unc-13 homolog A-like | F:GO:0005509; F:GO:0005543; P:GO:0007268; F:GO:0019992; P:GO:0035556 | |
| TRINITY_DN26973_c0_g1_i1_len_487_243368.p1_type_internal_len_163 | ANQ44705.1heat shock protein 70 kDa | F:GO:0005524 | |
| TRINITY_DN18736_c0_g1_i2_len_665_200551.p1_type_complete_len_162 | ---NA--- | | |
| TRINITY_DN15716_c0_g1_i3_len_559_67842.p1_type_3prime_partial_len_163 | ---NA--- | | |
| TRINITY_DN20652_c0_g1_i1_len_489_204577.p1_type_internal_len_163 | ---NA--- | | |
| TRINITY_DN32780_c0_g1_i1_len_487_243381.p1_type_internal_len_163 | ---NA--- | | |
| TRINITY_DN7846_c0_g1_i1_len_486_204668.p1_type_internal_len_163 | XP_018006676.1 glutamate receptor ionotropic, delta-2-like | | |
| TRINITY_DN11827_c0_g1_i1_len_489_204586.p1_type_internal_len_163 | AYC12381.1bone morphogenetic protein receptor type 1B | F:GO:0004675; F:GO:0005524; P:GO:0006468; P:GO:0007178; C:GO:0016021; F:GO:0046872 | EC:2.7.11 |
| TRINITY_DN8250_c0_g1_i1_len_486_204691.p1_type_internal_len_163 | ---NA--- | | |
| TRINITY_DN36912_c0_g1_i1_len_488_204619.p1_type_internal_len_163 | KXJ06669.1Retrovirus-related Pol polyprotein from transposon 17.6 | | |
| TRINITY_DN37636_c0_g1_i1_len_486_204670.p1_type_internal_len_163 | RAW24156.1hypothetical protein PC110_g19418 | | |

| | | | |
|---|---|---|---|
| TRINITY_DN4023_c0_g1_i1_len_488_167357.p1_type_internal_len_163 | ---NA--- | | |
| TRINITY_DN10075_c0_g1_i1_len_489_167340.p1_type_internal_len_163 | ---NA--- | | |
| TRINITY_DN20195_c0_g1_i1_len_802_97018.p3_type_complete_len_162 | ACN38704.1heat shock protein 70 | F:GO:0005524 | |
| TRINITY_DN30718_c0_g1_i1_len_489_167321.p1_type_internal_len_163 | ---NA--- | | |
| TRINITY_DN12022_c0_g1_i1_len_486_9167.p2_type_internal_len_163 | ---NA--- | | |
| TRINITY_DN11298_c0_g1_i1_len_507_68935.p1_type_5prime_partial_len_162 | ---NA--- | | |
| TRINITY_DN22785_c0_g1_i1_len_795_238009.p3_type_5prime_partial_len_162 | ---NA--- | | |
| TRINITY_DN16395_c0_g1_i1_len_674_163889.p1_type_complete_len_162 | ---NA--- | | |
| TRINITY_DN19780_c0_g1_i1_len_1790_266801.p2_type_5prime_partial_len_162 | KDR22713.1Cytosolic carboxypeptidase 2 | F:GO:0004180; P:GO:0006508 | |
| TRINITY_DN10046_c0_g1_i1_len_487_9147.p1_type_internal_len_163 | XP_018009231.1PREDICTED: uncharacterized protein LOC108666815 | | |
| TRINITY_DN28897_c0_g1_i4_len_1829_191561.p2_type_5prime_partial_len_162 | ---NA--- | | |
| TRINITY_DN17036_c0_g1_i2_len_564_165635.p1_type_5prime_partial_len_162 | OXA46791.1Protein big brother | F:GO:0003713; C:GO:0005634; P:GO:1903508 | |
| TRINITY_DN4872_c0_g1_i1_len_486_276361.p1_type_internal_len_163 | ---NA--- | | |
| TRINITY_DN3227_c0_g1_i1_len_638_201030.p1_type_5prime_partial_len_162 | XP_023704003.1oxysterol-binding protein-related protein 1-like isoform X2 | | |
| TRINITY_DN25848_c0_g3_i1_len_573_241309.p1_type_3prime_partial_len_163 | ---NA--- | | |
| TRINITY_DN13050_c0_g1_i1_len_487_132691.p2_type_internal_len_163 | ---NA--- | | |

| | | | |
|---|---|---|---|
| TRINITY_DN10645_c0_g1_i1_len_501_204202.p1_type_5prime_partial_len_162 | ---NA--- | | |
| TRINITY_DN23278_c0_g1_i1_len_490_276276.p1_type_5prime_partial_len_162 | ---NA--- | | |
| TRINITY_DN18814_c0_g1_i1_len_487_39002.p2_type_internal_len_163 | XP_018018358.1PREDICTED: uncharacterized protein LOC108674884 | | |
| TRINITY_DN14185_c0_g1_i1_len_1056_235674.p1_type_5prime_partial_len_162 | ---NA--- | | |
| TRINITY_DN13328_c0_g1_i3_len_822_271551.p2_type_5prime_partial_len_162 | ---NA--- | | |
| TRINITY_DN18879_c0_g1_i1_len_933_197152.p2_type_3prime_partial_len_163 | XP_018009540.1 integrator complex subunit 6-like | | |
| TRINITY_DN26584_c0_g2_i1_len_552_202815.p1_type_3prime_partial_len_163 | ---NA--- | | |
| TRINITY_DN16638_c0_g1_i1_len_1836_191533.p2_type_3prime_partial_len_163 | ---NA--- | | |
| TRINITY_DN26272_c0_g1_i1_len_486_304488.p1_type_internal_len_163 | ---NA--- | | |
| TRINITY_DN1002_c0_g1_i1_len_756_199189.p1_type_complete_len_162 | ---NA--- | | |
| TRINITY_DN2652_c0_g1_i1_len_896_236991.p1_type_5prime_partial_len_162 | ---NA--- | | |
| TRINITY_DN3127_c0_g1_i1_len_670_200466.p1_type_3prime_partial_len_163 | XP_022257629.1tyrosine-protein phosphatase Lar-like | | |
| TRINITY_DN20641_c0_g1_i1_len_1350_233773.p3_type_complete_len_162 | ---NA--- | | |
| TRINITY_DN25095_c0_g1_i1_len_2098_230795.p3_type_5prime_partial_len_161 | ---NA--- | | |
| TRINITY_DN35198_c0_g1_i1_len_483_204774.p1_type_internal_len_162 | ---NA--- | | |
| TRINITY_DN2197_c0_g1_i1_len_486_9175.p1_type_internal_len_162 | ---NA--- | | |

| | | | |
|---|---|---|---|
| TRINITY_DN24233_c0_g1_i9_len_3132_58085.p2_type_complete_len_161 | ---NA--- | | |
| TRINITY_DN27878_c0_g1_i1_len_485_132737.p1_type_internal_len_162 | ---NA--- | | |
| TRINITY_DN21336_c0_g1_i2_len_1135_2890.p1_type_5prime_partial_len_161 | PSN31119.1Structure-specific endonuclease subunit slx1 | P:GO:0006281; P:GO:0006310; F:GO:0017108; C:GO:0033557; P:GO:0090305 | EC:3.1.30; EC:3.1.21 |
| TRINITY_DN127_c0_g1_i1_len_564_130993.p1_type_5prime_partial_len_161 | ---NA--- | | |
| TRINITY_DN13267_c0_g1_i1_len_485_243423.p1_type_internal_len_162 | ---NA--- | | |
| TRINITY_DN33211_c0_g1_i1_len_486_167408.p1_type_3prime_partial_len_162 | PSN48792.1Collagen alpha-1(IV) chain | F:GO:0005201; C:GO:0005581 | |
| TRINITY_DN321_c0_g1_i1_len_483_69585.p1_type_internal_len_162 | ---NA--- | | |
| TRINITY_DN1633_c0_g1_i1_len_485_167426.p1_type_internal_len_162 | XP_012230614.1PREDICTED: uncharacterized protein LOC105676960 | C:GO:0005576; P:GO:0006030; F:GO:0008061 | |
| TRINITY_DN5165_c0_g1_i1_len_483_69577.p1_type_internal_len_162 | XP_018013614.1PREDICTED: uncharacterized protein LOC108670650 | | |
| TRINITY_DN5779_c0_g1_i1_len_484_243460.p1_type_internal_len_162 | XP_018015532.1 ras-specific guanine nucleotide-releasing factor RalGPS1-like | | |
| TRINITY_DN12379_c0_g1_i1_len_484_9221.p1_type_internal_len_162 | XP_022095908.1piggyBac transposable element-derived protein 4-like | | |
| TRINITY_DN36465_c0_g1_i1_len_484_243468.p1_type_internal_len_162 | XP_025110241.1protein tilB homolog | | |
| TRINITY_DN364_c0_g1_i1_len_484_243471.p1_type_internal_len_162 | ---NA--- | | |
| TRINITY_DN3353_c0_g1_i1_len_485_243436.p1_type_internal_len_162 | XP_018020402.1 transcription factor AP-2-epsilon-like isoform X2 | | |
| TRINITY_DN1476_c0_g1_i1_len_485_243450.p1_type_3prime_partial_len_162 | XP_018009204.1 delta(7)-sterol 5(6)-desaturase erg32-like isoform X1 | | |
| TRINITY_DN23249_c0_g1_i1_len_583_241121.p1_type_5prime_partial_len_161 | ---NA--- | | |

| | | | |
|---|---|---|---|
| TRINITY_DN41025_c0_g1_i1_len_487_204650.p1_type_internal_len_162 | ---NA--- | | |
| TRINITY_DN4553_c0_g1_i1_len_1140_235113.p2_type_complete_len_161 | ---NA--- | | |
| TRINITY_DN35017_c0_g1_i1_len_485_204722.p1_type_internal_len_162 | XP_018025400.1 calcium-dependent secretion activator-like | | |
| TRINITY_DN31082_c0_g1_i1_len_486_243401.p1_type_internal_len_162 | XP_018006424.1 sorting nexin-13-like | | |
| TRINITY_DN19628_c0_g1_i3_len_3640_153831.p1_type_5prime_partial_len_940 | XP_018015790.1 heterogeneous nuclear ribonucleoprotein U-like protein 1 | | |
| TRINITY_DN23441_c0_g1_i1_len_3144_188317.p1_type_complete_len_940 | XP_021003811.1protein FAN-like | | |
| 1728\|_\|c19812_f2p4_2820_isoform_c19812_full_length_coverage_2_non_full_length_coverage_4_isoform_length_2820_28993 | ---NA--- | | |
| 1729\|_\|c35020_f2p2_2819_isoform_c35020_full_length_coverage_2_non_full_length_coverage_2_isoform_length_2819_14425 | XP_018007337.1 exportin-7-A-like | C:GO:0005634; P:GO:0006886; F:GO:0008536; P:GO:0051169 | |
| 1730\|_\|c25347_f2p0_2818_isoform_c25347_full_length_coverage_2_non_full_length_coverage_0_isoform_length_2818_18354 | ---NA--- | | |
| 1731\|_\|c2974_f2p2_2818_isoform_c2974_full_length_coverage_2_non_full_length_coverage_2_isoform_length_2818_16727 | ---NA--- | | |
| TRINITY_DN6653_c0_g1_i1_len_3026_228963.p1_type_complete_len_939 | XP_018006440.1PREDICTED: uncharacterized protein LOC108664371 | | |
| TRINITY_DN18381_c0_g1_i1_len_2993_29488.p1_type_complete_len_939 | XP_018013306.1 C-1-tetrahydrofolate synthase, cytoplasmic-like isoform X1 | F:GO:0004329; F:GO:0004488; F:GO:0005524; P:GO:0055114 | EC:6.3.4.3; EC:1.5.1.5 |
| 1734\|_\|c2660_f3p2_2817_isoform_c2660_full_length_coverage_3_non_full_length_coverage_2_isoform_length_2817_21128 | XP_018026730.1 UDP-glucose:glycoprotein glucosyltransferase 1-like isoform X2 | | |
| TRINITY_DN30558_c0_g1_i4_len_2982_188567.p1_type_3prime_partial_len_940 | XP_026054784.1uncharacterized protein LOC113040739 | | |

| | | | |
|---|---|---|---|
| TRINITY_DN713_c0_g1_i1_len_485_167437.p1_type_internal_len_162 | ---NA--- | | |
| TRINITY_DN20223_c0_g1_i2_len_552_7789.p1_type_5prime_partial_len_161 | ---NA--- | | |
| TRINITY_DN22185_c0_g1_i2_len_675_65988.p1_type_5prime_partial_len_161 | ---NA--- | | |
| TRINITY_DN36058_c0_g1_i1_len_486_204697.p1_type_internal_len_162 | XP_015180346.1 nuclear factor of activated T-cells 5-like isoform X3 | | |
| TRINITY_DN22340_c0_g1_i2_len_487_9140.p1_type_internal_len_162 | ---NA--- | | |
| TRINITY_DN16512_c0_g1_i1_len_515_68733.p1_type_3prime_partial_len_162 | ---NA--- | | |
| TRINITY_DN30259_c1_g1_i1_len_2800_188856.p1_type_complete_len_161 | ---NA--- | | |
| TRINITY_DN26464_c0_g1_i1_len_1331_194073.p1_type_complete_len_161 | KZS03013.1Cytidine deaminase | F:GO:0004126; C:GO:0005829; F:GO:0008270; P:GO:0009972 | EC:3.5.4.5 |
| TRINITY_DN5458_c0_g1_i1_len_486_204672.p1_type_internal_len_162 | ---NA--- | | |
| TRINITY_DN21359_c0_g1_i2_len_626_6612.p1_type_complete_len_161 | XP_010121053.1 polyubiquitin isoform X2 | | |
| TRINITY_DN5906_c0_g1_i1_len_532_100594.p2_type_5prime_partial_len_161 | ---NA--- | | |
| TRINITY_DN13344_c0_g1_i1_len_486_204677.p1_type_internal_len_162 | ABX83902.1type II hemocyte transglutaminase | F:GO:0003810; P:GO:0018149; F:GO:0046872 | EC:2.3.2.13 |
| TRINITY_DN19829_c0_g1_i1_len_1594_192595.p1_type_5prime_partial_len_161 | ---NA--- | | |
| TRINITY_DN21479_c0_g1_i3_len_746_199334.p1_type_complete_len_161 | ---NA--- | | |
| TRINITY_DN16423_c0_g1_i1_len_595_67181.p1_type_5prime_partial_len_161 | XP_011669504.1 angiotensin-converting enzyme | F:GO:0070011 | |
| TRINITY_DN13715_c0_g1_i1_len_484_204736.p1_type_internal_len_162 | ---NA--- | | |

| | | | |
|---|---|---|---|
| TRINITY_DN26982_c0_g1_i1_len_2621_189192.p2_type_5prime_partial_len_161 | ---NA--- | | |
| TRINITY_DN11728_c0_g1_i1_len_631_164516.p1_type_5prime_partial_len_161 | ---NA--- | | |
| TRINITY_DN21564_c0_g1_i2_len_1640_232366.p1_type_complete_len_161 | ---NA--- | | |
| TRINITY_DN21980_c0_g1_i1_len_669_129366.p1_type_5prime_partial_len_161 | XP_001650427.2probable 39S ribosomal protein L23, mitochondrial | F:GO:0003735; C:GO:0005840; P:GO:0006412 | |
| TRINITY_DN1406_c0_g1_i1_len_486_204690.p1_type_internal_len_162 | ANA78437.1dopamine beta-hydroxylase | F:GO:0004497; C:GO:0016020 | |
| TRINITY_DN29188_c0_g1_i1_len_489_101559.p1_type_5prime_partial_len_161 | ---NA--- | | |
| TRINITY_DN6615_c0_g1_i1_len_486_204686.p1_type_internal_len_162 | XP_024892257.1tuberin isoform X2 | P:GO:0065007 | |
| TRINITY_DN10373_c0_g1_i1_len_487_204652.p1_type_internal_len_162 | ---NA--- | | |
| TRINITY_DN25930_c0_g1_i1_len_590_99585.p1_type_complete_len_161 | ---NA--- | | |
| TRINITY_DN16460_c0_g1_i1_len_1430_268007.p2_type_5prime_partial_len_161 | ---NA--- | | |
| TRINITY_DN8427_c0_g1_i1_len_484_204747.p2_type_internal_len_162 | EDS32075.1tubulointerstitial nephritis antigen | F:GO:0005044; P:GO:0006508; P:GO:0006898; P:GO:0006955; F:GO:0008234; F:GO:0030247 | |
| TRINITY_DN5086_c0_g1_i1_len_484_204750.p1_type_internal_len_162 | XP_015918377.1xylosyltransferase oxt-like | | |
| TRINITY_DN21505_c3_g3_i1_len_1661_296393.p2_type_complete_len_161 | XP_022307941.1uncharacterized protein LOC111113940 | | |
| TRINITY_DN9069_c0_g1_i1_len_641_240090.p1_type_complete_len_161 | XP_018026203.1 glucose dehydrogenase [FAD, quinone]-like | | |
| TRINITY_DN38930_c0_g1_i1_len_487_204656.p1_type_internal_len_162 | XP_018027333.1 low-density lipoprotein receptor-related protein 2-like | F:GO:0005509; C:GO:0016021 | |

| | | | |
|---|---|---|---|
| TRINITY_DN18442_c0_g1_i1_len_498_243082.p1_type_5prime_partial_len_161 | BAP28204.1copper/zinc superoxide dismutase isoform 5 | F:GO:0004784; C:GO:0016021; P:GO:0019430; F:GO:0046872; P:GO:0055114 | EC:1.15.1.1 |
| TRINITY_DN13355_c0_g1_i1_len_484_204731.p1_type_internal_len_162 | ---NA--- | | |
| TRINITY_DN13015_c0_g1_i1_len_485_204716.p1_type_internal_len_162 | EFN73293.1Dedicator of cytokinesis protein 3 | F:GO:0005085; P:GO:0007264 | |
| TRINITY_DN8573_c0_g1_i1_len_486_101643.p2_type_internal_len_162 | ---NA--- | | |
| TRINITY_DN31976_c0_g1_i1_len_485_204714.p1_type_internal_len_162 | AGZ84434.1heat shock protein 27 | | |
| TRINITY_DN19765_c0_g1_i1_len_1445_233291.p1_type_5prime_partial_len_161 | ---NA--- | | |
| TRINITY_DN552_c0_g1_i1_len_484_9211.p1_type_internal_len_162 | ---NA--- | | |
| TRINITY_DN10665_c0_g1_i1_len_485_9194.p1_type_internal_len_162 | ---NA--- | | |
| TRINITY_DN3235_c0_g1_i1_len_486_304491.p1_type_internal_len_162 | KZS19925.1Solute carrier family 12 member 8 | P:GO:0006884; F:GO:0015379; C:GO:0016020; P:GO:0055064; P:GO:0055075; P:GO:1902476; P:GO:1990573 | |
| TRINITY_DN1747_c0_g1_i1_len_575_241272.p1_type_5prime_partial_len_161 | XP_018007044.1 cytosolic purine 5'-nucleotidase-like isoform X1 | | |
| TRINITY_DN7024_c0_g1_i1_len_485_276387.p1_type_internal_len_162 | XP_018019085.1 paramyosin, long form-like isoform X2 | F:GO:0003774; C:GO:0016459 | EC:3.6.1.15 |
| TRINITY_DN8993_c0_g1_i1_len_515_166751.p1_type_5prime_partial_len_161 | XP_018007995.1 kinesin-like protein KIF20B | F:GO:0003777; F:GO:0005524; P:GO:0007018; F:GO:0008017 | EC:3.6.1.15 |
| TRINITY_DN8790_c0_g1_i1_len_483_276454.p1_type_internal_len_162 | ---NA--- | | |
| TRINITY_DN24063_c0_g1_i1_len_2630_229547.p2_type_5prime_partial_len_161 | XP_018012643.1 cyclin-L1-like | P:GO:0031323; P:GO:0051171; P:GO:0060255; P:GO:0080090 | |
| TRINITY_DN23961_c0_g1_i1_len_483_243505.p1_type_internal_len_162 | ESO98647.1hypothetical protein LOTGIDRAFT_180523 | F:GO:0003676 | |

| | | | |
|---|---|---|---|
| TRINITY_DN18738_c0_g1_i1_len_517_131963.p1_type_5prime_partial_len_161 | XP_018011191.1 copper-transporting ATPase 2-like | | |
| TRINITY_DN10135_c0_g1_i1_len_596_201854.p1_type_3prime_partial_len_162 | XP_018025035.1 inactive hydroxysteroid dehydrogenase-like protein 1 | C:GO:0016020 | |
| TRINITY_DN16514_c0_g1_i1_len_482_204802.p1_type_internal_len_161 | ---NA--- | | |
| TRINITY_DN13715_c0_g1_i1_len_1674_232231.p1_type_complete_len_160 | ---NA--- | | |
| TRINITY_DN8429_c0_g1_i1_len_2413_155154.p2_type_3prime_partial_len_161 | ---NA--- | | |
| TRINITY_DN12950_c0_g1_i2_len_483_39084.p2_type_internal_len_161 | XP_007620765.1 regulator of G-protein signaling 3-like | | |
| TRINITY_DN28883_c0_g1_i1_len_520_275590.p1_type_5prime_partial_len_160 | ---NA--- | | |
| TRINITY_DN26452_c0_g1_i1_len_557_202711.p1_type_5prime_partial_len_160 | ---NA--- | | |
| TRINITY_DN3834_c0_g1_i1_len_481_204825.p2_type_internal_len_161 | XP_013794399.1sushi, von Willebrand factor type A, EGF and pentraxin domain-containing protein 1-like | | |
| TRINITY_DN21120_c0_g1_i2_len_769_64893.p1_type_complete_len_160 | BAB85575.1cyclic AMP-regulated protein like protein | F:GO:0003779 | |
| TRINITY_DN19225_c0_g1_i1_len_723_5510.p1_type_complete_len_160 | ---NA--- | | |
| TRINITY_DN13645_c0_g1_i1_len_482_304590.p1_type_5prime_partial_len_160 | ---NA--- | | |
| TRINITY_DN23822_c0_g1_i1_len_1765_231887.p2_type_complete_len_160 | ---NA--- | | |
| TRINITY_DN23993_c0_g1_i1_len_2189_122803.p2_type_complete_len_160 | XP_008211868.1 flap endonuclease GEN | F:GO:0004518; P:GO:0006281; P:GO:0090305 | |
| TRINITY_DN33001_c0_g1_i1_len_481_132823.p1_type_internal_len_161 | ---NA--- | | |
| TRINITY_DN13777_c0_g1_i1_len_559_202673.p1_type_5prime_partial_len_160 | ---NA--- | | |

| | | | |
|---|---|---|---|
| TRINITY_DN7468_c0_g1_i1_len_1223_194780.p1_type_5prime_partial_len_160 | AFV99180.1autophagy-related protein 8 | C:GO:0005737; P:GO:0006914 | |
| TRINITY_DN24760_c0_g1_i1_len_481_276498.p1_type_internal_len_161 | XP_026466556.1phosphatidylinositol transfer protein alpha isoform | | |
| TRINITY_DN19899_c0_g1_i1_len_563_241514.p1_type_3prime_partial_len_161 | ---NA--- | | |
| TRINITY_DN18439_c0_g2_i1_len_484_39076.p1_type_internal_len_161 | ---NA--- | | |
| TRINITY_DN19603_c0_g1_i2_len_620_99124.p2_type_complete_len_160 | ---NA--- | | |
| TRINITY_DN33614_c0_g1_i1_len_543_203064.p1_type_3prime_partial_len_161 | XP_017778500.1 protein pangolin, isoforms A/H/I/S isoform X6 | | |
| TRINITY_DN7871_c0_g1_i1_len_481_276486.p1_type_internal_len_161 | ---NA--- | | |
| TRINITY_DN21504_c0_g1_i1_len_853_4464.p2_type_5prime_partial_len_160 | ---NA--- | | |
| TRINITY_DN18135_c0_g1_i1_len_910_127172.p2_type_complete_len_160 | BAM35674.1putative reverse transcriptase | F:GO:0003964; P:GO:0006278 | |
| TRINITY_DN37344_c0_g1_i1_len_625_201302.p1_type_5prime_partial_len_160 | XP_018022061.1 mucin-5AC-like | C:GO:0016020; C:GO:0016021 | |
| TRINITY_DN16847_c0_g1_i1_len_599_201800.p1_type_3prime_partial_len_161 | ---NA--- | | |
| TRINITY_DN18553_c0_g1_i1_len_1621_296471.p1_type_5prime_partial_len_160 | ---NA--- | | |
| TRINITY_DN11398_c0_g1_i1_len_482_204801.p2_type_internal_len_161 | XP_018011435.1 probable acyl-CoA dehydrogenase fadE25 | F:GO:0016491 | |
| TRINITY_DN23445_c0_g1_i2_len_1838_59697.p2_type_5prime_partial_len_160 | XP_018015635.1 src substrate protein p85-like | | |
| TRINITY_DN20644_c0_g1_i1_len_539_203183.p1_type_5prime_partial_len_160 | ---NA--- | | |
| TRINITY_DN17697_c0_g1_i2_len_480_204896.p1_type_internal_len_161 | XP_022241171.1arylsulfatase B-like | F:GO:0003824 | |

| | | | |
|---|---|---|---|
| TRINITY_DN5670_c0_g1_i1_len_481_204836.p1_type_internal_len_161 | XP_018012656.1PREDICTED: uncharacterized protein LOC108669763 | F:GO:0003676 | |
| TRINITY_DN23486_c0_g1_i3_len_1124_235221.p1_type_5prime_partial_len_160 | ---NA--- | | |
| TRINITY_DN15144_c0_g1_i1_len_483_204780.p1_type_internal_len_161 | PSN53146.1Protein DENND6B | F:GO:0017112 | |
| TRINITY_DN1870_c0_g1_i1_len_482_101741.p1_type_internal_len_161 | AFB81371.1MAP kinase-activated protein kinase 2 | F:GO:0004674; F:GO:0005524; P:GO:0006468 | EC:2.7.11 |
| TRINITY_DN24946_c0_g1_i1_len_622_201361.p2_type_3prime_partial_len_161 | ---NA--- | | |
| TRINITY_DN9094_c0_g1_i1_len_946_270586.p2_type_5prime_partial_len_160 | XP_018023920.1 calponin-3-like | | |
| TRINITY_DN19446_c0_g1_i1_len_852_34415.p2_type_5prime_partial_len_160 | ---NA--- | | |
| TRINITY_DN17088_c0_g1_i1_len_1073_235572.p1_type_5prime_partial_len_160 | ---NA--- | | |
| TRINITY_DN2689_c0_g1_i1_len_483_39103.p1_type_internal_len_161 | XP_026276882.1mitogen-activated protein kinase kinase kinase kinase 5 isoform X8 | F:GO:0004672; F:GO:0005524; P:GO:0006468 | |
| TRINITY_DN34491_c0_g1_i1_len_481_204838.p1_type_internal_len_161 | ---NA--- | | |
| TRINITY_DN18377_c0_g1_i1_len_617_240503.p1_type_complete_len_160 | ---NA--- | | |
| TRINITY_DN42304_c0_g1_i1_len_483_204753.p1_type_internal_len_161 | ---NA--- | | |
| TRINITY_DN38987_c0_g1_i1_len_484_204743.p1_type_internal_len_161 | XP_019959628.1 tubulin alpha chain-like | F:GO:0003924; F:GO:0005200; F:GO:0005525; C:GO:0005737; C:GO:0005874; P:GO:0007010; P:GO:0007017 | EC:3.6.1.15 |
| TRINITY_DN4258_c0_g1_i1_len_481_9287.p1_type_internal_len_161 | ---NA--- | | |
| TRINITY_DN41194_c0_g1_i1_len_482_204819.p1_type_internal_len_161 | XP_021936351.1protein crumbs | | |

| | | | |
|---|---|---|---|
| TRINITY_DN2033_c0_g1_i1_len_611_240611.p1_type_5prime_partial_len_160 | ---NA--- | | |
| TRINITY_DN34307_c0_g1_i1_len_483_204754.p1_type_internal_len_161 | ---NA--- | | |
| TRINITY_DN17720_c0_g1_i2_len_613_240571.p1_type_5prime_partial_len_160 | ---NA--- | | |
| TRINITY_DN4262_c0_g1_i1_len_535_203281.p1_type_5prime_partial_len_160 | ABP73291.1anti-lipopolysaccharide factor isoform 2 | | |
| TRINITY_DN2626_c0_g1_i1_len_482_167510.p1_type_internal_len_161 | ---NA--- | | |
| TRINITY_DN16089_c0_g2_i1_len_482_167506.p3_type_internal_len_161 | ---NA--- | | |
| TRINITY_DN19366_c0_g1_i1_len_481_69627.p1_type_internal_len_161 | XP_024613446.1peroxisome assembly protein 12-like | F:GO:0005488 | |
| TRINITY_DN24143_c0_g1_i4_len_3666_228358.p1_type_5prime_partial_len_939 | XP_023714866.1transcription elongation regulator 1 isoform X5 | | |
| 1737\|_\|c2795_f3p3_2816_isoform_c2795_full_length_coverage_3_non_full_length_coverage_3_isoform_length_2816_12168 | ---NA--- | | |
| TRINITY_DN30993_c0_g1_i2_len_4504_187312.p1_type_complete_len_938 | XP_023722155.1breast cancer anti-estrogen resistance protein 3 isoform X2 | F:GO:0005085; P:GO:0007264 | |
| TRINITY_DN19923_c0_g1_i1_len_2976_29497.p1_type_5prime_partial_len_938 | XP_022240875.1neutral alpha-glucosidase AB-like isoform X1 | | |
| 1740\|_\|c1662_f2p7_2813_isoform_c1662_full_length_coverage_2_non_full_length_coverage_7_isoform_length_2813_26013 | KDR16969.1Histone-lysine N-methyltransferase NSD3 | F:GO:0016740 | |
| TRINITY_DN19717_c0_g1_i1_len_3235_29397.p1_type_complete_len_937 | XP_021924492.1transmembrane and TPR repeat-containing protein CG4050-like | | |
| TRINITY_DN31002_c0_g1_i1_len_3427_188009.p1_type_complete_len_937 | XP_026272076.1piwi-like protein Ago3 | F:GO:0003676; P:GO:0031047 | |
| 1744\|_\|c1879_f2p4_2811_isoform_c1879_full_length_coverage_2_non_full_length_coverage_4_isoform_length_2811_25557 | XP_018023448.1 pollen-specific leucine-rich repeat extensin-like protein 2 isoform X1 | | |

| | | | |
|---|---|---|---|
| TRINITY_DN24481_c0_g1_i2_len_2904_58234.p1_type_5prime_partial_len_937 | ASW35097.1ATP-dependent 6-phosphofructokinase-like protein | F:GO:0003872; F:GO:0005524; C:GO:0005737; P:GO:0006002; F:GO:0046872; P:GO:0061615 | EC:2.7.1.11 |
| TRINITY_DN29081_c0_g1_i1_len_4058_187555.p1_type_complete_len_937 | AEK49107.1Tudor staphylococcal nuclease | F:GO:0004521; C:GO:0005737; C:GO:0016442; P:GO:0031047; P:GO:0090502 | |
| TRINITY_DN35104_c0_g1_i1_len_481_243560.p1_type_internal_len_161 | ---NA--- | | |
| TRINITY_DN4859_c0_g1_i1_len_480_243581.p1_type_internal_len_161 | XP_013786278.1mitochondrial coenzyme A transporter SLC25A42-like | | |
| TRINITY_DN27058_c0_g1_i1_len_3222_188221.p2_type_3prime_partial_len_161 | KMQ90581.1polycomb protein scm | C:GO:0005634; P:GO:0006355; F:GO:0008270 | |
| TRINITY_DN30470_c0_g1_i1_len_481_243576.p1_type_internal_len_161 | XP_022241301.1dystrophin-like | | |
| TRINITY_DN11104_c0_g1_i1_len_481_243552.p1_type_internal_len_161 | ---NA--- | | |
| TRINITY_DN7320_c0_g1_i1_len_482_243550.p2_type_internal_len_161 | XP_018014104.1 inactive pancreatic lipase-related protein 1-like | | |
| TRINITY_DN21117_c0_g1_i1_len_482_243548.p1_type_internal_len_161 | ---NA--- | | |
| TRINITY_DN30972_c0_g1_i1_len_482_243547.p3_type_internal_len_161 | ASJ26360.1IL-16-like protein | | |
| TRINITY_DN29153_c0_g1_i1_len_482_69602.p1_type_internal_len_161 | PNF35542.1hypothetical protein B7P43_G04105 | | |
| TRINITY_DN2636_c0_g1_i1_len_481_39149.p1_type_internal_len_161 | ---NA--- | | |
| TRINITY_DN10221_c0_g1_i1_len_482_69613.p1_type_internal_len_161 | ---NA--- | | |
| TRINITY_DN10498_c0_g1_i1_len_482_69611.p1_type_internal_len_161 | ---NA--- | | |
| TRINITY_DN17907_c0_g1_i1_len_484_9226.p1_type_internal_len_161 | ---NA--- | | |

| | | | |
|---|---|---|---|
| TRINITY_DN872_c0_g1_i1_len_483_132792.p1_type_internal_len_161 | ---NA--- | | |
| TRINITY_DN2010_c0_g1_i1_len_482_204807.p1_type_internal_len_161 | XP_018022821.1 ubiquitin carboxyl-terminal hydrolase 25-like | | |
| TRINITY_DN26369_c0_g1_i1_len_484_39071.p1_type_internal_len_161 | XP_018023090.1PREDICTED: uncharacterized protein LOC108679078 | C:GO:0016020; C:GO:0016021; F:GO:0022857; P:GO:0055085 | |
| TRINITY_DN33663_c0_g1_i1_len_494_167184.p1_type_5prime_partial_len_160 | XP_026688765.1histone deacetylase 4-like | P:GO:0006325; F:GO:0016787 | |
| TRINITY_DN4745_c0_g1_i1_len_480_243584.p2_type_internal_len_161 | ---NA--- | | |
| TRINITY_DN32518_c0_g1_i1_len_480_243583.p1_type_internal_len_161 | ---NA--- | | |
| TRINITY_DN5784_c0_g1_i2_len_481_167550.p1_type_internal_len_161 | ---NA--- | | |
| TRINITY_DN21106_c0_g1_i1_len_482_39117.p1_type_internal_len_161 | KFM62896.1Protein kibra | | |
| TRINITY_DN21896_c0_g1_i2_len_3003_154330.p2_type_complete_len_160 | ---NA--- | | |
| TRINITY_DN2209_c0_g1_i1_len_483_243510.p1_type_internal_len_161 | ---NA--- | | |
| TRINITY_DN16645_c0_g1_i1_len_483_243481.p1_type_internal_len_161 | ---NA--- | | |
| TRINITY_DN6665_c0_g1_i1_len_484_243475.p1_type_internal_len_161 | XP_018012588.1 fat-like cadherin-related tumor suppressor homolog | C:GO:0016020 | |
| TRINITY_DN6904_c0_g1_i1_len_482_204792.p1_type_internal_len_161 | XP_019769197.1 neurobeachin-like | | |
| TRINITY_DN26765_c0_g1_i1_len_483_243485.p1_type_internal_len_161 | XP_021916471.1filamin-A isoform X2 | | |
| TRINITY_DN38962_c0_g1_i1_len_481_204848.p1_type_internal_len_161 | XP_018018774.1PREDICTED: uncharacterized protein LOC108675285 | | |
| TRINITY_DN11539_c1_g1_i1_len_483_243484.p1_type_internal_len_161 | ---NA--- | | |

| | | | |
|---|---|---|---|
| TRINITY_DN30217_c0_g1_i1_len_482_39121.p1_type_internal_len_161 | ---NA--- | | |
| TRINITY_DN33306_c0_g1_i1_len_482_204791.p1_type_internal_len_161 | ---NA--- | | |
| TRINITY_DN20164_c0_g1_i1_len_752_199257.p1_type_3prime_partial_len_161 | ---NA--- | | |
| TRINITY_DN28669_c0_g1_i1_len_482_243545.p2_type_internal_len_161 | XP_014276494.1CUGBP Elav-like family member 1 isoform X9 | | |
| TRINITY_DN7165_c0_g1_i1_len_482_69597.p1_type_internal_len_161 | ---NA--- | | |
| TRINITY_DN21726_c0_g1_i2_len_706_239105.p1_type_complete_len_160 | ---NA--- | | |
| TRINITY_DN26877_c0_g1_i1_len_482_243543.p1_type_internal_len_161 | XP_011299174.1 neither inactivation nor afterpotential protein C isoform X2 | F:GO:0003774; F:GO:0005524 | |
| TRINITY_DN28878_c0_g1_i1_len_482_243542.p4_type_internal_len_161 | ---NA--- | | |
| TRINITY_DN15121_c0_g1_i1_len_482_243539.p1_type_internal_len_161 | ---NA--- | | |
| TRINITY_DN35019_c0_g1_i1_len_482_243536.p1_type_internal_len_161 | ---NA--- | | |
| TRINITY_DN12691_c0_g1_i1_len_481_304608.p1_type_internal_len_161 | XP_022198815.1zinc finger protein OZF-like | | |
| TRINITY_DN17429_c0_g1_i1_len_1045_126264.p3_type_5prime_partial_len_160 | ---NA--- | | |
| TRINITY_DN31469_c0_g1_i1_len_482_132809.p1_type_internal_len_161 | XP_018019085.1 paramyosin, long form-like isoform X2 | | |
| TRINITY_DN19572_c0_g1_i1_len_1340_194009.p1_type_5prime_partial_len_160 | ---NA--- | | |
| TRINITY_DN17641_c0_g1_i1_len_481_304607.p3_type_internal_len_161 | ---NA--- | | |
| TRINITY_DN11480_c0_g1_i1_len_721_238889.p1_type_5prime_partial_len_160 | ---NA--- | | |

| | | | |
|---|---|---|---|
| TRINITY_DN36963_c0_g1_i1_len_987_196663.p2_type_3prime_partial_len_161 | ---NA--- | | |
| TRINITY_DN14888_c0_g1_i1_len_481_132830.p1_type_internal_len_160 | ABN13875.1hemocyte transglutaminase | F:GO:0003810; P:GO:0018149; F:GO:0046872 | EC:2.3.2.13 |
| TRINITY_DN15049_c0_g1_i1_len_478_243650.p1_type_internal_len_160 | ---NA--- | | |
| TRINITY_DN33216_c0_g1_i1_len_478_243653.p1_type_internal_len_160 | XP_026680107.1glutathione S-transferase 1-like | F:GO:0016740 | |
| TRINITY_DN10296_c0_g1_i1_len_478_69741.p1_type_internal_len_160 | XP_012522663.1  E3 ubiquitin-protein ligase HUWE1 | | |
| TRINITY_DN15590_c0_g1_i2_len_550_100290.p1_type_complete_len_159 | XP_018015789.1 transmembrane protein 138-like | | |
| TRINITY_DN17178_c0_g1_i2_len_571_302829.p1_type_complete_len_159 | AEL23142.1RING finger protein 181 | | |
| TRINITY_DN12197_c0_g1_i1_len_480_204889.p2_type_internal_len_160 | ASA69502.1penlectin 5-2 | | |
| TRINITY_DN26415_c0_g1_i1_len_480_101799.p1_type_internal_len_160 | ---NA--- | | |
| TRINITY_DN33553_c0_g1_i1_len_481_204824.p1_type_internal_len_160 | ---NA--- | | |
| TRINITY_DN20462_c0_g1_i1_len_2612_154814.p1_type_complete_len_159 | XP_018020577.1PREDICTED: uncharacterized protein LOC108676929, partial | | |
| TRINITY_DN21541_c0_g1_i1_len_1074_235552.p1_type_3prime_partial_len_160 | ---NA--- | | |
| TRINITY_DN11702_c0_g1_i1_len_576_202295.p1_type_3prime_partial_len_160 | ---NA--- | | |
| TRINITY_DN8450_c0_g1_i1_len_479_101825.p1_type_internal_len_160 | XP_024613446.1peroxisome assembly protein 12-like | F:GO:0005488; C:GO:0005778; P:GO:0006625 | |
| TRINITY_DN13324_c0_g1_i1_len_479_101823.p1_type_internal_len_160 | PIK57074.1hypothetical protein BSL78_06014 | | |
| TRINITY_DN18842_c0_g1_i1_len_1065_95185.p1_type_complete_len_159 | XP_005957210.1 thioredoxin, mitochondrial | | |

| | | | |
|---|---|---|---|
| TRINITY_DN2908_c0_g1_i1_len_529_68423.p1_type_5prime_partial_len_159 | ---NA--- | | |
| TRINITY_DN29183_c0_g1_i1_len_1966_191019.p1_type_complete_len_159 | ---NA--- | | |
| TRINITY_DN32888_c0_g1_i1_len_478_243658.p1_type_internal_len_160 | KFM66581.1Myotubularin-related protein 2 | F:GO:0004725; P:GO:0035335 | EC:3.1.3.16; EC:3.1.3.48 |
| TRINITY_DN28934_c0_g1_i1_len_4563_187283.p2_type_complete_len_159 | ---NA--- | | |
| TRINITY_DN29034_c0_g1_i1_len_510_303963.p1_type_5prime_partial_len_159 | XP_018010857.1 glutaredoxin-related protein 5, mitochondrial-like | | |
| TRINITY_DN11285_c0_g1_i1_len_578_202252.p1_type_5prime_partial_len_159 | PIK40076.1hypothetical protein BSL78_23082 | | |
| TRINITY_DN14360_c0_g1_i2_len_479_204904.p1_type_internal_len_160 | ---NA--- | | |
| TRINITY_DN40634_c0_g1_i1_len_479_204903.p1_type_5prime_partial_len_159 | PNF15389.1Tubulin-specific chaperone cofactor E-like protein | | |
| TRINITY_DN22548_c0_g1_i1_len_1348_124812.p2_type_complete_len_159 | ---NA--- | | |
| TRINITY_DN19157_c0_g1_i1_len_3035_228953.p2_type_complete_len_159 | ---NA--- | | |
| TRINITY_DN28861_c0_g1_i1_len_479_243641.p1_type_internal_len_160 | XP_018015697.1 plexin-B-like | | |
| TRINITY_DN9227_c0_g1_i1_len_480_204869.p1_type_internal_len_160 | ---NA--- | | |
| TRINITY_DN4955_c0_g1_i1_len_762_238393.p2_type_complete_len_159 | XP_018006918.1 homer protein homolog 2-like | | |
| TRINITY_DN21168_c0_g1_i1_len_1220_194808.p2_type_5prime_partial_len_159 | XP_018015705.1 putative tricarboxylate transport protein, mitochondrial | | |
| TRINITY_DN2895_c0_g1_i1_len_522_68579.p1_type_5prime_partial_len_159 | ---NA--- | | |
| TRINITY_DN21988_c0_g2_i1_len_479_243636.p1_type_internal_len_160 | XP_021003064.1phosphatidylinositol 3-kinase regulatory subunit alpha | | |

| | | | |
|---|---|---|---|
| TRINITY_DN33520_c0_g1_i1_len_477_204984.p1_type_internal_len_160 | XP_018027164.1 probable phosphorylase b kinase regulatory subunit beta isoform X1 | | |
| TRINITY_DN25585_c0_g1_i1_len_1593_232574.p2_type_3prime_partial_len_160 | ---NA--- | | |
| TRINITY_DN12118_c0_g1_i1_len_718_5552.p1_type_5prime_partial_len_159 | ---NA--- | | |
| TRINITY_DN24569_c0_g1_i1_len_1551_192821.p1_type_complete_len_159 | ---NA--- | | |
| TRINITY_DN11184_c0_g1_i1_len_480_204877.p1_type_internal_len_160 | ---NA--- | | |
| TRINITY_DN21909_c1_g1_i3_len_480_101790.p1_type_internal_len_160 | ---NA--- | | |
| TRINITY_DN32367_c0_g1_i1_len_480_204880.p1_type_internal_len_160 | ---NA--- | | |
| TRINITY_DN22207_c0_g1_i1_len_2044_230980.p5_type_5prime_partial_len_159 | XP_023242482.1kelch-like protein 26 | | |
| TRINITY_DN2045_c0_g1_i1_len_680_163815.p1_type_5prime_partial_len_159 | XP_018010800.1 ADP-ribosylation factor-like protein 4C | | |
| TRINITY_DN27215_c0_g1_i1_len_479_243644.p1_type_internal_len_160 | ---NA--- | | |
| TRINITY_DN13770_c0_g1_i1_len_602_274112.p1_type_5prime_partial_len_159 | ---NA--- | | |
| TRINITY_DN22768_c0_g1_i1_len_2058_155949.p1_type_complete_len_159 | ---NA--- | | |
| TRINITY_DN26191_c0_g1_i1_len_3042_188467.p3_type_3prime_partial_len_160 | ---NA--- | | |
| TRINITY_DN15833_c0_g1_i2_len_480_132868.p1_type_internal_len_160 | ---NA--- | | |
| TRINITY_DN3866_c0_g1_i2_len_477_167646.p1_type_internal_len_160 | XP_018028125.1 retinol dehydrogenase 12-like | | |
| TRINITY_DN14828_c0_g1_i1_len_479_204929.p1_type_internal_len_160 | ---NA--- | | |

| | | | |
|---|---|---|---|
| TRINITY_DN8763_c0_g1_i1_len_478_204941.p1_type_internal_len_160 | XP_023324534.1sushi, von Willebrand factor type A, EGF and pentraxin domain-containing protein 1-like | | |
| TRINITY_DN4692_c0_g1_i1_len_481_167539.p1_type_internal_len_160 | ---NA--- | | |
| TRINITY_DN22744_c0_g1_i2_len_2813_188837.p1_type_5prime_partial_len_159 | ---NA--- | | |
| TRINITY_DN27412_c0_g1_i1_len_478_69718.p1_type_internal_len_160 | XP_018011453.1 nicastrin-like | | |
| TRINITY_DN9921_c0_g1_i1_len_477_167648.p1_type_internal_len_160 | ---NA--- | | |
| TRINITY_DN37280_c0_g1_i1_len_479_204917.p1_type_internal_len_160 | KDR08837.1SEC23-interacting protein | F:GO:0046872 | |
| TRINITY_DN17700_c0_g1_i1_len_550_131274.p1_type_5prime_partial_len_159 | XP_022909000.1protein ALP1-like isoform X1 | | |
| TRINITY_DN14208_c0_g1_i1_len_2814_188833.p1_type_internal_len_938 | KDR16213.1Huntingtin | C:GO:0005634; C:GO:0005737 | |
| TRINITY_DN21829_c0_g1_i1_len_2831_238.p1_type_5prime_partial_len_937 | XP_015905651.1zinc finger RNA-binding protein | | |
| 1749\|_\|c35032_f2p5_2809_isoform_c35032_full_length_coverage_2_non_full_length_coverage_5_isoform_length_2809_14430 | XP_017882140.1adenylosuccinate lyase | | |
| 1750\|_\|c8696_f4p2_2809_isoform_c8696_full_length_coverage_4_non_full_length_coverage_2_isoform_length_2809_17218 | XP_025107679.1protein rolling stone-like | | |
| 1751\|_\|c5205_f4p1_2809_isoform_c5205_full_length_coverage_4_non_full_length_coverage_1_isoform_length_2809_16940 | XP_018021978.1 RNA polymerase I-specific transcription initiation factor RRN3-like isoform X2 | | |
| 1752\|_\|c2311_f2p1_2808_isoform_c2311_full_length_coverage_2_non_full_length_coverage_1_isoform_length_2808_20836 | XP_005110357.2 SET and MYND domain-containing protein 4-like | | |
| TRINITY_DN30636_c0_g1_i1_len_3685_187781.p1_type_5prime_partial_len_936 | XP_022108866.1unconventional myosin-XIX-like isoform X2 | | |

| | | | |
|---|---|---|---|
| TRINITY_DN27108_c0_g1_i1_len_2819_188824.p1_type_5prime_partial_len_936 | ---NA--- | | |
| TRINITY_DN23136_c0_g1_i1_len_3768_153780.p1_type_5prime_partial_len_936 | ABO38434.1Toll receptor | P:GO:0007165; C:GO:0016021; P:GO:0045087 | |
| 1758\|_\|c22878_f2p1_2807_isoform_c22878_full_length_coverage_2_non_full_length_coverage_1_isoform_length_2807_13856 | XP_018023714.1 RNA-binding protein 25-like | P:GO:0006397 | |
| TRINITY_DN23522_c0_g1_i1_len_492_9016.p1_type_5prime_partial_len_159 | XP_018009322.1 transcription initiation factor TFIID subunit 1-like | F:GO:0004402; C:GO:0005669; P:GO:0016573; F:GO:0017025; F:GO:0043565; P:GO:0045944 | EC:2.3.1.5; EC:2.3.1.48 |
| TRINITY_DN6538_c0_g1_i1_len_478_204953.p1_type_internal_len_160 | XP_018903059.1PREDICTED: uncharacterized protein LOC109034392 | F:GO:0003676 | |
| TRINITY_DN33391_c0_g1_i1_len_479_204922.p1_type_internal_len_160 | XP_022241363.1enhancer of mRNA-decapping protein 4-like | | |
| TRINITY_DN9019_c0_g1_i1_len_479_167599.p4_type_internal_len_160 | ---NA--- | | |
| TRINITY_DN1828_c0_g1_i1_len_478_204954.p1_type_internal_len_160 | ---NA--- | | |
| TRINITY_DN24977_c1_g1_i2_len_2444_229912.p2_type_complete_len_159 | ---NA--- | | |
| TRINITY_DN29160_c0_g1_i1_len_2226_190168.p3_type_complete_len_159 | ---NA--- | | |
| TRINITY_DN13274_c0_g1_i1_len_479_132892.p1_type_internal_len_160 | ---NA--- | | |
| TRINITY_DN33026_c0_g1_i1_len_479_204912.p2_type_internal_len_160 | ---NA--- | | |
| TRINITY_DN33607_c0_g1_i1_len_478_204960.p1_type_internal_len_160 | KZS05741.1N(G),N(G)-dimethylarginine dimethylaminohydrolase 1 | F:GO:0016787 | |
| TRINITY_DN7282_c0_g1_i1_len_481_243569.p1_type_internal_len_160 | ---NA--- | | |
| TRINITY_DN21875_c0_g1_i1_len_1039_196201.p1_type_5prime_partial_len_159 | ---NA--- | | |

| | | | |
|---|---|---|---|
| TRINITY_DN9699_c0_g1_i1_len_479_243622.p1_type_internal_len_160 | XP_015924678.1ras-related protein Rab-4B-like | F:GO:0003924; F:GO:0005525 | EC:3.6.1.15 |
| TRINITY_DN20558_c0_g1_i2_len_1782_92570.p2_type_complete_len_159 | XP_018017488.1PREDICTED: uncharacterized protein LOC108674095 | | |
| TRINITY_DN9647_c1_g1_i1_len_478_276575.p1_type_internal_len_160 | XP_024124277.1folylpolyglutamate synthase, mitochondrial | | |
| TRINITY_DN26994_c0_g1_i1_len_478_39203.p1_type_internal_len_160 | XP_018012021.1 macrophage mannose receptor 1-like | C:GO:0016020; C:GO:0016021 | |
| TRINITY_DN13118_c0_g1_i1_len_478_276569.p1_type_internal_len_160 | ---NA--- | | |
| TRINITY_DN5136_c0_g1_i2_len_479_276548.p2_type_internal_len_160 | ---NA--- | | |
| TRINITY_DN37153_c0_g1_i1_len_478_243668.p1_type_internal_len_160 | KZS06758.1Tryptophan 2,3-dioxygenase | F:GO:0004833; P:GO:0019441; F:GO:0020037; F:GO:0046872; P:GO:0055114 | EC:1.13.11; EC:1.13.11.11 |
| TRINITY_DN4387_c0_g1_i1_len_480_243580.p1_type_internal_len_160 | ---NA--- | | |
| TRINITY_DN11706_c0_g1_i1_len_652_273391.p1_type_complete_len_159 | ---NA--- | | |
| TRINITY_DN20810_c0_g1_i1_len_1340_93878.p1_type_5prime_partial_len_158 | ---NA--- | | |
| TRINITY_DN19739_c0_g1_i1_len_898_127257.p1_type_complete_len_158 | XP_018023116.1 zinc finger protein Xfin-like | | |
| TRINITY_DN23981_c4_g1_i1_len_878_127401.p1_type_3prime_partial_len_159 | ---NA--- | | |
| TRINITY_DN24594_c0_g1_i1_len_477_9401.p1_type_internal_len_159 | EEB12539.1Rho GTPase regulating protein, putative | P:GO:0007165 | |
| TRINITY_DN8454_c0_g1_i1_len_512_203873.p1_type_3prime_partial_len_159 | ---NA--- | | |
| TRINITY_DN17666_c0_g1_i2_len_1161_234960.p1_type_5prime_partial_len_158 | ---NA--- | | |
| TRINITY_DN21141_c0_g1_i1_len_1637_1375.p3_type_5prime_partial_len_158 | ---NA--- | | |

| | | | |
|---|---|---|---|
| TRINITY_DN28959_c0_g1_i1_len_477_9400.p1_type_internal_len_159 | XP_018012983.1 cilia- and flagella-associated protein 57-like | | |
| TRINITY_DN3640_c0_g1_i1_len_476_304726.p1_type_internal_len_159 | ---NA--- | | |
| TRINITY_DN1211_c0_g1_i1_len_475_304734.p1_type_internal_len_159 | ---NA--- | | |
| TRINITY_DN21866_c0_g1_i1_len_793_198701.p1_type_5prime_partial_len_158 | AIU99749.1mitochondrial cytochrome c oxidase subunit VIb | C:GO:0005739 | |
| TRINITY_DN12828_c0_g1_i1_len_475_304741.p1_type_internal_len_159 | ---NA--- | | |
| TRINITY_DN5876_c0_g1_i1_len_477_204974.p1_type_internal_len_159 | PSN37510.1E3 ubiquitin-protein ligase HECTD1 | F:GO:0003824 | |
| TRINITY_DN16326_c0_g1_i1_len_1204_94459.p2_type_5prime_partial_len_158 | BAN20407.1histone H2A | C:GO:0000786; F:GO:0003677; C:GO:0005634; F:GO:0046982 | |
| TRINITY_DN42249_c0_g1_i1_len_474_205074.p1_type_internal_len_159 | APO14259.1juvenile hormone esterase-like carboxylesterase 1 | F:GO:0016787 | |
| TRINITY_DN4249_c0_g1_i1_len_477_204979.p1_type_internal_len_159 | ---NA--- | | |
| TRINITY_DN23294_c0_g1_i1_len_1467_233183.p1_type_complete_len_158 | ---NA--- | | |
| TRINITY_DN47_c0_g1_i1_len_478_204962.p1_type_internal_len_159 | AUC64090.1phosphatidylinositol transfer protein | F:GO:0005548; P:GO:0015914; F:GO:0046872 | |
| TRINITY_DN41619_c0_g1_i1_len_474_205089.p1_type_internal_len_159 | XP_010782447.1 myotubularin-related protein 3-like | | |
| TRINITY_DN12835_c0_g1_i1_len_475_276666.p1_type_internal_len_159 | ---NA--- | | |
| TRINITY_DN23935_c0_g1_i1_len_478_167629.p1_type_internal_len_159 | XP_023230709.1neurogenic protein big brain-like | C:GO:0016020 | |
| TRINITY_DN10032_c0_g1_i1_len_475_276653.p1_type_internal_len_159 | ---NA--- | | |
| TRINITY_DN17866_c0_g1_i1_len_477_167668.p1_type_internal_len_159 | ---NA--- | | |

| | | | |
|---|---|---|---|
| TRINITY_DN9958_c0_g1_i1_len_476_276630.p1_type_internal_len_159 | ---NA--- | | |
| TRINITY_DN20565_c0_g2_i1_len_476_132968.p1_type_internal_len_159 | ---NA--- | | |
| TRINITY_DN7399_c0_g1_i1_len_476_205011.p2_type_internal_len_159 | PNF27755.1Zinc finger with UFM1-specific peptidase domain protein | F:GO:0003676 | |
| TRINITY_DN32118_c0_g1_i1_len_476_132959.p1_type_internal_len_159 | ---NA--- | | |
| TRINITY_DN34280_c0_g1_i1_len_475_205034.p1_type_internal_len_159 | XP_022191769.1F-box/LRR-repeat protein 6-like | P:GO:0044267 | |
| TRINITY_DN20987_c0_g1_i2_len_1624_296463.p2_type_complete_len_158 | XP_010879543.1 mitotic spindle-associated MMXD complex subunit MIP18 | | |
| TRINITY_DN35242_c0_g1_i1_len_475_205028.p1_type_internal_len_159 | ---NA--- | | |
| TRINITY_DN31998_c0_g1_i1_len_477_204990.p1_type_internal_len_159 | XP_023327503.1uncharacterized protein LOC111700723 | | |
| TRINITY_DN2754_c0_g1_i1_len_476_204998.p2_type_internal_len_159 | ---NA--- | | |
| TRINITY_DN5418_c0_g1_i1_len_477_204992.p2_type_5prime_partial_len_158 | XP_018017480.1 lysyl oxidase homolog 2-like isoform X3 | | |
| TRINITY_DN37037_c0_g1_i1_len_477_204991.p1_type_internal_len_159 | ---NA--- | | |
| TRINITY_DN17977_c0_g1_i1_len_492_204503.p1_type_5prime_partial_len_158 | XP_022254646.1transmembrane protein 8A-like | | |
| TRINITY_DN25108_c0_g1_i1_len_478_132919.p1_type_internal_len_159 | ---NA--- | | |
| TRINITY_DN10837_c0_g1_i1_len_537_166224.p1_type_3prime_partial_len_159 | ---NA--- | | |
| TRINITY_DN4067_c0_g1_i1_len_476_205003.p2_type_internal_len_159 | ---NA--- | | |
| TRINITY_DN40900_c0_g1_i1_len_476_205001.p1_type_internal_len_159 | XP_013866165.1 lysosomal acid phosphatase isoform X1 | C:GO:0016020; C:GO:0016021 | |

| | | | |
|---|---|---|---|
| TRINITY_DN39142_c0_g1_i1_len_475_205057.p1_type_internal_len_159 | ---NA--- | | |
| TRINITY_DN8786_c0_g1_i1_len_520_68619.p1_type_5prime_partial_len_158 | ---NA--- | | |
| TRINITY_DN4965_c0_g1_i1_len_498_167114.p1_type_5prime_partial_len_158 | KGB42380.1Tubulin alpha chain | F:GO:0003924; F:GO:0005200; F:GO:0005525; C:GO:0005737; C:GO:0005874; P:GO:0007010; P:GO:0007017 | EC:3.6.1.15 |
| TRINITY_DN17500_c0_g1_i1_len_537_100522.p1_type_5prime_partial_len_158 | XP_018019714.1 organic cation transporter protein-like | | |
| TRINITY_DN25575_c0_g1_i2_len_7866_227483.p3_type_5prime_partial_len_158 | ---NA--- | | |
| TRINITY_DN39524_c0_g1_i1_len_477_204987.p1_type_internal_len_159 | XP_018014994.1 rho GTPase-activating protein 1-like | | |
| TRINITY_DN25646_c1_g1_i2_len_477_204988.p1_type_internal_len_159 | XP_019615798.1 craniofacial development protein 2-like | | |
| TRINITY_DN19211_c0_g1_i1_len_1361_233722.p1_type_5prime_partial_len_158 | ---NA--- | | |
| TRINITY_DN14636_c0_g1_i1_len_475_205050.p1_type_internal_len_159 | KNE57957.1hypothetical protein AMAG_18360 | F:GO:0005488 | |
| TRINITY_DN9759_c0_g1_i1_len_474_133011.p1_type_internal_len_159 | ---NA--- | | |
| TRINITY_DN23371_c0_g1_i7_len_3177_154150.p3_type_complete_len_158 | ---NA--- | | |
| TRINITY_DN25947_c0_g1_i2_len_999_196556.p2_type_complete_len_158 | KZS02768.1Uncharacterized protein APZ42_000054, partial | F:GO:0003677; P:GO:0006313; P:GO:0015074 | |
| TRINITY_DN25375_c0_g1_i1_len_476_101915.p1_type_internal_len_159 | ---NA--- | | |
| TRINITY_DN6063_c0_g1_i1_len_475_101936.p1_type_internal_len_159 | ---NA--- | | |
| TRINITY_DN13367_c0_g1_i1_len_517_303823.p1_type_5prime_partial_len_158 | XP_018027484.1 DNA-directed RNA polymerases I and III subunit RPAC2-like | | |

| | | | |
|---|---|---|---|
| TRINITY_DN14598_c0_g1_i1_len_518_38325.p1_type_5prime_partial_len_158 | ---NA--- | | |
| TRINITY_DN9219_c0_g1_i1_len_538_275197.p1_type_5prime_partial_len_158 | XP_022094939.1acetyl-coenzyme A synthetase 2-like, mitochondrial isoform X2 | F:GO:0003824 | |
| TRINITY_DN17908_c0_g1_i2_len_477_9387.p1_type_internal_len_159 | XP_018006912.1 SOSS complex subunit B1-B-like | | |
| TRINITY_DN24940_c0_g1_i1_len_477_9385.p1_type_internal_len_159 | ABE02605.1plexin-like protein | C:GO:0016020; F:GO:0017154; P:GO:0071526 | |
| TRINITY_DN15078_c0_g1_i2_len_477_101887.p1_type_internal_len_159 | ---NA--- | | |
| TRINITY_DN21849_c1_g2_i1_len_478_243654.p1_type_internal_len_159 | PRD19005.1zinc finger protein | F:GO:0003676 | |
| TRINITY_DN14782_c0_g1_i1_len_478_243661.p1_type_internal_len_159 | ---NA--- | | |
| TRINITY_DN28987_c0_g1_i1_len_478_243663.p1_type_internal_len_159 | ---NA--- | | |
| TRINITY_DN29927_c0_g1_i1_len_477_243676.p1_type_internal_len_159 | ---NA--- | | |
| TRINITY_DN36726_c0_g1_i1_len_477_243680.p1_type_internal_len_159 | XP_023707924.1protein groucho-like isoform X2 | | |
| TRINITY_DN14933_c0_g1_i1_len_477_243699.p1_type_internal_len_159 | ---NA--- | | |
| TRINITY_DN15315_c0_g1_i1_len_629_273727.p1_type_5prime_partial_len_158 | KDR11796.1Zinc finger HIT domain-containing protein 3 | F:GO:0046872 | |
| TRINITY_DN20382_c0_g1_i1_len_2709_229415.p2_type_complete_len_158 | ---NA--- | | |
| TRINITY_DN37049_c0_g1_i1_len_475_243751.p1_type_internal_len_159 | XP_018026562.1PREDICTED: uncharacterized protein LOC108681986, partial | F:GO:0004867; C:GO:0005576; P:GO:0010466; P:GO:0010951; F:GO:0030414 | |
| TRINITY_DN1784_c0_g1_i1_len_474_243762.p1_type_internal_len_159 | ---NA--- | | |

| | | | |
|---|---|---|---|
| TRINITY_DN10862_c0_g1_i1_len_474_243771.p1_type_5prime_partial_len_158 | ---NA--- | | |
| TRINITY_DN26950_c0_g1_i1_len_474_243791.p1_type_internal_len_159 | ---NA--- | | |
| TRINITY_DN28228_c0_g1_i1_len_476_243702.p1_type_internal_len_159 | ---NA--- | | |
| TRINITY_DN34855_c0_g1_i1_len_476_243704.p1_type_internal_len_159 | ---NA--- | | |
| TRINITY_DN12334_c0_g1_i1_len_476_243729.p1_type_internal_len_159 | XP_018017197.1PREDICTED: uncharacterized protein LOC108673827 | | |
| TRINITY_DN829_c0_g1_i1_len_476_243735.p1_type_internal_len_159 | XP_021940399.1protein furry | P:GO:0000902 | |
| TRINITY_DN22424_c0_g1_i1_len_482_69603.p1_type_5prime_partial_len_158 | ---NA--- | | |
| TRINITY_DN10605_c0_g1_i1_len_483_9236.p1_type_5prime_partial_len_158 | XP_018008597.1 39S ribosomal protein L42, mitochondrial-like | | |
| TRINITY_DN2223_c0_g1_i1_len_493_243211.p1_type_5prime_partial_len_158 | XP_017491375.1PREDICTED: uncharacterized protein LOC108379532 | C:GO:0016021 | |
| TRINITY_DN5927_c0_g1_i1_len_471_9547.p1_type_internal_len_158 | XP_018020778.1 xylose isomerase-like | P:GO:0005975; F:GO:0009045; F:GO:0016853; P:GO:0042732; F:GO:0046872 | |
| TRINITY_DN30324_c0_g1_i1_len_6915_186822.p1_type_5prime_partial_len_1668 | EEC00457.1conserved hypothetical protein | P:GO:0007416; P:GO:0009653; P:GO:0009966; F:GO:0030159; C:GO:0030425; C:GO:0044309; C:GO:0044459; P:GO:0048699; P:GO:0050806; P:GO:0051240; P:GO:0065008; C:GO:0098794; P:GO:0099173 | |
| TRINITY_DN27746_c1_g1_i1_len_7065_186809.p1_type_complete_len_1667 | XP_018022368.1 AT-rich interactive domain-containing protein 2-like isoform X2 | | |
| TRINITY_DN30691_c0_g1_i1_len_6048_186924.p1_type_complete_len_1666 | XP_025077672.1ras GTPase-activating-like protein IQGAP1 isoform X4 | P:GO:0007165; P:GO:0043087 | |

| | | | |
|---|---|---|---|
| TRINITY_DN26316_c0_g1_i1_len_5254_227735.p1_type_complete_len_1662 | XP_018007311.1PREDICTED: uncharacterized protein LOC108665100 | | |
| TRINITY_DN23453_c0_g1_i1_len_5532_153404.p1_type_complete_len_1659 | ---NA--- | | |
| TRINITY_DN23591_c0_g1_i4_len_5100_121778.p1_type_5prime_partial_len_1656 | KFM70134.1Insulin receptor substrate 2-B | F:GO:0005158; P:GO:0008286 | |
| TRINITY_DN30602_c0_g1_i2_len_5285_187065.p1_type_complete_len_1653 | XP_018015325.1 putative GPI-anchored protein PB15E9.01c isoform X3 | | |
| TRINITY_DN20912_c0_g1_i1_len_5787_264346.p1_type_complete_len_1648 | XP_014786678.1 putative helicase Mov10l1 isoform X2 | | |
| TRINITY_DN31191_c0_g1_i2_len_5530_187007.p1_type_5prime_partial_len_1638 | ---NA--- | | |
| TRINITY_DN26137_c0_g1_i1_len_5302_227724.p1_type_5prime_partial_len_1636 | XP_018013031.1 condensin-2 complex subunit D3-like | | |
| 1759\|_\|c9803_f3p1_2807_isoform_c9803_full_length_coverage_3_non_full_length_coverage_1_isoform_length_2807_17310 | ---NA--- | | |
| 1761\|_\|c17849_f3p4_2806_isoform_c17849_full_length_coverage_3_non_full_length_coverage_4_isoform_length_2806_22510 | XP_008559226.1 glycine--tRNA ligase | | |
| 1762\|_\|c2401_f2p0_2806_isoform_c2401_full_length_coverage_2_non_full_length_coverage_0_isoform_length_2806_20911 | PNF14070.1T-complex protein 11-like protein 1 | | |
| 1763\|_\|c47030_f3p1_2806_isoform_c47030_full_length_coverage_3_non_full_length_coverage_1_isoform_length_2806_19399 | XP_014225487.2zinc finger protein 540-like | | |
| 1764\|_\|c8861_f2p7_2806_isoform_c8861_full_length_coverage_2_non_full_length_coverage_7_isoform_length_2806_12755 | XP_018011098.1 kinesin light chain-like isoform X4 | | |
| 1765\|_\|c2588_f3p0_2806_isoform_c2588_full_length_coverage_3_non_full_length_coverage_0_isoform_length_2806_21066 | XP_023220170.1protein FAM214A-like isoform X2 | | |

| | | | |
|---|---|---|---|
| TRINITY_DN26132_c0_g1_i2_len_3144_228824.p1_type_complete_len_935 | PNF41917.1SCY1-like protein 2 | F:GO:0004672; F:GO:0005524; P:GO:0006468 | |
| TRINITY_DN27825_c0_g1_i2_len_3354_188072.p1_type_complete_len_935 | PSN35638.1WD repeat-containing protein 47 | | |
| 1769\|_\|c5931_f2p13_2805_isoform_c5931_full_length_coverage_2_non_full_length_coverage_13_isoform_length_2805_17003 | ---NA--- | | |
| 1770\|_\|c3151_f2p0_2805_isoform_c3151_full_length_coverage_2_non_full_length_coverage_0_isoform_length_2805_16744 | ---NA--- | | |
| TRINITY_DN22012_c0_g1_i3_len_1124_195498.p1_type_complete_len_157 | ---NA--- | | |
| TRINITY_DN17016_c0_g1_i2_len_870_197805.p1_type_5prime_partial_len_157 | AEB54651.1ribosomal protein S9 | | |
| TRINITY_DN30358_c0_g1_i3_len_3059_188435.p2_type_complete_len_157 | ---NA--- | | |
| TRINITY_DN21352_c0_g1_i1_len_2646_91371.p2_type_complete_len_157 | ABY66597.1pyruvate kinase 2 | F:GO:0000287; F:GO:0004743; P:GO:0006096; F:GO:0016301; F:GO:0030955 | EC:2.7.1.40 |
| TRINITY_DN19827_c0_g1_i2_len_1402_193641.p2_type_complete_len_157 | XP_018027220.1 cyclin-dependent kinase 20-like | | |
| TRINITY_DN9627_c0_g1_i1_len_486_204692.p1_type_5prime_partial_len_157 | XP_018014926.1 tyrosine-protein phosphatase non-receptor type 13-like | | |
| TRINITY_DN26918_c0_g1_i1_len_1793_191692.p2_type_5prime_partial_len_157 | XP_022191943.1gastrula zinc finger protein XlCGF57.1-like | | |
| TRINITY_DN4127_c0_g1_i2_len_1168_32681.p2_type_complete_len_157 | XP_008556309.1 alkylated DNA repair protein alkB homolog 1 | F:GO:0016491; P:GO:0055114 | |
| TRINITY_DN23215_c0_g1_i1_len_700_199996.p1_type_5prime_partial_len_157 | KXJ10292.1putative uncharacterized transposon-derived protein F52C9.6 | | |
| TRINITY_DN17732_c0_g1_i1_len_692_200126.p1_type_3prime_partial_len_158 | XP_022166233.1E3 SUMO-protein ligase KIAA1586-like | | |
| TRINITY_DN19288_c0_g1_i1_len_616_66844.p1_type_3prime_partial_len_158 | ---NA--- | | |

| | | | |
|---|---|---|---|
| TRINITY_DN4060_c0_g1_i1_len_600_99428.p2_type_5prime_partial_len_157 | ---NA--- | | |
| TRINITY_DN20293_c0_g1_i3_len_3170_91079.p3_type_complete_len_157 | ---NA--- | | |
| TRINITY_DN9275_c0_g1_i1_len_672_273128.p1_type_5prime_partial_len_157 | XP_021923285.1cytoplasmic dynein 2 light intermediate chain 1 isoform X3 | C:GO:0005868; C:GO:0005929; P:GO:0035721; P:GO:0035735 | |
| TRINITY_DN33519_c0_g1_i1_len_473_243799.p2_type_internal_len_158 | XP_018019235.1PREDICTED: uncharacterized protein LOC108675721 | | |
| TRINITY_DN31085_c0_g1_i1_len_473_243798.p1_type_internal_len_158 | ---NA--- | | |
| TRINITY_DN28957_c0_g1_i1_len_473_243793.p1_type_internal_len_158 | XP_018025432.1 E3 ubiquitin-protein ligase SHPRH-like | | |
| TRINITY_DN37481_c0_g1_i1_len_474_243781.p1_type_internal_len_158 | XP_014257268.1interference hedgehog-like isoform X2 | | |
| TRINITY_DN29959_c0_g1_i1_len_474_243778.p1_type_internal_len_158 | AFJ59952.1cation-independent mannose-6-phosphate receptor | P:GO:0007041; F:GO:0038023; F:GO:0051219 | |
| TRINITY_DN14795_c0_g1_i1_len_474_243776.p1_type_internal_len_158 | ---NA--- | | |
| TRINITY_DN5062_c0_g1_i1_len_474_243767.p1_type_internal_len_158 | XP_018016317.1 kelch-like protein 18 | | |
| TRINITY_DN35436_c0_g1_i1_len_474_243765.p1_type_internal_len_158 | XP_023023185.1RNA-binding protein 27 | | |
| TRINITY_DN19150_c0_g1_i4_len_2941_154403.p2_type_complete_len_157 | ---NA--- | | |
| TRINITY_DN11903_c0_g1_i2_len_472_69887.p1_type_internal_len_158 | OAD55718.1Protein Malvolio | C:GO:0016021; P:GO:0030001; F:GO:0046873 | |
| TRINITY_DN3753_c0_g1_i1_len_471_69936.p1_type_internal_len_158 | ---NA--- | | |
| TRINITY_DN20056_c0_g1_i4_len_1434_31674.p1_type_complete_len_157 | AEN94573.1clathrin coat assembly protein ap19 | P:GO:0006886; P:GO:0016192; C:GO:0030117 | |
| TRINITY_DN28669_c0_g1_i1_len_2196_190259.p2_type_complete_len_157 | ---NA--- | | |

| | | | |
|---|---|---|---|
| TRINITY_DN21192_c0_g1_i1_len_2858_154500.p2_type_complete_len_157 | XP_018027119.1 transmembrane emp24 domain-containing protein 7-like | C:GO:0016021 | |
| TRINITY_DN3180_c0_g1_i1_len_472_243847.p1_type_internal_len_158 | ---NA--- | | |
| TRINITY_DN11394_c0_g1_i1_len_472_243846.p1_type_internal_len_158 | XP_018012551.1PREDICTED: uncharacterized protein LOC108669665 | | |
| TRINITY_DN6427_c0_g1_i1_len_472_243825.p1_type_internal_len_158 | KFM72725.1Hemicentin-1, partial | F:GO:0005509 | |
| TRINITY_DN8689_c0_g1_i1_len_472_243823.p1_type_internal_len_158 | ---NA--- | | |
| TRINITY_DN31069_c0_g1_i10_len_3572_187869.p1_type_complete_len_157 | ---NA--- | | |
| TRINITY_DN17963_c0_g1_i1_len_473_133029.p1_type_internal_len_158 | ---NA--- | | |
| TRINITY_DN5339_c0_g1_i1_len_473_205105.p1_type_internal_len_158 | ---NA--- | | |
| TRINITY_DN21345_c0_g1_i2_len_485_9192.p1_type_5prime_partial_len_157 | ---NA--- | | |
| TRINITY_DN10927_c0_g1_i1_len_1287_32172.p3_type_3prime_partial_len_158 | ---NA--- | | |
| TRINITY_DN4581_c0_g1_i2_len_631_201175.p1_type_5prime_partial_len_157 | XP_018007480.1 protein FAM179A-like | | |
| TRINITY_DN11838_c0_g1_i1_len_473_39317.p1_type_internal_len_158 | XP_013062667.1 canalicular multispecific organic anion transporter 2-like | F:GO:0005524; C:GO:0005887; F:GO:0008514; P:GO:0015711; F:GO:0042626; P:GO:0055085 | EC:3.6.1.3; EC:3.6.1.15 |
| TRINITY_DN6559_c0_g1_i1_len_473_205101.p2_type_internal_len_158 | XP_018027486.1 connectin-like | | |
| TRINITY_DN41416_c0_g1_i1_len_473_205104.p1_type_internal_len_158 | ---NA--- | | |
| TRINITY_DN24584_c0_g1_i2_len_473_205124.p1_type_internal_len_158 | ---NA--- | | |
| TRINITY_DN4915_c0_g1_i1_len_564_202554.p1_type_5prime_partial_len_157 | ---NA--- | | |

| | | | |
|---|---|---|---|
| TRINITY_DN4802_c0_g1_i1_len_1018_196401.p1_type_3prime_partial_len_158 | XP_018023492.1 lateral signaling target protein 2 homolog | | |
| TRINITY_DN31510_c0_g1_i1_len_474_276688.p1_type_internal_len_158 | ---NA--- | | |
| TRINITY_DN26267_c0_g1_i1_len_475_167729.p1_type_internal_len_158 | ---NA--- | | |
| TRINITY_DN32603_c0_g1_i1_len_474_101974.p1_type_internal_len_158 | XP_021959448.1BMP-binding endothelial regulator protein-like | | |
| TRINITY_DN10986_c0_g1_i1_len_472_276751.p1_type_internal_len_158 | ---NA--- | | |
| TRINITY_DN28806_c0_g1_i1_len_474_101970.p1_type_internal_len_158 | AVP26799.1C-type lectin | F:GO:0030246 | |
| TRINITY_DN11244_c0_g1_i1_len_1032_196285.p1_type_5prime_partial_len_157 | ---NA--- | | |
| TRINITY_DN7544_c0_g1_i1_len_565_202540.p1_type_5prime_partial_len_157 | XP_018027327.1PREDICTED: uncharacterized protein LOC108682635 | C:GO:0016020; C:GO:0016021 | |
| TRINITY_DN33291_c0_g1_i1_len_471_205190.p1_type_internal_len_158 | XP_018011803.1 dachshund homolog 1-like isoform X3 | | |
| TRINITY_DN22168_c0_g1_i1_len_491_204540.p1_type_5prime_partial_len_157 | XP_018014155.1 U6 snRNA-associated Sm-like protein LSm1 | | |
| TRINITY_DN8063_c0_g1_i1_len_472_39348.p1_type_internal_len_158 | ---NA--- | | |
| TRINITY_DN3246_c0_g1_i1_len_473_205119.p1_type_internal_len_158 | XP_018019765.1 protocadherin Fat 1-like | | |
| TRINITY_DN38830_c0_g1_i1_len_500_204276.p1_type_3prime_partial_len_158 | XP_018332337.1BTB/POZ domain-containing protein KCTD5 | P:GO:0051260 | |
| TRINITY_DN26420_c0_g1_i1_len_471_304843.p1_type_internal_len_158 | EFN81739.1Hermansky-Pudlak syndrome 3 protein | | |
| TRINITY_DN13590_c0_g1_i1_len_474_205078.p2_type_internal_len_158 | XP_018011984.1 delta-like protein B | F:GO:0005509; P:GO:0007219; P:GO:0007275; C:GO:0016021 | |
| TRINITY_DN4819_c0_g1_i1_len_611_164817.p1_type_5prime_partial_len_157 | AHN10846.1viral responsive protein | C:GO:0016021 | |

| | | | |
|---|---|---|---|
| TRINITY_DN34865_c0_g1_i1_len_861_237324.p1_type_5prime_partial_len_157 | ---NA--- | | |
| TRINITY_DN23146_c0_g1_i2_len_2197_230526.p2_type_complete_len_157 | ---NA--- | | |
| TRINITY_DN17622_c0_g1_i1_len_690_129115.p1_type_5prime_partial_len_157 | ---NA--- | | |
| TRINITY_DN20530_c0_g1_i1_len_2558_265394.p3_type_complete_len_157 | XP_018022215.1 solute carrier family 28 member 3-like | | |
| TRINITY_DN10248_c0_g1_i1_len_609_6853.p1_type_3prime_partial_len_158 | ---NA--- | | |
| TRINITY_DN15597_c0_g1_i1_len_474_205098.p1_type_internal_len_158 | ---NA--- | | |
| TRINITY_DN12805_c0_g1_i2_len_472_304828.p1_type_internal_len_158 | XP_017260359.1probable palmitoyltransferase ZDHHC14 | | |
| TRINITY_DN2949_c0_g1_i1_len_475_205031.p1_type_internal_len_158 | ---NA--- | | |
| TRINITY_DN13171_c0_g1_i1_len_498_204334.p1_type_3prime_partial_len_158 | XP_014871920.1 SH3 domain-containing kinase-binding protein 1-like isoform X3 | | |
| TRINITY_DN13814_c0_g1_i1_len_473_304798.p1_type_internal_len_158 | ---NA--- | | |
| TRINITY_DN24292_c0_g1_i1_len_509_203957.p1_type_5prime_partial_len_157 | ---NA--- | | |
| TRINITY_DN3897_c0_g1_i1_len_472_205135.p1_type_internal_len_158 | XP_023237631.1low-density lipoprotein receptor-related protein 6-like | C:GO:0016020 | |
| TRINITY_DN930_c0_g1_i1_len_728_238782.p1_type_complete_len_157 | ---NA--- | | |
| TRINITY_DN30634_c0_g1_i3_len_995_196594.p1_type_complete_len_157 | XP_018026285.1 heat shock protein 67B2-like | | |
| TRINITY_DN18567_c0_g1_i1_len_473_205125.p1_type_3prime_partial_len_158 | ---NA--- | | |
| TRINITY_DN21813_c0_g2_i2_len_3221_91062.p3_type_complete_len_157 | ---NA--- | | |

| | | | |
|---|---|---|---|
| TRINITY_DN10601_c0_g1_i1_len_471_167834.p1_type_internal_len_158 | ---NA--- | | |
| TRINITY_DN14259_c0_g1_i2_len_471_102062.p1_type_internal_len_158 | ---NA--- | | |
| TRINITY_DN13336_c0_g1_i1_len_473_9499.p2_type_internal_len_158 | ---NA--- | | |
| TRINITY_DN836_c0_g1_i1_len_548_7850.p1_type_5prime_partial_len_157 | ---NA--- | | |
| TRINITY_DN26877_c0_g1_i1_len_472_102043.p1_type_internal_len_158 | AKL71620.1juvenile hormone epoxide hydrolase | C:GO:0005789; P:GO:0019439; F:GO:0033961 | EC:3.3.2.9 |
| TRINITY_DN25519_c0_g1_i1_len_651_239935.p1_type_5prime_partial_len_157 | ---NA--- | | |
| TRINITY_DN30866_c0_g1_i1_len_473_167774.p1_type_internal_len_158 | ---NA--- | | |
| TRINITY_DN1959_c0_g1_i2_len_473_167760.p1_type_internal_len_158 | ---NA--- | | |
| TRINITY_DN18063_c0_g1_i1_len_473_9481.p1_type_internal_len_158 | XP_019866683.1 zwei Ig domain protein zig-8 isoform X3 | | |
| TRINITY_DN19169_c0_g1_i1_len_472_205153.p1_type_internal_len_157 | ---NA--- | | |
| TRINITY_DN16126_c0_g1_i1_len_1093_195754.p1_type_complete_len_156 | XP_018027505.1 protein SYS1 homolog | C:GO:0005794; C:GO:0016020 | |
| TRINITY_DN29580_c0_g1_i1_len_2002_190893.p2_type_complete_len_156 | PNF20513.1Protein rogdi | | |
| TRINITY_DN33409_c0_g1_i1_len_470_205214.p1_type_internal_len_157 | ---NA--- | | |
| TRINITY_DN21856_c1_g1_i1_len_470_205204.p2_type_internal_len_157 | ---NA--- | | |
| TRINITY_DN20929_c0_g1_i1_len_2037_190770.p4_type_complete_len_156 | XP_019731092.1 polycomb complex protein BMI-1 | | |
| TRINITY_DN5058_c0_g1_i1_len_472_304820.p1_type_internal_len_157 | ---NA--- | | |

| | | | |
|---|---|---|---|
| TRINITY_DN32143_c0_g1_i1_len_471_205162.p1_type_internal_len_157 | XP_018027076.1 otoferlin-like | | |
| TRINITY_DN10923_c0_g1_i1_len_469_304888.p2_type_internal_len_157 | ---NA--- | | |
| TRINITY_DN13940_c0_g1_i2_len_490_243306.p1_type_5prime_partial_len_156 | ---NA--- | | |
| TRINITY_DN25387_c0_g1_i1_len_1982_190971.p2_type_5prime_partial_len_156 | XP_018016242.1 calcium uptake protein 1 homolog, mitochondrial-like | F:GO:0005509; P:GO:0006851 | |
| TRINITY_DN10521_c0_g1_i1_len_469_304903.p1_type_internal_len_157 | ---NA--- | | |
| TRINITY_DN12692_c0_g1_i1_len_469_304899.p1_type_internal_len_157 | ---NA--- | | |
| TRINITY_DN16720_c0_g1_i1_len_471_205164.p1_type_internal_len_157 | XP_015794848.1mucin-5AC-like isoform X2 | C:GO:0005576; P:GO:0006030; F:GO:0008061 | |
| TRINITY_DN19840_c0_g1_i1_len_1050_298536.p2_type_complete_len_156 | ---NA--- | | |
| TRINITY_DN37208_c0_g1_i1_len_470_205211.p1_type_internal_len_157 | ABP97431.1alpha 2 macroglobulin | F:GO:0004866; C:GO:0005615; P:GO:0010951 | |
| 1772\|_\|c2520_f3p0_2804_isoform_c2520_full_length_coverage_3_non_full_length_coverage_0_isoform_length_2804_16477 | XP_018015918.1 ATP-dependent RNA helicase abstrakt-like | | |
| 1773\|_\|c11163_f2p2_2803_isoform_c11163_full_length_coverage_2_non_full_length_coverage_2_isoform_length_2803_12948 | XP_018019666.1 structural maintenance of chromosomes protein 3-like isoform X1 | | |
| TRINITY_DN25831_c0_g1_i1_len_4316_228019.p1_type_5prime_partial_len_934 | XP_023714578.1ankyrin repeat domain-containing protein 27-like | | |
| 1776\|_\|c9398_f2p1_2802_isoform_c9398_full_length_coverage_2_non_full_length_coverage_1_isoform_length_2802_12800 | XP_018027249.1 protein-cysteine N-palmitoyltransferase HHAT-like | | |
| TRINITY_DN30511_c0_g1_i1_len_6934_186821.p1_type_complete_len_934 | AIN75603.1disintegrin and metalloproteinase domain-containing protein 10 | F:GO:0004222; P:GO:0006508; P:GO:0007229; C:GO:0016021 | EC:3.4.24 |

| | | | |
|---|---|---|---|
| TRINITY_DN27510_c0_g1_i1_len_3094_188383.p1_type_complete_len_934 | XP_021914641.1protein tamozhennic | P:GO:0007275; P:GO:0015031; F:GO:0046872 | |
| 1779\|_\|c1893_f2p6_2802_isoform_c1893_full_length_coverage_2_non_full_length_coverage_6_isoform_length_2802_28054 | PNF34955.1hypothetical protein B7P43_G15617 | P:GO:0035556 | |
| 1780\|_\|c17807_f3p2_2802_isoform_c17807_full_length_coverage_3_non_full_length_coverage_2_isoform_length_2802_17805 | ---NA--- | | |
| 1781\|_\|c17981_f2p6_2802_isoform_c17981_full_length_coverage_2_non_full_length_coverage_6_isoform_length_2802_22607 | ---NA--- | | |
| 1782\|_\|c2658_f2p1_2801_isoform_c2658_full_length_coverage_2_non_full_length_coverage_1_isoform_length_2801_21126 | ---NA--- | | |
| TRINITY_DN25489_c0_g1_i1_len_1360_193896.p2_type_3prime_partial_len_157 | XP_018014525.1 haloalkane dehalogenase-like | | |
| TRINITY_DN29103_c0_g1_i1_len_769_272069.p1_type_complete_len_156 | XP_023716859.1UPF0687 protein C20orf27 homolog isoform X1 | | |
| TRINITY_DN30442_c0_g1_i1_len_468_243961.p1_type_internal_len_157 | ---NA--- | | |
| TRINITY_DN29534_c0_g1_i1_len_470_276801.p1_type_internal_len_157 | ---NA--- | | |
| TRINITY_DN18183_c0_g1_i1_len_1656_123823.p2_type_complete_len_156 | XP_018020386.1 solute carrier family 25 member 38-A-like isoform X2 | C:GO:0005739; C:GO:0016020; F:GO:0022857; P:GO:0055085 | |
| TRINITY_DN13292_c0_g1_i1_len_469_243954.p1_type_internal_len_157 | ---NA--- | | |
| TRINITY_DN36917_c0_g1_i1_len_470_243916.p1_type_internal_len_157 | XP_018019355.1 myosin-IA-like | F:GO:0003774; F:GO:0003779; F:GO:0005524; C:GO:0016459 | EC:3.6.1.15 |
| TRINITY_DN7484_c0_g1_i1_len_470_243913.p1_type_internal_len_157 | KFM80855.1SPRY domain-containing protein 3 | | |
| TRINITY_DN2619_c0_g1_i1_len_470_276789.p1_type_internal_len_157 | ---NA--- | | |

| | | | |
|---|---|---|---|
| TRINITY_DN35695_c0_g1_i1_len_471_205182.p1_type_internal_len_157 | ---NA--- | | |
| TRINITY_DN33066_c0_g1_i1_len_468_243978.p1_type_internal_len_157 | ---NA--- | | |
| TRINITY_DN31139_c0_g1_i1_len_468_243971.p1_type_internal_len_157 | ADE44090.1phosphoinositide 3-kinase isoform a | F:GO:0005524; F:GO:0016303; P:GO:0036092; P:GO:0046854; F:GO:0046934; P:GO:0048015 | EC:2.7.1.153; EC:2.7.1.137 |
| TRINITY_DN34959_c0_g1_i1_len_472_243845.p1_type_internal_len_157 | ---NA--- | | |
| TRINITY_DN26711_c0_g1_i1_len_472_243835.p1_type_internal_len_157 | XP_018007126.1 transient receptor potential channel pyrexia-like | | |
| TRINITY_DN25065_c0_g2_i1_len_2050_230960.p1_type_complete_len_156 | ---NA--- | | |
| TRINITY_DN30963_c0_g1_i1_len_470_243911.p1_type_internal_len_157 | XP_018022814.1 nucleosome-remodeling factor subunit NURF301-like | C:GO:0016589; F:GO:0035064; F:GO:0046872 | |
| TRINITY_DN32825_c0_g1_i1_len_470_243910.p1_type_internal_len_157 | XP_008559134.1PREDICTED: uncharacterized protein LOC103579497 isoform X2 | | |
| TRINITY_DN3168_c0_g1_i1_len_471_243882.p1_type_internal_len_157 | XP_018010812.1 dual specificity protein phosphatase 22-like | P:GO:0006470; F:GO:0008138 | EC:3.1.3.16 |
| TRINITY_DN10814_c0_g1_i1_len_471_243880.p1_type_internal_len_157 | ---NA--- | | |
| TRINITY_DN100_c0_g1_i1_len_471_243871.p1_type_internal_len_157 | AAC61691.1inositol 1,4,5-trisphosphate receptor | F:GO:0005220; C:GO:0005783; C:GO:0016021; P:GO:0048016; P:GO:0070588; F:GO:0070679 | |
| TRINITY_DN8100_c0_g1_i1_len_472_243853.p1_type_internal_len_157 | ---NA--- | | |
| TRINITY_DN10972_c0_g1_i1_len_602_201735.p1_type_5prime_partial_len_156 | AJO70032.1neuroglobin isoform 2 | F:GO:0005344; P:GO:0015671; F:GO:0019825; F:GO:0020037; F:GO:0046872 | |
| TRINITY_DN19365_c0_g1_i1_len_2628_29680.p2_type_complete_len_156 | ---NA--- | | |

| | | | |
|---|---|---|---|
| TRINITY_DN36137_c0_g1_i1_len_481_243577.p1_type_5prime_partial_len_156 | AEX08659.1lipopolysaccharide and beta-1,3-glucan binding protein | F:GO:0001872; F:GO:0004553; C:GO:0005576; P:GO:0005975; P:GO:0045087 | |
| TRINITY_DN7917_c0_g1_i1_len_468_70047.p1_type_internal_len_157 | XP_018023003.1 transmembrane protein 184C-like | C:GO:0016021 | |
| TRINITY_DN3017_c0_g1_i1_len_472_102032.p1_type_internal_len_157 | ---NA--- | | |
| TRINITY_DN33202_c0_g1_i1_len_472_205139.p1_type_internal_len_157 | XP_026672112.1transmembrane protein KIAA1109 homolog | C:GO:0016020 | |
| TRINITY_DN22956_c1_g1_i2_len_3482_228496.p2_type_complete_len_156 | ---NA--- | | |
| TRINITY_DN26540_c0_g1_i1_len_615_240542.p2_type_complete_len_156 | XP_019643620.1 ryncolin-1-like isoform X3 | | |
| TRINITY_DN6985_c0_g1_i1_len_469_276818.p1_type_internal_len_157 | XP_018011432.1 plastin-1-like | F:GO:0005509; F:GO:0051015; P:GO:0051017 | |
| TRINITY_DN25555_c0_g1_i1_len_469_70003.p1_type_internal_len_157 | XP_018015529.1 protein ABHD17B-like | F:GO:0016787 | |
| TRINITY_DN40983_c0_g1_i1_len_471_205194.p1_type_internal_len_157 | ---NA--- | | |
| TRINITY_DN15134_c0_g1_i1_len_723_199672.p1_type_5prime_partial_len_156 | KFM57640.1Sorting nexin-6 | P:GO:0015031; F:GO:0035091 | |
| TRINITY_DN5711_c0_g1_i1_len_468_133149.p1_type_internal_len_157 | ---NA--- | | |
| TRINITY_DN26545_c0_g1_i1_len_469_9589.p1_type_3prime_partial_len_157 | XP_018015914.1PREDICTED: uncharacterized protein LOC108672708 | | |
| TRINITY_DN16758_c0_g1_i1_len_469_205256.p1_type_internal_len_157 | ---NA--- | | |
| TRINITY_DN15927_c0_g1_i3_len_481_132849.p1_type_complete_len_156 | AHL26189.1lipoprotein receptor 1 | F:GO:0005509; C:GO:0016020; C:GO:0016021 | |
| TRINITY_DN18679_c0_g1_i1_len_469_205241.p1_type_internal_len_157 | ---NA--- | | |
| TRINITY_DN20293_c0_g1_i1_len_2169_30111.p2_type_5prime_partial_len_156 | ---NA--- | | |

| | | | |
|---|---|---|---|
| TRINITY_DN10852_c0_g1_i1_len_641_66466.p1_type_5prime_partial_len_156 | XP_018019761.1 flocculation protein FLO11-like | | |
| TRINITY_DN11149_c0_g1_i1_len_472_133060.p1_type_internal_len_157 | ---NA--- | | |
| TRINITY_DN4683_c0_g1_i1_len_470_39391.p1_type_internal_len_157 | ---NA--- | | |
| TRINITY_DN1537_c0_g1_i1_len_553_241717.p1_type_3prime_partial_len_157 | PNF21813.1Membrane-bound transcription factor site-1 protease | F:GO:0004252; P:GO:0006508 | EC:3.4.21 |
| TRINITY_DN27444_c0_g1_i1_len_553_241710.p1_type_3prime_partial_len_157 | XP_021914302.1transmembrane protein 181 isoform X1 | C:GO:0016020 | |
| TRINITY_DN28739_c0_g1_i2_len_1789_191705.p2_type_3prime_partial_len_157 | ---NA--- | | |
| TRINITY_DN33592_c0_g1_i1_len_468_205265.p1_type_internal_len_157 | XP_018016072.1 active breakpoint cluster region-related protein-like | | |
| TRINITY_DN9133_c0_g1_i1_len_471_9556.p1_type_internal_len_157 | AKZ75938.1cuticle protein 2 | F:GO:0042302 | |
| TRINITY_DN9441_c0_g1_i4_len_469_39431.p1_type_internal_len_157 | ---NA--- | | |
| TRINITY_DN27074_c1_g1_i2_len_1076_195882.p1_type_3prime_partial_len_157 | ---NA--- | | |
| TRINITY_DN13232_c0_g1_i1_len_469_39423.p1_type_internal_len_157 | ---NA--- | | |
| TRINITY_DN26441_c0_g1_i1_len_468_167934.p1_type_internal_len_157 | ---NA--- | | |
| TRINITY_DN26984_c0_g1_i1_len_468_167939.p1_type_internal_len_157 | PSN54724.1E3 ubiquitin-protein ligase TRIM23 | F:GO:0003676; F:GO:0005525; F:GO:0008270; F:GO:0016874 | |
| TRINITY_DN36723_c0_g1_i1_len_566_241468.p1_type_5prime_partial_len_156 | ---NA--- | | |
| TRINITY_DN25528_c0_g1_i1_len_4516_227935.p2_type_3prime_partial_len_157 | ---NA--- | | |
| TRINITY_DN4575_c0_g1_i1_len_470_205216.p1_type_internal_len_157 | ---NA--- | | |

| | | | |
|---|---|---|---|
| TRINITY_DN26338_c0_g1_i5_len_1156_234995.p1_type_3prime_partial_len_157 | ---NA--- | | |
| TRINITY_DN16491_c0_g1_i2_len_470_205217.p2_type_internal_len_157 | ---NA--- | | |
| TRINITY_DN19910_c0_g2_i1_len_776_97273.p1_type_complete_len_156 | PRD25906.1zinc finger protein | F:GO:0003677 | |
| TRINITY_DN13041_c0_g1_i1_len_593_240930.p1_type_5prime_partial_len_156 | XP_018020121.1 vesicle-associated membrane protein 4-like | C:GO:0016020 | |
| TRINITY_DN25681_c0_g1_i2_len_1448_193372.p1_type_complete_len_156 | XP_013776647.1PEST proteolytic signal-containing nuclear protein-like isoform X2 | | |
| TRINITY_DN24004_c0_g1_i3_len_3858_228256.p2_type_complete_len_156 | AKV88638.1TGF-beta-activated kinase 1 and MAP3K7-binding protein 2 | F:GO:0016301; P:GO:0016310; F:GO:0046872 | |
| TRINITY_DN22393_c0_g1_i1_len_1708_1217.p3_type_5prime_partial_len_156 | ---NA--- | | |
| TRINITY_DN19175_c0_g1_i1_len_1558_267523.p2_type_3prime_partial_len_157 | XP_018025051.1 sulfotransferase 1A3-like | | |
| TRINITY_DN34061_c0_g1_i1_len_465_168031.p1_type_internal_len_156 | PSN33675.1putative E3 ubiquitin-protein ligase HERC1 | F:GO:0004842; P:GO:0016567; F:GO:0016874 | |
| TRINITY_DN11242_c0_g1_i2_len_466_304972.p1_type_internal_len_156 | ---NA--- | | |
| TRINITY_DN9219_c0_g1_i1_len_467_304960.p2_type_internal_len_156 | ---NA--- | | |
| TRINITY_DN14728_c0_g1_i2_len_467_276873.p1_type_internal_len_156 | ---NA--- | | |
| TRINITY_DN17771_c0_g1_i1_len_467_304964.p1_type_3prime_partial_len_156 | XP_026051402.1uncharacterized protein LOC113038303 | | |
| TRINITY_DN3838_c0_g1_i1_len_465_168035.p1_type_internal_len_156 | ---NA--- | | |
| TRINITY_DN31004_c0_g1_i1_len_467_304945.p1_type_internal_len_156 | AEE36486.1protein disulfide isomerase 2 | F:GO:0003756; C:GO:0005623; P:GO:0045454 | EC:5.3.4.1 |
| TRINITY_DN8254_c0_g1_i1_len_498_304229.p1_type_3prime_partial_len_156 | ---NA--- | | |

| | | | |
|---|---|---|---|
| TRINITY_DN22219_c0_g1_i1_len_468_304937.p1_type_internal_len_156 | AWU67130.1putative phospholipase A2 | | |
| TRINITY_DN24822_c0_g1_i1_len_468_276848.p1_type_internal_len_156 | ---NA--- | | |
| TRINITY_DN29247_c0_g1_i1_len_646_273472.p1_type_complete_len_155 | XP_018026648.1 plasminogen receptor (KT)-like isoform X1 | | |
| TRINITY_DN29249_c0_g1_i1_len_467_167987.p1_type_internal_len_156 | PSN43169.1hypothetical protein C0J52_09893 | F:GO:0005096; C:GO:0005634; P:GO:0032007; C:GO:0033596; P:GO:0043547; P:GO:0051056 | |
| TRINITY_DN8749_c0_g1_i1_len_466_205331.p1_type_internal_len_156 | ADM88556.1fatty acid synthase | F:GO:0004312; P:GO:0009058; F:GO:0016491; F:GO:0016788; F:GO:0031177; P:GO:0055114 | EC:2.3.1.85 |
| TRINITY_DN8380_c0_g1_i1_len_934_161213.p1_type_5prime_partial_len_155 | ---NA--- | | |
| TRINITY_DN2942_c0_g1_i1_len_465_102223.p1_type_internal_len_156 | AFA42361.1clip domain serine proteinase 3 | F:GO:0008233 | |
| TRINITY_DN17885_c0_g1_i1_len_897_299431.p2_type_complete_len_155 | XP_005089073.1 tubulin-folding cofactor B-like | | |
| TRINITY_DN31980_c0_g1_i1_len_465_205391.p1_type_internal_len_156 | OWR44115.1WD repeat protein 7 | | |
| TRINITY_DN23270_c0_g1_i1_len_605_201661.p1_type_3prime_partial_len_156 | ---NA--- | | |
| TRINITY_DN15597_c0_g1_i1_len_514_68775.p1_type_5prime_partial_len_155 | ---NA--- | | |
| TRINITY_DN22401_c0_g1_i2_len_2281_155425.p2_type_complete_len_155 | XP_026688809.1protein ALP1-like | | |
| TRINITY_DN20445_c0_g1_i1_len_465_205394.p1_type_internal_len_156 | ---NA--- | | |
| TRINITY_DN5113_c0_g1_i1_len_465_205387.p1_type_internal_len_156 | XP_016837097.1 venom serine protease Bi-VSP | F:GO:0008233; C:GO:0044459 | |
| TRINITY_DN23142_c0_g1_i1_len_466_276903.p1_type_internal_len_156 | ABO33174.1serine protease-like protein 3 | F:GO:0004252; P:GO:0006508 | EC:3.4.21 |

| | | | |
|---|---|---|---|
| TRINITY_DN35878_c0_g1_i1_len_466_205333.p2_type_internal_len_156 | ---NA--- | | |
| TRINITY_DN31695_c0_g1_i1_len_578_202247.p1_type_5prime_partial_len_155 | ANW81830.1calcium-calmodulin dependent protein kinase I | F:GO:0004674; F:GO:0005524; P:GO:0006468 | EC:2.7.11 |
| TRINITY_DN19177_c0_g1_i1_len_1628_232418.p1_type_5prime_partial_len_155 | XP_018007614.1 golgin subfamily A member 7-like isoform X2 | | |
| TRINITY_DN17540_c0_g1_i4_len_901_161485.p1_type_5prime_partial_len_155 | ---NA--- | | |
| TRINITY_DN21196_c0_g1_i2_len_613_99237.p1_type_5prime_partial_len_155 | ---NA--- | | |
| TRINITY_DN26504_c0_g1_i1_len_467_244015.p2_type_internal_len_156 | ---NA--- | | |
| TRINITY_DN37812_c0_g1_i1_len_467_243998.p1_type_internal_len_156 | ---NA--- | | |
| TRINITY_DN36897_c0_g1_i1_len_468_243986.p2_type_internal_len_156 | ATU31747.1Calcium-activated chloride channel regulator 2 | | |
| TRINITY_DN36529_c0_g1_i1_len_466_244034.p1_type_internal_len_156 | XP_026277867.1oxysterol-binding protein-related protein 11-like | | |
| TRINITY_DN36539_c0_g1_i1_len_466_244032.p1_type_internal_len_156 | XP_018022782.1 glutamine-dependent NAD(+) synthetase-like | F:GO:0000166; P:GO:0006807; F:GO:0016874 | |
| TRINITY_DN18626_c0_g1_i4_len_924_96101.p1_type_5prime_partial_len_155 | ---NA--- | | |
| TRINITY_DN28685_c0_g1_i1_len_466_244031.p1_type_internal_len_156 | ACU31810.1alpha2 macroglobulin isoform 2 | F:GO:0004866; C:GO:0005615; P:GO:0010951 | |
| TRINITY_DN29140_c0_g1_i1_len_467_244020.p2_type_internal_len_156 | ---NA--- | | |
| TRINITY_DN37113_c0_g1_i1_len_467_244018.p1_type_internal_len_156 | ---NA--- | | |
| 1783\|_\|c4539_f2p15_2801_isoform_c4539_full_length_coverage_2_non_full_length_coverage_15_isoform_length_2801_25727 | ---NA--- | | |

| | | | |
|---|---|---|---|
| 1785\|_\|c10069_f2p1_2800_isoform_c10069_full_length_coverage_2_non_full_length_coverage_1_isoform_length_2800_17326 | XP_018023130.1 protein abrupt-like isoform X3 | | |
| TRINITY_DN24575_c0_g1_i2_len_3089_228895.p1_type_5prime_partial_len_933 | ---NA--- | | |
| 1787\|_\|c5920_f2p2_2799_isoform_c5920_full_length_coverage_2_non_full_length_coverage_2_isoform_length_2799_21566 | ---NA--- | | |
| 1788\|_\|c2759_f4p4_2799_isoform_c2759_full_length_coverage_4_non_full_length_coverage_4_isoform_length_2799_12137 | ---NA--- | | |
| TRINITY_DN25191_c0_g1_i1_len_2936_188638.p1_type_5prime_partial_len_933 | AVN99027.1eye-specific diacylglycerol kinase isoform X3 | F:GO:0003951; F:GO:0004143; F:GO:0005524; P:GO:0007205; P:GO:0016310; P:GO:0035556 | EC:2.7.1.23; EC:2.7.1.107 |
| TRINITY_DN20542_c0_g1_i2_len_3208_264820.p1_type_5prime_partial_len_933 | XP_018011683.1 tyrosine-protein phosphatase non-receptor type 23-like | F:GO:0004721; F:GO:0004725; P:GO:0006470; P:GO:0016311; F:GO:0016787; F:GO:0016791; P:GO:0035335 | |
| TRINITY_DN26970_c0_g1_i2_len_3345_188083.p1_type_complete_len_933 | XP_013393278.1peroxisome assembly factor 2 | F:GO:0000166; F:GO:0005524 | |
| 1793\|_\|c2371_f5p1_2799_isoform_c2371_full_length_coverage_5_non_full_length_coverage_1_isoform_length_2799_16360 | XP_022249325.1integrator complex subunit 14-like | | |
| 1794\|_\|c4461_f2p5_2798_isoform_c4461_full_length_coverage_2_non_full_length_coverage_5_isoform_length_2798_21431 | ---NA--- | | |
| TRINITY_DN31451_c0_g1_i2_len_468_133142.p1_type_internal_len_156 | ---NA--- | | |
| TRINITY_DN39270_c0_g1_i1_len_466_205351.p1_type_internal_len_156 | ---NA--- | | |
| TRINITY_DN33718_c0_g1_i1_len_468_243972.p1_type_internal_len_156 | ---NA--- | | |

| | | | |
|---|---|---|---|
| TRINITY_DN9125_c0_g1_i1_len_477_276602.p1_type_5prime_partial_len_155 | ---NA--- | | |
| TRINITY_DN15406_c0_g1_i1_len_606_99327.p1_type_5prime_partial_len_155 | ---NA--- | | |
| TRINITY_DN29691_c0_g1_i1_len_734_65272.p1_type_5prime_partial_len_155 | AGW80518.1elongation factor 1-alpha 1 | F:GO:0003746; P:GO:0006414 | |
| TRINITY_DN34161_c0_g1_i1_len_466_244054.p1_type_internal_len_156 | ---NA--- | | |
| TRINITY_DN37207_c0_g1_i1_len_465_244070.p2_type_internal_len_156 | XP_018007685.1 peptidyl-prolyl cis-trans isomerase FKBP9-like | P:GO:0000413; F:GO:0003755; F:GO:0016853 | |
| TRINITY_DN32131_c0_g1_i1_len_465_244064.p1_type_internal_len_156 | ---NA--- | | |
| TRINITY_DN33404_c0_g1_i1_len_465_205362.p1_type_internal_len_156 | XP_022200606.1palmitoyltransferase Hip14-like | C:GO:0016020; F:GO:0016409 | |
| TRINITY_DN947_c0_g1_i1_len_466_205344.p1_type_internal_len_156 | ---NA--- | | |
| TRINITY_DN16089_c0_g1_i1_len_930_236701.p1_type_complete_len_155 | ---NA--- | | |
| TRINITY_DN9146_c0_g1_i1_len_494_304305.p1_type_5prime_partial_len_155 | ---NA--- | | |
| TRINITY_DN9536_c0_g1_i1_len_726_97779.p1_type_complete_len_155 | ADD70028.1myosin light chain | F:GO:0005509 | |
| TRINITY_DN13073_c0_g1_i1_len_651_129589.p1_type_complete_len_155 | XP_018014714.1 troponin C-like | F:GO:0005509 | |
| TRINITY_DN25655_c0_g1_i1_len_468_304910.p1_type_internal_len_156 | ---NA--- | | |
| TRINITY_DN16430_c1_g1_i1_len_1880_266582.p6_type_5prime_partial_len_155 | ---NA--- | | |
| TRINITY_DN5021_c0_g1_i1_len_467_133174.p1_type_internal_len_156 | ---NA--- | | |
| TRINITY_DN1950_c0_g1_i1_len_582_130668.p1_type_5prime_partial_len_155 | ---NA--- | | |

| | | | |
|---|---|---|---|
| TRINITY_DN29394_c0_g1_i1_len_500_276047.p1_type_5prime_partial_len_155 | ---NA--- | | |
| TRINITY_DN21072_c0_g1_i2_len_2668_229490.p2_type_complete_len_155 | ---NA--- | | |
| TRINITY_DN13209_c0_g1_i1_len_759_5175.p1_type_5prime_partial_len_155 | ---NA--- | | |
| TRINITY_DN17078_c0_g1_i1_len_797_162448.p1_type_complete_len_155 | XP_018009507.1 transmembrane protein 42-like | | |
| TRINITY_DN18503_c0_g1_i1_len_729_199592.p1_type_complete_len_155 | ---NA--- | | |
| TRINITY_DN2088_c0_g1_i1_len_468_70036.p1_type_internal_len_156 | ---NA--- | | |
| TRINITY_DN9795_c0_g1_i1_len_467_205310.p1_type_internal_len_156 | XP_010879262.1 NCK-interacting protein with SH3 domain | | |
| TRINITY_DN10615_c0_g1_i1_len_574_202327.p1_type_3prime_partial_len_156 | ---NA--- | | |
| TRINITY_DN18309_c0_g1_i1_len_465_9699.p1_type_internal_len_156 | ---NA--- | | |
| TRINITY_DN25178_c0_g1_i2_len_3609_228395.p1_type_3prime_partial_len_156 | ---NA--- | | |
| TRINITY_DN19511_c0_g1_i3_len_665_98510.p2_type_5prime_partial_len_155 | ---NA--- | | |
| TRINITY_DN37325_c0_g1_i1_len_468_205298.p1_type_internal_len_156 | EEB18891.1jumonji/arid domain-containing protein, putative | | |
| TRINITY_DN12647_c0_g1_i1_len_467_205302.p2_type_internal_len_156 | ---NA--- | | |
| TRINITY_DN31791_c0_g1_i1_len_467_205319.p1_type_internal_len_156 | XP_018019495.1 alpha-aminoadipic semialdehyde synthase, mitochondrial-like | | |
| TRINITY_DN2592_c0_g1_i1_len_467_205320.p1_type_internal_len_156 | KZS20145.1Protein SZT2 | C:GO:0005777 | |
| TRINITY_DN217_c0_g1_i1_len_467_205313.p1_type_internal_len_156 | ---NA--- | | |

| | | | |
|---|---|---|---|
| TRINITY_DN4666_c0_g1_i1_len_468_70008.p1_type_internal_len_156 | CAB85963.1alpha glucosidase | P:GO:0000023; F:GO:0004558; F:GO:0030246; F:GO:0032450 | EC:3.2.1.20 |
| TRINITY_DN20878_c0_g2_i1_len_468_70017.p1_type_internal_len_156 | XP_023726981.1glycoprotein 3-alpha-L-fucosyltransferase A-like | | |
| TRINITY_DN35751_c0_g1_i1_len_468_70032.p1_type_internal_len_156 | XP_013399086.1guanine deaminase-like isoform X2 | F:GO:0016810 | |
| TRINITY_DN21867_c0_g2_i1_len_715_97896.p1_type_complete_len_155 | ---NA--- | | |
| TRINITY_DN12621_c0_g1_i1_len_468_205286.p1_type_internal_len_156 | ---NA--- | | |
| TRINITY_DN10383_c0_g1_i1_len_468_39462.p1_type_internal_len_156 | XP_026274471.1homeodomain-interacting protein kinase 2 | F:GO:0003677; F:GO:0004672; F:GO:0005524; P:GO:0006468 | |
| TRINITY_DN20497_c0_g1_i1_len_1950_295827.p2_type_5prime_partial_len_155 | PSN42690.1Ubiquinol-cytochrome-c reductase complex assembly factor 2 | C:GO:0005739; P:GO:0034551 | |
| TRINITY_DN19039_c0_g2_i1_len_468_39452.p1_type_internal_len_156 | ---NA--- | | |
| TRINITY_DN20388_c0_g1_i11_len_1646_31104.p1_type_complete_len_155 | ---NA--- | | |
| TRINITY_DN20338_c0_g1_i1_len_858_197950.p1_type_complete_len_155 | ---NA--- | | |
| TRINITY_DN21344_c0_g1_i4_len_1309_158710.p2_type_complete_len_155 | ---NA--- | | |
| TRINITY_DN36484_c0_g1_i1_len_566_241456.p1_type_5prime_partial_len_155 | ---NA--- | | |
| TRINITY_DN5143_c0_g1_i1_len_468_205296.p1_type_internal_len_156 | KMQ83515.1integrase core domain protein | P:GO:0006807; P:GO:0043170; P:GO:0044238 | |
| TRINITY_DN10397_c0_g1_i1_len_466_39508.p1_type_internal_len_156 | XP_018027683.1 alpha-tocopherol transfer protein-like isoform X4 | | |
| TRINITY_DN18009_c0_g1_i1_len_465_39519.p1_type_internal_len_156 | ---NA--- | | |
| TRINITY_DN18755_c1_g1_i3_len_465_39511.p1_type_internal_len_156 | ---NA--- | | |

| | | | |
|---|---|---|---|
| TRINITY_DN32085_c0_g1_i1_len_468_205291.p1_type_internal_len_156 | AAD45201.1prophenoloxidase | F:GO:0016491; F:GO:0046872; P:GO:0055114 | |
| TRINITY_DN13634_c0_g1_i1_len_468_205289.p1_type_internal_len_156 | ---NA--- | | |
| TRINITY_DN15540_c0_g1_i1_len_467_39476.p1_type_internal_len_156 | ---NA--- | | |
| TRINITY_DN15315_c0_g1_i1_len_573_202350.p1_type_5prime_partial_len_155 | ---NA--- | | |
| TRINITY_DN40793_c0_g1_i1_len_469_205237.p1_type_internal_len_156 | EEB16736.1Negative elongation factor A, putative | F:GO:0003746; P:GO:0006414; C:GO:0032021; P:GO:0034244 | |
| TRINITY_DN14264_c0_g1_i5_len_983_270315.p1_type_5prime_partial_len_155 | ---NA--- | | |
| TRINITY_DN22412_c0_g1_i1_len_467_102175.p3_type_internal_len_156 | ---NA--- | | |
| TRINITY_DN33168_c0_g1_i1_len_976_160866.p2_type_5prime_partial_len_155 | XP_023223227.1inositol polyphosphate 1-phosphatase-like | P:GO:0046854 | |
| TRINITY_DN26168_c0_g1_i1_len_468_102157.p1_type_internal_len_156 | AFQ62792.1inositol-requiring enzyme-1 | F:GO:0004540; F:GO:0004672; F:GO:0005524; P:GO:0006397; P:GO:0006468; C:GO:0016021; P:GO:0090501 | |
| TRINITY_DN26106_c0_g1_i1_len_2198_190251.p2_type_3prime_partial_len_155 | XP_018008676.1 serine/arginine-rich splicing factor 3-like | | |
| TRINITY_DN8025_c0_g1_i1_len_464_244096.p1_type_internal_len_155 | XP_026760199.1WD repeat and FYVE domain-containing protein 3-like | | |
| TRINITY_DN13039_c0_g1_i1_len_463_276961.p1_type_internal_len_155 | ---NA--- | | |
| TRINITY_DN7923_c0_g1_i1_len_465_244082.p2_type_internal_len_155 | ---NA--- | | |
| TRINITY_DN31383_c0_g1_i1_len_943_299139.p2_type_complete_len_154 | ---NA--- | | |
| TRINITY_DN9189_c0_g1_i1_len_463_39565.p1_type_internal_len_155 | ---NA--- | | |

| | | | |
|---|---|---|---|
| TRINITY_DN31384_c0_g1_i1_len_534_242158.p1_type_5prime_partial_len_154 | KZC14936.1putative oxidoreductase dhs-27 | | |
| TRINITY_DN14484_c0_g1_i1_len_464_205430.p1_type_internal_len_155 | XP_015518334.1 transformation/transcription domain-associated protein | | |
| TRINITY_DN15936_c0_g1_i1_len_1058_196044.p1_type_5prime_partial_len_154 | ---NA--- | | |
| TRINITY_DN3532_c0_g1_i1_len_464_205431.p1_type_internal_len_155 | ---NA--- | | |
| TRINITY_DN22687_c0_g1_i1_len_2532_229718.p1_type_complete_len_154 | ---NA--- | | |
| TRINITY_DN12186_c0_g1_i1_len_742_65165.p1_type_complete_len_154 | ---NA--- | | |
| TRINITY_DN37238_c0_g1_i1_len_465_244080.p1_type_internal_len_155 | XP_018024804.1PREDICTED: uncharacterized protein LOC108680490 | | |
| TRINITY_DN18043_c0_g1_i1_len_570_130876.p1_type_5prime_partial_len_154 | ---NA--- | | |
| TRINITY_DN7015_c0_g1_i1_len_462_244182.p1_type_internal_len_155 | PSN36733.1Syntaxin-binding protein 5 | C:GO:0016021 | |
| TRINITY_DN30670_c0_g1_i1_len_462_244165.p1_type_internal_len_155 | ---NA--- | | |
| TRINITY_DN19491_c0_g1_i1_len_475_205044.p1_type_5prime_partial_len_154 | XP_021936008.1trimethyllysine dioxygenase, mitochondrial isoform X2 | F:GO:0016491 | |
| TRINITY_DN19587_c0_g1_i6_len_464_205426.p1_type_internal_len_155 | ---NA--- | | |
| TRINITY_DN4008_c0_g1_i1_len_462_244183.p1_type_internal_len_155 | ---NA--- | | |
| TRINITY_DN30897_c0_g1_i1_len_6000_186933.p4_type_3prime_partial_len_155 | ---NA--- | | |
| TRINITY_DN865_c0_g1_i1_len_463_70158.p1_type_internal_len_155 | KFM66581.1Myotubularin-related protein 2 | F:GO:0004725; P:GO:0035335 | EC:3.1.3.16; EC:3.1.3.48 |
| TRINITY_DN11542_c0_g1_i1_len_463_244132.p1_type_internal_len_155 | ---NA--- | | |

| | | | |
|---|---|---|---|
| TRINITY_DN1719_c0_g1_i1_len_630_129880.p2_type_5prime_partial_len_154 | ---NA--- | | |
| TRINITY_DN16356_c0_g1_i1_len_655_273346.p1_type_5prime_partial_len_154 | ---NA--- | | |
| TRINITY_DN10445_c0_g1_i2_len_514_242673.p1_type_5prime_partial_len_154 | ---NA--- | | |
| TRINITY_DN11595_c0_g1_i1_len_462_276998.p1_type_internal_len_155 | XP_018566520.1regulator of G-protein signaling 7 | | |
| TRINITY_DN129_c0_g1_i1_len_463_244145.p1_type_internal_len_155 | XP_016840841.1 multidrug resistance-associated protein 1 isoform X8 | | |
| TRINITY_DN7452_c0_g1_i1_len_463_244143.p1_type_internal_len_155 | ---NA--- | | |
| TRINITY_DN3953_c0_g1_i1_len_464_205427.p1_type_internal_len_155 | ---NA--- | | |
| TRINITY_DN25258_c0_g1_i1_len_1593_232571.p2_type_3prime_partial_len_155 | ---NA--- | | |
| TRINITY_DN8000_c0_g1_i1_len_463_205444.p1_type_internal_len_155 | XP_012535920.1 protein sprint-like | P:GO:0007165 | |
| TRINITY_DN19494_c0_g1_i3_len_1179_94594.p2_type_complete_len_154 | AFW99840.1troponin I | C:GO:0005861 | |
| TRINITY_DN18092_c0_g1_i1_len_471_243875.p1_type_5prime_partial_len_154 | ---NA--- | | |
| TRINITY_DN24037_c0_g1_i3_len_1893_59575.p2_type_3prime_partial_len_155 | ---NA--- | | |
| TRINITY_DN19864_c0_g1_i2_len_1137_195400.p2_type_5prime_partial_len_154 | ---NA--- | | |
| TRINITY_DN28374_c0_g1_i2_len_2793_188865.p3_type_complete_len_154 | XP_018018089.1 small G protein signaling modulator 2-like | | |
| TRINITY_DN35443_c0_g1_i1_len_466_244040.p1_type_internal_len_155 | ---NA--- | | |
| TRINITY_DN27260_c0_g1_i3_len_904_197446.p1_type_5prime_partial_len_154 | ---NA--- | | |

| | | | |
|---|---|---|---|
| TRINITY_DN20639_c0_g1_i2_len_462_276993.p1_type_internal_len_155 | ---NA--- | | |
| TRINITY_DN19786_c0_g1_i1_len_637_164427.p1_type_5prime_partial_len_154 | ---NA--- | | |
| 1795|_|c4504_f2p4_2798_isoform_c4504_full_length_coverage_2_non_full_length_coverage_4_isoform_length_2798_21440 | XP_018009604.1 NF-kappa-B inhibitor-interacting Ras-like protein 2 | F:GO:0000166 | |
| 1796|_|c2326_f4p4_2798_isoform_c2326_full_length_coverage_4_non_full_length_coverage_4_isoform_length_2798_20850 | XP_019643630.1 26S proteasome non-ATPase regulatory subunit 5-like | C:GO:0008540; P:GO:0043248 | |
| 1797|_|c9100_f2p4_2798_isoform_c9100_full_length_coverage_2_non_full_length_coverage_4_isoform_length_2798_21835 | XP_018027119.1 transmembrane emp24 domain-containing protein 7-like | C:GO:0016021 | |
| 1798|_|c2466_f3p0_2798_isoform_c2466_full_length_coverage_3_non_full_length_coverage_0_isoform_length_2798_20960 | XP_018009919.1 tetratricopeptide repeat protein 27-like | | |
| 1799|_|c2939_f2p2_2797_isoform_c2939_full_length_coverage_2_non_full_length_coverage_2_isoform_length_2797_16723 | ---NA--- | | |
| 1802|_|c2340_f6p16_2796_isoform_c2340_full_length_coverage_6_non_full_length_coverage_16_isoform_length_2796_16338 | ASR74813.1leucine-rich repeat-containing protein | | |
| 1803|_|c2468_f3p4_2796_isoform_c2468_full_length_coverage_3_non_full_length_coverage_4_isoform_length_2796_16435 | ---NA--- | | |
| 1804|_|c2723_f3p2_2796_isoform_c2723_full_length_coverage_3_non_full_length_coverage_2_isoform_length_2796_12105 | ---NA--- | | |
| 1806|_|c4467_f2p1_2796_isoform_c4467_full_length_coverage_2_non_full_length_coverage_1_isoform_length_2796_21433 | EFX67445.1hypothetical protein DAPPUDRAFT_63917 | P:GO:0035556 | |

| | | | |
|---|---|---|---|
| 1807\|_\|c3221_f2p0_2796_isoform_c3221_full_length_coverage_2_non_full_length_coverage_0_isoform_length_2796_21324 | ---NA--- | | |
| TRINITY_DN31038_c0_g1_i1_len_463_102280.p1_type_internal_len_155 | ---NA--- | | |
| TRINITY_DN5315_c0_g1_i2_len_464_305057.p1_type_internal_len_155 | ---NA--- | | |
| TRINITY_DN16914_c0_g1_i1_len_643_200926.p1_type_3prime_partial_len_155 | XP_015834256.1 transient receptor potential cation channel subfamily A member 1 isoform X3 | P:GO:0006810 | |
| TRINITY_DN8235_c0_g1_i1_len_465_205392.p1_type_internal_len_155 | XP_015791418.1arfGAP with SH3 domain, ANK repeat and PH domain-containing protein isoform X2 | | |
| TRINITY_DN23390_c0_g1_i2_len_1140_235111.p1_type_3prime_partial_len_155 | ---NA--- | | |
| TRINITY_DN10724_c0_g1_i1_len_464_39541.p1_type_internal_len_155 | ---NA--- | | |
| TRINITY_DN27676_c0_g1_i1_len_1120_159840.p2_type_complete_len_154 | XP_008205124.1 liprin-beta-1 isoform X6 | P:GO:0007528 | |
| TRINITY_DN29534_c0_g1_i1_len_2215_190191.p2_type_complete_len_154 | ---NA--- | | |
| TRINITY_DN39285_c0_g1_i1_len_465_205375.p1_type_internal_len_155 | XP_018024759.1 protein unc-79 homolog | | |
| TRINITY_DN5905_c0_g1_i1_len_464_102258.p1_type_internal_len_155 | ---NA--- | | |
| TRINITY_DN16712_c0_g1_i1_len_464_205402.p1_type_internal_len_155 | ---NA--- | | |
| TRINITY_DN10077_c0_g1_i1_len_466_205357.p1_type_internal_len_155 | ---NA--- | | |
| TRINITY_DN41171_c0_g1_i1_len_464_205403.p1_type_internal_len_155 | XP_018025433.1 probable peroxisomal acyl-coenzyme A oxidase 1 | | |
| TRINITY_DN20051_c0_g1_i2_len_1136_269427.p1_type_complete_len_154 | ---NA--- | | |

| | | | |
|---|---|---|---|
| TRINITY_DN6873_c0_g1_i1_len_465_205370.p1_type_internal_len_155 | XP_023727484.1dual specificity protein phosphatase 3 | P:GO:0016311; F:GO:0016791 | |
| TRINITY_DN4292_c0_g1_i1_len_464_205414.p1_type_internal_len_155 | ---NA--- | | |
| TRINITY_DN31677_c0_g1_i1_len_464_205415.p1_type_internal_len_155 | XP_018023308.1 sodium channel protein 60E-like | | |
| TRINITY_DN18476_c0_g1_i2_len_464_102256.p1_type_internal_len_155 | XP_018014271.1 zinc finger SWIM domain-containing protein 7-like | F:GO:0008270 | |
| TRINITY_DN11414_c0_g1_i1_len_464_205419.p1_type_internal_len_155 | XP_018026183.1 protein stoned-B-like | P:GO:0006886; P:GO:0030100; C:GO:0030131 | |
| TRINITY_DN4543_c0_g1_i1_len_612_201533.p1_type_5prime_partial_len_154 | XP_018017684.1 tryptophan 2,3-dioxygenase-like | F:GO:0004833; P:GO:0019441; P:GO:0019442; F:GO:0020037; F:GO:0046872; P:GO:0055114 | EC:1.13.11; EC:1.13.11.11 |
| TRINITY_DN14194_c0_g1_i1_len_464_205408.p1_type_internal_len_155 | ---NA--- | | |
| TRINITY_DN27803_c0_g1_i2_len_719_199717.p1_type_complete_len_154 | ---NA--- | | |
| TRINITY_DN24088_c0_g1_i1_len_1973_231201.p3_type_complete_len_154 | XP_014219157.1four and a half LIM domains protein 2 isoform X8 | | |
| TRINITY_DN13439_c0_g1_i1_len_465_9698.p1_type_internal_len_155 | ---NA--- | | |
| TRINITY_DN38183_c0_g1_i1_len_465_205398.p1_type_internal_len_155 | ---NA--- | | |
| TRINITY_DN566_c0_g1_i1_len_465_205358.p1_type_internal_len_155 | ---NA--- | | |
| TRINITY_DN15420_c0_g1_i1_len_464_205399.p1_type_internal_len_155 | ---NA--- | | |
| TRINITY_DN27958_c0_g1_i1_len_466_9682.p1_type_internal_len_155 | ---NA--- | | |
| TRINITY_DN33124_c0_g1_i1_len_498_69161.p1_type_5prime_partial_len_154 | ---NA--- | | |
| TRINITY_DN23181_c0_g1_i1_len_465_9694.p1_type_internal_len_155 | ---NA--- | | |

| | | | |
|---|---|---|---|
| TRINITY_DN1092_c0_g1_i1_len_531_203396.p1_type_5prime_partial_len_154 | ---NA--- | | |
| TRINITY_DN21458_c0_g1_i1_len_2938_154407.p3_type_complete_len_154 | ---NA--- | | |
| TRINITY_DN10985_c0_g1_i1_len_466_168002.p1_type_internal_len_155 | XP_018019958.1 carbohydrate sulfotransferase 11-like isoform X3 | | |
| TRINITY_DN22789_c0_g1_i2_len_2383_230042.p3_type_complete_len_154 | ---NA--- | | |
| TRINITY_DN16934_c0_g1_i1_len_537_275221.p1_type_3prime_partial_len_155 | ---NA--- | | |
| TRINITY_DN17522_c0_g1_i2_len_661_239768.p1_type_complete_len_154 | ---NA--- | | |
| TRINITY_DN26587_c0_g1_i1_len_1581_192652.p2_type_complete_len_154 | ---NA--- | | |
| TRINITY_DN13434_c0_g1_i2_len_603_201710.p1_type_5prime_partial_len_154 | XP_022198815.1zinc finger protein OZF-like | | |
| TRINITY_DN22686_c0_g1_i2_len_462_205477.p1_type_internal_len_155 | ---NA--- | | |
| TRINITY_DN2088_c0_g1_i1_len_729_238777.p1_type_3prime_partial_len_155 | ---NA--- | | |
| TRINITY_DN16374_c0_g1_i1_len_677_98356.p1_type_complete_len_154 | ---NA--- | | |
| TRINITY_DN14638_c1_g1_i1_len_577_202282.p1_type_5prime_partial_len_154 | XP_021927523.1prostate stem cell antigen | | |
| TRINITY_DN25330_c0_g1_i1_len_1430_193477.p2_type_3prime_partial_len_155 | ---NA--- | | |
| TRINITY_DN14093_c0_g1_i1_len_462_205500.p1_type_internal_len_155 | KFM66797.1RNA-binding protein 42 | F:GO:0003723; F:GO:0005199 | |
| TRINITY_DN8165_c0_g1_i1_len_462_205480.p1_type_internal_len_155 | ---NA--- | | |
| TRINITY_DN561_c0_g1_i1_len_611_273981.p1_type_5prime_partial_len_154 | ALT10383.1juvenile hormone esterase-like protein 1 | F:GO:0016787 | |

| | | | |
|---|---|---|---|
| TRINITY_DN29736_c0_g1_i1_len_463_168078.p3_type_internal_len_155 | XP_018014662.1 teneurin-m-like | | |
| TRINITY_DN29406_c0_g1_i4_len_4818_187190.p2_type_complete_len_154 | ---NA--- | | |
| TRINITY_DN33164_c0_g1_i1_len_463_205454.p1_type_internal_len_155 | AYF59256.1toll 3 | P:GO:0007165; C:GO:0016021; P:GO:0045087 | |
| TRINITY_DN8642_c0_g1_i1_len_622_240429.p1_type_complete_len_154 | XP_022194177.1zinc finger protein 26-like isoform X2 | | |
| TRINITY_DN584_c0_g1_i1_len_463_205471.p1_type_internal_len_155 | ---NA--- | | |
| TRINITY_DN25949_c0_g1_i1_len_466_133218.p1_type_internal_len_155 | ---NA--- | | |
| TRINITY_DN11566_c0_g1_i1_len_463_205473.p1_type_internal_len_155 | XP_018015540.1 TWiK family of potassium channels protein 18-like isoform X3 | | |
| TRINITY_DN22205_c0_g1_i1_len_474_101968.p1_type_5prime_partial_len_154 | ---NA--- | | |
| TRINITY_DN24783_c0_g1_i1_len_525_8311.p1_type_5prime_partial_len_154 | ---NA--- | | |
| TRINITY_DN7588_c0_g1_i1_len_466_205348.p1_type_internal_len_155 | ---NA--- | | |
| TRINITY_DN10226_c0_g1_i1_len_666_239717.p1_type_5prime_partial_len_154 | ---NA--- | | |
| TRINITY_DN15865_c0_g1_i1_len_550_202875.p1_type_5prime_partial_len_154 | ---NA--- | | |
| TRINITY_DN9330_c0_g1_i1_len_462_70190.p1_type_internal_len_154 | XP_023715289.1CUE domain-containing protein 2 | | |
| TRINITY_DN15037_c0_g1_i1_len_490_204571.p1_type_5prime_partial_len_153 | ---NA--- | | |
| TRINITY_DN7433_c0_g1_i1_len_485_204708.p1_type_5prime_partial_len_153 | XP_022195771.1gastrula zinc finger protein XlCGF57.1-like isoform X1 | | |
| TRINITY_DN14176_c0_g1_i1_len_460_9805.p1_type_internal_len_154 | XP_020707579.1zinc finger protein 625-like | | |

| | | | |
|---|---|---|---|
| TRINITY_DN5931_c0_g1_i1_len_461_168122.p2_type_internal_len_154 | ---NA--- | | |
| TRINITY_DN39221_c0_g1_i1_len_461_205529.p1_type_internal_len_154 | ---NA--- | | |
| TRINITY_DN12735_c0_g1_i1_len_550_241772.p1_type_complete_len_153 | ---NA--- | | |
| TRINITY_DN17771_c0_g1_i2_len_607_66981.p1_type_complete_len_153 | ---NA--- | | |
| TRINITY_DN15395_c0_g1_i1_len_1160_234965.p1_type_complete_len_153 | ---NA--- | | |
| TRINITY_DN18958_c0_g1_i1_len_459_205603.p1_type_internal_len_154 | ---NA--- | | |
| TRINITY_DN24694_c0_g1_i1_len_497_276124.p1_type_complete_len_153 | AEB54642.1ribosomal protein L35 | F:GO:0003735; C:GO:0005840; P:GO:0006412 | |
| TRINITY_DN10279_c0_g1_i1_len_658_98576.p1_type_5prime_partial_len_153 | AHL26189.1lipoprotein receptor 1 | F:GO:0005509; C:GO:0016021 | |
| TRINITY_DN14122_c0_g1_i1_len_964_299010.p3_type_complete_len_153 | ---NA--- | | |
| TRINITY_DN28350_c0_g1_i1_len_713_128869.p1_type_5prime_partial_len_153 | XP_018021692.1 GATA zinc finger domain-containing protein 14-like | | |
| TRINITY_DN26455_c0_g1_i1_len_1320_233933.p1_type_complete_len_153 | XP_018015971.1 ubiquitin-conjugating enzyme E2 N | F:GO:0005524; C:GO:0005634; P:GO:0006301; F:GO:0061631; P:GO:0070534 | |
| TRINITY_DN40859_c0_g1_i1_len_461_205514.p1_type_internal_len_154 | ---NA--- | | |
| TRINITY_DN31707_c0_g1_i1_len_462_205502.p1_type_internal_len_154 | XP_023711129.1uncharacterized protein LOC111866191 isoform X2 | | |
| TRINITY_DN21135_c0_g1_i1_len_3003_181.p2_type_3prime_partial_len_154 | AJD07838.1calcineurin subunit A | F:GO:0004721; P:GO:0006470 | EC:3.1.3.16 |
| TRINITY_DN8451_c0_g1_i1_len_462_205510.p1_type_internal_len_154 | ---NA--- | | |

| | | | |
|---|---|---|---|
| TRINITY_DN4529_c0_g1_i1_len_462_205493.p1_type_internal_len_154 | XP_018016962.1 membrane metallo-endopeptidase-like 1 | F:GO:0004222; P:GO:0006508; F:GO:0008237; C:GO:0016020; C:GO:0016021 | |
| TRINITY_DN25999_c0_g1_i1_len_480_204874.p1_type_5prime_partial_len_153 | ---NA--- | | |
| TRINITY_DN28955_c0_g1_i4_len_6084_186922.p2_type_complete_len_153 | ---NA--- | | |
| TRINITY_DN34183_c0_g1_i1_len_461_205524.p1_type_internal_len_154 | XP_010137462.1 exportin-6-like | C:GO:0005623; P:GO:0006611; F:GO:0008565 | |
| TRINITY_DN4628_c0_g1_i1_len_461_168134.p1_type_internal_len_154 | ---NA--- | | |
| TRINITY_DN40801_c0_g1_i1_len_461_205528.p1_type_internal_len_154 | ---NA--- | | |
| TRINITY_DN31432_c0_g1_i1_len_461_168136.p1_type_internal_len_154 | ---NA--- | | |
| TRINITY_DN8720_c0_g1_i2_len_722_97815.p2_type_5prime_partial_len_153 | ---NA--- | | |
| TRINITY_DN39734_c0_g1_i1_len_461_205520.p1_type_internal_len_154 | XP_012221768.1 protein-S-isoprenylcysteine O-methyltransferase | C:GO:0005783; F:GO:0008168; P:GO:0032259; C:GO:0044425 | |
| TRINITY_DN27598_c0_g1_i2_len_2126_190469.p1_type_complete_len_153 | XP_018010254.1 nuclear nucleic acid-binding protein C1D-like | | |
| TRINITY_DN23364_c0_g1_i1_len_461_205523.p2_type_internal_len_154 | ---NA--- | | |
| TRINITY_DN20671_c0_g1_i1_len_480_39165.p1_type_5prime_partial_len_153 | ---NA--- | | |
| TRINITY_DN17372_c0_g1_i1_len_688_200183.p1_type_5prime_partial_len_153 | XP_026692024.1transcription factor protein isoform X1 | F:GO:0000981; C:GO:0005634; P:GO:0006357; P:GO:0030154; F:GO:0043565 | |
| TRINITY_DN8598_c0_g1_i1_len_460_205575.p3_type_internal_len_154 | ---NA--- | | |
| TRINITY_DN14091_c0_g1_i1_len_461_133352.p1_type_internal_len_154 | ---NA--- | | |

| | | | |
|---|---|---|---|
| TRINITY_DN39012_c0_g1_i1_len_460_205562.p1_type_internal_len_154 | XP_018026183.1 protein stoned-B-like | | |
| TRINITY_DN40868_c0_g1_i1_len_460_205576.p1_type_internal_len_154 | XP_018011718.1 probable E3 ubiquitin-protein ligase makorin-1 | F:GO:0046872 | |
| TRINITY_DN34458_c0_g1_i1_len_461_133356.p1_type_internal_len_154 | ---NA--- | | |
| TRINITY_DN14876_c0_g1_i1_len_1329_158599.p1_type_complete_len_153 | ---NA--- | | |
| TRINITY_DN16770_c0_g1_i2_len_851_127609.p1_type_5prime_partial_len_153 | KFM74079.1Wilms tumor protein-like protein | F:GO:0003676 | |
| TRINITY_DN23534_c0_g1_i1_len_460_9810.p1_type_internal_len_154 | ---NA--- | | |
| TRINITY_DN18569_c0_g1_i1_len_583_7260.p1_type_5prime_partial_len_153 | ---NA--- | | |
| TRINITY_DN15584_c0_g1_i1_len_1128_195466.p1_type_complete_len_153 | ---NA--- | | |
| 1809\|_\|c22460_f3p1_2794_isoform_c22460_full_length_coverage_3_non_full_length_coverage_1_isoform_length_2794_18261 | ---NA--- | | |
| 1810\|_\|c5419_f2p0_2794_isoform_c5419_full_length_coverage_2_non_full_length_coverage_0_isoform_length_2794_21522 | ATU31746.1Talin-2-like protein | C:GO:0001726; F:GO:0005200; C:GO:0005856; C:GO:0005925; P:GO:0007016; P:GO:0007155; F:GO:0051015 | |
| 1811\|_\|c10516_f2p28_2794_isoform_c10516_full_length_coverage_2_non_full_length_coverage_28_isoform_length_2794_26080 | XP_018018313.1 long-chain-fatty-acid--CoA ligase ACSBG2-like isoform X4 | | |
| 1813\|_\|c18005_f2p1_2794_isoform_c18005_full_length_coverage_2_non_full_length_coverage_1_isoform_length_2794_13504 | XP_023711715.1AT-rich interactive domain-containing protein 4B | F:GO:0003677; C:GO:0005634 | |
| 1814\|_\|c9181_f2p3_2794_isoform_c9181_full_length_coverage_2_non_full_length_coverage_3_isoform_length_2794_17267 | XP_020619828.1electron transfer flavoprotein-ubiquinone oxidoreductase, mitochondrial-like | | |

| | | | |
|---|---|---|---|
| TRINITY_DN23071_c0_g1_i1_len_2955_58201.p1_type_5prime_partial_len_931 | XP_023704908.1lysine-specific demethylase 7B-like | | |
| 1817\|_\|c2157_f3p2_2793_isoform_c2157_full_length_coverage_3_non_full_length_coverage_2_isoform_length_2793_20744 | ---NA--- | | |
| 1818\|_\|c2534_f5p8_2793_isoform_c2534_full_length_coverage_5_non_full_length_coverage_8_isoform_length_2793_11948 | XP_013383918.1dihydrolipoyllysine-residue acetyltransferase component of pyruvate dehydrogenase complex, mitochondrial-like | F:GO:0004742; C:GO:0005759; P:GO:0006090; C:GO:0045254 | EC:2.3.1.38; EC:2.3.1.12 |
| TRINITY_DN31129_c0_g1_i1_len_3209_188241.p1_type_complete_len_931 | PNF40648.1putative leucine--tRNA ligase, mitochondrial | P:GO:0006399; P:GO:0006412; F:GO:0016874; F:GO:0140101 | |
| 1820\|_\|c2343_f3p4_2792_isoform_c2343_full_length_coverage_3_non_full_length_coverage_4_isoform_length_2792_16340 | KDR12063.1SH3 domain-binding protein 5-like | P:GO:0035556 | |
| TRINITY_DN21604_c0_g1_i1_len_1905_904.p2_type_3prime_partial_len_154 | ---NA--- | | |
| TRINITY_DN17666_c0_g1_i2_len_481_167533.p1_type_5prime_partial_len_153 | ---NA--- | | |
| TRINITY_DN16677_c0_g1_i2_len_617_36718.p1_type_3prime_partial_len_154 | ---NA--- | | |
| TRINITY_DN1267_c0_g1_i1_len_460_205564.p1_type_internal_len_154 | XP_018023018.1 GRIP1-associated protein 1-like | | |
| TRINITY_DN15563_c0_g1_i2_len_460_9809.p1_type_internal_len_154 | ---NA--- | | |
| TRINITY_DN8857_c0_g1_i1_len_460_205565.p1_type_internal_len_154 | ---NA--- | | |
| TRINITY_DN19878_c0_g1_i1_len_683_65887.p3_type_5prime_partial_len_153 | XP_018015635.1 src substrate protein p85-like | | |
| TRINITY_DN16576_c0_g1_i1_len_459_133398.p2_type_internal_len_154 | XP_018008381.1 dysferlin-like | C:GO:0016021 | |
| TRINITY_DN16167_c1_g1_i1_len_461_205533.p1_type_internal_len_154 | XP_022253209.1mitochondrial tRNA-specific 2-thiouridylase 1-like | | |

| | | | |
|---|---|---|---|
| TRINITY_DN31182_c0_g1_i1_len_4266_187441.p1_type_complete_len_153 | ---NA--- | | |
| TRINITY_DN37718_c0_g1_i1_len_463_205442.p1_type_internal_len_154 | XP_006814551.1 tectonic-1 | | |
| TRINITY_DN12250_c0_g1_i1_len_681_273008.p1_type_5prime_partial_len_153 | ---NA--- | | |
| TRINITY_DN4764_c0_g1_i2_len_579_241175.p1_type_5prime_partial_len_153 | XP_018013798.1 LIM domain only protein 3-like | | |
| TRINITY_DN17780_c0_g1_i3_len_2565_229658.p2_type_complete_len_153 | XP_018019839.1PREDICTED: uncharacterized protein LOC108676289 | | |
| TRINITY_DN36934_c0_g1_i1_len_461_205538.p1_type_internal_len_154 | ---NA--- | | |
| TRINITY_DN19195_c0_g1_i1_len_2132_265996.p3_type_complete_len_153 | XP_014261664.1hydroxysteroid dehydrogenase-like protein 2 | | |
| TRINITY_DN10747_c0_g1_i1_len_1085_3156.p2_type_5prime_partial_len_153 | XP_008829823.1 NAD-dependent protein deacylase sirtuin-5, mitochondrial isoform X2 | | |
| TRINITY_DN18010_c0_g2_i1_len_1198_61956.p2_type_5prime_partial_len_153 | ACY43156.1transmembrane protein | C:GO:0016021 | |
| TRINITY_DN38103_c0_g1_i1_len_459_244281.p1_type_internal_len_154 | XP_018028132.1 protein ECT2-like | P:GO:0065007 | |
| TRINITY_DN3082_c0_g1_i1_len_1453_193343.p1_type_complete_len_153 | ---NA--- | | |
| TRINITY_DN34832_c0_g1_i1_len_460_244251.p1_type_internal_len_154 | ---NA--- | | |
| TRINITY_DN15701_c0_g1_i2_len_460_244254.p1_type_internal_len_154 | ---NA--- | | |
| TRINITY_DN28009_c0_g1_i1_len_467_244004.p1_type_5prime_partial_len_153 | ---NA--- | | |
| TRINITY_DN18115_c0_g1_i1_len_460_277052.p1_type_internal_len_154 | ---NA--- | | |
| TRINITY_DN19137_c0_g1_i1_len_463_39552.p1_type_internal_len_154 | ---NA--- | | |

| | | | |
|---|---|---|---|
| TRINITY_DN9447_c0_g1_i1_len_459_102377.p1_type_internal_len_154 | ---NA--- | | |
| TRINITY_DN21236_c0_g1_i1_len_600_99425.p1_type_3prime_partial_len_154 | KOF62742.1hypothetical protein OCBIM_22021999mg | | |
| TRINITY_DN5220_c0_g1_i1_len_459_277064.p1_type_internal_len_154 | XP_018023688.1 probable ubiquitin carboxyl-terminal hydrolase FAF-X | | |
| TRINITY_DN22008_c0_g1_i1_len_2067_230907.p2_type_3prime_partial_len_154 | XP_013082040.1 rabenosyn-5-like | F:GO:0003676; F:GO:0046872 | |
| TRINITY_DN29204_c0_g1_i1_len_461_244189.p1_type_internal_len_154 | ---NA--- | | |
| TRINITY_DN23573_c0_g1_i2_len_2456_189561.p2_type_complete_len_153 | ---NA--- | | |
| TRINITY_DN8504_c0_g1_i1_len_461_277009.p2_type_internal_len_154 | ATU31747.1Calcium-activated chloride channel regulator 2 | C:GO:0016020; C:GO:0016021 | |
| TRINITY_DN7595_c0_g1_i1_len_461_244196.p1_type_internal_len_154 | XP_018020023.1 spectrin beta chain, non-erythrocytic 1-like | F:GO:0005543 | |
| TRINITY_DN3460_c0_g1_i1_len_461_244206.p1_type_internal_len_154 | ---NA--- | | |
| TRINITY_DN28832_c0_g1_i1_len_462_244152.p1_type_internal_len_154 | XP_018007455.1 longitudinals lacking protein, isoforms H/M/V-like | | |
| TRINITY_DN1022_c0_g1_i1_len_463_244138.p1_type_internal_len_154 | ---NA--- | | |
| TRINITY_DN6648_c0_g1_i1_len_462_244163.p1_type_internal_len_154 | PCG71067.1hypothetical protein B5V51_2281 | C:GO:0016020 | |
| TRINITY_DN4324_c0_g1_i1_len_462_244167.p1_type_internal_len_154 | ---NA--- | | |
| TRINITY_DN19307_c0_g1_i1_len_1119_94901.p2_type_5prime_partial_len_153 | ---NA--- | | |
| TRINITY_DN17623_c0_g1_i2_len_784_271916.p2_type_5prime_partial_len_153 | XP_018006527.1 glutaminase kidney isoform, mitochondrial-like isoform X3 | | |
| TRINITY_DN9300_c0_g1_i1_len_462_244177.p1_type_internal_len_154 | EFX75378.1hypothetical protein DAPPUDRAFT_323288 | | |

| | | | |
|---|---|---|---|
| TRINITY_DN11148_c0_g1_i1_len_460_244226.p1_type_internal_len_154 | XP_018021123.1 protein furry-like | | |
| TRINITY_DN22462_c0_g1_i1_len_3148_149.p2_type_5prime_partial_len_153 | ---NA--- | | |
| TRINITY_DN35618_c0_g1_i1_len_460_244241.p1_type_internal_len_154 | XP_014300690.1 transcription-associated protein 1 | | |
| TRINITY_DN6868_c0_g1_i1_len_752_162913.p3_type_5prime_partial_len_153 | XP_023722160.1serum response factor homolog isoform X2 | | |
| TRINITY_DN28480_c0_g1_i1_len_460_244248.p1_type_internal_len_154 | ---NA--- | | |
| TRINITY_DN32599_c0_g1_i1_len_461_244207.p1_type_internal_len_154 | XP_018022358.1 zinc finger homeobox protein 3-like isoform X1 | | |
| TRINITY_DN29418_c0_g1_i1_len_460_244214.p1_type_internal_len_154 | XP_018022458.1 kinesin-like protein KIF17 | F:GO:0000166 | |
| TRINITY_DN23786_c0_g1_i1_len_493_304319.p1_type_5prime_partial_len_153 | ---NA--- | | |
| TRINITY_DN11682_c0_g1_i1_len_462_276995.p1_type_internal_len_154 | ---NA--- | | |
| TRINITY_DN16095_c0_g1_i1_len_460_9816.p1_type_internal_len_154 | AXR98495.1amphiphysin isoform X4 | C:GO:0005737 | |
| TRINITY_DN22987_c0_g1_i1_len_543_203088.p1_type_5prime_partial_len_153 | ---NA--- | | |
| TRINITY_DN18498_c0_g1_i2_len_521_242487.p1_type_5prime_partial_len_153 | XP_023939539.1RNA-binding protein Musashi homolog Rbp6 isoform X2 | | |
| TRINITY_DN7486_c0_g1_i1_len_615_201476.p1_type_3prime_partial_len_154 | ---NA--- | | |
| TRINITY_DN7088_c0_g1_i1_len_466_304974.p1_type_5prime_partial_len_153 | ---NA--- | | |
| TRINITY_DN30939_c0_g1_i1_len_500_69108.p1_type_5prime_partial_len_153 | XP_018025696.1PREDICTED: uncharacterized protein LOC108681201 | | |
| TRINITY_DN30169_c0_g1_i1_len_508_132192.p1_type_5prime_partial_len_152 | ---NA--- | | |

| | | | |
|---|---|---|---|
| TRINITY_DN17499_c0_g1_i1_len_643_301786.p1_type_5prime_partial_len_152 | ---NA--- | | |
| TRINITY_DN17397_c0_g1_i1_len_949_236535.p1_type_5prime_partial_len_152 | ABE77153.1intracellular fatty acid binding protein | F:GO:0008289 | |
| TRINITY_DN35111_c0_g1_i1_len_459_205585.p1_type_internal_len_153 | ---NA--- | | |
| TRINITY_DN12126_c0_g1_i1_len_734_97693.p1_type_complete_len_152 | OWF38304.1Fucose mutarotase | P:GO:0005996; F:GO:0016853; F:GO:0048029 | |
| TRINITY_DN6195_c0_g1_i1_len_4072_228144.p3_type_complete_len_152 | ---NA--- | | |
| TRINITY_DN2650_c0_g1_i1_len_619_6698.p1_type_5prime_partial_len_152 | ---NA--- | | |
| TRINITY_DN1151_c0_g1_i1_len_459_277080.p1_type_internal_len_153 | ---NA--- | | |
| TRINITY_DN26105_c0_g1_i1_len_459_70283.p1_type_internal_len_153 | ---NA--- | | |
| TRINITY_DN24161_c0_g1_i2_len_835_198206.p1_type_5prime_partial_len_152 | ---NA--- | | |
| TRINITY_DN8514_c0_g1_i1_len_577_37308.p1_type_complete_len_152 | XP_023324719.1ADP-ribosylation factor-like protein 2-binding protein | | |
| TRINITY_DN28857_c0_g1_i2_len_2332_189862.p2_type_complete_len_152 | ---NA--- | | |
| TRINITY_DN27046_c1_g1_i2_len_1003_196529.p1_type_complete_len_152 | ---NA--- | | |
| TRINITY_DN18972_c0_g1_i1_len_907_197412.p1_type_complete_len_152 | XP_018009918.1 MICOS complex subunit Mic10-like | | |
| TRINITY_DN32553_c0_g1_i1_len_610_240633.p1_type_3prime_partial_len_153 | XP_018027296.1 SNF-related serine/threonine-protein kinase-like | | |
| TRINITY_DN22944_c0_g1_i1_len_537_203235.p1_type_5prime_partial_len_152 | XP_019636516.1 histone-lysine N-methyltransferase, H3 lysine-79 specific-like | | |

| | | | |
|---|---|---|---|
| TRINITY_DN35008_c0_g1_i1_len_458_205633.p1_type_internal_len_153 | XP_018021907.1 V-type proton ATPase 16 kDa proteolipid subunit | C:GO:0005774; F:GO:0015078; P:GO:0015991; C:GO:0016021; C:GO:0033179 | |
| TRINITY_DN11613_c0_g1_i1_len_1152_235020.p1_type_complete_len_152 | ---NA--- | | |
| TRINITY_DN22528_c0_g1_i9_len_1171_125553.p2_type_3prime_partial_len_153 | ---NA--- | | |
| TRINITY_DN2473_c0_g1_i1_len_459_205589.p1_type_internal_len_153 | XP_024220068.1glutamate receptor ionotropic, NMDA 2B isoform X1 | | |
| TRINITY_DN16883_c0_g1_i2_len_562_202607.p1_type_5prime_partial_len_152 | XP_019877533.1PREDICTED: uncharacterized protein LOC109605405, partial | | |
| TRINITY_DN36919_c0_g1_i1_len_459_205607.p1_type_internal_len_153 | ---NA--- | | |
| TRINITY_DN16371_c0_g1_i1_len_851_96640.p1_type_complete_len_152 | ---NA--- | | |
| TRINITY_DN32515_c0_g1_i1_len_459_205596.p1_type_internal_len_153 | ---NA--- | | |
| TRINITY_DN6791_c0_g1_i1_len_459_205593.p1_type_internal_len_153 | ---NA--- | | |
| TRINITY_DN13911_c0_g1_i1_len_785_271909.p2_type_5prime_partial_len_152 | ---NA--- | | |
| TRINITY_DN20166_c0_g1_i1_len_2693_295021.p2_type_complete_len_152 | ---NA--- | | |
| TRINITY_DN21088_c0_g1_i9_len_470_167861.p1_type_5prime_partial_len_152 | ---NA--- | | |
| TRINITY_DN2951_c0_g1_i1_len_457_39699.p1_type_internal_len_153 | ---NA--- | | |
| TRINITY_DN6197_c0_g1_i1_len_459_205617.p1_type_internal_len_153 | ---NA--- | | |
| TRINITY_DN14427_c0_g1_i1_len_541_241990.p1_type_5prime_partial_len_152 | ---NA--- | | |

| | | | |
|---|---|---|---|
| TRINITY_DN16763_c0_g1_i2_len_594_37056.p1_type_complete_len_152 | ---NA--- | | |
| TRINITY_DN23946_c0_g1_i1_len_459_277069.p2_type_internal_len_153 | ANG08894.1toll family protein LongTollB | P:GO:0007165; C:GO:0016021 | |
| TRINITY_DN5324_c0_g1_i1_len_543_68132.p1_type_5prime_partial_len_152 | ---NA--- | | |
| TRINITY_DN37354_c0_g1_i1_len_459_205588.p1_type_internal_len_153 | ---NA--- | | |
| TRINITY_DN6773_c0_g1_i1_len_459_205608.p1_type_internal_len_153 | ---NA--- | | |
| TRINITY_DN30133_c0_g1_i1_len_4636_187255.p2_type_complete_len_152 | ---NA--- | | |
| TRINITY_DN20834_c0_g1_i1_len_1832_296009.p3_type_5prime_partial_len_152 | ---NA--- | | |
| TRINITY_DN30822_c0_g1_i1_len_457_70351.p1_type_internal_len_153 | AJC52502.1estrogen sulfotransferase | F:GO:0008146; F:GO:0016740 | |
| TRINITY_DN33311_c0_g1_i1_len_458_70304.p1_type_internal_len_153 | ---NA--- | | |
| TRINITY_DN12119_c0_g1_i1_len_457_244328.p1_type_internal_len_153 | ---NA--- | | |
| TRINITY_DN36428_c0_g1_i1_len_457_244326.p1_type_internal_len_153 | XP_018022358.1 zinc finger homeobox protein 3-like isoform X1 | | |
| TRINITY_DN1941_c0_g1_i1_len_458_244301.p1_type_internal_len_153 | ---NA--- | | |
| TRINITY_DN21115_c0_g3_i2_len_801_97030.p1_type_complete_len_152 | XP_022207428.1GDNF-inducible zinc finger protein 1-like | | |
| 1821\|_\|c3237_f2p9_2791_isoform_c3237_full_length_coverage_2_non_full_length_coverage_9_isoform_length_2791_21325 | XP_021922448.1dihydropyrimidinase isoform X6 | | |
| 1822\|_\|c1829_f7p3_2791_isoform_c1829_full_length_coverage_7_non_full_length_coverage_3_isoform_length_2791_20559 | XP_022234987.1chromatin assembly factor 1 subunit B-like | | |

| | | | |
|---|---|---|---|
| 1823\|_\|c6110_f2p0_2790_isoform_c6110_full_length_coverage_2_non_full_length_coverage_0_isoform_length_2790_21584 | XP_023712316.1dual specificity protein phosphatase CDC14AB-like | F:GO:0004725; F:GO:0008138; P:GO:0035335 | EC:3.1.3.16; EC:3.1.3.48 |
| 1824\|_\|c1740_f3p12_2790_isoform_c1740_full_length_coverage_3_non_full_length_coverage_12_isoform_length_2790_27940 | AEI83215.1arsenite-resistance protein 2-like protein | F:GO:0003676; P:GO:0006397 | |
| 1825\|_\|c24491_f6p5_2789_isoform_c24491_full_length_coverage_6_non_full_length_coverage_5_isoform_length_2789_13902 | XP_023225961.1dnaJ homolog subfamily A member 2-like | | |
| 1826\|_\|c3344_f3p1_2789_isoform_c3344_full_length_coverage_3_non_full_length_coverage_1_isoform_length_2789_21337 | ---NA--- | | |
| 1827\|_\|c18529_f2p9_2789_isoform_c18529_full_length_coverage_2_non_full_length_coverage_9_isoform_length_2789_22742 | XP_018006803.1 longitudinals lacking protein, isoforms H/M/V-like isoform X39 | F:GO:0003676 | |
| 1829\|_\|c1664_f2p4_2788_isoform_c1664_full_length_coverage_2_non_full_length_coverage_4_isoform_length_2788_27885 | ---NA--- | | |
| TRINITY_DN31007_c0_g1_i1_len_2889_188721.p1_type_5prime_partial_len_929 | XP_022290595.1molybdenum cofactor sulfurase-like | | |
| 1831\|_\|c2440_f3p29_2787_isoform_c2440_full_length_coverage_3_non_full_length_coverage_29_isoform_length_2787_16413 | XP_018023075.1 dolichyl-diphosphooligosaccharide--protein glycosyltransferase subunit 2-like isoform X2 | | |
| TRINITY_DN31523_c0_g1_i1_len_456_205717.p1_type_internal_len_153 | XP_018022351.1 fibroblast growth factor receptor 2-like | | |
| TRINITY_DN780_c0_g1_i1_len_515_68745.p1_type_5prime_partial_len_152 | XP_020616061.1cytidine deaminase-like | F:GO:0004126; F:GO:0008270; P:GO:0009972 | EC:3.5.4.5 |
| TRINITY_DN26370_c0_g1_i1_len_1777_191749.p2_type_complete_len_152 | ACP19558.1prophenoloxidase-activating enzyme | F:GO:0004252; P:GO:0006508 | EC:3.4.21 |
| TRINITY_DN4269_c0_g1_i1_len_457_205666.p2_type_internal_len_153 | ---NA--- | | |

| | | | |
|---|---|---|---|
| TRINITY_DN30422_c0_g1_i1_len_457_244340.p1_type_internal_len_153 | XP_018023552.1 membrane-bound transcription factor site-1 protease-like | | |
| TRINITY_DN10312_c0_g1_i1_len_459_244279.p1_type_internal_len_153 | XP_024215122.1probable Ras GTPase-activating protein isoform X2 | P:GO:0065007 | |
| TRINITY_DN7999_c0_g1_i1_len_459_244277.p1_type_internal_len_153 | KYM99513.1Engulfment and cell motility protein 1 | P:GO:0006909; P:GO:0016477; P:GO:0030036 | |
| TRINITY_DN35422_c0_g1_i1_len_538_203208.p1_type_3prime_partial_len_153 | ---NA--- | | |
| TRINITY_DN33955_c0_g1_i1_len_458_244289.p1_type_internal_len_153 | ---NA--- | | |
| TRINITY_DN13220_c0_g1_i2_len_459_305172.p1_type_internal_len_153 | ---NA--- | | |
| TRINITY_DN21358_c0_g2_i1_len_457_168223.p1_type_internal_len_153 | XP_018012656.1PREDICTED: uncharacterized protein LOC108669763 | | |
| TRINITY_DN492_c0_g1_i1_len_457_205691.p1_type_internal_len_153 | EEC00328.1cysteine and histidine-rich protein 1B, putative | F:GO:0046872 | |
| TRINITY_DN22464_c0_g1_i7_len_1838_1001.p2_type_5prime_partial_len_152 | ---NA--- | | |
| TRINITY_DN30589_c0_g1_i1_len_457_277131.p1_type_internal_len_153 | ---NA--- | | |
| TRINITY_DN33522_c0_g1_i1_len_457_205695.p1_type_internal_len_153 | ---NA--- | | |
| TRINITY_DN36023_c0_g1_i1_len_457_205680.p1_type_internal_len_153 | ---NA--- | | |
| TRINITY_DN29125_c0_g1_i14_len_679_200325.p1_type_3prime_partial_len_153 | ---NA--- | | |
| TRINITY_DN32902_c0_g1_i1_len_457_205676.p1_type_internal_len_153 | XP_007445520.1tubulin beta chain | | |
| TRINITY_DN4796_c0_g1_i1_len_457_205682.p1_type_internal_len_153 | KRT84413.1Fibronectin domain-containing protein | | |
| TRINITY_DN16976_c0_g1_i2_len_1278_194404.p2_type_5prime_partial_len_152 | ---NA--- | | |

| | | | |
|---|---|---|---|
| TRINITY_DN9677_c0_g1_i1_len_502_304115.p1_type_5prime_partial_len_152 | XP_026496652.1complex III assembly factor LYRM7 | | |
| TRINITY_DN19981_c0_g1_i1_len_854_96621.p2_type_5prime_partial_len_152 | ---NA--- | | |
| TRINITY_DN15696_c0_g1_i1_len_456_9948.p1_type_internal_len_153 | ---NA--- | | |
| TRINITY_DN42003_c0_g1_i1_len_456_205712.p1_type_internal_len_153 | ---NA--- | | |
| TRINITY_DN20990_c0_g1_i1_len_1140_235103.p1_type_5prime_partial_len_152 | ---NA--- | | |
| TRINITY_DN19865_c0_g1_i1_len_457_205670.p1_type_internal_len_153 | ---NA--- | | |
| TRINITY_DN3674_c0_g1_i1_len_456_133493.p1_type_internal_len_153 | ---NA--- | | |
| TRINITY_DN16784_c0_g1_i1_len_668_98463.p1_type_5prime_partial_len_152 | ---NA--- | | |
| TRINITY_DN14952_c0_g1_i1_len_644_6369.p1_type_5prime_partial_len_152 | ---NA--- | | |
| TRINITY_DN25556_c0_g1_i1_len_460_102365.p1_type_internal_len_153 | ---NA--- | | |
| TRINITY_DN1229_c0_g1_i1_len_456_277151.p1_type_internal_len_153 | XP_018019721.1 G-protein coupled receptor Mth2-like | F:GO:0004888; F:GO:0004930; P:GO:0007166; P:GO:0007186; C:GO:0016020; C:GO:0016021 | |
| TRINITY_DN3516_c0_g1_i1_len_460_244224.p1_type_internal_len_153 | KPI99710.1DmX-like protein 2 | | |
| TRINITY_DN24524_c0_g1_i1_len_459_133417.p1_type_internal_len_153 | ---NA--- | | |
| TRINITY_DN14693_c0_g1_i1_len_458_133432.p1_type_internal_len_153 | ---NA--- | | |
| TRINITY_DN22440_c0_g1_i1_len_458_277107.p1_type_internal_len_153 | XP_021347442.1uncharacterized protein LOC110446564 | | |
| TRINITY_DN25994_c0_g1_i1_len_458_133431.p1_type_internal_len_153 | XP_013779316.1probable ATP-dependent RNA helicase YTHDC2 isoform X2 | F:GO:0003723; F:GO:0004386; F:GO:0005524 | EC:3.6.1.15 |

| | | | |
|---|---|---|---|
| TRINITY_DN35056_c0_g1_i1_len_458_205637.p1_type_internal_len_153 | ---NA--- | | |
| TRINITY_DN6191_c0_g1_i1_len_458_133430.p1_type_internal_len_153 | XP_015488173.1 canalicular multispecific organic anion transporter 1 | | |
| TRINITY_DN3413_c0_g1_i2_len_458_205645.p1_type_internal_len_153 | ---NA--- | | |
| TRINITY_DN8757_c0_g1_i1_len_459_133418.p1_type_3prime_partial_len_153 | ---NA--- | | |
| TRINITY_DN16510_c0_g1_i1_len_765_199064.p1_type_complete_len_152 | ---NA--- | | |
| TRINITY_DN3187_c0_g1_i1_len_458_205643.p1_type_internal_len_153 | XP_015904215.1inositol-trisphosphate 3-kinase homolog isoform X4 | | |
| TRINITY_DN4696_c0_g1_i1_len_598_201824.p1_type_5prime_partial_len_152 | XP_025089954.1voltage-gated hydrogen channel 1-like | P:GO:0006810; C:GO:0016020 | |
| TRINITY_DN4788_c0_g1_i1_len_456_205734.p1_type_internal_len_153 | XP_018010735.1 multiple epidermal growth factor-like domains protein 8 | F:GO:0005509; C:GO:0016020; C:GO:0016021 | |
| TRINITY_DN37763_c0_g1_i1_len_460_244222.p1_type_3prime_partial_len_153 | ---NA--- | | |
| TRINITY_DN6471_c0_g1_i1_len_458_102412.p1_type_3prime_partial_len_153 | XP_018027696.1PREDICTED: uncharacterized protein LOC108682943 isoform X1 | | |
| TRINITY_DN33685_c0_g1_i1_len_459_168170.p1_type_internal_len_153 | ---NA--- | | |
| TRINITY_DN12396_c0_g1_i1_len_458_305209.p1_type_internal_len_153 | AEU11364.1Broad-complex protein isoform 6 variant 1 | F:GO:0003676 | |
| TRINITY_DN3012_c0_g1_i1_len_460_244253.p1_type_internal_len_153 | ---NA--- | | |
| TRINITY_DN19265_c0_g1_i4_len_801_64579.p1_type_3prime_partial_len_153 | ---NA--- | | |
| TRINITY_DN6291_c0_g1_i1_len_458_205647.p1_type_internal_len_153 | XP_023283191.1fanconi-associated nuclease 1-like | | |
| TRINITY_DN1616_c0_g1_i1_len_587_274337.p1_type_5prime_partial_len_151 | ---NA--- | | |

| | | | |
|---|---|---|---|
| TRINITY_DN32581_c0_g1_i1_len_455_205769.p2_type_internal_len_152 | ---NA--- | | |
| TRINITY_DN28616_c1_g1_i1_len_1422_193524.p1_type_complete_len_151 | ---NA--- | | |
| TRINITY_DN21592_c0_g1_i1_len_559_241590.p1_type_5prime_partial_len_151 | ---NA--- | | |
| TRINITY_DN18163_c0_g1_i1_len_2220_265843.p2_type_5prime_partial_len_151 | ---NA--- | | |
| TRINITY_DN18380_c0_g1_i1_len_599_99434.p1_type_5prime_partial_len_151 | XP_017767858.1 COX assembly mitochondrial protein homolog | C:GO:0005739 | |
| TRINITY_DN19412_c0_g1_i1_len_454_205792.p1_type_internal_len_152 | ---NA--- | | |
| TRINITY_DN39690_c0_g1_i1_len_564_202574.p1_type_3prime_partial_len_152 | XP_018014415.1 galactosylceramide sulfotransferase-like | | |
| TRINITY_DN6336_c0_g1_i1_len_1551_232768.p1_type_5prime_partial_len_151 | XP_019927449.1 transcription elongation factor B polypeptide 1 | P:GO:0006511 | |
| TRINITY_DN21061_c0_g1_i2_len_2280_155426.p2_type_complete_len_151 | ---NA--- | | |
| TRINITY_DN14622_c0_g1_i1_len_955_270505.p2_type_complete_len_151 | ATY51984.1cAMP-responsive element binding protein-3 | P:GO:0006355 | |
| TRINITY_DN41538_c0_g1_i1_len_454_205804.p1_type_internal_len_152 | ---NA--- | | |
| TRINITY_DN21122_c0_g1_i1_len_2176_586.p2_type_complete_len_151 | ---NA--- | | |
| TRINITY_DN31798_c0_g1_i1_len_455_205774.p2_type_internal_len_152 | XP_018007711.1PREDICTED: uncharacterized protein LOC108665467 | | |
| TRINITY_DN12813_c0_g1_i1_len_457_102435.p1_type_internal_len_152 | ---NA--- | | |
| TRINITY_DN12231_c0_g1_i1_len_455_70403.p1_type_internal_len_152 | XP_023214802.1autophagy-related protein 16-like | | |
| TRINITY_DN22919_c2_g1_i1_len_2411_229968.p2_type_complete_len_151 | ---NA--- | | |

| | | | |
|---|---|---|---|
| TRINITY_DN8180_c0_g1_i1_len_456_168256.p1_type_internal_len_152 | ---NA--- | | |
| TRINITY_DN2767_c0_g1_i1_len_456_168254.p1_type_internal_len_152 | ABE02605.1plexin-like protein | F:GO:0017154; P:GO:0071526 | |
| TRINITY_DN20618_c0_g1_i1_len_1004_236083.p1_type_complete_len_151 | XP_022194177.1zinc finger protein 26-like isoform X2 | | |
| TRINITY_DN3271_c0_g1_i1_len_499_101319.p1_type_5prime_partial_len_151 | PWA18025.1hypothetical protein CCH79_00004166 | C:GO:0016020; C:GO:0016021 | |
| TRINITY_DN4091_c0_g1_i1_len_453_70469.p1_type_internal_len_152 | ---NA--- | | |
| TRINITY_DN20133_c0_g1_i3_len_845_96679.p1_type_3prime_partial_len_152 | ---NA--- | | |
| TRINITY_DN18665_c0_g1_i2_len_808_34721.p1_type_complete_len_151 | ---NA--- | | |
| TRINITY_DN3033_c0_g1_i1_len_454_205802.p1_type_internal_len_152 | ---NA--- | | |
| TRINITY_DN12141_c0_g1_i1_len_453_70453.p1_type_internal_len_152 | ---NA--- | | |
| TRINITY_DN5682_c0_g1_i1_len_454_70442.p1_type_internal_len_152 | ---NA--- | | |
| TRINITY_DN6015_c0_g1_i1_len_454_168334.p1_type_internal_len_152 | XP_018019275.1 NAD-dependent protein deacetylase sirtuin-1-like | F:GO:0070403 | |
| TRINITY_DN5159_c0_g1_i1_len_862_127533.p2_type_5prime_partial_len_151 | ---NA--- | | |
| TRINITY_DN26008_c0_g1_i1_len_454_168319.p1_type_internal_len_152 | ---NA--- | | |
| TRINITY_DN28431_c0_g1_i1_len_453_205848.p1_type_internal_len_152 | ---NA--- | | |
| TRINITY_DN18753_c0_g1_i3_len_505_204060.p1_type_5prime_partial_len_151 | ---NA--- | | |
| TRINITY_DN28828_c0_g1_i1_len_5224_187079.p3_type_complete_len_151 | XP_018006539.1 flocculation protein FLO11-like | | |

| | | | |
|---|---|---|---|
| TRINITY_DN28135_c0_g1_i1_len_952_95904.p2_type_5prime_partial_len_151 | XP_026572618.1deoxyribonuclease-2-alpha isoform X2 | | |
| TRINITY_DN10596_c0_g1_i1_len_455_168278.p1_type_internal_len_152 | ---NA--- | | |
| TRINITY_DN32522_c0_g1_i1_len_456_102482.p1_type_internal_len_152 | XP_018011706.1 serine/threonine-protein kinase fused-like | F:GO:0000166; F:GO:0004672 | |
| TRINITY_DN6352_c0_g1_i1_len_455_168302.p5_type_internal_len_152 | ---NA--- | | |
| TRINITY_DN8490_c1_g1_i1_len_455_168299.p1_type_internal_len_152 | ---NA--- | | |
| TRINITY_DN31129_c0_g1_i1_len_3209_188241.p2_type_5prime_partial_len_151 | XP_011671821.1 probable leucine--tRNA ligase, mitochondrial | | |
| TRINITY_DN1136_c0_g1_i1_len_455_168293.p1_type_internal_len_152 | ---NA--- | | |
| TRINITY_DN10055_c0_g1_i1_len_456_205714.p1_type_internal_len_152 | XP_018011028.1 general transcription factor 3C polypeptide 1-like | | |
| TRINITY_DN28869_c0_g1_i1_len_586_241068.p1_type_5prime_partial_len_151 | ---NA--- | | |
| TRINITY_DN10357_c0_g1_i1_len_454_244430.p1_type_internal_len_152 | ---NA--- | | |
| TRINITY_DN16761_c0_g1_i1_len_458_205638.p1_type_5prime_partial_len_151 | ---NA--- | | |
| TRINITY_DN13518_c0_g1_i1_len_454_305276.p1_type_internal_len_152 | ---NA--- | | |
| TRINITY_DN16760_c0_g1_i1_len_454_244419.p1_type_internal_len_152 | XP_023213934.1probable phosphorylase b kinase regulatory subunit beta | | |
| TRINITY_DN31416_c0_g1_i1_len_454_244416.p1_type_3prime_partial_len_152 | XP_018016324.1 mitogen-activated protein kinase kinase kinase 1-like | | |
| TRINITY_DN30924_c0_g1_i4_len_2575_189275.p2_type_complete_len_151 | ---NA--- | | |
| TRINITY_DN32213_c0_g1_i1_len_454_305288.p1_type_internal_len_152 | AIJ02081.1amylase | F:GO:0004556; P:GO:0005975; F:GO:0043169; F:GO:0103025 | EC:3.2.1.1 |

| | | | |
|---|---|---|---|
| 1832\|_\|c2065_f3p4_2787_isoform_c2065_full_length_coverage_3_non_full_length_coverage_4_isoform_length_2787_16161 | AHK05660.1ATP-binding cassette transporter sub-family F member 1 | F:GO:0005524; F:GO:0016887 | EC:3.6.1.3; EC:3.6.1.15 |
| TRINITY_DN28126_c0_g1_i3_len_2964_188601.p1_type_5prime_partial_len_929 | XP_018022139.1 dedicator of cytokinesis protein 3-like | | |
| TRINITY_DN24366_c0_g1_i2_len_3089_58112.p1_type_complete_len_929 | XP_023706712.1E3 ubiquitin-protein ligase TRIM37-like | F:GO:0008270; P:GO:0016567 | |
| TRINITY_DN24890_c0_g1_i3_len_3520_228453.p1_type_complete_len_929 | PSN46108.1Protein unc-45 B | | |
| 1837\|_\|c15463_f18p9_2786_isoform_c15463_full_length_coverage_18_non_full_length_coverage_9_isoform_length_2786_13241 | XP_023716139.1BUB3-interacting and GLEBS motif-containing protein ZNF207 isoform X2 | F:GO:0003677 | |
| TRINITY_DN22709_c0_g1_i1_len_3174_228800.p1_type_5prime_partial_len_928 | PNF30331.1Ubiquitin conjugation factor E4 A | C:GO:0000151; F:GO:0004842; P:GO:0006511; P:GO:0016567; F:GO:0034450 | |
| TRINITY_DN30593_c0_g1_i1_len_4064_187552.p1_type_5prime_partial_len_928 | XP_022252105.1cytosolic 10-formyltetrahydrofolate dehydrogenase-like | | |
| 1840\|_\|c1704_f4p1_2783_isoform_c1704_full_length_coverage_4_non_full_length_coverage_1_isoform_length_2783_11446 | XP_006812396.1 aurora kinase A isoform X1 | F:GO:0004674; F:GO:0005524; P:GO:0006468 | EC:2.7.11 |
| 1841\|_\|c1332_f4p33_2783_isoform_c1332_full_length_coverage_4_non_full_length_coverage_33_isoform_length_2783_25685 | ARS88259.1ATP binding cassette transporter 3 | F:GO:0005524; C:GO:0016021; F:GO:0016887 | EC:3.6.1.3; EC:3.6.1.15 |
| TRINITY_DN30544_c0_g1_i1_len_3570_187870.p1_type_complete_len_927 | ---NA--- | | |
| TRINITY_DN7553_c0_g1_i1_len_482_304592.p1_type_5prime_partial_len_151 | ---NA--- | | |
| TRINITY_DN21436_c0_g1_i5_len_455_205739.p1_type_internal_len_152 | ---NA--- | | |
| TRINITY_DN2026_c0_g1_i1_len_455_205742.p1_type_internal_len_152 | AAQ92356.1serine proteinase | F:GO:0004252; P:GO:0006508 | EC:3.4.21 |

| | | | |
|---|---|---|---|
| TRINITY_DN10727_c0_g1_i1_len_553_165885.p1_type_3prime_partial_len_152 | ---NA--- | | |
| TRINITY_DN6272_c0_g1_i1_len_482_304576.p1_type_5prime_partial_len_151 | ---NA--- | | |
| TRINITY_DN10585_c0_g1_i1_len_743_163001.p2_type_5prime_partial_len_151 | ---NA--- | | |
| TRINITY_DN6623_c0_g1_i1_len_453_244448.p1_type_internal_len_152 | KYB28305.1Otoferlin-like Protein | F:GO:0005509; C:GO:0016021; P:GO:0016079; C:GO:0098793 | |
| TRINITY_DN4528_c0_g1_i1_len_456_205705.p1_type_internal_len_152 | ---NA--- | | |
| TRINITY_DN31736_c0_g1_i1_len_456_205709.p1_type_internal_len_152 | XP_018013606.1 WD repeat-containing protein 19-like | C:GO:0005929; P:GO:0035721 | |
| TRINITY_DN13778_c0_g1_i2_len_492_132564.p1_type_5prime_partial_len_151 | ---NA--- | | |
| TRINITY_DN11765_c0_g1_i1_len_457_205663.p1_type_internal_len_152 | ---NA--- | | |
| TRINITY_DN19762_c0_g1_i1_len_945_197042.p1_type_5prime_partial_len_151 | XP_018019429.1 B-cell CLL/lymphoma 7 protein family member A-like | | |
| TRINITY_DN13248_c0_g1_i1_len_457_205664.p1_type_internal_len_152 | ---NA--- | | |
| TRINITY_DN32768_c0_g1_i1_len_455_244393.p3_type_internal_len_152 | ---NA--- | | |
| TRINITY_DN25686_c0_g1_i1_len_454_39773.p1_type_internal_len_152 | ---NA--- | | |
| TRINITY_DN16197_c0_g1_i3_len_456_277157.p1_type_internal_len_152 | ---NA--- | | |
| TRINITY_DN3675_c0_g1_i1_len_456_205718.p2_type_internal_len_152 | ATA66961.1glass bottom boat | C:GO:0005576; F:GO:0008083; P:GO:0010469; C:GO:0016021 | |
| TRINITY_DN29632_c0_g1_i1_len_455_244385.p1_type_internal_len_152 | XP_019870215.1 serine/threonine-protein kinase greatwall | | |
| TRINITY_DN35135_c0_g1_i1_len_457_244322.p1_type_internal_len_152 | ---NA--- | | |

| | | | |
|---|---|---|---|
| TRINITY_DN25711_c0_g1_i1_len_457_277112.p1_type_internal_len_152 | ALT10384.1juvenile hormone esterase-like protein 2 | F:GO:0016787 | |
| TRINITY_DN19958_c0_g1_i2_len_3467_264694.p3_type_5prime_partial_len_151 | ---NA--- | | |
| TRINITY_DN4653_c0_g1_i1_len_454_244413.p2_type_internal_len_152 | ACT64631.1delta protein | F:GO:0005509; P:GO:0007219; P:GO:0007275; C:GO:0016021 | |
| TRINITY_DN21200_c0_g1_i1_len_732_65294.p1_type_5prime_partial_len_151 | ---NA--- | | |
| TRINITY_DN11862_c0_g1_i3_len_455_244402.p1_type_internal_len_152 | KYN20737.1Cysteine-rich protein 1 | P:GO:0007165; F:GO:0046872 | |
| TRINITY_DN20016_c0_g1_i1_len_454_10006.p1_type_internal_len_152 | ---NA--- | | |
| TRINITY_DN6521_c0_g1_i1_len_454_244411.p1_type_internal_len_152 | ---NA--- | | |
| TRINITY_DN16306_c0_g1_i1_len_454_244407.p1_type_internal_len_152 | XP_008546557.1PREDICTED: uncharacterized protein LOC103570555 | | |
| TRINITY_DN11495_c0_g1_i1_len_516_242626.p1_type_complete_len_151 | ---NA--- | | |
| TRINITY_DN31645_c0_g1_i1_len_497_276104.p1_type_5prime_partial_len_151 | ---NA--- | | |
| TRINITY_DN38121_c0_g1_i1_len_455_205753.p2_type_internal_len_152 | XP_018008386.1 myoferlin-like | C:GO:0016020; C:GO:0016021 | |
| TRINITY_DN30301_c0_g1_i1_len_4351_187386.p3_type_5prime_partial_len_151 | XP_021935704.1ankyrin repeat domain-containing protein 27-like | | |
| TRINITY_DN29202_c0_g1_i1_len_2627_189178.p2_type_3prime_partial_len_152 | XP_018024972.1 AP-3 complex subunit mu-1-like | | |
| TRINITY_DN19698_c0_g1_i1_len_1051_298534.p2_type_5prime_partial_len_151 | ---NA--- | | |
| TRINITY_DN11603_c0_g1_i1_len_572_274599.p1_type_5prime_partial_len_151 | ---NA--- | | |
| TRINITY_DN8375_c0_g1_i1_len_526_38180.p1_type_3prime_partial_len_152 | ---NA--- | | |

| | | | |
|---|---|---|---|
| TRINITY_DN34834_c0_g1_i1_len_457_70339.p1_type_internal_len_152 | ---NA--- | | |
| TRINITY_DN7276_c0_g1_i1_len_482_9275.p1_type_5prime_partial_len_151 | ---NA--- | | |
| TRINITY_DN9048_c0_g1_i2_len_500_167056.p1_type_5prime_partial_len_151 | ---NA--- | | |
| TRINITY_DN14135_c0_g1_i1_len_453_277236.p1_type_internal_len_152 | ---NA--- | | |
| TRINITY_DN22923_c0_g1_i2_len_1658_123813.p1_type_5prime_partial_len_151 | XP_018015626.1PREDICTED: uncharacterized protein LOC108672472 | | |
| TRINITY_DN29337_c0_g1_i1_len_2246_190111.p1_type_5prime_partial_len_151 | ---NA--- | | |
| TRINITY_DN14561_c0_g1_i2_len_469_243942.p1_type_5prime_partial_len_151 | AEO16982.1anti-lipopolysaccharide factor | C:GO:0005576; P:GO:0010466; F:GO:0030414 | |
| TRINITY_DN33255_c0_g1_i1_len_457_205686.p1_type_internal_len_152 | XP_018006476.1PREDICTED: uncharacterized protein LOC108664411 | | |
| TRINITY_DN30658_c0_g1_i1_len_454_277230.p2_type_internal_len_152 | XP_024082889.1esterase FE4-like isoform X2 | F:GO:0016787 | |
| TRINITY_DN10264_c0_g1_i1_len_456_277144.p1_type_internal_len_152 | ---NA--- | | |
| TRINITY_DN34118_c0_g1_i1_len_455_205752.p1_type_internal_len_152 | ---NA--- | | |
| TRINITY_DN4817_c0_g1_i2_len_455_205767.p2_type_internal_len_152 | XP_015790513.1sodium-coupled monocarboxylate transporter 1 | C:GO:0016020; C:GO:0016021; F:GO:0022857; P:GO:0055085 | |
| TRINITY_DN4750_c0_g1_i1_len_632_273687.p1_type_3prime_partial_len_152 | ---NA--- | | |
| TRINITY_DN8887_c0_g1_i1_len_646_6342.p1_type_complete_len_151 | ---NA--- | | |
| TRINITY_DN4828_c0_g1_i1_len_457_39709.p1_type_internal_len_152 | ---NA--- | | |
| TRINITY_DN28162_c0_g1_i1_len_2019_190837.p2_type_complete_len_151 | ---NA--- | | |

| | | | |
|---|---|---|---|
| TRINITY_DN22205_c0_g1_i1_len_701_129020.p1_type_5prime_partial_len_151 | ---NA--- | | |
| TRINITY_DN2554_c0_g1_i2_len_609_6841.p1_type_5prime_partial_len_151 | ---NA--- | | |
| TRINITY_DN29108_c0_g1_i1_len_2193_190265.p1_type_5prime_partial_len_151 | EDS33334.1cornichon protein | C:GO:0016021; P:GO:0016192 | |
| TRINITY_DN13272_c0_g1_i1_len_455_205751.p1_type_internal_len_152 | ---NA--- | | |
| TRINITY_DN19706_c0_g1_i1_len_645_164298.p2_type_5prime_partial_len_151 | ---NA--- | | |
| TRINITY_DN20012_c0_g1_i1_len_2191_230535.p2_type_5prime_partial_len_151 | ---NA--- | | |
| TRINITY_DN26090_c0_g1_i1_len_1248_194606.p2_type_5prime_partial_len_151 | ---NA--- | | |
| TRINITY_DN4756_c0_g1_i1_len_485_304513.p1_type_5prime_partial_len_151 | ---NA--- | | |
| TRINITY_DN29429_c0_g1_i1_len_453_244472.p1_type_internal_len_152 | ---NA--- | | |
| TRINITY_DN7013_c0_g1_i1_len_673_239599.p1_type_5prime_partial_len_151 | ---NA--- | | |
| TRINITY_DN37090_c0_g1_i1_len_456_205702.p1_type_internal_len_152 | XP_021349063.1deoxynucleoside triphosphate triphosphohydrolase SAMHD1-like | | |
| TRINITY_DN4701_c0_g1_i2_len_456_39718.p1_type_internal_len_152 | AOR07239.1potassium voltage-gated channel protein EAG | F:GO:0005249; C:GO:0016021; P:GO:0071805 | |
| TRINITY_DN32156_c0_g1_i1_len_456_205701.p1_type_internal_len_152 | XP_023718620.1post-GPI attachment to proteins factor 6-like | C:GO:0016020 | |
| TRINITY_DN27332_c0_g1_i2_len_902_197463.p1_type_5prime_partial_len_151 | XP_022337856.1adenine phosphoribosyltransferase-like | F:GO:0016740 | |
| TRINITY_DN27253_c0_g1_i1_len_535_242129.p1_type_5prime_partial_len_151 | ---NA--- | | |
| TRINITY_DN56_c0_g1_i1_len_453_205863.p1_type_internal_len_152 | XP_018015556.1 trypsin-1-like | F:GO:0004252; P:GO:0006508 | |

| | | | |
|---|---|---|---|
| TRINITY_DN16634_c0_g1_i1_len_1659_267214.p1_type_complete_len_150 | PSN42819.1Mitochondrial fission 1 protein | P:GO:0000266; C:GO:0005741; C:GO:0016021 | |
| TRINITY_DN8580_c0_g1_i1_len_452_70504.p2_type_internal_len_151 | ---NA--- | | |
| TRINITY_DN27388_c0_g1_i2_len_1944_191115.p1_type_complete_len_150 | XP_018016278.1 FUN14 domain-containing protein 1-like isoform X2 | | |
| TRINITY_DN2527_c0_g1_i1_len_904_299394.p1_type_5prime_partial_len_150 | XP_019870284.1PREDICTED: uncharacterized protein C20orf24 homolog | | |
| TRINITY_DN7389_c0_g1_i1_len_451_277308.p1_type_internal_len_151 | XP_007902864.1 E3 ubiquitin-protein ligase RFWD3 | | |
| TRINITY_DN13098_c0_g1_i1_len_1341_124834.p1_type_complete_len_150 | XP_018021739.1 endothelial differentiation-related factor 1-like | F:GO:0043565 | |
| TRINITY_DN12685_c0_g1_i1_len_452_70506.p1_type_internal_len_151 | BAU19444.1serine racemase | P:GO:0006520; F:GO:0030170 | |
| TRINITY_DN17194_c0_g1_i1_len_591_201970.p1_type_5prime_partial_len_150 | ---NA--- | | |
| TRINITY_DN659_c0_g1_i2_len_518_203690.p1_type_5prime_partial_len_150 | ---NA--- | | |
| TRINITY_DN6957_c0_g1_i1_len_451_70521.p1_type_internal_len_151 | XP_018021910.1 probable zinc transporter protein DDB_G0269332 | | |
| TRINITY_DN21079_c0_g1_i1_len_451_133609.p1_type_internal_len_151 | ---NA--- | | |
| TRINITY_DN29769_c0_g1_i1_len_452_277272.p1_type_internal_len_151 | CDW60978.1pol polyprotein | F:GO:0003676; F:GO:0008270; P:GO:0015074 | |
| TRINITY_DN28642_c0_g2_i1_len_585_202106.p1_type_5prime_partial_len_150 | XP_018010987.1 pro-resilin-like | | |
| TRINITY_DN2116_c0_g1_i1_len_514_275727.p1_type_5prime_partial_len_150 | XP_018018731.1 heme-binding protein 2-like | | |
| TRINITY_DN3893_c0_g1_i2_len_638_273586.p1_type_complete_len_150 | XP_018008676.1 serine/arginine-rich splicing factor 3-like | | |
| TRINITY_DN23149_c0_g1_i1_len_454_277208.p1_type_internal_len_151 | ---NA--- | | |

| | | | |
|---|---|---|---|
| TRINITY_DN2309_c0_g1_i1_len_515_275691.p1_type_5prime_partial_len_150 | ---NA--- | | |
| TRINITY_DN20433_c0_g1_i1_len_550_68012.p1_type_5prime_partial_len_150 | ---NA--- | | |
| TRINITY_DN23581_c1_g1_i2_len_1000_126549.p1_type_complete_len_150 | ---NA--- | | |
| TRINITY_DN15641_c0_g1_i1_len_690_200149.p1_type_5prime_partial_len_150 | XP_015515448.1 phosphatidate cytidylyltransferase, photoreceptor-specific isoform X2 | | |
| TRINITY_DN17481_c0_g1_i1_len_744_97595.p1_type_5prime_partial_len_150 | XP_018008490.1PREDICTED: uncharacterized protein LOC108666179 | | |
| TRINITY_DN15914_c0_g1_i1_len_698_200027.p1_type_5prime_partial_len_150 | XP_020555803.2oocyte zinc finger protein XlCOF22-like | C:GO:0005634 | |
| TRINITY_DN17779_c0_g1_i1_len_555_7737.p1_type_5prime_partial_len_150 | ---NA--- | | |
| TRINITY_DN24344_c0_g1_i1_len_697_200055.p1_type_complete_len_150 | EGD79843.16-pyruvoyl tetrahydrobiopterin synthase | F:GO:0003874; P:GO:0006729; F:GO:0046872 | EC:4.2.3.12 |
| TRINITY_DN6366_c0_g1_i1_len_453_70477.p1_type_internal_len_151 | ---NA--- | | |
| TRINITY_DN2951_c0_g1_i1_len_1185_234803.p2_type_complete_len_150 | ---NA--- | | |
| TRINITY_DN12808_c0_g1_i1_len_453_205831.p1_type_5prime_partial_len_150 | ---NA--- | | |
| TRINITY_DN28982_c0_g1_i1_len_5553_187002.p3_type_complete_len_150 | ---NA--- | | |
| TRINITY_DN13963_c0_g1_i1_len_815_162272.p1_type_5prime_partial_len_150 | XP_013135497.1 transcription initiation factor IIA subunit 2 | C:GO:0005672; P:GO:0006367 | |
| TRINITY_DN24687_c0_g2_i1_len_451_205934.p1_type_internal_len_151 | ---NA--- | | |
| TRINITY_DN10472_c0_g1_i1_len_540_242002.p1_type_3prime_partial_len_151 | ---NA--- | | |
| TRINITY_DN1841_c0_g1_i1_len_453_205827.p1_type_internal_len_151 | ---NA--- | | |

| | | | |
|---|---|---|---|
| TRINITY_DN8884_c0_g1_i1_len_638_240147.p1_type_3prime_partial_len_151 | ---NA--- | | |
| 1844\|_\|c2436_f3p9_2781_isoform_c2436_full_length_coverage_3_non_full_length_coverage_9_isoform_length_2781_11865 | PNF14125.1hypothetical protein B7P43_G01048 | F:GO:0003676 | |
| TRINITY_DN29502_c0_g1_i1_len_5302_187059.p1_type_complete_len_927 | XP_018017398.1 ras GTPase-activating protein 1-like | F:GO:0005096; P:GO:0043547; P:GO:0046580 | |
| TRINITY_DN23359_c0_g1_i1_len_3712_153800.p1_type_5prime_partial_len_927 | ---NA--- | | |
| TRINITY_DN21802_c0_g1_i1_len_2789_294976.p1_type_5prime_partial_len_927 | XP_026286831.1WD repeat-containing protein 36 | P:GO:0006364; C:GO:0032040 | |
| 1848\|_\|c26558_f2p0_2780_isoform_c26558_full_length_coverage_2_non_full_length_coverage_0_isoform_length_2780_18399 | EKC35405.1hypothetical protein CGI_10021010 | | |
| TRINITY_DN20178_c0_g1_i1_len_2826_294961.p1_type_5prime_partial_len_926 | XP_018013028.1 protein transport protein Sec24A-like | | |
| TRINITY_DN29130_c1_g1_i1_len_4077_187544.p1_type_3prime_partial_len_927 | ---NA--- | | |
| 1853\|_\|c11548_f2p2_2778_isoform_c11548_full_length_coverage_2_non_full_length_coverage_2_isoform_length_2778_12990 | ---NA--- | | |
| 1854\|_\|c15344_f6p3_2777_isoform_c15344_full_length_coverage_6_non_full_length_coverage_3_isoform_length_2777_22324 | XP_018006352.1PREDICTED: uncharacterized protein LOC108664297 | | |
| 1855\|_\|c10773_f2p6_2776_isoform_c10773_full_length_coverage_2_non_full_length_coverage_6_isoform_length_2776_21995 | XP_018007604.1 vacuolar protein sorting-associated protein 4A-like | F:GO:0005524; C:GO:0005634; P:GO:0007033; F:GO:0016887; P:GO:0042476 | EC:3.6.1.3; EC:3.6.1.15 |
| TRINITY_DN16612_c0_g1_i1_len_1573_192691.p3_type_complete_len_150 | ---NA--- | | |
| TRINITY_DN13842_c0_g1_i1_len_782_238165.p1_type_complete_len_150 | ---NA--- | | |
| TRINITY_DN6023_c0_g1_i1_len_453_205851.p1_type_internal_len_151 | XP_018019355.1 myosin-IA-like | F:GO:0003774; F:GO:0003779; F:GO:0005524; C:GO:0016459 | EC:3.6.1.15 |

| | | | |
|---|---|---|---|
| TRINITY_DN12094_c0_g1_i1_len_781_238178.p1_type_complete_len_150 | ---NA--- | | |
| TRINITY_DN4093_c0_g1_i1_len_451_205911.p1_type_internal_len_151 | XP_015909384.1low-density lipoprotein receptor-related protein 3 | | |
| TRINITY_DN39172_c0_g1_i1_len_451_205931.p1_type_internal_len_151 | ---NA--- | | |
| TRINITY_DN14722_c0_g1_i1_len_770_238293.p1_type_5prime_partial_len_150 | XP_018027633.1 carbohydrate sulfotransferase 1-like | | |
| TRINITY_DN39622_c0_g1_i1_len_453_205842.p1_type_internal_len_151 | ---NA--- | | |
| TRINITY_DN28460_c0_g1_i2_len_1294_194288.p2_type_5prime_partial_len_150 | ---NA--- | | |
| TRINITY_DN18068_c0_g1_i1_len_451_205922.p1_type_internal_len_151 | ---NA--- | | |
| TRINITY_DN10010_c0_g1_i1_len_508_38547.p1_type_5prime_partial_len_150 | XP_018023849.1PREDICTED: uncharacterized protein LOC108679670 | | |
| TRINITY_DN9787_c0_g1_i1_len_451_205926.p1_type_internal_len_151 | XP_006821411.1 E3 ubiquitin-protein ligase HERC2-like | F:GO:0003824 | |
| TRINITY_DN13079_c0_g1_i1_len_450_102671.p1_type_internal_len_151 | XP_023342972.1estradiol 17-beta-dehydrogenase 11-like isoform X1 | | |
| TRINITY_DN24804_c0_g1_i1_len_451_168390.p1_type_internal_len_151 | XP_018025162.1 mitochondrial coenzyme A transporter SLC25A42-like | | |
| TRINITY_DN32338_c0_g1_i1_len_454_205811.p1_type_internal_len_151 | XP_018008465.1 lysine histidine transporter 2-like | | |
| TRINITY_DN19765_c0_g1_i1_len_755_199195.p1_type_complete_len_150 | ---NA--- | | |
| TRINITY_DN4157_c0_g1_i1_len_627_201261.p1_type_5prime_partial_len_150 | KZS12303.1N-acetylgalactosamine kinase | F:GO:0004335; C:GO:0005829; P:GO:0016310; P:GO:0033499; F:GO:0036094 | EC:2.7.1.6 |
| TRINITY_DN9047_c0_g1_i1_len_485_243431.p1_type_5prime_partial_len_150 | AAZ29608.1C-type lectin | F:GO:0030246 | |
| TRINITY_DN6996_c0_g1_i2_len_454_205805.p1_type_3prime_partial_len_151 | XP_020914563.1prolyl 4-hydroxylase subunit alpha-1-like | F:GO:0016491 | |

| | | | |
|---|---|---|---|
| TRINITY_DN34499_c0_g1_i1_len_451_168383.p1_type_internal_len_151 | XP_018019925.1 plexin A3-like isoform X2 | | |
| TRINITY_DN24588_c0_g1_i1_len_452_102610.p1_type_internal_len_151 | XP_018016741.1 dynein heavy chain 5, axonemal-like | F:GO:0003777; F:GO:0005524; P:GO:0007018; C:GO:0030286 | EC:3.6.1.15 |
| TRINITY_DN34980_c0_g1_i1_len_662_239753.p1_type_5prime_partial_len_150 | AAV48573.15-HT1 receptor | F:GO:0004930; P:GO:0007186; C:GO:0016021 | |
| TRINITY_DN17574_c0_g1_i1_len_700_98077.p2_type_5prime_partial_len_150 | ---NA--- | | |
| TRINITY_DN27641_c0_g1_i1_len_452_39826.p1_type_internal_len_151 | ---NA--- | | |
| TRINITY_DN18297_c0_g1_i1_len_1094_235412.p1_type_complete_len_150 | ---NA--- | | |
| TRINITY_DN31985_c0_g1_i1_len_452_205903.p1_type_internal_len_151 | XP_018025267.1 facilitated trehalose transporter Tret1-2 homolog isoform X2 | F:GO:0005215; C:GO:0016020 | |
| TRINITY_DN14464_c0_g1_i1_len_1030_235872.p1_type_complete_len_150 | ---NA--- | | |
| TRINITY_DN26887_c0_g1_i1_len_452_244484.p1_type_internal_len_151 | ---NA--- | | |
| TRINITY_DN39496_c0_g1_i1_len_452_205891.p1_type_internal_len_151 | ---NA--- | | |
| TRINITY_DN34213_c0_g1_i1_len_452_205881.p1_type_internal_len_151 | ---NA--- | | |
| TRINITY_DN12868_c1_g1_i1_len_452_205900.p1_type_internal_len_151 | AUI80370.1cryptochrome 1 | | |
| TRINITY_DN8370_c0_g1_i1_len_454_244415.p1_type_internal_len_151 | ACJ54486.1nitric oxide synthase | F:GO:0004517; F:GO:0005516; P:GO:0006809; F:GO:0010181; F:GO:0020037; F:GO:0046872; F:GO:0050660; F:GO:0050661; P:GO:0055114 | EC:1.14.13.39; EC:1.14.13 |
| TRINITY_DN8828_c0_g1_i1_len_453_244461.p1_type_internal_len_151 | ---NA--- | | |
| TRINITY_DN15243_c0_g1_i1_len_501_167041.p1_type_5prime_partial_len_150 | AAQ22771.1serine proteinase inhibitor | P:GO:0006508; F:GO:0008233 | |

| | | | |
|---|---|---|---|
| TRINITY_DN26399_c0_g2_i2_len_1377_233636.p2_type_complete_len_150 | ---NA--- | | |
| TRINITY_DN1076_c0_g1_i1_len_453_244465.p1_type_internal_len_151 | ---NA--- | | |
| TRINITY_DN27146_c0_g1_i1_len_597_240860.p1_type_5prime_partial_len_150 | XP_014770907.1 dual specificity protein phosphatase 19-like | P:GO:0016311; F:GO:0016791 | |
| TRINITY_DN31117_c0_g1_i8_len_3088_188392.p1_type_complete_len_150 | ---NA--- | | |
| TRINITY_DN1969_c0_g1_i1_len_452_244476.p1_type_internal_len_151 | ---NA--- | | |
| TRINITY_DN22918_c0_g1_i1_len_452_10051.p1_type_internal_len_151 | XP_013860411.1 zinc finger protein OZF-like | F:GO:0003676 | |
| TRINITY_DN20110_c0_g1_i1_len_453_10011.p1_type_internal_len_151 | ---NA--- | | |
| TRINITY_DN10115_c0_g1_i1_len_453_244457.p1_type_internal_len_151 | ---NA--- | | |
| TRINITY_DN31066_c0_g1_i1_len_451_244537.p1_type_internal_len_151 | ---NA--- | | |
| TRINITY_DN29756_c0_g1_i1_len_451_244542.p1_type_internal_len_151 | ---NA--- | | |
| TRINITY_DN14433_c0_g1_i4_len_453_244446.p1_type_internal_len_151 | ---NA--- | | |
| TRINITY_DN31386_c0_g1_i1_len_755_199194.p2_type_5prime_partial_len_150 | ---NA--- | | |
| TRINITY_DN16242_c0_g1_i1_len_451_244545.p1_type_internal_len_151 | XP_012723786.1uncharacterized protein LOC105930231 | | |
| TRINITY_DN15925_c0_g1_i1_len_452_205887.p1_type_internal_len_151 | ---NA--- | | |
| TRINITY_DN2003_c0_g1_i1_len_452_205882.p1_type_internal_len_151 | ---NA--- | | |
| TRINITY_DN30661_c0_g1_i1_len_450_10105.p1_type_internal_len_151 | ---NA--- | | |

| | | | |
|---|---|---|---|
| TRINITY_DN3768_c0_g1_i1_len_452_205905.p1_type_internal_len_151 | ---NA--- | | |
| TRINITY_DN5484_c0_g1_i1_len_452_244485.p1_type_internal_len_151 | ---NA--- | | |
| TRINITY_DN32468_c0_g1_i1_len_452_205886.p1_type_internal_len_151 | XP_014665810.1 aminopeptidase O-like isoform X5 | | |
| TRINITY_DN15064_c0_g1_i1_len_454_9988.p1_type_internal_len_151 | BAB85575.1cyclic AMP-regulated protein like protein | F:GO:0003779; C:GO:0016020 | |
| TRINITY_DN6439_c0_g1_i1_len_450_244580.p1_type_internal_len_151 | ---NA--- | | |
| TRINITY_DN37064_c0_g1_i1_len_450_244569.p1_type_internal_len_151 | XP_018016421.1 four and a half LIM domains protein 2-like isoform X3 | | |
| TRINITY_DN7461_c0_g1_i1_len_449_205987.p1_type_internal_len_150 | ---NA--- | | |
| TRINITY_DN15371_c0_g1_i4_len_447_244651.p1_type_internal_len_150 | XP_018916919.1 disks large homolog 4 isoform X9 | | |
| TRINITY_DN37392_c0_g1_i1_len_448_244630.p1_type_internal_len_150 | ---NA--- | | |
| TRINITY_DN7895_c0_g1_i1_len_448_244633.p1_type_internal_len_150 | XP_015110572.1 histone deacetylase 6 isoform X1 | | |
| TRINITY_DN4538_c0_g1_i1_len_448_244640.p1_type_internal_len_150 | AEB00643.1glucose transporter 1 | P:GO:0008643; C:GO:0016021; F:GO:0022857; P:GO:0055085 | |
| TRINITY_DN11859_c0_g1_i1_len_448_244634.p1_type_internal_len_150 | XP_018021013.1PREDICTED: uncharacterized protein LOC108677311 | | |
| TRINITY_DN2329_c0_g1_i1_len_448_244642.p1_type_internal_len_150 | XP_018019976.1 kynureninase-like | | |
| TRINITY_DN5013_c0_g1_i1_len_449_205989.p1_type_internal_len_150 | XP_026470485.1long-chain-fatty-acid--CoA ligase 1-like | F:GO:0003824 | |
| TRINITY_DN7949_c0_g1_i1_len_450_102669.p1_type_internal_len_150 | XP_018024139.1 UDP-glucuronosyltransferase-like isoform X3 | | |
| TRINITY_DN14203_c0_g1_i1_len_450_102658.p1_type_internal_len_150 | ---NA--- | | |

| | | | |
|---|---|---|---|
| TRINITY_DN18741_c0_g1_i1_len_593_240927.p1_type_complete_len_149 | KPI91664.1UPF0545 protein C22orf39-like | | |
| TRINITY_DN6363_c0_g1_i1_len_613_273960.p1_type_5prime_partial_len_149 | ---NA--- | | |
| TRINITY_DN4268_c0_g1_i1_len_450_244586.p1_type_internal_len_150 | ---NA--- | | |
| TRINITY_DN12886_c0_g1_i2_len_668_273177.p1_type_5prime_partial_len_149 | ---NA--- | | |
| TRINITY_DN35438_c0_g1_i1_len_447_206070.p1_type_internal_len_150 | ---NA--- | | |
| TRINITY_DN15110_c0_g1_i1_len_447_206065.p1_type_internal_len_150 | ---NA--- | | |
| TRINITY_DN33222_c0_g1_i1_len_450_244568.p1_type_internal_len_150 | OXA37293.1Histone deacetylase 6 | F:GO:0008270; F:GO:0016787 | |
| TRINITY_DN3724_c0_g1_i1_len_450_244561.p2_type_internal_len_150 | XP_018335741.1uncharacterized protein LOC108744461 isoform X1 | | |
| TRINITY_DN6264_c0_g1_i1_len_591_240976.p1_type_5prime_partial_len_149 | PNF28954.1Phosphorylase b kinase gamma catalytic chain, skeletal muscle/heart isoform | F:GO:0004689; F:GO:0005516; F:GO:0005524; C:GO:0005964; P:GO:0005978; P:GO:0006468 | EC:2.7.11; EC:2.7.11.19; EC:2.7.11.17 |
| TRINITY_DN4342_c0_g1_i1_len_451_244530.p1_type_internal_len_150 | ---NA--- | | |
| TRINITY_DN29967_c0_g1_i1_len_450_133632.p1_type_internal_len_150 | XP_023335652.1general transcription factor 3C polypeptide 1-like isoform X1 | | |
| TRINITY_DN35722_c0_g1_i1_len_448_244620.p1_type_3prime_partial_len_150 | XP_018013595.1 chloride channel protein 2-like isoform X4 | | |
| TRINITY_DN7768_c0_g1_i1_len_449_244617.p1_type_internal_len_150 | ---NA--- | | |
| TRINITY_DN36695_c0_g1_i1_len_449_244615.p1_type_internal_len_150 | ---NA--- | | |
| TRINITY_DN34649_c0_g1_i1_len_449_244609.p2_type_internal_len_150 | ---NA--- | | |
| TRINITY_DN17365_c0_g1_i2_len_448_102717.p1_type_internal_len_150 | ---NA--- | | |

| | | | |
|---|---|---|---|
| TRINITY_DN16972_c0_g1_i1_len_450_205980.p1_type_internal_len_150 | ---NA--- | | |
| TRINITY_DN31485_c0_g1_i1_len_448_244623.p1_type_internal_len_150 | ---NA--- | | |
| TRINITY_DN13294_c0_g1_i2_len_448_244622.p1_type_internal_len_150 | ---NA--- | | |
| TRINITY_DN16254_c0_g1_i4_len_714_65507.p1_type_complete_len_149 | XP_022255310.1protein CutA homolog isoform X3 | | |
| TRINITY_DN18528_c0_g1_i1_len_449_102703.p1_type_internal_len_150 | XP_021937373.1RUN and FYVE domain-containing protein 2 isoform X3 | F:GO:0046872 | |
| TRINITY_DN18795_c0_g1_i1_len_2638_29671.p3_type_complete_len_149 | ---NA--- | | |
| TRINITY_DN28436_c0_g1_i1_len_558_165773.p1_type_5prime_partial_len_149 | EFX86930.1GST-N-Metaxin-like protein | C:GO:0016020; C:GO:0016021 | |
| TRINITY_DN41250_c0_g1_i1_len_508_203978.p1_type_5prime_partial_len_149 | ---NA--- | | |
| TRINITY_DN19734_c0_g1_i1_len_892_197579.p2_type_complete_len_149 | ---NA--- | | |
| TRINITY_DN25889_c0_g1_i1_len_448_305464.p2_type_internal_len_150 | ---NA--- | | |
| TRINITY_DN407_c0_g1_i1_len_467_167960.p1_type_5prime_partial_len_149 | ---NA--- | | |
| TRINITY_DN4590_c0_g1_i1_len_534_203303.p1_type_3prime_partial_len_150 | XP_018023320.1 H(+)/Cl(-) exchange transporter 5-like isoform X2 | | |
| TRINITY_DN41047_c0_g1_i1_len_448_206015.p1_type_internal_len_150 | ---NA--- | | |
| TRINITY_DN12560_c0_g1_i1_len_468_167921.p1_type_3prime_partial_len_150 | XP_018006430.1 7,8-dihydro-8-oxoguanine triphosphatase-like | F:GO:0016787 | |
| TRINITY_DN24239_c0_g1_i1_len_1060_196025.p1_type_complete_len_149 | ---NA--- | | |
| TRINITY_DN10003_c0_g1_i2_len_1080_62622.p1_type_5prime_partial_len_149 | EEB17447.1Neuronal PAS domain-containing protein, putative | | |

| | | | |
|---|---|---|---|
| TRINITY_DN30348_c0_g1_i1_len_813_198457.p1_type_5prime_partial_len_149 | ---NA--- | | |
| TRINITY_DN31853_c0_g1_i1_len_448_206025.p1_type_internal_len_150 | ---NA--- | | |
| 1857\|_\|c8813_f3p0_2776_isoform_c8813_full_length_coverage_3_non_full_length_coverage_0_isoform_length_2776_17227 | PNF16400.1DNA replication licensing factor MCM4 | F:GO:0003677; F:GO:0003678; F:GO:0005524; C:GO:0005634; P:GO:0006270; P:GO:0032508; C:GO:0042555 | EC:3.6.1.15 |
| 1858\|_\|c1350_f7p3_2775_isoform_c1350_full_length_coverage_7_non_full_length_coverage_3_isoform_length_2775_15837 | XP_018018202.1 glomulin-like | | |
| 1859\|_\|c10575_f2p0_2775_isoform_c10575_full_length_coverage_2_non_full_length_coverage_0_isoform_length_2775_12902 | XP_015187987.1 non-canonical poly(A) RNA polymerase PAPD5-like | F:GO:0016779 | |
| 1860\|_\|c7368_f2p0_2775_isoform_c7368_full_length_coverage_2_non_full_length_coverage_0_isoform_length_2775_17132 | XP_025601873.1ubiquitin thioesterase OTU1 | | |
| TRINITY_DN24382_c0_g1_i2_len_3158_188307.p1_type_5prime_partial_len_925 | ATV91623.1serine/threonine-protein kinase ULK1 | F:GO:0004674; F:GO:0005524; P:GO:0006468 | EC:2.7.11 |
| 1862\|_\|c2770_f2p8_2774_isoform_c2770_full_length_coverage_2_non_full_length_coverage_8_isoform_length_2774_16687 | XP_021934825.1protein CNPPD1 isoform X1 | P:GO:0000079; F:GO:0019901 | |
| 1863\|_\|c3036_f2p2_2774_isoform_c3036_full_length_coverage_2_non_full_length_coverage_2_isoform_length_2774_21309 | ---NA--- | | |
| 1866\|_\|c12849_f2p8_2773_isoform_c12849_full_length_coverage_2_non_full_length_coverage_8_isoform_length_2773_28604 | ---NA--- | | |
| 1867\|_\|c11460_f2p0_2771_isoform_c11460_full_length_coverage_2_non_full_length_coverage_0_isoform_length_2771_22059 | XP_015931215.1gastrula zinc finger protein XlCGF8.2DB-like | | |

| | | | |
|---|---|---|---|
| 1868\|_\|c18035_f2p2_2771_isoform_c18035_full_length_coverage_2_non_full_length_coverage_2_isoform_length_2771_13525 | XP_018020938.1 1-acyl-sn-glycerol-3-phosphate acyltransferase epsilon-like isoform X1 | C:GO:0016020; C:GO:0016021; F:GO:0016740; F:GO:0016746 | |
| TRINITY_DN41212_c0_g1_i1_len_447_206052.p1_type_internal_len_150 | XP_021913228.1DNA polymerase zeta catalytic subunit | F:GO:0000166; F:GO:0003677; F:GO:0003887; C:GO:0016035; P:GO:0019985 | EC:2.7.7.7 |
| TRINITY_DN35115_c0_g1_i1_len_449_205998.p1_type_internal_len_150 | ---NA--- | | |
| TRINITY_DN17179_c0_g1_i1_len_449_205997.p1_type_internal_len_150 | ---NA--- | | |
| TRINITY_DN23177_c0_g1_i4_len_488_69437.p1_type_5prime_partial_len_149 | ---NA--- | | |
| TRINITY_DN21611_c0_g1_i3_len_447_244653.p1_type_internal_len_150 | ---NA--- | | |
| TRINITY_DN32236_c0_g1_i1_len_449_205991.p1_type_internal_len_150 | XP_018024855.1 serine/threonine-protein kinase PKH3-like | | |
| TRINITY_DN33686_c0_g1_i1_len_449_70576.p1_type_internal_len_150 | ---NA--- | | |
| TRINITY_DN11559_c0_g1_i1_len_491_167280.p1_type_5prime_partial_len_149 | ---NA--- | | |
| TRINITY_DN24249_c0_g1_i1_len_2152_230638.p3_type_complete_len_149 | ---NA--- | | |
| TRINITY_DN12465_c0_g1_i1_len_449_305431.p1_type_internal_len_150 | XP_018019714.1 organic cation transporter protein-like | | |
| TRINITY_DN19770_c0_g1_i1_len_449_206003.p1_type_internal_len_150 | ---NA--- | | |
| TRINITY_DN4486_c0_g1_i1_len_560_241580.p1_type_3prime_partial_len_150 | ---NA--- | | |
| TRINITY_DN20233_c0_g1_i1_len_449_305415.p1_type_internal_len_150 | ---NA--- | | |
| TRINITY_DN16785_c0_g1_i1_len_448_70633.p1_type_internal_len_150 | ---NA--- | | |

| | | | |
|---|---|---|---|
| TRINITY_DN19380_c0_g1_i1_len_1428_233358.p2_type_complete_len_149 | ---NA--- | | |
| TRINITY_DN3427_c0_g1_i1_len_447_168509.p1_type_internal_len_150 | XP_018014298.1 equilibrative nucleoside transporter 3-like | F:GO:0005337; C:GO:0016020; C:GO:0016021; P:GO:1901642 | |
| TRINITY_DN22743_c1_g1_i2_len_461_205537.p1_type_5prime_partial_len_149 | ---NA--- | | |
| TRINITY_DN30854_c0_g1_i1_len_448_39932.p1_type_internal_len_150 | KZS03013.1Cytidine deaminase | F:GO:0004126; C:GO:0005829; F:GO:0008270; P:GO:0009972 | EC:3.5.4.5 |
| TRINITY_DN16644_c1_g1_i1_len_687_163710.p1_type_5prime_partial_len_149 | ---NA--- | | |
| TRINITY_DN17933_c0_g1_i8_len_448_277394.p1_type_internal_len_150 | XP_015273707.1 zinc finger protein 397-like | | |
| TRINITY_DN28901_c0_g1_i1_len_448_39939.p1_type_internal_len_150 | XP_023225903.1PEST proteolytic signal-containing nuclear protein-like | | |
| TRINITY_DN16253_c0_g1_i1_len_864_127519.p1_type_5prime_partial_len_149 | ---NA--- | | |
| TRINITY_DN16993_c0_g1_i1_len_451_205936.p1_type_internal_len_150 | ---NA--- | | |
| TRINITY_DN25555_c0_g1_i1_len_448_39959.p1_type_internal_len_150 | XP_018012981.1 alpha-mannosidase 2x-like | | |
| TRINITY_DN13726_c0_g1_i1_len_450_205948.p2_type_internal_len_150 | ---NA--- | | |
| TRINITY_DN29742_c0_g1_i1_len_3308_188126.p3_type_3prime_partial_len_150 | ---NA--- | | |
| TRINITY_DN34865_c0_g1_i1_len_861_237324.p2_type_3prime_partial_len_150 | XP_023709053.1mothers against decapentaplegic homolog 6 | | |
| TRINITY_DN26799_c0_g1_i1_len_1292_194306.p1_type_5prime_partial_len_149 | ---NA--- | | |
| TRINITY_DN2745_c0_g1_i1_len_448_168486.p1_type_internal_len_150 | ---NA--- | | |
| TRINITY_DN27724_c1_g1_i1_len_1487_193176.p1_type_complete_len_149 | ---NA--- | | |

| | | | |
|---|---|---|---|
| TRINITY_DN16638_c0_g1_i3_len_448_168492.p1_type_internal_len_150 | ---NA--- | | |
| TRINITY_DN21232_c0_g1_i1_len_450_39881.p1_type_internal_len_150 | ALC78853.1adenosine monophosphate deaminase 2 | | |
| TRINITY_DN24647_c0_g1_i1_len_1229_194758.p2_type_5prime_partial_len_149 | ---NA--- | | |
| TRINITY_DN39616_c0_g1_i1_len_779_198869.p1_type_3prime_partial_len_150 | ---NA--- | | |
| TRINITY_DN5379_c0_g1_i1_len_611_201544.p1_type_5prime_partial_len_149 | XP_015517664.1 protein unc-79 homolog isoform X5 | | |
| TRINITY_DN9231_c0_g1_i1_len_539_203174.p1_type_5prime_partial_len_149 | ---NA--- | | |
| TRINITY_DN468_c0_g1_i1_len_706_163460.p2_type_complete_len_149 | ---NA--- | | |
| TRINITY_DN7303_c1_g1_i1_len_448_10163.p1_type_internal_len_150 | ---NA--- | | |
| TRINITY_DN23022_c0_g1_i1_len_448_10162.p1_type_internal_len_150 | AUB30147.1fast-type skeletal muscle actin 2 | F:GO:0005524 | |
| TRINITY_DN19095_c0_g1_i1_len_800_237943.p1_type_5prime_partial_len_149 | AUI80370.1cryptochrome 1 | | |
| TRINITY_DN33942_c0_g1_i1_len_450_205968.p1_type_internal_len_150 | XP_023239272.1 E3 ubiquitin-protein ligase MYCBP2-like | F:GO:0016874 | |
| TRINITY_DN17866_c0_g1_i2_len_1894_266555.p1_type_5prime_partial_len_149 | ---NA--- | | |
| TRINITY_DN11073_c0_g1_i1_len_450_205957.p1_type_internal_len_150 | XP_018020177.1 syndetin-like | C:GO:0005829; P:GO:0032456; P:GO:0042147; C:GO:1990745 | |
| TRINITY_DN1103_c0_g1_i1_len_448_133672.p1_type_internal_len_150 | ---NA--- | | |
| TRINITY_DN15847_c0_g2_i1_len_449_168449.p1_type_internal_len_150 | XP_018016657.1 glycerol-3-phosphate acyltransferase 3-like | | |
| TRINITY_DN17164_c0_g1_i1_len_1132_235168.p1_type_5prime_partial_len_149 | ---NA--- | | |

| | | | |
|---|---|---|---|
| TRINITY_DN14482_c0_g1_i1_len_722_238864.p2_type_complete_len_149 | ---NA--- | | |
| TRINITY_DN11952_c0_g1_i1_len_448_133675.p1_type_internal_len_150 | AWU67222.1putative chitinase | F:GO:0004553; P:GO:0008152 | |
| TRINITY_DN32049_c0_g1_i1_len_450_205955.p1_type_internal_len_150 | KZS15673.1Uncharacterized protein APZ42_018896 | F:GO:0005272; P:GO:0006811; P:GO:0006814; C:GO:0016020; C:GO:0016021; P:GO:0035725 | |
| TRINITY_DN9134_c0_g1_i1_len_500_204246.p1_type_5prime_partial_len_149 | XP_008991150.1 3-hydroxybutyrate dehydrogenase type 2 | C:GO:0005737; P:GO:0009987; F:GO:0016491 | |
| TRINITY_DN710_c0_g1_i1_len_634_273648.p1_type_complete_len_149 | ---NA--- | | |
| TRINITY_DN15025_c0_g1_i1_len_543_241950.p1_type_5prime_partial_len_149 | XP_013408869.1copine-8 isoform X2 | | |
| TRINITY_DN8174_c0_g1_i1_len_479_204902.p1_type_5prime_partial_len_149 | ---NA--- | | |
| TRINITY_DN15235_c0_g1_i1_len_450_205965.p1_type_internal_len_150 | XP_018012290.1 protein turtle homolog A-like | | |
| TRINITY_DN3158_c0_g1_i1_len_672_98411.p1_type_5prime_partial_len_149 | ---NA--- | | |
| TRINITY_DN14374_c0_g1_i1_len_536_100533.p1_type_5prime_partial_len_149 | ALJ10568.1rhodanese 1-like protein | | |
| TRINITY_DN15462_c0_g1_i2_len_567_202484.p1_type_5prime_partial_len_149 | XP_023340407.1myotubularin-related protein 4-like | F:GO:0046872 | |
| TRINITY_DN12634_c0_g1_i1_len_625_129970.p1_type_5prime_partial_len_149 | XP_018014250.1 PXMP2/4 family protein 4-like | | |
| TRINITY_DN22415_c0_g1_i1_len_557_202720.p1_type_5prime_partial_len_149 | ---NA--- | | |
| TRINITY_DN17198_c0_g1_i1_len_2994_264962.p2_type_5prime_partial_len_149 | ---NA--- | | |
| TRINITY_DN6990_c0_g1_i1_len_451_168386.p1_type_internal_len_150 | ---NA--- | | |
| TRINITY_DN1914_c0_g1_i1_len_468_39447.p1_type_5prime_partial_len_149 | XP_015510998.1 IDLSRF-like peptide | | |

| | | | |
|---|---|---|---|
| TRINITY_DN26305_c0_g1_i3_len_595_201870.p1_type_5prime_partial_len_149 | ---NA--- | | |
| TRINITY_DN30847_c0_g1_i1_len_448_168467.p1_type_internal_len_150 | XP_018020234.1 E3 ubiquitin-protein ligase MYCBP2-like | | |
| TRINITY_DN7332_c1_g1_i1_len_450_205954.p1_type_internal_len_150 | ACU30154.1hypoxia inducible factor 1 alpha | F:GO:0003677; C:GO:0005634; P:GO:0006355; F:GO:0046983 | |
| TRINITY_DN12748_c0_g1_i1_len_448_133671.p1_type_internal_len_150 | ---NA--- | | |
| TRINITY_DN19081_c0_g1_i1_len_528_8261.p1_type_5prime_partial_len_149 | ---NA--- | | |
| TRINITY_DN24046_c0_g2_i1_len_1808_191632.p1_type_complete_len_149 | ---NA--- | | |
| TRINITY_DN10566_c0_g1_i2_len_482_101735.p1_type_5prime_partial_len_149 | ---NA--- | | |
| TRINITY_DN39454_c0_g1_i1_len_604_201695.p1_type_5prime_partial_len_148 | KDQ71515.1Protein O-mannosyl-transferase 2 | F:GO:0000030; P:GO:0006493; C:GO:0016020; C:GO:0016021; F:GO:0016740; P:GO:0097502 | |
| TRINITY_DN17867_c0_g1_i1_len_1694_232155.p2_type_complete_len_148 | XP_026490173.126S proteasome regulatory subunit 8 | | |
| TRINITY_DN14943_c0_g1_i1_len_713_65515.p1_type_complete_len_148 | XP_022248715.1retinal rod rhodopsin-sensitive cGMP 3',5'-cyclic phosphodiesterase subunit delta isoform X1 | | |
| TRINITY_DN12868_c0_g1_i1_len_447_206063.p1_type_5prime_partial_len_148 | AUI80370.1cryptochrome 1 | | |
| TRINITY_DN30309_c1_g1_i1_len_1774_191761.p4_type_5prime_partial_len_148 | ---NA--- | | |
| TRINITY_DN6624_c0_g1_i1_len_447_277433.p1_type_internal_len_149 | ---NA--- | | |
| TRINITY_DN31410_c0_g1_i1_len_447_206067.p1_type_internal_len_149 | ---NA--- | | |
| TRINITY_DN18071_c0_g1_i1_len_1477_193217.p2_type_5prime_partial_len_148 | ---NA--- | | |

| | | | |
|---|---|---|---|
| TRINITY_DN27681_c0_g1_i1_len_447_168546.p1_type_internal_len_149 | ---NA--- | | |
| TRINITY_DN6853_c0_g1_i1_len_446_168547.p1_type_internal_len_149 | ---NA--- | | |
| TRINITY_DN32560_c0_g1_i1_len_447_168539.p1_type_internal_len_149 | AIT97138.1innexin 7 | C:GO:0016020; C:GO:0030054 | |
| TRINITY_DN22191_c0_g1_i1_len_2180_122819.p2_type_5prime_partial_len_148 | ---NA--- | | |
| TRINITY_DN9180_c0_g1_i1_len_447_133709.p1_type_internal_len_149 | APG53783.1transient receptor potential cation channel subfamily M member 3 | C:GO:0016020; C:GO:0016021 | |
| TRINITY_DN33495_c0_g1_i1_len_889_127324.p3_type_complete_len_148 | ---NA--- | | |
| TRINITY_DN25879_c0_g1_i2_len_2348_189827.p1_type_5prime_partial_len_148 | ---NA--- | | |
| TRINITY_DN20250_c0_g1_i1_len_988_236212.p2_type_complete_len_148 | XP_018026869.1PREDICTED: uncharacterized protein LOC108682249 | C:GO:0016020; C:GO:0016021 | |
| TRINITY_DN14726_c0_g1_i1_len_958_3842.p3_type_complete_len_148 | XP_018017426.1 putative cysteine proteinase CG12163 isoform X2 | | |
| TRINITY_DN34455_c0_g1_i1_len_445_70734.p1_type_internal_len_149 | PNF27755.1Zinc finger with UFM1-specific peptidase domain protein | F:GO:0003676 | |
| TRINITY_DN8070_c0_g1_i1_len_445_70730.p1_type_internal_len_149 | ---NA--- | | |
| TRINITY_DN24688_c0_g1_i3_len_535_203277.p1_type_5prime_partial_len_148 | ---NA--- | | |
| TRINITY_DN16809_c0_g1_i2_len_861_197913.p2_type_5prime_partial_len_148 | ---NA--- | | |
| TRINITY_DN25797_c0_g1_i1_len_445_277505.p1_type_internal_len_149 | ---NA--- | | |
| TRINITY_DN23001_c0_g1_i4_len_2232_230424.p4_type_complete_len_148 | ---NA--- | | |
| TRINITY_DN1931_c0_g1_i1_len_448_168481.p1_type_5prime_partial_len_148 | XP_023238733.1constitutive coactivator of PPAR-gamma-like protein 2 | C:GO:0005634 | |

| | | | |
|---|---|---|---|
| TRINITY_DN27659_c0_g1_i1_len_448_168476.p1_type_internal_len_149 | ---NA--- | | |
| TRINITY_DN4103_c0_g1_i2_len_444_168625.p1_type_internal_len_149 | ---NA--- | | |
| TRINITY_DN28030_c0_g1_i1_len_497_243107.p1_type_5prime_partial_len_148 | ---NA--- | | |
| TRINITY_DN8860_c0_g1_i1_len_446_277491.p1_type_internal_len_149 | XP_008198216.1 trafficking protein particle complex subunit 11 isoform X2 | C:GO:0005737 | |
| TRINITY_DN29747_c0_g1_i1_len_446_70692.p2_type_internal_len_149 | XP_023223078.1zinc finger with UFM1-specific peptidase domain protein-like | F:GO:0003676 | |
| TRINITY_DN31821_c0_g1_i1_len_527_203481.p1_type_complete_len_148 | ---NA--- | | |
| TRINITY_DN22553_c0_g1_i1_len_1082_195829.p1_type_complete_len_148 | ---NA--- | | |
| TRINITY_DN26875_c0_g1_i1_len_3156_188309.p1_type_3prime_partial_len_924 | ---NA--- | | |
| 1870\|_\|c1605_f8p3_2769_isoform_c1605_full_length_coverage_8_non_full_length_coverage_3_isoform_length_2769_15917 | XP_013775579.1ATP-dependent RNA helicase DDX51-like | F:GO:0097159; F:GO:1901363 | |
| 1873\|_\|c1740_f8p4_2767_isoform_c1740_full_length_coverage_8_non_full_length_coverage_4_isoform_length_2767_15966 | XP_022324322.1retinoic acid receptor beta-like isoform X3 | | |
| 1874\|_\|c36165_f3p2_2767_isoform_c36165_full_length_coverage_3_non_full_length_coverage_2_isoform_length_2767_18911 | XP_023344051.1chromodomain-helicase-DNA-binding protein 7-like isoform X4 | | |
| 1875\|_\|c18211_f2p2_2767_isoform_c18211_full_length_coverage_2_non_full_length_coverage_2_isoform_length_2767_13649 | XP_018018153.1 ATP-dependent RNA helicase SUV3 homolog, mitochondrial-like | | |
| 1876\|_\|c2555_f5p2_2766_isoform_c2555_full_length_coverage_5_non_full_length_coverage_2_isoform_length_2766_16507 | XP_018018327.1 probable cytosolic oligopeptidase A isoform X2 | F:GO:0008233 | |

| | | | |
|---|---|---|---|
| TRINITY_DN24342_c1_g1_i1_len_2849_58273.p1_type_5prime_partial_len_922 | XP_014661540.1 nucleolar complex protein 3 homolog | C:GO:0005634; C:GO:0005730; C:GO:0005739; C:GO:0016607; P:GO:0045444 | |
| 1878\|_\|c36240_f2p0_2764_isoform_c36240_full_length_coverage_2_non_full_length_coverage_0_isoform_length_2764_23585 | ---NA--- | | |
| 1879\|_\|c2141_f2p1_2764_isoform_c2141_full_length_coverage_2_non_full_length_coverage_1_isoform_length_2764_28247 | ---NA--- | | |
| 1880\|_\|c3808_f2p7_2763_isoform_c3808_full_length_coverage_2_non_full_length_coverage_7_isoform_length_2763_12326 | ---NA--- | | |
| TRINITY_DN20283_c0_g1_i1_len_1455_1797.p2_type_5prime_partial_len_148 | ---NA--- | | |
| TRINITY_DN29684_c0_g1_i1_len_446_133762.p1_type_internal_len_149 | XP_018022215.1 solute carrier family 28 member 3-like | F:GO:0005415; C:GO:0005887; P:GO:1901642 | |
| TRINITY_DN15891_c0_g1_i3_len_1133_32845.p2_type_5prime_partial_len_148 | XP_019614669.1 cell cycle checkpoint protein RAD1-like | P:GO:0000077; C:GO:0005634 | |
| TRINITY_DN25029_c0_g1_i1_len_448_277406.p1_type_internal_len_149 | ATU31747.1Calcium-activated chloride channel regulator 2 | | |
| TRINITY_DN12126_c0_g1_i1_len_446_277464.p1_type_internal_len_149 | ---NA--- | | |
| TRINITY_DN19260_c0_g1_i1_len_1385_268193.p2_type_5prime_partial_len_148 | ---NA--- | | |
| TRINITY_DN8636_c0_g1_i1_len_727_65357.p1_type_complete_len_148 | XP_018009886.1 15 kDa selenoprotein-like | | |
| TRINITY_DN12028_c0_g1_i1_len_666_200522.p1_type_5prime_partial_len_148 | ---NA--- | | |
| TRINITY_DN23376_c0_g1_i2_len_1760_156815.p2_type_complete_len_148 | ---NA--- | | |
| TRINITY_DN28344_c0_g1_i1_len_447_168513.p1_type_internal_len_149 | AKG54423.1interferon regulatory factor | F:GO:0044212 | |

| | | | |
|---|---|---|---|
| TRINITY_DN5859_c0_g1_i1_len_479_204913.p1_type_5prime_partial_len_148 | ---NA--- | | |
| TRINITY_DN10601_c0_g1_i1_len_527_242342.p1_type_5prime_partial_len_148 | ---NA--- | | |
| TRINITY_DN19809_c0_g1_i3_len_445_133775.p1_type_internal_len_149 | ---NA--- | | |
| TRINITY_DN18783_c0_g1_i1_len_762_97418.p1_type_5prime_partial_len_148 | XP_018027570.1 PAXIP1-associated glutamate-rich protein 1-like | | |
| TRINITY_DN24761_c0_g1_i2_len_659_200640.p1_type_5prime_partial_len_148 | XP_018012426.1PREDICTED: uncharacterized protein LOC108669564 | | |
| TRINITY_DN24388_c0_g3_i4_len_830_64321.p1_type_3prime_partial_len_149 | ---NA--- | | |
| TRINITY_DN17309_c0_g1_i1_len_1179_195104.p2_type_complete_len_148 | ---NA--- | | |
| TRINITY_DN25908_c0_g2_i1_len_3721_228334.p2_type_complete_len_148 | ---NA--- | | |
| TRINITY_DN9622_c0_g1_i1_len_445_168585.p1_type_internal_len_149 | ---NA--- | | |
| TRINITY_DN33691_c0_g1_i1_len_462_244157.p1_type_3prime_partial_len_149 | AJC52502.1estrogen sulfotransferase | F:GO:0008146; F:GO:0016740 | |
| TRINITY_DN11630_c0_g1_i1_len_446_102795.p1_type_internal_len_149 | ---NA--- | | |
| TRINITY_DN12352_c0_g1_i1_len_446_102777.p4_type_internal_len_149 | ---NA--- | | |
| TRINITY_DN2692_c0_g1_i1_len_445_206138.p1_type_internal_len_149 | XP_018020366.1 glutathione synthetase-like | | |
| TRINITY_DN4117_c0_g1_i1_len_445_206134.p1_type_internal_len_149 | ---NA--- | | |
| TRINITY_DN33161_c0_g1_i1_len_445_206150.p1_type_internal_len_149 | XP_022250474.1deleted in autism-related protein 1 homolog isoform X3 | | |
| TRINITY_DN29114_c0_g1_i1_len_445_10260.p1_type_internal_len_149 | AAQ94739.1signal transducer and activator of transcription | F:GO:0003677; F:GO:0003700; C:GO:0005634; C:GO:0005737; P:GO:0006355; P:GO:0060397 | |

| | | | |
|---|---|---|---|
| TRINITY_DN21910_c1_g1_i1_len_974_3752.p2_type_5prime_partial_len_148 | ---NA--- | | |
| TRINITY_DN38155_c0_g1_i1_len_445_206140.p1_type_internal_len_149 | XP_005689120.1 60S ribosomal protein L7 | | |
| TRINITY_DN17124_c0_g1_i1_len_448_305458.p1_type_internal_len_149 | ---NA--- | | |
| TRINITY_DN18172_c0_g1_i1_len_804_128010.p1_type_5prime_partial_len_148 | XP_025424841.1transmembrane protein 14C isoform X2 | | |
| TRINITY_DN14966_c0_g1_i1_len_448_305455.p1_type_internal_len_149 | ---NA--- | | |
| TRINITY_DN13650_c0_g1_i1_len_444_102859.p1_type_internal_len_149 | ---NA--- | | |
| TRINITY_DN18841_c0_g1_i1_len_457_205662.p1_type_5prime_partial_len_148 | ---NA--- | | |
| TRINITY_DN3994_c0_g1_i1_len_446_206103.p1_type_internal_len_149 | XP_018023256.1 motile sperm domain-containing protein 2-like | | |
| TRINITY_DN16763_c0_g1_i1_len_1242_234422.p2_type_3prime_partial_len_149 | XP_018006463.1PREDICTED: LOW QUALITY PROTEIN: uncharacterized protein LOC108664398 | | |
| TRINITY_DN28227_c0_g1_i1_len_444_102850.p1_type_internal_len_149 | ALT10384.1juvenile hormone esterase-like protein 2 | F:GO:0016787 | |
| TRINITY_DN15880_c0_g1_i1_len_678_273051.p2_type_3prime_partial_len_149 | ---NA--- | | |
| TRINITY_DN13594_c0_g1_i1_len_446_206119.p1_type_internal_len_149 | ---NA--- | | |
| TRINITY_DN5165_c0_g1_i1_len_445_206121.p1_type_internal_len_149 | XP_024869093.12-aminoethanethiol dioxygenase | F:GO:0016702; P:GO:0055114 | |
| TRINITY_DN2866_c0_g1_i1_len_445_206123.p1_type_internal_len_149 | ---NA--- | | |
| TRINITY_DN25220_c0_g1_i1_len_493_132537.p1_type_5prime_partial_len_148 | APC26118.1metabotropic glutamate receptor type 7 | F:GO:0004930; C:GO:0005886; P:GO:0007186; C:GO:0016021 | |
| TRINITY_DN39239_c0_g1_i1_len_446_206106.p1_type_internal_len_149 | XP_023324769.1monoacylglycerol lipase ABHD12-like | | |

| | | | |
|---|---|---|---|
| TRINITY_DN13421_c0_g1_i1_len_446_206109.p1_type_internal_len_149 | XP_018007308.1 von Willebrand factor A domain-containing protein 9-like | C:GO:0032039 | |
| TRINITY_DN20158_c0_g1_i1_len_446_206112.p1_type_internal_len_149 | XP_023237574.1rab GTPase-binding effector protein 1-like isoform X4 | | |
| TRINITY_DN33365_c0_g1_i1_len_446_206118.p1_type_internal_len_149 | XP_018014164.1 bestrophin-3-like | | |
| TRINITY_DN32925_c0_g1_i1_len_704_239136.p1_type_complete_len_148 | ---NA--- | | |
| TRINITY_DN6726_c0_g1_i1_len_447_39973.p1_type_internal_len_149 | XP_018016582.1 zinc finger and BTB domain-containing protein 44-like isoform X2 | | |
| TRINITY_DN3078_c0_g1_i1_len_478_9360.p1_type_5prime_partial_len_148 | ---NA--- | | |
| TRINITY_DN13617_c0_g1_i1_len_589_165199.p1_type_complete_len_148 | ---NA--- | | |
| TRINITY_DN37367_c0_g1_i1_len_444_206188.p1_type_internal_len_149 | ---NA--- | | |
| TRINITY_DN30153_c0_g1_i1_len_447_39999.p2_type_internal_len_149 | XP_018008896.1PREDICTED: uncharacterized protein LOC108666520 isoform X1 | | |
| TRINITY_DN78_c0_g1_i1_len_448_102720.p1_type_internal_len_149 | ---NA--- | | |
| TRINITY_DN37158_c0_g1_i1_len_444_206173.p1_type_internal_len_149 | AYD78165.1nuclear respiratory factor-1/DNA-binding protein Ewg isoform | F:GO:0003677; F:GO:0003700; P:GO:0006357 | |
| TRINITY_DN31763_c0_g1_i1_len_448_102715.p1_type_internal_len_149 | ---NA--- | | |
| TRINITY_DN22866_c0_g1_i1_len_449_102692.p1_type_5prime_partial_len_148 | ---NA--- | | |
| TRINITY_DN20658_c0_g1_i1_len_706_199897.p1_type_3prime_partial_len_149 | ---NA--- | | |
| TRINITY_DN13974_c0_g1_i1_len_668_239672.p1_type_5prime_partial_len_148 | ---NA--- | | |

| | | | |
|---|---|---|---|
| TRINITY_DN4741_c0_g1_i1_len_446_244709.p1_type_internal_len_149 | ---NA--- | | |
| TRINITY_DN36537_c0_g1_i1_len_445_244723.p1_type_internal_len_149 | ---NA--- | | |
| TRINITY_DN6203_c0_g1_i1_len_602_240767.p2_type_5prime_partial_len_148 | ---NA--- | | |
| TRINITY_DN10886_c0_g1_i1_len_445_244730.p1_type_internal_len_149 | XP_021932819.1vacuolar protein sorting-associated protein 13D isoform X2 | | |
| TRINITY_DN7818_c0_g1_i1_len_445_244733.p1_type_internal_len_149 | XP_018014830.1 ethanolaminephosphotransferase 1-like | | |
| TRINITY_DN31303_c1_g1_i1_len_446_206089.p1_type_internal_len_149 | ---NA--- | | |
| TRINITY_DN30419_c0_g1_i1_len_445_244744.p1_type_internal_len_149 | XP_023702383.1GTPase-activating protein CdGAPr | | |
| TRINITY_DN7114_c0_g1_i1_len_445_244747.p1_type_internal_len_149 | ---NA--- | | |
| TRINITY_DN34405_c0_g1_i1_len_445_244748.p1_type_internal_len_149 | ---NA--- | | |
| TRINITY_DN34266_c0_g1_i1_len_446_206087.p1_type_internal_len_149 | CRL04901.1CLUMA_CG017952, isoform A | C:GO:0016020; C:GO:0016021 | |
| TRINITY_DN12742_c0_g1_i1_len_446_244692.p1_type_internal_len_149 | XP_018018067.1 sorting nexin-6-like | | |
| TRINITY_DN16732_c0_g1_i3_len_448_10176.p1_type_internal_len_149 | XP_022187165.1gastrula zinc finger protein XlCGF57.1-like | F:GO:0000981; F:GO:0003676; C:GO:0005634; P:GO:0006357; P:GO:0040034 | |
| TRINITY_DN20577_c0_g1_i3_len_529_100659.p1_type_5prime_partial_len_148 | ---NA--- | | |
| TRINITY_DN17168_c0_g1_i1_len_903_197457.p1_type_5prime_partial_len_148 | XP_018008897.1PREDICTED: uncharacterized protein LOC108666520 isoform X2 | | |
| TRINITY_DN11593_c0_g1_i1_len_448_10177.p1_type_internal_len_149 | ---NA--- | | |

| | | | |
|---|---|---|---|
| TRINITY_DN4075_c0_g1_i1_len_445_244751.p1_type_internal_len_149 | XP_018018361.1 cubilin-like | F:GO:0005509; C:GO:0016020; C:GO:0016021 | |
| TRINITY_DN37123_c0_g1_i1_len_444_244771.p1_type_internal_len_149 | XP_023706958.1tigger transposable element-derived protein 1 isoform X4 | F:GO:0003676 | |
| TRINITY_DN23909_c0_g1_i1_len_446_206090.p1_type_internal_len_149 | ---NA--- | | |
| TRINITY_DN40862_c0_g1_i1_len_715_199774.p1_type_3prime_partial_len_149 | XP_018026912.1 solute carrier family 23 member 1-like | C:GO:0016020; C:GO:0016021; F:GO:0022857; P:GO:0055085 | |
| TRINITY_DN34653_c0_g1_i1_len_592_67240.p2_type_5prime_partial_len_148 | XP_006815228.1 solute carrier family 23 member 1-like | F:GO:0005215; C:GO:0016020; C:GO:0016021; F:GO:0022857; P:GO:0055085 | |
| TRINITY_DN13935_c0_g1_i1_len_718_163297.p1_type_5prime_partial_len_148 | ---NA--- | | |
| TRINITY_DN24902_c0_g1_i1_len_515_166743.p1_type_5prime_partial_len_148 | ---NA--- | | |
| TRINITY_DN6110_c0_g1_i1_len_447_244659.p1_type_internal_len_149 | ---NA--- | | |
| TRINITY_DN30190_c0_g1_i1_len_447_244666.p1_type_internal_len_149 | ---NA--- | | |
| TRINITY_DN38395_c0_g1_i1_len_446_206085.p1_type_internal_len_149 | ---NA--- | | |
| TRINITY_DN29828_c0_g1_i1_len_447_244678.p1_type_internal_len_149 | ---NA--- | | |
| TRINITY_DN17038_c0_g1_i1_len_625_302030.p1_type_5prime_partial_len_148 | ---NA--- | | |
| TRINITY_DN23372_c0_g1_i3_len_1550_232773.p3_type_3prime_partial_len_149 | ---NA--- | | |
| TRINITY_DN21611_c0_g1_i3_len_447_244653.p2_type_internal_len_149 | ---NA--- | | |
| TRINITY_DN12987_c0_g1_i1_len_447_244658.p1_type_internal_len_149 | ---NA--- | | |
| TRINITY_DN13111_c0_g1_i1_len_446_206098.p1_type_internal_len_149 | ---NA--- | | |

| | | | |
|---|---|---|---|
| TRINITY_DN4162_c0_g1_i1_len_446_206100.p1_type_3prime_partial_len_149 | ---NA--- | | |
| TRINITY_DN12000_c0_g1_i1_len_615_240547.p1_type_5prime_partial_len_148 | XP_018027317.1PREDICTED: uncharacterized protein LOC108682624 isoform X2 | P:GO:0006810; F:GO:0008028; C:GO:0016020 | |
| TRINITY_DN5958_c0_g1_i1_len_446_305492.p1_type_internal_len_149 | ---NA--- | | |
| TRINITY_DN28379_c0_g1_i1_len_498_101331.p1_type_5prime_partial_len_148 | ---NA--- | | |
| TRINITY_DN15083_c0_g1_i1_len_1363_193877.p2_type_3prime_partial_len_149 | ---NA--- | | |
| TRINITY_DN20553_c0_g1_i1_len_1470_296858.p2_type_complete_len_147 | AST14855.1Bcl2-associated X protein | C:GO:0016021; P:GO:0042981 | |
| TRINITY_DN18863_c1_g1_i1_len_1739_266964.p2_type_complete_len_147 | XP_018016994.1 protoporphyrinogen oxidase-like | | |
| TRINITY_DN35564_c0_g1_i1_len_489_204574.p1_type_3prime_partial_len_148 | KZS04440.1Major facilitator superfamily transporter 17 | C:GO:0016021 | |
| TRINITY_DN36045_c0_g1_i1_len_441_206273.p1_type_internal_len_148 | EEN59997.1hypothetical protein BRAFLDRAFT_71726 | C:GO:0005634; P:GO:0050789 | |
| TRINITY_DN15312_c0_g1_i1_len_477_276600.p1_type_5prime_partial_len_147 | ---NA--- | | |
| TRINITY_DN2695_c0_g1_i1_len_464_70150.p1_type_5prime_partial_len_147 | XP_013043109.1 histone deacetylase 11 isoform X2 | F:GO:0016787 | |
| TRINITY_DN34553_c0_g1_i1_len_797_237993.p1_type_5prime_partial_len_147 | ---NA--- | | |
| TRINITY_DN26292_c0_g1_i1_len_7506_227496.p1_type_5prime_partial_len_1636 | XP_018018981.1PREDICTED: uncharacterized protein LOC108675468 | | |
| TRINITY_DN22677_c0_g1_i1_len_5262_153426.p1_type_complete_len_1633 | ---NA--- | | |
| TRINITY_DN24989_c0_g1_i1_len_5214_57711.p1_type_complete_len_1631 | XP_014681230.1 nucleoporin NUP188 homolog | | |

| | | | |
|---|---|---|---|
| TRINITY_DN31215_c0_g1_i1_len_7781_186777.p1_type_5prime_partial_len_1630 | AAC61691.1inositol 1,4,5-trisphosphate receptor | F:GO:0005220; C:GO:0005783; C:GO:0016021; P:GO:0048016; P:GO:0070588; F:GO:0070679 | |
| TRINITY_DN29007_c0_g1_i1_len_5291_187060.p1_type_5prime_partial_len_1621 | XP_018021439.1 zinc finger protein 729-like | | |
| TRINITY_DN30790_c0_g1_i2_len_6671_186838.p1_type_5prime_partial_len_1620 | XP_020707997.1chromatin-remodeling ATPase INO80 isoform X2 | | |
| 204\|_\|c3853_f2p27_4856_isoform_c3853_full_length_coverage_2_non_full_length_coverage_27_isoform_length_4856_25700 | XP_018017935.1 M-phase inducer phosphatase-like | | |
| TRINITY_DN26211_c0_g1_i1_len_7133_186805.p1_type_complete_len_1618 | XP_018012654.1 glycogen debranching enzyme-like isoform X2 | F:GO:0004133; P:GO:0005977 | |
| TRINITY_DN22170_c0_g1_i2_len_6865_227524.p1_type_complete_len_1611 | XP_018009396.1 disco-interacting protein 2 homolog C-like | | |
| TRINITY_DN30554_c0_g1_i1_len_4940_187145.p1_type_5prime_partial_len_1606 | XP_018011694.1 laminin subunit alpha-1-like | P:GO:0007155 | |
| TRINITY_DN25392_c0_g1_i1_len_3791_187712.p1_type_5prime_partial_len_921 | XP_023233508.1zinc finger protein 850-like | F:GO:0003676 | |
| 1884\|_\|c18224_f2p0_2762_isoform_c18224_full_length_coverage_2_non_full_length_coverage_0_isoform_length_2762_18044 | ---NA--- | | |
| 1885\|_\|c11428_f2p1_2762_isoform_c11428_full_length_coverage_2_non_full_length_coverage_1_isoform_length_2762_17445 | ---NA--- | | |
| 1886\|_\|c36218_f2p5_2762_isoform_c36218_full_length_coverage_2_non_full_length_coverage_5_isoform_length_2762_23579 | XP_020619828.1electron transfer flavoprotein-ubiquinone oxidoreductase, mitochondrial-like | | |
| 1887\|_\|c3373_f2p2_2762_isoform_c3373_full_length_coverage_2_non_full_length_coverage_2_isoform_length_2762_21342 | XP_018024367.1 pre-mRNA-processing-splicing factor 8 | P:GO:0000398; C:GO:0005681; F:GO:0017070; F:GO:0030623 | |
| 1889\|_\|c2533_f6p2_2761_isoform_c2533_full_length_coverage_6_non_full_length_coverage_2_isoform_length_2761_21020 | XP_018025315.1 mitochondrial import receptor subunit TOM40 homolog 1-like | | |

<table>
<tr><td>1890|_|c18218_f2p6_2761_isoform_c18218_full_length_coverage_2_non_full_length_coverage_6_isoform_length_2761_13654</td><td>XP_018010583.1 transcriptional adapter 3-like isoform X2</td><td></td><td></td></tr>
<tr><td>1891|_|c2656_f3p3_2760_isoform_c2656_full_length_coverage_3_non_full_length_coverage_3_isoform_length_2760_16590</td><td>---NA---</td><td></td><td></td></tr>
<tr><td>TRINITY_DN25969_c1_g1_i5_len_4776_227850.p1_type_complete_len_920</td><td>AHL26189.1lipoprotein receptor 1</td><td>C:GO:0016020</td><td></td></tr>
<tr><td>1895|_|c17972_f2p4_2759_isoform_c17972_full_length_coverage_2_non_full_length_coverage_4_isoform_length_2759_17866</td><td>XP_019554478.1 elongation factor G, mitochondrial</td><td>F:GO:0003746; F:GO:0003924; F:GO:0005525; C:GO:0005739; P:GO:0070125</td><td>EC:3.6.1.15</td></tr>
<tr><td>TRINITY_DN9834_c0_g1_i1_len_691_163661.p1_type_complete_len_147</td><td>XP_018019756.1 mediator of RNA polymerase II transcription subunit 22-like</td><td></td><td></td></tr>
<tr><td>TRINITY_DN2673_c0_g1_i1_len_490_204549.p1_type_5prime_partial_len_147</td><td>XP_023226835.1transmembrane protein 229B-like</td><td></td><td></td></tr>
<tr><td>TRINITY_DN25248_c0_g1_i1_len_1232_194731.p2_type_complete_len_147</td><td>---NA---</td><td></td><td></td></tr>
<tr><td>TRINITY_DN13146_c0_g1_i1_len_442_206267.p1_type_internal_len_148</td><td>---NA---</td><td></td><td></td></tr>
<tr><td>TRINITY_DN12685_c0_g1_i1_len_757_238449.p2_type_5prime_partial_len_147</td><td>---NA---</td><td></td><td></td></tr>
<tr><td>TRINITY_DN2113_c0_g1_i1_len_441_206281.p1_type_internal_len_148</td><td>XP_010718169.1 UPF0505 protein C16orf62-like</td><td>C:GO:0005768; C:GO:0016020; P:GO:0016197; P:GO:0098876</td><td></td></tr>
<tr><td>TRINITY_DN18973_c0_g1_i1_len_1083_3163.p2_type_5prime_partial_len_147</td><td>---NA---</td><td></td><td></td></tr>
<tr><td>TRINITY_DN8311_c0_g1_i1_len_525_242374.p1_type_3prime_partial_len_148</td><td>---NA---</td><td></td><td></td></tr>
<tr><td>TRINITY_DN16374_c0_g1_i1_len_677_98356.p2_type_5prime_partial_len_147</td><td>---NA---</td><td></td><td></td></tr>
<tr><td>TRINITY_DN34026_c0_g1_i1_len_441_206286.p1_type_internal_len_148</td><td>---NA---</td><td></td><td></td></tr>
<tr><td>TRINITY_DN41273_c0_g1_i1_len_441_206282.p1_type_internal_len_148</td><td>---NA---</td><td></td><td></td></tr>
</table>

| | | | |
|---|---|---|---|
| TRINITY_DN8459_c0_g1_i1_len_540_203163.p1_type_complete_len_147 | XP_018021241.1 UBA-like domain-containing protein 1 | | |
| TRINITY_DN13659_c0_g1_i3_len_518_242580.p1_type_5prime_partial_len_147 | ---NA--- | | |
| TRINITY_DN22001_c0_g1_i1_len_4573_90844.p5_type_5prime_partial_len_147 | ---NA--- | | |
| TRINITY_DN25562_c0_g1_i2_len_1737_231999.p2_type_5prime_partial_len_147 | ---NA--- | | |
| TRINITY_DN2324_c1_g1_i1_len_443_70800.p1_type_internal_len_148 | ---NA--- | | |
| TRINITY_DN14079_c0_g1_i2_len_445_305542.p1_type_internal_len_148 | ---NA--- | | |
| TRINITY_DN9108_c0_g1_i2_len_442_70823.p1_type_internal_len_148 | XP_018018093.1 kinesin-like protein KIF13A isoform X1 | | |
| TRINITY_DN35313_c0_g1_i1_len_442_70822.p1_type_internal_len_148 | XP_018011507.1 SLIT-ROBO Rho GTPase-activating protein 1-like | F:GO:0005096; P:GO:0007165; P:GO:0043547 | |
| TRINITY_DN24857_c0_g1_i1_len_1653_60192.p3_type_5prime_partial_len_147 | XP_018027163.1 UDP-glucuronosyltransferase 2B14-like | F:GO:0016740 | |
| TRINITY_DN10424_c0_g1_i1_len_511_275788.p1_type_5prime_partial_len_147 | ---NA--- | | |
| TRINITY_DN7336_c0_g1_i1_len_442_305629.p1_type_internal_len_148 | ---NA--- | | |
| TRINITY_DN23027_c0_g1_i2_len_1846_123411.p2_type_3prime_partial_len_148 | ---NA--- | | |
| TRINITY_DN16819_c0_g1_i2_len_1991_231149.p2_type_complete_len_147 | ---NA--- | | |
| TRINITY_DN1919_c0_g1_i1_len_444_206154.p1_type_internal_len_148 | AXR98514.1keratin, partial | | |
| TRINITY_DN9480_c0_g1_i1_len_990_160745.p3_type_complete_len_147 | ---NA--- | | |
| TRINITY_DN38101_c0_g1_i1_len_444_206158.p1_type_internal_len_148 | ---NA--- | | |

| | | | |
|---|---|---|---|
| TRINITY_DN19221_c0_g1_i1_len_444_206174.p1_type_internal_len_148 | XP_023725517.1netrin receptor DCC-like | C:GO:0016020; C:GO:0016021 | |
| TRINITY_DN35241_c0_g1_i1_len_444_206180.p1_type_internal_len_148 | KDR07169.1Pleckstrin-like proteiny domain-containing family A member 5 | | |
| TRINITY_DN37060_c0_g1_i1_len_444_206184.p1_type_internal_len_148 | XP_018009310.1PREDICTED: uncharacterized protein LOC108666881 | | |
| TRINITY_DN9721_c0_g1_i1_len_443_206217.p1_type_internal_len_148 | XP_018020025.1 spermine oxidase-like | | |
| TRINITY_DN41560_c0_g1_i1_len_443_206219.p1_type_internal_len_148 | ---NA--- | | |
| TRINITY_DN20931_c0_g1_i1_len_443_206232.p1_type_internal_len_148 | ---NA--- | | |
| TRINITY_DN20566_c0_g1_i1_len_3042_264928.p8_type_5prime_partial_len_147 | XP_021914577.1papilin isoform X3 | P:GO:0010466; F:GO:0030414 | |
| TRINITY_DN41642_c0_g1_i1_len_443_206202.p1_type_internal_len_148 | AKS04548.1cysteine sulfinic acid decarboxylase | F:GO:0016831; P:GO:0019752; F:GO:0030170 | |
| TRINITY_DN3235_c0_g1_i1_len_443_206210.p1_type_internal_len_148 | XP_016146496.1 tubulin alpha chain | F:GO:0003924; F:GO:0005200; F:GO:0005525; C:GO:0005874; P:GO:0007010; P:GO:0007017 | EC:3.6.1.15 |
| TRINITY_DN11987_c0_g1_i3_len_443_206212.p1_type_internal_len_148 | ---NA--- | | |
| TRINITY_DN16211_c0_g1_i1_len_617_240510.p1_type_5prime_partial_len_147 | ---NA--- | | |
| TRINITY_DN29354_c0_g1_i1_len_444_70751.p1_type_internal_len_148 | ---NA--- | | |
| TRINITY_DN31106_c0_g1_i2_len_5657_186984.p4_type_5prime_partial_len_147 | ---NA--- | | |
| TRINITY_DN3507_c0_g1_i1_len_524_203556.p1_type_5prime_partial_len_147 | XP_026757636.1NCK-interacting protein with SH3 domain | | |
| TRINITY_DN9775_c0_g1_i2_len_996_160695.p1_type_5prime_partial_len_147 | XP_018014885.1 axonemal dynein light intermediate polypeptide 1-like | | |
| TRINITY_DN25855_c0_g1_i6_len_5214_227744.p2_type_complete_len_147 | ---NA--- | | |

| | | | |
|---|---|---|---|
| TRINITY_DN18647_c0_g1_i2_len_1019_196397.p1_type_5prime_partial_len_147 | XP_022204808.1probable G-protein coupled receptor 158 isoform X1 | | |
| TRINITY_DN37374_c0_g1_i1_len_622_240427.p1_type_5prime_partial_len_147 | ---NA--- | | |
| TRINITY_DN25969_c1_g1_i5_len_4776_227850.p5_type_complete_len_147 | ---NA--- | | |
| TRINITY_DN20388_c0_g5_i1_len_572_37387.p1_type_complete_len_147 | ---NA--- | | |
| TRINITY_DN18626_c0_g1_i1_len_486_243398.p1_type_5prime_partial_len_147 | ---NA--- | | |
| TRINITY_DN5275_c0_g1_i1_len_443_168651.p2_type_internal_len_148 | ---NA--- | | |
| TRINITY_DN14054_c0_g1_i1_len_444_10302.p1_type_internal_len_148 | XP_018022458.1 kinesin-like protein KIF17 | F:GO:0005524; C:GO:0005871; C:GO:0005874; C:GO:0005929; F:GO:0008017; F:GO:0008574; P:GO:0030030; C:GO:0032839; P:GO:0098971 | EC:3.6.1.3; EC:3.6.1.15; EC:3.6.4.4 |
| TRINITY_DN21085_c0_g1_i1_len_443_277553.p1_type_internal_len_148 | ---NA--- | | |
| TRINITY_DN3394_c0_g1_i1_len_483_243479.p1_type_3prime_partial_len_148 | XP_017769938.1 E3 ubiquitin-protein ligase TRIM56-like isoform X1 | | |
| TRINITY_DN16081_c0_g1_i2_len_2163_91873.p3_type_5prime_partial_len_147 | ---NA--- | | |
| TRINITY_DN12130_c0_g1_i1_len_443_10327.p1_type_internal_len_148 | ---NA--- | | |
| TRINITY_DN29470_c0_g1_i1_len_442_168688.p1_type_internal_len_148 | ---NA--- | | |
| TRINITY_DN7376_c0_g1_i1_len_443_168660.p1_type_internal_len_148 | ---NA--- | | |
| TRINITY_DN8661_c0_g1_i1_len_445_244742.p1_type_internal_len_148 | ---NA--- | | |
| TRINITY_DN1876_c0_g1_i1_len_445_244728.p1_type_internal_len_148 | ALK82309.1acetyl-CoA carboxylase | F:GO:0003989; F:GO:0005524; P:GO:0006633; F:GO:0046872 | EC:6.4.1.2 |

| | | | |
|---|---|---|---|
| TRINITY_DN3314_c0_g1_i1_len_445_244726.p1_type_internal_len_148 | XP_026282094.1C2 domain-containing protein 5 isoform X2 | | |
| TRINITY_DN17638_c0_g1_i3_len_442_102896.p1_type_internal_len_148 | ---NA--- | | |
| TRINITY_DN3533_c0_g1_i1_len_443_40094.p1_type_internal_len_148 | XP_013860411.1 zinc finger protein OZF-like | F:GO:0003676 | |
| TRINITY_DN8139_c0_g1_i1_len_443_244813.p1_type_internal_len_148 | ---NA--- | | |
| TRINITY_DN33971_c0_g1_i1_len_443_244809.p1_type_internal_len_148 | ---NA--- | | |
| TRINITY_DN31119_c0_g1_i1_len_443_244807.p1_type_internal_len_148 | EFX86052.1hypothetical protein DAPPUDRAFT_309043 | F:GO:0000254; F:GO:0005506; C:GO:0005789; C:GO:0016021; P:GO:0016126; P:GO:0055114 | EC:1.14.13.72; EC:1.14.13 |
| TRINITY_DN1979_c0_g1_i1_len_443_244800.p1_type_internal_len_148 | ---NA--- | | |
| TRINITY_DN13811_c1_g1_i1_len_443_244779.p1_type_internal_len_148 | XP_018017374.1 fat-like cadherin-related tumor suppressor homolog | | |
| TRINITY_DN32794_c0_g1_i1_len_443_244776.p1_type_internal_len_148 | ---NA--- | | |
| TRINITY_DN14851_c0_g1_i1_len_442_102918.p1_type_internal_len_148 | ACU31809.1alpha2 macroglobulin isoform 3 | F:GO:0004866; C:GO:0005615; P:GO:0010951 | |
| TRINITY_DN34966_c0_g1_i1_len_793_198698.p1_type_5prime_partial_len_147 | XP_018572814.1protein FAM32A | | |
| TRINITY_DN34045_c0_g1_i1_len_513_203841.p1_type_5prime_partial_len_147 | AYN63914.1amine oxidase | | |
| TRINITY_DN26696_c0_g1_i1_len_441_244863.p1_type_internal_len_148 | ---NA--- | | |
| TRINITY_DN32689_c0_g1_i1_len_442_244836.p3_type_internal_len_148 | AAP59794.1slow muscle myosin S1 heavy chain | F:GO:0003774; C:GO:0016459 | EC:3.6.1.15 |
| TRINITY_DN37161_c0_g1_i1_len_442_244825.p1_type_internal_len_148 | ---NA--- | | |
| TRINITY_DN26684_c0_g1_i1_len_442_244822.p2_type_internal_len_148 | ---NA--- | | |

| | | | |
|---|---|---|---|
| TRINITY_DN22545_c0_g1_i1_len_668_200496.p2_type_5prime_partial_len_147 | ---NA--- | | |
| TRINITY_DN7635_c0_g1_i1_len_443_102868.p1_type_internal_len_148 | ---NA--- | | |
| TRINITY_DN25355_c0_g1_i1_len_443_102872.p1_type_internal_len_148 | AXN72354.1Wnt10 | F:GO:0005102; C:GO:0005576; P:GO:0007275; P:GO:0016055 | |
| TRINITY_DN4197_c0_g1_i1_len_518_68672.p1_type_5prime_partial_len_147 | XP_018019453.1 mitochondrial amidoxime-reducing component 1-like | | |
| TRINITY_DN18666_c0_g1_i3_len_443_133844.p5_type_internal_len_148 | OWF36882.1Trophinin | C:GO:0016020; C:GO:0016021 | |
| TRINITY_DN19899_c0_g2_i1_len_448_39954.p1_type_5prime_partial_len_147 | AUB30144.1slow-type skeletal muscle actin 9 | F:GO:0005524 | |
| TRINITY_DN29521_c0_g1_i1_len_442_133859.p1_type_internal_len_148 | XP_021919250.1tensin-4-like isoform X3 | | |
| TRINITY_DN11851_c0_g1_i1_len_442_133863.p1_type_internal_len_148 | ---NA--- | | |
| TRINITY_DN22332_c0_g1_i1_len_1089_195769.p2_type_complete_len_147 | ---NA--- | | |
| TRINITY_DN25092_c1_g2_i1_len_565_67716.p1_type_5prime_partial_len_147 | ---NA--- | | |
| TRINITY_DN7236_c0_g1_i1_len_454_9979.p1_type_5prime_partial_len_147 | ---NA--- | | |
| TRINITY_DN16697_c0_g1_i2_len_601_67080.p1_type_5prime_partial_len_147 | XP_018026542.1 guanine nucleotide exchange factor MSS4 homolog | F:GO:0005085; P:GO:0007264 | |
| TRINITY_DN5215_c0_g1_i1_len_442_133865.p1_type_internal_len_148 | AAM90311.1elongation factor 1-alpha | F:GO:0003746; P:GO:0006414 | |
| TRINITY_DN32151_c0_g1_i1_len_442_133872.p1_type_internal_len_148 | ---NA--- | | |
| TRINITY_DN11582_c0_g1_i1_len_442_133876.p1_type_internal_len_148 | XP_018016182.1 protein DGCR14-like | | |
| TRINITY_DN6482_c0_g1_i1_len_2144_91904.p2_type_5prime_partial_len_147 | ---NA--- | | |

| | | | |
|---|---|---|---|
| TRINITY_DN29417_c0_g1_i1_len_441_133921.p1_type_internal_len_148 | XP_022690760.1sushi, von Willebrand factor type A, EGF and pentraxin domain-containing protein 1-like | | |
| TRINITY_DN31041_c0_g1_i1_len_480_69667.p1_type_5prime_partial_len_147 | XP_022198815.1zinc finger protein OZF-like | | |
| TRINITY_DN18848_c0_g1_i1_len_1497_157822.p2_type_3prime_partial_len_148 | AGN98124.1heat shock protein | | |
| TRINITY_DN8646_c0_g1_i1_len_444_277520.p1_type_internal_len_148 | XP_018013858.1 alkylglycerol monooxygenase-like | P:GO:0008152; C:GO:0016020; F:GO:0016491 | |
| TRINITY_DN25505_c0_g1_i1_len_1118_195546.p1_type_complete_len_147 | ---NA--- | | |
| TRINITY_DN17643_c0_g1_i1_len_632_98938.p1_type_complete_len_147 | XP_018014487.1PREDICTED: uncharacterized protein LOC108671450 | | |
| TRINITY_DN25105_c0_g2_i4_len_569_67633.p1_type_5prime_partial_len_147 | ---NA--- | | |
| TRINITY_DN9945_c0_g1_i1_len_442_244823.p1_type_internal_len_147 | ---NA--- | | |
| TRINITY_DN13294_c0_g1_i1_len_440_206310.p1_type_internal_len_147 | ---NA--- | | |
| TRINITY_DN40173_c0_g1_i1_len_440_206317.p1_type_internal_len_147 | XP_018026842.1PREDICTED: uncharacterized protein LOC108682222 | | |
| 1896\|_\|c3952_f2p0_2759_isoform_c3952_full_length_coverage_2_non_full_length_coverage_0_isoform_length_2759_16811 | ---NA--- | | |
| 1898\|_\|c1335_f15p15_2757_isoform_c1335_full_length_coverage_15_non_full_length_coverage_15_isoform_length_2757_11372 | XP_018006524.1 sialin-like | | |
| 1899\|_\|c1879_f7p2_2757_isoform_c1879_full_length_coverage_7_non_full_length_coverage_2_isoform_length_2757_20585 | XP_018024362.1 twinkle protein, mitochondrial-like | | |
| 1900\|_\|c36400_f2p51_2757_isoform_c36400_full_length_coverage_2_non_full_length_coverage_51_isoform_length_2757_23600 | APA05127.1polephole-like protein | | |

| | | | |
|---|---|---|---|
| TRINITY_DN26214_c0_g1_i1_len_3057_188439.p1_type_complete_len_919 | XP_018009310.1PREDICTED: uncharacterized protein LOC108666881 | | |
| TRINITY_DN26050_c0_g1_i1_len_3101_188374.p1_type_5prime_partial_len_919 | XP_018028047.1 DNA replication licensing factor mcm2-like | F:GO:0003677; F:GO:0004386; F:GO:0005524; C:GO:0005634; P:GO:0006270; C:GO:0042555; P:GO:1905775 | EC:3.6.1.15 |
| 1904\|_\|c2952_f2p2_2757_isoform_c2952_full_length_coverage_2_non_full_length_coverage_2_isoform_length_2757_16725 | XP_018015707.1PREDICTED: uncharacterized protein LOC108672531 isoform X1 | | |
| 1906\|_\|c7585_f3p14_2756_isoform_c7585_full_length_coverage_3_non_full_length_coverage_14_isoform_length_2756_21705 | XP_018019692.1 dihydropyrimidinase-like isoform X3 | | |
| TRINITY_DN2343_c0_g1_i1_len_2968_194.p1_type_5prime_partial_len_918 | XP_018027647.1 DNA replication licensing factor MCM4-like | F:GO:0003677; F:GO:0003678; F:GO:0005524; C:GO:0005634; P:GO:0006270; P:GO:0032508; C:GO:0042555 | EC:3.6.1.15 |
| 1913\|_\|c2844_f2p0_2754_isoform_c2844_full_length_coverage_2_non_full_length_coverage_0_isoform_length_2754_21281 | XP_024052402.1histone H4 transcription factor isoform X1 | | |
| TRINITY_DN21197_c0_g1_i1_len_656_6234.p1_type_5prime_partial_len_146 | ---NA--- | | |
| TRINITY_DN9984_c1_g1_i1_len_440_206309.p1_type_internal_len_147 | ---NA--- | | |
| TRINITY_DN8092_c0_g1_i1_len_439_277671.p1_type_internal_len_147 | ---NA--- | | |
| TRINITY_DN30569_c0_g1_i1_len_5140_187095.p2_type_3prime_partial_len_147 | ---NA--- | | |
| TRINITY_DN36845_c0_g1_i1_len_442_244819.p2_type_internal_len_147 | XP_022252404.1probable phospholipid-transporting ATPase VA isoform X2 | | |
| TRINITY_DN3317_c0_g1_i1_len_442_244830.p1_type_internal_len_147 | ---NA--- | | |

| | | | |
|---|---|---|---|
| TRINITY_DN41826_c0_g1_i1_len_440_206321.p1_type_internal_len_147 | RDD39982.1ATP-binding cassette sub-family A member 5 | F:GO:0000166; F:GO:0005524; C:GO:0016020; C:GO:0016021; F:GO:0016887; F:GO:0042626; P:GO:0055085 | |
| TRINITY_DN9935_c0_g1_i2_len_657_6218.p1_type_complete_len_146 | XP_013409845.1AP-4 complex subunit sigma-1 | P:GO:0006810 | |
| TRINITY_DN34678_c0_g1_i1_len_442_244833.p1_type_internal_len_147 | ---NA--- | | |
| TRINITY_DN4767_c0_g1_i1_len_438_277721.p1_type_internal_len_147 | ---NA--- | | |
| TRINITY_DN21947_c0_g1_i2_len_1078_62635.p2_type_3prime_partial_len_147 | ---NA--- | | |
| TRINITY_DN420_c0_g1_i1_len_440_244888.p1_type_internal_len_147 | ---NA--- | | |
| TRINITY_DN23489_c0_g2_i2_len_1228_125307.p1_type_complete_len_146 | XP_006622544.1 bis(5'-nucleosyl)-tetraphosphatase [asymmetrical]-like | F:GO:0016787 | |
| TRINITY_DN10219_c0_g1_i1_len_440_244893.p1_type_internal_len_147 | ---NA--- | | |
| TRINITY_DN34417_c0_g1_i1_len_440_244895.p1_type_internal_len_147 | ---NA--- | | |
| TRINITY_DN20206_c0_g1_i1_len_440_244892.p3_type_internal_len_147 | EDO36870.1predicted protein | | |
| TRINITY_DN18682_c0_g1_i1_len_440_206312.p1_type_internal_len_147 | ADG86240.1triosephosphate isomerase | F:GO:0004807; P:GO:0006094; P:GO:0006096 | EC:5.3.1.1 |
| TRINITY_DN13194_c0_g1_i1_len_438_277704.p2_type_internal_len_147 | XP_018026016.1 filaggrin-2-like isoform X3 | | |
| TRINITY_DN14191_c0_g1_i1_len_439_244897.p1_type_internal_len_147 | ---NA--- | | |
| TRINITY_DN7582_c0_g1_i1_len_438_244941.p1_type_internal_len_147 | ---NA--- | | |
| TRINITY_DN27010_c0_g1_i1_len_441_244864.p1_type_internal_len_147 | XP_023258550.1long-chain fatty acid transport protein 4-like | | |

| | | | |
|---|---|---|---|
| TRINITY_DN1079_c0_g1_i1_len_441_244859.p1_type_internal_len_147 | ---NA--- | | |
| TRINITY_DN28728_c0_g1_i1_len_442_244839.p1_type_internal_len_147 | XP_014242077.1negative elongation factor A | | |
| TRINITY_DN35396_c0_g1_i1_len_439_244913.p1_type_internal_len_147 | ---NA--- | | |
| TRINITY_DN36441_c0_g1_i1_len_440_244878.p1_type_internal_len_147 | KDR24324.1PH and SEC7 domain-containing protein 3 | F:GO:0005086; F:GO:0005543; P:GO:0032012 | |
| TRINITY_DN11493_c0_g1_i1_len_440_206336.p1_type_internal_len_147 | ---NA--- | | |
| TRINITY_DN13487_c0_g1_i2_len_809_237856.p1_type_5prime_partial_len_146 | XP_018009721.1 BTB/POZ domain-containing protein 10-like isoform X1 | | |
| TRINITY_DN13559_c0_g1_i1_len_440_206335.p1_type_internal_len_147 | ---NA--- | | |
| TRINITY_DN8564_c0_g1_i1_len_439_206348.p1_type_internal_len_147 | ---NA--- | | |
| TRINITY_DN25626_c0_g1_i4_len_677_200348.p1_type_complete_len_146 | ---NA--- | | |
| TRINITY_DN28421_c0_g1_i1_len_519_275618.p1_type_5prime_partial_len_146 | XP_018012973.1PREDICTED: uncharacterized protein LOC108670034 | | |
| TRINITY_DN37405_c0_g1_i1_len_439_206350.p1_type_internal_len_147 | ---NA--- | | |
| TRINITY_DN3895_c0_g1_i1_len_442_102927.p1_type_5prime_partial_len_146 | XP_021917146.1phosphofurin acidic cluster sorting protein 1 isoform X1 | | |
| TRINITY_DN14963_c0_g1_i1_len_957_270492.p1_type_5prime_partial_len_146 | XP_018016123.1 UPF0538 protein C2orf76 homolog | | |
| TRINITY_DN1405_c0_g1_i1_len_440_206340.p1_type_internal_len_147 | ---NA--- | | |
| TRINITY_DN21956_c0_g1_i1_len_439_277668.p1_type_internal_len_147 | XP_023349466.1myocyte-specific enhancer factor 2-like isoform X2 | | |
| TRINITY_DN16606_c0_g1_i2_len_595_99500.p1_type_5prime_partial_len_146 | XP_021373185.1protein yippee-like 5 isoform X1 | | |

| | | | |
|---|---|---|---|
| TRINITY_DN23344_c0_g1_i1_len_4197_228089.p2_type_complete_len_146 | ---NA--- | | |
| TRINITY_DN29400_c0_g1_i1_len_440_277657.p1_type_internal_len_147 | ---NA--- | | |
| TRINITY_DN27203_c0_g1_i1_len_439_277669.p1_type_internal_len_147 | ---NA--- | | |
| TRINITY_DN39536_c0_g1_i1_len_440_206327.p1_type_internal_len_147 | XP_018026912.1 solute carrier family 23 member 1-like | | |
| TRINITY_DN1009_c0_g1_i1_len_440_206331.p1_type_internal_len_147 | XP_018016633.1 intraflagellar transport protein 140 homolog | C:GO:0031514 | |
| TRINITY_DN9312_c0_g1_i1_len_712_163379.p1_type_3prime_partial_len_147 | ---NA--- | | |
| TRINITY_DN14850_c0_g1_i1_len_440_206329.p1_type_3prime_partial_len_147 | AIE45536.1prophenoloxidase 2-like protein | F:GO:0016491; F:GO:0046872; P:GO:0055114 | |
| TRINITY_DN2978_c0_g1_i1_len_497_8898.p1_type_5prime_partial_len_146 | ---NA--- | | |
| TRINITY_DN12826_c0_g1_i1_len_492_38864.p1_type_5prime_partial_len_146 | ---NA--- | | |
| TRINITY_DN2402_c0_g1_i1_len_438_206402.p1_type_internal_len_147 | XP_018020970.1 synaptojanin-1-like isoform X2 | | |
| TRINITY_DN41722_c0_g1_i1_len_441_206278.p1_type_internal_len_147 | XP_018025352.1 tyrosine-protein phosphatase 99A-like isoform X2 | | |
| TRINITY_DN7030_c0_g1_i1_len_441_206279.p1_type_internal_len_147 | ACL36280.1Kazal-type serine proteinase inhibitor 1 | | |
| TRINITY_DN15996_c0_g1_i1_len_442_206270.p1_type_internal_len_147 | ---NA--- | | |
| TRINITY_DN24157_c1_g1_i1_len_1859_231528.p3_type_complete_len_146 | ---NA--- | | |
| TRINITY_DN16899_c0_g1_i1_len_2068_123003.p2_type_5prime_partial_len_146 | AIG59666.1phosphopyruvate hydratase | C:GO:0000015; F:GO:0000287; F:GO:0004634; P:GO:0006096 | EC:4.2.1.11 |
| TRINITY_DN15811_c0_g1_i2_len_441_10373.p1_type_3prime_partial_len_147 | ---NA--- | | |

| | | | |
|---|---|---|---|
| TRINITY_DN16080_c0_g1_i1_len_539_68209.p1_type_5prime_partial_len_146 | XP_014774179.1 tektin-2-like isoform X1 | | |
| TRINITY_DN18881_c0_g1_i2_len_865_237291.p1_type_3prime_partial_len_147 | ---NA--- | | |
| TRINITY_DN32435_c0_g1_i1_len_438_206434.p1_type_internal_len_147 | XP_023702383.1GTPase-activating protein CdGAPr | | |
| TRINITY_DN6858_c0_g1_i1_len_461_305118.p1_type_5prime_partial_len_146 | ---NA--- | | |
| TRINITY_DN41913_c0_g1_i1_len_442_206253.p1_type_internal_len_147 | ---NA--- | | |
| TRINITY_DN40813_c0_g1_i1_len_442_206252.p1_type_internal_len_147 | ---NA--- | | |
| TRINITY_DN30464_c0_g1_i1_len_732_238739.p1_type_5prime_partial_len_146 | AAZ29608.1C-type lectin | F:GO:0030246 | |
| TRINITY_DN35501_c0_g1_i1_len_442_206250.p1_type_internal_len_147 | XP_018008617.1 matrix metalloproteinase-2-like | | |
| TRINITY_DN36356_c0_g1_i1_len_442_206246.p1_type_internal_len_147 | ---NA--- | | |
| TRINITY_DN9313_c0_g1_i1_len_438_206412.p2_type_internal_len_147 | XP_018018462.1 cell adhesion molecule 3-like | | |
| TRINITY_DN39732_c0_g1_i1_len_499_204281.p1_type_5prime_partial_len_146 | ---NA--- | | |
| TRINITY_DN2815_c0_g1_i1_len_725_238827.p1_type_3prime_partial_len_147 | ---NA--- | | |
| TRINITY_DN1985_c0_g1_i1_len_727_238804.p1_type_5prime_partial_len_146 | ---NA--- | | |
| TRINITY_DN205_c0_g1_i1_len_442_206258.p1_type_internal_len_147 | AAL05973.1peroxinectin | F:GO:0004601; P:GO:0006979; F:GO:0020037; P:GO:0055114; P:GO:0098869 | EC:1.11.1.7 |
| TRINITY_DN14564_c0_g1_i1_len_915_96153.p2_type_5prime_partial_len_146 | AUW64507.1serine protease 3 | F:GO:0004252; P:GO:0006508 | EC:3.4.21 |
| TRINITY_DN20314_c0_g1_i4_len_2258_295379.p2_type_complete_len_146 | XP_015796939.1 gastrula zinc finger protein XlCGF57.1-like | | |

| | | | |
|---|---|---|---|
| TRINITY_DN19395_c0_g1_i1_len_438_206417.p1_type_internal_len_147 | ACT64631.1delta protein | F:GO:0005509; P:GO:0007219; P:GO:0007275; C:GO:0016021 | |
| TRINITY_DN10493_c0_g1_i1_len_497_204344.p1_type_5prime_partial_len_146 | ---NA--- | | |
| TRINITY_DN41697_c0_g1_i1_len_441_206298.p1_type_internal_len_147 | ---NA--- | | |
| TRINITY_DN6254_c0_g1_i1_len_439_206380.p1_type_internal_len_147 | XP_023226349.1serine/threonine-protein phosphatase 4 regulatory subunit 4-like | | |
| TRINITY_DN15428_c0_g1_i2_len_754_35168.p1_type_5prime_partial_len_146 | ---NA--- | | |
| TRINITY_DN5515_c0_g1_i1_len_438_10497.p2_type_internal_len_147 | ---NA--- | | |
| TRINITY_DN7558_c0_g1_i1_len_439_206379.p1_type_internal_len_147 | ---NA--- | | |
| TRINITY_DN22043_c0_g1_i2_len_1028_196329.p1_type_5prime_partial_len_146 | ---NA--- | | |
| TRINITY_DN10620_c0_g1_i2_len_916_197332.p2_type_5prime_partial_len_146 | XP_018011231.1 zinc finger protein 513-like | | |
| TRINITY_DN39455_c0_g1_i1_len_441_206303.p1_type_internal_len_147 | ---NA--- | | |
| TRINITY_DN10234_c0_g1_i1_len_492_132582.p1_type_5prime_partial_len_146 | ---NA--- | | |
| TRINITY_DN17875_c0_g1_i1_len_440_206305.p1_type_internal_len_147 | ---NA--- | | |
| TRINITY_DN21473_c0_g1_i1_len_1887_191323.p2_type_5prime_partial_len_146 | ---NA--- | | |
| TRINITY_DN11454_c0_g1_i1_len_441_10391.p1_type_internal_len_147 | XP_018017531.1PREDICTED: uncharacterized protein LOC108674134 | | |
| TRINITY_DN22734_c0_g1_i2_len_1291_194308.p2_type_5prime_partial_len_146 | ---NA--- | | |
| TRINITY_DN19535_c0_g1_i1_len_973_3762.p4_type_5prime_partial_len_146 | XP_018011092.1 delta-1-pyrroline-5-carboxylate synthase-like | | |

| | | | |
|---|---|---|---|
| TRINITY_DN20626_c0_g1_i1_len_439_206381.p1_type_internal_len_147 | ---NA--- | | |
| TRINITY_DN16363_c0_g1_i1_len_1240_234432.p1_type_complete_len_146 | ---NA--- | | |
| TRINITY_DN7774_c0_g1_i1_len_439_206390.p1_type_internal_len_147 | ---NA--- | | |
| TRINITY_DN21020_c0_g1_i1_len_854_237390.p1_type_complete_len_146 | XP_022192271.1coiled-coil domain-containing protein 58-like | | |
| TRINITY_DN31443_c0_g1_i1_len_440_10412.p1_type_internal_len_147 | ---NA--- | | |
| TRINITY_DN10809_c0_g1_i1_len_440_70898.p2_type_internal_len_147 | XP_020720664.1histone demethylase UTY isoform X1 | | |
| TRINITY_DN14445_c0_g1_i1_len_440_70897.p1_type_5prime_partial_len_146 | ---NA--- | | |
| TRINITY_DN8338_c0_g1_i1_len_450_168414.p1_type_5prime_partial_len_146 | ---NA--- | | |
| TRINITY_DN12702_c0_g1_i2_len_489_243342.p1_type_3prime_partial_len_147 | ---NA--- | | |
| TRINITY_DN5165_c0_g1_i1_len_441_277606.p1_type_internal_len_147 | APF30038.1ionotropic glutamate receptor NMDA 2A | F:GO:0004970; C:GO:0016021; C:GO:0030054; P:GO:0034220; P:GO:0035235; C:GO:0045211 | |
| TRINITY_DN3478_c0_g1_i1_len_441_70868.p1_type_internal_len_147 | XP_014350417.1 xaa-Pro aminopeptidase 1 isoform X3 | F:GO:0016787 | |
| TRINITY_DN16536_c0_g1_i4_len_440_70892.p3_type_internal_len_147 | ---NA--- | | |
| TRINITY_DN17371_c0_g1_i1_len_554_303114.p1_type_5prime_partial_len_146 | ---NA--- | | |
| TRINITY_DN11820_c0_g1_i1_len_520_100855.p1_type_3prime_partial_len_147 | AAQ94739.1signal transducer and activator of transcription | F:GO:0003677; F:GO:0003700; C:GO:0005634; C:GO:0005737; P:GO:0006355; P:GO:0060397 | |
| TRINITY_DN8376_c0_g1_i1_len_449_168442.p1_type_5prime_partial_len_146 | ---NA--- | | |

<table>
<tr><td>1914|_|c2870_f2p2_2753_isoform_c2870_full_length_coverage_2_non_full_length_coverage_2_isoform_length_2753_12233</td><td>XP_018020719.1 phosphatidylinositol 3-kinase regulatory subunit alpha-like</td><td>C:GO:0005942; F:GO:0035014; P:GO:0043551</td><td></td></tr>
<tr><td>1915|_|c2506_f3p4_2753_isoform_c2506_full_length_coverage_3_non_full_length_coverage_4_isoform_length_2753_11923</td><td>XP_022246978.1FERM domain-containing protein 4A-like</td><td>C:GO:0005856</td><td></td></tr>
<tr><td>1916|_|c2103_f7p3_2752_isoform_c2103_full_length_coverage_7_non_full_length_coverage_3_isoform_length_2752_20707</td><td>XP_018024584.1 transmembrane protein 47-like isoform X1</td><td></td><td></td></tr>
<tr><td>1917|_|c13244_f3p3_2752_isoform_c13244_full_length_coverage_3_non_full_length_coverage_3_isoform_length_2752_17578</td><td>XP_023323894.1MICOS complex subunit MIC60-1-like</td><td>C:GO:0016020</td><td></td></tr>
<tr><td>1919|_|c24568_f2p1_2752_isoform_c24568_full_length_coverage_2_non_full_length_coverage_1_isoform_length_2752_13905</td><td>ABO38431.1inhibitor of apoptosis protein</td><td></td><td></td></tr>
<tr><td>1921|_|c1982_f2p2_2751_isoform_c1982_full_length_coverage_2_non_full_length_coverage_2_isoform_length_2751_25636</td><td>---NA---</td><td></td><td></td></tr>
<tr><td>TRINITY_DN23900_c0_g1_i1_len_3001_228988.p1_type_complete_len_917</td><td>AJR21491.1RNA helicase</td><td>F:GO:0003723; F:GO:0004386; F:GO:0032575; P:GO:0035194</td><td></td></tr>
<tr><td>1923|_|c2449_f2p1_2751_isoform_c2449_full_length_coverage_2_non_full_length_coverage_1_isoform_length_2751_16420</td><td>AEL23047.1dystroglycan, partial</td><td>F:GO:0005509; P:GO:0007016; C:GO:0016010; C:GO:0016021</td><td></td></tr>
<tr><td>TRINITY_DN26237_c0_g1_i1_len_4207_228084.p1_type_complete_len_917</td><td>XP_026291635.1DNA polymerase eta</td><td>F:GO:0003684; P:GO:0006281</td><td></td></tr>
<tr><td>1926|_|c17901_f2p2_2750_isoform_c17901_full_length_coverage_2_non_full_length_coverage_2_isoform_length_2750_22554</td><td>XP_025087011.1 tripartite motif-containing protein 2-like</td><td></td><td></td></tr>
<tr><td>TRINITY_DN20071_c0_g1_i3_len_792_97103.p1_type_complete_len_146</td><td>---NA---</td><td></td><td></td></tr>
<tr><td>TRINITY_DN27752_c0_g1_i1_len_438_70977.p1_type_internal_len_147</td><td>XP_018016524.1 neural-cadherin-like isoform X4</td><td></td><td></td></tr>
</table>

| | | | |
|---|---|---|---|
| TRINITY_DN25909_c0_g1_i1_len_440_168773.p1_type_3prime_partial_len_147 | PRD19726.1zinc finger protein | F:GO:0003677 | |
| TRINITY_DN1231_c0_g1_i1_len_440_168774.p1_type_internal_len_147 | XP_026676833.1E3 ubiquitin-protein ligase KCMF1-like | F:GO:0008270; P:GO:0016567; F:GO:0016874; F:GO:0046872; F:GO:0061630 | |
| TRINITY_DN16381_c0_g1_i1_len_439_168784.p1_type_internal_len_147 | ---NA--- | | |
| TRINITY_DN1079_c0_g1_i1_len_440_168767.p1_type_internal_len_147 | ODM98676.1Transmembrane protein | C:GO:0016021; F:GO:0016787 | |
| TRINITY_DN19461_c0_g1_i1_len_445_70717.p1_type_5prime_partial_len_146 | ---NA--- | | |
| TRINITY_DN26705_c0_g1_i1_len_440_102984.p1_type_internal_len_147 | ---NA--- | | |
| TRINITY_DN10483_c0_g1_i1_len_585_99671.p1_type_complete_len_146 | ---NA--- | | |
| TRINITY_DN11427_c0_g1_i1_len_441_70860.p1_type_internal_len_147 | ---NA--- | | |
| TRINITY_DN30863_c0_g1_i1_len_481_243567.p1_type_5prime_partial_len_146 | ---NA--- | | |
| TRINITY_DN9553_c0_g1_i1_len_440_40205.p1_type_internal_len_147 | ---NA--- | | |
| TRINITY_DN32764_c0_g1_i1_len_441_102954.p1_type_internal_len_147 | ---NA--- | | |
| TRINITY_DN8347_c0_g1_i1_len_440_133943.p1_type_internal_len_147 | ---NA--- | | |
| TRINITY_DN14569_c0_g1_i1_len_440_40185.p1_type_internal_len_147 | ---NA--- | | |
| TRINITY_DN25080_c0_g1_i1_len_843_64191.p2_type_5prime_partial_len_146 | ---NA--- | | |
| TRINITY_DN1059_c0_g1_i1_len_439_103025.p1_type_internal_len_147 | ---NA--- | | |
| TRINITY_DN15756_c0_g1_i2_len_577_302707.p1_type_5prime_partial_len_146 | ---NA--- | | |

| | | | |
|---|---|---|---|
| TRINITY_DN15455_c0_g1_i1_len_467_70073.p1_type_complete_len_146 | ---NA--- | | |
| TRINITY_DN4219_c0_g1_i1_len_441_277619.p1_type_internal_len_147 | XP_018008513.1 bone morphogenetic protein 10-like | | |
| TRINITY_DN28167_c0_g1_i2_len_1570_192706.p2_type_5prime_partial_len_146 | ---NA--- | | |
| TRINITY_DN6038_c0_g1_i1_len_440_133963.p1_type_internal_len_147 | ---NA--- | | |
| TRINITY_DN16954_c0_g1_i7_len_439_133969.p1_type_internal_len_147 | ---NA--- | | |
| TRINITY_DN22945_c0_g2_i1_len_439_133975.p1_type_internal_len_147 | XP_020382909.1transmembrane protein 5 isoform X3 | | |
| TRINITY_DN14608_c0_g1_i1_len_453_205846.p1_type_5prime_partial_len_146 | ---NA--- | | |
| TRINITY_DN414_c0_g1_i1_len_473_69869.p1_type_5prime_partial_len_146 | ---NA--- | | |
| TRINITY_DN35464_c0_g1_i1_len_438_206431.p2_type_internal_len_146 | AGC12524.1abdominal-B isoform II | C:GO:0005634; P:GO:0006355; F:GO:0043565 | |
| TRINITY_DN29729_c0_g1_i1_len_3972_187602.p3_type_5prime_partial_len_145 | ---NA--- | | |
| TRINITY_DN24948_c0_g1_i1_len_3999_228185.p3_type_5prime_partial_len_145 | ---NA--- | | |
| TRINITY_DN16936_c0_g1_i1_len_1350_61266.p1_type_complete_len_145 | ---NA--- | | |
| TRINITY_DN23965_c0_g1_i4_len_438_206435.p2_type_internal_len_146 | ---NA--- | | |
| TRINITY_DN19099_c0_g1_i3_len_1021_235943.p1_type_complete_len_145 | ---NA--- | | |
| TRINITY_DN9570_c0_g1_i1_len_436_134071.p1_type_internal_len_146 | ---NA--- | | |
| TRINITY_DN11553_c0_g1_i2_len_673_239606.p1_type_3prime_partial_len_146 | XP_018015220.1 protein Tob1-like | | |

| | | | |
|---|---|---|---|
| TRINITY_DN23590_c0_g1_i1_len_449_277359.p1_type_5prime_partial_len_145 | ---NA--- | | |
| TRINITY_DN19606_c0_g1_i3_len_634_6498.p1_type_complete_len_145 | XP_023715282.1nuclear envelope phosphatase-regulatory subunit 1 | C:GO:0044425 | |
| TRINITY_DN21203_c0_g1_i1_len_1030_126368.p2_type_5prime_partial_len_145 | ---NA--- | | |
| TRINITY_DN23620_c0_g1_i1_len_701_199979.p1_type_5prime_partial_len_145 | ---NA--- | | |
| TRINITY_DN13824_c0_g1_i1_len_438_206429.p1_type_internal_len_146 | ---NA--- | | |
| TRINITY_DN22591_c0_g1_i2_len_666_200520.p1_type_5prime_partial_len_145 | XP_018016474.1 beta-sarcoglycan-like | P:GO:0007517; C:GO:0016012; C:GO:0016020; C:GO:0016021 | |
| TRINITY_DN21687_c0_g1_i1_len_498_204317.p1_type_5prime_partial_len_145 | ---NA--- | | |
| TRINITY_DN631_c0_g1_i1_len_1115_298199.p1_type_complete_len_145 | ---NA--- | | |
| TRINITY_DN26514_c0_g1_i2_len_1268_194462.p1_type_complete_len_145 | XP_024217969.1zinc finger protein 425-like isoform X1 | | |
| TRINITY_DN27841_c0_g1_i1_len_466_9684.p1_type_5prime_partial_len_145 | PNF39666.1hypothetical protein B7P43_G05631 | F:GO:0045127; P:GO:0046835 | |
| TRINITY_DN1896_c0_g1_i1_len_439_206364.p1_type_internal_len_146 | ---NA--- | | |
| TRINITY_DN20406_c0_g1_i3_len_1407_233464.p1_type_complete_len_145 | ---NA--- | | |
| TRINITY_DN26419_c0_g1_i1_len_437_134050.p1_type_internal_len_146 | ---NA--- | | |
| TRINITY_DN4902_c0_g1_i1_len_549_7837.p2_type_5prime_partial_len_145 | XP_018022816.1 anoctamin-10-like | C:GO:0016020 | |
| TRINITY_DN8241_c0_g1_i1_len_437_206455.p1_type_3prime_partial_len_146 | ---NA--- | | |
| TRINITY_DN5075_c0_g1_i1_len_516_242624.p1_type_5prime_partial_len_145 | ADC42878.1serine proteinase inhibitor 7 | C:GO:0005615; P:GO:0006508; F:GO:0008233 | |

| | | | |
|---|---|---|---|
| TRINITY_DN8653_c0_g1_i1_len_437_206458.p1_type_internal_len_146 | ---NA--- | | |
| TRINITY_DN13553_c0_g1_i1_len_628_36560.p1_type_5prime_partial_len_145 | XP_018010425.1 CDGSH iron-sulfur domain-containing protein 2 homolog B-like | C:GO:0016020; C:GO:0016021; C:GO:0043231; F:GO:0051537 | |
| TRINITY_DN39378_c0_g1_i1_len_542_203101.p1_type_5prime_partial_len_145 | XP_013920798.1 tubulointerstitial nephritis antigen-like | P:GO:0006508; F:GO:0008234 | |
| TRINITY_DN13370_c0_g1_i1_len_437_134040.p1_type_internal_len_146 | ---NA--- | | |
| TRINITY_DN24870_c0_g1_i1_len_1236_194702.p1_type_3prime_partial_len_146 | ---NA--- | | |
| TRINITY_DN18240_c0_g1_i1_len_437_206450.p3_type_internal_len_146 | ---NA--- | | |
| TRINITY_DN18020_c0_g1_i2_len_610_201573.p2_type_5prime_partial_len_145 | ---NA--- | | |
| TRINITY_DN11328_c0_g1_i1_len_437_206438.p1_type_internal_len_146 | ---NA--- | | |
| TRINITY_DN1215_c0_g1_i2_len_438_103040.p2_type_internal_len_146 | ANQ44707.1heat shock protein 70 kDa | F:GO:0005524 | |
| TRINITY_DN30700_c0_g1_i1_len_5954_186939.p2_type_complete_len_145 | ---NA--- | | |
| TRINITY_DN9224_c0_g1_i1_len_436_103098.p1_type_internal_len_146 | XP_020456813.1haloacid dehalogenase-like hydrolase domain-containing protein 2 isoform X1 | | |
| TRINITY_DN31067_c0_g1_i2_len_6016_186930.p4_type_complete_len_145 | ADM88556.1fatty acid synthase | F:GO:0004312; P:GO:0009058; F:GO:0016491; F:GO:0016788; F:GO:0031177; P:GO:0055114 | EC:2.3.1.85 |
| TRINITY_DN10805_c0_g1_i1_len_1323_194118.p1_type_complete_len_145 | ---NA--- | | |
| TRINITY_DN16048_c0_g1_i1_len_967_270418.p2_type_5prime_partial_len_145 | AEB40303.1mitochondrial ATP synthase delta subunit precursor | P:GO:0015986; C:GO:0045261; F:GO:0046933; P:GO:0099132 | EC:3.6.1.3; EC:3.6.1.15 |
| TRINITY_DN21038_c0_g1_i1_len_453_205832.p1_type_3prime_partial_len_146 | ---NA--- | | |

| | | | |
|---|---|---|---|
| TRINITY_DN21209_c0_g1_i6_len_900_96264.p1_type_3prime_partial_len_146 | ---NA--- | | |
| TRINITY_DN16845_c0_g1_i1_len_708_199870.p1_type_5prime_partial_len_145 | XP_018023119.1 zinc finger and BTB domain-containing protein 17-like isoform X1 | | |
| TRINITY_DN14642_c0_g1_i1_len_700_5729.p1_type_5prime_partial_len_145 | ---NA--- | | |
| TRINITY_DN23478_c0_g1_i1_len_3210_154126.p3_type_complete_len_145 | ---NA--- | | |
| TRINITY_DN5179_c0_g1_i1_len_555_303089.p1_type_5prime_partial_len_145 | ---NA--- | | |
| TRINITY_DN35816_c0_g1_i1_len_540_242011.p1_type_5prime_partial_len_145 | ---NA--- | | |
| TRINITY_DN19194_c0_g1_i1_len_875_197758.p1_type_3prime_partial_len_146 | ---NA--- | | |
| TRINITY_DN19038_c0_g1_i1_len_439_206382.p1_type_internal_len_146 | ---NA--- | | |
| TRINITY_DN26143_c0_g1_i1_len_1301_194255.p3_type_5prime_partial_len_145 | ---NA--- | | |
| TRINITY_DN38993_c0_g1_i1_len_438_206410.p1_type_internal_len_146 | ---NA--- | | |
| TRINITY_DN20707_c0_g1_i2_len_1300_194262.p3_type_complete_len_145 | ---NA--- | | |
| TRINITY_DN11852_c0_g1_i2_len_464_305053.p1_type_5prime_partial_len_145 | XP_022207311.1gastrula zinc finger protein XlCGF57.1-like | | |
| TRINITY_DN17581_c0_g1_i1_len_825_162170.p1_type_5prime_partial_len_145 | AEI25985.1thioredoxin | C:GO:0005623; P:GO:0006662; F:GO:0015035; P:GO:0045454; P:GO:0055114 | |
| TRINITY_DN29360_c0_g1_i1_len_755_238473.p1_type_5prime_partial_len_145 | ---NA--- | | |
| TRINITY_DN39664_c0_g1_i1_len_438_206423.p2_type_internal_len_146 | XP_018014662.1 teneurin-m-like | C:GO:0016020; C:GO:0016021 | |

| | | | |
|---|---|---|---|
| TRINITY_DN2954_c0_g1_i1_len_438_206421.p2_type_internal_len_146 | XP_018011575.1 hemocytin-like | | |
| TRINITY_DN10915_c0_g1_i1_len_438_206418.p1_type_internal_len_146 | XP_018013569.1PREDICTED: uncharacterized protein LOC108670608 | | |
| TRINITY_DN13727_c0_g1_i1_len_436_40318.p1_type_internal_len_146 | ---NA--- | | |
| TRINITY_DN17596_c0_g1_i2_len_1094_235416.p2_type_complete_len_145 | ---NA--- | | |
| TRINITY_DN21906_c0_g1_i1_len_436_40314.p1_type_internal_len_146 | XP_018011060.1 mucin-5AC-like | | |
| TRINITY_DN17206_c0_g1_i1_len_730_128668.p1_type_complete_len_145 | XP_791682.2 thioredoxin domain-containing protein 12 | C:GO:0005623; P:GO:0045454 | |
| TRINITY_DN30645_c0_g1_i1_len_471_304850.p1_type_5prime_partial_len_145 | ---NA--- | | |
| TRINITY_DN19998_c0_g1_i1_len_443_168652.p1_type_5prime_partial_len_145 | ---NA--- | | |
| TRINITY_DN34093_c0_g1_i1_len_439_206367.p1_type_internal_len_146 | XP_011684481.1 zinc finger MYM-type protein 3 isoform X2 | | |
| TRINITY_DN33260_c0_g1_i1_len_438_206403.p1_type_internal_len_146 | KZS16380.1Sodium-coupled neutral amino acid transporter 9 | C:GO:0016020 | |
| TRINITY_DN12809_c0_g1_i1_len_438_206416.p2_type_internal_len_146 | PNF18332.1hypothetical protein B7P43_G14500 | F:GO:0005543 | |
| TRINITY_DN4768_c0_g1_i1_len_435_206519.p1_type_internal_len_146 | ---NA--- | | |
| TRINITY_DN14777_c0_g1_i1_len_435_206520.p1_type_internal_len_146 | ---NA--- | | |
| TRINITY_DN1265_c0_g1_i1_len_577_241220.p1_type_3prime_partial_len_146 | ---NA--- | | |
| TRINITY_DN1212_c0_g1_i1_len_439_277692.p1_type_internal_len_146 | BAV56779.1immunoglobulin kappa light chain | | |
| TRINITY_DN2756_c0_g1_i1_len_435_206533.p1_type_internal_len_146 | ---NA--- | | |

| | | | |
|---|---|---|---|
| TRINITY_DN16282_c0_g1_i1_len_437_71006.p1_type_internal_len_146 | XP_018014555.1PREDICTED: uncharacterized protein LOC108671513 | | |
| TRINITY_DN22610_c0_g1_i3_len_959_236458.p2_type_complete_len_145 | ---NA--- | | |
| TRINITY_DN2734_c0_g1_i1_len_437_71010.p1_type_internal_len_146 | KFM79960.1DnaJ-like protein subfamily C member 16 | C:GO:0016020 | |
| TRINITY_DN1405_c0_g1_i1_len_438_70981.p1_type_internal_len_146 | ---NA--- | | |
| 1927\|_\|c3135_f3p4_2750_isoform_c3135_full_length_coverage_3_non_full_length_coverage_4_isoform_length_2750_21318 | ---NA--- | | |
| 1928\|_\|c34198_f2p1_2749_isoform_c34198_full_length_coverage_2_non_full_length_coverage_1_isoform_length_2749_23393 | ---NA--- | | |
| 1929\|_\|c4234_f2p2_2749_isoform_c4234_full_length_coverage_2_non_full_length_coverage_2_isoform_length_2749_28336 | ---NA--- | | |
| 1930\|_\|c1866_f2p7_2749_isoform_c1866_full_length_coverage_2_non_full_length_coverage_7_isoform_length_2749_25549 | ---NA--- | | |
| 1931\|_\|c8233_f2p12_2748_isoform_c8233_full_length_coverage_2_non_full_length_coverage_12_isoform_length_2748_25898 | ---NA--- | | |
| 1932\|_\|c2757_f3p1_2748_isoform_c2757_full_length_coverage_3_non_full_length_coverage_1_isoform_length_2748_21210 | XP_018027039.1 sec1 family domain-containing protein 1-like | | |
| 1935\|_\|c2367_f2p0_2746_isoform_c2367_full_length_coverage_2_non_full_length_coverage_0_isoform_length_2746_11812 | XP_015237809.1 gamma-tubulin complex component 6-like | | |
| 1936\|_\|c1615_f6p9_2746_isoform_c1615_full_length_coverage_6_non_full_length_coverage_9_isoform_length_2746_11423 | PNF42738.1Neutral alpha-glucosidase AB | F:GO:0016798 | |

| | | | |
|---|---|---|---|
| TRINITY_DN17720_c0_g1_i2_len_2964_264985.p1_type_complete_len_915 | AGN52246.1female sterile homeotic protein | | |
| 1939\|_\|c3715_f2p1_2745_isoform_c3715_full_length_coverage_2_non_full_length_coverage_1_isoform_length_2745_12321 | XP_018022210.1 pre-mRNA-splicing factor SYF1-like isoform X2 | | |
| TRINITY_DN39637_c0_g1_i1_len_435_206532.p1_type_internal_len_146 | ---NA--- | | |
| TRINITY_DN19899_c0_g1_i1_len_437_40283.p1_type_internal_len_146 | AUB30158.1fast-type skeletal muscle actin 13 | F:GO:0005524 | |
| TRINITY_DN29442_c0_g1_i1_len_437_244987.p1_type_internal_len_146 | XP_018016750.1 U8 snoRNA-decapping enzyme-like isoform X1 | | |
| TRINITY_DN36981_c0_g1_i1_len_436_206498.p1_type_internal_len_146 | ---NA--- | | |
| TRINITY_DN15484_c0_g1_i2_len_437_244977.p3_type_internal_len_146 | ---NA--- | | |
| TRINITY_DN3228_c0_g1_i1_len_436_245010.p1_type_internal_len_146 | XP_013776691.1E3 ubiquitin-protein ligase UBR2-like isoform X2 | | |
| TRINITY_DN11497_c0_g1_i2_len_437_10516.p1_type_internal_len_146 | ---NA--- | | |
| TRINITY_DN14755_c0_g1_i1_len_436_245009.p1_type_internal_len_146 | XP_023216113.1uncharacterized protein LOC111618774 | | |
| TRINITY_DN1613_c0_g1_i1_len_437_277740.p2_type_internal_len_146 | ---NA--- | | |
| TRINITY_DN30363_c0_g1_i1_len_437_277737.p3_type_internal_len_146 | ---NA--- | | |
| TRINITY_DN12994_c0_g1_i1_len_437_245001.p1_type_internal_len_146 | ---NA--- | | |
| TRINITY_DN10341_c0_g1_i2_len_437_244998.p1_type_internal_len_146 | ---NA--- | | |
| TRINITY_DN33329_c0_g1_i1_len_436_206496.p1_type_internal_len_146 | ---NA--- | | |
| TRINITY_DN31149_c0_g1_i1_len_439_244899.p1_type_internal_len_146 | ---NA--- | | |

| | | | |
|---|---|---|---|
| TRINITY_DN2006_c0_g1_i1_len_437_244975.p1_type_internal_len_146 | ---NA--- | | |
| TRINITY_DN33400_c0_g1_i1_len_437_244970.p1_type_internal_len_146 | XP_018007608.1 protein PTHB1-like isoform X2 | C:GO:0034464 | |
| TRINITY_DN16277_c0_g1_i2_len_558_165767.p1_type_5prime_partial_len_145 | AHH25647.1lipopolysaccharide-induced TNF-alpha factor | C:GO:0016021 | |
| TRINITY_DN14_c0_g1_i1_len_438_244944.p1_type_internal_len_146 | XP_018013703.1 ras-GEF domain-containing family member 1B-like isoform X4 | | |
| TRINITY_DN4254_c0_g1_i1_len_439_244916.p1_type_internal_len_146 | XP_019638930.1 protein FAM91A1-like | | |
| TRINITY_DN25833_c0_g1_i2_len_4723_187225.p3_type_complete_len_145 | ---NA--- | | |
| TRINITY_DN2703_c0_g1_i1_len_437_305789.p1_type_internal_len_146 | ---NA--- | | |
| TRINITY_DN2948_c0_g1_i1_len_528_203467.p1_type_3prime_partial_len_146 | ---NA--- | | |
| TRINITY_DN16268_c0_g1_i1_len_438_305752.p1_type_internal_len_146 | XP_021942559.1histone-lysine N-methyltransferase E(z) isoform X2 | F:GO:0008168; P:GO:0032259 | |
| TRINITY_DN8904_c0_g1_i1_len_435_305827.p1_type_internal_len_146 | ---NA--- | | |
| TRINITY_DN3175_c0_g1_i2_len_436_305807.p1_type_internal_len_146 | XP_018015395.1 8-oxo-dGDP phosphatase NUDT18-like | F:GO:0016787 | |
| TRINITY_DN18514_c0_g1_i1_len_975_33623.p2_type_5prime_partial_len_145 | ---NA--- | | |
| TRINITY_DN13739_c0_g1_i2_len_441_277625.p1_type_5prime_partial_len_145 | ---NA--- | | |
| TRINITY_DN8635_c0_g1_i2_len_442_102934.p1_type_5prime_partial_len_145 | XP_018016706.1 serine-rich adhesin for platelets-like | P:GO:0006796; F:GO:0016740 | |
| TRINITY_DN29398_c0_g1_i1_len_435_71064.p1_type_internal_len_146 | XP_015784166.1death-associated protein kinase 1 isoform X1 | | |
| TRINITY_DN14864_c0_g1_i1_len_508_203973.p1_type_3prime_partial_len_146 | AAB17008.1immunoglobulin kappa chain constant region | | |

| | | | |
|---|---|---|---|
| TRINITY_DN14938_c0_g1_i1_len_437_40275.p1_type_internal_len_146 | ---NA--- | | |
| TRINITY_DN12359_c1_g1_i1_len_522_166579.p1_type_5prime_partial_len_145 | ---NA--- | | |
| TRINITY_DN14308_c0_g1_i1_len_438_10469.p1_type_internal_len_146 | XP_018016004.1PREDICTED: uncharacterized protein LOC108672784 | | |
| TRINITY_DN32589_c0_g1_i1_len_436_168891.p1_type_internal_len_146 | ---NA--- | | |
| TRINITY_DN15999_c0_g1_i2_len_436_168892.p1_type_3prime_partial_len_146 | ---NA--- | | |
| TRINITY_DN27892_c0_g1_i1_len_436_168896.p1_type_internal_len_146 | ---NA--- | | |
| TRINITY_DN37760_c0_g1_i1_len_436_206472.p1_type_internal_len_146 | ---NA--- | | |
| TRINITY_DN5758_c0_g1_i1_len_438_10480.p1_type_internal_len_146 | ---NA--- | | |
| TRINITY_DN8175_c0_g1_i1_len_437_168846.p1_type_internal_len_146 | ---NA--- | | |
| TRINITY_DN30137_c0_g1_i1_len_467_243991.p1_type_5prime_partial_len_145 | ---NA--- | | |
| TRINITY_DN31125_c0_g1_i1_len_641_240098.p1_type_5prime_partial_len_145 | ---NA--- | | |
| TRINITY_DN23984_c0_g1_i1_len_436_103104.p1_type_internal_len_146 | AHX84170.1target of rapamycin | F:GO:0004674; F:GO:0005524; P:GO:0006468; F:GO:0044877 | EC:2.7.11 |
| TRINITY_DN5973_c0_g1_i1_len_438_168821.p1_type_internal_len_146 | ---NA--- | | |
| TRINITY_DN8349_c0_g1_i3_len_438_168834.p1_type_internal_len_146 | ---NA--- | | |
| TRINITY_DN6885_c0_g1_i1_len_438_168836.p1_type_internal_len_146 | ---NA--- | | |
| TRINITY_DN15903_c0_g1_i4_len_436_168887.p1_type_internal_len_146 | ---NA--- | | |

| | | | |
|---|---|---|---|
| TRINITY_DN10717_c0_g1_i1_len_533_203340.p1_type_5prime_partial_len_145 | ---NA--- | | |
| TRINITY_DN41931_c0_g1_i1_len_437_206465.p1_type_5prime_partial_len_145 | ---NA--- | | |
| TRINITY_DN31714_c0_g1_i1_len_436_206487.p1_type_internal_len_146 | ---NA--- | | |
| TRINITY_DN28895_c0_g1_i1_len_435_245075.p1_type_internal_len_146 | ---NA--- | | |
| TRINITY_DN3185_c0_g1_i1_len_437_10510.p1_type_internal_len_146 | XP_023715289.1CUE domain-containing protein 2 | | |
| TRINITY_DN1831_c0_g1_i1_len_436_245030.p1_type_internal_len_146 | ---NA--- | | |
| TRINITY_DN7140_c0_g1_i1_len_437_10515.p1_type_internal_len_146 | XP_018013573.1 protein msta-like | | |
| TRINITY_DN26458_c0_g1_i1_len_435_245046.p1_type_internal_len_146 | ---NA--- | | |
| TRINITY_DN2729_c0_g1_i1_len_437_277750.p1_type_internal_len_146 | ---NA--- | | |
| TRINITY_DN6837_c0_g1_i1_len_436_206480.p1_type_internal_len_146 | ADC42878.1serine proteinase inhibitor 7 | C:GO:0005615; P:GO:0006508; F:GO:0008233 | |
| TRINITY_DN31212_c0_g1_i1_len_502_101245.p1_type_5prime_partial_len_144 | AHH25647.1lipopolysaccharide-induced TNF-alpha factor | C:GO:0016021 | |
| TRINITY_DN28852_c0_g1_i1_len_2664_189102.p1_type_5prime_partial_len_144 | ---NA--- | | |
| TRINITY_DN37427_c0_g1_i1_len_436_206473.p1_type_internal_len_145 | ---NA--- | | |
| TRINITY_DN20608_c0_g1_i2_len_433_168983.p2_type_internal_len_145 | ---NA--- | | |
| TRINITY_DN10749_c0_g1_i1_len_956_196941.p1_type_complete_len_144 | ODM88837.1Cytochrome b5 | C:GO:0016021; F:GO:0020037; F:GO:0046872 | |
| TRINITY_DN17305_c0_g1_i1_len_657_6220.p1_type_3prime_partial_len_145 | ---NA--- | | |

| | | | |
|---|---|---|---|
| TRINITY_DN25138_c0_g1_i4_len_3225_228746.p2_type_complete_len_144 | XP_022111115.1DNA polymerase theta-like | F:GO:0003677; F:GO:0003887; P:GO:0006260; P:GO:0006261; P:GO:0071897 | |
| TRINITY_DN7428_c0_g1_i1_len_434_168953.p1_type_internal_len_145 | ---NA--- | | |
| TRINITY_DN18876_c0_g1_i1_len_771_97330.p2_type_complete_len_144 | ---NA--- | | |
| TRINITY_DN16574_c0_g1_i1_len_622_36654.p1_type_complete_len_144 | XP_014666851.1 PDZ domain-containing protein 11-like isoform X1 | | |
| TRINITY_DN26176_c0_g1_i3_len_519_203657.p1_type_5prime_partial_len_144 | ---NA--- | | |
| TRINITY_DN20898_c0_g1_i1_len_1898_266548.p2_type_complete_len_144 | ---NA--- | | |
| TRINITY_DN25760_c0_g1_i1_len_1951_191089.p1_type_5prime_partial_len_144 | ---NA--- | | |
| TRINITY_DN33969_c0_g1_i1_len_434_206561.p1_type_internal_len_145 | XP_022918477.1TOM1-like protein 2 isoform X2 | | |
| TRINITY_DN27583_c0_g1_i1_len_434_168961.p1_type_internal_len_145 | ---NA--- | | |
| TRINITY_DN12429_c0_g1_i1_len_433_206616.p1_type_internal_len_145 | XP_015605196.1E3 ubiquitin-protein ligase UBR2 isoform X1 | | |
| TRINITY_DN31580_c0_g1_i1_len_434_134133.p1_type_internal_len_145 | ---NA--- | | |
| TRINITY_DN37508_c0_g1_i1_len_433_206610.p1_type_internal_len_145 | XP_018016422.1 anoctamin-1-like | | |
| TRINITY_DN33523_c0_g1_i1_len_434_134141.p1_type_internal_len_145 | ---NA--- | | |
| TRINITY_DN21202_c0_g1_i1_len_1429_297007.p3_type_complete_len_144 | ---NA--- | | |
| TRINITY_DN23166_c0_g1_i1_len_873_197779.p1_type_complete_len_144 | ---NA--- | | |

| | | | |
|---|---|---|---|
| TRINITY_DN7402_c0_g1_i1_len_435_305826.p1_type_internal_len_145 | XP_018018138.1 serine/threonine-protein phosphatase 2A 56 kDa regulatory subunit epsilon isoform-like | C:GO:0000159; P:GO:0007165; F:GO:0019888; P:GO:0043666 | |
| TRINITY_DN17628_c0_g1_i4_len_434_103160.p3_type_internal_len_145 | ---NA--- | | |
| TRINITY_DN18453_c0_g1_i1_len_670_129341.p2_type_5prime_partial_len_144 | ---NA--- | | |
| TRINITY_DN37118_c0_g1_i1_len_433_206589.p1_type_internal_len_145 | ---NA--- | | |
| TRINITY_DN22285_c0_g1_i3_len_1348_233781.p2_type_complete_len_144 | ---NA--- | | |
| TRINITY_DN35614_c0_g1_i1_len_493_204460.p1_type_5prime_partial_len_144 | ---NA--- | | |
| TRINITY_DN10445_c0_g1_i1_len_434_206574.p1_type_internal_len_145 | XP_018018330.1 kelch-like protein diablo | | |
| TRINITY_DN23384_c0_g1_i1_len_434_206572.p1_type_internal_len_145 | XP_013772099.1collagen alpha-1(IV) chain-like | F:GO:0005201; C:GO:0005581 | |
| TRINITY_DN12876_c0_g1_i1_len_436_134092.p1_type_internal_len_145 | ---NA--- | | |
| TRINITY_DN8733_c0_g1_i1_len_435_103153.p1_type_internal_len_145 | XP_018007289.1 ATP-binding cassette sub-family G member 4-like | F:GO:0005524; F:GO:0015416; P:GO:0015716; P:GO:0015748; C:GO:0016021; P:GO:0099133 | EC:3.6.1.3; EC:3.6.1.15; EC:3.6.3.28 |
| TRINITY_DN9163_c0_g1_i1_len_592_37092.p1_type_5prime_partial_len_144 | ADM21460.1anti-lipopolysaccharide factor isoform 6 | | |
| TRINITY_DN19350_c0_g1_i1_len_689_129140.p1_type_complete_len_144 | ---NA--- | | |
| TRINITY_DN19646_c0_g1_i1_len_933_236681.p2_type_5prime_partial_len_144 | ---NA--- | | |
| TRINITY_DN10516_c0_g1_i1_len_475_205051.p1_type_5prime_partial_len_144 | XP_003726848.1 mitochondrial tRNA-specific 2-thiouridylase 1 isoform X1 | P:GO:0002143; C:GO:0005739 | |
| TRINITY_DN19577_c0_g1_i1_len_1026_95438.p3_type_5prime_partial_len_144 | ---NA--- | | |

<table>
<tr><td></td><td></td><td></td><td></td></tr>
<tr><td>TRINITY_DN10613_c0_g1_i1_len_1247_2454.p2_type_5prime_partial_len_144</td><td>---NA---</td><td></td><td></td></tr>
<tr><td>TRINITY_DN16737_c0_g1_i1_len_437_103083.p1_type_5prime_partial_len_144</td><td>---NA---</td><td></td><td></td></tr>
<tr><td>TRINITY_DN29672_c0_g1_i1_len_435_168916.p1_type_internal_len_145</td><td>XP_018012981.1 alpha-mannosidase 2x-like</td><td>F:GO:0004559; P:GO:0006013</td><td>EC:3.2.1.24</td></tr>
<tr><td>TRINITY_DN38335_c0_g1_i1_len_435_206534.p1_type_internal_len_145</td><td>---NA---</td><td></td><td></td></tr>
<tr><td>TRINITY_DN15689_c0_g1_i1_len_432_169004.p1_type_internal_len_145</td><td>---NA---</td><td></td><td></td></tr>
<tr><td>TRINITY_DN15995_c0_g1_i1_len_791_4922.p2_type_complete_len_144</td><td>XP_018023456.1 bifunctional 3'-phosphoadenosine 5'-phosphosulfate synthase-like isoform X2</td><td></td><td></td></tr>
<tr><td>TRINITY_DN15265_c0_g1_i1_len_1216_234599.p2_type_5prime_partial_len_144</td><td>---NA---</td><td></td><td></td></tr>
<tr><td>TRINITY_DN30469_c0_g1_i1_len_434_103170.p2_type_internal_len_145</td><td>---NA---</td><td></td><td></td></tr>
<tr><td>1941|_|c13770_f2p11_2744_isoform_c13770_full_length_coverage_2_non_full_length_coverage_11_isoform_length_2744_28634</td><td>XP_018018321.1 inositol monophosphatase 3-like isoform X1</td><td></td><td></td></tr>
<tr><td>1942|_|c2339_f2p0_2743_isoform_c2339_full_length_coverage_2_non_full_length_coverage_0_isoform_length_2743_20863</td><td>XP_018020400.1 protein catecholamines up-like</td><td></td><td></td></tr>
<tr><td>TRINITY_DN27301_c0_g1_i1_len_4411_187356.p1_type_5prime_partial_len_914</td><td>XP_021353376.1nuclear pore membrane glycoprotein 210-like isoform X2</td><td>C:GO:0016020; C:GO:0016021</td><td></td></tr>
<tr><td>1946|_|c15304_f10p5_2742_isoform_c15304_full_length_coverage_10_non_full_length_coverage_5_isoform_length_2742_22309</td><td>XP_018015721.1 solute carrier family 35 member F6-like isoform X2</td><td></td><td></td></tr>
<tr><td>1947|_|c1814_f7p3_2741_isoform_c1814_full_length_coverage_7_non_full_length_coverage_3_isoform_length_2741_11503</td><td>KYN34145.1Protein neuralized</td><td></td><td></td></tr>
</table>

| | | | |
|---|---|---|---|
| 1948\|_\|c7497_f2p4_2741_isoform_c7497_full_length_coverage_2_non_full_length_coverage_4_isoform_length_2741_12640 | XP_018009613.1 ferrochelatase, mitochondrial-like | | |
| 1949\|_\|c2512_f5p6_2741_isoform_c2512_full_length_coverage_5_non_full_length_coverage_6_isoform_length_2741_11928 | ---NA--- | | |
| TRINITY_DN20641_c0_g1_i1_len_3489_94.p1_type_complete_len_913 | XP_018023993.1 gamma-tubulin complex component 3-like | | |
| TRINITY_DN19026_c0_g1_i1_len_2740_29603.p1_type_internal_len_914 | XP_018018657.1 bifunctional glutamate/proline--tRNA ligase-like | | |
| TRINITY_DN23492_c1_g1_i1_len_2774_154602.p1_type_3prime_partial_len_914 | XP_022240688.1transducin beta-like protein 3 isoform X2 | P:GO:0006364; C:GO:0032040 | |
| TRINITY_DN41917_c0_g1_i1_len_434_206542.p1_type_internal_len_145 | ---NA--- | | |
| TRINITY_DN15723_c0_g1_i1_len_479_204926.p1_type_5prime_partial_len_144 | ---NA--- | | |
| TRINITY_DN31500_c0_g1_i1_len_434_206541.p1_type_internal_len_145 | ---NA--- | | |
| TRINITY_DN23593_c0_g1_i1_len_432_206653.p1_type_internal_len_145 | ---NA--- | | |
| TRINITY_DN15945_c0_g1_i2_len_435_206502.p1_type_internal_len_145 | XP_024942397.1beta-galactosidase-1-like protein 2 | | |
| TRINITY_DN1618_c0_g1_i1_len_435_206527.p1_type_internal_len_145 | XP_018026253.1 syntaxin-binding protein 5-like | | |
| TRINITY_DN5557_c0_g1_i1_len_432_10678.p1_type_internal_len_145 | AOR07218.1two pore potassium channel subfamily K member 1 | P:GO:0006813; C:GO:0016020 | |
| TRINITY_DN3709_c0_g1_i1_len_435_168933.p1_type_internal_len_145 | XP_018012872.1 histone deacetylase 4-like | | |
| TRINITY_DN23707_c0_g1_i3_len_436_206501.p1_type_internal_len_145 | ---NA--- | | |
| TRINITY_DN23066_c0_g1_i1_len_1831_231639.p4_type_complete_len_144 | ---NA--- | | |

| | | | |
|---|---|---|---|
| TRINITY_DN15002_c0_g1_i1_len_433_103192.p3_type_internal_len_145 | ---NA--- | | |
| TRINITY_DN160_c0_g1_i1_len_432_206636.p1_type_internal_len_145 | XP_018026562.1PREDICTED: uncharacterized protein LOC108681986, partial | | |
| TRINITY_DN11698_c0_g1_i1_len_432_134198.p1_type_internal_len_145 | AVN99023.1sortilin-related receptor-like | C:GO:0016020 | |
| TRINITY_DN15138_c0_g1_i1_len_432_134213.p1_type_internal_len_145 | AKQ22256.1CTLDcp1 | | |
| TRINITY_DN7217_c0_g1_i1_len_433_134183.p1_type_internal_len_145 | AAP59794.1slow muscle myosin S1 heavy chain | F:GO:0003774; C:GO:0016459 | EC:3.6.1.15 |
| TRINITY_DN40750_c0_g1_i1_len_432_206649.p1_type_3prime_partial_len_145 | ---NA--- | | |
| TRINITY_DN37610_c0_g1_i1_len_434_206543.p1_type_internal_len_145 | ---NA--- | | |
| TRINITY_DN21390_c0_g1_i1_len_851_237423.p1_type_3prime_partial_len_145 | ---NA--- | | |
| TRINITY_DN6953_c0_g1_i1_len_434_206545.p1_type_internal_len_145 | ---NA--- | | |
| TRINITY_DN15175_c0_g1_i1_len_435_103133.p1_type_internal_len_145 | XP_018006803.1 longitudinals lacking protein, isoforms H/M/V-like isoform X39 | | |
| TRINITY_DN8215_c0_g1_i1_len_448_39963.p1_type_3prime_partial_len_145 | ---NA--- | | |
| TRINITY_DN36830_c0_g1_i1_len_432_245190.p1_type_internal_len_145 | ---NA--- | | |
| TRINITY_DN20398_c0_g1_i1_len_1597_267405.p1_type_complete_len_144 | ---NA--- | | |
| TRINITY_DN22386_c0_g1_i1_len_1143_195357.p2_type_5prime_partial_len_144 | ---NA--- | | |
| TRINITY_DN26218_c0_g1_i1_len_7546_227494.p2_type_5prime_partial_len_144 | ---NA--- | | |
| TRINITY_DN2055_c0_g1_i1_len_500_304161.p1_type_5prime_partial_len_144 | ---NA--- | | |

| | | | |
|---|---|---|---|
| TRINITY_DN16589_c0_g1_i1_len_433_245123.p1_type_internal_len_145 | ---NA--- | | |
| TRINITY_DN14641_c0_g1_i2_len_433_245129.p1_type_internal_len_145 | ---NA--- | | |
| TRINITY_DN29552_c0_g1_i1_len_3317_188119.p2_type_3prime_partial_len_145 | ---NA--- | | |
| TRINITY_DN9547_c0_g1_i2_len_434_245109.p1_type_internal_len_145 | ---NA--- | | |
| TRINITY_DN27927_c0_g1_i1_len_434_245110.p1_type_internal_len_145 | ---NA--- | | |
| TRINITY_DN11393_c0_g1_i1_len_805_271706.p1_type_3prime_partial_len_145 | ---NA--- | | |
| TRINITY_DN5124_c0_g1_i1_len_433_71125.p1_type_internal_len_145 | ---NA--- | | |
| TRINITY_DN37323_c0_g1_i1_len_433_245151.p2_type_internal_len_145 | ---NA--- | | |
| TRINITY_DN16191_c0_g1_i1_len_433_245152.p1_type_internal_len_145 | KDR19041.1SID1 transmembrane family member 1 | C:GO:0016020; C:GO:0016021; P:GO:0033227; F:GO:0051033 | |
| TRINITY_DN5863_c0_g1_i1_len_432_245182.p1_type_internal_len_145 | ---NA--- | | |
| TRINITY_DN15027_c0_g1_i1_len_432_245187.p1_type_internal_len_145 | ---NA--- | | |
| TRINITY_DN13639_c0_g1_i1_len_433_245130.p1_type_internal_len_145 | ---NA--- | | |
| TRINITY_DN26741_c0_g1_i1_len_2257_190079.p2_type_complete_len_144 | ---NA--- | | |
| TRINITY_DN22903_c0_g1_i1_len_433_277886.p1_type_internal_len_145 | XP_021968317.1cartilage oligomeric matrix protein-like | F:GO:0005509; C:GO:0005576; P:GO:0007155 | |
| TRINITY_DN23265_c0_g1_i1_len_432_277903.p1_type_internal_len_145 | ---NA--- | | |
| TRINITY_DN26409_c0_g1_i1_len_537_37962.p1_type_3prime_partial_len_145 | XP_021912826.1voltage-dependent calcium channel subunit alpha-2/delta-3-like | | |

| | | | |
|---|---|---|---|
| TRINITY_DN17112_c0_g1_i2_len_1506_93323.p2_type_complete_len_144 | XP_018020604.1 serine/arginine-rich splicing factor 2-like | F:GO:0003723; P:GO:0043484 | |
| TRINITY_DN12071_c0_g1_i2_len_469_39418.p1_type_5prime_partial_len_144 | ---NA--- | | |
| TRINITY_DN6576_c0_g1_i1_len_559_202682.p1_type_5prime_partial_len_144 | ---NA--- | | |
| TRINITY_DN15007_c0_g1_i1_len_560_100110.p1_type_5prime_partial_len_144 | ---NA--- | | |
| TRINITY_DN3486_c0_g1_i1_len_438_244960.p1_type_5prime_partial_len_144 | XP_018011038.1 putative polypeptide N-acetylgalactosaminyltransferase 9 isoform X6 | | |
| TRINITY_DN18255_c0_g1_i1_len_2063_30294.p2_type_5prime_partial_len_144 | ---NA--- | | |
| TRINITY_DN31738_c0_g1_i1_len_462_205499.p1_type_5prime_partial_len_144 | ---NA--- | | |
| TRINITY_DN14693_c0_g2_i2_len_435_245050.p1_type_internal_len_145 | XP_018023552.1 membrane-bound transcription factor site-1 protease-like | | |
| TRINITY_DN22136_c0_g1_i9_len_940_95994.p1_type_5prime_partial_len_144 | ---NA--- | | |
| TRINITY_DN17040_c0_g1_i1_len_435_71052.p1_type_internal_len_145 | ---NA--- | | |
| TRINITY_DN32030_c0_g1_i1_len_434_245097.p1_type_internal_len_145 | XP_015929967.1type I inositol 3,4-bisphosphate 4-phosphatase-like | F:GO:0016316; P:GO:0036092 | |
| TRINITY_DN30960_c0_g1_i1_len_435_245063.p1_type_internal_len_145 | XP_018012650.1 fibrillin-2-like | F:GO:0005201; F:GO:0005509; C:GO:0062023 | |
| TRINITY_DN27899_c0_g1_i1_len_436_245003.p1_type_internal_len_145 | ---NA--- | | |
| TRINITY_DN20487_c0_g1_i2_len_896_63795.p2_type_3prime_partial_len_145 | ---NA--- | | |
| TRINITY_DN627_c0_g1_i1_len_480_243588.p1_type_5prime_partial_len_143 | XP_023346423.1transforming growth factor-beta-induced protein ig-h3-like | | |
| TRINITY_DN30247_c0_g1_i1_len_429_169126.p1_type_internal_len_144 | XP_018020121.1 vesicle-associated membrane protein 4-like | C:GO:0016020 | |

| | | | |
|---|---|---|---|
| TRINITY_DN9003_c0_g1_i1_len_611_240615.p1_type_complete_len_143 | XP_018012369.1 nuclear receptor 2C2-associated protein-like | | |
| TRINITY_DN28071_c0_g1_i1_len_429_103327.p1_type_internal_len_144 | ---NA--- | | |
| TRINITY_DN26383_c0_g1_i4_len_2308_230220.p3_type_5prime_partial_len_143 | ---NA--- | | |
| TRINITY_DN6106_c0_g1_i1_len_430_206742.p1_type_internal_len_144 | XP_018015723.1 copine-8-like | | |
| TRINITY_DN11792_c0_g1_i1_len_444_305554.p1_type_5prime_partial_len_143 | XP_023226835.1transmembrane protein 229B-like | | |
| TRINITY_DN5826_c0_g1_i1_len_667_200505.p2_type_5prime_partial_len_143 | ---NA--- | | |
| TRINITY_DN19603_c0_g1_i1_len_433_10640.p1_type_5prime_partial_len_143 | ---NA--- | | |
| TRINITY_DN3256_c0_g1_i2_len_468_243982.p1_type_5prime_partial_len_143 | CCP46946.1onychopsin | P:GO:0007165; C:GO:0016020; F:GO:0038023 | |
| TRINITY_DN5558_c0_g1_i1_len_430_206737.p1_type_internal_len_144 | ---NA--- | | |
| TRINITY_DN9546_c0_g1_i1_len_713_199791.p1_type_5prime_partial_len_143 | ATN38697.1cuticle-like protein | F:GO:0042302 | |
| TRINITY_DN21904_c0_g1_i1_len_1718_156968.p2_type_5prime_partial_len_143 | ---NA--- | | |
| TRINITY_DN31175_c0_g1_i1_len_430_169103.p1_type_internal_len_144 | ---NA--- | | |
| TRINITY_DN24567_c0_g1_i2_len_2083_230849.p2_type_complete_len_143 | XP_018013464.1 serine incorporator 1-like isoform X2 | C:GO:0016021 | |
| TRINITY_DN10530_c0_g1_i1_len_597_302404.p1_type_complete_len_143 | XP_022240874.1thioredoxin-like protein 4A | P:GO:0000398; C:GO:0046540 | |
| TRINITY_DN30016_c0_g1_i1_len_539_166199.p1_type_5prime_partial_len_143 | ---NA--- | | |
| TRINITY_DN3159_c0_g1_i1_len_431_169052.p1_type_internal_len_144 | ---NA--- | | |

| | | | |
|---|---|---|---|
| TRINITY_DN16123_c0_g1_i3_len_1646_157255.p3_type_complete_len_143 | OWF41566.1Nitrogen permease regulator 2-like protein | P:GO:0009267; P:GO:0010506 | |
| TRINITY_DN9525_c0_g1_i1_len_446_206093.p1_type_5prime_partial_len_143 | XP_018023119.1 zinc finger and BTB domain-containing protein 17-like isoform X1 | F:GO:0003676; C:GO:0005634 | |
| TRINITY_DN10182_c0_g1_i1_len_432_206642.p1_type_internal_len_144 | XP_018018127.1 F-box/LRR-repeat protein 7-like | | |
| TRINITY_DN27227_c0_g1_i3_len_1562_192748.p2_type_3prime_partial_len_144 | ---NA--- | | |
| TRINITY_DN33550_c0_g1_i1_len_432_206633.p1_type_internal_len_144 | XP_005031645.1extracellular matrix protein FRAS1-like | P:GO:0007154; C:GO:0016020; C:GO:0016021 | |
| TRINITY_DN21454_c0_g2_i1_len_558_100147.p1_type_5prime_partial_len_143 | ---NA--- | | |
| TRINITY_DN1133_c0_g1_i2_len_432_206643.p1_type_internal_len_144 | XP_022300217.1GRIP1-associated protein 1-like | | |
| TRINITY_DN5393_c0_g1_i2_len_432_103229.p1_type_internal_len_144 | ---NA--- | | |
| TRINITY_DN35278_c0_g1_i1_len_432_71148.p1_type_internal_len_144 | KFB51359.1homeodomain interacting protein kinase, isoform A | F:GO:0003677; F:GO:0004672; F:GO:0005524; P:GO:0006468 | |
| TRINITY_DN26147_c0_g1_i1_len_432_71133.p1_type_internal_len_144 | ---NA--- | | |
| TRINITY_DN11609_c0_g1_i1_len_430_305974.p1_type_internal_len_144 | ---NA--- | | |
| TRINITY_DN13940_c0_g1_i1_len_433_206615.p1_type_internal_len_144 | ---NA--- | | |
| TRINITY_DN29873_c0_g1_i1_len_431_305960.p1_type_internal_len_144 | XP_018007528.1PREDICTED: uncharacterized protein LOC108665301 | | |
| TRINITY_DN22612_c1_g1_i1_len_430_10709.p1_type_internal_len_144 | ---NA--- | | |
| TRINITY_DN7064_c0_g1_i1_len_431_206699.p1_type_internal_len_144 | ---NA--- | | |

| | | | |
|---|---|---|---|
| TRINITY_DN4935_c0_g1_i1_len_431_206700.p1_type_internal_len_144 | KZS14476.1Peripheral-type benzodiazepine receptor-associated 1-like protein | P:GO:0007274 | |
| TRINITY_DN19523_c0_g1_i1_len_558_241597.p1_type_5prime_partial_len_143 | ---NA--- | | |
| TRINITY_DN1873_c0_g1_i1_len_430_206705.p1_type_internal_len_144 | XP_018018204.1 ubiquitin carboxyl-terminal hydrolase 36-like | P:GO:0016579; F:GO:0036459 | |
| TRINITY_DN32565_c0_g1_i1_len_430_206706.p1_type_internal_len_144 | AHN53426.1reverse transcriptase/ribonuclease H | F:GO:0003676; F:GO:0003964; P:GO:0006278; P:GO:0015074 | |
| TRINITY_DN2572_c0_g1_i1_len_430_206707.p1_type_internal_len_144 | AKK75092.1tet methylcytosine dioxygenase | P:GO:0006211; P:GO:0055114; F:GO:0070579; P:GO:0080111 | EC:1.14.11 |
| TRINITY_DN33357_c0_g1_i1_len_431_206683.p1_type_internal_len_144 | ---NA--- | | |
| TRINITY_DN6239_c0_g1_i1_len_430_206709.p1_type_internal_len_144 | PSN43743.1Kinesin-like protein KIF13A | F:GO:0003777; C:GO:0005623; P:GO:0072383 | EC:3.6.1.15 |
| TRINITY_DN14787_c0_g1_i1_len_543_303333.p1_type_complete_len_143 | XP_021346951.1uncharacterized protein LOC110446237 | | |
| TRINITY_DN14470_c0_g1_i1_len_570_241385.p1_type_3prime_partial_len_144 | ---NA--- | | |
| TRINITY_DN41243_c0_g1_i1_len_431_206691.p1_type_internal_len_144 | XP_026548217.1lethal(2) giant larvae protein homolog 2-like | | |
| TRINITY_DN23302_c0_g1_i2_len_431_206695.p1_type_internal_len_144 | ---NA--- | | |
| 1954\|_\|c1979_f2p48_2739_isoform_c1979_full_length_coverage_2_non_full_length_coverage_48_isoform_length_2739_25633 | KDR14213.1Serine/threonine-protein phosphatase 6 regulatory subunit 3 | | |
| 1955\|_\|c12673_f2p14_2739_isoform_c12673_full_length_coverage_2_non_full_length_coverage_14_isoform_length_2739_13084 | XP_018024676.1 threonine--tRNA ligase, cytoplasmic-like | F:GO:0004829; F:GO:0005524; C:GO:0005737; P:GO:0006435 | EC:6.1.1.3 |
| 1956\|_\|c43274_f8p7_2739_isoform_c43274_full_length_coverage_8_non_full_length_coverage_7_isoform_length_2739_19221 | XP_018008518.1 26S proteasome non-ATPase regulatory subunit 1-like isoform X1 | C:GO:0000502; C:GO:0016021; F:GO:0030234; P:GO:0042176; P:GO:0050790 | |

| | | | |
|---|---|---|---|
| 1958\|_\|c2825_f3p4_2738_isoform_c2825_full_length_coverage_3_non_full_length_coverage_4_isoform_length_2738_12194 | ---NA--- | | |
| 1959\|_\|c2486_f2p0_2738_isoform_c2486_full_length_coverage_2_non_full_length_coverage_0_isoform_length_2738_20978 | XP_018012588.1 fat-like cadherin-related tumor suppressor homolog | | |
| 1962\|_\|c8788_f6p12_2737_isoform_c8788_full_length_coverage_6_non_full_length_coverage_12_isoform_length_2737_12748 | XP_018015307.1 GPI mannosyltransferase 1-like | C:GO:0005783; C:GO:0005789; P:GO:0006506; C:GO:0016020; C:GO:0016021; F:GO:0016740; F:GO:0016757; F:GO:0016758 | |
| TRINITY_DN14995_c0_g1_i1_len_4186_153625.p1_type_complete_len_912 | PSN55111.1hypothetical protein C0J52_01880 | P:GO:0034773; F:GO:0042799 | EC:2.1.1.43 |
| 1964\|_\|c2578_f11p2_2736_isoform_c2578_full_length_coverage_11_non_full_length_coverage_2_isoform_length_2736_21058 | XP_018013660.1 zinc finger CCHC domain-containing protein 8 homolog | | |
| 1965\|_\|c15266_f7p6_2736_isoform_c15266_full_length_coverage_7_non_full_length_coverage_6_isoform_length_2736_22297 | ---NA--- | | |
| 1966\|_\|c12121_f3p1_2736_isoform_c12121_full_length_coverage_3_non_full_length_coverage_1_isoform_length_2736_22108 | XP_018331519.1AP-1 complex subunit mu-1 | P:GO:0006886; P:GO:0016192; C:GO:0030131 | |
| TRINITY_DN41576_c0_g1_i1_len_431_206689.p1_type_internal_len_144 | ---NA--- | | |
| TRINITY_DN34081_c0_g1_i1_len_431_206688.p1_type_internal_len_144 | XP_018011029.1 serine/threonine-protein kinase tousled-like 2 | | |
| TRINITY_DN4431_c0_g1_i1_len_430_206712.p1_type_internal_len_144 | ---NA--- | | |
| TRINITY_DN24356_c1_g1_i1_len_1619_232458.p1_type_complete_len_143 | ---NA--- | | |
| TRINITY_DN9746_c0_g1_i1_len_429_10747.p1_type_internal_len_144 | ---NA--- | | |
| TRINITY_DN18530_c0_g1_i1_len_917_63649.p1_type_complete_len_143 | XP_018010621.1 trafficking protein particle complex subunit 2-like | C:GO:0005737; P:GO:0006888 | |

| | | | |
|---|---|---|---|
| TRINITY_DN23237_c0_g1_i2_len_2221_230456.p2_type_complete_len_143 | XP_018012208.1 diphosphomevalonate decarboxylase-like | F:GO:0004163; F:GO:0005524; C:GO:0005829; P:GO:0006695; P:GO:0019287 | EC:4.1.1.33 |
| TRINITY_DN4087_c0_g1_i1_len_429_10731.p1_type_internal_len_144 | ---NA--- | | |
| TRINITY_DN14444_c0_g1_i1_len_790_271861.p1_type_complete_len_143 | ACY66625.1eukaryotic translation elongation factor 1 epsilon 1 | C:GO:0016020 | |
| TRINITY_DN671_c0_g1_i1_len_431_206669.p1_type_internal_len_144 | ---NA--- | | |
| TRINITY_DN41140_c0_g1_i1_len_431_206666.p1_type_internal_len_144 | XP_018010798.1 SLIT and NTRK-like protein 4 | | |
| TRINITY_DN29532_c0_g1_i4_len_2345_189832.p2_type_complete_len_143 | XP_026724912.1G protein-activated inward rectifier potassium channel 3-like isoform X2 | P:GO:0006811 | |
| TRINITY_DN3301_c0_g1_i1_len_430_206710.p1_type_internal_len_144 | XP_022115707.1protein shifted isoform X1 | | |
| TRINITY_DN7219_c0_g1_i1_len_469_205250.p1_type_5prime_partial_len_143 | EFX86930.1GST-N-Metaxin-like protein | | |
| TRINITY_DN14_c0_g1_i1_len_456_277161.p1_type_5prime_partial_len_143 | ---NA--- | | |
| TRINITY_DN19878_c0_g1_i1_len_431_10691.p1_type_internal_len_144 | ---NA--- | | |
| TRINITY_DN12716_c0_g1_i1_len_430_103290.p1_type_internal_len_144 | ---NA--- | | |
| TRINITY_DN23328_c0_g1_i2_len_429_71260.p1_type_internal_len_144 | XP_018010987.1 pro-resilin-like | | |
| TRINITY_DN14643_c0_g1_i1_len_493_243220.p1_type_5prime_partial_len_143 | ---NA--- | | |
| TRINITY_DN31351_c0_g1_i1_len_430_103299.p1_type_internal_len_144 | ---NA--- | | |
| TRINITY_DN29717_c0_g1_i1_len_430_103303.p1_type_internal_len_144 | XP_023228024.1uncharacterized protein LOC111628460 isoform X1 | | |

| | | | |
|---|---|---|---|
| TRINITY_DN7723_c0_g1_i1_len_431_71181.p1_type_internal_len_144 | ---NA--- | | |
| TRINITY_DN33762_c0_g1_i1_len_431_134261.p1_type_internal_len_144 | ---NA--- | | |
| TRINITY_DN25568_c0_g1_i1_len_432_103246.p1_type_internal_len_144 | ---NA--- | | |
| TRINITY_DN12359_c0_g1_i1_len_433_134187.p1_type_internal_len_144 | XP_018007716.1 zeta-sarcoglycan-like | C:GO:0016012; C:GO:0016020; C:GO:0016021 | |
| TRINITY_DN33158_c0_g1_i1_len_430_103276.p2_type_internal_len_144 | ---NA--- | | |
| TRINITY_DN5340_c0_g1_i1_len_432_134208.p1_type_internal_len_144 | ---NA--- | | |
| TRINITY_DN29731_c0_g1_i1_len_431_71184.p1_type_internal_len_144 | ---NA--- | | |
| TRINITY_DN21728_c0_g1_i2_len_1186_297864.p2_type_5prime_partial_len_143 | ---NA--- | | |
| TRINITY_DN5268_c0_g1_i1_len_500_304171.p1_type_5prime_partial_len_143 | ---NA--- | | |
| TRINITY_DN10881_c0_g1_i3_len_430_277961.p1_type_internal_len_144 | ---NA--- | | |
| TRINITY_DN23794_c0_g1_i1_len_939_197105.p1_type_5prime_partial_len_143 | ---NA--- | | |
| TRINITY_DN11182_c0_g1_i1_len_484_101688.p1_type_5prime_partial_len_143 | ---NA--- | | |
| TRINITY_DN21272_c0_g1_i1_len_1851_231562.p1_type_5prime_partial_len_143 | ---NA--- | | |
| TRINITY_DN25565_c0_g1_i1_len_962_196877.p2_type_5prime_partial_len_143 | XP_025834688.1BLOC-1-related complex subunit 7 | | |
| TRINITY_DN9152_c0_g1_i1_len_1101_235362.p4_type_3prime_partial_len_144 | ---NA--- | | |
| TRINITY_DN19797_c1_g1_i1_len_952_161072.p2_type_3prime_partial_len_144 | ---NA--- | | |

| | | | |
|---|---|---|---|
| TRINITY_DN30244_c0_g1_i1_len_6040_186927.p2_type_3prime_partial_len_144 | ---NA--- | | |
| TRINITY_DN4650_c0_g1_i1_len_923_197258.p3_type_complete_len_143 | ---NA--- | | |
| TRINITY_DN12755_c0_g1_i1_len_592_165134.p1_type_5prime_partial_len_143 | XP_015437372.1 NF-kappa-B-repressing factor | F:GO:0003676 | |
| TRINITY_DN35040_c0_g1_i1_len_923_197252.p1_type_5prime_partial_len_143 | XP_018011322.1PREDICTED: uncharacterized protein LOC108668592 | | |
| TRINITY_DN4365_c0_g1_i1_len_431_277938.p1_type_internal_len_144 | ---NA--- | | |
| TRINITY_DN19839_c0_g1_i1_len_2327_155321.p2_type_5prime_partial_len_143 | XP_018017707.1 transcription factor mef2A-like | | |
| TRINITY_DN28385_c0_g1_i1_len_3065_188430.p2_type_complete_len_143 | ---NA--- | | |
| TRINITY_DN8176_c0_g1_i1_len_431_40466.p1_type_internal_len_144 | XP_018018731.1 heme-binding protein 2-like | | |
| TRINITY_DN12875_c0_g1_i1_len_493_204458.p1_type_5prime_partial_len_143 | ---NA--- | | |
| TRINITY_DN15265_c0_g1_i1_len_491_304374.p1_type_5prime_partial_len_143 | ---NA--- | | |
| TRINITY_DN12857_c0_g1_i1_len_539_203180.p1_type_5prime_partial_len_143 | ---NA--- | | |
| TRINITY_DN2625_c0_g1_i1_len_430_40499.p1_type_internal_len_144 | ---NA--- | | |
| TRINITY_DN12063_c0_g1_i1_len_430_40507.p1_type_internal_len_144 | ---NA--- | | |
| TRINITY_DN24981_c0_g1_i1_len_1175_234863.p1_type_complete_len_143 | XP_023609225.1tigger transposable element-derived protein 1 isoform X4 | | |
| TRINITY_DN23568_c0_g1_i1_len_850_198030.p1_type_5prime_partial_len_143 | PRD19005.1zinc finger protein | F:GO:0003676 | |
| TRINITY_DN8054_c0_g1_i1_len_429_245306.p1_type_internal_len_144 | ---NA--- | | |

| | | | |
|---|---|---|---|
| TRINITY_DN3657_c0_g1_i1_len_431_245211.p2_type_internal_len_144 | ---NA--- | | |
| TRINITY_DN12760_c0_g1_i1_len_465_205379.p1_type_5prime_partial_len_143 | ---NA--- | | |
| TRINITY_DN34762_c0_g1_i1_len_429_245295.p1_type_internal_len_144 | XP_018024624.1 type-2 ice-structuring protein-like | | |
| TRINITY_DN27609_c0_g1_i1_len_429_245294.p1_type_internal_len_144 | ---NA--- | | |
| TRINITY_DN9609_c0_g1_i1_len_429_245279.p1_type_internal_len_144 | XP_023342619.1E3 ubiquitin-protein ligase HERC2-like | | |
| TRINITY_DN3421_c0_g1_i1_len_432_245192.p1_type_internal_len_144 | ---NA--- | | |
| TRINITY_DN29292_c0_g1_i1_len_431_245207.p1_type_internal_len_144 | XP_018008623.1 Niemann-Pick C1 protein-like isoform X4 | | |
| TRINITY_DN32463_c0_g1_i1_len_431_245210.p1_type_internal_len_144 | KZS02949.1Multidrug resistance protein 4 | P:GO:0006811; F:GO:0022857; P:GO:0055085 | |
| TRINITY_DN30443_c0_g1_i1_len_430_245265.p1_type_internal_len_144 | ---NA--- | | |
| TRINITY_DN7246_c0_g1_i1_len_430_245243.p1_type_internal_len_144 | AFJ11391.1cytosolic phospholipase A2 | F:GO:0004623; C:GO:0005737; P:GO:0009395; F:GO:0046872; F:GO:0102567; F:GO:0102568 | EC:3.1.1.1; EC:3.1.1.4 |
| TRINITY_DN25920_c0_g1_i1_len_5167_227754.p4_type_complete_len_143 | ---NA--- | | |
| TRINITY_DN27550_c0_g1_i1_len_430_245260.p1_type_internal_len_144 | ---NA--- | | |
| TRINITY_DN12823_c0_g1_i1_len_430_245274.p1_type_internal_len_144 | ---NA--- | | |
| TRINITY_DN1073_c0_g1_i1_len_430_245268.p1_type_internal_len_144 | XP_018566746.1trafficking protein particle complex subunit 8 | | |
| TRINITY_DN17766_c0_g1_i1_len_740_97636.p2_type_5prime_partial_len_143 | XP_008473394.1cysteine sulfinic acid decarboxylase | F:GO:0016831; P:GO:0019752; F:GO:0030170 | |
| TRINITY_DN27303_c0_g1_i1_len_430_245267.p1_type_internal_len_144 | ---NA--- | | |

| | | | |
|---|---|---|---|
| TRINITY_DN3974_c0_g1_i1_len_433_277870.p1_type_internal_len_144 | XP_012266923.1UPF0183 protein CG7083 isoform X1 | | |
| TRINITY_DN1713_c0_g1_i1_len_632_240245.p1_type_5prime_partial_len_143 | ---NA--- | | |
| TRINITY_DN34822_c0_g1_i1_len_433_245147.p1_type_internal_len_144 | ACU31810.1alpha2 macroglobulin isoform 2 | F:GO:0004866; C:GO:0005615; P:GO:0010951 | |
| TRINITY_DN11458_c0_g1_i1_len_428_134369.p1_type_internal_len_143 | ---NA--- | | |
| TRINITY_DN23367_c0_g1_i1_len_735_199513.p1_type_5prime_partial_len_142 | XP_021956375.1partitioning defective 3 homolog isoform X1 | | |
| TRINITY_DN3664_c0_g1_i1_len_429_10736.p1_type_internal_len_143 | ---NA--- | | |
| TRINITY_DN22837_c0_g1_i1_len_858_127554.p1_type_complete_len_142 | XP_019878639.1 ribonuclease P/MRP protein subunit POP5-like | | |
| TRINITY_DN39274_c0_g1_i1_len_429_206771.p2_type_internal_len_143 | KDR07375.1hypothetical protein L798_03183 | P:GO:0005975; F:GO:0016810 | |
| TRINITY_DN3453_c0_g1_i1_len_1536_192913.p1_type_5prime_partial_len_142 | XP_015916379.1cAMP-specific 3',5'-cyclic phosphodiesterase | F:GO:0004114; P:GO:0007165 | EC:3.1.4.17 |
| TRINITY_DN32038_c0_g1_i1_len_632_201164.p1_type_5prime_partial_len_142 | ---NA--- | | |
| TRINITY_DN28248_c0_g1_i1_len_536_203263.p1_type_3prime_partial_len_143 | ---NA--- | | |
| TRINITY_DN15479_c0_g1_i1_len_742_5333.p2_type_5prime_partial_len_142 | ---NA--- | | |
| TRINITY_DN29851_c0_g1_i1_len_448_168494.p1_type_5prime_partial_len_142 | ADM45305.1Dmc1 | F:GO:0000150; F:GO:0003677; F:GO:0005524; C:GO:0005634; P:GO:0006281; P:GO:0007131; F:GO:0008094 | EC:3.6.1.3; EC:3.6.1.15 |
| TRINITY_DN17278_c0_g1_i1_len_428_206781.p1_type_internal_len_143 | XP_023335653.1general transcription factor 3C polypeptide 1-like isoform X2 | | |
| TRINITY_DN29979_c0_g1_i1_len_2105_190538.p3_type_complete_len_142 | ---NA--- | | |

| | | | |
|---|---|---|---|
| TRINITY_DN403_c0_g1_i1_len_429_206752.p1_type_internal_len_143 | XP_018020275.1 ATP-binding cassette sub-family D member 1-like | F:GO:0005524; C:GO:0005778; P:GO:0006635; C:GO:0016021; F:GO:0042626; P:GO:0055085 | EC:3.6.1.3; EC:3.6.1.15 |
| TRINITY_DN11467_c0_g1_i1_len_428_206782.p1_type_internal_len_143 | ---NA--- | | |
| TRINITY_DN24058_c0_g1_i1_len_1170_195167.p3_type_5prime_partial_len_142 | ---NA--- | | |
| TRINITY_DN28389_c0_g1_i1_len_428_169163.p1_type_internal_len_143 | XP_018012095.1 kinesin-like protein KIF12 | | |
| TRINITY_DN7180_c0_g1_i1_len_429_169128.p1_type_internal_len_143 | ---NA--- | | |
| TRINITY_DN21192_c0_g1_i1_len_2203_230501.p2_type_complete_len_142 | ---NA--- | | |
| TRINITY_DN24708_c0_g1_i1_len_428_169153.p1_type_internal_len_143 | XP_018014100.1 protein dopey-1-like | | |
| TRINITY_DN15640_c0_g1_i1_len_431_206693.p1_type_5prime_partial_len_142 | ---NA--- | | |
| TRINITY_DN18267_c0_g1_i1_len_624_273794.p1_type_5prime_partial_len_142 | AAZ29608.1C-type lectin | F:GO:0030246 | |
| TRINITY_DN26572_c0_g1_i1_len_427_169189.p1_type_internal_len_143 | ---NA--- | | |
| TRINITY_DN10127_c0_g1_i1_len_461_205517.p1_type_complete_len_142 | ---NA--- | | |
| TRINITY_DN20044_c0_g1_i2_len_1372_124729.p2_type_3prime_partial_len_143 | ALM25772.1cyclin G2 | | |
| TRINITY_DN1279_c0_g1_i1_len_427_10789.p1_type_internal_len_143 | ---NA--- | | |
| TRINITY_DN24857_c0_g1_i1_len_600_274144.p1_type_5prime_partial_len_142 | ---NA--- | | |
| TRINITY_DN15296_c0_g1_i1_len_428_10756.p1_type_internal_len_143 | XP_018027071.1PREDICTED: uncharacterized protein LOC108682421 isoform X2 | | |

| | | | |
|---|---|---|---|
| TRINITY_DN22135_c0_g1_i2_len_600_201790.p1_type_5prime_partial_len_142 | ---NA--- | | |
| TRINITY_DN29843_c0_g1_i1_len_2974_188582.p1_type_complete_len_912 | ---NA--- | | |
| TRINITY_DN19918_c0_g1_i1_len_3619_264621.p1_type_5prime_partial_len_912 | XP_001510722.1 TBC domain-containing protein kinase-like protein | | |
| TRINITY_DN28038_c0_g1_i3_len_3231_188207.p1_type_5prime_partial_len_912 | KDR17261.1Tetratricopeptide repeat protein 7B | | |
| TRINITY_DN29763_c0_g1_i1_len_3625_187832.p1_type_complete_len_912 | XP_023708153.1pyruvate dehydrogenase phosphatase regulatory subunit, mitochondrial isoform X1 | F:GO:0016491; P:GO:0055114 | |
| 1972\|_\|c17633_f7p4_2735_isoform_c17633_full_length_coverage_7_non_full_length_coverage_4_isoform_length_2735_17778 | ADK94870.1saposin isoform 1 | C:GO:0005764; P:GO:0006665 | |
| 1974\|_\|c7039_f2p6_2734_isoform_c7039_full_length_coverage_2_non_full_length_coverage_6_isoform_length_2734_21651 | ---NA--- | | |
| 1975\|_\|c7463_f2p5_2734_isoform_c7463_full_length_coverage_2_non_full_length_coverage_5_isoform_length_2734_21693 | XP_021000641.1zinc finger protein 271-like | F:GO:0003676 | |
| TRINITY_DN16559_c0_g1_i1_len_2929_91192.p1_type_complete_len_911 | XP_018009087.1 protein MCM10 homolog | F:GO:0003690; F:GO:0003697; C:GO:0005634; P:GO:0006260; P:GO:0006270 | |
| 1979\|_\|c2353_f2p1_2733_isoform_c2353_full_length_coverage_2_non_full_length_coverage_1_isoform_length_2733_16347 | XP_018017801.1 cytospin-A-like isoform X2 | | |
| 1980\|_\|c18056_f2p4_2733_isoform_c18056_full_length_coverage_2_non_full_length_coverage_4_isoform_length_2733_13541 | BAJ78981.1heat shock protein 40 | F:GO:0005524; P:GO:0006457; P:GO:0009408; F:GO:0031072; F:GO:0046872; F:GO:0051082 | |
| TRINITY_DN24472_c0_g1_i1_len_426_10828.p1_type_internal_len_143 | XP_019961702.1 BTB/POZ domain-containing protein 2 | | |
| TRINITY_DN14510_c0_g1_i1_len_446_244712.p1_type_5prime_partial_len_142 | ---NA--- | | |

| | | | |
|---|---|---|---|
| TRINITY_DN29887_c0_g1_i1_len_638_6443.p1_type_complete_len_142 | ---NA--- | | |
| TRINITY_DN26318_c0_g1_i1_len_427_10792.p1_type_internal_len_143 | ---NA--- | | |
| TRINITY_DN13939_c0_g1_i1_len_599_274157.p1_type_complete_len_142 | ---NA--- | | |
| TRINITY_DN2749_c0_g1_i1_len_429_245303.p1_type_internal_len_143 | ---NA--- | | |
| TRINITY_DN31576_c0_g1_i1_len_429_245300.p1_type_internal_len_143 | XP_018025851.1 neurofibromin-like | | |
| TRINITY_DN38192_c0_g1_i1_len_428_206794.p1_type_internal_len_143 | ---NA--- | | |
| TRINITY_DN16054_c0_g1_i1_len_573_99861.p1_type_5prime_partial_len_142 | ACO11196.1Hydroxyacyl-coenzyme A dehydrogenase, mitochondrial precursor | F:GO:0003857; P:GO:0006631; P:GO:0055114; F:GO:0070403 | EC:1.1.1.35 |
| TRINITY_DN37621_c0_g1_i1_len_427_206834.p1_type_internal_len_143 | ---NA--- | | |
| TRINITY_DN33426_c0_g1_i1_len_427_206824.p1_type_internal_len_143 | AJR21491.1RNA helicase | F:GO:0003723; F:GO:0032575; P:GO:0035194 | EC:3.6.1.3; EC:3.6.1.15 |
| TRINITY_DN3728_c0_g1_i1_len_428_206798.p1_type_internal_len_143 | XP_018007216.1 exostosin-3-like | | |
| TRINITY_DN36551_c0_g1_i1_len_427_206825.p1_type_internal_len_143 | ---NA--- | | |
| TRINITY_DN3445_c0_g1_i1_len_697_98123.p2_type_complete_len_142 | AGC96522.1heat shock protein 70 | F:GO:0005524 | |
| TRINITY_DN409_c0_g1_i1_len_428_245321.p1_type_internal_len_143 | ---NA--- | | |
| TRINITY_DN6370_c0_g1_i2_len_428_206796.p1_type_internal_len_143 | XP_018028241.1 calpain-5-like | F:GO:0004198; P:GO:0006508 | EC:3.4.22 |
| TRINITY_DN17308_c0_g1_i1_len_427_206841.p1_type_internal_len_143 | ---NA--- | | |
| TRINITY_DN11959_c0_g1_i1_len_544_68112.p2_type_5prime_partial_len_142 | XP_013784100.160S ribosomal protein L12-like | F:GO:0003735; C:GO:0005840; P:GO:0006412 | |

| | | | |
|---|---|---|---|
| TRINITY_DN28641_c0_g1_i1_len_429_277980.p1_type_internal_len_143 | XP_018007090.1PREDICTED: uncharacterized protein LOC108664903 isoform X2 | | |
| TRINITY_DN16145_c0_g1_i1_len_629_6556.p2_type_3prime_partial_len_143 | ---NA--- | | |
| TRINITY_DN16161_c0_g1_i1_len_430_206724.p2_type_internal_len_143 | ALH22586.1fibrinogen-related protein isoform 1 | | |
| TRINITY_DN2460_c0_g1_i1_len_430_206734.p1_type_internal_len_143 | ---NA--- | | |
| TRINITY_DN33027_c0_g1_i1_len_430_245277.p1_type_internal_len_143 | XP_018023901.1 histone-lysine N-methyltransferase ASH1L-like | F:GO:0005488; F:GO:0016740 | |
| TRINITY_DN41934_c0_g1_i1_len_428_206792.p1_type_internal_len_143 | XP_023223130.1KICSTOR complex protein SZT2-like | | |
| TRINITY_DN19566_c0_g1_i1_len_428_206804.p1_type_internal_len_143 | ---NA--- | | |
| TRINITY_DN6133_c0_g1_i1_len_428_206808.p1_type_internal_len_143 | ADQ00194.1smoothened receptor | P:GO:0007166; C:GO:0016020 | |
| TRINITY_DN37806_c0_g1_i1_len_427_245369.p1_type_internal_len_143 | ---NA--- | | |
| TRINITY_DN14847_c0_g1_i2_len_430_206728.p1_type_internal_len_143 | ---NA--- | | |
| TRINITY_DN36632_c0_g1_i1_len_427_245363.p1_type_internal_len_143 | ---NA--- | | |
| TRINITY_DN18654_c0_g1_i1_len_427_245370.p1_type_5prime_partial_len_142 | ---NA--- | | |
| TRINITY_DN19150_c0_g1_i1_len_427_245383.p1_type_internal_len_143 | ---NA--- | | |
| TRINITY_DN13380_c0_g1_i1_len_576_202294.p1_type_5prime_partial_len_142 | ---NA--- | | |
| TRINITY_DN21910_c1_g1_i1_len_974_3752.p3_type_5prime_partial_len_142 | ---NA--- | | |
| TRINITY_DN885_c0_g1_i1_len_426_245417.p1_type_internal_len_143 | EEB15163.1kif1, putative | F:GO:0003777; F:GO:0005524; P:GO:0007018; F:GO:0008017 | EC:3.6.1.15 |

| | | | |
|---|---|---|---|
| TRINITY_DN4354_c0_g1_i1_len_595_130451.p1_type_complete_len_142 | AEB00643.1glucose transporter 1 | P:GO:0006810; C:GO:0016020 | |
| TRINITY_DN26462_c0_g1_i1_len_428_245337.p1_type_internal_len_143 | ---NA--- | | |
| TRINITY_DN5641_c0_g1_i1_len_428_245349.p1_type_internal_len_143 | XP_025077319.1 probable ATP-dependent RNA helicase YTHDC2 | F:GO:0097159; F:GO:1901363 | |
| TRINITY_DN35011_c0_g1_i1_len_427_206821.p1_type_internal_len_143 | ---NA--- | | |
| TRINITY_DN33493_c0_g1_i1_len_477_69758.p1_type_5prime_partial_len_142 | ---NA--- | | |
| TRINITY_DN30980_c0_g1_i1_len_518_203699.p1_type_5prime_partial_len_142 | XP_018016875.1 beta-glucuronidase-like | | |
| TRINITY_DN31509_c0_g1_i1_len_428_206803.p1_type_internal_len_143 | AXR98466.1fibrillin 2 | F:GO:0005201; F:GO:0005509; C:GO:0062023 | |
| TRINITY_DN19645_c0_g1_i2_len_735_65252.p1_type_5prime_partial_len_142 | XP_018018467.1 larval cuticle protein LCP-22-like | | |
| TRINITY_DN34272_c0_g1_i1_len_428_206784.p1_type_internal_len_143 | ---NA--- | | |
| TRINITY_DN33603_c0_g1_i1_len_430_134294.p1_type_internal_len_143 | ---NA--- | | |
| TRINITY_DN5003_c0_g1_i1_len_458_244285.p1_type_5prime_partial_len_142 | ---NA--- | | |
| TRINITY_DN13784_c0_g1_i1_len_565_302922.p1_type_complete_len_142 | ---NA--- | | |
| TRINITY_DN6857_c0_g1_i1_len_428_134330.p1_type_internal_len_143 | ---NA--- | | |
| TRINITY_DN18114_c0_g1_i2_len_492_132560.p1_type_5prime_partial_len_142 | ALJ10568.1rhodanese 1-like protein | | |
| TRINITY_DN8161_c0_g1_i1_len_426_206859.p1_type_internal_len_143 | XP_021926182.1synaptotagmin-5-like isoform X2 | F:GO:0005544; P:GO:0006887; C:GO:0016021 | |
| TRINITY_DN4215_c0_g1_i1_len_429_278001.p1_type_internal_len_143 | ---NA--- | | |

| | | | |
|---|---|---|---|
| TRINITY_DN16565_c0_g1_i1_len_426_206867.p1_type_internal_len_143 | ---NA--- | | |
| TRINITY_DN15750_c0_g1_i1_len_643_66430.p1_type_5prime_partial_len_142 | ---NA--- | | |
| TRINITY_DN23114_c0_g1_i1_len_428_278023.p1_type_internal_len_143 | XP_018007087.1 chromodomain-helicase-DNA-binding protein 1-like | | |
| TRINITY_DN7505_c0_g1_i1_len_430_245250.p1_type_internal_len_143 | ---NA--- | | |
| TRINITY_DN28924_c0_g1_i1_len_430_245248.p1_type_internal_len_143 | ---NA--- | | |
| TRINITY_DN18214_c0_g1_i2_len_1603_192540.p2_type_3prime_partial_len_143 | ---NA--- | | |
| TRINITY_DN4667_c0_g1_i2_len_427_103385.p1_type_internal_len_143 | XP_013173613.1 glutamate-gated chloride channel isoform X5 | | |
| TRINITY_DN25246_c0_g1_i1_len_508_203989.p1_type_3prime_partial_len_143 | XP_019755462.1 glutamate-rich WD repeat-containing protein 1 | C:GO:0005634 | |
| TRINITY_DN20695_c0_g1_i1_len_429_71244.p1_type_internal_len_143 | ---NA--- | | |
| TRINITY_DN18119_c0_g1_i1_len_634_164472.p2_type_5prime_partial_len_142 | ANJ03341.1vascular endothelial growth factor 3 | | |
| TRINITY_DN7372_c0_g1_i1_len_585_241080.p1_type_5prime_partial_len_142 | XP_018019424.1 nephrin-like | | |
| TRINITY_DN30937_c0_g1_i1_len_430_103275.p1_type_internal_len_143 | XP_018013547.1 adenylate kinase isoenzyme 1-like | F:GO:0004017; F:GO:0005524; C:GO:0005737; P:GO:0016310; P:GO:0046034; P:GO:0046940 | EC:2.7.4.4; EC:2.7.4.3 |
| TRINITY_DN9963_c0_g1_i3_len_426_40653.p1_type_internal_len_143 | XP_018011122.1 troponin I-like isoform X6 | | |
| TRINITY_DN20692_c0_g1_i1_len_597_7043.p1_type_5prime_partial_len_142 | ---NA--- | | |
| TRINITY_DN19332_c0_g1_i2_len_776_238228.p1_type_complete_len_142 | ---NA--- | | |
| TRINITY_DN35362_c0_g1_i1_len_426_71371.p1_type_internal_len_143 | ---NA--- | | |

| | | | |
|---|---|---|---|
| TRINITY_DN13504_c0_g1_i1_len_426_71375.p1_type_internal_len_143 | ---NA--- | | |
| TRINITY_DN10001_c0_g1_i1_len_428_103356.p1_type_internal_len_143 | ---NA--- | | |
| TRINITY_DN23332_c0_g1_i1_len_430_103285.p1_type_5prime_partial_len_142 | XP_018008089.1 huntingtin-interacting protein 1-like | | |
| TRINITY_DN29579_c0_g1_i1_len_429_71261.p1_type_internal_len_143 | ---NA--- | | |
| TRINITY_DN11282_c0_g1_i1_len_427_71326.p1_type_internal_len_143 | ---NA--- | | |
| TRINITY_DN12580_c0_g1_i2_len_427_71331.p1_type_internal_len_143 | ---NA--- | | |
| TRINITY_DN35036_c0_g1_i1_len_427_71320.p1_type_internal_len_143 | ---NA--- | | |
| TRINITY_DN22484_c0_g1_i1_len_1687_232181.p1_type_complete_len_142 | XP_021346951.1uncharacterized protein LOC110446237 | F:GO:0005488 | |
| TRINITY_DN3246_c0_g1_i1_len_426_306113.p1_type_internal_len_143 | ---NA--- | | |
| TRINITY_DN10421_c0_g1_i1_len_602_164968.p2_type_3prime_partial_len_143 | ---NA--- | | |
| TRINITY_DN9377_c0_g1_i1_len_566_165592.p1_type_5prime_partial_len_142 | ---NA--- | | |
| TRINITY_DN11097_c0_g1_i1_len_638_201040.p1_type_5prime_partial_len_142 | ---NA--- | | |
| TRINITY_DN14834_c0_g1_i2_len_502_204188.p1_type_complete_len_142 | BBC20610.1hypothetical protein | | |
| TRINITY_DN30277_c1_g1_i1_len_6785_186831.p2_type_complete_len_142 | ---NA--- | | |
| TRINITY_DN18192_c0_g1_i2_len_458_305192.p1_type_5prime_partial_len_142 | ACG60902.1cathepsin C | P:GO:0006508; F:GO:0008234 | |
| TRINITY_DN4108_c0_g1_i1_len_439_206355.p1_type_3prime_partial_len_143 | ---NA--- | | |

| | | | |
|---|---|---|---|
| TRINITY_DN25019_c0_g1_i1_len_732_199554.p1_type_5prime_partial_len_142 | ---NA--- | | |
| TRINITY_DN26802_c0_g1_i1_len_424_245503.p1_type_internal_len_142 | APO14260.1juvenile hormone esterase-like carboxylesterase 2 | F:GO:0016787 | |
| TRINITY_DN17093_c0_g1_i1_len_425_206913.p2_type_internal_len_142 | ---NA--- | | |
| TRINITY_DN22194_c0_g1_i1_len_7069_227512.p3_type_3prime_partial_len_142 | XP_018022368.1 AT-rich interactive domain-containing protein 2-like isoform X2 | | |
| TRINITY_DN22021_c0_g1_i1_len_1272_2375.p3_type_complete_len_141 | ---NA--- | | |
| TRINITY_DN11925_c0_g1_i1_len_583_165309.p1_type_5prime_partial_len_141 | ---NA--- | | |
| TRINITY_DN13393_c0_g1_i1_len_945_161123.p1_type_complete_len_141 | ---NA--- | | |
| TRINITY_DN7878_c1_g1_i1_len_425_206908.p3_type_internal_len_142 | ---NA--- | | |
| TRINITY_DN17185_c0_g1_i5_len_423_103497.p1_type_internal_len_142 | ---NA--- | | |
| TRINITY_DN24354_c1_g1_i1_len_738_65221.p2_type_5prime_partial_len_141 | ---NA--- | | |
| TRINITY_DN1404_c0_g1_i1_len_426_245416.p1_type_internal_len_142 | KZS04237.1Collagen type IV alpha-3-binding protein | C:GO:0005794; C:GO:0005829; C:GO:0016020; P:GO:0035621; F:GO:0070273; P:GO:0120009; F:GO:1902387; F:GO:1902388; P:GO:1902389 | |
| TRINITY_DN5942_c0_g1_i1_len_530_166394.p2_type_5prime_partial_len_141 | XP_023705420.1ATP synthase-coupling factor 6, mitochondrial | C:GO:0000276; F:GO:0015078; P:GO:0015986 | |
| TRINITY_DN37335_c0_g1_i1_len_426_245421.p1_type_internal_len_142 | ---NA--- | | |
| TRINITY_DN37712_c0_g1_i1_len_425_245445.p1_type_internal_len_142 | XP_018024938.1 nucleolysin TIA-1-like isoform X3 | | |

| | | | |
|---|---|---|---|
| TRINITY_DN33566_c0_g1_i1_len_425_206930.p1_type_internal_len_142 | XP_018016072.1 active breakpoint cluster region-related protein-like | F:GO:0005096; P:GO:0007165; P:GO:0043547 | |
| TRINITY_DN6301_c0_g1_i1_len_426_245393.p1_type_internal_len_142 | XP_018016212.1 polycystic kidney disease protein 1-like 2 | | |
| TRINITY_DN25734_c0_g1_i1_len_426_278084.p1_type_internal_len_142 | XP_020845150.1myotubularin-related protein 9 | | |
| TRINITY_DN35167_c0_g1_i1_len_425_245451.p1_type_internal_len_142 | ---NA--- | | |
| 1982\|_\|c9364_f3p2_2733_isoform_c9364_full_length_coverage_3_non_full_length_coverage_2_isoform_length_2733_21858 | XP_018022019.1 calcium-binding mitochondrial carrier protein SCaMC-1-like isoform X3 | | |
| 1984\|_\|c2332_f2p0_2732_isoform_c2332_full_length_coverage_2_non_full_length_coverage_0_isoform_length_2732_20856 | ---NA--- | | |
| 1985\|_\|c2591_f2p5_2731_isoform_c2591_full_length_coverage_2_non_full_length_coverage_5_isoform_length_2731_16538 | XP_018015721.1 solute carrier family 35 member F6-like isoform X2 | | |
| TRINITY_DN21284_c0_g1_i1_len_2731_229381.p1_type_5prime_partial_len_910 | XP_019927129.1 rotatin isoform X2 | C:GO:0005813; C:GO:0036064; P:GO:0044782 | |
| 1987\|_\|c17924_f2p0_2730_isoform_c17924_full_length_coverage_2_non_full_length_coverage_0_isoform_length_2730_22570 | ---NA--- | | |
| 1988\|_\|c2098_f8p3_2730_isoform_c2098_full_length_coverage_8_non_full_length_coverage_3_isoform_length_2730_20704 | XP_023219758.1protein fem-1 homolog A-like | | |
| TRINITY_DN22397_c0_g1_i1_len_3087_228899.p1_type_complete_len_910 | AGZ84438.1G-protein signaling modulator | F:GO:0030695; P:GO:0050790 | |
| TRINITY_DN24269_c0_g1_i2_len_4332_228008.p1_type_5prime_partial_len_910 | XP_013382421.1DNA topoisomerase I, mitochondrial isoform X2 | F:GO:0003677; F:GO:0003917; C:GO:0005694; P:GO:0006265 | EC:5.99.1.2 |
| 1991\|_\|c26387_f2p4_2729_isoform_c26387_full_length_coverage_2_non_full_length_coverage_4_isoform_length_2729_23009 | ---NA--- | | |

| | | | |
|---|---|---|---|
| 1992\|_\|c18157_f3p1_2729_isoform_c18157_full_length_coverage_3_non_full_length_coverage_1_isoform_length_2729_17990 | XP_018027897.1 ubiquitin-like protein 4A | | |
| TRINITY_DN10167_c0_g1_i1_len_426_245399.p1_type_internal_len_142 | PSN36835.1Myotubularin-related protein 2 | | |
| TRINITY_DN32364_c0_g1_i1_len_426_245400.p1_type_internal_len_142 | XP_021927756.1CD109 antigen-like isoform X2 | | |
| TRINITY_DN8913_c0_g1_i1_len_426_245412.p1_type_internal_len_142 | KFM59313.1E3 ubiquitin-protein ligase MIB1 | F:GO:0004842; F:GO:0008270; P:GO:0016567; F:GO:0016874 | |
| TRINITY_DN18935_c0_g1_i1_len_1248_125220.p2_type_complete_len_141 | XP_018024032.1 protein arginine N-methyltransferase 6-like isoform X2 | | |
| TRINITY_DN4217_c0_g1_i1_len_425_245426.p1_type_internal_len_142 | XP_018027154.1PREDICTED: uncharacterized protein LOC108682497 | | |
| TRINITY_DN30356_c0_g1_i1_len_425_245436.p1_type_internal_len_142 | ---NA--- | | |
| TRINITY_DN30810_c0_g1_i1_len_426_278089.p1_type_internal_len_142 | ---NA--- | | |
| TRINITY_DN1579_c0_g1_i1_len_425_245441.p1_type_internal_len_142 | XP_018012344.1 dystonin-like isoform X2 | | |
| TRINITY_DN5300_c0_g1_i1_len_425_206933.p1_type_internal_len_142 | ---NA--- | | |
| TRINITY_DN14219_c0_g1_i1_len_441_10395.p1_type_5prime_partial_len_141 | ---NA--- | | |
| TRINITY_DN2867_c0_g1_i1_len_425_245443.p1_type_internal_len_142 | XP_019627295.1 multidrug resistance-associated protein 7-like isoform X2 | F:GO:0005524; C:GO:0016021; F:GO:0042626; P:GO:0055085 | EC:3.6.1.3; EC:3.6.1.15 |
| TRINITY_DN28879_c0_g1_i1_len_1605_192527.p2_type_5prime_partial_len_141 | PSN43440.1Pyridine nucleotide-disulfide oxidoreductase domain-containing protein 1 | F:GO:0003735; C:GO:0005840; P:GO:0006412; F:GO:0016491; P:GO:0055114 | |
| TRINITY_DN16544_c0_g1_i1_len_438_206411.p1_type_5prime_partial_len_141 | XP_018008076.1 sphingosine kinase 2-like isoform X4 | | |
| TRINITY_DN9298_c0_g1_i1_len_653_239896.p1_type_5prime_partial_len_141 | ---NA--- | | |

| | | | |
|---|---|---|---|
| TRINITY_DN8635_c0_g1_i1_len_424_245483.p1_type_internal_len_142 | AUW71485.1Rabconnectin-3A, partial | | |
| TRINITY_DN124_c0_g1_i1_len_424_245467.p1_type_internal_len_142 | ---NA--- | | |
| TRINITY_DN26534_c0_g1_i1_len_424_245464.p1_type_internal_len_142 | ---NA--- | | |
| TRINITY_DN2388_c0_g1_i1_len_425_245463.p1_type_internal_len_142 | XP_021938403.1PHD finger protein 19 isoform X1 | F:GO:0046872 | |
| TRINITY_DN5655_c0_g1_i1_len_423_103524.p1_type_internal_len_142 | ---NA--- | | |
| TRINITY_DN38179_c0_g1_i1_len_425_206923.p2_type_internal_len_142 | ---NA--- | | |
| TRINITY_DN12493_c0_g1_i1_len_600_240804.p1_type_5prime_partial_len_141 | ---NA--- | | |
| TRINITY_DN19055_c0_g1_i1_len_1026_235907.p1_type_complete_len_141 | ---NA--- | | |
| TRINITY_DN32958_c0_g1_i1_len_654_239883.p2_type_5prime_partial_len_141 | ---NA--- | | |
| TRINITY_DN5115_c0_g1_i1_len_427_245361.p1_type_internal_len_142 | ---NA--- | | |
| TRINITY_DN21558_c0_g2_i4_len_1057_62771.p1_type_complete_len_141 | XP_018010741.1 DNA-directed RNA polymerase II 16 kDa polypeptide-like | | |
| TRINITY_DN14515_c0_g1_i1_len_424_245470.p1_type_internal_len_142 | XP_017480264.1 potassium voltage-gated channel protein eag-like | F:GO:0005249; C:GO:0016021; P:GO:0034765; P:GO:0071805 | |
| TRINITY_DN38887_c0_g1_i1_len_427_206835.p1_type_internal_len_142 | ---NA--- | | |
| TRINITY_DN22789_c0_g1_i2_len_2383_230042.p5_type_complete_len_141 | ---NA--- | | |
| TRINITY_DN25378_c0_g1_i1_len_426_169242.p1_type_internal_len_142 | ---NA--- | | |
| TRINITY_DN25609_c0_g1_i1_len_424_169296.p1_type_internal_len_142 | AIE45536.1prophenoloxidase 2-like protein | F:GO:0016491; F:GO:0046872; P:GO:0055114 | |

| | | | |
|---|---|---|---|
| TRINITY_DN29419_c0_g1_i1_len_425_169275.p1_type_internal_len_142 | ---NA--- | | |
| TRINITY_DN9862_c1_g1_i1_len_555_165824.p1_type_3prime_partial_len_142 | ---NA--- | | |
| TRINITY_DN107_c0_g1_i1_len_557_274858.p1_type_5prime_partial_len_141 | ---NA--- | | |
| TRINITY_DN12979_c0_g1_i1_len_454_102537.p1_type_5prime_partial_len_141 | XP_018009697.1 DNA translocase FtsK-like | F:GO:0042302 | |
| TRINITY_DN25684_c0_g1_i1_len_712_239016.p1_type_5prime_partial_len_141 | ---NA--- | | |
| TRINITY_DN15909_c0_g1_i1_len_425_103436.p1_type_internal_len_142 | ---NA--- | | |
| TRINITY_DN39686_c0_g1_i1_len_426_206868.p1_type_internal_len_142 | XP_018011998.1 semaphorin-5A-like | | |
| TRINITY_DN5849_c0_g1_i1_len_425_103439.p1_type_internal_len_142 | XP_022188684.1probable E3 ubiquitin-protein ligase HERC4 | F:GO:0016874 | |
| TRINITY_DN28273_c0_g1_i1_len_461_102327.p1_type_5prime_partial_len_141 | XP_023232966.1WD repeat-containing protein 26-like | | |
| TRINITY_DN6861_c0_g1_i1_len_427_206839.p1_type_internal_len_142 | ---NA--- | | |
| TRINITY_DN23711_c0_g1_i1_len_2071_59188.p2_type_complete_len_141 | XP_015908111.1WD repeat-containing protein 24 isoform X3 | | |
| TRINITY_DN26362_c0_g1_i1_len_5498_227682.p4_type_complete_len_141 | ---NA--- | | |
| TRINITY_DN20703_c0_g1_i1_len_1482_124347.p2_type_complete_len_141 | ---NA--- | | |
| TRINITY_DN1547_c0_g1_i1_len_427_206840.p1_type_internal_len_142 | ---NA--- | | |
| TRINITY_DN23114_c0_g1_i5_len_1029_196310.p2_type_complete_len_141 | ---NA--- | | |
| TRINITY_DN15195_c0_g1_i1_len_423_169329.p2_type_internal_len_142 | ---NA--- | | |

| | | | |
|---|---|---|---|
| TRINITY_DN19662_c0_g1_i1_len_1787_266811.p1_type_5prime_partial_len_141 | ---NA--- | | |
| TRINITY_DN17322_c0_g1_i1_len_500_204255.p1_type_5prime_partial_len_141 | XP_017276034.1hemicentin-1 isoform X1 | | |
| TRINITY_DN19994_c0_g1_i1_len_1005_298784.p2_type_5prime_partial_len_141 | XP_013406597.1golgin subfamily A member 5 isoform X2 | C:GO:0016020 | |
| TRINITY_DN16028_c0_g1_i1_len_782_198840.p1_type_5prime_partial_len_141 | XP_022099413.1piggyBac transposable element-derived protein 4-like | | |
| TRINITY_DN24982_c0_g1_i1_len_423_169337.p1_type_internal_len_142 | XP_018011797.1 E3 ubiquitin-protein ligase HUWE1-like | P:GO:0000209; C:GO:0005634; C:GO:0005737; P:GO:0006511; C:GO:0016021; F:GO:0061630 | |
| TRINITY_DN20554_c0_g2_i2_len_697_129057.p1_type_5prime_partial_len_141 | ---NA--- | | |
| TRINITY_DN14585_c0_g1_i1_len_634_164479.p1_type_complete_len_141 | ---NA--- | | |
| TRINITY_DN30067_c0_g1_i1_len_1869_191401.p2_type_5prime_partial_len_141 | ---NA--- | | |
| TRINITY_DN6359_c0_g1_i1_len_425_206907.p1_type_internal_len_142 | XP_023709053.1mothers against decapentaplegic homolog 6 | P:GO:0006355 | |
| TRINITY_DN11946_c0_g1_i1_len_1121_235236.p1_type_3prime_partial_len_142 | ---NA--- | | |
| TRINITY_DN4563_c0_g1_i1_len_427_278036.p1_type_internal_len_142 | XP_023332368.1DNA topoisomerase 2-binding protein 1-A-like | | |
| TRINITY_DN37001_c0_g1_i1_len_425_206904.p1_type_internal_len_142 | ---NA--- | | |
| TRINITY_DN10871_c0_g1_i1_len_423_245530.p1_type_internal_len_142 | XP_018011489.1 heparan sulfate glucosamine 3-O-sulfotransferase 1-like | F:GO:0016740 | |
| TRINITY_DN14622_c0_g1_i1_len_834_299879.p1_type_3prime_partial_len_142 | ---NA--- | | |
| TRINITY_DN22287_c0_g1_i1_len_427_103399.p1_type_internal_len_142 | ---NA--- | | |
| TRINITY_DN5952_c0_g1_i1_len_426_206889.p1_type_internal_len_142 | ---NA--- | | |

| | | | |
|---|---|---|---|
| TRINITY_DN4885_c0_g1_i1_len_528_203472.p1_type_5prime_partial_len_141 | ---NA--- | | |
| TRINITY_DN673_c0_g1_i1_len_423_207014.p1_type_internal_len_142 | ---NA--- | | |
| TRINITY_DN19846_c0_g1_i1_len_913_63684.p1_type_5prime_partial_len_141 | ---NA--- | | |
| TRINITY_DN37346_c0_g1_i1_len_423_207003.p1_type_internal_len_142 | ---NA--- | | |
| TRINITY_DN2681_c0_g1_i1_len_423_207005.p1_type_internal_len_142 | PNF18256.1Bifunctional heparan sulfate N-deacetylase/N-sulfotransferase | F:GO:0008146; C:GO:0016020 | |
| TRINITY_DN17475_c0_g1_i1_len_450_205963.p1_type_5prime_partial_len_141 | XP_019879341.1 protein SERAC1-like | | |
| TRINITY_DN25199_c0_g1_i1_len_425_278125.p2_type_internal_len_142 | ATU31747.1Calcium-activated chloride channel regulator 2 | | |
| TRINITY_DN26996_c0_g1_i1_len_430_10711.p1_type_5prime_partial_len_141 | ---NA--- | | |
| TRINITY_DN3129_c0_g1_i1_len_436_71049.p1_type_5prime_partial_len_141 | ---NA--- | | |
| TRINITY_DN9199_c0_g1_i1_len_608_66957.p1_type_5prime_partial_len_141 | ---NA--- | | |
| TRINITY_DN13759_c0_g1_i1_len_423_206989.p1_type_internal_len_142 | ---NA--- | | |
| TRINITY_DN16385_c1_g1_i2_len_424_40722.p1_type_internal_len_142 | ---NA--- | | |
| TRINITY_DN11690_c0_g1_i1_len_425_134466.p1_type_internal_len_142 | ANA74997.1tubulin alpha-1A chain | F:GO:0003924; F:GO:0005200; F:GO:0005525; C:GO:0005874; P:GO:0007010; P:GO:0007017 | EC:3.6.1.15 |
| TRINITY_DN1529_c0_g1_i2_len_425_134486.p1_type_internal_len_142 | XP_026694932.1protein NATD1-like | | |
| TRINITY_DN17399_c0_g1_i1_len_462_205503.p1_type_5prime_partial_len_141 | ---NA--- | | |
| TRINITY_DN28920_c0_g1_i1_len_424_306174.p1_type_internal_len_142 | ---NA--- | | |

| | | | |
|---|---|---|---|
| TRINITY_DN22605_c0_g2_i1_len_1589_192614.p3_type_complete_len_141 | XP_013782708.2signal transducing adapter molecule 1-like | | |
| TRINITY_DN1698_c0_g1_i1_len_465_168022.p1_type_5prime_partial_len_141 | ---NA--- | | |
| TRINITY_DN14473_c0_g1_i1_len_763_128333.p1_type_5prime_partial_len_141 | ---NA--- | | |
| TRINITY_DN30659_c1_g1_i5_len_4549_187290.p2_type_complete_len_141 | ---NA--- | | |
| TRINITY_DN11976_c0_g1_i1_len_424_10896.p1_type_5prime_partial_len_141 | ---NA--- | | |
| TRINITY_DN22923_c0_g1_i1_len_1524_192970.p2_type_complete_len_141 | ---NA--- | | |
| TRINITY_DN17126_c0_g1_i1_len_814_237801.p1_type_5prime_partial_len_141 | ---NA--- | | |
| TRINITY_DN20535_c1_g1_i1_len_1236_159130.p3_type_3prime_partial_len_142 | ---NA--- | | |
| TRINITY_DN23513_c0_g2_i2_len_2209_230482.p2_type_5prime_partial_len_141 | XP_018006779.1 longitudinals lacking protein, isoforms A/B/D/L-like isoform X19 | | |
| TRINITY_DN27587_c0_g1_i2_len_3131_188335.p3_type_5prime_partial_len_141 | ---NA--- | | |
| TRINITY_DN20823_c1_g1_i1_len_425_306140.p1_type_internal_len_142 | ---NA--- | | |
| TRINITY_DN30634_c0_g1_i1_len_824_237694.p1_type_5prime_partial_len_141 | XP_022900636.1iron-sulfur cluster assembly 1 homolog, mitochondrial | F:GO:0005198; F:GO:0051536; P:GO:0097428 | |
| TRINITY_DN29794_c0_g1_i1_len_425_306157.p1_type_internal_len_142 | ---NA--- | | |
| TRINITY_DN995_c0_g1_i1_len_424_278165.p1_type_internal_len_142 | XP_021001385.1gastrula zinc finger protein XlCGF71.1-like | | |
| TRINITY_DN12104_c0_g1_i1_len_451_39866.p1_type_5prime_partial_len_141 | ---NA--- | | |
| TRINITY_DN6796_c0_g1_i1_len_424_206960.p2_type_internal_len_142 | XP_018019205.1 acyl-CoA-binding domain-containing protein 5-like | F:GO:0000062; C:GO:0016021 | |

<table>
<tr><td></td><td></td><td></td><td></td></tr>
<tr><td>TRINITY_DN20081_c0_g2_i1_len_515_242650.p1_type_5prime_partial_len_141</td><td>XP_022197570.1zinc finger protein 501-like</td><td></td><td></td></tr>
<tr><td>TRINITY_DN8506_c0_g1_i1_len_425_71388.p1_type_internal_len_142</td><td>---NA---</td><td></td><td></td></tr>
<tr><td>TRINITY_DN8294_c0_g1_i1_len_424_206964.p1_type_internal_len_142</td><td>---NA---</td><td></td><td></td></tr>
<tr><td>TRINITY_DN10714_c0_g1_i1_len_425_206939.p1_type_internal_len_142</td><td>---NA---</td><td></td><td></td></tr>
<tr><td>TRINITY_DN20052_c0_g1_i1_len_1057_269886.p1_type_5prime_partial_len_141</td><td>---NA---</td><td></td><td></td></tr>
<tr><td>TRINITY_DN6896_c0_g1_i1_len_425_278103.p1_type_internal_len_142</td><td>---NA---</td><td></td><td></td></tr>
<tr><td>1993|_|c2047_f3p5_2729_isoform_c2047_full_length_coverage_3_non_full_length_coverage_5_isoform_length_2729_16148</td><td>---NA---</td><td></td><td></td></tr>
<tr><td>1994|_|c2328_f7p3_2728_isoform_c2328_full_length_coverage_7_non_full_length_coverage_3_isoform_length_2728_20852</td><td>---NA---</td><td></td><td></td></tr>
<tr><td>1997|_|c5463_f5p4_2726_isoform_c5463_full_length_coverage_5_non_full_length_coverage_4_isoform_length_2726_21527</td><td>XP_018024844.1 trithorax group protein osa-like isoform X2</td><td>P:GO:0006338; C:GO:0070603</td><td></td></tr>
<tr><td>1998|_|c10973_f3p1_2725_isoform_c10973_full_length_coverage_3_non_full_length_coverage_1_isoform_length_2725_17410</td><td>---NA---</td><td></td><td></td></tr>
<tr><td>TRINITY_DN18414_c0_g1_i2_len_2801_265147.p1_type_complete_len_908</td><td>XP_023727495.1pyruvate dehydrogenase phosphatase regulatory subunit, mitochondrial-like isoform X2</td><td>F:GO:0016491; P:GO:0055114</td><td></td></tr>
<tr><td>2001|_|c10500_f2p1_2724_isoform_c10500_full_length_coverage_2_non_full_length_coverage_1_isoform_length_2724_17370</td><td>XP_018906501.1 tectonin beta-propeller repeat-containing protein</td><td></td><td></td></tr>
<tr><td>2002|_|c11737_f2p1_2724_isoform_c11737_full_length_coverage_2_non_full_length_coverage_1_isoform_length_2724_13007</td><td>XP_012610364.1cysteine desulfurase, mitochondrial isoform X1</td><td>C:GO:0005634; C:GO:0005759; P:GO:0018283; F:GO:0030170; F:GO:0031071; P:GO:0044571</td><td>EC:2.8.1.7</td></tr>
</table>

| | | | |
|---|---|---|---|
| 2003\|_\|c2204_f3p1_2723_isoform_c2204_full_length_coverage_3_non_full_length_coverage_1_isoform_length_2723_20777 | ---NA--- | | |
| 2005\|_\|c2065_f2p11_2722_isoform_c2065_full_length_coverage_2_non_full_length_coverage_11_isoform_length_2722_26969 | BAO20827.1DNA polymerase delta catalytic subunit | F:GO:0000166; F:GO:0003677; F:GO:0003887; C:GO:0005634; P:GO:0006260; F:GO:0046872; F:GO:0051539; P:GO:0071897 | EC:2.7.7.7 |
| 2006\|_\|c2678_f7p2_2722_isoform_c2678_full_length_coverage_7_non_full_length_coverage_2_isoform_length_2722_16609 | XP_013778368.22-oxoisovalerate dehydrogenase subunit beta, mitochondrial-like isoform X1 | F:GO:0003824 | |
| TRINITY_DN13345_c0_g1_i1_len_609_201591.p1_type_5prime_partial_len_141 | XP_018014829.1 phosphoglucomutase-2-like | | |
| TRINITY_DN40884_c0_g1_i1_len_424_206950.p1_type_internal_len_142 | ---NA--- | | |
| TRINITY_DN13684_c0_g1_i1_len_446_168566.p1_type_3prime_partial_len_142 | ---NA--- | | |
| TRINITY_DN23273_c0_g1_i8_len_2268_58846.p3_type_5prime_partial_len_141 | ---NA--- | | |
| TRINITY_DN11049_c0_g1_i1_len_424_206947.p1_type_internal_len_142 | ---NA--- | | |
| TRINITY_DN37153_c0_g1_i1_len_425_206937.p1_type_internal_len_142 | ---NA--- | | |
| TRINITY_DN15916_c0_g1_i1_len_1386_193735.p1_type_complete_len_141 | ---NA--- | | |
| TRINITY_DN24079_c0_g1_i2_len_1723_232055.p2_type_5prime_partial_len_141 | ---NA--- | | |
| TRINITY_DN25381_c0_g1_i1_len_425_40662.p1_type_internal_len_142 | OQR73050.1hypothetical protein BIW11_03661 | F:GO:0003676; F:GO:0008270 | |
| TRINITY_DN35595_c0_g1_i1_len_424_206973.p1_type_internal_len_142 | ---NA--- | | |
| TRINITY_DN1711_c0_g1_i1_len_424_40691.p1_type_internal_len_142 | XP_018015044.1PREDICTED: uncharacterized protein LOC108671954 | | |

| | | | |
|---|---|---|---|
| TRINITY_DN21938_c0_g1_i1_len_454_205803.p1_type_3prime_partial_len_142 | ---NA--- | | |
| TRINITY_DN8659_c0_g1_i1_len_426_40643.p1_type_internal_len_142 | CAA57964.1putative serine proteinase inhibitor | C:GO:0005615 | |
| TRINITY_DN30946_c1_g3_i1_len_611_201540.p1_type_5prime_partial_len_141 | ---NA--- | | |
| TRINITY_DN6123_c0_g1_i1_len_523_242452.p1_type_3prime_partial_len_142 | XP_011451816.1PREDICTED: uncharacterized protein LOC105345383 | | |
| TRINITY_DN3733_c0_g1_i1_len_424_206971.p1_type_internal_len_142 | ---NA--- | | |
| TRINITY_DN3082_c0_g1_i2_len_463_70160.p1_type_5prime_partial_len_141 | ACZ63471.1i-type lysozyme-like protein 1 | F:GO:0003796 | EC:3.2.1.17 |
| TRINITY_DN856_c0_g1_i1_len_424_206941.p1_type_internal_len_141 | ---NA--- | | |
| TRINITY_DN7861_c0_g1_i1_len_422_207058.p1_type_internal_len_141 | ---NA--- | | |
| TRINITY_DN5169_c0_g1_i1_len_483_204777.p1_type_5prime_partial_len_140 | AKF17681.1insulin-like receptor | F:GO:0004714; F:GO:0005524; C:GO:0005887; P:GO:0007169; P:GO:0018108; P:GO:0071469 | EC:2.7.10; EC:2.7.10.1; EC:2.7.11 |
| TRINITY_DN15342_c0_g1_i1_len_422_207063.p1_type_internal_len_141 | ---NA--- | | |
| TRINITY_DN37651_c0_g1_i1_len_422_207044.p1_type_internal_len_141 | ---NA--- | | |
| TRINITY_DN1521_c0_g1_i1_len_423_278191.p1_type_5prime_partial_len_140 | ---NA--- | | |
| TRINITY_DN36944_c0_g1_i1_len_423_245538.p1_type_internal_len_141 | ---NA--- | | |
| TRINITY_DN14114_c0_g1_i1_len_865_237283.p1_type_complete_len_140 | AGC24178.1inhibition of apoptosis protein | | |
| TRINITY_DN12593_c0_g1_i1_len_424_245504.p1_type_internal_len_141 | ---NA--- | | |
| TRINITY_DN25747_c0_g1_i1_len_1650_192313.p1_type_complete_len_140 | ---NA--- | | |

| | | | |
|---|---|---|---|
| TRINITY_DN32479_c0_g1_i1_len_424_245507.p1_type_internal_len_141 | ---NA--- | | |
| TRINITY_DN14388_c0_g1_i1_len_1516_267687.p2_type_complete_len_140 | ---NA--- | | |
| TRINITY_DN1866_c0_g1_i1_len_422_207072.p1_type_internal_len_141 | XP_021924784.1tryptophan 2,3-dioxygenase | F:GO:0005488; P:GO:0006569; F:GO:0051213 | |
| TRINITY_DN19330_c0_g1_i1_len_1158_32732.p1_type_complete_len_140 | AAL04108.1olfactory enriched transcipt 14.35 | F:GO:0030246 | |
| TRINITY_DN3197_c0_g1_i1_len_423_278204.p1_type_internal_len_141 | ---NA--- | | |
| TRINITY_DN9475_c0_g1_i1_len_876_237173.p2_type_complete_len_140 | ---NA--- | | |
| TRINITY_DN6023_c0_g1_i1_len_424_245491.p1_type_internal_len_141 | ---NA--- | | |
| TRINITY_DN37826_c0_g1_i1_len_422_207043.p1_type_internal_len_141 | ---NA--- | | |
| TRINITY_DN25612_c0_g1_i4_len_2591_229625.p2_type_complete_len_140 | OXA64138.1Programmed cell death protein 6 | F:GO:0005509 | |
| TRINITY_DN2568_c0_g1_i1_len_422_207069.p1_type_internal_len_141 | PSN45160.1Hydroxymethylglutaryl-CoA synthase 1 | F:GO:0004421; P:GO:0008299; P:GO:0016126 | EC:2.3.3.10 |
| TRINITY_DN20130_c0_g1_i1_len_1424_124550.p2_type_complete_len_140 | PIK40800.1putative hydroxypyruvate isomerase | F:GO:0016853 | |
| TRINITY_DN30140_c0_g1_i1_len_1669_192212.p2_type_complete_len_140 | ---NA--- | | |
| TRINITY_DN22233_c0_g1_i1_len_2751_229346.p3_type_complete_len_140 | ---NA--- | | |
| TRINITY_DN24676_c0_g1_i1_len_622_201354.p1_type_complete_len_140 | ---NA--- | | |
| TRINITY_DN20245_c0_g1_i1_len_1173_234877.p1_type_complete_len_140 | XP_018022823.1 glia maturation factor gamma-like | F:GO:0005488; P:GO:0032956 | |
| TRINITY_DN19628_c0_g1_i1_len_465_305009.p1_type_5prime_partial_len_140 | ---NA--- | | |

| | | | |
|---|---|---|---|
| TRINITY_DN21414_c0_g1_i1_len_716_199757.p1_type_5prime_partial_len_140 | ---NA--- | | |
| TRINITY_DN27503_c0_g1_i1_len_423_278177.p1_type_internal_len_141 | ---NA--- | | |
| TRINITY_DN25016_c0_g1_i1_len_422_103544.p1_type_internal_len_141 | XP_018902871.1 peroxidase-like | F:GO:0004601; P:GO:0006979; F:GO:0020037; C:GO:0042600; P:GO:0055114; P:GO:0098869 | |
| TRINITY_DN11086_c0_g1_i1_len_485_204706.p1_type_3prime_partial_len_141 | XP_013774992.1beta-1-syntrophin-like isoform X1 | F:GO:0005198; C:GO:0016010; P:GO:0050808 | |
| TRINITY_DN22837_c0_g1_i4_len_855_237374.p1_type_3prime_partial_len_141 | ---NA--- | | |
| TRINITY_DN800_c0_g1_i1_len_420_103609.p1_type_internal_len_141 | AFK65730.1myosin heavy chain | F:GO:0003774; C:GO:0016459 | EC:3.6.1.15 |
| TRINITY_DN18467_c0_g1_i1_len_423_206988.p2_type_internal_len_141 | ---NA--- | | |
| TRINITY_DN20577_c0_g1_i2_len_768_199017.p1_type_complete_len_140 | ---NA--- | | |
| TRINITY_DN34972_c0_g1_i1_len_422_207075.p1_type_internal_len_141 | ---NA--- | | |
| TRINITY_DN40758_c0_g1_i1_len_423_207000.p1_type_internal_len_141 | ---NA--- | | |
| TRINITY_DN25488_c0_g1_i1_len_3509_187922.p1_type_5prime_partial_len_140 | ---NA--- | | |
| TRINITY_DN40711_c0_g1_i1_len_421_207080.p1_type_internal_len_141 | ---NA--- | | |
| TRINITY_DN15300_c0_g1_i1_len_424_206981.p1_type_internal_len_141 | ---NA--- | | |
| TRINITY_DN22337_c0_g1_i2_len_3964_51.p4_type_complete_len_140 | ---NA--- | | |
| TRINITY_DN37676_c0_g1_i1_len_421_207078.p1_type_internal_len_141 | KFM62368.1Pecanex-like protein 1 | C:GO:0016020 | |
| TRINITY_DN13754_c0_g1_i1_len_468_276847.p1_type_5prime_partial_len_140 | XP_018014155.1 U6 snRNA-associated Sm-like protein LSm1 | | |

| | | | |
|---|---|---|---|
| TRINITY_DN31090_c0_g1_i3_len_1213_194860.p2_type_5prime_partial_len_140 | ---NA--- | | |
| TRINITY_DN16142_c0_g1_i2_len_1808_231709.p2_type_complete_len_140 | ---NA--- | | |
| TRINITY_DN3864_c0_g1_i1_len_421_207089.p1_type_internal_len_141 | ---NA--- | | |
| TRINITY_DN284_c0_g1_i1_len_421_207081.p1_type_internal_len_141 | ---NA--- | | |
| TRINITY_DN18693_c0_g1_i8_len_948_126885.p1_type_complete_len_140 | ---NA--- | | |
| TRINITY_DN4526_c0_g1_i2_len_424_206974.p1_type_internal_len_141 | ---NA--- | | |
| TRINITY_DN287_c1_g1_i1_len_422_245578.p2_type_internal_len_141 | XP_023017137.1synaptotagmin-like protein 5 | C:GO:0016020 | |
| TRINITY_DN25857_c0_g2_i1_len_422_245583.p1_type_internal_len_141 | ---NA--- | | |
| TRINITY_DN12729_c0_g1_i1_len_422_245588.p1_type_internal_len_141 | ---NA--- | | |
| TRINITY_DN36937_c0_g1_i1_len_422_245595.p1_type_internal_len_141 | CRK94795.1CLUMA_CG008289, isoform A | F:GO:0003677; F:GO:0042800; C:GO:0044666; F:GO:0046872; P:GO:0051568 | EC:2.1.1.43 |
| TRINITY_DN14331_c0_g1_i1_len_1132_94833.p1_type_5prime_partial_len_140 | ---NA--- | | |
| TRINITY_DN3282_c0_g1_i1_len_421_207082.p1_type_internal_len_141 | ---NA--- | | |
| TRINITY_DN21837_c0_g1_i1_len_931_197173.p1_type_complete_len_140 | ---NA--- | | |
| TRINITY_DN4962_c0_g1_i1_len_421_245614.p1_type_internal_len_141 | ---NA--- | | |
| TRINITY_DN31257_c0_g1_i10_len_3028_188494.p3_type_5prime_partial_len_140 | KGG50728.1hypothetical protein DI09_596p10, partial | | |
| TRINITY_DN24976_c0_g1_i1_len_421_103577.p1_type_internal_len_141 | ---NA--- | | |

| | | | |
|---|---|---|---|
| TRINITY_DN35101_c0_g1_i1_len_422_207041.p2_type_internal_len_141 | XP_022905330.1probable 3',5'-cyclic phosphodiesterase pde-5 | F:GO:0004118; P:GO:0010754; F:GO:0030552; F:GO:0046872 | EC:3.1.4.17 |
| TRINITY_DN26635_c0_g1_i1_len_422_245557.p1_type_internal_len_141 | XP_018027370.1PREDICTED: uncharacterized protein LOC108682669 | | |
| TRINITY_DN31786_c0_g1_i1_len_421_207094.p1_type_internal_len_141 | PNF31736.1Sialin | C:GO:0016020 | |
| TRINITY_DN29789_c0_g1_i1_len_652_66308.p1_type_5prime_partial_len_140 | ---NA--- | | |
| TRINITY_DN3146_c0_g1_i1_len_421_207083.p1_type_internal_len_141 | EFA02360.2putative multidrug resistance-associated protein lethal(2)03659-like Protein | F:GO:0000166; F:GO:0005524; C:GO:0016020; C:GO:0016021; F:GO:0016887; F:GO:0042626; P:GO:0055085 | |
| TRINITY_DN37951_c0_g1_i1_len_422_245573.p1_type_internal_len_141 | EFA00753.1N-acetylgalactosamine kinase-like Protein | F:GO:0004335; F:GO:0005524; F:GO:0005534; C:GO:0005829; P:GO:0033499; P:GO:0046835 | EC:2.7.1.6 |
| TRINITY_DN1719_c0_g1_i1_len_422_245565.p1_type_internal_len_141 | XP_006614227.1 protein split ends-like | F:GO:0003723 | |
| TRINITY_DN10317_c0_g1_i1_len_420_245645.p1_type_internal_len_141 | CDJ89140.1RNA-directed DNA polymerase (reverse transcriptase) domain containing protein | | |
| TRINITY_DN16786_c0_g1_i1_len_422_207033.p1_type_internal_len_141 | XP_023237523.1inositol hexakisphosphate and diphosphoinositol-pentakisphosphate kinase-like | F:GO:0000829; F:GO:0000832; F:GO:0005524; C:GO:0005829; P:GO:0016310; F:GO:0033857; F:GO:0052723; F:GO:0052724; F:GO:0102092 | EC:2.7.4.24; EC:2.7.4.21 |
| TRINITY_DN3478_c0_g1_i1_len_420_103586.p1_type_internal_len_141 | ---NA--- | | |
| TRINITY_DN22251_c0_g1_i2_len_1556_192787.p2_type_5prime_partial_len_140 | ---NA--- | | |
| TRINITY_DN18229_c0_g1_i1_len_1304_234022.p1_type_complete_len_140 | XP_012943245.1 craniofacial development protein 2-like | | |
| TRINITY_DN32580_c0_g1_i1_len_423_207019.p1_type_internal_len_141 | XP_018010174.1 proton-coupled folate transporter-like | | |

| | | | |
|---|---|---|---|
| TRINITY_DN17940_c0_g1_i1_len_566_67698.p1_type_3prime_partial_len_141 | ---NA--- | | |
| TRINITY_DN23035_c0_g4_i2_len_754_128411.p1_type_5prime_partial_len_140 | XP_018348421.1 cAMP-responsive element-binding protein-like 2 isoform X1 | | |
| TRINITY_DN6754_c0_g1_i1_len_422_207073.p1_type_internal_len_141 | ACP19563.1masquerade-like serine proteinase-like protein 3 | F:GO:0004252; P:GO:0006508 | EC:3.4.21 |
| TRINITY_DN21212_c0_g1_i1_len_2177_91855.p2_type_complete_len_140 | ---NA--- | | |
| TRINITY_DN6353_c0_g1_i1_len_422_40796.p1_type_internal_len_141 | ---NA--- | | |
| TRINITY_DN8647_c0_g1_i1_len_440_103000.p1_type_5prime_partial_len_140 | AQU12522.1cytochrome P450 | F:GO:0016491; F:GO:0046872 | |
| TRINITY_DN12037_c0_g1_i1_len_421_71537.p1_type_internal_len_141 | ---NA--- | | |
| TRINITY_DN29279_c0_g1_i1_len_462_244171.p1_type_5prime_partial_len_140 | ---NA--- | | |
| TRINITY_DN29293_c0_g1_i1_len_422_71495.p1_type_internal_len_141 | APF30033.1ionotropic glutamate receptor NMDA 2-like protein | F:GO:0004970; C:GO:0016021; C:GO:0030054; P:GO:0034220; P:GO:0035235; C:GO:0045211 | |
| TRINITY_DN7501_c0_g1_i1_len_422_71488.p1_type_internal_len_141 | ---NA--- | | |
| TRINITY_DN22384_c0_g1_i2_len_431_134253.p1_type_5prime_partial_len_140 | ---NA--- | | |
| TRINITY_DN12559_c0_g1_i1_len_525_275472.p1_type_3prime_partial_len_141 | EDO48894.1predicted protein | C:GO:0016020; C:GO:0016021; C:GO:0043231; P:GO:0055085 | |
| TRINITY_DN17820_c0_g1_i2_len_2931_91188.p1_type_5prime_partial_len_907 | ---NA--- | | |
| TRINITY_DN26148_c1_g1_i6_len_2884_229163.p1_type_complete_len_907 | ---NA--- | | |
| 2009\|_\|c17724_f3p2_2720_isoform_c17724_full_length_coverage_3_non_full_length_coverage_2_isoform_length_2720_22474 | XP_021938821.1tumor protein D54 isoform X5 | | |

| | | | |
|---|---|---|---|
| 2010\|_\|c28768_f2p2_2720_isoform_c28768_full_length_coverage_2_non_full_length_coverage_2_isoform_length_2720_18473 | XP_018018591.1 ubiquitin carboxyl-terminal hydrolase 1-like | F:GO:0004197; F:GO:0004843; P:GO:0006511; P:GO:0016579; F:GO:0036459 | |
| 2011\|_\|c1967_f4p3_2720_isoform_c1967_full_length_coverage_4_non_full_length_coverage_3_isoform_length_2720_20630 | XP_018020838.1 cell adhesion molecule 2-like isoform X1 | | |
| 2012\|_\|c18264_f2p0_2720_isoform_c18264_full_length_coverage_2_non_full_length_coverage_0_isoform_length_2720_18067 | XP_018007910.1 valine--tRNA ligase-like | P:GO:0006399; P:GO:0006412; F:GO:0016874; F:GO:0140101 | |
| 2013\|_\|c7767_f5p2_2720_isoform_c7767_full_length_coverage_5_non_full_length_coverage_2_isoform_length_2720_21722 | XP_021199457.1probable RNA-binding protein EIF1AD | F:GO:0003743; P:GO:0006413 | |
| 2015\|_\|c3033_f6p7_2719_isoform_c3033_full_length_coverage_6_non_full_length_coverage_7_isoform_length_2719_21308 | KZS17456.1Succinate dehydrogenase [ubiquinone] flavoprotein subunit, mitochondrial | C:GO:0005749; P:GO:0006099; P:GO:0006121; F:GO:0008177; F:GO:0009055; C:GO:0045282; F:GO:0050660 | EC:1.3.99.1; EC:1.3.5.1 |
| TRINITY_DN28192_c0_g1_i1_len_3501_187930.p1_type_complete_len_906 | XP_022235403.1tyrosine-protein kinase transmembrane receptor Ror2-like | | |
| TRINITY_DN21716_c0_g1_i1_len_3808_153756.p1_type_5prime_partial_len_906 | XP_018008474.1 protein transport protein Sec24C-like | | |
| TRINITY_DN24864_c0_g1_i1_len_422_40778.p2_type_internal_len_141 | ---NA--- | | |
| TRINITY_DN11245_c0_g1_i2_len_423_134571.p1_type_internal_len_141 | XP_006615783.1 transmembrane protein 8A-like isoform X4 | | |
| TRINITY_DN12075_c0_g1_i1_len_423_134550.p1_type_internal_len_141 | ---NA--- | | |
| TRINITY_DN26294_c0_g1_i1_len_423_134555.p1_type_internal_len_141 | ---NA--- | | |
| TRINITY_DN14601_c0_g1_i1_len_423_10943.p1_type_internal_len_141 | XP_018018445.1 heterogeneous nuclear ribonucleoprotein 27C-like isoform X2 | F:GO:0003723 | |
| TRINITY_DN9960_c0_g1_i1_len_1162_195225.p2_type_complete_len_140 | XP_018025173.1 tubulin glycylase 3A-like | | |

| | | | |
|---|---|---|---|
| TRINITY_DN23247_c0_g1_i1_len_525_38193.p1_type_5prime_partial_len_140 | ---NA--- | | |
| TRINITY_DN14337_c0_g1_i2_len_541_241993.p1_type_complete_len_140 | ---NA--- | | |
| TRINITY_DN21286_c0_g2_i1_len_1432_158124.p2_type_5prime_partial_len_140 | XP_018026438.1 ribonuclease H1-like | | |
| TRINITY_DN20172_c0_g1_i2_len_498_167100.p1_type_3prime_partial_len_141 | ---NA--- | | |
| TRINITY_DN17341_c0_g1_i1_len_783_97194.p1_type_3prime_partial_len_141 | ---NA--- | | |
| TRINITY_DN17656_c0_g1_i1_len_1163_195214.p2_type_complete_len_140 | ---NA--- | | |
| TRINITY_DN16197_c0_g1_i1_len_421_134636.p1_type_internal_len_141 | ---NA--- | | |
| TRINITY_DN9158_c0_g1_i2_len_517_242593.p1_type_3prime_partial_len_141 | AKM12664.1Janus kinase | F:GO:0004715; F:GO:0005524; C:GO:0005856; C:GO:0016020; P:GO:0018108; P:GO:0035556 | EC:2.7.10; EC:2.7.10.2 |
| TRINITY_DN27736_c0_g1_i2_len_429_206780.p1_type_5prime_partial_len_140 | XP_018014271.1 zinc finger SWIM domain-containing protein 7-like | F:GO:0008270 | |
| TRINITY_DN18887_c0_g1_i1_len_421_40816.p1_type_internal_len_141 | ---NA--- | | |
| TRINITY_DN26542_c0_g1_i1_len_1622_192446.p1_type_5prime_partial_len_140 | XP_022095908.1piggyBac transposable element-derived protein 4-like | | |
| TRINITY_DN18769_c0_g1_i1_len_436_10530.p1_type_5prime_partial_len_140 | ---NA--- | | |
| TRINITY_DN24474_c0_g1_i1_len_423_306226.p1_type_internal_len_141 | ---NA--- | | |
| TRINITY_DN9716_c0_g1_i1_len_570_165524.p1_type_3prime_partial_len_141 | ---NA--- | | |
| TRINITY_DN14462_c1_g1_i1_len_420_169457.p1_type_internal_len_141 | PRD22648.1zinc finger protein | F:GO:0003676 | |

| | | | |
|---|---|---|---|
| TRINITY_DN8541_c0_g1_i1_len_504_204099.p1_type_3prime_partial_len_141 | XP_013397824.1nascent polypeptide-associated complex subunit alpha, muscle-specific form | | |
| TRINITY_DN29833_c0_g1_i1_len_421_169419.p1_type_internal_len_141 | ---NA--- | | |
| TRINITY_DN25841_c0_g1_i1_len_422_306259.p1_type_internal_len_141 | ---NA--- | | |
| TRINITY_DN19111_c0_g1_i2_len_420_207135.p1_type_internal_len_141 | XP_018008051.1PREDICTED: uncharacterized protein LOC108665766 | | |
| TRINITY_DN17857_c0_g1_i1_len_1134_195423.p1_type_complete_len_140 | ---NA--- | | |
| TRINITY_DN22043_c0_g1_i2_len_1028_196329.p2_type_complete_len_140 | ---NA--- | | |
| TRINITY_DN124_c0_g1_i1_len_596_99475.p1_type_5prime_partial_len_140 | AEU11366.1Broad-complex protein isoform 4 | F:GO:0003676 | |
| TRINITY_DN33147_c0_g1_i1_len_421_207105.p1_type_internal_len_141 | EFX77641.1hypothetical protein DAPPUDRAFT_105878 | C:GO:0016021; P:GO:0055085 | |
| TRINITY_DN19475_c0_g1_i3_len_663_66142.p2_type_5prime_partial_len_140 | ---NA--- | | |
| TRINITY_DN30323_c0_g1_i1_len_3660_187800.p2_type_complete_len_140 | ---NA--- | | |
| TRINITY_DN10041_c0_g1_i1_len_452_277260.p1_type_5prime_partial_len_140 | ---NA--- | | |
| TRINITY_DN40734_c0_g1_i1_len_421_207112.p1_type_internal_len_141 | XP_025083893.1WD repeat domain phosphoinositide-interacting protein 3-like isoform X3 | | |
| TRINITY_DN24497_c0_g1_i1_len_498_132417.p1_type_5prime_partial_len_140 | ADE42874.1ADP-ribosylation factor | F:GO:0000166 | |
| TRINITY_DN10212_c0_g1_i1_len_645_240040.p1_type_5prime_partial_len_140 | ---NA--- | | |
| TRINITY_DN17545_c0_g1_i2_len_528_203458.p1_type_5prime_partial_len_140 | ---NA--- | | |

| | | | |
|---|---|---|---|
| TRINITY_DN32310_c0_g1_i1_len_643_240073.p1_type_complete_len_140 | ---NA--- | | |
| TRINITY_DN38960_c0_g1_i1_len_421_207109.p1_type_internal_len_141 | XP_018021559.1 ATP-binding cassette sub-family A member 5-like | F:GO:0005215; P:GO:0006810; F:GO:0016887 | EC:3.6.1.3; EC:3.6.1.15 |
| TRINITY_DN32303_c0_g1_i1_len_421_207110.p1_type_internal_len_141 | ---NA--- | | |
| TRINITY_DN5419_c0_g1_i2_len_641_240099.p1_type_3prime_partial_len_141 | ---NA--- | | |
| TRINITY_DN17186_c0_g1_i2_len_420_306304.p1_type_internal_len_141 | ---NA--- | | |
| TRINITY_DN16426_c0_g1_i3_len_434_10586.p1_type_5prime_partial_len_140 | ---NA--- | | |
| TRINITY_DN486_c0_g1_i1_len_420_207152.p1_type_internal_len_141 | XP_018025305.1 sodium/glucose cotransporter 4-like | C:GO:0016021; F:GO:0022857; P:GO:0055085 | |
| TRINITY_DN6741_c0_g1_i1_len_558_241608.p1_type_5prime_partial_len_140 | ---NA--- | | |
| TRINITY_DN32019_c0_g1_i1_len_655_98616.p2_type_5prime_partial_len_140 | ---NA--- | | |
| TRINITY_DN17422_c0_g2_i2_len_420_71576.p1_type_internal_len_141 | ---NA--- | | |
| TRINITY_DN27987_c0_g1_i1_len_650_129599.p1_type_5prime_partial_len_140 | XP_018028251.1 ubiquitin-like protein 3 | | |
| TRINITY_DN31756_c0_g1_i1_len_420_207158.p1_type_internal_len_141 | ---NA--- | | |
| TRINITY_DN25201_c0_g1_i1_len_910_197384.p1_type_complete_len_140 | AGA83300.1glutathione peroxidase | F:GO:0004602; P:GO:0006979; P:GO:0055114; P:GO:0098869 | EC:1.11.1.9; EC:1.11.1.7 |
| TRINITY_DN32619_c0_g1_i1_len_423_169316.p2_type_internal_len_141 | ---NA--- | | |
| TRINITY_DN4406_c0_g1_i1_len_420_207146.p1_type_internal_len_141 | ---NA--- | | |
| TRINITY_DN30995_c0_g1_i1_len_422_169344.p1_type_internal_len_141 | ---NA--- | | |

| | | | |
|---|---|---|---|
| TRINITY_DN25291_c0_g1_i1_len_421_169372.p1_type_internal_len_141 | ---NA--- | | |
| TRINITY_DN8528_c0_g1_i1_len_421_40810.p1_type_internal_len_141 | ---NA--- | | |
| TRINITY_DN29534_c0_g1_i1_len_2215_190191.p3_type_5prime_partial_len_140 | ---NA--- | | |
| TRINITY_DN21837_c0_g1_i5_len_421_169396.p1_type_internal_len_141 | ---NA--- | | |
| TRINITY_DN5156_c0_g1_i1_len_421_306273.p1_type_internal_len_141 | XP_022648313.1low-density lipoprotein receptor-related protein 4-like isoform X1 | | |
| TRINITY_DN15264_c0_g1_i2_len_424_169283.p1_type_5prime_partial_len_140 | ---NA--- | | |
| TRINITY_DN6822_c0_g1_i1_len_421_169386.p1_type_internal_len_141 | EKC33360.1Mitochondrial sodium/hydrogen exchanger NHA2 | P:GO:0006812; C:GO:0016020; P:GO:0055085 | |
| TRINITY_DN17386_c0_g1_i1_len_418_207246.p1_type_internal_len_140 | ---NA--- | | |
| TRINITY_DN11700_c0_g1_i1_len_418_207236.p1_type_internal_len_140 | ---NA--- | | |
| TRINITY_DN19574_c0_g1_i1_len_1521_192987.p1_type_complete_len_139 | ---NA--- | | |
| TRINITY_DN13656_c0_g1_i1_len_839_198152.p1_type_5prime_partial_len_139 | ---NA--- | | |
| TRINITY_DN34333_c0_g1_i1_len_417_207293.p1_type_internal_len_140 | XP_022830415.1protein embryonic gonad-like | F:GO:0003700; C:GO:0005634; P:GO:0006355; F:GO:0008270; F:GO:0043565 | |
| TRINITY_DN3375_c0_g1_i1_len_417_207273.p1_type_internal_len_140 | XP_026461564.1transmembrane protein adipocyte-associated 1 homolog isoform X2 | | |
| TRINITY_DN34378_c0_g1_i1_len_417_207290.p1_type_internal_len_140 | AEL23072.1phosphoenolpyruvate carboxykinase | F:GO:0004613; F:GO:0005525; P:GO:0006094; F:GO:0016301; P:GO:0016310 | EC:4.1.1.32 |
| TRINITY_DN30106_c0_g1_i1_len_2865_188755.p3_type_complete_len_139 | XP_004921714.1delta-1-pyrroline-5-carboxylate synthase isoform X1 | | |

| | | | |
|---|---|---|---|
| TRINITY_DN19173_c0_g1_i3_len_1245_297625.p1_type_complete_len_139 | KOC67994.1Protein phosphatase 1 regulatory subunit 14B | C:GO:0005737; P:GO:0042325 | |
| TRINITY_DN5412_c0_g1_i1_len_417_207297.p1_type_internal_len_140 | ---NA--- | | |
| TRINITY_DN10295_c0_g1_i1_len_418_207217.p1_type_internal_len_140 | AAT77812.1calpain T | F:GO:0004198; P:GO:0006508 | EC:3.4.22 |
| TRINITY_DN3276_c0_g1_i1_len_417_207262.p1_type_internal_len_140 | XP_018016096.1 irregular chiasm C-roughest protein-like | C:GO:0016020 | |
| TRINITY_DN19816_c0_g1_i2_len_1856_266648.p2_type_complete_len_139 | ---NA--- | | |
| TRINITY_DN1698_c0_g1_i1_len_418_40912.p1_type_internal_len_140 | ---NA--- | | |
| TRINITY_DN691_c0_g1_i1_len_418_207226.p1_type_internal_len_140 | ---NA--- | | |
| TRINITY_DN3237_c0_g1_i1_len_417_207266.p1_type_internal_len_140 | ---NA--- | | |
| TRINITY_DN6144_c0_g1_i1_len_419_207205.p1_type_internal_len_140 | XP_021927085.1spectrin beta chain, non-erythrocytic 1 | F:GO:0005543 | |
| TRINITY_DN17531_c0_g1_i2_len_1585_267433.p1_type_complete_len_139 | ---NA--- | | |
| TRINITY_DN32462_c0_g1_i1_len_420_207147.p1_type_internal_len_140 | ---NA--- | | |
| TRINITY_DN2991_c0_g1_i1_len_419_11064.p1_type_internal_len_140 | ---NA--- | | |
| TRINITY_DN18843_c0_g1_i2_len_616_130114.p1_type_complete_len_139 | BBC20610.1hypothetical protein | | |
| TRINITY_DN19509_c0_g1_i1_len_507_204018.p1_type_5prime_partial_len_139 | ---NA--- | | |
| TRINITY_DN6006_c0_g1_i1_len_818_127883.p1_type_complete_len_139 | ---NA--- | | |
| TRINITY_DN9696_c0_g1_i3_len_590_274284.p2_type_3prime_partial_len_140 | AFW99840.1troponin I | C:GO:0005861 | |

| | | | |
|---|---|---|---|
| TRINITY_DN26237_c0_g1_i1_len_419_11066.p1_type_internal_len_140 | ---NA--- | | |
| TRINITY_DN24161_c0_g1_i2_len_835_198206.p2_type_complete_len_139 | XP_018006956.1 U7 snRNA-associated Sm-like protein LSm10 | | |
| TRINITY_DN9579_c0_g1_i1_len_420_207156.p1_type_internal_len_140 | EEN50887.1hypothetical protein BRAFLDRAFT_86044 | F:GO:0003676 | |
| TRINITY_DN14997_c0_g1_i1_len_417_11117.p1_type_internal_len_140 | ACO11196.1Hydroxyacyl-coenzyme A dehydrogenase, mitochondrial precursor | F:GO:0003857; P:GO:0006631; P:GO:0055114; F:GO:0070403 | EC:1.1.1.35 |
| TRINITY_DN9518_c0_g1_i1_len_501_204230.p1_type_5prime_partial_len_139 | ---NA--- | | |
| TRINITY_DN12301_c0_g1_i3_len_501_204211.p1_type_5prime_partial_len_139 | ---NA--- | | |
| TRINITY_DN6871_c0_g1_i1_len_421_207108.p1_type_internal_len_140 | XP_020898339.1uncharacterized protein LOC110237103 | | |
| TRINITY_DN27158_c0_g1_i1_len_1752_191851.p2_type_complete_len_139 | ---NA--- | | |
| TRINITY_DN13423_c0_g1_i1_len_420_207142.p1_type_internal_len_140 | XP_025424441.1zinc finger MYM-type protein 1-like | | |
| TRINITY_DN9211_c0_g1_i1_len_503_204140.p1_type_3prime_partial_len_140 | ---NA--- | | |
| TRINITY_DN9134_c0_g1_i2_len_606_130273.p1_type_complete_len_139 | ---NA--- | | |
| TRINITY_DN6129_c0_g1_i1_len_420_207131.p1_type_internal_len_140 | ---NA--- | | |
| TRINITY_DN29957_c0_g1_i1_len_4223_187470.p3_type_complete_len_139 | ---NA--- | | |
| TRINITY_DN6150_c0_g1_i1_len_420_11020.p1_type_internal_len_140 | PSN55872.1Laminin subunit beta-1 | | |
| TRINITY_DN32037_c0_g1_i1_len_419_207189.p1_type_internal_len_140 | PIK35125.1hypothetical protein BSL78_28051 | F:GO:0003676; P:GO:0015074 | |
| TRINITY_DN3989_c0_g1_i1_len_419_207178.p1_type_internal_len_140 | XP_018022039.1 midasin-like | | |

| | | | |
|---|---|---|---|
| TRINITY_DN20293_c1_g1_i1_len_572_99889.p1_type_5prime_partial_len_139 | ---NA--- | | |
| TRINITY_DN30807_c0_g1_i1_len_4772_187209.p1_type_internal_len_1591 | XP_018016854.1 mucin-17-like | | |
| TRINITY_DN23996_c0_g1_i1_len_5028_227777.p1_type_complete_len_1590 | KDR16784.1putative methyltransferase TARBP1 | P:GO:0001510; F:GO:0003723; P:GO:0006396; F:GO:0008168; F:GO:0008173; F:GO:0016740; P:GO:0032259 | |
| TRINITY_DN23347_c0_g1_i1_len_4892_227816.p1_type_5prime_partial_len_1589 | XP_018023279.1 receptor-type tyrosine-protein phosphatase alpha-like | | |
| TRINITY_DN26357_c0_g1_i2_len_5993_227613.p1_type_complete_len_1584 | XP_012339244.1PREDICTED: uncharacterized protein LOC100871926 | | |
| TRINITY_DN26304_c0_g1_i1_len_7700_186780.p1_type_complete_len_1578 | XP_018015537.1 STE20-like serine/threonine-protein kinase isoform X2 | | |
| TRINITY_DN30315_c0_g1_i1_len_5348_187047.p1_type_complete_len_1575 | XP_003248963.1 zinc finger protein 62 homolog | F:GO:0003676 | |
| TRINITY_DN30455_c0_g1_i1_len_4806_187194.p1_type_5prime_partial_len_1569 | XP_018006409.1 histone-lysine N-methyltransferase 2D-like | | |
| 219\|_\|c14814_f2p8_4706_isoform_c14814_full_length_coverage_2_non_full_length_coverage_8_isoform_length_4706_28661 | XP_018019275.1 NAD-dependent protein deacetylase sirtuin-1-like | F:GO:0002039; F:GO:0003714; C:GO:0005637; C:GO:0005654; C:GO:0005719; C:GO:0005720; F:GO:0008022; P:GO:0016575; F:GO:0019904; C:GO:0033553; F:GO:0035257; F:GO:0043425; F:GO:0046969; F:GO:0051019; F:GO:0070403 | EC:3.5.1.98 |
| TRINITY_DN29947_c0_g2_i2_len_5071_187106.p1_type_complete_len_1568 | XP_023933273.1KAT8 regulatory NSL complex subunit 3 | | |
| TRINITY_DN27765_c0_g1_i1_len_5362_187042.p1_type_5prime_partial_len_1563 | XP_023222250.1spectrin beta chain, non-erythrocytic 1-like | | |
| TRINITY_DN29215_c0_g1_i1_len_2949_188619.p1_type_5prime_partial_len_906 | XP_018903748.1PREDICTED: uncharacterized protein LOC109034831 | | |

| | | | |
|---|---|---|---|
| TRINITY_DN26232_c2_g1_i1_len_4387_227988.p1_type_complete_len_906 | ---NA--- | | |
| 2021\|_\|c2322_f3p7_2717_isoform_c2322_full_length_coverage_3_non_full_length_coverage_7_isoform_length_2717_16323 | ---NA--- | | |
| 2022\|_\|c18772_f2p0_2716_isoform_c18772_full_length_coverage_2_non_full_length_coverage_0_isoform_length_2716_18109 | ---NA--- | | |
| TRINITY_DN23586_c0_g1_i1_len_3472_153934.p1_type_complete_len_905 | XP_016370761.1 gastrula zinc finger protein XlCGF57.1-like | | |
| 2025\|_\|c1855_f14p10_2715_isoform_c1855_full_length_coverage_14_non_full_length_coverage_10_isoform_length_2715_11516 | XP_018011435.1 probable acyl-CoA dehydrogenase fadE25 | F:GO:0016491; F:GO:0016627; F:GO:0050660; P:GO:0055114 | |
| 2026\|_\|c18013_f2p2_2715_isoform_c18013_full_length_coverage_2_non_full_length_coverage_2_isoform_length_2715_17895 | XP_014674932.1 cell division cycle protein 27 homolog | P:GO:0051301 | |
| 2027\|_\|c7103_f2p3_2715_isoform_c7103_full_length_coverage_2_non_full_length_coverage_3_isoform_length_2715_12600 | XP_018026228.1 protein hu-li tai shao-like | | |
| TRINITY_DN20395_c2_g1_i3_len_2735_29611.p1_type_5prime_partial_len_905 | AGI56293.2thrombospondin II | F:GO:0005509; C:GO:0005576; P:GO:0006030; P:GO:0007155; F:GO:0008061 | |
| TRINITY_DN24662_c0_g1_i1_len_2835_188803.p1_type_5prime_partial_len_905 | XP_018008120.1 lysosomal alpha-mannosidase-like | | |
| TRINITY_DN28326_c0_g1_i1_len_421_10996.p1_type_internal_len_140 | XP_018023168.1 protein tramtrack, beta isoform-like isoform X38 | | |
| TRINITY_DN16566_c0_g1_i1_len_748_5276.p1_type_complete_len_139 | XP_023224580.1transmembrane protein 107-like | | |
| TRINITY_DN27799_c0_g1_i1_len_859_197949.p1_type_5prime_partial_len_139 | ---NA--- | | |
| TRINITY_DN6938_c0_g1_i1_len_419_11045.p1_type_5prime_partial_len_139 | ---NA--- | | |

| | | | |
|---|---|---|---|
| TRINITY_DN34123_c0_g1_i1_len_419_207166.p1_type_internal_len_140 | ---NA--- | | |
| TRINITY_DN22385_c0_g2_i1_len_2109_230757.p2_type_5prime_partial_len_139 | PSN34217.1Mitochondrial import inner membrane translocase subunit Tim8 A | C:GO:0005758; F:GO:0046872; P:GO:0072321 | |
| TRINITY_DN7080_c0_g1_i1_len_516_203768.p1_type_5prime_partial_len_139 | XP_022207428.1GDNF-inducible zinc finger protein 1-like | | |
| TRINITY_DN12236_c0_g1_i1_len_568_274669.p1_type_5prime_partial_len_139 | ---NA--- | | |
| TRINITY_DN3113_c0_g1_i1_len_584_99692.p1_type_5prime_partial_len_139 | ---NA--- | | |
| TRINITY_DN6140_c0_g1_i1_len_764_199069.p1_type_5prime_partial_len_139 | XP_023937943.1zinc finger protein 43-like | | |
| TRINITY_DN15854_c0_g1_i1_len_954_33740.p1_type_5prime_partial_len_139 | XP_018009028.1 prefoldin subunit 6-like | P:GO:0006457; C:GO:0016272; F:GO:0051082 | |
| TRINITY_DN18974_c0_g1_i1_len_925_197233.p1_type_complete_len_139 | ---NA--- | | |
| TRINITY_DN3027_c0_g1_i1_len_528_203465.p1_type_3prime_partial_len_140 | XP_018025042.1 transmembrane protein 39A-like isoform X1 | | |
| TRINITY_DN28368_c0_g1_i1_len_1895_191282.p2_type_3prime_partial_len_140 | ---NA--- | | |
| TRINITY_DN15679_c0_g1_i1_len_555_37644.p1_type_5prime_partial_len_139 | XP_018027891.1 bolA-like protein 3 | | |
| TRINITY_DN14342_c0_g1_i1_len_506_275907.p2_type_5prime_partial_len_139 | ---NA--- | | |
| TRINITY_DN15334_c0_g1_i1_len_782_34932.p1_type_3prime_partial_len_140 | ---NA--- | | |
| TRINITY_DN19545_c0_g1_i1_len_2653_265284.p3_type_5prime_partial_len_139 | XP_014236267.1non-canonical poly(A) RNA polymerase protein Trf4-1-like isoform X2 | | |
| TRINITY_DN18511_c0_g1_i1_len_1188_94540.p3_type_5prime_partial_len_139 | ---NA--- | | |
| TRINITY_DN32861_c0_g1_i1_len_947_236550.p2_type_5prime_partial_len_139 | ---NA--- | | |

| | | | |
|---|---|---|---|
| TRINITY_DN13918_c0_g1_i1_len_650_273406.p2_type_5prime_partial_len_139 | AAM73800.1SCYLLA-like protein | C:GO:0005737; P:GO:0009968 | |
| TRINITY_DN21121_c1_g1_i1_len_445_305538.p1_type_5prime_partial_len_139 | ---NA--- | | |
| TRINITY_DN7171_c0_g1_i1_len_419_103656.p2_type_internal_len_140 | XP_022193556.1oxysterol-binding protein-related protein 1-like | | |
| TRINITY_DN31545_c0_g1_i1_len_419_103648.p1_type_internal_len_140 | APG53783.1transient receptor potential cation channel subfamily M member 3 | C:GO:0016020; C:GO:0016021 | |
| TRINITY_DN12020_c0_g1_i1_len_419_103643.p1_type_internal_len_140 | ---NA--- | | |
| TRINITY_DN5512_c0_g1_i1_len_419_103641.p1_type_internal_len_140 | ACN38704.1heat shock protein 70 | F:GO:0005524 | |
| TRINITY_DN4151_c0_g1_i1_len_868_237247.p1_type_5prime_partial_len_139 | XP_018015107.1 cohesin subunit SA-2-like isoform X1 | | |
| TRINITY_DN20052_c0_g1_i2_len_646_164262.p1_type_complete_len_139 | ---NA--- | | |
| TRINITY_DN4097_c0_g1_i1_len_418_245713.p1_type_internal_len_140 | XP_014601339.1 multiple epidermal growth factor-like domains protein 8 isoform X3 | | |
| TRINITY_DN12953_c0_g1_i1_len_418_134736.p1_type_internal_len_140 | ---NA--- | | |
| TRINITY_DN13817_c0_g1_i1_len_419_245674.p1_type_internal_len_140 | ---NA--- | | |
| TRINITY_DN27049_c0_g1_i1_len_419_245681.p1_type_internal_len_140 | XP_018014478.1 TRAF3-interacting protein 1-like | F:GO:0008017 | |
| TRINITY_DN29850_c0_g1_i1_len_418_245703.p1_type_internal_len_140 | ACI96113.1A-type potassium channel | F:GO:0005249; C:GO:0008076; P:GO:0034765; P:GO:0051260; P:GO:0071805 | |
| TRINITY_DN10296_c0_g1_i2_len_417_134795.p1_type_internal_len_140 | ---NA--- | | |
| TRINITY_DN16396_c0_g1_i1_len_417_134771.p1_type_internal_len_140 | ---NA--- | | |

| | | | |
|---|---|---|---|
| TRINITY_DN12578_c0_g1_i1_len_421_134644.p1_type_internal_len_140 | ---NA--- | | |
| TRINITY_DN5912_c0_g1_i1_len_421_134614.p1_type_internal_len_140 | KMQ83515.1integrase core domain protein | | |
| TRINITY_DN3397_c0_g1_i1_len_421_134609.p1_type_internal_len_140 | ---NA--- | | |
| TRINITY_DN11135_c0_g1_i1_len_419_134705.p3_type_internal_len_140 | ---NA--- | | |
| TRINITY_DN14435_c0_g1_i1_len_659_66197.p1_type_5prime_partial_len_139 | ---NA--- | | |
| TRINITY_DN24205_c0_g1_i3_len_1733_59966.p2_type_complete_len_139 | XP_018022437.1 NAD kinase-like isoform X2 | F:GO:0003951; P:GO:0006741; P:GO:0016310; P:GO:0019674 | EC:2.7.1.23 |
| TRINITY_DN15047_c0_g1_i1_len_420_278278.p1_type_internal_len_140 | ---NA--- | | |
| TRINITY_DN37442_c0_g1_i1_len_522_203605.p1_type_3prime_partial_len_140 | ---NA--- | | |
| TRINITY_DN20474_c0_g1_i1_len_430_245261.p1_type_5prime_partial_len_139 | ---NA--- | | |
| TRINITY_DN10166_c0_g1_i1_len_581_202175.p1_type_5prime_partial_len_139 | XP_023218029.1kelch-like protein 18 | | |
| TRINITY_DN20173_c0_g1_i2_len_428_245335.p1_type_5prime_partial_len_139 | ---NA--- | | |
| TRINITY_DN15593_c0_g1_i2_len_421_245599.p1_type_5prime_partial_len_139 | XP_009127069.1 thiamine thiazole synthase, chloroplastic-like | | |
| TRINITY_DN11494_c0_g2_i1_len_420_245633.p1_type_internal_len_140 | ---NA--- | | |
| TRINITY_DN11591_c0_g1_i1_len_469_167893.p1_type_5prime_partial_len_139 | ---NA--- | | |
| TRINITY_DN823_c0_g1_i1_len_432_277917.p1_type_5prime_partial_len_139 | ---NA--- | | |
| TRINITY_DN25349_c0_g1_i1_len_421_278266.p1_type_internal_len_140 | ---NA--- | | |

| | | | |
|---|---|---|---|
| TRINITY_DN3032_c0_g1_i1_len_421_71519.p1_type_internal_len_140 | XP_007061180.1 hemicentin-2 | F:GO:0005509 | |
| TRINITY_DN11041_c0_g1_i1_len_420_71566.p1_type_internal_len_140 | ---NA--- | | |
| TRINITY_DN34427_c0_g1_i1_len_420_71578.p1_type_internal_len_140 | ---NA--- | | |
| TRINITY_DN12426_c0_g1_i1_len_627_240330.p1_type_5prime_partial_len_139 | ---NA--- | | |
| TRINITY_DN2502_c0_g1_i1_len_526_203496.p1_type_5prime_partial_len_139 | XP_018008496.1 alpha-1,6-mannosyl-glycoprotein 2-beta-N-acetylglucosaminyltransferase-like | F:GO:0016740 | |
| TRINITY_DN10654_c0_g1_i1_len_470_39402.p1_type_5prime_partial_len_139 | ---NA--- | | |
| TRINITY_DN9102_c0_g1_i1_len_561_241556.p1_type_5prime_partial_len_139 | ---NA--- | | |
| TRINITY_DN3300_c0_g1_i2_len_425_71387.p1_type_5prime_partial_len_139 | PSN52665.1Dosage compensation regulator | F:GO:0003723 | |
| TRINITY_DN11086_c0_g1_i1_len_528_242307.p1_type_5prime_partial_len_139 | ---NA--- | | |
| TRINITY_DN10290_c0_g1_i1_len_487_243389.p1_type_3prime_partial_len_140 | ---NA--- | | |
| TRINITY_DN4477_c0_g1_i1_len_473_205115.p1_type_5prime_partial_len_139 | ---NA--- | | |
| TRINITY_DN4464_c0_g1_i2_len_586_37173.p1_type_3prime_partial_len_140 | ---NA--- | | |
| TRINITY_DN3969_c0_g1_i1_len_417_103709.p1_type_internal_len_140 | AET87131.1myosin light chain | F:GO:0005509 | |
| TRINITY_DN3510_c0_g1_i1_len_417_103703.p1_type_internal_len_140 | XP_018024889.1 transcriptional activator protein Pur-beta-B-like isoform X4 | | |
| TRINITY_DN6324_c0_g1_i1_len_703_239153.p1_type_5prime_partial_len_139 | ---NA--- | | |
| TRINITY_DN12180_c0_g1_i1_len_647_6330.p1_type_5prime_partial_len_139 | ---NA--- | | |

| | | | |
|---|---|---|---|
| TRINITY_DN25249_c0_g1_i1_len_517_131965.p1_type_5prime_partial_len_139 | ---NA--- | | |
| TRINITY_DN10541_c0_g1_i2_len_418_169523.p1_type_internal_len_140 | PIK51349.1putative major facilitator superfamily domain-containing protein 7 | C:GO:0016020; C:GO:0016021; P:GO:0055085 | |
| TRINITY_DN29065_c0_g1_i1_len_419_169487.p5_type_internal_len_140 | ---NA--- | | |
| TRINITY_DN1875_c0_g1_i1_len_418_169515.p1_type_internal_len_140 | ---NA--- | | |
| TRINITY_DN30404_c0_g1_i1_len_418_169500.p1_type_internal_len_140 | XP_022104328.1carbohydrate sulfotransferase 11-like | | |
| TRINITY_DN11340_c0_g1_i1_len_421_40831.p1_type_internal_len_140 | ---NA--- | | |
| TRINITY_DN28093_c0_g1_i1_len_417_169542.p1_type_5prime_partial_len_139 | ---NA--- | | |
| TRINITY_DN16401_c0_g1_i2_len_417_169578.p1_type_internal_len_140 | ---NA--- | | |
| TRINITY_DN21075_c0_g1_i1_len_512_132115.p1_type_5prime_partial_len_139 | ---NA--- | | |
| TRINITY_DN20291_c0_g1_i1_len_638_6446.p2_type_3prime_partial_len_140 | KDR20307.1Histidine triad nucleotide-binding protein 3 | F:GO:0003824 | |
| TRINITY_DN20263_c0_g1_i1_len_1288_194327.p1_type_5prime_partial_len_139 | XP_019490632.1 S-adenosylmethionine synthase-like | | |
| TRINITY_DN30941_c0_g1_i1_len_418_169526.p1_type_internal_len_140 | XP_023339719.1chitooligosaccharidolytic beta-N-acetylglucosaminidase-like | | |
| TRINITY_DN4609_c0_g1_i1_len_699_200011.p1_type_5prime_partial_len_139 | ---NA--- | | |
| TRINITY_DN22800_c0_g1_i1_len_1119_195540.p1_type_5prime_partial_len_139 | ---NA--- | | |
| TRINITY_DN23382_c0_g1_i1_len_418_306399.p1_type_internal_len_140 | XP_018014885.1 axonemal dynein light intermediate polypeptide 1-like | | |
| TRINITY_DN1181_c0_g1_i1_len_417_245746.p1_type_internal_len_140 | EEC07162.1zinc finger protein, putative | C:GO:0031462; P:GO:1902667 | |

| | | | |
|---|---|---|---|
| TRINITY_DN34037_c0_g1_i1_len_419_169469.p1_type_internal_len_140 | ---NA--- | | |
| TRINITY_DN28835_c0_g1_i1_len_2101_190549.p2_type_3prime_partial_len_140 | ---NA--- | | |
| TRINITY_DN28074_c0_g1_i1_len_419_169466.p1_type_internal_len_140 | ---NA--- | | |
| TRINITY_DN2523_c0_g1_i1_len_425_206901.p1_type_5prime_partial_len_139 | ---NA--- | | |
| TRINITY_DN22275_c0_g1_i5_len_527_131738.p1_type_5prime_partial_len_139 | ---NA--- | | |
| TRINITY_DN3827_c0_g1_i1_len_421_169385.p1_type_internal_len_140 | ADZ96217.1JHE-like carboxylesterase 1 | F:GO:0016787 | |
| TRINITY_DN26576_c0_g1_i1_len_421_169420.p1_type_internal_len_140 | ---NA--- | | |
| TRINITY_DN16289_c0_g1_i1_len_764_238365.p1_type_5prime_partial_len_138 | KFM74079.1Wilms tumor protein-like protein | F:GO:0003676 | |
| TRINITY_DN23630_c1_g1_i4_len_919_161339.p1_type_5prime_partial_len_138 | ---NA--- | | |
| TRINITY_DN14729_c0_g1_i5_len_1292_268633.p2_type_complete_len_138 | ---NA--- | | |
| TRINITY_DN25362_c0_g1_i7_len_3190_228779.p3_type_5prime_partial_len_138 | ---NA--- | | |
| TRINITY_DN20949_c0_g1_i1_len_1352_297265.p2_type_complete_len_138 | ---NA--- | | |
| TRINITY_DN16529_c0_g1_i1_len_1214_194846.p1_type_complete_len_138 | ---NA--- | | |
| TRINITY_DN15674_c0_g1_i1_len_780_128207.p3_type_5prime_partial_len_138 | XP_015520636.1PREDICTED: uncharacterized protein LOC107224904 | | |
| TRINITY_DN9847_c0_g1_i1_len_460_205572.p1_type_3prime_partial_len_139 | ---NA--- | | |
| TRINITY_DN22518_c0_g1_i1_len_416_103777.p1_type_internal_len_139 | ---NA--- | | |

| | | | |
|---|---|---|---|
| TRINITY_DN8786_c0_g1_i1_len_415_103792.p1_type_internal_len_139 | AAZ29608.1C-type lectin | F:GO:0030246 | |
| 2030\|_\|c20364_f2p5_2714_isoform_c20364_full_length_coverage_2_non_full_length_coverage_5_isoform_length_2714_9386 | ---NA--- | | |
| 2031\|_\|c6121_f2p3_2713_isoform_c6121_full_length_coverage_2_non_full_length_coverage_3_isoform_length_2713_21586 | PNF43862.1Protein-L-isoaspartate O-methyltransferase domain-containing protein 1 | P:GO:0006464; F:GO:0008168; P:GO:0032259 | |
| 2032\|_\|c2310_f3p2_2713_isoform_c2310_full_length_coverage_3_non_full_length_coverage_2_isoform_length_2713_20835 | XP_008323225.1uncharacterized protein LOC103389552 | | |
| 2033\|_\|c12273_f3p14_2712_isoform_c12273_full_length_coverage_3_non_full_length_coverage_14_isoform_length_2712_22121 | ---NA--- | | |
| TRINITY_DN17134_c0_g1_i1_len_5115_29201.p1_type_5prime_partial_len_904 | XP_018018815.1 importin subunit beta-1-like | C:GO:0005623; P:GO:0006606; F:GO:0008536 | |
| 2036\|_\|c11664_f5p11_2712_isoform_c11664_full_length_coverage_5_non_full_length_coverage_11_isoform_length_2712_12999 | ---NA--- | | |
| TRINITY_DN24266_c0_g1_i1_len_2977_229016.p1_type_5prime_partial_len_904 | CAB41634.1iron regulatory protein 1-like protein | F:GO:0003994; C:GO:0005737; P:GO:0006879; P:GO:0010040; F:GO:0051539 | EC:4.2.1.3 |
| TRINITY_DN31198_c0_g1_i1_len_4800_187197.p1_type_5prime_partial_len_904 | XP_018022828.1 ER membrane protein complex subunit 1-like | C:GO:0072546 | |
| TRINITY_DN24132_c0_g1_i1_len_3241_228724.p1_type_5prime_partial_len_904 | KDR18594.1Glutamyl aminopeptidase | F:GO:0070011 | |
| TRINITY_DN29953_c0_g1_i2_len_3188_188268.p1_type_5prime_partial_len_904 | ADK25179.1SID-1-like protein | C:GO:0016021; P:GO:0033227; F:GO:0051033 | |
| TRINITY_DN13065_c0_g1_i1_len_471_304844.p1_type_5prime_partial_len_138 | ---NA--- | | |
| TRINITY_DN4499_c0_g1_i1_len_860_237341.p1_type_complete_len_138 | ---NA--- | | |

| | | | |
|---|---|---|---|
| TRINITY_DN15013_c0_g1_i2_len_1935_191135.p3_type_5prime_partial_len_138 | ---NA--- | | |
| TRINITY_DN14809_c0_g1_i1_len_565_274719.p3_type_5prime_partial_len_138 | AHH32888.1integrin alpha 5 | P:GO:0007155; P:GO:0007229; C:GO:0008305 | |
| TRINITY_DN25114_c0_g1_i3_len_494_69275.p1_type_complete_len_138 | ---NA--- | | |
| TRINITY_DN22984_c0_g1_i1_len_417_103702.p1_type_internal_len_139 | ANG08894.1toll family protein LongTollB | P:GO:0007165; C:GO:0016021 | |
| TRINITY_DN25419_c0_g1_i1_len_977_196757.p2_type_complete_len_138 | ---NA--- | | |
| TRINITY_DN901_c0_g1_i1_len_773_97298.p2_type_complete_len_138 | ---NA--- | | |
| TRINITY_DN24450_c0_g1_i1_len_675_163874.p2_type_complete_len_138 | AXU05739.1 kinase C | F:GO:0004697; F:GO:0005524; P:GO:0006468; P:GO:0035556; F:GO:0046872 | EC:2.7.11; EC:2.7.11.13 |
| TRINITY_DN9215_c0_g1_i1_len_542_203116.p1_type_5prime_partial_len_138 | ---NA--- | | |
| TRINITY_DN22007_c0_g1_i1_len_496_204383.p1_type_3prime_partial_len_139 | ---NA--- | | |
| TRINITY_DN6100_c0_g1_i1_len_483_276449.p1_type_5prime_partial_len_138 | XP_011877444.1 UPF0528 protein CG10038 | | |
| TRINITY_DN19207_c0_g1_i3_len_1327_61373.p2_type_complete_len_138 | ---NA--- | | |
| TRINITY_DN28737_c0_g1_i1_len_2670_189086.p2_type_5prime_partial_len_138 | APB08719.1APN-like 1 protein | F:GO:0004177; P:GO:0006508; F:GO:0008237; F:GO:0008270 | EC:3.4.11 |
| TRINITY_DN31351_c0_g1_i1_len_484_69559.p1_type_5prime_partial_len_138 | ---NA--- | | |
| TRINITY_DN30914_c0_g1_i1_len_3066_188429.p4_type_5prime_partial_len_138 | APF30033.1ionotropic glutamate receptor NMDA 2-like protein | F:GO:0004970; C:GO:0016021; C:GO:0030054; P:GO:0034220; P:GO:0035235; C:GO:0045211 | |
| TRINITY_DN21723_c0_g1_i1_len_1633_157300.p1_type_5prime_partial_len_138 | ---NA--- | | |

| | | | |
|---|---|---|---|
| TRINITY_DN19349_c0_g1_i1_len_825_237688.p1_type_complete_len_138 | ---NA--- | | |
| TRINITY_DN13576_c0_g1_i1_len_414_103817.p1_type_internal_len_139 | ---NA--- | | |
| TRINITY_DN17104_c0_g1_i1_len_2118_155782.p2_type_complete_len_138 | XP_023236784.1apoptosis-resistant E3 ubiquitin protein ligase 1-like isoform X1 | | |
| TRINITY_DN23055_c0_g1_i1_len_1334_194040.p2_type_complete_len_138 | ---NA--- | | |
| TRINITY_DN21336_c0_g1_i1_len_422_71477.p1_type_5prime_partial_len_138 | ---NA--- | | |
| TRINITY_DN15100_c0_g1_i1_len_415_40991.p1_type_internal_len_139 | ---NA--- | | |
| TRINITY_DN5200_c0_g1_i1_len_668_273179.p1_type_3prime_partial_len_139 | ---NA--- | | |
| TRINITY_DN25254_c0_g1_i1_len_4497_227942.p3_type_5prime_partial_len_138 | ---NA--- | | |
| TRINITY_DN31843_c0_g1_i1_len_547_100330.p1_type_5prime_partial_len_138 | ---NA--- | | |
| TRINITY_DN9124_c0_g1_i2_len_418_71650.p1_type_internal_len_139 | ---NA--- | | |
| TRINITY_DN18932_c0_g1_i1_len_1240_268886.p2_type_complete_len_138 | XP_018023840.1 PDZ and LIM domain protein 5-like | | |
| TRINITY_DN29167_c0_g1_i1_len_3207_188243.p2_type_3prime_partial_len_139 | ---NA--- | | |
| TRINITY_DN19942_c0_g1_i1_len_1637_1374.p2_type_3prime_partial_len_139 | ---NA--- | | |
| TRINITY_DN1906_c0_g1_i1_len_548_241806.p2_type_3prime_partial_len_139 | XP_023216198.1BRCA2-interacting transcriptional repressor EMSY-like isoform X1 | | |
| TRINITY_DN20878_c0_g1_i1_len_444_70770.p1_type_5prime_partial_len_138 | ---NA--- | | |
| TRINITY_DN18042_c0_g1_i1_len_646_200879.p1_type_5prime_partial_len_138 | XP_022196638.1gastrula zinc finger protein XlCGF57.1-like | | |

| | | | |
|---|---|---|---|
| TRINITY_DN22612_c0_g1_i2_len_3731_228326.p2_type_complete_len_138 | XP_015912870.1myocyte-specific enhancer factor 2C isoform X2 | F:GO:0000977; C:GO:0005634; P:GO:0045944; F:GO:0046983 | |
| TRINITY_DN12331_c0_g1_i1_len_1038_160385.p1_type_complete_len_138 | ---NA--- | | |
| TRINITY_DN21390_c0_g1_i13_len_1994_190935.p2_type_complete_len_138 | ---NA--- | | |
| TRINITY_DN23765_c0_g1_i1_len_1038_196209.p1_type_5prime_partial_len_138 | XP_018014905.1PREDICTED: uncharacterized protein LOC108671825 | C:GO:0016020; C:GO:0016021 | |
| TRINITY_DN19445_c0_g1_i3_len_783_97193.p1_type_complete_len_138 | AQW41381.1selenium-dependent glutathione peroxidase | F:GO:0004602; P:GO:0006979; P:GO:0055114; P:GO:0098869 | EC:1.11.1.9; EC:1.11.1.7 |
| TRINITY_DN23553_c0_g1_i2_len_2159_59020.p2_type_complete_len_138 | ---NA--- | | |
| TRINITY_DN37924_c0_g1_i1_len_453_205859.p1_type_complete_len_138 | XP_018019153.1PREDICTED: uncharacterized protein LOC108675634, partial | | |
| TRINITY_DN25611_c0_g1_i2_len_2692_229445.p2_type_3prime_partial_len_139 | ---NA--- | | |
| TRINITY_DN1221_c0_g1_i1_len_416_71712.p1_type_internal_len_139 | ---NA--- | | |
| TRINITY_DN29003_c0_g1_i1_len_415_71741.p1_type_internal_len_139 | ---NA--- | | |
| TRINITY_DN18727_c0_g2_i1_len_414_41023.p1_type_internal_len_139 | ---NA--- | | |
| TRINITY_DN2169_c0_g1_i1_len_415_71753.p1_type_internal_len_139 | XP_013779349.1ankyrin-2-like isoform X4 | | |
| TRINITY_DN3388_c0_g1_i1_len_416_207334.p1_type_internal_len_139 | XP_018019910.1 GRIP and coiled-coil domain-containing protein 2-like | | |
| TRINITY_DN12859_c0_g1_i1_len_416_207339.p2_type_internal_len_139 | ---NA--- | | |
| TRINITY_DN23445_c0_g1_i1_len_1552_192814.p2_type_complete_len_138 | XP_011435464.1 derlin-2 | C:GO:0005789; C:GO:0016021 | |
| TRINITY_DN7513_c0_g1_i1_len_445_305527.p1_type_5prime_partial_len_138 | ---NA--- | | |

| | | | |
|---|---|---|---|
| TRINITY_DN15029_c0_g1_i2_len_622_130036.p1_type_5prime_partial_len_138 | ---NA--- | | |
| TRINITY_DN16946_c0_g1_i1_len_416_207331.p1_type_internal_len_139 | ---NA--- | | |
| TRINITY_DN25519_c0_g1_i1_len_542_68150.p2_type_5prime_partial_len_138 | ASW35087.1CBP80/20-dependent translation initiation factor isoform X1 | F:GO:0003743; P:GO:0006413 | |
| TRINITY_DN1736_c0_g1_i1_len_416_207328.p1_type_internal_len_139 | XP_024215463.1forkhead box protein N3-like | F:GO:0003677 | |
| TRINITY_DN15400_c0_g1_i2_len_424_10887.p1_type_5prime_partial_len_138 | XP_018027576.1 3-mercaptopyruvate sulfurtransferase-like | | |
| TRINITY_DN24620_c0_g1_i1_len_416_11149.p2_type_internal_len_139 | XP_018011463.1 cytochrome P450 302a1, mitochondrial-like | | |
| TRINITY_DN922_c0_g1_i1_len_415_207351.p2_type_internal_len_139 | ---NA--- | | |
| TRINITY_DN28887_c0_g1_i1_len_416_245772.p1_type_internal_len_139 | XP_013087018.1 metallophosphoesterase 1-like | C:GO:0005783; P:GO:0006506; C:GO:0016020; C:GO:0016021; F:GO:0016787 | |
| TRINITY_DN40405_c0_g1_i1_len_418_207252.p1_type_internal_len_139 | ---NA--- | | |
| TRINITY_DN9325_c0_g1_i1_len_416_11166.p2_type_internal_len_139 | ---NA--- | | |
| TRINITY_DN10772_c0_g1_i1_len_926_236733.p1_type_complete_len_138 | ---NA--- | | |
| TRINITY_DN1370_c0_g1_i1_len_416_306444.p1_type_5prime_partial_len_138 | ---NA--- | | |
| TRINITY_DN7894_c0_g1_i1_len_415_207349.p1_type_internal_len_139 | XP_023721400.1microtubule-actin cross-linking factor 1 isoform X4 | C:GO:0005856; P:GO:0007010; F:GO:0008092 | |
| TRINITY_DN3170_c0_g1_i1_len_415_207348.p1_type_internal_len_139 | ---NA--- | | |
| TRINITY_DN4220_c0_g1_i1_len_415_207345.p3_type_3prime_partial_len_139 | XP_023220057.1frizzled-4-like | F:GO:0004888; P:GO:0007166; P:GO:0007275; C:GO:0016020; C:GO:0016021; P:GO:0016055; F:GO:0042813 | |

| | | | |
|---|---|---|---|
| TRINITY_DN14693_c0_g1_i1_len_788_97130.p2_type_5prime_partial_len_138 | APP91325.1glutathione S-transferase | F:GO:0004364 | EC:2.5.1.18 |
| TRINITY_DN14557_c0_g1_i1_len_988_236213.p2_type_5prime_partial_len_138 | XP_018013994.1 spondin-2-like | | |
| TRINITY_DN7235_c0_g1_i1_len_414_245833.p1_type_internal_len_139 | XP_018021258.1 zinc finger SWIM domain-containing protein 8-like | F:GO:0008270; C:GO:0031462; P:GO:1902667 | |
| TRINITY_DN11125_c0_g1_i3_len_433_305897.p1_type_5prime_partial_len_138 | ---NA--- | | |
| TRINITY_DN40187_c0_g1_i1_len_416_207305.p1_type_internal_len_139 | ---NA--- | | |
| TRINITY_DN27937_c0_g1_i1_len_416_245798.p1_type_internal_len_139 | XP_014256161.1histone deacetylase 6 isoform X1 | F:GO:0016787 | |
| TRINITY_DN12368_c0_g1_i1_len_415_245806.p1_type_internal_len_139 | XP_018006730.1 calcium homeostasis endoplasmic reticulum protein-like | F:GO:0003723; C:GO:0005623; P:GO:0006396; P:GO:0006874 | |
| TRINITY_DN36782_c0_g1_i1_len_415_245825.p1_type_internal_len_139 | ---NA--- | | |
| TRINITY_DN36937_c0_g1_i1_len_417_207298.p1_type_internal_len_139 | XP_012279157.2sushi, von Willebrand factor type A, EGF and pentraxin domain-containing protein 1 | | |
| TRINITY_DN37701_c0_g1_i1_len_415_245823.p1_type_internal_len_139 | ---NA--- | | |
| TRINITY_DN6389_c0_g1_i1_len_415_245803.p1_type_internal_len_139 | XP_018025022.1 brefeldin A-inhibited guanine nucleotide-exchange protein 1-like isoform X2 | | |
| TRINITY_DN29773_c0_g1_i1_len_414_245829.p1_type_internal_len_139 | ATU31752.1basement membrane-specific heparan sulfate proteoglycan core protein | F:GO:0005509 | |
| TRINITY_DN11431_c0_g1_i1_len_1674_232230.p2_type_complete_len_138 | XP_011449912.1 ankyrin repeat domain-containing protein 29 | | |
| TRINITY_DN9816_c0_g1_i1_len_416_207322.p1_type_internal_len_139 | AGK89909.1nicotinic acetylcholine receptor subunit alpha 10 | F:GO:0004888; F:GO:0005216; F:GO:0005230; P:GO:0006811; C:GO:0016020; C:GO:0016021; P:GO:0034220 | |

| | | | |
|---|---|---|---|
| TRINITY_DN36155_c0_g1_i1_len_416_207319.p1_type_internal_len_139 | XP_018012811.1 N-acetylgalactosamine-6-sulfatase-like | | |
| TRINITY_DN19881_c0_g1_i1_len_609_6838.p1_type_5prime_partial_len_138 | ---NA--- | | |
| TRINITY_DN13156_c0_g1_i1_len_416_245785.p1_type_internal_len_139 | ---NA--- | | |
| TRINITY_DN6235_c0_g1_i1_len_417_207285.p1_type_internal_len_139 | ---NA--- | | |
| TRINITY_DN24071_c1_g1_i1_len_417_207289.p1_type_internal_len_139 | ---NA--- | | |
| TRINITY_DN27280_c0_g1_i1_len_416_245786.p1_type_internal_len_139 | AQX43455.1sodium bicarbonate cotransporter NBC | F:GO:0005452; P:GO:0015698; C:GO:0016021; P:GO:0098656 | |
| TRINITY_DN5161_c0_g1_i1_len_648_273444.p1_type_5prime_partial_len_138 | XP_018025454.1 rho GTPase-activating protein 190-like isoform X1 | | |
| TRINITY_DN14744_c0_g1_i1_len_416_207310.p1_type_internal_len_139 | ---NA--- | | |
| TRINITY_DN27530_c0_g1_i1_len_431_245199.p1_type_5prime_partial_len_138 | ---NA--- | | |
| TRINITY_DN275_c1_g1_i1_len_417_207278.p1_type_internal_len_139 | ---NA--- | | |
| TRINITY_DN38764_c0_g1_i1_len_417_207282.p1_type_internal_len_139 | XP_014262156.1coiled-coil domain-containing protein 85C | | |
| TRINITY_DN13893_c0_g1_i1_len_414_207397.p1_type_internal_len_139 | ---NA--- | | |
| TRINITY_DN13220_c0_g1_i1_len_414_207396.p1_type_internal_len_139 | ---NA--- | | |
| TRINITY_DN39727_c0_g1_i1_len_414_207394.p1_type_internal_len_139 | ---NA--- | | |
| TRINITY_DN12021_c0_g1_i1_len_415_169646.p1_type_internal_len_139 | ---NA--- | | |
| TRINITY_DN24857_c0_g1_i1_len_414_169675.p1_type_internal_len_139 | ---NA--- | | |

| | | | |
|---|---|---|---|
| TRINITY_DN35273_c0_g1_i1_len_459_244259.p1_type_3prime_partial_len_139 | ---NA--- | | |
| TRINITY_DN33481_c0_g1_i1_len_414_207408.p1_type_internal_len_139 | ATU31747.1Calcium-activated chloride channel regulator 2 | | |
| TRINITY_DN25351_c0_g1_i1_len_414_169671.p1_type_internal_len_139 | XP_013785922.1serine/threonine-protein kinase pim-1-like | | |
| TRINITY_DN7649_c0_g1_i1_len_414_169667.p1_type_internal_len_139 | ---NA--- | | |
| TRINITY_DN34320_c0_g1_i1_len_415_169651.p1_type_internal_len_139 | PNF36244.1Pantothenate kinase 4 | F:GO:0004594; F:GO:0005524; P:GO:0015937; P:GO:0016310 | EC:2.7.1.33 |
| TRINITY_DN36405_c0_g1_i1_len_415_207384.p1_type_internal_len_139 | XP_023704479.1procollagen-lysine,2-oxoglutarate 5-dioxygenase 1 isoform X2 | F:GO:0005506; F:GO:0016705; F:GO:0031418; F:GO:0051213; P:GO:0055114 | |
| 2041\|_\|c1917_f7p1_2712_isoform_c1917_full_length_coverage_7_non_full_length_coverage_1_isoform_length_2712_20607 | KYN34145.1Protein neuralized | | |
| 2042\|_\|c1926_f7p11_2711_isoform_c1926_full_length_coverage_7_non_full_length_coverage_11_isoform_length_2711_11568 | ---NA--- | | |
| 2044\|_\|c2567_f3p3_2710_isoform_c2567_full_length_coverage_3_non_full_length_coverage_3_isoform_length_2710_11977 | XP_018024751.1 nitrogen permease regulator 2-like protein | | |
| TRINITY_DN22812_c0_g1_i2_len_3364_228600.p1_type_complete_len_903 | XP_018018882.1 sarcosine dehydrogenase, mitochondrial-like | | |
| 2046\|_\|c1963_f5p2_2709_isoform_c1963_full_length_coverage_5_non_full_length_coverage_2_isoform_length_2709_11590 | XP_018014413.1PREDICTED: uncharacterized protein LOC108671383 | | |
| 2047\|_\|c17837_f3p0_2709_isoform_c17837_full_length_coverage_3_non_full_length_coverage_0_isoform_length_2709_13434 | XP_018017763.1 vacuolar protein sorting-associated protein 26B-like | C:GO:0005768; C:GO:0005829; P:GO:0006886; F:GO:0008565; C:GO:0030904; P:GO:0042147 | |
| TRINITY_DN30768_c0_g1_i3_len_4141_187510.p1_type_5prime_partial_len_903 | XP_018012118.1 kinesin-like protein KIF21B | | |

| | | | |
|---|---|---|---|
| 2050\|_\|c2105_f4p1_2708_isoform_c2105_full_length_coverage_4_non_full_length_coverage_1_isoform_length_2708_16190 | XP_022344913.1acyl-CoA dehydrogenase family member 11-like | F:GO:0016491 | |
| 2051\|_\|c1868_f9p3_2708_isoform_c1868_full_length_coverage_9_non_full_length_coverage_3_isoform_length_2708_20577 | ---NA--- | | |
| 2052\|_\|c24896_f2p3_2707_isoform_c24896_full_length_coverage_2_non_full_length_coverage_3_isoform_length_2707_18339 | XP_018026731.1 X-ray repair cross-complementing protein 5-like | P:GO:0009987; F:GO:0097159; F:GO:1901363 | |
| TRINITY_DN13297_c0_g1_i1_len_417_134782.p1_type_internal_len_139 | XP_018013869.1PREDICTED: uncharacterized protein LOC108670892, partial | | |
| TRINITY_DN14170_c0_g1_i1_len_415_169633.p1_type_internal_len_139 | ---NA--- | | |
| TRINITY_DN10921_c0_g1_i1_len_415_169639.p1_type_internal_len_139 | XP_018008840.1 collagen alpha-5(IV) chain-like | F:GO:0005201; C:GO:0005581 | |
| TRINITY_DN23259_c0_g1_i1_len_416_278416.p1_type_internal_len_139 | ---NA--- | | |
| TRINITY_DN10746_c0_g1_i1_len_596_201857.p1_type_3prime_partial_len_139 | ---NA--- | | |
| TRINITY_DN37283_c0_g1_i1_len_414_207429.p1_type_internal_len_139 | XP_017443160.1 zinc finger protein 431-like | F:GO:0003676 | |
| TRINITY_DN9618_c0_g1_i1_len_414_207425.p1_type_internal_len_139 | XP_018014164.1 bestrophin-3-like | | |
| TRINITY_DN2840_c0_g1_i1_len_417_245726.p2_type_internal_len_139 | ---NA--- | | |
| TRINITY_DN33529_c0_g1_i1_len_417_245732.p1_type_internal_len_139 | XP_018025700.1 irregular chiasm C-roughest protein-like | | |
| TRINITY_DN8503_c0_g1_i1_len_417_245733.p1_type_internal_len_139 | ---NA--- | | |
| TRINITY_DN23714_c0_g1_i1_len_887_197628.p1_type_5prime_partial_len_138 | ---NA--- | | |

| | | | |
|---|---|---|---|
| TRINITY_DN27341_c0_g1_i1_len_417_245718.p1_type_internal_len_139 | XP_018022865.1 ATP-binding cassette sub-family A member 3-like | F:GO:0005524; C:GO:0016021; F:GO:0042626; P:GO:0055085 | |
| TRINITY_DN33608_c0_g1_i1_len_414_207415.p1_type_internal_len_139 | ACN38704.1heat shock protein 70 | F:GO:0005524 | |
| TRINITY_DN35126_c0_g1_i1_len_414_207410.p1_type_internal_len_139 | ---NA--- | | |
| TRINITY_DN10915_c0_g1_i1_len_414_169682.p1_type_internal_len_139 | ---NA--- | | |
| TRINITY_DN24003_c0_g1_i1_len_414_169676.p1_type_internal_len_139 | ---NA--- | | |
| TRINITY_DN20620_c0_g1_i1_len_764_5134.p2_type_complete_len_138 | ---NA--- | | |
| TRINITY_DN36433_c0_g1_i1_len_417_245747.p1_type_internal_len_139 | ---NA--- | | |
| TRINITY_DN28872_c0_g1_i1_len_417_245748.p1_type_internal_len_139 | XP_018006687.1PREDICTED: uncharacterized protein LOC108664581 isoform X4 | | |
| TRINITY_DN1840_c0_g1_i1_len_418_245686.p1_type_internal_len_139 | PNF32141.1hypothetical protein B7P43_G02839 | | |
| TRINITY_DN26635_c0_g1_i1_len_416_169585.p1_type_internal_len_139 | ---NA--- | | |
| TRINITY_DN39358_c0_g1_i1_len_415_207370.p1_type_internal_len_139 | AJF94636.1cytochrome P450 CYP315a1 | F:GO:0004497; F:GO:0005506; F:GO:0016705; F:GO:0020037; P:GO:0055114 | |
| TRINITY_DN17847_c0_g1_i2_len_415_11181.p1_type_internal_len_139 | XP_018026238.1 E3 ubiquitin-protein ligase RBBP6-like | F:GO:0008270 | |
| TRINITY_DN18511_c0_g1_i1_len_1109_195631.p2_type_5prime_partial_len_138 | ---NA--- | | |
| TRINITY_DN10214_c0_g1_i1_len_415_207360.p1_type_internal_len_139 | ---NA--- | | |
| TRINITY_DN1048_c0_g1_i1_len_418_207243.p1_type_internal_len_139 | XP_013782928.1peroxisomal targeting signal 1 receptor-like | | |

| | | | |
|---|---|---|---|
| TRINITY_DN25579_c0_g1_i1_len_414_134875.p1_type_internal_len_139 | ---NA--- | | |
| TRINITY_DN41800_c0_g1_i1_len_415_207361.p1_type_internal_len_139 | XP_018021006.1 mucin-17-like | | |
| TRINITY_DN24196_c0_g1_i1_len_414_134877.p1_type_internal_len_139 | XP_018013460.1 leucine-rich repeat-containing protein 20-like | | |
| TRINITY_DN2559_c0_g1_i1_len_415_169617.p1_type_internal_len_139 | ---NA--- | | |
| TRINITY_DN12618_c0_g1_i1_len_619_164668.p1_type_5prime_partial_len_138 | ---NA--- | | |
| TRINITY_DN28036_c0_g1_i1_len_415_169628.p1_type_internal_len_139 | ---NA--- | | |
| TRINITY_DN942_c0_g1_i1_len_415_134819.p1_type_internal_len_139 | XP_018022538.1PREDICTED: uncharacterized protein LOC108678590 | | |
| TRINITY_DN31559_c0_g1_i1_len_415_207377.p1_type_internal_len_139 | ---NA--- | | |
| TRINITY_DN9420_c0_g1_i1_len_416_169612.p1_type_internal_len_139 | ---NA--- | | |
| TRINITY_DN17345_c0_g1_i4_len_537_68248.p1_type_5prime_partial_len_137 | ---NA--- | | |
| TRINITY_DN23215_c0_g1_i1_len_700_199996.p2_type_5prime_partial_len_137 | KXJ10292.1putative uncharacterized transposon-derived protein F52C9.6 | | |
| TRINITY_DN2986_c0_g1_i1_len_412_278566.p1_type_internal_len_138 | ---NA--- | | |
| TRINITY_DN9760_c0_g1_i1_len_452_70497.p1_type_5prime_partial_len_137 | ABI78947.1serine protease-like protein isoform 1 | F:GO:0004252; P:GO:0006508 | EC:3.4.21 |
| TRINITY_DN861_c0_g1_i1_len_421_245605.p1_type_5prime_partial_len_137 | XP_018023116.1 zinc finger protein Xfin-like | | |
| TRINITY_DN19757_c0_g1_i1_len_688_301258.p2_type_complete_len_137 | PNF21573.1NudC domain-containing protein 2 | | |
| TRINITY_DN20422_c0_g1_i1_len_411_41102.p1_type_internal_len_138 | ---NA--- | | |

| | | | |
|---|---|---|---|
| TRINITY_DN25647_c0_g1_i1_len_2886_188727.p3_type_5prime_partial_len_137 | AKE50481.1AMP-activated protein kinase subunit gamma | F:GO:0016301; F:GO:0032559; P:GO:0071900 | |
| TRINITY_DN23822_c0_g1_i1_len_4291_187428.p3_type_complete_len_137 | XP_022541067.1zinc finger protein OZF-like | F:GO:0003676 | |
| TRINITY_DN31069_c0_g1_i10_len_3572_187869.p2_type_complete_len_137 | ---NA--- | | |
| TRINITY_DN15799_c0_g3_i1_len_411_278607.p1_type_internal_len_138 | ---NA--- | | |
| TRINITY_DN27031_c0_g1_i1_len_654_66281.p1_type_5prime_partial_len_137 | ---NA--- | | |
| TRINITY_DN21373_c0_g1_i1_len_935_63525.p2_type_5prime_partial_len_137 | ODM93600.1NAD(P)H-dependent FMN reductase C4B3.06c | C:GO:0005829; F:GO:0010181; F:GO:0052873 | EC:1.5.1.38; EC:1.5.1.39 |
| TRINITY_DN1942_c0_g1_i1_len_411_278605.p1_type_internal_len_138 | AJD14729.1glutathione s-transferase | F:GO:0016740 | |
| TRINITY_DN10942_c0_g1_i1_len_529_203445.p1_type_5prime_partial_len_137 | AVI10170.1inhibitor of nuclear factor kappa-B kinase epsilon isoform 2 | F:GO:0004672; F:GO:0005524; P:GO:0006468 | |
| TRINITY_DN21531_c0_g1_i1_len_618_66816.p1_type_complete_len_137 | PFX20510.1Ribonuclease UK114 | | |
| TRINITY_DN27139_c0_g1_i1_len_647_66366.p1_type_complete_len_137 | ---NA--- | | |
| TRINITY_DN30044_c0_g2_i1_len_1114_195586.p1_type_5prime_partial_len_137 | ---NA--- | | |
| TRINITY_DN19103_c0_g1_i1_len_777_64807.p1_type_complete_len_137 | XP_015181811.1 zinc finger protein 593 homolog isoform X2 | | |
| TRINITY_DN24293_c0_g1_i2_len_2522_229739.p2_type_5prime_partial_len_137 | ---NA--- | | |
| TRINITY_DN29197_c0_g1_i1_len_411_245959.p1_type_internal_len_138 | ---NA--- | | |
| TRINITY_DN5104_c0_g1_i1_len_412_245905.p1_type_internal_len_138 | ---NA--- | | |
| TRINITY_DN11191_c0_g1_i1_len_503_69047.p1_type_5prime_partial_len_137 | ---NA--- | | |

| | | | |
|---|---|---|---|
| TRINITY_DN19350_c0_g1_i1_len_890_4225.p2_type_5prime_partial_len_137 | XP_018022486.1 spindle and kinetochore-associated protein 1-like | P:GO:0009987 | |
| TRINITY_DN16450_c2_g1_i1_len_651_98686.p1_type_5prime_partial_len_137 | ---NA--- | | |
| TRINITY_DN2005_c0_g1_i1_len_413_245904.p1_type_internal_len_138 | ---NA--- | | |
| TRINITY_DN12072_c0_g1_i2_len_781_64764.p2_type_5prime_partial_len_137 | ---NA--- | | |
| TRINITY_DN20661_c0_g1_i3_len_509_68870.p1_type_5prime_partial_len_137 | ---NA--- | | |
| TRINITY_DN10844_c0_g1_i1_len_440_277649.p1_type_5prime_partial_len_137 | ---NA--- | | |
| TRINITY_DN5742_c0_g1_i1_len_412_245916.p1_type_internal_len_138 | XP_022252685.1E3 ubiquitin-protein ligase PDZRN3-like isoform X1 | | |
| TRINITY_DN28667_c0_g1_i1_len_412_245931.p1_type_internal_len_138 | XP_018011221.1 NFX1-type zinc finger-containing protein 1-like isoform X3 | | |
| TRINITY_DN10405_c0_g1_i1_len_587_202056.p1_type_5prime_partial_len_137 | ---NA--- | | |
| TRINITY_DN254_c0_g1_i1_len_412_245926.p1_type_internal_len_138 | ---NA--- | | |
| TRINITY_DN11450_c0_g1_i1_len_457_244337.p1_type_5prime_partial_len_137 | ---NA--- | | |
| TRINITY_DN8851_c0_g1_i1_len_473_243794.p1_type_5prime_partial_len_137 | ---NA--- | | |
| TRINITY_DN4382_c0_g1_i1_len_2328_230176.p2_type_complete_len_137 | XP_008540270.1 ADP-ribosylation factor 1 isoform X3 | | |
| TRINITY_DN12275_c0_g1_i1_len_412_245908.p1_type_internal_len_138 | XP_022196639.1zinc finger protein 64 homolog, isoforms 3 and 4-like | | |
| TRINITY_DN25932_c0_g1_i1_len_413_278532.p1_type_internal_len_138 | XP_018023090.1PREDICTED: uncharacterized protein LOC108679078 | | |
| TRINITY_DN12700_c0_g1_i2_len_1697_157049.p2_type_5prime_partial_len_137 | ---NA--- | | |

| | | | |
|---|---|---|---|
| TRINITY_DN27844_c1_g1_i2_len_1162_195220.p1_type_complete_len_137 | ---NA--- | | |
| TRINITY_DN33340_c0_g1_i1_len_527_68474.p1_type_3prime_partial_len_138 | ---NA--- | | |
| TRINITY_DN79_c0_g1_i1_len_415_278465.p1_type_internal_len_138 | AAL36898.1putative antimicrobial peptide | C:GO:0005576; P:GO:0010466; F:GO:0030414 | |
| TRINITY_DN32761_c0_g1_i1_len_413_245891.p1_type_internal_len_138 | ---NA--- | | |
| TRINITY_DN17665_c0_g1_i1_len_3318_188116.p2_type_5prime_partial_len_137 | ---NA--- | | |
| TRINITY_DN27472_c0_g1_i1_len_413_245881.p1_type_internal_len_138 | ---NA--- | | |
| TRINITY_DN19390_c0_g1_i5_len_849_64152.p2_type_complete_len_137 | AGW23856.1nitric oxide synthase adaptor protein | | |
| TRINITY_DN30111_c1_g2_i1_len_714_199781.p1_type_complete_len_137 | ---NA--- | | |
| TRINITY_DN25788_c2_g1_i1_len_2429_229931.p2_type_3prime_partial_len_138 | ---NA--- | | |
| TRINITY_DN7395_c0_g1_i1_len_414_245850.p1_type_internal_len_138 | XP_022200157.1dystrophin, isoforms A/C/F/G/H-like | F:GO:0005488 | |
| TRINITY_DN11139_c0_g1_i2_len_556_202735.p1_type_3prime_partial_len_138 | ---NA--- | | |
| TRINITY_DN26346_c1_g1_i2_len_414_245845.p1_type_internal_len_138 | ---NA--- | | |
| TRINITY_DN13789_c0_g1_i1_len_444_40075.p1_type_5prime_partial_len_137 | ---NA--- | | |
| TRINITY_DN29872_c0_g1_i1_len_412_41073.p1_type_internal_len_138 | ---NA--- | | |
| TRINITY_DN15539_c0_g1_i1_len_413_245895.p1_type_internal_len_138 | APU54662.1suppressor of cytokine signaling 2 | P:GO:0001558; F:GO:0005159; C:GO:0005737; F:GO:0008269; P:GO:0035556; P:GO:0046426 | |
| TRINITY_DN9961_c0_g1_i1_len_413_278533.p1_type_internal_len_138 | ---NA--- | | |

| | | | |
|---|---|---|---|
| TRINITY_DN32203_c0_g1_i1_len_413_245893.p1_type_internal_len_138 | ---NA--- | | |
| TRINITY_DN4708_c0_g1_i1_len_504_242925.p1_type_5prime_partial_len_137 | ---NA--- | | |
| TRINITY_DN17246_c0_g1_i1_len_521_203611.p1_type_3prime_partial_len_138 | ---NA--- | | |
| TRINITY_DN8725_c0_g1_i1_len_467_167967.p1_type_5prime_partial_len_137 | XP_013791900.2hemicentin-1-like | F:GO:0005509 | |
| TRINITY_DN5759_c0_g1_i1_len_463_168072.p1_type_5prime_partial_len_137 | ---NA--- | | |
| TRINITY_DN5387_c0_g1_i1_len_518_275642.p1_type_5prime_partial_len_137 | XP_018010398.1 B-cell lymphoma 6 protein homolog isoform X1 | | |
| TRINITY_DN12999_c0_g1_i1_len_524_275500.p1_type_5prime_partial_len_137 | XP_022196639.1zinc finger protein 64 homolog, isoforms 3 and 4-like | | |
| TRINITY_DN24604_c0_g1_i1_len_413_134887.p1_type_internal_len_138 | ADM45305.1Dmc1 | F:GO:0000150; F:GO:0003677; F:GO:0005524; C:GO:0005634; P:GO:0006281; P:GO:0007131; F:GO:0008094 | EC:3.6.1.3; EC:3.6.1.15 |
| TRINITY_DN11204_c0_g1_i1_len_451_168389.p1_type_5prime_partial_len_137 | ---NA--- | | |
| TRINITY_DN11981_c0_g1_i1_len_685_200237.p1_type_complete_len_137 | ---NA--- | | |
| 2053\|_\|c2649_f2p1_2707_isoform_c2649_full_length_coverage_2_non_full_length_coverage_1_isoform_length_2707_21119 | KDR18536.1McKusick-Kaufman/Bardet-Biedl syndromes putative chaperonin | F:GO:0005524; P:GO:0006457; F:GO:0051082; P:GO:0060271 | |
| 2055\|_\|c11423_f2p0_2706_isoform_c11423_full_length_coverage_2_non_full_length_coverage_0_isoform_length_2706_12973 | ALU86069.1mitogen-activated protein kinase kinase 7 | F:GO:0004674; F:GO:0005524; P:GO:0006468 | EC:2.7.11 |
| 2056\|_\|c2657_f7p5_2706_isoform_c2657_full_length_coverage_7_non_full_length_coverage_5_isoform_length_2706_16591 | CCI71879.1gelsolin | C:GO:0005737; C:GO:0005856; F:GO:0046872; F:GO:0051015; P:GO:0051693 | |

<table>
<tr><td>2057|_|c4265_f2p0_2706_isoform_c4265_full_length_coverage_2_non_full_length_coverage_0_isoform_length_2706_21418</td><td>XP_018017843.1 signal recognition particle receptor subunit alpha homolog isoform X2</td><td>F:GO:0003924; F:GO:0005047; F:GO:0005525; C:GO:0005785; P:GO:0006614</td><td>EC:3.6.1.15</td></tr>
<tr><td>TRINITY_DN27001_c0_g1_i1_len_2862_188759.p1_type_5prime_partial_len_902</td><td>ACU31810.1alpha2 macroglobulin isoform 2</td><td>F:GO:0004866; C:GO:0005615; P:GO:0010951; C:GO:0016020</td><td></td></tr>
<tr><td>TRINITY_DN24287_c0_g1_i1_len_2741_188970.p1_type_3prime_partial_len_903</td><td>XP_015113573.1 biotin--protein ligase</td><td></td><td></td></tr>
<tr><td>2061|_|c11480_f3p1_2705_isoform_c11480_full_length_coverage_3_non_full_length_coverage_1_isoform_length_2705_12976</td><td>---NA---</td><td></td><td></td></tr>
<tr><td>2062|_|c1888_f2p10_2705_isoform_c1888_full_length_coverage_2_non_full_length_coverage_10_isoform_length_2705_25564</td><td>XP_018013579.1 syntaxin-5-like</td><td></td><td></td></tr>
<tr><td>2063|_|c5752_f2p0_2704_isoform_c5752_full_length_coverage_2_non_full_length_coverage_0_isoform_length_2704_21553</td><td>---NA---</td><td></td><td></td></tr>
<tr><td>2065|_|c1404_f12p7_2704_isoform_c1404_full_length_coverage_12_non_full_length_coverage_7_isoform_length_2704_20420</td><td>XP_018009492.1 ADP-ribosylation factor GTPase-activating protein 2-like isoform X2</td><td></td><td></td></tr>
<tr><td>TRINITY_DN14451_c0_g2_i1_len_516_38374.p1_type_5prime_partial_len_137</td><td>---NA---</td><td></td><td></td></tr>
<tr><td>TRINITY_DN15318_c0_g2_i1_len_413_134913.p1_type_internal_len_138</td><td>---NA---</td><td></td><td></td></tr>
<tr><td>TRINITY_DN9958_c0_g1_i1_len_413_134911.p2_type_internal_len_138</td><td>---NA---</td><td></td><td></td></tr>
<tr><td>TRINITY_DN33682_c0_g1_i1_len_413_134910.p1_type_internal_len_138</td><td>KFM62623.1Retrovirus-related Pol polyprotein from transposon 412</td><td>P:GO:0006807; P:GO:0043170; P:GO:0044238</td><td></td></tr>
<tr><td>TRINITY_DN30650_c0_g1_i1_len_414_11222.p1_type_internal_len_138</td><td>XP_018026078.1 multiple PDZ domain protein-like</td><td></td><td></td></tr>
<tr><td>TRINITY_DN15507_c0_g1_i1_len_1793_231766.p1_type_5prime_partial_len_137</td><td>XP_018028024.1 cartilage oligomeric matrix protein-like isoform X3</td><td></td><td></td></tr>
<tr><td>TRINITY_DN11499_c0_g1_i1_len_415_207366.p1_type_internal_len_138</td><td>---NA---</td><td></td><td></td></tr>
</table>

| | | | |
|---|---|---|---|
| TRINITY_DN18192_c0_g1_i2_len_458_305192.p2_type_5prime_partial_len_137 | ACG60902.1cathepsin C | P:GO:0006508; F:GO:0008234 | |
| TRINITY_DN8400_c0_g1_i1_len_413_11261.p1_type_internal_len_138 | ---NA--- | | |
| TRINITY_DN9179_c0_g1_i1_len_413_11246.p2_type_internal_len_138 | ---NA--- | | |
| TRINITY_DN40632_c0_g1_i1_len_953_196956.p3_type_5prime_partial_len_137 | ---NA--- | | |
| TRINITY_DN7141_c0_g1_i1_len_447_305469.p1_type_5prime_partial_len_137 | XP_018012407.1 probable ATP-dependent RNA helicase DDX46 isoform X2 | | |
| TRINITY_DN20149_c1_g4_i1_len_508_275838.p1_type_5prime_partial_len_137 | ---NA--- | | |
| TRINITY_DN1093_c0_g1_i1_len_415_207352.p1_type_internal_len_138 | XP_008545673.1 phosphoinositide 3-kinase regulatory subunit 4 isoform X3 | | |
| TRINITY_DN19078_c0_g1_i1_len_1457_296916.p2_type_5prime_partial_len_137 | ---NA--- | | |
| TRINITY_DN26342_c0_g1_i2_len_5120_227762.p3_type_complete_len_137 | ---NA--- | | |
| TRINITY_DN30751_c0_g1_i1_len_1267_194463.p2_type_5prime_partial_len_137 | ---NA--- | | |
| TRINITY_DN28042_c0_g1_i1_len_1391_193702.p1_type_3prime_partial_len_138 | ---NA--- | | |
| TRINITY_DN15194_c0_g1_i1_len_450_10097.p1_type_3prime_partial_len_138 | ---NA--- | | |
| TRINITY_DN27752_c0_g1_i1_len_479_9354.p1_type_5prime_partial_len_137 | ---NA--- | | |
| TRINITY_DN29953_c0_g1_i1_len_429_169134.p1_type_3prime_partial_len_138 | ---NA--- | | |
| TRINITY_DN418_c0_g1_i1_len_413_306556.p1_type_internal_len_138 | ---NA--- | | |
| TRINITY_DN3305_c0_g1_i1_len_425_134470.p1_type_5prime_partial_len_137 | XP_018015416.1 glycine receptor subunit alpha-2-like | | |

| | | | |
|---|---|---|---|
| TRINITY_DN27077_c0_g1_i1_len_413_169696.p1_type_internal_len_138 | ---NA--- | | |
| TRINITY_DN28970_c0_g1_i1_len_413_169709.p1_type_internal_len_138 | XP_018910904.1 rho guanine nucleotide exchange factor 11 isoform X1 | | |
| TRINITY_DN10270_c0_g1_i1_len_412_169754.p1_type_internal_len_138 | ---NA--- | | |
| TRINITY_DN15640_c0_g1_i1_len_412_169768.p1_type_internal_len_138 | ---NA--- | | |
| TRINITY_DN5472_c0_g1_i1_len_413_306575.p1_type_internal_len_138 | ---NA--- | | |
| TRINITY_DN28635_c0_g1_i1_len_422_169355.p1_type_5prime_partial_len_137 | ---NA--- | | |
| TRINITY_DN4533_c0_g1_i1_len_413_71824.p1_type_internal_len_138 | ---NA--- | | |
| TRINITY_DN28944_c0_g1_i1_len_413_71852.p1_type_internal_len_138 | ---NA--- | | |
| TRINITY_DN8017_c0_g1_i1_len_785_198801.p1_type_5prime_partial_len_137 | ---NA--- | | |
| TRINITY_DN19361_c0_g1_i2_len_412_71882.p1_type_internal_len_138 | XP_004929161.1BTB/POZ domain-containing protein 9 isoform X2 | | |
| TRINITY_DN16895_c0_g1_i2_len_412_71891.p1_type_internal_len_138 | ACL36283.1Kazal-type serine proteinase inhibitor 4 | | |
| TRINITY_DN13601_c0_g1_i2_len_537_203224.p1_type_3prime_partial_len_138 | ---NA--- | | |
| TRINITY_DN14162_c0_g1_i1_len_415_71770.p1_type_internal_len_138 | ---NA--- | | |
| TRINITY_DN31765_c0_g1_i1_len_414_71788.p1_type_internal_len_138 | XP_018027616.1 1-acyl-sn-glycerol-3-phosphate acyltransferase alpha-like | | |
| TRINITY_DN22719_c0_g1_i1_len_1631_192402.p2_type_3prime_partial_len_138 | XP_018015079.1 ruvB-like helicase 1 | F:GO:0005524; P:GO:0006281; P:GO:0006325; C:GO:0031011; P:GO:0032508; C:GO:0035267; F:GO:0043141; C:GO:0097255 | EC:3.6.1.3; EC:3.6.1.15 |

| | | | |
|---|---|---|---|
| TRINITY_DN27214_c0_g1_i1_len_414_71804.p1_type_internal_len_138 | XP_018011584.1 UDP-glucuronosyltransferase 2B14-like | | |
| TRINITY_DN17335_c0_g1_i1_len_730_199589.p1_type_5prime_partial_len_137 | ---NA--- | | |
| TRINITY_DN22617_c0_g1_i2_len_656_239849.p2_type_complete_len_137 | ---NA--- | | |
| TRINITY_DN29224_c0_g1_i1_len_411_71912.p1_type_internal_len_138 | ---NA--- | | |
| TRINITY_DN9298_c0_g1_i1_len_440_70890.p1_type_5prime_partial_len_137 | ---NA--- | | |
| TRINITY_DN26292_c0_g1_i1_len_470_39393.p1_type_3prime_partial_len_138 | ---NA--- | | |
| TRINITY_DN17937_c0_g1_i1_len_1250_159037.p1_type_complete_len_137 | ---NA--- | | |
| TRINITY_DN12834_c0_g1_i2_len_603_67047.p2_type_3prime_partial_len_138 | ---NA--- | | |
| TRINITY_DN22184_c0_g1_i1_len_1191_195025.p1_type_5prime_partial_len_137 | ---NA--- | | |
| TRINITY_DN8424_c0_g1_i1_len_412_207506.p1_type_internal_len_138 | AVP26799.1C-type lectin | F:GO:0030246 | |
| TRINITY_DN4365_c0_g1_i1_len_412_207495.p1_type_internal_len_138 | ---NA--- | | |
| TRINITY_DN31884_c0_g1_i1_len_412_207488.p1_type_internal_len_138 | XP_021343454.1uncharacterized protein LOC110443514 | | |
| TRINITY_DN24032_c0_g3_i1_len_749_128461.p1_type_5prime_partial_len_137 | ---NA--- | | |
| TRINITY_DN3275_c0_g1_i1_len_413_207481.p1_type_internal_len_138 | ---NA--- | | |
| TRINITY_DN11544_c0_g1_i2_len_413_207478.p1_type_internal_len_138 | ---NA--- | | |
| TRINITY_DN11301_c0_g1_i1_len_413_207477.p1_type_internal_len_138 | ---NA--- | | |

| | | | |
|---|---|---|---|
| TRINITY_DN6674_c0_g1_i1_len_413_207468.p1_type_internal_len_138 | XP_018017453.1 nuclear pore complex protein DDB_G0274915-like | F:GO:0004252; P:GO:0006508 | EC:3.4.21 |
| TRINITY_DN12558_c0_g1_i1_len_413_207459.p1_type_internal_len_138 | AUC64082.1arrestin 2 | P:GO:0007165 | |
| TRINITY_DN39154_c0_g1_i1_len_413_207457.p1_type_internal_len_138 | ---NA--- | | |
| TRINITY_DN32157_c0_g1_i1_len_413_207452.p1_type_internal_len_138 | XP_023212063.1dnaJ homolog subfamily C member 13-like | P:GO:0006898; C:GO:0010008 | |
| TRINITY_DN36997_c0_g1_i1_len_411_207550.p1_type_internal_len_138 | XP_018012041.1 transcription factor LBX1-like | | |
| TRINITY_DN893_c0_g1_i1_len_411_207549.p1_type_internal_len_138 | XP_018020943.1 tyrosine-protein phosphatase 69D-like | | |
| TRINITY_DN23347_c0_g1_i5_len_411_207546.p1_type_internal_len_138 | ---NA--- | | |
| TRINITY_DN15609_c0_g1_i1_len_414_103838.p1_type_internal_len_138 | ---NA--- | | |
| TRINITY_DN4870_c0_g1_i1_len_414_103832.p1_type_internal_len_138 | XP_018027149.1 neurogenic locus Notch protein-like | F:GO:0005509; P:GO:0007219; P:GO:0007275; C:GO:0016021; P:GO:0030154; P:GO:0050793 | |
| TRINITY_DN13031_c0_g1_i1_len_412_207509.p1_type_internal_len_138 | AXR98478.1techylectin-5B-like protein | | |
| TRINITY_DN13466_c0_g1_i1_len_412_207507.p2_type_internal_len_138 | ASW35087.1CBP80/20-dependent translation initiation factor isoform X1 | F:GO:0003743; P:GO:0006413 | |
| TRINITY_DN37287_c0_g1_i1_len_414_207409.p1_type_internal_len_138 | ---NA--- | | |
| TRINITY_DN15288_c0_g1_i3_len_412_11298.p1_type_internal_len_138 | ---NA--- | | |
| TRINITY_DN33423_c0_g1_i1_len_414_207407.p2_type_internal_len_138 | ---NA--- | | |
| TRINITY_DN4102_c0_g1_i1_len_414_207393.p2_type_internal_len_138 | ---NA--- | | |
| TRINITY_DN15207_c0_g1_i2_len_961_196888.p1_type_5prime_partial_len_137 | ---NA--- | | |

| | | | |
|---|---|---|---|
| TRINITY_DN31901_c0_g1_i1_len_413_207443.p1_type_internal_len_138 | ---NA--- | | |
| TRINITY_DN40797_c0_g1_i1_len_413_207441.p1_type_internal_len_138 | ---NA--- | | |
| TRINITY_DN1263_c0_g1_i1_len_414_207427.p1_type_internal_len_138 | ---NA--- | | |
| TRINITY_DN1354_c0_g1_i1_len_414_207423.p1_type_internal_len_138 | XP_018010174.1 proton-coupled folate transporter-like | | |
| TRINITY_DN7402_c0_g1_i1_len_414_207418.p1_type_internal_len_138 | XP_018013657.1 5'-3' exoribonuclease 1-like isoform X2 | | |
| TRINITY_DN33328_c0_g1_i1_len_414_207416.p1_type_internal_len_138 | AAX48011.1prophenoloxidase, partial | F:GO:0016491; F:GO:0046872; P:GO:0055114 | |
| TRINITY_DN30430_c0_g1_i1_len_6463_186861.p5_type_3prime_partial_len_138 | ---NA--- | | |
| TRINITY_DN10432_c0_g1_i1_len_984_196697.p1_type_5prime_partial_len_137 | XP_018908074.1 V-type proton ATPase subunit G | | |
| TRINITY_DN22421_c0_g1_i1_len_682_239471.p1_type_3prime_partial_len_138 | ---NA--- | | |
| TRINITY_DN12762_c0_g1_i1_len_412_103888.p1_type_internal_len_138 | AFB71156.1serine protease-like protein 1 | F:GO:0008233 | |
| TRINITY_DN10402_c0_g1_i1_len_413_103880.p1_type_internal_len_138 | ---NA--- | | |
| TRINITY_DN9613_c0_g1_i1_len_413_103868.p1_type_internal_len_138 | KMQ83515.1integrase core domain protein | F:GO:0003676; P:GO:0015074 | |
| TRINITY_DN18575_c0_g1_i1_len_413_103863.p1_type_internal_len_138 | ---NA--- | | |
| TRINITY_DN13293_c0_g1_i1_len_2044_92070.p3_type_complete_len_136 | ---NA--- | | |
| TRINITY_DN17116_c0_g1_i1_len_856_271280.p1_type_complete_len_136 | ---NA--- | | |
| TRINITY_DN28101_c0_g1_i1_len_410_41162.p2_type_internal_len_137 | AOD27418.1Notch | F:GO:0005509; P:GO:0007219; P:GO:0007275; C:GO:0016021; P:GO:0030154; P:GO:0050793 | |

| | | | |
|---|---|---|---|
| TRINITY_DN19200_c0_g1_i1_len_520_203625.p1_type_5prime_partial_len_136 | XP_018007716.1 zeta-sarcoglycan-like | C:GO:0016012; C:GO:0016020; C:GO:0016021 | |
| TRINITY_DN24442_c0_g1_i1_len_763_199087.p1_type_3prime_partial_len_137 | ---NA--- | | |
| TRINITY_DN9850_c0_g1_i1_len_779_198870.p2_type_3prime_partial_len_137 | ---NA--- | | |
| TRINITY_DN16815_c0_g1_i1_len_542_203114.p1_type_3prime_partial_len_137 | ---NA--- | | |
| TRINITY_DN14815_c0_g1_i1_len_410_41135.p1_type_internal_len_137 | ---NA--- | | |
| TRINITY_DN22920_c0_g1_i1_len_410_41137.p1_type_internal_len_137 | XP_018018028.1  CTP synthase-like | F:GO:0003883; P:GO:0006221 | EC:6.3.4.2 |
| TRINITY_DN8668_c0_g1_i1_len_410_41150.p2_type_internal_len_137 | AHL26189.1lipoprotein receptor 1 | F:GO:0005509; C:GO:0016021 | |
| TRINITY_DN13301_c0_g1_i1_len_880_271085.p2_type_5prime_partial_len_136 | ---NA--- | | |
| TRINITY_DN19958_c0_g1_i2_len_753_199229.p1_type_5prime_partial_len_136 | ---NA--- | | |
| TRINITY_DN14210_c0_g1_i1_len_736_199489.p1_type_complete_len_136 | XP_026464826.1mediator of RNA polymerase II transcription subunit 11 | F:GO:0003712; P:GO:0006357; C:GO:0016592 | |
| TRINITY_DN14136_c0_g1_i1_len_409_41186.p3_type_internal_len_137 | ---NA--- | | |
| TRINITY_DN19072_c0_g1_i1_len_1450_1811.p2_type_5prime_partial_len_136 | ---NA--- | | |
| TRINITY_DN4063_c0_g1_i1_len_833_34527.p1_type_complete_len_136 | XP_019871495.1 putative peptidyl-tRNA hydrolase PTRHD1 | F:GO:0004045 | EC:3.1.1.29; EC:3.1.1.1 |
| TRINITY_DN13352_c0_g1_i2_len_894_270977.p1_type_5prime_partial_len_136 | XP_018023119.1 zinc finger and BTB domain-containing protein 17-like isoform X1 | | |
| TRINITY_DN26330_c0_g2_i4_len_4298_228027.p1_type_complete_len_901 | XP_021939378.1epidermal growth factor receptor kinase substrate 8-like isoform X1 | | |

| | | | |
|---|---|---|---|
| 2067\|_\|c47827_f67p23_2703_isoform_c47827_full_length_coverage_67_non_full_length_coverage_23_isoform_length_2703_19695 | XP_018020868.1 transketolase-like isoform X2 | | |
| TRINITY_DN12399_c0_g1_i1_len_2785_229306.p1_type_5prime_partial_len_901 | XP_011699998.1 protein aurora borealis | | |
| 2070\|_\|c1603_f9p11_2703_isoform_c1603_full_length_coverage_9_non_full_length_coverage_11_isoform_length_2703_20471 | XP_014277147.1serine-arginine protein 55 isoform X2 | F:GO:0003676 | |
| 2071\|_\|c12230_f3p1_2703_isoform_c12230_full_length_coverage_3_non_full_length_coverage_1_isoform_length_2703_17494 | XP_024890042.1CCR4-NOT transcription complex subunit 1 isoform X2 | P:GO:0006417; C:GO:0030015 | |
| 2073\|_\|c11103_f3p4_2702_isoform_c11103_full_length_coverage_3_non_full_length_coverage_4_isoform_length_2702_22021 | XP_018021609.1 kinetochore protein NDC80 homolog | | |
| 2074\|_\|c27745_f2p2_2702_isoform_c27745_full_length_coverage_2_non_full_length_coverage_2_isoform_length_2702_23057 | XP_023323894.1MICOS complex subunit MIC60-1-like | C:GO:0016020 | |
| 2075\|_\|c2493_f4p0_2702_isoform_c2493_full_length_coverage_4_non_full_length_coverage_0_isoform_length_2702_16456 | XP_021702333.1zinc finger protein 585B isoform X18 | F:GO:0003676 | |
| TRINITY_DN31154_c0_g1_i3_len_3340_188086.p1_type_complete_len_900 | XP_018025100.1 protein inturned-like | | |
| TRINITY_DN25362_c0_g1_i7_len_3190_228779.p1_type_5prime_partial_len_900 | XP_018016572.1 mucin-17-like | | |
| TRINITY_DN18742_c0_g1_i2_len_2007_295733.p4_type_5prime_partial_len_136 | PVD28401.1hypothetical protein C0Q70_10988 | F:GO:0003779; C:GO:0005856 | |
| TRINITY_DN18448_c0_g1_i1_len_1319_2216.p2_type_complete_len_136 | XP_011674282.1 centromere protein J-like | | |
| TRINITY_DN28273_c0_g2_i6_len_1070_195936.p1_type_complete_len_136 | ---NA--- | | |
| TRINITY_DN5320_c0_g1_i2_len_435_71067.p1_type_5prime_partial_len_136 | ---NA--- | | |

| | | | |
|---|---|---|---|
| TRINITY_DN5076_c0_g1_i1_len_482_101739.p1_type_complete_len_136 | XP_013784372.1microsomal glutathione S-transferase 3-like | C:GO:0016020; F:GO:0016740 | |
| TRINITY_DN23239_c1_g1_i3_len_3474_153930.p2_type_complete_len_136 | ---NA--- | | |
| TRINITY_DN8646_c0_g1_i2_len_495_38794.p1_type_5prime_partial_len_136 | ---NA--- | | |
| TRINITY_DN34968_c0_g1_i1_len_409_71998.p1_type_internal_len_137 | XP_018012981.1 alpha-mannosidase 2x-like | | |
| TRINITY_DN1186_c0_g1_i1_len_1275_234210.p1_type_3prime_partial_len_137 | ---NA--- | | |
| TRINITY_DN2338_c0_g1_i1_len_411_71933.p1_type_internal_len_137 | ---NA--- | | |
| TRINITY_DN2946_c0_g1_i1_len_410_71938.p1_type_internal_len_137 | ---NA--- | | |
| TRINITY_DN27099_c0_g1_i1_len_410_71940.p1_type_internal_len_137 | XP_008560289.1PREDICTED: uncharacterized protein KIAA1841 | | |
| TRINITY_DN6202_c0_g1_i1_len_410_71959.p1_type_internal_len_137 | ---NA--- | | |
| TRINITY_DN230_c0_g1_i1_len_410_71964.p1_type_internal_len_137 | ---NA--- | | |
| TRINITY_DN2708_c0_g1_i1_len_410_169848.p1_type_internal_len_137 | XP_018024684.1 protein unc-13 homolog A-like | F:GO:0005509; F:GO:0005543; P:GO:0007268; F:GO:0019992; P:GO:0035556 | |
| TRINITY_DN21061_c0_g1_i2_len_2280_155426.p3_type_complete_len_136 | ---NA--- | | |
| TRINITY_DN5465_c0_g1_i1_len_409_169882.p1_type_internal_len_137 | ---NA--- | | |
| TRINITY_DN35760_c0_g1_i1_len_681_200294.p1_type_3prime_partial_len_137 | ---NA--- | | |
| TRINITY_DN13378_c0_g1_i1_len_412_169756.p1_type_internal_len_137 | EFX90234.1hypothetical protein DAPPUDRAFT_299942 | F:GO:0008483; P:GO:1901605 | |
| TRINITY_DN10435_c0_g1_i1_len_408_306737.p1_type_internal_len_137 | ---NA--- | | |

| | | | |
|---|---|---|---|
| TRINITY_DN13278_c0_g1_i1_len_472_69915.p1_type_5prime_partial_len_136 | ---NA--- | | |
| TRINITY_DN15888_c0_g1_i1_len_685_200235.p1_type_5prime_partial_len_136 | ---NA--- | | |
| TRINITY_DN7337_c0_g1_i1_len_408_169900.p1_type_internal_len_137 | ---NA--- | | |
| TRINITY_DN23350_c0_g1_i1_len_694_200102.p1_type_3prime_partial_len_137 | ---NA--- | | |
| TRINITY_DN2226_c0_g1_i1_len_408_169929.p1_type_internal_len_137 | ---NA--- | | |
| TRINITY_DN15264_c0_g1_i1_len_463_205446.p1_type_3prime_partial_len_137 | ---NA--- | | |
| TRINITY_DN38758_c0_g1_i1_len_411_207545.p1_type_internal_len_137 | ---NA--- | | |
| TRINITY_DN7292_c0_g1_i1_len_430_40520.p1_type_5prime_partial_len_136 | ---NA--- | | |
| TRINITY_DN41823_c0_g1_i1_len_411_207548.p1_type_internal_len_137 | XP_022199050.1 zinc finger protein 26-like | | |
| TRINITY_DN25116_c0_g1_i2_len_1926_231338.p2_type_5prime_partial_len_136 | ---NA--- | | |
| TRINITY_DN14538_c0_g1_i1_len_911_197374.p1_type_complete_len_136 | ---NA--- | | |
| TRINITY_DN701_c0_g1_i1_len_411_207558.p1_type_internal_len_137 | ---NA--- | | |
| TRINITY_DN18771_c0_g1_i1_len_411_207566.p1_type_internal_len_137 | ---NA--- | | |
| TRINITY_DN16493_c0_g1_i4_len_410_207570.p1_type_internal_len_137 | EDO32584.1predicted protein | | |
| TRINITY_DN30277_c1_g1_i1_len_6785_186831.p3_type_complete_len_136 | ---NA--- | | |
| TRINITY_DN14892_c0_g1_i1_len_408_11425.p1_type_internal_len_137 | ---NA--- | | |

| | | | |
|---|---|---|---|
| TRINITY_DN6832_c0_g1_i1_len_412_207484.p1_type_internal_len_137 | PNY15768.1retrotransposon-related protein | F:GO:0003676; F:GO:0004190; P:GO:0006508; P:GO:0015074 | |
| TRINITY_DN23824_c0_g1_i1_len_3094_58109.p6_type_5prime_partial_len_136 | XP_018017966.1 collagen alpha-1(I) chain-like isoform X2 | | |
| TRINITY_DN380_c0_g1_i1_len_408_11435.p1_type_internal_len_137 | ---NA--- | | |
| TRINITY_DN4801_c0_g1_i1_len_412_207518.p1_type_internal_len_137 | XP_018018093.1 kinesin-like protein KIF13A isoform X1 | F:GO:0003777; F:GO:0005524; C:GO:0005623; F:GO:0008017; P:GO:0072383 | EC:3.6.1.15 |
| TRINITY_DN24514_c0_g1_i1_len_933_197155.p1_type_complete_len_136 | XP_026465565.1cytochrome c oxidase assembly protein COX16 homolog, mitochondrial | C:GO:0016020 | |
| TRINITY_DN32337_c0_g1_i2_len_410_207601.p1_type_internal_len_137 | XP_015928354.1protein quiver | C:GO:0016020; P:GO:0050789 | |
| TRINITY_DN272_c0_g1_i1_len_410_207602.p1_type_internal_len_137 | ---NA--- | | |
| TRINITY_DN40099_c0_g1_i1_len_409_207605.p1_type_internal_len_137 | XP_018020234.1 E3 ubiquitin-protein ligase MYCBP2-like | | |
| TRINITY_DN8629_c0_g1_i1_len_409_207607.p1_type_internal_len_137 | ---NA--- | | |
| TRINITY_DN1287_c0_g1_i2_len_409_207609.p1_type_internal_len_137 | XP_022908158.1bifunctional heparan sulfate N-deacetylase/N-sulfotransferase | F:GO:0016740 | |
| TRINITY_DN38539_c0_g1_i1_len_409_207625.p1_type_internal_len_137 | ---NA--- | | |
| TRINITY_DN36040_c0_g1_i1_len_409_207627.p1_type_internal_len_137 | APG53779.1transient receptor potential cation channel subfamily A member 1 | F:GO:0005216; C:GO:0016021; P:GO:0034220 | |
| TRINITY_DN13719_c0_g2_i1_len_409_207634.p1_type_internal_len_137 | XP_018010562.1 vacuolar protein sorting-associated protein 13D-like | | |
| TRINITY_DN936_c0_g1_i1_len_409_207650.p1_type_internal_len_137 | ---NA--- | | |
| TRINITY_DN35566_c0_g1_i1_len_409_207656.p1_type_internal_len_137 | XP_023328593.1putative uncharacterized protein DDB_G0268364 | | |

| | | | |
|---|---|---|---|
| TRINITY_DN8470_c0_g1_i1_len_408_207659.p1_type_internal_len_137 | ---NA--- | | |
| TRINITY_DN24432_c0_g1_i2_len_3335_57991.p5_type_5prime_partial_len_136 | XP_018012406.1 probable ATP-dependent RNA helicase DDX46 isoform X1 | | |
| TRINITY_DN7613_c0_g1_i1_len_408_207664.p1_type_internal_len_137 | XP_018011143.1 triple functional domain protein-like | | |
| TRINITY_DN11841_c1_g1_i1_len_408_207674.p1_type_internal_len_137 | ---NA--- | | |
| TRINITY_DN38239_c0_g1_i1_len_410_207573.p1_type_internal_len_137 | ---NA--- | | |
| TRINITY_DN34933_c0_g1_i1_len_410_207575.p1_type_internal_len_137 | ---NA--- | | |
| TRINITY_DN2645_c0_g1_i1_len_410_207576.p1_type_internal_len_137 | ---NA--- | | |
| TRINITY_DN41213_c0_g1_i1_len_410_207578.p1_type_internal_len_137 | ANG08894.1toll family protein LongTollB | P:GO:0007165; C:GO:0016021 | |
| TRINITY_DN7573_c0_g1_i1_len_410_207586.p1_type_internal_len_137 | ---NA--- | | |
| TRINITY_DN18593_c0_g1_i2_len_410_207592.p1_type_internal_len_137 | ---NA--- | | |
| TRINITY_DN31843_c0_g1_i1_len_410_207595.p1_type_internal_len_137 | XP_018025433.1 probable peroxisomal acyl-coenzyme A oxidase 1 | | |
| TRINITY_DN25765_c0_g1_i3_len_1650_232328.p2_type_complete_len_136 | ---NA--- | | |
| TRINITY_DN2263_c0_g1_i1_len_416_207330.p1_type_5prime_partial_len_136 | ---NA--- | | |
| TRINITY_DN23374_c0_g1_i4_len_1834_231628.p3_type_complete_len_136 | ---NA--- | | |
| TRINITY_DN5439_c0_g1_i2_len_411_11324.p1_type_internal_len_137 | XP_018026155.1 mediator of RNA polymerase II transcription subunit 13-like | | |
| TRINITY_DN25470_c0_g1_i1_len_1282_234167.p2_type_complete_len_136 | ---NA--- | | |

| | | | |
|---|---|---|---|
| TRINITY_DN1295_c0_g1_i1_len_1361_193888.p1_type_complete_len_136 | ---NA--- | | |
| TRINITY_DN9246_c0_g1_i1_len_568_202457.p1_type_5prime_partial_len_136 | XP_018016751.1 U8 snoRNA-decapping enzyme-like isoform X2 | | |
| TRINITY_DN16505_c0_g1_i1_len_478_276585.p1_type_5prime_partial_len_136 | ---NA--- | | |
| TRINITY_DN25657_c0_g1_i1_len_410_11374.p1_type_internal_len_137 | ---NA--- | | |
| TRINITY_DN8281_c0_g1_i1_len_410_104002.p2_type_internal_len_137 | XP_013794399.1sushi, von Willebrand factor type A, EGF and pentraxin domain-containing protein 1-like | F:GO:0005509 | |
| TRINITY_DN19841_c1_g1_i4_len_410_103984.p3_type_internal_len_137 | ---NA--- | | |
| TRINITY_DN4319_c0_g1_i1_len_409_104047.p1_type_internal_len_137 | XP_015119032.1 mortality factor 4-like protein 1 isoform X1 | | |
| TRINITY_DN2262_c0_g1_i1_len_409_104027.p1_type_internal_len_137 | ---NA--- | | |
| TRINITY_DN13205_c0_g1_i1_len_409_104024.p1_type_internal_len_137 | ---NA--- | | |
| TRINITY_DN12246_c0_g1_i1_len_409_104014.p1_type_internal_len_137 | ---NA--- | | |
| TRINITY_DN37349_c0_g1_i1_len_603_201718.p1_type_complete_len_136 | ---NA--- | | |
| TRINITY_DN31981_c0_g1_i1_len_412_103913.p1_type_internal_len_137 | AJG06864.1laccase-1-like protein | F:GO:0005507; F:GO:0016491; P:GO:0055114 | |
| TRINITY_DN12622_c0_g1_i1_len_459_244261.p1_type_5prime_partial_len_136 | XP_018011231.1 zinc finger protein 513-like | | |
| TRINITY_DN38448_c0_g1_i1_len_439_206353.p1_type_3prime_partial_len_137 | XP_010150533.1 transmembrane protein 17-like | | |
| TRINITY_DN18573_c0_g1_i6_len_695_163611.p1_type_complete_len_136 | ---NA--- | | |
| TRINITY_DN16519_c0_g1_i1_len_465_244078.p1_type_5prime_partial_len_136 | ---NA--- | | |

| | | | |
|---|---|---|---|
| TRINITY_DN10188_c0_g1_i1_len_446_206092.p1_type_5prime_partial_len_136 | XP_026679627.1peroxisomal carnitine O-octanoyltransferase-like | F:GO:0016740 | |
| TRINITY_DN19185_c0_g1_i1_len_617_240513.p1_type_complete_len_136 | ---NA--- | | |
| TRINITY_DN8555_c0_g1_i1_len_1011_160579.p2_type_5prime_partial_len_136 | ---NA--- | | |
| TRINITY_DN7306_c0_g1_i1_len_647_240008.p1_type_complete_len_136 | ---NA--- | | |
| TRINITY_DN10918_c0_g1_i1_len_666_200528.p1_type_5prime_partial_len_136 | ---NA--- | | |
| TRINITY_DN24701_c0_g1_i1_len_666_200526.p1_type_complete_len_136 | ---NA--- | | |
| TRINITY_DN12973_c0_g1_i1_len_841_162008.p1_type_complete_len_136 | ---NA--- | | |
| TRINITY_DN26536_c0_g1_i1_len_513_242692.p1_type_3prime_partial_len_137 | ---NA--- | | |
| TRINITY_DN17475_c0_g1_i1_len_410_246001.p1_type_internal_len_137 | ---NA--- | | |
| TRINITY_DN36427_c0_g1_i1_len_410_246011.p1_type_3prime_partial_len_137 | AAT42131.1mas-like protein | F:GO:0004252; P:GO:0006508 | EC:3.4.21 |
| TRINITY_DN9715_c0_g1_i1_len_410_246014.p1_type_internal_len_137 | ---NA--- | | |
| TRINITY_DN28183_c0_g1_i1_len_410_246019.p1_type_internal_len_137 | AAC61691.1inositol 1,4,5-trisphosphate receptor | F:GO:0005220; C:GO:0005783; C:GO:0016021; P:GO:0048016; P:GO:0070588; F:GO:0070679 | |
| TRINITY_DN12595_c0_g1_i1_len_409_246036.p1_type_internal_len_137 | ---NA--- | | |
| TRINITY_DN6507_c0_g1_i1_len_409_246052.p1_type_internal_len_137 | ---NA--- | | |
| TRINITY_DN10949_c0_g1_i1_len_409_246056.p1_type_internal_len_137 | ---NA--- | | |
| TRINITY_DN7305_c0_g1_i1_len_409_246057.p1_type_internal_len_137 | ---NA--- | | |

<table>
<tr><td>TRINITY_DN3758_c0_g1_i1_len_408_246062.p1_type_internal_len_137</td><td>---NA---</td><td></td><td></td></tr>
<tr><td>2078|_|c13396_f2p1_2700_isoform_c13396_full_length_coverage_2_non_full_length_coverage_1_isoform_length_2700_13135</td><td>AXF35700.1cryptochrome 2</td><td></td><td></td></tr>
<tr><td>2079|_|c18082_f2p0_2700_isoform_c18082_full_length_coverage_2_non_full_length_coverage_0_isoform_length_2700_22677</td><td>---NA---</td><td></td><td></td></tr>
<tr><td>2080|_|c2740_f4p10_2699_isoform_c2740_full_length_coverage_4_non_full_length_coverage_10_isoform_length_2699_16661</td><td>XP_016915213.1 E3 ubiquitin-protein ligase RNF126-A isoform X1</td><td></td><td></td></tr>
<tr><td>2081|_|c3792_f2p3_2699_isoform_c3792_full_length_coverage_2_non_full_length_coverage_3_isoform_length_2699_21378</td><td>---NA---</td><td></td><td></td></tr>
<tr><td>2082|_|c7279_f3p2_2698_isoform_c7279_full_length_coverage_3_non_full_length_coverage_2_isoform_length_2698_21671</td><td>---NA---</td><td></td><td></td></tr>
<tr><td>2083|_|c19769_f2p16_2698_isoform_c19769_full_length_coverage_2_non_full_length_coverage_16_isoform_length_2698_28975</td><td>AVN99029.1DIS3-like exonuclease 2</td><td>P:GO:0000291; F:GO:0004527; F:GO:0004540; F:GO:0005488; P:GO:0090501</td><td></td></tr>
<tr><td>2084|_|c1744_f6p3_2698_isoform_c1744_full_length_coverage_6_non_full_length_coverage_3_isoform_length_2698_11469</td><td>XP_026272674.1ubiquitin carboxyl-terminal hydrolase 30 homolog isoform X2</td><td></td><td></td></tr>
<tr><td>2085|_|c2316_f4p5_2698_isoform_c2316_full_length_coverage_4_non_full_length_coverage_5_isoform_length_2698_11792</td><td>XP_023705347.1transcriptional adapter 2B isoform X3</td><td>P:GO:0031323; P:GO:0051171; P:GO:0060255; P:GO:0080090</td><td></td></tr>
<tr><td>2086|_|c33815_f5p1_2698_isoform_c33815_full_length_coverage_5_non_full_length_coverage_1_isoform_length_2698_23321</td><td>XP_018023993.1 gamma-tubulin complex component 3-like</td><td>C:GO:0015630; C:GO:0044430</td><td></td></tr>
<tr><td>2087|_|c17847_f3p0_2698_isoform_c17847_full_length_coverage_3_non_full_length_coverage_0_isoform_length_2698_22509</td><td>XP_018006888.1 transport and Golgi organization protein 1-like isoform X5</td><td></td><td></td></tr>
</table>

| | | | |
|---|---|---|---|
| TRINITY_DN35099_c0_g1_i1_len_408_246074.p1_type_internal_len_137 | AUC64090.1phosphatidylinositol transfer protein | F:GO:0005548; P:GO:0015914; F:GO:0046872 | |
| TRINITY_DN5281_c0_g1_i1_len_468_167954.p1_type_5prime_partial_len_136 | ---NA--- | | |
| TRINITY_DN19843_c0_g1_i1_len_1761_59886.p2_type_5prime_partial_len_136 | ---NA--- | | |
| TRINITY_DN37304_c0_g1_i1_len_412_245929.p3_type_5prime_partial_len_136 | AMD09631.1glutamine synthetase | F:GO:0004356; F:GO:0005524; P:GO:0006542 | EC:6.3.1.2 |
| TRINITY_DN13779_c0_g1_i1_len_411_245946.p1_type_internal_len_137 | XP_018009184.1 serine-rich adhesin for platelets-like | | |
| TRINITY_DN36928_c0_g1_i1_len_411_245950.p1_type_internal_len_137 | AVI10170.1inhibitor of nuclear factor kappa-B kinase epsilon isoform 2 | F:GO:0004672; F:GO:0005524; P:GO:0006468 | |
| TRINITY_DN12389_c0_g1_i1_len_411_245955.p1_type_internal_len_137 | ---NA--- | | |
| TRINITY_DN28689_c0_g1_i1_len_411_245965.p1_type_internal_len_137 | ---NA--- | | |
| TRINITY_DN11430_c0_g1_i1_len_411_245971.p1_type_internal_len_137 | ---NA--- | | |
| TRINITY_DN18936_c0_g1_i1_len_411_245977.p1_type_internal_len_137 | ---NA--- | | |
| TRINITY_DN5501_c0_g1_i1_len_411_245982.p1_type_internal_len_137 | ---NA--- | | |
| TRINITY_DN13325_c0_g1_i2_len_410_245986.p1_type_internal_len_137 | ---NA--- | | |
| TRINITY_DN36977_c0_g1_i1_len_410_245993.p1_type_internal_len_137 | XP_023708627.1nostrin isoform X4 | | |
| TRINITY_DN29826_c0_g1_i1_len_410_246000.p1_type_internal_len_137 | XP_012558008.1 N-acetylgalactosamine kinase-like | F:GO:0016772 | |
| TRINITY_DN15410_c0_g1_i1_len_420_169436.p1_type_5prime_partial_len_136 | XP_018022783.1PREDICTED: uncharacterized protein LOC108678811 | | |
| TRINITY_DN37656_c0_g1_i1_len_438_244956.p1_type_5prime_partial_len_136 | ---NA--- | | |

| | | | |
|---|---|---|---|
| TRINITY_DN21572_c0_g1_i1_len_408_278696.p1_type_internal_len_137 | XP_018026078.1 multiple PDZ domain protein-like | | |
| TRINITY_DN28699_c0_g1_i1_len_432_245162.p1_type_5prime_partial_len_136 | ---NA--- | | |
| TRINITY_DN9515_c0_g1_i1_len_409_278668.p2_type_internal_len_137 | ---NA--- | | |
| TRINITY_DN2387_c0_g1_i1_len_409_278661.p1_type_internal_len_137 | ---NA--- | | |
| TRINITY_DN6616_c0_g1_i1_len_467_304955.p1_type_5prime_partial_len_136 | XP_018012166.1PREDICTED: uncharacterized protein LOC108669362 isoform X2 | | |
| TRINITY_DN823_c0_g1_i1_len_448_244629.p1_type_5prime_partial_len_136 | ---NA--- | | |
| TRINITY_DN1785_c0_g1_i1_len_412_278576.p1_type_internal_len_137 | ---NA--- | | |
| TRINITY_DN9628_c0_g1_i1_len_412_278563.p1_type_internal_len_137 | ---NA--- | | |
| TRINITY_DN32914_c0_g1_i1_len_412_245910.p1_type_internal_len_137 | ---NA--- | | |
| TRINITY_DN19713_c0_g1_i1_len_412_245918.p1_type_internal_len_137 | KZR99292.140S ribosomal protein S2 | F:GO:0003723; F:GO:0003735; P:GO:0006412; C:GO:0022627 | |
| TRINITY_DN23993_c0_g1_i2_len_1582_60405.p2_type_3prime_partial_len_137 | ---NA--- | | |
| TRINITY_DN14345_c0_g1_i1_len_482_204816.p1_type_5prime_partial_len_136 | ---NA--- | | |
| TRINITY_DN10531_c0_g1_i1_len_510_203940.p1_type_5prime_partial_len_136 | ---NA--- | | |
| TRINITY_DN24237_c0_g1_i2_len_3092_228891.p2_type_complete_len_136 | ---NA--- | | |
| TRINITY_DN30556_c0_g1_i1_len_409_135041.p1_type_internal_len_137 | ---NA--- | | |
| TRINITY_DN21485_c0_g1_i2_len_2198_568.p3_type_5prime_partial_len_136 | ---NA--- | | |

| | | | |
|---|---|---|---|
| TRINITY_DN16586_c0_g1_i1_len_753_128427.p1_type_5prime_partial_len_136 | ---NA--- | | |
| TRINITY_DN9404_c0_g1_i1_len_561_202641.p1_type_complete_len_136 | KGG50728.1hypothetical protein DI09_596p10, partial | | |
| TRINITY_DN29387_c0_g1_i1_len_2365_189781.p2_type_3prime_partial_len_137 | ---NA--- | | |
| TRINITY_DN22049_c0_g1_i2_len_3531_228444.p3_type_complete_len_136 | ---NA--- | | |
| TRINITY_DN8883_c0_g1_i1_len_411_134967.p2_type_internal_len_137 | ---NA--- | | |
| TRINITY_DN15528_c0_g1_i1_len_929_299231.p3_type_5prime_partial_len_136 | ---NA--- | | |
| TRINITY_DN10605_c0_g1_i1_len_410_134998.p1_type_internal_len_137 | XP_018017488.1PREDICTED: uncharacterized protein LOC108674095 | | |
| TRINITY_DN27151_c0_g1_i1_len_410_135008.p1_type_internal_len_137 | XP_018014104.1 inactive pancreatic lipase-related protein 1-like | | |
| TRINITY_DN5827_c0_g1_i1_len_502_204182.p1_type_3prime_partial_len_137 | XP_007434271.1regulatory-associated protein of mTOR-like | P:GO:0031929; C:GO:0031931 | |
| TRINITY_DN2931_c0_g1_i1_len_411_134956.p1_type_internal_len_137 | XP_014341861.1 armadillo repeat-containing protein 2 | | |
| TRINITY_DN16850_c0_g1_i1_len_835_198198.p2_type_complete_len_136 | XP_023235830.1tetratricopeptide repeat protein 36 homolog | | |
| TRINITY_DN23713_c0_g1_i1_len_544_131397.p1_type_3prime_partial_len_137 | KOF62742.1hypothetical protein OCBIM_22021999mg | | |
| TRINITY_DN33482_c0_g1_i1_len_408_246082.p1_type_internal_len_136 | XP_018023307.1 nipped-B-like protein A | F:GO:0003682; C:GO:0005634; P:GO:0007049; P:GO:0010468 | |
| TRINITY_DN5629_c0_g1_i1_len_407_246138.p3_type_internal_len_136 | XP_018006837.1 cadherin-89D-like | | |
| TRINITY_DN2259_c0_g1_i1_len_408_246064.p1_type_internal_len_136 | ---NA--- | | |
| TRINITY_DN9403_c0_g1_i1_len_408_246079.p1_type_internal_len_136 | ---NA--- | | |

| | | | |
|---|---|---|---|
| TRINITY_DN12154_c0_g1_i1_len_408_246077.p1_type_internal_len_136 | ---NA--- | | |
| TRINITY_DN5845_c0_g1_i1_len_406_170003.p1_type_internal_len_136 | ---NA--- | | |
| TRINITY_DN34350_c0_g1_i1_len_407_246137.p1_type_internal_len_136 | ---NA--- | | |
| TRINITY_DN8825_c0_g1_i1_len_407_246129.p1_type_internal_len_136 | XP_018016675.1 transmembrane and coiled-coil domain-containing protein 4-like | C:GO:0016021 | |
| TRINITY_DN31520_c0_g1_i1_len_406_169977.p1_type_internal_len_136 | ---NA--- | | |
| TRINITY_DN21119_c0_g1_i6_len_1633_296442.p3_type_complete_len_135 | ---NA--- | | |
| TRINITY_DN8374_c0_g1_i1_len_407_246100.p1_type_internal_len_136 | XP_023326419.1WD repeat-containing protein 35-like | | |
| TRINITY_DN6571_c0_g1_i1_len_407_246109.p1_type_internal_len_136 | XP_018017479.1 lysyl oxidase homolog 2-like isoform X2 | | |
| TRINITY_DN36951_c0_g1_i1_len_407_246123.p1_type_internal_len_136 | ---NA--- | | |
| TRINITY_DN5433_c0_g1_i1_len_407_246126.p1_type_internal_len_136 | ---NA--- | | |
| TRINITY_DN2509_c0_g1_i1_len_406_306825.p2_type_internal_len_136 | ---NA--- | | |
| TRINITY_DN9294_c0_g1_i1_len_433_206590.p1_type_5prime_partial_len_135 | XP_005182833.1 translocator protein | C:GO:0016020; C:GO:0016021 | |
| TRINITY_DN27330_c0_g1_i1_len_522_68589.p1_type_5prime_partial_len_135 | ---NA--- | | |
| TRINITY_DN26883_c0_g1_i4_len_2489_189476.p2_type_5prime_partial_len_135 | ---NA--- | | |
| TRINITY_DN22233_c0_g1_i1_len_2100_155831.p3_type_complete_len_135 | ---NA--- | | |
| TRINITY_DN3530_c0_g1_i1_len_1217_268990.p1_type_5prime_partial_len_135 | ---NA--- | | |

| | | | |
|---|---|---|---|
| TRINITY_DN5430_c0_g1_i2_len_579_202242.p1_type_5prime_partial_len_135 | AIU96362.1peroxinectin | F:GO:0004601; P:GO:0006979; F:GO:0020037; P:GO:0055114; P:GO:0098869 | EC:1.11.1.7 |
| TRINITY_DN9122_c0_g1_i1_len_412_278582.p1_type_5prime_partial_len_135 | XP_013783175.1RING finger and SPRY domain-containing protein 1-like | | |
| TRINITY_DN5334_c0_g1_i1_len_405_170026.p1_type_internal_len_136 | ---NA--- | | |
| TRINITY_DN17841_c0_g1_i1_len_691_129103.p1_type_5prime_partial_len_135 | ALO61081.1C-jun-amino-terminal kinase-interacting protein 4 | F:GO:0005078; F:GO:0016301; P:GO:0043410 | |
| TRINITY_DN685_c0_g1_i1_len_408_246059.p1_type_internal_len_136 | ---NA--- | | |
| TRINITY_DN26899_c0_g1_i1_len_494_167209.p1_type_5prime_partial_len_135 | ---NA--- | | |
| TRINITY_DN24108_c0_g1_i2_len_3156_122041.p2_type_complete_len_135 | ---NA--- | | |
| TRINITY_DN31069_c0_g1_i10_len_3572_187869.p3_type_complete_len_135 | ---NA--- | | |
| TRINITY_DN34146_c0_g1_i1_len_409_169879.p1_type_internal_len_136 | XP_018027289.1 sushi, von Willebrand factor type A, EGF and pentraxin domain-containing protein 1-like | | |
| TRINITY_DN14917_c0_g1_i1_len_587_130576.p1_type_complete_len_135 | AEB54641.1ribosomal protein L32 | F:GO:0003735; C:GO:0005840; P:GO:0006412 | |
| TRINITY_DN19040_c0_g1_i1_len_408_306719.p1_type_internal_len_136 | ---NA--- | | |
| TRINITY_DN4889_c0_g1_i1_len_407_306757.p1_type_internal_len_136 | XP_018025308.1 sodium/glucose cotransporter 4-like | C:GO:0016021; F:GO:0022857; P:GO:0055085 | |
| TRINITY_DN30668_c0_g1_i1_len_407_306763.p1_type_internal_len_136 | XP_023216226.1fibrillin-2-like isoform X2 | | |
| TRINITY_DN14958_c0_g1_i1_len_428_206791.p1_type_5prime_partial_len_135 | ---NA--- | | |
| TRINITY_DN31441_c0_g1_i1_len_406_246172.p1_type_internal_len_136 | ---NA--- | | |

| | | | |
|---|---|---|---|
| TRINITY_DN21723_c0_g1_i1_len_1812_59754.p2_type_5prime_partial_len_135 | XP_018027589.1 U4/U6 small nuclear ribonucleoprotein Prp31-like | | |
| TRINITY_DN6964_c0_g1_i1_len_408_169931.p1_type_internal_len_136 | ---NA--- | | |
| TRINITY_DN18682_c0_g1_i1_len_787_4948.p3_type_complete_len_135 | ---NA--- | | |
| TRINITY_DN25091_c0_g1_i1_len_408_169930.p2_type_internal_len_136 | AUW64506.2serine protease 5 | F:GO:0008233 | |
| TRINITY_DN2535_c0_g1_i1_len_405_246203.p1_type_internal_len_136 | ---NA--- | | |
| TRINITY_DN2787_c0_g1_i1_len_405_246210.p1_type_internal_len_136 | ---NA--- | | |
| TRINITY_DN432_c0_g1_i1_len_405_246214.p1_type_internal_len_136 | ---NA--- | | |
| TRINITY_DN31868_c0_g1_i1_len_407_169943.p2_type_internal_len_136 | ---NA--- | | |
| TRINITY_DN1545_c0_g1_i1_len_406_246150.p1_type_internal_len_136 | ---NA--- | | |
| TRINITY_DN7896_c0_g1_i1_len_406_246152.p1_type_internal_len_136 | XP_021955202.1zinc finger SWIM domain-containing protein 8-like isoform X1 | F:GO:0008270 | |
| TRINITY_DN35446_c0_g1_i1_len_406_246155.p1_type_internal_len_136 | ---NA--- | | |
| TRINITY_DN28511_c0_g1_i1_len_406_246157.p1_type_internal_len_136 | ---NA--- | | |
| TRINITY_DN2267_c0_g1_i1_len_407_306784.p1_type_internal_len_136 | ---NA--- | | |
| TRINITY_DN12975_c1_g1_i2_len_420_169459.p1_type_5prime_partial_len_135 | ---NA--- | | |
| TRINITY_DN25873_c0_g1_i1_len_408_169895.p1_type_internal_len_136 | ---NA--- | | |
| TRINITY_DN3089_c0_g1_i1_len_685_301292.p1_type_complete_len_135 | ---NA--- | | |

| | | | |
|---|---|---|---|
| TRINITY_DN17537_c0_g1_i1_len_407_306787.p1_type_internal_len_136 | ---NA--- | | |
| TRINITY_DN20516_c0_g1_i3_len_499_304184.p1_type_5prime_partial_len_135 | ---NA--- | | |
| TRINITY_DN33822_c0_g1_i1_len_487_243378.p1_type_5prime_partial_len_135 | ---NA--- | | |
| TRINITY_DN31351_c0_g1_i1_len_481_243556.p1_type_5prime_partial_len_135 | ---NA--- | | |
| TRINITY_DN17346_c0_g1_i1_len_582_302617.p1_type_5prime_partial_len_135 | ---NA--- | | |
| 2088\|_\|c17841_f3p4_2698_isoform_c17841_full_length_coverage_3_non_full_length_coverage_4_isoform_length_2698_17816 | ---NA--- | | |
| 2089\|_\|c2310_f7p2_2698_isoform_c2310_full_length_coverage_7_non_full_length_coverage_2_isoform_length_2698_16315 | ---NA--- | | |
| TRINITY_DN26368_c1_g1_i3_len_2697_229434.p1_type_internal_len_900 | EEB19050.1papilin, putative | F:GO:0004867; C:GO:0005576; P:GO:0006508; F:GO:0008233; P:GO:0010466; P:GO:0010951; F:GO:0016787; F:GO:0030414 | |
| 2091\|_\|c8985_f3p2_2697_isoform_c8985_full_length_coverage_3_non_full_length_coverage_2_isoform_length_2697_21821 | ---NA--- | | |
| TRINITY_DN29474_c1_g1_i1_len_2806_188847.p1_type_complete_len_899 | XP_018006730.1 calcium homeostasis endoplasmic reticulum protein-like | F:GO:0003676; F:GO:0003723; C:GO:0005623; P:GO:0006396; P:GO:0006874 | |
| 2093\|_\|c11645_f3p4_2696_isoform_c11645_full_length_coverage_3_non_full_length_coverage_4_isoform_length_2696_12996 | ---NA--- | | |
| 2095\|_\|c937_f20p15_2696_isoform_c937_full_length_coverage_20_non_full_length_coverage_15_isoform_length_2696_15794 | XP_018016329.1 ATP-binding cassette sub-family E member 1-like | F:GO:0005524; F:GO:0016887 | EC:3.6.1.3; EC:3.6.1.15 |

<table>
<tr><td></td><td></td><td></td><td></td></tr>
<tr><td>2096|_|c5582_f2p3_2696_isoform_c5582_full_length_coverage_2_non_full_length_coverage_3_isoform_length_2696_12481</td><td>XP_018024966.1 polycystic kidney disease protein 1-like 2</td><td>F:GO:0005509; C:GO:0016020; C:GO:0016021</td><td></td></tr>
<tr><td>2097|_|c5169_f3p8_2695_isoform_c5169_full_length_coverage_3_non_full_length_coverage_8_isoform_length_2695_16932</td><td>---NA---</td><td></td><td></td></tr>
<tr><td>2099|_|c2738_f2p1_2694_isoform_c2738_full_length_coverage_2_non_full_length_coverage_1_isoform_length_2694_16659</td><td>BAH98113.1GTP binding protein alpha subunit Gi</td><td>F:GO:0003924; F:GO:0005525; P:GO:0007188; F:GO:0031683; F:GO:0046872</td><td>EC:3.6.1.15</td></tr>
<tr><td>TRINITY_DN19519_c0_g1_i1_len_1559_157552.p1_type_complete_len_135</td><td>---NA---</td><td></td><td></td></tr>
<tr><td>TRINITY_DN16161_c0_g1_i1_len_405_104216.p1_type_internal_len_136</td><td>---NA---</td><td></td><td></td></tr>
<tr><td>TRINITY_DN6000_c0_g1_i1_len_408_104073.p1_type_internal_len_136</td><td>EEZ97506.1Carbohydrate sulfotransferase 5-like Protein</td><td>F:GO:0008146; P:GO:0008152</td><td></td></tr>
<tr><td>TRINITY_DN1515_c0_g1_i1_len_407_104111.p1_type_internal_len_136</td><td>---NA---</td><td></td><td></td></tr>
<tr><td>TRINITY_DN9272_c0_g1_i1_len_407_104100.p5_type_internal_len_136</td><td>---NA---</td><td></td><td></td></tr>
<tr><td>TRINITY_DN24735_c0_g1_i1_len_1792_191694.p2_type_5prime_partial_len_135</td><td>---NA---</td><td></td><td></td></tr>
<tr><td>TRINITY_DN2246_c0_g1_i1_len_545_241899.p1_type_3prime_partial_len_136</td><td>XP_018014298.1 equilibrative nucleoside transporter 3-like</td><td>F:GO:0005337; C:GO:0016020; C:GO:0016021; P:GO:1901642</td><td></td></tr>
<tr><td>TRINITY_DN20294_c0_g1_i2_len_977_63260.p1_type_complete_len_135</td><td>---NA---</td><td></td><td></td></tr>
<tr><td>TRINITY_DN5653_c0_g1_i1_len_564_241494.p1_type_5prime_partial_len_135</td><td>---NA---</td><td></td><td></td></tr>
<tr><td>TRINITY_DN19885_c0_g1_i1_len_585_241081.p1_type_complete_len_135</td><td>---NA---</td><td></td><td></td></tr>
<tr><td>TRINITY_DN9401_c0_g1_i1_len_567_37471.p1_type_5prime_partial_len_135</td><td>---NA---</td><td></td><td></td></tr>
<tr><td>TRINITY_DN25148_c0_g1_i1_len_1712_192033.p2_type_5prime_partial_len_135</td><td>XP_014300410.1 serine palmitoyltransferase 2</td><td></td><td></td></tr>
</table>

| | | | |
|---|---|---|---|
| TRINITY_DN17354_c0_g1_i2_len_593_240931.p1_type_5prime_partial_len_135 | ---NA--- | | |
| TRINITY_DN9904_c0_g1_i1_len_972_160900.p3_type_3prime_partial_len_136 | ---NA--- | | |
| TRINITY_DN21743_c0_g1_i1_len_832_198240.p1_type_5prime_partial_len_135 | ---NA--- | | |
| TRINITY_DN1400_c0_g1_i1_len_661_239770.p1_type_5prime_partial_len_135 | ---NA--- | | |
| TRINITY_DN19483_c0_g1_i2_len_938_161187.p2_type_5prime_partial_len_135 | ---NA--- | | |
| TRINITY_DN25969_c0_g1_i1_len_682_239465.p1_type_5prime_partial_len_135 | ---NA--- | | |
| TRINITY_DN18313_c1_g1_i1_len_896_63797.p2_type_5prime_partial_len_135 | ---NA--- | | |
| TRINITY_DN16032_c0_g1_i1_len_1050_160306.p2_type_5prime_partial_len_135 | ---NA--- | | |
| TRINITY_DN2021_c0_g1_i1_len_866_64020.p1_type_complete_len_135 | ---NA--- | | |
| TRINITY_DN587_c0_g1_i1_len_406_278752.p1_type_internal_len_136 | ---NA--- | | |
| TRINITY_DN15171_c0_g1_i1_len_406_278741.p1_type_internal_len_136 | ---NA--- | | |
| TRINITY_DN12442_c1_g1_i1_len_557_202726.p1_type_5prime_partial_len_135 | XP_011206752.2 IDLSRF-like peptide | | |
| TRINITY_DN18346_c0_g1_i1_len_406_278735.p1_type_internal_len_136 | ---NA--- | | |
| TRINITY_DN30017_c0_g1_i1_len_633_301915.p1_type_5prime_partial_len_135 | ---NA--- | | |
| TRINITY_DN29476_c0_g1_i1_len_407_278721.p3_type_internal_len_136 | ---NA--- | | |
| TRINITY_DN16097_c0_g1_i2_len_630_164527.p2_type_5prime_partial_len_135 | ---NA--- | | |

| | | | |
|---|---|---|---|
| TRINITY_DN24201_c0_g1_i1_len_651_200787.p1_type_5prime_partial_len_135 | KYN09248.1Glycerol-3-phosphate acyltransferase 3 | C:GO:0016020; C:GO:0016021; F:GO:0016740; F:GO:0016746 | |
| TRINITY_DN8753_c0_g1_i1_len_440_244877.p1_type_5prime_partial_len_135 | APF30033.1ionotropic glutamate receptor NMDA 2-like protein | F:GO:0004970; C:GO:0016021; C:GO:0030054; P:GO:0034220; P:GO:0035235; C:GO:0045211 | |
| TRINITY_DN4568_c0_g1_i1_len_406_41258.p1_type_internal_len_136 | AAI54534.1LOC562037 protein | F:GO:0004827; F:GO:0005524; C:GO:0005737; P:GO:0006433; C:GO:0017101 | EC:6.1.1.15 |
| TRINITY_DN16521_c0_g1_i1_len_541_166146.p1_type_3prime_partial_len_136 | ---NA--- | | |
| TRINITY_DN26041_c0_g1_i1_len_541_166152.p1_type_5prime_partial_len_135 | ---NA--- | | |
| TRINITY_DN19630_c0_g1_i1_len_1967_156202.p1_type_complete_len_135 | ---NA--- | | |
| TRINITY_DN11495_c0_g1_i2_len_408_278689.p1_type_internal_len_136 | ---NA--- | | |
| TRINITY_DN16108_c0_g1_i1_len_560_202645.p1_type_5prime_partial_len_135 | ---NA--- | | |
| TRINITY_DN2558_c0_g1_i1_len_409_104035.p1_type_internal_len_136 | XP_013788008.2two pore calcium channel protein 1-like isoform X1 | | |
| TRINITY_DN3747_c0_g1_i1_len_409_104020.p1_type_internal_len_136 | XP_018014885.1 axonemal dynein light intermediate polypeptide 1-like | | |
| TRINITY_DN17903_c1_g1_i1_len_1731_156923.p2_type_complete_len_135 | ---NA--- | | |
| TRINITY_DN7708_c0_g1_i1_len_646_164266.p1_type_5prime_partial_len_135 | XP_018007236.1 enhancer of rudimentary homolog isoform X1 | P:GO:0006221; P:GO:0007049; P:GO:0045747 | |
| TRINITY_DN26764_c0_g1_i1_len_2128_190462.p2_type_3prime_partial_len_136 | ---NA--- | | |
| TRINITY_DN632_c0_g1_i1_len_405_72147.p1_type_internal_len_136 | ---NA--- | | |
| TRINITY_DN38727_c0_g1_i1_len_743_199386.p1_type_5prime_partial_len_135 | XP_011142568.2homeotic protein empty spiracles | C:GO:0005634; P:GO:0006355; F:GO:0043565 | |

| | | | |
|---|---|---|---|
| TRINITY_DN26280_c1_g1_i1_len_1414_193572.p1_type_complete_len_135 | ---NA--- | | |
| TRINITY_DN24559_c0_g1_i1_len_525_275471.p1_type_5prime_partial_len_135 | ---NA--- | | |
| TRINITY_DN24286_c0_g3_i1_len_523_203568.p1_type_5prime_partial_len_135 | ---NA--- | | |
| TRINITY_DN17430_c0_g1_i1_len_3600_294731.p3_type_5prime_partial_len_135 | ---NA--- | | |
| TRINITY_DN5468_c0_g1_i1_len_465_133240.p1_type_5prime_partial_len_135 | XP_018026962.1 LIM domain-containing protein jub-like isoform X3 | | |
| TRINITY_DN23530_c0_g1_i1_len_1968_231210.p2_type_complete_len_135 | ---NA--- | | |
| TRINITY_DN5081_c0_g1_i1_len_408_72029.p1_type_internal_len_136 | XP_018011644.1 irregular chiasm C-roughest protein-like | C:GO:0016020; C:GO:0016021 | |
| TRINITY_DN19715_c0_g1_i2_len_963_95820.p3_type_5prime_partial_len_135 | ---NA--- | | |
| TRINITY_DN24757_c0_g1_i1_len_467_133168.p1_type_5prime_partial_len_135 | ---NA--- | | |
| TRINITY_DN17727_c0_g1_i1_len_407_72086.p2_type_internal_len_136 | ---NA--- | | |
| TRINITY_DN9213_c0_g1_i1_len_878_271095.p1_type_5prime_partial_len_135 | XP_018022130.1 transcription elongation factor SPT4-like | F:GO:0003746; C:GO:0005634; P:GO:0006414; F:GO:0008270; P:GO:0032786 | |
| TRINITY_DN35005_c0_g1_i1_len_406_72108.p1_type_internal_len_136 | ---NA--- | | |
| TRINITY_DN25908_c0_g1_i1_len_406_72116.p1_type_internal_len_136 | XP_018024207.1 protein O-mannosyl-transferase 2-like | C:GO:0016020; C:GO:0016021 | |
| TRINITY_DN18593_c0_g1_i1_len_407_72076.p1_type_internal_len_136 | ---NA--- | | |
| TRINITY_DN8253_c0_g1_i1_len_407_72082.p1_type_internal_len_136 | ---NA--- | | |
| TRINITY_DN20707_c0_g1_i2_len_407_72085.p1_type_internal_len_136 | ---NA--- | | |

| | | | |
|---|---|---|---|
| TRINITY_DN31801_c0_g1_i1_len_408_11442.p1_type_internal_len_136 | ---NA--- | | |
| TRINITY_DN31018_c0_g1_i1_len_406_11496.p2_type_internal_len_136 | ---NA--- | | |
| TRINITY_DN16327_c0_g1_i2_len_577_7372.p2_type_complete_len_135 | ---NA--- | | |
| TRINITY_DN4750_c0_g1_i1_len_407_11475.p1_type_internal_len_136 | ---NA--- | | |
| TRINITY_DN33567_c0_g1_i1_len_411_207535.p1_type_5prime_partial_len_135 | AQT26399.1chitin-binding protein | F:GO:0042302 | |
| TRINITY_DN33533_c0_g1_i1_len_406_135130.p1_type_internal_len_136 | XP_019768858.1 high-affinity choline transporter 1-like | C:GO:0016021; F:GO:0022857; P:GO:0055085 | |
| TRINITY_DN27895_c0_g1_i1_len_405_11524.p1_type_internal_len_136 | ---NA--- | | |
| TRINITY_DN22034_c0_g1_i2_len_3068_91116.p4_type_complete_len_135 | ---NA--- | | |
| TRINITY_DN17085_c0_g1_i1_len_426_134437.p1_type_5prime_partial_len_135 | KFM83382.1Retrovirus-related Pol polyprotein from transposon 412 | F:GO:0003676; F:GO:0004190; P:GO:0006508; P:GO:0015074 | |
| TRINITY_DN14136_c0_g1_i1_len_409_41186.p5_type_internal_len_136 | ---NA--- | | |
| TRINITY_DN2954_c0_g1_i1_len_483_276440.p1_type_3prime_partial_len_136 | ACD74843.1trehalose-6-phosphate synthase | F:GO:0003825; P:GO:0005992 | EC:2.4.1.15 |
| TRINITY_DN10721_c0_g1_i1_len_554_100229.p1_type_5prime_partial_len_135 | ---NA--- | | |
| TRINITY_DN27453_c0_g1_i1_len_480_276523.p1_type_5prime_partial_len_135 | XP_026737637.1ras-related protein Rab-8A | F:GO:0003924; F:GO:0005525 | EC:3.6.1.15 |
| TRINITY_DN32206_c0_g1_i1_len_406_207782.p1_type_internal_len_136 | ABC94530.1receptor guanylyl cyclase II +18 isoform | F:GO:0000166; P:GO:0009190; C:GO:0016020; F:GO:0016849; P:GO:1901564 | |
| TRINITY_DN36985_c0_g1_i1_len_406_207765.p2_type_internal_len_136 | XP_021968317.1cartilage oligomeric matrix protein-like | F:GO:0005509; C:GO:0005576; P:GO:0007155 | |
| TRINITY_DN2074_c0_g1_i1_len_407_207708.p1_type_internal_len_136 | ---NA--- | | |

| | | | |
|---|---|---|---|
| TRINITY_DN16846_c0_g1_i1_len_406_207766.p1_type_internal_len_136 | ---NA--- | | |
| TRINITY_DN29930_c0_g1_i1_len_5434_187025.p4_type_5prime_partial_len_135 | ---NA--- | | |
| TRINITY_DN32470_c0_g1_i1_len_483_69579.p1_type_3prime_partial_len_136 | ---NA--- | | |
| TRINITY_DN21841_c0_g1_i1_len_406_207784.p1_type_internal_len_136 | ---NA--- | | |
| TRINITY_DN30653_c0_g1_i1_len_405_306849.p1_type_internal_len_136 | AHK23065.1double-stranded RNA-specific adenosine deaminase | F:GO:0003723; F:GO:0004000; P:GO:0006396 | EC:3.5.4.4 |
| TRINITY_DN8863_c0_g1_i1_len_408_207669.p1_type_internal_len_136 | XP_023705494.1glucoside xylosyltransferase 1 | C:GO:0016021; F:GO:0016757 | |
| TRINITY_DN4375_c0_g1_i1_len_408_207667.p1_type_internal_len_136 | XP_018024896.1 fat-like cadherin-related tumor suppressor homolog | F:GO:0005509; C:GO:0005886; P:GO:0007155; P:GO:0007156; C:GO:0016020; C:GO:0016021 | |
| TRINITY_DN6449_c0_g1_i1_len_407_207732.p1_type_internal_len_136 | XP_018021461.1 Niemann-Pick C1 protein-like | F:GO:0005319; P:GO:0006869; C:GO:0016021 | |
| TRINITY_DN37360_c0_g1_i1_len_407_207726.p1_type_internal_len_136 | XP_018025851.1 neurofibromin-like | | |
| TRINITY_DN6301_c0_g1_i1_len_406_207749.p1_type_internal_len_136 | KZS06468.1RNA-binding protein 45 | F:GO:0003723 | |
| TRINITY_DN1293_c0_g1_i1_len_407_207725.p1_type_internal_len_136 | ---NA--- | | |
| TRINITY_DN521_c0_g1_i1_len_407_207723.p1_type_internal_len_136 | ---NA--- | | |
| TRINITY_DN17155_c0_g1_i1_len_469_69992.p1_type_5prime_partial_len_135 | XP_018017488.1PREDICTED: uncharacterized protein LOC108674095 | | |
| TRINITY_DN20932_c0_g1_i3_len_979_3723.p1_type_5prime_partial_len_135 | ---NA--- | | |
| TRINITY_DN7612_c0_g1_i1_len_406_207745.p1_type_internal_len_136 | ---NA--- | | |

| | | | |
|---|---|---|---|
| TRINITY_DN16368_c0_g1_i1_len_407_207710.p1_type_internal_len_136 | OAD56514.1Disintegrin and metalloproteinase domain-containing protein 12 | F:GO:0004222; P:GO:0006508; P:GO:0007229; C:GO:0016020; C:GO:0016021 | |
| TRINITY_DN6621_c0_g1_i1_len_407_207709.p1_type_internal_len_136 | ---NA--- | | |
| TRINITY_DN4872_c0_g1_i1_len_649_200826.p1_type_5prime_partial_len_135 | XP_018026900.1 cat eye syndrome critical region protein 5-like | | |
| TRINITY_DN31046_c0_g1_i2_len_3757_187732.p2_type_3prime_partial_len_136 | ---NA--- | | |
| TRINITY_DN41442_c0_g1_i1_len_407_207719.p1_type_internal_len_136 | XP_018015674.1 protocadherin-16-like isoform X1 | | |
| TRINITY_DN34064_c0_g1_i1_len_406_207762.p1_type_internal_len_136 | XP_022235568.1regulation of nuclear pre-mRNA domain-containing protein 1B-like isoform X1 | F:GO:0000993; C:GO:0016591; P:GO:0031124 | |
| TRINITY_DN35749_c0_g1_i1_len_407_207717.p1_type_internal_len_136 | XP_013379222.1DNA replication ATP-dependent helicase/nuclease DNA2 | F:GO:0017111 | EC:3.6.1.15 |
| TRINITY_DN5061_c0_g1_i1_len_407_207712.p1_type_internal_len_136 | XP_018011075.1PREDICTED: uncharacterized protein LOC108668383 isoform X1 | | |
| TRINITY_DN6836_c0_g1_i1_len_409_207641.p1_type_internal_len_136 | XP_018008078.1PREDICTED: uncharacterized protein KIAA0513-like | | |
| TRINITY_DN6734_c0_g1_i1_len_405_207800.p1_type_internal_len_136 | XP_011431192.2 kinesin-like protein KIF13A | F:GO:0003777; F:GO:0005524; C:GO:0005874; P:GO:0007018; F:GO:0008017 | EC:3.6.1.15 |
| TRINITY_DN23087_c0_g1_i1_len_2929_229087.p1_type_5prime_partial_len_898 | KZS18508.1Tolloid 2-like protein | F:GO:0004222; F:GO:0005509; P:GO:0006508; F:GO:0008270 | EC:3.4.24 |
| 2101\|_\|c10225_f3p51_2694_isoform_c10225_full_length_coverage_3_non_full_length_coverage_51_isoform_length_2694_21930 | ASC55678.1putative trypsin serine protease | F:GO:0004252; P:GO:0006508; F:GO:0008233 | |
| TRINITY_DN24699_c0_g1_i1_len_4040_228157.p1_type_5prime_partial_len_898 | XP_018015357.1PREDICTED: uncharacterized protein LOC108672229 isoform X3 | | |

| | | | |
|---|---|---|---|
| 2103\|_\|c1800_f6p6_2693_isoform_c1800_full_length_coverage_6_non_full_length_coverage_6_isoform_length_2693_11498 | XP_023702071.1polynucleotide 5'-hydroxyl-kinase NOL9 isoform X1 | F:GO:0000166; F:GO:0005524 | |
| 2104\|_\|c20175_f2p0_2693_isoform_c20175_full_length_coverage_2_non_full_length_coverage_0_isoform_length_2693_18175 | XP_018902772.1 high affinity copper uptake protein 1 isoform X2 | | |
| TRINITY_DN22023_c0_g1_i2_len_2891_154469.p1_type_complete_len_897 | XP_018014490.1PREDICTED: uncharacterized protein LOC108671453, partial | | |
| TRINITY_DN16079_c0_g1_i3_len_2693_91339.p1_type_internal_len_898 | PIK52130.1putative crossover junction endonuclease MUS81 | F:GO:0003677; F:GO:0004518; F:GO:0004519; P:GO:0090305 | |
| TRINITY_DN20583_c0_g1_i1_len_2694_91336.p1_type_internal_len_898 | XP_021928260.1puromycin-sensitive aminopeptidase isoform X2 | F:GO:0004177; P:GO:0006508; F:GO:0008237; F:GO:0008270 | EC:3.4.11 |
| TRINITY_DN21644_c0_g1_i1_len_2694_229440.p1_type_internal_len_898 | ---NA--- | | |
| TRINITY_DN24865_c0_g1_i1_len_3053_228939.p1_type_5prime_partial_len_897 | ---NA--- | | |
| TRINITY_DN19940_c0_g1_i1_len_525_203520.p1_type_5prime_partial_len_135 | ---NA--- | | |
| TRINITY_DN10610_c0_g1_i2_len_498_132395.p1_type_5prime_partial_len_135 | ---NA--- | | |
| TRINITY_DN11380_c0_g1_i1_len_503_69040.p1_type_3prime_partial_len_136 | XP_014782283.1PREDICTED: putative uncharacterized protein FLJ37770 | | |
| TRINITY_DN2932_c0_g1_i1_len_406_207785.p1_type_5prime_partial_len_135 | XP_011420820.1 protein ABHD4-like | | |
| TRINITY_DN35896_c0_g1_i1_len_546_202998.p1_type_3prime_partial_len_136 | ---NA--- | | |
| TRINITY_DN18077_c0_g1_i1_len_549_100307.p1_type_5prime_partial_len_135 | ---NA--- | | |
| TRINITY_DN17512_c0_g1_i2_len_403_41379.p1_type_internal_len_135 | ---NA--- | | |
| TRINITY_DN1545_c0_g1_i1_len_406_207759.p1_type_internal_len_135 | ---NA--- | | |

| | | | |
|---|---|---|---|
| TRINITY_DN7113_c0_g1_i1_len_404_207834.p1_type_internal_len_135 | XP_018012118.1 kinesin-like protein KIF21B | | |
| TRINITY_DN31787_c0_g1_i1_len_404_207841.p2_type_internal_len_135 | XP_018023076.1 putative mediator of RNA polymerase II transcription subunit 12 | | |
| TRINITY_DN15756_c0_g1_i1_len_594_240911.p1_type_complete_len_134 | ---NA--- | | |
| TRINITY_DN11729_c0_g1_i1_len_1048_62831.p1_type_complete_len_134 | ---NA--- | | |
| TRINITY_DN10191_c0_g1_i1_len_537_203222.p1_type_5prime_partial_len_134 | ---NA--- | | |
| TRINITY_DN28542_c0_g1_i1_len_595_240894.p1_type_5prime_partial_len_134 | XP_018020025.1 spermine oxidase-like | F:GO:0016491; P:GO:0030097; P:GO:0055114 | |
| TRINITY_DN5258_c0_g1_i1_len_756_97483.p1_type_5prime_partial_len_134 | ---NA--- | | |
| TRINITY_DN14932_c0_g1_i1_len_405_207819.p1_type_internal_len_135 | ---NA--- | | |
| TRINITY_DN11114_c0_g1_i1_len_406_207783.p1_type_internal_len_135 | ---NA--- | | |
| TRINITY_DN37787_c0_g1_i1_len_406_207778.p1_type_internal_len_135 | ---NA--- | | |
| TRINITY_DN17522_c2_g1_i1_len_824_271531.p1_type_complete_len_134 | ---NA--- | | |
| TRINITY_DN36128_c0_g1_i1_len_405_207796.p1_type_internal_len_135 | ---NA--- | | |
| TRINITY_DN35276_c0_g1_i1_len_404_207833.p1_type_internal_len_135 | AGK89908.1nicotinic acetylcholine receptor subunit alpha 3 | F:GO:0004888; C:GO:0016021; F:GO:0022848; C:GO:0030054; P:GO:0034220; C:GO:0045211; P:GO:0060079 | |
| TRINITY_DN30714_c0_g1_i1_len_5112_187098.p2_type_complete_len_134 | ---NA--- | | |
| TRINITY_DN5959_c0_g1_i1_len_405_41331.p1_type_internal_len_135 | ---NA--- | | |

| | | | |
|---|---|---|---|
| TRINITY_DN30682_c2_g1_i1_len_405_207826.p1_type_internal_len_135 | XP_018006762.1 longitudinals lacking protein, isoforms H/M/V-like isoform X3 | | |
| TRINITY_DN2793_c0_g1_i1_len_405_207823.p2_type_internal_len_135 | ---NA--- | | |
| TRINITY_DN20135_c0_g1_i1_len_538_303430.p1_type_3prime_partial_len_135 | ---NA--- | | |
| TRINITY_DN15883_c0_g1_i1_len_512_303922.p1_type_5prime_partial_len_134 | ---NA--- | | |
| TRINITY_DN11336_c0_g1_i1_len_404_104246.p1_type_5prime_partial_len_134 | ---NA--- | | |
| TRINITY_DN16835_c0_g1_i1_len_404_207846.p1_type_internal_len_135 | ---NA--- | | |
| TRINITY_DN10084_c0_g1_i2_len_402_104303.p1_type_internal_len_135 | ---NA--- | | |
| TRINITY_DN25113_c0_g2_i1_len_1861_191430.p1_type_complete_len_134 | ---NA--- | | |
| TRINITY_DN20039_c0_g1_i2_len_623_240404.p1_type_complete_len_134 | ---NA--- | | |
| TRINITY_DN2847_c0_g1_i2_len_727_238803.p2_type_complete_len_134 | ---NA--- | | |
| TRINITY_DN11480_c0_g1_i1_len_721_238889.p3_type_complete_len_134 | ---NA--- | | |
| TRINITY_DN7634_c0_g1_i1_len_404_207861.p2_type_internal_len_135 | ---NA--- | | |
| TRINITY_DN31294_c0_g1_i1_len_405_104207.p1_type_internal_len_135 | XP_018020560.1 vitelline membrane outer layer protein 1 homolog | | |
| TRINITY_DN24476_c0_g1_i1_len_1211_194869.p1_type_complete_len_134 | ---NA--- | | |
| TRINITY_DN25319_c0_g1_i1_len_404_104218.p1_type_internal_len_135 | ---NA--- | | |
| TRINITY_DN10587_c0_g1_i1_len_404_11560.p1_type_internal_len_135 | ---NA--- | | |

| | | | |
|---|---|---|---|
| TRINITY_DN17765_c0_g1_i1_len_2676_229471.p1_type_complete_len_134 | ---NA--- | | |
| TRINITY_DN23769_c0_g1_i1_len_417_169562.p1_type_5prime_partial_len_134 | ---NA--- | | |
| TRINITY_DN24774_c0_g1_i1_len_1485_193182.p2_type_complete_len_134 | ---NA--- | | |
| TRINITY_DN26123_c0_g1_i1_len_2260_230353.p3_type_complete_len_134 | ---NA--- | | |
| TRINITY_DN30799_c0_g1_i1_len_3984_187594.p4_type_complete_len_134 | ---NA--- | | |
| TRINITY_DN32591_c0_g1_i1_len_430_206725.p1_type_5prime_partial_len_134 | XP_018016072.1 active breakpoint cluster region-related protein-like | F:GO:0005096; P:GO:0007165; P:GO:0043547 | |
| TRINITY_DN24297_c0_g1_i1_len_406_306819.p1_type_internal_len_135 | XP_013164818.1 PH and SEC7 domain-containing protein 3 isoform X3 | F:GO:0005086; F:GO:0005543; P:GO:0032012 | |
| TRINITY_DN15658_c0_g1_i1_len_406_306816.p1_type_internal_len_135 | ---NA--- | | |
| TRINITY_DN11023_c0_g1_i2_len_404_306886.p2_type_internal_len_135 | ---NA--- | | |
| TRINITY_DN3819_c0_g1_i1_len_406_169994.p1_type_internal_len_135 | ---NA--- | | |
| TRINITY_DN12076_c0_g1_i1_len_404_306878.p1_type_internal_len_135 | ---NA--- | | |
| TRINITY_DN6955_c0_g1_i1_len_403_207891.p1_type_internal_len_135 | ---NA--- | | |
| TRINITY_DN15227_c0_g2_i1_len_403_207887.p1_type_internal_len_135 | AYC12381.1bone morphogenetic protein receptor type 1B | F:GO:0004672; F:GO:0005488; P:GO:0009987 | |
| TRINITY_DN17561_c0_g1_i1_len_403_207882.p1_type_internal_len_135 | ---NA--- | | |
| TRINITY_DN40583_c0_g1_i1_len_403_207881.p1_type_internal_len_135 | ---NA--- | | |
| TRINITY_DN2842_c0_g1_i1_len_403_207872.p1_type_internal_len_135 | ---NA--- | | |

| | | | |
|---|---|---|---|
| TRINITY_DN32846_c0_g1_i1_len_403_207871.p2_type_internal_len_135 | ---NA--- | | |
| TRINITY_DN22484_c0_g1_i1_len_1687_232181.p2_type_complete_len_134 | PIK44577.1putative retrovirus-related Pol polyprotein from transposon | | |
| TRINITY_DN23682_c0_g1_i1_len_583_165311.p1_type_5prime_partial_len_134 | ---NA--- | | |
| TRINITY_DN19091_c0_g1_i1_len_402_207905.p1_type_internal_len_135 | ---NA--- | | |
| TRINITY_DN4486_c0_g1_i1_len_403_207899.p2_type_internal_len_135 | XP_018011644.1 irregular chiasm C-roughest protein-like | C:GO:0016020; C:GO:0016021 | |
| TRINITY_DN34752_c0_g1_i1_len_403_207896.p1_type_internal_len_135 | ---NA--- | | |
| TRINITY_DN1795_c0_g1_i1_len_710_97969.p1_type_3prime_partial_len_135 | ---NA--- | | |
| TRINITY_DN39790_c0_g1_i1_len_403_207869.p1_type_internal_len_135 | XP_023245886.1protein furry | | |
| TRINITY_DN14831_c0_g1_i1_len_827_271498.p1_type_complete_len_134 | EEC03121.1NADH:ubiquinone oxidoreductase, NDUFC2/B14.5B subunit, putative | C:GO:0005739; C:GO:0044425 | |
| TRINITY_DN22926_c0_g1_i1_len_1366_61205.p2_type_5prime_partial_len_134 | ---NA--- | | |
| TRINITY_DN12054_c0_g1_i1_len_519_38313.p1_type_5prime_partial_len_134 | ---NA--- | | |
| TRINITY_DN14428_c0_g1_i1_len_505_166956.p1_type_5prime_partial_len_134 | ---NA--- | | |
| TRINITY_DN23906_c0_g1_i1_len_1367_193852.p1_type_complete_len_134 | ---NA--- | | |
| TRINITY_DN13243_c0_g1_i2_len_1299_234056.p1_type_3prime_partial_len_135 | AOD27420.1transcription factor CSL | F:GO:0000978; F:GO:0001228; C:GO:0005634; P:GO:0045944 | |
| TRINITY_DN31148_c0_g1_i1_len_435_10562.p1_type_5prime_partial_len_134 | ---NA--- | | |
| TRINITY_DN35601_c0_g1_i1_len_402_207938.p1_type_internal_len_135 | XP_014678859.1 probable helicase with zinc finger domain | F:GO:0004386; F:GO:0046872 | |

| | | | |
|---|---|---|---|
| TRINITY_DN20847_c0_g1_i3_len_450_10100.p1_type_5prime_partial_len_134 | ---NA--- | | |
| TRINITY_DN4389_c0_g1_i1_len_402_207944.p1_type_internal_len_135 | ---NA--- | | |
| TRINITY_DN31288_c0_g1_i1_len_463_205437.p1_type_complete_len_134 | ---NA--- | | |
| TRINITY_DN16162_c0_g1_i1_len_711_199825.p2_type_3prime_partial_len_135 | ---NA--- | | |
| TRINITY_DN3386_c0_g1_i1_len_402_246306.p1_type_internal_len_135 | ---NA--- | | |
| TRINITY_DN14744_c0_g1_i1_len_402_246300.p1_type_internal_len_135 | ---NA--- | | |
| TRINITY_DN7643_c0_g1_i1_len_403_246289.p1_type_internal_len_135 | PNF21610.1Autophagy-related protein 2-like protein B | P:GO:0006914 | |
| TRINITY_DN6443_c0_g1_i1_len_403_246272.p1_type_internal_len_135 | ---NA--- | | |
| TRINITY_DN23460_c0_g1_i2_len_1020_196393.p1_type_5prime_partial_len_134 | ODM97492.1Carboxypeptidase B | F:GO:0004180 | |
| TRINITY_DN12469_c0_g1_i2_len_414_71776.p1_type_5prime_partial_len_134 | ---NA--- | | |
| TRINITY_DN4448_c0_g1_i1_len_404_72159.p1_type_internal_len_135 | PNF27304.1Oxysterol-binding protein-related protein 8 | P:GO:0006869; C:GO:0016021 | |
| TRINITY_DN11217_c0_g1_i1_len_447_244648.p1_type_5prime_partial_len_134 | ---NA--- | | |
| TRINITY_DN23546_c0_g1_i1_len_404_278809.p1_type_internal_len_135 | ---NA--- | | |
| TRINITY_DN21555_c0_g1_i1_len_404_278838.p1_type_internal_len_135 | XP_018007219.1 2-hydroxy-3-oxopropionate reductase-like | | |
| TRINITY_DN24610_c0_g1_i1_len_405_278770.p1_type_internal_len_135 | ---NA--- | | |
| TRINITY_DN16524_c0_g1_i1_len_405_278772.p1_type_internal_len_135 | CDI59403.1Histone 1 | C:GO:0000786; F:GO:0003677; C:GO:0005634; P:GO:0006334 | |

| | | | |
|---|---|---|---|
| TRINITY_DN26143_c0_g1_i1_len_405_278790.p1_type_internal_len_135 | ---NA--- | | |
| TRINITY_DN20884_c0_g1_i1_len_1888_156441.p4_type_5prime_partial_len_134 | ---NA--- | | |
| TRINITY_DN30775_c0_g1_i1_len_458_244292.p1_type_3prime_partial_len_135 | ---NA--- | | |
| TRINITY_DN3732_c0_g1_i1_len_402_278908.p1_type_internal_len_135 | ---NA--- | | |
| TRINITY_DN11503_c0_g1_i1_len_463_244147.p1_type_5prime_partial_len_134 | ---NA--- | | |
| TRINITY_DN9439_c0_g1_i2_len_463_244122.p1_type_5prime_partial_len_134 | XP_015510998.1 IDLSRF-like peptide | | |
| TRINITY_DN21534_c0_g1_i1_len_535_203295.p1_type_5prime_partial_len_134 | ACD36030.1spatzle protein | | |
| TRINITY_DN8678_c0_g1_i3_len_403_278849.p1_type_internal_len_135 | ---NA--- | | |
| TRINITY_DN23739_c0_g1_i1_len_403_278865.p1_type_3prime_partial_len_135 | ---NA--- | | |
| TRINITY_DN5467_c0_g1_i1_len_405_246219.p2_type_internal_len_135 | ---NA--- | | |
| TRINITY_DN6823_c0_g1_i1_len_405_246208.p1_type_internal_len_135 | ---NA--- | | |
| TRINITY_DN473_c0_g1_i1_len_405_246204.p1_type_internal_len_135 | ---NA--- | | |
| TRINITY_DN33586_c0_g1_i1_len_405_246193.p1_type_internal_len_135 | OWK10999.1hypothetical protein Celaphus_00007046, partial | C:GO:0005623; P:GO:0006886 | |
| 2111\|_\|c5996_f2p0_2690_isoform_c5996_full_length_coverage_2_non_full_length_coverage_0_isoform_length_2690_17012 | ---NA--- | | |
| 2112\|_\|c4425_f2p5_2690_isoform_c4425_full_length_coverage_2_non_full_length_coverage_5_isoform_length_2690_12380 | XP_017882140.1adenylosuccinate lyase | | |

| | | | |
|---|---|---|---|
| 2113\|_\|c4346_f2p0_2690_isoform_c4346_full_length_coverage_2_non_full_length_coverage_0_isoform_length_2690_12369 | XP_023288319.1prolyl 4-hydroxylase subunit alpha-2 | | |
| 2114\|_\|c18209_f2p1_2689_isoform_c18209_full_length_coverage_2_non_full_length_coverage_1_isoform_length_2689_13647 | ---NA--- | | |
| 2115\|_\|c2451_f5p2_2689_isoform_c2451_full_length_coverage_5_non_full_length_coverage_2_isoform_length_2689_20947 | XP_021927433.1postreplication repair E3 ubiquitin-protein ligase rad18-like isoform X2 | P:GO:0006807; P:GO:0044238; P:GO:0044260 | |
| TRINITY_DN24541_c0_g1_i2_len_2969_229035.p1_type_complete_len_896 | XP_018018096.1 exocyst complex component 1-like | C:GO:0000145; P:GO:0006887 | |
| 2117\|_\|c5213_f2p1_2688_isoform_c5213_full_length_coverage_2_non_full_length_coverage_1_isoform_length_2688_12454 | ---NA--- | | |
| 2119\|_\|c18240_f2p0_2687_isoform_c18240_full_length_coverage_2_non_full_length_coverage_0_isoform_length_2687_18054 | XP_018007798.1 guanine deaminase-like | F:GO:0016810 | |
| 2120\|_\|c17862_f4p1_2686_isoform_c17862_full_length_coverage_4_non_full_length_coverage_1_isoform_length_2686_13447 | KZS05748.1Separase/extra spindle poles I-like protein | C:GO:0005634; P:GO:0006508; F:GO:0008233 | |
| TRINITY_DN23404_c0_g1_i2_len_2923_122168.p1_type_5prime_partial_len_895 | XP_018020308.1 kinesin-like protein KIF23 isoform X1 | F:GO:0005488; P:GO:0009987 | |
| TRINITY_DN32722_c0_g1_i1_len_405_246188.p1_type_internal_len_135 | ---NA--- | | |
| TRINITY_DN27166_c0_g1_i1_len_405_246182.p1_type_internal_len_135 | ---NA--- | | |
| TRINITY_DN9400_c0_g1_i1_len_404_246256.p1_type_internal_len_135 | XP_015925854.1spermine oxidase isoform X3 | | |
| TRINITY_DN5948_c0_g1_i1_len_404_246253.p1_type_internal_len_135 | XP_018007608.1 protein PTHB1-like isoform X2 | | |
| TRINITY_DN36992_c0_g1_i1_len_404_246252.p1_type_internal_len_135 | ---NA--- | | |

| | | | |
|---|---|---|---|
| TRINITY_DN17140_c1_g1_i1_len_404_246247.p3_type_internal_len_135 | ---NA--- | | |
| TRINITY_DN37366_c0_g1_i1_len_404_246242.p1_type_internal_len_135 | ---NA--- | | |
| TRINITY_DN33646_c0_g1_i1_len_404_246239.p1_type_internal_len_135 | XP_013139704.1 semaphorin-5B | | |
| TRINITY_DN31952_c0_g1_i1_len_404_246228.p1_type_internal_len_135 | XP_022235408.1myotubularin-related protein 4-like | P:GO:0016311; F:GO:0016791 | |
| TRINITY_DN27861_c0_g1_i1_len_404_246225.p1_type_internal_len_135 | ---NA--- | | |
| TRINITY_DN32097_c0_g1_i1_len_404_246223.p1_type_internal_len_135 | AMD09631.1glutamine synthetase | F:GO:0004356; F:GO:0005524; P:GO:0006542 | EC:6.3.1.2 |
| TRINITY_DN18147_c0_g1_i2_len_533_203344.p1_type_complete_len_134 | ---NA--- | | |
| TRINITY_DN13907_c0_g1_i1_len_439_244918.p1_type_5prime_partial_len_134 | ADN43412.1hemocyte homeostasis-associated protein | | |
| TRINITY_DN36033_c0_g1_i1_len_406_246153.p1_type_internal_len_135 | ---NA--- | | |
| TRINITY_DN13773_c0_g1_i2_len_406_246144.p1_type_internal_len_135 | ---NA--- | | |
| TRINITY_DN20990_c0_g1_i7_len_1558_1527.p2_type_5prime_partial_len_134 | ---NA--- | | |
| TRINITY_DN14511_c0_g1_i1_len_420_245635.p1_type_5prime_partial_len_134 | ---NA--- | | |
| TRINITY_DN6558_c0_g1_i1_len_539_131501.p1_type_5prime_partial_len_134 | XP_018009697.1 DNA translocase FtsK-like | F:GO:0042302 | |
| TRINITY_DN27832_c0_g1_i1_len_520_131910.p1_type_complete_len_134 | KFP49773.1Tripartite motif-containing protein 45 | F:GO:0008270 | |
| TRINITY_DN21537_c0_g1_i1_len_652_129574.p2_type_5prime_partial_len_134 | ---NA--- | | |
| TRINITY_DN27208_c0_g1_i1_len_870_197808.p1_type_5prime_partial_len_134 | ---NA--- | | |

| | | | |
|---|---|---|---|
| TRINITY_DN19824_c0_g1_i1_len_2073_92018.p1_type_5prime_partial_len_134 | ---NA--- | | |
| TRINITY_DN12958_c0_g1_i1_len_455_133501.p1_type_5prime_partial_len_134 | ---NA--- | | |
| TRINITY_DN7326_c0_g1_i1_len_1298_32134.p2_type_complete_len_134 | ---NA--- | | |
| TRINITY_DN163_c0_g1_i2_len_505_204065.p1_type_5prime_partial_len_134 | ---NA--- | | |
| TRINITY_DN18370_c0_g1_i4_len_1347_31964.p2_type_5prime_partial_len_134 | ---NA--- | | |
| TRINITY_DN23177_c1_g1_i3_len_491_204534.p1_type_5prime_partial_len_134 | ---NA--- | | |
| TRINITY_DN624_c0_g1_i1_len_475_205060.p1_type_5prime_partial_len_134 | ---NA--- | | |
| TRINITY_DN26372_c0_g1_i1_len_404_72183.p1_type_internal_len_135 | KFM63422.1putative inorganic phosphate cotransporter | C:GO:0016020; C:GO:0016021; P:GO:0055085 | |
| TRINITY_DN17718_c0_g2_i1_len_464_276942.p1_type_5prime_partial_len_134 | ---NA--- | | |
| TRINITY_DN8724_c0_g1_i1_len_403_72239.p1_type_internal_len_135 | ---NA--- | | |
| TRINITY_DN656_c0_g1_i1_len_403_135296.p1_type_internal_len_135 | ---NA--- | | |
| TRINITY_DN30611_c0_g1_i1_len_405_135199.p1_type_internal_len_135 | XP_013399086.1guanine deaminase-like isoform X2 | F:GO:0016810 | |
| TRINITY_DN15104_c0_g1_i1_len_476_205013.p1_type_5prime_partial_len_134 | ---NA--- | | |
| TRINITY_DN7455_c0_g1_i1_len_402_135309.p1_type_internal_len_135 | ---NA--- | | |
| TRINITY_DN5209_c0_g1_i1_len_564_202565.p2_type_5prime_partial_len_134 | ---NA--- | | |
| TRINITY_DN10352_c1_g1_i1_len_455_205779.p1_type_3prime_partial_len_135 | ---NA--- | | |

| | | | |
|---|---|---|---|
| TRINITY_DN19356_c0_g1_i2_len_460_39645.p1_type_5prime_partial_len_134 | ---NA--- | | |
| TRINITY_DN20379_c0_g1_i1_len_1607_157390.p2_type_5prime_partial_len_134 | ---NA--- | | |
| TRINITY_DN1931_c0_g1_i1_len_609_201587.p1_type_5prime_partial_len_134 | ---NA--- | | |
| TRINITY_DN16247_c0_g1_i2_len_401_104343.p1_type_internal_len_134 | ---NA--- | | |
| TRINITY_DN27640_c0_g1_i1_len_680_35955.p2_type_5prime_partial_len_133 | XP_018023503.1 V-type proton ATPase subunit F-like | P:GO:0015991; C:GO:0033180; F:GO:0046961 | EC:3.6.1.3; EC:3.6.1.15 |
| TRINITY_DN33277_c0_g1_i1_len_399_208054.p1_type_internal_len_134 | KFM71705.1Rho guanine nucleotide exchange factor 17 | F:GO:0005089 | |
| TRINITY_DN15202_c0_g1_i1_len_482_204813.p1_type_5prime_partial_len_133 | ---NA--- | | |
| TRINITY_DN24721_c0_g1_i1_len_1288_194335.p1_type_complete_len_133 | ---NA--- | | |
| TRINITY_DN18412_c0_g1_i2_len_681_35950.p1_type_complete_len_133 | XP_018026823.1 intraflagellar transport protein 20 homolog | | |
| TRINITY_DN26277_c0_g1_i1_len_1278_2347.p3_type_3prime_partial_len_134 | ---NA--- | | |
| TRINITY_DN18333_c0_g1_i1_len_400_208019.p1_type_internal_len_134 | XP_018008386.1 myoferlin-like | C:GO:0016021 | |
| TRINITY_DN6602_c0_g1_i1_len_400_208023.p1_type_internal_len_134 | XP_021942758.1C2 domain-containing protein 5 isoform X5 | F:GO:0005544 | |
| TRINITY_DN14936_c0_g1_i1_len_400_208025.p1_type_internal_len_134 | ---NA--- | | |
| TRINITY_DN11787_c0_g1_i1_len_505_132245.p1_type_complete_len_133 | XP_023701473.1zinc metalloproteinase nas-13-like | F:GO:0008233 | |
| TRINITY_DN35526_c0_g1_i1_len_399_208071.p1_type_internal_len_134 | ---NA--- | | |
| TRINITY_DN2435_c0_g1_i1_len_399_208080.p1_type_internal_len_134 | ---NA--- | | |

| | | | |
|---|---|---|---|
| TRINITY_DN35287_c0_g1_i1_len_399_208081.p1_type_internal_len_134 | XP_012229050.1 epidermal growth factor receptor | F:GO:0004714; F:GO:0005524; P:GO:0007169; C:GO:0016021; P:GO:0018108 | EC:2.7.10; EC:2.7.10.1; EC:2.7.11 |
| TRINITY_DN19228_c0_g1_i1_len_469_205234.p1_type_3prime_partial_len_134 | ---NA--- | | |
| TRINITY_DN20255_c0_g1_i2_len_459_9834.p1_type_5prime_partial_len_133 | ---NA--- | | |
| TRINITY_DN18966_c0_g1_i1_len_401_207980.p1_type_internal_len_134 | ---NA--- | | |
| TRINITY_DN37701_c0_g1_i1_len_401_207984.p1_type_internal_len_134 | XP_018011765.1PREDICTED: uncharacterized protein LOC108668992 | | |
| TRINITY_DN18513_c0_g2_i1_len_635_6484.p1_type_complete_len_133 | ---NA--- | | |
| TRINITY_DN21839_c0_g1_i1_len_1359_193907.p1_type_complete_len_133 | ---NA--- | | |
| TRINITY_DN4161_c0_g1_i1_len_399_41500.p1_type_internal_len_134 | XP_018022161.1 histone-lysine N-methyltransferase, H3 lysine-79 specific-like isoform X2 | | |
| TRINITY_DN27315_c0_g1_i1_len_401_104361.p1_type_internal_len_134 | ---NA--- | | |
| TRINITY_DN35468_c0_g1_i1_len_401_207986.p1_type_internal_len_134 | ---NA--- | | |
| TRINITY_DN23936_c0_g1_i1_len_1337_233825.p2_type_complete_len_133 | ---NA--- | | |
| TRINITY_DN7519_c0_g1_i1_len_456_133491.p1_type_5prime_partial_len_133 | XP_023346423.1transforming growth factor-beta-induced protein ig-h3-like | | |
| TRINITY_DN26232_c0_g1_i1_len_401_104348.p1_type_internal_len_134 | ---NA--- | | |
| TRINITY_DN34541_c0_g1_i1_len_400_207996.p2_type_internal_len_134 | XP_018015479.1PREDICTED: uncharacterized protein LOC108672345 isoform X1 | | |
| TRINITY_DN30911_c0_g1_i1_len_4224_187468.p3_type_complete_len_133 | ---NA--- | | |

| | | | |
|---|---|---|---|
| TRINITY_DN36667_c0_g1_i1_len_401_207990.p1_type_internal_len_134 | ---NA--- | | |
| TRINITY_DN9654_c0_g1_i2_len_401_135360.p1_type_5prime_partial_len_133 | ---NA--- | | |
| TRINITY_DN30947_c0_g1_i2_len_3319_188115.p3_type_complete_len_133 | ACD74843.1trehalose-6-phosphate synthase | F:GO:0003825; P:GO:0005992 | EC:2.4.1.15 |
| TRINITY_DN23029_c0_g1_i1_len_2626_229558.p2_type_complete_len_133 | ---NA--- | | |
| TRINITY_DN28687_c0_g1_i1_len_3278_188159.p2_type_complete_len_133 | ---NA--- | | |
| TRINITY_DN25117_c0_g1_i1_len_2537_229710.p1_type_5prime_partial_len_133 | ---NA--- | | |
| TRINITY_DN31537_c0_g1_i1_len_401_170204.p1_type_internal_len_134 | ---NA--- | | |
| TRINITY_DN24168_c0_g1_i1_len_402_170157.p1_type_internal_len_134 | ---NA--- | | |
| TRINITY_DN19931_c1_g1_i3_len_400_307052.p2_type_internal_len_134 | ---NA--- | | |
| TRINITY_DN3594_c0_g1_i1_len_652_6283.p1_type_5prime_partial_len_133 | ---NA--- | | |
| TRINITY_DN27003_c0_g1_i1_len_462_102296.p1_type_5prime_partial_len_133 | ALC78853.1adenosine monophosphate deaminase 2 | | |
| TRINITY_DN14437_c0_g1_i1_len_401_135372.p1_type_internal_len_134 | ---NA--- | | |
| TRINITY_DN26422_c0_g1_i1_len_401_306983.p1_type_internal_len_134 | ---NA--- | | |
| TRINITY_DN24033_c0_g1_i1_len_400_41468.p1_type_internal_len_134 | ---NA--- | | |
| TRINITY_DN9217_c0_g1_i1_len_402_306965.p1_type_internal_len_134 | ---NA--- | | |
| TRINITY_DN16572_c0_g1_i1_len_923_4028.p2_type_5prime_partial_len_133 | XP_013399963.1MOB-like protein phocein | F:GO:0016301; P:GO:0016310 | |

| | | | |
|---|---|---|---|
| TRINITY_DN12933_c0_g1_i1_len_401_306995.p1_type_internal_len_134 | ---NA--- | | |
| TRINITY_DN6225_c0_g1_i1_len_401_135380.p1_type_internal_len_134 | XP_018011683.1 tyrosine-protein phosphatase non-receptor type 23-like | F:GO:0004725; P:GO:0035335 | EC:3.1.3.16; EC:3.1.3.48 |
| TRINITY_DN15079_c0_g1_i1_len_943_197053.p3_type_5prime_partial_len_133 | ---NA--- | | |
| TRINITY_DN538_c0_g1_i1_len_520_203623.p1_type_5prime_partial_len_133 | ---NA--- | | |
| TRINITY_DN12491_c0_g1_i1_len_533_242171.p1_type_5prime_partial_len_133 | ---NA--- | | |
| TRINITY_DN6717_c0_g1_i1_len_971_196791.p1_type_3prime_partial_len_134 | ---NA--- | | |
| TRINITY_DN4231_c0_g1_i1_len_402_135329.p1_type_internal_len_134 | ---NA--- | | |
| TRINITY_DN2586_c0_g1_i1_len_399_135467.p1_type_internal_len_134 | XP_018013636.1 FERM domain-containing protein 8-like | | |
| TRINITY_DN1696_c0_g1_i1_len_400_11730.p1_type_internal_len_134 | ---NA--- | | |
| TRINITY_DN16752_c0_g1_i1_len_1187_125481.p2_type_complete_len_133 | ---NA--- | | |
| TRINITY_DN17361_c0_g1_i1_len_630_240282.p1_type_5prime_partial_len_133 | ---NA--- | | |
| TRINITY_DN23402_c0_g1_i1_len_399_11765.p1_type_internal_len_134 | ---NA--- | | |
| TRINITY_DN10883_c0_g1_i1_len_406_207775.p1_type_5prime_partial_len_133 | ---NA--- | | |
| TRINITY_DN13586_c0_g1_i1_len_402_72270.p1_type_internal_len_134 | ---NA--- | | |
| TRINITY_DN8909_c1_g1_i1_len_401_207948.p5_type_internal_len_134 | ---NA--- | | |
| TRINITY_DN36973_c0_g1_i1_len_401_207949.p1_type_3prime_partial_len_134 | XP_022317618.1 midasin-like | F:GO:0005524; F:GO:0016887 | |

| | | | |
|---|---|---|---|
| 2124\|_\|c3366_f3p1_2685_isoform_c3366_full_length_coverage_3_non_full_length_coverage_1_isoform_length_2685_21340 | XP_018010190.1 dynactin subunit 4-like | C:GO:0005869 | |
| 2125\|_\|c4181_f2p4_2685_isoform_c4181_full_length_coverage_2_non_full_length_coverage_4_isoform_length_2685_12359 | ---NA--- | | |
| 2127\|_\|c18083_f2p1_2683_isoform_c18083_full_length_coverage_2_non_full_length_coverage_1_isoform_length_2683_22678 | XP_018009609.1 condensin-2 complex subunit G2-like | C:GO:0005634 | |
| 2128\|_\|c18096_f2p0_2682_isoform_c18096_full_length_coverage_2_non_full_length_coverage_0_isoform_length_2682_13574 | XP_019638837.1 lon protease homolog 2, peroxisomal-like | | |
| 2129\|_\|c2579_f2p0_2682_isoform_c2579_full_length_coverage_2_non_full_length_coverage_0_isoform_length_2682_11985 | XP_018019753.1 gephyrin-like | F:GO:0016740; P:GO:0044267 | |
| 2130\|_\|c9902_f2p0_2682_isoform_c9902_full_length_coverage_2_non_full_length_coverage_0_isoform_length_2682_21901 | XP_023707594.1rho guanine nucleotide exchange factor 10 isoform X5 | F:GO:0005089 | |
| 2131\|_\|c2416_f2p3_2681_isoform_c2416_full_length_coverage_2_non_full_length_coverage_3_isoform_length_2681_16394 | BAJ78981.1heat shock protein 40 | F:GO:0005524; P:GO:0006457; P:GO:0009408; F:GO:0031072; F:GO:0046872; F:GO:0051082 | |
| 2132\|_\|c1655_f2p2_2681_isoform_c1655_full_length_coverage_2_non_full_length_coverage_2_isoform_length_2681_26009 | XP_026279122.1uncharacterized protein C1orf112-like | | |
| 2133\|_\|c29016_f2p1_2681_isoform_c29016_full_length_coverage_2_non_full_length_coverage_1_isoform_length_2681_18481 | XP_018026342.1 zinc finger protein 3 homolog | | |
| 2134\|_\|c2552_f3p0_2681_isoform_c2552_full_length_coverage_3_non_full_length_coverage_0_isoform_length_2681_16505 | XP_018007297.1 TBC1 domain family member 15-like isoform X2 | | |
| TRINITY_DN31837_c0_g1_i1_len_401_207953.p1_type_internal_len_134 | XP_020715811.1oxysterol-binding protein-related protein 8 isoform X6 | P:GO:0006869; C:GO:0016021 | |

| | | | |
|---|---|---|---|
| TRINITY_DN32214_c0_g1_i1_len_401_207955.p1_type_internal_len_134 | ---NA--- | | |
| TRINITY_DN20470_c0_g1_i2_len_431_10693.p1_type_3prime_partial_len_134 | XP_015610268.1histone H2B | | |
| TRINITY_DN41590_c0_g1_i1_len_401_207945.p1_type_internal_len_134 | XP_018023567.1 regulation of nuclear pre-mRNA domain-containing protein 1B-like | | |
| TRINITY_DN17656_c0_g1_i1_len_1163_195214.p3_type_complete_len_133 | ---NA--- | | |
| TRINITY_DN17147_c0_g1_i2_len_434_10617.p1_type_5prime_partial_len_133 | ---NA--- | | |
| TRINITY_DN17728_c0_g1_i1_len_402_72281.p1_type_internal_len_134 | ---NA--- | | |
| TRINITY_DN1693_c0_g1_i1_len_401_207972.p1_type_internal_len_134 | XP_019864802.1 sushi, von Willebrand factor type A, EGF and pentraxin domain-containing protein 1-like | | |
| TRINITY_DN8528_c0_g1_i1_len_401_207977.p1_type_internal_len_134 | XP_023932628.1uncharacterized protein LOC106177003 | F:GO:0003676 | |
| TRINITY_DN20217_c0_g2_i1_len_400_135397.p1_type_internal_len_134 | XP_018019054.1 alpha-L-fucosidase-like | | |
| TRINITY_DN15114_c0_g1_i1_len_817_198413.p2_type_complete_len_133 | XP_018009778.1 alpha-(1,3)-fucosyltransferase 4-like | | |
| TRINITY_DN960_c0_g1_i1_len_445_10241.p1_type_5prime_partial_len_133 | ---NA--- | | |
| TRINITY_DN3245_c0_g1_i1_len_401_207965.p1_type_internal_len_134 | ---NA--- | | |
| TRINITY_DN29866_c0_g1_i1_len_403_72230.p1_type_internal_len_134 | XP_022202585.1zinc finger protein 2 homolog isoform X1 | | |
| TRINITY_DN2312_c0_g1_i1_len_403_72221.p1_type_internal_len_134 | ---NA--- | | |
| TRINITY_DN40017_c0_g1_i1_len_401_207966.p1_type_internal_len_134 | ---NA--- | | |
| TRINITY_DN30069_c0_g1_i1_len_450_133634.p1_type_3prime_partial_len_134 | ---NA--- | | |

| | | | |
|---|---|---|---|
| TRINITY_DN7090_c0_g1_i1_len_419_134706.p1_type_5prime_partial_len_133 | ---NA--- | | |
| TRINITY_DN37204_c0_g1_i1_len_415_207368.p1_type_5prime_partial_len_133 | EEC11289.1polyprotein of retroviral origin, putative | F:GO:0016740 | |
| TRINITY_DN822_c0_g1_i1_len_402_207913.p1_type_internal_len_134 | ---NA--- | | |
| TRINITY_DN21131_c0_g1_i1_len_732_199558.p1_type_5prime_partial_len_133 | ---NA--- | | |
| TRINITY_DN33642_c0_g1_i1_len_403_207868.p1_type_internal_len_134 | XP_018026383.1PREDICTED: uncharacterized protein KIAA1109-like | | |
| TRINITY_DN25867_c0_g1_i1_len_2127_230713.p4_type_complete_len_133 | ---NA--- | | |
| TRINITY_DN21577_c1_g1_i1_len_438_134027.p1_type_5prime_partial_len_133 | PRD21701.1zinc finger protein | F:GO:0003676 | |
| TRINITY_DN20629_c0_g1_i1_len_2790_91276.p4_type_complete_len_133 | ---NA--- | | |
| TRINITY_DN24857_c0_g1_i1_len_1726_232038.p1_type_5prime_partial_len_133 | ---NA--- | | |
| TRINITY_DN20237_c0_g1_i1_len_402_207936.p1_type_internal_len_134 | ---NA--- | | |
| TRINITY_DN6328_c0_g1_i1_len_400_72365.p1_type_internal_len_134 | ---NA--- | | |
| TRINITY_DN26745_c0_g1_i1_len_400_72359.p1_type_internal_len_134 | ---NA--- | | |
| TRINITY_DN40430_c0_g1_i1_len_402_207941.p1_type_internal_len_134 | ---NA--- | | |
| TRINITY_DN24077_c0_g1_i1_len_399_41518.p1_type_internal_len_134 | ---NA--- | | |
| TRINITY_DN3848_c0_g1_i1_len_400_72347.p2_type_internal_len_134 | ---NA--- | | |
| TRINITY_DN21440_c0_g1_i2_len_648_66364.p1_type_5prime_partial_len_133 | ---NA--- | | |

| | | | |
|---|---|---|---|
| TRINITY_DN30990_c0_g1_i1_len_652_66311.p1_type_5prime_partial_len_133 | ---NA--- | | |
| TRINITY_DN9211_c0_g1_i1_len_544_166065.p1_type_5prime_partial_len_133 | ---NA--- | | |
| TRINITY_DN34858_c0_g1_i1_len_988_236208.p1_type_5prime_partial_len_133 | ---NA--- | | |
| TRINITY_DN19263_c0_g1_i1_len_700_98090.p2_type_complete_len_133 | ---NA--- | | |
| TRINITY_DN15741_c0_g1_i1_len_843_127666.p1_type_3prime_partial_len_134 | ---NA--- | | |
| TRINITY_DN3510_c0_g1_i1_len_640_301816.p2_type_5prime_partial_len_133 | ---NA--- | | |
| TRINITY_DN4663_c0_g1_i1_len_458_205635.p1_type_3prime_partial_len_134 | ---NA--- | | |
| TRINITY_DN4084_c0_g1_i1_len_489_9069.p1_type_5prime_partial_len_133 | ALJ10568.1rhodanese 1-like protein | | |
| TRINITY_DN8833_c0_g1_i1_len_2182_155636.p2_type_3prime_partial_len_134 | EEB10598.1Proliferation-associated protein 2G4, putative | F:GO:0004177; P:GO:0006508 | EC:3.4.11 |
| TRINITY_DN24428_c0_g1_i1_len_1753_59910.p2_type_5prime_partial_len_133 | KOX68921.1DNA-directed RNA polymerase III subunit RPC10 | F:GO:0003676; F:GO:0003899; C:GO:0005730; P:GO:0006351; F:GO:0008270; P:GO:0042779; P:GO:0061484 | EC:2.7.7.6 |
| TRINITY_DN7231_c0_g1_i1_len_502_204177.p1_type_5prime_partial_len_133 | ---NA--- | | |
| TRINITY_DN13604_c0_g1_i1_len_408_246092.p1_type_5prime_partial_len_133 | ---NA--- | | |
| TRINITY_DN18272_c0_g1_i2_len_528_8248.p1_type_5prime_partial_len_133 | ---NA--- | | |
| TRINITY_DN18272_c0_g1_i2_len_528_8248.p2_type_complete_len_133 | ---NA--- | | |
| TRINITY_DN4044_c0_g1_i1_len_401_278922.p1_type_internal_len_134 | XP_008480027.1synaptotagmin-like protein 5 | C:GO:0016020 | |

| | | | |
|---|---|---|---|
| TRINITY_DN30835_c0_g1_i1_len_402_278890.p1_type_internal_len_134 | PNF23018.1BTB/POZ domain-containing protein KCTD3 | P:GO:0051260 | |
| TRINITY_DN23056_c0_g1_i1_len_516_8490.p1_type_5prime_partial_len_133 | ---NA--- | | |
| TRINITY_DN8878_c0_g1_i3_len_616_273901.p1_type_5prime_partial_len_133 | XP_018028226.1 protein ecdysoneless homolog | | |
| TRINITY_DN6174_c0_g1_i1_len_399_279009.p1_type_internal_len_134 | ---NA--- | | |
| TRINITY_DN22986_c0_g1_i3_len_1216_159227.p1_type_complete_len_133 | ---NA--- | | |
| TRINITY_DN15017_c0_g1_i1_len_637_164430.p1_type_complete_len_133 | ---NA--- | | |
| TRINITY_DN10352_c1_g1_i1_len_455_205779.p2_type_complete_len_133 | ---NA--- | | |
| TRINITY_DN36332_c0_g1_i1_len_438_206405.p1_type_3prime_partial_len_134 | ---NA--- | | |
| TRINITY_DN16814_c0_g1_i2_len_839_198151.p1_type_complete_len_133 | ---NA--- | | |
| TRINITY_DN16662_c0_g1_i1_len_669_200481.p1_type_5prime_partial_len_133 | ---NA--- | | |
| TRINITY_DN23779_c0_g1_i1_len_1457_60858.p2_type_complete_len_133 | ---NA--- | | |
| TRINITY_DN9633_c0_g1_i1_len_774_64837.p2_type_5prime_partial_len_133 | AUW69684.1spenito, partial | F:GO:0003723 | |
| TRINITY_DN29432_c0_g1_i2_len_835_198203.p1_type_5prime_partial_len_133 | ---NA--- | | |
| TRINITY_DN10798_c0_g1_i1_len_402_278881.p1_type_internal_len_134 | XP_013399086.1guanine deaminase-like isoform X2 | P:GO:0006147; F:GO:0008270; F:GO:0008892 | EC:3.5.4.3 |
| TRINITY_DN21634_c0_g1_i1_len_4967_227792.p1_type_5prime_partial_len_133 | ---NA--- | | |
| TRINITY_DN35206_c0_g1_i1_len_600_201765.p1_type_5prime_partial_len_133 | XP_018009463.1 solute carrier family 25 member 48-like | C:GO:0016020; C:GO:0016021; F:GO:0022857; P:GO:0055085 | |

| | | | |
|---|---|---|---|
| TRINITY_DN153_c0_g1_i1_len_399_246445.p1_type_internal_len_134 | XP_026479179.1general transcription factor 3C polypeptide 1-like | | |
| TRINITY_DN2762_c0_g1_i1_len_399_246449.p1_type_internal_len_134 | XP_018019295.1 FYVE, RhoGEF and PH domain-containing protein 6-like isoform X2 | | |
| TRINITY_DN28349_c0_g1_i1_len_2600_189222.p2_type_5prime_partial_len_133 | ---NA--- | | |
| TRINITY_DN16581_c0_g1_i1_len_401_41439.p1_type_internal_len_134 | CAB53471.1polymerase polyprotein | P:GO:0006508; F:GO:0008233 | |
| TRINITY_DN10423_c0_g1_i1_len_400_246397.p1_type_internal_len_134 | ---NA--- | | |
| TRINITY_DN28031_c0_g1_i1_len_400_246398.p1_type_internal_len_134 | XP_018012290.1 protein turtle homolog A-like | | |
| TRINITY_DN9756_c0_g1_i1_len_400_246401.p1_type_internal_len_134 | ---NA--- | | |
| TRINITY_DN31767_c0_g1_i1_len_400_246404.p1_type_internal_len_134 | XP_018011644.1 irregular chiasm C-roughest protein-like | C:GO:0016020; C:GO:0016021 | |
| TRINITY_DN26928_c0_g1_i1_len_400_246405.p1_type_internal_len_134 | EEB18400.1SEC23-interacting protein, putative | F:GO:0046872 | |
| TRINITY_DN18607_c0_g1_i1_len_584_202109.p1_type_5prime_partial_len_133 | ---NA--- | | |
| TRINITY_DN26139_c0_g1_i2_len_684_239436.p1_type_5prime_partial_len_133 | ---NA--- | | |
| TRINITY_DN26674_c0_g1_i1_len_401_246344.p1_type_internal_len_134 | XP_014260253.1kinesin-like protein unc-104 isoform X7 | F:GO:0003777; F:GO:0005524; P:GO:0007018; F:GO:0008017 | EC:3.6.1.15 |
| TRINITY_DN17586_c0_g2_i2_len_429_206777.p1_type_5prime_partial_len_133 | ---NA--- | | |
| TRINITY_DN30216_c0_g1_i1_len_403_170117.p1_type_internal_len_134 | ---NA--- | | |
| TRINITY_DN27336_c0_g1_i1_len_401_246357.p1_type_internal_len_134 | KZS03498.1Myosin heavy chain 95F | F:GO:0003774; F:GO:0005524; C:GO:0016459; F:GO:0051015 | EC:3.6.1.15 |
| TRINITY_DN7055_c0_g1_i1_len_401_246360.p1_type_internal_len_134 | ---NA--- | | |

| | | | |
|---|---|---|---|
| TRINITY_DN14037_c0_g1_i1_len_401_246362.p1_type_internal_len_134 | XP_022520445.1uncharacterized protein LOC111190946 | | |
| TRINITY_DN30077_c0_g1_i1_len_401_246364.p1_type_internal_len_134 | KFM57431.1Laminin subunit alpha | F:GO:0005102; P:GO:0030155; P:GO:0030334; P:GO:0045995 | |
| TRINITY_DN37805_c0_g1_i1_len_402_246311.p1_type_internal_len_134 | ACC62400.2ribonuclease III | F:GO:0003723; F:GO:0004525; P:GO:0006396; P:GO:0016075; P:GO:0031047; P:GO:0090502 | EC:3.1.30; EC:3.1.26; EC:3.1.26.3 |
| TRINITY_DN5938_c0_g1_i1_len_402_246325.p1_type_internal_len_134 | KZS14475.1putative Peripheral-type benzodiazepine receptor-associated protein | P:GO:0007274 | |
| TRINITY_DN33046_c0_g1_i1_len_402_246332.p1_type_internal_len_134 | XP_022332945.1multidrug resistance-associated protein 7-like | | |
| TRINITY_DN33557_c0_g1_i1_len_402_246333.p1_type_internal_len_134 | XP_018017907.1 adenosine kinase-like | | |
| TRINITY_DN37907_c0_g1_i1_len_401_246372.p1_type_internal_len_134 | XP_022541067.1zinc finger protein OZF-like | | |
| TRINITY_DN7426_c0_g1_i1_len_400_246378.p1_type_internal_len_134 | ---NA--- | | |
| TRINITY_DN1367_c0_g1_i1_len_400_246384.p1_type_internal_len_134 | ---NA--- | | |
| TRINITY_DN31664_c0_g1_i1_len_400_246393.p1_type_internal_len_134 | ---NA--- | | |
| TRINITY_DN1792_c0_g1_i1_len_401_246367.p1_type_internal_len_134 | ---NA--- | | |
| TRINITY_DN7442_c1_g1_i1_len_397_208139.p1_type_internal_len_133 | XP_011674660.1 disheveled-associated activator of morphogenesis 1 isoform X3 | | |
| TRINITY_DN19882_c0_g1_i1_len_1043_160349.p1_type_complete_len_132 | ---NA--- | | |
| TRINITY_DN41592_c0_g1_i1_len_397_208137.p3_type_internal_len_133 | XP_022206249.1suppressor of cytokine signaling 5-like | P:GO:0035556 | |
| TRINITY_DN10682_c0_g1_i1_len_397_11825.p1_type_internal_len_133 | ---NA--- | | |

| | | | |
|---|---|---|---|
| TRINITY_DN31664_c0_g1_i1_len_397_208147.p1_type_internal_len_133 | XP_026761405.1histone-lysine N-methyltransferase trithorax | | |
| TRINITY_DN17761_c0_g1_i1_len_396_208183.p1_type_internal_len_133 | XP_003403377.2glucose dehydrogenase [FAD, quinone] | | |
| TRINITY_DN33512_c0_g1_i1_len_397_208142.p1_type_internal_len_133 | KZS07405.1Low-density lipoprotein receptor-related protein 2 | F:GO:0005509; C:GO:0016020; C:GO:0016021 | |
| TRINITY_DN19868_c0_g1_i1_len_1388_158318.p2_type_complete_len_132 | ---NA--- | | |
| TRINITY_DN11222_c0_g1_i2_len_396_11873.p1_type_internal_len_133 | ---NA--- | | |
| TRINITY_DN26318_c1_g1_i14_len_5925_227622.p1_type_5prime_partial_len_1563 | EEB13862.1conserved hypothetical protein | | |
| TRINITY_DN25766_c0_g1_i1_len_5773_227641.p1_type_5prime_partial_len_1558 | XP_021930318.1lysine-specific demethylase 5A-like isoform X1 | F:GO:0005488 | |
| TRINITY_DN31156_c0_g1_i2_len_4954_187139.p1_type_5prime_partial_len_1556 | XP_026284642.1breast cancer type 2 susceptibility protein homolog | | |
| TRINITY_DN26280_c0_g1_i2_len_6471_227552.p1_type_5prime_partial_len_1550 | EEB10173.1protein sidekick precursor, putative | C:GO:0016020 | |
| TRINITY_DN23057_c0_g1_i3_len_4827_227834.p1_type_5prime_partial_len_1549 | KDR13941.1Cohesin subunit SA-1 | | |
| TRINITY_DN24937_c2_g1_i3_len_8334_227475.p1_type_complete_len_1545 | XP_021929185.1 histone-lysine N-methyltransferase 2D | | |
| TRINITY_DN30256_c0_g1_i4_len_5600_186992.p1_type_complete_len_1545 | XP_023024863.1CAP-Gly domain-containing linker protein 2-like isoform X4 | | |
| TRINITY_DN30349_c0_g1_i3_len_5043_187112.p1_type_5prime_partial_len_1545 | PNI43328.1GIN1 isoform 6 | F:GO:0003676; P:GO:0015074 | |
| TRINITY_DN31203_c0_g1_i2_len_5434_187024.p1_type_complete_len_1545 | AKK75090.1DNA (cytosine-5-)-methyltransferase 1 | F:GO:0003677; F:GO:0003682; F:GO:0003886; C:GO:0005634; F:GO:0008270; P:GO:0090116 | EC:2.1.1.37 |
| TRINITY_DN26411_c0_g1_i2_len_6082_227600.p1_type_5prime_partial_len_1543 | KXJ06669.1Retrovirus-related Pol polyprotein from transposon 17.6 | | |
| TRINITY_DN23596_c0_g1_i1_len_3376_154000.p1_type_complete_len_893 | XP_007060189.1 tigger transposable element-derived protein 1-like | | |

| | | | |
|---|---|---|---|
| 2137\|_\|c2183_f3p0_2679_isoform_c2183_full_length_coverage_3_non_full_length_coverage_0_isoform_length_2679_11720 | XP_018007530.1PREDICTED: uncharacterized protein LOC108665303 | | |
| 2138\|_\|c18144_f3p0_2679_isoform_c18144_full_length_coverage_3_non_full_length_coverage_0_isoform_length_2679_22718 | XP_018013028.1 protein transport protein Sec24A-like | P:GO:0015031; P:GO:0046907 | |
| TRINITY_DN23893_c0_g1_i2_len_2783_122244.p1_type_3prime_partial_len_894 | ---NA--- | | |
| TRINITY_DN29112_c0_g1_i2_len_2855_188767.p1_type_5prime_partial_len_893 | XP_014271591.1peripheral plasma membrane protein CASK isoform X7 | | |
| 2142\|_\|c17866_f3p3_2678_isoform_c17866_full_length_coverage_3_non_full_length_coverage_3_isoform_length_2678_17825 | ---NA--- | | |
| 2143\|_\|c1408_f16p3_2678_isoform_c1408_full_length_coverage_16_non_full_length_coverage_3_isoform_length_2678_20424 | XP_014294367.1AFG3-like protein 2 | F:GO:0004222; F:GO:0005524; P:GO:0006508; F:GO:0008270; C:GO:0016021 | EC:3.4.24 |
| 2144\|_\|c2639_f6p2_2677_isoform_c2639_full_length_coverage_6_non_full_length_coverage_2_isoform_length_2677_21110 | OQR72141.1aspartate--tRNA ligase | F:GO:0003676; F:GO:0004815; F:GO:0005524; C:GO:0005737; P:GO:0006422 | EC:6.1.1.12 |
| TRINITY_DN21944_c0_g1_i2_len_2778_91283.p1_type_5prime_partial_len_892 | XP_018009234.1PREDICTED: uncharacterized protein LOC108666817 | | |
| TRINITY_DN22307_c0_g1_i1_len_2679_229468.p1_type_internal_len_893 | ---NA--- | | |
| TRINITY_DN20317_c0_g1_i2_len_749_5261.p3_type_5prime_partial_len_132 | ---NA--- | | |
| TRINITY_DN41924_c0_g1_i1_len_398_208123.p1_type_internal_len_133 | KRT83535.1Sterile alpha motif containing protein | F:GO:0003950 | EC:2.4.2.30 |
| TRINITY_DN19914_c0_g1_i3_len_813_271641.p1_type_complete_len_132 | ---NA--- | | |
| TRINITY_DN32672_c0_g1_i1_len_397_208134.p2_type_internal_len_133 | ---NA--- | | |
| TRINITY_DN21657_c0_g1_i1_len_615_273929.p1_type_5prime_partial_len_132 | ---NA--- | | |

| | | | |
|---|---|---|---|
| TRINITY_DN20927_c0_g1_i4_len_1263_94198.p2_type_complete_len_132 | ---NA--- | | |
| TRINITY_DN36916_c0_g1_i1_len_397_208155.p2_type_internal_len_133 | XP_018011576.1PREDICTED: uncharacterized protein LOC108668835 | C:GO:0005576; P:GO:0006030; F:GO:0008061 | |
| TRINITY_DN18958_c0_g1_i1_len_1597_267404.p2_type_complete_len_132 | ---NA--- | | |
| TRINITY_DN1945_c0_g1_i1_len_396_208182.p1_type_internal_len_133 | KDR23676.1Multiple epidermal growth factor-like domains 8 | F:GO:0005509; C:GO:0016020; C:GO:0016021 | |
| TRINITY_DN15655_c0_g1_i1_len_544_100381.p1_type_complete_len_132 | OWF42742.1Chromosome transmission fidelity protein 8-like | P:GO:0007064; C:GO:0031390 | |
| TRINITY_DN31099_c0_g1_i2_len_3373_188055.p3_type_complete_len_132 | ---NA--- | | |
| TRINITY_DN12268_c0_g1_i2_len_397_104520.p1_type_internal_len_133 | ---NA--- | | |
| TRINITY_DN26613_c0_g1_i1_len_2897_188701.p3_type_complete_len_132 | ---NA--- | | |
| TRINITY_DN17477_c0_g1_i1_len_399_208072.p1_type_internal_len_133 | ---NA--- | | |
| TRINITY_DN20907_c0_g1_i1_len_2179_584.p3_type_complete_len_132 | ---NA--- | | |
| TRINITY_DN17419_c0_g1_i1_len_720_5533.p1_type_complete_len_132 | XP_018011715.1 prefoldin subunit 4-like | | |
| TRINITY_DN37443_c0_g1_i1_len_541_241989.p2_type_5prime_partial_len_132 | ---NA--- | | |
| TRINITY_DN18439_c0_g1_i1_len_1271_94166.p2_type_5prime_partial_len_132 | ---NA--- | | |
| TRINITY_DN19034_c0_g1_i3_len_400_208013.p1_type_internal_len_133 | ---NA--- | | |
| TRINITY_DN4875_c0_g1_i1_len_578_274505.p1_type_5prime_partial_len_132 | ---NA--- | | |
| TRINITY_DN31131_c4_g1_i1_len_2479_189502.p1_type_complete_len_132 | KRX83908.1hypothetical protein T4E_7988, partial | | |

| | | | |
|---|---|---|---|
| TRINITY_DN1756_c0_g1_i1_len_396_41625.p1_type_internal_len_133 | XP_013776004.1ras association domain-containing protein 10-like | | |
| TRINITY_DN19098_c0_g1_i2_len_451_133596.p1_type_3prime_partial_len_133 | XP_015255935.1 gastrula zinc finger protein XlCGF8.2DB-like | | |
| TRINITY_DN24832_c0_g1_i1_len_3748_228318.p7_type_complete_len_132 | AKB96221.1heat shock protein | | |
| TRINITY_DN26547_c0_g1_i1_len_1737_266977.p2_type_complete_len_132 | ---NA--- | | |
| TRINITY_DN13037_c0_g1_i2_len_535_242125.p1_type_5prime_partial_len_132 | PRD19005.1zinc finger protein | F:GO:0003676 | |
| TRINITY_DN38722_c0_g1_i1_len_510_203936.p2_type_5prime_partial_len_132 | AEB54642.1ribosomal protein L35 | F:GO:0003735; C:GO:0005840; P:GO:0006412 | |
| TRINITY_DN17753_c0_g1_i1_len_568_130925.p2_type_5prime_partial_len_132 | AAL78166.1transglutaminase | F:GO:0003810; P:GO:0018149; F:GO:0046872 | EC:2.3.2.13 |
| TRINITY_DN32557_c0_g1_i1_len_398_208113.p1_type_3prime_partial_len_133 | ---NA--- | | |
| TRINITY_DN23635_c0_g1_i1_len_2017_190841.p1_type_5prime_partial_len_132 | ---NA--- | | |
| TRINITY_DN18099_c0_g1_i2_len_611_240609.p1_type_complete_len_132 | ---NA--- | | |
| TRINITY_DN1007_c0_g1_i1_len_396_208185.p1_type_internal_len_133 | KZS14202.1Tubulointerstitial nephritis antigen-like protein | F:GO:0005044; C:GO:0005615; P:GO:0006508; P:GO:0006898; P:GO:0006955; P:GO:0007155; F:GO:0008234; F:GO:0030247 | |
| TRINITY_DN15074_c0_g1_i1_len_520_203634.p1_type_5prime_partial_len_132 | ---NA--- | | |
| TRINITY_DN29179_c0_g1_i1_len_2105_190540.p1_type_5prime_partial_len_132 | ---NA--- | | |
| TRINITY_DN31609_c0_g1_i1_len_398_208095.p1_type_internal_len_133 | ---NA--- | | |
| TRINITY_DN38224_c0_g1_i1_len_398_208101.p1_type_internal_len_133 | ---NA--- | | |

| | | | |
|---|---|---|---|
| TRINITY_DN37140_c0_g1_i1_len_398_208109.p1_type_internal_len_133 | ---NA--- | | |
| TRINITY_DN31804_c0_g1_i1_len_438_206414.p1_type_5prime_partial_len_132 | ---NA--- | | |
| TRINITY_DN14430_c0_g1_i1_len_447_244655.p1_type_5prime_partial_len_132 | XP_023710781.1transmembrane protein 145 | P:GO:0007186; C:GO:0016021; P:GO:0019236 | |
| TRINITY_DN2684_c0_g1_i1_len_400_41460.p1_type_internal_len_133 | KDR21679.1PCTP-like protein | F:GO:0008289 | |
| TRINITY_DN18335_c0_g1_i1_len_1063_235622.p2_type_complete_len_132 | ---NA--- | | |
| TRINITY_DN26832_c0_g1_i1_len_400_170237.p1_type_internal_len_133 | ---NA--- | | |
| TRINITY_DN15641_c0_g1_i1_len_400_170208.p1_type_internal_len_133 | ---NA--- | | |
| TRINITY_DN23753_c1_g1_i1_len_907_236884.p1_type_3prime_partial_len_133 | ---NA--- | | |
| TRINITY_DN19531_c0_g1_i3_len_717_300946.p1_type_complete_len_132 | ---NA--- | | |
| TRINITY_DN15697_c0_g1_i2_len_398_170315.p1_type_internal_len_133 | ---NA--- | | |
| TRINITY_DN13648_c0_g1_i3_len_398_170301.p3_type_internal_len_133 | ---NA--- | | |
| TRINITY_DN11210_c0_g1_i1_len_443_277551.p1_type_5prime_partial_len_132 | ---NA--- | | |
| TRINITY_DN36990_c0_g1_i1_len_446_244705.p1_type_complete_len_132 | ---NA--- | | |
| TRINITY_DN13742_c0_g1_i1_len_398_170280.p1_type_internal_len_133 | ---NA--- | | |
| TRINITY_DN15051_c0_g1_i1_len_507_242860.p1_type_5prime_partial_len_132 | ---NA--- | | |
| TRINITY_DN9598_c0_g1_i1_len_397_279073.p1_type_internal_len_133 | ---NA--- | | |

| | | | |
|---|---|---|---|
| TRINITY_DN22534_c2_g1_i4_len_853_237401.p1_type_5prime_partial_len_132 | ---NA--- | | |
| TRINITY_DN5209_c0_g1_i1_len_397_307141.p1_type_internal_len_133 | ---NA--- | | |
| TRINITY_DN16530_c0_g1_i1_len_398_307116.p1_type_internal_len_133 | ACD74843.1trehalose-6-phosphate synthase | F:GO:0003825; P:GO:0005992 | EC:2.4.1.15 |
| TRINITY_DN23026_c0_g1_i1_len_4551_153521.p4_type_complete_len_132 | ---NA--- | | |
| TRINITY_DN11191_c0_g1_i1_len_416_103772.p1_type_5prime_partial_len_132 | ---NA--- | | |
| TRINITY_DN14729_c0_g1_i1_len_469_205240.p1_type_5prime_partial_len_132 | XP_015911965.1zinc finger protein 670-like | | |
| TRINITY_DN4959_c1_g1_i1_len_510_242773.p1_type_5prime_partial_len_132 | ---NA--- | | |
| TRINITY_DN19473_c0_g1_i2_len_470_304873.p1_type_5prime_partial_len_132 | ---NA--- | | |
| TRINITY_DN11004_c0_g1_i2_len_441_305650.p1_type_5prime_partial_len_132 | ---NA--- | | |
| TRINITY_DN6907_c0_g1_i1_len_424_169278.p1_type_5prime_partial_len_132 | AWP38853.1RING box protein 1 | F:GO:0008270 | |
| TRINITY_DN32381_c0_g1_i1_len_399_246420.p1_type_internal_len_133 | KZS09955.1Low-density lipoprotein receptor-related protein 6 | F:GO:0017147; F:GO:0042813; P:GO:0060070 | |
| TRINITY_DN30221_c0_g1_i1_len_399_246418.p1_type_internal_len_133 | ---NA--- | | |
| TRINITY_DN9230_c0_g1_i1_len_399_246416.p1_type_internal_len_133 | XP_023240656.1WD repeat domain phosphoinositide-interacting protein 3-like | | |
| TRINITY_DN40995_c0_g1_i1_len_432_206641.p1_type_5prime_partial_len_132 | ---NA--- | | |
| TRINITY_DN939_c0_g1_i1_len_482_167507.p1_type_3prime_partial_len_133 | APY20638.1long wavelength sensitive opsin | P:GO:0007165 | |
| TRINITY_DN26187_c0_g1_i1_len_435_103122.p1_type_5prime_partial_len_132 | ---NA--- | | |

| | | | |
|---|---|---|---|
| TRINITY_DN7144_c0_g1_i1_len_709_199854.p1_type_3prime_partial_len_133 | ---NA--- | | |
| TRINITY_DN8332_c0_g1_i1_len_589_202013.p1_type_3prime_partial_len_133 | ---NA--- | | |
| TRINITY_DN12355_c0_g1_i1_len_406_278751.p1_type_5prime_partial_len_132 | ---NA--- | | |
| TRINITY_DN24264_c0_g1_i1_len_408_169902.p1_type_5prime_partial_len_132 | ---NA--- | | |
| TRINITY_DN11136_c0_g1_i1_len_581_67389.p1_type_complete_len_132 | APG16029.1broad-complex | F:GO:0003676 | |
| TRINITY_DN29517_c0_g1_i1_len_397_279079.p1_type_internal_len_133 | ---NA--- | | |
| TRINITY_DN5330_c0_g1_i1_len_580_202205.p1_type_5prime_partial_len_132 | ---NA--- | | |
| TRINITY_DN30939_c0_g1_i1_len_501_243017.p1_type_3prime_partial_len_133 | ABK56704.1delta protein | F:GO:0005509; P:GO:0007219; P:GO:0007275; C:GO:0016021 | |
| TRINITY_DN29230_c0_g1_i1_len_397_246502.p1_type_internal_len_133 | ---NA--- | | |
| TRINITY_DN30815_c0_g1_i1_len_398_246492.p1_type_internal_len_133 | ---NA--- | | |
| TRINITY_DN1767_c0_g1_i1_len_398_246486.p1_type_internal_len_133 | ---NA--- | | |
| TRINITY_DN11774_c0_g1_i1_len_432_245185.p1_type_5prime_partial_len_132 | ---NA--- | | |
| TRINITY_DN13353_c0_g1_i1_len_398_246482.p1_type_internal_len_133 | ---NA--- | | |
| TRINITY_DN30424_c0_g1_i1_len_398_246478.p1_type_internal_len_133 | ---NA--- | | |
| TRINITY_DN32286_c0_g1_i1_len_398_246476.p1_type_internal_len_133 | ---NA--- | | |
| TRINITY_DN10346_c0_g1_i1_len_431_245208.p1_type_5prime_partial_len_132 | XP_018913639.1 histone H2A | | |

| | | | |
|---|---|---|---|
| TRINITY_DN13859_c0_g1_i1_len_398_246475.p1_type_internal_len_133 | ---NA--- | | |
| TRINITY_DN11909_c0_g1_i1_len_398_246471.p1_type_internal_len_133 | ---NA--- | | |
| TRINITY_DN33551_c0_g1_i1_len_398_246469.p1_type_internal_len_133 | XP_018014555.1PREDICTED: uncharacterized protein LOC108671513 | | |
| TRINITY_DN4002_c0_g1_i2_len_398_246468.p1_type_internal_len_133 | ---NA--- | | |
| TRINITY_DN34603_c0_g1_i1_len_398_246454.p1_type_internal_len_133 | ---NA--- | | |
| TRINITY_DN4884_c0_g1_i1_len_399_246442.p1_type_internal_len_133 | ---NA--- | | |
| TRINITY_DN1691_c0_g1_i1_len_399_246425.p1_type_internal_len_133 | ---NA--- | | |
| TRINITY_DN17895_c0_g1_i1_len_535_203278.p1_type_5prime_partial_len_132 | ---NA--- | | |
| TRINITY_DN7840_c0_g1_i1_len_396_246564.p1_type_internal_len_133 | ---NA--- | | |
| TRINITY_DN30705_c0_g1_i1_len_396_246562.p1_type_internal_len_133 | ---NA--- | | |
| TRINITY_DN29556_c0_g1_i1_len_396_246543.p1_type_internal_len_133 | XP_013784962.1MKRN2 opposite strand protein-like | | |
| TRINITY_DN36426_c0_g1_i1_len_396_246536.p1_type_internal_len_133 | XP_021936280.1rab GTPase-activating protein 1-like | F:GO:0005096; P:GO:0043547 | |
| TRINITY_DN8598_c0_g1_i1_len_397_246529.p1_type_internal_len_133 | ---NA--- | | |
| TRINITY_DN7825_c0_g1_i1_len_397_246516.p2_type_internal_len_133 | XP_018023308.1 sodium channel protein 60E-like | | |
| TRINITY_DN36445_c0_g1_i1_len_397_246515.p1_type_internal_len_133 | XP_018019672.1 toll-like receptor Tollo | P:GO:0007165; C:GO:0016021 | |
| TRINITY_DN3094_c0_g1_i1_len_397_246513.p1_type_internal_len_133 | XP_022246027.1leucine-rich repeat serine/threonine-protein kinase 1-like | F:GO:0016301; P:GO:0016310 | |

<table>
<tr><td>TRINITY_DN20486_c1_g1_i1_len_3746_264587.p1_type_5prime_partial_len_892</td><td>XP_018021230.1 histone demethylase UTY-like</td><td></td><td></td></tr>
<tr><td>2149|_|c12106_f5p4_2676_isoform_c12106_full_length_coverage_5_non_full_length_coverage_4_isoform_length_2676_17488</td><td>AGV28719.1casein kinase 1 epsilon</td><td>F:GO:0004674; F:GO:0005524; P:GO:0006468</td><td>EC:2.7.11</td></tr>
<tr><td>2150|_|c1705_f2p3_2676_isoform_c1705_full_length_coverage_2_non_full_length_coverage_3_isoform_length_2676_27915</td><td>---NA---</td><td></td><td></td></tr>
<tr><td>2151|_|c18285_f2p0_2676_isoform_c18285_full_length_coverage_2_non_full_length_coverage_0_isoform_length_2676_13695</td><td>---NA---</td><td></td><td></td></tr>
<tr><td>2152|_|c47494_f21p8_2676_isoform_c47494_full_length_coverage_21_non_full_length_coverage_8_isoform_length_2676_24168</td><td>XP_015173705.1 protein lines</td><td></td><td></td></tr>
<tr><td>2153|_|c2035_f5p3_2676_isoform_c2035_full_length_coverage_5_non_full_length_coverage_3_isoform_length_2676_11620</td><td>PSN52815.1Protein sel-1 1</td><td>C:GO:0016021</td><td></td></tr>
<tr><td>2154|_|c30953_f2p0_2675_isoform_c30953_full_length_coverage_2_non_full_length_coverage_0_isoform_length_2675_14109</td><td>XP_013385759.1GPI transamidase component PIG-S</td><td>C:GO:0016020; C:GO:0016021; P:GO:0016255; C:GO:0042765</td><td></td></tr>
<tr><td>2155|_|c2880_f3p0_2675_isoform_c2880_full_length_coverage_3_non_full_length_coverage_0_isoform_length_2675_12242</td><td>XP_015273028.1 zinc finger protein 14-like</td><td></td><td></td></tr>
<tr><td>2157|_|c5786_f3p0_2675_isoform_c5786_full_length_coverage_3_non_full_length_coverage_0_isoform_length_2675_21555</td><td>ARW71688.1NF-kappa-B-repressing factor</td><td>F:GO:0003723</td><td></td></tr>
<tr><td>2159|_|c18061_f2p1_2674_isoform_c18061_full_length_coverage_2_non_full_length_coverage_1_isoform_length_2674_13545</td><td>---NA---</td><td></td><td></td></tr>
<tr><td>TRINITY_DN30036_c0_g1_i1_len_398_104443.p1_type_internal_len_133</td><td>AJP09268.1elongation factor 1-alpha</td><td>F:GO:0003746; F:GO:0005525; P:GO:0006414</td><td></td></tr>
<tr><td>TRINITY_DN11503_c0_g1_i1_len_463_244147.p2_type_5prime_partial_len_132</td><td>---NA---</td><td></td><td></td></tr>
</table>

| | | | |
|---|---|---|---|
| TRINITY_DN24937_c0_g1_i1_len_1862_231522.p2_type_5prime_partial_len_132 | XP_014817019.1 transcription initiation factor TFIID subunit 10 | | |
| TRINITY_DN10401_c0_g1_i1_len_398_135472.p1_type_internal_len_133 | ---NA--- | | |
| TRINITY_DN6025_c0_g1_i2_len_398_135475.p2_type_internal_len_133 | ---NA--- | | |
| TRINITY_DN18833_c0_g1_i2_len_398_135476.p1_type_internal_len_133 | ---NA--- | | |
| TRINITY_DN10809_c0_g1_i1_len_398_135495.p1_type_internal_len_133 | ---NA--- | | |
| TRINITY_DN6935_c0_g1_i1_len_401_135385.p1_type_5prime_partial_len_132 | AAL68340.2RH06643p, partial | P:GO:0002181; F:GO:0003735; C:GO:0005791; F:GO:0008270; C:GO:0022627; C:GO:0042788 | |
| TRINITY_DN11371_c0_g1_i1_len_1392_268165.p2_type_5prime_partial_len_132 | ---NA--- | | |
| TRINITY_DN26335_c0_g1_i1_len_399_135439.p1_type_internal_len_133 | ---NA--- | | |
| TRINITY_DN4571_c0_g1_i1_len_399_135462.p1_type_internal_len_133 | ANA78437.1dopamine beta-hydroxylase | F:GO:0005507; C:GO:0016021; F:GO:0016715; P:GO:0055114 | EC:1.14.17 |
| TRINITY_DN15001_c0_g1_i1_len_523_275521.p1_type_5prime_partial_len_132 | ---NA--- | | |
| TRINITY_DN17445_c0_g1_i1_len_405_11539.p1_type_5prime_partial_len_132 | ---NA--- | | |
| TRINITY_DN30972_c0_g1_i2_len_899_197514.p1_type_complete_len_132 | ---NA--- | | |
| TRINITY_DN10856_c0_g1_i1_len_398_41538.p2_type_internal_len_133 | ---NA--- | | |
| TRINITY_DN31001_c0_g1_i1_len_397_135519.p1_type_internal_len_133 | BBC20717.1integrin alpha ps | P:GO:0007155; P:GO:0007229; C:GO:0008305 | |
| TRINITY_DN27407_c0_g1_i1_len_397_135544.p1_type_internal_len_133 | XP_014057789.1 multidrug resistance-associated protein 1-like | F:GO:0005524; C:GO:0005887; F:GO:0008514; P:GO:0015711; F:GO:0042626; P:GO:0055085 | EC:3.6.1.3; EC:3.6.1.15 |

| | | | |
|---|---|---|---|
| TRINITY_DN11406_c0_g1_i1_len_396_135564.p1_type_internal_len_133 | XP_018018467.1 larval cuticle protein LCP-22-like | | |
| TRINITY_DN27798_c0_g1_i1_len_453_10023.p1_type_5prime_partial_len_132 | ---NA--- | | |
| TRINITY_DN21696_c0_g1_i2_len_656_239848.p1_type_5prime_partial_len_132 | ---NA--- | | |
| TRINITY_DN21143_c0_g2_i1_len_1373_193822.p1_type_5prime_partial_len_132 | ---NA--- | | |
| TRINITY_DN7266_c0_g1_i1_len_442_10354.p1_type_3prime_partial_len_133 | ---NA--- | | |
| TRINITY_DN10991_c0_g1_i1_len_403_72199.p1_type_5prime_partial_len_132 | ---NA--- | | |
| TRINITY_DN14044_c0_g1_i1_len_804_198555.p1_type_5prime_partial_len_132 | XP_018027749.1 signal peptidase complex subunit 1-like | C:GO:0005787; P:GO:0006465; F:GO:0008233; C:GO:0016021 | |
| TRINITY_DN27621_c0_g1_i1_len_416_71706.p1_type_5prime_partial_len_132 | ---NA--- | | |
| TRINITY_DN6911_c0_g1_i1_len_471_9552.p2_type_5prime_partial_len_132 | XP_018009982.1PREDICTED: uncharacterized protein LOC108667470 isoform X1 | | |
| TRINITY_DN16091_c0_g1_i2_len_397_72476.p1_type_internal_len_133 | ---NA--- | | |
| TRINITY_DN22827_c0_g1_i1_len_1147_195324.p2_type_5prime_partial_len_132 | ETN66666.1mitochondrial glutamate carrier protein | C:GO:0016021; F:GO:0022857; P:GO:0055085 | |
| TRINITY_DN30993_c0_g1_i1_len_398_72435.p1_type_internal_len_133 | XP_018016653.1 SCY1-like protein 2 | | |
| TRINITY_DN13122_c0_g1_i2_len_398_72429.p1_type_internal_len_133 | ---NA--- | | |
| TRINITY_DN4974_c1_g1_i1_len_399_72396.p1_type_internal_len_133 | XP_025602031.1microtubule-associated protein futsch-like isoform X8 | | |
| TRINITY_DN33776_c0_g1_i1_len_399_72394.p1_type_internal_len_133 | XP_012341921.1  AP2-associated protein kinase 1-like | | |

| | | | |
|---|---|---|---|
| TRINITY_DN23413_c0_g1_i1_len_411_11345.p1_type_3prime_partial_len_133 | XP_021942947.1ankyrin repeat and sterile alpha motif domain-containing protein 1B-like | | |
| TRINITY_DN20314_c0_g1_i4_len_2258_295379.p3_type_complete_len_132 | ---NA--- | | |
| TRINITY_DN13721_c0_g1_i1_len_396_72541.p1_type_internal_len_133 | AUI80370.1cryptochrome 1 | | |
| TRINITY_DN31011_c0_g1_i1_len_396_72530.p1_type_internal_len_133 | ---NA--- | | |
| TRINITY_DN16243_c0_g1_i1_len_397_72486.p1_type_internal_len_133 | ANQ44704.1heat shock protein 70 kDa | F:GO:0005524 | |
| TRINITY_DN33144_c0_g1_i1_len_399_104412.p1_type_internal_len_133 | XP_018018541.1 probable serine/threonine-protein kinase DDB_G0282963 isoform X1 | | |
| TRINITY_DN9563_c0_g1_i2_len_399_41514.p1_type_5prime_partial_len_132 | ---NA--- | | |
| TRINITY_DN14702_c0_g1_i1_len_416_207307.p1_type_5prime_partial_len_132 | ---NA--- | | |
| TRINITY_DN7623_c0_g1_i1_len_394_41690.p1_type_internal_len_132 | ---NA--- | | |
| TRINITY_DN10136_c0_g1_i1_len_565_37493.p1_type_complete_len_131 | XP_015180727.1 biogenesis of lysosome-related organelles complex 1 subunit 1 | | |
| TRINITY_DN17817_c0_g1_i1_len_1187_94541.p2_type_complete_len_131 | ---NA--- | | |
| TRINITY_DN6330_c0_g1_i1_len_452_39833.p1_type_5prime_partial_len_131 | ---NA--- | | |
| TRINITY_DN18886_c0_g1_i1_len_720_97843.p1_type_complete_len_131 | XP_023721356.1uncharacterized protein LOC111872050 isoform X2 | C:GO:0005761; P:GO:0032543 | |
| TRINITY_DN25768_c0_g1_i1_len_407_278710.p1_type_5prime_partial_len_131 | ---NA--- | | |
| TRINITY_DN10632_c0_g1_i1_len_553_37675.p1_type_5prime_partial_len_131 | ---NA--- | | |

| | | | |
|---|---|---|---|
| TRINITY_DN14012_c0_g1_i1_len_396_279112.p1_type_internal_len_132 | ---NA--- | | |
| TRINITY_DN15371_c0_g1_i1_len_648_36310.p1_type_complete_len_131 | ---NA--- | | |
| TRINITY_DN19902_c0_g2_i1_len_1304_94007.p2_type_complete_len_131 | ---NA--- | | |
| TRINITY_DN6902_c0_g1_i1_len_486_101623.p1_type_5prime_partial_len_131 | ---NA--- | | |
| TRINITY_DN3558_c0_g1_i1_len_576_99812.p3_type_5prime_partial_len_131 | ---NA--- | | |
| TRINITY_DN4119_c0_g1_i1_len_452_277259.p1_type_complete_len_131 | ACD69542.1accessory gland protein | F:GO:0004252; P:GO:0006508 | |
| TRINITY_DN23619_c0_g1_i1_len_648_273442.p1_type_complete_len_131 | XP_018013022.1 DNA-directed RNA polymerases I, II, and III subunit RPABC2-like | F:GO:0003677; F:GO:0003899; C:GO:0005665; P:GO:0006351 | EC:2.7.7.6 |
| TRINITY_DN2356_c0_g1_i1_len_425_40686.p1_type_5prime_partial_len_131 | ---NA--- | | |
| TRINITY_DN1248_c0_g1_i1_len_1681_92818.p2_type_complete_len_131 | ---NA--- | | |
| TRINITY_DN13114_c0_g1_i1_len_396_104555.p1_type_internal_len_132 | ---NA--- | | |
| TRINITY_DN15628_c0_g1_i1_len_395_279156.p4_type_internal_len_132 | ---NA--- | | |
| TRINITY_DN22746_c0_g1_i1_len_394_279178.p1_type_internal_len_132 | ---NA--- | | |
| TRINITY_DN25843_c0_g1_i1_len_395_41669.p2_type_internal_len_132 | ---NA--- | | |
| TRINITY_DN24108_c0_g1_i2_len_3156_122041.p3_type_complete_len_131 | ---NA--- | | |
| TRINITY_DN29401_c0_g1_i1_len_393_104671.p1_type_internal_len_132 | ---NA--- | | |
| TRINITY_DN118_c0_g1_i1_len_393_104668.p1_type_internal_len_132 | XP_018022047.1 PRKCA-binding protein-like isoform X3 | | |

| | | | |
|---|---|---|---|
| TRINITY_DN9107_c0_g1_i1_len_395_41659.p1_type_internal_len_132 | ---NA--- | | |
| TRINITY_DN13311_c0_g1_i1_len_754_272212.p1_type_complete_len_131 | ---NA--- | | |
| TRINITY_DN2426_c0_g1_i1_len_395_279138.p1_type_internal_len_132 | ---NA--- | | |
| TRINITY_DN25592_c0_g1_i1_len_395_279154.p1_type_5prime_partial_len_131 | XP_018010798.1 SLIT and NTRK-like protein 4 | | |
| TRINITY_DN20780_c0_g1_i1_len_395_41645.p1_type_internal_len_132 | ---NA--- | | |
| TRINITY_DN8589_c0_g1_i1_len_395_41639.p1_type_internal_len_132 | ---NA--- | | |
| TRINITY_DN14897_c0_g1_i1_len_1597_93054.p2_type_complete_len_131 | ---NA--- | | |
| TRINITY_DN9924_c0_g1_i1_len_394_104629.p2_type_internal_len_132 | ---NA--- | | |
| TRINITY_DN15213_c0_g1_i1_len_419_278318.p1_type_5prime_partial_len_131 | ---NA--- | | |
| TRINITY_DN2308_c0_g1_i1_len_447_39990.p1_type_5prime_partial_len_131 | XP_026468286.128S ribosomal protein S33, mitochondrial | C:GO:0005840 | |
| TRINITY_DN20127_c0_g1_i1_len_734_199525.p1_type_5prime_partial_len_131 | ---NA--- | | |
| TRINITY_DN9082_c0_g1_i1_len_931_236696.p1_type_complete_len_131 | ---NA--- | | |
| TRINITY_DN7322_c0_g1_i1_len_438_206409.p1_type_5prime_partial_len_131 | ---NA--- | | |
| TRINITY_DN7596_c0_g1_i1_len_540_166171.p1_type_5prime_partial_len_131 | ---NA--- | | |
| TRINITY_DN7535_c0_g1_i1_len_395_135585.p1_type_internal_len_132 | ---NA--- | | |
| TRINITY_DN30464_c0_g1_i1_len_395_135615.p1_type_internal_len_132 | ---NA--- | | |

| | | | |
|---|---|---|---|
| TRINITY_DN33209_c0_g1_i1_len_396_135569.p1_type_internal_len_132 | AYD41582.1macrophage mannose receptor 1-like protein | C:GO:0016020; C:GO:0016021 | |
| TRINITY_DN34515_c0_g1_i1_len_395_135581.p1_type_internal_len_132 | PNF32219.1hypothetical protein B7P43_G18285, partial | | |
| TRINITY_DN22185_c0_g1_i1_len_1019_235963.p2_type_complete_len_131 | ---NA--- | | |
| TRINITY_DN20739_c0_g1_i3_len_1019_235965.p1_type_5prime_partial_len_131 | ---NA--- | | |
| TRINITY_DN17510_c0_g1_i1_len_412_306588.p1_type_5prime_partial_len_131 | ---NA--- | | |
| TRINITY_DN19442_c0_g1_i2_len_483_204779.p1_type_3prime_partial_len_132 | ---NA--- | | |
| TRINITY_DN20305_c0_g1_i1_len_1816_296041.p2_type_complete_len_131 | ---NA--- | | |
| TRINITY_DN4375_c0_g1_i1_len_468_133141.p1_type_3prime_partial_len_132 | ---NA--- | | |
| TRINITY_DN21053_c0_g3_i1_len_460_168163.p1_type_complete_len_131 | ---NA--- | | |
| TRINITY_DN1154_c0_g1_i1_len_460_168143.p1_type_5prime_partial_len_131 | XP_022661802.1zinc finger protein 384-like isoform X2 | | |
| TRINITY_DN19287_c0_g1_i1_len_500_132377.p1_type_5prime_partial_len_131 | ---NA--- | | |
| TRINITY_DN40766_c0_g1_i1_len_1192_195018.p2_type_complete_len_131 | XP_026274442.1probable protein phosphatase 2C T23F11.1 | F:GO:0004722; P:GO:0006470; F:GO:0046872 | EC:3.1.3.16 |
| TRINITY_DN30457_c0_g1_i2_len_3175_188286.p4_type_complete_len_131 | ---NA--- | | |
| TRINITY_DN454_c0_g1_i1_len_600_240802.p1_type_5prime_partial_len_131 | ---NA--- | | |
| TRINITY_DN7547_c0_g1_i1_len_397_208169.p1_type_internal_len_132 | XP_018024207.1 protein O-mannosyl-transferase 2-like | | |
| TRINITY_DN3300_c0_g1_i1_len_397_208133.p1_type_internal_len_132 | AAN11303.1vitellogenin VTG | F:GO:0005319; P:GO:0006869 | |

| | | | |
|---|---|---|---|
| TRINITY_DN10478_c0_g1_i1_len_1020_160507.p1_type_complete_len_131 | AHG94982.1vesicle-associated membrane protein | C:GO:0016021; P:GO:0016192 | |
| TRINITY_DN27123_c0_g1_i2_len_3328_188100.p2_type_3prime_partial_len_132 | XP_018021257.1 ubiquitin-conjugating enzyme E2 Q2-like | | |
| TRINITY_DN23484_c0_g2_i2_len_628_240322.p1_type_3prime_partial_len_132 | ---NA--- | | |
| TRINITY_DN21726_c0_g1_i2_len_903_197452.p1_type_complete_len_131 | ---NA--- | | |
| TRINITY_DN36881_c0_g1_i1_len_396_208197.p1_type_internal_len_132 | ---NA--- | | |
| TRINITY_DN18862_c0_g1_i1_len_4457_57760.p1_type_complete_len_891 | AVN99022.1alpha-actinin, sarcomeric-like isoform X1 | | |
| 2161\|_\|c8794_f2p7_2673_isoform_c8794_full_length_coverage_2_non_full_length_coverage_7_isoform_length_2673_12749 | XP_018023570.1 poly(ADP-ribose) glycohydrolase ARH3-like | F:GO:0016787 | |
| 2162\|_\|c2048_f8p42_2673_isoform_c2048_full_length_coverage_8_non_full_length_coverage_42_isoform_length_2673_16149 | ---NA--- | | |
| TRINITY_DN24784_c0_g1_i1_len_2997_228991.p1_type_5prime_partial_len_891 | ---NA--- | | |
| 2164\|_\|c47262_f2p0_2673_isoform_c47262_full_length_coverage_2_non_full_length_coverage_0_isoform_length_2673_19512 | XP_013789135.1protein unc-119 homolog B-like isoform X1 | | |
| TRINITY_DN25260_c0_g1_i3_len_3199_188263.p1_type_complete_len_891 | XP_023326913.1interaptin-like isoform X1 | | |
| 2167\|_\|c3819_f2p0_2672_isoform_c3819_full_length_coverage_2_non_full_length_coverage_0_isoform_length_2672_16797 | XP_018011295.1 nodal modulator 3-like | F:GO:0030246 | |
| 2168\|_\|c2724_f2p3_2672_isoform_c2724_full_length_coverage_2_non_full_length_coverage_3_isoform_length_2672_12106 | XP_018021609.1 kinetochore protein NDC80 homolog | | |

| | | | |
|---|---|---|---|
| 2169\|_\|c7184_f4p7_2671_isoform_c7184_full_length_coverage_4_non_full_length_coverage_7_isoform_length_2671_12607 | XP_014277147.1serine-arginine protein 55 isoform X2 | F:GO:0003723 | |
| 2170\|_\|c36228_f4p1_2671_isoform_c36228_full_length_coverage_4_non_full_length_coverage_1_isoform_length_2671_23582 | XP_013400066.1claspin | | |
| TRINITY_DN37769_c0_g1_i1_len_396_208203.p1_type_internal_len_132 | ---NA--- | | |
| TRINITY_DN13683_c1_g1_i1_len_396_208207.p1_type_internal_len_132 | XP_018308983.1 vacuolar protein sorting-associated protein 13A isoform X5 | | |
| TRINITY_DN28760_c0_g1_i2_len_2626_189182.p1_type_5prime_partial_len_131 | ---NA--- | | |
| TRINITY_DN31115_c0_g1_i1_len_540_242009.p1_type_3prime_partial_len_132 | ---NA--- | | |
| TRINITY_DN27311_c0_g1_i1_len_2812_188839.p3_type_3prime_partial_len_132 | ---NA--- | | |
| TRINITY_DN6805_c0_g1_i1_len_396_208195.p1_type_internal_len_132 | XP_012543343.1 zinc finger SWIM domain-containing protein 8 | F:GO:0008270 | |
| TRINITY_DN20173_c0_g1_i1_len_1257_158999.p2_type_complete_len_131 | ---NA--- | | |
| TRINITY_DN27048_c0_g1_i1_len_1230_194747.p2_type_5prime_partial_len_131 | ---NA--- | | |
| TRINITY_DN15232_c0_g1_i1_len_1230_194752.p2_type_complete_len_131 | ---NA--- | | |
| TRINITY_DN9677_c0_g1_i1_len_809_237858.p1_type_5prime_partial_len_131 | ---NA--- | | |
| TRINITY_DN19260_c0_g1_i1_len_393_135660.p1_type_internal_len_132 | ---NA--- | | |
| TRINITY_DN12756_c0_g1_i1_len_907_161432.p1_type_complete_len_131 | ---NA--- | | |
| TRINITY_DN16538_c0_g1_i3_len_444_206172.p1_type_5prime_partial_len_131 | ---NA--- | | |

| | | | |
|---|---|---|---|
| TRINITY_DN11966_c0_g1_i1_len_769_162731.p1_type_complete_len_131 | ---NA--- | | |
| TRINITY_DN9325_c0_g1_i1_len_760_238415.p4_type_3prime_partial_len_132 | SIW61372.1Lytic polysaccharide monooxygenase | F:GO:0004497; P:GO:0055114 | |
| TRINITY_DN22566_c0_g1_i1_len_395_307218.p1_type_internal_len_132 | ---NA--- | | |
| TRINITY_DN40779_c0_g1_i1_len_507_204022.p1_type_5prime_partial_len_131 | ---NA--- | | |
| TRINITY_DN23426_c0_g1_i6_len_2889_58252.p2_type_5prime_partial_len_131 | ---NA--- | | |
| TRINITY_DN23345_c0_g1_i1_len_394_307237.p1_type_internal_len_132 | PIK54499.1Retrovirus-related Pol polyprotein from transposon | | |
| TRINITY_DN21272_c0_g1_i1_len_1851_231562.p3_type_complete_len_131 | ---NA--- | | |
| TRINITY_DN24950_c0_g1_i1_len_397_307144.p1_type_internal_len_132 | CRK93495.1CLUMA_CG007031, isoform A | C:GO:0005576 | |
| TRINITY_DN12769_c0_g1_i1_len_397_307135.p1_type_internal_len_132 | ---NA--- | | |
| TRINITY_DN1538_c0_g1_i2_len_502_204173.p1_type_3prime_partial_len_132 | ---NA--- | | |
| TRINITY_DN10349_c0_g1_i1_len_1299_194264.p1_type_complete_len_131 | ---NA--- | | |
| TRINITY_DN11606_c0_g1_i1_len_1227_234525.p2_type_complete_len_131 | XP_018025267.1 facilitated trehalose transporter Tret1-2 homolog isoform X2 | | |
| TRINITY_DN15811_c0_g1_i1_len_1004_126519.p3_type_3prime_partial_len_132 | ---NA--- | | |
| TRINITY_DN23019_c0_g1_i1_len_394_307272.p1_type_internal_len_132 | XP_018009674.1PREDICTED: uncharacterized protein LOC108667192 | | |
| TRINITY_DN10453_c0_g1_i1_len_425_206898.p1_type_5prime_partial_len_131 | ---NA--- | | |
| TRINITY_DN17336_c0_g1_i1_len_1519_232930.p3_type_3prime_partial_len_132 | ---NA--- | | |

| | | | |
|---|---|---|---|
| TRINITY_DN25429_c0_g1_i1_len_396_11896.p1_type_internal_len_132 | ---NA--- | | |
| TRINITY_DN29574_c0_g1_i1_len_396_170390.p1_type_internal_len_132 | ---NA--- | | |
| TRINITY_DN24132_c0_g1_i1_len_395_170405.p1_type_internal_len_132 | XP_023712659.1WD repeat-containing protein 35 isoform X1 | | |
| TRINITY_DN12080_c0_g1_i1_len_394_11945.p1_type_internal_len_132 | ---NA--- | | |
| TRINITY_DN27122_c0_g1_i1_len_394_11946.p1_type_internal_len_132 | EEC08691.1protein phosphatase PP2A, putative | C:GO:0000159; F:GO:0005509; F:GO:0019888; P:GO:0043666 | |
| TRINITY_DN29562_c0_g1_i1_len_394_11947.p1_type_internal_len_132 | ---NA--- | | |
| TRINITY_DN761_c0_g1_i1_len_533_203350.p1_type_5prime_partial_len_131 | ---NA--- | | |
| TRINITY_DN18040_c0_g1_i1_len_2004_231110.p1_type_complete_len_131 | ---NA--- | | |
| TRINITY_DN25235_c1_g1_i1_len_1912_231375.p3_type_complete_len_131 | ---NA--- | | |
| TRINITY_DN16329_c0_g1_i3_len_998_298821.p2_type_complete_len_131 | XP_022237468.1serine/threonine-protein phosphatase 6 regulatory ankyrin repeat subunit A-like | | |
| TRINITY_DN18868_c0_g1_i1_len_394_170454.p3_type_internal_len_132 | ---NA--- | | |
| TRINITY_DN6211_c0_g1_i1_len_394_170447.p1_type_internal_len_132 | ---NA--- | | |
| TRINITY_DN27124_c0_g1_i1_len_394_170444.p1_type_internal_len_132 | ---NA--- | | |
| TRINITY_DN12845_c0_g1_i1_len_393_170492.p1_type_internal_len_132 | ---NA--- | | |
| TRINITY_DN31442_c0_g1_i1_len_394_170480.p2_type_internal_len_132 | ---NA--- | | |

| | | | |
|---|---|---|---|
| TRINITY_DN29770_c0_g1_i1_len_394_170467.p1_type_internal_len_132 | ODM99713.1Ribosomal protein S6 kinase delta-1 | F:GO:0004672; F:GO:0005524; P:GO:0006468; F:GO:0016301; P:GO:0016310; F:GO:0035091 | |
| TRINITY_DN34316_c0_g1_i1_len_394_170459.p1_type_internal_len_132 | AGV55415.1innexin 2 | C:GO:0005886; C:GO:0005921; P:GO:0006811; C:GO:0016021 | |
| TRINITY_DN24080_c0_g1_i4_len_393_208383.p1_type_internal_len_132 | ---NA--- | | |
| TRINITY_DN9388_c0_g1_i1_len_393_208386.p1_type_internal_len_132 | ---NA--- | | |
| TRINITY_DN7218_c0_g1_i1_len_393_72652.p1_type_internal_len_132 | ---NA--- | | |
| TRINITY_DN9094_c0_g1_i1_len_393_72651.p1_type_internal_len_132 | ---NA--- | | |
| TRINITY_DN4035_c0_g1_i2_len_393_72632.p2_type_internal_len_132 | XP_018020778.1 xylose isomerase-like | | |
| TRINITY_DN35187_c0_g1_i1_len_394_72627.p2_type_internal_len_132 | ---NA--- | | |
| TRINITY_DN39780_c0_g1_i1_len_393_208333.p1_type_internal_len_132 | ---NA--- | | |
| TRINITY_DN7939_c0_g1_i1_len_393_208362.p1_type_internal_len_132 | ---NA--- | | |
| TRINITY_DN29085_c0_g1_i3_len_393_208368.p1_type_internal_len_132 | ---NA--- | | |
| TRINITY_DN35224_c0_g1_i1_len_396_72538.p2_type_internal_len_132 | CAB85963.1alpha glucosidase | P:GO:0000023; F:GO:0004558; F:GO:0030246; F:GO:0032450 | EC:3.2.1.20 |
| TRINITY_DN9201_c0_g1_i1_len_396_72523.p1_type_internal_len_132 | XP_018020922.1PREDICTED: uncharacterized protein LOC108677237 | | |
| TRINITY_DN2298_c0_g1_i1_len_396_72519.p2_type_internal_len_132 | KYN09248.1Glycerol-3-phosphate acyltransferase 3 | C:GO:0016020; C:GO:0016021; F:GO:0016740; F:GO:0016746 | |
| TRINITY_DN31454_c0_g1_i1_len_397_72510.p1_type_internal_len_132 | ---NA--- | | |
| TRINITY_DN10420_c0_g1_i1_len_395_72551.p1_type_internal_len_132 | XP_018013573.1 protein msta-like | F:GO:0046872 | |

| | | | |
|---|---|---|---|
| TRINITY_DN36404_c0_g1_i2_len_466_244052.p1_type_5prime_partial_len_131 | ---NA--- | | |
| TRINITY_DN18657_c0_g1_i1_len_456_205736.p1_type_5prime_partial_len_131 | ARS88258.1ATP binding cassette transporter 6 | F:GO:0000166; C:GO:0016020 | |
| TRINITY_DN4129_c0_g1_i1_len_458_244290.p1_type_3prime_partial_len_132 | XP_023244525.1protein zyg-11 homolog B-like | C:GO:0031462 | |
| TRINITY_DN8876_c0_g1_i1_len_394_208283.p1_type_internal_len_132 | XP_018016790.1 venom dipeptidyl peptidase 4-like | P:GO:0006508; F:GO:0008236 | |
| TRINITY_DN39206_c0_g1_i1_len_394_208290.p1_type_internal_len_132 | XP_023407289.1multidrug resistance-associated protein 5 isoform X4 | | |
| TRINITY_DN39745_c0_g1_i1_len_394_208295.p1_type_internal_len_132 | ---NA--- | | |
| TRINITY_DN20731_c0_g1_i1_len_1151_195292.p1_type_5prime_partial_len_131 | ---NA--- | | |
| TRINITY_DN123_c0_g1_i1_len_394_208316.p1_type_internal_len_132 | AGW23859.1cAMP specific phosphodiesterase | F:GO:0004114; P:GO:0007165 | EC:3.1.4.17 |
| TRINITY_DN32864_c0_g1_i1_len_394_208318.p1_type_internal_len_132 | KDR09067.1Ubiquitin-conjugating enzyme E2 O | | |
| TRINITY_DN37476_c0_g1_i1_len_394_208310.p1_type_internal_len_132 | XP_018012344.1 dystonin-like isoform X2 | | |
| TRINITY_DN36219_c0_g1_i1_len_394_208311.p1_type_internal_len_132 | ---NA--- | | |
| TRINITY_DN20024_c0_g1_i2_len_542_68146.p2_type_5prime_partial_len_131 | AYA22651.1argininosuccinate lyase-like protein | F:GO:0016829 | |
| TRINITY_DN30386_c0_g1_i1_len_396_246573.p1_type_internal_len_132 | ---NA--- | | |
| TRINITY_DN1773_c0_g1_i1_len_395_246583.p1_type_internal_len_132 | ---NA--- | | |
| TRINITY_DN27593_c0_g1_i1_len_395_246590.p1_type_internal_len_132 | ---NA--- | | |
| TRINITY_DN1190_c0_g1_i1_len_395_246594.p1_type_internal_len_132 | ---NA--- | | |

| | | | |
|---|---|---|---|
| TRINITY_DN11653_c0_g1_i1_len_395_246602.p1_type_internal_len_132 | ---NA--- | | |
| TRINITY_DN33226_c0_g1_i1_len_395_246603.p1_type_internal_len_132 | XP_026294213.1 TOM1-like protein 2 | | |
| TRINITY_DN9004_c0_g1_i1_len_395_246607.p1_type_internal_len_132 | XP_023720005.1RIMS-binding protein 2 | | |
| TRINITY_DN15923_c0_g1_i1_len_399_246432.p1_type_5prime_partial_len_131 | XP_014677047.1PREDICTED: uncharacterized protein LOC106816918 | | |
| TRINITY_DN30394_c0_g1_i1_len_397_246500.p1_type_internal_len_132 | ---NA--- | | |
| TRINITY_DN1547_c0_g1_i1_len_397_246526.p1_type_internal_len_132 | ---NA--- | | |
| TRINITY_DN5447_c0_g1_i1_len_396_246533.p1_type_internal_len_132 | ---NA--- | | |
| TRINITY_DN2950_c0_g1_i1_len_396_246539.p1_type_internal_len_132 | ---NA--- | | |
| TRINITY_DN33050_c0_g1_i1_len_396_246559.p1_type_internal_len_132 | KZS13329.1Ankyrin repeat and ibr domain containing | F:GO:0016740 | |
| TRINITY_DN6272_c0_g1_i1_len_435_71053.p1_type_5prime_partial_len_131 | XP_023295524.1protein PET117 homolog, mitochondrial | C:GO:0016021 | |
| TRINITY_DN19601_c0_g1_i2_len_469_205242.p1_type_5prime_partial_len_131 | ---NA--- | | |
| TRINITY_DN37285_c0_g1_i1_len_395_246611.p1_type_internal_len_132 | ---NA--- | | |
| TRINITY_DN33855_c0_g1_i1_len_395_246615.p1_type_internal_len_132 | ---NA--- | | |
| TRINITY_DN35817_c0_g1_i1_len_395_246620.p1_type_internal_len_132 | XP_018017378.1 protocadherin Fat 1-like | F:GO:0005509; C:GO:0005886; P:GO:0007155; P:GO:0007156; C:GO:0016020 | |
| TRINITY_DN34515_c0_g1_i1_len_394_246623.p1_type_internal_len_132 | XP_019772143.1 follicle-stimulating hormone receptor-like | F:GO:0004930; P:GO:0007186; C:GO:0016021; F:GO:0016500 | |
| TRINITY_DN30340_c0_g1_i1_len_394_246629.p1_type_internal_len_132 | XP_018008386.1 myoferlin-like | | |

| | | | |
|---|---|---|---|
| TRINITY_DN1045_c0_g1_i1_len_394_246656.p1_type_internal_len_132 | XP_014216755.1phosphorylase b kinase gamma catalytic chain, skeletal muscle/heart isoform isoform X2 | F:GO:0004689; F:GO:0005516; F:GO:0005524; C:GO:0005964; P:GO:0005978; P:GO:0006468; C:GO:0016021 | EC:2.7.11; EC:2.7.11.19; EC:2.7.11.17 |
| TRINITY_DN32456_c0_g1_i1_len_393_246670.p1_type_internal_len_132 | KFM71705.1Rho guanine nucleotide exchange factor 17 | F:GO:0005089 | |
| TRINITY_DN6312_c0_g1_i1_len_393_246683.p1_type_internal_len_132 | XP_013082349.1 protein DENND6B-like | F:GO:0017112 | |
| TRINITY_DN12086_c1_g1_i1_len_393_246685.p1_type_internal_len_132 | ---NA--- | | |
| TRINITY_DN15265_c0_g1_i1_len_395_208248.p1_type_internal_len_132 | ---NA--- | | |
| TRINITY_DN603_c0_g1_i1_len_395_208277.p1_type_internal_len_132 | ---NA--- | | |
| TRINITY_DN35921_c0_g1_i1_len_395_208247.p1_type_internal_len_132 | ---NA--- | | |
| TRINITY_DN7254_c0_g1_i1_len_395_208241.p1_type_internal_len_132 | ---NA--- | | |
| 2171\|_\|c1589_f3p1_2670_isoform_c1589_full_length_coverage_3_non_full_length_coverage_1_isoform_length_2670_27850 | ---NA--- | | |
| TRINITY_DN20626_c0_g1_i1_len_2673_229483.p1_type_internal_len_891 | XP_023719239.1laminin subunit alpha | P:GO:0050789 | |
| TRINITY_DN30850_c0_g1_i1_len_2757_188941.p1_type_5prime_partial_len_890 | XP_005111446.2 sortilin-related receptor-like | | |
| TRINITY_DN21675_c0_g1_i1_len_2691_295022.p1_type_5prime_partial_len_890 | XP_023211649.1transcription factor 25-like | | |
| 2176\|_\|c2602_f2p1_2669_isoform_c2602_full_length_coverage_2_non_full_length_coverage_1_isoform_length_2669_21078 | ---NA--- | | |
| 2177\|_\|c2064_f8p3_2669_isoform_c2064_full_length_coverage_8_non_full_length_coverage_3_isoform_length_2669_16160 | XP_019645410.1 transmembrane protein 59-like | C:GO:0016020; C:GO:0016021 | |

<table>
<tr><td>2178|_|c5916_f6p4_2669_isoform_c5916_full_length_coverage_6_non_full_length_coverage_4_isoform_length_2669_21565</td><td>XP_781852.2 methylmalonyl-CoA mutase, mitochondrial isoform X2</td><td>F:GO:0004494; F:GO:0031419; F:GO:0046872</td><td>EC:5.4.99.2</td></tr>
<tr><td>2179|_|c2427_f2p1_2668_isoform_c2427_full_length_coverage_2_non_full_length_coverage_1_isoform_length_2668_11857</td><td>---NA---</td><td></td><td></td></tr>
<tr><td>2180|_|c1826_f2p3_2668_isoform_c1826_full_length_coverage_2_non_full_length_coverage_3_isoform_length_2668_28007</td><td>---NA---</td><td></td><td></td></tr>
<tr><td>TRINITY_DN25727_c1_g1_i3_len_2667_229492.p1_type_internal_len_890</td><td>OQV11796.1putative Serine proteinase stubble</td><td>F:GO:0004252; P:GO:0006508</td><td></td></tr>
<tr><td>TRINITY_DN39828_c0_g1_i1_len_394_208282.p1_type_internal_len_132</td><td>---NA---</td><td></td><td></td></tr>
<tr><td>TRINITY_DN5282_c0_g1_i1_len_395_208238.p1_type_internal_len_132</td><td>---NA---</td><td></td><td></td></tr>
<tr><td>TRINITY_DN37597_c0_g1_i1_len_395_208256.p1_type_internal_len_132</td><td>---NA---</td><td></td><td></td></tr>
<tr><td>TRINITY_DN35234_c0_g1_i1_len_451_205914.p1_type_5prime_partial_len_131</td><td>---NA---</td><td></td><td></td></tr>
<tr><td>TRINITY_DN21911_c0_g1_i1_len_521_242491.p1_type_5prime_partial_len_131</td><td>---NA---</td><td></td><td></td></tr>
<tr><td>TRINITY_DN9428_c0_g1_i1_len_3751_228315.p3_type_5prime_partial_len_131</td><td>---NA---</td><td></td><td></td></tr>
<tr><td>TRINITY_DN41209_c0_g1_i1_len_395_208230.p1_type_internal_len_132</td><td>---NA---</td><td></td><td></td></tr>
<tr><td>TRINITY_DN29852_c0_g1_i1_len_2571_189287.p1_type_5prime_partial_len_131</td><td>---NA---</td><td></td><td></td></tr>
<tr><td>TRINITY_DN8253_c0_g1_i1_len_396_208215.p1_type_internal_len_132</td><td>---NA---</td><td></td><td></td></tr>
<tr><td>TRINITY_DN34299_c0_g1_i1_len_395_208273.p1_type_internal_len_132</td><td>KDR11212.1GTP-binding protein Rit1</td><td>F:GO:0000166; F:GO:0003924; F:GO:0005525; P:GO:0007165; C:GO:0016020</td><td></td></tr>
</table>

| | | | |
|---|---|---|---|
| TRINITY_DN23358_c0_g1_i1_len_3985_228195.p2_type_5prime_partial_len_131 | ---NA--- | | |
| TRINITY_DN11272_c1_g1_i1_len_395_208250.p1_type_internal_len_132 | ADG85659.1lectin D | | |
| TRINITY_DN16284_c0_g1_i1_len_845_64180.p1_type_5prime_partial_len_131 | ---NA--- | | |
| TRINITY_DN19420_c0_g1_i1_len_1727_267003.p5_type_complete_len_130 | ---NA--- | | |
| TRINITY_DN32544_c0_g1_i1_len_440_70925.p1_type_complete_len_130 | ---NA--- | | |
| TRINITY_DN14208_c0_g1_i2_len_390_170614.p1_type_internal_len_131 | ---NA--- | | |
| TRINITY_DN19688_c0_g1_i1_len_4471_153539.p1_type_complete_len_130 | ---NA--- | | |
| TRINITY_DN2618_c0_g1_i1_len_390_246808.p1_type_internal_len_131 | ---NA--- | | |
| TRINITY_DN3672_c0_g1_i1_len_390_246827.p1_type_internal_len_131 | ---NA--- | | |
| TRINITY_DN16398_c0_g1_i1_len_1761_266892.p1_type_complete_len_130 | ---NA--- | | |
| TRINITY_DN1194_c0_g1_i1_len_390_246825.p2_type_internal_len_131 | ---NA--- | | |
| TRINITY_DN2351_c0_g1_i1_len_390_246815.p1_type_internal_len_131 | ---NA--- | | |
| TRINITY_DN1300_c0_g1_i1_len_390_104790.p1_type_internal_len_131 | XP_013102129.1 transmembrane GTPase Marf isoform X5 | F:GO:0003924; F:GO:0005525; C:GO:0005741; P:GO:0008053; C:GO:0016021 | EC:3.6.1.15 |
| TRINITY_DN33036_c0_g1_i1_len_390_246790.p1_type_internal_len_131 | ---NA--- | | |
| TRINITY_DN29289_c0_g1_i1_len_391_246754.p1_type_internal_len_131 | XP_023331424.1uncharacterized protein LOC111703643 isoform X3 | | |
| TRINITY_DN9160_c0_g1_i1_len_578_274502.p1_type_complete_len_130 | XP_026694932.1protein NATD1-like | | |

| | | | |
|---|---|---|---|
| TRINITY_DN3815_c0_g1_i1_len_391_104718.p1_type_internal_len_131 | ---NA--- | | |
| TRINITY_DN22449_c0_g1_i1_len_394_246659.p1_type_internal_len_131 | ---NA--- | | |
| TRINITY_DN19051_c0_g1_i1_len_1482_31507.p3_type_complete_len_130 | ---NA--- | | |
| TRINITY_DN1308_c0_g1_i1_len_393_246667.p1_type_internal_len_131 | XP_018011572.1 teneurin-m-like | | |
| TRINITY_DN31846_c0_g1_i1_len_391_104732.p1_type_internal_len_131 | ---NA--- | | |
| TRINITY_DN8521_c0_g1_i1_len_393_246682.p1_type_internal_len_131 | ---NA--- | | |
| TRINITY_DN19573_c0_g1_i1_len_573_7442.p1_type_complete_len_130 | ---NA--- | | |
| TRINITY_DN27454_c0_g1_i1_len_394_246622.p1_type_3prime_partial_len_131 | XP_018014624.1PREDICTED: uncharacterized protein LOC108671576 | | |
| TRINITY_DN2407_c0_g1_i2_len_392_246714.p1_type_internal_len_131 | ---NA--- | | |
| TRINITY_DN27990_c0_g1_i1_len_391_170586.p1_type_internal_len_131 | KFM81784.1Heparan sulfate glucosamine 3-O-sulfotransferase 5 | F:GO:0008146 | |
| TRINITY_DN5134_c0_g1_i1_len_392_246725.p1_type_internal_len_131 | ---NA--- | | |
| TRINITY_DN33087_c0_g1_i1_len_392_246728.p1_type_internal_len_131 | XP_018021058.1 protein polybromo-1-like | F:GO:0003677; F:GO:0003682; F:GO:0004540; P:GO:0006338; P:GO:0008033; C:GO:0016586; P:GO:0090501 | |
| TRINITY_DN17890_c0_g1_i1_len_1444_31641.p2_type_5prime_partial_len_130 | XP_008311455.1tRNA (adenine(58)-N(1))-methyltransferase non-catalytic subunit TRM6 | | |
| TRINITY_DN6385_c0_g1_i1_len_391_170601.p1_type_internal_len_131 | ---NA--- | | |
| TRINITY_DN15393_c0_g2_i1_len_1471_31542.p3_type_5prime_partial_len_130 | ---NA--- | | |

| | | | |
|---|---|---|---|
| TRINITY_DN4498_c0_g1_i1_len_393_246684.p1_type_internal_len_131 | ---NA--- | | |
| TRINITY_DN32889_c0_g1_i1_len_393_246689.p1_type_internal_len_131 | ---NA--- | | |
| TRINITY_DN15598_c0_g1_i1_len_1272_158922.p2_type_complete_len_130 | ANV22163.1telomerase reverse transcriptase | C:GO:0000781; F:GO:0003677; F:GO:0003721; C:GO:0005634; P:GO:0006278; F:GO:0046872 | EC:2.7.7.49 |
| TRINITY_DN19212_c0_g1_i1_len_391_307382.p1_type_internal_len_131 | ---NA--- | | |
| TRINITY_DN1188_c0_g1_i1_len_481_69620.p1_type_5prime_partial_len_130 | ---NA--- | | |
| TRINITY_DN14735_c0_g1_i1_len_392_307332.p1_type_internal_len_131 | ---NA--- | | |
| TRINITY_DN18309_c0_g1_i4_len_2692_265247.p3_type_complete_len_130 | ---NA--- | | |
| TRINITY_DN23530_c0_g1_i1_len_831_198252.p1_type_complete_len_130 | ABD36317.1mitochondria-associated granulocyte macrophage CSF signaling molecule | C:GO:0005744; P:GO:0030150 | |
| TRINITY_DN22301_c0_g1_i1_len_657_200684.p1_type_5prime_partial_len_130 | ---NA--- | | |
| TRINITY_DN18967_c0_g1_i1_len_567_202491.p1_type_complete_len_130 | XP_018025664.1 transcription initiation factor TFIID subunit 13-like | | |
| TRINITY_DN22986_c0_g1_i3_len_1216_159227.p2_type_complete_len_130 | ---NA--- | | |
| TRINITY_DN22766_c0_g1_i1_len_3356_154012.p2_type_3prime_partial_len_131 | XP_018015919.1 FGFR1 oncogene partner 2 homolog | | |
| TRINITY_DN22378_c0_g1_i2_len_589_202001.p1_type_complete_len_130 | ---NA--- | | |
| TRINITY_DN27942_c0_g1_i5_len_1078_195868.p1_type_5prime_partial_len_130 | AWK58823.1Chibby | | |
| TRINITY_DN12776_c0_g1_i2_len_1359_93801.p3_type_3prime_partial_len_131 | ---NA--- | | |

| | | | |
|---|---|---|---|
| TRINITY_DN2359_c0_g1_i1_len_392_307311.p1_type_internal_len_131 | AKM20741.1putative c-Fos protein | F:GO:0003677; F:GO:0003700; C:GO:0005634; P:GO:0006357 | |
| TRINITY_DN13685_c0_g1_i2_len_568_241426.p1_type_complete_len_130 | ---NA--- | | |
| TRINITY_DN16511_c0_g1_i2_len_548_68043.p1_type_5prime_partial_len_130 | ---NA--- | | |
| TRINITY_DN17680_c0_g1_i1_len_700_301114.p1_type_3prime_partial_len_131 | ---NA--- | | |
| TRINITY_DN6256_c0_g1_i1_len_535_68290.p1_type_5prime_partial_len_130 | AFE88209.1protaglandin reductase 1 | P:GO:0055114 | |
| TRINITY_DN10555_c0_g1_i1_len_598_165027.p1_type_5prime_partial_len_130 | XP_018010527.1 TATA element modulatory factor-like | | |
| TRINITY_DN28798_c0_g1_i1_len_392_279257.p1_type_internal_len_131 | ---NA--- | | |
| TRINITY_DN8194_c0_g1_i1_len_419_134722.p1_type_5prime_partial_len_130 | ---NA--- | | |
| TRINITY_DN33727_c0_g1_i1_len_396_135547.p1_type_5prime_partial_len_130 | EEN67923.1hypothetical protein BRAFLDRAFT_62306 | F:GO:0016831; P:GO:0019428 | |
| TRINITY_DN24727_c0_g1_i1_len_396_135546.p1_type_5prime_partial_len_130 | ---NA--- | | |
| TRINITY_DN24881_c0_g1_i1_len_392_279244.p1_type_internal_len_131 | ---NA--- | | |
| TRINITY_DN9034_c0_g1_i1_len_757_238447.p2_type_3prime_partial_len_131 | ---NA--- | | |
| TRINITY_DN11808_c0_g1_i2_len_391_279283.p1_type_internal_len_131 | ---NA--- | | |
| TRINITY_DN6273_c0_g1_i1_len_430_103289.p1_type_5prime_partial_len_130 | ALO17554.1G protein-coupled receptor kinase 2 | F:GO:0016301; P:GO:0016310 | |
| TRINITY_DN24783_c0_g1_i1_len_525_8311.p2_type_5prime_partial_len_130 | ---NA--- | | |
| TRINITY_DN13237_c0_g1_i1_len_768_238334.p1_type_complete_len_130 | ---NA--- | | |

| | | | |
|---|---|---|---|
| TRINITY_DN16382_c0_g1_i1_len_392_279274.p1_type_internal_len_131 | ---NA--- | | |
| TRINITY_DN10800_c0_g1_i1_len_393_135668.p1_type_internal_len_131 | ---NA--- | | |
| TRINITY_DN27055_c0_g1_i1_len_390_135785.p1_type_internal_len_131 | ACO12292.1Cell adhesion molecule 3 precursor | C:GO:0016020; C:GO:0016021 | |
| TRINITY_DN28884_c0_g1_i1_len_392_279238.p1_type_internal_len_131 | ---NA--- | | |
| TRINITY_DN7518_c0_g1_i1_len_394_135638.p1_type_internal_len_131 | ---NA--- | | |
| TRINITY_DN8500_c0_g1_i1_len_489_276301.p1_type_5prime_partial_len_130 | ---NA--- | | |
| TRINITY_DN13470_c0_g1_i1_len_516_275670.p1_type_5prime_partial_len_130 | ---NA--- | | |
| TRINITY_DN7683_c0_g1_i2_len_415_169653.p1_type_5prime_partial_len_130 | KZS15587.1E3 ubiquitin-protein ligase MARCH2 | F:GO:0008270; C:GO:0016021 | |
| TRINITY_DN7945_c0_g1_i1_len_393_72638.p1_type_internal_len_131 | ---NA--- | | |
| TRINITY_DN26407_c0_g1_i2_len_1205_194926.p2_type_5prime_partial_len_130 | ---NA--- | | |
| TRINITY_DN18778_c0_g1_i1_len_558_131084.p2_type_5prime_partial_len_130 | ---NA--- | | |
| TRINITY_DN4393_c0_g1_i2_len_394_72608.p1_type_internal_len_131 | ODM95030.1Acetyl-CoA acetyltransferase, cytosolic | F:GO:0016747 | |
| TRINITY_DN16837_c0_g2_i1_len_509_242804.p1_type_5prime_partial_len_130 | AAP92779.1hemocyte kazal-type proteinase inhibitor | | |
| TRINITY_DN10772_c0_g1_i1_len_844_64185.p1_type_5prime_partial_len_130 | XP_026687357.1stress-associated endoplasmic reticulum protein 2-like | C:GO:0005783; C:GO:0016021 | |
| TRINITY_DN5951_c0_g1_i2_len_393_104663.p1_type_internal_len_131 | ---NA--- | | |
| TRINITY_DN27387_c0_g1_i1_len_393_104672.p1_type_internal_len_131 | XP_023714210.1importin-7 isoform X1 | C:GO:0005634; P:GO:0006886; F:GO:0008536 | |

| | | | |
|---|---|---|---|
| TRINITY_DN6370_c0_g1_i1_len_503_242968.p1_type_3prime_partial_len_131 | XP_018021621.1PREDICTED: uncharacterized protein CG10915-like isoform X2 | | |
| TRINITY_DN3014_c0_g1_i2_len_402_72265.p1_type_5prime_partial_len_130 | ---NA--- | | |
| TRINITY_DN21047_c0_g4_i2_len_502_304121.p1_type_complete_len_130 | ---NA--- | | |
| TRINITY_DN10715_c0_g1_i1_len_683_239451.p1_type_5prime_partial_len_130 | ---NA--- | | |
| TRINITY_DN9782_c0_g1_i2_len_391_279306.p1_type_internal_len_131 | ---NA--- | | |
| TRINITY_DN22304_c0_g1_i2_len_816_64449.p2_type_complete_len_130 | XP_018018035.1 uncharacterized oxidoreductase YjmC-like | F:GO:0016491; P:GO:0055114 | |
| TRINITY_DN486_c0_g1_i1_len_405_104181.p1_type_5prime_partial_len_130 | ---NA--- | | |
| TRINITY_DN26126_c0_g1_i1_len_391_279315.p1_type_internal_len_131 | XP_013111191.1 H(+)/Cl(-) exchange transporter 5 isoform X2 | P:GO:0006810 | |
| TRINITY_DN20681_c0_g1_i1_len_1677_92834.p3_type_5prime_partial_len_130 | ---NA--- | | |
| TRINITY_DN9348_c0_g1_i1_len_392_72692.p1_type_internal_len_131 | ---NA--- | | |
| TRINITY_DN19611_c0_g1_i1_len_392_72689.p1_type_internal_len_131 | EZA48187.1Methyltransferase-like protein | P:GO:0006479; F:GO:0008168; F:GO:0008276; F:GO:0016740; P:GO:0032259 | |
| TRINITY_DN13521_c0_g1_i2_len_428_169157.p1_type_5prime_partial_len_130 | ---NA--- | | |
| 2183\|_\|c17994_f2p1_2666_isoform_c17994_full_length_coverage_2_non_full_length_coverage_1_isoform_length_2666_17882 | XP_018020149.1 transmembrane protein 184B-like isoform X3 | | |
| 2185\|_\|c18684_f3p2_2666_isoform_c18684_full_length_coverage_3_non_full_length_coverage_2_isoform_length_2666_28844 | ADK63101.1thrombospondin protein | F:GO:0005488 | |

| | | | |
|---|---|---|---|
| 2186\|_\|c10588_f3p0_2666_isoform_c10588_full_length_coverage_3_non_full_length_coverage_0_isoform_length_2666_12903 | XP_018008430.1PREDICTED: uncharacterized protein LOC108666125 isoform X1 | C:GO:0016020 | |
| 2187\|_\|c4369_f3p0_2666_isoform_c4369_full_length_coverage_3_non_full_length_coverage_0_isoform_length_2666_16848 | XP_018008424.1 actin-related protein 10-like | P:GO:0009987 | |
| 2188\|_\|c6089_f4p1_2665_isoform_c6089_full_length_coverage_4_non_full_length_coverage_1_isoform_length_2665_21579 | XP_014779603.1 TBC1 domain family member 31-like | | |
| 2189\|_\|c2281_f3p42_2665_isoform_c2281_full_length_coverage_3_non_full_length_coverage_42_isoform_length_2665_16290 | ---NA--- | | |
| 2190\|_\|c2682_f3p1_2665_isoform_c2682_full_length_coverage_3_non_full_length_coverage_1_isoform_length_2665_21147 | PNF26095.1RNA polymerase II subunit B1 CTD phosphatase Rpap2 | F:GO:0008420; F:GO:0043175; P:GO:0070940 | |
| 2191\|_\|c8790_f4p5_2665_isoform_c8790_full_length_coverage_4_non_full_length_coverage_5_isoform_length_2665_17226 | XP_018023262.1 chitobiosyldiphosphodolichol beta-mannosyltransferase-like | | |
| TRINITY_DN22236_c0_g1_i1_len_3398_153980.p1_type_5prime_partial_len_888 | XP_022338755.1piwi-like protein 1 isoform X2 | F:GO:0003676; P:GO:0031047 | |
| 2193\|_\|c20991_f2p1_2664_isoform_c20991_full_length_coverage_2_non_full_length_coverage_1_isoform_length_2664_22825 | XP_022241978.1mitotic checkpoint serine/threonine-protein kinase BUB1-like isoform X1 | | |
| TRINITY_DN14424_c0_g1_i1_len_928_161254.p1_type_complete_len_130 | XP_019742167.1 tigger transposable element-derived protein 1-like | | |
| TRINITY_DN30133_c0_g1_i1_len_393_72664.p1_type_internal_len_131 | XP_023018867.1laminin subunit gamma-1-like | | |
| TRINITY_DN13050_c0_g1_i2_len_393_72663.p1_type_internal_len_131 | ASK05861.1chitin-binding protein | F:GO:0042302 | |
| TRINITY_DN2463_c0_g1_i1_len_393_72662.p1_type_5prime_partial_len_130 | ---NA--- | | |
| TRINITY_DN10013_c0_g1_i1_len_391_72736.p2_type_internal_len_131 | ---NA--- | | |

| | | | |
|---|---|---|---|
| TRINITY_DN34969_c0_g1_i1_len_392_72713.p1_type_internal_len_131 | ---NA--- | | |
| TRINITY_DN25043_c0_g1_i1_len_2233_230421.p2_type_complete_len_130 | AHM88206.1pancreatic lipase | C:GO:0005576; P:GO:0006629; F:GO:0046872; F:GO:0052689 | EC:3.1.1.1 |
| TRINITY_DN18344_c0_g1_i7_len_2213_91800.p4_type_5prime_partial_len_130 | ---NA--- | | |
| TRINITY_DN23366_c0_g1_i1_len_391_12057.p1_type_internal_len_131 | ---NA--- | | |
| TRINITY_DN25340_c0_g2_i1_len_2098_230794.p2_type_5prime_partial_len_130 | ---NA--- | | |
| TRINITY_DN3622_c0_g1_i1_len_394_11944.p1_type_internal_len_131 | XP_019628476.1 sorting nexin-17-like | C:GO:0005769; C:GO:0005856; P:GO:0006886; P:GO:0007165; F:GO:0035091; P:GO:1990126 | |
| TRINITY_DN3300_c0_g1_i1_len_401_11682.p1_type_5prime_partial_len_130 | ---NA--- | | |
| TRINITY_DN14211_c0_g1_i1_len_392_12008.p1_type_internal_len_131 | ---NA--- | | |
| TRINITY_DN13938_c0_g1_i1_len_392_12016.p1_type_internal_len_131 | ---NA--- | | |
| TRINITY_DN31152_c0_g1_i1_len_4619_187263.p2_type_complete_len_130 | ---NA--- | | |
| TRINITY_DN22507_c0_g1_i5_len_736_199487.p1_type_5prime_partial_len_130 | ---NA--- | | |
| TRINITY_DN23960_c0_g1_i1_len_393_11989.p1_type_internal_len_131 | ---NA--- | | |
| TRINITY_DN16749_c0_g1_i1_len_392_12007.p1_type_internal_len_131 | ---NA--- | | |
| TRINITY_DN7328_c0_g1_i1_len_390_208499.p1_type_internal_len_131 | ---NA--- | | |
| TRINITY_DN39526_c0_g1_i1_len_390_208510.p1_type_internal_len_131 | ---NA--- | | |
| TRINITY_DN22050_c0_g1_i1_len_2741_91303.p2_type_complete_len_130 | ---NA--- | | |

| | | | |
|---|---|---|---|
| TRINITY_DN10751_c0_g1_i1_len_390_41838.p1_type_internal_len_131 | BBC20718.1integrin alpha V | C:GO:0016021 | |
| TRINITY_DN4369_c0_g1_i1_len_392_208413.p2_type_internal_len_131 | ---NA--- | | |
| TRINITY_DN13571_c0_g1_i1_len_392_208415.p1_type_internal_len_131 | ---NA--- | | |
| TRINITY_DN5157_c0_g1_i1_len_392_208418.p1_type_internal_len_131 | ---NA--- | | |
| TRINITY_DN18750_c0_g1_i1_len_392_208421.p1_type_internal_len_131 | ---NA--- | | |
| TRINITY_DN38017_c0_g1_i1_len_392_208422.p1_type_internal_len_131 | XP_023296228.1transient receptor potential cation channel subfamily A member 1 isoform X1 | P:GO:0006810 | |
| TRINITY_DN8795_c0_g1_i1_len_392_208425.p1_type_internal_len_131 | XP_018577624.1membrane-bound transcription factor site-1 protease isoform X1 | F:GO:0004252; P:GO:0006508; C:GO:0016021 | EC:3.4.21 |
| TRINITY_DN33570_c0_g1_i1_len_392_208429.p1_type_internal_len_131 | XP_013401496.1serine/threonine-protein kinase WNK4-like isoform X1 | F:GO:0004674; F:GO:0005524; C:GO:0005737; P:GO:0006468; P:GO:0010766; F:GO:0019869; F:GO:0019870; P:GO:0035556; P:GO:0050801; P:GO:1903288; P:GO:2000651 | EC:2.7.11 |
| TRINITY_DN13166_c0_g1_i1_len_392_208433.p1_type_5prime_partial_len_130 | ---NA--- | | |
| TRINITY_DN4462_c0_g1_i1_len_391_208443.p1_type_internal_len_131 | XP_018011788.1PREDICTED: uncharacterized protein LOC108669014 | | |
| TRINITY_DN34025_c0_g1_i1_len_391_208444.p1_type_internal_len_131 | ---NA--- | | |
| TRINITY_DN5348_c0_g1_i2_len_391_208448.p1_type_internal_len_131 | ALC78853.1adenosine monophosphate deaminase 2 | | |
| TRINITY_DN3144_c0_g1_i1_len_391_208452.p1_type_internal_len_131 | ---NA--- | | |

| | | | |
|---|---|---|---|
| TRINITY_DN2659_c1_g1_i1_len_391_208456.p1_type_internal_len_131 | ---NA--- | | |
| TRINITY_DN36070_c0_g1_i1_len_391_208457.p1_type_internal_len_131 | ---NA--- | | |
| TRINITY_DN6823_c0_g1_i1_len_391_208458.p1_type_internal_len_131 | XP_018904795.1 dystrobrevin beta-like isoform X5 | | |
| TRINITY_DN41449_c0_g1_i1_len_391_208463.p1_type_internal_len_131 | ---NA--- | | |
| TRINITY_DN24584_c1_g3_i2_len_391_208466.p1_type_internal_len_131 | ---NA--- | | |
| TRINITY_DN35062_c0_g1_i1_len_391_208472.p1_type_internal_len_131 | XP_018024941.1 endothelin-converting enzyme 1-like | | |
| TRINITY_DN1786_c0_g1_i1_len_391_208477.p1_type_internal_len_131 | ABD94502.1ubiquitin-conjugating BIR-domain enzyme | P:GO:0009987 | |
| TRINITY_DN17899_c0_g1_i1_len_390_208483.p1_type_internal_len_131 | XP_023227337.1A disintegrin and metalloproteinase with thrombospondin motifs 3-like | | |
| TRINITY_DN31663_c0_g1_i1_len_390_208489.p1_type_internal_len_131 | BAC56455.1 elongation factor 1 alpha | F:GO:0003746; F:GO:0005525; P:GO:0006414 | |
| TRINITY_DN19658_c0_g1_i6_len_5463_227693.p4_type_5prime_partial_len_130 | ---NA--- | | |
| TRINITY_DN2651_c0_g1_i1_len_399_41506.p1_type_5prime_partial_len_130 | CCP46946.1onychopsin | P:GO:0007165; C:GO:0016020; F:GO:0038023 | |
| TRINITY_DN12193_c0_g1_i1_len_394_208284.p1_type_internal_len_131 | XP_023237743.1disheveled-associated activator of morphogenesis 1-like isoform X2 | | |
| TRINITY_DN16655_c0_g1_i1_len_743_199385.p1_type_3prime_partial_len_131 | ---NA--- | | |
| TRINITY_DN5981_c0_g1_i1_len_394_208297.p1_type_internal_len_131 | BAO20841.1RNA polymerase II largest subunit | F:GO:0003677; F:GO:0003899; C:GO:0005665; P:GO:0006366 | EC:2.7.7.6 |
| TRINITY_DN29644_c0_g1_i2_len_2492_189466.p2_type_3prime_partial_len_131 | ---NA--- | | |

| | | | |
|---|---|---|---|
| TRINITY_DN3804_c0_g1_i1_len_393_208340.p1_type_internal_len_131 | XP_018016096.1 irregular chiasm C-roughest protein-like | C:GO:0016020; C:GO:0016021 | |
| TRINITY_DN30629_c0_g2_i2_len_393_208351.p1_type_internal_len_131 | ---NA--- | | |
| TRINITY_DN7491_c0_g1_i1_len_393_208358.p1_type_internal_len_131 | ---NA--- | | |
| TRINITY_DN12482_c0_g1_i1_len_393_208360.p1_type_internal_len_131 | ---NA--- | | |
| TRINITY_DN8142_c0_g1_i1_len_393_208365.p1_type_internal_len_131 | ---NA--- | | |
| TRINITY_DN9722_c0_g1_i1_len_393_208385.p1_type_internal_len_131 | ---NA--- | | |
| TRINITY_DN9071_c0_g1_i1_len_392_208391.p1_type_internal_len_131 | PSN36343.1ADP-dependent glucokinase | P:GO:0008152; F:GO:0016772 | |
| TRINITY_DN2317_c0_g1_i1_len_392_208395.p1_type_internal_len_131 | ASU87566.1sodium-hydrogen exchanger 3 | P:GO:0006885; F:GO:0015385; C:GO:0016021; P:GO:0035725; P:GO:1902600 | |
| TRINITY_DN40528_c0_g1_i1_len_392_208402.p1_type_internal_len_131 | ESO94103.1hypothetical protein LOTGIDRAFT_175496, partial | F:GO:0005506; P:GO:0006537; F:GO:0010181; F:GO:0016040; F:GO:0050660; F:GO:0051538; P:GO:0055114 | EC:1.4.1.14 |
| TRINITY_DN6513_c0_g1_i1_len_392_208407.p1_type_internal_len_131 | XP_018020972.1 synaptojanin-1-like isoform X4 | | |
| TRINITY_DN11904_c0_g1_i1_len_392_208411.p1_type_internal_len_131 | ---NA--- | | |
| TRINITY_DN40772_c0_g1_i1_len_392_208412.p1_type_5prime_partial_len_130 | ---NA--- | | |
| TRINITY_DN31130_c1_g1_i2_len_394_208300.p1_type_internal_len_131 | ---NA--- | | |
| TRINITY_DN21794_c0_g1_i3_len_1020_3497.p2_type_5prime_partial_len_130 | ---NA--- | | |
| TRINITY_DN11520_c0_g1_i1_len_393_208332.p1_type_internal_len_131 | ---NA--- | | |

| | | | |
|---|---|---|---|
| TRINITY_DN10097_c0_g1_i1_len_393_208339.p1_type_internal_len_131 | ---NA--- | | |
| TRINITY_DN24753_c0_g1_i1_len_446_10231.p1_type_5prime_partial_len_130 | ---NA--- | | |
| TRINITY_DN21143_c0_g2_i1_len_1373_193822.p2_type_complete_len_130 | ---NA--- | | |
| TRINITY_DN6027_c0_g1_i1_len_518_203689.p1_type_5prime_partial_len_130 | XP_003696850.1 mitochondrial amidoxime reducing component 2 | F:GO:0003824; F:GO:0030151; F:GO:0030170 | |
| TRINITY_DN13244_c0_g1_i1_len_465_9708.p1_type_5prime_partial_len_130 | XP_018015635.1 src substrate protein p85-like | | |
| TRINITY_DN27548_c0_g1_i1_len_391_279287.p1_type_internal_len_130 | ---NA--- | | |
| TRINITY_DN22049_c0_g1_i1_len_1280_194392.p1_type_5prime_partial_len_129 | ALG65274.1glucose transporter 2 | P:GO:0008643; C:GO:0016021; F:GO:0022857; P:GO:0055085 | |
| TRINITY_DN23878_c0_g1_i1_len_766_199048.p1_type_3prime_partial_len_130 | ---NA--- | | |
| TRINITY_DN3004_c0_g1_i1_len_389_279375.p1_type_internal_len_130 | XP_017762291.1 putative inorganic phosphate cotransporter isoform X3 | | |
| TRINITY_DN19690_c0_g1_i1_len_1261_94209.p2_type_5prime_partial_len_129 | XP_023230021.1glyoxylate reductase/hydroxypyruvate reductase-like isoform X2 | | |
| TRINITY_DN30498_c0_g1_i1_len_2405_189684.p2_type_complete_len_129 | XP_013109033.1 cytochrome b5 reductase 4 | F:GO:0016491; F:GO:0020037; F:GO:0046872; P:GO:0055114 | |
| TRINITY_DN4846_c0_g1_i1_len_395_208235.p1_type_5prime_partial_len_129 | ---NA--- | | |
| TRINITY_DN28397_c0_g1_i1_len_2411_189666.p2_type_complete_len_129 | ---NA--- | | |
| TRINITY_DN20122_c0_g1_i3_len_391_307380.p1_type_internal_len_130 | ---NA--- | | |
| TRINITY_DN6151_c0_g1_i1_len_388_279409.p1_type_internal_len_130 | ---NA--- | | |
| TRINITY_DN29820_c0_g1_i1_len_390_279340.p1_type_internal_len_130 | XP_023330579.1hemicentin-2-like | | |

| | | | |
|---|---|---|---|
| TRINITY_DN6566_c0_g1_i1_len_389_279349.p1_type_internal_len_130 | ---NA--- | | |
| TRINITY_DN9809_c0_g1_i1_len_980_196725.p1_type_complete_len_129 | ---NA--- | | |
| TRINITY_DN30260_c0_g1_i1_len_2381_189749.p2_type_complete_len_129 | ---NA--- | | |
| TRINITY_DN3112_c0_g1_i1_len_686_98249.p1_type_complete_len_129 | PNF21507.1Transcription initiation factor TFIID subunit 10 | F:GO:0003743; C:GO:0005634; P:GO:0006352; P:GO:0006413 | |
| TRINITY_DN35676_c0_g1_i1_len_446_244714.p1_type_complete_len_129 | ---NA--- | | |
| TRINITY_DN17577_c0_g1_i1_len_553_37681.p1_type_5prime_partial_len_129 | ---NA--- | | |
| TRINITY_DN9553_c0_g1_i1_len_404_278841.p1_type_5prime_partial_len_129 | ---NA--- | | |
| TRINITY_DN8739_c0_g1_i2_len_452_244482.p1_type_complete_len_129 | ---NA--- | | |
| TRINITY_DN13808_c0_g1_i2_len_998_160682.p2_type_3prime_partial_len_130 | ---NA--- | | |
| TRINITY_DN23308_c0_g1_i1_len_460_244256.p1_type_5prime_partial_len_129 | ---NA--- | | |
| TRINITY_DN21935_c0_g1_i5_len_1789_156725.p3_type_complete_len_129 | XP_018012091.1 protein FAM50A-like isoform X2 | | |
| TRINITY_DN19093_c0_g1_i1_len_993_160726.p1_type_complete_len_129 | ---NA--- | | |
| TRINITY_DN8854_c0_g1_i1_len_633_301917.p1_type_complete_len_129 | XP_022908367.1NHP2-like protein 1 homolog | F:GO:0003723; C:GO:0005730; P:GO:0042254; C:GO:1990904 | |
| TRINITY_DN24130_c0_g1_i1_len_406_278747.p1_type_5prime_partial_len_129 | ---NA--- | | |
| TRINITY_DN7729_c0_g1_i1_len_394_208292.p1_type_5prime_partial_len_129 | XP_005153004.1 peroxisomal carnitine O-octanoyltransferase | F:GO:0016746 | |
| TRINITY_DN7264_c0_g1_i1_len_487_243369.p1_type_5prime_partial_len_129 | ---NA--- | | |

<table>
<tr><td>TRINITY_DN25469_c0_g1_i3_len_1332_233862.p1_type_complete_len_129</td><td>---NA---</td><td></td><td></td></tr>
<tr><td>TRINITY_DN18040_c0_g1_i2_len_2532_29750.p2_type_complete_len_129</td><td>---NA---</td><td></td><td></td></tr>
<tr><td>TRINITY_DN39665_c0_g1_i1_len_603_201706.p2_type_complete_len_129</td><td>---NA---</td><td></td><td></td></tr>
<tr><td>TRINITY_DN17627_c0_g1_i4_len_1081_33072.p1_type_complete_len_129</td><td>---NA---</td><td></td><td></td></tr>
<tr><td>2194|_|c18085_f2p0_2664_isoform_c18085_full_length_coverage_2_non_full_length_coverage_0_isoform_length_2664_13564</td><td>XP_018916017.1 E3 ubiquitin-protein ligase TRIM9 isoform X2</td><td></td><td></td></tr>
<tr><td>2195|_|c2752_f7p7_2664_isoform_c2752_full_length_coverage_7_non_full_length_coverage_7_isoform_length_2664_21206</td><td>XP_014262214.1NADH-ubiquinone oxidoreductase 75 kDa subunit, mitochondrial</td><td>F:GO:0008137; F:GO:0009055; C:GO:0016020; P:GO:0042773; F:GO:0051536</td><td>EC:1.6.99.5; EC:1.6.99.3; EC:1.6.5.3</td></tr>
<tr><td>TRINITY_DN13119_c0_g1_i1_len_2664_229498.p1_type_internal_len_889</td><td>XP_015512896.1 phosphatidylinositide phosphatase SAC2-like isoform X1</td><td>F:GO:0042578</td><td></td></tr>
<tr><td>2197|_|c13440_f2p3_2664_isoform_c13440_full_length_coverage_2_non_full_length_coverage_3_isoform_length_2664_17592</td><td>XP_018024884.1 protein abrupt-like isoform X19</td><td></td><td></td></tr>
<tr><td>TRINITY_DN30484_c0_g1_i1_len_3324_188106.p1_type_5prime_partial_len_888</td><td>XP_019871859.1 ribosome biogenesis protein BOP1 homolog</td><td>C:GO:0005730; P:GO:0006364; C:GO:0030684; P:GO:0042273</td><td></td></tr>
<tr><td>2199|_|c28718_f2p6_2663_isoform_c28718_full_length_coverage_2_non_full_length_coverage_6_isoform_length_2663_23094</td><td>XP_018017521.1 nicotinamide/nicotinic acid mononucleotide adenylyltransferase 1-like</td><td>F:GO:0000309; F:GO:0004515; F:GO:0005524; P:GO:0009435</td><td>EC:2.7.7.1; EC:2.7.7.18</td></tr>
<tr><td>2200|_|c2663_f3p0_2663_isoform_c2663_full_length_coverage_3_non_full_length_coverage_0_isoform_length_2663_21130</td><td>XP_018025128.1PREDICTED: uncharacterized protein LOC108680745 isoform X4</td><td></td><td></td></tr>
<tr><td>2201|_|c1818_f2p5_2663_isoform_c1818_full_length_coverage_2_non_full_length_coverage_5_isoform_length_2663_28001</td><td>---NA---</td><td></td><td></td></tr>
<tr><td>2202|_|c22611_f2p0_2662_isoform_c22611_full_length_coverage_2_non_full_length_coverage_0_isoform_length_2662_18265</td><td>XP_023222966.1cytohesin-1-like isoform X1</td><td></td><td></td></tr>
</table>

| | | | |
|---|---|---|---|
| TRINITY_DN22808_c0_g1_i1_len_2784_58327.p1_type_complete_len_887 | PNF34613.1WD repeat-containing protein mio | | |
| TRINITY_DN9768_c0_g1_i1_len_566_202505.p1_type_5prime_partial_len_129 | ---NA--- | | |
| TRINITY_DN21813_c0_g1_i1_len_760_272152.p1_type_complete_len_129 | ---NA--- | | |
| TRINITY_DN26040_c0_g1_i1_len_3161_228809.p1_type_complete_len_129 | ---NA--- | | |
| TRINITY_DN7279_c0_g1_i1_len_392_307331.p1_type_5prime_partial_len_129 | ---NA--- | | |
| TRINITY_DN10016_c0_g1_i1_len_391_104726.p1_type_internal_len_130 | ---NA--- | | |
| TRINITY_DN14293_c0_g1_i1_len_389_104828.p2_type_internal_len_130 | ---NA--- | | |
| TRINITY_DN28624_c0_g1_i1_len_390_104794.p1_type_internal_len_130 | ---NA--- | | |
| TRINITY_DN6944_c0_g1_i1_len_1041_62872.p2_type_5prime_partial_len_129 | ---NA--- | | |
| TRINITY_DN4713_c0_g1_i1_len_390_104780.p1_type_5prime_partial_len_129 | ---NA--- | | |
| TRINITY_DN12450_c0_g1_i1_len_1099_159989.p2_type_complete_len_129 | ---NA--- | | |
| TRINITY_DN23893_c0_g1_i1_len_974_196768.p1_type_3prime_partial_len_130 | ---NA--- | | |
| TRINITY_DN39392_c0_g1_i1_len_609_201581.p1_type_5prime_partial_len_129 | ---NA--- | | |
| TRINITY_DN11807_c0_g2_i1_len_388_279424.p1_type_internal_len_130 | ---NA--- | | |
| TRINITY_DN4849_c0_g1_i1_len_387_279457.p1_type_internal_len_130 | ---NA--- | | |
| TRINITY_DN22239_c0_g1_i1_len_387_279475.p1_type_internal_len_130 | XP_026274221.1UHRF1-binding protein 1-like isoform X4 | | |

| | | | |
|---|---|---|---|
| TRINITY_DN18717_c0_g1_i3_len_1390_233561.p2_type_complete_len_129 | ---NA--- | | |
| TRINITY_DN1415_c0_g1_i1_len_511_203887.p1_type_3prime_partial_len_130 | ---NA--- | | |
| TRINITY_DN12482_c0_g1_i1_len_571_7464.p1_type_5prime_partial_len_129 | ---NA--- | | |
| TRINITY_DN28993_c2_g1_i2_len_2510_189429.p2_type_5prime_partial_len_129 | ---NA--- | | |
| TRINITY_DN29384_c1_g1_i17_len_2527_189394.p1_type_complete_len_129 | ---NA--- | | |
| TRINITY_DN22533_c0_g1_i5_len_3323_228630.p5_type_3prime_partial_len_130 | ---NA--- | | |
| TRINITY_DN3153_c0_g1_i1_len_390_246804.p1_type_internal_len_130 | ---NA--- | | |
| TRINITY_DN32817_c0_g1_i1_len_390_246792.p1_type_internal_len_130 | ANW81830.1calcium-calmodulin dependent protein kinase I | F:GO:0004683; F:GO:0005516; F:GO:0005524; P:GO:0006468; P:GO:0022404 | EC:2.7.11; EC:2.7.11.17 |
| TRINITY_DN10638_c0_g1_i4_len_857_271274.p2_type_3prime_partial_len_130 | ---NA--- | | |
| TRINITY_DN7128_c0_g1_i1_len_391_246745.p1_type_internal_len_130 | AET79205.1sterile-alpha and armadillo motif containing protein | P:GO:0009967; P:GO:0034128; F:GO:0035591; P:GO:0048678 | |
| TRINITY_DN26689_c0_g1_i1_len_391_246755.p1_type_internal_len_130 | XP_008560027.1 protein Jumonji | | |
| TRINITY_DN39200_c0_g1_i1_len_387_208662.p1_type_internal_len_130 | ---NA--- | | |
| TRINITY_DN26222_c0_g1_i1_len_426_71346.p1_type_complete_len_129 | ---NA--- | | |
| TRINITY_DN32631_c0_g1_i1_len_389_246855.p1_type_internal_len_130 | ---NA--- | | |
| TRINITY_DN14964_c0_g1_i2_len_1462_267884.p3_type_complete_len_129 | ---NA--- | | |
| TRINITY_DN9426_c0_g1_i1_len_390_246809.p1_type_internal_len_130 | ---NA--- | | |

| | | | |
|---|---|---|---|
| TRINITY_DN26590_c0_g1_i1_len_390_246821.p1_type_internal_len_130 | ---NA--- | | |
| TRINITY_DN26782_c0_g1_i1_len_389_246837.p1_type_internal_len_130 | XP_018027486.1 connectin-like | | |
| TRINITY_DN23602_c0_g1_i1_len_3288_122003.p4_type_complete_len_129 | ---NA--- | | |
| TRINITY_DN1652_c0_g1_i1_len_389_41876.p1_type_internal_len_130 | XP_018010071.1 cytochrome P450 9e2-like | | |
| TRINITY_DN1662_c0_g1_i1_len_388_208605.p1_type_internal_len_130 | XP_018012233.1 epidermal growth factor receptor substrate 15-like 1 isoform X2 | | |
| TRINITY_DN30303_c0_g1_i1_len_389_41880.p1_type_internal_len_130 | ---NA--- | | |
| TRINITY_DN35196_c0_g1_i1_len_388_208603.p1_type_internal_len_130 | ---NA--- | | |
| TRINITY_DN2474_c0_g1_i1_len_388_208599.p3_type_internal_len_130 | ---NA--- | | |
| TRINITY_DN38697_c0_g1_i1_len_389_208569.p1_type_internal_len_130 | ---NA--- | | |
| TRINITY_DN31673_c0_g1_i1_len_389_208572.p1_type_internal_len_130 | XP_018025771.1 cytoplasmic dynein 2 heavy chain 1-like | F:GO:0003777; F:GO:0005524; P:GO:0007018; C:GO:0030286 | |
| TRINITY_DN37623_c0_g1_i1_len_389_208578.p1_type_internal_len_130 | KYB26510.1Inositol hexakisphosphate and diphosphoinositol-pentakisphosphate kinase-like Protein | F:GO:0000829; P:GO:0016310 | |
| TRINITY_DN6950_c0_g1_i1_len_388_208583.p1_type_internal_len_130 | ---NA--- | | |
| TRINITY_DN19328_c0_g1_i2_len_388_208627.p1_type_internal_len_130 | ---NA--- | | |
| TRINITY_DN6505_c0_g1_i2_len_390_41860.p1_type_internal_len_130 | XP_019638053.1 zinc finger protein 239-like | | |
| TRINITY_DN4984_c0_g1_i1_len_387_208632.p1_type_internal_len_130 | ---NA--- | | |
| TRINITY_DN41091_c0_g1_i1_len_387_208639.p1_type_internal_len_130 | ---NA--- | | |

| | | | |
|---|---|---|---|
| TRINITY_DN25406_c0_g1_i1_len_387_208641.p1_type_internal_len_130 | ---NA--- | | |
| TRINITY_DN7712_c0_g1_i1_len_388_208614.p1_type_internal_len_130 | PRD25597.1zinc finger protein | F:GO:0003677 | |
| TRINITY_DN35006_c0_g1_i1_len_388_208615.p1_type_internal_len_130 | XP_018020861.1 sex determination protein fruitless-like isoform X2 | | |
| TRINITY_DN34370_c0_g1_i1_len_388_208618.p1_type_internal_len_130 | ---NA--- | | |
| TRINITY_DN25870_c0_g1_i1_len_5906_227626.p4_type_complete_len_129 | ---NA--- | | |
| TRINITY_DN9634_c0_g1_i2_len_653_36258.p1_type_complete_len_129 | ---NA--- | | |
| TRINITY_DN10008_c0_g1_i1_len_387_246926.p1_type_internal_len_130 | ---NA--- | | |
| TRINITY_DN29335_c0_g1_i1_len_388_246869.p1_type_internal_len_130 | AYA73279.1sirtuin 1 | F:GO:0008270; P:GO:0016575; F:GO:0017136; F:GO:0070403 | EC:3.5.1.98 |
| TRINITY_DN36309_c0_g1_i1_len_387_246909.p1_type_internal_len_130 | ---NA--- | | |
| TRINITY_DN13995_c0_g2_i1_len_387_246910.p1_type_internal_len_130 | ---NA--- | | |
| TRINITY_DN3091_c0_g1_i1_len_387_246911.p1_type_internal_len_130 | ---NA--- | | |
| TRINITY_DN20617_c0_g1_i1_len_574_202330.p1_type_complete_len_129 | ---NA--- | | |
| TRINITY_DN7175_c0_g1_i1_len_816_96911.p1_type_3prime_partial_len_130 | ---NA--- | | |
| TRINITY_DN9134_c0_g1_i1_len_390_72759.p1_type_internal_len_130 | ---NA--- | | |
| TRINITY_DN6448_c0_g1_i1_len_389_72812.p1_type_internal_len_130 | ---NA--- | | |
| TRINITY_DN6220_c0_g1_i1_len_389_72805.p1_type_internal_len_130 | XP_011663638.1 organic cation transporter protein | | |

| | | | |
|---|---|---|---|
| TRINITY_DN25557_c0_g1_i1_len_391_72738.p1_type_internal_len_130 | XP_022094844.1gastrula zinc finger protein XlCGF26.1-like isoform X1 | | |
| TRINITY_DN10184_c0_g1_i3_len_387_72897.p1_type_internal_len_130 | ---NA--- | | |
| TRINITY_DN34043_c0_g1_i1_len_387_72905.p1_type_internal_len_130 | ATU31752.1basement membrane-specific heparan sulfate proteoglycan core protein | F:GO:0005509 | |
| TRINITY_DN18038_c0_g1_i1_len_1920_191186.p5_type_5prime_partial_len_129 | ---NA--- | | |
| TRINITY_DN29491_c0_g1_i1_len_389_72826.p1_type_internal_len_130 | XP_021916301.1tyrosine-protein phosphatase non-receptor type 23-like isoform X3 | F:GO:0016787 | |
| TRINITY_DN15460_c0_g1_i1_len_389_72820.p1_type_internal_len_130 | ---NA--- | | |
| TRINITY_DN14732_c0_g1_i1_len_402_72253.p1_type_5prime_partial_len_129 | ---NA--- | | |
| TRINITY_DN36435_c0_g1_i1_len_389_208534.p1_type_internal_len_130 | KXJ06669.1Retrovirus-related Pol polyprotein from transposon 17.6 | | |
| TRINITY_DN11723_c0_g1_i1_len_390_208527.p1_type_internal_len_130 | XP_018011507.1 SLIT-ROBO Rho GTPase-activating protein 1-like | | |
| TRINITY_DN3703_c1_g1_i1_len_390_208517.p1_type_internal_len_130 | ---NA--- | | |
| TRINITY_DN30985_c0_g1_i1_len_397_72497.p1_type_5prime_partial_len_129 | XP_022194177.1zinc finger protein 26-like isoform X2 | | |
| TRINITY_DN9153_c0_g1_i1_len_613_66873.p1_type_5prime_partial_len_129 | ---NA--- | | |
| TRINITY_DN41277_c0_g1_i1_len_390_208525.p1_type_internal_len_130 | ---NA--- | | |
| TRINITY_DN17763_c0_g1_i1_len_409_71972.p1_type_5prime_partial_len_129 | ---NA--- | | |
| TRINITY_DN11170_c1_g1_i1_len_389_208563.p1_type_internal_len_130 | ---NA--- | | |
| TRINITY_DN16736_c0_g1_i1_len_389_208542.p1_type_internal_len_130 | ---NA--- | | |

| | | | |
|---|---|---|---|
| TRINITY_DN16583_c0_g1_i1_len_389_208546.p1_type_internal_len_130 | ---NA--- | | |
| TRINITY_DN9642_c0_g1_i1_len_389_208550.p1_type_internal_len_130 | ---NA--- | | |
| TRINITY_DN8249_c0_g1_i1_len_391_208446.p1_type_internal_len_130 | ---NA--- | | |
| TRINITY_DN11957_c0_g1_i2_len_387_41955.p1_type_internal_len_130 | ---NA--- | | |
| TRINITY_DN5971_c0_g1_i1_len_387_41957.p2_type_internal_len_130 | XP_018027149.1 neurogenic locus Notch protein-like | F:GO:0005509; P:GO:0007219; P:GO:0007275; C:GO:0016021; P:GO:0030154; P:GO:0050793 | |
| TRINITY_DN19493_c0_g1_i1_len_391_208480.p1_type_internal_len_130 | ---NA--- | | |
| TRINITY_DN14247_c0_g1_i2_len_388_41941.p1_type_internal_len_130 | XP_020632086.1 S-methylmethionine--homocysteine S-methyltransferase BHMT2-like | F:GO:0016740 | |
| TRINITY_DN29080_c0_g1_i1_len_387_307544.p1_type_internal_len_130 | ---NA--- | | |
| TRINITY_DN21815_c0_g1_i3_len_506_204035.p1_type_5prime_partial_len_129 | ---NA--- | | |
| TRINITY_DN13101_c0_g1_i1_len_465_133223.p1_type_3prime_partial_len_130 | ---NA--- | | |
| TRINITY_DN32034_c0_g1_i1_len_494_167185.p1_type_5prime_partial_len_129 | ASK05861.1chitin-binding protein | F:GO:0042302 | |
| TRINITY_DN20697_c0_g1_i1_len_1183_94568.p3_type_5prime_partial_len_129 | ---NA--- | | |
| TRINITY_DN14690_c0_g1_i1_len_729_272490.p1_type_5prime_partial_len_129 | ---NA--- | | |
| TRINITY_DN9964_c0_g1_i1_len_440_305696.p1_type_5prime_partial_len_129 | KMQ90581.1polycomb protein scm | C:GO:0005634; P:GO:0006355; F:GO:0008270 | |
| TRINITY_DN3045_c0_g1_i1_len_524_100784.p1_type_5prime_partial_len_129 | ---NA--- | | |

| | | | |
|---|---|---|---|
| TRINITY_DN40731_c0_g1_i1_len_503_204138.p1_type_5prime_partial_len_129 | ---NA--- | | |
| TRINITY_DN23300_c0_g1_i1_len_791_198721.p2_type_5prime_partial_len_129 | ---NA--- | | |
| TRINITY_DN1342_c0_g1_i1_len_415_207383.p1_type_5prime_partial_len_129 | ---NA--- | | |
| TRINITY_DN16339_c0_g1_i1_len_433_103212.p1_type_5prime_partial_len_129 | ---NA--- | | |
| TRINITY_DN18153_c0_g1_i1_len_1004_236088.p1_type_complete_len_129 | ---NA--- | | |
| TRINITY_DN29031_c0_g1_i1_len_388_12189.p1_type_internal_len_130 | ABW82154.1crustin-like protein | C:GO:0005576; P:GO:0010466; F:GO:0030414 | |
| 2204\|_\|c17636_f6p8_2661_isoform_c17636_full_length_coverage_6_non_full_length_coverage_8_isoform_length_2661_13380 | XP_018024251.1 methionine aminopeptidase 2-like | C:GO:0005737; P:GO:0006508; F:GO:0046872; F:GO:0070006; P:GO:0070084 | EC:3.4.11 |
| 2205\|_\|c2391_f2p3_2661_isoform_c2391_full_length_coverage_2_non_full_length_coverage_3_isoform_length_2661_11831 | AGC92010.1mitogen-activated protein kinase 14 | P:GO:0000165; F:GO:0004707; F:GO:0005524 | EC:2.7.11; EC:2.7.11.24 |
| TRINITY_DN28785_c0_g1_i3_len_5221_187080.p1_type_complete_len_887 | XP_018014382.1 fasciclin-2-like isoform X1 | | |
| TRINITY_DN30794_c0_g1_i1_len_4069_187549.p1_type_complete_len_887 | XP_018899169.1 ras association domain-containing protein 8 isoform X1 | | |
| TRINITY_DN27875_c0_g1_i1_len_3030_188490.p1_type_complete_len_887 | XP_013397109.1NHL repeat-containing protein 2 | C:GO:0005623; P:GO:0045454 | |
| 2209\|_\|c35039_f2p16_2660_isoform_c35039_full_length_coverage_2_non_full_length_coverage_16_isoform_length_2660_14431 | XP_018010631.1 transmembrane protein 209-like | | |
| TRINITY_DN26090_c0_g1_i1_len_3110_228873.p1_type_complete_len_886 | KFM81110.1DNA ligase 4 | P:GO:0006259; F:GO:0016874 | |
| TRINITY_DN23075_c0_g1_i1_len_3705_153807.p1_type_complete_len_886 | XP_018018231.1 LETM1 and EF-hand domain-containing protein 1, mitochondrial-like isoform X2 | | |

| | | | |
|---|---|---|---|
| 2213\|_\|c1427_f15p12_2658_isoform_c1427_full_length_coverage_15_non_full_length_coverage_12_isoform_length_2658_20430 | XP_018008579.1 carnitine O-acetyltransferase-like | F:GO:0016740 | |
| 2215\|_\|c1774_f5p6_2658_isoform_c1774_full_length_coverage_5_non_full_length_coverage_6_isoform_length_2658_15993 | ---NA--- | | |
| TRINITY_DN16121_c0_g1_i2_len_630_164522.p1_type_complete_len_129 | ---NA--- | | |
| TRINITY_DN31159_c0_g1_i1_len_388_12169.p1_type_internal_len_130 | AFP33455.1kinesin-like protein KIF3A | F:GO:0003777; F:GO:0005524; C:GO:0005874; P:GO:0007018; F:GO:0008017 | EC:3.6.1.15 |
| TRINITY_DN17140_c0_g1_i2_len_857_237362.p1_type_3prime_partial_len_130 | ---NA--- | | |
| TRINITY_DN16199_c0_g1_i2_len_466_304987.p1_type_complete_len_129 | AAP92779.1hemocyte kazal-type proteinase inhibitor | | |
| TRINITY_DN39145_c0_g1_i1_len_439_206392.p1_type_5prime_partial_len_129 | APA34037.1seminal fluid protein | F:GO:0016491; P:GO:0055114 | |
| TRINITY_DN7778_c0_g1_i1_len_390_12117.p1_type_5prime_partial_len_129 | ---NA--- | | |
| TRINITY_DN1672_c0_g1_i1_len_604_164928.p2_type_5prime_partial_len_129 | ABI98680.1ubiquitin-conjugating enzyme H1 | F:GO:0005524; F:GO:0016740 | |
| TRINITY_DN24482_c0_g1_i2_len_812_198474.p2_type_5prime_partial_len_129 | ---NA--- | | |
| TRINITY_DN29468_c0_g1_i1_len_389_12153.p1_type_internal_len_130 | XP_006820846.1 rab GTPase-activating protein 1-like | F:GO:0005096; P:GO:0043547 | |
| TRINITY_DN17519_c0_g2_i1_len_389_12131.p1_type_internal_len_130 | ---NA--- | | |
| TRINITY_DN28744_c0_g1_i1_len_387_170750.p1_type_internal_len_130 | ---NA--- | | |
| TRINITY_DN15456_c0_g1_i1_len_527_203480.p1_type_3prime_partial_len_130 | ---NA--- | | |

| | | | |
|---|---|---|---|
| TRINITY_DN6960_c0_g1_i1_len_486_204671.p1_type_3prime_partial_len_130 | XP_018022161.1 histone-lysine N-methyltransferase, H3 lysine-79 specific-like isoform X2 | | |
| TRINITY_DN15254_c0_g1_i1_len_388_170695.p1_type_internal_len_130 | ---NA--- | | |
| TRINITY_DN25873_c0_g1_i1_len_400_307020.p1_type_5prime_partial_len_129 | ---NA--- | | |
| TRINITY_DN8322_c0_g1_i1_len_388_170700.p1_type_internal_len_130 | ---NA--- | | |
| TRINITY_DN2574_c0_g1_i1_len_390_170631.p1_type_internal_len_130 | ---NA--- | | |
| TRINITY_DN4594_c0_g1_i1_len_390_170633.p1_type_internal_len_130 | ---NA--- | | |
| TRINITY_DN10533_c0_g1_i1_len_389_170653.p1_type_internal_len_130 | CZT16305.1related to non-ribosomal peptide synthetase | F:GO:0003677; F:GO:0003712; P:GO:0006352; P:GO:0006357; C:GO:0016592; F:GO:0016874; F:GO:0031177 | |
| TRINITY_DN4551_c0_g1_i1_len_498_204321.p1_type_complete_len_129 | BAM35674.1putative reverse transcriptase | F:GO:0003964; P:GO:0006278 | |
| TRINITY_DN1207_c0_g1_i3_len_551_131248.p1_type_5prime_partial_len_129 | EZA56243.1[Pyruvate dehydrogenase [lipoamide]] kinase, mitochondrial | F:GO:0004672; P:GO:0006468 | |
| TRINITY_DN28028_c0_g1_i1_len_411_306631.p1_type_5prime_partial_len_129 | ---NA--- | | |
| TRINITY_DN19405_c0_g1_i1_len_670_6063.p1_type_5prime_partial_len_129 | ---NA--- | | |
| TRINITY_DN17919_c0_g1_i1_len_406_207756.p1_type_5prime_partial_len_129 | ---NA--- | | |
| TRINITY_DN15978_c0_g1_i1_len_1452_193350.p1_type_complete_len_129 | ---NA--- | | |
| TRINITY_DN24680_c0_g1_i1_len_389_135844.p1_type_internal_len_130 | XP_020369211.1 sushi, von Willebrand factor type A, EGF and pentraxin domain-containing protein 1-like | | |

| | | | |
|---|---|---|---|
| TRINITY_DN33264_c0_g1_i1_len_389_135837.p1_type_internal_len_130 | ---NA--- | | |
| TRINITY_DN28388_c0_g1_i1_len_389_135826.p1_type_internal_len_130 | PFX13290.1Retrovirus-related Pol polyprotein | | |
| TRINITY_DN11799_c0_g1_i1_len_494_304307.p1_type_5prime_partial_len_129 | ---NA--- | | |
| TRINITY_DN13820_c0_g1_i1_len_388_135863.p1_type_internal_len_130 | ---NA--- | | |
| TRINITY_DN2220_c0_g1_i2_len_390_135772.p1_type_internal_len_130 | ---NA--- | | |
| TRINITY_DN8020_c0_g1_i2_len_387_135943.p1_type_internal_len_130 | XP_026273760.1endoplasmic reticulum junction formation protein lunapark-A isoform X2 | | |
| TRINITY_DN33631_c0_g1_i1_len_387_135924.p1_type_internal_len_130 | APG53779.1transient receptor potential cation channel subfamily A member 1 | F:GO:0005216; C:GO:0016021; P:GO:0034220 | |
| TRINITY_DN20022_c0_g1_i2_len_740_199426.p1_type_3prime_partial_len_130 | ---NA--- | | |
| TRINITY_DN14123_c0_g1_i2_len_506_304021.p1_type_complete_len_129 | XP_014298367.1 afadin-like isoform X2 | C:GO:0005911; P:GO:0007165 | |
| TRINITY_DN17375_c0_g1_i1_len_677_239531.p2_type_complete_len_129 | ---NA--- | | |
| TRINITY_DN20268_c0_g1_i1_len_1012_160570.p1_type_complete_len_128 | ---NA--- | | |
| TRINITY_DN3800_c0_g1_i1_len_387_208661.p1_type_internal_len_129 | ---NA--- | | |
| TRINITY_DN30555_c0_g1_i1_len_391_246762.p1_type_5prime_partial_len_128 | ---NA--- | | |
| TRINITY_DN31266_c0_g1_i1_len_392_246729.p1_type_complete_len_128 | ---NA--- | | |
| TRINITY_DN17442_c0_g1_i1_len_628_201238.p1_type_5prime_partial_len_128 | XP_018015996.1 dolichol-phosphate mannosyltransferase subunit 3-like | | |
| TRINITY_DN22526_c0_g1_i2_len_1594_232566.p2_type_complete_len_128 | XP_023711328.1hydroxylysine kinase isoform X3 | F:GO:0016301; P:GO:0016310 | |

| | | | |
|---|---|---|---|
| TRINITY_DN8313_c0_g1_i1_len_483_39095.p1_type_5prime_partial_len_128 | ---NA--- | | |
| TRINITY_DN21168_c0_g1_i1_len_3566_57919.p3_type_3prime_partial_len_129 | ---NA--- | | |
| TRINITY_DN4419_c0_g1_i1_len_386_170780.p1_type_internal_len_129 | ---NA--- | | |
| TRINITY_DN41102_c0_g1_i1_len_387_208655.p1_type_internal_len_129 | XP_018013126.1 multidrug resistance-associated protein 7-like | | |
| TRINITY_DN13251_c0_g1_i2_len_792_97100.p2_type_complete_len_128 | ---NA--- | | |
| TRINITY_DN24948_c0_g2_i2_len_2022_59298.p2_type_5prime_partial_len_128 | ---NA--- | | |
| TRINITY_DN11156_c0_g1_i1_len_807_162354.p1_type_5prime_partial_len_128 | ---NA--- | | |
| TRINITY_DN24258_c0_g1_i1_len_384_307663.p1_type_internal_len_129 | ---NA--- | | |
| TRINITY_DN2213_c0_g1_i1_len_386_208684.p1_type_internal_len_129 | XP_018018286.1 glycerol-3-phosphate acyltransferase 1, mitochondrial-like | | |
| TRINITY_DN41989_c0_g1_i1_len_386_208688.p1_type_internal_len_129 | AFJ11398.1thromboxane A synthase | F:GO:0005506; C:GO:0016021; F:GO:0016705; F:GO:0016853; F:GO:0020037; P:GO:0055114 | |
| TRINITY_DN9965_c0_g1_i1_len_386_208693.p1_type_internal_len_129 | ---NA--- | | |
| TRINITY_DN32634_c0_g1_i1_len_386_208695.p1_type_internal_len_129 | ---NA--- | | |
| TRINITY_DN9400_c0_g1_i1_len_998_95612.p1_type_complete_len_128 | XP_018023991.1 longitudinals lacking protein-like | C:GO:0005634; P:GO:0006357 | |
| TRINITY_DN41046_c0_g1_i1_len_387_208669.p1_type_internal_len_129 | XP_021936980.1unconventional myosin-XV-like isoform X3 | F:GO:0005488 | |
| TRINITY_DN24192_c0_g1_i3_len_2011_231089.p1_type_complete_len_128 | AYN63914.1amine oxidase | | |
| TRINITY_DN389_c0_g1_i1_len_386_208673.p1_type_internal_len_129 | XP_018016528.1 tankyrase-2-like | F:GO:0003950; P:GO:0016055; P:GO:0032212; P:GO:0051225 | EC:2.4.2.30 |

| | | | |
|---|---|---|---|
| TRINITY_DN19272_c0_g1_i2_len_550_100284.p1_type_5prime_partial_len_128 | ---NA--- | | |
| TRINITY_DN8485_c0_g1_i1_len_387_104895.p1_type_3prime_partial_len_129 | ACU00106.1troponin-C isoform 6 | F:GO:0005509 | |
| TRINITY_DN18136_c0_g1_i2_len_1036_62898.p2_type_5prime_partial_len_128 | ---NA--- | | |
| TRINITY_DN22387_c0_g1_i1_len_385_104956.p1_type_internal_len_129 | XP_024214437.1homeodomain-interacting protein kinase 2-like | | |
| TRINITY_DN26006_c0_g1_i1_len_386_104947.p2_type_internal_len_129 | ---NA--- | | |
| TRINITY_DN26634_c0_g1_i1_len_386_104941.p1_type_internal_len_129 | ---NA--- | | |
| TRINITY_DN30428_c0_g1_i1_len_388_170717.p1_type_internal_len_129 | ---NA--- | | |
| TRINITY_DN1249_c0_g1_i2_len_388_170713.p1_type_internal_len_129 | ---NA--- | | |
| TRINITY_DN16878_c0_g1_i1_len_405_207791.p1_type_5prime_partial_len_128 | ---NA--- | | |
| TRINITY_DN13952_c0_g1_i1_len_615_36746.p1_type_5prime_partial_len_128 | XP_018019993.1 protein TEX261-like | C:GO:0016021 | |
| TRINITY_DN15740_c0_g1_i1_len_589_241003.p1_type_complete_len_128 | ---NA--- | | |
| TRINITY_DN12238_c0_g1_i1_len_387_104888.p1_type_internal_len_129 | ---NA--- | | |
| TRINITY_DN24225_c0_g1_i1_len_386_170805.p1_type_internal_len_129 | ---NA--- | | |
| TRINITY_DN3281_c0_g1_i1_len_413_207470.p1_type_complete_len_128 | ---NA--- | | |
| TRINITY_DN9471_c0_g1_i2_len_388_208606.p1_type_internal_len_129 | ---NA--- | | |
| TRINITY_DN31989_c0_g1_i1_len_386_246991.p1_type_internal_len_129 | XP_013774871.2uncharacterized protein LOC106459765 | | |

| | | | |
|---|---|---|---|
| TRINITY_DN1072_c0_g1_i1_len_385_208737.p1_type_internal_len_129 | ---NA--- | | |
| TRINITY_DN28976_c0_g1_i1_len_385_246995.p1_type_internal_len_129 | ---NA--- | | |
| TRINITY_DN467_c0_g1_i1_len_385_246997.p1_type_internal_len_129 | ---NA--- | | |
| TRINITY_DN10441_c0_g1_i1_len_629_201228.p1_type_5prime_partial_len_128 | ---NA--- | | |
| TRINITY_DN30539_c0_g1_i7_len_3156_188308.p2_type_complete_len_128 | ---NA--- | | |
| TRINITY_DN18637_c0_g1_i1_len_535_68291.p1_type_5prime_partial_len_128 | ---NA--- | | |
| TRINITY_DN9119_c0_g1_i1_len_385_247000.p1_type_internal_len_129 | XP_018022821.1 ubiquitin carboxyl-terminal hydrolase 25-like | | |
| TRINITY_DN26437_c0_g1_i1_len_385_247003.p1_type_internal_len_129 | ---NA--- | | |
| TRINITY_DN35151_c0_g1_i1_len_385_208748.p1_type_internal_len_129 | ---NA--- | | |
| TRINITY_DN2118_c0_g1_i1_len_385_247012.p1_type_internal_len_129 | XP_018011424.1 DENN domain-containing protein 5A-like | | |
| TRINITY_DN31831_c0_g1_i1_len_386_246953.p1_type_internal_len_129 | ---NA--- | | |
| TRINITY_DN2918_c0_g1_i1_len_386_246956.p1_type_internal_len_129 | AVN99027.1eye-specific diacylglycerol kinase isoform X3 | F:GO:0003951; F:GO:0004143; F:GO:0005524; P:GO:0007205; P:GO:0016310; P:GO:0035556 | EC:2.7.1.23; EC:2.7.1.107 |
| TRINITY_DN10205_c0_g1_i1_len_386_246958.p1_type_internal_len_129 | XP_019872639.1 neuroligin-4, Y-linked | | |
| TRINITY_DN38914_c0_g1_i1_len_386_208731.p1_type_internal_len_129 | ---NA--- | | |
| TRINITY_DN1050_c0_g1_i1_len_389_170657.p1_type_5prime_partial_len_128 | ---NA--- | | |
| TRINITY_DN24393_c0_g1_i1_len_520_38301.p2_type_complete_len_128 | ---NA--- | | |

<table>
<tr><th></th><th></th><th></th><th></th></tr>
<tr><td>TRINITY_DN1789_c0_g1_i1_len_386_246960.p1_type_internal_len_129</td><td>---NA---</td><td></td><td></td></tr>
<tr><td>TRINITY_DN25853_c0_g1_i1_len_1531_232879.p2_type_5prime_partial_len_128</td><td>---NA---</td><td></td><td></td></tr>
<tr><td>TRINITY_DN17486_c0_g1_i1_len_3511_187921.p2_type_complete_len_128</td><td>---NA---</td><td></td><td></td></tr>
<tr><td>TRINITY_DN3746_c0_g1_i1_len_386_246968.p1_type_internal_len_129</td><td>KDR09067.1Ubiquitin-conjugating enzyme E2 O</td><td></td><td></td></tr>
<tr><td>TRINITY_DN21732_c0_g1_i1_len_1270_234241.p2_type_complete_len_128</td><td>---NA---</td><td></td><td></td></tr>
<tr><td>TRINITY_DN18548_c0_g1_i1_len_491_101517.p1_type_5prime_partial_len_128</td><td>---NA---</td><td></td><td></td></tr>
<tr><td>TRINITY_DN35063_c0_g1_i1_len_385_208762.p1_type_internal_len_129</td><td>XP_017768559.1 myotubularin-related protein 3 isoform X3</td><td>F:GO:0004725; P:GO:0016311; P:GO:0035335</td><td></td></tr>
<tr><td>TRINITY_DN13300_c0_g1_i1_len_385_208774.p1_type_internal_len_129</td><td>---NA---</td><td></td><td></td></tr>
<tr><td>TRINITY_DN36858_c0_g1_i1_len_385_208777.p1_type_internal_len_129</td><td>---NA---</td><td></td><td></td></tr>
<tr><td>TRINITY_DN1886_c0_g1_i1_len_385_208792.p1_type_internal_len_129</td><td>---NA---</td><td></td><td></td></tr>
<tr><td>2217|_|c21421_f3p5_2656_isoform_c21421_full_length_coverage_3_non_full_length_coverage_5_isoform_length_2656_13802</td><td>XP_011631276.1glycine--tRNA ligase</td><td></td><td></td></tr>
<tr><td>TRINITY_DN21941_c0_g1_i7_len_2847_91243.p1_type_5prime_partial_len_885</td><td>ASC55678.1putative trypsin serine protease</td><td>F:GO:0004252; P:GO:0006508; F:GO:0008233</td><td></td></tr>
<tr><td>2221|_|c5394_f2p0_2655_isoform_c5394_full_length_coverage_2_non_full_length_coverage_0_isoform_length_2655_12470</td><td>---NA---</td><td></td><td></td></tr>
<tr><td>2222|_|c12416_f2p0_2655_isoform_c12416_full_length_coverage_2_non_full_length_coverage_0_isoform_length_2655_17510</td><td>PFX15302.1WD repeat-containing protein 34</td><td></td><td></td></tr>
<tr><td>TRINITY_DN28199_c0_g1_i1_len_2906_188685.p1_type_complete_len_885</td><td>XP_018012467.1 protein artemis-like</td><td></td><td></td></tr>
</table>

| | | | |
|---|---|---|---|
| 2225\|_\|c17858_f5p1_2655_isoform_c17858_full_length_coverage_5_non_full_length_coverage_1_isoform_length_2655_13445 | ---NA--- | | |
| 2226\|_\|c5464_f2p1_2654_isoform_c5464_full_length_coverage_2_non_full_length_coverage_1_isoform_length_2654_12475 | XP_018026404.1 zinc finger protein 501-like | F:GO:0003676 | |
| 2227\|_\|c2308_f3p0_2654_isoform_c2308_full_length_coverage_3_non_full_length_coverage_0_isoform_length_2654_16313 | XP_023242730.1protein farnesyltransferase subunit beta-like | F:GO:0004659; F:GO:0046872 | |
| 2228\|_\|c12237_f2p0_2654_isoform_c12237_full_length_coverage_2_non_full_length_coverage_0_isoform_length_2654_22115 | XP_018006442.1PREDICTED: uncharacterized protein F54F2.9-like | F:GO:0003677; C:GO:0016020; C:GO:0016021 | |
| 2229\|_\|c10989_f2p7_2654_isoform_c10989_full_length_coverage_2_non_full_length_coverage_7_isoform_length_2654_17411 | XP_018008433.1 mitochondrial Rho GTPase 1-like | | |
| TRINITY_DN11359_c0_g1_i1_len_385_247017.p1_type_internal_len_129 | XP_018010632.1 proteoglycan 4-like | | |
| TRINITY_DN7289_c0_g1_i1_len_385_247019.p1_type_internal_len_129 | ---NA--- | | |
| TRINITY_DN27043_c0_g1_i1_len_384_247027.p1_type_internal_len_129 | ---NA--- | | |
| TRINITY_DN12861_c0_g1_i1_len_384_247040.p1_type_internal_len_129 | ---NA--- | | |
| TRINITY_DN35341_c0_g1_i1_len_384_247047.p1_type_internal_len_129 | ---NA--- | | |
| TRINITY_DN31138_c0_g1_i1_len_384_247058.p1_type_internal_len_129 | ---NA--- | | |
| TRINITY_DN32302_c0_g1_i1_len_384_247079.p1_type_internal_len_129 | ---NA--- | | |
| TRINITY_DN20569_c0_g1_i1_len_2398_155180.p1_type_5prime_partial_len_128 | ---NA--- | | |
| TRINITY_DN35965_c0_g1_i1_len_384_208813.p1_type_internal_len_129 | ---NA--- | | |

| | | | |
|---|---|---|---|
| TRINITY_DN39776_c0_g1_i1_len_442_206265.p1_type_5prime_partial_len_128 | ---NA--- | | |
| TRINITY_DN33996_c0_g1_i1_len_388_246868.p1_type_internal_len_129 | ---NA--- | | |
| TRINITY_DN41505_c0_g1_i1_len_386_208704.p1_type_internal_len_129 | XP_018012344.1 dystonin-like isoform X2 | | |
| TRINITY_DN14549_c0_g1_i1_len_386_208707.p1_type_internal_len_129 | OMO51981.1reverse transcriptase | F:GO:0003676; F:GO:0003964; P:GO:0006278; F:GO:0008270; P:GO:0015074 | |
| TRINITY_DN2196_c0_g1_i1_len_388_246886.p1_type_internal_len_129 | ---NA--- | | |
| TRINITY_DN103_c0_g1_i1_len_386_208697.p1_type_internal_len_129 | ---NA--- | | |
| TRINITY_DN11972_c0_g1_i1_len_516_68712.p1_type_5prime_partial_len_128 | ---NA--- | | |
| TRINITY_DN28728_c0_g1_i1_len_3651_187807.p2_type_complete_len_128 | XP_018017393.1 sodium bicarbonate transporter-like protein 11 | | |
| TRINITY_DN27739_c0_g1_i2_len_1749_191860.p2_type_5prime_partial_len_128 | ---NA--- | | |
| TRINITY_DN17013_c0_g1_i1_len_1005_95571.p2_type_complete_len_128 | ---NA--- | | |
| TRINITY_DN38916_c0_g1_i1_len_386_208722.p1_type_internal_len_129 | ---NA--- | | |
| TRINITY_DN18448_c0_g1_i2_len_386_208724.p1_type_internal_len_129 | ---NA--- | | |
| TRINITY_DN31495_c0_g1_i1_len_387_246923.p1_type_internal_len_129 | ---NA--- | | |
| TRINITY_DN30594_c0_g1_i1_len_387_246931.p1_type_internal_len_129 | ---NA--- | | |
| TRINITY_DN14280_c0_g1_i1_len_610_240627.p1_type_3prime_partial_len_129 | ---NA--- | | |
| TRINITY_DN32849_c0_g1_i1_len_388_246893.p1_type_internal_len_129 | ---NA--- | | |

| | | | |
|---|---|---|---|
| TRINITY_DN12009_c0_g1_i1_len_386_208708.p1_type_internal_len_129 | XP_023217315.1suppressor of lurcher protein 1-like isoform X4 | | |
| TRINITY_DN34585_c0_g1_i1_len_386_208714.p1_type_internal_len_129 | AFQ62792.1inositol-requiring enzyme-1 | F:GO:0004540; F:GO:0004672; F:GO:0005524; P:GO:0006397; P:GO:0006468; C:GO:0016021; P:GO:0090501 | |
| TRINITY_DN25779_c0_g3_i1_len_387_246908.p1_type_internal_len_129 | ALX81379.170 kDa heat shock protein isoform 1 | F:GO:0005524 | |
| TRINITY_DN2980_c0_g1_i1_len_386_208718.p1_type_internal_len_129 | ---NA--- | | |
| TRINITY_DN21653_c0_g1_i1_len_5073_57715.p4_type_complete_len_128 | ---NA--- | | |
| TRINITY_DN37134_c0_g1_i1_len_1162_195222.p2_type_5prime_partial_len_128 | AKS04548.1cysteine sulfinic acid decarboxylase | F:GO:0016831; P:GO:0019752; F:GO:0030170 | |
| TRINITY_DN40880_c0_g1_i1_len_452_205883.p1_type_5prime_partial_len_128 | XP_025096332.1alpha-1,6-mannosyl-glycoprotein 2-beta-N-acetylglucosaminyltransferase-like | C:GO:0005795; F:GO:0008455; P:GO:0009312; C:GO:0016021 | |
| TRINITY_DN30268_c0_g1_i2_len_2223_190173.p2_type_complete_len_128 | KDR24401.1Nucleoside diphosphate kinase 7 | | |
| TRINITY_DN35535_c0_g1_i1_len_887_237073.p1_type_5prime_partial_len_128 | EEC17106.1serine/threonine protein kinase, putative | F:GO:0004674; F:GO:0005524; C:GO:0005737; P:GO:0016477; P:GO:0031098; P:GO:0032147; P:GO:0043408; F:GO:0048365 | EC:2.7.11 |
| TRINITY_DN6799_c0_g1_i1_len_385_279529.p1_type_internal_len_129 | ---NA--- | | |
| TRINITY_DN17914_c0_g1_i2_len_2423_29850.p2_type_complete_len_128 | XP_026274343.1dolichyl-diphosphooligosaccharide--protein glycosyltransferase subunit STT3B | F:GO:0004576; P:GO:0006486; C:GO:0016021 | |
| TRINITY_DN23155_c0_g1_i1_len_384_12328.p1_type_internal_len_129 | ---NA--- | | |
| TRINITY_DN30965_c0_g3_i1_len_2501_189446.p1_type_complete_len_128 | XP_022289764.1transmembrane protein 230-like | C:GO:0016021 | |

| | | | |
|---|---|---|---|
| TRINITY_DN12568_c0_g1_i1_len_680_163819.p1_type_5prime_partial_len_128 | ---NA--- | | |
| TRINITY_DN15463_c0_g1_i2_len_1628_192422.p3_type_complete_len_128 | ---NA--- | | |
| TRINITY_DN4985_c0_g1_i1_len_513_242684.p1_type_3prime_partial_len_129 | XP_023336417.1phenazine biosynthesis-like domain-containing protein | F:GO:0003824; P:GO:0009058 | |
| TRINITY_DN26319_c0_g1_i1_len_384_12319.p2_type_internal_len_129 | AOR07228.1potassium voltage-gated channel subfamily H member 2 | F:GO:0005249; C:GO:0016021; P:GO:0071805 | |
| TRINITY_DN29187_c0_g1_i1_len_2098_190556.p1_type_5prime_partial_len_128 | ---NA--- | | |
| TRINITY_DN23069_c0_g1_i1_len_755_65054.p2_type_5prime_partial_len_128 | ---NA--- | | |
| TRINITY_DN17519_c0_g1_i1_len_386_12241.p2_type_internal_len_129 | ---NA--- | | |
| TRINITY_DN10241_c0_g1_i1_len_386_12242.p1_type_internal_len_129 | ---NA--- | | |
| TRINITY_DN22468_c0_g2_i2_len_386_12247.p1_type_internal_len_129 | ---NA--- | | |
| TRINITY_DN8011_c0_g1_i2_len_385_12296.p1_type_internal_len_129 | ---NA--- | | |
| TRINITY_DN28692_c0_g1_i1_len_388_279399.p1_type_internal_len_129 | ---NA--- | | |
| TRINITY_DN22369_c1_g2_i2_len_386_12248.p1_type_internal_len_129 | ---NA--- | | |
| TRINITY_DN23875_c0_g1_i1_len_1413_193576.p1_type_5prime_partial_len_128 | ---NA--- | | |
| TRINITY_DN25294_c0_g1_i1_len_388_279420.p1_type_internal_len_129 | ---NA--- | | |
| TRINITY_DN5923_c0_g1_i1_len_387_279456.p1_type_internal_len_129 | AYF59252.1beta-arrestin 1 | P:GO:0007165 | |
| TRINITY_DN14304_c0_g1_i1_len_385_12287.p1_type_internal_len_129 | ---NA--- | | |

| | | | |
|---|---|---|---|
| TRINITY_DN31040_c0_g1_i1_len_385_12291.p1_type_internal_len_129 | ---NA--- | | |
| TRINITY_DN12680_c0_g1_i1_len_385_12274.p1_type_internal_len_129 | ---NA--- | | |
| TRINITY_DN31486_c0_g1_i1_len_388_12183.p1_type_internal_len_129 | ---NA--- | | |
| TRINITY_DN23513_c0_g1_i2_len_1680_232212.p1_type_5prime_partial_len_128 | ---NA--- | | |
| TRINITY_DN15156_c0_g1_i1_len_422_207061.p1_type_5prime_partial_len_128 | ---NA--- | | |
| TRINITY_DN24458_c0_g1_i1_len_1370_233676.p2_type_complete_len_128 | XP_013787357.1very-long-chain (3R)-3-hydroxyacyl-CoA dehydratase-like | C:GO:0005789; P:GO:0006633; C:GO:0016021; F:GO:0102158; F:GO:0102343; F:GO:0102344; F:GO:0102345 | EC:4.2.1.134 |
| TRINITY_DN19201_c0_g1_i1_len_694_98158.p1_type_5prime_partial_len_128 | ---NA--- | | |
| TRINITY_DN12635_c0_g1_i1_len_386_279499.p1_type_internal_len_129 | ---NA--- | | |
| TRINITY_DN21120_c0_g1_i1_len_1485_193180.p1_type_complete_len_128 | ---NA--- | | |
| TRINITY_DN4538_c0_g1_i1_len_386_279507.p1_type_internal_len_129 | ---NA--- | | |
| TRINITY_DN17273_c0_g2_i1_len_452_168357.p1_type_5prime_partial_len_128 | XP_024219427.1zinc finger protein 711-like | | |
| TRINITY_DN38037_c0_g1_i1_len_419_207192.p1_type_5prime_partial_len_128 | XP_018010074.1 polypeptide N-acetylgalactosaminyltransferase 1-like | | |
| TRINITY_DN25807_c0_g1_i1_len_1389_193710.p2_type_5prime_partial_len_128 | ---NA--- | | |
| TRINITY_DN17275_c0_g1_i2_len_797_237987.p1_type_5prime_partial_len_128 | AFE88209.1protaglandin reductase 1 | P:GO:0055114 | |
| TRINITY_DN2951_c0_g1_i1_len_412_169767.p1_type_5prime_partial_len_128 | ---NA--- | | |

| | | | |
|---|---|---|---|
| TRINITY_DN40176_c0_g1_i1_len_390_208526.p1_type_5prime_partial_len_128 | ---NA--- | | |
| TRINITY_DN8354_c0_g1_i2_len_386_307552.p1_type_internal_len_129 | XP_025114560.1RING finger and SPRY domain-containing protein 1-like | F:GO:0046872 | |
| TRINITY_DN23544_c0_g1_i1_len_410_11362.p1_type_3prime_partial_len_129 | XP_017772483.1 disks large 1 tumor suppressor protein-like isoform X3 | | |
| TRINITY_DN23434_c0_g1_i1_len_385_104977.p1_type_internal_len_129 | ABB85237.1chitinase 1 | F:GO:0004568; C:GO:0005576; P:GO:0005975; P:GO:0006030; F:GO:0008061 | EC:3.2.1.14 |
| TRINITY_DN26017_c0_g1_i1_len_2604_189219.p2_type_complete_len_128 | ---NA--- | | |
| TRINITY_DN36093_c0_g1_i1_len_636_201088.p2_type_5prime_partial_len_128 | ---NA--- | | |
| TRINITY_DN22084_c0_g1_i1_len_657_66235.p1_type_3prime_partial_len_129 | ---NA--- | | |
| TRINITY_DN15348_c0_g1_i1_len_600_201781.p1_type_5prime_partial_len_128 | ---NA--- | | |
| TRINITY_DN12957_c0_g1_i2_len_918_63644.p2_type_5prime_partial_len_128 | ---NA--- | | |
| TRINITY_DN33550_c0_g1_i1_len_504_166977.p1_type_5prime_partial_len_128 | ANA91277.1crustinI-5 | C:GO:0005576; P:GO:0010466; F:GO:0030414 | |
| TRINITY_DN33792_c0_g1_i1_len_450_205958.p1_type_5prime_partial_len_128 | XP_018011605.1 UDP-N-acetylglucosamine transporter-like isoform X1 | | |
| TRINITY_DN14375_c0_g1_i3_len_1285_158845.p2_type_5prime_partial_len_128 | ---NA--- | | |
| TRINITY_DN17537_c0_g1_i1_len_498_204335.p1_type_5prime_partial_len_128 | ---NA--- | | |
| TRINITY_DN16928_c0_g1_i2_len_386_72957.p1_type_internal_len_129 | ---NA--- | | |
| TRINITY_DN20840_c0_g1_i1_len_539_203177.p1_type_5prime_partial_len_128 | ---NA--- | | |
| TRINITY_DN9699_c0_g1_i1_len_386_72946.p2_type_internal_len_129 | ---NA--- | | |

| | | | |
|---|---|---|---|
| TRINITY_DN26614_c0_g1_i1_len_466_133214.p1_type_5prime_partial_len_128 | XP_015271262.1 solute carrier family 12 member 9-like | F:GO:0005215; P:GO:0006811; C:GO:0016020 | |
| TRINITY_DN27782_c0_g1_i1_len_477_132951.p1_type_5prime_partial_len_128 | XP_018008078.1PREDICTED: uncharacterized protein KIAA0513-like | | |
| TRINITY_DN11027_c0_g1_i1_len_386_42000.p1_type_internal_len_129 | ---NA--- | | |
| TRINITY_DN28365_c0_g1_i1_len_384_73042.p2_type_internal_len_129 | XP_018018658.1PREDICTED: uncharacterized protein LOC108675177 | | |
| TRINITY_DN18429_c0_g1_i1_len_420_134680.p1_type_5prime_partial_len_128 | KRX83908.1hypothetical protein T4E_7988, partial | | |
| TRINITY_DN27749_c0_g1_i2_len_480_204897.p1_type_5prime_partial_len_128 | ---NA--- | | |
| TRINITY_DN28258_c0_g1_i1_len_385_72988.p1_type_internal_len_129 | ---NA--- | | |
| TRINITY_DN22962_c0_g1_i7_len_385_72986.p1_type_internal_len_129 | ---NA--- | | |
| TRINITY_DN25234_c0_g1_i1_len_385_72985.p1_type_internal_len_129 | ---NA--- | | |
| TRINITY_DN21440_c0_g1_i1_len_1807_123479.p2_type_3prime_partial_len_129 | ---NA--- | | |
| TRINITY_DN40663_c0_g1_i1_len_517_203730.p1_type_3prime_partial_len_129 | ---NA--- | | |
| TRINITY_DN15370_c0_g1_i4_len_1069_33138.p1_type_5prime_partial_len_128 | ---NA--- | | |
| TRINITY_DN3212_c0_g1_i2_len_384_42071.p1_type_internal_len_129 | ---NA--- | | |
| TRINITY_DN13485_c0_g1_i6_len_386_42014.p1_type_internal_len_129 | ---NA--- | | |
| TRINITY_DN17123_c0_g1_i1_len_586_7201.p2_type_5prime_partial_len_128 | XP_021940286.1plastin-3 isoform X3 | F:GO:0005509; F:GO:0051015; P:GO:0051017 | |
| 2230\|_\|c7048_f3p8_2653_isoform_c7048_full_length_coverage_3_non_full_length_coverage_8_isoform_length_2653_21653 | XP_023350276.1aurora kinase A | | |

<table>
<tr><td>2231|_|c4011_f2p6_2653_isoform_c4011_full_length_coverage_2_non_full_length_coverage_6_isoform_length_2653_12341</td><td>---NA---</td><td></td><td></td></tr>
<tr><td>2232|_|c2711_f2p0_2653_isoform_c2711_full_length_coverage_2_non_full_length_coverage_0_isoform_length_2653_21171</td><td>XP_018019486.1 myosin-IB-like</td><td>F:GO:0000166</td><td></td></tr>
<tr><td>TRINITY_DN29707_c0_g1_i1_len_4340_187395.p1_type_complete_len_884</td><td>---NA---</td><td></td><td></td></tr>
<tr><td>2236|_|c18009_f2p1_2651_isoform_c18009_full_length_coverage_2_non_full_length_coverage_1_isoform_length_2651_22627</td><td>---NA---</td><td></td><td></td></tr>
<tr><td>2237|_|c28774_f2p0_2650_isoform_c28774_full_length_coverage_2_non_full_length_coverage_0_isoform_length_2650_23097</td><td>XP_008756456.2 zinc finger protein 665-like</td><td></td><td></td></tr>
<tr><td>2238|_|c17985_f2p1_2650_isoform_c17985_full_length_coverage_2_non_full_length_coverage_1_isoform_length_2650_17876</td><td>XP_011432547.1 DNA-binding protein SMUBP-2 isoform X2</td><td>F:GO:0003677; F:GO:0004714; F:GO:0005524; C:GO:0016021; P:GO:0018108</td><td>EC:2.7.10; EC:2.7.10.1; EC:2.7.11</td></tr>
<tr><td>TRINITY_DN29966_c0_g1_i1_len_2977_188577.p1_type_5prime_partial_len_883</td><td>XP_015603966.1U3 small nucleolar RNA-associated protein 14 homolog A</td><td></td><td></td></tr>
<tr><td>TRINITY_DN26784_c0_g1_i1_len_2964_188600.p1_type_complete_len_883</td><td>XP_023227764.1zinc finger CCCH domain-containing protein 14-like</td><td>F:GO:0008143; P:GO:0043488; F:GO:0046872; P:GO:1900364</td><td></td></tr>
<tr><td>TRINITY_DN26938_c0_g1_i1_len_2831_188811.p1_type_5prime_partial_len_883</td><td>XP_018023410.1PREDICTED: uncharacterized protein LOC108679320 isoform X4</td><td></td><td></td></tr>
<tr><td>TRINITY_DN22431_c0_g1_i1_len_385_42064.p1_type_internal_len_129</td><td>---NA---</td><td></td><td></td></tr>
<tr><td>TRINITY_DN31816_c0_g1_i1_len_568_202450.p1_type_5prime_partial_len_128</td><td>---NA---</td><td></td><td></td></tr>
<tr><td>TRINITY_DN18422_c0_g1_i1_len_1025_196350.p2_type_3prime_partial_len_129</td><td>---NA---</td><td></td><td></td></tr>
<tr><td>TRINITY_DN14800_c0_g1_i1_len_475_205039.p1_type_5prime_partial_len_128</td><td>---NA---</td><td></td><td></td></tr>
</table>

| | | | |
|---|---|---|---|
| TRINITY_DN12391_c0_g1_i1_len_402_72271.p1_type_3prime_partial_len_129 | XP_018026786.1 protein FAM49B-like | | |
| TRINITY_DN12908_c0_g1_i1_len_489_204588.p1_type_3prime_partial_len_129 | XP_023716109.1sorting nexin-29 isoform X1 | | |
| TRINITY_DN8868_c0_g1_i1_len_455_277173.p1_type_5prime_partial_len_128 | ---NA--- | | |
| TRINITY_DN1790_c0_g1_i1_len_666_129406.p1_type_5prime_partial_len_128 | ---NA--- | | |
| TRINITY_DN16655_c0_g1_i1_len_743_199385.p2_type_5prime_partial_len_128 | ---NA--- | | |
| TRINITY_DN11627_c0_g2_i1_len_387_41959.p2_type_internal_len_129 | ---NA--- | | |
| TRINITY_DN20388_c0_g4_i1_len_387_41963.p1_type_internal_len_129 | ---NA--- | | |
| TRINITY_DN7108_c0_g1_i1_len_385_136036.p1_type_internal_len_129 | ---NA--- | | |
| TRINITY_DN30336_c0_g1_i1_len_385_136029.p1_type_internal_len_129 | ---NA--- | | |
| TRINITY_DN10105_c0_g1_i1_len_385_136025.p1_type_internal_len_129 | ---NA--- | | |
| TRINITY_DN10145_c0_g1_i1_len_970_3781.p1_type_5prime_partial_len_128 | ---NA--- | | |
| TRINITY_DN20417_c0_g1_i1_len_386_135965.p1_type_5prime_partial_len_128 | ---NA--- | | |
| TRINITY_DN13734_c0_g1_i1_len_387_135942.p1_type_internal_len_129 | ---NA--- | | |
| TRINITY_DN15754_c0_g1_i1_len_841_34475.p1_type_5prime_partial_len_128 | ---NA--- | | |
| TRINITY_DN29004_c0_g1_i1_len_386_135980.p1_type_internal_len_129 | PIK39424.1hypothetical protein BSL78_23736 | F:GO:0003676; P:GO:0015074 | |
| TRINITY_DN30445_c0_g1_i1_len_384_136084.p1_type_internal_len_129 | ---NA--- | | |

| | | | |
|---|---|---|---|
| TRINITY_DN24998_c0_g1_i1_len_384_136088.p1_type_internal_len_129 | XP_018968294.1 ezrin | F:GO:0003779; C:GO:0005856 | |
| TRINITY_DN22806_c0_g2_i1_len_510_203919.p1_type_5prime_partial_len_128 | ---NA--- | | |
| TRINITY_DN9186_c0_g1_i1_len_478_204964.p1_type_complete_len_128 | ---NA--- | | |
| TRINITY_DN7209_c0_g1_i1_len_406_41282.p1_type_5prime_partial_len_128 | XP_018024341.1 disks large 1 tumor suppressor protein-like | | |
| TRINITY_DN1754_c0_g1_i1_len_536_275254.p1_type_5prime_partial_len_128 | ---NA--- | | |
| TRINITY_DN5097_c0_g1_i1_len_387_135925.p1_type_internal_len_129 | ---NA--- | | |
| TRINITY_DN17362_c0_g1_i1_len_1422_31718.p3_type_5prime_partial_len_127 | ---NA--- | | |
| TRINITY_DN18845_c0_g1_i1_len_599_130392.p1_type_complete_len_127 | XP_022907319.1probable 28S ribosomal protein S16, mitochondrial | F:GO:0003735; C:GO:0005840; P:GO:0006412 | |
| TRINITY_DN14542_c0_g1_i1_len_391_208470.p1_type_5prime_partial_len_127 | EDO34495.1predicted protein | P:GO:0003333; F:GO:0015171; C:GO:0016020; C:GO:0016021 | |
| TRINITY_DN12656_c0_g1_i1_len_703_128992.p1_type_complete_len_127 | AYE56135.1thioredoxin domain-containing protein 14 | | |
| TRINITY_DN9028_c0_g1_i1_len_555_202766.p1_type_3prime_partial_len_128 | ---NA--- | | |
| TRINITY_DN16616_c0_g1_i1_len_1089_95058.p2_type_complete_len_127 | ---NA--- | | |
| TRINITY_DN11311_c0_g1_i1_len_452_102614.p1_type_5prime_partial_len_127 | ---NA--- | | |
| TRINITY_DN11167_c0_g1_i1_len_630_129890.p2_type_5prime_partial_len_127 | ---NA--- | | |
| TRINITY_DN26441_c0_g1_i1_len_1929_191156.p1_type_3prime_partial_len_128 | ---NA--- | | |
| TRINITY_DN16401_c0_g1_i3_len_571_274623.p1_type_5prime_partial_len_127 | ---NA--- | | |

| | | | |
|---|---|---|---|
| TRINITY_DN11708_c0_g1_i1_len_642_129719.p1_type_3prime_partial_len_128 | ---NA--- | | |
| TRINITY_DN20035_c0_g1_i1_len_3090_294847.p3_type_3prime_partial_len_128 | ---NA--- | | |
| TRINITY_DN11808_c0_g1_i1_len_453_205821.p1_type_5prime_partial_len_127 | XP_023248359.1ras association domain-containing protein 2-like | P:GO:0007165 | |
| TRINITY_DN17645_c0_g1_i2_len_1574_157501.p1_type_complete_len_127 | ---NA--- | | |
| TRINITY_DN32648_c0_g1_i1_len_394_170455.p1_type_5prime_partial_len_127 | ---NA--- | | |
| TRINITY_DN23716_c0_g1_i2_len_2395_230013.p3_type_complete_len_127 | ---NA--- | | |
| TRINITY_DN28593_c0_g1_i1_len_381_171006.p1_type_internal_len_128 | ---NA--- | | |
| TRINITY_DN31705_c0_g1_i1_len_383_279615.p1_type_internal_len_128 | ---NA--- | | |
| TRINITY_DN31181_c0_g1_i1_len_381_171028.p1_type_internal_len_128 | ---NA--- | | |
| TRINITY_DN11946_c0_g1_i1_len_383_170900.p1_type_internal_len_128 | XP_018019052.1 alpha-L-fucosidase-like | F:GO:0004560; C:GO:0005764; P:GO:0006004; P:GO:0016139 | EC:3.2.1.51 |
| TRINITY_DN12249_c0_g1_i1_len_385_279547.p1_type_internal_len_128 | ---NA--- | | |
| TRINITY_DN19896_c0_g1_i1_len_499_204285.p1_type_5prime_partial_len_127 | ---NA--- | | |
| TRINITY_DN31329_c0_g1_i1_len_384_279565.p1_type_internal_len_128 | ---NA--- | | |
| TRINITY_DN29995_c0_g1_i1_len_383_170920.p1_type_internal_len_128 | XP_022343500.1uncharacterized protein LOC111136715 | | |
| TRINITY_DN32778_c0_g1_i1_len_382_170977.p1_type_internal_len_128 | XP_023727048.1epidermal growth factor receptor-like | F:GO:0004714; F:GO:0005524; P:GO:0007169; C:GO:0016020; P:GO:0018108 | EC:2.7.10; EC:2.7.10.1; EC:2.7.11 |
| TRINITY_DN20928_c0_g2_i1_len_381_279677.p1_type_3prime_partial_len_128 | ---NA--- | | |

| | | | |
|---|---|---|---|
| TRINITY_DN10099_c0_g1_i1_len_760_199126.p2_type_5prime_partial_len_127 | XP_018022661.1 probable serine incorporator | | |
| TRINITY_DN29817_c0_g1_i1_len_381_279676.p1_type_internal_len_128 | ---NA--- | | |
| TRINITY_DN2701_c0_g1_i1_len_382_279662.p2_type_internal_len_128 | ---NA--- | | |
| TRINITY_DN26555_c0_g1_i1_len_384_170880.p1_type_internal_len_128 | XP_018011799.1 S phase cyclin A-associated protein in the endoplasmic reticulum-like | F:GO:0005488 | |
| TRINITY_DN839_c0_g1_i1_len_382_279657.p2_type_internal_len_128 | ---NA--- | | |
| TRINITY_DN33456_c0_g1_i1_len_384_170864.p1_type_internal_len_128 | AYJ71540.1cuticle protein-8 | F:GO:0042302 | |
| TRINITY_DN7623_c0_g1_i1_len_392_208420.p1_type_5prime_partial_len_127 | ---NA--- | | |
| TRINITY_DN23964_c0_g1_i2_len_2334_122611.p2_type_5prime_partial_len_127 | ---NA--- | | |
| TRINITY_DN10809_c0_g1_i1_len_381_279688.p1_type_internal_len_128 | ---NA--- | | |
| TRINITY_DN19422_c0_g1_i4_len_2815_229259.p3_type_complete_len_127 | ---NA--- | | |
| TRINITY_DN18293_c0_g1_i1_len_682_163786.p1_type_complete_len_127 | ---NA--- | | |
| TRINITY_DN33235_c0_g1_i1_len_564_202562.p1_type_5prime_partial_len_127 | XP_018009875.1 protein amalgam-like isoform X1 | | |
| TRINITY_DN21626_c0_g1_i2_len_3327_228621.p3_type_complete_len_127 | ---NA--- | | |
| TRINITY_DN19311_c0_g1_i1_len_670_163931.p1_type_5prime_partial_len_127 | ---NA--- | | |
| TRINITY_DN1263_c0_g1_i1_len_381_42190.p1_type_internal_len_128 | ---NA--- | | |
| TRINITY_DN30191_c0_g1_i2_len_3050_188451.p2_type_5prime_partial_len_127 | ---NA--- | | |

| | | | |
|---|---|---|---|
| TRINITY_DN31236_c0_g1_i1_len_597_240854.p1_type_5prime_partial_len_127 | XP_018514080.1 ribulose bisphosphate carboxylase/oxygenase activase, chloroplastic | F:GO:0005524 | |
| TRINITY_DN23329_c1_g1_i4_len_3142_228827.p2_type_5prime_partial_len_127 | ---NA--- | | |
| TRINITY_DN15796_c0_g1_i1_len_612_164791.p1_type_5prime_partial_len_127 | ---NA--- | | |
| TRINITY_DN12955_c0_g1_i2_len_383_105050.p1_type_internal_len_128 | XP_018014923.1 SCY1-like protein 2 | F:GO:0004672; F:GO:0005524; P:GO:0006468 | |
| TRINITY_DN32381_c0_g1_i1_len_382_105086.p1_type_internal_len_128 | ---NA--- | | |
| TRINITY_DN10396_c0_g1_i1_len_383_105059.p1_type_internal_len_128 | ---NA--- | | |
| TRINITY_DN4228_c0_g1_i1_len_385_104957.p1_type_internal_len_128 | ---NA--- | | |
| TRINITY_DN6447_c0_g1_i1_len_479_204927.p1_type_3prime_partial_len_128 | ---NA--- | | |
| TRINITY_DN14118_c0_g1_i1_len_383_136117.p1_type_internal_len_128 | ---NA--- | | |
| TRINITY_DN21121_c0_g1_i1_len_989_160759.p1_type_5prime_partial_len_127 | ---NA--- | | |
| TRINITY_DN30528_c0_g1_i1_len_383_136103.p1_type_internal_len_128 | ---NA--- | | |
| TRINITY_DN16839_c0_g1_i2_len_406_104156.p1_type_5prime_partial_len_127 | ---NA--- | | |
| TRINITY_DN13768_c0_g1_i1_len_719_238910.p1_type_5prime_partial_len_127 | XP_018016860.1 small ubiquitin-related modifier 2-like | C:GO:0005634; P:GO:0016925 | |
| TRINITY_DN3723_c0_g1_i1_len_383_136115.p1_type_internal_len_128 | ---NA--- | | |
| TRINITY_DN15284_c0_g1_i1_len_383_136116.p1_type_internal_len_128 | ---NA--- | | |
| TRINITY_DN6009_c0_g1_i1_len_381_136194.p1_type_internal_len_128 | ---NA--- | | |

| | | | |
|---|---|---|---|
| TRINITY_DN33652_c0_g1_i1_len_382_136172.p2_type_internal_len_128 | ---NA--- | | |
| TRINITY_DN15774_c0_g1_i2_len_382_136154.p1_type_internal_len_128 | XP_018017378.1 protocadherin Fat 1-like | | |
| TRINITY_DN7807_c0_g1_i1_len_444_206187.p1_type_5prime_partial_len_127 | ---NA--- | | |
| TRINITY_DN12885_c0_g1_i1_len_661_239766.p1_type_3prime_partial_len_128 | ---NA--- | | |
| TRINITY_DN14758_c0_g1_i1_len_747_35229.p2_type_5prime_partial_len_127 | ---NA--- | | |
| TRINITY_DN21002_c0_g1_i2_len_1093_62544.p2_type_complete_len_127 | ---NA--- | | |
| TRINITY_DN17907_c0_g2_i2_len_384_136073.p1_type_internal_len_128 | ---NA--- | | |
| TRINITY_DN24155_c0_g1_i1_len_1533_192929.p2_type_complete_len_127 | ---NA--- | | |
| TRINITY_DN9372_c0_g1_i1_len_384_136089.p1_type_internal_len_128 | ---NA--- | | |
| TRINITY_DN29463_c0_g1_i1_len_435_168937.p1_type_5prime_partial_len_127 | XP_022250342.1UDP-N-acetylglucosamine transporter-like isoform X2 | | |
| TRINITY_DN3390_c0_g1_i1_len_429_206756.p1_type_5prime_partial_len_127 | ---NA--- | | |
| TRINITY_DN15023_c0_g1_i1_len_494_132515.p1_type_5prime_partial_len_127 | ---NA--- | | |
| TRINITY_DN29602_c0_g1_i1_len_2278_190015.p2_type_5prime_partial_len_127 | XP_016914104.1 tubulin gamma-2 chain | C:GO:0000930; F:GO:0003924; F:GO:0005525; C:GO:0005874; P:GO:0007020; P:GO:0031122 | EC:3.6.1.15 |
| TRINITY_DN23126_c0_g1_i5_len_1784_59834.p2_type_5prime_partial_len_127 | EOB00197.1Mitochondrial chaperone BCS1 | F:GO:0000166 | |
| TRINITY_DN8156_c0_g1_i1_len_541_241980.p1_type_3prime_partial_len_128 | AYF59252.1beta-arrestin 1 | P:GO:0007165 | |
| TRINITY_DN19912_c0_g1_i1_len_2033_190787.p2_type_5prime_partial_len_127 | ---NA--- | | |

| | | | |
|---|---|---|---|
| 2243\|_\|c18153_f2p0_2649_isoform_c18153_full_length_coverage_2_non_full_length_coverage_0_isoform_length_2649_13614 | XP_013787947.1prolyl 3-hydroxylase OGFOD1-like isoform X2 | | |
| TRINITY_DN27318_c0_g1_i2_len_3089_188389.p1_type_complete_len_883 | XP_018025094.1 protein bicaudal C homolog 1-like | F:GO:0003676; F:GO:0003723 | |
| 2245\|_\|c2607_f2p9_2649_isoform_c2607_full_length_coverage_2_non_full_length_coverage_9_isoform_length_2649_12008 | XP_018015072.1 dipeptidyl peptidase 3-like isoform X1 | | |
| 2246\|_\|c43109_f4p3_2648_isoform_c43109_full_length_coverage_4_non_full_length_coverage_3_isoform_length_2648_15030 | ---NA--- | | |
| 2247\|_\|c12317_f3p3_2647_isoform_c12317_full_length_coverage_3_non_full_length_coverage_3_isoform_length_2647_22125 | XP_018014580.1 phosphatidylinositol 4-kinase type 2-beta-like isoform X2 | | |
| 2248\|_\|c18074_f2p0_2647_isoform_c18074_full_length_coverage_2_non_full_length_coverage_0_isoform_length_2647_13554 | XP_021001228.1zinc finger protein 271-like isoform X2 | | |
| TRINITY_DN25824_c0_g1_i1_len_3372_228596.p1_type_complete_len_882 | XP_018019364.1 microsomal triglyceride transfer protein large subunit-like isoform X3 | | |
| TRINITY_DN25652_c0_g1_i1_len_2696_189042.p1_type_5prime_partial_len_882 | XP_018023566.1PREDICTED: uncharacterized protein LOC108679461 | | |
| TRINITY_DN26041_c0_g1_i1_len_2773_188909.p1_type_complete_len_882 | XP_022250695.1next to BRCA1 gene 1 protein-like isoform X2 | | |
| 2253\|_\|c4377_f2p1_2645_isoform_c4377_full_length_coverage_2_non_full_length_coverage_1_isoform_length_2645_16850 | ---NA--- | | |
| TRINITY_DN3587_c0_g1_i1_len_588_7183.p1_type_complete_len_127 | ---NA--- | | |
| TRINITY_DN20752_c0_g1_i1_len_1063_235627.p1_type_5prime_partial_len_127 | ---NA--- | | |
| TRINITY_DN11757_c0_g1_i1_len_385_42052.p1_type_internal_len_128 | ---NA--- | | |

| | | | |
|---|---|---|---|
| TRINITY_DN26497_c0_g1_i2_len_2110_190521.p2_type_complete_len_127 | ---NA--- | | |
| TRINITY_DN18272_c0_g1_i1_len_712_35576.p3_type_complete_len_127 | ---NA--- | | |
| TRINITY_DN31512_c0_g1_i1_len_546_241858.p1_type_complete_len_127 | ---NA--- | | |
| TRINITY_DN19392_c0_g1_i1_len_425_103448.p1_type_3prime_partial_len_128 | ---NA--- | | |
| TRINITY_DN25698_c0_g1_i1_len_559_165753.p1_type_5prime_partial_len_127 | XP_003702561.1 protein-S-isoprenylcysteine O-methyltransferase | F:GO:0004671; C:GO:0005789; P:GO:0006481; C:GO:0016021 | EC:2.1.1.100 |
| TRINITY_DN13013_c0_g2_i1_len_536_166259.p1_type_5prime_partial_len_127 | ---NA--- | | |
| TRINITY_DN20852_c0_g1_i1_len_992_63170.p2_type_complete_len_127 | XP_002738882.1 out at first protein-like isoform X1 | | |
| TRINITY_DN14027_c0_g1_i1_len_436_134083.p1_type_5prime_partial_len_127 | ---NA--- | | |
| TRINITY_DN14960_c0_g1_i1_len_382_105117.p3_type_internal_len_128 | ---NA--- | | |
| TRINITY_DN24005_c0_g1_i1_len_382_105111.p1_type_internal_len_128 | AAD41899.1fushi tarazu-factor 1 | F:GO:0003700; F:GO:0003707; C:GO:0005634; P:GO:0006355; F:GO:0008270; P:GO:0043401; F:GO:0043565 | |
| TRINITY_DN16198_c0_g1_i1_len_382_105102.p1_type_internal_len_128 | ---NA--- | | |
| TRINITY_DN19112_c0_g1_i1_len_411_134966.p1_type_5prime_partial_len_127 | XP_019435462.1 histone H2A | C:GO:0000786; F:GO:0003677; C:GO:0005634; F:GO:0046982 | |
| TRINITY_DN18415_c0_g1_i1_len_395_208262.p1_type_5prime_partial_len_127 | ---NA--- | | |
| TRINITY_DN17780_c0_g1_i3_len_2565_229658.p3_type_complete_len_127 | ---NA--- | | |
| TRINITY_DN12698_c0_g1_i1_len_587_37154.p1_type_5prime_partial_len_127 | ---NA--- | | |

| | | | |
|---|---|---|---|
| TRINITY_DN25691_c0_g1_i4_len_2291_189974.p3_type_complete_len_127 | ---NA--- | | |
| TRINITY_DN18490_c1_g1_i2_len_909_299354.p2_type_5prime_partial_len_127 | ---NA--- | | |
| TRINITY_DN4542_c0_g1_i1_len_463_133313.p1_type_5prime_partial_len_127 | ---NA--- | | |
| TRINITY_DN37231_c0_g1_i1_len_382_208943.p1_type_internal_len_128 | XP_016927241.1 UPF0505 protein CG8202 | | |
| TRINITY_DN19364_c0_g1_i4_len_383_12365.p1_type_internal_len_128 | ---NA--- | | |
| TRINITY_DN2149_c0_g1_i1_len_382_208896.p1_type_internal_len_128 | ---NA--- | | |
| TRINITY_DN6194_c0_g1_i1_len_419_71610.p1_type_5prime_partial_len_127 | XP_018023119.1 zinc finger and BTB domain-containing protein 17-like isoform X1 | | |
| TRINITY_DN38868_c0_g1_i1_len_382_208898.p1_type_internal_len_128 | XP_023934795.1putative inorganic phosphate cotransporter | | |
| TRINITY_DN37919_c0_g1_i1_len_382_208899.p1_type_internal_len_128 | ---NA--- | | |
| TRINITY_DN40312_c0_g1_i1_len_382_208901.p1_type_internal_len_128 | ---NA--- | | |
| TRINITY_DN36664_c0_g1_i1_len_382_208906.p1_type_internal_len_128 | ---NA--- | | |
| TRINITY_DN4445_c0_g1_i1_len_382_208929.p1_type_internal_len_128 | XP_018017684.1 tryptophan 2,3-dioxygenase-like | F:GO:0005488; P:GO:0006569; F:GO:0051213 | |
| TRINITY_DN6744_c0_g1_i1_len_382_208915.p1_type_internal_len_128 | ELR14136.1integrase core domain containing protein | F:GO:0003676; P:GO:0015074 | |
| TRINITY_DN34442_c0_g1_i1_len_570_202416.p1_type_5prime_partial_len_127 | ---NA--- | | |
| TRINITY_DN31970_c0_g1_i1_len_385_246998.p1_type_internal_len_128 | XP_018118418.1 ADP-dependent glucokinase-like | P:GO:0005975; F:GO:0016773 | |
| TRINITY_DN13789_c0_g1_i1_len_384_12343.p1_type_internal_len_128 | ---NA--- | | |

| | | | |
|---|---|---|---|
| TRINITY_DN7976_c0_g1_i1_len_381_208987.p1_type_internal_len_128 | ---NA--- | | |
| TRINITY_DN29279_c0_g1_i1_len_381_307771.p1_type_5prime_partial_len_127 | ---NA--- | | |
| TRINITY_DN17102_c0_g1_i1_len_401_246337.p1_type_5prime_partial_len_127 | ---NA--- | | |
| TRINITY_DN30920_c3_g1_i1_len_820_198373.p1_type_complete_len_127 | ---NA--- | | |
| TRINITY_DN38602_c0_g1_i1_len_385_208734.p1_type_internal_len_128 | ABQ22345.1elongation factor 1 gamma-like protein | F:GO:0003746; P:GO:0006414 | |
| TRINITY_DN9178_c0_g1_i1_len_383_208850.p2_type_internal_len_128 | ---NA--- | | |
| TRINITY_DN33780_c0_g1_i1_len_383_208851.p1_type_internal_len_128 | EFN88948.1Protein Jumonji | F:GO:0003677; C:GO:0005634 | |
| TRINITY_DN14280_c0_g1_i1_len_383_208853.p1_type_internal_len_128 | XP_018022742.1 protein odd-skipped-related 2-like | F:GO:0003676 | |
| TRINITY_DN22013_c1_g2_i1_len_383_208857.p1_type_internal_len_128 | ---NA--- | | |
| TRINITY_DN18023_c0_g1_i1_len_383_208861.p1_type_internal_len_128 | ---NA--- | | |
| TRINITY_DN293_c0_g1_i1_len_383_208862.p1_type_internal_len_128 | XP_018014922.1PREDICTED: uncharacterized protein LOC108671838 | | |
| TRINITY_DN32354_c0_g1_i1_len_383_208888.p2_type_internal_len_128 | XP_018023308.1 sodium channel protein 60E-like | F:GO:0005216; C:GO:0016021; P:GO:0034220 | |
| TRINITY_DN2364_c0_g1_i1_len_383_208887.p1_type_internal_len_128 | ---NA--- | | |
| TRINITY_DN12012_c0_g1_i1_len_383_208882.p1_type_internal_len_128 | XP_023716726.1bicaudal D-related protein homolog isoform X2 | | |
| TRINITY_DN630_c0_g1_i1_len_383_208878.p1_type_internal_len_128 | ---NA--- | | |
| TRINITY_DN2399_c0_g1_i1_len_383_208875.p1_type_internal_len_128 | ---NA--- | | |

| | | | |
|---|---|---|---|
| TRINITY_DN39503_c0_g1_i1_len_383_208869.p1_type_internal_len_128 | XP_018012344.1 dystonin-like isoform X2 | | |
| TRINITY_DN12522_c0_g1_i1_len_383_208868.p1_type_internal_len_128 | ---NA--- | | |
| TRINITY_DN14201_c0_g1_i1_len_384_208822.p1_type_5prime_partial_len_127 | ---NA--- | | |
| TRINITY_DN7687_c0_g1_i1_len_384_208815.p1_type_internal_len_128 | ---NA--- | | |
| TRINITY_DN25163_c0_g1_i1_len_1787_191711.p2_type_3prime_partial_len_128 | ---NA--- | | |
| TRINITY_DN15113_c0_g1_i1_len_383_208846.p1_type_internal_len_128 | ---NA--- | | |
| TRINITY_DN40936_c0_g1_i1_len_384_208837.p1_type_internal_len_128 | ---NA--- | | |
| TRINITY_DN39008_c0_g1_i1_len_384_208830.p1_type_internal_len_128 | ---NA--- | | |
| TRINITY_DN34247_c0_g1_i1_len_384_208827.p1_type_internal_len_128 | XP_018011187.1 alpha-1,3-mannosyl-glycoprotein 2-beta-N-acetylglucosaminyltransferase-like | P:GO:0006486; F:GO:0008375; C:GO:0016021 | |
| TRINITY_DN23525_c0_g1_i1_len_2258_155462.p2_type_complete_len_127 | ---NA--- | | |
| TRINITY_DN36004_c0_g1_i1_len_406_207754.p1_type_5prime_partial_len_127 | ---NA--- | | |
| TRINITY_DN317_c0_g1_i1_len_467_205316.p1_type_3prime_partial_len_128 | ---NA--- | | |
| TRINITY_DN20746_c0_g1_i3_len_409_207639.p1_type_5prime_partial_len_127 | ---NA--- | | |
| TRINITY_DN18404_c0_g1_i2_len_2583_154858.p2_type_complete_len_127 | ---NA--- | | |
| TRINITY_DN2404_c0_g1_i1_len_796_198667.p2_type_complete_len_127 | ---NA--- | | |
| TRINITY_DN37021_c0_g1_i1_len_383_247107.p1_type_internal_len_128 | ---NA--- | | |

| | | | |
|---|---|---|---|
| TRINITY_DN37884_c0_g1_i1_len_383_247116.p1_type_internal_len_128 | KTF92594.1hypothetical protein cypCar_00026626 | C:GO:0032156; C:GO:0044430; C:GO:0044448 | |
| TRINITY_DN15397_c0_g1_i1_len_383_247120.p1_type_internal_len_128 | ---NA--- | | |
| TRINITY_DN37739_c0_g1_i1_len_382_247133.p1_type_internal_len_128 | XP_022236443.1rho GTPase-activating protein 45-like isoform X2 | F:GO:0005096; P:GO:0035556; P:GO:0043547 | |
| TRINITY_DN22079_c1_g1_i1_len_1582_232636.p3_type_3prime_partial_len_128 | XP_015837926.1 probable JmjC domain-containing histone demethylation protein 2C isoform X1 | C:GO:0000785; F:GO:0000976; C:GO:0005634; F:GO:0031490; F:GO:0032454; P:GO:0033169 | |
| TRINITY_DN12847_c0_g1_i1_len_464_70153.p1_type_5prime_partial_len_127 | ---NA--- | | |
| TRINITY_DN10168_c0_g1_i1_len_382_247139.p1_type_internal_len_128 | XP_018026005.1 ATPase family AAA domain-containing protein 1-like | | |
| TRINITY_DN14191_c1_g1_i1_len_384_247035.p1_type_internal_len_128 | ---NA--- | | |
| TRINITY_DN36048_c0_g1_i1_len_384_247038.p1_type_internal_len_128 | ---NA--- | | |
| TRINITY_DN36655_c0_g1_i1_len_384_247046.p1_type_internal_len_128 | XP_018006524.1 sialin-like | | |
| TRINITY_DN15315_c0_g1_i1_len_383_247099.p1_type_5prime_partial_len_127 | ---NA--- | | |
| TRINITY_DN5943_c0_g1_i2_len_420_207148.p1_type_3prime_partial_len_128 | ---NA--- | | |
| TRINITY_DN26915_c0_g1_i1_len_384_247075.p1_type_3prime_partial_len_128 | ---NA--- | | |
| TRINITY_DN7638_c0_g1_i1_len_384_247074.p2_type_internal_len_128 | XP_023231445.1retinol dehydrogenase 14-like isoform X5 | | |
| TRINITY_DN35757_c0_g1_i1_len_384_247073.p1_type_internal_len_128 | ---NA--- | | |
| TRINITY_DN8695_c0_g1_i1_len_384_247063.p1_type_internal_len_128 | XP_022099413.1piggyBac transposable element-derived protein 4-like | | |
| TRINITY_DN34491_c0_g1_i1_len_384_247051.p1_type_internal_len_128 | ---NA--- | | |

| | | | |
|---|---|---|---|
| TRINITY_DN21703_c0_g1_i2_len_1484_296819.p2_type_5prime_partial_len_127 | XP_011148417.1methyltransferase-like protein 4 isoform X1 | | |
| TRINITY_DN29194_c0_g1_i1_len_381_247194.p1_type_internal_len_128 | ---NA--- | | |
| TRINITY_DN6783_c0_g1_i1_len_382_247168.p1_type_internal_len_128 | ---NA--- | | |
| TRINITY_DN6756_c0_g1_i1_len_382_247167.p2_type_internal_len_128 | ---NA--- | | |
| TRINITY_DN30523_c0_g1_i1_len_382_247166.p2_type_internal_len_128 | XP_018011656.1 laminin subunit alpha-2-like | F:GO:0005488 | |
| TRINITY_DN27883_c0_g1_i1_len_382_247157.p1_type_internal_len_128 | AQV11927.1ABC transporter B family protein | F:GO:0005524; C:GO:0016021; F:GO:0042626; P:GO:0055085 | EC:3.6.1.3; EC:3.6.1.15 |
| TRINITY_DN18667_c0_g1_i1_len_383_73060.p1_type_internal_len_128 | ---NA--- | | |
| TRINITY_DN14016_c0_g1_i1_len_453_244447.p1_type_5prime_partial_len_127 | ---NA--- | | |
| TRINITY_DN22172_c0_g1_i3_len_383_73059.p1_type_internal_len_128 | XP_015262007.1 zinc finger protein OZF-like | | |
| TRINITY_DN9890_c0_g1_i1_len_384_73055.p1_type_internal_len_128 | ---NA--- | | |
| TRINITY_DN9320_c0_g1_i2_len_383_73081.p1_type_internal_len_128 | ---NA--- | | |
| TRINITY_DN9794_c0_g1_i1_len_383_73064.p1_type_internal_len_128 | XP_014481887.1 collagen alpha-1(I) chain-like | C:GO:0005576; P:GO:0006030; F:GO:0008061 | |
| TRINITY_DN16782_c0_g1_i1_len_384_307648.p1_type_internal_len_128 | ---NA--- | | |
| TRINITY_DN25429_c1_g1_i2_len_670_200473.p1_type_5prime_partial_len_127 | ---NA--- | | |
| TRINITY_DN23539_c0_g1_i1_len_1129_195456.p1_type_5prime_partial_len_127 | XP_019631525.1 zinc finger protein 37-like | F:GO:0003676; C:GO:0005634 | |
| TRINITY_DN1952_c0_g1_i1_len_443_244795.p1_type_5prime_partial_len_127 | ---NA--- | | |

| | | | |
|---|---|---|---|
| TRINITY_DN3198_c0_g1_i1_len_382_73147.p2_type_internal_len_128 | XP_018019672.1 toll-like receptor Tollo | P:GO:0007165; C:GO:0016021 | |
| TRINITY_DN18257_c0_g1_i1_len_2537_29748.p2_type_5prime_partial_len_127 | ---NA--- | | |
| TRINITY_DN30299_c0_g1_i1_len_4818_187191.p1_type_5prime_partial_len_1540 | KZS18124.1putative Nucleolar pre-ribosomal-associated protein 1 | | |
| TRINITY_DN30795_c0_g1_i1_len_4931_187149.p1_type_5prime_partial_len_1539 | ---NA--- | | |
| TRINITY_DN26410_c1_g1_i5_len_5008_227781.p1_type_complete_len_1534 | KXJ06669.1Retrovirus-related Pol polyprotein from transposon 17.6 | | |
| TRINITY_DN30804_c0_g1_i1_len_4986_187130.p1_type_complete_len_1531 | XP_012264714.1mediator of RNA polymerase II transcription subunit 23 | | |
| TRINITY_DN29695_c0_g1_i1_len_5383_187038.p1_type_5prime_partial_len_1531 | AGL08684.1dicer 2 | F:GO:0003677; F:GO:0003723; F:GO:0004525; F:GO:0005524; P:GO:0006396; P:GO:0031047; P:GO:0090502 | EC:3.1.30; EC:3.1.26; EC:3.1.26.3 |
| TRINITY_DN15260_c0_g1_i1_len_4735_227861.p1_type_complete_len_1528 | XP_022191822.1 protein mahjong-like | P:GO:0016567 | |
| TRINITY_DN21289_c0_g1_i1_len_4923_153455.p1_type_complete_len_1526 | XP_018026730.1 UDP-glucose:glycoprotein glucosyltransferase 1-like isoform X2 | | |
| TRINITY_DN30624_c0_g1_i1_len_5395_187036.p1_type_complete_len_1526 | XP_023724702.1Bloom syndrome protein homolog isoform X2 | F:GO:0000166; P:GO:0006259; F:GO:0008026 | EC:3.6.1.3; EC:3.6.1.15 |
| TRINITY_DN30700_c0_g1_i1_len_5954_186939.p1_type_complete_len_1525 | PNF19410.1Zinc finger FYVE domain-containing protein 9 | F:GO:0046872 | |
| TRINITY_DN24449_c0_g1_i2_len_6566_227543.p1_type_5prime_partial_len_1520 | KDR21141.1Paired amphipathic helix protein Sin3a | P:GO:1903506 | |
| 2254\|_\|c35003_f15p31_2645_isoform_c35003_full_length_coverage_15_non_full_length_coverage_31_isoform_length_2645_18809 | OXA58763.1Surfeit locus protein 4 | C:GO:0016021 | |
| TRINITY_DN24468_c0_g1_i2_len_3791_228297.p1_type_complete_len_881 | XP_011173335.1DDB1- and CUL4-associated factor 6-like | | |

<table>
<tr><td>TRINITY_DN22203_c0_g1_i1_len_4163_228104.p1_type_complete_len_881</td><td>KZS14224.1Protein wech</td><td>F:GO:0046872</td><td></td></tr>
<tr><td>2257|_|c25659_f2p0_2643_isoform_c25659_full_length_coverage_2_non_full_length_coverage_0_isoform_length_2643_22981</td><td>XP_018018231.1 LETM1 and EF-hand domain-containing protein 1, mitochondrial-like isoform X2</td><td></td><td></td></tr>
<tr><td>2258|_|c7983_f2p1_2642_isoform_c7983_full_length_coverage_2_non_full_length_coverage_1_isoform_length_2642_17177</td><td>XP_018024452.1 protein SpAN-like</td><td></td><td></td></tr>
<tr><td>2259|_|c2301_f3p1_2642_isoform_c2301_full_length_coverage_3_non_full_length_coverage_1_isoform_length_2642_16308</td><td>PNF19281.1tRNA (cytosine(34)-C(5))-methyltransferase</td><td>F:GO:0016740</td><td></td></tr>
<tr><td>2260|_|c2457_f2p2_2642_isoform_c2457_full_length_coverage_2_non_full_length_coverage_2_isoform_length_2642_16427</td><td>XP_018006541.1 nuclear pore complex protein Nup205-like</td><td>C:GO:0005643</td><td></td></tr>
<tr><td>2261|_|c1775_f6p5_2642_isoform_c1775_full_length_coverage_6_non_full_length_coverage_5_isoform_length_2642_11487</td><td>---NA---</td><td></td><td></td></tr>
<tr><td>2262|_|c13367_f2p0_2641_isoform_c13367_full_length_coverage_2_non_full_length_coverage_0_isoform_length_2641_17587</td><td>XP_016277811.1 zinc finger protein 665-like isoform X1</td><td>F:GO:0003677; C:GO:0005634</td><td></td></tr>
<tr><td>2263|_|c33229_f7p7_2641_isoform_c33229_full_length_coverage_7_non_full_length_coverage_7_isoform_length_2641_14214</td><td>XP_012144472.1 probable ATP-dependent RNA helicase DDX10</td><td></td><td></td></tr>
<tr><td>TRINITY_DN39789_c0_g1_i1_len_454_205813.p1_type_5prime_partial_len_127</td><td>---NA---</td><td></td><td></td></tr>
<tr><td>TRINITY_DN1103_c0_g1_i1_len_382_73111.p1_type_internal_len_128</td><td>---NA---</td><td></td><td></td></tr>
<tr><td>TRINITY_DN30405_c0_g1_i1_len_382_73109.p1_type_internal_len_128</td><td>---NA---</td><td></td><td></td></tr>
<tr><td>TRINITY_DN5545_c0_g1_i2_len_382_73133.p1_type_internal_len_128</td><td>---NA---</td><td></td><td></td></tr>
<tr><td>TRINITY_DN37884_c0_g1_i1_len_473_205121.p1_type_3prime_partial_len_128</td><td>KPJ19725.1Zinc finger protein ubi-d4 A</td><td></td><td></td></tr>
</table>

| | | | |
|---|---|---|---|
| TRINITY_DN33453_c0_g1_i1_len_555_202762.p1_type_complete_len_127 | ---NA--- | | |
| TRINITY_DN21917_c0_g1_i1_len_383_307685.p1_type_internal_len_128 | ---NA--- | | |
| TRINITY_DN4988_c0_g1_i1_len_383_307695.p2_type_internal_len_128 | XP_018006437.1 moesin/ezrin/radixin homolog 1-like isoform X4 | | |
| TRINITY_DN12514_c0_g1_i2_len_641_66461.p1_type_3prime_partial_len_128 | XP_024219538.1zinc finger Y-chromosomal protein-like isoform X5 | | |
| TRINITY_DN16028_c0_g1_i1_len_379_12574.p1_type_internal_len_127 | ---NA--- | | |
| TRINITY_DN18128_c0_g1_i1_len_2192_58951.p4_type_complete_len_126 | ---NA--- | | |
| TRINITY_DN41056_c0_g1_i1_len_478_204969.p1_type_3prime_partial_len_127 | PNF24359.1Ubiquitin carboxyl-terminal hydrolase 32 | F:GO:0004843; F:GO:0005509; P:GO:0006511; P:GO:0016579 | EC:3.4.19.12 |
| TRINITY_DN19042_c0_g1_i2_len_569_99965.p1_type_3prime_partial_len_127 | ---NA--- | | |
| TRINITY_DN18192_c0_g1_i1_len_1338_61323.p2_type_complete_len_126 | ---NA--- | | |
| TRINITY_DN1158_c0_g1_i2_len_379_42280.p1_type_internal_len_127 | ---NA--- | | |
| TRINITY_DN16912_c0_g1_i2_len_1229_32407.p2_type_5prime_partial_len_126 | ---NA--- | | |
| TRINITY_DN18988_c0_g1_i1_len_736_163081.p1_type_5prime_partial_len_126 | ---NA--- | | |
| TRINITY_DN20106_c0_g1_i1_len_784_198820.p1_type_complete_len_126 | ABU97474.1profilin | F:GO:0003779 | |
| TRINITY_DN8585_c0_g1_i1_len_1218_94405.p1_type_5prime_partial_len_126 | ---NA--- | | |
| TRINITY_DN5473_c0_g1_i2_len_417_207292.p1_type_5prime_partial_len_126 | XP_023212837.1sugar transporter SWEET1-like | P:GO:0008643; C:GO:0016020; F:GO:0051119 | |
| TRINITY_DN17478_c0_g1_i2_len_785_34902.p1_type_complete_len_126 | ---NA--- | | |

| | | | |
|---|---|---|---|
| TRINITY_DN5154_c0_g1_i1_len_646_164269.p1_type_5prime_partial_len_126 | ---NA--- | | |
| TRINITY_DN15317_c0_g1_i1_len_1187_125480.p1_type_complete_len_126 | ---NA--- | | |
| TRINITY_DN22757_c1_g1_i2_len_2794_229287.p4_type_5prime_partial_len_126 | ---NA--- | | |
| TRINITY_DN2270_c0_g1_i1_len_505_204077.p1_type_3prime_partial_len_127 | ---NA--- | | |
| TRINITY_DN924_c0_g1_i1_len_462_305078.p1_type_complete_len_126 | ---NA--- | | |
| TRINITY_DN24541_c0_g1_i1_len_1425_60972.p3_type_complete_len_126 | ALT10383.1juvenile hormone esterase-like protein 1 | F:GO:0016787 | |
| TRINITY_DN17115_c0_g1_i2_len_654_273352.p1_type_complete_len_126 | ---NA--- | | |
| TRINITY_DN16210_c0_g1_i1_len_2406_229982.p2_type_complete_len_126 | AEL23003.1multiprotein bridging factor 1 | | |
| TRINITY_DN8557_c0_g1_i1_len_589_274301.p2_type_complete_len_126 | ---NA--- | | |
| TRINITY_DN31194_c0_g2_i3_len_616_201455.p1_type_complete_len_126 | ---NA--- | | |
| TRINITY_DN17383_c0_g1_i1_len_1241_2479.p1_type_complete_len_126 | ---NA--- | | |
| TRINITY_DN981_c0_g1_i1_len_1283_194371.p4_type_3prime_partial_len_127 | XP_018013994.1 spondin-2-like | | |
| TRINITY_DN23421_c0_g1_i1_len_1847_231582.p2_type_3prime_partial_len_127 | ---NA--- | | |
| TRINITY_DN15132_c0_g1_i1_len_866_161795.p1_type_5prime_partial_len_126 | ---NA--- | | |
| TRINITY_DN16990_c0_g1_i1_len_1085_298349.p2_type_complete_len_126 | XP_018022170.1 dnaJ homolog subfamily C member 16-like isoform X1 | C:GO:0005623; C:GO:0016020; C:GO:0016021; P:GO:0045454 | |
| TRINITY_DN31270_c1_g2_i5_len_531_203392.p1_type_5prime_partial_len_126 | ---NA--- | | |

| | | | |
|---|---|---|---|
| TRINITY_DN18352_c0_g1_i1_len_380_12508.p1_type_internal_len_127 | ---NA--- | | |
| TRINITY_DN4643_c0_g1_i1_len_379_42262.p1_type_internal_len_127 | ---NA--- | | |
| TRINITY_DN19754_c0_g1_i1_len_512_203869.p1_type_5prime_partial_len_126 | ---NA--- | | |
| TRINITY_DN14689_c0_g1_i1_len_698_239227.p1_type_complete_len_126 | ---NA--- | | |
| TRINITY_DN32290_c0_g1_i1_len_487_204663.p1_type_3prime_partial_len_127 | XP_023235400.1ETS-like protein pointed isoform X3 | | |
| TRINITY_DN21021_c0_g1_i1_len_1136_235141.p2_type_5prime_partial_len_126 | ---NA--- | | |
| TRINITY_DN22722_c0_g1_i1_len_379_12556.p1_type_internal_len_127 | XP_016977495.1 serine proteinase stubble | F:GO:0004252; P:GO:0006508 | |
| TRINITY_DN15563_c0_g1_i2_len_379_42266.p1_type_internal_len_127 | ---NA--- | | |
| TRINITY_DN17120_c0_g1_i1_len_758_162846.p1_type_complete_len_126 | XP_015604623.1succinate dehydrogenase assembly factor 3, mitochondrial | | |
| TRINITY_DN10430_c0_g1_i1_len_484_304530.p1_type_3prime_partial_len_127 | ---NA--- | | |
| TRINITY_DN3027_c0_g1_i1_len_528_203465.p2_type_3prime_partial_len_127 | ---NA--- | | |
| TRINITY_DN24019_c0_g1_i2_len_836_198192.p1_type_5prime_partial_len_126 | ---NA--- | | |
| TRINITY_DN26892_c0_g1_i1_len_379_12543.p1_type_internal_len_127 | XP_016908713.1 glutamate-gated chloride channel isoform X2 | F:GO:0004888; F:GO:0005230; C:GO:0005886; C:GO:0016021; P:GO:0034220 | |
| TRINITY_DN10376_c0_g1_i1_len_432_206662.p1_type_5prime_partial_len_126 | ---NA--- | | |
| TRINITY_DN26145_c3_g1_i2_len_1358_233733.p1_type_complete_len_126 | ---NA--- | | |
| TRINITY_DN21713_c0_g1_i4_len_939_299174.p2_type_5prime_partial_len_126 | ---NA--- | | |

| | | | |
|---|---|---|---|
| TRINITY_DN22892_c0_g1_i1_len_482_204818.p1_type_5prime_partial_len_126 | XP_018008386.1 myoferlin-like | | |
| TRINITY_DN29006_c1_g1_i3_len_1264_194485.p1_type_5prime_partial_len_126 | ---NA--- | | |
| TRINITY_DN7650_c0_g1_i1_len_386_170802.p1_type_5prime_partial_len_126 | ---NA--- | | |
| TRINITY_DN24371_c0_g1_i1_len_381_12458.p1_type_internal_len_127 | ---NA--- | | |
| TRINITY_DN22637_c0_g1_i1_len_993_236169.p1_type_5prime_partial_len_126 | ---NA--- | | |
| TRINITY_DN24946_c0_g1_i1_len_2325_230179.p3_type_complete_len_126 | ---NA--- | | |
| TRINITY_DN1642_c0_g1_i1_len_379_307867.p1_type_internal_len_127 | ---NA--- | | |
| TRINITY_DN19876_c0_g1_i1_len_1286_234133.p2_type_complete_len_126 | ---NA--- | | |
| TRINITY_DN30005_c0_g1_i1_len_415_169656.p1_type_5prime_partial_len_126 | ---NA--- | | |
| TRINITY_DN31681_c0_g1_i1_len_380_307851.p1_type_internal_len_127 | ---NA--- | | |
| TRINITY_DN6095_c0_g1_i1_len_380_307843.p1_type_internal_len_127 | ABV65905.1sodium/potassium-transporting ATPase subunit beta | C:GO:0005890; P:GO:0006813; P:GO:0006814 | |
| TRINITY_DN27308_c0_g1_i1_len_1371_193833.p3_type_complete_len_126 | XP_014678597.1 zinc finger protein 271-like isoform X2 | | |
| TRINITY_DN19932_c0_g1_i7_len_1048_235735.p2_type_complete_len_126 | AFW99840.1troponin I | C:GO:0005861 | |
| TRINITY_DN17465_c0_g1_i1_len_1456_267913.p2_type_5prime_partial_len_126 | ---NA--- | | |
| TRINITY_DN11968_c0_g1_i2_len_493_38850.p1_type_5prime_partial_len_126 | ---NA--- | | |
| TRINITY_DN16776_c0_g1_i1_len_445_102801.p1_type_5prime_partial_len_126 | ---NA--- | | |

| | | | |
|---|---|---|---|
| TRINITY_DN31800_c0_g1_i1_len_439_168798.p1_type_5prime_partial_len_126 | ADC42879.1serine proteinase inhibitor 8 | C:GO:0005615 | |
| TRINITY_DN18844_c0_g1_i6_len_378_307896.p1_type_internal_len_127 | ---NA--- | | |
| TRINITY_DN4636_c0_g1_i1_len_461_102336.p1_type_5prime_partial_len_126 | ---NA--- | | |
| TRINITY_DN28181_c0_g1_i5_len_1367_193847.p1_type_5prime_partial_len_126 | ---NA--- | | |
| TRINITY_DN23099_c0_g1_i1_len_380_307838.p1_type_internal_len_127 | PRD22338.1zinc finger protein | F:GO:0003676 | |
| TRINITY_DN10199_c0_g1_i1_len_379_171072.p1_type_internal_len_127 | ---NA--- | | |
| TRINITY_DN25763_c0_g1_i1_len_379_171075.p1_type_internal_len_127 | ---NA--- | | |
| TRINITY_DN31059_c0_g1_i1_len_3126_188344.p3_type_5prime_partial_len_126 | ---NA--- | | |
| TRINITY_DN33037_c0_g1_i1_len_467_102176.p1_type_5prime_partial_len_126 | ---NA--- | | |
| TRINITY_DN18962_c0_g1_i1_len_380_171061.p1_type_internal_len_127 | ---NA--- | | |
| TRINITY_DN21004_c0_g1_i1_len_2270_230324.p2_type_5prime_partial_len_126 | ---NA--- | | |
| TRINITY_DN17741_c0_g1_i1_len_932_236683.p2_type_3prime_partial_len_127 | ---NA--- | | |
| TRINITY_DN24517_c0_g2_i2_len_1601_232535.p1_type_5prime_partial_len_126 | ---NA--- | | |
| TRINITY_DN34220_c0_g1_i1_len_521_203610.p1_type_3prime_partial_len_127 | ---NA--- | | |
| TRINITY_DN37842_c0_g1_i1_len_539_203178.p1_type_5prime_partial_len_126 | XP_022251018.1spondin-1-like isoform X3 | F:GO:0004867; P:GO:0010951 | |
| TRINITY_DN41885_c0_g1_i1_len_398_208115.p1_type_5prime_partial_len_126 | XP_018017462.1PREDICTED: uncharacterized protein LOC108674075 isoform X1 | C:GO:0005576; P:GO:0006030; F:GO:0008061 | |

| | | | |
|---|---|---|---|
| TRINITY_DN3284_c0_g1_i1_len_833_198228.p1_type_5prime_partial_len_126 | XP_014677747.1 heparan sulfate 2-O-sulfotransferase 1-like | | |
| TRINITY_DN22843_c0_g1_i1_len_2747_229353.p2_type_5prime_partial_len_126 | XP_003701626.1 transketolase-like protein 2 isoform X4 | | |
| TRINITY_DN7170_c0_g1_i1_len_379_171090.p1_type_internal_len_127 | ---NA--- | | |
| TRINITY_DN34409_c0_g1_i1_len_379_171094.p1_type_internal_len_127 | ---NA--- | | |
| TRINITY_DN26201_c0_g1_i1_len_381_170999.p1_type_internal_len_127 | AET79205.1sterile-alpha and armadillo motif containing protein | P:GO:0009967; P:GO:0034128; F:GO:0035591; P:GO:0048678 | |
| TRINITY_DN22672_c0_g1_i1_len_623_201346.p1_type_3prime_partial_len_127 | ---NA--- | | |
| TRINITY_DN31917_c0_g1_i1_len_511_166816.p1_type_5prime_partial_len_126 | ---NA--- | | |
| TRINITY_DN10223_c0_g1_i2_len_455_133523.p1_type_complete_len_126 | ---NA--- | | |
| TRINITY_DN10619_c0_g1_i1_len_380_171038.p1_type_internal_len_127 | ---NA--- | | |
| TRINITY_DN25999_c0_g1_i1_len_480_204874.p2_type_5prime_partial_len_126 | ---NA--- | | |
| TRINITY_DN1082_c0_g1_i1_len_381_208971.p1_type_internal_len_127 | ---NA--- | | |
| TRINITY_DN41829_c0_g1_i1_len_381_208984.p1_type_internal_len_127 | XP_018016665.1PREDICTED: uncharacterized protein LOC108673361 | | |
| TRINITY_DN3560_c0_g1_i1_len_381_208951.p1_type_internal_len_127 | ---NA--- | | |
| TRINITY_DN13491_c0_g1_i1_len_381_208953.p1_type_internal_len_127 | XP_018010068.1 cytochrome P450 9e2-like isoform X2 | | |
| TRINITY_DN36943_c0_g1_i1_len_381_208959.p1_type_internal_len_127 | XP_023229462.1alpha-1,3-mannosyl-glycoprotein 4-beta-N-acetylglucosaminyltransferase B-like | F:GO:0016757 | |

| | | | |
|---|---|---|---|
| TRINITY_DN19706_c0_g1_i2_len_2995_264961.p1_type_complete_len_880 | XP_018016760.1PREDICTED: uncharacterized protein LOC108673437 isoform X3 | | |
| TRINITY_DN25692_c1_g4_i1_len_2759_229332.p1_type_5prime_partial_len_880 | BAM37459.1argonaute 2 | F:GO:0003676; P:GO:0031047 | |
| 2266\|_\|c2862_f4p5_2640_isoform_c2862_full_length_coverage_4_non_full_length_coverage_5_isoform_length_2640_12225 | AEI83216.1partner of drosha-like protein | F:GO:0003723; F:GO:0020037; P:GO:0031053; F:GO:0042802; C:GO:0070877 | |
| 2268\|_\|c33172_f20p14_2639_isoform_c33172_full_length_coverage_20_non_full_length_coverage_14_isoform_length_2639_14194 | AIT97134.1innexin 2 | C:GO:0005886; C:GO:0005921; P:GO:0006811; C:GO:0016021 | |
| 2270\|_\|c2713_f2p1_2639_isoform_c2713_full_length_coverage_2_non_full_length_coverage_1_isoform_length_2639_16640 | PNF18279.1CAD protein | F:GO:0000166; F:GO:0016743; F:GO:0016810; F:GO:0043168; P:GO:0044237; P:GO:0044238; P:GO:0044281; P:GO:1901564 | |
| 2272\|_\|c2454_f5p6_2639_isoform_c2454_full_length_coverage_5_non_full_length_coverage_6_isoform_length_2639_11882 | ---NA--- | | |
| 2273\|_\|c2125_f4p6_2639_isoform_c2125_full_length_coverage_4_non_full_length_coverage_6_isoform_length_2639_11679 | ---NA--- | | |
| 2274\|_\|c29810_f2p1_2638_isoform_c29810_full_length_coverage_2_non_full_length_coverage_1_isoform_length_2638_23145 | ---NA--- | | |
| 2275\|_\|c2111_f2p10_2638_isoform_c2111_full_length_coverage_2_non_full_length_coverage_10_isoform_length_2638_28226 | ---NA--- | | |
| 2276\|_\|c12259_f4p3_2638_isoform_c12259_full_length_coverage_4_non_full_length_coverage_3_isoform_length_2638_17498 | XP_015682811.1 transcription factor E4F1 | F:GO:0003676; F:GO:0046872 | |
| TRINITY_DN26397_c0_g1_i1_len_1594_192596.p2_type_5prime_partial_len_126 | ---NA--- | | |

| | | | |
|---|---|---|---|
| TRINITY_DN16169_c0_g1_i1_len_769_97353.p1_type_5prime_partial_len_126 | ---NA--- | | |
| TRINITY_DN37967_c0_g1_i1_len_380_209031.p1_type_internal_len_127 | ---NA--- | | |
| TRINITY_DN3155_c0_g1_i1_len_380_209032.p1_type_internal_len_127 | PSN35406.1hypothetical protein C0J52_21266 | | |
| TRINITY_DN11959_c1_g1_i1_len_380_209041.p1_type_internal_len_127 | ---NA--- | | |
| TRINITY_DN22203_c0_g1_i1_len_4163_228104.p3_type_complete_len_126 | ---NA--- | | |
| TRINITY_DN40087_c0_g1_i1_len_380_209000.p1_type_internal_len_127 | XP_018014866.1PREDICTED: uncharacterized protein LOC108671794 isoform X2 | F:GO:0003779; P:GO:0016043 | |
| TRINITY_DN34486_c0_g1_i1_len_380_209008.p1_type_internal_len_127 | XP_021920327.1disks large homolog 5 isoform X1 | | |
| TRINITY_DN34055_c0_g1_i1_len_380_209014.p1_type_internal_len_127 | ---NA--- | | |
| TRINITY_DN35036_c0_g1_i1_len_380_209016.p1_type_internal_len_127 | XP_022252041.1formin-like protein CG32138 | F:GO:0005515; P:GO:0016043 | |
| TRINITY_DN7249_c0_g1_i1_len_380_209019.p1_type_internal_len_127 | AJR21491.1RNA helicase | F:GO:0003723; F:GO:0032575; P:GO:0035194 | EC:3.6.1.3; EC:3.6.1.15 |
| TRINITY_DN14889_c0_g1_i2_len_380_209024.p1_type_internal_len_127 | ---NA--- | | |
| TRINITY_DN20612_c0_g1_i3_len_573_67555.p1_type_5prime_partial_len_126 | ---NA--- | | |
| TRINITY_DN24789_c0_g1_i1_len_1042_196176.p1_type_5prime_partial_len_126 | ---NA--- | | |
| TRINITY_DN24731_c1_g1_i2_len_1120_195527.p3_type_5prime_partial_len_126 | ---NA--- | | |
| TRINITY_DN12955_c0_g1_i1_len_672_200427.p1_type_5prime_partial_len_126 | ---NA--- | | |
| TRINITY_DN41890_c0_g1_i1_len_382_208926.p1_type_internal_len_127 | ---NA--- | | |

| | | | |
|---|---|---|---|
| TRINITY_DN35404_c0_g1_i1_len_382_208928.p1_type_internal_len_127 | ---NA--- | | |
| TRINITY_DN35867_c0_g1_i1_len_382_208931.p1_type_internal_len_127 | XP_018010044.1 glucose dehydrogenase [FAD, quinone]-like | | |
| TRINITY_DN5281_c0_g1_i1_len_382_208942.p1_type_internal_len_127 | ---NA--- | | |
| TRINITY_DN8750_c0_g1_i1_len_912_197364.p1_type_complete_len_126 | ---NA--- | | |
| TRINITY_DN34184_c0_g1_i1_len_381_208947.p1_type_internal_len_127 | ---NA--- | | |
| TRINITY_DN42113_c0_g1_i1_len_381_208948.p1_type_internal_len_127 | ---NA--- | | |
| TRINITY_DN20744_c0_g1_i1_len_416_71702.p1_type_5prime_partial_len_126 | ---NA--- | | |
| TRINITY_DN7435_c0_g1_i1_len_380_247233.p1_type_internal_len_127 | ---NA--- | | |
| TRINITY_DN37942_c0_g1_i1_len_380_247235.p1_type_internal_len_127 | XP_021936522.1ankyrin-3-like isoform X8 | | |
| TRINITY_DN2260_c0_g1_i1_len_380_247240.p1_type_internal_len_127 | ---NA--- | | |
| TRINITY_DN34471_c0_g1_i1_len_380_247242.p1_type_3prime_partial_len_127 | XP_018013891.1 rho guanine nucleotide exchange factor 17-like | F:GO:0005089; P:GO:0030036 | |
| TRINITY_DN28098_c0_g1_i1_len_380_247244.p1_type_internal_len_127 | ---NA--- | | |
| TRINITY_DN451_c0_g1_i1_len_380_247252.p1_type_internal_len_127 | ---NA--- | | |
| TRINITY_DN28121_c0_g1_i3_len_2381_189748.p3_type_complete_len_126 | ---NA--- | | |
| TRINITY_DN14516_c0_g1_i4_len_465_70128.p1_type_3prime_partial_len_127 | ---NA--- | | |
| TRINITY_DN28676_c0_g1_i2_len_381_247196.p1_type_internal_len_127 | ---NA--- | | |

| | | | |
|---|---|---|---|
| TRINITY_DN7005_c1_g1_i1_len_379_209076.p1_type_internal_len_127 | XP_017771990.1 acyl-CoA synthetase family member 3, mitochondrial | | |
| TRINITY_DN34619_c0_g1_i1_len_379_209080.p1_type_internal_len_127 | ---NA--- | | |
| TRINITY_DN27797_c0_g1_i1_len_381_247205.p1_type_internal_len_127 | ---NA--- | | |
| TRINITY_DN23088_c0_g1_i1_len_378_105262.p1_type_internal_len_127 | XP_021941822.1tyrosine-protein phosphatase non-receptor type 9-like | | |
| TRINITY_DN3249_c0_g1_i1_len_379_247295.p1_type_internal_len_127 | ---NA--- | | |
| TRINITY_DN23604_c1_g1_i2_len_379_247310.p1_type_internal_len_127 | ---NA--- | | |
| TRINITY_DN26423_c0_g1_i1_len_379_247314.p1_type_internal_len_127 | ---NA--- | | |
| TRINITY_DN34654_c0_g1_i1_len_380_247255.p1_type_internal_len_127 | XP_018009983.1PREDICTED: uncharacterized protein LOC108667470 isoform X2 | | |
| TRINITY_DN36870_c0_g1_i1_len_380_247262.p1_type_internal_len_127 | ---NA--- | | |
| TRINITY_DN28592_c0_g1_i1_len_379_247288.p1_type_internal_len_127 | XP_026287248.1sialic acid synthase | F:GO:0003824; P:GO:0016051 | |
| TRINITY_DN11754_c0_g1_i1_len_380_209048.p1_type_internal_len_127 | ---NA--- | | |
| TRINITY_DN19238_c0_g1_i1_len_703_199944.p1_type_5prime_partial_len_126 | AHH32890.1integrin beta 1 | P:GO:0007155; P:GO:0007229; C:GO:0016021 | |
| TRINITY_DN24687_c0_g1_i4_len_4133_228118.p3_type_complete_len_126 | ---NA--- | | |
| TRINITY_DN13432_c0_g1_i1_len_380_209042.p1_type_internal_len_127 | ---NA--- | | |
| TRINITY_DN33129_c0_g1_i1_len_382_247144.p1_type_internal_len_127 | AJR22372.1serine proteinase inhibitor-2 | C:GO:0005615 | |
| TRINITY_DN37654_c0_g1_i1_len_382_247154.p1_type_internal_len_127 | PNF32380.1Peroxisomal targeting signal 1 receptor | | |

| | | | |
|---|---|---|---|
| TRINITY_DN12055_c0_g1_i1_len_382_247155.p1_type_internal_len_127 | ---NA--- | | |
| TRINITY_DN27148_c0_g1_i1_len_382_42128.p1_type_internal_len_127 | ACR15870.1serine protease | F:GO:0008233 | |
| TRINITY_DN2057_c0_g1_i1_len_379_209059.p1_type_internal_len_127 | XP_022255566.1thrombospondin type-1 domain-containing protein 4-like | P:GO:0006508; F:GO:0008233 | |
| TRINITY_DN31912_c0_g1_i1_len_379_209069.p1_type_internal_len_127 | ---NA--- | | |
| TRINITY_DN10660_c0_g1_i1_len_379_209070.p1_type_internal_len_127 | XP_018027696.1PREDICTED: uncharacterized protein LOC108682943 isoform X1 | | |
| TRINITY_DN16269_c0_g1_i1_len_900_270930.p1_type_complete_len_126 | ---NA--- | | |
| TRINITY_DN23589_c0_g1_i4_len_382_73141.p1_type_internal_len_127 | ---NA--- | | |
| TRINITY_DN20714_c0_g1_i2_len_835_64262.p2_type_3prime_partial_len_127 | OXA64138.1Programmed cell death protein 6 | F:GO:0005509 | |
| TRINITY_DN16033_c0_g1_i1_len_380_73201.p1_type_internal_len_127 | ACG60513.1chitinase 1 precursor | F:GO:0004568; C:GO:0005576; P:GO:0005975; P:GO:0006030; F:GO:0008061 | EC:3.2.1.14 |
| TRINITY_DN7908_c0_g1_i1_len_381_73168.p1_type_internal_len_127 | ---NA--- | | |
| TRINITY_DN30171_c0_g1_i1_len_381_73166.p1_type_internal_len_127 | ---NA--- | | |
| TRINITY_DN12168_c0_g1_i2_len_381_73163.p1_type_internal_len_127 | AYC12381.1bone morphogenetic protein receptor type 1B | F:GO:0004672; F:GO:0005488; P:GO:0009987 | |
| TRINITY_DN28035_c0_g1_i2_len_1668_192218.p3_type_5prime_partial_len_126 | XP_007229530.1cytochrome P450 2U1 | F:GO:0005488; F:GO:0016491 | |
| TRINITY_DN24230_c0_g1_i1_len_755_199208.p2_type_3prime_partial_len_127 | ---NA--- | | |
| TRINITY_DN20886_c0_g1_i1_len_392_208400.p1_type_5prime_partial_len_126 | ---NA--- | | |

| | | | |
|---|---|---|---|
| TRINITY_DN30104_c0_g1_i1_len_463_244134.p1_type_5prime_partial_len_126 | ---NA--- | | |
| TRINITY_DN27890_c0_g1_i1_len_379_73262.p2_type_internal_len_127 | AAX48011.1prophenoloxidase, partial | F:GO:0016491; F:GO:0046872; P:GO:0055114 | |
| TRINITY_DN10314_c0_g1_i1_len_605_201657.p1_type_3prime_partial_len_127 | ---NA--- | | |
| TRINITY_DN148_c0_g1_i1_len_378_73288.p1_type_internal_len_127 | ---NA--- | | |
| TRINITY_DN2398_c0_g1_i1_len_379_73236.p1_type_internal_len_127 | ---NA--- | | |
| TRINITY_DN16261_c0_g1_i1_len_380_73214.p1_type_internal_len_127 | ---NA--- | | |
| TRINITY_DN13187_c1_g1_i1_len_379_279783.p1_type_internal_len_127 | XP_018008897.1PREDICTED: uncharacterized protein LOC108666520 isoform X2 | | |
| TRINITY_DN4696_c0_g1_i1_len_426_245408.p1_type_5prime_partial_len_126 | ---NA--- | | |
| TRINITY_DN9811_c0_g1_i1_len_379_279756.p1_type_internal_len_127 | ---NA--- | | |
| TRINITY_DN17467_c0_g1_i2_len_408_246085.p1_type_5prime_partial_len_126 | ---NA--- | | |
| TRINITY_DN16579_c0_g1_i2_len_665_301524.p1_type_5prime_partial_len_126 | ---NA--- | | |
| TRINITY_DN14183_c0_g1_i1_len_3980_264522.p2_type_complete_len_126 | ---NA--- | | |
| TRINITY_DN20574_c0_g2_i1_len_528_242297.p1_type_5prime_partial_len_126 | ---NA--- | | |
| TRINITY_DN18003_c0_g2_i1_len_463_39562.p1_type_5prime_partial_len_126 | ---NA--- | | |
| TRINITY_DN11737_c0_g1_i1_len_1163_195215.p2_type_5prime_partial_len_126 | ---NA--- | | |
| TRINITY_DN15201_c0_g1_i1_len_573_302781.p1_type_5prime_partial_len_126 | ---NA--- | | |

| | | | |
|---|---|---|---|
| TRINITY_DN10653_c0_g1_i1_len_451_244536.p1_type_5prime_partial_len_126 | ---NA--- | | |
| TRINITY_DN8339_c0_g1_i1_len_380_279737.p1_type_internal_len_127 | ---NA--- | | |
| TRINITY_DN29202_c0_g1_i1_len_380_279742.p1_type_internal_len_127 | XP_018022865.1 ATP-binding cassette sub-family A member 3-like | | |
| TRINITY_DN25808_c0_g1_i1_len_380_279745.p1_type_internal_len_127 | ---NA--- | | |
| TRINITY_DN23007_c0_g1_i1_len_380_279749.p1_type_internal_len_127 | XP_018024056.1 ubiquitin-protein ligase E3B-like | F:GO:0004842; P:GO:0016567; F:GO:0016874 | |
| TRINITY_DN31012_c0_g1_i1_len_380_279750.p1_type_internal_len_127 | XP_018011235.1 mucin-22-like isoform X3 | | |
| TRINITY_DN31059_c0_g1_i1_len_381_279703.p1_type_internal_len_127 | ---NA--- | | |
| TRINITY_DN7442_c0_g1_i1_len_379_209090.p2_type_internal_len_127 | XP_023237780.1disheveled-associated activator of morphogenesis 1-like isoform X3 | | |
| TRINITY_DN153_c0_g1_i1_len_379_209096.p1_type_internal_len_127 | ---NA--- | | |
| TRINITY_DN8367_c0_g1_i1_len_379_209087.p1_type_internal_len_127 | XP_026490636.1homeobox protein GBX-1-like | | |
| TRINITY_DN12921_c0_g1_i2_len_378_209118.p1_type_internal_len_127 | ---NA--- | | |
| TRINITY_DN35324_c0_g1_i1_len_378_209112.p2_type_internal_len_127 | ---NA--- | | |
| TRINITY_DN5008_c0_g1_i1_len_379_209102.p1_type_internal_len_127 | XP_018019239.1PREDICTED: uncharacterized protein LOC108675722 | | |
| TRINITY_DN40067_c0_g1_i1_len_379_209100.p1_type_internal_len_127 | ---NA--- | | |
| TRINITY_DN29228_c0_g1_i1_len_516_68719.p1_type_5prime_partial_len_126 | ANA91277.1crustinI-5 | C:GO:0005576; P:GO:0010466; F:GO:0030414 | |

| | | | |
|---|---|---|---|
| TRINITY_DN22156_c0_g1_i4_len_1128_159789.p3_type_5prime_partial_len_126 | XP_002128232.2D-beta-hydroxybutyrate dehydrogenase, mitochondrial-like isoform X1 | C:GO:0016020; C:GO:0016021; F:GO:0016491; P:GO:0055114 | |
| TRINITY_DN7504_c0_g1_i1_len_378_209132.p2_type_internal_len_127 | ODM98238.1Epoxide hydrolase 4 | F:GO:0003824; C:GO:0016020; C:GO:0016021; F:GO:0016787 | |
| TRINITY_DN14379_c0_g1_i4_len_379_136284.p1_type_internal_len_127 | ---NA--- | | |
| TRINITY_DN14742_c0_g1_i1_len_465_205390.p1_type_3prime_partial_len_127 | ---NA--- | | |
| TRINITY_DN21290_c0_g1_i1_len_379_209099.p1_type_internal_len_127 | AYD41591.1mannose receptor | C:GO:0016020; C:GO:0016021; F:GO:0030246 | |
| 2278\|_\|c2280_f3p2_2638_isoform_c2280_full_length_coverage_3_non_full_length_coverage_2_isoform_length_2638_11773 | PNF17179.1Acyl-CoA dehydrogenase family member 9, mitochondrial | F:GO:0016627 | |
| TRINITY_DN30902_c0_g1_i1_len_3489_187944.p1_type_5prime_partial_len_879 | XP_018019143.1 periostin-like | | |
| TRINITY_DN30441_c0_g1_i1_len_4994_187126.p1_type_complete_len_879 | KDR11247.1AP-1 complex subunit gamma-1 | C:GO:0005794; P:GO:0015031; C:GO:0030117; C:GO:0031410 | |
| 2281\|_\|c19849_f2p5_2637_isoform_c19849_full_length_coverage_2_non_full_length_coverage_5_isoform_length_2637_26605 | ---NA--- | | |
| 2282\|_\|c30154_f2p0_2637_isoform_c30154_full_length_coverage_2_non_full_length_coverage_0_isoform_length_2637_18517 | ---NA--- | | |
| 2283\|_\|c2521_f2p2_2637_isoform_c2521_full_length_coverage_2_non_full_length_coverage_2_isoform_length_2637_11937 | PNF24626.1Metallophosphoesterase 1 | P:GO:0006506; C:GO:0016020; C:GO:0016021; F:GO:0016787 | |
| 2284\|_\|c17873_f2p2_2637_isoform_c17873_full_length_coverage_2_non_full_length_coverage_2_isoform_length_2637_22530 | ---NA--- | | |
| TRINITY_DN24979_c0_g1_i4_len_2915_58228.p1_type_complete_len_879 | XP_018014771.1 cyclin-dependent kinase 11B-like isoform X1 | | |

| | | | |
|---|---|---|---|
| 2287\|_\|c3708_f2p0_2636_isoform_c3708_full_length_coverage_2_non_full_length_coverage_0_isoform_length_2636_12320 | PNF43405.1Polypeptide N-acetylgalactosaminyltransferase 35A | C:GO:0016020 | |
| 2289\|_\|c2371_f3p0_2636_isoform_c2371_full_length_coverage_3_non_full_length_coverage_0_isoform_length_2636_20888 | PSN55948.1Golgi phosphoprotein 3 sauron | F:GO:0070273 | |
| TRINITY_DN28391_c0_g1_i1_len_379_136289.p1_type_internal_len_127 | ---NA--- | | |
| TRINITY_DN31398_c0_g1_i1_len_466_205347.p1_type_5prime_partial_len_126 | XP_018023298.1 CSC1-like protein 2 | | |
| TRINITY_DN24901_c0_g1_i1_len_380_105169.p1_type_internal_len_127 | ---NA--- | | |
| TRINITY_DN10634_c0_g1_i1_len_378_247343.p1_type_internal_len_127 | AEZ68611.1adenine nucleotide translocase | C:GO:0005743; C:GO:0016021; F:GO:0022857; P:GO:0055085 | |
| TRINITY_DN27898_c0_g1_i1_len_381_105126.p2_type_internal_len_127 | XP_014216560.1NFX1-type zinc finger-containing protein 1-like | F:GO:0003700; C:GO:0005634; P:GO:0006355; F:GO:0008270 | |
| TRINITY_DN1253_c0_g1_i1_len_381_105142.p1_type_internal_len_127 | ---NA--- | | |
| TRINITY_DN19137_c0_g1_i1_len_379_209085.p1_type_internal_len_127 | ---NA--- | | |
| TRINITY_DN11579_c0_g1_i2_len_380_42241.p1_type_internal_len_127 | ---NA--- | | |
| TRINITY_DN25378_c0_g1_i1_len_380_136217.p2_type_internal_len_127 | ---NA--- | | |
| TRINITY_DN11994_c0_g1_i2_len_379_105221.p1_type_internal_len_127 | ---NA--- | | |
| TRINITY_DN23371_c0_g1_i1_len_379_105229.p1_type_internal_len_127 | ---NA--- | | |
| TRINITY_DN10903_c0_g1_i1_len_380_42244.p2_type_internal_len_127 | ---NA--- | | |
| TRINITY_DN1807_c0_g1_i1_len_379_105231.p1_type_internal_len_127 | ---NA--- | | |

| | | | |
|---|---|---|---|
| TRINITY_DN29657_c0_g1_i1_len_379_247318.p1_type_5prime_partial_len_126 | XP_014224279.1bifunctional heparan sulfate N-deacetylase/N-sulfotransferase | F:GO:0016740 | |
| TRINITY_DN413_c0_g1_i1_len_378_247335.p1_type_internal_len_127 | XP_013774138.1beta-hexosaminidase subunit alpha-like | | |
| TRINITY_DN27284_c0_g1_i1_len_380_105205.p1_type_internal_len_127 | ---NA--- | | |
| TRINITY_DN31198_c0_g1_i1_len_382_105091.p1_type_internal_len_127 | XP_018017978.1 2-aminoethanethiol dioxygenase-like | F:GO:0016702; F:GO:0051213; P:GO:0055114 | |
| TRINITY_DN13861_c0_g1_i1_len_380_136221.p1_type_internal_len_127 | AWK58823.1Chibby | | |
| TRINITY_DN9618_c0_g1_i1_len_382_105095.p1_type_internal_len_127 | XP_018009970.1 DNA-directed RNA polymerase I subunit RPA1-like | F:GO:0016740 | |
| TRINITY_DN29348_c0_g1_i1_len_378_247363.p1_type_internal_len_127 | ---NA--- | | |
| TRINITY_DN17866_c0_g1_i1_len_830_34555.p1_type_5prime_partial_len_125 | ---NA--- | | |
| TRINITY_DN35193_c0_g1_i1_len_376_209236.p1_type_internal_len_126 | ---NA--- | | |
| TRINITY_DN13789_c0_g1_i1_len_740_199430.p2_type_5prime_partial_len_125 | ---NA--- | | |
| TRINITY_DN38168_c0_g1_i1_len_376_209227.p1_type_internal_len_126 | XP_015523621.1 dual specificity protein phosphatase 10-like isoform X2 | F:GO:0004725; F:GO:0008138; P:GO:0035335 | EC:3.1.3.16; EC:3.1.3.48 |
| TRINITY_DN15872_c0_g1_i2_len_398_11807.p1_type_5prime_partial_len_125 | ---NA--- | | |
| TRINITY_DN39864_c0_g1_i1_len_376_209228.p1_type_internal_len_126 | ---NA--- | | |
| TRINITY_DN18625_c0_g1_i1_len_1567_93147.p2_type_5prime_partial_len_125 | ---NA--- | | |
| TRINITY_DN31131_c0_g1_i2_len_375_209249.p1_type_internal_len_126 | ---NA--- | | |
| TRINITY_DN6901_c0_g1_i1_len_404_207835.p1_type_5prime_partial_len_125 | ---NA--- | | |

| | | | |
|---|---|---|---|
| TRINITY_DN19528_c0_g2_i4_len_405_41327.p1_type_5prime_partial_len_125 | ---NA--- | | |
| TRINITY_DN27454_c0_g1_i1_len_378_73279.p1_type_internal_len_126 | XP_007169839.1 elongation factor 1-alpha 1-like | F:GO:0003746; F:GO:0003924; F:GO:0005525; P:GO:0006414 | EC:3.6.1.15 |
| TRINITY_DN32104_c0_g1_i1_len_375_209251.p1_type_internal_len_126 | XP_015119985.1 transmembrane channel-like protein 7 isoform X2 | | |
| TRINITY_DN30186_c0_g1_i1_len_562_7627.p1_type_complete_len_125 | XP_018026160.1 multifunctional methyltransferase subunit TRM112-like protein | | |
| TRINITY_DN17254_c0_g1_i1_len_862_4394.p2_type_complete_len_125 | XP_026280262.1coiled-coil domain-containing protein 124 | | |
| TRINITY_DN20631_c0_g1_i1_len_1002_126529.p1_type_complete_len_125 | XP_018017318.1 brain protein I3-like | | |
| TRINITY_DN18693_c0_g1_i1_len_592_302474.p1_type_complete_len_125 | XP_018023668.1 INO80 complex subunit C-like | | |
| TRINITY_DN29939_c0_g2_i2_len_375_209245.p1_type_internal_len_126 | ---NA--- | | |
| TRINITY_DN12624_c0_g1_i1_len_387_208638.p1_type_5prime_partial_len_125 | ---NA--- | | |
| TRINITY_DN10282_c0_g1_i1_len_777_198897.p2_type_complete_len_125 | ---NA--- | | |
| TRINITY_DN12523_c0_g1_i1_len_386_208691.p1_type_5prime_partial_len_125 | ---NA--- | | |
| TRINITY_DN31352_c0_g1_i1_len_376_209194.p1_type_internal_len_126 | XP_018018489.1PREDICTED: uncharacterized protein LOC108675008 | P:GO:0006357 | |
| TRINITY_DN36861_c0_g1_i1_len_377_209175.p1_type_internal_len_126 | ---NA--- | | |
| TRINITY_DN13887_c0_g1_i1_len_377_209177.p1_type_internal_len_126 | ---NA--- | | |
| TRINITY_DN33454_c0_g1_i1_len_377_209182.p1_type_internal_len_126 | XP_024275640.1midasin-like isoform X2 | | |
| TRINITY_DN29469_c0_g1_i1_len_1605_157397.p2_type_complete_len_125 | ---NA--- | | |

| | | | |
|---|---|---|---|
| TRINITY_DN16006_c0_g1_i2_len_454_205809.p1_type_3prime_partial_len_126 | ---NA--- | | |
| TRINITY_DN25104_c1_g1_i5_len_1259_234305.p1_type_complete_len_125 | ---NA--- | | |
| TRINITY_DN18662_c0_g1_i1_len_636_301871.p1_type_complete_len_125 | ---NA--- | | |
| TRINITY_DN4143_c0_g1_i1_len_376_209206.p1_type_internal_len_126 | XP_014101131.1 putative polypeptide N-acetylgalactosaminyltransferase 9 | C:GO:0000139; P:GO:0006486; C:GO:0016021; F:GO:0016757; F:GO:0030246 | |
| TRINITY_DN23540_c0_g1_i2_len_376_209210.p1_type_internal_len_126 | XP_018009982.1PREDICTED: uncharacterized protein LOC108667470 isoform X1 | | |
| TRINITY_DN20138_c0_g1_i1_len_2001_156105.p3_type_3prime_partial_len_126 | ---NA--- | | |
| TRINITY_DN5738_c0_g1_i1_len_1147_298024.p3_type_3prime_partial_len_126 | ---NA--- | | |
| TRINITY_DN3306_c0_g1_i1_len_775_64823.p1_type_5prime_partial_len_125 | ---NA--- | | |
| TRINITY_DN9669_c0_g1_i1_len_735_199505.p1_type_3prime_partial_len_126 | XP_022102327.1dual specificity protein phosphatase 19-like isoform X1 | | |
| TRINITY_DN23330_c0_g1_i1_len_1276_194416.p2_type_complete_len_125 | ---NA--- | | |
| TRINITY_DN22879_c0_g1_i1_len_2887_154470.p2_type_complete_len_125 | ---NA--- | | |
| TRINITY_DN2992_c0_g1_i1_len_403_207880.p1_type_5prime_partial_len_125 | ---NA--- | | |
| TRINITY_DN22453_c0_g1_i1_len_1181_234821.p1_type_complete_len_125 | ---NA--- | | |
| TRINITY_DN25588_c0_g1_i1_len_1178_234838.p1_type_complete_len_125 | ---NA--- | | |
| TRINITY_DN12702_c0_g1_i2_len_489_243342.p2_type_5prime_partial_len_125 | ---NA--- | | |

| | | | |
|---|---|---|---|
| TRINITY_DN16912_c0_g1_i2_len_1229_32407.p3_type_complete_len_125 | ---NA--- | | |
| TRINITY_DN225_c0_g1_i1_len_379_73268.p1_type_internal_len_126 | XP_013379849.1kinesin-like protein KIF1C | F:GO:0003777; F:GO:0005524; C:GO:0005874; P:GO:0007018; F:GO:0008017 | EC:3.6.1.15 |
| TRINITY_DN3845_c0_g1_i1_len_375_209257.p1_type_internal_len_126 | ---NA--- | | |
| TRINITY_DN21225_c0_g1_i1_len_797_198658.p1_type_5prime_partial_len_125 | ---NA--- | | |
| TRINITY_DN35076_c0_g1_i1_len_455_244382.p1_type_3prime_partial_len_126 | XP_018007338.1 transmembrane protein 104-like | | |
| TRINITY_DN3619_c0_g1_i1_len_376_42404.p1_type_internal_len_126 | ---NA--- | | |
| TRINITY_DN3553_c0_g1_i1_len_377_247391.p1_type_internal_len_126 | ---NA--- | | |
| TRINITY_DN297_c0_g1_i1_len_421_207097.p1_type_5prime_partial_len_125 | XP_008313443.1carbohydrate sulfotransferase 11 | | |
| TRINITY_DN35225_c0_g1_i1_len_377_247402.p1_type_internal_len_126 | ---NA--- | | |
| TRINITY_DN15256_c0_g1_i2_len_377_247404.p1_type_internal_len_126 | ---NA--- | | |
| TRINITY_DN6720_c0_g1_i1_len_377_247408.p1_type_internal_len_126 | ---NA--- | | |
| TRINITY_DN15315_c0_g1_i1_len_573_202350.p2_type_5prime_partial_len_125 | ---NA--- | | |
| TRINITY_DN20522_c0_g1_i1_len_801_4850.p2_type_3prime_partial_len_126 | ---NA--- | | |
| TRINITY_DN25372_c0_g2_i1_len_377_247376.p1_type_internal_len_126 | ---NA--- | | |
| TRINITY_DN11688_c0_g1_i1_len_377_247379.p1_type_internal_len_126 | ---NA--- | | |
| TRINITY_DN15538_c0_g1_i1_len_377_247380.p1_type_internal_len_126 | ---NA--- | | |

| | | | |
|---|---|---|---|
| TRINITY_DN5186_c0_g1_i1_len_377_247426.p1_type_internal_len_126 | ---NA--- | | |
| TRINITY_DN28479_c0_g1_i1_len_376_247429.p1_type_internal_len_126 | XP_011254074.1protein draper isoform X2 | C:GO:0016020; C:GO:0016021 | |
| TRINITY_DN10508_c0_g1_i1_len_1075_195890.p2_type_5prime_partial_len_125 | XP_021003123.1discoidin domain-containing receptor 2-like | P:GO:0001952; F:GO:0005518; F:GO:0005524; C:GO:0005887; P:GO:0008284; P:GO:0018108; P:GO:0030154; F:GO:0038062; P:GO:0038063; C:GO:0043235; P:GO:1903053 | EC:2.7.10; EC:2.7.10.1; EC:2.7.11 |
| TRINITY_DN3824_c0_g1_i2_len_376_247432.p1_type_internal_len_126 | ---NA--- | | |
| TRINITY_DN17682_c0_g1_i2_len_572_202370.p1_type_5prime_partial_len_125 | ---NA--- | | |
| TRINITY_DN30893_c0_g1_i1_len_667_273190.p1_type_5prime_partial_len_125 | ---NA--- | | |
| TRINITY_DN16728_c0_g1_i1_len_1380_233621.p1_type_complete_len_125 | ---NA--- | | |
| TRINITY_DN34076_c0_g1_i1_len_377_247413.p1_type_internal_len_126 | ---NA--- | | |
| TRINITY_DN977_c0_g1_i1_len_377_247416.p1_type_internal_len_126 | ---NA--- | | |
| TRINITY_DN30825_c1_g2_i4_len_699_200023.p1_type_3prime_partial_len_126 | ---NA--- | | |
| TRINITY_DN20625_c0_g1_i1_len_1417_233424.p3_type_5prime_partial_len_125 | ---NA--- | | |
| TRINITY_DN34036_c0_g1_i1_len_524_203545.p1_type_3prime_partial_len_126 | ---NA--- | | |
| TRINITY_DN2014_c0_g1_i1_len_377_247422.p1_type_internal_len_126 | ---NA--- | | |
| TRINITY_DN6774_c0_g1_i1_len_379_247291.p1_type_internal_len_126 | ---NA--- | | |

<table>
<tr><td>TRINITY_DN21259_c0_g1_i1_len_379_247264.p1_type_internal_len_126</td><td>---NA---</td><td></td><td></td></tr>
<tr><td>TRINITY_DN27144_c0_g1_i1_len_378_247331.p1_type_internal_len_126</td><td>XP_018011792.1 transmembrane protein 164-like</td><td>C:GO:0016020</td><td></td></tr>
<tr><td>TRINITY_DN28118_c0_g1_i1_len_378_247337.p1_type_internal_len_126</td><td>XP_017064742.1 coronin-7 isoform X1</td><td>F:GO:0008092; P:GO:0016043; C:GO:0044444; C:GO:0099513</td><td></td></tr>
<tr><td>TRINITY_DN14601_c0_g1_i1_len_376_42422.p1_type_internal_len_126</td><td>KYP41059.1Transposon Ty3-G Gag-Pol polyprotein</td><td>F:GO:0003676</td><td></td></tr>
<tr><td>TRINITY_DN4862_c0_g1_i1_len_379_209081.p1_type_internal_len_126</td><td>---NA---</td><td></td><td></td></tr>
<tr><td>TRINITY_DN20980_c0_g1_i1_len_809_198502.p1_type_complete_len_125</td><td>---NA---</td><td></td><td></td></tr>
<tr><td>TRINITY_DN9264_c0_g1_i1_len_418_11107.p1_type_3prime_partial_len_126</td><td>---NA---</td><td></td><td></td></tr>
<tr><td>TRINITY_DN38087_c0_g1_i1_len_378_247324.p1_type_internal_len_126</td><td>XP_023707970.1glycosylated lysosomal membrane protein A-like isoform X1</td><td>C:GO:0016020; C:GO:0016021</td><td></td></tr>
<tr><td>TRINITY_DN35060_c0_g1_i1_len_378_247369.p1_type_internal_len_126</td><td>BAF64529.1peptidylglycine alpha-hydroxylating monooxygenase</td><td>F:GO:0005507; P:GO:0006518; C:GO:0016020; F:GO:0016715; P:GO:0055114</td><td>EC:1.14.17</td></tr>
<tr><td>TRINITY_DN23616_c0_g1_i4_len_1570_232687.p3_type_complete_len_125</td><td>---NA---</td><td></td><td></td></tr>
<tr><td>2290|_|c15274_f10p1_2635_isoform_c15274_full_length_coverage_10_non_full_length_coverage_1_isoform_length_2635_22300</td><td>XP_018007780.1 vacuolar protein sorting-associated protein 45-like</td><td></td><td></td></tr>
<tr><td>2291|_|c34105_f6p3_2634_isoform_c34105_full_length_coverage_6_non_full_length_coverage_3_isoform_length_2634_18685</td><td>XP_018009973.1 BRCA1-associated RING domain protein 1-like</td><td>P:GO:0016567; C:GO:0031436; P:GO:0043065</td><td></td></tr>
<tr><td>2292|_|c8164_f2p1_2634_isoform_c8164_full_length_coverage_2_non_full_length_coverage_1_isoform_length_2634_17188</td><td>PNF28796.1Non-canonical poly(A) RNA polymerase PAPD5</td><td>F:GO:0003723; F:GO:0004652; P:GO:0043631</td><td>EC:2.7.7.19</td></tr>
<tr><td>2293|_|c29310_f2p0_2633_isoform_c29310_full_length_coverage_2_non_full_length_coverage_0_isoform_length_2633_23125</td><td>XP_018018909.1PREDICTED: uncharacterized protein LOC108675412</td><td></td><td></td></tr>
</table>

| | | | |
|---|---|---|---|
| 2294\|_\|c8753_f2p4_2633_isoform_c8753_full_length_coverage_2_non_full_length_coverage_4_isoform_length_2633_12745 | XP_011554325.1 mitochondrial import inner membrane translocase subunit TIM50-C-like isoform X2 | C:GO:0005739; C:GO:0005743; C:GO:0005744; P:GO:0015031; C:GO:0016021 | |
| 2295\|_\|c18065_f2p2_2633_isoform_c18065_full_length_coverage_2_non_full_length_coverage_2_isoform_length_2633_13547 | ---NA--- | | |
| 2296\|_\|c5257_f2p0_2632_isoform_c5257_full_length_coverage_2_non_full_length_coverage_0_isoform_length_2632_21509 | ALC78849.1acid phosphatase | F:GO:0003993; P:GO:0016311; F:GO:0046872 | EC:3.1.3.2 |
| 2297\|_\|c2005_f3p0_2632_isoform_c2005_full_length_coverage_3_non_full_length_coverage_0_isoform_length_2632_16120 | XP_018011564.1 protein FAM136A-like isoform X2 | | |
| 2298\|_\|c1938_f2p16_2632_isoform_c1938_full_length_coverage_2_non_full_length_coverage_16_isoform_length_2632_28090 | ---NA--- | | |
| TRINITY_DN24309_c0_g1_i2_len_3251_228716.p1_type_complete_len_877 | EZA58100.1Putative homeodomain transcription factor | F:GO:0003677; C:GO:0016020; C:GO:0016021 | |
| TRINITY_DN27342_c0_g1_i1_len_378_247349.p1_type_internal_len_126 | ---NA--- | | |
| TRINITY_DN23374_c0_g1_i2_len_574_202333.p1_type_3prime_partial_len_126 | ---NA--- | | |
| TRINITY_DN16594_c0_g1_i1_len_1666_232257.p2_type_5prime_partial_len_125 | ---NA--- | | |
| TRINITY_DN29957_c0_g1_i1_len_4223_187470.p5_type_3prime_partial_len_126 | ---NA--- | | |
| TRINITY_DN37307_c0_g1_i1_len_378_247357.p1_type_internal_len_126 | XP_012939381.1 neurofibromin-like | | |
| TRINITY_DN29330_c0_g1_i1_len_378_247358.p1_type_internal_len_126 | ---NA--- | | |
| TRINITY_DN15668_c0_g1_i1_len_586_202078.p1_type_5prime_partial_len_125 | ---NA--- | | |
| TRINITY_DN16107_c0_g1_i1_len_1117_269535.p1_type_5prime_partial_len_125 | AOD27422.1presenilin enhancer 2 | C:GO:0016021 | |

| | | | |
|---|---|---|---|
| TRINITY_DN25395_c0_g1_i2_len_1020_196389.p2_type_complete_len_125 | XP_022323194.1uncharacterized protein LOC111124546 isoform X1 | | |
| TRINITY_DN6079_c0_g1_i1_len_378_209107.p1_type_internal_len_126 | ---NA--- | | |
| TRINITY_DN40061_c0_g1_i1_len_384_208808.p1_type_5prime_partial_len_125 | ---NA--- | | |
| TRINITY_DN1171_c0_g1_i1_len_397_246498.p1_type_5prime_partial_len_125 | ---NA--- | | |
| TRINITY_DN35228_c0_g1_i1_len_375_247526.p1_type_internal_len_126 | XP_018021461.1 Niemann-Pick C1 protein-like | C:GO:0016020; C:GO:0016021 | |
| TRINITY_DN2794_c0_g1_i1_len_378_307905.p2_type_internal_len_126 | ---NA--- | | |
| TRINITY_DN2488_c0_g1_i1_len_378_307898.p2_type_internal_len_126 | ---NA--- | | |
| TRINITY_DN41596_c0_g1_i1_len_377_209157.p1_type_internal_len_126 | ---NA--- | | |
| TRINITY_DN15414_c1_g1_i1_len_377_209163.p1_type_internal_len_126 | ---NA--- | | |
| TRINITY_DN35879_c0_g1_i1_len_377_209165.p1_type_internal_len_126 | KDR08121.1hypothetical protein L798_01654, partial | F:GO:0005089; P:GO:0035023 | |
| TRINITY_DN28482_c0_g1_i1_len_410_246022.p1_type_5prime_partial_len_125 | ---NA--- | | |
| TRINITY_DN17916_c0_g1_i1_len_799_300133.p2_type_5prime_partial_len_125 | ALV82310.1calcineurin B-like protein 1 | F:GO:0005509 | |
| TRINITY_DN35737_c0_g1_i1_len_377_209166.p1_type_internal_len_126 | AKI88007.1apoptosis signal-regulating kinase 1 | F:GO:0004672; F:GO:0005524; P:GO:0006468 | |
| TRINITY_DN16201_c0_g1_i1_len_951_33758.p1_type_complete_len_125 | ---NA--- | | |
| TRINITY_DN40718_c0_g1_i1_len_377_209172.p1_type_internal_len_126 | ---NA--- | | |
| TRINITY_DN15840_c0_g1_i1_len_405_246192.p1_type_5prime_partial_len_125 | XP_018007454.1 gastrula zinc finger protein XlCGF49.1-like | | |

| | | | |
|---|---|---|---|
| TRINITY_DN32177_c0_g1_i1_len_376_247465.p1_type_internal_len_126 | ---NA--- | | |
| TRINITY_DN36788_c0_g1_i1_len_376_247467.p1_type_internal_len_126 | XP_018006413.1 monoacylglycerol lipase ABHD12-like | C:GO:0016020 | |
| TRINITY_DN29088_c0_g1_i1_len_376_247473.p1_type_internal_len_126 | AAV48573.15-HT1 receptor | F:GO:0004930; P:GO:0007186; C:GO:0016021 | |
| TRINITY_DN29551_c0_g1_i1_len_377_42381.p1_type_internal_len_126 | ---NA--- | | |
| TRINITY_DN11066_c0_g1_i1_len_375_308054.p1_type_internal_len_126 | ---NA--- | | |
| TRINITY_DN26145_c0_g1_i1_len_390_246805.p1_type_5prime_partial_len_125 | ---NA--- | | |
| TRINITY_DN310_c0_g1_i1_len_375_308026.p1_type_internal_len_126 | XP_025829888.1DET1 homolog | | |
| TRINITY_DN17807_c0_g1_i1_len_1153_2808.p2_type_5prime_partial_len_125 | XP_018027675.1PREDICTED: uncharacterized protein LOC108682930 | | |
| TRINITY_DN36712_c0_g1_i1_len_376_247442.p1_type_internal_len_126 | XP_021923386.1vacuolar protein sorting-associated protein 13A-like isoform X4 | | |
| TRINITY_DN31258_c2_g1_i2_len_6700_186835.p2_type_complete_len_125 | ---NA--- | | |
| TRINITY_DN14107_c0_g1_i1_len_376_247443.p1_type_internal_len_126 | ---NA--- | | |
| TRINITY_DN11543_c0_g1_i1_len_376_247450.p1_type_internal_len_126 | ---NA--- | | |
| TRINITY_DN4009_c0_g1_i1_len_376_247457.p1_type_internal_len_126 | ---NA--- | | |
| TRINITY_DN29957_c0_g1_i1_len_376_247461.p1_type_internal_len_126 | ---NA--- | | |
| TRINITY_DN4511_c0_g1_i1_len_377_307957.p1_type_internal_len_126 | XP_018006365.1 ras GTPase-activating protein 3-like | P:GO:0035556 | |
| TRINITY_DN5892_c0_g1_i1_len_376_105359.p1_type_internal_len_126 | AEX08659.1lipopolysaccharide and beta-1,3-glucan binding protein | F:GO:0004553; P:GO:0005975 | |

| | | | |
|---|---|---|---|
| TRINITY_DN27980_c0_g1_i1_len_377_105302.p1_type_internal_len_126 | ---NA--- | | |
| TRINITY_DN28204_c0_g1_i1_len_377_105317.p1_type_internal_len_126 | ---NA--- | | |
| TRINITY_DN12380_c0_g1_i2_len_639_6429.p1_type_3prime_partial_len_126 | ---NA--- | | |
| TRINITY_DN19728_c0_g1_i2_len_947_236553.p2_type_3prime_partial_len_126 | ---NA--- | | |
| TRINITY_DN27227_c0_g1_i3_len_1562_192748.p3_type_5prime_partial_len_125 | ---NA--- | | |
| TRINITY_DN15091_c0_g1_i1_len_378_105274.p2_type_internal_len_126 | XP_003386140.1 radixin-like | F:GO:0003779; C:GO:0005856 | |
| TRINITY_DN12184_c0_g1_i1_len_543_166092.p1_type_5prime_partial_len_125 | ---NA--- | | |
| TRINITY_DN24272_c0_g1_i1_len_1581_60411.p2_type_5prime_partial_len_125 | ---NA--- | | |
| TRINITY_DN25092_c1_g1_i2_len_1842_59688.p2_type_complete_len_125 | ---NA--- | | |
| TRINITY_DN24099_c0_g1_i2_len_637_201059.p1_type_3prime_partial_len_126 | XP_019617495.1 betaine--homocysteine S-methyltransferase 1-like | F:GO:0008270; P:GO:0009086; P:GO:0032259; F:GO:0047150 | EC:2.1.1.5 |
| TRINITY_DN26933_c0_g1_i1_len_501_276025.p1_type_complete_len_125 | ---NA--- | | |
| TRINITY_DN18767_c0_g1_i1_len_1064_235619.p2_type_complete_len_125 | XP_018023119.1 zinc finger and BTB domain-containing protein 17-like isoform X1 | | |
| TRINITY_DN24577_c0_g1_i1_len_874_197766.p1_type_5prime_partial_len_125 | ---NA--- | | |
| TRINITY_DN6582_c0_g1_i1_len_555_131154.p1_type_5prime_partial_len_125 | ---NA--- | | |
| TRINITY_DN21047_c0_g1_i4_len_407_306760.p1_type_5prime_partial_len_125 | ---NA--- | | |
| TRINITY_DN19454_c0_g1_i2_len_915_96161.p2_type_complete_len_125 | ---NA--- | | |

| | | | |
|---|---|---|---|
| TRINITY_DN24076_c0_g1_i8_len_2508_229762.p3_type_complete_len_125 | ---NA--- | | |
| TRINITY_DN10639_c0_g1_i3_len_488_132660.p1_type_5prime_partial_len_125 | ---NA--- | | |
| TRINITY_DN21983_c0_g1_i1_len_2281_190003.p1_type_complete_len_125 | XP_018010488.1 mucin-5AC-like | C:GO:0005634; P:GO:0006357 | |
| TRINITY_DN30467_c0_g1_i5_len_3379_188049.p3_type_complete_len_125 | ---NA--- | | |
| TRINITY_DN26152_c0_g1_i1_len_468_167944.p1_type_3prime_partial_len_126 | XP_018577698.1GTP-binding protein Rit1-like | | |
| TRINITY_DN19520_c0_g1_i1_len_517_131960.p2_type_3prime_partial_len_126 | XP_018022788.1 methionine adenosyltransferase 2 subunit beta-like | F:GO:0043546; P:GO:0055114 | |
| TRINITY_DN3184_c0_g1_i1_len_454_168322.p1_type_5prime_partial_len_125 | ---NA--- | | |
| TRINITY_DN34439_c0_g1_i1_len_378_136313.p1_type_internal_len_126 | ACU31809.1alpha2 macroglobulin isoform 3 | F:GO:0004866; C:GO:0005615; P:GO:0010951 | |
| TRINITY_DN7113_c0_g1_i1_len_377_136343.p1_type_internal_len_126 | XP_018015220.1 protein Tob1-like | | |
| TRINITY_DN16375_c0_g1_i2_len_785_162555.p1_type_5prime_partial_len_125 | ---NA--- | | |
| TRINITY_DN13185_c0_g2_i2_len_615_240535.p1_type_5prime_partial_len_125 | XP_022256117.1huntingtin-interacting protein 1-like | F:GO:0005515 | |
| TRINITY_DN1393_c0_g1_i1_len_377_136357.p1_type_internal_len_126 | ---NA--- | | |
| TRINITY_DN18272_c0_g1_i2_len_528_8248.p3_type_complete_len_125 | ---NA--- | | |
| TRINITY_DN6912_c0_g1_i1_len_381_136207.p1_type_5prime_partial_len_125 | ---NA--- | | |
| TRINITY_DN23976_c0_g1_i1_len_4399_121823.p2_type_5prime_partial_len_125 | ---NA--- | | |
| TRINITY_DN5053_c0_g1_i1_len_401_135370.p1_type_5prime_partial_len_125 | ---NA--- | | |

| | | | |
|---|---|---|---|
| TRINITY_DN17390_c0_g2_i1_len_402_135317.p1_type_3prime_partial_len_126 | ASK05861.1chitin-binding protein | F:GO:0042302 | |
| TRINITY_DN1197_c0_g1_i1_len_931_236695.p2_type_complete_len_125 | XP_022286175.1serine/threonine-protein kinase ULK2-like isoform X3 | | |
| TRINITY_DN19075_c0_g1_i2_len_400_41488.p1_type_5prime_partial_len_125 | ---NA--- | | |
| TRINITY_DN19075_c0_g1_i2_len_400_41488.p2_type_5prime_partial_len_125 | ---NA--- | | |
| TRINITY_DN21198_c0_g1_i1_len_903_236925.p1_type_complete_len_125 | ---NA--- | | |
| TRINITY_DN28951_c0_g1_i1_len_611_164819.p1_type_complete_len_125 | ---NA--- | | |
| TRINITY_DN11505_c1_g1_i1_len_1548_192843.p4_type_5prime_partial_len_125 | XP_018023064.1 serine/threonine-protein kinase OSR1-like isoform X2 | | |
| TRINITY_DN2266_c0_g1_i1_len_375_136436.p1_type_internal_len_126 | ---NA--- | | |
| TRINITY_DN2813_c0_g1_i1_len_511_275786.p1_type_5prime_partial_len_125 | ---NA--- | | |
| TRINITY_DN2134_c0_g1_i1_len_447_206079.p1_type_5prime_partial_len_125 | XP_018027727.1PREDICTED: uncharacterized protein LOC108682971 | | |
| TRINITY_DN11749_c0_g1_i1_len_386_135970.p1_type_5prime_partial_len_125 | AXC43906.1glutathione S-transferase 1-like protein | F:GO:0004364 | EC:2.5.1.18 |
| TRINITY_DN18054_c0_g1_i1_len_867_237266.p2_type_3prime_partial_len_126 | ---NA--- | | |
| TRINITY_DN29229_c0_g1_i1_len_375_136477.p1_type_internal_len_126 | ---NA--- | | |
| TRINITY_DN15768_c0_g1_i3_len_692_163655.p2_type_5prime_partial_len_125 | ---NA--- | | |
| TRINITY_DN28097_c0_g1_i1_len_377_12645.p2_type_internal_len_126 | ---NA--- | | |
| TRINITY_DN4104_c0_g1_i1_len_505_242891.p1_type_3prime_partial_len_126 | XP_020289637.1UPF0183 protein CG7083 isoform X1 | | |

<table>
<tr><td>TRINITY_DN7703_c0_g1_i1_len_498_38727.p1_type_5prime_partial_len_125</td><td>XP_018027767.1 protein sprint-like</td><td>P:GO:0007165</td><td></td></tr>
<tr><td>TRINITY_DN10565_c0_g1_i1_len_378_171115.p1_type_internal_len_126</td><td>---NA---</td><td></td><td></td></tr>
<tr><td>TRINITY_DN31801_c0_g1_i1_len_378_171126.p1_type_internal_len_126</td><td>---NA---</td><td></td><td></td></tr>
<tr><td>TRINITY_DN7546_c0_g1_i1_len_375_73486.p1_type_internal_len_126</td><td>XP_023231960.1BTB/POZ domain-containing protein kctd15-like isoform X5</td><td>P:GO:0051260</td><td></td></tr>
<tr><td>TRINITY_DN1391_c0_g1_i1_len_377_12673.p2_type_internal_len_126</td><td>---NA---</td><td></td><td></td></tr>
<tr><td>TRINITY_DN3107_c0_g1_i1_len_377_73339.p1_type_internal_len_126</td><td>---NA---</td><td></td><td></td></tr>
<tr><td>TRINITY_DN10702_c0_g1_i1_len_376_73382.p1_type_internal_len_126</td><td>---NA---</td><td></td><td></td></tr>
<tr><td>TRINITY_DN1164_c0_g1_i1_len_376_73375.p2_type_internal_len_126</td><td>---NA---</td><td></td><td></td></tr>
<tr><td>TRINITY_DN18223_c0_g1_i1_len_377_42353.p1_type_internal_len_126</td><td>CAA66038.1proline rich protein</td><td>F:GO:0005199</td><td></td></tr>
<tr><td>TRINITY_DN35787_c0_g1_i1_len_378_73303.p1_type_internal_len_126</td><td>---NA---</td><td></td><td></td></tr>
<tr><td>TRINITY_DN4583_c0_g1_i1_len_378_73333.p2_type_internal_len_126</td><td>---NA---</td><td></td><td></td></tr>
<tr><td>TRINITY_DN18258_c0_g2_i1_len_378_73325.p1_type_internal_len_126</td><td>---NA---</td><td></td><td></td></tr>
<tr><td>2300|_|c2616_f7p2_2631_isoform_c2616_full_length_coverage_7_non_full_length_coverage_2_isoform_length_2631_12015</td><td>KDR08420.1putative elongator complex protein 2</td><td>P:GO:0002098; C:GO:0033588</td><td></td></tr>
<tr><td>TRINITY_DN23172_c0_g1_i6_len_2948_122147.p1_type_complete_len_877</td><td>XP_018016760.1PREDICTED: uncharacterized protein LOC108673437 isoform X3</td><td></td><td></td></tr>
<tr><td>2302|_|c17708_f3p1_2631_isoform_c17708_full_length_coverage_3_non_full_length_coverage_1_isoform_length_2631_22467</td><td>AAD38810.1ankyrin repeat-containing protein Asb-3</td><td>P:GO:0008150; P:GO:0016567; P:GO:0035556</td><td></td></tr>
</table>

| | | | |
|---|---|---|---|
| 2303\|_\|c2079_f5p2_2631_isoform_c2079_full_length_coverage_5_non_full_length_coverage_2_isoform_length_2631_16172 | XP_007902122.1 short/branched chain specific acyl-CoA dehydrogenase, mitochondrial | F:GO:0003995; C:GO:0005739; F:GO:0050660; P:GO:0055114 | EC:1.3.99.3 |
| TRINITY_DN26210_c0_g1_i7_len_3065_228921.p1_type_complete_len_877 | XP_025109287.1trimethylguanosine synthase-like | P:GO:0016070 | |
| TRINITY_DN27673_c0_g1_i2_len_3443_187996.p1_type_5prime_partial_len_877 | XP_013421684.1neuroblastoma-amplified sequence | | |
| 2308\|_\|c1725_f6p0_2630_isoform_c1725_full_length_coverage_6_non_full_length_coverage_0_isoform_length_2630_15959 | ---NA--- | | |
| 2309\|_\|c18064_f2p0_2629_isoform_c18064_full_length_coverage_2_non_full_length_coverage_0_isoform_length_2629_13546 | ---NA--- | | |
| 2311\|_\|c7088_f2p1_2629_isoform_c7088_full_length_coverage_2_non_full_length_coverage_1_isoform_length_2629_17109 | XP_013784519.1dehydrogenase/reductase SDR family member 12-like | | |
| 2312\|_\|c1950_f8p5_2628_isoform_c1950_full_length_coverage_8_non_full_length_coverage_5_isoform_length_2628_20624 | XP_012168548.1CCR4-NOT transcription complex subunit 6-like isoform X1 | | |
| TRINITY_DN26351_c0_g1_i1_len_377_171196.p1_type_internal_len_126 | KZS09319.1Alpha-1,3-mannosyl-glycoprotein 2-beta-N-acetylglucosaminyltransferase | F:GO:0003827; C:GO:0005794; P:GO:0006487 | EC:2.4.1.101 |
| TRINITY_DN21646_c0_g1_i2_len_453_205837.p1_type_5prime_partial_len_125 | ---NA--- | | |
| TRINITY_DN4706_c0_g1_i1_len_376_171231.p1_type_internal_len_126 | ---NA--- | | |
| TRINITY_DN8850_c0_g1_i2_len_377_171161.p1_type_internal_len_126 | ---NA--- | | |
| TRINITY_DN12178_c0_g1_i1_len_377_171162.p1_type_internal_len_126 | ---NA--- | | |
| TRINITY_DN25866_c0_g1_i1_len_377_171174.p1_type_internal_len_126 | AFP33455.1kinesin-like protein KIF3A | F:GO:0003777; F:GO:0005524; C:GO:0005874; P:GO:0007018; F:GO:0008017 | EC:3.6.1.15 |

| | | | |
|---|---|---|---|
| TRINITY_DN4615_c0_g1_i1_len_376_73410.p1_type_internal_len_126 | ---NA--- | | |
| TRINITY_DN29827_c0_g1_i1_len_378_12599.p1_type_5prime_partial_len_125 | ---NA--- | | |
| TRINITY_DN575_c0_g1_i1_len_376_171242.p1_type_internal_len_126 | XP_013810361.1 dimethylglycine dehydrogenase, mitochondrial-like | | |
| TRINITY_DN26941_c0_g1_i1_len_377_279888.p2_type_internal_len_126 | ---NA--- | | |
| TRINITY_DN5805_c0_g1_i1_len_376_279926.p2_type_3prime_partial_len_126 | ---NA--- | | |
| TRINITY_DN9534_c0_g1_i1_len_375_279935.p1_type_internal_len_126 | ---NA--- | | |
| TRINITY_DN4746_c0_g1_i1_len_664_6144.p1_type_5prime_partial_len_125 | ---NA--- | | |
| TRINITY_DN684_c0_g1_i1_len_537_242082.p1_type_3prime_partial_len_126 | ---NA--- | | |
| TRINITY_DN28940_c0_g1_i1_len_375_279942.p1_type_internal_len_126 | ---NA--- | | |
| TRINITY_DN4239_c0_g1_i1_len_377_279865.p1_type_internal_len_126 | XP_018011463.1 cytochrome P450 302a1, mitochondrial-like | | |
| TRINITY_DN4451_c0_g1_i1_len_568_130922.p1_type_complete_len_125 | ---NA--- | | |
| TRINITY_DN25408_c0_g1_i1_len_376_279922.p1_type_internal_len_126 | ANA78437.1dopamine beta-hydroxylase | F:GO:0003824; F:GO:0004497; F:GO:0005507; C:GO:0016020; C:GO:0016021; F:GO:0016715; P:GO:0055114 | |
| TRINITY_DN34025_c0_g1_i1_len_389_170659.p1_type_5prime_partial_len_125 | ---NA--- | | |
| TRINITY_DN19899_c0_g1_i1_len_374_12780.p1_type_internal_len_125 | ABB73282.1reverse transcriptase | F:GO:0003964; P:GO:0006278 | EC:2.7.7.49 |
| TRINITY_DN14664_c0_g1_i1_len_372_209386.p1_type_internal_len_125 | ---NA--- | | |

| | | | |
|---|---|---|---|
| TRINITY_DN2829_c0_g1_i1_len_375_209243.p1_type_internal_len_125 | ---NA--- | | |
| TRINITY_DN15914_c0_g1_i1_len_793_97089.p1_type_complete_len_124 | ---NA--- | | |
| TRINITY_DN17660_c0_g1_i1_len_917_270790.p1_type_5prime_partial_len_124 | ---NA--- | | |
| TRINITY_DN30090_c0_g1_i4_len_2168_190343.p2_type_5prime_partial_len_124 | XP_018025959.1 syntaxin-7-like isoform X1 | | |
| TRINITY_DN12464_c0_g1_i1_len_372_209419.p1_type_internal_len_125 | ---NA--- | | |
| TRINITY_DN29701_c0_g1_i2_len_2709_189022.p2_type_3prime_partial_len_125 | OQV11806.1putative Nucleolar protein 10 | | |
| TRINITY_DN23046_c0_g1_i1_len_537_203215.p1_type_5prime_partial_len_124 | ---NA--- | | |
| TRINITY_DN37059_c0_g1_i1_len_372_209428.p1_type_internal_len_125 | ---NA--- | | |
| TRINITY_DN30447_c1_g1_i1_len_4102_187529.p2_type_complete_len_124 | XP_022197742.1transmembrane protein 87A-like | C:GO:0016020 | |
| TRINITY_DN12541_c0_g1_i1_len_384_208810.p1_type_5prime_partial_len_124 | AWS21480.1stylicin 1 | | |
| TRINITY_DN15221_c0_g1_i1_len_835_271437.p2_type_complete_len_124 | XP_017344132.1 peptidyl-prolyl cis-trans isomerase CWC27 homolog isoform X3 | P:GO:0000413; F:GO:0003755; P:GO:0006457 | EC:5.2.1.8 |
| TRINITY_DN17882_c0_g1_i2_len_375_12743.p1_type_3prime_partial_len_125 | XP_018955047.1PREDICTED: uncharacterized protein LOC109084860 isoform X1 | F:GO:0005488 | |
| TRINITY_DN21863_c0_g1_i1_len_375_12740.p1_type_internal_len_125 | ---NA--- | | |
| TRINITY_DN25102_c0_g1_i2_len_4328_228011.p2_type_complete_len_124 | ---NA--- | | |
| TRINITY_DN39238_c0_g1_i1_len_376_209220.p1_type_internal_len_125 | XP_019868521.1 lachesin-like isoform X2 | | |
| TRINITY_DN35251_c0_g1_i1_len_376_209219.p1_type_internal_len_125 | ---NA--- | | |

| | | | |
|---|---|---|---|
| TRINITY_DN21910_c1_g1_i1_len_974_3752.p4_type_complete_len_124 | ---NA--- | | |
| TRINITY_DN24131_c0_g1_i1_len_7416_227498.p2_type_complete_len_124 | ---NA--- | | |
| TRINITY_DN5861_c0_g1_i1_len_373_209342.p1_type_internal_len_125 | XP_018020252.1 RNA-directed DNA polymerase from mobile element jockey-like | F:GO:0003676; F:GO:0003964; F:GO:0004523; P:GO:0006278; P:GO:0090502 | |
| TRINITY_DN25184_c0_g1_i1_len_374_12767.p1_type_internal_len_125 | XP_015610405.1neither inactivation nor afterpotential protein C isoform X2 | | |
| TRINITY_DN24521_c0_g1_i1_len_2025_190816.p3_type_5prime_partial_len_124 | ---NA--- | | |
| TRINITY_DN38400_c0_g1_i1_len_373_209337.p1_type_internal_len_125 | EAA06867.5AGAP000699-PA | | |
| TRINITY_DN28511_c0_g1_i2_len_2819_188825.p3_type_3prime_partial_len_125 | ---NA--- | | |
| TRINITY_DN41910_c0_g1_i1_len_373_209355.p1_type_internal_len_125 | XP_018022655.1 alpha-mannosidase 2-like isoform X1 | | |
| TRINITY_DN14507_c0_g1_i2_len_375_12750.p1_type_internal_len_125 | ---NA--- | | |
| TRINITY_DN37682_c0_g1_i1_len_373_209357.p1_type_internal_len_125 | ---NA--- | | |
| TRINITY_DN34162_c0_g1_i1_len_373_209352.p2_type_internal_len_125 | ---NA--- | | |
| TRINITY_DN35296_c0_g1_i1_len_373_209350.p1_type_internal_len_125 | XP_018024896.1 fat-like cadherin-related tumor suppressor homolog | | |
| TRINITY_DN8615_c0_g1_i1_len_373_209351.p1_type_internal_len_125 | ---NA--- | | |
| TRINITY_DN26693_c0_g1_i2_len_1985_190957.p2_type_complete_len_124 | ---NA--- | | |
| TRINITY_DN32718_c0_g1_i1_len_374_209287.p2_type_internal_len_125 | XP_018566738.1plexin-B | | |

| | | | |
|---|---|---|---|
| TRINITY_DN32901_c0_g1_i1_len_374_209285.p1_type_3prime_partial_len_125 | AFJ11391.1cytosolic phospholipase A2 | F:GO:0004623; C:GO:0005737; P:GO:0009395; F:GO:0046872; F:GO:0102567; F:GO:0102568 | EC:3.1.1.1; EC:3.1.1.4 |
| TRINITY_DN41797_c0_g1_i1_len_375_209277.p1_type_internal_len_125 | XP_016366618.1 acid ceramidase-like | | |
| TRINITY_DN14461_c0_g1_i1_len_373_209373.p1_type_internal_len_125 | ---NA--- | | |
| TRINITY_DN29723_c0_g1_i1_len_374_12779.p2_type_internal_len_125 | XP_016854744.1 protein unc-13 homolog A | P:GO:0007268; F:GO:0019992 | |
| TRINITY_DN5530_c0_g1_i1_len_375_209252.p1_type_internal_len_125 | ---NA--- | | |
| TRINITY_DN39055_c0_g1_i1_len_374_209311.p2_type_internal_len_125 | ---NA--- | | |
| TRINITY_DN6087_c0_g1_i1_len_374_209306.p1_type_internal_len_125 | ---NA--- | | |
| TRINITY_DN20968_c0_g2_i1_len_658_98577.p1_type_3prime_partial_len_125 | ---NA--- | | |
| TRINITY_DN38802_c0_g1_i1_len_374_209302.p1_type_internal_len_125 | ---NA--- | | |
| TRINITY_DN5130_c0_g1_i1_len_374_209301.p1_type_5prime_partial_len_124 | ---NA--- | | |
| TRINITY_DN14205_c0_g1_i1_len_374_209290.p1_type_internal_len_125 | ---NA--- | | |
| TRINITY_DN8688_c0_g1_i1_len_374_209295.p1_type_internal_len_125 | ---NA--- | | |
| TRINITY_DN15100_c0_g1_i1_len_2296_189961.p2_type_5prime_partial_len_124 | ---NA--- | | |
| TRINITY_DN20026_c0_g1_i2_len_1728_123658.p3_type_5prime_partial_len_124 | ---NA--- | | |
| TRINITY_DN4708_c0_g1_i1_len_385_208759.p1_type_5prime_partial_len_124 | ---NA--- | | |
| TRINITY_DN37992_c0_g1_i1_len_385_208758.p1_type_5prime_partial_len_124 | ---NA--- | | |

| | | | |
|---|---|---|---|
| TRINITY_DN1059_c0_g1_i1_len_376_42417.p1_type_internal_len_125 | ---NA--- | | |
| TRINITY_DN19694_c0_g1_i1_len_752_199258.p1_type_5prime_partial_len_124 | ---NA--- | | |
| TRINITY_DN23842_c0_g1_i1_len_372_12858.p1_type_internal_len_125 | ---NA--- | | |
| TRINITY_DN15153_c0_g1_i1_len_803_271735.p1_type_5prime_partial_len_124 | ---NA--- | | |
| TRINITY_DN23974_c0_g1_i1_len_373_12808.p1_type_5prime_partial_len_124 | ---NA--- | | |
| TRINITY_DN2576_c0_g1_i1_len_374_42467.p2_type_internal_len_125 | ---NA--- | | |
| TRINITY_DN38119_c0_g1_i1_len_389_208536.p1_type_3prime_partial_len_125 | ---NA--- | | |
| TRINITY_DN8889_c0_g1_i1_len_374_42462.p1_type_internal_len_125 | ---NA--- | | |
| TRINITY_DN13022_c0_g1_i1_len_373_12815.p1_type_internal_len_125 | ---NA--- | | |
| TRINITY_DN20797_c0_g1_i1_len_553_202797.p2_type_5prime_partial_len_124 | ---NA--- | | |
| TRINITY_DN9216_c0_g1_i1_len_374_12794.p1_type_internal_len_125 | XP_018019937.1 flotillin-2-like isoform X3 | | |
| TRINITY_DN16763_c0_g1_i2_len_594_37056.p2_type_5prime_partial_len_124 | ---NA--- | | |
| TRINITY_DN25984_c0_g1_i2_len_1741_191900.p2_type_complete_len_124 | ---NA--- | | |
| TRINITY_DN24795_c0_g1_i3_len_1840_191514.p2_type_complete_len_124 | KZC09929.1GSK3-beta interaction protein | F:GO:0051018; P:GO:0060828 | |
| TRINITY_DN9608_c0_g1_i1_len_374_42457.p1_type_internal_len_125 | ---NA--- | | |
| TRINITY_DN25823_c0_g1_i2_len_3299_228657.p2_type_5prime_partial_len_124 | ---NA--- | | |

| | | | |
|---|---|---|---|
| TRINITY_DN5098_c0_g1_i1_len_375_42449.p1_type_internal_len_125 | XP_025829888.1DET1 homolog | | |
| TRINITY_DN10248_c0_g1_i1_len_609_6853.p2_type_5prime_partial_len_124 | ---NA--- | | |
| TRINITY_DN27258_c0_g1_i1_len_373_105501.p1_type_internal_len_125 | ---NA--- | | |
| TRINITY_DN29145_c0_g1_i1_len_373_105491.p2_type_internal_len_125 | XP_018009676.1 beta,beta-carotene 15,15'-dioxygenase-like isoform X2 | F:GO:0004497; F:GO:0016702; F:GO:0046872; P:GO:0055114 | |
| TRINITY_DN4535_c0_g1_i1_len_374_105441.p1_type_internal_len_125 | XP_018010562.1 vacuolar protein sorting-associated protein 13D-like | | |
| TRINITY_DN20921_c0_g1_i4_len_373_136563.p1_type_internal_len_125 | EDO36870.1predicted protein | | |
| TRINITY_DN21782_c1_g1_i1_len_373_136550.p1_type_internal_len_125 | ABC33914.1transglutaminase | F:GO:0003810; P:GO:0018149; F:GO:0046872 | EC:2.3.2.13 |
| TRINITY_DN3905_c0_g1_i1_len_373_136546.p1_type_internal_len_125 | ---NA--- | | |
| TRINITY_DN26653_c0_g1_i1_len_374_136518.p1_type_internal_len_125 | ---NA--- | | |
| TRINITY_DN17402_c0_g1_i3_len_2381_265615.p2_type_5prime_partial_len_124 | ---NA--- | | |
| TRINITY_DN32363_c0_g1_i1_len_373_136574.p1_type_internal_len_125 | XP_018016421.1 four and a half LIM domains protein 2-like isoform X3 | | |
| TRINITY_DN11536_c0_g1_i1_len_373_136565.p1_type_internal_len_125 | ---NA--- | | |
| TRINITY_DN26731_c0_g1_i1_len_374_280006.p1_type_internal_len_125 | ---NA--- | | |
| TRINITY_DN9108_c0_g1_i1_len_375_279947.p1_type_internal_len_125 | XP_023224634.1oxysterol-binding protein-related protein 3-like | | |
| TRINITY_DN21764_c0_g2_i1_len_372_105540.p1_type_internal_len_125 | ---NA--- | | |
| TRINITY_DN13259_c0_g1_i1_len_373_105532.p1_type_internal_len_125 | ---NA--- | | |

| | | | |
|---|---|---|---|
| 2313\|_\|c2459_f4p5_2628_isoform_c2459_full_length_coverage_4_non_full_length_coverage_5_isoform_length_2628_16428 | XP_018008603.1 zinc finger protein 184-like isoform X2 | | |
| TRINITY_DN30419_c0_g1_i2_len_4974_187135.p1_type_complete_len_876 | XP_018026993.1 N-alpha-acetyltransferase 15, NatA auxiliary subunit-like | | |
| TRINITY_DN22689_c0_g1_i3_len_3248_228717.p1_type_complete_len_876 | XP_018008283.1 katanin p80 WD40 repeat-containing subunit B1-like isoform X1 | C:GO:0015630; C:GO:0044430 | |
| 2316\|_\|c47178_f3p1_2627_isoform_c47178_full_length_coverage_3_non_full_length_coverage_1_isoform_length_2627_24014 | XP_018013260.1 GDP-D-glucose phosphorylase 1-like | | |
| 2317\|_\|c48226_f3p2_2627_isoform_c48226_full_length_coverage_3_non_full_length_coverage_2_isoform_length_2627_15751 | XP_006817934.1 spore wall protein 2-like | | |
| 2318\|_\|c15464_f16p8_2627_isoform_c15464_full_length_coverage_16_non_full_length_coverage_8_isoform_length_2627_13242 | XP_023703478.1zinc finger protein 367-like | F:GO:0003676 | |
| 2321\|_\|c5744_f2p2_2626_isoform_c5744_full_length_coverage_2_non_full_length_coverage_2_isoform_length_2626_21551 | XP_026462167.1 cold shock domain-containing protein E1-like | | |
| 2322\|_\|c2648_f3p1_2626_isoform_c2648_full_length_coverage_3_non_full_length_coverage_1_isoform_length_2626_12039 | XP_018016237.1 ceramide synthase 6-like | | |
| 2323\|_\|c4775_f2p10_2626_isoform_c4775_full_length_coverage_2_non_full_length_coverage_10_isoform_length_2626_28366 | ---NA--- | | |
| 2324\|_\|c9915_f3p0_2625_isoform_c9915_full_length_coverage_3_non_full_length_coverage_0_isoform_length_2625_21903 | XP_018021171.1 proline dehydrogenase 1, mitochondrial-like | F:GO:0004657; P:GO:0006562; P:GO:0055114 | EC:1.5.99.8 |
| TRINITY_DN25201_c0_g1_i1_len_910_197384.p2_type_5prime_partial_len_124 | AXP33454.1selenium-dependent glutathione peroxidase | F:GO:0004602; P:GO:0006979; P:GO:0055114; P:GO:0098869 | EC:1.11.1.9; EC:1.11.1.7 |
| TRINITY_DN13927_c0_g1_i1_len_555_303083.p1_type_5prime_partial_len_124 | ---NA--- | | |

| | | | |
|---|---|---|---|
| TRINITY_DN11232_c0_g1_i1_len_912_197366.p1_type_5prime_partial_len_124 | ---NA--- | | |
| TRINITY_DN16225_c0_g1_i1_len_548_241813.p1_type_5prime_partial_len_124 | AJC52502.1estrogen sulfotransferase | F:GO:0008146; F:GO:0016740 | |
| TRINITY_DN25348_c0_g1_i1_len_447_305466.p1_type_5prime_partial_len_124 | ---NA--- | | |
| TRINITY_DN20314_c0_g1_i1_len_439_206354.p1_type_5prime_partial_len_124 | ---NA--- | | |
| TRINITY_DN27365_c0_g1_i11_len_1232_194734.p1_type_5prime_partial_len_124 | ---NA--- | | |
| TRINITY_DN13286_c0_g1_i1_len_845_237486.p1_type_5prime_partial_len_124 | ---NA--- | | |
| TRINITY_DN28752_c0_g1_i2_len_682_163780.p1_type_5prime_partial_len_124 | ---NA--- | | |
| TRINITY_DN14585_c0_g1_i1_len_874_237189.p3_type_5prime_partial_len_124 | ---NA--- | | |
| TRINITY_DN38972_c0_g1_i1_len_433_206597.p1_type_3prime_partial_len_125 | XP_018006918.1 homer protein homolog 2-like | | |
| TRINITY_DN11341_c0_g1_i1_len_542_166112.p1_type_complete_len_124 | ACC86067.1anti-lipopolysaccharide factor | | |
| TRINITY_DN510_c0_g1_i1_len_520_166620.p1_type_complete_len_124 | ---NA--- | | |
| TRINITY_DN36460_c0_g1_i1_len_433_206580.p1_type_5prime_partial_len_124 | ---NA--- | | |
| TRINITY_DN27728_c0_g1_i1_len_375_136432.p1_type_internal_len_125 | XP_018023308.1 sodium channel protein 60E-like | F:GO:0005272; C:GO:0005886; P:GO:0006814; C:GO:0016021; P:GO:0034220 | |
| TRINITY_DN33830_c0_g1_i1_len_375_136425.p1_type_internal_len_125 | ---NA--- | | |
| TRINITY_DN13470_c0_g1_i1_len_516_275670.p2_type_5prime_partial_len_124 | ---NA--- | | |
| TRINITY_DN666_c0_g1_i1_len_374_136488.p2_type_internal_len_125 | ---NA--- | | |

| | | | |
|---|---|---|---|
| TRINITY_DN2917_c0_g1_i1_len_375_136460.p1_type_internal_len_125 | AAB03106.1cytochrome P450 | F:GO:0005488; C:GO:0016020; F:GO:0016491; C:GO:0043231 | |
| TRINITY_DN16100_c0_g1_i1_len_653_200759.p1_type_complete_len_124 | ---NA--- | | |
| TRINITY_DN15831_c0_g1_i1_len_730_238758.p1_type_5prime_partial_len_124 | ---NA--- | | |
| TRINITY_DN39776_c0_g1_i1_len_442_206265.p2_type_5prime_partial_len_124 | ---NA--- | | |
| TRINITY_DN15059_c0_g1_i1_len_750_162932.p1_type_5prime_partial_len_124 | ---NA--- | | |
| TRINITY_DN26758_c0_g1_i1_len_803_237918.p1_type_5prime_partial_len_124 | ---NA--- | | |
| TRINITY_DN9209_c0_g1_i1_len_1013_160561.p2_type_5prime_partial_len_124 | ---NA--- | | |
| TRINITY_DN15257_c0_g1_i1_len_994_160709.p2_type_5prime_partial_len_124 | EDS37545.1microsomal dipeptidase | C:GO:0016020; F:GO:0070011 | |
| TRINITY_DN26896_c0_g1_i1_len_660_239783.p1_type_5prime_partial_len_124 | ---NA--- | | |
| TRINITY_DN23033_c0_g1_i2_len_1158_62191.p2_type_complete_len_124 | ---NA--- | | |
| TRINITY_DN9156_c0_g1_i1_len_381_136188.p1_type_5prime_partial_len_124 | AYE56137.1thioredoxin-interacting protein | | |
| TRINITY_DN13853_c0_g1_i2_len_374_42482.p1_type_internal_len_125 | ---NA--- | | |
| TRINITY_DN2294_c0_g1_i1_len_373_42510.p1_type_internal_len_125 | ---NA--- | | |
| TRINITY_DN26247_c0_g1_i2_len_471_205185.p1_type_3prime_partial_len_125 | ---NA--- | | |
| TRINITY_DN17604_c0_g1_i1_len_1006_196505.p1_type_5prime_partial_len_124 | ---NA--- | | |
| TRINITY_DN3366_c0_g1_i1_len_372_42552.p1_type_internal_len_125 | ---NA--- | | |

| | | | |
|---|---|---|---|
| TRINITY_DN14533_c0_g1_i1_len_374_42499.p1_type_internal_len_125 | ---NA--- | | |
| TRINITY_DN27901_c0_g1_i1_len_373_42505.p1_type_internal_len_125 | ---NA--- | | |
| TRINITY_DN2457_c0_g1_i1_len_372_247676.p2_type_internal_len_125 | XP_018021062.1 longitudinals lacking protein, isoforms A/B/D/L-like | | |
| TRINITY_DN33240_c0_g1_i1_len_372_247664.p1_type_internal_len_125 | ---NA--- | | |
| TRINITY_DN15073_c0_g1_i1_len_372_247659.p1_type_internal_len_125 | ---NA--- | | |
| TRINITY_DN38043_c0_g1_i1_len_372_247655.p1_type_internal_len_125 | ---NA--- | | |
| TRINITY_DN35199_c0_g1_i1_len_372_247644.p1_type_internal_len_125 | ACU31810.1alpha2 macroglobulin isoform 2 | F:GO:0004866; C:GO:0005615; P:GO:0010951; C:GO:0016020 | |
| TRINITY_DN5509_c0_g1_i1_len_372_247643.p1_type_internal_len_125 | PRD20967.1zinc finger protein | F:GO:0003676 | |
| TRINITY_DN4052_c0_g1_i1_len_372_247635.p1_type_internal_len_125 | ---NA--- | | |
| TRINITY_DN34484_c0_g1_i1_len_373_247621.p1_type_internal_len_125 | ---NA--- | | |
| TRINITY_DN26801_c0_g1_i1_len_373_247609.p2_type_internal_len_125 | ABP97431.1alpha 2 macroglobulin | F:GO:0004866; C:GO:0005615; P:GO:0010951 | |
| TRINITY_DN33172_c0_g1_i1_len_373_247605.p1_type_internal_len_125 | ---NA--- | | |
| TRINITY_DN32437_c0_g1_i1_len_373_247600.p1_type_internal_len_125 | ---NA--- | | |
| TRINITY_DN29184_c0_g1_i1_len_373_247598.p1_type_internal_len_125 | ---NA--- | | |
| TRINITY_DN13016_c0_g1_i1_len_578_202254.p1_type_5prime_partial_len_124 | EEC13186.1RAS-related protein, putative | F:GO:0003924; F:GO:0004767; F:GO:0005525; C:GO:0005768; P:GO:0006886; P:GO:0030100; P:GO:0032482 | EC:3.1.4.12; EC:3.6.1.15 |

| | | | |
|---|---|---|---|
| TRINITY_DN17069_c0_g1_i1_len_1108_195641.p1_type_complete_len_124 | ---NA--- | | |
| TRINITY_DN11213_c0_g1_i1_len_444_277536.p1_type_5prime_partial_len_124 | ---NA--- | | |
| TRINITY_DN31994_c0_g1_i1_len_375_247505.p1_type_internal_len_125 | XP_023312978.1E3 ubiquitin-protein ligase Ubr3-like | P:GO:0016567; F:GO:0061630; P:GO:0071596 | |
| TRINITY_DN36948_c0_g1_i1_len_376_247434.p1_type_internal_len_125 | XP_022239564.1microtubule-actin cross-linking factor 1-like isoform X10 | | |
| TRINITY_DN11275_c0_g1_i1_len_373_247583.p2_type_internal_len_125 | XP_023712837.1tricalbin-3-like | | |
| TRINITY_DN33212_c0_g1_i1_len_373_247580.p1_type_internal_len_125 | XP_018017090.1 GPI mannosyltransferase 4-like | | |
| TRINITY_DN8129_c0_g1_i1_len_374_247579.p1_type_internal_len_125 | ---NA--- | | |
| TRINITY_DN33601_c0_g1_i1_len_374_247566.p2_type_internal_len_125 | XP_009325046.1 meiotic recombination protein SPO11 | | |
| TRINITY_DN30388_c0_g1_i1_len_374_247556.p1_type_internal_len_125 | ---NA--- | | |
| TRINITY_DN6852_c0_g1_i1_len_374_247550.p2_type_internal_len_125 | XP_018027561.1 metal transporter CNNM4-like | | |
| TRINITY_DN37710_c0_g1_i1_len_374_247549.p1_type_internal_len_125 | XP_013780462.1prolow-density lipoprotein receptor-related protein 1-like | F:GO:0005509; C:GO:0016020; C:GO:0016021 | |
| TRINITY_DN15912_c0_g1_i1_len_374_247539.p1_type_internal_len_125 | ---NA--- | | |
| TRINITY_DN30955_c0_g1_i1_len_374_247538.p1_type_internal_len_125 | ---NA--- | | |
| TRINITY_DN10007_c0_g1_i1_len_375_247531.p1_type_internal_len_125 | ---NA--- | | |
| TRINITY_DN14391_c0_g1_i1_len_374_73493.p1_type_internal_len_125 | ---NA--- | | |
| TRINITY_DN35875_c0_g1_i1_len_374_73521.p1_type_internal_len_125 | ---NA--- | | |

| | | | |
|---|---|---|---|
| TRINITY_DN8801_c0_g1_i2_len_507_242854.p1_type_5prime_partial_len_124 | ---NA--- | | |
| TRINITY_DN27750_c0_g1_i1_len_373_73546.p1_type_internal_len_125 | ---NA--- | | |
| TRINITY_DN22579_c0_g1_i6_len_1226_2528.p1_type_complete_len_124 | ---NA--- | | |
| TRINITY_DN32797_c0_g1_i1_len_376_73422.p1_type_internal_len_125 | XP_018028145.1 fibrillin-1-like isoform X3 | | |
| TRINITY_DN996_c0_g1_i1_len_497_276099.p1_type_3prime_partial_len_125 | ---NA--- | | |
| TRINITY_DN21117_c0_g1_i2_len_1346_93854.p2_type_5prime_partial_len_124 | ---NA--- | | |
| TRINITY_DN28845_c0_g1_i1_len_373_73575.p1_type_internal_len_125 | ---NA--- | | |
| TRINITY_DN17765_c0_g1_i1_len_372_73599.p1_type_internal_len_125 | ---NA--- | | |
| TRINITY_DN13836_c0_g1_i1_len_510_242776.p1_type_3prime_partial_len_125 | ---NA--- | | |
| TRINITY_DN10577_c0_g1_i1_len_576_7394.p1_type_5prime_partial_len_124 | XP_013771843.1double-stranded RNA-binding protein Staufen homolog 1-like | C:GO:0016020 | |
| TRINITY_DN15863_c0_g1_i1_len_959_196908.p1_type_5prime_partial_len_124 | AAD45201.1prophenoloxidase | F:GO:0016491; F:GO:0046872; P:GO:0055114 | |
| TRINITY_DN32044_c0_g1_i1_len_433_245144.p1_type_5prime_partial_len_124 | ---NA--- | | |
| TRINITY_DN22042_c0_g1_i1_len_372_42577.p1_type_internal_len_125 | ---NA--- | | |
| TRINITY_DN38213_c0_g1_i1_len_602_201734.p1_type_5prime_partial_len_124 | ---NA--- | | |
| TRINITY_DN12156_c0_g1_i1_len_399_278990.p1_type_5prime_partial_len_124 | ---NA--- | | |
| TRINITY_DN2590_c0_g1_i2_len_394_279193.p1_type_5prime_partial_len_124 | ---NA--- | | |

| | | | |
|---|---|---|---|
| TRINITY_DN9241_c0_g1_i1_len_383_73058.p1_type_5prime_partial_len_124 | ---NA--- | | |
| TRINITY_DN28321_c0_g1_i1_len_1288_194329.p1_type_complete_len_124 | ---NA--- | | |
| TRINITY_DN14932_c0_g1_i1_len_1257_234322.p1_type_complete_len_124 | ---NA--- | | |
| TRINITY_DN17204_c0_g1_i1_len_2051_92060.p2_type_5prime_partial_len_124 | ---NA--- | | |
| TRINITY_DN6162_c0_g1_i1_len_375_308033.p1_type_internal_len_125 | ---NA--- | | |
| TRINITY_DN31880_c0_g1_i1_len_376_171230.p1_type_internal_len_125 | XP_012232426.1 myotubularin-related protein 2 isoform X2 | | |
| TRINITY_DN26718_c0_g1_i1_len_375_171266.p1_type_internal_len_125 | AXY87731.1carnitine O-palmitoyltransferase 1b | F:GO:0016746 | |
| TRINITY_DN32550_c0_g1_i1_len_374_171302.p1_type_internal_len_125 | ---NA--- | | |
| TRINITY_DN22914_c0_g1_i1_len_1128_235197.p2_type_complete_len_124 | ---NA--- | | |
| TRINITY_DN7111_c0_g1_i1_len_374_171321.p1_type_internal_len_125 | ---NA--- | | |
| TRINITY_DN3820_c0_g1_i1_len_373_171354.p1_type_internal_len_125 | BAG71401.1beta tubulin | F:GO:0003924; F:GO:0005200; F:GO:0005525; C:GO:0005874; P:GO:0007010; P:GO:0007017 | EC:3.6.1.15 |
| TRINITY_DN26272_c0_g1_i1_len_1644_1356.p2_type_3prime_partial_len_125 | ---NA--- | | |
| TRINITY_DN13574_c0_g1_i1_len_670_273145.p2_type_5prime_partial_len_124 | ---NA--- | | |
| TRINITY_DN4173_c0_g1_i2_len_552_100253.p1_type_5prime_partial_len_124 | ---NA--- | | |
| TRINITY_DN22382_c0_g1_i3_len_1494_1670.p2_type_5prime_partial_len_124 | ---NA--- | | |
| TRINITY_DN29829_c0_g1_i1_len_372_308162.p1_type_internal_len_125 | ---NA--- | | |

| | | | |
|---|---|---|---|
| TRINITY_DN8592_c0_g1_i2_len_513_101001.p2_type_5prime_partial_len_124 | ---NA--- | | |
| TRINITY_DN6715_c0_g1_i1_len_372_308191.p1_type_internal_len_125 | ---NA--- | | |
| TRINITY_DN31666_c0_g1_i1_len_374_308078.p1_type_internal_len_125 | KYQ49024.1DNA-binding protein D-ETS-4 | F:GO:0003700; C:GO:0005634; P:GO:0006355; F:GO:0043565 | |
| 2325\|_\|c2061_f4p9_2625_isoform_c2061_full_length_coverage_4_non_full_length_coverage_9_isoform_length_2625_11626 | AAU12181.1G protein s alpha subunit | F:GO:0003924; F:GO:0005525; P:GO:0007186; F:GO:0031683; F:GO:0046872 | EC:3.6.1.15 |
| 2327\|_\|c2806_f3p0_2625_isoform_c2806_full_length_coverage_3_non_full_length_coverage_0_isoform_length_2625_12178 | ---NA--- | | |
| 2328\|_\|c8693_f4p2_2625_isoform_c8693_full_length_coverage_4_non_full_length_coverage_2_isoform_length_2625_12743 | PNF37124.1Zinc finger protein 76 | F:GO:0003676 | |
| 2329\|_\|c27528_f2p0_2625_isoform_c27528_full_length_coverage_2_non_full_length_coverage_0_isoform_length_2625_18434 | XP_013400767.1folliculin | F:GO:0005085 | |
| 2330\|_\|c5922_f2p1_2625_isoform_c5922_full_length_coverage_2_non_full_length_coverage_1_isoform_length_2625_21567 | XP_018021245.1 UV-stimulated scaffold protein A-like | | |
| TRINITY_DN29922_c0_g1_i1_len_3257_188183.p1_type_complete_len_875 | XP_021950434.1integral membrane protein GPR155-like | C:GO:0016020; C:GO:0016021; P:GO:0035556; P:GO:0055085 | |
| TRINITY_DN29372_c0_g1_i3_len_3687_187780.p1_type_5prime_partial_len_875 | XP_018011570.1 mediator of RNA polymerase II transcription subunit 16-like | C:GO:0005634; C:GO:0016592 | |
| TRINITY_DN25995_c0_g1_i1_len_3067_228918.p1_type_5prime_partial_len_875 | XP_016911621.1 type I inositol 3,4-bisphosphate 4-phosphatase isoform X4 | | |
| TRINITY_DN22837_c0_g1_i1_len_3138_58081.p1_type_5prime_partial_len_875 | ANJ04737.1glycoprotein 94 | F:GO:0005524; P:GO:0006457; F:GO:0051082 | |
| 2336\|_\|c3131_f2p3_2624_isoform_c3131_full_length_coverage_2_non_full_length_coverage_3_isoform_length_2624_12269 | XP_018009792.1 E3 ubiquitin-protein ligase TRIP12-like | | |

| | | | |
|---|---|---|---|
| TRINITY_DN1854_c0_g1_i1_len_374_308082.p1_type_internal_len_125 | ---NA--- | | |
| TRINITY_DN27624_c0_g1_i1_len_374_308100.p1_type_internal_len_125 | ---NA--- | | |
| TRINITY_DN15167_c0_g1_i1_len_373_308127.p1_type_internal_len_125 | ACL36280.1Kazal-type serine proteinase inhibitor 1 | | |
| TRINITY_DN21504_c0_g1_i1_len_1267_194467.p1_type_5prime_partial_len_124 | ---NA--- | | |
| TRINITY_DN26220_c0_g1_i1_len_461_168124.p1_type_5prime_partial_len_124 | ---NA--- | | |
| TRINITY_DN26255_c0_g1_i1_len_413_169705.p1_type_5prime_partial_len_124 | XP_018022084.1 hydroxymethylglutaryl-CoA lyase, mitochondrial-like | F:GO:0004419 | EC:4.1.3.4 |
| TRINITY_DN13070_c0_g1_i1_len_417_306416.p1_type_5prime_partial_len_124 | ---NA--- | | |
| TRINITY_DN16458_c0_g1_i1_len_504_132277.p1_type_5prime_partial_len_124 | ---NA--- | | |
| TRINITY_DN379_c0_g1_i1_len_407_169959.p1_type_5prime_partial_len_124 | ---NA--- | | |
| TRINITY_DN3121_c0_g1_i1_len_401_170170.p1_type_5prime_partial_len_124 | ---NA--- | | |
| TRINITY_DN30853_c0_g1_i2_len_4240_187462.p4_type_complete_len_123 | ---NA--- | | |
| TRINITY_DN6434_c0_g1_i1_len_2254_230364.p3_type_5prime_partial_len_123 | ---NA--- | | |
| TRINITY_DN29210_c0_g1_i1_len_414_245861.p1_type_5prime_partial_len_123 | XP_018011093.1 actin-binding LIM protein 1-like | F:GO:0003779; P:GO:0007010; F:GO:0016740; F:GO:0046872 | |
| TRINITY_DN25219_c0_g1_i1_len_1594_232565.p2_type_complete_len_123 | ---NA--- | | |
| TRINITY_DN36945_c0_g1_i1_len_425_245446.p1_type_5prime_partial_len_123 | XP_022194177.1zinc finger protein 26-like isoform X2 | | |
| TRINITY_DN20397_c0_g1_i6_len_2210_30066.p2_type_complete_len_123 | ---NA--- | | |

| | | | |
|---|---|---|---|
| TRINITY_DN15593_c0_g1_i1_len_431_169046.p1_type_5prime_partial_len_123 | ---NA--- | | |
| TRINITY_DN18489_c4_g1_i1_len_1015_33412.p1_type_complete_len_123 | ---NA--- | | |
| TRINITY_DN10443_c0_g1_i1_len_524_131823.p1_type_5prime_partial_len_123 | ---NA--- | | |
| TRINITY_DN9708_c0_g1_i1_len_525_166516.p1_type_3prime_partial_len_124 | ---NA--- | | |
| TRINITY_DN18551_c0_g1_i2_len_411_11357.p1_type_5prime_partial_len_123 | ---NA--- | | |
| TRINITY_DN11538_c0_g1_i1_len_650_273412.p1_type_5prime_partial_len_123 | XP_017777920.1 protein Wnt-10b | F:GO:0005102; C:GO:0005576; P:GO:0007275; P:GO:0016055 | |
| TRINITY_DN17249_c0_g1_i1_len_409_246029.p1_type_3prime_partial_len_124 | ---NA--- | | |
| TRINITY_DN22144_c2_g1_i1_len_523_100788.p1_type_complete_len_123 | ---NA--- | | |
| TRINITY_DN4196_c0_g1_i1_len_370_12970.p2_type_internal_len_124 | ---NA--- | | |
| TRINITY_DN22938_c0_g1_i1_len_1747_231959.p2_type_5prime_partial_len_123 | ---NA--- | | |
| TRINITY_DN22749_c0_g1_i2_len_1379_193782.p4_type_5prime_partial_len_123 | XP_013139709.1 LIM and senescent cell antigen-like-containing domain protein 2 isoform X1 | | |
| TRINITY_DN11442_c0_g1_i1_len_492_243240.p1_type_5prime_partial_len_123 | ---NA--- | | |
| TRINITY_DN28973_c0_g1_i1_len_507_242845.p1_type_5prime_partial_len_123 | ---NA--- | | |
| TRINITY_DN33800_c0_g1_i1_len_409_135045.p1_type_5prime_partial_len_123 | XP_018020911.1 ankyrin-3-like | | |
| TRINITY_DN29724_c0_g1_i1_len_3204_188252.p3_type_complete_len_123 | ---NA--- | | |
| TRINITY_DN28641_c0_g1_i1_len_419_134701.p1_type_5prime_partial_len_123 | ---NA--- | | |

| | | | |
|---|---|---|---|
| TRINITY_DN9735_c0_g1_i1_len_573_165471.p2_type_3prime_partial_len_124 | ---NA--- | | |
| TRINITY_DN2819_c0_g1_i1_len_544_166063.p1_type_complete_len_123 | ---NA--- | | |
| TRINITY_DN24019_c0_g1_i2_len_2580_122382.p2_type_complete_len_123 | ---NA--- | | |
| TRINITY_DN22216_c0_g1_i1_len_1787_156729.p2_type_complete_len_123 | ---NA--- | | |
| TRINITY_DN16834_c0_g1_i1_len_445_10263.p1_type_5prime_partial_len_123 | ---NA--- | | |
| TRINITY_DN8504_c0_g1_i1_len_614_164752.p2_type_complete_len_123 | XP_017772218.1 protein NDRG3 isoform X5 | | |
| TRINITY_DN9284_c0_g1_i1_len_372_247677.p1_type_internal_len_124 | ---NA--- | | |
| TRINITY_DN32882_c0_g1_i1_len_372_247651.p1_type_internal_len_124 | XP_020937726.1von Willebrand factor A domain-containing protein 8-like | | |
| TRINITY_DN35537_c0_g1_i1_len_371_247705.p1_type_internal_len_124 | XP_018011507.1 SLIT-ROBO Rho GTPase-activating protein 1-like | | |
| TRINITY_DN31649_c0_g1_i1_len_371_247703.p2_type_internal_len_124 | XP_023722453.1CUB and sushi domain-containing protein 3 | | |
| TRINITY_DN26507_c0_g1_i1_len_371_247699.p1_type_internal_len_124 | XP_023709257.1ras-related protein Rab-34-like isoform X3 | F:GO:0003924; F:GO:0005525 | |
| TRINITY_DN11646_c0_g1_i1_len_371_247695.p1_type_internal_len_124 | ---NA--- | | |
| TRINITY_DN10988_c0_g1_i1_len_371_247693.p1_type_internal_len_124 | XP_018013212.1PREDICTED: uncharacterized protein LOC108670263 | | |
| TRINITY_DN4727_c0_g1_i1_len_371_247684.p2_type_internal_len_124 | ---NA--- | | |
| TRINITY_DN1139_c0_g1_i1_len_371_247681.p1_type_internal_len_124 | ---NA--- | | |
| TRINITY_DN5722_c0_g1_i1_len_372_12855.p2_type_internal_len_124 | AWU67222.1putative chitinase | F:GO:0004568; P:GO:0005975; F:GO:0008061 | EC:3.2.1.14 |

| | | | |
|---|---|---|---|
| TRINITY_DN2240_c0_g1_i1_len_373_247581.p1_type_internal_len_124 | EFN76354.1E3 ubiquitin-protein ligase mind-bomb | F:GO:0004842; F:GO:0008270; P:GO:0016567; F:GO:0016874 | |
| TRINITY_DN19901_c0_g1_i1_len_2075_230879.p2_type_5prime_partial_len_123 | ---NA--- | | |
| TRINITY_DN37199_c0_g1_i1_len_373_247631.p1_type_internal_len_124 | ---NA--- | | |
| TRINITY_DN22367_c0_g1_i1_len_1254_194561.p2_type_complete_len_123 | ---NA--- | | |
| TRINITY_DN10618_c0_g1_i1_len_369_247810.p1_type_internal_len_124 | ---NA--- | | |
| TRINITY_DN22894_c0_g1_i1_len_2568_154886.p2_type_complete_len_123 | ---NA--- | | |
| TRINITY_DN34551_c0_g1_i1_len_369_247794.p1_type_internal_len_124 | ---NA--- | | |
| TRINITY_DN6929_c0_g1_i1_len_370_247747.p1_type_internal_len_124 | KRT81117.1AAA protein | F:GO:0005524; F:GO:0016887 | EC:3.6.1.3; EC:3.6.1.15 |
| TRINITY_DN7325_c0_g1_i2_len_370_247740.p1_type_internal_len_124 | XP_023708009.1MAGUK p55 subfamily member 5 isoform X2 | C:GO:0001917; C:GO:0002142; C:GO:0005886; P:GO:0007605; C:GO:0032391; C:GO:0032426; P:GO:0042052; P:GO:0050953 | |
| TRINITY_DN15365_c0_g1_i1_len_370_247738.p2_type_internal_len_124 | ---NA--- | | |
| TRINITY_DN37157_c0_g1_i1_len_371_247722.p1_type_internal_len_124 | ---NA--- | | |
| TRINITY_DN26607_c0_g1_i1_len_371_247716.p1_type_internal_len_124 | ---NA--- | | |
| TRINITY_DN29484_c0_g1_i1_len_371_247709.p1_type_internal_len_124 | AFJ59952.1cation-independent mannose-6-phosphate receptor | P:GO:0007041; F:GO:0038023; F:GO:0051219 | |
| TRINITY_DN27095_c0_g1_i1_len_369_247790.p1_type_internal_len_124 | ---NA--- | | |
| TRINITY_DN4582_c0_g1_i1_len_369_247789.p2_type_internal_len_124 | ---NA--- | | |

| | | | |
|---|---|---|---|
| TRINITY_DN5347_c0_g1_i1_len_370_247783.p1_type_internal_len_124 | XP_019638841.1 G-protein coupled receptor 161-like | | |
| TRINITY_DN751_c0_g1_i1_len_370_247780.p1_type_internal_len_124 | ---NA--- | | |
| TRINITY_DN16865_c2_g1_i1_len_370_247779.p1_type_internal_len_124 | ---NA--- | | |
| TRINITY_DN37517_c0_g1_i1_len_370_247775.p1_type_internal_len_124 | ---NA--- | | |
| TRINITY_DN5130_c0_g1_i1_len_370_247756.p1_type_internal_len_124 | ---NA--- | | |
| TRINITY_DN10065_c0_g1_i1_len_738_272381.p2_type_complete_len_123 | XP_013161728.1 26S proteasome non-ATPase regulatory subunit 9 | | |
| TRINITY_DN10027_c0_g1_i1_len_533_100579.p1_type_5prime_partial_len_123 | ---NA--- | | |
| TRINITY_DN14025_c0_g1_i2_len_371_12915.p1_type_internal_len_124 | ---NA--- | | |
| TRINITY_DN12945_c0_g1_i1_len_371_12903.p1_type_internal_len_124 | ---NA--- | | |
| TRINITY_DN31491_c0_g1_i1_len_405_11517.p1_type_5prime_partial_len_123 | ---NA--- | | |
| TRINITY_DN15415_c0_g1_i1_len_677_200360.p2_type_complete_len_123 | XP_022237072.1organic cation transporter protein-like isoform X1 | | |
| TRINITY_DN30352_c0_g1_i1_len_371_12923.p1_type_internal_len_124 | ---NA--- | | |
| TRINITY_DN14980_c0_g1_i1_len_371_12877.p1_type_internal_len_124 | ---NA--- | | |
| TRINITY_DN16201_c0_g1_i1_len_951_33758.p2_type_complete_len_123 | ---NA--- | | |
| TRINITY_DN13544_c0_g1_i1_len_537_100512.p1_type_5prime_partial_len_123 | XP_021947749.1uncharacterized protein LOC110845604 isoform X2 | P:GO:0008152; F:GO:0016671 | |
| TRINITY_DN27088_c0_g1_i1_len_370_136723.p1_type_internal_len_124 | AUB30135.1cardiac-type actin 3 | F:GO:0005524 | |

| | | | |
|---|---|---|---|
| TRINITY_DN31086_c0_g1_i2_len_2396_189704.p1_type_complete_len_123 | ---NA--- | | |
| TRINITY_DN33643_c0_g1_i1_len_372_171424.p1_type_internal_len_124 | ---NA--- | | |
| TRINITY_DN15354_c0_g1_i1_len_808_162346.p1_type_complete_len_123 | ---NA--- | | |
| TRINITY_DN23877_c0_g4_i1_len_742_199400.p1_type_3prime_partial_len_124 | ---NA--- | | |
| TRINITY_DN8695_c0_g1_i1_len_371_136657.p1_type_internal_len_124 | XP_023222932.1tankyrase-1-like | F:GO:0016740 | |
| TRINITY_DN17625_c0_g1_i1_len_370_136683.p1_type_internal_len_124 | ---NA--- | | |
| TRINITY_DN18927_c0_g1_i2_len_369_171546.p1_type_internal_len_124 | ---NA--- | | |
| TRINITY_DN5150_c0_g1_i1_len_371_171442.p1_type_internal_len_124 | AXR98466.1fibrillin 2 | F:GO:0005044; F:GO:0005201; F:GO:0005509; P:GO:0006898; C:GO:0016020; C:GO:0062023 | |
| TRINITY_DN20080_c0_g1_i2_len_599_99441.p1_type_3prime_partial_len_124 | ---NA--- | | |
| TRINITY_DN28902_c0_g1_i3_len_2688_189058.p2_type_5prime_partial_len_123 | ---NA--- | | |
| TRINITY_DN1579_c0_g1_i1_len_378_136315.p1_type_5prime_partial_len_123 | XP_014252512.1BTB/POZ domain-containing protein 17 isoform X2 | | |
| TRINITY_DN18687_c0_g1_i1_len_699_5751.p1_type_5prime_partial_len_123 | XP_001121986.2cAMP-responsive element-binding protein-like 2 isoform X1 | | |
| TRINITY_DN5925_c0_g1_i1_len_620_240459.p1_type_5prime_partial_len_123 | ---NA--- | | |
| TRINITY_DN10959_c0_g1_i1_len_1107_159931.p1_type_5prime_partial_len_123 | ---NA--- | | |
| TRINITY_DN16397_c0_g1_i2_len_850_198029.p1_type_complete_len_123 | ---NA--- | | |
| TRINITY_DN19018_c0_g3_i1_len_395_104589.p1_type_5prime_partial_len_123 | ABW82154.1crustin-like protein | C:GO:0005576; P:GO:0010466; F:GO:0030414 | |

| | | | |
|---|---|---|---|
| TRINITY_DN9819_c0_g1_i1_len_372_136614.p2_type_internal_len_124 | ---NA--- | | |
| TRINITY_DN970_c0_g1_i1_len_373_171367.p1_type_internal_len_124 | ---NA--- | | |
| TRINITY_DN3398_c0_g1_i1_len_373_171363.p1_type_internal_len_124 | ---NA--- | | |
| TRINITY_DN31269_c0_g1_i1_len_372_136603.p1_type_internal_len_124 | ---NA--- | | |
| TRINITY_DN11303_c0_g1_i1_len_373_171347.p1_type_internal_len_124 | PRD22648.1zinc finger protein | F:GO:0003677 | |
| 2337\|_\|c25608_f3p3_2623_isoform_c25608_full_length_coverage_3_non_full_length_coverage_3_isoform_length_2623_13931 | AJO70002.1V-ATPase A | F:GO:0005524; P:GO:0015991; C:GO:0033180; P:GO:0046034; F:GO:0046961 | EC:3.6.1.3; EC:3.6.1.15 |
| 2338\|_\|c2357_f3p2_2622_isoform_c2357_full_length_coverage_3_non_full_length_coverage_2_isoform_length_2622_16350 | XP_018009461.1 optineurin-like isoform X2 | | |
| TRINITY_DN28239_c0_g1_i1_len_2894_188705.p1_type_5prime_partial_len_874 | XP_018014971.1 zinc finger protein 699-like | F:GO:0003676; P:GO:0006355; F:GO:0046872 | |
| TRINITY_DN26277_c0_g1_i1_len_2895_229143.p1_type_5prime_partial_len_874 | XP_018027994.1 exosome component 10-like | P:GO:0044237 | |
| 2343\|_\|c3535_f3p2_2621_isoform_c3535_full_length_coverage_3_non_full_length_coverage_2_isoform_length_2621_12306 | ---NA--- | | |
| 2344\|_\|c10602_f2p11_2620_isoform_c10602_full_length_coverage_2_non_full_length_coverage_11_isoform_length_2620_4851 | XP_018008735.1 protein lin-7 homolog C-like | | |
| 2345\|_\|c17905_f2p3_2620_isoform_c17905_full_length_coverage_2_non_full_length_coverage_3_isoform_length_2620_22558 | XP_011303763.1 probable tRNA (uracil-O(2)-)-methyltransferase | F:GO:0008168; F:GO:0016740; P:GO:0032259 | |
| 2346\|_\|c2317_f2p0_2619_isoform_c2317_full_length_coverage_2_non_full_length_coverage_0_isoform_length_2619_20842 | ---NA--- | | |

| | | | |
|---|---|---|---|
| 2348\|_\|c17693_f3p3_2618_isoform_c17693_full_length_coverage_3_non_full_length_coverage_3_isoform_length_2618_22460 | XP_006817934.1 spore wall protein 2-like | C:GO:0016020; C:GO:0016021 | |
| 2350\|_\|c10879_f3p4_2616_isoform_c10879_full_length_coverage_3_non_full_length_coverage_4_isoform_length_2616_22002 | XP_013379529.1Fanconi anemia group M protein | F:GO:0003676; F:GO:0016787; P:GO:0090304 | |
| TRINITY_DN15110_c0_g1_i1_len_371_136643.p2_type_internal_len_124 | ---NA--- | | |
| TRINITY_DN16518_c0_g1_i1_len_673_239608.p1_type_5prime_partial_len_123 | ---NA--- | | |
| TRINITY_DN22781_c0_g1_i4_len_1553_192809.p2_type_complete_len_123 | ---NA--- | | |
| TRINITY_DN701_c0_g1_i1_len_386_135978.p1_type_5prime_partial_len_123 | XP_019631525.1 zinc finger protein 37-like | C:GO:0005634 | |
| TRINITY_DN11539_c0_g1_i2_len_609_274016.p1_type_5prime_partial_len_123 | ---NA--- | | |
| TRINITY_DN24370_c0_g1_i1_len_372_280088.p1_type_internal_len_124 | EEC06499.1fucosyltransferase, putative | | |
| TRINITY_DN17098_c0_g1_i1_len_777_198888.p1_type_complete_len_123 | ---NA--- | | |
| TRINITY_DN25363_c0_g1_i1_len_373_280042.p1_type_internal_len_124 | ---NA--- | | |
| TRINITY_DN12036_c0_g1_i1_len_1280_234177.p1_type_complete_len_123 | XP_018024071.1 dnaJ homolog subfamily C member 27-like | F:GO:0003924; F:GO:0005525 | EC:3.6.1.15 |
| TRINITY_DN15994_c0_g1_i1_len_507_8669.p1_type_5prime_partial_len_123 | XP_018015252.1 immediate early response 3-interacting protein 1-like | C:GO:0016021 | |
| TRINITY_DN26005_c0_g1_i1_len_941_197073.p1_type_complete_len_123 | XP_013786261.1microtubule-associated proteins 1A/1B light chain 3A-like | C:GO:0005737; P:GO:0006914 | |
| TRINITY_DN24535_c0_g1_i1_len_377_279843.p1_type_5prime_partial_len_123 | ---NA--- | | |
| TRINITY_DN17889_c0_g1_i2_len_371_105568.p2_type_internal_len_124 | KFB42598.1muscle-specific actin-like protein | F:GO:0005524 | |

| | | | |
|---|---|---|---|
| TRINITY_DN29925_c0_g1_i1_len_371_105598.p1_type_internal_len_124 | ---NA--- | | |
| TRINITY_DN192_c0_g1_i1_len_373_105503.p1_type_internal_len_124 | ---NA--- | | |
| TRINITY_DN7763_c0_g1_i1_len_369_171555.p1_type_internal_len_124 | ---NA--- | | |
| TRINITY_DN3697_c0_g1_i1_len_369_171554.p1_type_internal_len_124 | ---NA--- | | |
| TRINITY_DN24991_c0_g1_i1_len_371_280129.p1_type_internal_len_124 | XP_021962531.1mucin-5AC-like isoform X1 | C:GO:0016020 | |
| TRINITY_DN31128_c0_g1_i1_len_369_105698.p1_type_internal_len_124 | ---NA--- | | |
| TRINITY_DN12294_c0_g1_i1_len_372_280093.p1_type_internal_len_124 | ---NA--- | | |
| TRINITY_DN315_c0_g1_i1_len_805_237902.p1_type_5prime_partial_len_123 | ---NA--- | | |
| TRINITY_DN11715_c0_g1_i1_len_607_274039.p1_type_complete_len_123 | ---NA--- | | |
| TRINITY_DN32083_c0_g1_i1_len_741_163036.p2_type_5prime_partial_len_123 | ---NA--- | | |
| TRINITY_DN24427_c0_g1_i1_len_370_280177.p1_type_internal_len_124 | XP_018024208.1 protein O-mannosyl-transferase 2-like | | |
| TRINITY_DN15142_c0_g1_i1_len_370_105666.p1_type_internal_len_124 | ---NA--- | | |
| TRINITY_DN28529_c0_g1_i1_len_370_105658.p1_type_internal_len_124 | ---NA--- | | |
| TRINITY_DN26268_c0_g1_i1_len_370_105656.p1_type_internal_len_124 | ---NA--- | | |
| TRINITY_DN41362_c0_g1_i1_len_369_209600.p1_type_internal_len_124 | XP_018013901.1 leucine-rich repeats and immunoglobulin-like domains protein 2 | | |
| TRINITY_DN12380_c0_g1_i1_len_427_306078.p1_type_5prime_partial_len_123 | ---NA--- | | |

| | | | |
|---|---|---|---|
| TRINITY_DN34148_c0_g1_i1_len_369_209611.p1_type_internal_len_124 | KMQ95831.1nucleolar protein 4 | | |
| TRINITY_DN18460_c1_g1_i1_len_369_209612.p1_type_internal_len_124 | ---NA--- | | |
| TRINITY_DN12039_c0_g1_i1_len_494_204438.p1_type_5prime_partial_len_123 | XP_018013900.1 ras-related protein Rab-9A-like | F:GO:0003924; F:GO:0005525 | EC:3.6.1.15 |
| TRINITY_DN23244_c0_g1_i1_len_496_204379.p1_type_5prime_partial_len_123 | ---NA--- | | |
| TRINITY_DN34431_c0_g1_i1_len_380_73232.p1_type_5prime_partial_len_123 | ---NA--- | | |
| TRINITY_DN221_c0_g1_i1_len_453_205858.p1_type_5prime_partial_len_123 | XP_018021179.1 protein abrupt-like | | |
| TRINITY_DN15525_c0_g1_i1_len_497_204346.p1_type_5prime_partial_len_123 | ACB47428.1Kazal-type proteinase inhibitor | | |
| TRINITY_DN31368_c0_g1_i1_len_373_73551.p2_type_internal_len_124 | ---NA--- | | |
| TRINITY_DN9363_c0_g1_i1_len_393_41717.p1_type_5prime_partial_len_123 | ---NA--- | | |
| TRINITY_DN12694_c0_g1_i1_len_369_209589.p1_type_internal_len_124 | ODM87514.1histone H3 | C:GO:0000786; F:GO:0003677; C:GO:0005634; F:GO:0046982 | |
| TRINITY_DN21142_c0_g1_i1_len_476_205004.p1_type_5prime_partial_len_123 | ---NA--- | | |
| TRINITY_DN38041_c0_g1_i1_len_369_209594.p1_type_internal_len_124 | XP_018011572.1 teneurin-m-like | | |
| TRINITY_DN29024_c0_g2_i1_len_404_207839.p2_type_5prime_partial_len_123 | ---NA--- | | |
| TRINITY_DN29024_c0_g2_i1_len_404_207839.p1_type_5prime_partial_len_123 | ---NA--- | | |
| TRINITY_DN15862_c0_g1_i1_len_593_201934.p1_type_complete_len_123 | ---NA--- | | |
| TRINITY_DN9794_c0_g1_i1_len_407_207707.p1_type_3prime_partial_len_124 | XP_018014829.1 phosphoglucomutase-2-like | F:GO:0000287; P:GO:0005975; F:GO:0016868 | |

| | | | |
|---|---|---|---|
| TRINITY_DN17616_c0_g1_i1_len_504_204122.p1_type_3prime_partial_len_124 | ---NA--- | | |
| TRINITY_DN28776_c0_g1_i1_len_370_73694.p1_type_internal_len_124 | ---NA--- | | |
| TRINITY_DN39772_c0_g1_i1_len_370_209521.p1_type_internal_len_124 | ---NA--- | | |
| TRINITY_DN2393_c0_g1_i1_len_370_209523.p1_type_internal_len_124 | XP_018020023.1 spectrin beta chain, non-erythrocytic 1-like | | |
| TRINITY_DN15613_c0_g1_i1_len_370_209524.p1_type_internal_len_124 | ---NA--- | | |
| TRINITY_DN33515_c0_g1_i1_len_370_73683.p1_type_internal_len_124 | ---NA--- | | |
| TRINITY_DN37450_c0_g1_i1_len_370_209528.p1_type_internal_len_124 | ---NA--- | | |
| TRINITY_DN35827_c0_g1_i1_len_370_209534.p1_type_internal_len_124 | ---NA--- | | |
| TRINITY_DN38173_c0_g1_i1_len_370_209536.p1_type_internal_len_124 | ---NA--- | | |
| TRINITY_DN38108_c0_g1_i1_len_371_209478.p2_type_internal_len_124 | ---NA--- | | |
| TRINITY_DN13227_c0_g1_i1_len_369_73728.p1_type_internal_len_124 | ---NA--- | | |
| TRINITY_DN238_c0_g1_i1_len_371_209493.p1_type_internal_len_124 | ---NA--- | | |
| TRINITY_DN41355_c0_g1_i1_len_370_209499.p1_type_internal_len_124 | ---NA--- | | |
| TRINITY_DN13508_c0_g1_i1_len_370_209508.p1_type_internal_len_124 | ---NA--- | | |
| TRINITY_DN974_c0_g1_i1_len_370_209510.p1_type_internal_len_124 | ---NA--- | | |
| TRINITY_DN36678_c0_g1_i1_len_370_209513.p1_type_internal_len_124 | ---NA--- | | |

| | | | |
|---|---|---|---|
| TRINITY_DN31510_c0_g1_i1_len_370_209548.p1_type_internal_len_124 | ---NA--- | | |
| TRINITY_DN3861_c0_g1_i1_len_372_73610.p1_type_internal_len_124 | ---NA--- | | |
| TRINITY_DN3262_c0_g1_i1_len_370_209552.p1_type_internal_len_124 | PIK40076.1hypothetical protein BSL78_23082 | | |
| TRINITY_DN1030_c0_g1_i1_len_370_209553.p1_type_internal_len_124 | ---NA--- | | |
| TRINITY_DN12942_c0_g1_i1_len_369_209556.p1_type_internal_len_124 | AHN53443.1reverse transcriptase | F:GO:0003676; F:GO:0003700; F:GO:0003964; P:GO:0006278; P:GO:0006355; F:GO:0008270; P:GO:0015074 | |
| TRINITY_DN19022_c0_g1_i1_len_550_202891.p1_type_5prime_partial_len_123 | ---NA--- | | |
| TRINITY_DN32158_c0_g1_i1_len_369_209568.p1_type_internal_len_124 | ABP97431.1alpha 2 macroglobulin | F:GO:0004866; C:GO:0005576; C:GO:0005615; P:GO:0010951 | |
| TRINITY_DN17888_c0_g1_i1_len_370_209537.p1_type_internal_len_124 | ---NA--- | | |
| TRINITY_DN21125_c1_g1_i1_len_370_209541.p1_type_internal_len_124 | ---NA--- | | |
| TRINITY_DN15108_c0_g1_i1_len_986_298879.p1_type_5prime_partial_len_123 | ---NA--- | | |
| TRINITY_DN29450_c0_g1_i1_len_371_73638.p1_type_internal_len_124 | ---NA--- | | |
| TRINITY_DN9300_c0_g1_i2_len_374_308077.p1_type_5prime_partial_len_123 | XP_018026113.1PREDICTED: uncharacterized protein LOC108681578 | | |
| TRINITY_DN16038_c0_g1_i2_len_630_201201.p1_type_3prime_partial_len_124 | XP_018021508.1 sialic acid synthase-like | F:GO:0003824; P:GO:0016051 | |
| TRINITY_DN11789_c0_g1_i1_len_373_308103.p1_type_internal_len_124 | XP_018021226.1 organic cation transporter protein-like | | |
| TRINITY_DN6771_c0_g1_i1_len_370_308267.p1_type_internal_len_124 | XP_018024691.1PREDICTED: uncharacterized protein LOC108680392 | F:GO:0003824 | |

| | | | |
|---|---|---|---|
| TRINITY_DN20997_c0_g1_i3_len_431_71193.p1_type_5prime_partial_len_123 | ---NA--- | | |
| TRINITY_DN24876_c0_g1_i2_len_2631_58440.p2_type_5prime_partial_len_123 | ---NA--- | | |
| TRINITY_DN20996_c1_g1_i1_len_469_205236.p1_type_3prime_partial_len_124 | XP_018019976.1 kynureninase-like | | |
| TRINITY_DN15008_c0_g1_i1_len_581_202183.p1_type_5prime_partial_len_123 | ---NA--- | | |
| TRINITY_DN34191_c0_g1_i1_len_411_207559.p1_type_5prime_partial_len_123 | ---NA--- | | |
| TRINITY_DN5891_c0_g1_i1_len_520_203638.p1_type_5prime_partial_len_123 | ---NA--- | | |
| TRINITY_DN38216_c0_g1_i1_len_372_209400.p2_type_internal_len_124 | ABC86571.1vitellogenin | F:GO:0005319; P:GO:0006869; F:GO:0008289; F:GO:0045735; P:GO:0048477 | |
| TRINITY_DN25622_c0_g1_i3_len_371_209460.p2_type_internal_len_124 | ---NA--- | | |
| TRINITY_DN28586_c0_g1_i1_len_370_42623.p1_type_internal_len_124 | ---NA--- | | |
| TRINITY_DN36798_c0_g1_i1_len_371_209466.p2_type_internal_len_124 | ---NA--- | | |
| TRINITY_DN11739_c0_g1_i1_len_373_209340.p1_type_internal_len_124 | ---NA--- | | |
| TRINITY_DN35144_c0_g1_i1_len_371_209465.p1_type_internal_len_124 | ---NA--- | | |
| TRINITY_DN31829_c0_g1_i1_len_373_209362.p1_type_internal_len_124 | ---NA--- | | |
| TRINITY_DN640_c0_g1_i1_len_467_205306.p1_type_5prime_partial_len_123 | ---NA--- | | |
| TRINITY_DN21552_c0_g1_i1_len_2414_295244.p2_type_5prime_partial_len_123 | ---NA--- | | |
| TRINITY_DN34011_c0_g1_i1_len_371_209463.p1_type_internal_len_124 | KFM81092.1Roundabout-like protein | | |

| | | | |
|---|---|---|---|
| TRINITY_DN32216_c0_g1_i1_len_372_209432.p1_type_internal_len_124 | XP_018007219.1 2-hydroxy-3-oxopropionate reductase-like | F:GO:0016491; F:GO:0050661; F:GO:0051287; P:GO:0055114 | |
| TRINITY_DN35852_c0_g1_i1_len_371_209453.p1_type_internal_len_124 | ---NA--- | | |
| TRINITY_DN7455_c0_g1_i1_len_371_209441.p1_type_internal_len_124 | ---NA--- | | |
| TRINITY_DN36932_c0_g1_i1_len_373_209374.p1_type_internal_len_124 | XP_020293032.1alpha-(1,3)-fucosyltransferase 7-like | | |
| TRINITY_DN14075_c1_g1_i1_len_372_209380.p1_type_internal_len_124 | ---NA--- | | |
| TRINITY_DN40140_c0_g1_i1_len_371_209442.p1_type_internal_len_124 | ---NA--- | | |
| TRINITY_DN22469_c0_g2_i1_len_375_209267.p1_type_5prime_partial_len_123 | ---NA--- | | |
| TRINITY_DN9351_c0_g1_i1_len_379_209086.p1_type_5prime_partial_len_123 | XP_018118418.1 ADP-dependent glucokinase-like | P:GO:0005975; F:GO:0016773 | |
| 2351\|_\|c1221_f19p12_2615_isoform_c1221_full_length_coverage_19_non_full_length_coverage_12_isoform_length_2615_15824 | AQW44865.1thiredoxin reductase | F:GO:0004791; C:GO:0005739; F:GO:0009055; F:GO:0015035; P:GO:0022900; P:GO:0045454; F:GO:0050660; P:GO:0098869 | EC:1.8.1.9 |
| 2352\|_\|c4424_f2p4_2615_isoform_c4424_full_length_coverage_2_non_full_length_coverage_4_isoform_length_2615_12379 | XP_023719153.1protein wings apart-like | | |
| 2353\|_\|c26343_f2p0_2615_isoform_c26343_full_length_coverage_2_non_full_length_coverage_0_isoform_length_2615_23005 | XP_021931753.1glycerol-3-phosphate acyltransferase 4 isoform X2 | | |
| 2354\|_\|c1648_f14p7_2614_isoform_c1648_full_length_coverage_14_non_full_length_coverage_7_isoform_length_2614_15937 | XP_018025737.1 NECAP-like protein CG9132 | | |
| TRINITY_DN17144_c0_g1_i1_len_3645_29305.p1_type_complete_len_871 | XP_021937878.1cell division cycle protein 27 homolog isoform X2 | P:GO:0051301 | |
| TRINITY_DN27834_c0_g1_i1_len_3051_188450.p1_type_complete_len_871 | XP_022208009.1UV radiation resistance associated protein | | |

| | | | |
|---|---|---|---|
| TRINITY_DN25806_c1_g1_i2_len_4324_228013.p1_type_5prime_partial_len_871 | ---NA--- | | |
| TRINITY_DN27416_c0_g2_i2_len_3296_188140.p1_type_5prime_partial_len_871 | ---NA--- | | |
| 2360\|_\|c1632_f2p5_2613_isoform_c1632_full_length_coverage_2_non_full_length_coverage_5_isoform_length_2613_25460 | ---NA--- | | |
| 2361\|_\|c2844_f3p2_2613_isoform_c2844_full_length_coverage_3_non_full_length_coverage_2_isoform_length_2613_12207 | XP_021928897.1mitochondrial import inner membrane translocase subunit TIM44 isoform X2 | C:GO:0005743; P:GO:0030150; F:GO:0051087 | |
| TRINITY_DN3592_c0_g1_i1_len_371_209454.p3_type_internal_len_124 | ---NA--- | | |
| TRINITY_DN36895_c0_g1_i1_len_371_209439.p1_type_internal_len_124 | XP_018023322.1 H(+)/Cl(-) exchange transporter 5-like isoform X3 | | |
| TRINITY_DN4819_c0_g1_i1_len_372_209413.p1_type_internal_len_124 | ---NA--- | | |
| TRINITY_DN13197_c0_g1_i1_len_369_73737.p1_type_internal_len_124 | ---NA--- | | |
| TRINITY_DN54_c0_g1_i1_len_372_209417.p1_type_internal_len_124 | ---NA--- | | |
| TRINITY_DN37272_c0_g1_i1_len_371_209474.p2_type_5prime_partial_len_123 | ---NA--- | | |
| TRINITY_DN37872_c0_g1_i1_len_371_209472.p1_type_internal_len_124 | AFB71156.1serine protease-like protein 1 | F:GO:0004252; P:GO:0006508; F:GO:0008233 | |
| TRINITY_DN26468_c0_g1_i1_len_368_136780.p1_type_internal_len_123 | ---NA--- | | |
| TRINITY_DN13397_c0_g1_i1_len_516_303835.p3_type_complete_len_122 | XP_018010166.1 cuticle protein 7-like | F:GO:0042302 | |
| TRINITY_DN2556_c0_g1_i1_len_368_136778.p2_type_internal_len_123 | ---NA--- | | |
| TRINITY_DN366_c0_g1_i1_len_567_202486.p1_type_5prime_partial_len_122 | ---NA--- | | |

| | | | |
|---|---|---|---|
| TRINITY_DN20287_c0_g2_i2_len_413_11272.p1_type_5prime_partial_len_122 | ---NA--- | | |
| TRINITY_DN20261_c0_g1_i1_len_622_99097.p1_type_complete_len_122 | ---NA--- | | |
| TRINITY_DN14411_c0_g1_i1_len_1392_297123.p2_type_complete_len_122 | ---NA--- | | |
| TRINITY_DN18655_c0_g1_i1_len_1534_267615.p2_type_5prime_partial_len_122 | ---NA--- | | |
| TRINITY_DN9118_c0_g1_i1_len_370_280174.p1_type_internal_len_123 | ---NA--- | | |
| TRINITY_DN24441_c0_g1_i1_len_1601_192554.p1_type_5prime_partial_len_122 | XP_018006795.1 longitudinals lacking protein, isoforms H/M/V-like isoform X33 | F:GO:0003676 | |
| TRINITY_DN23248_c0_g1_i2_len_1907_231392.p1_type_5prime_partial_len_122 | ---NA--- | | |
| TRINITY_DN25713_c1_g1_i2_len_1769_231870.p2_type_complete_len_122 | ---NA--- | | |
| TRINITY_DN20504_c0_g1_i1_len_890_96330.p1_type_complete_len_122 | ---NA--- | | |
| TRINITY_DN5725_c0_g1_i1_len_367_13070.p1_type_internal_len_123 | XP_023705553.1peroxisomal membrane protein PMP34 isoform X2 | C:GO:0016021; F:GO:0022857; P:GO:0055085 | |
| TRINITY_DN19677_c0_g1_i9_len_1050_95291.p2_type_complete_len_122 | ---NA--- | | |
| TRINITY_DN13011_c0_g1_i1_len_368_136810.p1_type_internal_len_123 | ---NA--- | | |
| TRINITY_DN16158_c0_g1_i2_len_1129_159786.p2_type_complete_len_122 | ---NA--- | | |
| TRINITY_DN15241_c0_g1_i1_len_1138_159705.p1_type_complete_len_122 | ---NA--- | | |
| TRINITY_DN24416_c0_g1_i2_len_569_202430.p1_type_3prime_partial_len_123 | ---NA--- | | |
| TRINITY_DN14309_c0_g1_i1_len_368_136813.p1_type_internal_len_123 | ---NA--- | | |

| | | | |
|---|---|---|---|
| TRINITY_DN13912_c0_g1_i1_len_366_209757.p1_type_internal_len_123 | XP_001509296.2 magnesium transporter NIPA2 | F:GO:0015095; C:GO:0016021; P:GO:1903830 | |
| TRINITY_DN17224_c0_g1_i1_len_593_240940.p1_type_3prime_partial_len_123 | ---NA--- | | |
| TRINITY_DN10951_c0_g1_i1_len_367_136834.p1_type_internal_len_123 | ---NA--- | | |
| TRINITY_DN30097_c0_g1_i1_len_2395_189707.p2_type_complete_len_122 | ---NA--- | | |
| TRINITY_DN20949_c0_g1_i1_len_1352_297265.p3_type_5prime_partial_len_122 | ---NA--- | | |
| TRINITY_DN15698_c0_g1_i2_len_586_241070.p2_type_complete_len_122 | ---NA--- | | |
| TRINITY_DN13277_c1_g1_i2_len_484_276426.p2_type_5prime_partial_len_122 | XP_014775064.1 zinc finger protein 239-like | F:GO:0003676 | |
| TRINITY_DN27295_c1_g1_i1_len_368_136800.p2_type_internal_len_123 | ---NA--- | | |
| TRINITY_DN23784_c0_g1_i2_len_3052_58141.p2_type_complete_len_122 | ---NA--- | | |
| TRINITY_DN29098_c0_g1_i1_len_395_246601.p1_type_5prime_partial_len_122 | ---NA--- | | |
| TRINITY_DN365_c0_g1_i1_len_473_133046.p1_type_5prime_partial_len_122 | ---NA--- | | |
| TRINITY_DN16626_c0_g1_i2_len_580_202200.p1_type_5prime_partial_len_122 | ---NA--- | | |
| TRINITY_DN13133_c1_g1_i1_len_367_308386.p1_type_internal_len_123 | EDL75408.1tubulin, alpha 4, isoform CRA_b | | |
| TRINITY_DN21965_c0_g1_i1_len_1523_232915.p3_type_5prime_partial_len_122 | ---NA--- | | |
| TRINITY_DN11539_c0_g1_i2_len_609_274016.p2_type_5prime_partial_len_122 | ---NA--- | | |
| TRINITY_DN30241_c0_g1_i1_len_3348_188078.p2_type_complete_len_122 | AEE25939.1V-H-ATPase subunit A | C:GO:0000220; F:GO:0015078; P:GO:0015991; C:GO:0016021 | |

| | | | |
|---|---|---|---|
| TRINITY_DN22743_c0_g1_i1_len_4221_153614.p2_type_complete_len_122 | ---NA--- | | |
| TRINITY_DN9146_c0_g1_i1_len_494_304305.p2_type_5prime_partial_len_122 | ---NA--- | | |
| TRINITY_DN23857_c0_g1_i2_len_1547_232787.p1_type_complete_len_122 | ---NA--- | | |
| TRINITY_DN19938_c0_g1_i6_len_1334_93898.p2_type_5prime_partial_len_122 | ---NA--- | | |
| TRINITY_DN15659_c0_g1_i1_len_367_308393.p1_type_internal_len_123 | ---NA--- | | |
| TRINITY_DN28299_c0_g1_i1_len_368_105720.p1_type_internal_len_123 | XP_018010866.1PREDICTED: uncharacterized protein LOC108668205 | | |
| TRINITY_DN4503_c0_g1_i2_len_369_308299.p1_type_internal_len_123 | OXA50206.1Sedoheptulokinase | P:GO:0005975; F:GO:0016301; P:GO:0016310; F:GO:0016773 | |
| TRINITY_DN17791_c0_g1_i1_len_367_105772.p1_type_internal_len_123 | ---NA--- | | |
| TRINITY_DN9265_c0_g1_i1_len_368_308331.p2_type_internal_len_123 | ---NA--- | | |
| TRINITY_DN595_c0_g1_i1_len_366_105809.p1_type_internal_len_123 | ---NA--- | | |
| TRINITY_DN4865_c0_g1_i1_len_366_105811.p1_type_internal_len_123 | ---NA--- | | |
| TRINITY_DN11281_c0_g1_i1_len_489_304422.p1_type_complete_len_122 | ---NA--- | | |
| TRINITY_DN22875_c0_g1_i1_len_368_308325.p1_type_internal_len_123 | XP_018015529.1 protein ABHD17B-like | F:GO:0016787 | |
| TRINITY_DN17627_c0_g1_i1_len_793_162482.p1_type_5prime_partial_len_122 | ---NA--- | | |
| TRINITY_DN11907_c0_g1_i1_len_367_105796.p1_type_internal_len_123 | ---NA--- | | |
| TRINITY_DN15103_c0_g1_i1_len_425_10873.p1_type_5prime_partial_len_122 | ---NA--- | | |

| | | | |
|---|---|---|---|
| TRINITY_DN19255_c1_g1_i1_len_418_207232.p1_type_5prime_partial_len_122 | ---NA--- | | |
| TRINITY_DN3558_c0_g1_i1_len_460_133375.p1_type_5prime_partial_len_122 | XP_018015723.1 copine-8-like | | |
| TRINITY_DN8410_c0_g1_i1_len_671_239622.p1_type_3prime_partial_len_123 | ---NA--- | | |
| TRINITY_DN30919_c0_g1_i1_len_3108_188363.p6_type_5prime_partial_len_122 | ---NA--- | | |
| TRINITY_DN19623_c0_g1_i1_len_1377_268227.p3_type_5prime_partial_len_122 | ---NA--- | | |
| TRINITY_DN6096_c0_g1_i2_len_366_13123.p1_type_internal_len_123 | ---NA--- | | |
| TRINITY_DN32047_c0_g1_i1_len_370_280165.p1_type_internal_len_123 | XP_018017552.1 serine/threonine-protein kinase 3-like | F:GO:0004672; F:GO:0005524; P:GO:0006468 | |
| TRINITY_DN26204_c0_g1_i1_len_366_308425.p1_type_internal_len_123 | ---NA--- | | |
| TRINITY_DN21752_c0_g1_i1_len_1572_232673.p2_type_complete_len_122 | ---NA--- | | |
| TRINITY_DN20266_c0_g1_i1_len_494_304294.p1_type_5prime_partial_len_122 | ---NA--- | | |
| TRINITY_DN3279_c0_g1_i1_len_370_280149.p1_type_internal_len_123 | ---NA--- | | |
| TRINITY_DN1110_c0_g1_i1_len_367_209724.p1_type_3prime_partial_len_123 | ---NA--- | | |
| TRINITY_DN13774_c0_g1_i1_len_431_10689.p1_type_3prime_partial_len_123 | ---NA--- | | |
| TRINITY_DN22397_c0_g1_i1_len_368_42729.p1_type_internal_len_123 | ---NA--- | | |
| TRINITY_DN8713_c0_g1_i2_len_367_280294.p1_type_internal_len_123 | XP_013788008.2two pore calcium channel protein 1-like isoform X1 | F:GO:0005216; C:GO:0016021; P:GO:0034220 | |
| TRINITY_DN36035_c0_g1_i1_len_367_247922.p2_type_internal_len_123 | ---NA--- | | |

| | | | |
|---|---|---|---|
| TRINITY_DN6332_c0_g1_i1_len_368_247881.p1_type_3prime_partial_len_123 | XP_012559954.1 proprotein convertase subtilisin/kexin type 7-like | F:GO:0004252; P:GO:0006508 | EC:3.4.21 |
| TRINITY_DN19462_c0_g1_i1_len_368_247878.p1_type_internal_len_123 | ---NA--- | | |
| TRINITY_DN1782_c0_g1_i1_len_368_247876.p2_type_internal_len_123 | ---NA--- | | |
| TRINITY_DN8407_c0_g1_i1_len_368_42740.p1_type_internal_len_123 | ---NA--- | | |
| TRINITY_DN16311_c0_g1_i1_len_1474_31532.p3_type_5prime_partial_len_122 | ---NA--- | | |
| TRINITY_DN36774_c0_g1_i1_len_367_247892.p1_type_internal_len_123 | ---NA--- | | |
| TRINITY_DN29623_c0_g1_i1_len_367_280292.p1_type_internal_len_123 | XP_018011207.1 ileal sodium/bile acid cotransporter-like | C:GO:0016020; C:GO:0016021 | |
| TRINITY_DN21799_c0_g1_i9_len_1693_157070.p2_type_complete_len_122 | ---NA--- | | |
| TRINITY_DN15487_c0_g1_i1_len_369_42676.p1_type_internal_len_123 | ---NA--- | | |
| TRINITY_DN25940_c0_g1_i1_len_740_65202.p1_type_5prime_partial_len_122 | ---NA--- | | |
| TRINITY_DN6320_c0_g1_i1_len_366_209781.p1_type_internal_len_123 | ---NA--- | | |
| TRINITY_DN37274_c0_g1_i1_len_367_247947.p1_type_internal_len_123 | ---NA--- | | |
| TRINITY_DN35340_c0_g1_i1_len_367_247942.p1_type_internal_len_123 | ---NA--- | | |
| TRINITY_DN15508_c0_g1_i1_len_465_244069.p1_type_3prime_partial_len_123 | ---NA--- | | |
| TRINITY_DN27098_c0_g1_i1_len_367_247929.p1_type_3prime_partial_len_123 | ---NA--- | | |
| TRINITY_DN35580_c0_g1_i1_len_367_247928.p1_type_internal_len_123 | ---NA--- | | |

| | | | |
|---|---|---|---|
| TRINITY_DN29249_c0_g1_i1_len_368_280250.p1_type_internal_len_123 | ---NA--- | | |
| TRINITY_DN17498_c0_g1_i1_len_677_65954.p1_type_5prime_partial_len_122 | ---NA--- | | |
| TRINITY_DN15805_c0_g1_i1_len_434_245096.p1_type_5prime_partial_len_122 | ---NA--- | | |
| TRINITY_DN27071_c0_g1_i1_len_370_247768.p1_type_internal_len_123 | ---NA--- | | |
| TRINITY_DN33753_c0_g1_i1_len_370_247742.p1_type_internal_len_123 | XP_026274218.1UHRF1-binding protein 1-like isoform X1 | | |
| TRINITY_DN2487_c0_g1_i1_len_370_247786.p1_type_internal_len_123 | XP_018020970.1 synaptojanin-1-like isoform X2 | | |
| TRINITY_DN4323_c0_g1_i1_len_370_247773.p1_type_internal_len_123 | XP_022202820.1fat-like cadherin-related tumor suppressor homolog | | |
| TRINITY_DN23455_c0_g1_i1_len_370_12951.p1_type_internal_len_123 | XP_023222726.1neurotrypsin-like isoform X1 | | |
| TRINITY_DN31947_c0_g1_i1_len_368_247868.p1_type_internal_len_123 | ---NA--- | | |
| 2362\|_\|c36258_f2p33_2612_isoform_c36258_full_length_coverage_2_non_full_length_coverage_33_isoform_length_2612_23589 | AMQ13578.1p53 protein | F:GO:0003700; C:GO:0005634; P:GO:0006355; P:GO:0006915; F:GO:0044212 | |
| 2363\|_\|c47185_f2p1_2611_isoform_c47185_full_length_coverage_2_non_full_length_coverage_1_isoform_length_2611_24021 | ---NA--- | | |
| 2364\|_\|c2717_f4p2_2611_isoform_c2717_full_length_coverage_4_non_full_length_coverage_2_isoform_length_2611_21176 | XP_018024283.1 N-acetylglucosamine-6-sulfatase-like | F:GO:0003824; F:GO:0008449; F:GO:0008484; P:GO:0030203 | |
| 2365\|_\|c10499_f4p1_2611_isoform_c10499_full_length_coverage_4_non_full_length_coverage_1_isoform_length_2611_21966 | XP_018022934.1 cleavage stimulation factor subunit 3-like | C:GO:0005634; P:GO:0006397 | |
| 2366\|_\|c3586_f2p2_2610_isoform_c3586_full_length_coverage_2_non_full_length_coverage_2_isoform_length_2610_12312 | XP_013415188.1diphthine methyl ester synthase | F:GO:0004164; P:GO:0017183; P:GO:0032259 | EC:2.1.1.98 |

| | | | |
|---|---|---|---|
| TRINITY_DN29724_c0_g1_i1_len_3204_188252.p1_type_complete_len_870 | KZS13472.1Vacuolar protein sorting-associated protein 41 | C:GO:0005768; P:GO:0006810; C:GO:0016020; C:GO:0043231 | |
| TRINITY_DN27353_c0_g1_i1_len_2870_188745.p1_type_5prime_partial_len_870 | XP_019877420.1  DNA topoisomerase 3-beta-1 | | |
| 2370\|_\|c13229_f2p1_2609_isoform_c13229_full_length_coverage_2_non_full_length_coverage_1_isoform_length_2609_22197 | XP_018017683.1PREDICTED: uncharacterized protein LOC108674257 | | |
| 2371\|_\|c27312_f2p1_2608_isoform_c27312_full_length_coverage_2_non_full_length_coverage_1_isoform_length_2608_23038 | XP_018025223.1 histone-lysine N-methyltransferase NSD2-like isoform X2 | | |
| 2372\|_\|c6316_f3p2_2608_isoform_c6316_full_length_coverage_3_non_full_length_coverage_2_isoform_length_2608_12544 | ACM47362.1Sox14 protein | F:GO:0003677; C:GO:0005634 | |
| TRINITY_DN26304_c0_g1_i1_len_367_42768.p1_type_internal_len_123 | ---NA--- | | |
| TRINITY_DN11716_c0_g1_i1_len_368_247865.p1_type_internal_len_123 | ---NA--- | | |
| TRINITY_DN33194_c0_g1_i1_len_368_247860.p4_type_internal_len_123 | CAD44571.1runt protein 1b | F:GO:0003677; F:GO:0003700; F:GO:0005524; C:GO:0005634; P:GO:0006355 | |
| TRINITY_DN26275_c0_g1_i2_len_2241_190125.p3_type_complete_len_122 | ---NA--- | | |
| TRINITY_DN6282_c0_g1_i1_len_369_247825.p1_type_internal_len_123 | ODM95427.1Trans-1,2-dihydrobenzene-1,2-diol dehydrogenase | F:GO:0016491; P:GO:0055114 | |
| TRINITY_DN28386_c0_g1_i1_len_368_280262.p1_type_internal_len_123 | ---NA--- | | |
| TRINITY_DN13673_c0_g1_i1_len_369_247801.p1_type_internal_len_123 | ---NA--- | | |
| TRINITY_DN6218_c0_g1_i1_len_369_247800.p1_type_internal_len_123 | ---NA--- | | |
| TRINITY_DN32009_c0_g1_i1_len_661_200595.p1_type_5prime_partial_len_122 | ---NA--- | | |

| | | | |
|---|---|---|---|
| TRINITY_DN36469_c0_g1_i1_len_368_247845.p2_type_3prime_partial_len_123 | AGY46351.1domeless | C:GO:0016021 | |
| TRINITY_DN21854_c0_g2_i1_len_524_100767.p1_type_5prime_partial_len_122 | ---NA--- | | |
| TRINITY_DN33063_c0_g1_i1_len_368_247843.p1_type_internal_len_123 | ---NA--- | | |
| TRINITY_DN6838_c0_g1_i1_len_594_201895.p2_type_complete_len_122 | ---NA--- | | |
| TRINITY_DN38162_c0_g1_i1_len_366_209771.p1_type_internal_len_123 | ---NA--- | | |
| TRINITY_DN4075_c0_g1_i1_len_366_280363.p1_type_internal_len_123 | ---NA--- | | |
| TRINITY_DN15033_c0_g1_i1_len_366_136859.p1_type_internal_len_123 | ---NA--- | | |
| TRINITY_DN8315_c0_g1_i1_len_481_204827.p1_type_5prime_partial_len_122 | ---NA--- | | |
| TRINITY_DN22640_c0_g1_i1_len_368_13029.p1_type_internal_len_123 | ADK12657.1ryanodine receptor | F:GO:0005219; F:GO:0005509; C:GO:0005623; C:GO:0016021; P:GO:0051209 | |
| TRINITY_DN36544_c0_g1_i1_len_546_241868.p1_type_3prime_partial_len_123 | XP_013783646.1zinc finger protein 704-like isoform X2 | | |
| TRINITY_DN23127_c0_g1_i1_len_3388_57968.p2_type_complete_len_122 | ---NA--- | | |
| TRINITY_DN24186_c0_g1_i1_len_368_13037.p1_type_internal_len_123 | ACP19560.1masquerade-like serine proteinase-like protein 2 | F:GO:0004252; P:GO:0006508 | EC:3.4.21 |
| TRINITY_DN11324_c0_g1_i2_len_492_204506.p1_type_5prime_partial_len_122 | ---NA--- | | |
| TRINITY_DN11722_c0_g1_i1_len_370_42660.p1_type_internal_len_123 | ---NA--- | | |
| TRINITY_DN14615_c0_g1_i1_len_1519_157725.p2_type_3prime_partial_len_123 | ---NA--- | | |
| TRINITY_DN16353_c0_g1_i2_len_588_99625.p1_type_5prime_partial_len_122 | ---NA--- | | |

| | | | |
|---|---|---|---|
| TRINITY_DN8509_c0_g1_i1_len_366_248005.p1_type_internal_len_123 | XP_021941381.1sialin isoform X2 | | |
| TRINITY_DN31097_c3_g8_i1_len_399_208033.p1_type_5prime_partial_len_122 | ---NA--- | | |
| TRINITY_DN8741_c0_g1_i1_len_454_205799.p1_type_5prime_partial_len_122 | ---NA--- | | |
| TRINITY_DN30880_c0_g1_i1_len_367_280321.p1_type_internal_len_123 | XP_018024062.1 kinase D-interacting substrate of 220 kDa-like isoform X2 | | |
| TRINITY_DN22546_c0_g1_i1_len_367_280319.p1_type_internal_len_123 | ---NA--- | | |
| TRINITY_DN23385_c1_g1_i1_len_1471_193242.p1_type_complete_len_122 | ---NA--- | | |
| TRINITY_DN18664_c0_g1_i1_len_366_209741.p1_type_internal_len_123 | ---NA--- | | |
| TRINITY_DN23193_c0_g2_i3_len_484_132761.p1_type_complete_len_122 | ---NA--- | | |
| TRINITY_DN19073_c0_g1_i2_len_1307_32091.p2_type_5prime_partial_len_122 | ---NA--- | | |
| TRINITY_DN1261_c0_g1_i1_len_366_209745.p1_type_internal_len_123 | ---NA--- | | |
| TRINITY_DN9702_c0_g1_i1_len_510_242777.p1_type_5prime_partial_len_122 | XP_021961592.1mitochondrial ornithine transporter 1-like | F:GO:0000064; C:GO:0016021; P:GO:1990575 | |
| TRINITY_DN24685_c0_g1_i1_len_366_280344.p1_type_internal_len_123 | XP_026320269.1zinc finger protein 40 isoform X2 | | |
| TRINITY_DN37038_c0_g1_i1_len_453_205862.p1_type_5prime_partial_len_122 | ---NA--- | | |
| TRINITY_DN30252_c0_g1_i1_len_4409_187358.p3_type_3prime_partial_len_123 | ---NA--- | | |
| TRINITY_DN10784_c0_g1_i1_len_503_204129.p1_type_5prime_partial_len_122 | ---NA--- | | |
| TRINITY_DN9214_c0_g1_i1_len_459_9832.p2_type_5prime_partial_len_122 | ---NA--- | | |

| | | | |
|---|---|---|---|
| TRINITY_DN28879_c0_g1_i1_len_367_73836.p2_type_internal_len_123 | ---NA--- | | |
| TRINITY_DN18315_c0_g2_i1_len_958_196918.p2_type_5prime_partial_len_122 | ---NA--- | | |
| TRINITY_DN2525_c0_g1_i1_len_423_207026.p1_type_5prime_partial_len_122 | ---NA--- | | |
| TRINITY_DN35687_c0_g1_i1_len_367_73841.p1_type_internal_len_123 | ---NA--- | | |
| TRINITY_DN22274_c0_g1_i1_len_368_73798.p2_type_internal_len_123 | ---NA--- | | |
| TRINITY_DN35681_c0_g1_i1_len_367_73825.p2_type_internal_len_123 | ---NA--- | | |
| TRINITY_DN14083_c0_g1_i1_len_369_171534.p2_type_internal_len_123 | ---NA--- | | |
| TRINITY_DN28745_c0_g1_i1_len_368_73818.p1_type_internal_len_123 | ---NA--- | | |
| TRINITY_DN39145_c0_g1_i1_len_439_206392.p2_type_5prime_partial_len_122 | PNF29295.1Aldo-keto reductase family 1 member B10 | F:GO:0016491; P:GO:0055114 | |
| TRINITY_DN25739_c0_g1_i1_len_1032_196283.p1_type_complete_len_122 | ---NA--- | | |
| TRINITY_DN18280_c0_g1_i1_len_1899_191264.p1_type_5prime_partial_len_122 | ---NA--- | | |
| TRINITY_DN18864_c0_g2_i1_len_374_73500.p1_type_5prime_partial_len_122 | ---NA--- | | |
| TRINITY_DN4779_c0_g1_i1_len_449_102687.p1_type_5prime_partial_len_122 | XP_018026869.1PREDICTED: uncharacterized protein LOC108682249 | C:GO:0016020; C:GO:0016021 | |
| TRINITY_DN7313_c0_g1_i1_len_367_73846.p1_type_internal_len_123 | ---NA--- | | |
| TRINITY_DN7209_c0_g1_i1_len_406_41282.p2_type_5prime_partial_len_122 | XP_018024341.1 disks large 1 tumor suppressor protein-like | | |
| TRINITY_DN8902_c0_g1_i1_len_368_171612.p1_type_internal_len_123 | ---NA--- | | |

| | | | |
|---|---|---|---|
| TRINITY_DN12161_c0_g1_i1_len_370_209497.p1_type_internal_len_123 | AJO70197.1sodium-calcium exchanger 3 | F:GO:0005432; P:GO:0006816; P:GO:0007154; C:GO:0016021; P:GO:0035725 | |
| TRINITY_DN8866_c0_g1_i1_len_367_209689.p1_type_internal_len_123 | ---NA--- | | |
| TRINITY_DN18434_c0_g1_i1_len_367_209696.p1_type_internal_len_123 | ---NA--- | | |
| TRINITY_DN25582_c0_g1_i1_len_369_73720.p1_type_internal_len_123 | ---NA--- | | |
| TRINITY_DN19869_c0_g1_i1_len_892_197578.p2_type_5prime_partial_len_122 | XP_018008465.1 lysine histidine transporter 2-like | | |
| TRINITY_DN10646_c0_g1_i1_len_367_209701.p1_type_internal_len_123 | ---NA--- | | |
| TRINITY_DN16667_c0_g1_i1_len_1079_235528.p1_type_5prime_partial_len_122 | ---NA--- | | |
| TRINITY_DN17764_c0_g1_i1_len_406_135145.p1_type_3prime_partial_len_123 | ---NA--- | | |
| TRINITY_DN9002_c0_g1_i2_len_596_165054.p2_type_5prime_partial_len_122 | ---NA--- | | |
| TRINITY_DN4893_c0_g1_i1_len_367_209691.p1_type_internal_len_123 | ---NA--- | | |
| TRINITY_DN31421_c0_g1_i1_len_370_209538.p1_type_internal_len_123 | ---NA--- | | |
| TRINITY_DN13745_c0_g1_i1_len_386_307557.p1_type_5prime_partial_len_122 | XP_018021203.1 crossover junction endonuclease MUS81-like | F:GO:0003677; F:GO:0004519; P:GO:0090305 | |
| TRINITY_DN31212_c0_g1_i2_len_2693_189048.p1_type_complete_len_122 | ---NA--- | | |
| TRINITY_DN39036_c0_g1_i1_len_369_209570.p1_type_internal_len_123 | ---NA--- | | |
| TRINITY_DN6502_c0_g1_i1_len_547_202965.p1_type_5prime_partial_len_122 | ---NA--- | | |
| TRINITY_DN12514_c0_g1_i1_len_768_199006.p2_type_complete_len_122 | ---NA--- | | |

| | | | |
|---|---|---|---|
| TRINITY_DN31298_c0_g1_i1_len_369_209558.p1_type_internal_len_123 | OWF35392.1Tetratricopeptide repeat protein 17 | | |
| TRINITY_DN27912_c0_g1_i1_len_370_171496.p1_type_internal_len_123 | XP_023723189.1OTU domain-containing protein 7B-like isoform X1 | F:GO:0005488 | |
| TRINITY_DN17097_c0_g1_i1_len_422_103530.p2_type_5prime_partial_len_122 | PNF44055.1Histone H2A | C:GO:0000786; F:GO:0003677; C:GO:0005634; F:GO:0046982 | |
| TRINITY_DN34632_c0_g1_i1_len_368_73779.p1_type_internal_len_123 | XP_018008089.1 huntingtin-interacting protein 1-like | F:GO:0003779; F:GO:0005543; P:GO:0006897; F:GO:0030276 | |
| TRINITY_DN26706_c0_g1_i4_len_2669_189089.p4_type_complete_len_122 | ---NA--- | | |
| TRINITY_DN23331_c2_g1_i1_len_367_209702.p2_type_internal_len_123 | ---NA--- | | |
| TRINITY_DN2917_c0_g1_i1_len_367_209688.p1_type_internal_len_123 | ---NA--- | | |
| TRINITY_DN30820_c0_g1_i1_len_369_73742.p1_type_internal_len_123 | ---NA--- | | |
| TRINITY_DN26755_c0_g1_i1_len_635_164459.p1_type_5prime_partial_len_122 | BAW78903.1single VWC domain protein 6 | | |
| TRINITY_DN937_c0_g1_i1_len_373_308114.p1_type_5prime_partial_len_122 | ---NA--- | | |
| TRINITY_DN17233_c0_g1_i1_len_480_204892.p1_type_5prime_partial_len_122 | ---NA--- | | |
| TRINITY_DN11299_c0_g1_i1_len_434_206565.p1_type_5prime_partial_len_122 | ---NA--- | | |
| TRINITY_DN15153_c0_g1_i1_len_803_271735.p2_type_5prime_partial_len_122 | ---NA--- | | |
| TRINITY_DN31735_c0_g1_i1_len_369_209610.p1_type_internal_len_123 | XP_017858565.1 patatin-like phospholipase domain-containing protein 3 isoform X2 | P:GO:0006629; P:GO:0016042; F:GO:0016787 | |
| TRINITY_DN14040_c0_g1_i1_len_369_209604.p1_type_internal_len_123 | XP_018019423.1PREDICTED: uncharacterized protein LOC108675887 | | |
| TRINITY_DN33063_c0_g1_i1_len_368_209620.p1_type_internal_len_123 | ---NA--- | | |

| | | | |
|---|---|---|---|
| TRINITY_DN1512_c0_g1_i1_len_368_209663.p1_type_internal_len_123 | ---NA--- | | |
| TRINITY_DN7159_c0_g1_i1_len_369_209601.p1_type_internal_len_123 | XP_014670462.1 protein FAM102A-like | | |
| TRINITY_DN37239_c0_g1_i1_len_369_209602.p1_type_internal_len_123 | XP_023718871.1multidrug resistance-associated protein 5-like isoform X2 | F:GO:0005524; C:GO:0016021; F:GO:0042626; P:GO:0055085 | EC:3.6.1.3; EC:3.6.1.15 |
| TRINITY_DN37842_c0_g1_i1_len_539_203178.p2_type_complete_len_122 | ---NA--- | | |
| TRINITY_DN34078_c0_g1_i1_len_368_209653.p1_type_internal_len_123 | ---NA--- | | |
| TRINITY_DN15021_c0_g1_i1_len_745_162981.p2_type_5prime_partial_len_122 | ---NA--- | | |
| TRINITY_DN17943_c0_g1_i2_len_421_71533.p1_type_5prime_partial_len_122 | EGD79843.16-pyruvoyl tetrahydrobiopterin synthase | F:GO:0003874; P:GO:0006729; F:GO:0046872 | |
| TRINITY_DN22852_c0_g1_i1_len_3286_228677.p2_type_complete_len_122 | ---NA--- | | |
| TRINITY_DN40875_c0_g1_i1_len_368_209654.p1_type_internal_len_123 | XP_023346886.1ras-related protein Rab-28-like | F:GO:0003924; F:GO:0005525 | |
| TRINITY_DN3184_c0_g1_i1_len_417_103738.p1_type_5prime_partial_len_122 | ---NA--- | | |
| TRINITY_DN39662_c0_g1_i1_len_368_209656.p1_type_internal_len_123 | ---NA--- | | |
| TRINITY_DN25675_c0_g1_i6_len_5010_227780.p1_type_complete_len_1518 | XP_018025468.1 CLIP-associating protein 2-like isoform X2 | | |
| TRINITY_DN29266_c0_g1_i1_len_4745_187219.p1_type_5prime_partial_len_1517 | XP_022904556.1myotubularin-related protein 13 isoform X3 | | |
| TRINITY_DN30153_c0_g1_i5_len_5327_187051.p1_type_5prime_partial_len_1517 | XP_018010243.1 tyrosine-protein kinase Abl-like | | |
| 253\|_\|c3798_f3p12_4550_isoform_c3798_full_length_coverage_3_non_full_length_coverage_12_isoform_length_4550_25697 | XP_018021212.1 LIM and SH3 domain protein 1-like | | |
| TRINITY_DN29709_c0_g1_i1_len_5478_187018.p1_type_complete_len_1515 | XP_021916393.1ral GTPase-activating protein subunit beta isoform X10 | | |

| | | | |
|---|---|---|---|
| TRINITY_DN31077_c0_g1_i1_len_4682_187240.p1_type_5prime_partial_len_1514 | ---NA--- | | |
| TRINITY_DN20195_c0_g1_i1_len_5408_227708.p1_type_5prime_partial_len_1514 | XP_023706406.1bromodomain adjacent to zinc finger domain protein 1A-like isoform X1 | C:GO:0005634; F:GO:0046872 | |
| TRINITY_DN23116_c0_g1_i1_len_4834_227832.p1_type_complete_len_1513 | KFM65608.1Zinc finger protein 84 | F:GO:0003676; F:GO:0003677 | |
| TRINITY_DN29732_c0_g1_i3_len_5094_187100.p1_type_complete_len_1511 | XP_014469894.1 ataxin-2-like protein | | |
| TRINITY_DN26339_c0_g1_i1_len_5724_227649.p1_type_5prime_partial_len_1509 | XP_018015696.1 condensin complex subunit 1-like isoform X2 | P:GO:0009987 | |
| 2373\|_\|c2701_f2p2_2608_isoform_c2701_full_length_coverage_2_non_full_length_coverage_2_isoform_length_2608_12087 | XP_018015409.1 AP-3 complex subunit delta-1-like isoform X1 | | |
| TRINITY_DN24847_c0_g1_i5_len_4489_187327.p1_type_complete_len_869 | XP_026288287.1transcriptional repressor p66-alpha isoform X1 | | |
| TRINITY_DN26665_c0_g1_i1_len_3143_188318.p1_type_5prime_partial_len_869 | PNF16968.1Regulatory-associated protein of mTOR | P:GO:0031929; C:GO:0031931 | |
| 2376\|_\|c2394_f3p2_2607_isoform_c2394_full_length_coverage_3_non_full_length_coverage_2_isoform_length_2607_20905 | XP_013406712.1sec1 family domain-containing protein 2 isoform X2 | P:GO:0006904; P:GO:0016192 | |
| TRINITY_DN25172_c0_g1_i1_len_2609_229591.p1_type_internal_len_870 | XP_018016130.1 serine-rich adhesin for platelets-like | | |
| 2378\|_\|c17859_f2p0_2606_isoform_c17859_full_length_coverage_2_non_full_length_coverage_0_isoform_length_2606_22519 | XP_018011297.1 hyaluronan mediated motility receptor-like | | |
| 2379\|_\|c5753_f2p2_2606_isoform_c5753_full_length_coverage_2_non_full_length_coverage_2_isoform_length_2606_12501 | XP_018019951.1 ran-binding protein 3-like isoform X2 | | |
| 2380\|_\|c17867_f2p5_2605_isoform_c17867_full_length_coverage_2_non_full_length_coverage_5_isoform_length_2605_22526 | ---NA--- | | |

| | | | |
|---|---|---|---|
| 2381\|_\|c1969_f8p4_2605_isoform_c1969_full_length_coverage_8_non_full_length_coverage_4_isoform_length_2605_11591 | PSN37237.1Anaphase-promoting complex subunit 4 | C:GO:0005680; P:GO:0030071; P:GO:0031145; P:GO:0070979 | |
| 2382\|_\|c18178_f3p0_2605_isoform_c18178_full_length_coverage_3_non_full_length_coverage_0_isoform_length_2605_18010 | XP_011418271.1 structural maintenance of chromosomes protein 6 isoform X1 | | |
| TRINITY_DN14559_c0_g1_i1_len_1546_1554.p2_type_5prime_partial_len_122 | ---NA--- | | |
| TRINITY_DN4820_c0_g1_i1_len_521_166595.p1_type_5prime_partial_len_122 | ---NA--- | | |
| TRINITY_DN30650_c0_g1_i2_len_1933_191146.p1_type_3prime_partial_len_123 | ---NA--- | | |
| TRINITY_DN14910_c0_g1_i3_len_956_3853.p1_type_5prime_partial_len_122 | ---NA--- | | |
| TRINITY_DN20615_c0_g1_i1_len_368_209618.p1_type_internal_len_123 | ---NA--- | | |
| TRINITY_DN41531_c0_g1_i1_len_368_209645.p1_type_internal_len_123 | XP_019875092.1 protein unc-79 homolog | | |
| TRINITY_DN28966_c0_g1_i1_len_367_171648.p1_type_internal_len_123 | ---NA--- | | |
| TRINITY_DN31858_c0_g1_i1_len_622_201365.p1_type_5prime_partial_len_122 | ---NA--- | | |
| TRINITY_DN27314_c0_g1_i1_len_367_171658.p1_type_internal_len_123 | OWR51737.1putative protein-O-xylosyltransferase | P:GO:0006024; F:GO:0008375; C:GO:0016020; C:GO:0016021; F:GO:0030158 | |
| TRINITY_DN6813_c0_g1_i1_len_367_171653.p1_type_internal_len_123 | ---NA--- | | |
| TRINITY_DN33830_c0_g1_i1_len_367_209672.p1_type_internal_len_123 | ---NA--- | | |
| TRINITY_DN7774_c0_g1_i2_len_366_73917.p1_type_internal_len_123 | ---NA--- | | |
| TRINITY_DN30113_c0_g1_i1_len_2890_188717.p1_type_5prime_partial_len_122 | XP_008483966.1heat shock factor-binding protein 1-like | F:GO:0003714; P:GO:1903507 | |

| | | | |
|---|---|---|---|
| TRINITY_DN26145_c3_g1_i2_len_1358_233733.p2_type_5prime_partial_len_122 | ---NA--- | | |
| TRINITY_DN11420_c0_g1_i1_len_367_209679.p1_type_internal_len_123 | ---NA--- | | |
| TRINITY_DN2990_c1_g1_i1_len_367_209680.p1_type_internal_len_123 | PFX11949.1Retrovirus-related Pol polyprotein from transposon opus | | |
| TRINITY_DN19387_c0_g1_i2_len_2584_189261.p4_type_5prime_partial_len_122 | ---NA--- | | |
| TRINITY_DN33902_c0_g1_i1_len_368_209649.p1_type_internal_len_123 | ---NA--- | | |
| TRINITY_DN14011_c0_g1_i1_len_366_171683.p1_type_internal_len_123 | GBC34766.1gag-pol polyprotein | F:GO:0016787 | |
| TRINITY_DN41671_c0_g1_i1_len_368_209648.p1_type_internal_len_123 | XP_013390909.1glucosylceramidase-like | P:GO:0006629; C:GO:0016020; F:GO:0016798 | |
| TRINITY_DN14856_c0_g1_i1_len_368_209664.p1_type_internal_len_123 | ---NA--- | | |
| TRINITY_DN3413_c0_g1_i1_len_422_134580.p1_type_5prime_partial_len_122 | ---NA--- | | |
| TRINITY_DN21766_c0_g1_i1_len_1942_191122.p2_type_5prime_partial_len_122 | ---NA--- | | |
| TRINITY_DN7864_c0_g1_i1_len_365_248014.p1_type_internal_len_122 | ---NA--- | | |
| TRINITY_DN5086_c0_g1_i1_len_490_167313.p1_type_complete_len_121 | XP_022246120.1maternal embryonic leucine zipper kinase-like | | |
| TRINITY_DN40963_c0_g1_i1_len_365_209785.p1_type_internal_len_122 | ACO52050.1Formimidoyltransferase-cyclodeaminase | F:GO:0005542; F:GO:0016740 | |
| TRINITY_DN16507_c0_g1_i1_len_2585_91415.p4_type_complete_len_121 | XP_018026458.1 mitochondrial inner membrane protease subunit 2-like isoform X1 | P:GO:0006465; P:GO:0006627; F:GO:0008236; C:GO:0042720 | |
| TRINITY_DN25078_c0_g1_i1_len_412_306593.p2_type_5prime_partial_len_121 | ALG65274.1glucose transporter 2 | P:GO:0008643; C:GO:0016021; F:GO:0022857; P:GO:0055085 | |
| TRINITY_DN10541_c1_g1_i1_len_414_169687.p1_type_5prime_partial_len_121 | ---NA--- | | |

| | | | |
|---|---|---|---|
| TRINITY_DN15365_c0_g1_i2_len_746_97572.p2_type_complete_len_121 | ---NA--- | | |
| TRINITY_DN37043_c0_g1_i1_len_365_209823.p1_type_internal_len_122 | ---NA--- | | |
| TRINITY_DN25849_c0_g1_i1_len_403_170130.p1_type_5prime_partial_len_121 | ---NA--- | | |
| TRINITY_DN20546_c0_g1_i1_len_705_65610.p1_type_5prime_partial_len_121 | ---NA--- | | |
| TRINITY_DN18539_c0_g1_i1_len_767_199031.p1_type_complete_len_121 | ---NA--- | | |
| TRINITY_DN26445_c0_g1_i1_len_2704_189025.p1_type_complete_len_121 | ---NA--- | | |
| TRINITY_DN17014_c0_g1_i3_len_380_105202.p1_type_complete_len_121 | ---NA--- | | |
| TRINITY_DN35418_c0_g1_i1_len_365_209813.p1_type_internal_len_122 | XP_022245675.1vacuolar protein sorting-associated protein 13D-like | | |
| TRINITY_DN24144_c0_g1_i1_len_4291_228031.p2_type_complete_len_121 | PNF30596.1Core histone macro-H2A.1 | C:GO:0000786; F:GO:0003677; C:GO:0005634; F:GO:0046982 | |
| TRINITY_DN14612_c0_g1_i1_len_779_34963.p1_type_complete_len_121 | XP_018916142.1 mitochondrial pyruvate carrier 2-like | C:GO:0005743; P:GO:0006850 | |
| TRINITY_DN29939_c0_g1_i1_len_571_7470.p1_type_complete_len_121 | ---NA--- | | |
| TRINITY_DN235_c0_g1_i1_len_365_209806.p1_type_internal_len_122 | ---NA--- | | |
| TRINITY_DN1984_c0_g1_i1_len_467_167974.p1_type_complete_len_121 | ---NA--- | | |
| TRINITY_DN9506_c0_g1_i1_len_603_201714.p1_type_5prime_partial_len_121 | ---NA--- | | |
| TRINITY_DN17408_c0_g1_i1_len_367_280307.p1_type_internal_len_122 | ---NA--- | | |
| TRINITY_DN21461_c0_g1_i1_len_1021_235947.p1_type_complete_len_121 | ---NA--- | | |

| | | | |
|---|---|---|---|
| TRINITY_DN18781_c0_g1_i1_len_2332_230164.p2_type_complete_len_121 | BAH98113.1GTP binding protein alpha subunit Gi | F:GO:0003924; F:GO:0005525; P:GO:0007188; F:GO:0031683; F:GO:0046872 | EC:3.6.1.15 |
| TRINITY_DN17011_c0_g1_i1_len_366_247972.p1_type_internal_len_122 | ADK25181.1argonaute 2 | F:GO:0003676; P:GO:0031047 | |
| TRINITY_DN12337_c0_g1_i1_len_366_247971.p1_type_internal_len_122 | ---NA--- | | |
| TRINITY_DN27425_c0_g1_i2_len_1577_192669.p1_type_5prime_partial_len_121 | ---NA--- | | |
| TRINITY_DN3117_c0_g1_i1_len_479_243639.p1_type_5prime_partial_len_121 | ---NA--- | | |
| TRINITY_DN34945_c0_g1_i1_len_365_209787.p1_type_internal_len_122 | ---NA--- | | |
| TRINITY_DN2137_c0_g1_i2_len_1151_195294.p1_type_5prime_partial_len_121 | ---NA--- | | |
| TRINITY_DN12398_c0_g1_i1_len_366_247953.p1_type_internal_len_122 | ---NA--- | | |
| TRINITY_DN20122_c0_g1_i2_len_1793_266800.p1_type_complete_len_121 | ---NA--- | | |
| TRINITY_DN31687_c0_g1_i1_len_365_209792.p1_type_internal_len_122 | KXJ10292.1putative uncharacterized transposon-derived protein F52C9.6 | | |
| TRINITY_DN2990_c0_g1_i1_len_366_209746.p1_type_internal_len_122 | ---NA--- | | |
| TRINITY_DN33020_c0_g1_i1_len_1932_123249.p2_type_complete_len_121 | PSN36202.1putative elongator complex protein 3 | F:GO:0004402; P:GO:0016573; F:GO:0046872; F:GO:0051536 | EC:2.3.1.5; EC:2.3.1.48 |
| TRINITY_DN4472_c0_g1_i2_len_454_205810.p1_type_3prime_partial_len_122 | ---NA--- | | |
| TRINITY_DN11737_c0_g1_i1_len_651_36289.p1_type_complete_len_121 | ---NA--- | | |
| TRINITY_DN36860_c0_g1_i1_len_366_247952.p1_type_internal_len_122 | XP_018023046.1 ras-related protein Rab-32-like isoform X2 | F:GO:0003924; F:GO:0005525; C:GO:0005802; P:GO:0016192; C:GO:0031982 | EC:3.6.1.15 |

| | | | |
|---|---|---|---|
| TRINITY_DN39048_c0_g1_i1_len_369_209573.p1_type_5prime_partial_len_121 | XP_018011718.1 probable E3 ubiquitin-protein ligase makorin-1 | F:GO:0046872 | |
| TRINITY_DN18657_c0_g1_i1_len_456_205736.p2_type_5prime_partial_len_121 | ARS88258.1ATP binding cassette transporter 6 | F:GO:0000166; F:GO:0005524; C:GO:0016020; C:GO:0016021; F:GO:0016887; P:GO:0055085 | |
| TRINITY_DN28789_c0_g1_i1_len_436_168910.p1_type_5prime_partial_len_121 | ---NA--- | | |
| TRINITY_DN25627_c0_g1_i1_len_3129_228841.p4_type_complete_len_121 | ---NA--- | | |
| TRINITY_DN18472_c0_g1_i2_len_365_209801.p1_type_internal_len_122 | ---NA--- | | |
| TRINITY_DN22367_c0_g1_i1_len_1254_194561.p3_type_5prime_partial_len_121 | ---NA--- | | |
| TRINITY_DN7805_c0_g1_i1_len_366_280336.p1_type_internal_len_122 | ---NA--- | | |
| TRINITY_DN25041_c0_g1_i4_len_2229_190164.p4_type_complete_len_121 | ---NA--- | | |
| TRINITY_DN18403_c0_g1_i1_len_429_206757.p1_type_5prime_partial_len_121 | ---NA--- | | |
| TRINITY_DN20204_c1_g1_i1_len_748_199319.p1_type_5prime_partial_len_121 | ---NA--- | | |
| TRINITY_DN40666_c0_g1_i1_len_365_209786.p1_type_internal_len_122 | XP_018010735.1 multiple epidermal growth factor-like domains protein 8 | F:GO:0005509; C:GO:0016020; C:GO:0016021 | |
| TRINITY_DN2121_c0_g1_i1_len_366_247982.p1_type_internal_len_122 | XP_018007045.1 cytosolic purine 5'-nucleotidase-like isoform X2 | | |
| TRINITY_DN35393_c0_g1_i1_len_366_247973.p2_type_internal_len_122 | XP_018017581.1 RNA-binding protein 45-like | F:GO:0003723 | |
| TRINITY_DN31293_c0_g1_i1_len_366_247994.p1_type_internal_len_122 | ---NA--- | | |
| TRINITY_DN35585_c0_g1_i1_len_366_247991.p1_type_internal_len_122 | ---NA--- | | |
| TRINITY_DN20715_c0_g1_i1_len_650_200816.p1_type_5prime_partial_len_121 | PRD20723.1zinc finger protein | F:GO:0003677 | |

| | | | |
|---|---|---|---|
| TRINITY_DN12283_c0_g1_i1_len_366_247988.p2_type_internal_len_122 | XP_018023391.1 ADP-ribosylation factor-like | | |
| TRINITY_DN12150_c0_g1_i1_len_540_203142.p1_type_3prime_partial_len_122 | ---NA--- | | |
| TRINITY_DN7389_c0_g1_i1_len_365_209797.p1_type_internal_len_122 | ---NA--- | | |
| TRINITY_DN15249_c0_g1_i1_len_378_73305.p1_type_5prime_partial_len_121 | AWM95782.1type 1 phosphodiesterase | F:GO:0004114; P:GO:0007165; F:GO:0046872 | EC:3.1.4.17 |
| TRINITY_DN29734_c0_g1_i1_len_365_171733.p1_type_internal_len_122 | ---NA--- | | |
| TRINITY_DN32092_c0_g1_i1_len_363_74100.p1_type_internal_len_122 | XP_018022425.1 kinesin-like protein unc-104 | | |
| TRINITY_DN1093_c0_g1_i1_len_365_171709.p1_type_internal_len_122 | ---NA--- | | |
| TRINITY_DN11397_c0_g1_i1_len_366_209750.p1_type_internal_len_122 | ---NA--- | | |
| TRINITY_DN16424_c0_g1_i1_len_538_68224.p1_type_5prime_partial_len_121 | ---NA--- | | |
| TRINITY_DN28460_c0_g1_i1_len_364_73982.p1_type_internal_len_122 | ---NA--- | | |
| TRINITY_DN31726_c0_g1_i1_len_364_73986.p1_type_3prime_partial_len_122 | ---NA--- | | |
| TRINITY_DN11944_c0_g1_i1_len_365_73963.p1_type_internal_len_122 | XP_018025305.1 sodium/glucose cotransporter 4-like | F:GO:0005412; C:GO:0005886; C:GO:0016021; P:GO:0055085 | |
| TRINITY_DN30025_c0_g1_i1_len_365_171734.p1_type_internal_len_122 | ---NA--- | | |
| TRINITY_DN11451_c0_g1_i1_len_365_73970.p1_type_internal_len_122 | ---NA--- | | |
| TRINITY_DN17921_c0_g1_i1_len_365_171738.p1_type_internal_len_122 | ---NA--- | | |
| TRINITY_DN23881_c0_g1_i1_len_367_171646.p1_type_internal_len_122 | ---NA--- | | |

| | | | |
|---|---|---|---|
| TRINITY_DN3821_c0_g1_i1_len_366_209777.p1_type_internal_len_122 | OPL21697.1tubulin chain alpha-1c | F:GO:0003924; F:GO:0005200; F:GO:0005525; C:GO:0005737; C:GO:0005874; P:GO:0007010; P:GO:0007017 | EC:3.6.1.15 |
| TRINITY_DN21704_c0_g1_i2_len_600_67090.p1_type_5prime_partial_len_121 | ---NA--- | | |
| TRINITY_DN31718_c0_g1_i1_len_366_171669.p1_type_internal_len_122 | ---NA--- | | |
| TRINITY_DN13956_c0_g1_i1_len_367_136819.p1_type_internal_len_122 | XP_018017486.1 Niemann-Pick C1 protein-like | | |
| TRINITY_DN33947_c0_g1_i1_len_366_171674.p1_type_internal_len_122 | ETE73596.1Lysosomal alpha-glucosidase | F:GO:0003824; F:GO:0004553; P:GO:0005975; F:GO:0030246; P:GO:0042981 | |
| TRINITY_DN15550_c0_g1_i5_len_375_247495.p1_type_5prime_partial_len_121 | ---NA--- | | |
| TRINITY_DN4792_c0_g1_i1_len_367_136843.p1_type_internal_len_122 | ---NA--- | | |
| TRINITY_DN24559_c0_g1_i1_len_366_171660.p1_type_internal_len_122 | ---NA--- | | |
| TRINITY_DN25020_c0_g1_i1_len_2835_229233.p1_type_complete_len_868 | KDR12431.1NMDA receptor-regulated protein 2 | C:GO:0008023 | |
| TRINITY_DN30914_c0_g1_i1_len_3066_188429.p1_type_complete_len_868 | APF30033.1ionotropic glutamate receptor NMDA 2-like protein | F:GO:0004970; C:GO:0016021; C:GO:0030054; P:GO:0034220; P:GO:0035235; C:GO:0045211 | |
| TRINITY_DN8506_c0_g1_i1_len_2798_229283.p1_type_5prime_partial_len_868 | XP_018009919.1 tetratricopeptide repeat protein 27-like | | |
| TRINITY_DN23487_c0_g1_i2_len_6983_153364.p2_type_5prime_partial_len_868 | ---NA--- | | |
| 2387\|_\|c2815_f3p1_2604_isoform_c2815_full_length_coverage_3_non_full_length_coverage_1_isoform_length_2604_12186 | ACY82398.1integrin | P:GO:0007155; P:GO:0007229; C:GO:0016021 | |

| | | | |
|---|---|---|---|
| 2389\|_\|c12186_f2p0_2603_isoform_c12186_full_length_coverage_2_non_full_length_coverage_0_isoform_length_2603_13044 | ---NA--- | | |
| 2390\|_\|c2295_f5p9_2603_isoform_c2295_full_length_coverage_5_non_full_length_coverage_9_isoform_length_2603_11782 | ---NA--- | | |
| 2391\|_\|c18721_f2p2_2603_isoform_c18721_full_length_coverage_2_non_full_length_coverage_2_isoform_length_2603_18106 | ---NA--- | | |
| 2392\|_\|c12018_f2p2_2602_isoform_c12018_full_length_coverage_2_non_full_length_coverage_2_isoform_length_2602_22099 | ---NA--- | | |
| 2394\|_\|c2764_f6p1_2601_isoform_c2764_full_length_coverage_6_non_full_length_coverage_1_isoform_length_2601_16681 | ---NA--- | | |
| TRINITY_DN26526_c0_g1_i1_len_1714_192027.p2_type_complete_len_121 | AYD41600.1B-cell receptor-associated protein 31-like protein | C:GO:0005783; P:GO:0006886; C:GO:0016021 | |
| TRINITY_DN9778_c0_g1_i1_len_365_105864.p1_type_internal_len_122 | ---NA--- | | |
| TRINITY_DN25137_c0_g1_i1_len_365_105850.p1_type_internal_len_122 | ACU31809.1alpha2 macroglobulin isoform 3 | C:GO:0005576 | |
| TRINITY_DN29005_c0_g1_i1_len_2608_189213.p2_type_complete_len_121 | XP_018027866.1PREDICTED: uncharacterized protein LOC108683095 | | |
| TRINITY_DN24248_c0_g1_i1_len_366_105841.p1_type_internal_len_122 | ---NA--- | | |
| TRINITY_DN6987_c0_g1_i1_len_365_105888.p1_type_internal_len_122 | PNF20553.1H(+)/Cl(-) exchange transporter 3 | F:GO:0005247; C:GO:0016021; P:GO:1902476 | |
| TRINITY_DN1537_c0_g1_i1_len_365_105879.p1_type_internal_len_122 | ---NA--- | | |
| TRINITY_DN4478_c0_g1_i1_len_367_105779.p1_type_internal_len_122 | APG53781.1transient receptor potential cation channel subfamily M-like protein | P:GO:0006811; C:GO:0016020; P:GO:0055085 | |
| TRINITY_DN31949_c0_g1_i1_len_367_105766.p1_type_internal_len_122 | ---NA--- | | |

| | | | |
|---|---|---|---|
| TRINITY_DN8606_c0_g1_i1_len_366_105828.p1_type_internal_len_122 | ---NA--- | | |
| TRINITY_DN26699_c0_g1_i1_len_372_136608.p1_type_5prime_partial_len_121 | ---NA--- | | |
| TRINITY_DN23182_c0_g1_i1_len_366_105827.p1_type_internal_len_122 | ---NA--- | | |
| TRINITY_DN39528_c0_g1_i1_len_373_209343.p1_type_5prime_partial_len_121 | ---NA--- | | |
| TRINITY_DN30885_c0_g1_i1_len_363_74044.p1_type_internal_len_122 | ---NA--- | | |
| TRINITY_DN30689_c0_g1_i2_len_2591_189242.p2_type_complete_len_121 | ---NA--- | | |
| TRINITY_DN34553_c0_g1_i1_len_363_171837.p1_type_internal_len_122 | ACO83357.1heat shock protein 90 | F:GO:0005524; P:GO:0006457; F:GO:0051082 | |
| TRINITY_DN21158_c0_g2_i1_len_364_74006.p1_type_internal_len_122 | ---NA--- | | |
| TRINITY_DN37980_c0_g1_i1_len_482_204787.p1_type_5prime_partial_len_121 | ---NA--- | | |
| TRINITY_DN40382_c0_g1_i1_len_366_209772.p1_type_internal_len_122 | ---NA--- | | |
| TRINITY_DN15706_c0_g1_i1_len_363_171801.p1_type_internal_len_122 | ---NA--- | | |
| TRINITY_DN13958_c0_g1_i1_len_578_7344.p2_type_complete_len_121 | XP_018025541.1 glutaminase liver isoform, mitochondrial-like | | |
| TRINITY_DN4924_c0_g1_i1_len_364_105911.p1_type_internal_len_122 | ---NA--- | | |
| TRINITY_DN8194_c0_g1_i1_len_364_74014.p1_type_internal_len_122 | ---NA--- | | |
| TRINITY_DN3431_c0_g1_i1_len_363_105950.p1_type_internal_len_122 | XP_004929161.1BTB/POZ domain-containing protein 9 isoform X2 | | |
| TRINITY_DN25624_c0_g2_i1_len_427_245382.p1_type_5prime_partial_len_121 | ---NA--- | | |

| | | | |
|---|---|---|---|
| TRINITY_DN1356_c0_g1_i1_len_366_209765.p1_type_internal_len_122 | XP_023215648.1probable Ras GTPase-activating protein | | |
| TRINITY_DN13592_c0_g1_i1_len_366_209747.p1_type_internal_len_122 | ---NA--- | | |
| TRINITY_DN16929_c0_g1_i2_len_582_130675.p2_type_5prime_partial_len_121 | ---NA--- | | |
| TRINITY_DN14338_c0_g1_i2_len_423_245537.p1_type_5prime_partial_len_121 | ---NA--- | | |
| TRINITY_DN41470_c0_g1_i1_len_366_209782.p1_type_internal_len_122 | XP_018011335.1 serine/threonine-protein kinase DCLK1-like | | |
| TRINITY_DN35560_c0_g1_i1_len_425_206916.p1_type_3prime_partial_len_122 | ---NA--- | | |
| TRINITY_DN20105_c0_g1_i1_len_1086_235486.p2_type_complete_len_121 | ---NA--- | | |
| TRINITY_DN6739_c0_g1_i1_len_425_245453.p1_type_3prime_partial_len_122 | ---NA--- | | |
| TRINITY_DN25955_c0_g1_i1_len_397_170335.p1_type_5prime_partial_len_121 | ---NA--- | | |
| TRINITY_DN1792_c0_g1_i1_len_363_42977.p1_type_internal_len_122 | AEX08659.1lipopolysaccharide and beta-1,3-glucan binding protein | F:GO:0001872; F:GO:0004553; C:GO:0005576; P:GO:0005975; P:GO:0045087 | |
| TRINITY_DN14240_c0_g1_i1_len_1302_94021.p2_type_complete_len_121 | ---NA--- | | |
| TRINITY_DN32643_c0_g1_i1_len_376_12692.p1_type_5prime_partial_len_121 | ---NA--- | | |
| TRINITY_DN4354_c0_g1_i1_len_595_130451.p2_type_5prime_partial_len_121 | AEB00643.1glucose transporter 1 | P:GO:0008643; C:GO:0016021; F:GO:0022857; P:GO:0055085 | |
| TRINITY_DN30946_c1_g2_i2_len_2263_190063.p4_type_complete_len_121 | ---NA--- | | |
| TRINITY_DN3112_c0_g1_i1_len_366_209778.p1_type_internal_len_122 | ---NA--- | | |
| TRINITY_DN20455_c0_g1_i1_len_1360_93798.p2_type_complete_len_121 | ---NA--- | | |

| | | | |
|---|---|---|---|
| TRINITY_DN20484_c0_g1_i1_len_364_136969.p1_type_internal_len_122 | ---NA--- | | |
| TRINITY_DN24847_c0_g1_i1_len_366_136904.p1_type_internal_len_122 | ---NA--- | | |
| TRINITY_DN39059_c0_g1_i1_len_366_209756.p1_type_internal_len_122 | XP_018025576.1 intraflagellar transport protein 88 homolog | | |
| TRINITY_DN27644_c0_g1_i1_len_366_136888.p1_type_internal_len_122 | ---NA--- | | |
| TRINITY_DN6424_c0_g1_i1_len_366_136878.p1_type_internal_len_122 | XP_018021514.1 heparan sulfate 2-O-sulfotransferase 1-like | | |
| TRINITY_DN25762_c0_g1_i1_len_365_136942.p1_type_internal_len_122 | ALG65274.1glucose transporter 2 | P:GO:0008643; C:GO:0016021; F:GO:0022857; P:GO:0055085 | |
| TRINITY_DN2374_c0_g1_i1_len_365_136935.p1_type_internal_len_122 | ---NA--- | | |
| TRINITY_DN26447_c0_g1_i1_len_365_136923.p1_type_internal_len_122 | ---NA--- | | |
| TRINITY_DN33402_c0_g1_i1_len_426_206870.p1_type_3prime_partial_len_122 | ---NA--- | | |
| TRINITY_DN4421_c0_g1_i1_len_364_137005.p1_type_internal_len_122 | XP_023719832.1rho guanine nucleotide exchange factor 17-like | F:GO:0005089; P:GO:0035023 | |
| TRINITY_DN26133_c0_g1_i1_len_1742_191897.p2_type_complete_len_121 | KFV91237.1Serine/threonine-protein kinase greatwall | F:GO:0016301 | |
| TRINITY_DN19660_c0_g1_i1_len_723_199680.p1_type_3prime_partial_len_122 | ---NA--- | | |
| TRINITY_DN12999_c0_g1_i1_len_524_275500.p2_type_5prime_partial_len_121 | XP_022186264.1zinc finger protein 239-like | | |
| TRINITY_DN9287_c0_g1_i1_len_646_98746.p3_type_3prime_partial_len_122 | XP_026674123.1calmodulin-like protein 4 | | |
| TRINITY_DN23841_c0_g1_i1_len_378_307918.p1_type_5prime_partial_len_121 | ---NA--- | | |
| TRINITY_DN3194_c0_g1_i1_len_697_163593.p2_type_5prime_partial_len_121 | ---NA--- | | |

| | | | |
|---|---|---|---|
| TRINITY_DN19525_c0_g1_i1_len_1202_194940.p1_type_5prime_partial_len_121 | ---NA--- | | |
| TRINITY_DN22746_c0_g2_i3_len_1368_193845.p4_type_complete_len_121 | ---NA--- | | |
| TRINITY_DN9582_c0_g1_i1_len_366_209758.p1_type_internal_len_122 | PNF26538.1hypothetical protein B7P43_G13959 | C:GO:0016020 | |
| TRINITY_DN34606_c0_g1_i1_len_663_239750.p2_type_5prime_partial_len_121 | XP_023713435.1zinc finger with UFM1-specific peptidase domain protein-like isoform X2 | F:GO:0003676 | |
| TRINITY_DN13548_c0_g1_i1_len_542_37868.p3_type_5prime_partial_len_121 | ---NA--- | | |
| TRINITY_DN11932_c0_g1_i1_len_1901_92342.p4_type_complete_len_121 | ---NA--- | | |
| TRINITY_DN21780_c0_g1_i1_len_795_198671.p2_type_complete_len_121 | ---NA--- | | |
| TRINITY_DN11257_c0_g1_i2_len_709_239066.p1_type_complete_len_121 | ---NA--- | | |
| TRINITY_DN36522_c0_g1_i1_len_367_209718.p1_type_internal_len_122 | ---NA--- | | |
| TRINITY_DN16074_c0_g1_i2_len_886_161620.p3_type_5prime_partial_len_121 | ---NA--- | | |
| TRINITY_DN13218_c0_g1_i1_len_696_239262.p1_type_5prime_partial_len_121 | ---NA--- | | |
| TRINITY_DN13045_c0_g1_i1_len_895_161536.p1_type_3prime_partial_len_122 | ---NA--- | | |
| TRINITY_DN12573_c0_g1_i1_len_646_200880.p1_type_3prime_partial_len_122 | ---NA--- | | |
| TRINITY_DN17259_c0_g1_i1_len_629_164542.p1_type_5prime_partial_len_121 | ---NA--- | | |
| TRINITY_DN16605_c0_g1_i5_len_474_276689.p1_type_complete_len_121 | ---NA--- | | |

| | | | |
|---|---|---|---|
| TRINITY_DN28755_c0_g1_i1_len_363_280485.p1_type_internal_len_122 | XP_018016477.1PREDICTED: uncharacterized protein LOC108673200 isoform X2 | | |
| TRINITY_DN39848_c0_g1_i1_len_366_209731.p1_type_internal_len_122 | ---NA--- | | |
| TRINITY_DN19710_c0_g4_i2_len_455_277180.p1_type_5prime_partial_len_121 | KRX83908.1hypothetical protein T4E_7988, partial | | |
| TRINITY_DN23758_c0_g1_i1_len_461_277022.p2_type_5prime_partial_len_121 | ---NA--- | | |
| TRINITY_DN17964_c0_g1_i1_len_1193_195017.p3_type_complete_len_121 | XP_018007298.1 PIH1 domain-containing protein 1-like | | |
| TRINITY_DN2802_c0_g1_i1_len_400_72358.p1_type_5prime_partial_len_121 | EFA05430.1Serine/threonine-protein kinase TAO1-like Protein | F:GO:0016301; P:GO:0016310 | |
| TRINITY_DN18150_c0_g1_i1_len_648_239980.p1_type_5prime_partial_len_121 | ---NA--- | | |
| TRINITY_DN9496_c0_g1_i1_len_429_40543.p1_type_3prime_partial_len_122 | ---NA--- | | |
| TRINITY_DN17927_c0_g1_i1_len_991_160739.p1_type_5prime_partial_len_121 | ---NA--- | | |
| TRINITY_DN17623_c0_g1_i1_len_651_200785.p1_type_5prime_partial_len_121 | ---NA--- | | |
| TRINITY_DN12216_c0_g1_i1_len_367_209711.p1_type_internal_len_122 | ---NA--- | | |
| TRINITY_DN18426_c0_g1_i4_len_469_133120.p1_type_3prime_partial_len_122 | ---NA--- | | |
| TRINITY_DN19910_c0_g1_i1_len_1234_268914.p2_type_complete_len_121 | XP_025105539.1 putative glutathione-specific gamma-glutamylcyclotransferase 2 | F:GO:0003839; C:GO:0005737; P:GO:0006751; F:GO:0061928 | EC:4.3.2.7; EC:4.3.2.9 |
| TRINITY_DN30683_c0_g1_i1_len_744_238595.p1_type_5prime_partial_len_121 | ---NA--- | | |
| TRINITY_DN30063_c3_g1_i4_len_1651_192306.p1_type_complete_len_121 | ---NA--- | | |

<table>
<tr><td>TRINITY_DN10611_c0_g1_i1_len_1062_196009.p1_type_complete_len_121</td><td>---NA---</td><td></td><td></td></tr>
<tr><td>TRINITY_DN23041_c0_g1_i2_len_772_238274.p1_type_3prime_partial_len_122</td><td>ABN58714.1pol-like protein</td><td>F:GO:0003824; F:GO:0005488; P:GO:0090304</td><td></td></tr>
<tr><td>TRINITY_DN30364_c0_g1_i1_len_364_13238.p1_type_internal_len_122</td><td>XP_019392221.1 TATA element modulatory factor</td><td></td><td></td></tr>
<tr><td>TRINITY_DN11712_c0_g1_i1_len_366_209760.p1_type_internal_len_122</td><td>---NA---</td><td></td><td></td></tr>
<tr><td>TRINITY_DN3055_c0_g1_i1_len_364_13247.p1_type_internal_len_122</td><td>---NA---</td><td></td><td></td></tr>
<tr><td>TRINITY_DN34908_c0_g1_i1_len_428_71307.p1_type_5prime_partial_len_121</td><td>---NA---</td><td></td><td></td></tr>
<tr><td>TRINITY_DN17797_c0_g1_i1_len_776_238237.p1_type_complete_len_121</td><td>---NA---</td><td></td><td></td></tr>
<tr><td>TRINITY_DN28896_c0_g1_i1_len_364_13233.p1_type_internal_len_122</td><td>XP_012555914.1 transcription factor BYE1-like</td><td>F:GO:0046872</td><td></td></tr>
<tr><td>TRINITY_DN24356_c0_g1_i1_len_792_238046.p1_type_5prime_partial_len_121</td><td>---NA---</td><td></td><td></td></tr>
<tr><td>TRINITY_DN7784_c0_g1_i1_len_529_203438.p1_type_5prime_partial_len_121</td><td>---NA---</td><td></td><td></td></tr>
<tr><td>TRINITY_DN6946_c0_g1_i1_len_396_208214.p1_type_5prime_partial_len_121</td><td>---NA---</td><td></td><td></td></tr>
<tr><td>TRINITY_DN30074_c0_g1_i1_len_365_13184.p3_type_internal_len_122</td><td>XP_018008897.1PREDICTED: uncharacterized protein LOC108666520 isoform X2</td><td></td><td></td></tr>
<tr><td>TRINITY_DN28631_c0_g1_i1_len_365_308477.p1_type_internal_len_122</td><td>XP_018020947.1 dynein intermediate chain 2, ciliary-like</td><td></td><td></td></tr>
<tr><td>2399|_|c2267_f6p25_2600_isoform_c2267_full_length_coverage_6_non_full_length_coverage_25_isoform_length_2600_11766</td><td>AHI85755.1cytoplasmic dynein intermediate chain</td><td>C:GO:0005868; P:GO:0007018</td><td></td></tr>
<tr><td>2400|_|c11371_f3p2_2599_isoform_c11371_full_length_coverage_3_non_full_length_coverage_2_isoform_length_2599_17437</td><td>XP_021365327.1vacuolar fusion protein CCZ1 homolog</td><td></td><td></td></tr>
</table>

| | | | |
|---|---|---|---|
| 2401\|_\|c1465_f3p5_2599_isoform_c1465_full_length_coverage_3_non_full_length_coverage_5_isoform_length_2599_27797 | PNF43639.1CCR4-NOT transcription complex subunit 6-like-B | | |
| 2402\|_\|c2346_f4p6_2598_isoform_c2346_full_length_coverage_4_non_full_length_coverage_6_isoform_length_2598_20869 | XP_018016824.1 dihydrolipoyl dehydrogenase, mitochondrial-like | F:GO:0004148; C:GO:0005623; F:GO:0009055; P:GO:0022900; P:GO:0045454; F:GO:0050660 | EC:1.8.1.4 |
| TRINITY_DN31089_c0_g1_i1_len_4941_187143.p1_type_complete_len_866 | PNF17198.1Transforming growth factor-beta receptor-associated protein 1 | C:GO:0005623; P:GO:0006886; P:GO:0016192 | |
| TRINITY_DN15665_c0_g1_i1_len_4248_153603.p1_type_5prime_partial_len_866 | AGN52246.1female sterile homeotic protein | | |
| 2406\|_\|c13279_f4p9_2597_isoform_c13279_full_length_coverage_4_non_full_length_coverage_9_isoform_length_2597_13126 | ---NA--- | | |
| 2407\|_\|c12347_f2p17_2597_isoform_c12347_full_length_coverage_2_non_full_length_coverage_17_isoform_length_2597_17506 | ABB89491.1forkhead transcription factor N1/4 transcript variant 1 | F:GO:0003700; C:GO:0005634; P:GO:0006355; F:GO:0043565 | |
| 2408\|_\|c2521_f9p5_2595_isoform_c2521_full_length_coverage_9_non_full_length_coverage_5_isoform_length_2595_16478 | PNF17762.1Anaphase-promoting complex subunit 2 | P:GO:0006511; F:GO:0031625 | |
| 2410\|_\|c2108_f5p5_2595_isoform_c2108_full_length_coverage_5_non_full_length_coverage_5_isoform_length_2595_11664 | XP_018025327.1 cell division cycle protein 20 homolog isoform X1 | P:GO:0009987 | |
| TRINITY_DN8313_c0_g1_i1_len_483_39095.p2_type_5prime_partial_len_121 | ---NA--- | | |
| TRINITY_DN30959_c0_g1_i1_len_1211_194866.p2_type_5prime_partial_len_121 | ---NA--- | | |
| TRINITY_DN9130_c0_g1_i1_len_366_308430.p1_type_internal_len_122 | ---NA--- | | |
| TRINITY_DN23430_c0_g1_i1_len_366_308432.p1_type_internal_len_122 | XP_018024610.1 galactosylceramide sulfotransferase-like | | |
| TRINITY_DN22511_c0_g1_i1_len_1911_191219.p2_type_5prime_partial_len_121 | ---NA--- | | |

| | | | |
|---|---|---|---|
| TRINITY_DN5614_c0_g1_i1_len_365_308448.p1_type_internal_len_122 | ---NA--- | | |
| TRINITY_DN21787_c0_g1_i1_len_984_63214.p2_type_5prime_partial_len_121 | ---NA--- | | |
| TRINITY_DN35839_c0_g1_i1_len_365_248064.p1_type_internal_len_122 | ---NA--- | | |
| TRINITY_DN26409_c0_g1_i1_len_537_37962.p2_type_3prime_partial_len_122 | XP_018025794.1 voltage-dependent calcium channel subunit alpha-2/delta-3-like | | |
| TRINITY_DN35606_c0_g1_i1_len_365_248062.p1_type_internal_len_122 | XP_018011187.1 alpha-1,3-mannosyl-glycoprotein 2-beta-N-acetylglucosaminyltransferase-like | F:GO:0003827; C:GO:0005794; P:GO:0006487 | EC:2.4.1.101 |
| TRINITY_DN27818_c0_g1_i1_len_363_248152.p1_type_internal_len_122 | ---NA--- | | |
| TRINITY_DN11069_c0_g1_i1_len_364_280436.p1_type_internal_len_122 | ---NA--- | | |
| TRINITY_DN9098_c0_g1_i1_len_363_248161.p1_type_internal_len_122 | ---NA--- | | |
| TRINITY_DN3499_c0_g1_i1_len_364_209872.p1_type_internal_len_122 | ---NA--- | | |
| TRINITY_DN14454_c0_g1_i1_len_365_248054.p1_type_internal_len_122 | XP_018028171.1 semaphorin-1A-like | | |
| TRINITY_DN28485_c0_g1_i1_len_1602_192551.p1_type_complete_len_121 | ---NA--- | | |
| TRINITY_DN2010_c0_g1_i1_len_365_248051.p3_type_internal_len_122 | ---NA--- | | |
| TRINITY_DN20158_c0_g1_i1_len_2114_230746.p2_type_5prime_partial_len_121 | ---NA--- | | |
| TRINITY_DN5213_c0_g1_i1_len_363_209905.p1_type_internal_len_122 | ---NA--- | | |
| TRINITY_DN14806_c0_g1_i3_len_364_209870.p1_type_internal_len_122 | ---NA--- | | |

| | | | |
|---|---|---|---|
| TRINITY_DN10193_c0_g1_i1_len_364_280407.p3_type_internal_len_122 | ---NA--- | | |
| TRINITY_DN31818_c0_g1_i1_len_366_209739.p1_type_internal_len_122 | ---NA--- | | |
| TRINITY_DN12266_c0_g1_i1_len_364_209885.p2_type_internal_len_122 | ---NA--- | | |
| TRINITY_DN11743_c0_g1_i1_len_363_248134.p1_type_internal_len_122 | AXY87731.1carnitine O-palmitoyltransferase 1b | F:GO:0016746 | |
| TRINITY_DN6638_c0_g1_i1_len_363_209939.p1_type_internal_len_122 | XP_018025771.1 cytoplasmic dynein 2 heavy chain 1-like | C:GO:0005868; C:GO:0005930; F:GO:0008569; C:GO:0031514; P:GO:0035721; F:GO:0045503; F:GO:0045505; F:GO:0051959; P:GO:0060271 | EC:3.6.1.3; EC:3.6.4.5; EC:3.6.1.15 |
| TRINITY_DN12789_c0_g1_i1_len_363_209944.p1_type_internal_len_122 | ---NA--- | | |
| TRINITY_DN1372_c0_g1_i1_len_363_248121.p1_type_internal_len_122 | ---NA--- | | |
| TRINITY_DN35165_c0_g1_i1_len_364_209888.p1_type_internal_len_122 | ---NA--- | | |
| TRINITY_DN23218_c0_g1_i2_len_935_63524.p1_type_5prime_partial_len_121 | ---NA--- | | |
| TRINITY_DN11438_c0_g1_i1_len_392_72688.p1_type_5prime_partial_len_121 | ---NA--- | | |
| TRINITY_DN34146_c0_g1_i1_len_364_209875.p1_type_internal_len_122 | XP_006122343.2peroxisomal membrane protein 2 | C:GO:0016020 | |
| TRINITY_DN18893_c0_g2_i1_len_435_206509.p1_type_3prime_partial_len_122 | ---NA--- | | |
| TRINITY_DN31537_c0_g1_i1_len_364_248114.p1_type_internal_len_122 | ---NA--- | | |
| TRINITY_DN22605_c0_g2_i1_len_906_4126.p2_type_complete_len_121 | ---NA--- | | |
| TRINITY_DN36383_c0_g1_i1_len_365_248031.p1_type_internal_len_122 | ---NA--- | | |

| | | | |
|---|---|---|---|
| TRINITY_DN7907_c0_g1_i1_len_364_209835.p1_type_internal_len_122 | XP_024083164.1myelin regulatory factor isoform X3 | | |
| TRINITY_DN30506_c0_g1_i1_len_364_248095.p2_type_internal_len_122 | ---NA--- | | |
| TRINITY_DN39583_c0_g1_i1_len_363_209896.p1_type_internal_len_122 | ODN04892.1Peroxisomal acyl-coenzyme A oxidase 3 | F:GO:0003997; C:GO:0005777; P:GO:0006635; F:GO:0071949 | EC:1.3.3.6 |
| TRINITY_DN12947_c0_g1_i1_len_364_209836.p1_type_internal_len_122 | ---NA--- | | |
| TRINITY_DN31245_c0_g1_i1_len_365_248033.p1_type_internal_len_122 | ---NA--- | | |
| TRINITY_DN1935_c0_g1_i1_len_438_206430.p1_type_5prime_partial_len_121 | ---NA--- | | |
| TRINITY_DN10133_c0_g1_i1_len_366_280345.p1_type_internal_len_122 | XP_020707262.1glycosaminoglycan xylosylkinase | | |
| TRINITY_DN20617_c0_g1_i1_len_574_202330.p2_type_complete_len_121 | ---NA--- | | |
| TRINITY_DN2014_c0_g1_i1_len_365_209828.p1_type_internal_len_122 | AHH32887.1integrin alpha 4 | P:GO:0007155; P:GO:0007229; C:GO:0008305 | |
| TRINITY_DN10497_c0_g1_i1_len_366_209767.p1_type_internal_len_122 | ---NA--- | | |
| TRINITY_DN39464_c0_g1_i1_len_594_201902.p1_type_5prime_partial_len_121 | XP_018010845.1 B9 domain-containing protein 2-like | | |
| TRINITY_DN35204_c0_g1_i1_len_365_248048.p1_type_internal_len_122 | ---NA--- | | |
| TRINITY_DN1005_c0_g1_i2_len_384_73029.p1_type_5prime_partial_len_121 | XP_015509234.1 probable Ras GTPase-activating protein isoform X5 | | |
| TRINITY_DN13475_c0_g1_i1_len_4038_29258.p2_type_5prime_partial_len_121 | ---NA--- | | |
| TRINITY_DN33782_c0_g1_i1_len_364_209867.p1_type_internal_len_122 | XP_019641338.1 major facilitator superfamily domain-containing protein 10-like | | |
| TRINITY_DN37186_c0_g1_i1_len_364_209864.p1_type_internal_len_122 | ---NA--- | | |

| | | | |
|---|---|---|---|
| TRINITY_DN20152_c1_g1_i1_len_365_248039.p1_type_internal_len_122 | ---NA--- | | |
| TRINITY_DN39564_c0_g1_i1_len_363_209900.p1_type_internal_len_122 | XP_018017486.1 Niemann-Pick C1 protein-like | C:GO:0016020; C:GO:0016021 | |
| TRINITY_DN5963_c0_g1_i1_len_365_248037.p2_type_internal_len_122 | XP_015177358.1 myosin-IIIb-like isoform X5 | | |
| TRINITY_DN19072_c0_g1_i1_len_1450_1811.p3_type_complete_len_121 | ---NA--- | | |
| TRINITY_DN36955_c0_g1_i1_len_366_209770.p1_type_internal_len_122 | ABD65301.1zinc proteinase Mpc1 | F:GO:0004222; P:GO:0006508; F:GO:0008270 | EC:3.4.24 |
| TRINITY_DN42329_c0_g1_i1_len_364_209857.p1_type_internal_len_122 | XP_018027333.1 low-density lipoprotein receptor-related protein 2-like | | |
| TRINITY_DN35572_c0_g1_i1_len_364_209853.p1_type_internal_len_122 | XP_018022134.1 RNA pseudouridylate synthase domain-containing protein 2-like | P:GO:0001522; F:GO:0003723; F:GO:0009982 | |
| TRINITY_DN24241_c0_g1_i1_len_3276_228686.p3_type_complete_len_120 | ---NA--- | | |
| TRINITY_DN8580_c0_g1_i1_len_392_135704.p1_type_5prime_partial_len_120 | XP_019634724.1 gastrula zinc finger protein XlCGF57.1-like | | |
| TRINITY_DN32735_c0_g1_i1_len_364_248076.p1_type_internal_len_121 | BBC20717.1integrin alpha ps | P:GO:0007155; P:GO:0007229; C:GO:0008305 | |
| TRINITY_DN25756_c0_g1_i1_len_2598_229609.p3_type_complete_len_120 | XP_018022215.1 solute carrier family 28 member 3-like | F:GO:0005337; C:GO:0016021; P:GO:1901642 | |
| TRINITY_DN29238_c0_g1_i1_len_363_13276.p1_type_internal_len_121 | XP_022709754.1uncharacterized protein LOC111272519 isoform X2 | | |
| TRINITY_DN1586_c0_g1_i1_len_427_134413.p1_type_5prime_partial_len_120 | ---NA--- | | |
| TRINITY_DN23048_c0_g1_i1_len_2594_229619.p2_type_complete_len_120 | ---NA--- | | |
| TRINITY_DN13605_c0_g1_i1_len_362_248192.p1_type_internal_len_121 | ---NA--- | | |
| TRINITY_DN18730_c0_g1_i1_len_362_13323.p2_type_internal_len_121 | ---NA--- | | |

| | | | |
|---|---|---|---|
| TRINITY_DN20157_c0_g1_i2_len_362_13324.p1_type_internal_len_121 | ---NA--- | | |
| TRINITY_DN21650_c0_g1_i1_len_3060_154267.p3_type_complete_len_120 | ---NA--- | | |
| TRINITY_DN31834_c0_g1_i1_len_362_248184.p1_type_internal_len_121 | ---NA--- | | |
| TRINITY_DN32028_c0_g1_i1_len_362_248191.p1_type_internal_len_121 | XP_014258728.1E3 ubiquitin-protein ligase UBR4 isoform X3 | | |
| TRINITY_DN4710_c0_g1_i1_len_360_248264.p1_type_internal_len_121 | XP_018012267.1 dystrophin, isoforms A/C/F/G/H-like | | |
| TRINITY_DN29418_c0_g1_i1_len_361_13347.p1_type_internal_len_121 | ALH22586.1fibrinogen-related protein isoform 1 | | |
| TRINITY_DN29436_c0_g1_i1_len_360_248256.p2_type_internal_len_121 | XP_018027026.1 glycine receptor subunit alpha-2-like | | |
| TRINITY_DN28914_c0_g1_i1_len_363_280478.p1_type_internal_len_121 | ---NA--- | | |
| TRINITY_DN25769_c0_g1_i1_len_362_13335.p1_type_internal_len_121 | ---NA--- | | |
| TRINITY_DN14190_c0_g1_i2_len_495_132493.p1_type_5prime_partial_len_120 | ---NA--- | | |
| TRINITY_DN33523_c0_g1_i1_len_360_248270.p1_type_internal_len_121 | XP_018024062.1 kinase D-interacting substrate of 220 kDa-like isoform X2 | | |
| TRINITY_DN35654_c0_g1_i1_len_361_248216.p1_type_internal_len_121 | XP_018020234.1 E3 ubiquitin-protein ligase MYCBP2-like | | |
| TRINITY_DN12125_c0_g1_i1_len_361_248218.p1_type_internal_len_121 | ---NA--- | | |
| TRINITY_DN28792_c0_g1_i1_len_363_248133.p1_type_internal_len_121 | XP_018019355.1 myosin-IA-like | F:GO:0003774; F:GO:0003779; F:GO:0005524; C:GO:0016459 | EC:3.6.1.15 |
| TRINITY_DN3067_c0_g1_i1_len_360_13395.p1_type_internal_len_121 | ---NA--- | | |
| TRINITY_DN658_c0_g1_i1_len_360_280634.p1_type_internal_len_121 | OQV23915.1Sorbitol dehydrogenase | F:GO:0046872 | |

| | | | |
|---|---|---|---|
| TRINITY_DN29673_c0_g1_i1_len_3303_188133.p2_type_complete_len_120 | ---NA--- | | |
| TRINITY_DN31930_c0_g1_i1_len_878_197721.p2_type_3prime_partial_len_121 | ---NA--- | | |
| TRINITY_DN21522_c0_g1_i1_len_364_280404.p1_type_internal_len_121 | ---NA--- | | |
| TRINITY_DN27026_c0_g1_i1_len_363_13286.p1_type_internal_len_121 | ASQ43185.1Src | F:GO:0004715; F:GO:0005524; P:GO:0018108 | EC:2.7.10; EC:2.7.10.2 |
| TRINITY_DN1536_c0_g1_i1_len_365_13170.p1_type_5prime_partial_len_120 | ---NA--- | | |
| TRINITY_DN26532_c0_g1_i1_len_363_248122.p1_type_internal_len_121 | ---NA--- | | |
| TRINITY_DN16810_c0_g1_i3_len_363_13291.p1_type_internal_len_121 | ---NA--- | | |
| TRINITY_DN4145_c0_g1_i1_len_362_248179.p1_type_internal_len_121 | ACY44140.1acetyl-CoA carboxylase | F:GO:0005524; F:GO:0046872 | |
| TRINITY_DN20568_c1_g1_i2_len_2766_154617.p3_type_complete_len_120 | ---NA--- | | |
| TRINITY_DN31725_c0_g1_i1_len_362_248180.p1_type_internal_len_121 | XP_018021838.1PREDICTED: uncharacterized protein LOC108678019 | | |
| TRINITY_DN13979_c0_g1_i1_len_1817_30718.p1_type_5prime_partial_len_120 | ---NA--- | | |
| TRINITY_DN27761_c0_g1_i2_len_3116_188356.p2_type_3prime_partial_len_121 | ---NA--- | | |
| TRINITY_DN11018_c0_g1_i1_len_361_280567.p1_type_internal_len_121 | AAX63905.1C-type lectin protein | F:GO:0030246 | |
| TRINITY_DN23875_c0_g1_i1_len_360_280612.p1_type_internal_len_121 | ASW35106.1FH1/FH2 domain-containing protein 1 | P:GO:0007015 | |
| TRINITY_DN9335_c0_g1_i1_len_363_248157.p1_type_internal_len_121 | XP_023222252.1spectrin alpha chain, non-erythrocytic 1-like | | |
| TRINITY_DN14724_c0_g1_i3_len_361_280597.p2_type_internal_len_121 | ---NA--- | | |

| | | | |
|---|---|---|---|
| TRINITY_DN9404_c0_g1_i1_len_561_202641.p2_type_complete_len_120 | KZS09412.1Uncharacterized protein APZ42_026526, partial | F:GO:0003676; P:GO:0015074 | |
| 2411\|_\|c33147_f21p10_2595_isoform_c33147_full_length_coverage_21_non_full_length_coverage_10_isoform_length_2595_14180 | XP_006016862.1ATP-dependent RNA helicase DDX54 | F:GO:0016787; F:GO:0097159; F:GO:1901363 | |
| TRINITY_DN24782_c0_g1_i2_len_3107_58101.p1_type_5prime_partial_len_865 | ACD74843.1trehalose-6-phosphate synthase | F:GO:0003825; P:GO:0005992 | EC:2.4.1.15 |
| 2413\|_\|c17865_f3p0_2595_isoform_c17865_full_length_coverage_3_non_full_length_coverage_0_isoform_length_2595_17824 | PNF33725.1Adapter molecule Crk | | |
| 2415\|_\|c9755_f2p3_2594_isoform_c9755_full_length_coverage_2_non_full_length_coverage_3_isoform_length_2594_12829 | AEU11365.1Broad-complex protein isoform 6 variant 2 | F:GO:0003676 | |
| 2417\|_\|c9343_f3p1_2594_isoform_c9343_full_length_coverage_3_non_full_length_coverage_1_isoform_length_2594_17277 | XP_018026342.1 zinc finger protein 3 homolog | | |
| 2419\|_\|c2416_f2p0_2593_isoform_c2416_full_length_coverage_2_non_full_length_coverage_0_isoform_length_2593_11850 | ---NA--- | | |
| 2420\|_\|c1720_f2p0_2593_isoform_c1720_full_length_coverage_2_non_full_length_coverage_0_isoform_length_2593_27925 | ---NA--- | | |
| TRINITY_DN31227_c0_g1_i1_len_3543_187894.p1_type_5prime_partial_len_864 | XP_018022792.1 probable serine/threonine-protein kinase DDB_G0268876 | F:GO:0016740 | |
| TRINITY_DN19977_c0_g1_i1_len_2673_295.p1_type_5prime_partial_len_864 | XP_018017904.1 sphingomyelin phosphodiesterase 4-like isoform X2 | C:GO:0016020; C:GO:0016021; F:GO:0050290 | |
| TRINITY_DN30436_c0_g1_i1_len_2692_189050.p1_type_5prime_partial_len_864 | XP_021916129.1CWF19-like protein 2 isoform X3 | | |
| TRINITY_DN21692_c0_g1_i2_len_742_238626.p2_type_complete_len_120 | XP_026472186.1BLOC-1-related complex subunit 8 homolog isoform X2 | | |

| | | | |
|---|---|---|---|
| TRINITY_DN15193_c0_g1_i2_len_595_274217.p1_type_5prime_partial_len_120 | ---NA--- | | |
| TRINITY_DN13193_c0_g1_i1_len_589_274303.p1_type_5prime_partial_len_120 | ---NA--- | | |
| TRINITY_DN27676_c0_g1_i1_len_440_206338.p1_type_5prime_partial_len_120 | ---NA--- | | |
| TRINITY_DN18147_c0_g1_i2_len_533_203344.p2_type_3prime_partial_len_121 | ---NA--- | | |
| TRINITY_DN19698_c0_g1_i4_len_908_34028.p2_type_5prime_partial_len_120 | ---NA--- | | |
| TRINITY_DN28576_c0_g1_i1_len_780_238186.p1_type_5prime_partial_len_120 | XP_018024236.1PREDICTED: uncharacterized protein LOC108679998 | | |
| TRINITY_DN32548_c0_g1_i1_len_441_206302.p1_type_5prime_partial_len_120 | ACR23314.1chitinase 4 precursor | F:GO:0004568; C:GO:0005576; P:GO:0005975; P:GO:0006030; F:GO:0008061 | EC:3.2.1.14 |
| TRINITY_DN162_c0_g1_i1_len_1836_231620.p3_type_complete_len_120 | ---NA--- | | |
| TRINITY_DN12265_c0_g1_i1_len_433_40427.p1_type_5prime_partial_len_120 | XP_023931222.1methyltransferase-like protein 27 isoform X2 | F:GO:0008168; C:GO:0016021; P:GO:0032259 | |
| TRINITY_DN16605_c0_g1_i3_len_791_4920.p1_type_complete_len_120 | XP_018007416.1 probable protein BRICK1-B | P:GO:0007015; C:GO:0031209; F:GO:0044877 | |
| TRINITY_DN8044_c0_g1_i1_len_360_210087.p1_type_internal_len_121 | ---NA--- | | |
| TRINITY_DN32728_c0_g1_i1_len_360_210076.p1_type_internal_len_121 | XP_018009669.1 SET and MYND domain-containing protein DDB_G0284059-like | | |
| TRINITY_DN1917_c0_g1_i1_len_360_210108.p1_type_internal_len_121 | XP_026678380.1carbohydrate sulfotransferase 11 | C:GO:0005794; P:GO:0005975; C:GO:0016020; F:GO:0016740 | |
| TRINITY_DN38160_c0_g1_i1_len_360_210101.p1_type_internal_len_121 | XP_018019355.1 myosin-IA-like | F:GO:0003774; F:GO:0003779; F:GO:0005524; C:GO:0016459 | EC:3.6.1.15 |
| TRINITY_DN41582_c0_g1_i1_len_360_210096.p1_type_internal_len_121 | ---NA--- | | |
| TRINITY_DN13503_c0_g1_i1_len_630_240279.p1_type_complete_len_120 | ---NA--- | | |

| | | | |
|---|---|---|---|
| TRINITY_DN3672_c0_g1_i1_len_361_210060.p2_type_internal_len_121 | ---NA--- | | |
| TRINITY_DN34959_c0_g1_i1_len_361_210055.p2_type_internal_len_121 | XP_018009237.1 phospholipid-transporting ATPase ID-like | | |
| TRINITY_DN38854_c0_g1_i1_len_361_210052.p3_type_internal_len_121 | ---NA--- | | |
| TRINITY_DN16021_c0_g1_i1_len_621_240444.p1_type_5prime_partial_len_120 | ---NA--- | | |
| TRINITY_DN2073_c0_g1_i1_len_474_276675.p1_type_5prime_partial_len_120 | ---NA--- | | |
| TRINITY_DN31774_c0_g1_i1_len_361_210067.p3_type_internal_len_121 | XP_018012882.1 epithelial chloride channel protein-like | | |
| TRINITY_DN12210_c0_g1_i1_len_361_308645.p1_type_internal_len_121 | ---NA--- | | |
| TRINITY_DN5473_c0_g1_i2_len_417_207292.p2_type_5prime_partial_len_120 | XP_023212837.1sugar transporter SWEET1-like | P:GO:0008643; C:GO:0016020; F:GO:0051119 | |
| TRINITY_DN9371_c0_g1_i1_len_362_308605.p1_type_internal_len_121 | ---NA--- | | |
| TRINITY_DN16991_c0_g1_i1_len_362_308621.p1_type_internal_len_121 | ---NA--- | | |
| TRINITY_DN24976_c0_g1_i1_len_362_308627.p1_type_internal_len_121 | AQW41381.1selenium-dependent glutathione peroxidase | F:GO:0004602; P:GO:0006979; P:GO:0055114; P:GO:0098869 | EC:1.11.1.9; EC:1.11.1.7 |
| TRINITY_DN12756_c0_g1_i1_len_907_161432.p2_type_complete_len_120 | ---NA--- | | |
| TRINITY_DN23336_c0_g1_i1_len_1764_231889.p3_type_5prime_partial_len_120 | XP_018024664.1 trimeric intracellular cation channel type B-like | | |
| TRINITY_DN22256_c0_g1_i4_len_360_210114.p2_type_internal_len_121 | ---NA--- | | |
| TRINITY_DN11072_c0_g1_i1_len_1053_235694.p2_type_5prime_partial_len_120 | ABX54737.1elongation factor 1 beta' | F:GO:0003746; C:GO:0005853; P:GO:0006414 | |
| TRINITY_DN7184_c0_g1_i2_len_426_206855.p1_type_3prime_partial_len_121 | XP_018023119.1 zinc finger and BTB domain-containing protein 17-like isoform X1 | | |

| | | | |
|---|---|---|---|
| TRINITY_DN9910_c0_g1_i1_len_1080_235516.p1_type_complete_len_120 | XP_018016491.1 ras-related protein Rab-30-like isoform X3 | | |
| TRINITY_DN20095_c0_g1_i2_len_2638_91379.p3_type_5prime_partial_len_120 | ---NA--- | | |
| TRINITY_DN10435_c0_g1_i2_len_391_170603.p1_type_5prime_partial_len_120 | ---NA--- | | |
| TRINITY_DN23800_c0_g1_i1_len_1828_59720.p1_type_5prime_partial_len_120 | ---NA--- | | |
| TRINITY_DN12067_c0_g1_i1_len_410_306655.p1_type_complete_len_120 | ---NA--- | | |
| TRINITY_DN20672_c0_g1_i1_len_1065_3240.p4_type_3prime_partial_len_121 | ---NA--- | | |
| TRINITY_DN9280_c0_g1_i1_len_362_171878.p3_type_internal_len_121 | ---NA--- | | |
| TRINITY_DN33355_c0_g1_i1_len_362_171873.p1_type_internal_len_121 | ---NA--- | | |
| TRINITY_DN6785_c0_g1_i1_len_362_171864.p1_type_internal_len_121 | ---NA--- | | |
| TRINITY_DN6282_c0_g1_i1_len_361_171927.p1_type_internal_len_121 | ---NA--- | | |
| TRINITY_DN24287_c0_g1_i1_len_361_171928.p1_type_internal_len_121 | ---NA--- | | |
| TRINITY_DN12113_c0_g1_i1_len_361_171925.p1_type_internal_len_121 | XP_018014155.1 U6 snRNA-associated Sm-like protein LSm1 | | |
| TRINITY_DN9827_c1_g1_i1_len_361_171922.p1_type_internal_len_121 | KMQ95366.1putative glutamine-dependent nad+ synthetase-like protein | F:GO:0003952; F:GO:0004359; F:GO:0005524; C:GO:0005737; P:GO:0009435 | EC:3.5.1.2; EC:6.3.5.1 |
| TRINITY_DN1841_c0_g1_i1_len_362_171852.p1_type_internal_len_121 | XP_018011235.1 mucin-22-like isoform X3 | | |
| TRINITY_DN6421_c0_g1_i1_len_362_171848.p2_type_internal_len_121 | ---NA--- | | |
| TRINITY_DN8412_c0_g1_i1_len_363_171834.p1_type_internal_len_121 | XP_021941676.1E3 ubiquitin-protein ligase HUWE1-like | F:GO:0016740 | |

| | | | |
|---|---|---|---|
| TRINITY_DN18884_c0_g1_i1_len_899_236967.p2_type_5prime_partial_len_120 | ABI93175.1keratinocyte associated protein 2 | C:GO:0016021 | |
| TRINITY_DN19740_c0_g1_i2_len_455_305258.p1_type_5prime_partial_len_120 | EFN62657.1E3 ubiquitin-protein ligase rififylin | F:GO:0016874; F:GO:0046872 | |
| TRINITY_DN14104_c0_g1_i1_len_556_202746.p1_type_3prime_partial_len_121 | XP_018018729.1PREDICTED: uncharacterized protein LOC108675246 | | |
| TRINITY_DN1199_c0_g1_i1_len_890_237040.p1_type_5prime_partial_len_120 | ---NA--- | | |
| TRINITY_DN18529_c0_g1_i1_len_882_237111.p2_type_5prime_partial_len_120 | ---NA--- | | |
| TRINITY_DN19587_c0_g1_i2_len_2418_91566.p2_type_complete_len_120 | ---NA--- | | |
| TRINITY_DN14714_c0_g1_i1_len_439_305720.p1_type_5prime_partial_len_120 | ---NA--- | | |
| TRINITY_DN9538_c0_g1_i1_len_362_43000.p1_type_internal_len_121 | ---NA--- | | |
| TRINITY_DN7476_c0_g1_i1_len_362_43019.p1_type_internal_len_121 | ---NA--- | | |
| TRINITY_DN20356_c0_g1_i1_len_661_200605.p1_type_3prime_partial_len_121 | ---NA--- | | |
| TRINITY_DN31069_c0_g1_i10_len_3572_187869.p4_type_5prime_partial_len_120 | ---NA--- | | |
| TRINITY_DN20551_c0_g1_i1_len_363_42971.p1_type_internal_len_121 | XP_023716831.1ecto-NOX disulfide-thiol exchanger 2 isoform X2 | F:GO:0003723; P:GO:0007624; C:GO:0009897; F:GO:0016491; P:GO:0055114 | |
| TRINITY_DN29446_c0_g1_i1_len_669_6070.p1_type_5prime_partial_len_120 | ---NA--- | | |
| TRINITY_DN3979_c0_g1_i1_len_363_42950.p2_type_internal_len_121 | ---NA--- | | |
| TRINITY_DN14932_c0_g1_i1_len_1687_157096.p2_type_5prime_partial_len_120 | ---NA--- | | |
| TRINITY_DN25975_c0_g1_i1_len_361_43061.p1_type_internal_len_121 | ---NA--- | | |

| | | | |
|---|---|---|---|
| TRINITY_DN2193_c0_g1_i1_len_455_244392.p1_type_5prime_partial_len_120 | ---NA--- | | |
| TRINITY_DN197_c0_g1_i1_len_360_43119.p1_type_internal_len_121 | KRX76135.1hypothetical protein T4E_12067, partial | | |
| TRINITY_DN11710_c0_g1_i1_len_361_106080.p1_type_internal_len_121 | ---NA--- | | |
| TRINITY_DN2761_c0_g1_i1_len_360_106132.p1_type_internal_len_121 | XP_022780414.1intraflagellar transport protein 122 homolog isoform X2 | C:GO:0031514; P:GO:0060271 | |
| TRINITY_DN28994_c0_g1_i1_len_362_106002.p1_type_internal_len_121 | PSN35281.1Oxysterol-binding protein-related protein 2 | P:GO:0006869 | |
| TRINITY_DN17276_c0_g1_i1_len_428_245334.p3_type_5prime_partial_len_120 | ---NA--- | | |
| TRINITY_DN6170_c0_g1_i1_len_485_204703.p1_type_5prime_partial_len_120 | ---NA--- | | |
| TRINITY_DN21061_c0_g1_i2_len_2280_155426.p4_type_5prime_partial_len_120 | ---NA--- | | |
| TRINITY_DN6373_c0_g1_i1_len_390_246818.p1_type_5prime_partial_len_120 | XP_018009917.1 tubulin alpha-8 chain-like | F:GO:0003924; F:GO:0005200; F:GO:0005525; C:GO:0005874; P:GO:0007010; P:GO:0007017 | EC:3.6.1.15 |
| TRINITY_DN379_c0_g1_i1_len_362_209984.p1_type_internal_len_121 | ---NA--- | | |
| TRINITY_DN14312_c0_g1_i1_len_362_209980.p1_type_internal_len_121 | ---NA--- | | |
| TRINITY_DN33697_c0_g1_i1_len_362_209971.p1_type_internal_len_121 | XP_018017219.1 melanotransferrin-like | | |
| TRINITY_DN20854_c1_g1_i1_len_1290_158821.p3_type_complete_len_120 | ALA09086.1pacifastin light chain-like serine proteinase inhibitor | F:GO:0004867; P:GO:0010951 | |
| TRINITY_DN34262_c0_g1_i1_len_362_209952.p3_type_internal_len_121 | ---NA--- | | |
| TRINITY_DN10978_c0_g1_i1_len_362_209994.p1_type_internal_len_121 | XP_020710579.1putative fatty acyl-CoA reductase CG5065 | | |
| TRINITY_DN41271_c0_g1_i1_len_362_209991.p1_type_internal_len_121 | XP_018013901.1 leucine-rich repeats and immunoglobulin-like domains protein 2 | | |

| | | | |
|---|---|---|---|
| TRINITY_DN7783_c0_g1_i1_len_362_209990.p1_type_internal_len_121 | ---NA--- | | |
| TRINITY_DN20390_c0_g1_i1_len_362_209987.p1_type_internal_len_121 | ---NA--- | | |
| TRINITY_DN371_c0_g1_i1_len_363_209912.p1_type_internal_len_121 | XP_012530240.1 trafficking protein particle complex subunit 8 | | |
| TRINITY_DN37526_c0_g1_i1_len_363_209908.p1_type_internal_len_121 | ---NA--- | | |
| TRINITY_DN29865_c0_g1_i2_len_5553_187003.p4_type_complete_len_120 | ---NA--- | | |
| TRINITY_DN26262_c0_g1_i1_len_1456_193329.p4_type_3prime_partial_len_121 | ---NA--- | | |
| TRINITY_DN31781_c0_g1_i1_len_363_209901.p1_type_internal_len_121 | APU88436.1hypoxia-inducible factor 1 beta | F:GO:0003677; F:GO:0003700; C:GO:0005634; C:GO:0005667; C:GO:0005737; P:GO:0006355; F:GO:0046983 | |
| TRINITY_DN6143_c0_g1_i1_len_362_209949.p1_type_internal_len_121 | XP_019634649.1 von Willebrand factor A domain-containing protein 8-like | F:GO:0005524; F:GO:0016887 | EC:3.6.1.3; EC:3.6.1.15 |
| TRINITY_DN4537_c0_g1_i1_len_363_209945.p2_type_internal_len_121 | ---NA--- | | |
| TRINITY_DN31773_c0_g1_i1_len_545_241887.p1_type_5prime_partial_len_120 | ---NA--- | | |
| TRINITY_DN38726_c0_g1_i1_len_361_210045.p3_type_internal_len_121 | ---NA--- | | |
| TRINITY_DN1090_c0_g1_i1_len_361_210038.p2_type_internal_len_121 | ---NA--- | | |
| TRINITY_DN13693_c0_g1_i1_len_361_210049.p1_type_internal_len_121 | ---NA--- | | |
| TRINITY_DN1863_c0_g1_i1_len_412_207508.p1_type_5prime_partial_len_120 | ---NA--- | | |
| TRINITY_DN22246_c0_g1_i2_len_571_202387.p1_type_complete_len_120 | ---NA--- | | |

| | | | |
|---|---|---|---|
| TRINITY_DN2560_c0_g1_i1_len_583_241117.p1_type_5prime_partial_len_120 | XP_018027139.1 signal recognition particle 9 kDa protein-like | | |
| TRINITY_DN23889_c0_g1_i1_len_3203_58040.p2_type_complete_len_120 | ADK94870.1saposin isoform 1 | C:GO:0005764; P:GO:0006665 | |
| TRINITY_DN36938_c0_g1_i1_len_572_241331.p1_type_3prime_partial_len_121 | XP_018012435.1 neuroglian-like isoform X1 | C:GO:0016021 | |
| TRINITY_DN17214_c0_g1_i1_len_361_210010.p1_type_internal_len_121 | XP_026292725.1protein tilB-like | | |
| 2425\|_\|c17857_f3p1_2592_isoform_c17857_full_length_coverage_3_non_full_length_coverage_1_isoform_length_2592_22517 | ---NA--- | | |
| 2426\|_\|c2175_f5p0_2592_isoform_c2175_full_length_coverage_5_non_full_length_coverage_0_isoform_length_2592_20758 | XP_018023643.1 BRCA1-associated protein-like | F:GO:0003676; F:GO:0008270 | |
| 2427\|_\|c4794_f2p3_2592_isoform_c4794_full_length_coverage_2_non_full_length_coverage_3_isoform_length_2592_12415 | ---NA--- | | |
| 2428\|_\|c29071_f2p10_2592_isoform_c29071_full_length_coverage_2_non_full_length_coverage_10_isoform_length_2592_14043 | XP_018008609.1 DNA replication licensing factor Mcm5-like | F:GO:0003688; F:GO:0004386; F:GO:0005524; C:GO:0005634; P:GO:0006270; C:GO:0042555 | EC:3.6.1.15 |
| 2431\|_\|c4872_f2p4_2590_isoform_c4872_full_length_coverage_2_non_full_length_coverage_4_isoform_length_2590_21473 | CFW94149.1Csa-calmodulin 1 | F:GO:0005509 | |
| 2432\|_\|c17908_f3p0_2590_isoform_c17908_full_length_coverage_3_non_full_length_coverage_0_isoform_length_2590_17847 | AKE50479.1AMP-activated protein kinase subunit alpha | F:GO:0004674; F:GO:0005524; P:GO:0006468 | EC:2.7.11 |
| 2433\|_\|c18011_f2p1_2590_isoform_c18011_full_length_coverage_2_non_full_length_coverage_1_isoform_length_2590_17894 | ---NA--- | | |
| 2434\|_\|c21526_f2p0_2590_isoform_c21526_full_length_coverage_2_non_full_length_coverage_0_isoform_length_2590_18226 | XP_018024019.1 DNA-directed RNA polymerase I subunit RPA1-like | F:GO:0001054; F:GO:0003682; F:GO:0005515; P:GO:0006360; C:GO:0031981; P:GO:1904750 | EC:2.7.7.6 |

<table>
<tr><td>2435|_|c2537_f6p0_2590_isoform_c2537_full_length_coverage_6_non_full_length_coverage_0_isoform_length_2590_16492</td><td>XP_018026035.1 alpha-soluble NSF attachment protein-like</td><td>C:GO:0005623; P:GO:0006886</td><td></td></tr>
<tr><td>2436|_|c2475_f3p5_2589_isoform_c2475_full_length_coverage_3_non_full_length_coverage_5_isoform_length_2589_20969</td><td>XP_018025728.1 tropomyosin-1, isoforms 9A/A/B isoform X30</td><td></td><td></td></tr>
<tr><td>TRINITY_DN39074_c0_g1_i1_len_362_209997.p1_type_internal_len_121</td><td>XP_018006413.1 monoacylglycerol lipase ABHD12-like</td><td>C:GO:0016020</td><td></td></tr>
<tr><td>TRINITY_DN39396_c0_g1_i1_len_361_210036.p1_type_internal_len_121</td><td>---NA---</td><td></td><td></td></tr>
<tr><td>TRINITY_DN31778_c0_g1_i1_len_361_210032.p1_type_internal_len_121</td><td>XP_022257746.1ubiquitin carboxyl-terminal hydrolase 16-like isoform X2</td><td></td><td></td></tr>
<tr><td>TRINITY_DN8339_c0_g1_i1_len_361_210030.p1_type_internal_len_121</td><td>---NA---</td><td></td><td></td></tr>
<tr><td>TRINITY_DN31469_c0_g1_i1_len_361_210024.p1_type_internal_len_121</td><td>XP_022248669.1acyl-CoA synthetase family member 3, mitochondrial-like</td><td>F:GO:0003824</td><td></td></tr>
<tr><td>TRINITY_DN17188_c0_g2_i1_len_495_204415.p1_type_5prime_partial_len_120</td><td>---NA---</td><td></td><td></td></tr>
<tr><td>TRINITY_DN9863_c0_g1_i1_len_601_201752.p1_type_5prime_partial_len_120</td><td>---NA---</td><td></td><td></td></tr>
<tr><td>TRINITY_DN3956_c0_g1_i1_len_491_204531.p1_type_5prime_partial_len_120</td><td>---NA---</td><td></td><td></td></tr>
<tr><td>TRINITY_DN37038_c0_g1_i1_len_453_205862.p2_type_complete_len_120</td><td>---NA---</td><td></td><td></td></tr>
<tr><td>TRINITY_DN19669_c0_g1_i1_len_2397_230008.p4_type_5prime_partial_len_120</td><td>XP_026280088.1myotubularin-related protein 6 isoform X2</td><td></td><td></td></tr>
<tr><td>TRINITY_DN4856_c0_g1_i1_len_499_243074.p1_type_5prime_partial_len_120</td><td>XP_018022104.1 protein FAM57A-like</td><td></td><td></td></tr>
<tr><td>TRINITY_DN20357_c0_g1_i1_len_453_205833.p1_type_5prime_partial_len_120</td><td>---NA---</td><td></td><td></td></tr>
<tr><td>TRINITY_DN11442_c0_g1_i1_len_492_243240.p2_type_complete_len_120</td><td>---NA---</td><td></td><td></td></tr>
</table>

| | | | |
|---|---|---|---|
| TRINITY_DN2928_c0_g1_i1_len_364_209873.p1_type_internal_len_121 | ---NA--- | | |
| TRINITY_DN8376_c0_g1_i1_len_509_203949.p1_type_5prime_partial_len_120 | ---NA--- | | |
| TRINITY_DN3780_c0_g1_i1_len_364_209871.p2_type_internal_len_121 | ---NA--- | | |
| TRINITY_DN21308_c0_g1_i1_len_1353_158467.p2_type_complete_len_120 | ---NA--- | | |
| TRINITY_DN18100_c0_g1_i1_len_3048_29463.p5_type_complete_len_120 | ANF06986.1myocyte enhancer factor 2 a variant | F:GO:0000977; C:GO:0005634; P:GO:0045944; F:GO:0046983 | |
| TRINITY_DN9761_c0_g1_i1_len_364_209865.p1_type_internal_len_121 | ---NA--- | | |
| TRINITY_DN11552_c0_g1_i1_len_403_207885.p1_type_5prime_partial_len_120 | ---NA--- | | |
| TRINITY_DN29302_c0_g1_i1_len_362_137080.p1_type_internal_len_121 | ---NA--- | | |
| TRINITY_DN22032_c0_g2_i2_len_944_95965.p2_type_5prime_partial_len_120 | ---NA--- | | |
| TRINITY_DN16846_c0_g2_i1_len_363_137027.p2_type_internal_len_121 | XP_025086094.1multidrug resistance-associated protein 1-like isoform X1 | F:GO:0005524; C:GO:0016021; F:GO:0042626; P:GO:0055085 | EC:3.6.1.3; EC:3.6.1.15 |
| TRINITY_DN17818_c0_g1_i2_len_363_137041.p1_type_internal_len_121 | ---NA--- | | |
| TRINITY_DN31008_c0_g1_i1_len_361_137136.p1_type_internal_len_121 | ---NA--- | | |
| TRINITY_DN22919_c0_g1_i1_len_3404_153977.p3_type_5prime_partial_len_120 | ---NA--- | | |
| TRINITY_DN1265_c0_g1_i1_len_361_137147.p1_type_internal_len_121 | EEB16843.1class D atypical G-protein coupled receptor GPRfz3, putative | P:GO:0007275; C:GO:0016021; P:GO:0016055; F:GO:0042813 | |
| TRINITY_DN7772_c0_g1_i1_len_362_137108.p1_type_internal_len_121 | ---NA--- | | |
| TRINITY_DN8295_c0_g1_i1_len_363_74078.p1_type_internal_len_121 | ---NA--- | | |

| | | | |
|---|---|---|---|
| TRINITY_DN18004_c0_g1_i2_len_361_137112.p1_type_internal_len_121 | ---NA--- | | |
| TRINITY_DN31214_c0_g1_i1_len_361_137111.p2_type_internal_len_121 | ---NA--- | | |
| TRINITY_DN12201_c0_g1_i1_len_940_197089.p1_type_3prime_partial_len_121 | ---NA--- | | |
| TRINITY_DN22460_c0_g1_i1_len_2353_189812.p2_type_5prime_partial_len_120 | ---NA--- | | |
| TRINITY_DN17269_c0_g1_i4_len_546_37797.p1_type_complete_len_120 | ---NA--- | | |
| TRINITY_DN25403_c0_g1_i1_len_364_136989.p1_type_internal_len_121 | KFM78306.1Low-density lipoprotein receptor-related protein 6 | | |
| TRINITY_DN18531_c0_g1_i1_len_911_197381.p1_type_5prime_partial_len_120 | ---NA--- | | |
| TRINITY_DN10611_c0_g1_i1_len_1062_196009.p2_type_5prime_partial_len_120 | ---NA--- | | |
| TRINITY_DN31097_c3_g1_i2_len_1630_192406.p1_type_3prime_partial_len_121 | ---NA--- | | |
| TRINITY_DN26404_c0_g1_i1_len_363_74068.p1_type_internal_len_121 | ---NA--- | | |
| TRINITY_DN18428_c0_g1_i3_len_362_74118.p1_type_internal_len_121 | ---NA--- | | |
| TRINITY_DN14159_c0_g1_i1_len_572_37395.p1_type_5prime_partial_len_120 | ---NA--- | | |
| TRINITY_DN6774_c0_g1_i1_len_371_73643.p1_type_5prime_partial_len_120 | ---NA--- | | |
| TRINITY_DN2999_c0_g1_i1_len_364_74001.p2_type_internal_len_121 | ---NA--- | | |
| TRINITY_DN21894_c0_g1_i2_len_752_300582.p1_type_complete_len_120 | ---NA--- | | |
| TRINITY_DN2936_c0_g1_i1_len_633_98925.p1_type_complete_len_120 | ---NA--- | | |

| | | | |
|---|---|---|---|
| TRINITY_DN25825_c0_g1_i1_len_406_72089.p1_type_3prime_partial_len_121 | AAF19002.1clottable protein | F:GO:0005319; C:GO:0005576; P:GO:0006869; P:GO:0042381 | |
| TRINITY_DN25628_c0_g1_i1_len_360_137194.p1_type_internal_len_121 | ---NA--- | | |
| TRINITY_DN35230_c0_g1_i1_len_389_72838.p1_type_5prime_partial_len_119 | ---NA--- | | |
| TRINITY_DN14363_c0_g1_i1_len_777_271991.p1_type_complete_len_119 | XP_018013852.1 DNA-directed RNA polymerase II subunit RPB11-like | F:GO:0001055; F:GO:0003677; C:GO:0005665; P:GO:0006366; F:GO:0046983 | EC:2.7.7.6 |
| TRINITY_DN2194_c0_g1_i1_len_721_97829.p1_type_complete_len_119 | ---NA--- | | |
| TRINITY_DN813_c0_g1_i1_len_358_137292.p1_type_internal_len_120 | ---NA--- | | |
| TRINITY_DN16448_c0_g1_i2_len_552_202817.p1_type_5prime_partial_len_119 | ---NA--- | | |
| TRINITY_DN28188_c0_g1_i1_len_358_137281.p1_type_internal_len_120 | ---NA--- | | |
| TRINITY_DN32082_c0_g1_i1_len_358_137319.p1_type_internal_len_120 | ---NA--- | | |
| TRINITY_DN27954_c0_g1_i1_len_358_137305.p1_type_internal_len_120 | ---NA--- | | |
| TRINITY_DN11301_c0_g1_i1_len_436_245032.p1_type_5prime_partial_len_119 | AAC84145.1hyperglycemic hormone homolog PmSGP-IV precursor | F:GO:0005184; C:GO:0005576; P:GO:0006006; P:GO:0007218; P:GO:0010469 | |
| TRINITY_DN36217_c0_g1_i1_len_390_208494.p1_type_3prime_partial_len_120 | ---NA--- | | |
| TRINITY_DN29220_c0_g1_i1_len_390_208498.p1_type_5prime_partial_len_119 | ---NA--- | | |
| TRINITY_DN6203_c1_g1_i1_len_518_166693.p1_type_5prime_partial_len_119 | ---NA--- | | |
| TRINITY_DN23532_c0_g1_i2_len_2276_155432.p6_type_5prime_partial_len_119 | ---NA--- | | |

| | | | |
|---|---|---|---|
| TRINITY_DN29605_c0_g1_i1_len_445_244734.p1_type_complete_len_119 | ---NA--- | | |
| TRINITY_DN32848_c0_g1_i1_len_358_210243.p2_type_internal_len_120 | XP_018026040.1PREDICTED: uncharacterized protein LOC108681512 isoform X1 | | |
| TRINITY_DN35346_c0_g1_i1_len_465_70129.p1_type_5prime_partial_len_119 | ---NA--- | | |
| TRINITY_DN9475_c0_g1_i1_len_359_74256.p3_type_internal_len_120 | XP_023722160.1serum response factor homolog isoform X2 | F:GO:0000981; F:GO:0000987; C:GO:0005634; P:GO:0007275; F:GO:0008134; P:GO:0045944; F:GO:0046983 | |
| TRINITY_DN13271_c0_g1_i1_len_2016_156065.p2_type_complete_len_119 | ---NA--- | | |
| TRINITY_DN14235_c0_g1_i1_len_359_137234.p1_type_internal_len_120 | ---NA--- | | |
| TRINITY_DN17662_c0_g1_i1_len_900_236951.p1_type_complete_len_119 | ---NA--- | | |
| TRINITY_DN24259_c0_g1_i2_len_392_208398.p1_type_3prime_partial_len_120 | ---NA--- | | |
| TRINITY_DN40126_c0_g1_i1_len_424_206956.p1_type_3prime_partial_len_120 | XP_018016802.1 transcription factor SPT20 homolog | | |
| TRINITY_DN30664_c0_g1_i1_len_969_298979.p2_type_5prime_partial_len_119 | XP_018009239.1 peripheral plasma membrane protein CASK-like | | |
| TRINITY_DN37357_c0_g1_i1_len_424_245487.p2_type_complete_len_119 | ---NA--- | | |
| TRINITY_DN7695_c0_g1_i1_len_610_164826.p1_type_5prime_partial_len_119 | ---NA--- | | |
| TRINITY_DN16988_c0_g1_i1_len_553_131205.p1_type_complete_len_119 | XP_014771883.1PREDICTED: uncharacterized protein LOC106870361 | | |
| TRINITY_DN8437_c0_g1_i1_len_890_237045.p2_type_complete_len_119 | ---NA--- | | |
| TRINITY_DN10840_c0_g1_i1_len_451_305374.p1_type_5prime_partial_len_119 | ---NA--- | | |

| | | | |
|---|---|---|---|
| TRINITY_DN3269_c0_g1_i2_len_577_165409.p2_type_3prime_partial_len_120 | XP_018008089.1 huntingtin-interacting protein 1-like | | |
| TRINITY_DN24808_c0_g1_i1_len_359_137238.p2_type_3prime_partial_len_120 | ---NA--- | | |
| TRINITY_DN20153_c0_g1_i1_len_359_137235.p1_type_internal_len_120 | ---NA--- | | |
| TRINITY_DN31432_c0_g1_i1_len_431_245230.p1_type_5prime_partial_len_119 | XP_016825420.1 exportin-6 isoform X4 | C:GO:0005623; P:GO:0006611; F:GO:0008565 | |
| TRINITY_DN31608_c0_g1_i1_len_360_74244.p1_type_internal_len_120 | ADG22162.1chitinase 1 precursor | F:GO:0004553; C:GO:0005576; P:GO:0005975; P:GO:0006030; F:GO:0008061 | |
| TRINITY_DN35196_c0_g1_i1_len_359_248304.p3_type_internal_len_120 | ---NA--- | | |
| TRINITY_DN4385_c0_g1_i1_len_359_248302.p2_type_internal_len_120 | ---NA--- | | |
| TRINITY_DN12751_c0_g1_i1_len_359_248285.p1_type_internal_len_120 | XP_018022039.1 midasin-like | F:GO:0005524; F:GO:0016887 | |
| TRINITY_DN34207_c0_g1_i1_len_360_248276.p1_type_internal_len_120 | ---NA--- | | |
| TRINITY_DN37562_c0_g1_i1_len_360_248274.p1_type_internal_len_120 | XP_018023086.1 E3 ubiquitin-protein ligase TRIM23-like | F:GO:0003676; F:GO:0005525; F:GO:0008270 | |
| TRINITY_DN37306_c0_g1_i1_len_383_208849.p1_type_5prime_partial_len_119 | ---NA--- | | |
| TRINITY_DN10074_c0_g1_i1_len_358_248371.p1_type_internal_len_120 | ODM87514.1histone H3 | C:GO:0000786; F:GO:0003677; C:GO:0005634; F:GO:0046982 | |
| TRINITY_DN12896_c0_g1_i1_len_358_248359.p2_type_internal_len_120 | ---NA--- | | |
| TRINITY_DN30510_c0_g1_i1_len_358_248358.p2_type_internal_len_120 | XP_023715375.1NACHT and WD repeat domain-containing protein 2 isoform X3 | | |
| TRINITY_DN29397_c0_g1_i1_len_358_248348.p1_type_internal_len_120 | XP_018026643.1 extracellular matrix protein FRAS1-like | | |

| | | | |
|---|---|---|---|
| TRINITY_DN33626_c0_g1_i1_len_359_248319.p2_type_internal_len_120 | AEB00643.1glucose transporter 1 | P:GO:0008643; C:GO:0016020; C:GO:0016021; F:GO:0022857; P:GO:0055085 | |
| TRINITY_DN7780_c0_g1_i1_len_390_104802.p1_type_5prime_partial_len_119 | XP_018015547.1PREDICTED: uncharacterized protein LOC108672409 isoform X3 | | |
| TRINITY_DN20304_c0_g1_i2_len_1152_235024.p1_type_complete_len_119 | ---NA--- | | |
| TRINITY_DN1054_c0_g1_i1_len_470_69977.p1_type_5prime_partial_len_119 | ---NA--- | | |
| TRINITY_DN4183_c0_g1_i2_len_360_248272.p1_type_internal_len_120 | XP_018013175.1 probable serine/threonine-protein kinase nek3 | F:GO:0003676 | |
| TRINITY_DN39_c0_g1_i1_len_360_248251.p1_type_internal_len_120 | ---NA--- | | |
| TRINITY_DN5242_c0_g1_i1_len_360_248248.p1_type_5prime_partial_len_119 | XP_022290249.1uncharacterized protein LOC111101890 | | |
| TRINITY_DN11394_c1_g1_i1_len_360_248243.p1_type_internal_len_120 | ---NA--- | | |
| TRINITY_DN36580_c0_g1_i1_len_361_248225.p1_type_internal_len_120 | ---NA--- | | |
| TRINITY_DN28889_c0_g1_i1_len_3174_188288.p3_type_5prime_partial_len_119 | ---NA--- | | |
| TRINITY_DN25583_c0_g1_i2_len_3252_228711.p1_type_5prime_partial_len_863 | XP_022309567.1zinc finger protein 665-like | | |
| 2438\|_\|c19202_f2p1_2589_isoform_c19202_full_length_coverage_2_non_full_length_coverage_1_isoform_length_2589_22767 | XP_018022039.1 midasin-like | | |
| 2439\|_\|c2604_f4p1_2589_isoform_c2604_full_length_coverage_4_non_full_length_coverage_1_isoform_length_2589_21080 | XP_018571317.1calcium uptake protein 3, mitochondrial isoform X2 | | |
| TRINITY_DN24815_c0_g1_i1_len_2589_189248.p1_type_internal_len_864 | KZS18217.1putative Kelch domain-containing protein 2 | C:GO:0048188; C:GO:0071339 | |

| | | | |
|---|---|---|---|
| TRINITY_DN21074_c0_g1_i1_len_3490_90975.p1_type_5prime_partial_len_863 | AMJ52356.1cullin 3 | P:GO:0006511; C:GO:0031461; F:GO:0031625 | |
| 2442\|_\|c2924_f2p9_2588_isoform_c2924_full_length_coverage_2_non_full_length_coverage_9_isoform_length_2588_16721 | XP_017948482.1 ubiquitin carboxyl-terminal hydrolase 10 isoform X2 | P:GO:0006508; F:GO:0036459; P:GO:0044267 | EC:3.4.19.12 |
| 2443\|_\|c19617_f2p2_2588_isoform_c19617_full_length_coverage_2_non_full_length_coverage_2_isoform_length_2588_22787 | EZA53219.1Lysine-specific demethylase 3B | F:GO:0008168; P:GO:0032259 | |
| 2444\|_\|c2436_f3p12_2588_isoform_c2436_full_length_coverage_3_non_full_length_coverage_12_isoform_length_2588_20933 | XP_026279169.1ras-like GTP-binding protein Rho1 isoform X1 | F:GO:0003924; F:GO:0005525; P:GO:0007264 | EC:3.6.1.15 |
| 2445\|_\|c6643_f2p4_2587_isoform_c6643_full_length_coverage_2_non_full_length_coverage_4_isoform_length_2587_21619 | XP_018014233.1 TAF6-like RNA polymerase II p300/CBP-associated factor-associated factor 65 kDa subunit 6L | | |
| 2447\|_\|c18287_f2p3_2586_isoform_c18287_full_length_coverage_2_non_full_length_coverage_3_isoform_length_2586_13697 | ---NA--- | | |
| TRINITY_DN8044_c0_g1_i1_len_361_248215.p1_type_internal_len_120 | XP_014298924.1 solute carrier family 12 member 8 isoform X2 | C:GO:0016021; F:GO:0022857; P:GO:0055085 | |
| TRINITY_DN9630_c0_g1_i1_len_359_280685.p1_type_internal_len_120 | ---NA--- | | |
| TRINITY_DN23049_c0_g1_i1_len_359_280684.p1_type_internal_len_120 | ---NA--- | | |
| TRINITY_DN31498_c0_g1_i1_len_359_280668.p1_type_internal_len_120 | ---NA--- | | |
| TRINITY_DN11955_c0_g1_i1_len_361_74171.p1_type_internal_len_120 | XP_016521803.1 gastrula zinc finger protein XlCGF8.2DB-like isoform X1 | F:GO:0003676 | |
| TRINITY_DN5018_c0_g1_i1_len_358_280720.p2_type_internal_len_120 | XP_011416110.1 xaa-Pro aminopeptidase 1 isoform X1 | F:GO:0016787; F:GO:0046872 | |
| TRINITY_DN41543_c0_g1_i1_len_357_210314.p1_type_internal_len_120 | KDR10374.1putative ATP-dependent RNA helicase YTHDC2 | F:GO:0003723; F:GO:0004386; F:GO:0005524 | EC:3.6.1.15 |
| TRINITY_DN22468_c0_g1_i1_len_410_103967.p1_type_5prime_partial_len_119 | ---NA--- | | |

| | | | |
|---|---|---|---|
| TRINITY_DN30191_c0_g1_i2_len_3050_188451.p4_type_complete_len_119 | ---NA--- | | |
| TRINITY_DN30407_c0_g1_i1_len_358_280702.p1_type_internal_len_120 | XP_018027080.1 cubilin-like isoform X2 | | |
| TRINITY_DN18724_c0_g1_i1_len_498_101322.p1_type_5prime_partial_len_119 | ---NA--- | | |
| TRINITY_DN30991_c0_g1_i1_len_357_248419.p1_type_internal_len_120 | ---NA--- | | |
| TRINITY_DN29265_c0_g1_i1_len_357_248406.p1_type_internal_len_120 | ACC62400.2ribonuclease III | F:GO:0003723; F:GO:0004525; P:GO:0006396; P:GO:0016075; P:GO:0031047; P:GO:0090502 | EC:3.1.30; EC:3.1.26; EC:3.1.26.3 |
| TRINITY_DN14801_c0_g1_i1_len_361_280593.p1_type_internal_len_120 | ---NA--- | | |
| TRINITY_DN518_c0_g1_i1_len_408_169913.p1_type_5prime_partial_len_119 | ---NA--- | | |
| TRINITY_DN4359_c0_g1_i1_len_358_248395.p1_type_internal_len_120 | ---NA--- | | |
| TRINITY_DN5383_c0_g1_i1_len_496_69203.p1_type_3prime_partial_len_120 | ---NA--- | | |
| TRINITY_DN30181_c0_g1_i1_len_358_248388.p1_type_internal_len_120 | XP_014673088.1 speedy protein A-like | | |
| TRINITY_DN35124_c0_g1_i1_len_358_248381.p2_type_internal_len_120 | KZS05987.1 WSCD family member | F:GO:0008146; C:GO:0016020 | |
| TRINITY_DN33413_c0_g1_i1_len_358_248376.p1_type_internal_len_120 | XP_018011710.1 venom phosphodiesterase 2-like | | |
| TRINITY_DN16961_c0_g1_i1_len_359_280656.p1_type_internal_len_120 | ---NA--- | | |
| TRINITY_DN23055_c0_g1_i1_len_359_280649.p2_type_internal_len_120 | ---NA--- | | |
| TRINITY_DN11827_c0_g1_i1_len_494_69259.p1_type_complete_len_119 | ---NA--- | | |
| TRINITY_DN10869_c0_g1_i2_len_360_280641.p1_type_internal_len_120 | XP_023227811.1ubiquitin domain-containing protein 2-like | | |

| | | | |
|---|---|---|---|
| TRINITY_DN9108_c0_g1_i1_len_361_106069.p1_type_internal_len_120 | ---NA--- | | |
| TRINITY_DN8029_c0_g1_i1_len_361_106089.p1_type_internal_len_120 | XP_021962532.1mucin-5AC-like isoform X2 | C:GO:0016020 | |
| TRINITY_DN12148_c0_g1_i4_len_360_106115.p1_type_internal_len_120 | XP_022206325.1proteoglycan 4-like | | |
| TRINITY_DN12588_c0_g1_i1_len_360_106122.p1_type_internal_len_120 | ---NA--- | | |
| TRINITY_DN28711_c0_g2_i1_len_2347_189829.p3_type_5prime_partial_len_119 | ---NA--- | | |
| TRINITY_DN23947_c0_g1_i1_len_431_305947.p1_type_5prime_partial_len_119 | ---NA--- | | |
| TRINITY_DN28243_c0_g1_i1_len_644_36379.p1_type_complete_len_119 | ---NA--- | | |
| TRINITY_DN23847_c0_g1_i2_len_1251_194578.p2_type_complete_len_119 | ---NA--- | | |
| TRINITY_DN7649_c0_g1_i1_len_358_106213.p1_type_internal_len_120 | ---NA--- | | |
| TRINITY_DN14820_c0_g1_i2_len_807_300061.p2_type_5prime_partial_len_119 | ---NA--- | | |
| TRINITY_DN16048_c0_g1_i1_len_391_246760.p1_type_5prime_partial_len_119 | ---NA--- | | |
| TRINITY_DN13021_c0_g1_i2_len_387_246932.p1_type_5prime_partial_len_119 | ---NA--- | | |
| TRINITY_DN30660_c0_g1_i1_len_359_106177.p1_type_internal_len_120 | ---NA--- | | |
| TRINITY_DN20367_c0_g1_i1_len_1416_1921.p3_type_5prime_partial_len_119 | ---NA--- | | |
| TRINITY_DN3175_c0_g1_i1_len_1299_94038.p2_type_3prime_partial_len_120 | ---NA--- | | |
| TRINITY_DN15465_c0_g1_i1_len_369_209583.p1_type_5prime_partial_len_119 | ---NA--- | | |

| | | | |
|---|---|---|---|
| TRINITY_DN26380_c0_g1_i1_len_369_12994.p1_type_5prime_partial_len_119 | ---NA--- | | |
| TRINITY_DN29632_c0_g2_i1_len_1107_195648.p1_type_5prime_partial_len_119 | ---NA--- | | |
| TRINITY_DN32868_c0_g1_i1_len_377_247407.p1_type_5prime_partial_len_119 | ---NA--- | | |
| TRINITY_DN11530_c0_g1_i1_len_378_247365.p1_type_5prime_partial_len_119 | ---NA--- | | |
| TRINITY_DN940_c0_g1_i2_len_625_201306.p1_type_5prime_partial_len_119 | ---NA--- | | |
| TRINITY_DN12866_c0_g1_i1_len_469_167911.p1_type_5prime_partial_len_119 | ---NA--- | | |
| TRINITY_DN35259_c0_g1_i1_len_376_247476.p1_type_5prime_partial_len_119 | ---NA--- | | |
| TRINITY_DN13383_c0_g1_i1_len_773_35012.p1_type_5prime_partial_len_119 | ---NA--- | | |
| TRINITY_DN12038_c0_g1_i1_len_482_204803.p1_type_5prime_partial_len_119 | ---NA--- | | |
| TRINITY_DN5849_c0_g1_i1_len_374_247558.p1_type_5prime_partial_len_119 | ---NA--- | | |
| TRINITY_DN12353_c0_g1_i1_len_504_132276.p1_type_5prime_partial_len_119 | ---NA--- | | |
| TRINITY_DN39003_c0_g1_i1_len_359_210147.p1_type_internal_len_120 | XP_018026562.1PREDICTED: uncharacterized protein LOC108681986, partial | F:GO:0004857; F:GO:0004867; C:GO:0005576; P:GO:0010466; P:GO:0010951; F:GO:0030414; P:GO:0043086 | |
| TRINITY_DN5206_c0_g1_i1_len_417_207274.p1_type_5prime_partial_len_119 | ---NA--- | | |
| TRINITY_DN37936_c0_g1_i1_len_359_210145.p2_type_internal_len_120 | XP_021927155.1rho GTPase-activating protein 45-like isoform X1 | | |
| TRINITY_DN2809_c0_g1_i1_len_359_210138.p1_type_internal_len_120 | ---NA--- | | |

| | | | |
|---|---|---|---|
| TRINITY_DN31522_c0_g1_i1_len_359_210153.p1_type_internal_len_120 | ---NA--- | | |
| TRINITY_DN30818_c0_g1_i1_len_1508_296759.p3_type_5prime_partial_len_119 | ---NA--- | | |
| TRINITY_DN13615_c0_g1_i1_len_359_210148.p1_type_internal_len_120 | ---NA--- | | |
| TRINITY_DN31948_c0_g1_i1_len_409_207630.p1_type_3prime_partial_len_120 | XP_023713941.1tyrosine-protein kinase Dnt-like | | |
| TRINITY_DN37023_c0_g1_i1_len_554_241690.p1_type_5prime_partial_len_119 | ---NA--- | | |
| TRINITY_DN36622_c0_g1_i1_len_359_210135.p1_type_internal_len_120 | ---NA--- | | |
| TRINITY_DN21112_c0_g1_i2_len_1460_296910.p2_type_complete_len_119 | ---NA--- | | |
| TRINITY_DN22795_c0_g1_i1_len_604_99353.p1_type_complete_len_119 | ---NA--- | | |
| TRINITY_DN14909_c0_g1_i1_len_2073_230885.p2_type_5prime_partial_len_119 | ---NA--- | | |
| TRINITY_DN26304_c0_g1_i1_len_4816_227835.p3_type_5prime_partial_len_119 | ---NA--- | | |
| TRINITY_DN23388_c0_g1_i1_len_898_63778.p1_type_complete_len_119 | ---NA--- | | |
| TRINITY_DN12477_c0_g1_i2_len_359_210160.p1_type_internal_len_120 | ---NA--- | | |
| TRINITY_DN12773_c0_g1_i1_len_609_99297.p3_type_5prime_partial_len_119 | ---NA--- | | |
| TRINITY_DN7213_c0_g1_i1_len_359_210158.p1_type_internal_len_120 | ---NA--- | | |
| TRINITY_DN18668_c0_g1_i3_len_1672_1296.p1_type_5prime_partial_len_119 | ---NA--- | | |
| TRINITY_DN16248_c1_g1_i1_len_359_210163.p1_type_internal_len_120 | ADZ96217.1JHE-like carboxylesterase 1 | F:GO:0016787 | |

| | | | |
|---|---|---|---|
| TRINITY_DN16766_c0_g1_i3_len_560_274821.p1_type_3prime_partial_len_120 | ---NA--- | | |
| TRINITY_DN15925_c0_g1_i2_len_987_33565.p3_type_5prime_partial_len_119 | ACJ36226.1spermatogonial stem-cell renewal factor | F:GO:0004645; P:GO:0006206; P:GO:0006213; F:GO:0009032; F:GO:0016154 | EC:2.4.1.1; EC:2.4.2.2; EC:2.4.2.4 |
| TRINITY_DN13714_c0_g1_i1_len_359_210162.p1_type_internal_len_120 | ---NA--- | | |
| TRINITY_DN8619_c0_g1_i1_len_406_207777.p1_type_5prime_partial_len_119 | ---NA--- | | |
| TRINITY_DN38766_c0_g1_i1_len_450_205961.p1_type_5prime_partial_len_119 | ---NA--- | | |
| TRINITY_DN16377_c0_g1_i2_len_357_308839.p1_type_internal_len_120 | XP_026292635.1myc box-dependent-interacting protein 1-like | C:GO:0005737 | |
| TRINITY_DN41501_c0_g1_i1_len_360_210103.p1_type_internal_len_120 | ---NA--- | | |
| TRINITY_DN31777_c0_g1_i1_len_361_210063.p1_type_internal_len_120 | ---NA--- | | |
| TRINITY_DN41587_c0_g1_i1_len_361_210068.p1_type_internal_len_120 | ---NA--- | | |
| TRINITY_DN16635_c0_g1_i2_len_634_98907.p1_type_3prime_partial_len_120 | ---NA--- | | |
| TRINITY_DN21239_c0_g1_i1_len_437_40284.p1_type_5prime_partial_len_119 | ---NA--- | | |
| TRINITY_DN1684_c0_g1_i1_len_360_210094.p1_type_internal_len_120 | ---NA--- | | |
| TRINITY_DN19080_c0_g1_i1_len_446_206091.p1_type_5prime_partial_len_119 | ---NA--- | | |
| TRINITY_DN17718_c0_g2_i1_len_464_276942.p2_type_5prime_partial_len_119 | ---NA--- | | |
| TRINITY_DN40341_c0_g1_i1_len_361_210012.p1_type_internal_len_120 | ---NA--- | | |
| TRINITY_DN10629_c0_g1_i1_len_542_303339.p1_type_complete_len_119 | ---NA--- | | |

| | | | |
|---|---|---|---|
| TRINITY_DN3328_c0_g1_i1_len_359_308758.p3_type_internal_len_120 | ---NA--- | | |
| TRINITY_DN19446_c0_g1_i2_len_1551_267547.p1_type_complete_len_119 | ---NA--- | | |
| TRINITY_DN24394_c0_g1_i1_len_587_202040.p1_type_5prime_partial_len_119 | ---NA--- | | |
| TRINITY_DN18459_c0_g1_i2_len_361_210011.p1_type_internal_len_120 | ---NA--- | | |
| TRINITY_DN6346_c0_g1_i1_len_359_210123.p1_type_internal_len_120 | XP_018015211.1 leucine-rich repeat serine/threonine-protein kinase 1-like | | |
| TRINITY_DN13475_c0_g1_i1_len_4038_29258.p3_type_complete_len_119 | ---NA--- | | |
| TRINITY_DN6747_c0_g1_i1_len_358_308795.p1_type_internal_len_120 | XP_023236053.1xaa-Pro aminopeptidase 1-like isoform X2 | | |
| TRINITY_DN11628_c0_g1_i1_len_360_210111.p1_type_internal_len_120 | ---NA--- | | |
| TRINITY_DN15997_c0_g1_i1_len_457_39684.p1_type_5prime_partial_len_119 | ---NA--- | | |
| TRINITY_DN27902_c0_g1_i1_len_682_163787.p1_type_5prime_partial_len_119 | XP_021930063.1MFS-type transporter SLC18B1-like | C:GO:0016021; P:GO:0055085 | |
| TRINITY_DN12251_c0_g1_i1_len_683_163768.p1_type_5prime_partial_len_119 | ---NA--- | | |
| TRINITY_DN32583_c0_g1_i1_len_358_210221.p1_type_internal_len_120 | KZS21895.1putative Rho GTPase-activating protein 20 | P:GO:0007165 | |
| TRINITY_DN13932_c0_g1_i1_len_358_210237.p2_type_3prime_partial_len_120 | ---NA--- | | |
| TRINITY_DN24935_c0_g1_i1_len_2799_58320.p1_type_5prime_partial_len_862 | ABG82044.1ALG-2 interacting protein x | | |
| 2449\|_\|c26002_f2p1_2585_isoform_c26002_full_length_coverage_2_non_full_length_coverage_1_isoform_length_2585_18377 | ---NA--- | | |

| | | | |
|---|---|---|---|
| 2450\|_\|c1884_f5p3_2585_isoform_c1884_full_length_coverage_5_non_full_length_coverage_3_isoform_length_2585_20588 | ---NA--- | | |
| 2451\|_\|c1825_f2p6_2585_isoform_c1825_full_length_coverage_2_non_full_length_coverage_6_isoform_length_2585_25516 | XP_018006398.1 focadhesin-like | | |
| 2453\|_\|c2279_f7p1_2584_isoform_c2279_full_length_coverage_7_non_full_length_coverage_1_isoform_length_2584_20823 | PCG78848.1hypothetical protein B5V51_2879 | | |
| TRINITY_DN29811_c1_g1_i1_len_2868_188752.p1_type_complete_len_861 | XP_015834770.1 otopetrin-2 isoform X2 | C:GO:0016020; C:GO:0016021 | |
| 2455\|_\|c26264_f2p0_2583_isoform_c26264_full_length_coverage_2_non_full_length_coverage_0_isoform_length_2583_23002 | ---NA--- | | |
| 2456\|_\|c1683_f2p2_2583_isoform_c1683_full_length_coverage_2_non_full_length_coverage_2_isoform_length_2583_27898 | ---NA--- | | |
| 2457\|_\|c1779_f8p8_2583_isoform_c1779_full_length_coverage_8_non_full_length_coverage_8_isoform_length_2583_11490 | ---NA--- | | |
| TRINITY_DN24200_c0_g2_i2_len_3748_228317.p1_type_complete_len_861 | EEB20268.1conserved hypothetical protein | F:GO:0003723; P:GO:0006396 | |
| TRINITY_DN31139_c3_g1_i1_len_1888_191319.p3_type_complete_len_119 | ---NA--- | | |
| TRINITY_DN19023_c0_g1_i1_len_851_299752.p1_type_complete_len_119 | ---NA--- | | |
| TRINITY_DN3306_c0_g1_i2_len_357_43258.p1_type_internal_len_120 | ---NA--- | | |
| TRINITY_DN16550_c0_g1_i1_len_2501_29777.p5_type_complete_len_119 | ---NA--- | | |
| TRINITY_DN5662_c0_g1_i1_len_359_74287.p1_type_internal_len_120 | ---NA--- | | |

| | | | |
|---|---|---|---|
| TRINITY_DN31220_c1_g1_i1_len_1145_195339.p2_type_5prime_partial_len_119 | ---NA--- | | |
| TRINITY_DN11732_c0_g1_i1_len_627_201265.p2_type_5prime_partial_len_119 | ---NA--- | | |
| TRINITY_DN15396_c0_g1_i1_len_358_74302.p1_type_internal_len_120 | ---NA--- | | |
| TRINITY_DN33879_c0_g1_i1_len_489_243326.p1_type_3prime_partial_len_120 | ---NA--- | | |
| TRINITY_DN19005_c0_g1_i1_len_457_9904.p1_type_5prime_partial_len_119 | ---NA--- | | |
| TRINITY_DN33245_c0_g1_i1_len_795_198684.p1_type_complete_len_119 | ---NA--- | | |
| TRINITY_DN21735_c0_g1_i2_len_1613_157373.p2_type_complete_len_119 | AVD29880.1B cell lymphoma 2 | C:GO:0016021; P:GO:0042981 | |
| TRINITY_DN35392_c0_g1_i1_len_358_210238.p1_type_internal_len_120 | ---NA--- | | |
| TRINITY_DN12056_c0_g1_i1_len_451_244543.p1_type_complete_len_119 | ---NA--- | | |
| TRINITY_DN25367_c0_g1_i1_len_359_43161.p1_type_internal_len_120 | ---NA--- | | |
| TRINITY_DN5509_c0_g1_i1_len_359_43153.p1_type_internal_len_120 | ---NA--- | | |
| TRINITY_DN3405_c0_g1_i1_len_359_74268.p2_type_internal_len_120 | PRD25906.1zinc finger protein | F:GO:0003677 | |
| TRINITY_DN13675_c0_g1_i1_len_361_43064.p1_type_internal_len_120 | ---NA--- | | |
| TRINITY_DN25380_c0_g1_i1_len_357_43238.p1_type_3prime_partial_len_120 | XP_014665059.1 proprotein convertase subtilisin/kexin type 7-like | F:GO:0008236 | |
| TRINITY_DN16447_c0_g1_i1_len_358_43187.p1_type_internal_len_120 | AWU67222.1putative chitinase | F:GO:0004553; P:GO:0008152 | |
| TRINITY_DN1284_c0_g1_i1_len_358_43175.p1_type_internal_len_120 | ---NA--- | | |

| | | | |
|---|---|---|---|
| TRINITY_DN11877_c0_g1_i1_len_358_43191.p1_type_internal_len_120 | XP_020288627.1BTB/POZ domain-containing protein 17 | | |
| TRINITY_DN13359_c1_g1_i1_len_520_242518.p1_type_3prime_partial_len_120 | ---NA--- | | |
| TRINITY_DN21246_c0_g1_i1_len_458_70328.p1_type_5prime_partial_len_119 | ---NA--- | | |
| TRINITY_DN14347_c0_g1_i1_len_758_162840.p1_type_5prime_partial_len_119 | ---NA--- | | |
| TRINITY_DN34164_c0_g1_i1_len_358_210198.p1_type_internal_len_120 | ---NA--- | | |
| TRINITY_DN21510_c0_g1_i2_len_358_210195.p1_type_internal_len_120 | ---NA--- | | |
| TRINITY_DN11966_c0_g1_i1_len_769_162731.p2_type_complete_len_119 | ---NA--- | | |
| TRINITY_DN13210_c0_g1_i1_len_426_71348.p1_type_complete_len_119 | XP_014663259.1 multidrug resistance-associated protein 7-like | | |
| TRINITY_DN14432_c0_g1_i2_len_357_74395.p1_type_internal_len_120 | ---NA--- | | |
| TRINITY_DN25318_c0_g1_i3_len_4364_227998.p4_type_complete_len_119 | ---NA--- | | |
| TRINITY_DN13697_c0_g1_i1_len_413_278513.p1_type_5prime_partial_len_119 | ---NA--- | | |
| TRINITY_DN23854_c0_g1_i1_len_1516_232952.p2_type_5prime_partial_len_119 | ---NA--- | | |
| TRINITY_DN10635_c0_g1_i1_len_358_210184.p1_type_internal_len_120 | ---NA--- | | |
| TRINITY_DN29071_c0_g1_i3_len_1218_194817.p3_type_5prime_partial_len_119 | ---NA--- | | |
| TRINITY_DN25833_c0_g1_i2_len_4723_187225.p4_type_complete_len_119 | ---NA--- | | |
| TRINITY_DN17060_c0_g1_i1_len_413_71810.p1_type_5prime_partial_len_119 | ODM91117.1Bladder cancer-associated protein | C:GO:0016021 | |

| | | | |
|---|---|---|---|
| TRINITY_DN18228_c0_g1_i1_len_941_95989.p1_type_5prime_partial_len_119 | ---NA--- | | |
| TRINITY_DN19105_c0_g1_i1_len_720_163271.p1_type_complete_len_119 | ---NA--- | | |
| TRINITY_DN8240_c0_g1_i1_len_358_74313.p1_type_internal_len_120 | ODN02314.1Protein ovo | F:GO:0003676 | |
| TRINITY_DN4031_c0_g1_i1_len_365_209795.p1_type_5prime_partial_len_119 | ---NA--- | | |
| TRINITY_DN15846_c0_g1_i1_len_375_308024.p1_type_5prime_partial_len_119 | ---NA--- | | |
| TRINITY_DN2906_c0_g1_i1_len_1042_196173.p1_type_5prime_partial_len_119 | ---NA--- | | |
| TRINITY_DN8519_c0_g1_i1_len_358_74306.p1_type_internal_len_120 | ---NA--- | | |
| TRINITY_DN18830_c0_g1_i1_len_668_200503.p1_type_5prime_partial_len_119 | PSN37584.1von Willebrand factor A domain-containing protein 9 | C:GO:0032039 | |
| TRINITY_DN25679_c0_g1_i1_len_1424_233379.p2_type_5prime_partial_len_119 | ---NA--- | | |
| TRINITY_DN11110_c0_g1_i1_len_358_74337.p1_type_internal_len_120 | ---NA--- | | |
| TRINITY_DN12063_c0_g1_i1_len_423_71473.p1_type_5prime_partial_len_119 | XP_023221827.1ubiquitin-like-conjugating enzyme ATG3 isoform X2 | | |
| TRINITY_DN12791_c0_g1_i2_len_1455_158004.p3_type_5prime_partial_len_119 | ---NA--- | | |
| TRINITY_DN13121_c0_g1_i2_len_782_128186.p1_type_5prime_partial_len_119 | ---NA--- | | |
| TRINITY_DN24932_c0_g1_i2_len_3260_228698.p3_type_5prime_partial_len_119 | ---NA--- | | |
| TRINITY_DN7265_c0_g1_i2_len_357_210294.p1_type_internal_len_120 | ---NA--- | | |
| TRINITY_DN7089_c0_g1_i1_len_379_171091.p1_type_5prime_partial_len_119 | ---NA--- | | |

| | | | |
|---|---|---|---|
| TRINITY_DN26427_c0_g1_i1_len_359_13466.p1_type_internal_len_120 | ARK36624.1G-protein coupled receptor GRL101 | F:GO:0004930; P:GO:0007186; C:GO:0016021 | |
| TRINITY_DN3748_c0_g1_i1_len_357_172091.p2_type_internal_len_120 | XP_018015635.1 src substrate protein p85-like | | |
| TRINITY_DN31949_c0_g1_i1_len_360_13413.p1_type_internal_len_120 | ---NA--- | | |
| TRINITY_DN27456_c0_g1_i1_len_357_172128.p1_type_internal_len_120 | ---NA--- | | |
| TRINITY_DN24510_c0_g1_i1_len_357_172099.p1_type_internal_len_120 | ABC94529.1NO-sensitive soluble guanylyl cyclase beta 1 short isoform | F:GO:0000166; F:GO:0004383; P:GO:0006182; F:GO:0020037; P:GO:0035556 | EC:4.6.1.2 |
| TRINITY_DN23874_c0_g1_i1_len_357_13546.p1_type_internal_len_120 | ---NA--- | | |
| TRINITY_DN10500_c0_g1_i2_len_486_39013.p1_type_5prime_partial_len_119 | KNC20967.1S-adenosylmethionine synthase | F:GO:0000166; F:GO:0016740; F:GO:0043167; P:GO:0044237 | |
| TRINITY_DN18016_c0_g1_i2_len_394_307255.p1_type_5prime_partial_len_119 | ---NA--- | | |
| TRINITY_DN7872_c0_g1_i1_len_358_13497.p1_type_internal_len_120 | ---NA--- | | |
| TRINITY_DN11985_c0_g1_i3_len_357_210299.p1_type_internal_len_120 | XP_018007512.1 CAS1 domain-containing protein 1-like | | |
| TRINITY_DN6098_c0_g1_i2_len_361_171899.p1_type_internal_len_120 | ---NA--- | | |
| TRINITY_DN17299_c0_g1_i1_len_357_280735.p1_type_internal_len_120 | ---NA--- | | |
| TRINITY_DN29110_c0_g1_i1_len_361_171905.p1_type_internal_len_120 | ---NA--- | | |
| TRINITY_DN25708_c0_g1_i1_len_360_171948.p2_type_internal_len_120 | ---NA--- | | |
| TRINITY_DN29997_c0_g1_i1_len_360_171944.p1_type_internal_len_120 | ---NA--- | | |
| TRINITY_DN16215_c0_g1_i1_len_714_35559.p1_type_complete_len_119 | ---NA--- | | |

| | | | |
|---|---|---|---|
| TRINITY_DN8971_c0_g1_i1_len_357_210303.p1_type_internal_len_120 | ---NA--- | | |
| TRINITY_DN18427_c0_g1_i1_len_429_103320.p1_type_5prime_partial_len_119 | ---NA--- | | |
| TRINITY_DN3527_c0_g1_i1_len_360_171950.p1_type_internal_len_120 | ---NA--- | | |
| TRINITY_DN6407_c0_g1_i1_len_428_134344.p1_type_5prime_partial_len_119 | ---NA--- | | |
| TRINITY_DN18366_c0_g1_i2_len_393_307280.p1_type_5prime_partial_len_119 | ---NA--- | | |
| TRINITY_DN9158_c0_g1_i1_len_358_172045.p1_type_internal_len_120 | ---NA--- | | |
| TRINITY_DN31091_c0_g1_i1_len_359_172020.p1_type_internal_len_120 | ---NA--- | | |
| TRINITY_DN28617_c0_g1_i1_len_355_137448.p1_type_internal_len_119 | ---NA--- | | |
| TRINITY_DN34531_c0_g1_i1_len_356_210390.p1_type_internal_len_119 | ---NA--- | | |
| TRINITY_DN18907_c0_g1_i1_len_868_237248.p2_type_5prime_partial_len_118 | ---NA--- | | |
| TRINITY_DN39122_c0_g1_i1_len_355_210395.p1_type_internal_len_119 | XP_018020602.1 furin-like protease 2 | | |
| TRINITY_DN14948_c0_g1_i1_len_393_208345.p1_type_5prime_partial_len_118 | ---NA--- | | |
| TRINITY_DN10618_c0_g1_i1_len_657_164103.p2_type_complete_len_118 | XP_015912772.1retinal rod rhodopsin-sensitive cGMP 3',5'-cyclic phosphodiesterase subunit delta | F:GO:0004114; P:GO:0050953 | EC:3.1.4.17 |
| TRINITY_DN29889_c0_g1_i1_len_357_43243.p1_type_internal_len_119 | ---NA--- | | |
| TRINITY_DN4502_c0_g1_i1_len_355_210447.p1_type_internal_len_119 | XP_018020452.1 polypeptide N-acetylgalactosaminyltransferase 1-like | C:GO:0000139; P:GO:0006486; C:GO:0016021; F:GO:0016757; F:GO:0030246 | |

| | | | |
|---|---|---|---|
| TRINITY_DN892_c0_g1_i1_len_355_210398.p1_type_internal_len_119 | XP_013095076.1 neuroendocrine convertase 1-like isoform X3 | F:GO:0008236 | |
| TRINITY_DN38720_c0_g1_i1_len_358_210242.p1_type_internal_len_119 | ---NA--- | | |
| TRINITY_DN25755_c0_g1_i1_len_356_137411.p1_type_internal_len_119 | ---NA--- | | |
| TRINITY_DN38141_c0_g1_i1_len_355_210439.p2_type_internal_len_119 | ---NA--- | | |
| TRINITY_DN31300_c0_g1_i1_len_358_13522.p1_type_internal_len_119 | XP_023221379.1equilibrative nucleoside transporter 4-like | | |
| TRINITY_DN19870_c0_g1_i1_len_584_7237.p2_type_5prime_partial_len_118 | ---NA--- | | |
| TRINITY_DN27850_c0_g1_i1_len_1847_191492.p2_type_5prime_partial_len_118 | ---NA--- | | |
| TRINITY_DN37620_c0_g1_i1_len_355_210440.p1_type_internal_len_119 | ---NA--- | | |
| TRINITY_DN28431_c0_g2_i2_len_357_210271.p1_type_internal_len_119 | ---NA--- | | |
| TRINITY_DN26807_c0_g1_i1_len_1553_192800.p1_type_5prime_partial_len_118 | ---NA--- | | |
| TRINITY_DN12537_c0_g1_i3_len_2076_30264.p4_type_complete_len_118 | ---NA--- | | |
| TRINITY_DN14425_c0_g1_i1_len_453_244439.p1_type_3prime_partial_len_119 | ---NA--- | | |
| TRINITY_DN1445_c0_g1_i1_len_356_210381.p1_type_internal_len_119 | ---NA--- | | |
| TRINITY_DN27865_c0_g1_i1_len_1137_195402.p1_type_complete_len_118 | ---NA--- | | |
| TRINITY_DN14109_c0_g1_i1_len_457_205677.p1_type_3prime_partial_len_119 | ---NA--- | | |
| TRINITY_DN37857_c0_g1_i1_len_358_210222.p1_type_internal_len_119 | XP_013399832.1transformation/transcription domain-associated protein-like | C:GO:0000123; F:GO:0016301; P:GO:0016310; P:GO:0016573 | |

<table>
<tr><td>TRINITY_DN19803_c0_g1_i1_len_4174_187493.p1_type_complete_len_861</td><td>KZS22073.1putative G-protein coupled receptor Mth 12</td><td>F:GO:0004888; F:GO:0004930; P:GO:0007166; P:GO:0007186; C:GO:0016020; C:GO:0016021</td><td></td></tr>
<tr><td>2460|_|c18160_f2p1_2582_isoform_c18160_full_length_coverage_2_non_full_length_coverage_1_isoform_length_2582_13618</td><td>XP_018014490.1PREDICTED: uncharacterized protein LOC108671453, partial</td><td></td><td></td></tr>
<tr><td>2461|_|c2279_f6p8_2582_isoform_c2279_full_length_coverage_6_non_full_length_coverage_8_isoform_length_2582_11772</td><td>---NA---</td><td></td><td></td></tr>
<tr><td>2462|_|c18110_f2p1_2582_isoform_c18110_full_length_coverage_2_non_full_length_coverage_1_isoform_length_2582_22695</td><td>PFX29899.1Riboflavin kinase</td><td>F:GO:0008531; P:GO:0009231; P:GO:0016310</td><td>EC:2.7.1.26</td></tr>
<tr><td>2463|_|c2549_f3p3_2582_isoform_c2549_full_length_coverage_3_non_full_length_coverage_3_isoform_length_2582_16502</td><td>XP_018024884.1 protein abrupt-like isoform X19</td><td></td><td></td></tr>
<tr><td>2465|_|c2528_f4p1_2581_isoform_c2528_full_length_coverage_4_non_full_length_coverage_1_isoform_length_2581_16484</td><td>XP_018024221.1 E3 SUMO-protein ligase RanBP2-like isoform X2</td><td></td><td></td></tr>
<tr><td>2466|_|c10495_f2p3_2581_isoform_c10495_full_length_coverage_2_non_full_length_coverage_3_isoform_length_2581_17369</td><td>XP_023243255.1tubulin gamma-1 chain</td><td>C:GO:0000930; F:GO:0003924; F:GO:0005525; C:GO:0005874; P:GO:0007020; P:GO:0031122</td><td>EC:3.6.1.15</td></tr>
<tr><td>2468|_|c2022_f2p30_2581_isoform_c2022_full_length_coverage_2_non_full_length_coverage_30_isoform_length_2581_28157</td><td>AEB00820.1vasa-like protein</td><td>F:GO:0003676; F:GO:0004386; F:GO:0005524; F:GO:0008270</td><td>EC:3.6.1.15</td></tr>
<tr><td>2469|_|c10149_f2p4_2581_isoform_c10149_full_length_coverage_2_non_full_length_coverage_4_isoform_length_2581_17333</td><td>XP_023717986.1rab-like protein 2A</td><td>F:GO:0003924; F:GO:0005525</td><td>EC:3.6.1.15</td></tr>
<tr><td>2470|_|c2566_f2p0_2581_isoform_c2566_full_length_coverage_2_non_full_length_coverage_0_isoform_length_2581_16518</td><td>XP_018016678.1 choline transporter-like protein 2</td><td>C:GO:0016020; C:GO:0016021</td><td></td></tr>
<tr><td>TRINITY_DN20012_c0_g1_i1_len_801_97028.p1_type_complete_len_118</td><td>---NA---</td><td></td><td></td></tr>
</table>

| | | | |
|---|---|---|---|
| TRINITY_DN27134_c0_g1_i2_len_997_196571.p1_type_complete_len_118 | ---NA--- | | |
| TRINITY_DN337_c0_g1_i1_len_471_243869.p1_type_3prime_partial_len_119 | ---NA--- | | |
| TRINITY_DN13832_c0_g1_i1_len_354_137511.p1_type_internal_len_119 | ---NA--- | | |
| TRINITY_DN14583_c0_g1_i1_len_358_210231.p1_type_internal_len_119 | ---NA--- | | |
| TRINITY_DN19308_c0_g2_i1_len_356_13585.p1_type_internal_len_119 | ACL79884.1putative articulin p60 | | |
| TRINITY_DN25688_c1_g1_i3_len_1309_233994.p1_type_complete_len_118 | ---NA--- | | |
| TRINITY_DN1305_c0_g1_i1_len_473_205102.p1_type_5prime_partial_len_118 | ---NA--- | | |
| TRINITY_DN39204_c0_g1_i1_len_356_210376.p1_type_internal_len_119 | XP_018009194.1 tankyrase-1-like | | |
| TRINITY_DN30462_c0_g1_i1_len_357_43250.p1_type_internal_len_119 | ---NA--- | | |
| TRINITY_DN14488_c0_g1_i1_len_556_202753.p1_type_complete_len_118 | ---NA--- | | |
| TRINITY_DN16171_c0_g1_i1_len_356_210391.p1_type_internal_len_119 | ---NA--- | | |
| TRINITY_DN30764_c0_g1_i1_len_463_244120.p1_type_3prime_partial_len_119 | XP_018010966.1 histone-lysine N-methyltransferase SETD1B-A-like | F:GO:0003723 | |
| TRINITY_DN24596_c0_g1_i1_len_1251_194581.p2_type_5prime_partial_len_118 | ---NA--- | | |
| TRINITY_DN30002_c0_g1_i1_len_355_43318.p1_type_internal_len_119 | ---NA--- | | |
| TRINITY_DN1854_c0_g1_i2_len_355_43337.p1_type_3prime_partial_len_119 | ---NA--- | | |
| TRINITY_DN15031_c0_g1_i3_len_356_106320.p2_type_internal_len_119 | ---NA--- | | |

| | | | |
|---|---|---|---|
| TRINITY_DN13044_c0_g1_i1_len_595_165076.p1_type_5prime_partial_len_118 | ---NA--- | | |
| TRINITY_DN22597_c0_g1_i1_len_3389_153989.p2_type_complete_len_118 | ---NA--- | | |
| TRINITY_DN39982_c0_g1_i1_len_357_210262.p1_type_internal_len_119 | ASW35084.1Nesprin-1, partial | C:GO:0016021; C:GO:0034993; F:GO:0051015; P:GO:0090286 | |
| TRINITY_DN13789_c1_g1_i1_len_355_210435.p1_type_internal_len_119 | ---NA--- | | |
| TRINITY_DN21261_c0_g1_i1_len_960_236436.p1_type_5prime_partial_len_118 | ---NA--- | | |
| TRINITY_DN3841_c0_g1_i1_len_356_106302.p1_type_internal_len_119 | APF30038.1ionotropic glutamate receptor NMDA 2A | F:GO:0004970; C:GO:0016021; C:GO:0030054; P:GO:0034220; P:GO:0035235; C:GO:0045211 | |
| TRINITY_DN4582_c0_g1_i1_len_356_106296.p1_type_internal_len_119 | ---NA--- | | |
| TRINITY_DN24894_c0_g1_i1_len_356_106291.p1_type_5prime_partial_len_118 | ---NA--- | | |
| TRINITY_DN13929_c0_g1_i2_len_421_245604.p1_type_5prime_partial_len_118 | ---NA--- | | |
| TRINITY_DN5231_c0_g1_i1_len_355_210408.p1_type_internal_len_119 | ---NA--- | | |
| TRINITY_DN31551_c0_g1_i1_len_356_106308.p1_type_internal_len_119 | ---NA--- | | |
| TRINITY_DN22432_c0_g1_i1_len_1718_123679.p2_type_5prime_partial_len_118 | ---NA--- | | |
| TRINITY_DN25414_c0_g1_i1_len_1666_192228.p1_type_complete_len_118 | ---NA--- | | |
| TRINITY_DN18521_c1_g1_i3_len_354_106395.p1_type_internal_len_119 | ---NA--- | | |
| TRINITY_DN33211_c0_g1_i1_len_532_131649.p2_type_5prime_partial_len_118 | ---NA--- | | |
| TRINITY_DN19480_c0_g1_i2_len_1750_191858.p1_type_complete_len_118 | ---NA--- | | |

| | | | |
|---|---|---|---|
| TRINITY_DN2070_c0_g1_i1_len_354_106428.p1_type_internal_len_119 | ---NA--- | | |
| TRINITY_DN13391_c0_g1_i1_len_402_246327.p1_type_5prime_partial_len_118 | ---NA--- | | |
| TRINITY_DN23944_c0_g1_i1_len_784_198817.p1_type_complete_len_118 | ---NA--- | | |
| TRINITY_DN22736_c0_g1_i1_len_1244_194639.p1_type_complete_len_118 | ---NA--- | | |
| TRINITY_DN31228_c0_g1_i1_len_355_106351.p1_type_internal_len_119 | ---NA--- | | |
| TRINITY_DN973_c0_g1_i2_len_355_210417.p1_type_internal_len_119 | XP_018011765.1PREDICTED: uncharacterized protein LOC108668992 | | |
| TRINITY_DN21820_c0_g1_i6_len_1270_194449.p5_type_complete_len_118 | ---NA--- | | |
| TRINITY_DN23558_c1_g1_i2_len_2221_155545.p2_type_complete_len_118 | ABB73282.1reverse transcriptase | F:GO:0003964; P:GO:0006278 | |
| TRINITY_DN7927_c0_g1_i1_len_355_210421.p1_type_internal_len_119 | ---NA--- | | |
| TRINITY_DN35334_c0_g1_i1_len_472_205138.p1_type_complete_len_118 | ATQ64223.1hyperglycemic hormone 1-like protein | | |
| TRINITY_DN11458_c0_g1_i1_len_398_41558.p1_type_5prime_partial_len_118 | ---NA--- | | |
| TRINITY_DN12937_c0_g1_i1_len_357_210251.p1_type_internal_len_119 | XP_013118452.1 E3 ubiquitin-protein ligase MIB2 | F:GO:0046872 | |
| TRINITY_DN22925_c0_g1_i1_len_1959_156233.p3_type_3prime_partial_len_119 | ---NA--- | | |
| TRINITY_DN10420_c0_g1_i1_len_357_210250.p1_type_internal_len_119 | ---NA--- | | |
| TRINITY_DN27177_c0_g1_i1_len_357_137323.p1_type_internal_len_119 | ---NA--- | | |
| TRINITY_DN299_c0_g1_i1_len_356_210385.p1_type_internal_len_119 | XP_005742809.1 thrombospondin-1 | F:GO:0005488 | |

| | | | |
|---|---|---|---|
| TRINITY_DN40687_c0_g1_i1_len_356_210382.p1_type_internal_len_119 | XP_018008988.1 WD repeat and FYVE domain-containing protein 3-like | | |
| TRINITY_DN27699_c0_g1_i1_len_357_137333.p1_type_internal_len_119 | ---NA--- | | |
| TRINITY_DN31405_c0_g1_i1_len_358_106226.p1_type_5prime_partial_len_118 | ---NA--- | | |
| TRINITY_DN1904_c0_g1_i1_len_580_130694.p1_type_5prime_partial_len_118 | XP_017765529.1 piggyBac transposable element-derived protein 4-like | | |
| TRINITY_DN37462_c0_g1_i1_len_425_245456.p1_type_5prime_partial_len_118 | ---NA--- | | |
| TRINITY_DN9739_c0_g1_i1_len_390_208490.p1_type_3prime_partial_len_119 | AFU61129.1antilipopolysaccharide factor isoform 6 | | |
| TRINITY_DN15704_c0_g1_i1_len_395_307214.p1_type_5prime_partial_len_118 | ---NA--- | | |
| TRINITY_DN8314_c0_g1_i1_len_509_166875.p3_type_complete_len_118 | ---NA--- | | |
| TRINITY_DN41815_c0_g1_i1_len_357_210253.p1_type_internal_len_119 | ---NA--- | | |
| TRINITY_DN32743_c0_g1_i1_len_357_106254.p2_type_internal_len_119 | ---NA--- | | |
| TRINITY_DN13345_c0_g1_i1_len_609_201591.p2_type_complete_len_118 | XP_018014829.1 phosphoglucomutase-2-like | F:GO:0000287; P:GO:0005975; F:GO:0016868 | |
| TRINITY_DN23644_c0_g1_i1_len_1129_195458.p1_type_complete_len_118 | ---NA--- | | |
| TRINITY_DN15486_c0_g1_i1_len_542_37865.p1_type_5prime_partial_len_118 | ---NA--- | | |
| TRINITY_DN28892_c0_g1_i1_len_356_172141.p1_type_internal_len_119 | XP_018018741.1 sushi, von Willebrand factor type A, EGF and pentraxin domain-containing protein 1-like | | |
| TRINITY_DN24624_c0_g1_i1_len_356_172140.p1_type_internal_len_119 | XP_012348257.1 eukaryotic translation initiation factor 2-alpha kinase 4-like | | |
| TRINITY_DN24339_c0_g1_i1_len_356_172136.p1_type_internal_len_119 | XP_021177892.1sodium/glucose cotransporter 5 | C:GO:0016021; F:GO:0022857; P:GO:0055085 | |

| | | | |
|---|---|---|---|
| TRINITY_DN17983_c0_g1_i1_len_835_198207.p1_type_complete_len_118 | ---NA--- | | |
| TRINITY_DN9958_c0_g1_i1_len_354_210517.p1_type_internal_len_119 | ---NA--- | | |
| TRINITY_DN10474_c0_g1_i1_len_477_101890.p1_type_5prime_partial_len_118 | PNF26900.1Cytokine receptor-like factor 3 | | |
| TRINITY_DN25235_c0_g1_i3_len_1951_231260.p1_type_complete_len_118 | ---NA--- | | |
| TRINITY_DN32165_c0_g1_i1_len_354_210521.p1_type_internal_len_119 | ---NA--- | | |
| TRINITY_DN22982_c0_g1_i1_len_722_199687.p2_type_5prime_partial_len_118 | ---NA--- | | |
| TRINITY_DN24195_c0_g1_i1_len_357_172125.p1_type_internal_len_119 | ---NA--- | | |
| TRINITY_DN31167_c0_g1_i1_len_357_172115.p2_type_internal_len_119 | ---NA--- | | |
| TRINITY_DN812_c0_g1_i1_len_354_74517.p1_type_internal_len_119 | ---NA--- | | |
| TRINITY_DN21646_c0_g1_i1_len_354_13722.p1_type_internal_len_119 | AIB55756.1chlorophyll a-b binding protein 1C (chloroplast) (chloroplast) | C:GO:0009522; C:GO:0009523; C:GO:0009535; P:GO:0009765; C:GO:0016021; F:GO:0016168; P:GO:0018298 | |
| TRINITY_DN11454_c0_g1_i1_len_355_172241.p1_type_internal_len_119 | ---NA--- | | |
| TRINITY_DN10358_c0_g1_i1_len_549_275015.p1_type_5prime_partial_len_118 | ---NA--- | | |
| TRINITY_DN15980_c0_g1_i1_len_407_207727.p1_type_5prime_partial_len_118 | ---NA--- | | |
| TRINITY_DN8477_c0_g1_i1_len_354_210507.p1_type_internal_len_119 | ---NA--- | | |
| TRINITY_DN1537_c0_g1_i2_len_354_13712.p1_type_internal_len_119 | XP_022668513.1RNA-binding protein Musashi homolog Rbp6-like isoform X7 | | |

| | | | |
|---|---|---|---|
| TRINITY_DN3724_c0_g1_i1_len_372_209395.p1_type_5prime_partial_len_118 | XP_018010166.1 cuticle protein 7-like | | |
| TRINITY_DN30391_c0_g1_i1_len_355_172207.p1_type_internal_len_119 | ---NA--- | | |
| TRINITY_DN14797_c0_g1_i1_len_1294_158800.p2_type_5prime_partial_len_118 | ---NA--- | | |
| TRINITY_DN3053_c0_g1_i1_len_355_172204.p1_type_internal_len_119 | ---NA--- | | |
| TRINITY_DN30033_c0_g1_i1_len_2111_190517.p1_type_complete_len_118 | ---NA--- | | |
| TRINITY_DN28921_c0_g1_i1_len_355_172220.p1_type_internal_len_119 | ---NA--- | | |
| TRINITY_DN16585_c0_g1_i1_len_696_239280.p1_type_5prime_partial_len_118 | XP_018025307.1 mucin-17-like | | |
| TRINITY_DN14417_c0_g1_i1_len_356_308874.p1_type_internal_len_119 | ---NA--- | | |
| TRINITY_DN23644_c0_g1_i5_len_1093_160036.p2_type_5prime_partial_len_118 | ---NA--- | | |
| TRINITY_DN1140_c0_g1_i1_len_360_171974.p1_type_5prime_partial_len_118 | ---NA--- | | |
| TRINITY_DN10936_c0_g1_i1_len_361_171890.p1_type_5prime_partial_len_118 | ---NA--- | | |
| TRINITY_DN18746_c0_g2_i1_len_357_308859.p1_type_internal_len_119 | ANH56805.1sulfotransferase 3 | F:GO:0008146; F:GO:0016740 | |
| TRINITY_DN25273_c1_g1_i1_len_356_308860.p1_type_internal_len_119 | ---NA--- | | |
| TRINITY_DN27464_c0_g1_i2_len_1435_193443.p2_type_3prime_partial_len_119 | ---NA--- | | |
| TRINITY_DN7279_c0_g1_i1_len_415_207386.p1_type_5prime_partial_len_118 | ---NA--- | | |
| TRINITY_DN22833_c0_g1_i1_len_1878_231466.p1_type_complete_len_118 | ---NA--- | | |

<table>
<tr><td>TRINITY_DN950_c0_g1_i2_len_577_202266.p1_type_complete_len_118</td><td>---NA---</td><td></td><td></td></tr>
<tr><td>TRINITY_DN28904_c0_g1_i2_len_1693_192116.p1_type_complete_len_118</td><td>---NA---</td><td></td><td></td></tr>
<tr><td>TRINITY_DN21909_c0_g1_i1_len_510_275808.p1_type_5prime_partial_len_118</td><td>---NA---</td><td></td><td></td></tr>
<tr><td>TRINITY_DN2319_c0_g1_i1_len_446_206082.p2_type_5prime_partial_len_118</td><td>---NA---</td><td></td><td></td></tr>
<tr><td>TRINITY_DN21851_c0_g1_i1_len_3081_294855.p1_type_complete_len_860</td><td>KZS05877.1putative E3 ubiquitin-protein ligase Topors</td><td>F:GO:0016874</td><td></td></tr>
<tr><td>TRINITY_DN24257_c0_g1_i1_len_3079_188407.p1_type_complete_len_860</td><td>XP_023714271.1transmembrane channel-like protein 5</td><td>C:GO:0005887; C:GO:0016020; C:GO:0016021</td><td></td></tr>
<tr><td>TRINITY_DN27859_c0_g1_i1_len_2786_188880.p1_type_5prime_partial_len_860</td><td>KFM77482.1Cyclin-D-binding Myb-like transcription factor 1</td><td>F:GO:0003677</td><td></td></tr>
<tr><td>2475|_|c5437_f2p0_2580_isoform_c5437_full_length_coverage_2_non_full_length_coverage_0_isoform_length_2580_21524</td><td>---NA---</td><td></td><td></td></tr>
<tr><td>TRINITY_DN29611_c0_g1_i2_len_3155_188310.p1_type_complete_len_860</td><td>XP_018018963.1 poly [ADP-ribose] polymerase 2-like isoform X2</td><td></td><td></td></tr>
<tr><td>2477|_|c10306_f2p0_2580_isoform_c10306_full_length_coverage_2_non_full_length_coverage_0_isoform_length_2580_17345</td><td>XP_018013670.1 conserved oligomeric Golgi complex subunit 4-like isoform X1</td><td></td><td></td></tr>
<tr><td>2478|_|c20593_f2p8_2579_isoform_c20593_full_length_coverage_2_non_full_length_coverage_8_isoform_length_2579_22815</td><td>---NA---</td><td></td><td></td></tr>
<tr><td>2479|_|c2760_f2p2_2579_isoform_c2760_full_length_coverage_2_non_full_length_coverage_2_isoform_length_2579_12138</td><td>---NA---</td><td></td><td></td></tr>
<tr><td>2480|_|c2625_f3p0_2579_isoform_c2625_full_length_coverage_3_non_full_length_coverage_0_isoform_length_2579_21097</td><td>XP_026142221.1ketohexokinase-like isoform X2</td><td></td><td></td></tr>
</table>

| | | | |
|---|---|---|---|
| 2482\|_\|c2574_f3p1_2579_isoform_c2574_full_length_coverage_3_non_full_length_coverage_1_isoform_length_2579_11980 | ---NA--- | | |
| TRINITY_DN10554_c0_g1_i1_len_1117_159860.p1_type_complete_len_118 | ---NA--- | | |
| TRINITY_DN25134_c0_g1_i1_len_354_309012.p1_type_internal_len_119 | XP_018021206.1 neurogenic protein big brain-like | | |
| TRINITY_DN20713_c0_g1_i1_len_1016_126449.p2_type_5prime_partial_len_118 | ---NA--- | | |
| TRINITY_DN5206_c0_g1_i1_len_498_276093.p1_type_complete_len_118 | ---NA--- | | |
| TRINITY_DN1660_c0_g1_i1_len_656_273326.p1_type_complete_len_118 | ACY64754.1double WAP domain-containing protein | C:GO:0005576; P:GO:0010466; F:GO:0030414 | |
| TRINITY_DN16977_c0_g1_i1_len_842_64204.p1_type_complete_len_118 | ---NA--- | | |
| TRINITY_DN717_c0_g1_i1_len_354_210473.p1_type_internal_len_119 | XP_019618009.1PREDICTED: uncharacterized protein LOC109465272 | | |
| TRINITY_DN36656_c0_g1_i1_len_365_209816.p1_type_5prime_partial_len_118 | ---NA--- | | |
| TRINITY_DN17571_c0_g1_i1_len_924_270740.p1_type_5prime_partial_len_118 | ---NA--- | | |
| TRINITY_DN8718_c0_g1_i2_len_479_204923.p1_type_5prime_partial_len_118 | ---NA--- | | |
| TRINITY_DN26336_c0_g2_i1_len_2192_230533.p2_type_complete_len_118 | ---NA--- | | |
| TRINITY_DN998_c0_g1_i1_len_354_210487.p1_type_internal_len_119 | KZS11050.1Protein FAM91A1 | | |
| TRINITY_DN4403_c0_g1_i1_len_358_210188.p1_type_internal_len_119 | XP_022242960.1arfGAP with SH3 domain, ANK repeat and PH domain-containing protein-like isoform X2 | | |
| TRINITY_DN18900_c0_g1_i1_len_365_209822.p1_type_5prime_partial_len_118 | ---NA--- | | |

| | | | |
|---|---|---|---|
| TRINITY_DN5102_c0_g1_i1_len_355_13631.p1_type_internal_len_119 | ---NA--- | | |
| TRINITY_DN35984_c0_g1_i1_len_354_210482.p1_type_internal_len_119 | ALT10384.1juvenile hormone esterase-like protein 2 | F:GO:0016787 | |
| TRINITY_DN19564_c0_g1_i2_len_823_237705.p1_type_3prime_partial_len_119 | ---NA--- | | |
| TRINITY_DN7813_c0_g1_i1_len_358_210220.p1_type_internal_len_119 | ---NA--- | | |
| TRINITY_DN14597_c0_g1_i1_len_1156_195263.p1_type_5prime_partial_len_118 | BAF75375.1ecdysone receptor | F:GO:0003707; F:GO:0004879; C:GO:0005634; P:GO:0006355; F:GO:0008270; P:GO:0035076; F:GO:0035100; F:GO:0043565 | |
| TRINITY_DN10209_c0_g1_i1_len_354_210462.p1_type_internal_len_119 | ---NA--- | | |
| TRINITY_DN10530_c0_g1_i1_len_504_242933.p1_type_3prime_partial_len_119 | ---NA--- | | |
| TRINITY_DN3176_c0_g1_i2_len_495_204408.p1_type_5prime_partial_len_118 | ---NA--- | | |
| TRINITY_DN21120_c0_g1_i1_len_1485_193180.p3_type_complete_len_118 | ---NA--- | | |
| TRINITY_DN21249_c0_g1_i1_len_2303_230232.p2_type_complete_len_118 | ---NA--- | | |
| TRINITY_DN18759_c0_g1_i1_len_404_207829.p1_type_5prime_partial_len_118 | ---NA--- | | |
| TRINITY_DN8213_c0_g1_i1_len_397_11830.p1_type_5prime_partial_len_118 | ---NA--- | | |
| TRINITY_DN36628_c0_g1_i1_len_358_210174.p2_type_internal_len_119 | ANH58178.1delta class glutathione S-transferase | F:GO:0016740 | |
| TRINITY_DN23249_c0_g1_i10_len_1311_124962.p4_type_complete_len_118 | ---NA--- | | |
| TRINITY_DN28014_c0_g1_i1_len_356_74428.p1_type_internal_len_119 | ---NA--- | | |

| | | | |
|---|---|---|---|
| TRINITY_DN33646_c0_g1_i1_len_356_74433.p1_type_internal_len_119 | ---NA--- | | |
| TRINITY_DN34893_c0_g1_i1_len_356_74450.p1_type_internal_len_119 | ---NA--- | | |
| TRINITY_DN3443_c0_g1_i1_len_356_74452.p1_type_internal_len_119 | ---NA--- | | |
| TRINITY_DN2594_c0_g1_i1_len_356_74453.p1_type_internal_len_119 | ---NA--- | | |
| TRINITY_DN29873_c0_g1_i1_len_1995_190928.p2_type_complete_len_118 | ---NA--- | | |
| TRINITY_DN35362_c0_g1_i1_len_354_210500.p1_type_internal_len_119 | ---NA--- | | |
| TRINITY_DN24433_c0_g1_i1_len_605_99338.p1_type_5prime_partial_len_118 | ---NA--- | | |
| TRINITY_DN24657_c0_g1_i1_len_838_198166.p2_type_5prime_partial_len_118 | ---NA--- | | |
| TRINITY_DN17537_c0_g1_i2_len_795_162467.p1_type_complete_len_118 | ---NA--- | | |
| TRINITY_DN31395_c0_g1_i1_len_357_74386.p1_type_internal_len_119 | XP_023226357.1tetratricopeptide repeat protein 7B-like | | |
| TRINITY_DN8134_c0_g1_i1_len_354_210494.p1_type_internal_len_119 | ---NA--- | | |
| TRINITY_DN21998_c0_g1_i1_len_357_74389.p1_type_internal_len_119 | ABB73282.1reverse transcriptase | F:GO:0003964; P:GO:0006278 | EC:2.7.7.49 |
| TRINITY_DN71_c0_g1_i1_len_354_210497.p1_type_internal_len_119 | ---NA--- | | |
| TRINITY_DN25100_c0_g2_i1_len_357_74368.p1_type_internal_len_119 | ---NA--- | | |
| TRINITY_DN26010_c0_g1_i1_len_356_74412.p1_type_internal_len_119 | ---NA--- | | |
| TRINITY_DN3384_c0_g1_i1_len_355_13679.p1_type_internal_len_119 | ---NA--- | | |

| | | | |
|---|---|---|---|
| TRINITY_DN23294_c0_g1_i1_len_384_42093.p1_type_5prime_partial_len_118 | ---NA--- | | |
| TRINITY_DN6815_c0_g1_i2_len_356_210340.p1_type_internal_len_119 | ---NA--- | | |
| TRINITY_DN32588_c0_g1_i1_len_354_248573.p1_type_internal_len_119 | XP_018014922.1PREDICTED: uncharacterized protein LOC108671838 | | |
| TRINITY_DN28612_c0_g1_i1_len_355_280838.p1_type_internal_len_119 | XP_018006837.1 cadherin-89D-like | | |
| TRINITY_DN1265_c0_g1_i1_len_356_210359.p1_type_internal_len_119 | ABP97431.1alpha 2 macroglobulin | F:GO:0004866; C:GO:0005615; P:GO:0010951 | |
| TRINITY_DN29425_c0_g1_i1_len_358_248394.p1_type_internal_len_119 | ---NA--- | | |
| TRINITY_DN22020_c0_g1_i1_len_1900_59566.p2_type_5prime_partial_len_118 | XP_018022500.1 EH domain-containing protein 1-like isoform X2 | | |
| TRINITY_DN31277_c0_g1_i1_len_356_248476.p1_type_internal_len_119 | AKG54423.1interferon regulatory factor | F:GO:0044212 | |
| TRINITY_DN32581_c0_g1_i1_len_358_248391.p1_type_internal_len_119 | KZS09360.1Protein crumbs | C:GO:0016020 | |
| TRINITY_DN19678_c0_g1_i1_len_3645_228373.p2_type_5prime_partial_len_118 | ---NA--- | | |
| TRINITY_DN31277_c0_g1_i1_len_356_210364.p1_type_internal_len_119 | XP_018023970.1 DBH-like monooxygenase protein 1 | | |
| TRINITY_DN32844_c0_g1_i1_len_356_248479.p1_type_internal_len_119 | XP_018010562.1 vacuolar protein sorting-associated protein 13D-like | | |
| TRINITY_DN27425_c0_g1_i1_len_356_248481.p1_type_internal_len_119 | PNJ70490.1OTX2 isoform 5 | F:GO:0003700; C:GO:0005634; P:GO:0006355; P:GO:0007275; F:GO:0043565 | |
| TRINITY_DN3733_c0_g1_i1_len_356_248485.p1_type_internal_len_119 | ---NA--- | | |
| TRINITY_DN7797_c0_g1_i1_len_356_210369.p2_type_internal_len_119 | ---NA--- | | |
| TRINITY_DN8813_c0_g1_i1_len_356_248457.p1_type_internal_len_119 | ---NA--- | | |

| | | | |
|---|---|---|---|
| TRINITY_DN27082_c0_g1_i1_len_520_100850.p1_type_3prime_partial_len_119 | ---NA--- | | |
| TRINITY_DN3151_c0_g1_i1_len_356_248462.p1_type_internal_len_119 | ---NA--- | | |
| TRINITY_DN27292_c0_g1_i1_len_355_280825.p1_type_internal_len_119 | ---NA--- | | |
| TRINITY_DN36436_c0_g1_i1_len_357_248451.p2_type_internal_len_119 | ADE44090.1phosphoinositide 3-kinase isoform a | F:GO:0005524; C:GO:0005737; C:GO:0005886; C:GO:0005942; P:GO:0006468; P:GO:0014065; F:GO:0016303; P:GO:0016477; P:GO:0030168; F:GO:0035005; P:GO:0036092; P:GO:0046854; F:GO:0046934 | EC:2.7.1.153; EC:2.7.1.154; EC:2.7.1.137 |
| TRINITY_DN504_c0_g1_i1_len_356_210372.p1_type_internal_len_119 | ---NA--- | | |
| TRINITY_DN42236_c0_g1_i1_len_357_210323.p1_type_internal_len_119 | ODM98856.1Spondin-1 | F:GO:0004867; P:GO:0010951 | |
| TRINITY_DN28471_c0_g1_i1_len_356_248464.p1_type_internal_len_119 | ---NA--- | | |
| TRINITY_DN32608_c0_g1_i1_len_423_103518.p1_type_5prime_partial_len_118 | ---NA--- | | |
| TRINITY_DN28759_c0_g1_i1_len_354_248592.p1_type_internal_len_119 | XP_017875266.1cAMP-specific 3',5'-cyclic phosphodiesterase | F:GO:0004114; P:GO:0007165; C:GO:0016021; F:GO:0046872 | EC:3.1.4.17 |
| TRINITY_DN33625_c0_g1_i1_len_354_248586.p1_type_internal_len_119 | PNF22200.1SPRY domain-containing protein 3 | | |
| TRINITY_DN9095_c0_g1_i1_len_356_248463.p1_type_internal_len_119 | XP_018012233.1 epidermal growth factor receptor substrate 15-like 1 isoform X2 | | |
| TRINITY_DN22635_c0_g1_i1_len_766_199038.p1_type_5prime_partial_len_118 | ---NA--- | | |
| TRINITY_DN37858_c0_g1_i1_len_355_248535.p2_type_internal_len_119 | XP_021946086.1zinc finger protein 320-like | F:GO:0003676 | |
| TRINITY_DN39959_c0_g1_i1_len_356_210331.p1_type_internal_len_119 | ---NA--- | | |

| | | | |
|---|---|---|---|
| TRINITY_DN6300_c0_g1_i2_len_355_248498.p2_type_internal_len_119 | ---NA--- | | |
| TRINITY_DN35123_c0_g1_i1_len_355_248544.p1_type_internal_len_119 | ---NA--- | | |
| TRINITY_DN18032_c0_g1_i1_len_378_73284.p1_type_5prime_partial_len_118 | ---NA--- | | |
| TRINITY_DN38724_c0_g1_i1_len_645_200902.p1_type_5prime_partial_len_118 | ---NA--- | | |
| TRINITY_DN18805_c0_g1_i4_len_356_210329.p2_type_internal_len_119 | ---NA--- | | |
| TRINITY_DN81_c0_g1_i1_len_355_248525.p1_type_internal_len_119 | ---NA--- | | |
| TRINITY_DN32730_c0_g1_i1_len_358_248353.p1_type_internal_len_119 | ---NA--- | | |
| TRINITY_DN9545_c0_g1_i1_len_356_280782.p1_type_internal_len_119 | ---NA--- | | |
| TRINITY_DN9020_c0_g1_i1_len_370_105621.p1_type_5prime_partial_len_118 | PSN36432.1hypothetical protein C0J52_11657 | C:GO:0016020 | |
| TRINITY_DN29158_c0_g1_i1_len_355_248534.p1_type_internal_len_119 | ---NA--- | | |
| TRINITY_DN2115_c0_g1_i1_len_355_248532.p1_type_internal_len_119 | ---NA--- | | |
| TRINITY_DN36531_c0_g1_i1_len_358_248363.p1_type_internal_len_119 | ---NA--- | | |
| TRINITY_DN37784_c0_g1_i1_len_355_248516.p1_type_internal_len_119 | ---NA--- | | |
| TRINITY_DN11316_c0_g1_i1_len_357_210305.p1_type_internal_len_119 | ---NA--- | | |
| TRINITY_DN29939_c0_g1_i1_len_356_248489.p1_type_internal_len_119 | XP_021346125.1DNA repair protein REV1-like isoform X1 | P:GO:0006281 | |
| TRINITY_DN10187_c0_g1_i1_len_355_248556.p2_type_internal_len_119 | ---NA--- | | |

<table>
<tr><td></td><td></td><td></td><td></td></tr>
<tr><td>TRINITY_DN2584_c0_g1_i1_len_355_248557.p1_type_internal_len_119</td><td>---NA---</td><td></td><td></td></tr>
<tr><td>TRINITY_DN32559_c0_g1_i1_len_358_248387.p1_type_internal_len_119</td><td>---NA---</td><td></td><td></td></tr>
<tr><td>TRINITY_DN788_c0_g1_i1_len_356_248487.p1_type_internal_len_119</td><td>XP_022207524.1geranylgeranyl transferase type-1 subunit beta-like</td><td>F:GO:0003824</td><td></td></tr>
<tr><td>TRINITY_DN27682_c0_g1_i1_len_356_280801.p1_type_internal_len_119</td><td>XP_018011187.1 alpha-1,3-mannosyl-glycoprotein 2-beta-N-acetylglucosaminyltransferase-like</td><td>P:GO:0006486; F:GO:0008375; C:GO:0016021</td><td></td></tr>
<tr><td>TRINITY_DN31332_c0_g1_i1_len_355_280857.p1_type_internal_len_119</td><td>---NA---</td><td></td><td></td></tr>
<tr><td>TRINITY_DN35707_c0_g1_i1_len_355_248546.p1_type_internal_len_119</td><td>CRK93649.1CLUMA_CG007178, isoform A</td><td>C:GO:0016020; C:GO:0016021; P:GO:0055085</td><td></td></tr>
<tr><td>TRINITY_DN39376_c0_g1_i1_len_356_210342.p1_type_internal_len_119</td><td>KDR23772.1hypothetical protein L798_11774</td><td></td><td></td></tr>
<tr><td>TRINITY_DN2307_c0_g1_i1_len_376_73415.p1_type_5prime_partial_len_118</td><td>---NA---</td><td></td><td></td></tr>
<tr><td>TRINITY_DN32514_c0_g1_i1_len_356_248492.p1_type_internal_len_119</td><td>XP_022203755.1protein sprint-like</td><td></td><td></td></tr>
<tr><td>2483|_|c4641_f2p34_2579_isoform_c4641_full_length_coverage_2_non_full_length_coverage_34_isoform_length_2579_21457</td><td>ARE72527.1importin subunit alpha-1</td><td>C:GO:0005634; C:GO:0005737; P:GO:0006606; F:GO:0008565; F:GO:0061608</td><td></td></tr>
<tr><td>2485|_|c25347_f2p1_2578_isoform_c25347_full_length_coverage_2_non_full_length_coverage_1_isoform_length_2578_22967</td><td>---NA---</td><td></td><td></td></tr>
<tr><td>2486|_|c18103_f3p2_2577_isoform_c18103_full_length_coverage_3_non_full_length_coverage_2_isoform_length_2577_22691</td><td>XP_015750951.1 transcription factor Dp-1-like</td><td>P:GO:0000278; F:GO:0003700; F:GO:0003712; C:GO:0005667; P:GO:0006355; F:GO:0008134; P:GO:0051726</td><td></td></tr>
<tr><td>2487|_|c2710_f3p15_2577_isoform_c2710_full_length_coverage_3_non_full_length_coverage_15_isoform_length_2577_16637</td><td>AYJ76527.1cell division cycle 42 protein</td><td>F:GO:0003924; F:GO:0005525; P:GO:0007264; P:GO:0051301</td><td>EC:3.6.1.15</td></tr>
</table>

| | | | |
|---|---|---|---|
| 2488\|_\|c18102_f2p1_2577_isoform_c18102_full_length_coverage_2_non_full_length_coverage_1_isoform_length_2577_17954 | ---NA--- | | |
| TRINITY_DN23479_c0_g1_i4_len_4262_228051.p1_type_complete_len_859 | ATU31743.1extended synaptotagmin-1-like protein | P:GO:0006869; F:GO:0008289; C:GO:0016021; P:GO:0061817 | |
| TRINITY_DN20990_c0_g1_i1_len_2866_58260.p1_type_complete_len_859 | XP_018009920.1 tRNA (uracil-5-)-methyltransferase homolog A-like | P:GO:0008152; F:GO:0016740 | |
| 2491\|_\|c9602_f2p2_2576_isoform_c9602_full_length_coverage_2_non_full_length_coverage_2_isoform_length_2576_21882 | XP_026280814.1acylamino-acid-releasing enzyme-like isoform X2 | | |
| 2492\|_\|c15379_f34p18_2576_isoform_c15379_full_length_coverage_34_non_full_length_coverage_18_isoform_length_2576_13224 | XP_018023312.1 DNA replication licensing factor MCM6-like | | |
| 2493\|_\|c17855_f4p2_2576_isoform_c17855_full_length_coverage_4_non_full_length_coverage_2_isoform_length_2576_17821 | ---NA--- | | |
| TRINITY_DN33307_c0_g1_i1_len_357_210304.p1_type_internal_len_119 | ---NA--- | | |
| TRINITY_DN32523_c0_g1_i1_len_356_210327.p1_type_internal_len_119 | ---NA--- | | |
| TRINITY_DN16598_c0_g1_i1_len_553_67954.p2_type_5prime_partial_len_118 | ---NA--- | | |
| TRINITY_DN15351_c0_g1_i2_len_747_35231.p2_type_5prime_partial_len_118 | XP_024222695.1probable E3 ubiquitin-protein ligase MGRN1 isoform X2 | | |
| TRINITY_DN17897_c0_g1_i1_len_771_198978.p1_type_3prime_partial_len_119 | ---NA--- | | |
| TRINITY_DN18788_c0_g1_i1_len_402_306973.p1_type_5prime_partial_len_118 | ---NA--- | | |
| TRINITY_DN31910_c0_g1_i1_len_357_248439.p1_type_internal_len_119 | ---NA--- | | |
| TRINITY_DN40751_c0_g1_i1_len_356_210348.p2_type_internal_len_119 | ---NA--- | | |

| | | | |
|---|---|---|---|
| TRINITY_DN2280_c0_g1_i1_len_356_210362.p2_type_internal_len_119 | ACX47560.1ryanodine receptor | F:GO:0005219; F:GO:0005509; C:GO:0005623; C:GO:0016021; P:GO:0051209 | |
| TRINITY_DN6454_c0_g1_i1_len_356_210347.p1_type_internal_len_119 | ---NA--- | | |
| TRINITY_DN29224_c0_g1_i1_len_357_248416.p2_type_internal_len_119 | ---NA--- | | |
| TRINITY_DN26652_c0_g1_i1_len_357_248434.p1_type_internal_len_119 | XP_018022681.1 major facilitator superfamily domain-containing protein 6-like | | |
| TRINITY_DN5435_c0_g1_i1_len_378_73327.p1_type_5prime_partial_len_118 | ---NA--- | | |
| TRINITY_DN24590_c0_g1_i1_len_446_133741.p1_type_5prime_partial_len_118 | ---NA--- | | |
| TRINITY_DN6695_c0_g1_i1_len_357_210324.p1_type_internal_len_119 | XP_025086787.1 E3 ubiquitin-protein ligase HERC2-like | F:GO:0004842; F:GO:0008270; P:GO:0016567 | |
| TRINITY_DN2168_c0_g1_i1_len_356_280818.p1_type_internal_len_119 | ---NA--- | | |
| TRINITY_DN34287_c0_g1_i1_len_356_210349.p1_type_internal_len_119 | ---NA--- | | |
| TRINITY_DN30318_c0_g1_i1_len_357_280733.p1_type_internal_len_119 | ---NA--- | | |
| TRINITY_DN8355_c0_g1_i1_len_356_210326.p1_type_internal_len_119 | ---NA--- | | |
| TRINITY_DN23544_c0_g1_i1_len_356_280764.p1_type_internal_len_119 | ---NA--- | | |
| TRINITY_DN14126_c0_g1_i1_len_357_248411.p2_type_internal_len_119 | ---NA--- | | |
| TRINITY_DN36010_c0_g1_i1_len_357_248427.p1_type_internal_len_119 | ---NA--- | | |
| TRINITY_DN33148_c0_g1_i1_len_354_172270.p2_type_internal_len_118 | ARK19985.1regucalcin-like protein 2 | F:GO:0005509; F:GO:0030234; P:GO:0050790 | |

| | | | |
|---|---|---|---|
| TRINITY_DN10572_c0_g1_i1_len_351_248726.p1_type_internal_len_118 | ---NA--- | | |
| TRINITY_DN29526_c0_g1_i1_len_376_136382.p1_type_5prime_partial_len_117 | ---NA--- | | |
| TRINITY_DN31259_c0_g1_i1_len_354_13714.p1_type_internal_len_118 | XP_015840547.1 fat-like cadherin-related tumor suppressor homolog isoform X6 | | |
| TRINITY_DN25979_c0_g1_i1_len_351_172425.p1_type_internal_len_118 | ---NA--- | | |
| TRINITY_DN36848_c0_g1_i1_len_1007_236064.p1_type_complete_len_117 | ---NA--- | | |
| TRINITY_DN17509_c0_g1_i1_len_353_137553.p1_type_internal_len_118 | ---NA--- | | |
| TRINITY_DN17835_c0_g1_i1_len_500_204245.p1_type_3prime_partial_len_118 | ---NA--- | | |
| TRINITY_DN24790_c0_g1_i1_len_351_281037.p1_type_internal_len_118 | ---NA--- | | |
| TRINITY_DN16543_c0_g1_i1_len_1009_236042.p1_type_complete_len_117 | XP_018026817.1 mitochondrial import inner membrane translocase subunit TIM14-like | | |
| TRINITY_DN22348_c0_g1_i1_len_351_281033.p1_type_internal_len_118 | ---NA--- | | |
| TRINITY_DN23801_c0_g1_i1_len_3405_228563.p3_type_complete_len_117 | ---NA--- | | |
| TRINITY_DN20675_c0_g1_i2_len_1083_235501.p1_type_complete_len_117 | ---NA--- | | |
| TRINITY_DN24956_c0_g1_i1_len_1021_196382.p2_type_5prime_partial_len_117 | ---NA--- | | |
| TRINITY_DN30965_c0_g3_i1_len_2501_189446.p2_type_complete_len_117 | XP_014780028.1 transmembrane protein 230-like isoform X1 | C:GO:0016021 | |
| TRINITY_DN21966_c0_g1_i1_len_1337_158551.p2_type_5prime_partial_len_117 | ---NA--- | | |
| TRINITY_DN16641_c0_g1_i2_len_1624_232435.p3_type_complete_len_117 | ---NA--- | | |

| | | | |
|---|---|---|---|
| TRINITY_DN1126_c0_g1_i1_len_353_137556.p1_type_internal_len_118 | XP_018027076.1 otoferlin-like | | |
| TRINITY_DN19544_c0_g1_i1_len_351_281035.p1_type_internal_len_118 | ---NA--- | | |
| TRINITY_DN18663_c0_g1_i1_len_635_201119.p1_type_5prime_partial_len_117 | ---NA--- | | |
| TRINITY_DN10874_c0_g1_i3_len_354_172263.p1_type_internal_len_118 | ---NA--- | | |
| TRINITY_DN33273_c0_g1_i1_len_351_248729.p1_type_internal_len_118 | ---NA--- | | |
| TRINITY_DN39388_c0_g1_i1_len_352_210626.p1_type_internal_len_118 | XP_026284270.1fat-like cadherin-related tumor suppressor homolog | | |
| TRINITY_DN24956_c0_g1_i1_len_354_13701.p1_type_internal_len_118 | ---NA--- | | |
| TRINITY_DN18913_c0_g1_i1_len_468_133158.p1_type_5prime_partial_len_117 | ---NA--- | | |
| TRINITY_DN16061_c0_g1_i2_len_354_74534.p1_type_3prime_partial_len_118 | ---NA--- | | |
| TRINITY_DN22582_c0_g1_i1_len_401_104331.p1_type_5prime_partial_len_117 | ---NA--- | | |
| TRINITY_DN17795_c0_g1_i2_len_474_205064.p1_type_5prime_partial_len_117 | ---NA--- | | |
| TRINITY_DN8692_c0_g1_i1_len_354_280862.p1_type_internal_len_118 | ---NA--- | | |
| TRINITY_DN25872_c0_g1_i1_len_2476_229841.p1_type_complete_len_117 | ---NA--- | | |
| TRINITY_DN14952_c0_g1_i1_len_644_6369.p2_type_5prime_partial_len_117 | ---NA--- | | |
| TRINITY_DN17602_c0_g1_i1_len_1034_298622.p2_type_complete_len_117 | ---NA--- | | |
| TRINITY_DN20290_c0_g1_i2_len_935_63518.p1_type_complete_len_117 | ---NA--- | | |

| | | | |
|---|---|---|---|
| TRINITY_DN18884_c0_g1_i1_len_2023_156044.p4_type_complete_len_117 | ---NA--- | | |
| TRINITY_DN32504_c0_g1_i1_len_352_137579.p1_type_internal_len_118 | ---NA--- | | |
| TRINITY_DN14689_c0_g1_i1_len_698_239227.p2_type_complete_len_117 | ---NA--- | | |
| TRINITY_DN25253_c0_g1_i1_len_354_13725.p1_type_internal_len_118 | XP_018020023.1 spectrin beta chain, non-erythrocytic 1-like | | |
| TRINITY_DN8830_c0_g1_i1_len_352_137576.p1_type_internal_len_118 | ---NA--- | | |
| TRINITY_DN7229_c0_g1_i2_len_441_133906.p1_type_complete_len_117 | ---NA--- | | |
| TRINITY_DN10553_c0_g1_i1_len_351_172417.p1_type_internal_len_118 | XP_018009219.1 putative glycine-rich cell wall structural protein 1 | F:GO:0030246 | |
| TRINITY_DN32012_c0_g1_i1_len_355_172203.p1_type_internal_len_118 | ---NA--- | | |
| TRINITY_DN18071_c0_g1_i6_len_1246_94286.p3_type_5prime_partial_len_117 | ---NA--- | | |
| TRINITY_DN16163_c0_g1_i1_len_1008_270151.p1_type_complete_len_117 | ---NA--- | | |
| TRINITY_DN15980_c0_g1_i1_len_410_169830.p1_type_complete_len_117 | ---NA--- | | |
| TRINITY_DN997_c0_g1_i1_len_421_245613.p1_type_5prime_partial_len_117 | ---NA--- | | |
| TRINITY_DN30174_c0_g1_i5_len_2828_188814.p4_type_complete_len_117 | ---NA--- | | |
| TRINITY_DN22035_c0_g1_i4_len_944_95962.p1_type_3prime_partial_len_118 | ---NA--- | | |
| TRINITY_DN29876_c0_g1_i3_len_2120_190490.p1_type_5prime_partial_len_117 | ---NA--- | | |
| TRINITY_DN23406_c0_g1_i1_len_442_102914.p1_type_5prime_partial_len_117 | ---NA--- | | |

| | | | |
|---|---|---|---|
| TRINITY_DN12935_c0_g1_i1_len_472_102034.p1_type_5prime_partial_len_117 | ---NA--- | | |
| TRINITY_DN19173_c0_g1_i1_len_483_243504.p1_type_5prime_partial_len_117 | ---NA--- | | |
| TRINITY_DN4040_c0_g1_i1_len_1246_94284.p2_type_5prime_partial_len_117 | ---NA--- | | |
| TRINITY_DN16192_c0_g1_i2_len_406_207748.p2_type_3prime_partial_len_118 | ---NA--- | | |
| TRINITY_DN3437_c0_g1_i1_len_419_169476.p1_type_5prime_partial_len_117 | ---NA--- | | |
| TRINITY_DN29217_c0_g1_i2_len_1960_191046.p1_type_complete_len_117 | ---NA--- | | |
| TRINITY_DN29100_c0_g1_i1_len_485_304505.p1_type_3prime_partial_len_118 | XP_013868404.1 gastrula zinc finger protein XlCGF8.2DB-like | F:GO:0003676 | |
| TRINITY_DN32168_c0_g1_i1_len_353_172348.p1_type_internal_len_118 | XP_023715499.1histone demethylase UTY isoform X1 | | |
| TRINITY_DN18508_c0_g1_i1_len_353_137518.p3_type_internal_len_118 | ---NA--- | | |
| TRINITY_DN38896_c0_g1_i1_len_351_210703.p1_type_internal_len_118 | ---NA--- | | |
| TRINITY_DN4163_c0_g1_i1_len_353_43416.p1_type_internal_len_118 | ---NA--- | | |
| TRINITY_DN15893_c0_g1_i1_len_1061_95223.p2_type_5prime_partial_len_117 | ---NA--- | | |
| TRINITY_DN28783_c0_g1_i1_len_352_172356.p1_type_internal_len_118 | ---NA--- | | |
| TRINITY_DN25915_c0_g1_i1_len_353_43419.p1_type_internal_len_118 | ---NA--- | | |
| TRINITY_DN21903_c0_g1_i3_len_1283_234159.p3_type_5prime_partial_len_117 | ---NA--- | | |
| TRINITY_DN7450_c0_g1_i1_len_441_206274.p1_type_5prime_partial_len_117 | ---NA--- | | |

| | | | |
|---|---|---|---|
| TRINITY_DN8646_c0_g1_i1_len_478_243657.p1_type_5prime_partial_len_117 | ---NA--- | | |
| TRINITY_DN34174_c0_g1_i1_len_353_172337.p1_type_internal_len_118 | ---NA--- | | |
| TRINITY_DN18662_c0_g1_i1_len_888_63855.p1_type_3prime_partial_len_118 | ANC55855.1selenoprotein M | | |
| TRINITY_DN5958_c0_g1_i1_len_351_210683.p1_type_internal_len_118 | ---NA--- | | |
| TRINITY_DN27185_c0_g1_i1_len_3701_187773.p2_type_complete_len_117 | ---NA--- | | |
| TRINITY_DN12602_c0_g1_i1_len_388_41912.p1_type_5prime_partial_len_117 | XP_015518273.1 integrator complex subunit 2 isoform X2 | P:GO:0016180; C:GO:0032039 | |
| TRINITY_DN14953_c0_g1_i1_len_403_41380.p1_type_5prime_partial_len_117 | ---NA--- | | |
| TRINITY_DN35502_c0_g1_i1_len_351_210706.p1_type_internal_len_118 | XP_018013547.1 adenylate kinase isoenzyme 1-like | F:GO:0004017; F:GO:0004550; F:GO:0005524; C:GO:0005737; P:GO:0006165; P:GO:0006172; P:GO:0009142; P:GO:0046033; P:GO:0046034; P:GO:0046940 | EC:2.7.4.4; EC:2.7.4.3; EC:2.7.4.6 |
| TRINITY_DN12280_c0_g1_i1_len_405_72139.p1_type_5prime_partial_len_117 | ---NA--- | | |
| TRINITY_DN14487_c0_g1_i2_len_860_4409.p1_type_5prime_partial_len_117 | ---NA--- | | |
| TRINITY_DN8146_c0_g1_i1_len_352_172395.p1_type_internal_len_118 | ---NA--- | | |
| TRINITY_DN16579_c0_g1_i1_len_354_210491.p1_type_internal_len_118 | ---NA--- | | |
| TRINITY_DN424_c0_g1_i1_len_353_137517.p1_type_internal_len_118 | ---NA--- | | |
| TRINITY_DN29677_c0_g1_i1_len_9793_186750.p1_type_complete_len_2471 | XP_025266074.1CCR4-NOT transcription complex subunit 1 isoform X2 | P:GO:0006417; C:GO:0030015 | |
| TRINITY_DN30235_c0_g1_i1_len_9670_186751.p1_type_complete_len_2463 | XP_023705168.1multiple PDZ domain protein-like isoform X7 | | |

| | | | |
|---|---|---|---|
| TRINITY_DN24922_c0_g1_i4_len_8450_227473.p1_type_complete_len_2434 | PNF34874.1hypothetical protein B7P43_G03117, partial | F:GO:0005096; P:GO:0007165; P:GO:0043547 | |
| TRINITY_DN26114_c0_g1_i1_len_8274_227476.p1_type_5prime_partial_len_2421 | AHX84170.1target of rapamycin | F:GO:0004674; F:GO:0005524; P:GO:0006468; F:GO:0044877 | EC:2.7.11 |
| TRINITY_DN30953_c0_g1_i1_len_8577_186759.p1_type_complete_len_2416 | XP_018020900.1 spectrin alpha chain-like isoform X2 | F:GO:0005509 | |
| TRINITY_DN30458_c0_g1_i2_len_7455_186786.p1_type_5prime_partial_len_2414 | XP_018008782.1 laminin subunit alpha-like | | |
| TRINITY_DN25605_c0_g1_i2_len_8066_227478.p1_type_5prime_partial_len_2387 | XP_021001770.1E3 ubiquitin-protein ligase UBR4 | F:GO:0008270; F:GO:0016874 | |
| TRINITY_DN31098_c0_g1_i1_len_7531_186783.p1_type_complete_len_2375 | XP_018024367.1 pre-mRNA-processing-splicing factor 8 | P:GO:0000398; C:GO:0005681; F:GO:0017070; F:GO:0030623 | |
| TRINITY_DN25978_c1_g1_i2_len_7225_227506.p1_type_3prime_partial_len_2350 | XP_023704416.1helicase domino isoform X6 | | |
| TRINITY_DN30981_c0_g1_i1_len_7377_186789.p1_type_5prime_partial_len_2304 | XP_018025826.1 DNA-dependent protein kinase catalytic subunit-like | F:GO:0000166; F:GO:0003677; F:GO:0004677; F:GO:0005524; C:GO:0005634; P:GO:0006281; P:GO:0006303; P:GO:0006468; F:GO:0016301; P:GO:0016310; F:GO:0016740 | |
| TRINITY_DN24937_c2_g2_i1_len_7075_227511.p1_type_5prime_partial_len_1503 | XP_023704810.1rho guanine nucleotide exchange factor 18 isoform X3 | | |
| TRINITY_DN27659_c0_g1_i2_len_4821_187188.p1_type_3prime_partial_len_1503 | XP_026064555.1zinc finger protein 208-like | | |
| TRINITY_DN24956_c0_g1_i1_len_5071_227766.p1_type_complete_len_1501 | BAM76968.1dual oxidase | F:GO:0004601; F:GO:0005509; P:GO:0006979; C:GO:0016021; F:GO:0016174; F:GO:0020037; P:GO:0050665; P:GO:0055114; P:GO:0098869 | EC:1.6.3.1; EC:1.11.1.7 |
| TRINITY_DN30828_c0_g1_i3_len_6044_186925.p1_type_complete_len_1496 | XP_023723256.1mitogen-activated protein kinase-binding protein 1 isoform X2 | | |

| | | | |
|---|---|---|---|
| 267\|_\|c6295_f2p30_4484_isoform_c6295_full_length_coverage_2_non_full_length_coverage_30_isoform_length_4484_28433 | ---NA--- | | |
| TRINITY_DN24580_c0_g1_i1_len_4964_227793.p1_type_complete_len_1492 | XP_014474042.1 PERQ amino acid-rich with GYF domain-containing protein CG11148 isoform X2 | | |
| TRINITY_DN25481_c0_g1_i1_len_4465_227957.p1_type_internal_len_1488 | ---NA--- | | |
| TRINITY_DN31167_c0_g1_i1_len_4927_187153.p1_type_5prime_partial_len_1487 | XP_013789347.1gastrula zinc finger protein XlCGF57.1-like | | |
| TRINITY_DN25855_c0_g1_i6_len_5214_227744.p1_type_3prime_partial_len_1487 | XP_018025456.1 rho GTPase-activating protein 190-like isoform X3 | | |
| TRINITY_DN26388_c0_g1_i2_len_4989_227786.p1_type_complete_len_1485 | XP_019638879.1 coiled-coil and C2 domain-containing protein 2A-like isoform X2 | | |
| 2495\|_\|c17982_f2p2_2576_isoform_c17982_full_length_coverage_2_non_full_length_coverage_2_isoform_length_2576_13486 | XP_024052402.1histone H4 transcription factor isoform X1 | | |
| 2496\|_\|c18127_f2p7_2576_isoform_c18127_full_length_coverage_2_non_full_length_coverage_7_isoform_length_2576_13593 | AAU12181.1G protein s alpha subunit | F:GO:0003924; F:GO:0005525; P:GO:0007186; F:GO:0031683; F:GO:0046872 | EC:3.6.1.15 |
| 2497\|_\|c12011_f2p0_2576_isoform_c12011_full_length_coverage_2_non_full_length_coverage_0_isoform_length_2576_13032 | XP_018007679.1 AP-4 complex subunit beta-1-like | P:GO:0006886; P:GO:0015031; C:GO:0016020; P:GO:0016192; C:GO:0030117; F:GO:0030276 | |
| 2499\|_\|c18124_f2p1_2575_isoform_c18124_full_length_coverage_2_non_full_length_coverage_1_isoform_length_2575_13590 | XP_025836948.1nitric oxide-associated protein 1 | F:GO:0005525 | |
| TRINITY_DN21422_c0_g1_i1_len_2889_91221.p1_type_complete_len_858 | PNF43621.1ATP-binding cassette sub-family B member 6, mitochondrial | F:GO:0005524; C:GO:0016021; F:GO:0042626; P:GO:0055085 | EC:3.6.1.3; EC:3.6.1.15 |
| TRINITY_DN28374_c0_g1_i2_len_2793_188865.p1_type_complete_len_858 | XP_025089141.1small G protein signaling modulator 1-like isoform X8 | F:GO:0005096; P:GO:0043547 | |
| 2502\|_\|c2364_f5p3_2574_isoform_c2364_full_length_coverage_5_non_full_length_coverage_3_isoform_length_2574_20882 | XP_018020339.1 protein flightless-1-like | P:GO:0030036; P:GO:0051014; F:GO:0051015 | |

| | | | |
|---|---|---|---|
| 2503\|_\|c4521_f2p2_2574_isoform_c4521_full_length_coverage_2_non_full_length_coverage_2_isoform_length_2574_12389 | PNF39467.1Tubulin gamma-2 chain | C:GO:0000930; F:GO:0003924; F:GO:0005525; C:GO:0005874; P:GO:0007020; P:GO:0031122 | EC:3.6.1.15 |
| 2504\|_\|c11151_f7p0_2574_isoform_c11151_full_length_coverage_7_non_full_length_coverage_0_isoform_length_2574_22029 | XP_026470945.1probable protein phosphatase CG10417 | | |
| TRINITY_DN25908_c0_g2_i1_len_3721_228334.p1_type_complete_len_858 | XP_018015554.1 zinc transporter foi-like | | |
| TRINITY_DN12742_c0_g1_i1_len_353_43405.p2_type_internal_len_118 | ---NA--- | | |
| TRINITY_DN22816_c0_g1_i4_len_1705_157010.p1_type_complete_len_117 | ---NA--- | | |
| TRINITY_DN34384_c0_g1_i1_len_351_210695.p1_type_internal_len_118 | KZS10881.1Sulfotransferase 4A1 | F:GO:0008146; F:GO:0016740 | |
| TRINITY_DN24438_c0_g1_i1_len_352_172385.p1_type_internal_len_118 | ---NA--- | | |
| TRINITY_DN7360_c0_g1_i1_len_352_172386.p1_type_internal_len_118 | ---NA--- | | |
| TRINITY_DN8457_c0_g1_i1_len_354_210489.p1_type_internal_len_118 | ---NA--- | | |
| TRINITY_DN22946_c0_g1_i1_len_2631_154781.p3_type_5prime_partial_len_117 | ---NA--- | | |
| TRINITY_DN31299_c0_g1_i1_len_354_210493.p1_type_internal_len_118 | ---NA--- | | |
| TRINITY_DN3847_c0_g1_i1_len_355_43315.p1_type_internal_len_118 | ---NA--- | | |
| TRINITY_DN11956_c0_g1_i1_len_352_172371.p1_type_internal_len_118 | ---NA--- | | |
| TRINITY_DN37324_c0_g1_i1_len_351_210698.p2_type_internal_len_118 | XP_018016102.1 protein bric-a-brac 2-like isoform X2 | | |
| TRINITY_DN31105_c0_g1_i2_len_4501_187314.p4_type_5prime_partial_len_117 | ---NA--- | | |

| | | | |
|---|---|---|---|
| TRINITY_DN428_c0_g1_i1_len_354_210455.p1_type_internal_len_118 | PSN47158.1Histone deacetylase 4 | C:GO:0000118; F:GO:0032041; F:GO:0046872; P:GO:0070932 | EC:3.5.1.98 |
| TRINITY_DN7558_c0_g1_i1_len_470_243915.p1_type_5prime_partial_len_117 | XP_014783424.1 piggyBac transposable element-derived protein 4-like | | |
| TRINITY_DN21635_c0_g1_i9_len_3242_154096.p4_type_3prime_partial_len_118 | ---NA--- | | |
| TRINITY_DN4632_c0_g1_i1_len_352_172372.p1_type_internal_len_118 | XP_018017758.1 UDP-N-acetylglucosamine/UDP-glucose/GDP-mannose transporter-like isoform X2 | | |
| TRINITY_DN11757_c0_g1_i1_len_352_210643.p1_type_internal_len_118 | ODN03888.1DNA polymerase subunit gamma-1 | P:GO:0034645 | |
| TRINITY_DN11804_c0_g1_i2_len_592_7124.p1_type_complete_len_117 | ---NA--- | | |
| TRINITY_DN18250_c0_g2_i1_len_987_298876.p1_type_complete_len_117 | ---NA--- | | |
| TRINITY_DN30382_c2_g1_i1_len_651_200789.p1_type_complete_len_117 | ---NA--- | | |
| TRINITY_DN6123_c0_g1_i1_len_402_41404.p1_type_complete_len_117 | ---NA--- | | |
| TRINITY_DN2552_c0_g1_i1_len_353_172303.p1_type_internal_len_118 | AYH52220.1putative peptidyl-alpha-hydroxyglycine-alpha-amidating lyase I | F:GO:0003824 | |
| TRINITY_DN24396_c0_g1_i1_len_561_302986.p1_type_complete_len_117 | ---NA--- | | |
| TRINITY_DN3540_c0_g1_i2_len_353_137538.p2_type_internal_len_118 | ---NA--- | | |
| TRINITY_DN40373_c0_g1_i1_len_351_210714.p2_type_internal_len_118 | ---NA--- | | |
| TRINITY_DN31449_c0_g1_i1_len_353_137542.p1_type_internal_len_118 | ---NA--- | | |
| TRINITY_DN18547_c0_g1_i1_len_734_300768.p2_type_5prime_partial_len_117 | ---NA--- | | |
| TRINITY_DN13079_c0_g1_i1_len_354_172289.p1_type_internal_len_118 | CDI59403.1Histone 1 | C:GO:0000786; F:GO:0003677; C:GO:0005634; P:GO:0006334 | |

| | | | |
|---|---|---|---|
| TRINITY_DN13587_c0_g1_i1_len_355_280854.p1_type_internal_len_118 | ---NA--- | | |
| TRINITY_DN15828_c0_g1_i1_len_351_13845.p1_type_internal_len_118 | ---NA--- | | |
| TRINITY_DN16194_c0_g1_i1_len_417_278390.p1_type_5prime_partial_len_117 | ---NA--- | | |
| TRINITY_DN35811_c0_g1_i1_len_352_210628.p1_type_internal_len_118 | ---NA--- | | |
| TRINITY_DN30231_c0_g1_i1_len_351_248741.p1_type_internal_len_118 | XP_014611009.1 protein furry isoform X4 | | |
| TRINITY_DN34003_c0_g1_i1_len_354_210457.p1_type_internal_len_118 | ADM88556.1fatty acid synthase | F:GO:0004312; P:GO:0009058; F:GO:0016491; F:GO:0016788; F:GO:0031177; P:GO:0055114 | EC:2.3.1.85 |
| TRINITY_DN38989_c0_g1_i1_len_352_210634.p1_type_internal_len_118 | ---NA--- | | |
| TRINITY_DN40301_c0_g1_i1_len_352_210636.p1_type_internal_len_118 | XP_022900363.1serine proteinase stubble isoform X2 | F:GO:0004252; P:GO:0006508 | EC:3.4.21 |
| TRINITY_DN20174_c0_g1_i1_len_1811_156654.p1_type_complete_len_117 | ---NA--- | | |
| TRINITY_DN30773_c0_g1_i1_len_4428_187347.p6_type_5prime_partial_len_117 | ---NA--- | | |
| TRINITY_DN19640_c0_g1_i1_len_1167_195187.p1_type_complete_len_117 | ---NA--- | | |
| TRINITY_DN1043_c0_g1_i1_len_354_210456.p1_type_internal_len_118 | AAM47019.1dopamine beta hydroxylase | F:GO:0004497; C:GO:0016020 | |
| TRINITY_DN7195_c0_g1_i1_len_841_127684.p1_type_5prime_partial_len_117 | ---NA--- | | |
| TRINITY_DN19114_c0_g1_i1_len_596_302418.p1_type_complete_len_117 | ---NA--- | | |
| TRINITY_DN7092_c0_g1_i2_len_452_39837.p1_type_3prime_partial_len_118 | ---NA--- | | |
| TRINITY_DN11067_c0_g1_i1_len_359_43149.p1_type_3prime_partial_len_118 | ---NA--- | | |

| | | | |
|---|---|---|---|
| TRINITY_DN31804_c0_g1_i1_len_353_172315.p1_type_internal_len_118 | XP_018023391.1 ADP-ribosylation factor-like | F:GO:0005525 | |
| TRINITY_DN14769_c0_g1_i1_len_419_207162.p1_type_5prime_partial_len_117 | ---NA--- | | |
| TRINITY_DN25447_c0_g1_i1_len_353_137530.p1_type_internal_len_118 | XP_012263808.1ubiquitin-protein ligase E3B isoform X2 | | |
| TRINITY_DN27483_c0_g1_i1_len_359_248299.p1_type_5prime_partial_len_117 | ---NA--- | | |
| TRINITY_DN24024_c1_g1_i2_len_1455_233242.p3_type_5prime_partial_len_117 | ---NA--- | | |
| TRINITY_DN18771_c0_g1_i1_len_719_65463.p1_type_5prime_partial_len_117 | ---NA--- | | |
| TRINITY_DN4396_c0_g1_i2_len_591_274274.p1_type_5prime_partial_len_117 | ---NA--- | | |
| TRINITY_DN38011_c0_g1_i1_len_351_248752.p1_type_internal_len_118 | ---NA--- | | |
| TRINITY_DN22870_c0_g1_i1_len_386_104921.p1_type_5prime_partial_len_117 | ---NA--- | | |
| TRINITY_DN13559_c0_g1_i1_len_453_168352.p1_type_5prime_partial_len_117 | ---NA--- | | |
| TRINITY_DN5843_c0_g1_i1_len_353_106489.p1_type_internal_len_118 | ---NA--- | | |
| TRINITY_DN3070_c0_g1_i1_len_352_106519.p1_type_internal_len_118 | XP_018026245.1 solute carrier family 35 member F2-like | C:GO:0016020 | |
| TRINITY_DN29310_c0_g1_i1_len_354_248570.p1_type_internal_len_118 | AJR21491.1RNA helicase | F:GO:0003723; F:GO:0032575; P:GO:0035194 | EC:3.6.1.3; EC:3.6.1.15 |
| TRINITY_DN5170_c0_g1_i1_len_351_106562.p1_type_internal_len_118 | ---NA--- | | |
| TRINITY_DN19836_c1_g1_i4_len_1025_298680.p2_type_5prime_partial_len_117 | XP_023212524.1gastrula zinc finger protein XlCGF49.1-like | | |
| TRINITY_DN20717_c0_g1_i1_len_616_201461.p1_type_5prime_partial_len_117 | ---NA--- | | |

| | | | |
|---|---|---|---|
| TRINITY_DN28752_c0_g1_i1_len_354_248562.p1_type_internal_len_118 | ---NA--- | | |
| TRINITY_DN40206_c0_g1_i1_len_353_210580.p1_type_internal_len_118 | ---NA--- | | |
| TRINITY_DN13976_c0_g1_i1_len_353_210576.p1_type_internal_len_118 | ---NA--- | | |
| TRINITY_DN31567_c0_g1_i1_len_352_248690.p2_type_internal_len_118 | ---NA--- | | |
| TRINITY_DN29643_c0_g1_i1_len_354_248574.p1_type_internal_len_118 | ---NA--- | | |
| TRINITY_DN35250_c0_g1_i1_len_353_210542.p1_type_internal_len_118 | ---NA--- | | |
| TRINITY_DN29042_c0_g1_i1_len_354_106427.p2_type_internal_len_118 | ---NA--- | | |
| TRINITY_DN2402_c0_g1_i1_len_352_13791.p1_type_internal_len_118 | AFQ62792.1inositol-requiring enzyme-1 | F:GO:0004540; F:GO:0004672; F:GO:0005524; P:GO:0006397; P:GO:0006468; C:GO:0016021; P:GO:0090501 | |
| TRINITY_DN31086_c0_g1_i1_len_354_106419.p1_type_internal_len_118 | ---NA--- | | |
| TRINITY_DN6326_c0_g1_i1_len_354_309018.p1_type_internal_len_118 | ---NA--- | | |
| TRINITY_DN137_c0_g1_i1_len_579_202224.p1_type_5prime_partial_len_117 | ---NA--- | | |
| TRINITY_DN16658_c0_g1_i1_len_1148_159649.p1_type_complete_len_117 | ---NA--- | | |
| TRINITY_DN35898_c0_g1_i1_len_354_210472.p1_type_internal_len_118 | XP_018007939.1PREDICTED: uncharacterized protein LOC108665675 | | |
| TRINITY_DN19620_c0_g1_i3_len_996_3621.p1_type_complete_len_117 | ---NA--- | | |
| TRINITY_DN27724_c1_g1_i1_len_1487_193176.p2_type_complete_len_117 | ---NA--- | | |

| | | | |
|---|---|---|---|
| TRINITY_DN35724_c0_g1_i1_len_353_210536.p1_type_internal_len_118 | ---NA--- | | |
| TRINITY_DN503_c0_g1_i1_len_353_309063.p1_type_internal_len_118 | ---NA--- | | |
| TRINITY_DN37956_c0_g1_i1_len_353_210590.p1_type_internal_len_118 | ---NA--- | | |
| TRINITY_DN18773_c0_g1_i1_len_351_210652.p1_type_internal_len_118 | ---NA--- | | |
| TRINITY_DN7837_c0_g1_i1_len_353_210531.p1_type_internal_len_118 | ---NA--- | | |
| TRINITY_DN7968_c0_g1_i1_len_353_210597.p1_type_internal_len_118 | ---NA--- | | |
| TRINITY_DN32310_c0_g1_i1_len_643_240073.p2_type_3prime_partial_len_118 | ---NA--- | | |
| TRINITY_DN12151_c0_g1_i1_len_353_309041.p1_type_internal_len_118 | ---NA--- | | |
| TRINITY_DN5113_c0_g1_i2_len_352_74672.p1_type_5prime_partial_len_117 | XP_023710828.1transmembrane protein 65 isoform X3 | | |
| TRINITY_DN18469_c0_g1_i1_len_1627_60266.p3_type_complete_len_117 | ---NA--- | | |
| TRINITY_DN31513_c0_g1_i1_len_353_210540.p1_type_internal_len_118 | ---NA--- | | |
| TRINITY_DN7364_c0_g1_i2_len_352_248697.p1_type_internal_len_118 | XP_023710354.1protein SON-like isoform X1 | F:GO:0003676; F:GO:0003723; P:GO:0043484; P:GO:0051726 | |
| TRINITY_DN6721_c0_g1_i1_len_484_167452.p1_type_5prime_partial_len_117 | ---NA--- | | |
| TRINITY_DN31328_c0_g1_i1_len_352_248699.p1_type_internal_len_118 | ---NA--- | | |
| TRINITY_DN794_c0_g1_i1_len_646_240022.p1_type_3prime_partial_len_118 | ---NA--- | | |
| TRINITY_DN41877_c0_g1_i1_len_353_210538.p1_type_internal_len_118 | ---NA--- | | |

<table>
<tr><td>TRINITY_DN19545_c0_g1_i3_len_1871_59621.p4_type_5prime_partial_len_117</td><td>---NA---</td><td></td><td></td></tr>
<tr><td>TRINITY_DN34204_c0_g1_i1_len_353_248650.p1_type_internal_len_118</td><td>XP_015905250.1integrin alpha-8-like</td><td></td><td></td></tr>
<tr><td>TRINITY_DN23581_c0_g1_i2_len_353_210551.p1_type_internal_len_118</td><td>---NA---</td><td></td><td></td></tr>
<tr><td>TRINITY_DN16300_c0_g1_i2_len_352_74646.p1_type_internal_len_118</td><td>---NA---</td><td></td><td></td></tr>
<tr><td>TRINITY_DN31716_c0_g1_i1_len_434_168955.p1_type_3prime_partial_len_118</td><td>---NA---</td><td></td><td></td></tr>
<tr><td>TRINITY_DN8215_c0_g1_i1_len_352_13817.p1_type_internal_len_118</td><td>---NA---</td><td></td><td></td></tr>
<tr><td>TRINITY_DN21386_c0_g1_i1_len_1843_231597.p2_type_complete_len_117</td><td>---NA---</td><td></td><td></td></tr>
<tr><td>TRINITY_DN4494_c0_g1_i1_len_353_248656.p1_type_3prime_partial_len_118</td><td>XP_023025538.1arf-GAP with coiled-coil, ANK repeat and PH domain-containing protein 2</td><td></td><td></td></tr>
<tr><td>TRINITY_DN7946_c0_g1_i1_len_353_248643.p1_type_3prime_partial_len_118</td><td>AHH32890.1integrin beta 1</td><td>P:GO:0007155; P:GO:0007229; C:GO:0016021</td><td></td></tr>
<tr><td>2507|_|c18114_f2p1_2574_isoform_c18114_full_length_coverage_2_non_full_length_coverage_1_isoform_length_2574_13584</td><td>XP_026492897.1uncharacterized protein LOC113398387</td><td></td><td></td></tr>
<tr><td>2509|_|c10420_f2p9_2572_isoform_c10420_full_length_coverage_2_non_full_length_coverage_9_isoform_length_2572_21956</td><td>XP_018012515.1 longitudinals lacking protein, isoforms A/B/D/L-like isoform X2</td><td>F:GO:0003676</td><td></td></tr>
<tr><td>2510|_|c18026_f2p0_2572_isoform_c18026_full_length_coverage_2_non_full_length_coverage_0_isoform_length_2572_22641</td><td>AAB03106.1cytochrome P450</td><td>F:GO:0016491; C:GO:0043227</td><td></td></tr>
<tr><td>2511|_|c9761_f2p2_2571_isoform_c9761_full_length_coverage_2_non_full_length_coverage_2_isoform_length_2571_12831</td><td>XP_018579916.1protein pelota</td><td>F:GO:0004519; C:GO:0005634; C:GO:0005737; F:GO:0046872; P:GO:0070481; P:GO:0070966; P:GO:0071025; P:GO:0090305</td><td></td></tr>
</table>

| | | | |
|---|---|---|---|
| 2512\|_\|c2029_f6p2_2571_isoform_c2029_full_length_coverage_6_non_full_length_coverage_2_isoform_length_2571_20653 | PNF27664.1Dymeclin | | |
| TRINITY_DN24406_c0_g1_i1_len_2573_189282.p1_type_internal_len_858 | XP_011433250.1 ubiquitin carboxyl-terminal hydrolase 20 | | |
| 2514\|_\|c2320_f3p1_2571_isoform_c2320_full_length_coverage_3_non_full_length_coverage_1_isoform_length_2571_16321 | ---NA--- | | |
| 2516\|_\|c2362_f5p3_2571_isoform_c2362_full_length_coverage_5_non_full_length_coverage_3_isoform_length_2571_11807 | XP_018018894.1 serine/threonine-protein kinase PLK1-like | | |
| 2517\|_\|c9498_f3p1_2570_isoform_c9498_full_length_coverage_3_non_full_length_coverage_1_isoform_length_2570_17287 | XP_004652711.1 cell division cycle-associated 7-like protein | P:GO:0006355; P:GO:0008284; P:GO:0051301 | |
| 2519\|_\|c18295_f2p0_2570_isoform_c18295_full_length_coverage_2_non_full_length_coverage_0_isoform_length_2570_18088 | ---NA--- | | |
| TRINITY_DN28023_c0_g1_i1_len_2352_189814.p2_type_complete_len_117 | ---NA--- | | |
| TRINITY_DN4901_c0_g1_i1_len_354_280899.p1_type_internal_len_118 | ---NA--- | | |
| TRINITY_DN4396_c0_g1_i1_len_364_105920.p1_type_5prime_partial_len_117 | ---NA--- | | |
| TRINITY_DN36885_c0_g1_i1_len_353_248665.p1_type_internal_len_118 | ---NA--- | | |
| TRINITY_DN28557_c0_g1_i1_len_355_308927.p1_type_internal_len_118 | XP_018027727.1PREDICTED: uncharacterized protein LOC108682971 | | |
| TRINITY_DN24928_c0_g1_i1_len_4610_227906.p2_type_5prime_partial_len_117 | ---NA--- | | |
| TRINITY_DN6247_c0_g1_i1_len_353_248663.p1_type_internal_len_118 | ---NA--- | | |
| TRINITY_DN18027_c0_g1_i2_len_1374_31871.p2_type_complete_len_117 | ---NA--- | | |

| | | | |
|---|---|---|---|
| TRINITY_DN16672_c0_g1_i1_len_537_100504.p1_type_3prime_partial_len_118 | ---NA--- | | |
| TRINITY_DN28985_c0_g1_i1_len_352_248667.p1_type_internal_len_118 | XP_018010735.1 multiple epidermal growth factor-like domains protein 8 | | |
| TRINITY_DN27229_c0_g1_i1_len_353_248612.p1_type_internal_len_118 | ---NA--- | | |
| TRINITY_DN21879_c0_g1_i1_len_850_198035.p1_type_complete_len_117 | ---NA--- | | |
| TRINITY_DN40259_c0_g1_i1_len_389_208560.p1_type_5prime_partial_len_117 | ---NA--- | | |
| TRINITY_DN23004_c0_g1_i1_len_1487_193169.p2_type_5prime_partial_len_117 | ---NA--- | | |
| TRINITY_DN27409_c0_g1_i1_len_2339_189847.p2_type_5prime_partial_len_117 | ---NA--- | | |
| TRINITY_DN5335_c0_g1_i1_len_393_170521.p1_type_5prime_partial_len_117 | ---NA--- | | |
| TRINITY_DN12161_c0_g1_i1_len_353_248609.p1_type_internal_len_118 | EUB62981.1Tubulin beta chain | | |
| TRINITY_DN15006_c0_g1_i1_len_899_197510.p2_type_5prime_partial_len_117 | ---NA--- | | |
| TRINITY_DN7515_c0_g1_i1_len_353_210570.p1_type_internal_len_118 | ---NA--- | | |
| TRINITY_DN11269_c1_g1_i2_len_353_248620.p1_type_internal_len_118 | ---NA--- | | |
| TRINITY_DN12482_c0_g1_i1_len_352_248686.p1_type_internal_len_118 | ---NA--- | | |
| TRINITY_DN27827_c0_g1_i1_len_352_248688.p1_type_internal_len_118 | ---NA--- | | |
| TRINITY_DN21726_c2_g1_i2_len_1338_93882.p2_type_complete_len_117 | ---NA--- | | |
| TRINITY_DN12107_c0_g1_i1_len_569_202425.p1_type_complete_len_117 | ---NA--- | | |

| | | | |
|---|---|---|---|
| TRINITY_DN2205_c0_g1_i1_len_353_248625.p1_type_internal_len_118 | ---NA--- | | |
| TRINITY_DN3271_c0_g1_i1_len_492_167249.p1_type_5prime_partial_len_117 | ---NA--- | | |
| TRINITY_DN33664_c0_g1_i1_len_353_248615.p1_type_internal_len_118 | ---NA--- | | |
| TRINITY_DN8370_c0_g1_i1_len_351_137634.p1_type_internal_len_118 | XP_018010863.1 FGGY carbohydrate kinase domain-containing protein-like | | |
| TRINITY_DN18449_c2_g1_i1_len_354_308987.p1_type_internal_len_118 | ---NA--- | | |
| TRINITY_DN4424_c0_g1_i1_len_353_248618.p1_type_internal_len_118 | ---NA--- | | |
| TRINITY_DN24720_c0_g1_i1_len_353_280925.p1_type_internal_len_118 | ---NA--- | | |
| TRINITY_DN28583_c0_g1_i1_len_358_106197.p1_type_5prime_partial_len_117 | ---NA--- | | |
| TRINITY_DN30665_c0_g1_i1_len_406_246166.p1_type_3prime_partial_len_118 | ---NA--- | | |
| TRINITY_DN21902_c0_g1_i4_len_993_63165.p2_type_5prime_partial_len_117 | ---NA--- | | |
| TRINITY_DN9780_c0_g1_i1_len_351_210664.p2_type_internal_len_118 | ---NA--- | | |
| TRINITY_DN32359_c0_g1_i1_len_355_210428.p2_type_internal_len_118 | XP_018917412.1 gamma-butyrobetaine dioxygenase isoform X2 | | |
| TRINITY_DN21212_c0_g1_i1_len_2167_155665.p1_type_complete_len_117 | ---NA--- | | |
| TRINITY_DN7542_c0_g1_i1_len_785_198800.p1_type_complete_len_117 | ---NA--- | | |
| TRINITY_DN40444_c0_g1_i1_len_352_210602.p1_type_internal_len_118 | ---NA--- | | |
| TRINITY_DN8358_c0_g1_i2_len_353_210529.p1_type_internal_len_118 | ---NA--- | | |

| | | | |
|---|---|---|---|
| TRINITY_DN18245_c0_g1_i2_len_351_172436.p1_type_internal_len_118 | ---NA--- | | |
| TRINITY_DN8904_c0_g1_i1_len_352_280969.p1_type_internal_len_118 | ---NA--- | | |
| TRINITY_DN29185_c0_g2_i1_len_829_198270.p1_type_5prime_partial_len_117 | ---NA--- | | |
| TRINITY_DN1849_c0_g1_i1_len_433_134172.p1_type_5prime_partial_len_117 | ---NA--- | | |
| TRINITY_DN16191_c0_g1_i1_len_352_137593.p1_type_internal_len_118 | ---NA--- | | |
| TRINITY_DN30647_c0_g1_i4_len_3012_188528.p2_type_complete_len_117 | ---NA--- | | |
| TRINITY_DN6683_c0_g1_i1_len_353_74567.p1_type_internal_len_118 | ---NA--- | | |
| TRINITY_DN30634_c0_g1_i1_len_351_74697.p1_type_internal_len_118 | ---NA--- | | |
| TRINITY_DN15214_c0_g1_i2_len_354_210519.p1_type_internal_len_118 | ---NA--- | | |
| TRINITY_DN16381_c0_g1_i1_len_561_241548.p1_type_complete_len_117 | ---NA--- | | |
| TRINITY_DN12222_c0_g1_i1_len_352_210617.p1_type_3prime_partial_len_118 | AEI88122.1hypothetical protein, partial | F:GO:0003676 | |
| TRINITY_DN31251_c0_g1_i2_len_931_197169.p2_type_5prime_partial_len_117 | ---NA--- | | |
| TRINITY_DN39866_c0_g1_i1_len_355_210431.p1_type_3prime_partial_len_118 | ---NA--- | | |
| TRINITY_DN32072_c0_g1_i1_len_352_280991.p2_type_internal_len_118 | ---NA--- | | |
| TRINITY_DN41816_c0_g1_i1_len_352_210606.p1_type_internal_len_118 | XP_018015557.1 palmitoyltransferase Hip14-like | F:GO:0015095; C:GO:0016021; F:GO:0019706; P:GO:1903830 | EC:2.3.1.225 |
| TRINITY_DN13302_c0_g1_i1_len_351_74688.p1_type_internal_len_118 | ---NA--- | | |

| | | | |
|---|---|---|---|
| TRINITY_DN19234_c0_g1_i1_len_1375_268237.p1_type_3prime_partial_len_118 | ---NA--- | | |
| TRINITY_DN280_c0_g1_i1_len_353_280950.p1_type_internal_len_118 | ---NA--- | | |
| TRINITY_DN35183_c0_g1_i1_len_355_210410.p1_type_internal_len_118 | ---NA--- | | |
| TRINITY_DN23634_c0_g1_i1_len_351_309127.p1_type_internal_len_118 | ---NA--- | | |
| TRINITY_DN3964_c0_g1_i1_len_352_309104.p1_type_internal_len_118 | ---NA--- | | |
| TRINITY_DN16084_c0_g1_i3_len_940_95997.p1_type_5prime_partial_len_117 | ---NA--- | | |
| TRINITY_DN30688_c0_g1_i1_len_351_248705.p1_type_internal_len_118 | ---NA--- | | |
| TRINITY_DN34298_c0_g1_i1_len_353_74602.p1_type_internal_len_118 | ---NA--- | | |
| TRINITY_DN22627_c0_g1_i4_len_1234_2502.p1_type_5prime_partial_len_117 | ---NA--- | | |
| TRINITY_DN26306_c0_g1_i1_len_6531_227547.p3_type_complete_len_117 | ---NA--- | | |
| TRINITY_DN39926_c0_g1_i1_len_353_210598.p1_type_internal_len_118 | ---NA--- | | |
| TRINITY_DN35468_c0_g1_i1_len_352_74678.p1_type_internal_len_118 | ---NA--- | | |
| TRINITY_DN39296_c0_g1_i1_len_386_208729.p1_type_5prime_partial_len_117 | ---NA--- | | |
| TRINITY_DN41349_c0_g1_i1_len_353_210600.p1_type_internal_len_118 | XP_018027333.1 low-density lipoprotein receptor-related protein 2-like | F:GO:0005509; C:GO:0016021 | |
| TRINITY_DN34458_c0_g1_i1_len_391_208462.p1_type_5prime_partial_len_117 | ---NA--- | | |
| TRINITY_DN39151_c0_g1_i1_len_355_210411.p2_type_internal_len_118 | ---NA--- | | |

| | | | |
|---|---|---|---|
| TRINITY_DN3525_c0_g1_i1_len_351_210651.p1_type_5prime_partial_len_117 | ---NA--- | | |
| TRINITY_DN17587_c0_g1_i1_len_353_210599.p1_type_internal_len_118 | ---NA--- | | |
| TRINITY_DN18893_c0_g2_i1_len_435_206509.p2_type_5prime_partial_len_117 | ---NA--- | | |
| TRINITY_DN30760_c0_g1_i1_len_355_248522.p1_type_internal_len_118 | ---NA--- | | |
| TRINITY_DN35089_c0_g1_i1_len_350_248768.p1_type_internal_len_117 | XP_023318213.1neurobeachin isoform X8 | | |
| TRINITY_DN18998_c0_g1_i1_len_821_237728.p1_type_complete_len_116 | ---NA--- | | |
| TRINITY_DN12794_c0_g1_i2_len_350_137687.p3_type_internal_len_117 | ---NA--- | | |
| TRINITY_DN22293_c0_g1_i2_len_1465_157962.p4_type_complete_len_116 | ---NA--- | | |
| TRINITY_DN32197_c0_g1_i1_len_351_172458.p1_type_internal_len_117 | ---NA--- | | |
| TRINITY_DN32541_c0_g1_i1_len_350_248771.p2_type_internal_len_117 | ---NA--- | | |
| TRINITY_DN22039_c0_g1_i1_len_1363_233708.p1_type_complete_len_116 | ---NA--- | | |
| TRINITY_DN22760_c0_g1_i2_len_2244_230396.p2_type_complete_len_116 | ---NA--- | | |
| TRINITY_DN23329_c0_g4_i1_len_750_128448.p1_type_5prime_partial_len_116 | ---NA--- | | |
| TRINITY_DN22631_c0_g3_i3_len_1276_2356.p2_type_complete_len_116 | ---NA--- | | |
| TRINITY_DN19907_c0_g1_i2_len_1319_61405.p1_type_complete_len_116 | ---NA--- | | |
| TRINITY_DN30769_c0_g1_i1_len_348_13977.p1_type_internal_len_117 | ---NA--- | | |

| | | | |
|---|---|---|---|
| TRINITY_DN6333_c0_g1_i2_len_348_281193.p1_type_internal_len_117 | ---NA--- | | |
| TRINITY_DN22982_c0_g1_i2_len_1471_157925.p3_type_5prime_partial_len_116 | ---NA--- | | |
| TRINITY_DN30467_c0_g1_i1_len_351_137662.p1_type_internal_len_117 | ---NA--- | | |
| TRINITY_DN17321_c0_g1_i1_len_351_210653.p1_type_internal_len_117 | ---NA--- | | |
| TRINITY_DN22761_c0_g1_i1_len_1476_193226.p2_type_3prime_partial_len_117 | ---NA--- | | |
| TRINITY_DN11753_c0_g1_i1_len_350_137717.p1_type_internal_len_117 | ---NA--- | | |
| TRINITY_DN2885_c0_g1_i1_len_351_172438.p1_type_internal_len_117 | ---NA--- | | |
| TRINITY_DN25514_c0_g1_i1_len_350_137712.p1_type_internal_len_117 | ---NA--- | | |
| TRINITY_DN1826_c0_g1_i1_len_616_36731.p1_type_complete_len_116 | ---NA--- | | |
| TRINITY_DN19463_c0_g1_i1_len_758_128373.p1_type_complete_len_116 | ---NA--- | | |
| TRINITY_DN31476_c0_g1_i1_len_421_207090.p1_type_complete_len_116 | XP_018010166.1 cuticle protein 7-like | F:GO:0042302 | |
| TRINITY_DN15228_c0_g1_i1_len_502_242998.p1_type_5prime_partial_len_116 | ---NA--- | | |
| 2520\|_\|c6934_f3p1_2569_isoform_c6934_full_length_coverage_3_non_full_length_coverage_1_isoform_length_2569_21643 | ---NA--- | | |
| 2521\|_\|c1786_f6p4_2569_isoform_c1786_full_length_coverage_6_non_full_length_coverage_4_isoform_length_2569_20540 | XP_018014310.1PREDICTED: uncharacterized protein LOC108671307 | | |
| TRINITY_DN31157_c1_g1_i1_len_7828_186774.p1_type_complete_len_856 | XP_013141232.1 constitutive coactivator of PPAR-gamma-like protein 1 isoform X4 | C:GO:0005634 | |

<table>
<tr><td>2526|_|c1408_f10p5_2567_isoform_c1408_full_length_coverage_10_non_full_length_coverage_5_isoform_length_2567_11380</td><td>PNF29235.1Prolyl endopeptidase</td><td>F:GO:0004252; P:GO:0006508; F:GO:0070008</td><td>EC:3.4.21</td></tr>
<tr><td>2527|_|c10543_f4p8_2567_isoform_c10543_full_length_coverage_4_non_full_length_coverage_8_isoform_length_2567_21970</td><td>XP_023350276.1aurora kinase A</td><td></td><td></td></tr>
<tr><td>2529|_|c2595_f3p0_2567_isoform_c2595_full_length_coverage_3_non_full_length_coverage_0_isoform_length_2567_11999</td><td>---NA---</td><td></td><td></td></tr>
<tr><td>2530|_|c17921_f3p0_2567_isoform_c17921_full_length_coverage_3_non_full_length_coverage_0_isoform_length_2567_13461</td><td>PSN55948.1Golgi phosphoprotein 3 sauron</td><td>F:GO:0070273</td><td></td></tr>
<tr><td>TRINITY_DN26087_c0_g1_i4_len_2975_188580.p1_type_complete_len_855</td><td>XP_018007760.1 trafficking kinesin-binding protein 1-like isoform X1</td><td></td><td></td></tr>
<tr><td>TRINITY_DN29746_c0_g1_i1_len_3067_188425.p1_type_5prime_partial_len_855</td><td>PNF15969.1hypothetical protein B7P43_G07491</td><td>C:GO:0016020; C:GO:0016021</td><td></td></tr>
<tr><td>2535|_|c17882_f4p1_2564_isoform_c17882_full_length_coverage_4_non_full_length_coverage_1_isoform_length_2564_17836</td><td>XP_001603169.1 beta-1,4-galactosyltransferase 1</td><td>P:GO:0005975; F:GO:0016757</td><td></td></tr>
<tr><td>TRINITY_DN27741_c0_g1_i1_len_350_248767.p1_type_internal_len_117</td><td>---NA---</td><td></td><td></td></tr>
<tr><td>TRINITY_DN32518_c0_g1_i1_len_351_210657.p1_type_internal_len_117</td><td>---NA---</td><td></td><td></td></tr>
<tr><td>TRINITY_DN32671_c0_g1_i1_len_683_98272.p1_type_complete_len_116</td><td>---NA---</td><td></td><td></td></tr>
<tr><td>TRINITY_DN5020_c0_g1_i1_len_969_126754.p1_type_complete_len_116</td><td>---NA---</td><td></td><td></td></tr>
<tr><td>TRINITY_DN25224_c0_g1_i1_len_1185_195064.p1_type_5prime_partial_len_116</td><td>---NA---</td><td></td><td></td></tr>
<tr><td>TRINITY_DN16622_c0_g1_i1_len_522_203602.p1_type_5prime_partial_len_116</td><td>XP_018014830.1 ethanolaminephosphotransferase 1-like</td><td>P:GO:0008654; C:GO:0016021; F:GO:0016780</td><td></td></tr>
<tr><td>TRINITY_DN6222_c0_g1_i1_len_351_248715.p1_type_internal_len_117</td><td>XP_018023492.1 lateral signaling target protein 2 homolog</td><td></td><td></td></tr>
</table>

| | | | |
|---|---|---|---|
| TRINITY_DN3903_c0_g1_i1_len_356_248471.p1_type_3prime_partial_len_117 | ---NA--- | | |
| TRINITY_DN3653_c0_g1_i1_len_363_171838.p1_type_5prime_partial_len_116 | ---NA--- | | |
| TRINITY_DN32124_c0_g1_i1_len_404_306870.p1_type_5prime_partial_len_116 | ---NA--- | | |
| TRINITY_DN27184_c0_g1_i1_len_351_248708.p1_type_internal_len_117 | XP_013780543.1probable phosphorylase b kinase regulatory subunit alpha | | |
| TRINITY_DN23911_c0_g1_i1_len_2094_190570.p2_type_5prime_partial_len_116 | ---NA--- | | |
| TRINITY_DN13690_c0_g1_i1_len_429_277975.p1_type_3prime_partial_len_117 | ---NA--- | | |
| TRINITY_DN21684_c0_g1_i1_len_688_200186.p1_type_complete_len_116 | ---NA--- | | |
| TRINITY_DN21015_c0_g1_i7_len_351_248719.p1_type_internal_len_117 | ---NA--- | | |
| TRINITY_DN34402_c0_g1_i1_len_352_210616.p2_type_internal_len_117 | ---NA--- | | |
| TRINITY_DN19042_c0_g1_i1_len_820_4701.p2_type_complete_len_116 | XP_014249271.1lipoma-preferred partner homolog | F:GO:0046872 | |
| TRINITY_DN13908_c0_g1_i1_len_409_207614.p1_type_5prime_partial_len_116 | ---NA--- | | |
| TRINITY_DN16309_c0_g1_i1_len_373_209370.p1_type_5prime_partial_len_116 | ---NA--- | | |
| TRINITY_DN21042_c0_g1_i2_len_758_199144.p1_type_5prime_partial_len_116 | ---NA--- | | |
| TRINITY_DN26375_c1_g1_i4_len_1884_231455.p2_type_complete_len_116 | ---NA--- | | |
| TRINITY_DN31080_c0_g1_i4_len_761_199119.p1_type_complete_len_116 | ---NA--- | | |
| TRINITY_DN10249_c0_g1_i1_len_348_210866.p1_type_internal_len_117 | ---NA--- | | |

| | | | |
|---|---|---|---|
| TRINITY_DN21205_c0_g1_i1_len_4136_294678.p3_type_complete_len_116 | XP_018019508.1 isoleucine--tRNA ligase, cytoplasmic-like | F:GO:0004822; F:GO:0005524; P:GO:0006428 | EC:6.1.1.5 |
| TRINITY_DN27197_c0_g1_i1_len_352_248676.p1_type_internal_len_117 | ---NA--- | | |
| TRINITY_DN18603_c0_g1_i1_len_568_202451.p1_type_complete_len_116 | ---NA--- | | |
| TRINITY_DN15008_c0_g1_i1_len_351_13826.p1_type_internal_len_117 | ---NA--- | | |
| TRINITY_DN19239_c0_g1_i3_len_1072_160156.p4_type_3prime_partial_len_117 | ---NA--- | | |
| TRINITY_DN35152_c0_g1_i1_len_352_248668.p1_type_internal_len_117 | ---NA--- | | |
| TRINITY_DN24295_c0_g1_i1_len_1440_193421.p1_type_5prime_partial_len_116 | ---NA--- | | |
| TRINITY_DN36432_c0_g1_i1_len_351_248700.p1_type_internal_len_117 | ---NA--- | | |
| TRINITY_DN17479_c0_g1_i1_len_2527_265424.p3_type_complete_len_116 | XP_018022816.1 anoctamin-10-like | | |
| TRINITY_DN5653_c0_g1_i1_len_442_277580.p1_type_complete_len_116 | ---NA--- | | |
| TRINITY_DN25049_c0_g1_i1_len_350_13907.p1_type_internal_len_117 | ---NA--- | | |
| TRINITY_DN18566_c0_g1_i1_len_1156_159600.p2_type_complete_len_116 | ---NA--- | | |
| TRINITY_DN13288_c0_g1_i1_len_641_240088.p1_type_5prime_partial_len_116 | ---NA--- | | |
| TRINITY_DN35055_c0_g1_i1_len_358_210178.p1_type_5prime_partial_len_116 | AFJ11393.1glutathione-dependent prostaglandin D synthase | F:GO:0016740 | |
| TRINITY_DN33278_c0_g1_i1_len_350_248755.p1_type_internal_len_117 | KZS22087.1WD repeat-containing protein 7 | | |
| TRINITY_DN8423_c0_g1_i1_len_384_170891.p1_type_5prime_partial_len_116 | ---NA--- | | |

| | | | |
|---|---|---|---|
| TRINITY_DN30730_c0_g1_i2_len_4893_187161.p3_type_3prime_partial_len_117 | ---NA--- | | |
| TRINITY_DN206_c0_g1_i1_len_2482_189491.p2_type_complete_len_116 | ---NA--- | | |
| TRINITY_DN17349_c0_g1_i1_len_728_199608.p1_type_complete_len_116 | ---NA--- | | |
| TRINITY_DN18061_c0_g1_i1_len_528_242310.p1_type_5prime_partial_len_116 | ---NA--- | | |
| TRINITY_DN28637_c0_g1_i1_len_350_248757.p1_type_internal_len_117 | XP_018012106.1PREDICTED: uncharacterized protein LOC108669313 | | |
| TRINITY_DN35143_c0_g1_i1_len_352_210638.p1_type_internal_len_117 | ACC62400.2ribonuclease III | F:GO:0003723; F:GO:0004525; P:GO:0006396; P:GO:0016075; P:GO:0031047; P:GO:0090502 | EC:3.1.30; EC:3.1.26; EC:3.1.26.3 |
| TRINITY_DN13070_c0_g1_i1_len_350_13895.p1_type_5prime_partial_len_116 | ---NA--- | | |
| TRINITY_DN30314_c0_g1_i1_len_351_248748.p1_type_internal_len_117 | PIK54585.1putative thy-1 membrane glycoprotein isoform X1 | | |
| TRINITY_DN31565_c0_g1_i1_len_526_203498.p1_type_5prime_partial_len_116 | ---NA--- | | |
| TRINITY_DN32096_c0_g1_i1_len_348_210892.p1_type_internal_len_117 | XP_023714619.1UPF0577 protein KIAA1324-like homolog isoform X2 | | |
| TRINITY_DN11290_c0_g1_i2_len_432_71136.p1_type_5prime_partial_len_116 | ---NA--- | | |
| TRINITY_DN10129_c0_g1_i1_len_349_210838.p1_type_internal_len_117 | XP_021941112.1phospholipase DDHD1-like isoform X1 | | |
| TRINITY_DN26486_c0_g1_i1_len_352_172377.p1_type_internal_len_117 | ---NA--- | | |
| TRINITY_DN21160_c0_g1_i1_len_403_207883.p1_type_5prime_partial_len_116 | ---NA--- | | |
| TRINITY_DN34963_c0_g1_i1_len_348_210862.p1_type_internal_len_117 | ---NA--- | | |
| TRINITY_DN20394_c0_g1_i1_len_1469_193255.p2_type_complete_len_116 | ---NA--- | | |

| | | | |
|---|---|---|---|
| TRINITY_DN35940_c0_g1_i1_len_348_210894.p1_type_internal_len_117 | ---NA--- | | |
| TRINITY_DN2850_c0_g1_i1_len_348_309309.p1_type_internal_len_117 | XP_013792251.2protein farnesyltransferase subunit beta-like | F:GO:0003824 | |
| TRINITY_DN18862_c0_g1_i2_len_349_13935.p2_type_internal_len_117 | ---NA--- | | |
| TRINITY_DN35125_c0_g1_i1_len_351_74705.p3_type_internal_len_117 | XP_023725938.1methionine-R-sulfoxide reductase B1 isoform X8 | | |
| TRINITY_DN18247_c0_g1_i1_len_2032_231027.p3_type_5prime_partial_len_116 | ---NA--- | | |
| TRINITY_DN18418_c0_g1_i1_len_2053_702.p2_type_5prime_partial_len_116 | ---NA--- | | |
| TRINITY_DN23314_c0_g1_i1_len_3837_153743.p2_type_complete_len_116 | XP_022191793.1regulator of telomere elongation helicase 1 homolog | F:GO:0004386; F:GO:0097159; F:GO:1901363 | EC:3.6.1.15 |
| TRINITY_DN3199_c0_g1_i1_len_549_275003.p1_type_5prime_partial_len_116 | ---NA--- | | |
| TRINITY_DN30358_c0_g1_i1_len_351_248721.p1_type_5prime_partial_len_116 | XP_018012135.1 S1 RNA-binding domain-containing protein 1-like isoform X2 | | |
| TRINITY_DN4263_c0_g1_i1_len_349_309252.p1_type_internal_len_117 | ---NA--- | | |
| TRINITY_DN15760_c0_g1_i1_len_409_207657.p1_type_5prime_partial_len_116 | XP_015112091.1 acetyl-CoA acetyltransferase, mitochondrial isoform X1 | F:GO:0016746 | |
| TRINITY_DN15543_c0_g1_i1_len_348_309278.p1_type_internal_len_117 | XP_014284054.1protein big brother isoform X2 | F:GO:0003713; C:GO:0005634; P:GO:1903508 | |
| TRINITY_DN16437_c0_g1_i1_len_349_309267.p1_type_internal_len_117 | ---NA--- | | |
| TRINITY_DN22194_c0_g1_i1_len_540_275168.p1_type_complete_len_116 | ---NA--- | | |
| TRINITY_DN16954_c0_g1_i1_len_408_207683.p1_type_5prime_partial_len_116 | ---NA--- | | |
| TRINITY_DN35745_c0_g1_i1_len_348_210884.p1_type_internal_len_117 | ---NA--- | | |

| | | | |
|---|---|---|---|
| TRINITY_DN2832_c0_g1_i1_len_351_248745.p1_type_internal_len_117 | ---NA--- | | |
| TRINITY_DN15747_c0_g1_i1_len_526_203507.p1_type_5prime_partial_len_116 | ---NA--- | | |
| TRINITY_DN13765_c0_g1_i2_len_1051_235718.p2_type_5prime_partial_len_116 | ---NA--- | | |
| TRINITY_DN21093_c0_g1_i1_len_444_70769.p1_type_5prime_partial_len_116 | ---NA--- | | |
| TRINITY_DN10874_c0_g1_i1_len_500_304170.p2_type_5prime_partial_len_116 | ---NA--- | | |
| TRINITY_DN27242_c0_g1_i1_len_725_199639.p1_type_3prime_partial_len_117 | ---NA--- | | |
| TRINITY_DN7955_c0_g1_i1_len_469_102132.p2_type_5prime_partial_len_116 | ---NA--- | | |
| TRINITY_DN26513_c0_g1_i1_len_351_248732.p1_type_internal_len_117 | ---NA--- | | |
| TRINITY_DN34036_c0_g1_i1_len_524_203545.p2_type_complete_len_116 | ---NA--- | | |
| TRINITY_DN30799_c0_g1_i1_len_504_101195.p1_type_complete_len_116 | ---NA--- | | |
| TRINITY_DN30764_c0_g1_i1_len_463_244120.p2_type_complete_len_116 | XP_015512541.1 histone-lysine N-methyltransferase SETD1 | | |
| TRINITY_DN31367_c0_g1_i1_len_348_172588.p1_type_internal_len_117 | ---NA--- | | |
| TRINITY_DN32505_c0_g1_i1_len_349_248837.p2_type_internal_len_117 | XP_021928072.1 synaptotagmin-4-like | C:GO:0016020 | |
| TRINITY_DN21755_c0_g2_i2_len_958_299036.p1_type_complete_len_116 | ---NA--- | | |
| TRINITY_DN29013_c0_g1_i1_len_384_105035.p1_type_5prime_partial_len_116 | ---NA--- | | |
| TRINITY_DN37575_c0_g1_i1_len_350_248785.p1_type_internal_len_117 | XP_021930425.1laminin subunit alpha | | |

| | | | |
|---|---|---|---|
| TRINITY_DN13106_c0_g1_i1_len_433_103221.p1_type_5prime_partial_len_116 | ---NA--- | | |
| TRINITY_DN22092_c0_g1_i3_len_3910_228236.p3_type_5prime_partial_len_116 | ---NA--- | | |
| TRINITY_DN38877_c0_g1_i1_len_350_210768.p1_type_internal_len_117 | ---NA--- | | |
| TRINITY_DN15693_c0_g1_i3_len_349_281121.p1_type_internal_len_117 | ---NA--- | | |
| TRINITY_DN3140_c0_g1_i1_len_652_164174.p1_type_complete_len_116 | ---NA--- | | |
| TRINITY_DN14046_c0_g1_i3_len_350_210766.p2_type_internal_len_117 | ACI04663.1tropomyosin, partial | | |
| TRINITY_DN14475_c0_g1_i1_len_424_206986.p1_type_5prime_partial_len_116 | ---NA--- | | |
| TRINITY_DN32904_c0_g1_i1_len_460_244228.p1_type_3prime_partial_len_117 | ---NA--- | | |
| TRINITY_DN28697_c0_g1_i1_len_449_168464.p1_type_3prime_partial_len_117 | AIT97138.1innexin 7 | C:GO:0005886; C:GO:0005921; P:GO:0006811; C:GO:0016021 | |
| TRINITY_DN10388_c0_g1_i2_len_350_210770.p1_type_internal_len_117 | ---NA--- | | |
| TRINITY_DN19779_c0_g1_i2_len_350_248789.p1_type_internal_len_117 | APO14259.1juvenile hormone esterase-like carboxylesterase 1 | F:GO:0016787 | |
| TRINITY_DN7056_c0_g1_i1_len_350_210771.p1_type_internal_len_117 | XP_018012069.1 zinc finger protein 2 homolog | | |
| TRINITY_DN32340_c0_g1_i1_len_350_248788.p1_type_internal_len_117 | XP_011335913.1 dystrobrevin beta isoform X5 | | |
| 2536\|_\|c2478_f3p2_2563_isoform_c2478_full_length_coverage_3_non_full_length_coverage_2_isoform_length_2563_16444 | KZS15921.1XK-related protein | C:GO:0016020; C:GO:0016021 | |
| 2537\|_\|c23649_f2p17_2563_isoform_c23649_full_length_coverage_2_non_full_length_coverage_17_isoform_length_2563_22916 | PSN45792.1CCR4-NOT transcription complex subunit 10 | C:GO:0030014 | |

<table>
<tr><td>2538|_|c4233_f3p2_2563_isoform_c4233_full_length_coverage_3_non_full_length_coverage_2_isoform_length_2563_16837</td><td>---NA---</td><td></td><td></td></tr>
<tr><td>2539|_|c4122_f2p1_2562_isoform_c4122_full_length_coverage_2_non_full_length_coverage_1_isoform_length_2562_12351</td><td>---NA---</td><td></td><td></td></tr>
<tr><td>2540|_|c2313_f3p2_2562_isoform_c2313_full_length_coverage_3_non_full_length_coverage_2_isoform_length_2562_20838</td><td>XP_023719030.1protein PRRC2C-like isoform X4</td><td></td><td></td></tr>
<tr><td>2541|_|c2116_f6p10_2562_isoform_c2116_full_length_coverage_6_non_full_length_coverage_10_isoform_length_2562_20717</td><td>XP_018025321.1 SLAIN motif-containing protein 2-like</td><td></td><td></td></tr>
<tr><td>2543|_|c2837_f2p5_2562_isoform_c2837_full_length_coverage_2_non_full_length_coverage_5_isoform_length_2562_21276</td><td>XP_018013254.1 eukaryotic peptide chain release factor GTP-binding subunit ERF3A-like</td><td>P:GO:0000288; F:GO:0003746; F:GO:0003747; F:GO:0003924; F:GO:0005525; P:GO:0006414; P:GO:0006415</td><td>EC:3.6.1.15</td></tr>
<tr><td>TRINITY_DN23122_c0_g1_i1_len_2675_229477.p1_type_5prime_partial_len_854</td><td>OXA51005.1Smith-Magenis syndrome chromosomal region candidate gene 8 protein</td><td></td><td></td></tr>
<tr><td>TRINITY_DN22232_c0_g1_i1_len_2663_154737.p1_type_5prime_partial_len_854</td><td>XP_018020841.1 ligand of Numb protein X 2-like</td><td></td><td></td></tr>
<tr><td>2547|_|c2324_f3p1_2561_isoform_c2324_full_length_coverage_3_non_full_length_coverage_1_isoform_length_2561_20848</td><td>XP_018023231.1 condensin complex subunit 3-like</td><td>C:GO:0000796; P:GO:0007076</td><td></td></tr>
<tr><td>TRINITY_DN11897_c0_g1_i1_len_349_172542.p1_type_internal_len_117</td><td>---NA---</td><td></td><td></td></tr>
<tr><td>TRINITY_DN31986_c0_g1_i1_len_349_106647.p2_type_internal_len_117</td><td>---NA---</td><td></td><td></td></tr>
<tr><td>TRINITY_DN7916_c0_g1_i1_len_350_248779.p2_type_internal_len_117</td><td>---NA---</td><td></td><td></td></tr>
<tr><td>TRINITY_DN22276_c0_g1_i1_len_349_106691.p1_type_internal_len_117</td><td>---NA---</td><td></td><td></td></tr>
</table>

| | | | |
|---|---|---|---|
| TRINITY_DN916_c0_g1_i1_len_349_210780.p1_type_internal_len_117 | XP_021927794.1cubilin-like isoform X1 | F:GO:0005509 | |
| TRINITY_DN30307_c2_g1_i5_len_1377_193795.p1_type_complete_len_116 | ---NA--- | | |
| TRINITY_DN30907_c0_g1_i1_len_349_106678.p1_type_internal_len_117 | ---NA--- | | |
| TRINITY_DN4355_c0_g1_i1_len_349_210782.p1_type_internal_len_117 | ---NA--- | | |
| TRINITY_DN17819_c0_g1_i1_len_351_106583.p1_type_internal_len_117 | ---NA--- | | |
| TRINITY_DN38609_c0_g1_i2_len_349_210787.p1_type_internal_len_117 | ---NA--- | | |
| TRINITY_DN30190_c0_g1_i1_len_350_74767.p2_type_internal_len_117 | OXA44641.1Williams-Beuren syndrome chromosomal region 27 protein | | |
| TRINITY_DN12898_c0_g1_i1_len_542_131447.p1_type_5prime_partial_len_116 | XP_020634102.1heme-binding protein 2 | | |
| TRINITY_DN19350_c0_g1_i1_len_425_206932.p1_type_5prime_partial_len_116 | ---NA--- | | |
| TRINITY_DN30476_c0_g1_i1_len_348_172562.p1_type_internal_len_117 | XP_018023091.1 vacuolar protein sorting-associated protein 13B-like | | |
| TRINITY_DN28271_c0_g1_i1_len_350_74766.p1_type_internal_len_117 | AWT50625.1neuropeptide receptor | F:GO:0004930; P:GO:0007186; C:GO:0016021 | |
| TRINITY_DN1856_c0_g1_i1_len_349_248849.p1_type_internal_len_117 | XP_018026900.1 cat eye syndrome critical region protein 5-like | | |
| TRINITY_DN40871_c0_g1_i1_len_439_206376.p1_type_5prime_partial_len_116 | AFP33455.1kinesin-like protein KIF3A | F:GO:0003777; F:GO:0005524; C:GO:0005874; P:GO:0007018; F:GO:0008017 | EC:3.6.1.15 |
| TRINITY_DN15913_c0_g1_i1_len_351_210691.p1_type_internal_len_117 | ---NA--- | | |
| TRINITY_DN37088_c0_g1_i1_len_350_210748.p1_type_internal_len_117 | ---NA--- | | |
| TRINITY_DN36027_c0_g1_i1_len_349_74823.p2_type_internal_len_117 | ---NA--- | | |

| | | | |
|---|---|---|---|
| TRINITY_DN8694_c0_g1_i1_len_349_248821.p1_type_internal_len_117 | XP_018012974.1PREDICTED: uncharacterized protein LOC108670035 | | |
| TRINITY_DN31264_c0_g1_i1_len_350_74789.p1_type_internal_len_117 | XP_018011388.1 ral GTPase-activating protein subunit alpha-1-like | | |
| TRINITY_DN33451_c0_g1_i1_len_350_210751.p1_type_internal_len_117 | XP_018017230.1 dmX-like protein 2 isoform X1 | | |
| TRINITY_DN37092_c0_g1_i1_len_349_210814.p2_type_internal_len_117 | XP_013860411.1 zinc finger protein OZF-like | F:GO:0003676 | |
| TRINITY_DN1749_c0_g1_i1_len_350_210742.p1_type_internal_len_117 | ---NA--- | | |
| TRINITY_DN2126_c0_g1_i2_len_350_281085.p1_type_internal_len_117 | XP_018012287.1 otopetrin-2-like isoform X10 | | |
| TRINITY_DN38996_c0_g1_i1_len_350_210739.p1_type_internal_len_117 | ---NA--- | | |
| TRINITY_DN19473_c0_g1_i1_len_349_210825.p1_type_internal_len_117 | ---NA--- | | |
| TRINITY_DN28160_c0_g1_i1_len_350_281092.p2_type_internal_len_117 | ENN76034.1hypothetical protein YQE_07407 | | |
| TRINITY_DN26066_c0_g1_i2_len_3986_228194.p2_type_complete_len_116 | ---NA--- | | |
| TRINITY_DN32758_c0_g1_i1_len_350_248797.p1_type_internal_len_117 | ---NA--- | | |
| TRINITY_DN37095_c0_g1_i1_len_350_210753.p1_type_internal_len_117 | XP_013086292.1 G/T mismatch-specific thymine DNA glycosylase-like | P:GO:0006281; F:GO:0019104 | |
| TRINITY_DN11313_c0_g1_i1_len_351_281046.p1_type_internal_len_117 | ---NA--- | | |
| TRINITY_DN27247_c0_g1_i1_len_419_134698.p1_type_5prime_partial_len_116 | XP_018015338.1 zinc finger protein ubi-d4 B-like | F:GO:0046872 | |
| TRINITY_DN37317_c0_g1_i1_len_350_210728.p1_type_internal_len_117 | ---NA--- | | |
| TRINITY_DN41334_c0_g1_i1_len_350_210743.p2_type_internal_len_117 | EFX89755.1transcriptional factor scalloped, isoform 1 | F:GO:0003677; F:GO:0003700; C:GO:0005634; P:GO:0006355; P:GO:0035329 | |

| | | | |
|---|---|---|---|
| TRINITY_DN8425_c0_g1_i1_len_468_167924.p1_type_5prime_partial_len_116 | ---NA--- | | |
| TRINITY_DN22795_c3_g1_i3_len_350_248798.p1_type_internal_len_117 | PRD18460.1zinc finger protein | F:GO:0003676 | |
| TRINITY_DN19531_c0_g1_i3_len_717_300946.p2_type_complete_len_116 | ---NA--- | | |
| TRINITY_DN5866_c0_g1_i1_len_458_205634.p1_type_3prime_partial_len_117 | XP_018019004.1 oxysterol-binding protein-related protein 11-like | | |
| TRINITY_DN11282_c0_g1_i1_len_349_210823.p1_type_internal_len_117 | ---NA--- | | |
| TRINITY_DN15017_c0_g1_i1_len_434_245116.p1_type_5prime_partial_len_116 | ---NA--- | | |
| TRINITY_DN20037_c0_g1_i1_len_1303_94018.p2_type_5prime_partial_len_116 | ---NA--- | | |
| TRINITY_DN27445_c0_g1_i1_len_350_281111.p1_type_internal_len_117 | ---NA--- | | |
| TRINITY_DN25723_c0_g1_i1_len_350_281097.p1_type_internal_len_117 | ---NA--- | | |
| TRINITY_DN15625_c1_g1_i1_len_349_210810.p1_type_internal_len_117 | ---NA--- | | |
| TRINITY_DN31860_c0_g1_i1_len_454_168325.p1_type_complete_len_116 | ---NA--- | | |
| TRINITY_DN32107_c0_g1_i1_len_350_210764.p1_type_internal_len_117 | XP_014247904.1pleckstrin homology domain-containing family G member 3 isoform X1 | | |
| TRINITY_DN21768_c0_g1_i1_len_592_165131.p2_type_5prime_partial_len_116 | PRD17995.1zinc finger protein | F:GO:0003676 | |
| TRINITY_DN32138_c0_g1_i1_len_348_172603.p2_type_internal_len_117 | ---NA--- | | |
| TRINITY_DN20528_c0_g1_i3_len_2431_265548.p1_type_complete_len_116 | ---NA--- | | |
| TRINITY_DN23185_c0_g1_i1_len_446_244716.p1_type_5prime_partial_len_116 | ---NA--- | | |

| | | | |
|---|---|---|---|
| TRINITY_DN33537_c0_g1_i1_len_350_248795.p3_type_internal_len_117 | ---NA--- | | |
| TRINITY_DN16096_c0_g1_i1_len_385_307607.p3_type_5prime_partial_len_116 | ---NA--- | | |
| TRINITY_DN9468_c0_g1_i2_len_573_99882.p1_type_complete_len_116 | ---NA--- | | |
| TRINITY_DN34995_c0_g1_i1_len_350_210756.p1_type_internal_len_117 | XP_018025827.1 DNA-dependent protein kinase catalytic subunit-like | | |
| TRINITY_DN1613_c0_g1_i1_len_350_210724.p1_type_internal_len_117 | ---NA--- | | |
| TRINITY_DN4933_c0_g1_i1_len_350_281095.p1_type_internal_len_117 | ---NA--- | | |
| TRINITY_DN7402_c0_g1_i1_len_396_72536.p1_type_5prime_partial_len_116 | ---NA--- | | |
| TRINITY_DN1449_c0_g1_i2_len_350_210757.p1_type_internal_len_117 | AKL71381.1IGF-binding protein | F:GO:0005520; C:GO:0005576 | |
| TRINITY_DN34909_c0_g1_i1_len_350_210720.p1_type_internal_len_117 | ---NA--- | | |
| TRINITY_DN1482_c0_g1_i1_len_581_37236.p1_type_3prime_partial_len_117 | ---NA--- | | |
| TRINITY_DN18385_c0_g1_i1_len_437_103075.p1_type_5prime_partial_len_116 | ---NA--- | | |
| TRINITY_DN26166_c2_g1_i1_len_349_248834.p1_type_internal_len_117 | ---NA--- | | |
| TRINITY_DN7805_c0_g1_i1_len_348_210848.p1_type_internal_len_117 | ---NA--- | | |
| TRINITY_DN13598_c0_g1_i3_len_350_172475.p1_type_5prime_partial_len_116 | ---NA--- | | |
| TRINITY_DN29640_c0_g1_i1_len_349_172519.p1_type_internal_len_117 | ---NA--- | | |
| TRINITY_DN25262_c0_g1_i1_len_350_172474.p1_type_internal_len_117 | XP_018016317.1 kelch-like protein 18 | | |

| | | | |
|---|---|---|---|
| TRINITY_DN39742_c0_g1_i1_len_349_210801.p1_type_internal_len_117 | EEN44003.1smoothend-like protein protein | F:GO:0004888; P:GO:0007166; P:GO:0007224; P:GO:0007275; C:GO:0016020; C:GO:0016021 | |
| TRINITY_DN29068_c0_g1_i1_len_348_248858.p1_type_internal_len_117 | ---NA--- | | |
| TRINITY_DN24853_c0_g1_i1_len_3789_228298.p3_type_5prime_partial_len_116 | ---NA--- | | |
| TRINITY_DN23890_c0_g1_i2_len_1650_60201.p2_type_complete_len_116 | ATU31736.1LIM domain-binding protein 2 | | |
| TRINITY_DN28032_c0_g1_i1_len_350_248773.p1_type_internal_len_117 | ---NA--- | | |
| TRINITY_DN25040_c0_g1_i1_len_2949_229061.p4_type_complete_len_116 | ---NA--- | | |
| TRINITY_DN34848_c0_g1_i1_len_349_248856.p2_type_internal_len_117 | ---NA--- | | |
| TRINITY_DN27832_c0_g1_i1_len_520_131910.p2_type_complete_len_116 | KFP49773.1Tripartite motif-containing protein 45 | F:GO:0008270 | |
| TRINITY_DN4065_c0_g1_i1_len_562_67770.p1_type_5prime_partial_len_116 | ---NA--- | | |
| TRINITY_DN18454_c0_g1_i1_len_899_197502.p2_type_complete_len_116 | KFM81476.1Soluble guanylate cyclase 88E | F:GO:0004383; P:GO:0006182; F:GO:0020037; P:GO:0035556 | EC:4.6.1.2 |
| TRINITY_DN29721_c0_g1_i1_len_350_43558.p1_type_internal_len_117 | ---NA--- | | |
| TRINITY_DN10050_c0_g1_i1_len_349_210794.p1_type_internal_len_117 | ---NA--- | | |
| TRINITY_DN30642_c0_g1_i1_len_350_172496.p1_type_internal_len_117 | ---NA--- | | |
| TRINITY_DN6656_c0_g1_i1_len_349_210792.p1_type_internal_len_117 | ---NA--- | | |
| TRINITY_DN9588_c0_g1_i1_len_1025_196354.p3_type_complete_len_116 | ---NA--- | | |
| TRINITY_DN16684_c0_g1_i2_len_500_167050.p1_type_5prime_partial_len_116 | ---NA--- | | |

| | | | |
|---|---|---|---|
| TRINITY_DN12933_c0_g1_i1_len_489_243329.p1_type_complete_len_116 | ---NA--- | | |
| TRINITY_DN4974_c0_g1_i1_len_348_74873.p1_type_internal_len_117 | XP_018012386.1 ankyrin-3-like | | |
| TRINITY_DN30468_c0_g1_i1_len_350_43539.p2_type_internal_len_117 | ---NA--- | | |
| TRINITY_DN21146_c0_g1_i1_len_348_281159.p1_type_internal_len_117 | ---NA--- | | |
| TRINITY_DN39770_c0_g1_i1_len_351_210671.p1_type_internal_len_117 | ---NA--- | | |
| TRINITY_DN2256_c0_g1_i1_len_429_103329.p1_type_5prime_partial_len_116 | ---NA--- | | |
| TRINITY_DN16278_c0_g1_i1_len_350_74749.p1_type_internal_len_117 | ---NA--- | | |
| TRINITY_DN2569_c0_g1_i1_len_351_210680.p1_type_internal_len_117 | ---NA--- | | |
| TRINITY_DN32349_c0_g1_i1_len_348_248889.p1_type_internal_len_117 | XP_018020695.1 zinc finger protein 813-like | F:GO:0003676 | |
| TRINITY_DN18525_c0_g1_i1_len_587_67324.p1_type_3prime_partial_len_117 | ---NA--- | | |
| TRINITY_DN18233_c0_g1_i1_len_593_302462.p1_type_5prime_partial_len_116 | ---NA--- | | |
| TRINITY_DN18913_c0_g2_i1_len_352_137566.p1_type_5prime_partial_len_116 | ---NA--- | | |
| TRINITY_DN119_c0_g1_i1_len_348_248912.p1_type_internal_len_117 | ---NA--- | | |
| TRINITY_DN2838_c0_g1_i1_len_351_43492.p1_type_internal_len_117 | ---NA--- | | |
| TRINITY_DN464_c0_g1_i1_len_351_210661.p1_type_internal_len_117 | ---NA--- | | |
| TRINITY_DN14362_c0_g1_i1_len_677_301381.p2_type_5prime_partial_len_116 | ---NA--- | | |

| | | | |
|---|---|---|---|
| 2548\|_\|c19878_f2p0_2561_isoform_c19878_full_length_coverage_2_non_full_length_coverage_0_isoform_length_2561_22796 | ---NA--- | | |
| 2552\|_\|c13911_f2p0_2560_isoform_c13911_full_length_coverage_2_non_full_length_coverage_0_isoform_length_2560_28642 | XP_018017948.1 actin-binding protein anillin-like | | |
| TRINITY_DN27358_c0_g1_i1_len_2912_188674.p1_type_5prime_partial_len_853 | XP_025833685.1glycine--tRNA ligase-like | F:GO:0000166; F:GO:0004812; P:GO:0006418 | |
| 2554\|_\|c2707_f7p1_2559_isoform_c2707_full_length_coverage_7_non_full_length_coverage_1_isoform_length_2559_21167 | XP_018020708.1 uridine-cytidine kinase-like 1 | F:GO:0004849; F:GO:0005524; P:GO:0009116; P:GO:0016310; P:GO:0044206; P:GO:0044211 | EC:2.7.1.48 |
| TRINITY_DN18972_c0_g1_i1_len_4015_153682.p1_type_complete_len_853 | KDR08233.1E3 ubiquitin-protein ligase RNF19A | F:GO:0004842; C:GO:0016021; P:GO:0016567; F:GO:0016874; F:GO:0046872 | |
| 2556\|_\|c18292_f2p5_2559_isoform_c18292_full_length_coverage_2_non_full_length_coverage_5_isoform_length_2559_13700 | XP_025996575.1protein downstream neighbor of son homolog | | |
| TRINITY_DN27861_c0_g1_i1_len_3021_188504.p1_type_5prime_partial_len_853 | XP_018018348.1 U4/U6.U5 tri-snRNP-associated protein 1-like | P:GO:0000398 | |
| 2558\|_\|c2808_f2p0_2558_isoform_c2808_full_length_coverage_2_non_full_length_coverage_0_isoform_length_2558_21255 | XP_026286226.1DNA polymerase alpha catalytic subunit isoform X1 | | |
| 2559\|_\|c1818_f7p3_2558_isoform_c1818_full_length_coverage_7_non_full_length_coverage_3_isoform_length_2558_20550 | KZS19834.1Fidgetin protein 1 | C:GO:0000228; P:GO:0001649; F:GO:0005524; P:GO:0007140; P:GO:0010569; P:GO:0033687; C:GO:0048471; P:GO:0051726; P:GO:0071479; P:GO:2001243 | |
| 2560\|_\|c5191_f2p1_2558_isoform_c5191_full_length_coverage_2_non_full_length_coverage_1_isoform_length_2558_16937 | XP_018027299.1PREDICTED: uncharacterized protein LOC108682613 | | |
| TRINITY_DN27934_c0_g1_i1_len_2204_190226.p3_type_5prime_partial_len_116 | ---NA--- | | |

| | | | |
|---|---|---|---|
| TRINITY_DN1168_c0_g1_i1_len_352_106530.p1_type_internal_len_117 | AUB13319.1C-type lectin containing low-density lipoprotein receptor domain | F:GO:0030246 | |
| TRINITY_DN12461_c0_g1_i1_len_351_106532.p1_type_5prime_partial_len_116 | ---NA--- | | |
| TRINITY_DN9851_c0_g1_i1_len_352_137601.p1_type_internal_len_117 | ---NA--- | | |
| TRINITY_DN12828_c0_g1_i1_len_430_206732.p1_type_5prime_partial_len_116 | ---NA--- | | |
| TRINITY_DN11411_c0_g1_i1_len_429_103342.p1_type_5prime_partial_len_116 | ---NA--- | | |
| TRINITY_DN4817_c0_g1_i1_len_349_43606.p1_type_internal_len_117 | ---NA--- | | |
| TRINITY_DN33200_c0_g1_i1_len_351_137644.p2_type_internal_len_117 | XP_022204716.1protein shifted-like | | |
| TRINITY_DN26544_c0_g1_i1_len_348_248888.p1_type_internal_len_117 | XP_022237072.1organic cation transporter protein-like isoform X1 | | |
| TRINITY_DN29421_c0_g1_i1_len_349_43613.p1_type_internal_len_117 | ---NA--- | | |
| TRINITY_DN4918_c0_g1_i1_len_349_210796.p1_type_internal_len_117 | ---NA--- | | |
| TRINITY_DN27712_c0_g1_i1_len_2724_188994.p3_type_complete_len_116 | ---NA--- | | |
| TRINITY_DN14115_c0_g1_i1_len_351_210674.p1_type_internal_len_117 | ---NA--- | | |
| TRINITY_DN16699_c0_g1_i1_len_811_96962.p2_type_complete_len_115 | ---NA--- | | |
| TRINITY_DN17710_c0_g1_i2_len_590_201988.p1_type_5prime_partial_len_115 | ---NA--- | | |
| TRINITY_DN17126_c0_g1_i1_len_917_299302.p1_type_5prime_partial_len_115 | ---NA--- | | |
| TRINITY_DN25262_c0_g1_i5_len_1712_232098.p3_type_complete_len_115 | EKC19904.1Threonine synthase-like 2 | | |

| | | | |
|---|---|---|---|
| TRINITY_DN23678_c0_g1_i1_len_345_172727.p1_type_internal_len_116 | ---NA--- | | |
| TRINITY_DN26098_c0_g1_i1_len_3658_228365.p2_type_complete_len_115 | ---NA--- | | |
| TRINITY_DN2102_c0_g1_i1_len_690_200161.p1_type_3prime_partial_len_116 | ALC78850.15'-nucleotidase domain-containing protein 3 | F:GO:0046872 | |
| TRINITY_DN17157_c0_g1_i1_len_347_210929.p2_type_internal_len_116 | ABX83902.1type II hemocyte transglutaminase | F:GO:0003810; P:GO:0018149; F:GO:0046872 | EC:2.3.2.13 |
| TRINITY_DN14370_c0_g1_i3_len_907_197405.p1_type_5prime_partial_len_115 | ---NA--- | | |
| TRINITY_DN19605_c0_g1_i3_len_810_198492.p1_type_complete_len_115 | XP_012782187.1 protein Churchill | P:GO:0007275; F:GO:0008270; P:GO:0045893 | |
| TRINITY_DN20550_c0_g1_i2_len_932_161228.p2_type_complete_len_115 | ---NA--- | | |
| TRINITY_DN4101_c0_g1_i1_len_449_206006.p1_type_5prime_partial_len_115 | ALT10383.1juvenile hormone esterase-like protein 1 | F:GO:0016787 | |
| TRINITY_DN21916_c1_g1_i2_len_464_205420.p1_type_5prime_partial_len_115 | ---NA--- | | |
| TRINITY_DN4494_c0_g1_i1_len_590_201993.p1_type_5prime_partial_len_115 | ---NA--- | | |
| TRINITY_DN19180_c0_g1_i1_len_640_66482.p1_type_5prime_partial_len_115 | XP_018017895.1 protein lin-52 homolog | P:GO:0006351; C:GO:0070176 | |
| TRINITY_DN7547_c0_g1_i1_len_347_74943.p1_type_internal_len_116 | ---NA--- | | |
| TRINITY_DN31389_c0_g1_i1_len_348_248882.p1_type_internal_len_116 | ---NA--- | | |
| TRINITY_DN24699_c0_g1_i4_len_2232_58895.p3_type_complete_len_115 | ---NA--- | | |
| TRINITY_DN37555_c0_g1_i1_len_375_209276.p1_type_5prime_partial_len_115 | XP_018016444.1 serine-rich adhesin for platelets-like | | |
| TRINITY_DN29678_c0_g1_i1_len_424_278154.p1_type_5prime_partial_len_115 | ---NA--- | | |

| | | | |
|---|---|---|---|
| TRINITY_DN25655_c0_g1_i1_len_348_74846.p1_type_internal_len_116 | ---NA--- | | |
| TRINITY_DN9507_c0_g1_i1_len_356_172166.p1_type_complete_len_115 | AOG14389.1histamine receptor type 4 | F:GO:0004888; F:GO:0005230; C:GO:0005886; C:GO:0016021; P:GO:0034220 | |
| TRINITY_DN17420_c0_g1_i1_len_3283_29382.p5_type_complete_len_115 | ---NA--- | | |
| TRINITY_DN21504_c0_g1_i2_len_349_74818.p1_type_internal_len_116 | XP_018008496.1 alpha-1,6-mannosyl-glycoprotein 2-beta-N-acetylglucosaminyltransferase-like | | |
| TRINITY_DN8928_c0_g1_i1_len_347_210954.p1_type_internal_len_116 | ---NA--- | | |
| TRINITY_DN21026_c0_g1_i1_len_1880_92385.p2_type_complete_len_115 | ---NA--- | | |
| TRINITY_DN34269_c0_g1_i1_len_346_210983.p2_type_internal_len_116 | ---NA--- | | |
| TRINITY_DN22072_c0_g1_i1_len_346_281300.p1_type_internal_len_116 | ---NA--- | | |
| TRINITY_DN8466_c0_g1_i2_len_349_248815.p2_type_internal_len_116 | XP_018011046.1 pollen-specific leucine-rich repeat extensin-like protein 2 | C:GO:0005576; P:GO:0006030; F:GO:0008061 | |
| TRINITY_DN30108_c0_g1_i1_len_348_309297.p1_type_internal_len_116 | XP_018018224.1 protein O-linked-mannose beta-1,2-N-acetylglucosaminyltransferase 1-like | P:GO:0006486; F:GO:0008375; F:GO:0016740 | |
| TRINITY_DN2656_c0_g1_i2_len_347_74925.p1_type_internal_len_116 | ABY77968.1tetraspanin-like protein CD9 | C:GO:0016021 | |
| TRINITY_DN24682_c0_g1_i1_len_3741_228321.p3_type_5prime_partial_len_115 | ---NA--- | | |
| TRINITY_DN1428_c0_g1_i1_len_347_309338.p1_type_internal_len_116 | ---NA--- | | |
| TRINITY_DN20975_c0_g1_i1_len_1412_193583.p2_type_3prime_partial_len_116 | XP_018016688.1 tRNA selenocysteine 1-associated protein 1-like | F:GO:0003723 | |
| TRINITY_DN862_c0_g1_i1_len_636_98893.p1_type_5prime_partial_len_115 | ---NA--- | | |

| | | | |
|---|---|---|---|
| TRINITY_DN5689_c0_g1_i1_len_348_248872.p1_type_internal_len_116 | XP_018008840.1 collagen alpha-5(IV) chain-like | F:GO:0005201; C:GO:0005581 | |
| TRINITY_DN28015_c0_g1_i1_len_1872_191381.p5_type_complete_len_115 | ---NA--- | | |
| TRINITY_DN12381_c0_g1_i1_len_347_309344.p1_type_internal_len_116 | ---NA--- | | |
| TRINITY_DN24715_c0_g1_i1_len_409_169865.p1_type_5prime_partial_len_115 | XP_018018089.1 small G protein signaling modulator 2-like | | |
| TRINITY_DN13558_c0_g1_i1_len_349_13938.p1_type_internal_len_116 | XP_018017173.1 degenerin mec-10-like | | |
| TRINITY_DN15973_c0_g1_i1_len_974_196769.p2_type_complete_len_115 | XP_011448421.1 UPF0538 protein C2orf76 homolog isoform X1 | | |
| TRINITY_DN6031_c0_g1_i1_len_433_206600.p1_type_3prime_partial_len_116 | ---NA--- | | |
| TRINITY_DN21517_c0_g1_i2_len_1611_192503.p2_type_complete_len_115 | ---NA--- | | |
| TRINITY_DN38936_c0_g1_i1_len_347_210958.p1_type_internal_len_116 | ---NA--- | | |
| TRINITY_DN19771_c0_g1_i1_len_1557_192779.p1_type_complete_len_115 | ---NA--- | | |
| TRINITY_DN24428_c0_g1_i1_len_2823_229243.p2_type_complete_len_115 | ---NA--- | | |
| TRINITY_DN20496_c0_g1_i1_len_1096_235401.p1_type_complete_len_115 | ---NA--- | | |
| TRINITY_DN30883_c0_g1_i1_len_345_249084.p1_type_internal_len_116 | XP_018025139.1 meckelin-like | | |
| TRINITY_DN22870_c0_g1_i1_len_345_249057.p1_type_internal_len_116 | ---NA--- | | |
| TRINITY_DN19019_c0_g1_i1_len_739_5355.p1_type_complete_len_115 | AON76443.1defender against apoptotic death | F:GO:0004579; P:GO:0006486; C:GO:0008250; C:GO:0016021 | EC:2.4.1.119 |
| TRINITY_DN12344_c0_g1_i1_len_348_74867.p1_type_5prime_partial_len_115 | ---NA--- | | |

| | | | |
|---|---|---|---|
| TRINITY_DN40689_c0_g1_i1_len_348_210855.p1_type_internal_len_116 | ---NA--- | | |
| TRINITY_DN14241_c0_g1_i1_len_362_106007.p1_type_5prime_partial_len_115 | ---NA--- | | |
| TRINITY_DN29594_c0_g1_i2_len_3039_188475.p2_type_3prime_partial_len_116 | ---NA--- | | |
| TRINITY_DN3914_c0_g1_i1_len_348_74889.p1_type_internal_len_116 | ---NA--- | | |
| TRINITY_DN4519_c0_g1_i1_len_346_211026.p1_type_internal_len_116 | ---NA--- | | |
| TRINITY_DN27264_c0_g1_i1_len_375_105402.p1_type_3prime_partial_len_116 | ---NA--- | | |
| TRINITY_DN23658_c0_g1_i1_len_905_161452.p1_type_complete_len_115 | ---NA--- | | |
| TRINITY_DN16352_c0_g1_i2_len_345_75061.p1_type_internal_len_116 | ---NA--- | | |
| TRINITY_DN12769_c0_g1_i3_len_346_172693.p1_type_internal_len_116 | ---NA--- | | |
| TRINITY_DN2554_c0_g1_i1_len_345_106843.p1_type_internal_len_116 | ---NA--- | | |
| TRINITY_DN41570_c0_g1_i1_len_425_206928.p1_type_complete_len_115 | ---NA--- | | |
| TRINITY_DN15653_c0_g1_i1_len_1760_123577.p2_type_3prime_partial_len_116 | ---NA--- | | |
| TRINITY_DN38956_c0_g1_i1_len_349_210803.p2_type_internal_len_116 | ---NA--- | | |
| TRINITY_DN340_c0_g1_i1_len_434_206552.p1_type_3prime_partial_len_116 | ---NA--- | | |
| TRINITY_DN26957_c0_g1_i3_len_1201_194959.p2_type_complete_len_115 | ---NA--- | | |
| TRINITY_DN23791_c0_g1_i2_len_825_198309.p1_type_5prime_partial_len_115 | ---NA--- | | |

| | | | |
|---|---|---|---|
| TRINITY_DN21689_c0_g1_i1_len_520_203648.p1_type_5prime_partial_len_115 | ---NA--- | | |
| TRINITY_DN36700_c0_g1_i1_len_346_211013.p1_type_internal_len_116 | ---NA--- | | |
| TRINITY_DN16555_c0_g1_i2_len_468_205283.p1_type_5prime_partial_len_115 | ---NA--- | | |
| TRINITY_DN7069_c0_g1_i1_len_431_277950.p1_type_5prime_partial_len_115 | ---NA--- | | |
| TRINITY_DN9834_c0_g1_i1_len_652_200766.p2_type_complete_len_115 | XP_018008597.1 39S ribosomal protein L42, mitochondrial-like | | |
| TRINITY_DN18557_c0_g1_i1_len_465_70118.p2_type_5prime_partial_len_115 | ---NA--- | | |
| TRINITY_DN10337_c0_g1_i1_len_377_171173.p1_type_5prime_partial_len_115 | ---NA--- | | |
| TRINITY_DN27925_c0_g1_i1_len_347_248957.p1_type_internal_len_116 | BBC20717.1integrin alpha ps | C:GO:0016021 | |
| TRINITY_DN12745_c0_g1_i1_len_370_280175.p1_type_5prime_partial_len_115 | PRD23668.1zinc finger protein | F:GO:0003676 | |
| TRINITY_DN19098_c0_g1_i1_len_584_241097.p1_type_complete_len_115 | ---NA--- | | |
| TRINITY_DN27652_c0_g1_i3_len_1692_192120.p2_type_complete_len_115 | ---NA--- | | |
| TRINITY_DN27328_c0_g1_i1_len_346_14098.p1_type_internal_len_116 | ---NA--- | | |
| TRINITY_DN31125_c0_g1_i1_len_641_240098.p2_type_complete_len_115 | ---NA--- | | |
| TRINITY_DN12625_c0_g1_i1_len_448_277417.p1_type_5prime_partial_len_115 | ABB73282.1reverse transcriptase | F:GO:0003964; P:GO:0006278 | |
| TRINITY_DN23061_c0_g1_i1_len_359_280689.p1_type_5prime_partial_len_115 | ---NA--- | | |
| TRINITY_DN727_c0_g1_i1_len_349_248855.p1_type_internal_len_116 | XP_018022200.1 sodium-independent sulfate anion transporter-like | P:GO:0008272; F:GO:0015116; C:GO:0016020; P:GO:0055085 | |

| | | | |
|---|---|---|---|
| TRINITY_DN33421_c0_g1_i1_len_349_210789.p1_type_3prime_partial_len_116 | ---NA--- | | |
| TRINITY_DN11712_c0_g1_i2_len_362_171863.p4_type_5prime_partial_len_115 | ---NA--- | | |
| TRINITY_DN3108_c0_g1_i1_len_347_106755.p1_type_internal_len_116 | ---NA--- | | |
| TRINITY_DN26338_c1_g1_i1_len_347_248946.p1_type_internal_len_116 | ---NA--- | | |
| 2561\|_\|c33207_f15p16_2557_isoform_c33207_full_length_coverage_15_non_full_length_coverage_16_isoform_length_2557_14204 | XP_018014547.1 FACT complex subunit Ssrp1-like | F:GO:0003677; C:GO:0005634; C:GO:0005694; P:GO:0006260; P:GO:0006281 | |
| 2562\|_\|c13899_f2p16_2557_isoform_c13899_full_length_coverage_2_non_full_length_coverage_16_isoform_length_2557_28640 | XP_018018310.1 long-chain-fatty-acid--CoA ligase ACSBG2-like isoform X2 | | |
| 2563\|_\|c4019_f2p3_2557_isoform_c4019_full_length_coverage_2_non_full_length_coverage_3_isoform_length_2557_16818 | ---NA--- | | |
| TRINITY_DN17198_c0_g1_i1_len_2994_264962.p1_type_complete_len_852 | XP_018013093.1 inactive rhomboid protein 1-like | F:GO:0004252; P:GO:0006508; C:GO:0016020; C:GO:0016021 | |
| TRINITY_DN30008_c0_g1_i1_len_4292_187426.p1_type_5prime_partial_len_852 | ---NA--- | | |
| TRINITY_DN22608_c0_g1_i2_len_2685_229456.p1_type_5prime_partial_len_852 | ---NA--- | | |
| 2568\|_\|c2629_f3p3_2556_isoform_c2629_full_length_coverage_3_non_full_length_coverage_3_isoform_length_2556_21100 | ---NA--- | | |
| 2569\|_\|c11586_f2p0_2556_isoform_c11586_full_length_coverage_2_non_full_length_coverage_0_isoform_length_2556_22066 | XP_018018626.1 lipoamide acyltransferase component of branched-chain alpha-keto acid dehydrogenase complex, mitochondrial-like | F:GO:0016746 | |

| | | | |
|---|---|---|---|
| 2570\|_\|c36198_f2p11_2555_isoform_c36198_full_length_coverage_2_non_full_length_coverage_11_isoform_length_2555_23567 | AWG41959.1glycogen synthase kinase 3 beta | F:GO:0004674; F:GO:0005524; P:GO:0006468 | EC:2.7.11 |
| 2571\|_\|c7555_f2p0_2555_isoform_c7555_full_length_coverage_2_non_full_length_coverage_0_isoform_length_2555_12642 | XP_018009489.1 beta-galactosidase-like | F:GO:0004565; C:GO:0005773; P:GO:0008152 | EC:3.2.1.23 |
| TRINITY_DN1881_c0_g1_i1_len_938_270637.p2_type_5prime_partial_len_115 | ---NA--- | | |
| TRINITY_DN33682_c0_g1_i1_len_346_74999.p1_type_internal_len_116 | ---NA--- | | |
| TRINITY_DN13694_c0_g1_i1_len_992_160732.p1_type_5prime_partial_len_115 | ---NA--- | | |
| TRINITY_DN4949_c0_g1_i2_len_347_106746.p2_type_internal_len_116 | ---NA--- | | |
| TRINITY_DN14667_c0_g1_i1_len_347_106745.p1_type_internal_len_116 | ---NA--- | | |
| TRINITY_DN25638_c0_g2_i1_len_346_249000.p1_type_internal_len_116 | XP_014358086.1 glycerol-3-phosphate acyltransferase 4 isoform X1 | | |
| TRINITY_DN22468_c0_g1_i1_len_2503_189441.p2_type_5prime_partial_len_115 | XP_022184534.1protein bicaudal D isoform X2 | | |
| TRINITY_DN26308_c0_g1_i1_len_811_198476.p1_type_3prime_partial_len_116 | ---NA--- | | |
| TRINITY_DN37233_c0_g1_i1_len_346_249003.p1_type_internal_len_116 | ---NA--- | | |
| TRINITY_DN22354_c0_g1_i1_len_359_308750.p1_type_5prime_partial_len_115 | ---NA--- | | |
| TRINITY_DN26552_c0_g1_i1_len_346_248997.p1_type_internal_len_116 | ---NA--- | | |
| TRINITY_DN22310_c0_g1_i1_len_1552_192818.p2_type_complete_len_115 | ---NA--- | | |
| TRINITY_DN7358_c0_g1_i2_len_746_199336.p1_type_complete_len_115 | ---NA--- | | |

| | | | |
|---|---|---|---|
| TRINITY_DN5845_c0_g1_i1_len_348_248898.p1_type_internal_len_116 | XP_022128176.1spectrin beta chain-like | F:GO:0003779 | |
| TRINITY_DN32674_c0_g1_i1_len_348_248911.p1_type_internal_len_116 | ---NA--- | | |
| TRINITY_DN28962_c0_g1_i1_len_347_74944.p1_type_internal_len_116 | XP_015522966.1 esterase E4-like | | |
| TRINITY_DN5907_c0_g1_i1_len_346_14079.p1_type_internal_len_116 | ---NA--- | | |
| TRINITY_DN9210_c0_g1_i1_len_345_211050.p1_type_internal_len_116 | XP_018019054.1 alpha-L-fucosidase-like | F:GO:0004560; P:GO:0006004 | EC:3.2.1.51 |
| TRINITY_DN41407_c0_g1_i1_len_518_203685.p1_type_5prime_partial_len_115 | ---NA--- | | |
| TRINITY_DN38746_c0_g1_i1_len_345_211029.p1_type_internal_len_116 | XP_018020695.1 zinc finger protein 813-like | | |
| TRINITY_DN21239_c0_g1_i1_len_437_40284.p2_type_5prime_partial_len_115 | ---NA--- | | |
| TRINITY_DN16262_c0_g1_i1_len_699_200006.p1_type_complete_len_115 | ---NA--- | | |
| TRINITY_DN24683_c0_g1_i1_len_2906_188684.p3_type_complete_len_115 | ---NA--- | | |
| TRINITY_DN10796_c0_g1_i2_len_554_165857.p1_type_5prime_partial_len_115 | ---NA--- | | |
| TRINITY_DN4045_c0_g1_i2_len_391_208469.p1_type_5prime_partial_len_115 | ---NA--- | | |
| TRINITY_DN26767_c0_g1_i1_len_489_276296.p1_type_5prime_partial_len_115 | ---NA--- | | |
| TRINITY_DN26988_c0_g1_i1_len_1523_192975.p1_type_complete_len_115 | ---NA--- | | |
| TRINITY_DN21066_c0_g1_i1_len_345_281364.p1_type_internal_len_116 | XP_014778830.1 rab GTPase-activating protein 1-like isoform X4 | F:GO:0005096; P:GO:0043547 | |
| TRINITY_DN2729_c0_g1_i1_len_345_211049.p2_type_internal_len_116 | ---NA--- | | |

| | | | |
|---|---|---|---|
| TRINITY_DN11688_c0_g1_i1_len_539_303408.p1_type_5prime_partial_len_115 | ---NA--- | | |
| TRINITY_DN10606_c0_g1_i1_len_546_166040.p1_type_5prime_partial_len_115 | ---NA--- | | |
| TRINITY_DN29052_c0_g1_i1_len_347_248968.p1_type_internal_len_116 | XP_025410476.1multidrug resistance-associated protein 1 isoform X1 | | |
| TRINITY_DN16701_c0_g1_i1_len_633_36498.p2_type_5prime_partial_len_115 | BAP28203.1copper/zinc superoxide dismutase isoform 4 | F:GO:0004784; P:GO:0019430; F:GO:0046872; P:GO:0055114 | EC:1.15.1.1 |
| TRINITY_DN34134_c0_g1_i1_len_571_202393.p1_type_5prime_partial_len_115 | ---NA--- | | |
| TRINITY_DN33150_c0_g1_i1_len_348_210872.p1_type_internal_len_116 | ---NA--- | | |
| TRINITY_DN37314_c0_g1_i1_len_346_248980.p1_type_internal_len_116 | ---NA--- | | |
| TRINITY_DN6207_c0_g1_i1_len_346_248992.p1_type_internal_len_116 | ---NA--- | | |
| TRINITY_DN35552_c0_g1_i1_len_346_248989.p1_type_3prime_partial_len_116 | ---NA--- | | |
| TRINITY_DN20226_c0_g1_i1_len_1521_60616.p4_type_3prime_partial_len_116 | ---NA--- | | |
| TRINITY_DN18997_c0_g1_i2_len_568_165567.p1_type_5prime_partial_len_115 | ---NA--- | | |
| TRINITY_DN32971_c0_g1_i1_len_347_248933.p1_type_internal_len_116 | XP_018018992.1 sulfotransferase 1 family member D1-like | | |
| TRINITY_DN19914_c0_g1_i1_len_709_199850.p1_type_5prime_partial_len_115 | ---NA--- | | |
| TRINITY_DN35605_c0_g1_i1_len_346_248976.p1_type_internal_len_116 | ---NA--- | | |
| TRINITY_DN29143_c1_g1_i1_len_812_198466.p1_type_complete_len_115 | ---NA--- | | |
| TRINITY_DN22523_c0_g1_i1_len_2334_122613.p2_type_3prime_partial_len_116 | ---NA--- | | |

| | | | |
|---|---|---|---|
| TRINITY_DN24393_c0_g1_i1_len_373_136575.p1_type_5prime_partial_len_115 | ---NA--- | | |
| TRINITY_DN8076_c0_g1_i1_len_346_248979.p1_type_internal_len_116 | ---NA--- | | |
| TRINITY_DN28513_c0_g1_i2_len_1373_193821.p4_type_complete_len_115 | ---NA--- | | |
| TRINITY_DN11671_c0_g1_i1_len_423_169323.p1_type_5prime_partial_len_115 | ---NA--- | | |
| TRINITY_DN18948_c0_g1_i1_len_404_135248.p1_type_5prime_partial_len_115 | ---NA--- | | |
| TRINITY_DN363_c0_g1_i1_len_455_205756.p1_type_3prime_partial_len_116 | ---NA--- | | |
| TRINITY_DN8421_c0_g1_i1_len_349_210831.p1_type_internal_len_116 | AIE15914.1argonaute 4 | F:GO:0003676; P:GO:0031047 | |
| TRINITY_DN28961_c0_g1_i1_len_347_137842.p1_type_internal_len_116 | ---NA--- | | |
| TRINITY_DN39230_c0_g1_i1_len_352_210644.p1_type_5prime_partial_len_115 | KFM67140.1Dedicator of cytokinesis protein 11 | F:GO:0005085; P:GO:0007264 | |
| TRINITY_DN33271_c0_g1_i1_len_346_211018.p1_type_internal_len_116 | XP_019865710.1 protein shifted-like isoform X3 | | |
| TRINITY_DN13499_c0_g1_i1_len_348_13969.p1_type_internal_len_116 | ---NA--- | | |
| TRINITY_DN12362_c0_g1_i1_len_348_210897.p1_type_internal_len_116 | ---NA--- | | |
| TRINITY_DN16075_c0_g1_i1_len_783_162577.p1_type_5prime_partial_len_115 | ---NA--- | | |
| TRINITY_DN17887_c0_g1_i1_len_347_137862.p1_type_internal_len_116 | ---NA--- | | |
| TRINITY_DN8914_c0_g1_i1_len_347_14022.p1_type_internal_len_116 | ---NA--- | | |
| TRINITY_DN27334_c0_g1_i2_len_945_197044.p2_type_complete_len_115 | ---NA--- | | |

| | | | |
|---|---|---|---|
| TRINITY_DN18248_c0_g1_i1_len_348_137815.p1_type_internal_len_116 | ACC86067.1anti-lipopolysaccharide factor | | |
| TRINITY_DN31466_c0_g1_i1_len_347_210903.p1_type_internal_len_116 | KDR24458.1Protein HEXIM | P:GO:0000122; F:GO:0004861; C:GO:0005634; C:GO:0005737; F:GO:0017069; P:GO:0045736 | |
| TRINITY_DN23716_c0_g1_i1_len_349_172533.p1_type_internal_len_116 | ---NA--- | | |
| TRINITY_DN14993_c0_g1_i3_len_693_5821.p5_type_3prime_partial_len_116 | ---NA--- | | |
| TRINITY_DN40967_c0_g1_i1_len_348_210899.p1_type_internal_len_116 | ---NA--- | | |
| TRINITY_DN5626_c0_g1_i1_len_480_204873.p1_type_5prime_partial_len_115 | AAC84145.1hyperglycemic hormone homolog PmSGP-IV precursor | F:GO:0005184; C:GO:0005576; P:GO:0006006; P:GO:0007218; P:GO:0010469 | |
| TRINITY_DN1110_c1_g1_i1_len_346_210988.p1_type_internal_len_116 | ---NA--- | | |
| TRINITY_DN27459_c0_g1_i1_len_348_137828.p2_type_internal_len_116 | ---NA--- | | |
| TRINITY_DN32294_c0_g1_i1_len_431_71191.p1_type_5prime_partial_len_115 | ---NA--- | | |
| TRINITY_DN16197_c0_g1_i1_len_1121_235237.p2_type_5prime_partial_len_115 | ---NA--- | | |
| TRINITY_DN23136_c0_g1_i2_len_728_199606.p1_type_3prime_partial_len_116 | ---NA--- | | |
| TRINITY_DN6608_c0_g1_i1_len_346_137927.p1_type_internal_len_116 | ---NA--- | | |
| TRINITY_DN36953_c0_g1_i1_len_347_210971.p1_type_internal_len_116 | ---NA--- | | |
| TRINITY_DN22296_c1_g1_i1_len_345_211048.p1_type_internal_len_116 | ---NA--- | | |
| TRINITY_DN33133_c0_g1_i1_len_456_205737.p1_type_5prime_partial_len_115 | ---NA--- | | |

| | | | |
|---|---|---|---|
| TRINITY_DN3121_c0_g1_i1_len_347_281239.p1_type_internal_len_116 | XP_018008896.1PREDICTED: uncharacterized protein LOC108666520 isoform X1 | | |
| TRINITY_DN31231_c0_g1_i1_len_346_137935.p1_type_internal_len_116 | XP_018010528.1 serine-rich adhesin for platelets-like | | |
| TRINITY_DN38792_c0_g1_i1_len_348_210873.p1_type_internal_len_116 | ---NA--- | | |
| TRINITY_DN6210_c0_g1_i1_len_392_12036.p1_type_5prime_partial_len_115 | ---NA--- | | |
| TRINITY_DN18925_c0_g1_i1_len_881_63917.p2_type_5prime_partial_len_115 | ---NA--- | | |
| TRINITY_DN31192_c0_g1_i1_len_365_73937.p1_type_5prime_partial_len_115 | ---NA--- | | |
| TRINITY_DN14904_c0_g1_i1_len_792_198711.p4_type_complete_len_115 | ---NA--- | | |
| TRINITY_DN21704_c0_g1_i1_len_1367_124749.p2_type_5prime_partial_len_115 | ---NA--- | | |
| TRINITY_DN336_c0_g1_i2_len_370_209543.p1_type_5prime_partial_len_115 | ---NA--- | | |
| TRINITY_DN7337_c0_g1_i1_len_347_210925.p1_type_internal_len_116 | XP_023222648.1GATOR complex protein NPRL3-like | | |
| TRINITY_DN24002_c0_g1_i1_len_348_172572.p1_type_internal_len_116 | ---NA--- | | |
| TRINITY_DN38095_c0_g1_i1_len_354_210465.p1_type_5prime_partial_len_115 | XP_018025890.1 piggyBac transposable element-derived protein 3-like | | |
| TRINITY_DN41785_c0_g1_i1_len_346_210981.p1_type_internal_len_116 | CEF34375.1Variant Ionotropic Glutamate Receptor | F:GO:0004970; C:GO:0016020; C:GO:0016021; P:GO:0035235 | |
| TRINITY_DN24418_c0_g1_i1_len_4216_187476.p2_type_complete_len_115 | ---NA--- | | |
| TRINITY_DN20677_c0_g1_i1_len_1484_157878.p2_type_3prime_partial_len_116 | XP_002732534.1 saccharopine dehydrogenase-like oxidoreductase-like | | |
| TRINITY_DN20313_c0_g1_i2_len_1485_124334.p3_type_3prime_partial_len_116 | ---NA--- | | |

| | | | |
|---|---|---|---|
| TRINITY_DN37349_c0_g1_i1_len_603_201718.p2_type_5prime_partial_len_115 | ---NA--- | | |
| TRINITY_DN27575_c0_g1_i1_len_1052_196089.p1_type_5prime_partial_len_115 | ---NA--- | | |
| TRINITY_DN18769_c0_g2_i2_len_347_43696.p1_type_internal_len_116 | ---NA--- | | |
| TRINITY_DN24313_c0_g1_i1_len_348_281181.p1_type_internal_len_116 | KMQ86077.1krab-a domain-containing protein | F:GO:0003676; P:GO:0015074; F:GO:0016787 | |
| TRINITY_DN12844_c0_g1_i2_len_347_43674.p3_type_internal_len_116 | ---NA--- | | |
| TRINITY_DN28556_c0_g1_i1_len_348_43650.p1_type_internal_len_116 | XP_018022161.1 histone-lysine N-methyltransferase, H3 lysine-79 specific-like isoform X2 | | |
| TRINITY_DN6363_c0_g1_i1_len_377_209164.p1_type_5prime_partial_len_115 | PNF30600.1zer-1-like protein | C:GO:0031462 | |
| TRINITY_DN4443_c0_g1_i1_len_347_43720.p1_type_internal_len_116 | ---NA--- | | |
| 2572\|_\|c4937_f3p3_2555_isoform_c4937_full_length_coverage_3_non_full_length_coverage_3_isoform_length_2555_16906 | ---NA--- | | |
| 2573\|_\|c5014_f2p0_2554_isoform_c5014_full_length_coverage_2_non_full_length_coverage_0_isoform_length_2554_12435 | XP_018013106.1 X-ray repair cross-complementing protein 6-like | F:GO:0003677; P:GO:0009987 | |
| 2574\|_\|c17798_f3p1_2554_isoform_c17798_full_length_coverage_3_non_full_length_coverage_1_isoform_length_2554_22496 | ---NA--- | | |
| 2575\|_\|c6662_f2p1_2554_isoform_c6662_full_length_coverage_2_non_full_length_coverage_1_isoform_length_2554_21623 | XP_018006560.1 tubulin polyglutamylase TTLL5-like | | |
| TRINITY_DN20471_c0_g1_i1_len_2610_265329.p1_type_5prime_partial_len_851 | XP_018016426.1 digestive organ expansion factor homolog | C:GO:0005634 | |
| TRINITY_DN29661_c0_g1_i1_len_2844_188785.p1_type_complete_len_851 | PNF27563.1Zinc finger CCCH domain-containing protein 10 | F:GO:0046872 | |

<table>
<tr><th></th><th></th><th></th><th></th></tr>
<tr><td>TRINITY_DN26027_c0_g1_i1_len_3115_228862.p1_type_5prime_partial_len_851</td><td>EFX76039.1hypothetical protein DAPPUDRAFT_322760</td><td>F:GO:0005515; P:GO:0010605; C:GO:0030687; C:GO:0030689; P:GO:0031324; C:GO:0031981; P:GO:0051172; P:GO:0080090</td><td></td></tr>
<tr><td>2579|_|c12582_f4p1_2553_isoform_c12582_full_length_coverage_4_non_full_length_coverage_1_isoform_length_2553_17527</td><td>XP_021931437.1heterogeneous nuclear ribonucleoprotein L isoform X1</td><td></td><td></td></tr>
<tr><td>TRINITY_DN28315_c0_g1_i2_len_2585_189256.p1_type_5prime_partial_len_851</td><td>AHH32888.1integrin alpha 5</td><td>P:GO:0007155; P:GO:0007229; C:GO:0008305</td><td></td></tr>
<tr><td>TRINITY_DN30730_c0_g1_i2_len_4893_187161.p1_type_complete_len_851</td><td>PNF16632.1ARF GTPase-activating protein GIT2</td><td>F:GO:0005096; P:GO:0043547; F:GO:0046872</td><td></td></tr>
<tr><td>TRINITY_DN21910_c1_g1_i1_len_494_204437.p1_type_5prime_partial_len_115</td><td>---NA---</td><td></td><td></td></tr>
<tr><td>TRINITY_DN22135_c0_g1_i4_len_2298_230250.p4_type_5prime_partial_len_115</td><td>---NA---</td><td></td><td></td></tr>
<tr><td>TRINITY_DN8731_c0_g1_i1_len_384_12349.p1_type_5prime_partial_len_115</td><td>---NA---</td><td></td><td></td></tr>
<tr><td>TRINITY_DN10569_c0_g1_i1_len_348_43657.p1_type_internal_len_116</td><td>---NA---</td><td></td><td></td></tr>
<tr><td>TRINITY_DN17519_c0_g1_i1_len_1461_124413.p2_type_5prime_partial_len_115</td><td>---NA---</td><td></td><td></td></tr>
<tr><td>TRINITY_DN23145_c0_g1_i1_len_1657_192271.p1_type_complete_len_115</td><td>---NA---</td><td></td><td></td></tr>
<tr><td>TRINITY_DN30242_c0_g1_i1_len_347_14012.p1_type_internal_len_116</td><td>---NA---</td><td></td><td></td></tr>
<tr><td>TRINITY_DN1165_c0_g1_i1_len_345_211034.p1_type_internal_len_116</td><td>---NA---</td><td></td><td></td></tr>
<tr><td>TRINITY_DN26505_c0_g1_i1_len_348_137781.p1_type_internal_len_116</td><td>XP_022245126.1ras-related and estrogen-regulated growth inhibitor-like isoform X1</td><td>F:GO:0000166</td><td></td></tr>
<tr><td>TRINITY_DN41893_c0_g1_i1_len_347_210911.p1_type_internal_len_116</td><td>---NA---</td><td></td><td></td></tr>
<tr><td>TRINITY_DN6798_c0_g1_i1_len_368_171601.p1_type_5prime_partial_len_115</td><td>AEK86518.1IKKbeta</td><td>F:GO:0004672; F:GO:0005524; P:GO:0006468</td><td></td></tr>
</table>

| | | | |
|---|---|---|---|
| TRINITY_DN18388_c0_g1_i1_len_370_308249.p1_type_5prime_partial_len_115 | ---NA--- | | |
| TRINITY_DN30515_c0_g1_i1_len_2748_188957.p3_type_complete_len_115 | ---NA--- | | |
| TRINITY_DN5236_c0_g1_i1_len_348_281204.p1_type_internal_len_116 | XP_010141949.1 lysophospholipase-like protein 1 | F:GO:0016787 | |
| TRINITY_DN40338_c0_g1_i1_len_347_210962.p1_type_internal_len_116 | AYF59256.1toll 3 | P:GO:0007165; C:GO:0016021; P:GO:0045087 | |
| TRINITY_DN21125_c0_g1_i1_len_346_211016.p1_type_internal_len_116 | ---NA--- | | |
| TRINITY_DN9480_c0_g1_i1_len_671_273142.p1_type_5prime_partial_len_115 | ---NA--- | | |
| TRINITY_DN12965_c0_g1_i1_len_347_210914.p1_type_internal_len_116 | APP91326.1retinoid X receptor | F:GO:0003700; F:GO:0003707; C:GO:0005634; P:GO:0006355; F:GO:0008270; P:GO:0043401; F:GO:0043565 | |
| TRINITY_DN39731_c0_g1_i1_len_348_210890.p1_type_internal_len_116 | XP_015517234.1 hemicentin-2-like | | |
| TRINITY_DN6603_c0_g1_i2_len_347_172637.p1_type_internal_len_116 | ---NA--- | | |
| TRINITY_DN25491_c0_g1_i1_len_1998_190910.p2_type_3prime_partial_len_116 | ---NA--- | | |
| TRINITY_DN2800_c0_g1_i1_len_347_210967.p1_type_internal_len_116 | XP_018022161.1 histone-lysine N-methyltransferase, H3 lysine-79 specific-like isoform X2 | | |
| TRINITY_DN12340_c0_g1_i1_len_347_172616.p1_type_internal_len_116 | ---NA--- | | |
| TRINITY_DN16262_c1_g1_i1_len_435_134097.p2_type_5prime_partial_len_115 | ---NA--- | | |
| TRINITY_DN23908_c0_g1_i1_len_380_105196.p1_type_5prime_partial_len_115 | XP_018010131.1 peroxidase-like | F:GO:0004601; P:GO:0006979; F:GO:0020037; P:GO:0055114; P:GO:0098869 | |

| | | | |
|---|---|---|---|
| TRINITY_DN27865_c0_g1_i1_len_1137_195402.p2_type_3prime_partial_len_116 | ---NA--- | | |
| TRINITY_DN24735_c0_g1_i1_len_347_172613.p1_type_internal_len_116 | ---NA--- | | |
| TRINITY_DN37563_c0_g1_i1_len_346_210980.p1_type_internal_len_116 | XP_026808524.1carboxypeptidase B-like | F:GO:0004180; F:GO:0004181; P:GO:0006508; F:GO:0008270 | |
| TRINITY_DN28172_c0_g1_i1_len_361_43070.p1_type_5prime_partial_len_115 | ---NA--- | | |
| TRINITY_DN19255_c0_g1_i2_len_1646_267255.p1_type_5prime_partial_len_115 | ---NA--- | | |
| TRINITY_DN30104_c0_g1_i1_len_346_309382.p1_type_internal_len_116 | XP_018013069.1 transient receptor potential protein-like | F:GO:0005262; C:GO:0016021; P:GO:0070588 | |
| TRINITY_DN28352_c0_g1_i1_len_346_281288.p1_type_internal_len_116 | ---NA--- | | |
| TRINITY_DN18953_c0_g1_i2_len_625_164595.p1_type_3prime_partial_len_116 | ---NA--- | | |
| TRINITY_DN24520_c0_g1_i4_len_1764_59882.p2_type_complete_len_115 | XP_018012091.1 protein FAM50A-like isoform X2 | C:GO:0005634; C:GO:0016020; F:GO:0017154; P:GO:0071526 | |
| TRINITY_DN4824_c0_g1_i1_len_543_241940.p1_type_5prime_partial_len_115 | ---NA--- | | |
| TRINITY_DN8628_c0_g1_i1_len_346_172663.p1_type_internal_len_116 | ---NA--- | | |
| TRINITY_DN5006_c0_g1_i1_len_346_281275.p1_type_internal_len_116 | XP_018016507.1PREDICTED: uncharacterized protein LOC108673227 | F:GO:0003824; P:GO:0009116 | |
| TRINITY_DN16377_c0_g1_i1_len_506_8698.p1_type_5prime_partial_len_115 | ---NA--- | | |
| TRINITY_DN31898_c0_g1_i1_len_347_172648.p1_type_internal_len_116 | ---NA--- | | |
| TRINITY_DN28668_c0_g1_i1_len_347_14038.p1_type_internal_len_116 | ---NA--- | | |
| TRINITY_DN6347_c0_g1_i1_len_347_281243.p2_type_internal_len_116 | XP_018014164.1 bestrophin-3-like | | |

| | | | |
|---|---|---|---|
| TRINITY_DN30012_c0_g1_i1_len_359_248300.p1_type_5prime_partial_len_115 | ---NA--- | | |
| TRINITY_DN15553_c0_g1_i2_len_349_13952.p1_type_internal_len_116 | ---NA--- | | |
| TRINITY_DN20785_c0_g1_i1_len_1558_232733.p5_type_5prime_partial_len_115 | ---NA--- | | |
| TRINITY_DN9870_c0_g1_i1_len_456_244353.p1_type_5prime_partial_len_115 | ---NA--- | | |
| TRINITY_DN34360_c0_g1_i1_len_345_137942.p1_type_internal_len_116 | XP_018010158.1 protein mab-21-like 1 | | |
| TRINITY_DN17389_c0_g1_i2_len_455_244377.p1_type_5prime_partial_len_115 | ---NA--- | | |
| TRINITY_DN21479_c1_g1_i1_len_461_9795.p2_type_5prime_partial_len_115 | ---NA--- | | |
| TRINITY_DN25226_c0_g1_i1_len_390_135806.p1_type_5prime_partial_len_115 | ---NA--- | | |
| TRINITY_DN1118_c0_g1_i1_len_493_167213.p2_type_5prime_partial_len_114 | ACY64754.1double WAP domain-containing protein | C:GO:0005576; P:GO:0010466; F:GO:0030414 | |
| TRINITY_DN20897_c1_g1_i1_len_372_209401.p1_type_5prime_partial_len_114 | ---NA--- | | |
| TRINITY_DN23796_c0_g1_i1_len_343_172836.p1_type_internal_len_115 | ---NA--- | | |
| TRINITY_DN27351_c0_g1_i1_len_392_72696.p1_type_3prime_partial_len_115 | ---NA--- | | |
| TRINITY_DN17490_c0_g1_i1_len_757_300533.p1_type_complete_len_114 | XP_018025283.1 DNA polymerase epsilon subunit 4-like | C:GO:0008622; F:GO:0046982 | |
| TRINITY_DN14719_c0_g1_i1_len_933_236680.p1_type_complete_len_114 | ---NA--- | | |
| TRINITY_DN18863_c0_g1_i1_len_1832_231632.p2_type_complete_len_114 | ---NA--- | | |
| TRINITY_DN15031_c0_g1_i1_len_909_197393.p2_type_3prime_partial_len_115 | ---NA--- | | |

| | | | |
|---|---|---|---|
| TRINITY_DN20387_c0_g2_i1_len_419_40881.p1_type_5prime_partial_len_114 | ---NA--- | | |
| TRINITY_DN1705_c0_g1_i1_len_472_167802.p1_type_5prime_partial_len_114 | ---NA--- | | |
| TRINITY_DN5008_c0_g1_i1_len_343_172854.p2_type_internal_len_115 | KZS05717.1Opsin Rh6 | P:GO:0007165 | |
| TRINITY_DN20486_c1_g1_i1_len_3746_264587.p2_type_complete_len_114 | EEB20500.1ubiquitously transcribed X chromosome tetratricopeptide repeat protein, putative | P:GO:0010468; F:GO:0046872; P:GO:0071557; F:GO:0071558 | |
| TRINITY_DN16335_c0_g1_i1_len_439_168797.p1_type_5prime_partial_len_114 | ---NA--- | | |
| TRINITY_DN1003_c0_g1_i1_len_661_273261.p1_type_complete_len_114 | ---NA--- | | |
| TRINITY_DN26507_c0_g1_i4_len_1806_191640.p5_type_complete_len_114 | ---NA--- | | |
| TRINITY_DN30098_c0_g1_i1_len_931_197174.p1_type_complete_len_114 | ---NA--- | | |
| TRINITY_DN19298_c0_g1_i1_len_1815_231687.p4_type_5prime_partial_len_114 | ---NA--- | | |
| TRINITY_DN27422_c0_g1_i1_len_1054_196078.p1_type_5prime_partial_len_114 | ---NA--- | | |
| TRINITY_DN2021_c0_g1_i1_len_820_162211.p1_type_complete_len_114 | ---NA--- | | |
| TRINITY_DN28255_c0_g1_i1_len_467_167969.p1_type_5prime_partial_len_114 | ---NA--- | | |
| TRINITY_DN24863_c0_g1_i2_len_931_197176.p2_type_complete_len_114 | ---NA--- | | |
| TRINITY_DN8507_c0_g1_i1_len_597_37001.p1_type_complete_len_114 | ---NA--- | | |
| TRINITY_DN16985_c0_g1_i1_len_401_307002.p1_type_5prime_partial_len_114 | ---NA--- | | |
| TRINITY_DN34190_c0_g1_i1_len_346_172667.p1_type_internal_len_115 | XP_018025541.1 glutaminase liver isoform, mitochondrial-like | F:GO:0004359; P:GO:0006541 | |

| | | | |
|---|---|---|---|
| TRINITY_DN18770_c0_g1_i1_len_1468_124394.p2_type_complete_len_114 | ---NA--- | | |
| TRINITY_DN26231_c0_g1_i1_len_1639_192359.p2_type_complete_len_114 | ---NA--- | | |
| TRINITY_DN24108_c0_g1_i2_len_3156_122041.p4_type_complete_len_114 | ---NA--- | | |
| TRINITY_DN14026_c0_g1_i1_len_1014_235999.p2_type_5prime_partial_len_114 | XP_018011898.1 JNK-interacting protein 1-like | | |
| TRINITY_DN29800_c0_g1_i2_len_1864_191419.p2_type_complete_len_114 | ---NA--- | | |
| TRINITY_DN26221_c0_g1_i1_len_531_8176.p1_type_5prime_partial_len_114 | ---NA--- | | |
| TRINITY_DN36692_c0_g1_i1_len_826_237683.p1_type_5prime_partial_len_114 | ---NA--- | | |
| TRINITY_DN21549_c0_g1_i5_len_1184_125491.p1_type_5prime_partial_len_114 | ---NA--- | | |
| TRINITY_DN22215_c0_g1_i1_len_1055_235682.p1_type_complete_len_114 | ---NA--- | | |
| TRINITY_DN13250_c0_g1_i1_len_1272_268723.p2_type_complete_len_114 | XP_023231630.1bifunctional coenzyme A synthase-like | F:GO:0003824; P:GO:0006796; P:GO:0009058 | |
| TRINITY_DN12973_c0_g1_i1_len_841_162008.p2_type_3prime_partial_len_115 | ---NA--- | | |
| TRINITY_DN19958_c0_g1_i2_len_1567_232697.p2_type_3prime_partial_len_115 | ---NA--- | | |
| TRINITY_DN27945_c0_g1_i1_len_343_172820.p1_type_internal_len_115 | ---NA--- | | |
| TRINITY_DN13775_c0_g1_i2_len_577_274520.p1_type_5prime_partial_len_114 | ---NA--- | | |
| TRINITY_DN10411_c0_g1_i1_len_388_72849.p1_type_5prime_partial_len_114 | ---NA--- | | |
| TRINITY_DN13920_c0_g1_i1_len_380_209010.p1_type_5prime_partial_len_114 | PSN41146.1Death-associated protein kinase 1 | F:GO:0016301; P:GO:0016310 | |

| | | | |
|---|---|---|---|
| TRINITY_DN19426_c0_g1_i1_len_388_72855.p1_type_5prime_partial_len_114 | ---NA--- | | |
| TRINITY_DN10085_c0_g1_i1_len_664_164002.p1_type_complete_len_114 | ---NA--- | | |
| TRINITY_DN20811_c0_g1_i2_len_379_171096.p1_type_5prime_partial_len_114 | ---NA--- | | |
| TRINITY_DN2289_c0_g1_i1_len_444_168633.p2_type_5prime_partial_len_114 | ---NA--- | | |
| TRINITY_DN19855_c0_g1_i1_len_639_201024.p1_type_5prime_partial_len_114 | ---NA--- | | |
| TRINITY_DN28326_c0_g1_i1_len_1854_191467.p1_type_complete_len_114 | ---NA--- | | |
| TRINITY_DN12519_c0_g1_i1_len_429_206763.p1_type_5prime_partial_len_114 | ---NA--- | | |
| TRINITY_DN12392_c0_g1_i2_len_345_172718.p1_type_internal_len_115 | ---NA--- | | |
| TRINITY_DN22103_c0_g1_i9_len_2211_91803.p3_type_complete_len_114 | XP_023953356.1beta-glucuronidase-like, partial | | |
| TRINITY_DN17826_c0_g1_i2_len_2633_58438.p2_type_5prime_partial_len_114 | ---NA--- | | |
| TRINITY_DN1668_c0_g1_i1_len_1008_160611.p1_type_complete_len_114 | ---NA--- | | |
| TRINITY_DN26845_c0_g1_i1_len_2747_188960.p1_type_complete_len_851 | XP_023723000.1DNA-binding protein RFX2-like isoform X3 | | |
| 2584\|_\|c2228_f12p1_2552_isoform_c2228_full_length_coverage_12_non_full_length_coverage_1_isoform_length_2552_20793 | XP_018016915.1 condensin-2 complex subunit H2-like isoform X2 | | |
| 2585\|_\|c9019_f3p2_2552_isoform_c9019_full_length_coverage_3_non_full_length_coverage_2_isoform_length_2552_17244 | XP_019872910.1 importin-4-like | | |
| 2586\|_\|c5546_f2p0_2551_isoform_c5546_full_length_coverage_2_non_full_length_coverage_0_isoform_length_2551_21535 | XP_012529444.1 probable ATP-dependent RNA helicase DHX34 | F:GO:0003676; F:GO:0004386; F:GO:0005524 | EC:3.6.1.15 |

| | | | |
|---|---|---|---|
| 2588\|_\|c1993_f3p6_2551_isoform_c1993_full_length_coverage_3_non_full_length_coverage_6_isoform_length_2551_16110 | ---NA--- | | |
| 2589\|_\|c8624_f2p3_2551_isoform_c8624_full_length_coverage_2_non_full_length_coverage_3_isoform_length_2551_17214 | ---NA--- | | |
| 2590\|_\|c7248_f3p2_2551_isoform_c7248_full_length_coverage_3_non_full_length_coverage_2_isoform_length_2551_25833 | ---NA--- | | |
| 2591\|_\|c2980_f2p0_2550_isoform_c2980_full_length_coverage_2_non_full_length_coverage_0_isoform_length_2550_16729 | ---NA--- | | |
| 2592\|_\|c2514_f3p0_2550_isoform_c2514_full_length_coverage_3_non_full_length_coverage_0_isoform_length_2550_21004 | ---NA--- | | |
| 2594\|_\|c5934_f4p1_2550_isoform_c5934_full_length_coverage_4_non_full_length_coverage_1_isoform_length_2550_12513 | ---NA--- | | |
| TRINITY_DN17178_c0_g1_i2_len_1641_60219.p2_type_complete_len_114 | XP_018026166.1 probable phospholipid-transporting ATPase IIA | | |
| TRINITY_DN12965_c0_g1_i2_len_1131_235175.p2_type_3prime_partial_len_115 | ---NA--- | | |
| TRINITY_DN25915_c0_g1_i1_len_362_171884.p1_type_5prime_partial_len_114 | XP_018026815.1 geranylgeranyl transferase type-1 subunit beta-like | F:GO:0003824 | |
| TRINITY_DN6100_c0_g1_i1_len_841_237538.p2_type_complete_len_114 | ---NA--- | | |
| TRINITY_DN22593_c0_g1_i2_len_1225_194772.p2_type_5prime_partial_len_114 | ---NA--- | | |
| TRINITY_DN41438_c0_g1_i1_len_377_209179.p1_type_5prime_partial_len_114 | ---NA--- | | |
| TRINITY_DN30585_c0_g1_i1_len_342_172856.p1_type_internal_len_115 | ---NA--- | | |

| | | | |
|---|---|---|---|
| TRINITY_DN19498_c0_g1_i1_len_972_196778.p1_type_5prime_partial_len_114 | ---NA--- | | |
| TRINITY_DN33964_c0_g1_i1_len_346_172708.p1_type_internal_len_115 | ---NA--- | | |
| TRINITY_DN12888_c0_g1_i1_len_1213_234620.p1_type_5prime_partial_len_114 | ---NA--- | | |
| TRINITY_DN32712_c0_g1_i1_len_725_238832.p1_type_5prime_partial_len_114 | ---NA--- | | |
| TRINITY_DN9842_c0_g1_i1_len_410_306658.p1_type_3prime_partial_len_115 | ---NA--- | | |
| TRINITY_DN18582_c0_g3_i1_len_637_273603.p1_type_5prime_partial_len_114 | ---NA--- | | |
| TRINITY_DN4938_c0_g1_i2_len_405_170022.p1_type_5prime_partial_len_114 | ---NA--- | | |
| TRINITY_DN17855_c0_g1_i1_len_948_236542.p1_type_3prime_partial_len_115 | ---NA--- | | |
| TRINITY_DN22249_c0_g1_i1_len_980_196721.p2_type_complete_len_114 | ---NA--- | | |
| TRINITY_DN21721_c0_g1_i1_len_548_202927.p1_type_5prime_partial_len_114 | ---NA--- | | |
| TRINITY_DN32211_c0_g1_i1_len_344_14195.p1_type_internal_len_115 | XP_018022904.1 putative glutamate synthase [NADPH] | F:GO:0005506; P:GO:0006537; F:GO:0010181; F:GO:0016040; F:GO:0050660; F:GO:0051538; P:GO:0055114 | EC:1.4.1.14 |
| TRINITY_DN11729_c0_g1_i1_len_346_106810.p1_type_internal_len_115 | ---NA--- | | |
| TRINITY_DN5975_c0_g1_i1_len_542_131444.p2_type_5prime_partial_len_114 | ---NA--- | | |
| TRINITY_DN32350_c0_g1_i1_len_346_106791.p1_type_internal_len_115 | ---NA--- | | |
| TRINITY_DN22491_c0_g1_i1_len_346_106807.p1_type_internal_len_115 | XP_018016353.1 interaptin-like isoform X2 | | |

| | | | |
|---|---|---|---|
| TRINITY_DN11254_c0_g1_i2_len_921_63622.p2_type_5prime_partial_len_114 | ---NA--- | | |
| TRINITY_DN31418_c0_g1_i1_len_344_138027.p2_type_internal_len_115 | ---NA--- | | |
| TRINITY_DN20861_c1_g1_i1_len_1630_267302.p1_type_5prime_partial_len_114 | ---NA--- | | |
| TRINITY_DN28007_c0_g1_i1_len_345_14170.p1_type_internal_len_115 | ---NA--- | | |
| TRINITY_DN29738_c0_g1_i1_len_344_138042.p1_type_3prime_partial_len_115 | XP_021934029.1kelch-like ECH-associated protein 1 isoform X8 | | |
| TRINITY_DN412_c0_g1_i1_len_344_309522.p1_type_internal_len_115 | ---NA--- | | |
| TRINITY_DN30640_c0_g1_i1_len_1086_298344.p2_type_5prime_partial_len_114 | ---NA--- | | |
| TRINITY_DN8704_c0_g1_i1_len_925_96095.p1_type_5prime_partial_len_114 | ---NA--- | | |
| TRINITY_DN22790_c0_g1_i1_len_345_137968.p2_type_internal_len_115 | ---NA--- | | |
| TRINITY_DN12654_c0_g1_i2_len_344_106934.p1_type_internal_len_115 | ---NA--- | | |
| TRINITY_DN7218_c0_g1_i1_len_343_106995.p1_type_5prime_partial_len_114 | ---NA--- | | |
| TRINITY_DN27774_c0_g1_i1_len_343_309549.p1_type_internal_len_115 | XP_018015398.1 facilitated trehalose transporter Tret1-like isoform X2 | | |
| TRINITY_DN6399_c0_g1_i1_len_343_106974.p1_type_internal_len_115 | ---NA--- | | |
| TRINITY_DN27693_c0_g1_i1_len_345_137962.p1_type_internal_len_115 | XP_012590373.1 formimidoyltransferase-cyclodeaminase-like | | |
| TRINITY_DN33403_c0_g1_i1_len_345_106878.p1_type_internal_len_115 | ---NA--- | | |
| TRINITY_DN29315_c0_g1_i1_len_344_106883.p1_type_internal_len_115 | ---NA--- | | |

| | | | |
|---|---|---|---|
| TRINITY_DN22883_c0_g1_i1_len_345_106870.p1_type_internal_len_115 | APC26113.1metabotropic glutamate receptor type 7 | F:GO:0001642; C:GO:0005887; P:GO:0007196; C:GO:0042734; P:GO:0051966 | |
| TRINITY_DN23992_c0_g1_i1_len_345_106876.p1_type_internal_len_115 | ---NA--- | | |
| TRINITY_DN16052_c0_g1_i2_len_344_309506.p1_type_internal_len_115 | ---NA--- | | |
| TRINITY_DN24808_c0_g1_i1_len_604_67031.p1_type_5prime_partial_len_114 | ---NA--- | | |
| TRINITY_DN40602_c0_g1_i1_len_344_211093.p1_type_internal_len_115 | ---NA--- | | |
| TRINITY_DN8510_c0_g1_i1_len_344_75110.p1_type_internal_len_115 | ---NA--- | | |
| TRINITY_DN27118_c0_g1_i1_len_494_132523.p1_type_3prime_partial_len_115 | ---NA--- | | |
| TRINITY_DN33349_c0_g1_i1_len_345_75048.p1_type_internal_len_115 | ---NA--- | | |
| TRINITY_DN38493_c0_g1_i1_len_345_211080.p1_type_internal_len_115 | XP_015367706.1 kinesin-like protein unc-104 isoform X3 | | |
| TRINITY_DN37895_c0_g1_i1_len_345_211081.p1_type_internal_len_115 | ---NA--- | | |
| TRINITY_DN30319_c0_g1_i1_len_345_75038.p2_type_internal_len_115 | ---NA--- | | |
| TRINITY_DN26283_c0_g1_i1_len_344_75077.p1_type_internal_len_115 | ---NA--- | | |
| TRINITY_DN18177_c0_g2_i1_len_548_241826.p1_type_5prime_partial_len_114 | ---NA--- | | |
| TRINITY_DN10087_c0_g1_i1_len_683_200266.p1_type_complete_len_114 | ---NA--- | | |
| TRINITY_DN26227_c0_g1_i1_len_380_136228.p2_type_5prime_partial_len_114 | XP_018027633.1 carbohydrate sulfotransferase 1-like | | |
| TRINITY_DN7979_c0_g1_i1_len_367_247908.p1_type_5prime_partial_len_114 | ---NA--- | | |

| | | | |
|---|---|---|---|
| TRINITY_DN34926_c0_g1_i1_len_343_75142.p1_type_internal_len_115 | XP_018026155.1 mediator of RNA polymerase II transcription subunit 13-like | | |
| TRINITY_DN34627_c0_g1_i1_len_343_75179.p1_type_internal_len_115 | ---NA--- | | |
| TRINITY_DN11730_c0_g1_i1_len_345_211083.p1_type_internal_len_115 | ---NA--- | | |
| TRINITY_DN9428_c0_g1_i1_len_345_14123.p1_type_internal_len_115 | ---NA--- | | |
| TRINITY_DN29477_c0_g1_i1_len_344_138054.p1_type_internal_len_115 | ---NA--- | | |
| TRINITY_DN22460_c0_g1_i1_len_2353_189812.p3_type_complete_len_114 | ---NA--- | | |
| TRINITY_DN20674_c0_g1_i2_len_345_211084.p1_type_internal_len_115 | ---NA--- | | |
| TRINITY_DN29625_c0_g1_i1_len_345_75019.p1_type_internal_len_115 | XP_018012905.1 death-associated protein kinase 3-like | F:GO:0004674; F:GO:0005524; P:GO:0006468 | EC:2.7.11 |
| TRINITY_DN40072_c0_g1_i1_len_345_211082.p2_type_internal_len_115 | ---NA--- | | |
| TRINITY_DN33759_c0_g1_i1_len_378_247354.p1_type_5prime_partial_len_114 | ---NA--- | | |
| TRINITY_DN33260_c0_g1_i1_len_345_75009.p2_type_internal_len_115 | PNF20836.1hypothetical protein B7P43_G12063, partial | F:GO:0004114; P:GO:0007165; F:GO:0046872 | EC:3.1.4.17 |
| TRINITY_DN4809_c0_g1_i1_len_346_14108.p1_type_internal_len_115 | ---NA--- | | |
| TRINITY_DN21577_c0_g1_i1_len_343_138074.p1_type_internal_len_115 | PRD27860.1zinc finger protein | F:GO:0003676 | |
| TRINITY_DN3789_c0_g1_i1_len_346_74991.p1_type_internal_len_115 | ---NA--- | | |
| TRINITY_DN36392_c0_g1_i1_len_401_207974.p1_type_3prime_partial_len_115 | ---NA--- | | |
| TRINITY_DN20444_c0_g1_i1_len_351_43505.p1_type_5prime_partial_len_114 | ---NA--- | | |

| | | | |
|---|---|---|---|
| TRINITY_DN39952_c0_g1_i1_len_343_211158.p1_type_internal_len_115 | XP_018012343.1 dystonin-like isoform X1 | C:GO:0005856; P:GO:0007010; F:GO:0008092 | |
| TRINITY_DN33447_c0_g1_i1_len_344_211146.p3_type_internal_len_115 | ---NA--- | | |
| TRINITY_DN41207_c0_g1_i1_len_344_211149.p1_type_internal_len_115 | XP_018012588.1 fat-like cadherin-related tumor suppressor homolog | | |
| TRINITY_DN30718_c0_g1_i1_len_344_43863.p1_type_internal_len_115 | ---NA--- | | |
| TRINITY_DN2823_c0_g1_i2_len_344_43858.p2_type_internal_len_115 | ---NA--- | | |
| TRINITY_DN28495_c0_g1_i1_len_486_243402.p1_type_5prime_partial_len_114 | ---NA--- | | |
| TRINITY_DN9699_c0_g1_i1_len_343_43908.p1_type_internal_len_115 | ---NA--- | | |
| TRINITY_DN40076_c0_g1_i1_len_344_211145.p1_type_internal_len_115 | XP_018021126.1 inositol hexakisphosphate and diphosphoinositol-pentakisphosphate kinase-like | | |
| TRINITY_DN41559_c0_g1_i1_len_343_211206.p1_type_internal_len_115 | XP_021954740.1prolyl 4-hydroxylase subunit alpha-2-like isoform X2 | F:GO:0016491; F:GO:0043167 | |
| TRINITY_DN4046_c0_g1_i1_len_343_211208.p1_type_internal_len_115 | XP_015517131.1 ubiquitin-protein ligase E3B | | |
| TRINITY_DN31696_c0_g1_i1_len_342_211272.p1_type_internal_len_115 | XP_023708308.1RNA pseudouridylate synthase domain-containing protein 2-like | P:GO:0001522; F:GO:0003723; F:GO:0009982 | |
| TRINITY_DN39901_c0_g1_i1_len_343_211186.p1_type_internal_len_115 | XP_018008738.1 guanine nucleotide exchange factor subunit Rich-like | | |
| TRINITY_DN8113_c0_g1_i2_len_343_211194.p1_type_internal_len_115 | ---NA--- | | |
| TRINITY_DN11089_c0_g1_i1_len_343_211196.p1_type_internal_len_115 | ---NA--- | | |
| TRINITY_DN34337_c0_g1_i1_len_343_211200.p1_type_5prime_partial_len_114 | ---NA--- | | |
| TRINITY_DN40407_c0_g1_i1_len_343_211203.p1_type_internal_len_115 | ---NA--- | | |

| | | | |
|---|---|---|---|
| TRINITY_DN9288_c0_g1_i1_len_505_242897.p1_type_5prime_partial_len_114 | ---NA--- | | |
| TRINITY_DN34626_c0_g1_i1_len_342_211254.p1_type_internal_len_115 | KOF80876.1hypothetical protein OCBIM_22027274mg | F:GO:0046872 | |
| TRINITY_DN8244_c0_g1_i1_len_342_211237.p1_type_internal_len_115 | ---NA--- | | |
| TRINITY_DN30095_c0_g1_i1_len_4159_187500.p4_type_complete_len_114 | ---NA--- | | |
| TRINITY_DN40413_c0_g1_i1_len_342_211241.p1_type_internal_len_115 | ---NA--- | | |
| TRINITY_DN21962_c0_g2_i2_len_707_5656.p1_type_3prime_partial_len_115 | XP_018023185.1 longitudinals lacking protein, isoforms H/M/V-like isoform X9 | | |
| TRINITY_DN34428_c0_g1_i1_len_342_211256.p1_type_internal_len_115 | XP_018011893.1 dual specificity protein phosphatase 10-like | | |
| TRINITY_DN35160_c0_g1_i1_len_343_211162.p1_type_internal_len_115 | XP_026758542.1ATP-binding cassette sub-family A member 5-like | F:GO:0005524; C:GO:0016021; F:GO:0042626; P:GO:0055085 | EC:3.6.1.3; EC:3.6.1.15 |
| TRINITY_DN16051_c0_g1_i2_len_382_12425.p1_type_3prime_partial_len_115 | ---NA--- | | |
| TRINITY_DN15483_c0_g1_i2_len_345_43826.p1_type_internal_len_115 | ---NA--- | | |
| TRINITY_DN39985_c0_g1_i1_len_343_211167.p1_type_internal_len_115 | XP_018023096.1 PQ-loop repeat-containing protein 1-like | | |
| TRINITY_DN42313_c0_g1_i1_len_343_211177.p1_type_internal_len_115 | ---NA--- | | |
| TRINITY_DN8489_c0_g1_i1_len_343_211182.p1_type_internal_len_115 | XP_018024466.1PREDICTED: uncharacterized protein LOC108680203 | | |
| TRINITY_DN39309_c0_g1_i1_len_343_211184.p1_type_internal_len_115 | XP_012262835.1dedicator of cytokinesis protein 9 isoform X5 | F:GO:0005085; P:GO:0007264 | |
| TRINITY_DN28955_c0_g1_i4_len_6084_186922.p1_type_complete_len_850 | AJW83244.1dynamin superfamily protein | F:GO:0003924; F:GO:0005525 | EC:3.6.1.15 |
| TRINITY_DN30500_c0_g1_i2_len_3057_188440.p1_type_complete_len_850 | ---NA--- | | |

| | | | |
|---|---|---|---|
| 2597\|_\|c10612_f2p4_2549_isoform_c10612_full_length_coverage_2_non_full_length_coverage_4_isoform_length_2549_12906 | ---NA--- | | |
| 2598\|_\|c47571_f2p0_2549_isoform_c47571_full_length_coverage_2_non_full_length_coverage_0_isoform_length_2549_24229 | ---NA--- | | |
| 2599\|_\|c3729_f5p1_2549_isoform_c3729_full_length_coverage_5_non_full_length_coverage_1_isoform_length_2549_21373 | KDR14218.1Protein BANP | | |
| 2600\|_\|c12478_f2p0_2548_isoform_c12478_full_length_coverage_2_non_full_length_coverage_0_isoform_length_2548_17517 | XP_017880449.1serine/threonine-protein phosphatase 4 regulatory subunit 1-like isoform X1 | | |
| 2601\|_\|c1787_f2p3_2548_isoform_c1787_full_length_coverage_2_non_full_length_coverage_3_isoform_length_2548_27978 | ---NA--- | | |
| 2603\|_\|c6938_f3p9_2548_isoform_c6938_full_length_coverage_3_non_full_length_coverage_9_isoform_length_2548_21644 | ---NA--- | | |
| TRINITY_DN19203_c0_g1_i1_len_2904_265038.p1_type_complete_len_849 | ---NA--- | | |
| TRINITY_DN22824_c0_g1_i1_len_2793_58323.p1_type_complete_len_849 | XP_023230969.1crooked neck-like protein 1 | | |
| TRINITY_DN39829_c0_g1_i1_len_343_211173.p1_type_internal_len_115 | AJD14729.1glutathione s-transferase | F:GO:0016740 | |
| TRINITY_DN31784_c0_g1_i1_len_343_211174.p1_type_internal_len_115 | ---NA--- | | |
| TRINITY_DN39268_c0_g1_i1_len_669_200485.p1_type_complete_len_114 | ---NA--- | | |
| TRINITY_DN23624_c0_g1_i1_len_345_43815.p1_type_internal_len_115 | ---NA--- | | |
| TRINITY_DN33270_c0_g1_i1_len_483_204757.p1_type_5prime_partial_len_114 | ---NA--- | | |

| | | | |
|---|---|---|---|
| TRINITY_DN698_c0_g1_i1_len_890_63840.p4_type_5prime_partial_len_114 | ---NA--- | | |
| TRINITY_DN33076_c0_g1_i1_len_344_211095.p1_type_internal_len_115 | APU54663.1thioester-containing protein 1 | F:GO:0004866; C:GO:0005615; P:GO:0010951 | |
| TRINITY_DN2049_c0_g1_i1_len_344_211125.p1_type_internal_len_115 | ---NA--- | | |
| TRINITY_DN16634_c0_g1_i1_len_610_201562.p1_type_3prime_partial_len_115 | ---NA--- | | |
| TRINITY_DN4999_c0_g1_i1_len_344_211102.p1_type_internal_len_115 | ---NA--- | | |
| TRINITY_DN31945_c0_g1_i1_len_344_211103.p1_type_internal_len_115 | PIK35125.1hypothetical protein BSL78_28051 | F:GO:0003676; P:GO:0015074 | |
| TRINITY_DN40497_c0_g1_i1_len_344_211107.p1_type_internal_len_115 | ---NA--- | | |
| TRINITY_DN9925_c0_g1_i1_len_459_205580.p1_type_3prime_partial_len_115 | ---NA--- | | |
| TRINITY_DN34588_c0_g1_i1_len_345_137948.p1_type_internal_len_115 | XP_018015675.1 protocadherin-16-like isoform X2 | | |
| TRINITY_DN28411_c0_g1_i1_len_342_107038.p1_type_internal_len_115 | XP_018018361.1 cubilin-like | | |
| TRINITY_DN14108_c0_g1_i1_len_342_309619.p1_type_internal_len_115 | ---NA--- | | |
| TRINITY_DN40_c0_g1_i1_len_866_197851.p1_type_3prime_partial_len_115 | ---NA--- | | |
| TRINITY_DN33526_c0_g1_i1_len_437_244981.p1_type_complete_len_114 | ---NA--- | | |
| TRINITY_DN22186_c0_g1_i1_len_508_203988.p1_type_5prime_partial_len_114 | ---NA--- | | |
| TRINITY_DN1847_c0_g1_i1_len_344_211143.p1_type_internal_len_115 | ALJ96715.1transglutaminase II | F:GO:0003810; P:GO:0018149; F:GO:0046872 | EC:2.3.2.13 |
| TRINITY_DN4249_c0_g1_i1_len_343_14275.p1_type_internal_len_115 | ---NA--- | | |

| | | | |
|---|---|---|---|
| TRINITY_DN23302_c0_g1_i2_len_342_43966.p1_type_internal_len_115 | ---NA--- | | |
| TRINITY_DN7987_c0_g1_i1_len_344_211129.p1_type_internal_len_115 | ---NA--- | | |
| TRINITY_DN1669_c0_g1_i1_len_425_245444.p1_type_5prime_partial_len_114 | XP_018010018.1PREDICTED: uncharacterized protein LOC108667498 | | |
| TRINITY_DN18694_c0_g1_i2_len_346_137901.p1_type_internal_len_115 | ---NA--- | | |
| TRINITY_DN2821_c0_g1_i1_len_422_245552.p1_type_5prime_partial_len_114 | XP_018015673.1PREDICTED: uncharacterized protein LOC108672505 isoform X2 | | |
| TRINITY_DN23277_c0_g1_i1_len_344_211137.p1_type_internal_len_115 | ---NA--- | | |
| TRINITY_DN16453_c0_g2_i3_len_342_281522.p1_type_internal_len_115 | ---NA--- | | |
| TRINITY_DN27041_c0_g1_i1_len_352_309103.p2_type_5prime_partial_len_114 | XP_018016422.1 anoctamin-1-like | | |
| TRINITY_DN37238_c0_g1_i1_len_346_211022.p1_type_internal_len_115 | ADZ96217.1JHE-like carboxylesterase 1 | F:GO:0016787 | |
| TRINITY_DN30196_c0_g1_i1_len_342_281519.p1_type_internal_len_115 | ---NA--- | | |
| TRINITY_DN22340_c0_g1_i1_len_343_281460.p1_type_internal_len_115 | BBA53799.1crustacean female specific hormone | F:GO:0005125; C:GO:0005576; P:GO:0010469 | |
| TRINITY_DN442_c0_g1_i1_len_343_281462.p1_type_internal_len_115 | ---NA--- | | |
| TRINITY_DN19731_c0_g1_i2_len_6395_294630.p2_type_complete_len_114 | ---NA--- | | |
| TRINITY_DN24894_c0_g1_i1_len_3235_228732.p5_type_5prime_partial_len_114 | ---NA--- | | |
| TRINITY_DN3374_c0_g1_i1_len_345_211061.p1_type_internal_len_115 | ---NA--- | | |
| TRINITY_DN35632_c0_g1_i1_len_345_211058.p1_type_internal_len_115 | ---NA--- | | |

| | | | |
|---|---|---|---|
| TRINITY_DN12355_c0_g1_i1_len_355_248555.p1_type_5prime_partial_len_114 | ---NA--- | | |
| TRINITY_DN13238_c0_g1_i1_len_821_198364.p2_type_3prime_partial_len_115 | ---NA--- | | |
| TRINITY_DN16694_c0_g1_i2_len_407_104140.p1_type_complete_len_114 | ---NA--- | | |
| TRINITY_DN38368_c0_g1_i1_len_516_203757.p1_type_3prime_partial_len_115 | ---NA--- | | |
| TRINITY_DN6361_c0_g1_i1_len_342_249220.p1_type_internal_len_115 | AAC61691.1inositol 1,4,5-trisphosphate receptor | F:GO:0005220; C:GO:0005783; C:GO:0016021; P:GO:0048016; P:GO:0070588; F:GO:0070679 | |
| TRINITY_DN13393_c0_g1_i1_len_342_281486.p1_type_internal_len_115 | ---NA--- | | |
| TRINITY_DN29592_c0_g1_i1_len_342_249219.p1_type_internal_len_115 | XP_018012066.1 minor histocompatibility protein HA-1-like | | |
| TRINITY_DN65_c0_g1_i1_len_343_249195.p1_type_internal_len_115 | XP_018021372.1 serine/threonine-protein kinase tricorner-like | F:GO:0004674; F:GO:0005524; P:GO:0006468 | EC:2.7.11 |
| TRINITY_DN3817_c0_g1_i1_len_344_249128.p1_type_internal_len_115 | XP_018011038.1 putative polypeptide N-acetylgalactosaminyltransferase 9 isoform X6 | | |
| TRINITY_DN1041_c0_g1_i1_len_343_249193.p1_type_internal_len_115 | ---NA--- | | |
| TRINITY_DN30480_c0_g1_i1_len_345_249081.p1_type_internal_len_115 | AXR98511.1putative Niemann-Pick C1 protein | C:GO:0016021 | |
| TRINITY_DN28994_c0_g1_i1_len_345_249089.p1_type_internal_len_115 | ---NA--- | | |
| TRINITY_DN12790_c0_g1_i2_len_1098_195720.p3_type_5prime_partial_len_114 | ---NA--- | | |
| TRINITY_DN37132_c0_g1_i1_len_345_249103.p1_type_internal_len_115 | ---NA--- | | |
| TRINITY_DN26661_c0_g1_i1_len_344_249144.p1_type_internal_len_115 | ---NA--- | | |

| | | | |
|---|---|---|---|
| TRINITY_DN14326_c0_g1_i1_len_343_249175.p1_type_internal_len_115 | ---NA--- | | |
| TRINITY_DN27971_c0_g1_i1_len_343_249173.p1_type_internal_len_115 | XP_018026383.1PREDICTED: uncharacterized protein KIAA1109-like | | |
| TRINITY_DN27330_c0_g1_i1_len_344_249147.p1_type_internal_len_115 | XP_018013858.1 alkylglycerol monooxygenase-like | P:GO:0008152; C:GO:0016020 | |
| TRINITY_DN17415_c0_g1_i1_len_343_249170.p2_type_internal_len_115 | ---NA--- | | |
| TRINITY_DN5072_c0_g1_i1_len_344_249153.p1_type_internal_len_115 | ---NA--- | | |
| TRINITY_DN31660_c0_g1_i1_len_350_281096.p1_type_5prime_partial_len_114 | ---NA--- | | |
| TRINITY_DN12851_c0_g1_i1_len_344_249160.p1_type_internal_len_115 | ---NA--- | | |
| TRINITY_DN36050_c0_g1_i1_len_343_249190.p1_type_internal_len_115 | ---NA--- | | |
| TRINITY_DN27748_c0_g1_i1_len_344_249129.p1_type_internal_len_115 | ---NA--- | | |
| TRINITY_DN29880_c0_g1_i1_len_344_249134.p1_type_internal_len_115 | XP_018026041.1PREDICTED: uncharacterized protein LOC108681512 isoform X2 | | |
| TRINITY_DN5416_c0_g1_i1_len_344_249140.p1_type_internal_len_115 | XP_018027312.1PREDICTED: uncharacterized protein LOC108682621 | | |
| TRINITY_DN32746_c0_g1_i1_len_343_249176.p1_type_internal_len_115 | ---NA--- | | |
| TRINITY_DN13277_c0_g1_i1_len_453_102558.p1_type_5prime_partial_len_114 | ---NA--- | | |
| TRINITY_DN11899_c0_g1_i1_len_342_281497.p1_type_internal_len_115 | ---NA--- | | |
| TRINITY_DN4363_c0_g1_i1_len_346_249016.p1_type_internal_len_115 | ---NA--- | | |
| TRINITY_DN17274_c0_g1_i1_len_1105_159947.p3_type_5prime_partial_len_114 | ---NA--- | | |

| | | | |
|---|---|---|---|
| TRINITY_DN18549_c0_g1_i1_len_480_204885.p1_type_3prime_partial_len_115 | ---NA--- | | |
| TRINITY_DN38020_c0_g1_i1_len_342_249256.p1_type_internal_len_115 | ---NA--- | | |
| TRINITY_DN4330_c0_g1_i1_len_345_249056.p1_type_internal_len_115 | ---NA--- | | |
| TRINITY_DN30365_c0_g1_i1_len_342_249247.p1_type_internal_len_115 | ---NA--- | | |
| TRINITY_DN28762_c0_g1_i1_len_342_249245.p1_type_internal_len_115 | ---NA--- | | |
| TRINITY_DN34054_c0_g1_i1_len_345_249070.p1_type_internal_len_115 | XP_018007595.1 multidrug resistance-associated protein 5-like | | |
| TRINITY_DN37688_c0_g1_i1_len_342_249231.p1_type_internal_len_115 | ---NA--- | | |
| TRINITY_DN34497_c0_g1_i1_len_345_249077.p1_type_internal_len_115 | ---NA--- | | |
| TRINITY_DN13367_c0_g1_i1_len_577_202280.p1_type_3prime_partial_len_115 | ---NA--- | | |
| TRINITY_DN34446_c0_g1_i1_len_346_249041.p1_type_internal_len_115 | ---NA--- | | |
| TRINITY_DN4809_c0_g1_i1_len_346_249043.p1_type_internal_len_115 | ---NA--- | | |
| TRINITY_DN36862_c0_g1_i1_len_345_249047.p1_type_internal_len_115 | XP_020711663.1zinc finger protein 808-like | | |
| TRINITY_DN26969_c0_g1_i1_len_345_249049.p1_type_internal_len_115 | ---NA--- | | |
| TRINITY_DN7460_c0_g1_i1_len_345_281357.p1_type_internal_len_115 | ---NA--- | | |
| TRINITY_DN22700_c0_g1_i1_len_1558_192769.p2_type_3prime_partial_len_115 | ---NA--- | | |
| TRINITY_DN27468_c0_g1_i1_len_345_281345.p1_type_internal_len_115 | ---NA--- | | |

| | | | |
|---|---|---|---|
| TRINITY_DN3620_c0_g1_i1_len_345_281373.p1_type_internal_len_115 | ---NA--- | | |
| TRINITY_DN14451_c0_g1_i3_len_502_38653.p1_type_5prime_partial_len_114 | ---NA--- | | |
| TRINITY_DN11129_c0_g1_i1_len_566_241451.p1_type_5prime_partial_len_114 | ---NA--- | | |
| TRINITY_DN30061_c0_g1_i1_len_342_75217.p1_type_internal_len_115 | ---NA--- | | |
| TRINITY_DN30277_c0_g1_i1_len_343_281433.p1_type_internal_len_115 | XP_018021253.1 FH1/FH2 domain-containing protein 3-like | | |
| TRINITY_DN10871_c0_g1_i1_len_344_281384.p1_type_internal_len_115 | XP_002741199.1 kelch domain-containing protein 10-like | | |
| TRINITY_DN29194_c0_g1_i1_len_344_281412.p1_type_internal_len_115 | ---NA--- | | |
| TRINITY_DN30274_c0_g1_i1_len_344_281406.p3_type_internal_len_115 | AKQ22256.1CTLDcp1 | | |
| TRINITY_DN22734_c0_g1_i1_len_1079_62631.p2_type_complete_len_114 | ---NA--- | | |
| TRINITY_DN13491_c1_g1_i1_len_345_211067.p1_type_internal_len_115 | ---NA--- | | |
| TRINITY_DN7209_c0_g1_i1_len_349_13945.p1_type_complete_len_114 | XP_018917412.1 gamma-butyrobetaine dioxygenase isoform X2 | | |
| TRINITY_DN33484_c0_g1_i1_len_345_211031.p1_type_internal_len_115 | ---NA--- | | |
| TRINITY_DN29334_c2_g1_i1_len_2628_189176.p3_type_5prime_partial_len_114 | ---NA--- | | |
| TRINITY_DN287_c0_g1_i1_len_339_249444.p1_type_internal_len_114 | KRT84734.1C2 domain containing protein | C:GO:0016020 | |
| TRINITY_DN41801_c0_g1_i1_len_343_211205.p1_type_internal_len_114 | ---NA--- | | |
| TRINITY_DN19601_c0_g1_i1_len_1490_124317.p3_type_complete_len_113 | ---NA--- | | |

| | | | |
|---|---|---|---|
| TRINITY_DN23105_c0_g1_i2_len_2631_154780.p1_type_complete_len_849 | ---NA--- | | |
| TRINITY_DN22876_c1_g1_i1_len_2775_229317.p1_type_5prime_partial_len_849 | ---NA--- | | |
| TRINITY_DN25058_c0_g1_i1_len_2950_229058.p1_type_complete_len_849 | XP_021930369.1metabotropic glutamate receptor 5-like | F:GO:0004930; P:GO:0007165; P:GO:0007186; C:GO:0016020; C:GO:0016021 | |
| TRINITY_DN16476_c0_g1_i1_len_4322_228015.p1_type_5prime_partial_len_849 | XP_021928529.1striatin-interacting protein 1 isoform X1 | | |
| TRINITY_DN20334_c0_g1_i1_len_2911_29523.p1_type_5prime_partial_len_849 | XP_013773704.1transducin beta-like protein 3 isoform X1 | | |
| 2615\|_\|c2563_f5p2_2546_isoform_c2563_full_length_coverage_5_non_full_length_coverage_2_isoform_length_2546_11973 | ---NA--- | | |
| 2616\|_\|c1988_f2p8_2546_isoform_c1988_full_length_coverage_2_non_full_length_coverage_8_isoform_length_2546_28128 | ---NA--- | | |
| 2617\|_\|c42771_f24p17_2545_isoform_c42771_full_length_coverage_24_non_full_length_coverage_17_isoform_length_2545_14834 | ---NA--- | | |
| 2619\|_\|c1966_f2p16_2545_isoform_c1966_full_length_coverage_2_non_full_length_coverage_16_isoform_length_2545_28112 | EFX72164.1hypothetical protein DAPPUDRAFT_254801 | P:GO:0000122; F:GO:0003700; C:GO:0005634 | |
| 2620\|_\|c12780_f4p0_2545_isoform_c12780_full_length_coverage_4_non_full_length_coverage_0_isoform_length_2545_22160 | XP_018023106.1 peptidyl-prolyl cis-trans isomerase CWC27 homolog | P:GO:0009987; F:GO:0016853 | |
| TRINITY_DN33544_c0_g1_i1_len_343_211207.p1_type_internal_len_114 | XP_018009809.1 extracellular serine/threonine protein CG31145-like | | |
| TRINITY_DN29093_c0_g1_i1_len_339_249443.p1_type_internal_len_114 | XP_024887060.1solute carrier family 35 member G1 isoform X2 | | |
| TRINITY_DN2991_c0_g1_i1_len_340_249386.p1_type_internal_len_114 | ---NA--- | | |

| | | | |
|---|---|---|---|
| TRINITY_DN12833_c0_g1_i1_len_342_249238.p1_type_internal_len_114 | ---NA--- | | |
| TRINITY_DN34281_c0_g1_i1_len_342_249240.p1_type_internal_len_114 | ---NA--- | | |
| TRINITY_DN24825_c0_g1_i1_len_4087_228134.p4_type_5prime_partial_len_113 | ---NA--- | | |
| TRINITY_DN8156_c0_g1_i1_len_343_211188.p1_type_internal_len_114 | XP_021923219.1dystonin isoform X1 | C:GO:0005856; P:GO:0007010; F:GO:0008092 | |
| TRINITY_DN3919_c0_g1_i1_len_341_14332.p3_type_internal_len_114 | ---NA--- | | |
| TRINITY_DN34541_c0_g1_i1_len_341_249286.p1_type_internal_len_114 | XP_018009004.1 mucin-5AC-like | F:GO:0004722; P:GO:0006470; F:GO:0046872 | EC:3.1.3.16 |
| TRINITY_DN11265_c0_g1_i1_len_630_301956.p2_type_5prime_partial_len_113 | ---NA--- | | |
| TRINITY_DN35317_c0_g1_i1_len_341_249285.p1_type_internal_len_114 | ---NA--- | | |
| TRINITY_DN34047_c0_g1_i1_len_341_249284.p1_type_internal_len_114 | ---NA--- | | |
| TRINITY_DN18030_c0_g1_i3_len_879_34226.p2_type_complete_len_113 | ---NA--- | | |
| TRINITY_DN13140_c0_g1_i2_len_341_249287.p1_type_internal_len_114 | ---NA--- | | |
| TRINITY_DN33728_c0_g1_i1_len_342_249263.p1_type_internal_len_114 | ---NA--- | | |
| TRINITY_DN34667_c0_g1_i1_len_342_249269.p1_type_internal_len_114 | ---NA--- | | |
| TRINITY_DN5959_c0_g1_i1_len_342_249267.p1_type_internal_len_114 | XP_023721400.1microtubule-actin cross-linking factor 1 isoform X4 | | |
| TRINITY_DN18704_c0_g1_i1_len_363_42983.p1_type_5prime_partial_len_113 | ---NA--- | | |
| TRINITY_DN620_c0_g1_i1_len_342_249265.p1_type_internal_len_114 | ---NA--- | | |

| | | | |
|---|---|---|---|
| TRINITY_DN40597_c0_g1_i1_len_342_211231.p1_type_internal_len_114 | ---NA--- | | |
| TRINITY_DN3326_c0_g1_i1_len_342_211226.p1_type_internal_len_114 | ---NA--- | | |
| TRINITY_DN26373_c1_g1_i2_len_7303_227503.p8_type_complete_len_113 | ADI52575.1notch protein | F:GO:0005509; P:GO:0007219; P:GO:0007275; C:GO:0016021; P:GO:0030154; P:GO:0050793 | |
| TRINITY_DN20023_c0_g1_i1_len_1152_94738.p3_type_complete_len_113 | XP_013780661.1WD repeat-containing protein 92-like | | |
| TRINITY_DN20134_c2_g4_i1_len_911_270849.p1_type_complete_len_113 | ---NA--- | | |
| TRINITY_DN19629_c0_g1_i1_len_339_249472.p1_type_internal_len_114 | ---NA--- | | |
| TRINITY_DN32749_c0_g1_i1_len_343_249167.p1_type_internal_len_114 | ---NA--- | | |
| TRINITY_DN6856_c0_g1_i1_len_648_66360.p1_type_complete_len_113 | ---NA--- | | |
| TRINITY_DN21622_c0_g1_i1_len_1331_233875.p2_type_5prime_partial_len_113 | ---NA--- | | |
| TRINITY_DN4261_c0_g1_i1_len_343_14267.p1_type_internal_len_114 | ---NA--- | | |
| TRINITY_DN16776_c0_g1_i1_len_445_102801.p3_type_5prime_partial_len_113 | ---NA--- | | |
| TRINITY_DN23827_c0_g1_i1_len_343_281454.p1_type_internal_len_114 | XP_018020644.1 SEC14-like protein 2 isoform X2 | | |
| TRINITY_DN1306_c0_g1_i1_len_902_236936.p1_type_complete_len_113 | ---NA--- | | |
| TRINITY_DN23108_c1_g1_i1_len_2512_58577.p4_type_5prime_partial_len_113 | ---NA--- | | |
| TRINITY_DN38204_c0_g1_i1_len_342_249230.p1_type_internal_len_114 | ---NA--- | | |
| TRINITY_DN9707_c0_g1_i1_len_339_249453.p1_type_internal_len_114 | ---NA--- | | |

| | | | |
|---|---|---|---|
| TRINITY_DN9382_c0_g1_i1_len_339_249454.p1_type_internal_len_114 | ---NA--- | | |
| TRINITY_DN29000_c0_g1_i1_len_343_281428.p1_type_internal_len_114 | AYD41582.1macrophage mannose receptor 1-like protein | C:GO:0016020 | |
| TRINITY_DN17012_c0_g1_i1_len_400_135426.p1_type_5prime_partial_len_113 | ---NA--- | | |
| TRINITY_DN8814_c0_g1_i1_len_342_211221.p1_type_internal_len_114 | XP_018008782.1 laminin subunit alpha-like | | |
| TRINITY_DN10837_c0_g1_i1_len_342_211225.p1_type_internal_len_114 | ---NA--- | | |
| TRINITY_DN37740_c0_g1_i1_len_343_249209.p1_type_internal_len_114 | ---NA--- | | |
| TRINITY_DN14390_c0_g1_i1_len_2022_156048.p2_type_complete_len_113 | ---NA--- | | |
| TRINITY_DN20567_c0_g1_i2_len_2385_295274.p3_type_5prime_partial_len_113 | ---NA--- | | |
| TRINITY_DN27587_c0_g1_i1_len_340_249356.p1_type_internal_len_114 | AAT77812.1calpain T | F:GO:0004198; P:GO:0006508 | EC:3.4.22 |
| TRINITY_DN23886_c0_g1_i1_len_392_279234.p1_type_5prime_partial_len_113 | ---NA--- | | |
| TRINITY_DN9439_c0_g1_i2_len_463_244122.p2_type_5prime_partial_len_113 | XP_015510998.1 IDLSRF-like peptide | | |
| TRINITY_DN7606_c0_g1_i1_len_619_201396.p1_type_3prime_partial_len_114 | XP_018011035.1 putative polypeptide N-acetylgalactosaminyltransferase 9 isoform X4 | | |
| TRINITY_DN20398_c0_g1_i1_len_340_14387.p1_type_internal_len_114 | ---NA--- | | |
| TRINITY_DN31790_c0_g1_i1_len_340_249337.p1_type_internal_len_114 | ---NA--- | | |
| TRINITY_DN33990_c0_g1_i1_len_340_249399.p1_type_internal_len_114 | ---NA--- | | |
| TRINITY_DN27722_c1_g1_i1_len_1032_196278.p1_type_complete_len_113 | ---NA--- | | |

| | | | |
|---|---|---|---|
| TRINITY_DN31469_c0_g1_i1_len_340_14412.p1_type_internal_len_114 | KZS21375.1Ras-related protein Rab-23 | P:GO:0000045; F:GO:0003924; F:GO:0005525; C:GO:0005776; P:GO:0006886; C:GO:0010008; P:GO:0032482 | EC:3.6.1.15 |
| TRINITY_DN12936_c0_g1_i1_len_471_243872.p1_type_5prime_partial_len_113 | ---NA--- | | |
| TRINITY_DN13123_c0_g1_i1_len_1338_233819.p2_type_5prime_partial_len_113 | ---NA--- | | |
| TRINITY_DN34713_c0_g1_i1_len_340_249367.p1_type_internal_len_114 | ---NA--- | | |
| TRINITY_DN38343_c0_g1_i1_len_340_249366.p2_type_internal_len_114 | ---NA--- | | |
| TRINITY_DN25303_c0_g1_i1_len_1234_194715.p4_type_5prime_partial_len_113 | ---NA--- | | |
| TRINITY_DN21904_c0_g1_i1_len_1718_156968.p4_type_5prime_partial_len_113 | ---NA--- | | |
| TRINITY_DN6430_c0_g1_i1_len_340_249392.p1_type_internal_len_114 | XP_018017458.1PREDICTED: uncharacterized protein LOC108674070 | | |
| TRINITY_DN7310_c0_g1_i1_len_340_249375.p1_type_internal_len_114 | XP_018019355.1 myosin-IA-like | F:GO:0003774; F:GO:0003779; F:GO:0005524; C:GO:0016459 | EC:3.6.1.15 |
| TRINITY_DN3668_c0_g1_i1_len_340_249359.p1_type_internal_len_114 | ---NA--- | | |
| TRINITY_DN36133_c0_g1_i1_len_340_249357.p1_type_internal_len_114 | EDO42086.1predicted protein | C:GO:0005623; P:GO:0045454 | |
| TRINITY_DN37997_c0_g1_i1_len_340_249365.p1_type_internal_len_114 | ---NA--- | | |
| TRINITY_DN33943_c0_g1_i1_len_340_249393.p1_type_internal_len_114 | ---NA--- | | |
| TRINITY_DN10897_c0_g1_i1_len_341_249305.p1_type_internal_len_114 | BAM35674.1putative reverse transcriptase | F:GO:0003964; P:GO:0006278 | EC:2.7.7.49 |
| TRINITY_DN36828_c0_g1_i1_len_341_249319.p1_type_internal_len_114 | XP_018028173.1PREDICTED: uncharacterized protein LOC108683371 | | |

| | | | |
|---|---|---|---|
| TRINITY_DN36813_c0_g1_i1_len_341_249316.p1_type_internal_len_114 | ---NA--- | | |
| TRINITY_DN29434_c0_g1_i1_len_341_14346.p1_type_internal_len_114 | KDR19067.1Glycoprotein 3-alpha-L-fucosyltransferase A | C:GO:0016020; F:GO:0016740 | |
| TRINITY_DN31574_c0_g1_i1_len_339_249421.p1_type_internal_len_114 | ---NA--- | | |
| TRINITY_DN31917_c0_g1_i1_len_341_249292.p2_type_internal_len_114 | ---NA--- | | |
| TRINITY_DN26517_c0_g1_i2_len_1313_194183.p1_type_complete_len_113 | ---NA--- | | |
| TRINITY_DN30261_c0_g1_i1_len_341_249299.p1_type_internal_len_114 | ---NA--- | | |
| TRINITY_DN16694_c0_g1_i1_len_1046_196140.p1_type_5prime_partial_len_113 | ---NA--- | | |
| TRINITY_DN33068_c0_g1_i1_len_446_244695.p1_type_5prime_partial_len_113 | ---NA--- | | |
| TRINITY_DN32266_c0_g1_i1_len_341_249298.p1_type_internal_len_114 | XP_023953854.1tubulin beta chain | F:GO:0003924; F:GO:0005200; F:GO:0005525; C:GO:0005874; P:GO:0007010; P:GO:0007017 | EC:3.6.1.15 |
| TRINITY_DN21427_c0_g1_i1_len_1548_192841.p3_type_complete_len_113 | ---NA--- | | |
| TRINITY_DN3643_c0_g1_i1_len_341_249296.p1_type_internal_len_114 | XP_019867871.1 laminin subunit alpha-like | | |
| TRINITY_DN15328_c0_g1_i1_len_6484_264332.p2_type_complete_len_113 | ---NA--- | | |
| TRINITY_DN27464_c0_g1_i1_len_340_249335.p1_type_internal_len_114 | ---NA--- | | |
| TRINITY_DN6763_c0_g1_i1_len_341_249332.p1_type_3prime_partial_len_114 | XP_014676714.1 non-lysosomal glucosylceramidase-like isoform X2 | F:GO:0004348; P:GO:0006680; C:GO:0016020 | EC:3.2.1.45 |
| TRINITY_DN34902_c0_g1_i1_len_341_249330.p1_type_internal_len_114 | ---NA--- | | |

| | | | |
|---|---|---|---|
| TRINITY_DN8622_c0_g1_i1_len_341_249327.p3_type_internal_len_114 | KFM78548.1Discoidin domain-containing receptor 2 | F:GO:0004714; F:GO:0005524; P:GO:0007169; C:GO:0016021; P:GO:0018108 | EC:2.7.10; EC:2.7.10.1; EC:2.7.11 |
| TRINITY_DN2322_c0_g1_i1_len_700_163536.p2_type_5prime_partial_len_113 | ---NA--- | | |
| TRINITY_DN21696_c0_g1_i2_len_453_10014.p1_type_3prime_partial_len_114 | ---NA--- | | |
| TRINITY_DN23693_c0_g1_i1_len_341_14358.p1_type_internal_len_114 | XP_008845326.1 succinate-semialdehyde dehydrogenase, mitochondrial | P:GO:0008152; F:GO:0016620 | |
| TRINITY_DN7344_c1_g1_i1_len_343_211179.p1_type_internal_len_114 | ---NA--- | | |
| TRINITY_DN8148_c0_g1_i2_len_340_281604.p1_type_internal_len_114 | ---NA--- | | |
| TRINITY_DN26903_c0_g1_i1_len_504_101189.p1_type_5prime_partial_len_113 | ---NA--- | | |
| TRINITY_DN10549_c0_g1_i1_len_728_35401.p1_type_complete_len_113 | ---NA--- | | |
| TRINITY_DN19990_c0_g1_i1_len_1113_195593.p1_type_complete_len_113 | ---NA--- | | |
| TRINITY_DN24589_c1_g1_i1_len_340_75318.p2_type_internal_len_114 | ---NA--- | | |
| TRINITY_DN14284_c0_g1_i1_len_378_73301.p1_type_5prime_partial_len_113 | XP_021940286.1plastin-3 isoform X3 | F:GO:0005509; F:GO:0051015; P:GO:0051017 | |
| TRINITY_DN17437_c0_g1_i1_len_340_75316.p1_type_internal_len_114 | PIK35125.1hypothetical protein BSL78_28051 | F:GO:0003676; P:GO:0015074 | |
| TRINITY_DN25741_c0_g1_i1_len_398_104474.p1_type_5prime_partial_len_113 | ---NA--- | | |
| TRINITY_DN30486_c0_g1_i1_len_415_169645.p1_type_5prime_partial_len_113 | ---NA--- | | |
| TRINITY_DN25503_c0_g1_i1_len_341_281569.p1_type_internal_len_114 | ---NA--- | | |
| TRINITY_DN19198_c0_g1_i1_len_615_201462.p1_type_complete_len_113 | ---NA--- | | |

| | | | |
|---|---|---|---|
| TRINITY_DN15695_c0_g1_i1_len_388_246900.p1_type_5prime_partial_len_113 | XP_018010131.1 peroxidase-like | | |
| TRINITY_DN20644_c0_g1_i2_len_943_3920.p1_type_complete_len_113 | ---NA--- | | |
| TRINITY_DN28859_c0_g1_i1_len_341_281583.p1_type_internal_len_114 | XP_023240618.1CUE domain-containing protein 2-like | | |
| TRINITY_DN29733_c0_g1_i1_len_4651_187250.p1_type_5prime_partial_len_1482 | KZS17806.1RNA exonuclease 1 protein | F:GO:0003676; F:GO:0004527; F:GO:0046872; P:GO:0090305 | |
| TRINITY_DN31083_c0_g1_i2_len_5171_187088.p1_type_5prime_partial_len_1482 | XP_018011574.1 hemocytin-like | | |
| TRINITY_DN25548_c0_g1_i1_len_4747_227857.p1_type_3prime_partial_len_1481 | XP_018012596.1PREDICTED: uncharacterized protein LOC108669703 | | |
| TRINITY_DN31273_c1_g1_i2_len_4867_187169.p1_type_3prime_partial_len_1476 | XP_023218077.1uncharacterized protein LOC111620395 | | |
| TRINITY_DN25379_c0_g1_i1_len_4427_187348.p1_type_internal_len_1476 | ---NA--- | | |
| TRINITY_DN25405_c0_g1_i2_len_5494_227685.p1_type_complete_len_1472 | AYE56134.1timeless protein | | |
| TRINITY_DN29999_c0_g1_i1_len_6133_186917.p1_type_complete_len_1470 | XP_026287918.1rap guanine nucleotide exchange factor 6 isoform X3 | | |
| 286\|_\|c29764_f5p47_4385_isoform_c29764_full_length_coverage_5_non_full_length_coverage_47_isoform_length_4385_27002 | ACV60547.1polehole-like protein | | |
| TRINITY_DN29268_c0_g1_i2_len_6213_186906.p1_type_complete_len_1461 | XP_018009431.1 nuclear factor related to kappa-B-binding protein-like | C:GO:0031011 | |
| TRINITY_DN31075_c0_g1_i1_len_5197_187083.p1_type_complete_len_1461 | XP_012255239.1RB1-inducible coiled-coil protein 1 isoform X2 | P:GO:0000045; P:GO:0000422 | |
| 2621\|_\|c19375_f2p1_2545_isoform_c19375_full_length_coverage_2_non_full_length_coverage_1_isoform_length_2545_18144 | XP_023707981.1protein-associating with the carboxyl-terminal domain of ezrin | | |
| 2622\|_\|c3665_f2p1_2545_isoform_c3665_full_length_coverage_2_non_full_length_coverage_1_isoform_length_2545_21367 | ---NA--- | | |

| | | | |
|---|---|---|---|
| 2623\|_\|c1688_f2p16_2545_isoform_c1688_full_length_coverage_2_non_full_length_coverage_16_isoform_length_2545_27901 | XP_018007540.1 innexin inx2-like | | |
| TRINITY_DN27494_c0_g1_i2_len_2775_188905.p1_type_5prime_partial_len_848 | XP_018026693.1 testis-expressed sequence 2 protein-like | P:GO:0006869; F:GO:0008289; C:GO:0016020; C:GO:0016021 | |
| 2625\|_\|c2251_f4p8_2544_isoform_c2251_full_length_coverage_4_non_full_length_coverage_8_isoform_length_2544_11756 | XP_018016828.1 eukaryotic translation initiation factor 2D-like | P:GO:0001731; F:GO:0003723; F:GO:0003743; P:GO:0006413 | |
| 2627\|_\|c1993_f2p6_2543_isoform_c1993_full_length_coverage_2_non_full_length_coverage_6_isoform_length_2543_28133 | XP_018015149.1 coatomer subunit alpha-like isoform X2 | | |
| 2628\|_\|c2710_f4p2_2541_isoform_c2710_full_length_coverage_4_non_full_length_coverage_2_isoform_length_2541_21170 | ---NA--- | | |
| 2629\|_\|c46880_f5p0_2541_isoform_c46880_full_length_coverage_5_non_full_length_coverage_0_isoform_length_2541_19299 | ---NA--- | | |
| 2630\|_\|c17877_f4p2_2541_isoform_c17877_full_length_coverage_4_non_full_length_coverage_2_isoform_length_2541_17833 | ---NA--- | | |
| 2631\|_\|c2600_f4p1_2541_isoform_c2600_full_length_coverage_4_non_full_length_coverage_1_isoform_length_2541_12002 | XP_014678523.1 zinc finger protein 791-like | | |
| TRINITY_DN23529_c0_g1_i1_len_501_101279.p1_type_complete_len_113 | XP_006818893.1 cysteine-rich DPF motif domain-containing protein 1-like | | |
| TRINITY_DN11511_c0_g1_i1_len_390_208521.p1_type_5prime_partial_len_113 | ---NA--- | | |
| TRINITY_DN14105_c0_g1_i1_len_341_75243.p1_type_internal_len_114 | KDR17243.1Tyrosine-protein phosphatase non-receptor type 14 | F:GO:0004721; P:GO:0006470 | EC:3.1.3.16 |
| TRINITY_DN4729_c0_g1_i1_len_343_75178.p1_type_internal_len_114 | ---NA--- | | |
| TRINITY_DN26352_c0_g1_i3_len_5465_227692.p2_type_complete_len_113 | ---NA--- | | |

| | | | |
|---|---|---|---|
| TRINITY_DN11896_c0_g1_i1_len_341_75248.p1_type_internal_len_114 | ---NA--- | | |
| TRINITY_DN13446_c0_g1_i1_len_414_306509.p1_type_complete_len_113 | XP_001120529.2H/ACA ribonucleoprotein complex subunit 3 | P:GO:0001522; F:GO:0030515; P:GO:0042254 | |
| TRINITY_DN31332_c0_g1_i1_len_1972_123179.p4_type_5prime_partial_len_113 | ---NA--- | | |
| TRINITY_DN22605_c0_g1_i1_len_339_281692.p1_type_internal_len_114 | KZS21895.1putative Rho GTPase-activating protein 20 | P:GO:0007165 | |
| TRINITY_DN27382_c0_g1_i1_len_339_281686.p1_type_internal_len_114 | ---NA--- | | |
| TRINITY_DN7405_c0_g1_i1_len_576_37333.p2_type_5prime_partial_len_113 | ---NA--- | | |
| TRINITY_DN24987_c0_g1_i1_len_1819_191602.p1_type_complete_len_113 | ---NA--- | | |
| TRINITY_DN14347_c0_g1_i1_len_1021_235941.p1_type_5prime_partial_len_113 | ---NA--- | | |
| TRINITY_DN11482_c0_g1_i1_len_340_75296.p1_type_internal_len_114 | AAL96263.1immunoglobulin gamma-1 heavy chain constant region | | |
| TRINITY_DN16507_c0_g1_i2_len_341_75281.p1_type_internal_len_114 | ---NA--- | | |
| TRINITY_DN36682_c0_g1_i1_len_374_247561.p1_type_5prime_partial_len_113 | ---NA--- | | |
| TRINITY_DN32717_c0_g1_i1_len_340_75310.p1_type_internal_len_114 | XP_021913253.1enhancer of mRNA-decapping protein 4 isoform X2 | | |
| TRINITY_DN32994_c0_g1_i1_len_341_75269.p2_type_internal_len_114 | ---NA--- | | |
| TRINITY_DN29597_c0_g1_i1_len_341_75268.p2_type_internal_len_114 | XP_018008896.1PREDICTED: uncharacterized protein LOC108666520 isoform X1 | | |
| TRINITY_DN6092_c0_g1_i1_len_341_75267.p1_type_internal_len_114 | XP_018023208.1 cytochrome P450 3A24-like | F:GO:0005506; C:GO:0016021; F:GO:0016705; F:GO:0020037; P:GO:0055114 | |

| | | | |
|---|---|---|---|
| TRINITY_DN18824_c0_g1_i2_len_376_136407.p1_type_5prime_partial_len_113 | AHJ10947.1slow tropomyosin isoform | | |
| TRINITY_DN25341_c0_g1_i1_len_340_107145.p1_type_internal_len_114 | ---NA--- | | |
| TRINITY_DN17759_c0_g1_i1_len_435_206503.p1_type_5prime_partial_len_113 | ---NA--- | | |
| TRINITY_DN28682_c0_g1_i1_len_339_107193.p1_type_internal_len_114 | ABB73282.1reverse transcriptase | F:GO:0003964; P:GO:0006278 | |
| TRINITY_DN13323_c0_g1_i1_len_364_136967.p1_type_3prime_partial_len_114 | ---NA--- | | |
| TRINITY_DN23069_c0_g1_i1_len_340_107160.p1_type_internal_len_114 | ---NA--- | | |
| TRINITY_DN14360_c0_g1_i2_len_444_305547.p1_type_5prime_partial_len_113 | ---NA--- | | |
| TRINITY_DN31728_c0_g1_i1_len_342_107010.p1_type_internal_len_114 | ---NA--- | | |
| TRINITY_DN21557_c0_g1_i3_len_1426_1884.p2_type_complete_len_113 | ---NA--- | | |
| TRINITY_DN27124_c0_g1_i1_len_341_107090.p1_type_internal_len_114 | ACU31809.1alpha2 macroglobulin isoform 3 | C:GO:0005576 | |
| TRINITY_DN15310_c0_g1_i1_len_341_107066.p1_type_internal_len_114 | ---NA--- | | |
| TRINITY_DN5827_c0_g1_i2_len_346_249008.p1_type_5prime_partial_len_113 | ---NA--- | | |
| TRINITY_DN27993_c0_g2_i1_len_1665_192233.p2_type_5prime_partial_len_113 | ---NA--- | | |
| TRINITY_DN18695_c0_g1_i2_len_454_305283.p1_type_5prime_partial_len_113 | XP_005891283.2 histone H4 | C:GO:0000786; F:GO:0003677; C:GO:0005634; P:GO:0006352; F:GO:0046982 | |
| TRINITY_DN21801_c0_g1_i4_len_931_236697.p1_type_3prime_partial_len_114 | ---NA--- | | |
| TRINITY_DN22233_c0_g1_i1_len_444_102844.p1_type_3prime_partial_len_114 | ---NA--- | | |

| | | | |
|---|---|---|---|
| TRINITY_DN35138_c0_g1_i1_len_424_206945.p1_type_5prime_partial_len_113 | ---NA--- | | |
| TRINITY_DN22012_c0_g1_i3_len_1124_195498.p2_type_complete_len_113 | ---NA--- | | |
| TRINITY_DN21046_c0_g2_i1_len_339_107226.p1_type_internal_len_114 | ---NA--- | | |
| TRINITY_DN34001_c0_g1_i1_len_339_173028.p1_type_internal_len_114 | ---NA--- | | |
| TRINITY_DN16191_c0_g1_i1_len_339_173053.p1_type_internal_len_114 | ---NA--- | | |
| TRINITY_DN188_c0_g1_i1_len_339_173067.p1_type_internal_len_114 | ---NA--- | | |
| TRINITY_DN13773_c0_g1_i1_len_886_197640.p1_type_complete_len_113 | ---NA--- | | |
| TRINITY_DN2385_c0_g1_i1_len_340_172980.p1_type_internal_len_114 | ---NA--- | | |
| TRINITY_DN34182_c0_g1_i1_len_340_172982.p1_type_internal_len_114 | ---NA--- | | |
| TRINITY_DN10365_c0_g1_i1_len_340_172997.p1_type_internal_len_114 | ---NA--- | | |
| TRINITY_DN17985_c0_g1_i1_len_339_75394.p1_type_internal_len_114 | EDO42099.1predicted protein | P:GO:0006281 | |
| TRINITY_DN4164_c0_g1_i1_len_523_166570.p1_type_5prime_partial_len_113 | ---NA--- | | |
| TRINITY_DN13084_c0_g1_i2_len_342_281538.p1_type_internal_len_114 | AUB13319.1C-type lectin containing low-density lipoprotein receptor domain | F:GO:0030246 | |
| TRINITY_DN24183_c0_g1_i1_len_3451_228525.p3_type_complete_len_113 | ---NA--- | | |
| TRINITY_DN27919_c0_g1_i1_len_343_106970.p1_type_internal_len_114 | ---NA--- | | |
| TRINITY_DN28311_c0_g1_i1_len_550_100285.p2_type_5prime_partial_len_113 | ---NA--- | | |

| | | | |
|---|---|---|---|
| TRINITY_DN286_c0_g1_i1_len_545_131382.p1_type_5prime_partial_len_113 | XP_011557312.1 ankyrin repeat domain-containing protein 29 | | |
| TRINITY_DN31415_c0_g1_i1_len_342_172855.p1_type_internal_len_114 | ---NA--- | | |
| TRINITY_DN9021_c0_g1_i1_len_343_172823.p1_type_internal_len_114 | ---NA--- | | |
| TRINITY_DN30703_c0_g1_i1_len_343_106985.p1_type_internal_len_114 | ---NA--- | | |
| TRINITY_DN13395_c0_g1_i1_len_341_172932.p1_type_internal_len_114 | ---NA--- | | |
| TRINITY_DN28145_c0_g1_i1_len_341_172936.p1_type_internal_len_114 | ADV17348.1antiviral-like c type lectin | F:GO:0030246 | |
| TRINITY_DN19283_c0_g1_i1_len_1086_235480.p1_type_complete_len_113 | ---NA--- | | |
| TRINITY_DN28300_c0_g1_i1_len_341_172947.p1_type_internal_len_114 | ---NA--- | | |
| TRINITY_DN18842_c0_g1_i1_len_617_201439.p1_type_5prime_partial_len_113 | ---NA--- | | |
| TRINITY_DN10124_c0_g1_i1_len_433_134163.p1_type_5prime_partial_len_113 | ---NA--- | | |
| TRINITY_DN10583_c0_g1_i1_len_560_202663.p1_type_5prime_partial_len_113 | PSN43596.1hypothetical protein C0J52_15975 | C:GO:0016021 | |
| TRINITY_DN6952_c0_g1_i1_len_340_309704.p1_type_internal_len_114 | ---NA--- | | |
| TRINITY_DN28308_c0_g1_i1_len_340_138266.p1_type_internal_len_114 | ---NA--- | | |
| TRINITY_DN3319_c0_g1_i1_len_340_138289.p1_type_internal_len_114 | ---NA--- | | |
| TRINITY_DN40856_c0_g1_i1_len_339_211422.p1_type_internal_len_114 | XP_008193881.1 zinc finger protein 395 isoform X2 | | |
| TRINITY_DN27952_c0_g1_i1_len_393_11974.p1_type_5prime_partial_len_113 | ---NA--- | | |

| | | | |
|---|---|---|---|
| TRINITY_DN24746_c0_g1_i1_len_346_137929.p1_type_5prime_partial_len_113 | ---NA--- | | |
| TRINITY_DN3505_c0_g1_i1_len_341_138222.p1_type_internal_len_114 | ---NA--- | | |
| TRINITY_DN1052_c0_g1_i1_len_341_138249.p2_type_internal_len_114 | ABP97431.1alpha 2 macroglobulin | F:GO:0004866; C:GO:0005615; P:GO:0010951 | |
| TRINITY_DN804_c0_g1_i1_len_341_138255.p1_type_internal_len_114 | ---NA--- | | |
| TRINITY_DN23386_c0_g1_i1_len_2173_230586.p2_type_complete_len_113 | ---NA--- | | |
| TRINITY_DN21257_c0_g1_i1_len_343_43920.p1_type_internal_len_114 | ---NA--- | | |
| TRINITY_DN29693_c0_g1_i1_len_339_309760.p1_type_internal_len_114 | ---NA--- | | |
| TRINITY_DN23306_c0_g1_i1_len_503_275980.p1_type_5prime_partial_len_113 | ---NA--- | | |
| TRINITY_DN14552_c0_g1_i1_len_407_278708.p1_type_5prime_partial_len_113 | ---NA--- | | |
| TRINITY_DN24035_c0_g1_i2_len_1133_62326.p2_type_complete_len_113 | ---NA--- | | |
| TRINITY_DN18115_c0_g1_i2_len_339_138348.p1_type_internal_len_114 | ---NA--- | | |
| TRINITY_DN36463_c0_g1_i1_len_340_211390.p1_type_internal_len_114 | ---NA--- | | |
| TRINITY_DN943_c0_g1_i1_len_439_10461.p1_type_5prime_partial_len_113 | ---NA--- | | |
| TRINITY_DN18501_c0_g1_i3_len_339_309749.p1_type_internal_len_114 | ---NA--- | | |
| TRINITY_DN24419_c0_g1_i1_len_1952_191079.p2_type_complete_len_113 | XP_018016764.1PREDICTED: uncharacterized protein LOC108673440 | | |
| TRINITY_DN2468_c0_g1_i1_len_339_309754.p1_type_internal_len_114 | ---NA--- | | |

| | | | |
|---|---|---|---|
| TRINITY_DN23168_c0_g1_i1_len_340_309735.p1_type_internal_len_114 | ---NA--- | | |
| TRINITY_DN19809_c0_g1_i3_len_765_238355.p3_type_5prime_partial_len_113 | ---NA--- | | |
| TRINITY_DN14489_c0_g1_i1_len_611_36806.p1_type_5prime_partial_len_113 | ---NA--- | | |
| TRINITY_DN25045_c0_g1_i1_len_2000_231120.p2_type_5prime_partial_len_113 | ---NA--- | | |
| TRINITY_DN41420_c0_g1_i1_len_339_211468.p1_type_internal_len_114 | ---NA--- | | |
| TRINITY_DN16115_c0_g1_i1_len_343_138124.p1_type_internal_len_114 | ---NA--- | | |
| TRINITY_DN37149_c0_g1_i1_len_419_207163.p1_type_5prime_partial_len_113 | ---NA--- | | |
| TRINITY_DN30654_c0_g1_i1_len_340_44077.p1_type_internal_len_114 | ---NA--- | | |
| TRINITY_DN11339_c0_g1_i1_len_538_275189.p1_type_5prime_partial_len_113 | KMQ89417.1nuclear receptor interaction protein | | |
| TRINITY_DN6777_c0_g1_i2_len_339_211442.p2_type_internal_len_114 | ---NA--- | | |
| TRINITY_DN22783_c1_g1_i1_len_1672_232239.p1_type_5prime_partial_len_113 | ---NA--- | | |
| TRINITY_DN20725_c0_g1_i1_len_1297_158778.p1_type_5prime_partial_len_113 | ---NA--- | | |
| TRINITY_DN32847_c0_g1_i1_len_542_241956.p1_type_5prime_partial_len_113 | ---NA--- | | |
| TRINITY_DN18230_c0_g1_i1_len_2021_231056.p2_type_complete_len_113 | ---NA--- | | |
| TRINITY_DN27062_c0_g1_i1_len_341_138207.p1_type_internal_len_114 | XP_026279821.1nascent polypeptide-associated complex subunit alpha, muscle-specific form isoform X1 | C:GO:0016020 | |
| TRINITY_DN33389_c0_g1_i1_len_531_242218.p1_type_5prime_partial_len_113 | ---NA--- | | |

| | | | |
|---|---|---|---|
| 2632\|_\|c4640_f3p1_2541_isoform_c4640_full_length_coverage_3_non_full_length_coverage_1_isoform_length_2541_12397 | ---NA--- | | |
| TRINITY_DN27607_c0_g1_i3_len_3205_188251.p1_type_complete_len_847 | XP_023707059.1scaffold attachment factor B2 isoform X3 | F:GO:0003723 | |
| 2635\|_\|c5166_f2p5_2540_isoform_c5166_full_length_coverage_2_non_full_length_coverage_5_isoform_length_2540_16931 | ABB05537.1ferritin peptide | F:GO:0004322; C:GO:0005623; P:GO:0006826; P:GO:0006879; F:GO:0008199; P:GO:0055114 | EC:1.16.3.1; EC:1.16.3 |
| 2636\|_\|c11421_f2p0_2539_isoform_c11421_full_length_coverage_2_non_full_length_coverage_0_isoform_length_2539_12971 | ---NA--- | | |
| 2637\|_\|c18113_f4p2_2538_isoform_c18113_full_length_coverage_4_non_full_length_coverage_2_isoform_length_2538_17960 | ---NA--- | | |
| TRINITY_DN28533_c0_g1_i1_len_2646_189136.p1_type_5prime_partial_len_846 | XP_018327549.1N-terminal kinase-like protein isoform X2 | F:GO:0004672; F:GO:0005524; P:GO:0006468 | |
| TRINITY_DN29404_c0_g1_i1_len_3274_188165.p1_type_complete_len_845 | XP_018011706.1 serine/threonine-protein kinase fused-like | F:GO:0016301; P:GO:0016310 | |
| 2640\|_\|c40122_f2p14_2535_isoform_c40122_full_length_coverage_2_non_full_length_coverage_14_isoform_length_2535_23715 | XP_018019971.1PREDICTED: uncharacterized protein LOC108676407 | | |
| 2641\|_\|c5689_f3p5_2535_isoform_c5689_full_length_coverage_3_non_full_length_coverage_5_isoform_length_2535_21547 | XP_022240350.1protein NEDD1-like isoform X2 | | |
| 2642\|_\|c2342_f2p1_2535_isoform_c2342_full_length_coverage_2_non_full_length_coverage_1_isoform_length_2535_16339 | XP_018020216.1 integrator complex subunit 8-like | P:GO:0034472 | |
| TRINITY_DN25101_c1_g1_i1_len_962_63339.p2_type_complete_len_113 | ---NA--- | | |
| TRINITY_DN16123_c0_g1_i1_len_341_309647.p1_type_internal_len_114 | ABB73282.1reverse transcriptase | F:GO:0003964; P:GO:0006278 | EC:2.7.7.49 |
| TRINITY_DN1357_c0_g1_i1_len_339_211433.p1_type_internal_len_114 | XP_021935515.1protocadherin Fat 2 | F:GO:0005509; P:GO:0007156; C:GO:0016020; C:GO:0016021 | |

| | | | |
|---|---|---|---|
| TRINITY_DN1220_c0_g1_i1_len_341_138209.p1_type_3prime_partial_len_114 | ---NA--- | | |
| TRINITY_DN9987_c0_g1_i1_len_685_239426.p1_type_3prime_partial_len_114 | ---NA--- | | |
| TRINITY_DN2125_c0_g1_i1_len_342_309589.p1_type_internal_len_114 | ---NA--- | | |
| TRINITY_DN3294_c0_g1_i1_len_339_14484.p2_type_internal_len_114 | ---NA--- | | |
| TRINITY_DN16261_c0_g1_i1_len_842_127676.p2_type_5prime_partial_len_113 | ---NA--- | | |
| TRINITY_DN22484_c0_g1_i1_len_1687_232181.p4_type_complete_len_113 | XP_001186509.2PREDICTED: uncharacterized protein LOC754648 | F:GO:0005488 | |
| TRINITY_DN13429_c0_g1_i1_len_532_242196.p1_type_3prime_partial_len_114 | ---NA--- | | |
| TRINITY_DN11249_c1_g1_i1_len_342_211258.p1_type_internal_len_114 | ---NA--- | | |
| TRINITY_DN38182_c0_g1_i1_len_341_211278.p1_type_internal_len_114 | ---NA--- | | |
| TRINITY_DN3141_c0_g1_i2_len_341_211298.p2_type_internal_len_114 | XP_022332945.1multidrug resistance-associated protein 7-like | | |
| TRINITY_DN41053_c0_g1_i1_len_341_211279.p1_type_internal_len_114 | KRT78217.1Myosin head | F:GO:0003774; F:GO:0005524; C:GO:0016459; F:GO:0051015 | EC:3.6.1.15 |
| TRINITY_DN35485_c0_g1_i1_len_341_211299.p1_type_internal_len_114 | XP_018024061.1 kinase D-interacting substrate of 220 kDa-like isoform X1 | | |
| TRINITY_DN32698_c0_g1_i1_len_340_211365.p1_type_internal_len_114 | ---NA--- | | |
| TRINITY_DN36514_c0_g1_i1_len_341_211308.p1_type_internal_len_114 | APU54663.1thioester-containing protein 1 | F:GO:0004866; C:GO:0005615; P:GO:0010951 | |
| TRINITY_DN17606_c0_g1_i1_len_340_211355.p2_type_internal_len_114 | ---NA--- | | |
| TRINITY_DN39800_c0_g1_i1_len_340_211367.p1_type_internal_len_114 | ---NA--- | | |

| | | | |
|---|---|---|---|
| TRINITY_DN32817_c0_g1_i1_len_341_211280.p1_type_internal_len_114 | ---NA--- | | |
| TRINITY_DN19240_c0_g1_i1_len_1451_267931.p2_type_5prime_partial_len_113 | ---NA--- | | |
| TRINITY_DN22756_c0_g1_i1_len_339_14449.p1_type_internal_len_114 | ---NA--- | | |
| TRINITY_DN18235_c0_g1_i1_len_341_211293.p1_type_internal_len_114 | XP_023716242.1pleckstrin homology domain-containing family A member 8 isoform X3 | C:GO:0005737; P:GO:0120009; F:GO:0120013 | |
| TRINITY_DN30111_c1_g2_i1_len_714_199781.p2_type_complete_len_113 | ---NA--- | | |
| TRINITY_DN28709_c0_g1_i1_len_571_202399.p1_type_5prime_partial_len_113 | ---NA--- | | |
| TRINITY_DN35156_c0_g1_i1_len_341_211276.p1_type_internal_len_114 | EEC05053.1tgf(beta)-induced transcription factor, putative | F:GO:0003677; C:GO:0005634; P:GO:0006355 | |
| TRINITY_DN18460_c0_g1_i1_len_353_210564.p1_type_5prime_partial_len_113 | ---NA--- | | |
| TRINITY_DN39209_c0_g1_i1_len_341_211287.p1_type_internal_len_114 | ---NA--- | | |
| TRINITY_DN40571_c0_g1_i1_len_341_211316.p1_type_internal_len_114 | XP_024204735.1beta-2-microglobulin isoform X1 | | |
| TRINITY_DN39888_c0_g1_i1_len_340_211378.p1_type_internal_len_114 | XP_023708627.1nostrin isoform X4 | | |
| TRINITY_DN41990_c0_g1_i1_len_341_211325.p1_type_internal_len_114 | ---NA--- | | |
| TRINITY_DN14512_c0_g1_i1_len_342_211253.p1_type_internal_len_114 | ---NA--- | | |
| TRINITY_DN33767_c0_g1_i1_len_341_211291.p1_type_internal_len_114 | ---NA--- | | |
| TRINITY_DN19525_c1_g1_i1_len_458_70313.p4_type_5prime_partial_len_113 | ---NA--- | | |
| TRINITY_DN37127_c0_g1_i1_len_342_211243.p1_type_internal_len_114 | ---NA--- | | |

| | | | |
|---|---|---|---|
| TRINITY_DN39500_c0_g1_i1_len_342_211245.p1_type_internal_len_114 | XP_018027333.1 low-density lipoprotein receptor-related protein 2-like | | |
| TRINITY_DN37102_c0_g1_i1_len_341_211322.p2_type_internal_len_114 | XP_018021773.1 forkhead box C1-A-like | F:GO:0003700; C:GO:0005634; P:GO:0006355; F:GO:0043565 | |
| TRINITY_DN40613_c0_g1_i1_len_341_211286.p1_type_3prime_partial_len_114 | ---NA--- | | |
| TRINITY_DN13903_c0_g1_i2_len_340_211381.p1_type_internal_len_114 | ---NA--- | | |
| TRINITY_DN7260_c0_g1_i1_len_341_211317.p1_type_internal_len_114 | ---NA--- | | |
| TRINITY_DN21990_c0_g1_i2_len_2076_190634.p2_type_3prime_partial_len_114 | ---NA--- | | |
| TRINITY_DN38035_c0_g1_i1_len_341_211285.p1_type_internal_len_114 | ---NA--- | | |
| TRINITY_DN32740_c0_g1_i1_len_340_211356.p1_type_5prime_partial_len_113 | ---NA--- | | |
| TRINITY_DN16591_c0_g1_i1_len_341_211334.p1_type_internal_len_114 | ---NA--- | | |
| TRINITY_DN27277_c0_g1_i1_len_340_14431.p1_type_internal_len_114 | ---NA--- | | |
| TRINITY_DN28022_c1_g1_i1_len_340_211360.p1_type_internal_len_114 | ---NA--- | | |
| TRINITY_DN3602_c0_g1_i1_len_341_211313.p1_type_internal_len_114 | ---NA--- | | |
| TRINITY_DN40590_c0_g1_i1_len_342_211255.p1_type_internal_len_114 | KFM78306.1Low-density lipoprotein receptor-related protein 6 | C:GO:0016021 | |
| TRINITY_DN37224_c0_g1_i1_len_338_249492.p1_type_internal_len_113 | XP_024938112.1intersectin-1 isoform X25 | | |
| TRINITY_DN10259_c0_g1_i1_len_338_249510.p1_type_internal_len_113 | XP_018025352.1 tyrosine-protein phosphatase 99A-like isoform X2 | | |
| TRINITY_DN13779_c0_g1_i1_len_338_281714.p1_type_internal_len_113 | AAD32609.1phospholipid phospholipase C beta isoform | F:GO:0004435; F:GO:0005509; P:GO:0016042; P:GO:0035556 | EC:3.1.4.11; EC:3.1.4.3 |

| | | | |
|---|---|---|---|
| TRINITY_DN41805_c0_g1_i1_len_338_211479.p1_type_internal_len_113 | XP_025994791.1lysophospholipase D GDPD1-like | P:GO:0006629; F:GO:0008081 | |
| TRINITY_DN32702_c0_g1_i1_len_338_249509.p1_type_internal_len_113 | ---NA--- | | |
| TRINITY_DN20100_c0_g1_i2_len_535_275268.p1_type_3prime_partial_len_113 | ---NA--- | | |
| TRINITY_DN8555_c0_g1_i1_len_338_249515.p1_type_internal_len_113 | ---NA--- | | |
| TRINITY_DN16748_c0_g1_i2_len_1658_92887.p3_type_complete_len_112 | ---NA--- | | |
| TRINITY_DN9262_c0_g1_i1_len_1014_33416.p1_type_complete_len_112 | ---NA--- | | |
| TRINITY_DN3412_c0_g1_i1_len_338_281729.p1_type_internal_len_113 | ---NA--- | | |
| TRINITY_DN7226_c0_g1_i1_len_338_249497.p1_type_internal_len_113 | ---NA--- | | |
| TRINITY_DN31597_c0_g1_i1_len_337_211557.p1_type_internal_len_113 | XP_026272238.1epoxide hydrolase 3-like | F:GO:0003824; C:GO:0016021 | |
| TRINITY_DN8213_c0_g1_i1_len_337_75508.p1_type_internal_len_113 | ---NA--- | | |
| TRINITY_DN31225_c0_g1_i1_len_338_249502.p1_type_internal_len_113 | ---NA--- | | |
| TRINITY_DN32995_c0_g1_i1_len_338_249519.p1_type_5prime_partial_len_112 | ---NA--- | | |
| TRINITY_DN26446_c0_g1_i1_len_338_249505.p1_type_internal_len_113 | ---NA--- | | |
| TRINITY_DN42288_c0_g1_i1_len_338_211482.p1_type_internal_len_113 | XP_020557540.1retinol dehydrogenase 13-like isoform X3 | | |
| TRINITY_DN29610_c0_g1_i1_len_338_249523.p1_type_internal_len_113 | ---NA--- | | |
| TRINITY_DN34131_c0_g1_i1_len_338_249504.p1_type_internal_len_113 | ---NA--- | | |

| | | | |
|---|---|---|---|
| TRINITY_DN28960_c0_g1_i1_len_339_249429.p1_type_internal_len_113 | ---NA--- | | |
| TRINITY_DN34118_c0_g1_i1_len_339_249428.p1_type_internal_len_113 | ---NA--- | | |
| TRINITY_DN9048_c0_g1_i2_len_338_211499.p1_type_internal_len_113 | ASC55672.1putative complement component 1, q subcomponent protein | | |
| TRINITY_DN15917_c0_g1_i1_len_687_200209.p1_type_3prime_partial_len_113 | ---NA--- | | |
| TRINITY_DN37371_c0_g1_i1_len_339_249416.p1_type_internal_len_113 | ---NA--- | | |
| TRINITY_DN11065_c0_g1_i1_len_339_281651.p2_type_internal_len_113 | ---NA--- | | |
| TRINITY_DN35797_c0_g1_i1_len_337_211575.p1_type_internal_len_113 | ---NA--- | | |
| TRINITY_DN18462_c0_g1_i1_len_336_138517.p1_type_internal_len_113 | ---NA--- | | |
| TRINITY_DN8044_c0_g1_i1_len_372_73593.p1_type_5prime_partial_len_112 | ---NA--- | | |
| TRINITY_DN41284_c0_g1_i1_len_449_206004.p1_type_5prime_partial_len_112 | ---NA--- | | |
| TRINITY_DN21027_c1_g1_i2_len_938_161186.p2_type_3prime_partial_len_113 | ---NA--- | | |
| TRINITY_DN23739_c1_g1_i1_len_2029_123064.p2_type_3prime_partial_len_113 | ---NA--- | | |
| TRINITY_DN26385_c0_g1_i1_len_338_14536.p1_type_internal_len_113 | ---NA--- | | |
| TRINITY_DN37684_c0_g1_i1_len_336_249655.p1_type_internal_len_113 | XP_018024062.1 kinase D-interacting substrate of 220 kDa-like isoform X2 | | |
| TRINITY_DN24180_c0_g1_i1_len_550_202885.p1_type_5prime_partial_len_112 | ---NA--- | | |
| TRINITY_DN24870_c0_g1_i1_len_3296_58004.p4_type_complete_len_112 | ---NA--- | | |

| | | | |
|---|---|---|---|
| TRINITY_DN22402_c0_g2_i1_len_342_138184.p1_type_5prime_partial_len_112 | ---NA--- | | |
| TRINITY_DN12884_c0_g1_i1_len_817_271598.p1_type_complete_len_112 | ---NA--- | | |
| TRINITY_DN16153_c0_g1_i1_len_1822_59739.p2_type_5prime_partial_len_112 | ---NA--- | | |
| TRINITY_DN18079_c0_g1_i1_len_591_201972.p1_type_3prime_partial_len_113 | ---NA--- | | |
| TRINITY_DN33108_c0_g1_i1_len_339_249460.p1_type_internal_len_113 | XP_018015773.1 solute carrier organic anion transporter family member 4A1-like | F:GO:0005215; C:GO:0005886; P:GO:0006811; C:GO:0016021; P:GO:0055085 | |
| TRINITY_DN37768_c0_g1_i1_len_336_249650.p2_type_internal_len_113 | ---NA--- | | |
| TRINITY_DN36540_c0_g1_i1_len_338_211498.p2_type_internal_len_113 | EDM15768.1ribosomal protein L3, isoform CRA_a | F:GO:0003735; C:GO:0005840; P:GO:0006412 | |
| TRINITY_DN24859_c0_g1_i1_len_339_281697.p1_type_internal_len_113 | ---NA--- | | |
| TRINITY_DN23532_c0_g1_i3_len_1599_192569.p2_type_complete_len_112 | XP_018010540.1 endophilin-A-like isoform X2 | | |
| TRINITY_DN17761_c0_g1_i2_len_1030_95410.p1_type_complete_len_112 | ---NA--- | | |
| TRINITY_DN9886_c0_g1_i1_len_339_281691.p1_type_internal_len_113 | ---NA--- | | |
| TRINITY_DN13982_c0_g1_i1_len_339_249437.p1_type_internal_len_113 | ---NA--- | | |
| TRINITY_DN30581_c0_g1_i1_len_336_249632.p1_type_internal_len_113 | ---NA--- | | |
| TRINITY_DN3312_c0_g1_i1_len_337_44208.p1_type_internal_len_113 | ---NA--- | | |
| TRINITY_DN5988_c0_g1_i1_len_339_211460.p1_type_internal_len_113 | ---NA--- | | |
| TRINITY_DN23480_c0_g1_i7_len_2415_155148.p2_type_complete_len_112 | ---NA--- | | |

| | | | |
|---|---|---|---|
| TRINITY_DN16580_c0_g1_i1_len_551_67987.p1_type_complete_len_112 | ---NA--- | | |
| 2643\|_\|c9521_f2p0_2535_isoform_c9521_full_length_coverage_2_non_full_length_coverage_0_isoform_length_2535_21875 | XP_012135610.1 calcium-binding mitochondrial carrier protein Aralar1 isoform X5 | | |
| 2644\|_\|c11526_f2p0_2535_isoform_c11526_full_length_coverage_2_non_full_length_coverage_0_isoform_length_2535_12983 | ---NA--- | | |
| TRINITY_DN24952_c0_g1_i1_len_2719_229397.p1_type_3prime_partial_len_846 | XP_018027304.1 DNA N6-methyl adenine demethylase-like isoform X2 | | |
| 2647\|_\|c8686_f2p2_2534_isoform_c8686_full_length_coverage_2_non_full_length_coverage_2_isoform_length_2534_12741 | ---NA--- | | |
| 2648\|_\|c12894_f2p2_2534_isoform_c12894_full_length_coverage_2_non_full_length_coverage_2_isoform_length_2534_22175 | KZS08663.1DOMON domain-containing protein | | |
| 2649\|_\|c17989_f2p3_2533_isoform_c17989_full_length_coverage_2_non_full_length_coverage_3_isoform_length_2533_22613 | ---NA--- | | |
| 2650\|_\|c9042_f2p2_2533_isoform_c9042_full_length_coverage_2_non_full_length_coverage_2_isoform_length_2533_17248 | XP_023718552.1zinc finger protein 2-like isoform X4 | F:GO:0003676 | |
| 2651\|_\|c2787_f2p1_2533_isoform_c2787_full_length_coverage_2_non_full_length_coverage_1_isoform_length_2533_21235 | ---NA--- | | |
| 2652\|_\|c2667_f2p1_2533_isoform_c2667_full_length_coverage_2_non_full_length_coverage_1_isoform_length_2533_21133 | ---NA--- | | |
| TRINITY_DN18200_c0_g1_i1_len_2995_29486.p1_type_complete_len_844 | ---NA--- | | |
| TRINITY_DN34027_c0_g1_i1_len_339_211457.p1_type_internal_len_113 | XP_021938824.1eIF-2-alpha kinase GCN2 isoform X3 | | |

| | | | |
|---|---|---|---|
| TRINITY_DN36440_c0_g1_i1_len_336_249615.p1_type_internal_len_113 | ---NA--- | | |
| TRINITY_DN39521_c0_g1_i1_len_337_211561.p1_type_internal_len_113 | ---NA--- | | |
| TRINITY_DN16913_c0_g1_i1_len_1103_32982.p2_type_complete_len_112 | ---NA--- | | |
| TRINITY_DN20980_c0_g1_i2_len_2344_122604.p2_type_complete_len_112 | XP_018009117.1 protein tramtrack, beta isoform-like | | |
| TRINITY_DN12270_c0_g1_i1_len_337_44225.p1_type_internal_len_113 | ---NA--- | | |
| TRINITY_DN15124_c0_g1_i1_len_336_249609.p1_type_internal_len_113 | ---NA--- | | |
| TRINITY_DN19565_c0_g2_i1_len_339_211464.p1_type_5prime_partial_len_112 | ---NA--- | | |
| TRINITY_DN33780_c0_g1_i1_len_540_68185.p1_type_5prime_partial_len_112 | ---NA--- | | |
| TRINITY_DN18026_c0_g2_i1_len_539_100483.p2_type_complete_len_112 | ---NA--- | | |
| TRINITY_DN17169_c0_g1_i1_len_466_70092.p1_type_complete_len_112 | ---NA--- | | |
| TRINITY_DN3210_c0_g1_i1_len_502_167025.p1_type_complete_len_112 | ---NA--- | | |
| TRINITY_DN1606_c0_g1_i1_len_336_249624.p1_type_internal_len_113 | ---NA--- | | |
| TRINITY_DN1948_c0_g1_i1_len_336_249627.p1_type_internal_len_113 | ---NA--- | | |
| TRINITY_DN26875_c0_g1_i1_len_366_105832.p1_type_5prime_partial_len_112 | ---NA--- | | |
| TRINITY_DN3976_c0_g1_i1_len_381_73176.p1_type_5prime_partial_len_112 | XP_018027529.1 GPI mannosyltransferase 2-like | | |
| TRINITY_DN7795_c0_g1_i1_len_337_44201.p1_type_internal_len_113 | ---NA--- | | |

| | | | |
|---|---|---|---|
| TRINITY_DN38973_c0_g1_i1_len_433_206598.p1_type_5prime_partial_len_112 | ---NA--- | | |
| TRINITY_DN35581_c0_g1_i1_len_338_211475.p1_type_internal_len_113 | ---NA--- | | |
| TRINITY_DN1465_c0_g1_i1_len_1090_195765.p1_type_3prime_partial_len_113 | ---NA--- | | |
| TRINITY_DN29551_c0_g1_i1_len_337_249565.p1_type_internal_len_113 | ---NA--- | | |
| TRINITY_DN1789_c1_g1_i1_len_337_249560.p1_type_internal_len_113 | XP_018021807.1 E3 ubiquitin-protein ligase UBR4-like | | |
| TRINITY_DN34377_c0_g1_i1_len_337_249575.p1_type_internal_len_113 | ---NA--- | | |
| TRINITY_DN22929_c0_g1_i2_len_1665_232260.p2_type_complete_len_112 | ---NA--- | | |
| TRINITY_DN13832_c0_g1_i2_len_337_249574.p1_type_internal_len_113 | ---NA--- | | |
| TRINITY_DN28919_c0_g1_i1_len_337_281763.p1_type_internal_len_113 | KZS09868.1DDB1- and CUL4-associated factor 10 | | |
| TRINITY_DN27403_c0_g1_i1_len_338_249538.p1_type_internal_len_113 | ---NA--- | | |
| TRINITY_DN35423_c0_g1_i1_len_338_249537.p1_type_internal_len_113 | ---NA--- | | |
| TRINITY_DN11584_c0_g1_i1_len_338_249532.p1_type_internal_len_113 | ---NA--- | | |
| TRINITY_DN36268_c0_g1_i1_len_338_211478.p1_type_internal_len_113 | ---NA--- | | |
| TRINITY_DN26449_c0_g1_i2_len_337_281782.p2_type_internal_len_113 | ---NA--- | | |
| TRINITY_DN6250_c0_g1_i1_len_339_211467.p1_type_internal_len_113 | ---NA--- | | |
| TRINITY_DN16766_c0_g1_i1_len_1042_160354.p2_type_complete_len_112 | ---NA--- | | |

| | | | |
|---|---|---|---|
| TRINITY_DN23131_c0_g1_i2_len_568_202461.p1_type_5prime_partial_len_112 | ---NA--- | | |
| TRINITY_DN20310_c0_g1_i2_len_699_239208.p2_type_5prime_partial_len_112 | ---NA--- | | |
| TRINITY_DN41088_c0_g1_i1_len_336_211623.p1_type_internal_len_113 | XP_018009502.1 multidrug resistance-associated protein 5-like | | |
| TRINITY_DN33263_c0_g1_i1_len_336_249602.p1_type_internal_len_113 | ACL00586.1prophenoloxidase 2 | F:GO:0016491; F:GO:0046872; P:GO:0055114 | |
| TRINITY_DN18028_c0_g1_i1_len_826_4658.p2_type_5prime_partial_len_112 | ---NA--- | | |
| TRINITY_DN15531_c0_g1_i1_len_1224_2538.p3_type_5prime_partial_len_112 | ---NA--- | | |
| TRINITY_DN11112_c0_g1_i1_len_337_281774.p1_type_internal_len_113 | ---NA--- | | |
| TRINITY_DN32855_c0_g1_i1_len_376_105346.p1_type_5prime_partial_len_112 | ---NA--- | | |
| TRINITY_DN29325_c0_g1_i1_len_337_249578.p1_type_internal_len_113 | ---NA--- | | |
| TRINITY_DN3325_c0_g1_i1_len_337_249588.p1_type_internal_len_113 | ---NA--- | | |
| TRINITY_DN9650_c0_g1_i1_len_337_249584.p1_type_internal_len_113 | ---NA--- | | |
| TRINITY_DN7855_c0_g1_i1_len_470_167860.p1_type_complete_len_112 | ---NA--- | | |
| TRINITY_DN25768_c0_g1_i1_len_2623_229564.p4_type_complete_len_112 | ---NA--- | | |
| TRINITY_DN30998_c0_g1_i3_len_338_211535.p2_type_internal_len_113 | ---NA--- | | |
| TRINITY_DN26592_c0_g1_i1_len_346_172688.p1_type_5prime_partial_len_112 | XP_018013069.1 transient receptor potential protein-like | F:GO:0005262; C:GO:0016021; P:GO:0070588 | |
| TRINITY_DN32419_c0_g1_i1_len_338_173078.p2_type_internal_len_113 | ---NA--- | | |

| | | | |
|---|---|---|---|
| TRINITY_DN26849_c0_g1_i1_len_338_173077.p1_type_internal_len_113 | PSN47158.1Histone deacetylase 4 | C:GO:0000118; F:GO:0032041; F:GO:0046872; P:GO:0070932 | EC:3.5.1.98 |
| TRINITY_DN8047_c0_g1_i2_len_485_39036.p1_type_5prime_partial_len_112 | ---NA--- | | |
| TRINITY_DN23871_c0_g1_i1_len_2038_190769.p2_type_3prime_partial_len_113 | ---NA--- | | |
| TRINITY_DN2869_c0_g1_i1_len_338_173099.p1_type_internal_len_113 | ---NA--- | | |
| TRINITY_DN5610_c0_g1_i1_len_338_173096.p1_type_internal_len_113 | ---NA--- | | |
| TRINITY_DN11109_c0_g1_i2_len_338_173095.p1_type_internal_len_113 | XP_021003064.1phosphatidylinositol 3-kinase regulatory subunit alpha | | |
| TRINITY_DN27018_c0_g1_i1_len_338_173086.p2_type_internal_len_113 | ---NA--- | | |
| TRINITY_DN30337_c0_g1_i1_len_337_138423.p1_type_internal_len_113 | ---NA--- | | |
| TRINITY_DN9504_c0_g1_i1_len_339_173056.p1_type_internal_len_113 | ---NA--- | | |
| TRINITY_DN35459_c0_g1_i1_len_338_75467.p3_type_internal_len_113 | ASJ26360.1IL-16-like protein | | |
| TRINITY_DN38778_c0_g1_i1_len_338_211522.p1_type_internal_len_113 | ---NA--- | | |
| TRINITY_DN7822_c0_g1_i1_len_441_133918.p1_type_5prime_partial_len_112 | ---NA--- | | |
| TRINITY_DN28773_c0_g1_i1_len_338_173076.p1_type_internal_len_113 | ---NA--- | | |
| TRINITY_DN31992_c0_g1_i1_len_337_138429.p1_type_internal_len_113 | XP_018023298.1 CSC1-like protein 2 | C:GO:0016020 | |
| TRINITY_DN7165_c0_g1_i2_len_358_308762.p1_type_5prime_partial_len_112 | ---NA--- | | |
| TRINITY_DN26935_c0_g1_i1_len_337_173163.p1_type_internal_len_113 | XP_018016913.1PREDICTED: uncharacterized protein LOC108673573 | | |

| | | | |
|---|---|---|---|
| TRINITY_DN13191_c0_g1_i1_len_337_138451.p2_type_internal_len_113 | ---NA--- | | |
| TRINITY_DN2028_c0_g1_i1_len_336_173198.p1_type_internal_len_113 | ---NA--- | | |
| TRINITY_DN26858_c0_g1_i1_len_339_138309.p1_type_internal_len_113 | ---NA--- | | |
| TRINITY_DN5198_c0_g1_i1_len_337_211588.p1_type_internal_len_113 | ---NA--- | | |
| TRINITY_DN31062_c0_g2_i2_len_2047_190739.p2_type_3prime_partial_len_113 | ---NA--- | | |
| TRINITY_DN26730_c0_g1_i1_len_338_173116.p3_type_internal_len_113 | ---NA--- | | |
| TRINITY_DN23129_c0_g1_i1_len_968_196827.p1_type_complete_len_112 | ---NA--- | | |
| TRINITY_DN31825_c0_g1_i1_len_338_173111.p1_type_internal_len_113 | XP_018015698.1 plexin-B-like | P:GO:0007165 | |
| TRINITY_DN13927_c0_g1_i1_len_621_99109.p2_type_5prime_partial_len_112 | ---NA--- | | |
| TRINITY_DN17045_c0_g1_i1_len_338_211530.p3_type_internal_len_113 | ---NA--- | | |
| TRINITY_DN6754_c0_g1_i1_len_338_75461.p1_type_internal_len_113 | XP_018011507.1 SLIT-ROBO Rho GTPase-activating protein 1-like | | |
| TRINITY_DN19179_c0_g1_i1_len_1171_94628.p2_type_5prime_partial_len_112 | ---NA--- | | |
| TRINITY_DN27038_c0_g1_i1_len_2869_188750.p2_type_3prime_partial_len_113 | ---NA--- | | |
| TRINITY_DN24068_c0_g1_i2_len_2645_122329.p1_type_complete_len_112 | ---NA--- | | |
| TRINITY_DN12481_c0_g1_i1_len_411_207552.p1_type_5prime_partial_len_112 | ---NA--- | | |
| TRINITY_DN19685_c0_g1_i1_len_415_207357.p1_type_5prime_partial_len_112 | ---NA--- | | |

| | | | |
|---|---|---|---|
| TRINITY_DN2202_c0_g2_i1_len_338_75458.p1_type_internal_len_113 | ---NA--- | | |
| TRINITY_DN30436_c0_g1_i1_len_1775_231842.p2_type_3prime_partial_len_113 | AAF43383.1NADH dehydrogenase subunit 5 (mitochondrion) | C:GO:0005743; F:GO:0008137; C:GO:0016021; P:GO:0042773; C:GO:0070469 | EC:1.6.99.5; EC:1.6.99.3; EC:1.6.5.3 |
| TRINITY_DN7531_c0_g1_i2_len_338_138404.p1_type_internal_len_113 | ---NA--- | | |
| TRINITY_DN25132_c0_g1_i1_len_338_75452.p1_type_internal_len_113 | ---NA--- | | |
| TRINITY_DN5492_c0_g1_i1_len_339_173012.p1_type_internal_len_113 | ---NA--- | | |
| TRINITY_DN9475_c0_g1_i1_len_339_173011.p1_type_internal_len_113 | ---NA--- | | |
| TRINITY_DN10937_c0_g1_i1_len_582_202151.p1_type_5prime_partial_len_112 | ---NA--- | | |
| TRINITY_DN9004_c0_g1_i1_len_337_211581.p1_type_internal_len_113 | ---NA--- | | |
| TRINITY_DN4335_c0_g1_i1_len_436_103099.p1_type_5prime_partial_len_112 | ---NA--- | | |
| TRINITY_DN41379_c0_g1_i1_len_338_211509.p1_type_internal_len_113 | PIK54138.1Retrovirus-related Pol polyprotein from transposon | | |
| TRINITY_DN25320_c0_g1_i1_len_398_104454.p1_type_5prime_partial_len_112 | ---NA--- | | |
| TRINITY_DN23426_c0_g1_i6_len_2889_58252.p5_type_complete_len_112 | AGN52246.1female sterile homeotic protein | | |
| TRINITY_DN30107_c0_g1_i1_len_1547_192848.p1_type_complete_len_112 | ---NA--- | | |
| TRINITY_DN2647_c0_g1_i1_len_340_249347.p1_type_5prime_partial_len_112 | ---NA--- | | |
| TRINITY_DN10796_c0_g1_i1_len_1786_191715.p3_type_complete_len_112 | XP_013860411.1 zinc finger protein OZF-like | F:GO:0003676 | |
| TRINITY_DN4404_c0_g1_i1_len_337_14569.p1_type_internal_len_113 | ---NA--- | | |

| | | | |
|---|---|---|---|
| TRINITY_DN5750_c0_g1_i1_len_337_211538.p1_type_internal_len_113 | ---NA--- | | |
| TRINITY_DN35802_c0_g1_i1_len_337_211577.p1_type_internal_len_113 | ---NA--- | | |
| TRINITY_DN15171_c0_g1_i2_len_336_138487.p1_type_internal_len_113 | ---NA--- | | |
| 2655\|_\|c28263_f2p1_2532_isoform_c28263_full_length_coverage_2_non_full_length_coverage_1_isoform_length_2532_23077 | XP_011788239.1 endonuclease 8-like 1 | F:GO:0003676; F:GO:0003684; F:GO:0003906; C:GO:0005634; C:GO:0005654; C:GO:0005737; P:GO:0006281; P:GO:0006284; P:GO:0006289; P:GO:0006979; F:GO:0008022; F:GO:0008270; F:GO:0016798; F:GO:0016799; F:GO:0016829; F:GO:0019104; P:GO:0032074 | |
| 2656\|_\|c2632_f3p0_2531_isoform_c2632_full_length_coverage_3_non_full_length_coverage_0_isoform_length_2531_21103 | XP_018019263.1 josephin-1-like | F:GO:0016787 | |
| 2657\|_\|c17828_f3p1_2531_isoform_c17828_full_length_coverage_3_non_full_length_coverage_1_isoform_length_2531_17807 | XP_018025014.1 fukutin-related protein-like | C:GO:0016020; C:GO:0016021 | |
| 2659\|_\|c2081_f9p5_2529_isoform_c2081_full_length_coverage_9_non_full_length_coverage_5_isoform_length_2529_16174 | XP_023725279.1protein SMG9 isoform X2 | P:GO:0000184 | |
| 2660\|_\|c6145_f2p1_2529_isoform_c6145_full_length_coverage_2_non_full_length_coverage_1_isoform_length_2529_17024 | ---NA--- | | |
| 2661\|_\|c1206_f4p6_2529_isoform_c1206_full_length_coverage_4_non_full_length_coverage_6_isoform_length_2529_25353 | ---NA--- | | |
| 2662\|_\|c2006_f8p2_2528_isoform_c2006_full_length_coverage_8_non_full_length_coverage_2_isoform_length_2528_16121 | XP_025412858.1H/ACA ribonucleoprotein complex non-core subunit NAF1 isoform X2 | P:GO:0009987; P:GO:0022613 | |

| | | | |
|---|---|---|---|
| 2664\|_\|c3141_f2p3_2527_isoform_c3141_full_length_coverage_2_non_full_length_coverage_3_isoform_length_2527_12271 | XP_014293912.1BTB/POZ domain-containing protein KCTD9 | P:GO:0035556; P:GO:0051260 | |
| 2667\|_\|c8547_f2p4_2527_isoform_c8547_full_length_coverage_2_non_full_length_coverage_4_isoform_length_2527_17210 | KDR23413.1Neither inactivation nor afterpotential protein G | C:GO:0016020; C:GO:0016021; F:GO:0016614; F:GO:0050660; P:GO:0055114 | |
| 2669\|_\|c26511_f2p3_2526_isoform_c26511_full_length_coverage_2_non_full_length_coverage_3_isoform_length_2526_18396 | XP_023712159.1TBC1 domain family member 13 isoform X1 | | |
| TRINITY_DN19086_c0_g1_i1_len_1731_191950.p1_type_5prime_partial_len_112 | ---NA--- | | |
| TRINITY_DN31694_c0_g1_i1_len_340_249391.p1_type_internal_len_113 | OWF41021.1Intraflagellar transport protein 140 | C:GO:0031514 | |
| TRINITY_DN19660_c0_g1_i1_len_723_199680.p2_type_5prime_partial_len_112 | ---NA--- | | |
| TRINITY_DN36647_c0_g1_i1_len_340_249385.p1_type_internal_len_113 | ---NA--- | | |
| TRINITY_DN23589_c0_g1_i1_len_605_240715.p1_type_complete_len_112 | ---NA--- | | |
| TRINITY_DN28158_c0_g1_i1_len_336_138513.p1_type_internal_len_113 | ---NA--- | | |
| TRINITY_DN8282_c0_g1_i1_len_338_211507.p1_type_internal_len_113 | AGY46351.1domeless | C:GO:0016021 | |
| TRINITY_DN12573_c0_g1_i1_len_646_200880.p2_type_complete_len_112 | ---NA--- | | |
| TRINITY_DN14123_c0_g1_i2_len_506_304021.p2_type_5prime_partial_len_112 | XP_014298367.1 afadin-like isoform X2 | C:GO:0005911; P:GO:0007165 | |
| TRINITY_DN33485_c0_g1_i1_len_338_211518.p1_type_internal_len_113 | XP_022122563.1 hormone-sensitive lipase | P:GO:0008203; P:GO:0016042; F:GO:0016298; F:GO:0016787 | |
| TRINITY_DN30143_c0_g1_i1_len_426_278070.p1_type_5prime_partial_len_112 | KZS14347.1Xenotropic and polytropic retrovirus receptor 1 | C:GO:0016021 | |
| TRINITY_DN14980_c0_g1_i1_len_339_75372.p1_type_internal_len_113 | ---NA--- | | |

| | | | |
|---|---|---|---|
| TRINITY_DN13464_c0_g1_i2_len_337_138456.p2_type_internal_len_113 | ELU13343.1hypothetical protein CAPTEDRAFT_91049, partial | | |
| TRINITY_DN21747_c0_g1_i1_len_662_239756.p1_type_5prime_partial_len_112 | ---NA--- | | |
| TRINITY_DN24523_c0_g1_i1_len_336_173214.p1_type_internal_len_113 | ---NA--- | | |
| TRINITY_DN20990_c0_g1_i1_len_1140_235103.p4_type_5prime_partial_len_112 | ---NA--- | | |
| TRINITY_DN28521_c0_g1_i1_len_409_104033.p1_type_5prime_partial_len_112 | ---NA--- | | |
| TRINITY_DN33439_c0_g1_i1_len_339_75350.p2_type_internal_len_113 | ---NA--- | | |
| TRINITY_DN7673_c0_g1_i1_len_349_281136.p1_type_5prime_partial_len_112 | ---NA--- | | |
| TRINITY_DN31667_c0_g1_i1_len_339_75352.p1_type_internal_len_113 | ---NA--- | | |
| TRINITY_DN18517_c0_g1_i1_len_336_138467.p1_type_internal_len_113 | ---NA--- | | |
| TRINITY_DN13777_c0_g1_i1_len_408_169898.p1_type_3prime_partial_len_113 | ---NA--- | | |
| TRINITY_DN7043_c0_g1_i1_len_338_211513.p1_type_internal_len_113 | XP_024085611.1glutamate--cysteine ligase catalytic subunit | | |
| TRINITY_DN5048_c0_g1_i1_len_337_211595.p1_type_internal_len_113 | ---NA--- | | |
| TRINITY_DN39972_c0_g1_i1_len_338_211516.p2_type_internal_len_113 | XP_001605902.2 GTPase-activating protein and VPS9 domain-containing protein 1 isoform X1 | P:GO:0065007 | |
| TRINITY_DN20482_c0_g1_i8_len_3141_264859.p2_type_complete_len_112 | ---NA--- | | |
| TRINITY_DN36388_c0_g1_i1_len_357_248429.p1_type_5prime_partial_len_112 | ---NA--- | | |
| TRINITY_DN23280_c1_g1_i1_len_4356_228002.p3_type_3prime_partial_len_113 | ---NA--- | | |

| | | | |
|---|---|---|---|
| TRINITY_DN30855_c0_g1_i1_len_412_278574.p1_type_5prime_partial_len_112 | ABQ41245.1male reproductive-related LIM protein | F:GO:0046872 | |
| TRINITY_DN25385_c0_g1_i1_len_1126_195482.p1_type_complete_len_112 | XP_014674141.1 transmembrane protein 243-like | C:GO:0016020 | |
| TRINITY_DN21178_c0_g1_i1_len_1199_2640.p1_type_complete_len_112 | ---NA--- | | |
| TRINITY_DN41254_c0_g1_i1_len_339_211432.p1_type_internal_len_113 | PSN41591.1hypothetical protein C0J52_17617 | F:GO:0005509 | |
| TRINITY_DN13610_c0_g1_i2_len_339_211426.p1_type_internal_len_113 | ---NA--- | | |
| TRINITY_DN13442_c0_g1_i1_len_604_274089.p1_type_5prime_partial_len_112 | ACR54131.1NADH dehydrogenase | C:GO:0005747; P:GO:0022900 | |
| TRINITY_DN21378_c0_g1_i2_len_531_203395.p1_type_5prime_partial_len_112 | ---NA--- | | |
| TRINITY_DN1018_c0_g1_i1_len_347_210924.p1_type_5prime_partial_len_112 | ---NA--- | | |
| TRINITY_DN22719_c0_g1_i5_len_707_128943.p2_type_complete_len_112 | ---NA--- | | |
| TRINITY_DN22910_c0_g1_i1_len_787_198768.p1_type_complete_len_112 | ---NA--- | | |
| TRINITY_DN8838_c0_g1_i1_len_699_98103.p2_type_5prime_partial_len_112 | ---NA--- | | |
| TRINITY_DN26430_c0_g1_i1_len_426_245414.p1_type_3prime_partial_len_113 | XP_018015378.1 GTP-binding protein 2-like | | |
| TRINITY_DN17578_c0_g1_i1_len_339_211420.p1_type_internal_len_113 | XP_024946536.1uncharacterized protein LOC107273441 isoform X2 | | |
| TRINITY_DN31705_c0_g1_i1_len_508_203977.p1_type_3prime_partial_len_113 | ---NA--- | | |
| TRINITY_DN18981_c0_g1_i1_len_525_242396.p1_type_3prime_partial_len_113 | ---NA--- | | |
| TRINITY_DN20835_c0_g1_i1_len_790_238066.p1_type_complete_len_112 | ---NA--- | | |

| | | | |
|---|---|---|---|
| TRINITY_DN7068_c0_g1_i1_len_384_307635.p1_type_3prime_partial_len_113 | ---NA--- | | |
| TRINITY_DN30929_c0_g1_i1_len_422_245556.p1_type_3prime_partial_len_113 | KZS20145.1Protein SZT2 | C:GO:0005777 | |
| TRINITY_DN19113_c0_g1_i1_len_784_64737.p1_type_5prime_partial_len_112 | XP_018014480.1PREDICTED: uncharacterized protein LOC108671443 | | |
| TRINITY_DN23379_c0_g1_i1_len_1628_157321.p3_type_5prime_partial_len_112 | ---NA--- | | |
| TRINITY_DN18342_c0_g1_i2_len_811_64492.p2_type_complete_len_112 | ---NA--- | | |
| TRINITY_DN31674_c0_g1_i1_len_338_107284.p1_type_internal_len_113 | ---NA--- | | |
| TRINITY_DN15998_c0_g1_i1_len_828_237667.p1_type_complete_len_112 | ---NA--- | | |
| TRINITY_DN9430_c0_g2_i1_len_339_211435.p1_type_internal_len_113 | PFX24919.1putative RNA-directed DNA polymerase from transposon BS | F:GO:0003964; P:GO:0006278 | |
| TRINITY_DN20065_c0_g1_i2_len_2010_266279.p2_type_5prime_partial_len_112 | ---NA--- | | |
| TRINITY_DN16380_c0_g1_i1_len_746_162965.p2_type_5prime_partial_len_112 | ---NA--- | | |
| TRINITY_DN2854_c0_g1_i1_len_439_206360.p1_type_5prime_partial_len_112 | ---NA--- | | |
| TRINITY_DN6580_c0_g1_i1_len_383_279637.p1_type_5prime_partial_len_112 | ---NA--- | | |
| TRINITY_DN17225_c0_g1_i1_len_1393_31812.p2_type_5prime_partial_len_112 | ---NA--- | | |
| TRINITY_DN16718_c0_g1_i1_len_337_107325.p1_type_internal_len_113 | ---NA--- | | |
| TRINITY_DN29709_c0_g1_i1_len_337_107359.p1_type_internal_len_113 | XP_006751844.1 huntingtin-interacting protein 1-like | F:GO:0005515; P:GO:0006897; P:GO:0009987 | |
| TRINITY_DN9371_c0_g1_i1_len_404_72164.p1_type_5prime_partial_len_112 | ---NA--- | | |

| | | | |
|---|---|---|---|
| TRINITY_DN23580_c0_g1_i2_len_4693_153494.p3_type_complete_len_112 | ---NA--- | | |
| TRINITY_DN41227_c0_g1_i1_len_339_211396.p1_type_internal_len_113 | ---NA--- | | |
| TRINITY_DN12036_c0_g1_i1_len_1280_234177.p2_type_complete_len_112 | ---NA--- | | |
| TRINITY_DN39986_c0_g1_i1_len_339_211400.p1_type_internal_len_113 | XP_018899722.1 zinc finger and SCAN domain-containing protein 2-like | | |
| TRINITY_DN3227_c0_g1_i1_len_638_201030.p2_type_complete_len_112 | KPJ11974.1Oxysterol-binding protein-related protein 1 | P:GO:0006869 | |
| TRINITY_DN37146_c0_g1_i1_len_367_209710.p1_type_5prime_partial_len_112 | KRX83908.1hypothetical protein T4E_7988, partial | | |
| TRINITY_DN29557_c0_g1_i1_len_473_243817.p1_type_5prime_partial_len_112 | ---NA--- | | |
| TRINITY_DN22772_c0_g1_i1_len_736_272408.p1_type_complete_len_112 | ---NA--- | | |
| TRINITY_DN17003_c0_g1_i1_len_807_127993.p1_type_5prime_partial_len_112 | ---NA--- | | |
| TRINITY_DN10966_c0_g1_i1_len_517_242589.p1_type_3prime_partial_len_113 | ---NA--- | | |
| TRINITY_DN18552_c0_g1_i1_len_642_273536.p1_type_5prime_partial_len_112 | ---NA--- | | |
| TRINITY_DN4048_c0_g1_i1_len_518_242583.p1_type_complete_len_112 | ---NA--- | | |
| TRINITY_DN2209_c0_g1_i1_len_340_211389.p1_type_internal_len_113 | ---NA--- | | |
| TRINITY_DN1862_c0_g1_i1_len_339_309796.p1_type_internal_len_113 | ---NA--- | | |
| TRINITY_DN25959_c0_g1_i3_len_778_238208.p1_type_3prime_partial_len_113 | ---NA--- | | |
| TRINITY_DN301_c0_g1_i1_len_339_211411.p1_type_internal_len_113 | ---NA--- | | |

| | | | |
|---|---|---|---|
| TRINITY_DN117_c0_g1_i1_len_339_211403.p1_type_internal_len_113 | ---NA--- | | |
| TRINITY_DN497_c0_g1_i1_len_340_309734.p1_type_internal_len_113 | ---NA--- | | |
| TRINITY_DN35747_c0_g1_i1_len_339_211404.p1_type_internal_len_113 | ---NA--- | | |
| TRINITY_DN11744_c0_g1_i1_len_779_64791.p2_type_5prime_partial_len_112 | ---NA--- | | |
| TRINITY_DN15869_c0_g1_i1_len_1313_233972.p1_type_5prime_partial_len_112 | ---NA--- | | |
| TRINITY_DN16590_c0_g1_i1_len_435_71073.p1_type_5prime_partial_len_112 | ---NA--- | | |
| TRINITY_DN27800_c0_g1_i1_len_339_309751.p1_type_internal_len_113 | ---NA--- | | |
| TRINITY_DN11444_c0_g1_i1_len_450_244588.p1_type_complete_len_112 | ---NA--- | | |
| TRINITY_DN31405_c0_g1_i1_len_337_309873.p1_type_internal_len_113 | PIO74628.1nicotinate phosphoribosyltransferase, (NAPRTase) family | F:GO:0004514; P:GO:0009435 | EC:2.4.2.19 |
| TRINITY_DN15080_c0_g1_i2_len_1547_31329.p2_type_5prime_partial_len_112 | XP_018018015.1 mannose-1-phosphate guanyltransferase alpha-like isoform X2 | P:GO:0009058; F:GO:0016779 | |
| TRINITY_DN23927_c0_g1_i2_len_1435_60944.p3_type_5prime_partial_len_112 | ---NA--- | | |
| TRINITY_DN27166_c0_g1_i1_len_2244_190117.p1_type_5prime_partial_len_112 | ---NA--- | | |
| TRINITY_DN1850_c0_g1_i2_len_587_37151.p2_type_5prime_partial_len_112 | AKV88638.1TGF-beta-activated kinase 1 and MAP3K7-binding protein 2 | F:GO:0016301; P:GO:0016310; F:GO:0046872 | |
| TRINITY_DN35963_c0_g1_i1_len_336_211651.p1_type_internal_len_113 | ---NA--- | | |
| TRINITY_DN5814_c0_g1_i1_len_434_206547.p1_type_3prime_partial_len_113 | PSN44076.1Four and a half LIM domains protein 2 | F:GO:0046872 | |
| TRINITY_DN18851_c0_g1_i1_len_1576_232660.p1_type_complete_len_112 | ---NA--- | | |

| | | | |
|---|---|---|---|
| TRINITY_DN22932_c0_g1_i1_len_1366_233689.p1_type_complete_len_112 | ---NA--- | | |
| TRINITY_DN8075_c0_g1_i1_len_400_246394.p1_type_5prime_partial_len_112 | ---NA--- | | |
| TRINITY_DN32238_c0_g1_i1_len_341_211281.p1_type_5prime_partial_len_112 | ---NA--- | | |
| TRINITY_DN32612_c0_g1_i1_len_541_166155.p1_type_5prime_partial_len_112 | ---NA--- | | |
| TRINITY_DN24243_c0_g1_i1_len_416_40945.p2_type_5prime_partial_len_112 | BAD34945.1serine proteinase homologue | F:GO:0004252; P:GO:0006508 | EC:3.4.21 |
| TRINITY_DN18794_c0_g2_i1_len_399_11779.p1_type_5prime_partial_len_112 | XP_023966522.1histone H4-like | C:GO:0000786; F:GO:0003677; C:GO:0005634; P:GO:0006352; F:GO:0046982 | |
| TRINITY_DN29259_c0_g1_i1_len_391_246765.p1_type_3prime_partial_len_113 | ---NA--- | | |
| TRINITY_DN8872_c0_g1_i1_len_346_309397.p1_type_5prime_partial_len_112 | ---NA--- | | |
| TRINITY_DN22669_c0_g1_i1_len_6252_227576.p1_type_complete_len_842 | XP_023702596.1catenin delta-2 isoform X5 | | |
| TRINITY_DN24693_c0_g1_i1_len_2929_229086.p1_type_5prime_partial_len_842 | XP_003708110.1 WD repeat-containing protein 75 | | |
| 2673\|_\|c2542_f2p29_2526_isoform_c2542_full_length_coverage_2_non_full_length_coverage_29_isoform_length_2526_11955 | ---NA--- | | |
| TRINITY_DN22241_c0_g1_i4_len_3225_154113.p1_type_5prime_partial_len_842 | PNF27521.1WD repeat-containing protein 44 | | |
| TRINITY_DN27178_c0_g1_i1_len_2738_188976.p1_type_5prime_partial_len_842 | ---NA--- | | |
| TRINITY_DN24678_c0_g1_i2_len_2784_229307.p1_type_5prime_partial_len_842 | ---NA--- | | |
| 2677\|_\|c10261_f2p1_2525_isoform_c10261_full_length_coverage_2_non_full_length_coverage_1_isoform_length_2525_21933 | ---NA--- | | |

| | | | |
|---|---|---|---|
| 2678\|_\|c11967_f4p2_2525_isoform_c11967_full_length_coverage_4_non_full_length_coverage_2_isoform_length_2525_13029 | XP_013415078.1condensin complex subunit 1-like isoform X2 | C:GO:0005634; P:GO:0007076 | |
| 2679\|_\|c2381_f2p4_2525_isoform_c2381_full_length_coverage_2_non_full_length_coverage_4_isoform_length_2525_16368 | ---NA--- | | |
| 2680\|_\|c17828_f3p3_2525_isoform_c17828_full_length_coverage_3_non_full_length_coverage_3_isoform_length_2525_13430 | ---NA--- | | |
| TRINITY_DN14219_c0_g1_i1_len_2179_190313.p1_type_5prime_partial_len_112 | ---NA--- | | |
| TRINITY_DN27533_c0_g1_i1_len_336_14643.p1_type_internal_len_113 | ---NA--- | | |
| TRINITY_DN13386_c0_g1_i2_len_506_242888.p2_type_5prime_partial_len_112 | XP_020285487.1ATP-citrate synthase | | |
| TRINITY_DN1564_c0_g1_i1_len_396_246561.p1_type_3prime_partial_len_113 | PIK58224.1putative 5'-3' exoribonuclease 1-like | P:GO:0000723; C:GO:0000932; F:GO:0002151; F:GO:0004534; F:GO:0005515; C:GO:0005634; C:GO:0005829; C:GO:0005886; P:GO:0006396; P:GO:0007569; P:GO:0016075; P:GO:0017148; C:GO:0030425; C:GO:0043025; C:GO:0045202; P:GO:0051321; F:GO:0051880; P:GO:0071028; P:GO:0071044; P:GO:0090503 | EC:3.1.13; EC:3.1.15 |
| TRINITY_DN3792_c0_g1_i1_len_338_44162.p1_type_internal_len_113 | ---NA--- | | |
| TRINITY_DN12604_c0_g1_i1_len_395_246610.p1_type_3prime_partial_len_113 | KDR20207.1WD repeat domain phosphoinositide-interacting protein 4 | P:GO:0006914 | |
| TRINITY_DN40251_c0_g1_i1_len_336_211664.p2_type_internal_len_113 | ---NA--- | | |
| TRINITY_DN36273_c0_g1_i1_len_401_207985.p1_type_5prime_partial_len_112 | ---NA--- | | |

| | | | |
|---|---|---|---|
| TRINITY_DN902_c0_g1_i1_len_345_43810.p1_type_5prime_partial_len_112 | AAL96263.1immunoglobulin gamma-1 heavy chain constant region | | |
| TRINITY_DN29119_c0_g1_i1_len_392_72710.p1_type_5prime_partial_len_112 | AUC64084.1G-protein-coupled receptor kinase 2 | F:GO:0004703; F:GO:0005524; P:GO:0006468; P:GO:0007165 | EC:2.7.11; EC:2.7.11.16 |
| TRINITY_DN15416_c0_g1_i1_len_440_206328.p1_type_5prime_partial_len_112 | ---NA--- | | |
| TRINITY_DN1510_c0_g1_i1_len_339_14479.p1_type_5prime_partial_len_112 | ---NA--- | | |
| TRINITY_DN32850_c0_g1_i1_len_405_246212.p1_type_3prime_partial_len_113 | ---NA--- | | |
| TRINITY_DN15216_c0_g1_i1_len_414_278493.p1_type_5prime_partial_len_112 | ---NA--- | | |
| TRINITY_DN29388_c0_g1_i1_len_370_42640.p1_type_5prime_partial_len_112 | ---NA--- | | |
| TRINITY_DN14495_c0_g1_i3_len_549_165970.p1_type_3prime_partial_len_113 | ---NA--- | | |
| TRINITY_DN17499_c0_g1_i1_len_435_206522.p1_type_3prime_partial_len_113 | ---NA--- | | |
| TRINITY_DN18315_c0_g1_i1_len_336_75564.p1_type_internal_len_112 | ---NA--- | | |
| TRINITY_DN17375_c0_g1_i1_len_1537_157648.p1_type_complete_len_111 | ---NA--- | | |
| TRINITY_DN32623_c0_g1_i1_len_335_138546.p1_type_internal_len_112 | CRK94795.1CLUMA_CG008289, isoform A | F:GO:0005488; C:GO:0005634; F:GO:0018024; P:GO:0034968 | EC:2.1.1.43 |
| TRINITY_DN16190_c0_g1_i2_len_578_302698.p1_type_complete_len_111 | ---NA--- | | |
| TRINITY_DN5929_c0_g1_i1_len_336_249607.p1_type_internal_len_112 | ---NA--- | | |
| TRINITY_DN1370_c0_g1_i1_len_681_129223.p1_type_5prime_partial_len_111 | ---NA--- | | |
| TRINITY_DN40127_c0_g1_i1_len_334_211731.p1_type_internal_len_112 | ---NA--- | | |

| | | | |
|---|---|---|---|
| TRINITY_DN23567_c1_g1_i3_len_335_249684.p3_type_internal_len_112 | OWF38525.1Stress protein DDR48 | C:GO:0016020; C:GO:0016021 | |
| TRINITY_DN21894_c0_g1_i2_len_752_300582.p2_type_5prime_partial_len_111 | ---NA--- | | |
| TRINITY_DN12329_c0_g1_i1_len_336_249613.p1_type_internal_len_112 | ---NA--- | | |
| TRINITY_DN32717_c0_g1_i1_len_335_211697.p4_type_internal_len_112 | ---NA--- | | |
| TRINITY_DN10403_c0_g1_i1_len_336_138504.p1_type_internal_len_112 | ---NA--- | | |
| TRINITY_DN13842_c0_g1_i1_len_333_75752.p1_type_internal_len_112 | ---NA--- | | |
| TRINITY_DN1120_c0_g1_i1_len_336_249654.p1_type_internal_len_112 | ---NA--- | | |
| TRINITY_DN21211_c0_g1_i1_len_1148_195315.p2_type_5prime_partial_len_111 | ---NA--- | | |
| TRINITY_DN15038_c0_g1_i1_len_589_302515.p1_type_complete_len_111 | ---NA--- | | |
| TRINITY_DN3654_c0_g1_i1_len_335_211714.p1_type_internal_len_112 | XP_018026075.1 protein ROP-like | | |
| TRINITY_DN26309_c0_g1_i1_len_334_138636.p2_type_internal_len_112 | XP_018019085.1 paramyosin, long form-like isoform X2 | F:GO:0003774; C:GO:0016459 | EC:3.6.1.15 |
| TRINITY_DN10960_c0_g1_i1_len_335_211729.p1_type_internal_len_112 | ---NA--- | | |
| TRINITY_DN23751_c0_g1_i1_len_741_163024.p1_type_complete_len_111 | ---NA--- | | |
| TRINITY_DN731_c0_g1_i1_len_336_75575.p1_type_internal_len_112 | XP_014786622.1 25-hydroxyvitamin D-1 alpha hydroxylase, mitochondrial-like | F:GO:0004497; F:GO:0005506; F:GO:0016491; F:GO:0016705; F:GO:0020037; F:GO:0046872; P:GO:0055114 | |
| TRINITY_DN23567_c1_g1_i3_len_335_249684.p4_type_internal_len_112 | OWF38525.1Stress protein DDR48 | C:GO:0016020; C:GO:0016021 | |

| | | | |
|---|---|---|---|
| TRINITY_DN40990_c0_g1_i1_len_351_210660.p1_type_5prime_partial_len_111 | ---NA--- | | |
| TRINITY_DN24963_c0_g1_i1_len_853_198008.p1_type_5prime_partial_len_111 | ---NA--- | | |
| TRINITY_DN7741_c0_g1_i5_len_335_211700.p1_type_internal_len_112 | ---NA--- | | |
| TRINITY_DN30371_c0_g1_i1_len_336_249622.p1_type_internal_len_112 | KFM59031.1Two pore calcium channel protein 1 | F:GO:0005245; F:GO:0005509; C:GO:0016021; P:GO:0070588 | |
| TRINITY_DN16825_c0_g1_i1_len_336_138529.p1_type_internal_len_112 | ---NA--- | | |
| TRINITY_DN6442_c0_g1_i1_len_335_211699.p2_type_internal_len_112 | ---NA--- | | |
| TRINITY_DN23567_c1_g1_i3_len_335_249684.p1_type_internal_len_112 | OWF38525.1Stress protein DDR48 | C:GO:0016020; C:GO:0016021 | |
| TRINITY_DN4875_c0_g1_i1_len_366_73889.p1_type_complete_len_111 | ---NA--- | | |
| TRINITY_DN107_c0_g1_i1_len_334_138629.p2_type_internal_len_112 | ---NA--- | | |
| TRINITY_DN11445_c0_g1_i1_len_334_211735.p1_type_internal_len_112 | AYD41581.1angiopoietin-2-like protein | | |
| TRINITY_DN32708_c0_g1_i1_len_336_75531.p2_type_internal_len_112 | ---NA--- | | |
| TRINITY_DN19905_c0_g1_i1_len_760_199121.p1_type_complete_len_111 | ---NA--- | | |
| TRINITY_DN969_c0_g1_i1_len_337_138426.p1_type_internal_len_112 | XP_018055614.1 transcription factor Sp4-like | F:GO:0003676 | |
| TRINITY_DN18085_c0_g1_i3_len_337_75487.p1_type_internal_len_112 | ---NA--- | | |
| TRINITY_DN23567_c1_g1_i3_len_335_249684.p2_type_internal_len_112 | OWF38525.1Stress protein DDR48 | C:GO:0016020; C:GO:0016021 | |
| TRINITY_DN18955_c0_g1_i1_len_1868_952.p3_type_complete_len_111 | ---NA--- | | |

| | | | |
|---|---|---|---|
| TRINITY_DN26734_c0_g1_i1_len_351_137658.p1_type_5prime_partial_len_111 | ---NA--- | | |
| TRINITY_DN7585_c0_g1_i1_len_337_211539.p1_type_internal_len_112 | PFX32598.1Retrovirus-related Pol polyprotein from transposon 17.6 | F:GO:0003676; P:GO:0015074 | |
| TRINITY_DN21334_c0_g1_i1_len_1402_124624.p2_type_complete_len_111 | ---NA--- | | |
| TRINITY_DN27819_c0_g1_i1_len_339_249466.p1_type_5prime_partial_len_111 | ---NA--- | | |
| TRINITY_DN10351_c0_g1_i1_len_333_138713.p1_type_internal_len_112 | ---NA--- | | |
| TRINITY_DN20953_c0_g1_i2_len_1015_196426.p1_type_5prime_partial_len_111 | ---NA--- | | |
| TRINITY_DN19377_c0_g1_i1_len_498_204311.p1_type_5prime_partial_len_111 | ---NA--- | | |
| TRINITY_DN26213_c0_g1_i1_len_334_138642.p2_type_internal_len_112 | ---NA--- | | |
| TRINITY_DN29591_c0_g1_i1_len_337_249577.p1_type_internal_len_112 | ---NA--- | | |
| TRINITY_DN6920_c0_g1_i1_len_337_249572.p1_type_internal_len_112 | ---NA--- | | |
| TRINITY_DN25058_c0_g1_i3_len_1014_196438.p1_type_5prime_partial_len_111 | ---NA--- | | |
| TRINITY_DN18608_c0_g1_i3_len_579_302673.p2_type_complete_len_111 | BAO96441.1prophenoloxidase beta | F:GO:0016491; F:GO:0046872; P:GO:0055114 | |
| TRINITY_DN9519_c0_g1_i1_len_1008_160608.p2_type_complete_len_111 | ---NA--- | | |
| TRINITY_DN31812_c0_g1_i1_len_335_211698.p1_type_internal_len_112 | XP_022199129.1protein unc-13 homolog A-like | | |
| TRINITY_DN27487_c0_g1_i1_len_336_249636.p1_type_internal_len_112 | XP_018012859.1 rab GTPase-activating protein 1-like | F:GO:0005096; P:GO:0043547 | |
| TRINITY_DN7735_c0_g1_i1_len_337_249567.p1_type_internal_len_112 | ESO95252.1hypothetical protein LOTGIDRAFT_144696 | C:GO:0005634; P:GO:0006342; C:GO:0016020 | |

| | | | |
|---|---|---|---|
| TRINITY_DN33093_c0_g1_i1_len_334_138643.p1_type_internal_len_112 | ---NA--- | | |
| TRINITY_DN22200_c0_g1_i1_len_1613_157372.p3_type_complete_len_111 | ---NA--- | | |
| TRINITY_DN23567_c1_g1_i3_len_335_249684.p5_type_internal_len_112 | OWF38525.1Stress protein DDR48 | C:GO:0016020; C:GO:0016021 | |
| TRINITY_DN2905_c0_g1_i1_len_337_75493.p1_type_internal_len_112 | ---NA--- | | |
| TRINITY_DN792_c0_g1_i2_len_335_211688.p1_type_internal_len_112 | ---NA--- | | |
| TRINITY_DN20397_c0_g1_i1_len_862_64051.p1_type_5prime_partial_len_111 | ---NA--- | | |
| TRINITY_DN35864_c0_g1_i1_len_336_249638.p1_type_internal_len_112 | XP_018014869.1 F-box/LRR-repeat protein 2-like isoform X2 | | |
| TRINITY_DN37840_c0_g1_i1_len_335_249679.p1_type_internal_len_112 | ---NA--- | | |
| TRINITY_DN31728_c0_g1_i1_len_336_138471.p1_type_internal_len_112 | ---NA--- | | |
| TRINITY_DN3489_c0_g1_i1_len_337_249568.p1_type_internal_len_112 | ---NA--- | | |
| TRINITY_DN2799_c0_g1_i1_len_334_75697.p1_type_internal_len_112 | ---NA--- | | |
| TRINITY_DN2624_c0_g1_i1_len_336_249644.p1_type_internal_len_112 | ---NA--- | | |
| TRINITY_DN32403_c0_g1_i1_len_334_138607.p1_type_internal_len_112 | ---NA--- | | |
| TRINITY_DN13137_c0_g1_i1_len_337_138455.p1_type_internal_len_112 | ---NA--- | | |
| TRINITY_DN11012_c0_g1_i1_len_336_138519.p1_type_internal_len_112 | XP_022074288.1peroxisomal sarcosine oxidase isoform X1 | | |
| TRINITY_DN9512_c0_g1_i1_len_518_203684.p1_type_5prime_partial_len_111 | ---NA--- | | |

| | | | |
|---|---|---|---|
| TRINITY_DN30883_c0_g1_i1_len_333_138674.p1_type_internal_len_112 | XP_013856237.1PREDICTED: uncharacterized protein LOC106512091, partial | F:GO:0003676; P:GO:0015074 | |
| TRINITY_DN7584_c0_g1_i1_len_452_205884.p1_type_3prime_partial_len_112 | ---NA--- | | |
| TRINITY_DN39152_c0_g1_i1_len_337_211547.p1_type_internal_len_112 | ---NA--- | | |
| TRINITY_DN5731_c0_g1_i1_len_966_196842.p3_type_5prime_partial_len_111 | ---NA--- | | |
| TRINITY_DN6392_c0_g1_i1_len_335_211704.p1_type_internal_len_112 | XP_018014829.1 phosphoglucomutase-2-like | | |
| TRINITY_DN8321_c0_g1_i1_len_687_163718.p1_type_complete_len_111 | ---NA--- | | |
| TRINITY_DN20845_c0_g1_i1_len_1566_157520.p2_type_5prime_partial_len_111 | ---NA--- | | |
| TRINITY_DN29924_c0_g2_i1_len_342_211262.p1_type_5prime_partial_len_111 | ---NA--- | | |
| TRINITY_DN31839_c0_g1_i1_len_337_211566.p1_type_internal_len_112 | XP_021962130.1ATP-binding cassette sub-family A member 3-like | F:GO:0005524; C:GO:0016021; F:GO:0042626; P:GO:0055085 | EC:3.6.1.3; EC:3.6.1.15 |
| TRINITY_DN4796_c0_g1_i1_len_335_249692.p1_type_internal_len_112 | KXJ26768.1hypothetical protein AC249_AIPGENE26403 | | |
| TRINITY_DN19737_c0_g1_i1_len_1106_62462.p2_type_complete_len_111 | ---NA--- | | |
| TRINITY_DN5598_c0_g1_i1_len_336_138514.p1_type_internal_len_112 | XP_023716937.1myotubularin-related protein 8 isoform X1 | | |
| TRINITY_DN19312_c0_g1_i1_len_1023_160475.p1_type_complete_len_111 | ---NA--- | | |
| 2681\|_\|c17739_f4p0_2525_isoform_c17739_full_length_coverage_4_non_full_length_coverage_0_isoform_length_2525_22481 | XP_018018008.1 ubiquitin carboxyl-terminal hydrolase 5-like isoform X1 | P:GO:0006508; F:GO:0008234 | |
| 2683\|_\|c2337_f4p1_2524_isoform_c2337_full_length_coverage_4_non_full_length_coverage_1_isoform_length_2524_16336 | XP_002935667.1 tumor suppressor candidate 2 | | |

<table>
<tr><td>2684|_|c12682_f2p0_2524_isoform_c12682_full_length_coverage_2_non_full_length_coverage_0_isoform_length_2524_22153</td><td>KFM82470.1Rho guanine nucleotide exchange factor 26</td><td>F:GO:0005089; P:GO:0035023</td><td></td></tr>
<tr><td>2687|_|c10946_f3p1_2523_isoform_c10946_full_length_coverage_3_non_full_length_coverage_1_isoform_length_2523_12928</td><td>---NA---</td><td></td><td></td></tr>
<tr><td>2689|_|c9911_f2p5_2523_isoform_c9911_full_length_coverage_2_non_full_length_coverage_5_isoform_length_2523_12844</td><td>XP_018017521.1 nicotinamide/nicotinic acid mononucleotide adenylyltransferase 1-like</td><td>F:GO:0000166; P:GO:0019363; F:GO:0070566</td><td></td></tr>
<tr><td>2690|_|c11750_f2p1_2522_isoform_c11750_full_length_coverage_2_non_full_length_coverage_1_isoform_length_2522_28581</td><td>---NA---</td><td></td><td></td></tr>
<tr><td>2691|_|c11848_f4p8_2522_isoform_c11848_full_length_coverage_4_non_full_length_coverage_8_isoform_length_2522_13016</td><td>---NA---</td><td></td><td></td></tr>
<tr><td>2692|_|c25575_f2p2_2521_isoform_c25575_full_length_coverage_2_non_full_length_coverage_2_isoform_length_2521_5632</td><td>---NA---</td><td></td><td></td></tr>
<tr><td>2693|_|c18180_f2p2_2521_isoform_c18180_full_length_coverage_2_non_full_length_coverage_2_isoform_length_2521_13633</td><td>XP_018023627.1 glutathione S-transferase C-terminal domain-containing protein homolog</td><td></td><td></td></tr>
<tr><td>2694|_|c18055_f2p0_2521_isoform_c18055_full_length_coverage_2_non_full_length_coverage_0_isoform_length_2521_13540</td><td>KOC68905.1Integrator complex subunit 1</td><td>C:GO:0032039; P:GO:0034474</td><td></td></tr>
<tr><td>TRINITY_DN25634_c0_g1_i1_len_335_44305.p1_type_internal_len_112</td><td>---NA---</td><td></td><td></td></tr>
<tr><td>TRINITY_DN7967_c0_g1_i1_len_335_44301.p1_type_internal_len_112</td><td>---NA---</td><td></td><td></td></tr>
<tr><td>TRINITY_DN2732_c0_g1_i1_len_736_238693.p1_type_3prime_partial_len_112</td><td>---NA---</td><td></td><td></td></tr>
<tr><td>TRINITY_DN16937_c0_g1_i1_len_2002_772.p3_type_5prime_partial_len_111</td><td>---NA---</td><td></td><td></td></tr>
</table>

| | | | |
|---|---|---|---|
| TRINITY_DN2732_c0_g1_i1_len_736_238693.p2_type_complete_len_111 | ---NA--- | | |
| TRINITY_DN12778_c0_g1_i2_len_336_211609.p1_type_internal_len_112 | ---NA--- | | |
| TRINITY_DN20713_c0_g1_i1_len_1802_59780.p2_type_complete_len_111 | ---NA--- | | |
| TRINITY_DN11069_c0_g1_i1_len_1170_195168.p2_type_complete_len_111 | ---NA--- | | |
| TRINITY_DN28055_c0_g1_i1_len_783_198827.p1_type_complete_len_111 | ---NA--- | | |
| TRINITY_DN11301_c0_g1_i1_len_390_72800.p1_type_5prime_partial_len_111 | ---NA--- | | |
| TRINITY_DN23728_c0_g1_i1_len_335_44312.p1_type_internal_len_112 | ---NA--- | | |
| TRINITY_DN20868_c0_g1_i1_len_541_203120.p1_type_5prime_partial_len_111 | ---NA--- | | |
| TRINITY_DN13317_c0_g1_i1_len_465_70119.p2_type_5prime_partial_len_111 | ---NA--- | | |
| TRINITY_DN7752_c0_g1_i1_len_729_238770.p1_type_complete_len_111 | ---NA--- | | |
| TRINITY_DN31554_c0_g1_i1_len_400_246389.p1_type_5prime_partial_len_111 | ---NA--- | | |
| TRINITY_DN17101_c0_g1_i2_len_459_168187.p1_type_complete_len_111 | ---NA--- | | |
| TRINITY_DN24692_c0_g1_i1_len_1078_195865.p1_type_3prime_partial_len_112 | ---NA--- | | |
| TRINITY_DN19320_c0_g1_i1_len_333_249825.p1_type_internal_len_112 | ---NA--- | | |
| TRINITY_DN18608_c0_g1_i2_len_744_35257.p1_type_3prime_partial_len_112 | ---NA--- | | |
| TRINITY_DN32731_c0_g1_i1_len_333_249836.p1_type_internal_len_112 | ---NA--- | | |

| | | | |
|---|---|---|---|
| TRINITY_DN9158_c0_g1_i1_len_381_136191.p1_type_5prime_partial_len_111 | ---NA--- | | |
| TRINITY_DN12846_c0_g1_i2_len_799_300137.p2_type_3prime_partial_len_112 | ---NA--- | | |
| TRINITY_DN35577_c0_g1_i1_len_333_249801.p1_type_internal_len_112 | XP_018015944.1PREDICTED: uncharacterized protein LOC108672734 isoform X1 | | |
| TRINITY_DN26649_c0_g1_i1_len_333_249803.p1_type_internal_len_112 | ---NA--- | | |
| TRINITY_DN2366_c0_g1_i1_len_557_241630.p1_type_5prime_partial_len_111 | ---NA--- | | |
| TRINITY_DN11777_c0_g1_i4_len_335_44332.p1_type_internal_len_112 | ---NA--- | | |
| TRINITY_DN28951_c0_g1_i1_len_335_44331.p1_type_internal_len_112 | ---NA--- | | |
| TRINITY_DN6542_c0_g1_i1_len_336_211607.p1_type_internal_len_112 | AAI58467.1LOC100145057 protein | F:GO:0000166; F:GO:0004812; F:GO:0004827; F:GO:0005524; C:GO:0005737; P:GO:0006418; P:GO:0006433 | |
| TRINITY_DN20117_c0_g1_i2_len_1009_270148.p2_type_complete_len_111 | ---NA--- | | |
| TRINITY_DN16157_c0_g1_i1_len_1118_269524.p3_type_complete_len_111 | ---NA--- | | |
| TRINITY_DN2867_c0_g1_i1_len_336_211629.p1_type_internal_len_112 | ---NA--- | | |
| TRINITY_DN37495_c0_g1_i1_len_333_211805.p2_type_internal_len_112 | XP_018026744.1 myosin-IIIb-like | F:GO:0003774; F:GO:0003779; F:GO:0004672; F:GO:0005524; P:GO:0006468; C:GO:0016459 | EC:3.6.1.15 |
| TRINITY_DN16289_c0_g1_i1_len_2058_266165.p2_type_5prime_partial_len_111 | ---NA--- | | |
| TRINITY_DN16072_c0_g1_i1_len_873_161737.p1_type_complete_len_111 | ---NA--- | | |

| | | | |
|---|---|---|---|
| TRINITY_DN27657_c0_g1_i1_len_984_196700.p2_type_complete_len_111 | ---NA--- | | |
| TRINITY_DN15596_c0_g1_i1_len_1785_123523.p1_type_5prime_partial_len_111 | ---NA--- | | |
| TRINITY_DN29765_c0_g1_i1_len_514_132046.p1_type_5prime_partial_len_111 | ---NA--- | | |
| TRINITY_DN9226_c0_g1_i1_len_336_211628.p1_type_internal_len_112 | AWD73767.1chitinase 5 | F:GO:0004553; C:GO:0005576; P:GO:0005975; P:GO:0006030; F:GO:0008061 | |
| TRINITY_DN16741_c0_g1_i1_len_336_211648.p1_type_internal_len_112 | XP_018009335.1 hemicentin-1-like | | |
| TRINITY_DN5619_c0_g1_i1_len_336_211645.p1_type_internal_len_112 | ---NA--- | | |
| TRINITY_DN9956_c0_g1_i1_len_333_211803.p1_type_internal_len_112 | ---NA--- | | |
| TRINITY_DN22617_c0_g1_i1_len_567_67677.p2_type_5prime_partial_len_111 | XP_014771883.1PREDICTED: uncharacterized protein LOC106870361 | | |
| TRINITY_DN18416_c0_g1_i8_len_2055_266171.p2_type_complete_len_111 | ---NA--- | | |
| TRINITY_DN8978_c0_g1_i1_len_471_167849.p1_type_5prime_partial_len_111 | ---NA--- | | |
| TRINITY_DN20950_c0_g1_i1_len_1003_236094.p1_type_complete_len_111 | XP_018015253.1 protein aveugle-like | | |
| TRINITY_DN15762_c0_g1_i2_len_336_211615.p1_type_5prime_partial_len_111 | ---NA--- | | |
| TRINITY_DN29677_c0_g1_i1_len_425_306138.p1_type_5prime_partial_len_111 | ---NA--- | | |
| TRINITY_DN8616_c0_g1_i1_len_336_211622.p1_type_internal_len_112 | KDR18748.1Microtubule-associated serine/threonine-protein kinase-like | F:GO:0004674; F:GO:0005524; P:GO:0006468 | EC:2.7.11 |
| TRINITY_DN15105_c0_g1_i1_len_525_68520.p1_type_5prime_partial_len_111 | ---NA--- | | |
| TRINITY_DN10025_c0_g1_i1_len_388_72882.p2_type_5prime_partial_len_111 | ---NA--- | | |

| | | | |
|---|---|---|---|
| TRINITY_DN13627_c0_g1_i2_len_525_203517.p1_type_5prime_partial_len_111 | ---NA--- | | |
| TRINITY_DN19272_c2_g1_i2_len_2030_266232.p1_type_complete_len_111 | ---NA--- | | |
| TRINITY_DN26779_c0_g1_i1_len_397_246511.p1_type_3prime_partial_len_112 | ---NA--- | | |
| TRINITY_DN36684_c0_g1_i1_len_336_211625.p1_type_internal_len_112 | ---NA--- | | |
| TRINITY_DN34448_c0_g1_i1_len_386_72964.p1_type_5prime_partial_len_111 | ---NA--- | | |
| TRINITY_DN40581_c0_g1_i1_len_333_211808.p1_type_internal_len_112 | ---NA--- | | |
| TRINITY_DN32676_c0_g1_i1_len_334_211793.p1_type_internal_len_112 | ---NA--- | | |
| TRINITY_DN18108_c0_g1_i1_len_1189_61995.p2_type_complete_len_111 | ---NA--- | | |
| TRINITY_DN35090_c0_g1_i1_len_334_249730.p1_type_internal_len_112 | ---NA--- | | |
| TRINITY_DN36518_c0_g1_i1_len_334_249733.p1_type_internal_len_112 | XP_018012650.1 fibrillin-2-like | F:GO:0005044; F:GO:0005201; F:GO:0005509; P:GO:0006898; C:GO:0016020; C:GO:0062023 | |
| TRINITY_DN21638_c0_g1_i1_len_799_198617.p1_type_complete_len_111 | ---NA--- | | |
| TRINITY_DN12141_c0_g1_i1_len_335_211684.p2_type_internal_len_112 | ---NA--- | | |
| TRINITY_DN21260_c0_g1_i1_len_1836_156567.p1_type_5prime_partial_len_111 | ---NA--- | | |
| TRINITY_DN15027_c0_g1_i2_len_647_164260.p1_type_3prime_partial_len_112 | ---NA--- | | |
| TRINITY_DN41435_c0_g1_i1_len_337_211582.p1_type_internal_len_112 | ---NA--- | | |
| TRINITY_DN21048_c0_g1_i2_len_3542_153884.p3_type_complete_len_111 | ---NA--- | | |

| | | | |
|---|---|---|---|
| TRINITY_DN5892_c0_g1_i1_len_335_211681.p1_type_internal_len_112 | XP_018022039.1 midasin-like | | |
| TRINITY_DN33352_c0_g1_i1_len_334_249736.p3_type_internal_len_112 | ACP19563.1masquerade-like serine proteinase-like protein 3 | F:GO:0004252; P:GO:0006508 | EC:3.4.21 |
| TRINITY_DN31709_c0_g1_i1_len_335_211679.p1_type_internal_len_112 | ---NA--- | | |
| TRINITY_DN15769_c0_g1_i1_len_363_42962.p1_type_3prime_partial_len_112 | ---NA--- | | |
| TRINITY_DN37976_c0_g1_i1_len_435_245039.p1_type_5prime_partial_len_111 | ---NA--- | | |
| TRINITY_DN20367_c2_g1_i7_len_1679_267149.p2_type_complete_len_111 | ---NA--- | | |
| TRINITY_DN32609_c0_g1_i1_len_334_211741.p1_type_internal_len_112 | AHA44500.1cyclooxygenase | F:GO:0004601; P:GO:0006979; C:GO:0016021; F:GO:0020037; P:GO:0055114; P:GO:0098869 | EC:1.11.1.7 |
| TRINITY_DN18697_c0_g1_i1_len_334_211742.p1_type_internal_len_112 | ---NA--- | | |
| TRINITY_DN19542_c0_g1_i1_len_738_65220.p2_type_3prime_partial_len_112 | ---NA--- | | |
| TRINITY_DN31399_c0_g1_i1_len_334_211736.p1_type_5prime_partial_len_111 | ---NA--- | | |
| TRINITY_DN27428_c0_g1_i1_len_1098_195713.p1_type_5prime_partial_len_111 | ---NA--- | | |
| TRINITY_DN27161_c0_g2_i1_len_798_198625.p1_type_5prime_partial_len_111 | ---NA--- | | |
| TRINITY_DN17863_c0_g1_i2_len_854_237384.p1_type_5prime_partial_len_111 | ---NA--- | | |
| TRINITY_DN14794_c0_g1_i1_len_335_211686.p1_type_internal_len_112 | ---NA--- | | |
| TRINITY_DN15487_c0_g1_i1_len_464_244087.p1_type_3prime_partial_len_112 | ---NA--- | | |
| TRINITY_DN17173_c0_g1_i1_len_723_65402.p2_type_5prime_partial_len_111 | ---NA--- | | |

| | | | |
|---|---|---|---|
| TRINITY_DN30168_c0_g1_i1_len_453_244453.p1_type_5prime_partial_len_111 | ---NA--- | | |
| TRINITY_DN13900_c0_g1_i1_len_379_171084.p1_type_5prime_partial_len_111 | ---NA--- | | |
| TRINITY_DN16582_c0_g1_i1_len_350_248796.p1_type_3prime_partial_len_112 | BAJ88166.1predicted protein | F:GO:0004497; C:GO:0009507; P:GO:0009853; P:GO:0015977; P:GO:0015979; F:GO:0016984; P:GO:0055114 | EC:4.1.1.39 |
| TRINITY_DN5129_c0_g1_i1_len_338_138362.p1_type_5prime_partial_len_111 | ---NA--- | | |
| TRINITY_DN24876_c0_g1_i1_len_506_204044.p1_type_complete_len_111 | ---NA--- | | |
| TRINITY_DN39715_c0_g1_i1_len_334_211776.p1_type_internal_len_112 | XP_018024410.1 putative serine/threonine-protein kinase C05D10.2 | P:GO:0000165; F:GO:0004707; F:GO:0005524 | EC:2.7.11; EC:2.7.11.24 |
| TRINITY_DN16137_c0_g1_i2_len_1036_33305.p3_type_3prime_partial_len_112 | ---NA--- | | |
| TRINITY_DN39479_c0_g1_i1_len_337_211597.p1_type_internal_len_112 | ---NA--- | | |
| TRINITY_DN47_c0_g1_i1_len_334_249767.p1_type_internal_len_112 | ---NA--- | | |
| TRINITY_DN9444_c0_g1_i3_len_334_249753.p1_type_internal_len_112 | ---NA--- | | |
| TRINITY_DN35483_c0_g1_i1_len_334_211765.p2_type_internal_len_112 | XP_018018244.1 enhancer of split m7 protein-like | F:GO:0003677; C:GO:0005634; P:GO:0006355; F:GO:0046983 | |
| TRINITY_DN31580_c0_g1_i1_len_334_249756.p1_type_internal_len_112 | XP_018010174.1 proton-coupled folate transporter-like | | |
| TRINITY_DN2333_c0_g1_i1_len_334_249762.p1_type_internal_len_112 | ---NA--- | | |
| TRINITY_DN183_c0_g1_i1_len_334_211770.p1_type_internal_len_112 | ---NA--- | | |
| TRINITY_DN6356_c0_g1_i1_len_333_249783.p1_type_internal_len_112 | ---NA--- | | |

<table>
<tr><th></th><th></th><th></th><th></th></tr>
<tr><td>TRINITY_DN12780_c0_g1_i1_len_1182_195087.p2_type_complete_len_111</td><td>AEF33797.1Rab7</td><td>F:GO:0003924; F:GO:0005525; C:GO:0016021</td><td>EC:3.6.1.15</td></tr>
<tr><td>TRINITY_DN3523_c0_g1_i1_len_334_249772.p1_type_internal_len_112</td><td>---NA---</td><td></td><td></td></tr>
<tr><td>TRINITY_DN38194_c0_g1_i1_len_333_249781.p1_type_internal_len_112</td><td>---NA---</td><td></td><td></td></tr>
<tr><td>2695|_|c2527_f3p2_2521_isoform_c2527_full_length_coverage_3_non_full_length_coverage_2_isoform_length_2521_21015</td><td>XP_018018363.1 protein Red-like</td><td>C:GO:0005634; P:GO:0007094</td><td></td></tr>
<tr><td>2696|_|c2564_f2p1_2520_isoform_c2564_full_length_coverage_2_non_full_length_coverage_1_isoform_length_2520_11974</td><td>---NA---</td><td></td><td></td></tr>
<tr><td>TRINITY_DN29097_c0_g1_i1_len_3037_188478.p1_type_complete_len_840</td><td>AJO70186.1V-H-ATPase 116 kDa subunit A</td><td>C:GO:0000220; F:GO:0015078; P:GO:0015991; C:GO:0016021</td><td></td></tr>
<tr><td>2698|_|c10192_f3p4_2520_isoform_c10192_full_length_coverage_3_non_full_length_coverage_4_isoform_length_2520_17335</td><td>XP_012256477.1nucleolar protein 10</td><td></td><td></td></tr>
<tr><td>TRINITY_DN23212_c0_g1_i1_len_2675_229478.p1_type_5prime_partial_len_840</td><td>XP_014256161.1histone deacetylase 6 isoform X1</td><td>F:GO:0008270; F:GO:0016787</td><td></td></tr>
<tr><td>2700|_|c23922_f2p0_2519_isoform_c23922_full_length_coverage_2_non_full_length_coverage_0_isoform_length_2519_22924</td><td>---NA---</td><td></td><td></td></tr>
<tr><td>2701|_|c18293_f2p1_2519_isoform_c18293_full_length_coverage_2_non_full_length_coverage_1_isoform_length_2519_13701</td><td>---NA---</td><td></td><td></td></tr>
<tr><td>2703|_|c1404_f12p3_2518_isoform_c1404_full_length_coverage_12_non_full_length_coverage_3_isoform_length_2518_11377</td><td>XP_024086407.1bromodomain-containing protein 7 isoform X3</td><td></td><td></td></tr>
<tr><td>TRINITY_DN22812_c0_g1_i1_len_3302_58002.p1_type_complete_len_839</td><td>XP_021356472.1protein PAT1 homolog 1-like isoform X3</td><td>P:GO:0000290</td><td></td></tr>
<tr><td>TRINITY_DN20380_c0_g1_i1_len_2520_29756.p1_type_internal_len_840</td><td>KDR16553.1Myeloid zinc finger 1</td><td>F:GO:0003676</td><td></td></tr>
</table>

| | | | |
|---|---|---|---|
| TRINITY_DN9309_c0_g1_i2_len_336_211667.p1_type_internal_len_112 | XP_014278218.1type I inositol 3,4-bisphosphate 4-phosphatase isoform X3 | F:GO:0016316; P:GO:0036092 | |
| TRINITY_DN516_c0_g1_i1_len_333_211828.p1_type_internal_len_112 | EFX69628.1ABC protein, subfamily ABCG | F:GO:0000166; C:GO:0005886; F:GO:0042626; P:GO:0055085 | EC:3.6.1.3; EC:3.6.1.15 |
| TRINITY_DN30300_c0_g1_i1_len_333_249782.p1_type_internal_len_112 | OPL21697.1tubulin chain alpha-1c | | |
| TRINITY_DN20133_c0_g1_i1_len_1999_156111.p2_type_complete_len_111 | ---NA--- | | |
| TRINITY_DN20409_c0_g1_i1_len_1384_268200.p2_type_5prime_partial_len_111 | ---NA--- | | |
| TRINITY_DN30648_c0_g1_i1_len_801_162413.p2_type_complete_len_111 | ---NA--- | | |
| TRINITY_DN2198_c0_g1_i1_len_335_211675.p1_type_internal_len_112 | ---NA--- | | |
| TRINITY_DN23335_c0_g1_i6_len_1976_156173.p1_type_complete_len_111 | ---NA--- | | |
| TRINITY_DN15014_c0_g1_i1_len_333_211846.p1_type_internal_len_112 | ---NA--- | | |
| TRINITY_DN12933_c0_g1_i1_len_395_170410.p1_type_5prime_partial_len_111 | ---NA--- | | |
| TRINITY_DN28746_c0_g1_i1_len_1218_194815.p1_type_5prime_partial_len_111 | ---NA--- | | |
| TRINITY_DN39480_c0_g1_i1_len_334_211764.p1_type_internal_len_112 | XP_013389487.1dnaJ homolog subfamily C member 13 | | |
| TRINITY_DN8607_c0_g1_i1_len_333_211844.p1_type_internal_len_112 | ---NA--- | | |
| TRINITY_DN31406_c0_g1_i1_len_334_249748.p1_type_internal_len_112 | ---NA--- | | |
| TRINITY_DN41620_c0_g1_i1_len_350_210752.p1_type_5prime_partial_len_111 | ---NA--- | | |
| TRINITY_DN27255_c0_g1_i2_len_336_107405.p1_type_internal_len_112 | ---NA--- | | |

| | | | |
|---|---|---|---|
| TRINITY_DN16794_c0_g1_i1_len_336_107408.p1_type_internal_len_112 | ---NA--- | | |
| TRINITY_DN265_c0_g1_i1_len_336_107414.p1_type_internal_len_112 | ---NA--- | | |
| TRINITY_DN22597_c0_g2_i1_len_335_14708.p1_type_internal_len_112 | ---NA--- | | |
| TRINITY_DN33582_c0_g1_i1_len_335_173293.p1_type_internal_len_112 | ---NA--- | | |
| TRINITY_DN9003_c0_g1_i1_len_335_281906.p2_type_internal_len_112 | ---NA--- | | |
| TRINITY_DN23616_c0_g1_i1_len_1444_193401.p3_type_5prime_partial_len_111 | ---NA--- | | |
| TRINITY_DN26714_c0_g1_i1_len_334_173313.p1_type_internal_len_112 | ---NA--- | | |
| TRINITY_DN33976_c0_g1_i1_len_335_173300.p2_type_internal_len_112 | ---NA--- | | |
| TRINITY_DN30457_c0_g1_i1_len_334_281912.p1_type_internal_len_112 | XP_018022944.1 carboxypeptidase D-like | | |
| TRINITY_DN8079_c0_g1_i1_len_335_173298.p1_type_internal_len_112 | ---NA--- | | |
| TRINITY_DN22906_c0_g1_i1_len_358_308802.p1_type_5prime_partial_len_111 | ---NA--- | | |
| TRINITY_DN23858_c0_g1_i1_len_2012_231082.p2_type_complete_len_111 | ---NA--- | | |
| TRINITY_DN25095_c0_g1_i1_len_335_14682.p1_type_internal_len_112 | ---NA--- | | |
| TRINITY_DN24464_c0_g1_i1_len_333_281995.p1_type_internal_len_112 | AAS98884.1slow-tonic S2 myosin heavy chain | F:GO:0003774; C:GO:0016459 | EC:3.6.1.15 |
| TRINITY_DN24161_c0_g1_i1_len_333_173379.p1_type_internal_len_112 | ASQ43185.1Src | F:GO:0004715; F:GO:0005524; P:GO:0018108 | EC:2.7.10; EC:2.7.10.2 |
| TRINITY_DN31689_c0_g1_i1_len_337_107353.p1_type_internal_len_112 | ---NA--- | | |

| | | | |
|---|---|---|---|
| TRINITY_DN25936_c0_g1_i1_len_333_173375.p1_type_internal_len_112 | ---NA--- | | |
| TRINITY_DN354_c0_g1_i1_len_333_173371.p1_type_internal_len_112 | ---NA--- | | |
| TRINITY_DN11638_c0_g1_i1_len_334_281957.p1_type_internal_len_112 | BAA82101.1anti-Entamoeba histolytica immunoglobulin gamma heavy chain | | |
| TRINITY_DN24965_c0_g1_i1_len_333_173404.p1_type_internal_len_112 | ---NA--- | | |
| TRINITY_DN30575_c0_g1_i1_len_333_173401.p1_type_internal_len_112 | XP_018014826.1 myotubularin-related protein 10-A-like | | |
| TRINITY_DN861_c0_g1_i2_len_337_107335.p1_type_internal_len_112 | ---NA--- | | |
| TRINITY_DN17585_c0_g1_i1_len_1657_192270.p4_type_5prime_partial_len_111 | ---NA--- | | |
| TRINITY_DN9246_c0_g1_i1_len_336_173208.p1_type_internal_len_112 | ---NA--- | | |
| TRINITY_DN4216_c0_g1_i1_len_334_107511.p1_type_internal_len_112 | ---NA--- | | |
| TRINITY_DN7880_c0_g1_i1_len_336_173222.p1_type_internal_len_112 | ---NA--- | | |
| TRINITY_DN17387_c0_g1_i1_len_441_102964.p1_type_5prime_partial_len_111 | ---NA--- | | |
| TRINITY_DN25584_c0_g1_i1_len_334_14753.p1_type_internal_len_112 | ---NA--- | | |
| TRINITY_DN1208_c0_g1_i1_len_334_14767.p1_type_internal_len_112 | ---NA--- | | |
| TRINITY_DN16064_c1_g1_i1_len_337_173153.p1_type_internal_len_112 | ---NA--- | | |
| TRINITY_DN28621_c0_g1_i1_len_337_173182.p1_type_internal_len_112 | ---NA--- | | |
| TRINITY_DN4187_c0_g1_i1_len_335_173262.p1_type_internal_len_112 | ---NA--- | | |

| | | | |
|---|---|---|---|
| TRINITY_DN15269_c0_g1_i3_len_335_173261.p3_type_internal_len_112 | ---NA--- | | |
| TRINITY_DN27142_c0_g1_i1_len_335_173258.p2_type_internal_len_112 | ---NA--- | | |
| TRINITY_DN33210_c0_g1_i1_len_335_173257.p3_type_internal_len_112 | ---NA--- | | |
| TRINITY_DN32814_c0_g1_i1_len_335_107494.p1_type_internal_len_112 | XP_018008550.1 protein kinase C-binding protein NELL1-like | | |
| TRINITY_DN10979_c0_g1_i1_len_335_173278.p3_type_internal_len_112 | ---NA--- | | |
| TRINITY_DN24104_c0_g1_i1_len_335_173275.p1_type_internal_len_112 | XP_015605113.1non-lysosomal glucosylceramidase | | |
| TRINITY_DN7276_c0_g1_i1_len_335_173268.p1_type_internal_len_112 | ---NA--- | | |
| TRINITY_DN2725_c0_g1_i1_len_690_98198.p2_type_5prime_partial_len_111 | XP_018015832.1 ubiquitin carboxyl-terminal hydrolase CYLD-like isoform X2 | | |
| TRINITY_DN23216_c0_g1_i1_len_334_14747.p1_type_internal_len_112 | ---NA--- | | |
| TRINITY_DN18231_c0_g1_i3_len_334_14742.p1_type_internal_len_112 | ---NA--- | | |
| TRINITY_DN1859_c0_g1_i1_len_723_272564.p1_type_3prime_partial_len_112 | ---NA--- | | |
| TRINITY_DN32092_c0_g1_i1_len_336_173245.p1_type_internal_len_112 | ---NA--- | | |
| TRINITY_DN31126_c0_g1_i1_len_610_99273.p1_type_complete_len_111 | ---NA--- | | |
| TRINITY_DN15717_c0_g1_i2_len_427_40598.p1_type_5prime_partial_len_111 | ---NA--- | | |
| TRINITY_DN15758_c0_g1_i1_len_2203_230500.p2_type_3prime_partial_len_112 | XP_018006338.1 general transcription factor IIH subunit 1-like | | |
| TRINITY_DN25079_c0_g1_i1_len_6674_227532.p2_type_complete_len_111 | ---NA--- | | |

| | | | |
|---|---|---|---|
| TRINITY_DN25598_c0_g1_i1_len_336_14598.p1_type_internal_len_112 | ---NA--- | | |
| TRINITY_DN7039_c0_g1_i1_len_395_41670.p1_type_complete_len_111 | ---NA--- | | |
| TRINITY_DN26368_c0_g1_i1_len_2065_190673.p3_type_5prime_partial_len_111 | ---NA--- | | |
| TRINITY_DN11357_c0_g1_i1_len_337_14583.p1_type_5prime_partial_len_111 | AHE40497.1p38 mitogen-activated protein kinase | P:GO:0000165; F:GO:0004707; F:GO:0005524 | EC:2.7.11; EC:2.7.11.24 |
| TRINITY_DN3925_c0_g1_i1_len_476_9425.p1_type_5prime_partial_len_111 | ---NA--- | | |
| TRINITY_DN24556_c0_g1_i1_len_1698_232141.p2_type_5prime_partial_len_111 | ---NA--- | | |
| TRINITY_DN23665_c0_g1_i1_len_1352_233761.p2_type_complete_len_111 | ---NA--- | | |
| TRINITY_DN27342_c0_g1_i1_len_595_37022.p3_type_5prime_partial_len_111 | ---NA--- | | |
| TRINITY_DN20949_c0_g1_i1_len_421_207120.p1_type_5prime_partial_len_111 | ---NA--- | | |
| TRINITY_DN5125_c0_g1_i1_len_335_309965.p1_type_internal_len_112 | ---NA--- | | |
| TRINITY_DN38787_c0_g1_i1_len_406_207747.p1_type_complete_len_111 | ---NA--- | | |
| TRINITY_DN23934_c0_g1_i1_len_2019_190839.p1_type_5prime_partial_len_111 | XP_018024537.1 zinc finger protein 706-like | | |
| TRINITY_DN25181_c0_g1_i1_len_347_106765.p1_type_5prime_partial_len_111 | ---NA--- | | |
| TRINITY_DN9468_c0_g1_i2_len_573_99882.p2_type_5prime_partial_len_111 | ---NA--- | | |
| TRINITY_DN16865_c0_g1_i1_len_429_103336.p1_type_5prime_partial_len_111 | ---NA--- | | |
| TRINITY_DN25608_c0_g1_i1_len_335_309986.p1_type_internal_len_112 | XP_018012843.1 serine/threonine-protein phosphatase CPPED1-like | F:GO:0016787 | |

| | | | |
|---|---|---|---|
| TRINITY_DN11869_c0_g1_i1_len_425_10844.p1_type_5prime_partial_len_111 | XP_026756604.1luciferin 4-monooxygenase-like | | |
| TRINITY_DN16249_c0_g1_i1_len_414_103828.p1_type_5prime_partial_len_111 | ---NA--- | | |
| TRINITY_DN14232_c0_g1_i1_len_693_272899.p1_type_complete_len_111 | ---NA--- | | |
| TRINITY_DN37303_c0_g1_i1_len_397_208165.p1_type_3prime_partial_len_112 | AYA22652.1formin-like protein | F:GO:0003779; F:GO:0017048; P:GO:0030036 | |
| TRINITY_DN13117_c0_g1_i1_len_336_14620.p1_type_internal_len_112 | ---NA--- | | |
| TRINITY_DN21880_c0_g1_i1_len_1513_232971.p1_type_5prime_partial_len_111 | ---NA--- | | |
| TRINITY_DN8108_c0_g1_i1_len_388_208619.p1_type_5prime_partial_len_111 | XP_019644105.1 multidrug resistance-associated protein 5-like | | |
| TRINITY_DN21055_c0_g1_i2_len_1138_94810.p2_type_5prime_partial_len_111 | XP_018020759.1 putative N-acetylated-alpha-linked acidic dipeptidase | | |
| TRINITY_DN21614_c0_g1_i1_len_704_199920.p2_type_complete_len_111 | ---NA--- | | |
| TRINITY_DN25264_c0_g1_i2_len_708_199864.p1_type_3prime_partial_len_112 | ---NA--- | | |
| TRINITY_DN21217_c0_g1_i1_len_2685_91345.p3_type_complete_len_111 | ---NA--- | | |
| TRINITY_DN30483_c0_g1_i5_len_2435_189602.p4_type_5prime_partial_len_111 | ---NA--- | | |
| TRINITY_DN13534_c0_g1_i1_len_730_97732.p1_type_3prime_partial_len_112 | ---NA--- | | |
| TRINITY_DN21900_c0_g2_i1_len_344_172804.p1_type_5prime_partial_len_111 | ---NA--- | | |
| TRINITY_DN26172_c0_g1_i1_len_3497_228479.p2_type_5prime_partial_len_111 | ---NA--- | | |
| TRINITY_DN23222_c0_g1_i2_len_758_199140.p1_type_complete_len_111 | ---NA--- | | |

<table>
<tr><th></th><th></th><th></th><th></th></tr>
<tr><td>TRINITY_DN9623_c0_g1_i1_len_381_12450.p1_type_5prime_partial_len_111</td><td>---NA---</td><td></td><td></td></tr>
<tr><td>TRINITY_DN22613_c0_g1_i2_len_1615_232472.p4_type_5prime_partial_len_111</td><td>---NA---</td><td></td><td></td></tr>
<tr><td>TRINITY_DN21902_c0_g1_i1_len_387_41981.p1_type_5prime_partial_len_111</td><td>---NA---</td><td></td><td></td></tr>
<tr><td>TRINITY_DN29577_c0_g1_i3_len_2715_189005.p5_type_5prime_partial_len_111</td><td>---NA---</td><td></td><td></td></tr>
<tr><td>2709|_|c2409_f2p1_2516_isoform_c2409_full_length_coverage_2_non_full_length_coverage_1_isoform_length_2516_16388</td><td>---NA---</td><td></td><td></td></tr>
<tr><td>2712|_|c7422_f2p2_2515_isoform_c7422_full_length_coverage_2_non_full_length_coverage_2_isoform_length_2515_12627</td><td>---NA---</td><td></td><td></td></tr>
<tr><td>2715|_|c11630_f2p3_2514_isoform_c11630_full_length_coverage_2_non_full_length_coverage_3_isoform_length_2514_22069</td><td>---NA---</td><td></td><td></td></tr>
<tr><td>TRINITY_DN24686_c0_g1_i3_len_2601_229603.p1_type_5prime_partial_len_838</td><td>XP_021924978.1uncharacterized protein LOC110832365 isoform X1</td><td></td><td></td></tr>
<tr><td>TRINITY_DN28419_c0_g1_i1_len_2626_189183.p1_type_5prime_partial_len_838</td><td>---NA---</td><td></td><td></td></tr>
<tr><td>TRINITY_DN28327_c0_g1_i3_len_2799_188857.p1_type_5prime_partial_len_838</td><td>XP_018017863.1 transmembrane protein 245-like</td><td>C:GO:0016020; C:GO:0016021</td><td></td></tr>
<tr><td>TRINITY_DN30050_c0_g1_i4_len_3951_187614.p1_type_5prime_partial_len_838</td><td>XP_018007452.1 homeotic protein spalt-major-like</td><td>F:GO:0003676</td><td></td></tr>
<tr><td>TRINITY_DN26042_c0_g1_i1_len_3264_188174.p1_type_5prime_partial_len_838</td><td>XP_017770979.1 dipeptidyl peptidase 9</td><td>P:GO:0006508; F:GO:0008236</td><td></td></tr>
<tr><td>2722|_|c24656_f2p16_2514_isoform_c24656_full_length_coverage_2_non_full_length_coverage_16_isoform_length_2514_18330</td><td>PIK43963.1putative methyl-CpG-binding domain protein 4</td><td>F:GO:0003677; F:GO:0003824; C:GO:0005634; P:GO:0006281; F:GO:0008263</td><td></td></tr>
<tr><td>2724|_|c10161_f2p1_2512_isoform_c10161_full_length_coverage_2_non_full_length_coverage_1_isoform_length_2512_21924</td><td>---NA---</td><td></td><td></td></tr>
</table>

| | | | |
|---|---|---|---|
| TRINITY_DN29614_c0_g1_i1_len_2616_189196.p3_type_complete_len_111 | ---NA--- | | |
| TRINITY_DN29941_c0_g1_i1_len_6427_186871.p3_type_5prime_partial_len_111 | ---NA--- | | |
| TRINITY_DN22863_c0_g1_i1_len_1672_192200.p2_type_5prime_partial_len_111 | ---NA--- | | |
| TRINITY_DN3796_c0_g1_i1_len_468_102158.p1_type_5prime_partial_len_111 | ---NA--- | | |
| TRINITY_DN13359_c0_g1_i1_len_2372_295285.p2_type_5prime_partial_len_111 | ---NA--- | | |
| TRINITY_DN20613_c0_g1_i1_len_710_199838.p1_type_5prime_partial_len_111 | ---NA--- | | |
| TRINITY_DN26183_c0_g1_i1_len_376_12696.p1_type_5prime_partial_len_111 | XP_014781812.1 zinc finger protein 239-like | F:GO:0003676 | |
| TRINITY_DN20293_c0_g1_i10_len_334_14775.p2_type_internal_len_112 | ---NA--- | | |
| TRINITY_DN24612_c0_g1_i2_len_4104_187528.p4_type_complete_len_111 | ---NA--- | | |
| TRINITY_DN30946_c0_g1_i1_len_358_248396.p1_type_5prime_partial_len_110 | BBC28100.1aldehyde oxidase | F:GO:0005506; F:GO:0009055; P:GO:0022900; F:GO:0051537; F:GO:0071949 | |
| TRINITY_DN31551_c0_g1_i1_len_332_75803.p1_type_internal_len_111 | ---NA--- | | |
| TRINITY_DN34128_c0_g1_i1_len_345_137981.p1_type_5prime_partial_len_110 | AHY86474.1hemocyanin subunit L4 | F:GO:0016491; F:GO:0046872; P:GO:0055114 | |
| TRINITY_DN28219_c0_g1_i1_len_1548_192840.p2_type_complete_len_110 | ---NA--- | | |
| TRINITY_DN13475_c0_g1_i1_len_330_212056.p1_type_internal_len_111 | XP_018010562.1 vacuolar protein sorting-associated protein 13D-like | | |
| TRINITY_DN35426_c0_g1_i1_len_332_75811.p3_type_internal_len_111 | ---NA--- | | |
| TRINITY_DN24099_c0_g1_i1_len_2192_230531.p3_type_3prime_partial_len_111 | ---NA--- | | |

| | | | |
|---|---|---|---|
| TRINITY_DN27484_c0_g1_i1_len_332_75816.p1_type_internal_len_111 | ---NA--- | | |
| TRINITY_DN19637_c0_g1_i2_len_332_75820.p1_type_internal_len_111 | ---NA--- | | |
| TRINITY_DN8363_c0_g1_i1_len_345_137969.p1_type_5prime_partial_len_110 | XP_018026797.1 BET1-like protein | | |
| TRINITY_DN25638_c0_g1_i1_len_1588_192621.p3_type_complete_len_110 | ---NA--- | | |
| TRINITY_DN26470_c0_g1_i1_len_389_12144.p1_type_5prime_partial_len_110 | ---NA--- | | |
| TRINITY_DN21836_c0_g1_i1_len_333_75739.p1_type_internal_len_111 | ---NA--- | | |
| TRINITY_DN18937_c0_g2_i1_len_1052_33232.p2_type_complete_len_110 | ---NA--- | | |
| TRINITY_DN14448_c0_g1_i1_len_357_280725.p1_type_5prime_partial_len_110 | ---NA--- | | |
| TRINITY_DN27760_c0_g1_i1_len_887_4244.p1_type_complete_len_110 | ---NA--- | | |
| TRINITY_DN26185_c0_g1_i2_len_3031_188489.p3_type_complete_len_110 | XP_012134650.1 ADP-ribosylation factor-like protein 5B isoform X3 | | |
| TRINITY_DN26537_c0_g1_i1_len_330_249981.p1_type_internal_len_111 | XP_018017230.1 dmX-like protein 2 isoform X1 | | |
| TRINITY_DN19995_c0_g1_i1_len_2191_190278.p3_type_complete_len_110 | ---NA--- | | |
| TRINITY_DN18687_c0_g1_i2_len_333_75750.p1_type_internal_len_111 | ---NA--- | | |
| TRINITY_DN36145_c0_g1_i1_len_333_211802.p1_type_internal_len_111 | ---NA--- | | |
| TRINITY_DN27193_c0_g1_i1_len_333_75712.p1_type_internal_len_111 | PFX28980.1Retrovirus-related Pol polyprotein from transposon 17.6 | | |
| TRINITY_DN37955_c0_g1_i1_len_331_211955.p1_type_internal_len_111 | ---NA--- | | |

| | | | |
|---|---|---|---|
| TRINITY_DN16288_c0_g1_i1_len_2456_265520.p3_type_complete_len_110 | XP_026284003.1DNA repair and recombination protein RAD54-like | | |
| TRINITY_DN7603_c0_g1_i1_len_333_75724.p1_type_internal_len_111 | ---NA--- | | |
| TRINITY_DN24563_c1_g1_i1_len_3662_228360.p2_type_complete_len_110 | ---NA--- | | |
| TRINITY_DN27705_c0_g1_i1_len_332_75778.p3_type_internal_len_111 | EZA53314.1hypothetical protein X777_06393 | F:GO:0003676; C:GO:0005634; P:GO:0006974 | |
| TRINITY_DN23044_c0_g1_i1_len_1702_157025.p1_type_complete_len_110 | ---NA--- | | |
| TRINITY_DN7140_c0_g1_i1_len_489_204584.p1_type_5prime_partial_len_110 | ---NA--- | | |
| TRINITY_DN13164_c0_g1_i1_len_332_75792.p4_type_internal_len_111 | ---NA--- | | |
| TRINITY_DN22672_c0_g1_i2_len_1460_124424.p2_type_complete_len_110 | ---NA--- | | |
| TRINITY_DN23461_c0_g1_i2_len_2215_230464.p3_type_5prime_partial_len_110 | ---NA--- | | |
| TRINITY_DN18481_c0_g1_i3_len_1679_31024.p2_type_5prime_partial_len_110 | ---NA--- | | |
| TRINITY_DN6363_c0_g1_i1_len_332_75774.p1_type_internal_len_111 | ---NA--- | | |
| TRINITY_DN23373_c0_g1_i1_len_1043_126277.p1_type_complete_len_110 | ---NA--- | | |
| TRINITY_DN10399_c0_g1_i1_len_331_211935.p1_type_internal_len_111 | ---NA--- | | |
| TRINITY_DN9800_c0_g1_i1_len_363_248124.p1_type_5prime_partial_len_110 | ---NA--- | | |
| TRINITY_DN27938_c0_g1_i1_len_330_75924.p1_type_5prime_partial_len_110 | ---NA--- | | |
| TRINITY_DN6397_c0_g1_i1_len_331_211968.p1_type_internal_len_111 | ---NA--- | | |

| | | | |
|---|---|---|---|
| TRINITY_DN5426_c0_g1_i1_len_504_69009.p1_type_5prime_partial_len_110 | ---NA--- | | |
| TRINITY_DN17217_c0_g1_i2_len_606_36855.p2_type_5prime_partial_len_110 | XP_018018110.1 centrosomal protein of 19 kDa-like | | |
| TRINITY_DN630_c0_g1_i2_len_393_104664.p1_type_5prime_partial_len_110 | ---NA--- | | |
| TRINITY_DN12679_c0_g1_i1_len_518_203696.p1_type_5prime_partial_len_110 | ---NA--- | | |
| TRINITY_DN22579_c0_g1_i6_len_1226_2528.p2_type_complete_len_110 | ---NA--- | | |
| TRINITY_DN12933_c0_g1_i1_len_382_12429.p1_type_5prime_partial_len_110 | ---NA--- | | |
| TRINITY_DN15855_c0_g1_i1_len_872_237208.p1_type_5prime_partial_len_110 | ---NA--- | | |
| TRINITY_DN15073_c0_g1_i1_len_1139_32832.p1_type_complete_len_110 | ---NA--- | | |
| TRINITY_DN501_c0_g1_i1_len_485_132732.p1_type_5prime_partial_len_110 | ---NA--- | | |
| TRINITY_DN27839_c2_g1_i1_len_4847_187184.p3_type_complete_len_110 | ---NA--- | | |
| TRINITY_DN17792_c0_g1_i1_len_747_162961.p1_type_complete_len_110 | ---NA--- | | |
| TRINITY_DN22210_c0_g1_i1_len_1142_195369.p2_type_5prime_partial_len_110 | ---NA--- | | |
| TRINITY_DN19240_c0_g1_i1_len_331_211979.p1_type_internal_len_111 | ---NA--- | | |
| TRINITY_DN11329_c0_g1_i1_len_460_102358.p1_type_5prime_partial_len_110 | ---NA--- | | |
| TRINITY_DN3684_c0_g1_i1_len_331_211925.p1_type_internal_len_111 | ---NA--- | | |
| TRINITY_DN24098_c0_g1_i4_len_1055_126205.p4_type_complete_len_110 | XP_012534722.1 actin, alpha skeletal muscle | | |

| | | | |
|---|---|---|---|
| TRINITY_DN18401_c0_g1_i1_len_1418_193548.p1_type_5prime_partial_len_110 | ---NA--- | | |
| TRINITY_DN17244_c0_g1_i1_len_331_211924.p2_type_internal_len_111 | XP_018026833.1 peroxidase-like | F:GO:0004601; P:GO:0006979; F:GO:0020037; P:GO:0055114; P:GO:0098869 | EC:1.11.1.7 |
| TRINITY_DN20298_c0_g1_i2_len_472_133059.p1_type_5prime_partial_len_110 | ---NA--- | | |
| TRINITY_DN33560_c0_g1_i1_len_330_249991.p1_type_internal_len_111 | XP_018025310.1 sodium/myo-inositol cotransporter 2-like | | |
| TRINITY_DN17509_c0_g2_i2_len_1739_266965.p2_type_complete_len_110 | ---NA--- | | |
| TRINITY_DN4343_c0_g1_i1_len_401_207946.p1_type_5prime_partial_len_110 | ---NA--- | | |
| TRINITY_DN18126_c0_g1_i1_len_806_237886.p3_type_complete_len_110 | XP_022185832.160S ribosomal protein L15 | | |
| TRINITY_DN7512_c0_g1_i1_len_731_163138.p1_type_complete_len_110 | AWP38854.1RING box protein 2 | F:GO:0008270 | |
| TRINITY_DN26275_c2_g2_i3_len_361_248217.p1_type_5prime_partial_len_110 | ---NA--- | | |
| TRINITY_DN29027_c0_g1_i1_len_331_75861.p2_type_internal_len_111 | PNI68724.1RPL7A isoform 10 | C:GO:0005840 | |
| TRINITY_DN7432_c0_g1_i2_len_403_135301.p1_type_5prime_partial_len_110 | ---NA--- | | |
| TRINITY_DN18764_c0_g1_i1_len_2156_230631.p2_type_complete_len_110 | ---NA--- | | |
| TRINITY_DN17632_c0_g1_i1_len_2717_154671.p1_type_5prime_partial_len_110 | ---NA--- | | |
| TRINITY_DN36437_c0_g1_i1_len_331_211950.p1_type_internal_len_111 | XP_018904082.1 integrin alpha-PS2-like isoform X2 | | |
| TRINITY_DN10833_c0_g1_i1_len_364_136973.p1_type_5prime_partial_len_110 | EGD79843.16-pyruvoyl tetrahydrobiopterin synthase | F:GO:0003874; P:GO:0006729; F:GO:0046872 | |
| TRINITY_DN26219_c0_g1_i1_len_2865_188753.p1_type_5prime_partial_len_110 | XP_021931049.1uncharacterized protein LOC110835283 isoform X6 | | |

| | | | |
|---|---|---|---|
| TRINITY_DN25374_c1_g1_i2_len_644_200915.p1_type_5prime_partial_len_110 | ---NA--- | | |
| TRINITY_DN28105_c0_g1_i1_len_334_249735.p1_type_internal_len_111 | ETN66609.1dipeptidyl aminopeptidase-like protein 6 | F:GO:0004177; P:GO:0006508; F:GO:0008236; C:GO:0016020; C:GO:0016021 | |
| TRINITY_DN24271_c0_g1_i1_len_334_281956.p1_type_internal_len_111 | ---NA--- | | |
| TRINITY_DN32766_c0_g1_i1_len_334_249739.p1_type_internal_len_111 | ---NA--- | | |
| TRINITY_DN8069_c0_g1_i1_len_331_211929.p1_type_internal_len_111 | ---NA--- | | |
| TRINITY_DN26626_c0_g1_i2_len_399_208038.p1_type_complete_len_110 | ---NA--- | | |
| TRINITY_DN15229_c0_g1_i2_len_334_281941.p1_type_internal_len_111 | ---NA--- | | |
| TRINITY_DN27524_c0_g1_i1_len_334_249751.p1_type_internal_len_111 | XP_026674134.1phospholipid-transporting ATPase ID isoform X4 | | |
| TRINITY_DN5139_c0_g1_i1_len_334_249752.p1_type_internal_len_111 | XP_008193750.1 5'-3' exoribonuclease 1 isoform X3 | P:GO:0000956; F:GO:0003723; C:GO:0005737; F:GO:0008409; P:GO:0090305 | |
| TRINITY_DN26360_c0_g1_i1_len_332_310151.p1_type_internal_len_111 | AOR07239.1potassium voltage-gated channel protein EAG | F:GO:0005249; C:GO:0016021; P:GO:0034765; P:GO:0071805 | |
| TRINITY_DN29207_c0_g1_i1_len_334_249744.p1_type_internal_len_111 | ---NA--- | | |
| TRINITY_DN21997_c0_g1_i1_len_3465_228508.p2_type_complete_len_110 | ---NA--- | | |
| TRINITY_DN1931_c0_g1_i1_len_334_249745.p1_type_internal_len_111 | ---NA--- | | |
| TRINITY_DN10091_c0_g1_i1_len_331_211952.p1_type_internal_len_111 | ---NA--- | | |
| TRINITY_DN25024_c0_g1_i1_len_1236_194707.p2_type_3prime_partial_len_111 | XP_021930293.1lysoplasmalogenase-like protein TMEM86A isoform X2 | | |

| | | | |
|---|---|---|---|
| TRINITY_DN13492_c0_g1_i1_len_333_281985.p1_type_5prime_partial_len_110 | ---NA--- | | |
| TRINITY_DN12421_c0_g1_i1_len_331_211951.p1_type_internal_len_111 | ---NA--- | | |
| TRINITY_DN932_c0_g1_i1_len_334_281967.p1_type_internal_len_111 | ---NA--- | | |
| TRINITY_DN30152_c0_g1_i1_len_377_136365.p1_type_5prime_partial_len_110 | ---NA--- | | |
| TRINITY_DN30042_c0_g1_i1_len_556_165823.p1_type_3prime_partial_len_111 | ---NA--- | | |
| 2725\|_\|c9890_f4p1_2512_isoform_c9890_full_length_coverage_4_non_full_length_coverage_1_isoform_length_2512_12841 | XP_013402480.1tctex1 domain-containing protein 2-like isoform X1 | | |
| 2726\|_\|c4286_f3p0_2511_isoform_c4286_full_length_coverage_3_non_full_length_coverage_0_isoform_length_2511_16841 | XP_018025798.1 serine palmitoyltransferase 1-like | F:GO:0003824; P:GO:0009058; F:GO:0016740; F:GO:0030170 | |
| 2728\|_\|c4157_f2p4_2511_isoform_c4157_full_length_coverage_2_non_full_length_coverage_4_isoform_length_2511_21409 | PNF42826.1Translation initiation factor IF-2, mitochondrial | F:GO:0003743; F:GO:0003924; F:GO:0005525; C:GO:0005739; P:GO:0070124 | EC:3.6.1.15 |
| TRINITY_DN16101_c0_g1_i1_len_2770_294983.p1_type_5prime_partial_len_837 | XP_018012339.1 serine protease nudel-like | | |
| TRINITY_DN31025_c0_g1_i1_len_4757_187214.p1_type_5prime_partial_len_837 | XP_018562655.1transmembrane 7 superfamily member 3-like | | |
| TRINITY_DN22677_c0_g1_i1_len_2741_229361.p1_type_3prime_partial_len_838 | XP_013788370.1uncharacterized protein KIAA2026-like isoform X1 | | |
| 2733\|_\|c2185_f6p0_2511_isoform_c2185_full_length_coverage_6_non_full_length_coverage_0_isoform_length_2511_11721 | PNF34639.1AP-3 complex subunit mu-1 | | |
| 2734\|_\|c3699_f2p3_2510_isoform_c3699_full_length_coverage_2_non_full_length_coverage_3_isoform_length_2510_16789 | ---NA--- | | |

<table>
<tr><td>2736|_|c10903_f2p0_2510_isoform_c10903_full_length_coverage_2_non_full_length_coverage_0_isoform_length_2510_12925</td><td>XP_021928419.1A-kinase anchor protein 7-like isoform X2</td><td>F:GO:0004866; P:GO:0010951; C:GO:0016020; C:GO:0016021; F:GO:0016301; P:GO:0016310</td><td></td></tr>
<tr><td>2737|_|c20855_f2p2_2510_isoform_c20855_full_length_coverage_2_non_full_length_coverage_2_isoform_length_2510_22817</td><td>---NA---</td><td></td><td></td></tr>
<tr><td>TRINITY_DN15641_c0_g1_i1_len_334_249721.p1_type_internal_len_111</td><td>---NA---</td><td></td><td></td></tr>
<tr><td>TRINITY_DN33432_c0_g1_i1_len_332_249845.p1_type_internal_len_111</td><td>BAL49600.1NADPH oxidase</td><td></td><td></td></tr>
<tr><td>TRINITY_DN12097_c0_g1_i1_len_332_249851.p1_type_internal_len_111</td><td>XP_018015412.1 organic cation transporter protein-like</td><td></td><td></td></tr>
<tr><td>TRINITY_DN15347_c0_g1_i2_len_332_249894.p2_type_internal_len_111</td><td>---NA---</td><td></td><td></td></tr>
<tr><td>TRINITY_DN36102_c0_g1_i1_len_435_206512.p1_type_5prime_partial_len_110</td><td>---NA---</td><td></td><td></td></tr>
<tr><td>TRINITY_DN34537_c0_g1_i1_len_332_249852.p1_type_internal_len_111</td><td>---NA---</td><td></td><td></td></tr>
<tr><td>TRINITY_DN10304_c0_g1_i1_len_332_249858.p1_type_internal_len_111</td><td>---NA---</td><td></td><td></td></tr>
<tr><td>TRINITY_DN5043_c0_g1_i1_len_332_249859.p1_type_internal_len_111</td><td>AFV39705.1Bmal1a</td><td>F:GO:0003677; F:GO:0003700; C:GO:0005634; C:GO:0005667; C:GO:0005737; P:GO:0006355; F:GO:0046983</td><td></td></tr>
<tr><td>TRINITY_DN27761_c0_g1_i1_len_333_249826.p1_type_internal_len_111</td><td>---NA---</td><td></td><td></td></tr>
<tr><td>TRINITY_DN28100_c0_g1_i1_len_333_249829.p1_type_internal_len_111</td><td>---NA---</td><td></td><td></td></tr>
<tr><td>TRINITY_DN16883_c0_g1_i1_len_999_160677.p1_type_complete_len_110</td><td>---NA---</td><td></td><td></td></tr>
<tr><td>TRINITY_DN33466_c0_g1_i1_len_332_249872.p1_type_internal_len_111</td><td>XP_018017684.1 tryptophan 2,3-dioxygenase-like</td><td>F:GO:0004833; P:GO:0019441; P:GO:0019442; F:GO:0020037; F:GO:0046872; P:GO:0055114</td><td>EC:1.13.11; EC:1.13.11.11</td></tr>
</table>

| | | | |
|---|---|---|---|
| TRINITY_DN34563_c0_g1_i1_len_332_249874.p1_type_internal_len_111 | AFE88209.1protaglandin reductase 1 | F:GO:0016491 | |
| TRINITY_DN30483_c0_g1_i5_len_2435_189602.p5_type_complete_len_110 | ---NA--- | | |
| TRINITY_DN16084_c0_g1_i1_len_425_206935.p1_type_complete_len_110 | ---NA--- | | |
| TRINITY_DN15156_c0_g1_i1_len_814_34674.p1_type_5prime_partial_len_110 | ---NA--- | | |
| TRINITY_DN33156_c0_g1_i1_len_334_249776.p1_type_internal_len_111 | ---NA--- | | |
| TRINITY_DN20405_c0_g1_i1_len_716_199747.p1_type_complete_len_110 | ---NA--- | | |
| TRINITY_DN6169_c0_g1_i1_len_331_249923.p1_type_internal_len_111 | ---NA--- | | |
| TRINITY_DN23385_c0_g1_i1_len_334_211783.p1_type_internal_len_111 | XP_018009586.1 protein unzipped-like | | |
| TRINITY_DN41020_c0_g1_i1_len_331_211937.p1_type_internal_len_111 | XP_018019958.1 carbohydrate sulfotransferase 11-like isoform X3 | | |
| TRINITY_DN28938_c0_g1_i1_len_2486_189478.p5_type_5prime_partial_len_110 | ---NA--- | | |
| TRINITY_DN26338_c0_g2_i2_len_334_249764.p1_type_internal_len_111 | ---NA--- | | |
| TRINITY_DN32634_c0_g1_i1_len_333_249802.p1_type_internal_len_111 | XP_018023308.1 sodium channel protein 60E-like | | |
| TRINITY_DN32156_c0_g1_i1_len_331_249902.p1_type_internal_len_111 | ---NA--- | | |
| TRINITY_DN10260_c0_g1_i1_len_334_281913.p1_type_internal_len_111 | ---NA--- | | |
| TRINITY_DN14601_c0_g1_i1_len_404_278840.p2_type_5prime_partial_len_110 | ---NA--- | | |
| TRINITY_DN15565_c0_g1_i1_len_331_249906.p1_type_internal_len_111 | XP_018011424.1 DENN domain-containing protein 5A-like | | |

| | | | |
|---|---|---|---|
| TRINITY_DN3933_c0_g1_i1_len_334_281922.p1_type_internal_len_111 | ---NA--- | | |
| TRINITY_DN12832_c0_g1_i1_len_333_249794.p1_type_internal_len_111 | ---NA--- | | |
| TRINITY_DN27196_c0_g1_i1_len_331_249903.p1_type_internal_len_111 | ASW35099.1influenza virus NS1A-binding protein A-like protein | | |
| TRINITY_DN8421_c0_g1_i1_len_1498_60702.p3_type_5prime_partial_len_110 | ---NA--- | | |
| TRINITY_DN22001_c0_g1_i1_len_331_282137.p1_type_internal_len_111 | ---NA--- | | |
| TRINITY_DN40633_c0_g1_i1_len_331_211927.p1_type_internal_len_111 | ---NA--- | | |
| TRINITY_DN8407_c0_g1_i1_len_386_12251.p1_type_5prime_partial_len_110 | ---NA--- | | |
| TRINITY_DN12521_c0_g1_i2_len_421_103580.p1_type_5prime_partial_len_110 | ---NA--- | | |
| TRINITY_DN11341_c0_g1_i2_len_331_282127.p2_type_internal_len_111 | ---NA--- | | |
| TRINITY_DN840_c0_g1_i2_len_330_282187.p1_type_internal_len_111 | ---NA--- | | |
| TRINITY_DN24684_c0_g1_i2_len_3055_228937.p5_type_complete_len_110 | ---NA--- | | |
| TRINITY_DN34057_c0_g1_i1_len_330_249974.p1_type_internal_len_111 | XP_018022747.1 mannosylglucosyl-3-phosphoglycerate phosphatase-like isoform X1 | | |
| TRINITY_DN29125_c0_g1_i1_len_474_101959.p1_type_5prime_partial_len_110 | ---NA--- | | |
| TRINITY_DN17232_c0_g1_i2_len_1564_157532.p4_type_5prime_partial_len_110 | ---NA--- | | |
| TRINITY_DN37907_c0_g1_i1_len_331_211987.p1_type_internal_len_111 | ---NA--- | | |
| TRINITY_DN7350_c0_g1_i1_len_332_282036.p2_type_internal_len_111 | ---NA--- | | |

| | | | |
|---|---|---|---|
| TRINITY_DN29585_c0_g1_i1_len_331_310198.p1_type_internal_len_111 | PSN32989.1Sorting nexin-17 | C:GO:0005623; P:GO:0006886; F:GO:0035091 | |
| TRINITY_DN26369_c0_g1_i3_len_2275_190024.p3_type_complete_len_110 | ---NA--- | | |
| TRINITY_DN18233_c0_g1_i1_len_332_282059.p2_type_internal_len_111 | ---NA--- | | |
| TRINITY_DN28837_c0_g1_i2_len_2363_189791.p3_type_5prime_partial_len_110 | ---NA--- | | |
| TRINITY_DN28750_c0_g1_i1_len_427_103379.p1_type_5prime_partial_len_110 | ---NA--- | | |
| TRINITY_DN22214_c0_g1_i1_len_331_310200.p1_type_internal_len_111 | ---NA--- | | |
| TRINITY_DN28463_c0_g1_i1_len_2892_188710.p2_type_5prime_partial_len_110 | ---NA--- | | |
| TRINITY_DN7095_c0_g1_i1_len_347_248915.p1_type_5prime_partial_len_110 | ---NA--- | | |
| TRINITY_DN15539_c0_g1_i1_len_331_310193.p1_type_internal_len_111 | ---NA--- | | |
| TRINITY_DN13803_c0_g1_i1_len_331_282101.p2_type_internal_len_111 | ---NA--- | | |
| TRINITY_DN14427_c1_g1_i1_len_331_282098.p2_type_internal_len_111 | ---NA--- | | |
| TRINITY_DN9442_c0_g1_i2_len_528_166435.p1_type_5prime_partial_len_110 | ---NA--- | | |
| TRINITY_DN2065_c0_g1_i1_len_581_302632.p2_type_5prime_partial_len_110 | ---NA--- | | |
| TRINITY_DN19359_c0_g1_i1_len_423_103509.p1_type_5prime_partial_len_110 | ---NA--- | | |
| TRINITY_DN22371_c0_g1_i1_len_332_282066.p1_type_internal_len_111 | ---NA--- | | |
| TRINITY_DN9059_c0_g1_i1_len_332_282060.p1_type_internal_len_111 | AFJ11398.1thromboxane A synthase | F:GO:0005506; C:GO:0016021; F:GO:0016705; F:GO:0020037; P:GO:0055114 | |

| | | | |
|---|---|---|---|
| TRINITY_DN7881_c0_g1_i1_len_331_310204.p2_type_internal_len_111 | ---NA--- | | |
| TRINITY_DN36285_c0_g1_i1_len_330_249964.p2_type_internal_len_111 | ---NA--- | | |
| TRINITY_DN30416_c0_g1_i1_len_337_75515.p1_type_5prime_partial_len_110 | ---NA--- | | |
| TRINITY_DN30360_c0_g1_i1_len_333_173414.p1_type_internal_len_111 | ---NA--- | | |
| TRINITY_DN5452_c0_g1_i1_len_347_172653.p1_type_5prime_partial_len_110 | ---NA--- | | |
| TRINITY_DN27198_c0_g1_i1_len_332_173423.p1_type_internal_len_111 | ---NA--- | | |
| TRINITY_DN18058_c0_g1_i1_len_815_162257.p1_type_complete_len_110 | ---NA--- | | |
| TRINITY_DN23899_c0_g1_i1_len_333_173396.p1_type_internal_len_111 | ---NA--- | | |
| TRINITY_DN18802_c0_g1_i1_len_611_201558.p1_type_complete_len_110 | ---NA--- | | |
| TRINITY_DN39108_c0_g1_i1_len_332_211908.p2_type_internal_len_111 | ---NA--- | | |
| TRINITY_DN23768_c0_g1_i1_len_332_173467.p1_type_internal_len_111 | XP_023715388.1probable phospholipid-transporting ATPase IF isoform X6 | | |
| TRINITY_DN7761_c0_g1_i1_len_350_309201.p1_type_5prime_partial_len_110 | ---NA--- | | |
| TRINITY_DN2758_c0_g1_i1_len_332_173480.p1_type_internal_len_111 | ABW77320.1clottable protein 2 | F:GO:0005319; C:GO:0005576; P:GO:0006869; P:GO:0042381 | |
| TRINITY_DN10937_c0_g1_i1_len_582_202151.p2_type_5prime_partial_len_110 | ---NA--- | | |
| TRINITY_DN23781_c0_g1_i1_len_332_173455.p1_type_internal_len_111 | XP_018021607.1 arylsulfatase I-like | | |
| TRINITY_DN20815_c0_g1_i1_len_1453_193344.p2_type_3prime_partial_len_111 | ---NA--- | | |

| | | | |
|---|---|---|---|
| TRINITY_DN25725_c0_g1_i1_len_807_198524.p1_type_3prime_partial_len_111 | ---NA--- | | |
| TRINITY_DN23276_c0_g1_i1_len_1645_192329.p2_type_3prime_partial_len_111 | ---NA--- | | |
| TRINITY_DN40238_c0_g1_i1_len_332_211861.p1_type_internal_len_111 | ---NA--- | | |
| TRINITY_DN34732_c0_g1_i1_len_334_173320.p1_type_internal_len_111 | XP_018009917.1 tubulin alpha-8 chain-like | F:GO:0003924; F:GO:0005200; F:GO:0005525; C:GO:0005737; C:GO:0005874; P:GO:0007010; P:GO:0007017 | EC:3.6.1.15 |
| TRINITY_DN13471_c0_g1_i1_len_330_212011.p1_type_internal_len_111 | ---NA--- | | |
| TRINITY_DN39277_c0_g1_i1_len_333_211843.p1_type_internal_len_111 | ADV40232.1putative NPDC-1 | C:GO:0016021 | |
| TRINITY_DN1910_c0_g1_i1_len_330_212015.p1_type_internal_len_111 | ---NA--- | | |
| TRINITY_DN29332_c0_g1_i1_len_331_107723.p1_type_internal_len_111 | XP_023023971.1chorion peroxidase | F:GO:0004601; P:GO:0006979; C:GO:0016020; C:GO:0016021; F:GO:0020037; P:GO:0048477; P:GO:0055114; P:GO:0098869 | |
| TRINITY_DN14819_c0_g1_i1_len_1998_231126.p3_type_complete_len_110 | ---NA--- | | |
| TRINITY_DN11023_c0_g1_i1_len_333_173382.p1_type_internal_len_111 | ---NA--- | | |
| TRINITY_DN602_c0_g1_i1_len_334_14717.p1_type_internal_len_111 | ---NA--- | | |
| TRINITY_DN24903_c0_g1_i1_len_334_173345.p1_type_internal_len_111 | ---NA--- | | |
| TRINITY_DN20979_c1_g1_i1_len_331_107727.p1_type_internal_len_111 | ---NA--- | | |
| TRINITY_DN31699_c0_g1_i1_len_330_212004.p1_type_internal_len_111 | ---NA--- | | |

<table>
<tr><th></th><th></th><th></th><th></th></tr>
<tr><td>TRINITY_DN23647_c2_g1_i6_len_1245_159065.p1_type_complete_len_110</td><td>---NA---</td><td></td><td></td></tr>
<tr><td>TRINITY_DN28234_c0_g1_i2_len_1456_193332.p5_type_3prime_partial_len_111</td><td>---NA---</td><td></td><td></td></tr>
<tr><td>TRINITY_DN39174_c0_g1_i1_len_333_211826.p1_type_internal_len_111</td><td>---NA---</td><td></td><td></td></tr>
<tr><td>TRINITY_DN27518_c0_g1_i2_len_1674_192196.p3_type_complete_len_110</td><td>---NA---</td><td></td><td></td></tr>
<tr><td>TRINITY_DN2945_c0_g1_i1_len_331_173500.p1_type_internal_len_111</td><td>---NA---</td><td></td><td></td></tr>
<tr><td>TRINITY_DN30107_c0_g1_i1_len_331_173506.p1_type_internal_len_111</td><td>---NA---</td><td></td><td></td></tr>
<tr><td>TRINITY_DN14544_c0_g1_i1_len_373_73573.p1_type_5prime_partial_len_110</td><td>---NA---</td><td></td><td></td></tr>
<tr><td>TRINITY_DN2754_c0_g1_i1_len_331_173491.p1_type_internal_len_111</td><td>---NA---</td><td></td><td></td></tr>
<tr><td>TRINITY_DN23991_c1_g1_i1_len_2505_229767.p2_type_3prime_partial_len_111</td><td>---NA---</td><td></td><td></td></tr>
<tr><td>TRINITY_DN5753_c0_g1_i1_len_607_66980.p1_type_5prime_partial_len_110</td><td>---NA---</td><td></td><td></td></tr>
<tr><td>2738|_|c2911_f2p4_2509_isoform_c2911_full_length_coverage_2_non_full_length_coverage_4_isoform_length_2509_28300</td><td>---NA---</td><td></td><td></td></tr>
<tr><td>2739|_|c2450_f2p0_2509_isoform_c2450_full_length_coverage_2_non_full_length_coverage_0_isoform_length_2509_20946</td><td>---NA---</td><td></td><td></td></tr>
<tr><td>TRINITY_DN30903_c0_g1_i4_len_2983_188564.p1_type_5prime_partial_len_836</td><td>XP_021346938.1uncharacterized protein LOC110446222, partial</td><td></td><td></td></tr>
<tr><td>TRINITY_DN30209_c0_g1_i1_len_2877_188734.p1_type_complete_len_836</td><td>---NA---</td><td></td><td></td></tr>
<tr><td>2743|_|c36173_f2p8_2508_isoform_c36173_full_length_coverage_2_non_full_length_coverage_8_isoform_length_2508_23557</td><td>---NA---</td><td></td><td></td></tr>
</table>

| | | | |
|---|---|---|---|
| TRINITY_DN30072_c0_g1_i1_len_4132_187514.p1_type_5prime_partial_len_836 | XP_018018264.1 tensin-1-like | | |
| 2746\|_\|c4818_f2p1_2508_isoform_c4818_full_length_coverage_2_non_full_length_coverage_1_isoform_length_2508_21470 | PNF28940.1DDB1- and CUL4-associated factor 12-B | | |
| 2747\|_\|c10079_f2p1_2507_isoform_c10079_full_length_coverage_2_non_full_length_coverage_1_isoform_length_2507_17328 | ---NA--- | | |
| 2748\|_\|c11790_f2p0_2507_isoform_c11790_full_length_coverage_2_non_full_length_coverage_0_isoform_length_2507_22082 | XP_025093144.1coiled-coil domain-containing protein 93-like isoform X1 | | |
| 2749\|_\|c2848_f3p1_2507_isoform_c2848_full_length_coverage_3_non_full_length_coverage_1_isoform_length_2507_12211 | ---NA--- | | |
| TRINITY_DN17477_c0_g1_i1_len_332_14873.p1_type_internal_len_111 | ---NA--- | | |
| TRINITY_DN19906_c0_g1_i1_len_330_15015.p1_type_internal_len_111 | ---NA--- | | |
| TRINITY_DN15354_c0_g1_i1_len_808_162346.p2_type_5prime_partial_len_110 | ---NA--- | | |
| TRINITY_DN9828_c0_g1_i1_len_330_173549.p1_type_internal_len_111 | ---NA--- | | |
| TRINITY_DN24196_c0_g1_i1_len_330_173558.p1_type_internal_len_111 | ---NA--- | | |
| TRINITY_DN27388_c0_g1_i1_len_331_173521.p1_type_internal_len_111 | ---NA--- | | |
| TRINITY_DN40645_c0_g1_i1_len_330_212033.p1_type_internal_len_111 | XP_023239637.1WD repeat-containing protein 81-like | | |
| TRINITY_DN39684_c0_g1_i1_len_332_211853.p1_type_internal_len_111 | ---NA--- | | |

| | | | |
|---|---|---|---|
| TRINITY_DN28602_c0_g1_i1_len_331_107676.p1_type_internal_len_111 | ABH00987.1rhodopsin, partial | F:GO:0004930; P:GO:0007186; P:GO:0007601; P:GO:0007602; F:GO:0009881; C:GO:0016021; P:GO:0018298 | |
| TRINITY_DN31061_c0_g1_i1_len_332_211854.p1_type_internal_len_111 | ---NA--- | | |
| TRINITY_DN7086_c0_g1_i1_len_387_170752.p1_type_5prime_partial_len_110 | ---NA--- | | |
| TRINITY_DN17039_c0_g1_i1_len_330_212022.p2_type_internal_len_111 | ---NA--- | | |
| TRINITY_DN27337_c0_g1_i1_len_401_72328.p1_type_5prime_partial_len_110 | ---NA--- | | |
| TRINITY_DN1913_c0_g1_i1_len_333_107572.p1_type_internal_len_111 | AUB30148.1fast-type skeletal muscle actin 3 | F:GO:0005524 | |
| TRINITY_DN24437_c0_g1_i1_len_333_107571.p1_type_internal_len_111 | ---NA--- | | |
| TRINITY_DN35567_c0_g1_i1_len_332_211852.p1_type_internal_len_111 | ---NA--- | | |
| TRINITY_DN32455_c0_g1_i1_len_471_205159.p1_type_5prime_partial_len_110 | XP_018007455.1 longitudinals lacking protein, isoforms H/M/V-like | | |
| TRINITY_DN24665_c0_g1_i1_len_333_107585.p2_type_internal_len_111 | ABK56706.1notch protein | F:GO:0005509; P:GO:0007219; P:GO:0007275; C:GO:0016021; P:GO:0030154; P:GO:0050793 | |
| TRINITY_DN9498_c0_g1_i1_len_558_7686.p1_type_3prime_partial_len_111 | ---NA--- | | |
| TRINITY_DN19220_c0_g2_i1_len_864_271212.p1_type_5prime_partial_len_110 | ---NA--- | | |
| TRINITY_DN23645_c0_g1_i2_len_1633_232387.p3_type_5prime_partial_len_110 | ---NA--- | | |
| TRINITY_DN18014_c0_g1_i1_len_1088_235462.p1_type_5prime_partial_len_110 | ---NA--- | | |
| TRINITY_DN3634_c0_g1_i1_len_332_211897.p1_type_internal_len_111 | ---NA--- | | |

| | | | |
|---|---|---|---|
| TRINITY_DN20260_c0_g1_i2_len_3778_29285.p4_type_3prime_partial_len_111 | ---NA--- | | |
| TRINITY_DN18023_c0_g1_i2_len_334_44369.p1_type_internal_len_111 | ---NA--- | | |
| TRINITY_DN25221_c0_g1_i1_len_332_107666.p1_type_internal_len_111 | ---NA--- | | |
| TRINITY_DN7184_c0_g1_i2_len_426_206855.p2_type_complete_len_110 | XP_018023119.1 zinc finger and BTB domain-containing protein 17-like isoform X1 | | |
| TRINITY_DN32288_c0_g1_i1_len_333_14800.p2_type_internal_len_111 | ---NA--- | | |
| TRINITY_DN9931_c0_g1_i2_len_333_14836.p2_type_internal_len_111 | ---NA--- | | |
| TRINITY_DN22885_c0_g1_i1_len_1554_232747.p1_type_5prime_partial_len_110 | ---NA--- | | |
| TRINITY_DN14147_c0_g1_i2_len_2002_266303.p2_type_complete_len_110 | ---NA--- | | |
| TRINITY_DN3569_c0_g1_i1_len_332_211887.p2_type_internal_len_111 | XP_018006830.1 transmembrane protease serine 9-like | | |
| TRINITY_DN18798_c0_g1_i1_len_1293_268632.p2_type_3prime_partial_len_111 | ---NA--- | | |
| TRINITY_DN31220_c1_g3_i2_len_388_208607.p1_type_5prime_partial_len_110 | ---NA--- | | |
| TRINITY_DN12383_c0_g1_i1_len_375_171268.p2_type_5prime_partial_len_110 | ---NA--- | | |
| TRINITY_DN2943_c0_g1_i1_len_332_211907.p1_type_internal_len_111 | ---NA--- | | |
| TRINITY_DN35828_c0_g1_i1_len_332_211851.p1_type_internal_len_111 | ---NA--- | | |
| TRINITY_DN17723_c0_g1_i1_len_331_107680.p2_type_internal_len_111 | ---NA--- | | |
| TRINITY_DN19263_c0_g1_i3_len_1286_158838.p2_type_complete_len_110 | ---NA--- | | |

| | | | |
|---|---|---|---|
| TRINITY_DN19151_c0_g1_i1_len_550_241767.p1_type_complete_len_110 | ---NA--- | | |
| TRINITY_DN2495_c0_g1_i1_len_330_212028.p1_type_internal_len_111 | ---NA--- | | |
| TRINITY_DN33410_c0_g1_i1_len_332_211917.p1_type_internal_len_111 | ---NA--- | | |
| TRINITY_DN15428_c0_g1_i1_len_869_161771.p2_type_5prime_partial_len_110 | ---NA--- | | |
| TRINITY_DN10751_c0_g1_i2_len_331_14926.p1_type_internal_len_111 | ---NA--- | | |
| TRINITY_DN27291_c0_g1_i1_len_949_197002.p2_type_complete_len_110 | ---NA--- | | |
| TRINITY_DN5330_c0_g1_i1_len_533_275305.p1_type_5prime_partial_len_110 | ---NA--- | | |
| TRINITY_DN9363_c0_g1_i1_len_332_211863.p1_type_internal_len_111 | ---NA--- | | |
| TRINITY_DN30693_c0_g1_i1_len_333_138707.p1_type_internal_len_111 | ---NA--- | | |
| TRINITY_DN25869_c0_g2_i3_len_468_205275.p1_type_complete_len_110 | ---NA--- | | |
| TRINITY_DN13824_c0_g1_i1_len_773_238264.p1_type_5prime_partial_len_110 | ---NA--- | | |
| TRINITY_DN41372_c0_g1_i1_len_330_212041.p1_type_internal_len_111 | ---NA--- | | |
| TRINITY_DN3652_c0_g1_i1_len_332_211877.p1_type_internal_len_111 | XP_018021626.1 kielin/chordin-like protein | | |
| TRINITY_DN15390_c0_g1_i1_len_405_207802.p1_type_3prime_partial_len_111 | ---NA--- | | |
| TRINITY_DN21952_c0_g1_i2_len_724_199660.p1_type_3prime_partial_len_111 | ---NA--- | | |
| TRINITY_DN42201_c0_g1_i1_len_374_209326.p1_type_5prime_partial_len_110 | ---NA--- | | |

| | | | |
|---|---|---|---|
| TRINITY_DN6153_c0_g1_i1_len_332_138733.p1_type_internal_len_111 | ---NA--- | | |
| TRINITY_DN15482_c0_g1_i1_len_463_168082.p1_type_5prime_partial_len_110 | ---NA--- | | |
| TRINITY_DN5376_c0_g1_i1_len_331_14945.p1_type_internal_len_111 | BAD16776.1calcification-associated peptide-2 | F:GO:0042302 | |
| TRINITY_DN18637_c0_g1_i1_len_1713_1211.p3_type_5prime_partial_len_110 | SCN12114.1conserved Plasmodium protein, unknown function | F:GO:0046872 | |
| TRINITY_DN822_c0_g1_i1_len_331_138823.p1_type_internal_len_111 | ---NA--- | | |
| TRINITY_DN26203_c0_g1_i1_len_331_138825.p1_type_internal_len_111 | EDO42086.1predicted protein | C:GO:0005623; P:GO:0045454 | |
| TRINITY_DN14592_c0_g1_i2_len_2075_92014.p2_type_3prime_partial_len_111 | ---NA--- | | |
| TRINITY_DN146_c0_g1_i1_len_331_211922.p1_type_internal_len_111 | ---NA--- | | |
| TRINITY_DN18157_c0_g2_i1_len_422_306249.p1_type_5prime_partial_len_110 | ---NA--- | | |
| TRINITY_DN32300_c0_g1_i1_len_330_138883.p2_type_internal_len_111 | PNF17156.1hypothetical protein B7P43_G09088 | | |
| TRINITY_DN33899_c0_g1_i1_len_331_138837.p1_type_internal_len_111 | ---NA--- | | |
| TRINITY_DN18384_c0_g1_i4_len_762_162809.p1_type_5prime_partial_len_110 | ---NA--- | | |
| TRINITY_DN9975_c0_g1_i1_len_460_168167.p3_type_5prime_partial_len_110 | ---NA--- | | |
| TRINITY_DN6771_c0_g1_i1_len_376_247471.p1_type_5prime_partial_len_110 | ---NA--- | | |
| TRINITY_DN10139_c0_g1_i1_len_758_35145.p1_type_5prime_partial_len_110 | ---NA--- | | |
| TRINITY_DN26223_c0_g1_i1_len_342_309596.p1_type_5prime_partial_len_110 | ---NA--- | | |

| | | | |
|---|---|---|---|
| TRINITY_DN24461_c0_g1_i1_len_710_65547.p1_type_complete_len_110 | ---NA--- | | |
| TRINITY_DN5418_c0_g1_i1_len_377_247424.p1_type_5prime_partial_len_110 | ---NA--- | | |
| TRINITY_DN4229_c0_g1_i2_len_332_44458.p1_type_internal_len_111 | ---NA--- | | |
| TRINITY_DN4142_c0_g1_i1_len_782_97212.p2_type_5prime_partial_len_110 | ---NA--- | | |
| TRINITY_DN30177_c0_g1_i1_len_331_14901.p1_type_internal_len_111 | ---NA--- | | |
| TRINITY_DN4559_c0_g1_i1_len_330_211998.p1_type_internal_len_111 | ---NA--- | | |
| TRINITY_DN15603_c0_g1_i1_len_381_208954.p1_type_5prime_partial_len_110 | ---NA--- | | |
| TRINITY_DN3145_c0_g1_i1_len_333_44429.p1_type_internal_len_111 | XP_017781556.1 otoferlin-like isoform X1 | | |
| TRINITY_DN20538_c0_g1_i1_len_394_11937.p2_type_5prime_partial_len_110 | ---NA--- | | |
| TRINITY_DN19651_c0_g3_i2_len_333_44430.p1_type_internal_len_111 | ---NA--- | | |
| TRINITY_DN2334_c0_g1_i1_len_331_14899.p1_type_internal_len_111 | ---NA--- | | |
| TRINITY_DN30998_c0_g1_i1_len_333_44434.p1_type_internal_len_111 | ---NA--- | | |
| TRINITY_DN31388_c0_g1_i1_len_359_106174.p1_type_5prime_partial_len_110 | ---NA--- | | |
| TRINITY_DN19439_c0_g1_i1_len_1927_156330.p1_type_5prime_partial_len_110 | ---NA--- | | |
| TRINITY_DN36651_c0_g1_i1_len_332_211916.p1_type_internal_len_111 | XP_020383563.1DNA repair protein REV1 isoform X2 | | |
| TRINITY_DN28606_c0_g1_i1_len_332_14883.p1_type_internal_len_111 | ---NA--- | | |

| | | | |
|---|---|---|---|
| TRINITY_DN31207_c0_g1_i1_len_436_245027.p1_type_5prime_partial_len_110 | CBG22733.1northern shrimp nuclease | F:GO:0003676; F:GO:0016787; F:GO:0046872 | |
| TRINITY_DN22121_c0_g1_i1_len_1654_232302.p1_type_5prime_partial_len_110 | ---NA--- | | |
| TRINITY_DN11054_c0_g1_i1_len_641_200986.p1_type_complete_len_110 | ---NA--- | | |
| TRINITY_DN12401_c0_g1_i2_len_354_106433.p1_type_3prime_partial_len_111 | ---NA--- | | |
| TRINITY_DN15829_c0_g1_i1_len_442_206237.p1_type_3prime_partial_len_111 | XP_018027529.1 GPI mannosyltransferase 2-like | | |
| TRINITY_DN8669_c0_g1_i1_len_418_207211.p1_type_3prime_partial_len_111 | ---NA--- | | |
| TRINITY_DN20999_c0_g1_i1_len_1118_235255.p1_type_3prime_partial_len_111 | ---NA--- | | |
| TRINITY_DN25692_c0_g1_i1_len_362_74136.p1_type_5prime_partial_len_110 | ---NA--- | | |
| TRINITY_DN6112_c0_g1_i1_len_330_211996.p1_type_internal_len_111 | ---NA--- | | |
| TRINITY_DN19114_c0_g1_i1_len_332_211864.p1_type_internal_len_111 | ---NA--- | | |
| TRINITY_DN29038_c0_g1_i1_len_366_171679.p1_type_5prime_partial_len_110 | ---NA--- | | |
| TRINITY_DN7363_c0_g1_i1_len_360_106119.p1_type_5prime_partial_len_110 | ---NA--- | | |
| TRINITY_DN20800_c0_g1_i2_len_440_244879.p1_type_5prime_partial_len_110 | ---NA--- | | |
| TRINITY_DN25639_c0_g1_i2_len_7195_227507.p1_type_complete_len_1461 | XP_018017599.1 protein strawberry notch homolog 1-like isoform X4 | | |
| TRINITY_DN26345_c0_g1_i4_len_5166_227755.p1_type_complete_len_1458 | KZS08586.1DNA topoisomerase 2-binding protein 1 | F:GO:0016853; P:GO:0033314 | |
| 291\|_\|c29416_f27p3_4372_isoform_c29416_full_length_coverage_27_non_full_length_coverage_3_isoform_length_4372_29320 | ACV60547.1polehole-like protein | | |

<table>
<tr><td>TRINITY_DN31201_c0_g1_i1_len_4798_187199.p1_type_complete_len_1454</td><td>KZC05752.1snRNA-activating protein complex subunit 1</td><td></td><td></td></tr>
<tr><td>TRINITY_DN25457_c0_g1_i3_len_4646_227894.p1_type_5prime_partial_len_1450</td><td>XP_018007330.1 mucin-19-like</td><td></td><td></td></tr>
<tr><td>TRINITY_DN30626_c0_g1_i1_len_4848_187183.p1_type_complete_len_1447</td><td>XP_022104971.1Fanconi anemia group D2 protein-like isoform X2</td><td></td><td></td></tr>
<tr><td>296|_|c1615_f2p78_4326_isoform_c1615_full_length_coverage_2_non_full_length_coverage_78_isoform_length_4326_25444</td><td>EEB19444.1Heterogeneous nuclear ribonucleoprotein A1, putative</td><td>F:GO:0003723</td><td></td></tr>
<tr><td>TRINITY_DN29670_c0_g1_i1_len_7236_186794.p1_type_complete_len_1441</td><td>KZS21435.1LA-related protein 1</td><td>F:GO:0003723</td><td></td></tr>
<tr><td>TRINITY_DN24971_c0_g1_i1_len_4456_57761.p1_type_5prime_partial_len_1441</td><td>XP_022237497.1ninein-like protein isoform X1</td><td></td><td></td></tr>
<tr><td>TRINITY_DN25921_c0_g1_i1_len_4686_227878.p1_type_5prime_partial_len_1438</td><td>XP_018011797.1 E3 ubiquitin-protein ligase HUWE1-like</td><td></td><td></td></tr>
<tr><td>2750|_|c3806_f3p2_2507_isoform_c3806_full_length_coverage_3_non_full_length_coverage_2_isoform_length_2507_12325</td><td>AGI96976.1stretchin-Mlck, partial</td><td>F:GO:0004674; F:GO:0005524; P:GO:0006468</td><td>EC:2.7.11</td></tr>
<tr><td>2751|_|c8226_f2p2_2506_isoform_c8226_full_length_coverage_2_non_full_length_coverage_2_isoform_length_2506_21761</td><td>XP_018013920.1 host cell factor 2-like</td><td>C:GO:0035097</td><td></td></tr>
<tr><td>2752|_|c1568_f9p4_2506_isoform_c1568_full_length_coverage_9_non_full_length_coverage_4_isoform_length_2506_15900</td><td>ANJ04741.1heat shock 70 kDa protein cognate 5</td><td>F:GO:0005524; P:GO:0006457; F:GO:0051082</td><td></td></tr>
<tr><td>2753|_|c18120_f2p6_2506_isoform_c18120_full_length_coverage_2_non_full_length_coverage_6_isoform_length_2506_22701</td><td>XP_022059196.1poly [ADP-ribose] polymerase 2</td><td></td><td></td></tr>
<tr><td>2754|_|c3575_f2p1_2506_isoform_c3575_full_length_coverage_2_non_full_length_coverage_1_isoform_length_2506_12310</td><td>---NA---</td><td></td><td></td></tr>
<tr><td>2755|_|c18158_f2p0_2505_isoform_c18158_full_length_coverage_2_non_full_length_coverage_0_isoform_length_2505_17991</td><td>AXU05739.1 kinase C</td><td>F:GO:0004697; F:GO:0005524; P:GO:0006468; P:GO:0035556; F:GO:0046872</td><td>EC:2.7.11; EC:2.7.11.13</td></tr>
</table>

| | | | |
|---|---|---|---|
| TRINITY_DN22867_c0_g1_i1_len_2645_229526.p1_type_5prime_partial_len_835 | XP_013028606.1 N-acetylglucosamine-1-phosphotransferase subunits alpha/beta isoform X3 | | |
| 2758\|_\|c5963_f2p2_2504_isoform_c5963_full_length_coverage_2_non_full_length_coverage_2_isoform_length_2504_12516 | XP_025088334.1 N-alpha-acetyltransferase 15, NatA auxiliary subunit-like | | |
| 2761\|_\|c2731_f2p1_2503_isoform_c2731_full_length_coverage_2_non_full_length_coverage_1_isoform_length_2503_16653 | XP_018015947.1 ubiA prenyltransferase domain-containing protein 1 homolog | F:GO:0004659; C:GO:0016021 | |
| 2762\|_\|c18023_f3p0_2503_isoform_c18023_full_length_coverage_3_non_full_length_coverage_0_isoform_length_2503_22639 | XP_025191690.1glucose-6-phosphate exchanger SLC37A2 isoform X2 | | |
| TRINITY_DN1613_c0_g1_i2_len_333_138679.p1_type_internal_len_111 | XP_009903399.1 abhydrolase domain-containing protein 1 | | |
| TRINITY_DN17743_c0_g1_i1_len_1017_298724.p2_type_complete_len_109 | ---NA--- | | |
| TRINITY_DN31905_c0_g1_i1_len_328_107891.p1_type_internal_len_110 | ---NA--- | | |
| TRINITY_DN27599_c0_g1_i1_len_328_15093.p1_type_internal_len_110 | ---NA--- | | |
| TRINITY_DN10927_c0_g1_i1_len_890_197595.p3_type_5prime_partial_len_109 | ---NA--- | | |
| TRINITY_DN18594_c0_g1_i1_len_648_301727.p1_type_complete_len_109 | ---NA--- | | |
| TRINITY_DN19883_c1_g1_i1_len_530_8221.p1_type_5prime_partial_len_109 | ---NA--- | | |
| TRINITY_DN33915_c0_g1_i1_len_330_212027.p1_type_internal_len_110 | XP_023210970.1E3 ubiquitin-protein ligase ubr3-like | | |
| TRINITY_DN18435_c0_g1_i1_len_1584_232618.p1_type_complete_len_109 | ---NA--- | | |
| TRINITY_DN22608_c0_g1_i1_len_1274_125110.p3_type_5prime_partial_len_109 | ---NA--- | | |

| | | | |
|---|---|---|---|
| TRINITY_DN11800_c0_g1_i1_len_330_107740.p1_type_internal_len_110 | ---NA--- | | |
| TRINITY_DN5827_c0_g1_i1_len_329_107813.p1_type_internal_len_110 | ---NA--- | | |
| TRINITY_DN29894_c0_g1_i1_len_329_310288.p1_type_internal_len_110 | ---NA--- | | |
| TRINITY_DN13967_c0_g1_i1_len_3269_294795.p4_type_complete_len_109 | ENN80311.1hypothetical protein YQE_03304, partial | | |
| TRINITY_DN15046_c0_g1_i1_len_488_204624.p1_type_complete_len_109 | ---NA--- | | |
| TRINITY_DN3146_c0_g1_i1_len_329_107835.p2_type_internal_len_110 | ---NA--- | | |
| TRINITY_DN29942_c0_g1_i1_len_328_76071.p1_type_internal_len_110 | ---NA--- | | |
| TRINITY_DN32658_c0_g1_i1_len_491_243269.p1_type_5prime_partial_len_109 | BAM35674.1putative reverse transcriptase | F:GO:0003964; P:GO:0006278 | EC:2.7.7.49 |
| TRINITY_DN5511_c0_g1_i1_len_1134_125761.p3_type_complete_len_109 | ---NA--- | | |
| TRINITY_DN6071_c0_g1_i1_len_328_107952.p1_type_internal_len_110 | ---NA--- | | |
| TRINITY_DN30350_c0_g1_i1_len_327_76114.p2_type_internal_len_110 | ---NA--- | | |
| TRINITY_DN30325_c0_g1_i1_len_327_76092.p1_type_internal_len_110 | ---NA--- | | |
| TRINITY_DN20554_c0_g1_i1_len_637_240170.p1_type_complete_len_109 | ---NA--- | | |
| TRINITY_DN17824_c0_g1_i1_len_540_8014.p1_type_5prime_partial_len_109 | ---NA--- | | |
| TRINITY_DN12321_c0_g1_i1_len_330_107771.p1_type_internal_len_110 | ---NA--- | | |
| TRINITY_DN30588_c0_g1_i1_len_328_15126.p1_type_internal_len_110 | XP_026473720.1probable peroxisomal acyl-coenzyme A oxidase 1 | | |

| | | | |
|---|---|---|---|
| TRINITY_DN8780_c0_g1_i1_len_329_310287.p2_type_internal_len_110 | ---NA--- | | |
| TRINITY_DN35434_c0_g1_i1_len_344_211127.p1_type_5prime_partial_len_109 | ---NA--- | | |
| TRINITY_DN32051_c0_g1_i1_len_353_210544.p1_type_5prime_partial_len_109 | ---NA--- | | |
| TRINITY_DN31574_c0_g1_i1_len_328_76039.p1_type_internal_len_110 | ---NA--- | | |
| TRINITY_DN20280_c0_g1_i1_len_1214_194854.p1_type_5prime_partial_len_109 | ---NA--- | | |
| TRINITY_DN25913_c0_g1_i2_len_3155_228817.p2_type_complete_len_109 | ---NA--- | | |
| TRINITY_DN25684_c0_g1_i1_len_328_76048.p1_type_internal_len_110 | ---NA--- | | |
| TRINITY_DN32032_c0_g1_i1_len_330_212031.p1_type_internal_len_110 | ---NA--- | | |
| TRINITY_DN29967_c0_g1_i1_len_328_76046.p1_type_internal_len_110 | XP_018022538.1PREDICTED: uncharacterized protein LOC108678590 | | |
| TRINITY_DN11574_c0_g1_i2_len_397_208161.p1_type_5prime_partial_len_109 | XP_018023324.1 branched-chain-amino-acid aminotransferase, cytosolic-like | F:GO:0004084; C:GO:0005739; P:GO:0009098; P:GO:0009099 | EC:2.6.1.42 |
| TRINITY_DN15026_c0_g1_i1_len_487_204661.p1_type_5prime_partial_len_109 | ---NA--- | | |
| TRINITY_DN11682_c0_g1_i1_len_328_15114.p1_type_internal_len_110 | ---NA--- | | |
| TRINITY_DN26454_c0_g1_i1_len_383_247123.p1_type_5prime_partial_len_109 | XP_022195772.1gastrula zinc finger protein XlCGF57.1-like isoform X2 | | |
| TRINITY_DN33051_c0_g1_i1_len_686_239421.p1_type_complete_len_109 | ---NA--- | | |
| TRINITY_DN40675_c0_g1_i1_len_330_212051.p1_type_internal_len_110 | ---NA--- | | |
| TRINITY_DN20702_c0_g1_i1_len_946_299118.p2_type_complete_len_109 | ---NA--- | | |

| | | | |
|---|---|---|---|
| TRINITY_DN23913_c2_g1_i1_len_667_129386.p1_type_complete_len_109 | ---NA--- | | |
| TRINITY_DN24404_c0_g1_i3_len_1480_233115.p1_type_complete_len_109 | XP_018019282.1 transcription factor MafG-like | | |
| TRINITY_DN6778_c0_g1_i1_len_532_275343.p1_type_5prime_partial_len_109 | ---NA--- | | |
| TRINITY_DN16511_c0_g1_i2_len_548_68043.p3_type_complete_len_109 | ---NA--- | | |
| TRINITY_DN21336_c0_g1_i1_len_3017_188516.p5_type_5prime_partial_len_109 | ---NA--- | | |
| TRINITY_DN19222_c0_g2_i2_len_543_275112.p2_type_3prime_partial_len_110 | ---NA--- | | |
| TRINITY_DN8924_c0_g1_i1_len_418_103694.p1_type_5prime_partial_len_109 | XP_018023308.1 sodium channel protein 60E-like | C:GO:0001518; F:GO:0005244; F:GO:0005248; P:GO:0034765; P:GO:0035725 | |
| TRINITY_DN29695_c0_g1_i1_len_376_279897.p1_type_5prime_partial_len_109 | XP_018014905.1PREDICTED: uncharacterized protein LOC108671825 | | |
| TRINITY_DN4905_c0_g1_i1_len_366_209773.p1_type_5prime_partial_len_109 | ---NA--- | | |
| TRINITY_DN16913_c0_g1_i1_len_1787_92562.p4_type_5prime_partial_len_109 | XP_025415833.1prolyl 4-hydroxylase subunit alpha-1-like | | |
| TRINITY_DN17750_c0_g1_i1_len_1754_231930.p3_type_complete_len_109 | ---NA--- | | |
| TRINITY_DN974_c0_g1_i1_len_689_65806.p1_type_5prime_partial_len_109 | ---NA--- | | |
| TRINITY_DN6695_c0_g1_i1_len_521_131897.p1_type_complete_len_109 | ---NA--- | | |
| TRINITY_DN15234_c0_g1_i1_len_766_238351.p3_type_complete_len_109 | XP_020282282.1uncharacterized protein LOC109854019 isoform X1 | | |
| TRINITY_DN16651_c0_g1_i1_len_655_239870.p1_type_complete_len_109 | ---NA--- | | |
| TRINITY_DN4176_c0_g1_i1_len_692_239333.p1_type_5prime_partial_len_109 | ---NA--- | | |

| | | | |
|---|---|---|---|
| TRINITY_DN1334_c0_g1_i1_len_766_238343.p1_type_complete_len_109 | ---NA--- | | |
| TRINITY_DN4648_c0_g1_i1_len_412_103891.p1_type_5prime_partial_len_109 | ---NA--- | | |
| TRINITY_DN19600_c0_g1_i1_len_361_106078.p1_type_5prime_partial_len_109 | XP_018010987.1 pro-resilin-like | | |
| TRINITY_DN19179_c0_g1_i1_len_1428_233359.p1_type_5prime_partial_len_109 | ---NA--- | | |
| TRINITY_DN31270_c0_g1_i2_len_1140_195381.p2_type_5prime_partial_len_109 | ---NA--- | | |
| TRINITY_DN20461_c0_g1_i1_len_565_202542.p1_type_complete_len_109 | ---NA--- | | |
| TRINITY_DN1855_c0_g1_i1_len_458_244286.p1_type_5prime_partial_len_109 | ---NA--- | | |
| TRINITY_DN7558_c0_g1_i1_len_384_105019.p1_type_5prime_partial_len_109 | ---NA--- | | |
| TRINITY_DN21242_c0_g1_i1_len_942_161159.p1_type_complete_len_109 | ---NA--- | | |
| TRINITY_DN26167_c0_g1_i2_len_3227_188211.p2_type_complete_len_109 | ---NA--- | | |
| TRINITY_DN37839_c0_g1_i1_len_364_248111.p1_type_complete_len_109 | ---NA--- | | |
| TRINITY_DN24133_c0_g1_i2_len_1310_61450.p3_type_5prime_partial_len_109 | ---NA--- | | |
| TRINITY_DN31476_c0_g1_i1_len_421_207090.p2_type_complete_len_109 | XP_018010166.1 cuticle protein 7-like | F:GO:0042302 | |
| TRINITY_DN24669_c0_g1_i1_len_770_162713.p1_type_complete_len_109 | ---NA--- | | |
| TRINITY_DN22297_c0_g1_i1_len_722_199690.p1_type_3prime_partial_len_110 | ---NA--- | | |
| TRINITY_DN19514_c0_g1_i1_len_508_132198.p1_type_complete_len_109 | ---NA--- | | |

| | | | |
|---|---|---|---|
| TRINITY_DN14518_c0_g1_i1_len_372_105545.p1_type_5prime_partial_len_109 | ---NA--- | | |
| TRINITY_DN15079_c0_g1_i1_len_682_239463.p1_type_5prime_partial_len_109 | ---NA--- | | |
| TRINITY_DN25588_c0_g1_i1_len_370_105653.p1_type_3prime_partial_len_110 | ---NA--- | | |
| TRINITY_DN19676_c0_g1_i1_len_625_129980.p2_type_5prime_partial_len_109 | XP_014817019.1 transcription initiation factor TFIID subunit 10 | | |
| TRINITY_DN13842_c0_g1_i1_len_782_238165.p2_type_5prime_partial_len_109 | ---NA--- | | |
| TRINITY_DN23713_c0_g1_i1_len_588_274313.p1_type_5prime_partial_len_109 | XP_006619139.1 cytochrome c oxidase assembly factor 6 homolog isoform X2 | C:GO:0005739 | |
| TRINITY_DN902_c0_g1_i1_len_331_211980.p1_type_3prime_partial_len_110 | PNJ75059.1RPL15 isoform 13 | F:GO:0003735; C:GO:0005840; P:GO:0006412 | |
| TRINITY_DN15024_c0_g1_i1_len_329_212064.p1_type_internal_len_110 | ---NA--- | | |
| TRINITY_DN25746_c0_g1_i1_len_2492_229803.p3_type_5prime_partial_len_109 | ---NA--- | | |
| TRINITY_DN34414_c0_g1_i1_len_330_212018.p1_type_internal_len_110 | ---NA--- | | |
| TRINITY_DN35397_c0_g1_i1_len_330_212042.p1_type_5prime_partial_len_109 | ---NA--- | | |
| TRINITY_DN32335_c0_g1_i1_len_415_207359.p1_type_3prime_partial_len_110 | XP_012244328.1roquin-1 isoform X2 | | |
| TRINITY_DN10462_c0_g1_i1_len_327_310451.p1_type_internal_len_110 | ---NA--- | | |
| TRINITY_DN20391_c0_g1_i1_len_784_64736.p3_type_5prime_partial_len_109 | XP_019333824.1 L-xylulose reductase | | |
| TRINITY_DN20287_c0_g1_i1_len_1553_232759.p3_type_5prime_partial_len_109 | ---NA--- | | |
| TRINITY_DN40709_c0_g1_i1_len_331_211976.p1_type_internal_len_110 | XP_023321377.1uncharacterized protein LOC111696079 isoform X3 | | |

| | | | |
|---|---|---|---|
| TRINITY_DN27098_c0_g1_i1_len_328_310355.p1_type_internal_len_110 | ---NA--- | | |
| TRINITY_DN2853_c0_g1_i1_len_329_212066.p1_type_internal_len_110 | ---NA--- | | |
| TRINITY_DN19130_c0_g1_i2_len_3509_187924.p6_type_5prime_partial_len_109 | ---NA--- | | |
| TRINITY_DN29921_c0_g1_i1_len_443_102874.p1_type_3prime_partial_len_110 | ---NA--- | | |
| TRINITY_DN32174_c0_g1_i1_len_329_212065.p1_type_internal_len_110 | ---NA--- | | |
| TRINITY_DN10452_c0_g1_i1_len_738_199468.p3_type_5prime_partial_len_109 | ---NA--- | | |
| TRINITY_DN39327_c0_g1_i1_len_331_211978.p1_type_internal_len_110 | ---NA--- | | |
| TRINITY_DN13198_c0_g1_i1_len_417_207256.p1_type_5prime_partial_len_109 | ---NA--- | | |
| TRINITY_DN13631_c0_g1_i1_len_614_273934.p1_type_5prime_partial_len_109 | XP_018026292.1 sodium- and chloride-dependent glycine transporter 2-like | | |
| TRINITY_DN16798_c0_g1_i1_len_825_237690.p1_type_5prime_partial_len_109 | ---NA--- | | |
| TRINITY_DN23530_c0_g1_i1_len_2680_58404.p1_type_5prime_partial_len_834 | XP_019645031.1 conserved oligomeric Golgi complex subunit 3-like | P:GO:0006891; P:GO:0007030; F:GO:0008565; C:GO:0017119 | |
| TRINITY_DN18565_c0_g1_i1_len_2505_154997.p1_type_internal_len_835 | ---NA--- | | |
| 2766\|_\|c22602_f2p3_2502_isoform_c22602_full_length_coverage_2_non_full_length_coverage_3_isoform_length_2502_22881 | XP_023229328.1tyrosine-protein phosphatase non-receptor type 4-like isoform X2 | P:GO:0016311; F:GO:0016791 | |
| TRINITY_DN30025_c0_g1_i1_len_3025_188496.p1_type_5prime_partial_len_834 | XP_006822145.1 DNA helicase MCM8-like | F:GO:0000166 | |
| 2770\|_\|c7115_f3p1_2500_isoform_c7115_full_length_coverage_3_non_full_length_coverage_1_isoform_length_2500_21657 | XP_018016414.1  splicing factor 3B subunit 3-like | F:GO:0003676; C:GO:0005634 | |

| | | | |
|---|---|---|---|
| 2772\|_\|c13472_f2p0_2500_isoform_c13472_full_length_coverage_2_non_full_length_coverage_0_isoform_length_2500_17597 | ---NA--- | | |
| TRINITY_DN8379_c0_g1_i1_len_2583_229639.p1_type_5prime_partial_len_833 | XP_022817368.1neurochondrin homolog | | |
| 2774\|_\|c2133_f5p3_2499_isoform_c2133_full_length_coverage_5_non_full_length_coverage_3_isoform_length_2499_11686 | ---NA--- | | |
| 2775\|_\|c18038_f2p0_2499_isoform_c18038_full_length_coverage_2_non_full_length_coverage_0_isoform_length_2499_13527 | ---NA--- | | |
| 2776\|_\|c8366_f2p0_2499_isoform_c8366_full_length_coverage_2_non_full_length_coverage_0_isoform_length_2499_21769 | XP_018026669.1 calcium/calmodulin-dependent protein kinase kinase 2-like | | |
| TRINITY_DN33829_c0_g1_i1_len_354_210467.p1_type_5prime_partial_len_109 | ---NA--- | | |
| TRINITY_DN33705_c0_g1_i1_len_329_212058.p1_type_internal_len_110 | XP_018014164.1 bestrophin-3-like | F:GO:0005254; C:GO:0005886; C:GO:0034707; P:GO:1902476 | |
| TRINITY_DN17537_c0_g1_i2_len_795_162467.p2_type_complete_len_109 | ---NA--- | | |
| TRINITY_DN15226_c0_g1_i1_len_349_106672.p1_type_5prime_partial_len_109 | ---NA--- | | |
| TRINITY_DN21495_c0_g1_i1_len_2164_155675.p2_type_complete_len_109 | ---NA--- | | |
| TRINITY_DN22444_c0_g1_i6_len_1200_2637.p2_type_5prime_partial_len_109 | ---NA--- | | |
| TRINITY_DN22995_c0_g1_i3_len_329_212060.p1_type_internal_len_110 | ---NA--- | | |
| TRINITY_DN38268_c0_g1_i1_len_330_212006.p2_type_internal_len_110 | ---NA--- | | |
| TRINITY_DN2884_c0_g1_i1_len_563_202585.p1_type_5prime_partial_len_109 | ---NA--- | | |

| | | | |
|---|---|---|---|
| TRINITY_DN19937_c0_g1_i2_len_450_168426.p1_type_5prime_partial_len_109 | ---NA--- | | |
| TRINITY_DN25493_c0_g1_i1_len_327_139058.p1_type_internal_len_110 | ---NA--- | | |
| TRINITY_DN34389_c0_g1_i1_len_328_212156.p1_type_internal_len_110 | ---NA--- | | |
| TRINITY_DN25409_c0_g1_i1_len_327_139054.p1_type_internal_len_110 | ---NA--- | | |
| TRINITY_DN8959_c1_g1_i1_len_328_212177.p1_type_internal_len_110 | XP_026038551.1uncharacterized protein K02A2.6-like | | |
| TRINITY_DN7233_c0_g1_i1_len_328_212174.p1_type_internal_len_110 | ---NA--- | | |
| TRINITY_DN18855_c0_g1_i1_len_749_199288.p1_type_5prime_partial_len_109 | ---NA--- | | |
| TRINITY_DN28828_c0_g1_i1_len_5224_187079.p4_type_complete_len_109 | ---NA--- | | |
| TRINITY_DN17543_c0_g1_i1_len_594_201887.p1_type_5prime_partial_len_109 | KZS07296.1Beta,beta-carotene 15,15'-dioxygenase | F:GO:0003834; F:GO:0004744; F:GO:0010436; P:GO:0016121; P:GO:0042574 | EC:1.13.11; EC:1.14.99.36 |
| TRINITY_DN4438_c0_g1_i1_len_328_212163.p1_type_internal_len_110 | XP_018009598.1 mitoguardin-like | | |
| TRINITY_DN8221_c0_g1_i1_len_328_212158.p1_type_internal_len_110 | ---NA--- | | |
| TRINITY_DN72_c0_g1_i1_len_329_138958.p2_type_internal_len_110 | AEX08659.1lipopolysaccharide and beta-1,3-glucan binding protein | F:GO:0001872; F:GO:0004553; C:GO:0005576; P:GO:0005975; P:GO:0045087 | |
| TRINITY_DN28631_c0_g1_i1_len_329_138955.p2_type_internal_len_110 | XP_018019321.1 protein tipE-like | | |
| TRINITY_DN12815_c0_g1_i1_len_454_168314.p1_type_3prime_partial_len_110 | ---NA--- | | |
| TRINITY_DN4233_c0_g1_i2_len_329_138943.p1_type_internal_len_110 | ---NA--- | | |

| | | | |
|---|---|---|---|
| TRINITY_DN4627_c0_g1_i1_len_329_138918.p1_type_internal_len_110 | ---NA--- | | |
| TRINITY_DN32567_c0_g1_i1_len_328_139008.p1_type_internal_len_110 | XP_023211963.1sorting nexin-17-like | C:GO:0005623; P:GO:0006886; F:GO:0035091 | |
| TRINITY_DN31276_c0_g1_i1_len_328_138998.p1_type_internal_len_110 | ---NA--- | | |
| TRINITY_DN17156_c0_g1_i1_len_328_138986.p1_type_internal_len_110 | ---NA--- | | |
| TRINITY_DN25359_c0_g1_i1_len_328_138982.p1_type_internal_len_110 | ---NA--- | | |
| TRINITY_DN18586_c0_g1_i1_len_534_242161.p1_type_5prime_partial_len_109 | ---NA--- | | |
| TRINITY_DN34407_c0_g1_i1_len_328_173647.p1_type_internal_len_110 | ---NA--- | | |
| TRINITY_DN15133_c0_g1_i1_len_328_212198.p1_type_internal_len_110 | ---NA--- | | |
| TRINITY_DN16080_c0_g1_i2_len_328_173655.p1_type_internal_len_110 | ---NA--- | | |
| TRINITY_DN15302_c0_g1_i1_len_1325_158616.p3_type_complete_len_109 | ---NA--- | | |
| TRINITY_DN3367_c0_g1_i1_len_328_212197.p1_type_internal_len_110 | ---NA--- | | |
| TRINITY_DN13415_c0_g1_i1_len_1249_297612.p2_type_5prime_partial_len_109 | ---NA--- | | |
| TRINITY_DN5651_c0_g1_i1_len_328_173680.p1_type_internal_len_110 | ---NA--- | | |
| TRINITY_DN26317_c0_g1_i1_len_328_173681.p1_type_internal_len_110 | XP_018017271.1 carboxypeptidase B-like | F:GO:0004180 | |
| TRINITY_DN21565_c0_g1_i1_len_329_173615.p1_type_internal_len_110 | ---NA--- | | |
| TRINITY_DN28878_c0_g1_i1_len_329_173628.p1_type_internal_len_110 | APF30031.1ionotropic glutamate receptor NMDA 1A | F:GO:0004970; C:GO:0016021; C:GO:0030054; P:GO:0034220; P:GO:0035235; C:GO:0045211 | |

| | | | |
|---|---|---|---|
| TRINITY_DN35945_c0_g1_i1_len_339_211456.p1_type_5prime_partial_len_109 | ---NA--- | | |
| TRINITY_DN28033_c0_g1_i1_len_5593_186994.p2_type_complete_len_109 | ---NA--- | | |
| TRINITY_DN30973_c0_g1_i1_len_328_173640.p1_type_internal_len_110 | ---NA--- | | |
| TRINITY_DN1591_c0_g1_i2_len_327_173748.p1_type_internal_len_110 | ---NA--- | | |
| TRINITY_DN33802_c0_g1_i1_len_328_212187.p2_type_internal_len_110 | XP_018021087.1PREDICTED: uncharacterized protein LOC108677391 | | |
| TRINITY_DN14066_c0_g1_i1_len_443_40121.p1_type_complete_len_109 | ---NA--- | | |
| TRINITY_DN10482_c0_g1_i1_len_447_206072.p1_type_complete_len_109 | ---NA--- | | |
| TRINITY_DN22099_c0_g1_i2_len_1585_93089.p3_type_5prime_partial_len_109 | ---NA--- | | |
| TRINITY_DN15949_c0_g1_i2_len_442_168679.p1_type_5prime_partial_len_109 | ---NA--- | | |
| TRINITY_DN25760_c0_g1_i1_len_328_173690.p1_type_internal_len_110 | XP_018011507.1 SLIT-ROBO Rho GTPase-activating protein 1-like | P:GO:0065007 | |
| TRINITY_DN7494_c0_g1_i1_len_327_173711.p1_type_internal_len_110 | ---NA--- | | |
| TRINITY_DN9363_c0_g1_i1_len_393_41717.p2_type_5prime_partial_len_109 | ---NA--- | | |
| TRINITY_DN10806_c0_g1_i1_len_593_201912.p1_type_5prime_partial_len_109 | ---NA--- | | |
| TRINITY_DN22168_c0_g2_i1_len_328_212190.p2_type_internal_len_110 | ---NA--- | | |
| TRINITY_DN32095_c0_g1_i1_len_327_173733.p1_type_internal_len_110 | ---NA--- | | |
| TRINITY_DN20821_c0_g1_i2_len_767_5116.p2_type_5prime_partial_len_109 | ---NA--- | | |

| | | | |
|---|---|---|---|
| TRINITY_DN36274_c0_g1_i1_len_329_212105.p1_type_internal_len_110 | XP_018019976.1 kynureninase-like | C:GO:0005737; P:GO:0006569; P:GO:0019805; F:GO:0030170; F:GO:0030429; P:GO:0034354; P:GO:0043420; P:GO:0097053 | EC:3.7.1.3 |
| TRINITY_DN39657_c0_g1_i1_len_329_212104.p1_type_internal_len_110 | ---NA--- | | |
| TRINITY_DN37847_c0_g1_i1_len_329_212101.p1_type_internal_len_110 | XP_018026744.1 myosin-IIIb-like | | |
| TRINITY_DN19558_c0_g1_i2_len_1126_32874.p2_type_5prime_partial_len_109 | ---NA--- | | |
| TRINITY_DN24064_c0_g1_i2_len_4331_57780.p3_type_complete_len_109 | XP_026687606.1protein AF-9-like | C:GO:0005634; P:GO:0006355 | |
| TRINITY_DN5494_c0_g1_i1_len_329_212095.p1_type_internal_len_110 | KFM80028.1Protein jagged-1 | F:GO:0005488 | |
| TRINITY_DN20559_c0_g1_i1_len_2468_265497.p2_type_complete_len_109 | ---NA--- | | |
| TRINITY_DN5879_c0_g1_i1_len_327_282340.p1_type_internal_len_110 | AAW33781.1testis beta tubulin | F:GO:0003924; F:GO:0005200; F:GO:0005525; C:GO:0005874; P:GO:0007010; P:GO:0007017 | EC:3.6.1.15 |
| TRINITY_DN12427_c0_g1_i1_len_329_212092.p1_type_internal_len_110 | ---NA--- | | |
| TRINITY_DN29241_c0_g1_i1_len_1591_192604.p1_type_5prime_partial_len_109 | ---NA--- | | |
| TRINITY_DN18477_c0_g1_i1_len_506_204050.p1_type_complete_len_109 | AGC54636.1heat shock protein 90 | F:GO:0005524; P:GO:0006457; F:GO:0051082 | |
| TRINITY_DN4867_c0_g1_i1_len_327_15140.p1_type_internal_len_110 | ---NA--- | | |
| TRINITY_DN35863_c0_g1_i1_len_328_212146.p3_type_internal_len_110 | XP_009704006.1 cytosolic carboxypeptidase 1-like | | |
| TRINITY_DN41260_c0_g1_i1_len_328_212141.p2_type_internal_len_110 | ---NA--- | | |
| TRINITY_DN27965_c0_g1_i2_len_1614_192486.p2_type_complete_len_109 | ---NA--- | | |

| | | | |
|---|---|---|---|
| TRINITY_DN31463_c0_g1_i1_len_905_197441.p1_type_complete_len_109 | ---NA--- | | |
| TRINITY_DN23248_c0_g1_i2_len_1907_231392.p2_type_complete_len_109 | ---NA--- | | |
| TRINITY_DN1364_c0_g1_i1_len_328_212139.p1_type_internal_len_110 | XP_015516829.1 regulatory-associated protein of mTOR isoform X5 | | |
| TRINITY_DN28590_c0_g1_i1_len_2060_190686.p3_type_complete_len_109 | ---NA--- | | |
| TRINITY_DN12560_c0_g1_i1_len_468_167921.p3_type_5prime_partial_len_109 | ---NA--- | | |
| TRINITY_DN40460_c0_g1_i1_len_328_212147.p1_type_internal_len_110 | ---NA--- | | |
| TRINITY_DN30036_c0_g1_i1_len_330_138847.p1_type_internal_len_110 | ---NA--- | | |
| TRINITY_DN11506_c0_g1_i1_len_327_15143.p1_type_internal_len_110 | ---NA--- | | |
| TRINITY_DN38889_c0_g1_i1_len_329_212121.p1_type_internal_len_110 | ---NA--- | | |
| TRINITY_DN11368_c0_g1_i1_len_352_210642.p1_type_5prime_partial_len_109 | ---NA--- | | |
| TRINITY_DN11437_c0_g1_i1_len_329_212118.p2_type_internal_len_110 | ---NA--- | | |
| TRINITY_DN40459_c0_g1_i1_len_329_212114.p1_type_internal_len_110 | XP_018015390.1 laminin subunit gamma-1-like isoform X2 | | |
| TRINITY_DN34401_c0_g1_i1_len_403_207866.p1_type_5prime_partial_len_109 | XP_018023185.1 longitudinals lacking protein, isoforms H/M/V-like isoform X9 | | |
| TRINITY_DN36812_c0_g1_i1_len_328_212137.p1_type_internal_len_110 | ---NA--- | | |
| TRINITY_DN32725_c0_g1_i1_len_336_138516.p1_type_5prime_partial_len_109 | XP_017491708.1 G protein-coupled receptor kinase 2-like isoform X2 | F:GO:0004703; F:GO:0005524; P:GO:0006468; P:GO:0007165 | EC:2.7.11; EC:2.7.11.16 |
| TRINITY_DN25092_c0_g1_i1_len_327_15165.p2_type_internal_len_110 | XP_018028145.1 fibrillin-1-like isoform X3 | F:GO:0005509 | |

<table>
<tr><td>TRINITY_DN22817_c0_g1_i1_len_1841_191510.p2_type_complete_len_109</td><td>---NA---</td><td></td><td></td></tr>
<tr><td>TRINITY_DN6914_c0_g1_i1_len_382_73129.p1_type_5prime_partial_len_109</td><td>XP_023162638.1tubulin--tyrosine ligase-like protein 12</td><td>F:GO:0005524; P:GO:0006464</td><td></td></tr>
<tr><td>TRINITY_DN21481_c0_g1_i1_len_2403_295256.p3_type_5prime_partial_len_109</td><td>---NA---</td><td></td><td></td></tr>
<tr><td>TRINITY_DN35547_c0_g1_i1_len_327_212258.p1_type_internal_len_110</td><td>---NA---</td><td></td><td></td></tr>
<tr><td>TRINITY_DN31372_c0_g1_i1_len_327_212256.p1_type_internal_len_110</td><td>XP_018010218.1PREDICTED: uncharacterized protein LOC108667681</td><td></td><td></td></tr>
<tr><td>TRINITY_DN26202_c2_g1_i1_len_1136_235138.p1_type_5prime_partial_len_109</td><td>---NA---</td><td></td><td></td></tr>
<tr><td>TRINITY_DN19562_c0_g2_i1_len_329_44598.p1_type_internal_len_110</td><td>---NA---</td><td></td><td></td></tr>
<tr><td>TRINITY_DN28296_c0_g1_i1_len_388_72868.p1_type_5prime_partial_len_109</td><td>---NA---</td><td></td><td></td></tr>
<tr><td>TRINITY_DN23210_c0_g1_i1_len_685_200231.p1_type_5prime_partial_len_109</td><td>---NA---</td><td></td><td></td></tr>
<tr><td>TRINITY_DN38313_c0_g1_i1_len_327_212278.p1_type_internal_len_110</td><td>---NA---</td><td></td><td></td></tr>
<tr><td>TRINITY_DN35420_c0_g1_i1_len_327_212279.p1_type_internal_len_110</td><td>---NA---</td><td></td><td></td></tr>
<tr><td>TRINITY_DN33468_c0_g1_i1_len_327_212271.p2_type_internal_len_110</td><td>XP_018008763.1 zinc finger protein castor homolog 1-like isoform X5</td><td></td><td></td></tr>
<tr><td>TRINITY_DN15844_c0_g1_i1_len_368_73793.p1_type_5prime_partial_len_109</td><td>---NA---</td><td></td><td></td></tr>
<tr><td>2778|_|c9588_f2p1_2499_isoform_c9588_full_length_coverage_2_non_full_length_coverage_1_isoform_length_2499_21881</td><td>XP_018024055.1 dynein heavy chain, cytoplasmic-like</td><td></td><td></td></tr>
<tr><td>2779|_|c13647_f4p2_2499_isoform_c13647_full_length_coverage_4_non_full_length_coverage_2_isoform_length_2499_17615</td><td>KDR18536.1McKusick-Kaufman/Bardet-Biedl syndromes putative chaperonin</td><td>F:GO:0005524; P:GO:0006457; F:GO:0051082; P:GO:0060271</td><td></td></tr>
</table>

| | | | |
|---|---|---|---|
| TRINITY_DN25621_c0_g1_i3_len_2508_189434.p1_type_5prime_partial_len_833 | XP_018911907.1 zinc finger protein 879-like | | |
| TRINITY_DN29138_c0_g1_i1_len_2629_189173.p1_type_5prime_partial_len_833 | XP_024228480.11-phosphatidylinositol 4,5-bisphosphate phosphodiesterase gamma-1 isoform X2 | P:GO:0006629; P:GO:0009987; F:GO:0016787 | |
| TRINITY_DN21567_c0_g1_i1_len_3949_153705.p1_type_5prime_partial_len_833 | XP_023222507.1cap-specific mRNA (nucleoside-2'-O-)-methyltransferase 1-like | F:GO:0004483; C:GO:0005634; C:GO:0005737; P:GO:0006370; P:GO:0097309 | EC:2.1.1.57 |
| 2783\|_\|c18039_f2p1_2498_isoform_c18039_full_length_coverage_2_non_full_length_coverage_1_isoform_length_2498_22649 | ---NA--- | | |
| 2784\|_\|c2102_f4p8_2498_isoform_c2102_full_length_coverage_4_non_full_length_coverage_8_isoform_length_2498_11660 | XP_019638088.1 O-acetyl-ADP-ribose deacetylase 1-like | | |
| 2785\|_\|c10052_f3p1_2498_isoform_c10052_full_length_coverage_3_non_full_length_coverage_1_isoform_length_2498_17325 | KDR07796.1Lysophospholipid acyltransferase 5 | C:GO:0016020; F:GO:0016740 | |
| 2786\|_\|c22292_f2p1_2498_isoform_c22292_full_length_coverage_2_non_full_length_coverage_1_isoform_length_2498_22864 | ---NA--- | | |
| TRINITY_DN19232_c0_g1_i3_len_2566_265382.p1_type_3prime_partial_len_833 | XP_023706301.1serine/arginine repetitive matrix protein 1 isoform X2 | P:GO:0006397 | |
| TRINITY_DN25271_c0_g1_i1_len_331_14957.p1_type_internal_len_110 | ---NA--- | | |
| TRINITY_DN18810_c0_g1_i1_len_1196_297821.p1_type_5prime_partial_len_109 | ---NA--- | | |
| TRINITY_DN13058_c0_g1_i1_len_327_212274.p1_type_internal_len_110 | ASW35086.1fructose-1,6-bisphosphatase 1-like protein | P:GO:0005975; P:GO:0016311; F:GO:0042132 | EC:3.1.3.23; EC:3.1.3.11 |
| TRINITY_DN39506_c0_g1_i1_len_700_199994.p1_type_5prime_partial_len_109 | ---NA--- | | |
| TRINITY_DN28294_c0_g1_i2_len_1699_192091.p4_type_5prime_partial_len_109 | ---NA--- | | |

| | | | |
|---|---|---|---|
| TRINITY_DN14094_c0_g1_i1_len_327_212277.p1_type_internal_len_110 | ---NA--- | | |
| TRINITY_DN23771_c0_g1_i1_len_1714_192022.p2_type_5prime_partial_len_109 | ---NA--- | | |
| TRINITY_DN18743_c0_g1_i1_len_1210_297769.p2_type_5prime_partial_len_109 | ---NA--- | | |
| TRINITY_DN21099_c0_g1_i1_len_330_44536.p1_type_internal_len_110 | ABE03741.1prophenoloxidase activating factor | F:GO:0004252; P:GO:0006508 | EC:3.4.21 |
| TRINITY_DN20373_c0_g1_i2_len_426_206887.p1_type_3prime_partial_len_110 | ---NA--- | | |
| TRINITY_DN14033_c1_g1_i1_len_369_209564.p1_type_5prime_partial_len_109 | ---NA--- | | |
| TRINITY_DN22108_c0_g1_i1_len_758_199151.p1_type_5prime_partial_len_109 | ---NA--- | | |
| TRINITY_DN5378_c0_g1_i1_len_366_171685.p1_type_5prime_partial_len_109 | ---NA--- | | |
| TRINITY_DN27163_c0_g1_i1_len_826_198299.p2_type_5prime_partial_len_109 | ---NA--- | | |
| TRINITY_DN38925_c0_g1_i1_len_327_212223.p1_type_internal_len_110 | ---NA--- | | |
| TRINITY_DN24967_c0_g1_i1_len_328_44668.p1_type_internal_len_110 | ---NA--- | | |
| TRINITY_DN30887_c0_g1_i1_len_434_71087.p1_type_3prime_partial_len_110 | ---NA--- | | |
| TRINITY_DN14481_c0_g1_i3_len_411_169782.p1_type_5prime_partial_len_109 | ---NA--- | | |
| TRINITY_DN1110_c0_g1_i2_len_328_44672.p1_type_internal_len_110 | ---NA--- | | |
| TRINITY_DN12899_c0_g1_i1_len_330_173560.p1_type_internal_len_110 | ---NA--- | | |
| TRINITY_DN18973_c0_g1_i1_len_328_212202.p1_type_internal_len_110 | ---NA--- | | |

| | | | |
|---|---|---|---|
| TRINITY_DN28834_c0_g1_i1_len_330_173587.p1_type_3prime_partial_len_110 | OWF43792.1Phosphoglucomutase-2 | F:GO:0000287; P:GO:0005975; F:GO:0016868 | |
| TRINITY_DN36917_c0_g1_i1_len_328_212199.p1_type_internal_len_110 | XP_018020234.1 E3 ubiquitin-protein ligase MYCBP2-like | | |
| TRINITY_DN4597_c0_g1_i1_len_327_212208.p2_type_internal_len_110 | ---NA--- | | |
| TRINITY_DN14251_c0_g1_i1_len_330_15013.p1_type_internal_len_110 | ---NA--- | | |
| TRINITY_DN31908_c0_g1_i1_len_423_169338.p1_type_5prime_partial_len_109 | ---NA--- | | |
| TRINITY_DN10720_c0_g1_i2_len_330_173541.p1_type_internal_len_110 | ---NA--- | | |
| TRINITY_DN14276_c0_g1_i1_len_330_173559.p1_type_internal_len_110 | ---NA--- | | |
| TRINITY_DN36161_c0_g1_i1_len_327_212231.p1_type_internal_len_110 | ---NA--- | | |
| TRINITY_DN13702_c0_g1_i1_len_471_205160.p1_type_5prime_partial_len_109 | EDO46894.1predicted protein | C:GO:0005774; F:GO:0015184; P:GO:0015811 | |
| TRINITY_DN17111_c0_g1_i1_len_950_270543.p4_type_5prime_partial_len_109 | ---NA--- | | |
| TRINITY_DN18986_c0_g1_i3_len_5449_264355.p2_type_complete_len_109 | ---NA--- | | |
| TRINITY_DN10581_c0_g1_i2_len_544_203049.p1_type_5prime_partial_len_109 | ---NA--- | | |
| TRINITY_DN25014_c0_g1_i1_len_328_44654.p1_type_internal_len_110 | ---NA--- | | |
| TRINITY_DN36182_c0_g1_i1_len_408_207681.p1_type_5prime_partial_len_109 | ---NA--- | | |
| TRINITY_DN21802_c0_g1_i1_len_328_44659.p1_type_internal_len_110 | XP_018027756.1PREDICTED: uncharacterized protein C630.12-like | | |
| TRINITY_DN5268_c0_g1_i1_len_328_44666.p1_type_internal_len_110 | XP_022902452.1disintegrin and metalloproteinase domain-containing protein 12 isoform X1 | | |

| | | | |
|---|---|---|---|
| TRINITY_DN16708_c0_g1_i1_len_1487_93380.p4_type_5prime_partial_len_109 | ALM25771.1cyclin T | P:GO:0006355; F:GO:0019901 | |
| TRINITY_DN25637_c0_g1_i1_len_330_14994.p1_type_internal_len_110 | ---NA--- | | |
| TRINITY_DN41403_c0_g1_i1_len_327_212227.p1_type_internal_len_110 | XP_023209388.1transmembrane protein 151B-like | | |
| TRINITY_DN6386_c0_g1_i1_len_580_241169.p1_type_5prime_partial_len_109 | ---NA--- | | |
| TRINITY_DN33744_c0_g1_i1_len_327_212226.p1_type_internal_len_110 | ---NA--- | | |
| TRINITY_DN33124_c1_g1_i1_len_1650_192309.p4_type_5prime_partial_len_109 | ---NA--- | | |
| TRINITY_DN20892_c0_g1_i1_len_328_44651.p1_type_internal_len_110 | ---NA--- | | |
| TRINITY_DN29748_c0_g1_i1_len_329_250037.p2_type_internal_len_110 | ---NA--- | | |
| TRINITY_DN11577_c0_g1_i1_len_329_250035.p1_type_internal_len_110 | ---NA--- | | |
| TRINITY_DN29793_c0_g1_i1_len_329_250034.p2_type_internal_len_110 | ---NA--- | | |
| TRINITY_DN9246_c0_g1_i1_len_329_250033.p1_type_internal_len_110 | ---NA--- | | |
| TRINITY_DN26560_c0_g1_i1_len_329_250026.p1_type_internal_len_110 | XP_018024941.1 endothelin-converting enzyme 1-like | | |
| TRINITY_DN7413_c0_g1_i1_len_329_250022.p4_type_internal_len_110 | ---NA--- | | |
| TRINITY_DN13739_c0_g1_i1_len_533_100591.p1_type_complete_len_109 | ---NA--- | | |
| TRINITY_DN12902_c0_g1_i1_len_329_250018.p1_type_internal_len_110 | ---NA--- | | |
| TRINITY_DN13950_c0_g1_i2_len_329_250013.p1_type_internal_len_110 | ---NA--- | | |

| | | | |
|---|---|---|---|
| TRINITY_DN31509_c0_g1_i1_len_329_250065.p2_type_internal_len_110 | ---NA--- | | |
| TRINITY_DN2414_c0_g1_i1_len_329_212083.p1_type_internal_len_110 | ---NA--- | | |
| TRINITY_DN33654_c0_g1_i1_len_329_250054.p3_type_internal_len_110 | ---NA--- | | |
| TRINITY_DN29406_c0_g1_i1_len_329_250051.p1_type_internal_len_110 | XP_018011346.1 diacylglycerol kinase delta-like | F:GO:0003951; F:GO:0004143; F:GO:0005524; P:GO:0007205; P:GO:0016310; P:GO:0035556 | EC:2.7.1.23; EC:2.7.1.107 |
| TRINITY_DN32561_c0_g1_i1_len_329_250041.p1_type_internal_len_110 | ---NA--- | | |
| TRINITY_DN35843_c0_g1_i1_len_331_249956.p1_type_internal_len_110 | ---NA--- | | |
| TRINITY_DN29408_c0_g1_i1_len_329_15074.p1_type_internal_len_110 | XP_018026907.1 beta-galactoside alpha-2,6-sialyltransferase 1-like | C:GO:0016020; F:GO:0016757; P:GO:0043412 | |
| TRINITY_DN32605_c0_g1_i1_len_331_249924.p1_type_internal_len_110 | ---NA--- | | |
| TRINITY_DN15426_c0_g1_i1_len_331_211945.p1_type_internal_len_110 | ---NA--- | | |
| TRINITY_DN28921_c0_g1_i1_len_2431_189613.p2_type_5prime_partial_len_109 | ---NA--- | | |
| TRINITY_DN12767_c0_g1_i1_len_379_136278.p1_type_5prime_partial_len_109 | ---NA--- | | |
| TRINITY_DN32001_c0_g1_i1_len_346_14101.p1_type_5prime_partial_len_109 | ---NA--- | | |
| TRINITY_DN34454_c0_g1_i1_len_330_249983.p1_type_internal_len_110 | ---NA--- | | |
| TRINITY_DN19893_c0_g1_i1_len_1443_267963.p2_type_5prime_partial_len_109 | ---NA--- | | |
| TRINITY_DN35635_c0_g1_i1_len_330_249977.p1_type_internal_len_110 | ---NA--- | | |
| TRINITY_DN34389_c0_g1_i1_len_330_249969.p1_type_internal_len_110 | ---NA--- | | |

| | | | |
|---|---|---|---|
| TRINITY_DN3735_c0_g1_i1_len_330_249968.p1_type_internal_len_110 | XP_018013901.1 leucine-rich repeats and immunoglobulin-like domains protein 2 | | |
| TRINITY_DN26528_c0_g1_i3_len_2279_190013.p2_type_3prime_partial_len_110 | ---NA--- | | |
| TRINITY_DN2878_c0_g1_i1_len_328_250144.p1_type_internal_len_110 | ---NA--- | | |
| TRINITY_DN2778_c0_g1_i1_len_328_250132.p1_type_internal_len_110 | ---NA--- | | |
| TRINITY_DN7336_c0_g1_i1_len_328_250128.p1_type_internal_len_110 | ---NA--- | | |
| TRINITY_DN12327_c0_g1_i1_len_328_250127.p2_type_internal_len_110 | ---NA--- | | |
| TRINITY_DN4589_c0_g1_i1_len_328_250111.p1_type_internal_len_110 | ---NA--- | | |
| TRINITY_DN36304_c0_g1_i1_len_331_211928.p1_type_internal_len_110 | ---NA--- | | |
| TRINITY_DN30946_c1_g2_i2_len_2263_190063.p5_type_complete_len_109 | ---NA--- | | |
| TRINITY_DN16943_c0_g1_i1_len_413_207449.p1_type_complete_len_109 | ---NA--- | | |
| TRINITY_DN33106_c0_g1_i1_len_327_250169.p1_type_internal_len_110 | AYD41593.1transforming growth factor-beta-induced protein ig-h3-like protein | | |
| TRINITY_DN29949_c0_g1_i1_len_329_250081.p2_type_internal_len_110 | ---NA--- | | |
| TRINITY_DN32836_c0_g1_i1_len_331_211934.p1_type_internal_len_110 | ---NA--- | | |
| TRINITY_DN38131_c0_g1_i1_len_329_250069.p1_type_internal_len_110 | ATN38697.1cuticle-like protein | F:GO:0042302 | |
| TRINITY_DN6089_c0_g1_i1_len_328_250100.p1_type_internal_len_110 | XP_018022184.1 cytochrome P450 6k1-like | F:GO:0016491; F:GO:0046872 | |
| TRINITY_DN27327_c0_g1_i1_len_328_250090.p1_type_internal_len_110 | ---NA--- | | |

| | | | |
|---|---|---|---|
| TRINITY_DN33361_c0_g1_i1_len_328_250089.p4_type_internal_len_110 | ---NA--- | | |
| TRINITY_DN21603_c0_g1_i1_len_331_75842.p1_type_internal_len_110 | ---NA--- | | |
| TRINITY_DN25836_c0_g1_i1_len_461_305115.p1_type_5prime_partial_len_109 | ---NA--- | | |
| TRINITY_DN26118_c0_g1_i1_len_331_75843.p1_type_internal_len_110 | XP_018014662.1 teneurin-m-like | | |
| TRINITY_DN2546_c0_g1_i1_len_331_75858.p1_type_internal_len_110 | ---NA--- | | |
| TRINITY_DN1250_c0_g1_i1_len_328_15091.p1_type_internal_len_110 | XP_018008324.1 cadherin-23-like | | |
| TRINITY_DN39620_c0_g1_i1_len_329_212068.p1_type_internal_len_110 | XP_018012974.1PREDICTED: uncharacterized protein LOC108670035 | | |
| TRINITY_DN17857_c0_g1_i2_len_1083_33068.p3_type_5prime_partial_len_109 | ---NA--- | | |
| TRINITY_DN24136_c0_g1_i1_len_354_13693.p1_type_5prime_partial_len_109 | ---NA--- | | |
| TRINITY_DN26597_c0_g1_i1_len_330_75953.p1_type_internal_len_110 | ---NA--- | | |
| TRINITY_DN25071_c0_g1_i2_len_329_75954.p1_type_internal_len_110 | ---NA--- | | |
| TRINITY_DN25551_c0_g1_i1_len_329_75983.p1_type_internal_len_110 | APG53780.1transient receptor potential cation channel subfamily M member 1 | F:GO:0005216; C:GO:0016021; P:GO:0034220; P:GO:0051262 | |
| TRINITY_DN11108_c0_g1_i1_len_1320_194135.p3_type_3prime_partial_len_110 | ---NA--- | | |
| TRINITY_DN21603_c0_g1_i2_len_1815_231688.p2_type_complete_len_109 | ---NA--- | | |
| TRINITY_DN17434_c0_g1_i1_len_636_164443.p1_type_5prime_partial_len_109 | ---NA--- | | |
| 2790\|_\|c1435_f3p6_2496_isoform_c1435_full_length_coverage_3_non_full_length_coverage_6_isoform_length_2496_27778 | ---NA--- | | |

<table>
<tr><td></td><td></td><td></td><td></td></tr>
<tr><td>TRINITY_DN25591_c0_g1_i1_len_5131_227759.p1_type_complete_len_832</td><td>XP_018008100.1 breast cancer anti-estrogen resistance protein 1-like isoform X3</td><td></td><td></td></tr>
<tr><td>2792|_|c18294_f2p4_2496_isoform_c18294_full_length_coverage_2_non_full_length_coverage_4_isoform_length_2496_18087</td><td>---NA---</td><td></td><td></td></tr>
<tr><td>2794|_|c5775_f2p13_2496_isoform_c5775_full_length_coverage_2_non_full_length_coverage_13_isoform_length_2496_25775</td><td>XP_013411826.1eukaryotic translation initiation factor 2-alpha kinase 1</td><td>F:GO:0003743; F:GO:0004672; F:GO:0005524; P:GO:0006413; P:GO:0006468; F:GO:0016301; P:GO:0016310</td><td></td></tr>
<tr><td>2795|_|c2655_f9p5_2496_isoform_c2655_full_length_coverage_9_non_full_length_coverage_5_isoform_length_2496_12045</td><td>KDR08563.1Anaphase-promoting complex subunit 7</td><td></td><td></td></tr>
<tr><td>2796|_|c2661_f2p1_2495_isoform_c2661_full_length_coverage_2_non_full_length_coverage_1_isoform_length_2495_16595</td><td>---NA---</td><td></td><td></td></tr>
<tr><td>2797|_|c5012_f2p0_2495_isoform_c5012_full_length_coverage_2_non_full_length_coverage_0_isoform_length_2495_16915</td><td>---NA---</td><td></td><td></td></tr>
<tr><td>2799|_|c6558_f2p11_2495_isoform_c6558_full_length_coverage_2_non_full_length_coverage_11_isoform_length_2495_21614</td><td>PNF39008.1Muskelin</td><td></td><td></td></tr>
<tr><td>2800|_|c1758_f5p4_2494_isoform_c1758_full_length_coverage_5_non_full_length_coverage_4_isoform_length_2494_15981</td><td>XP_022911981.1protein bric-a-brac 2-like</td><td></td><td></td></tr>
<tr><td>2801|_|c2608_f4p9_2494_isoform_c2608_full_length_coverage_4_non_full_length_coverage_9_isoform_length_2494_12009</td><td>---NA---</td><td></td><td></td></tr>
<tr><td>TRINITY_DN1199_c0_g1_i1_len_330_75900.p1_type_internal_len_110</td><td>---NA---</td><td></td><td></td></tr>
<tr><td>TRINITY_DN21644_c0_g1_i1_len_785_198796.p1_type_5prime_partial_len_109</td><td>---NA---</td><td></td><td></td></tr>
</table>

| | | | |
|---|---|---|---|
| TRINITY_DN15256_c0_g1_i1_len_414_207420.p1_type_5prime_partial_len_109 | ---NA--- | | |
| TRINITY_DN10222_c0_g1_i1_len_328_15076.p1_type_internal_len_110 | ---NA--- | | |
| TRINITY_DN11624_c0_g1_i1_len_477_204983.p1_type_5prime_partial_len_109 | ---NA--- | | |
| TRINITY_DN14956_c0_g1_i2_len_928_236709.p1_type_5prime_partial_len_109 | ---NA--- | | |
| TRINITY_DN32904_c0_g1_i1_len_504_101194.p2_type_5prime_partial_len_109 | ---NA--- | | |
| TRINITY_DN30207_c0_g1_i2_len_2432_189607.p3_type_5prime_partial_len_109 | ---NA--- | | |
| TRINITY_DN21653_c0_g1_i1_len_3529_228447.p2_type_5prime_partial_len_109 | ---NA--- | | |
| TRINITY_DN17249_c0_g1_i1_len_329_212075.p1_type_internal_len_110 | ---NA--- | | |
| TRINITY_DN22184_c0_g1_i2_len_473_69883.p1_type_3prime_partial_len_110 | ---NA--- | | |
| TRINITY_DN17921_c0_g1_i1_len_331_310203.p1_type_internal_len_110 | ---NA--- | | |
| TRINITY_DN25849_c0_g1_i1_len_329_282243.p3_type_5prime_partial_len_109 | ---NA--- | | |
| TRINITY_DN27798_c0_g1_i1_len_329_282242.p1_type_internal_len_110 | XP_018007454.1 gastrula zinc finger protein XlCGF49.1-like | | |
| TRINITY_DN18328_c0_g1_i1_len_713_5597.p1_type_5prime_partial_len_109 | ---NA--- | | |
| TRINITY_DN9691_c0_g1_i1_len_329_212084.p1_type_internal_len_110 | ---NA--- | | |
| TRINITY_DN34980_c0_g1_i1_len_340_211344.p1_type_5prime_partial_len_109 | XP_018010673.1 delta(14)-sterol reductase-like | | |
| TRINITY_DN31265_c0_g1_i1_len_350_137698.p1_type_5prime_partial_len_109 | ---NA--- | | |

| | | | |
|---|---|---|---|
| TRINITY_DN5316_c0_g1_i1_len_329_282229.p2_type_internal_len_110 | ---NA--- | | |
| TRINITY_DN29981_c0_g1_i5_len_2208_190216.p3_type_5prime_partial_len_109 | AYD41579.1laccase-10-like protein | F:GO:0005507; F:GO:0052716; P:GO:0055114 | EC:1.10.3; EC:1.10.3.2 |
| TRINITY_DN1994_c0_g1_i1_len_327_250196.p1_type_internal_len_110 | ---NA--- | | |
| TRINITY_DN3618_c0_g1_i1_len_327_250201.p1_type_internal_len_110 | XP_018027338.1 adenomatous polyposis coli homolog | P:GO:0001708; C:GO:0005881; P:GO:0007026; P:GO:0007389; F:GO:0008013; P:GO:0016477; C:GO:0030877; F:GO:0045295; P:GO:0045595; P:GO:0045732; P:GO:0090090 | |
| TRINITY_DN24870_c0_g1_i1_len_328_282287.p2_type_internal_len_110 | ---NA--- | | |
| TRINITY_DN13132_c0_g1_i4_len_328_282301.p1_type_internal_len_110 | ---NA--- | | |
| TRINITY_DN25010_c0_g1_i1_len_568_99973.p1_type_5prime_partial_len_109 | ---NA--- | | |
| TRINITY_DN10162_c0_g1_i1_len_327_108019.p1_type_internal_len_109 | ---NA--- | | |
| TRINITY_DN31448_c0_g1_i1_len_326_212319.p1_type_internal_len_109 | XP_018007338.1 transmembrane protein 104-like | | |
| TRINITY_DN19796_c0_g1_i1_len_403_207886.p1_type_5prime_partial_len_108 | ---NA--- | | |
| TRINITY_DN13822_c0_g1_i1_len_492_204493.p2_type_5prime_partial_len_108 | ---NA--- | | |
| TRINITY_DN42326_c0_g1_i1_len_326_212320.p1_type_internal_len_109 | ---NA--- | | |
| TRINITY_DN5613_c0_g1_i1_len_327_108022.p1_type_internal_len_109 | XP_019617495.1 betaine--homocysteine S-methyltransferase 1-like | F:GO:0008270; P:GO:0009086; P:GO:0032259; F:GO:0047150 | EC:2.1.1.5 |
| TRINITY_DN501_c0_g1_i1_len_488_243358.p1_type_5prime_partial_len_108 | ---NA--- | | |

| | | | |
|---|---|---|---|
| TRINITY_DN34540_c0_g1_i1_len_327_212237.p1_type_internal_len_109 | ---NA--- | | |
| TRINITY_DN33993_c0_g1_i1_len_327_212238.p1_type_internal_len_109 | ADV17345.1caspase 3 | F:GO:0004197; P:GO:0006508 | EC:3.4.22 |
| TRINITY_DN21632_c0_g1_i2_len_1843_994.p2_type_complete_len_108 | ---NA--- | | |
| TRINITY_DN20905_c0_g1_i1_len_328_44667.p1_type_internal_len_109 | ---NA--- | | |
| TRINITY_DN16730_c0_g1_i1_len_1327_268459.p1_type_complete_len_108 | ---NA--- | | |
| TRINITY_DN17275_c0_g1_i1_len_326_212311.p1_type_internal_len_109 | ---NA--- | | |
| TRINITY_DN41230_c0_g1_i1_len_326_212310.p1_type_internal_len_109 | XP_023724187.1actin-binding LIM protein 1 isoform X8 | | |
| TRINITY_DN38438_c0_g1_i1_len_325_212384.p1_type_internal_len_109 | ---NA--- | | |
| TRINITY_DN15767_c0_g1_i1_len_326_212362.p1_type_internal_len_109 | ---NA--- | | |
| TRINITY_DN21192_c0_g1_i1_len_1262_125161.p3_type_complete_len_108 | ---NA--- | | |
| TRINITY_DN24757_c0_g1_i1_len_332_310152.p1_type_5prime_partial_len_108 | ---NA--- | | |
| TRINITY_DN11526_c0_g1_i1_len_516_242613.p1_type_3prime_partial_len_109 | ---NA--- | | |
| TRINITY_DN42089_c0_g1_i1_len_326_212313.p1_type_internal_len_109 | ---NA--- | | |
| TRINITY_DN28916_c0_g1_i1_len_640_240111.p1_type_5prime_partial_len_108 | ---NA--- | | |
| TRINITY_DN5310_c0_g1_i2_len_324_212468.p1_type_internal_len_109 | XP_018025996.1 peroxidasin-like | F:GO:0004601; P:GO:0006979; P:GO:0007155; C:GO:0016020; F:GO:0020037; P:GO:0055114; P:GO:0098869 | EC:1.11.1.7 |

| | | | |
|---|---|---|---|
| TRINITY_DN18308_c0_g1_i1_len_1231_32397.p2_type_5prime_partial_len_108 | ---NA--- | | |
| TRINITY_DN15339_c0_g1_i1_len_491_204518.p1_type_complete_len_108 | ---NA--- | | |
| TRINITY_DN3827_c0_g1_i1_len_326_212321.p3_type_internal_len_109 | ---NA--- | | |
| TRINITY_DN14043_c0_g1_i1_len_326_108043.p1_type_internal_len_109 | ---NA--- | | |
| TRINITY_DN37837_c0_g1_i1_len_326_212361.p1_type_internal_len_109 | ---NA--- | | |
| TRINITY_DN7864_c0_g1_i1_len_327_212224.p1_type_internal_len_109 | XP_018016349.1 protein KIAA0100-like | | |
| TRINITY_DN20665_c0_g1_i1_len_1412_193588.p1_type_5prime_partial_len_108 | ---NA--- | | |
| TRINITY_DN1046_c0_g1_i2_len_325_212409.p1_type_internal_len_109 | XP_018011093.1 actin-binding LIM protein 1-like | F:GO:0003779; P:GO:0007010; F:GO:0016740; F:GO:0046872 | |
| TRINITY_DN26205_c0_g1_i1_len_2141_230675.p3_type_5prime_partial_len_108 | ---NA--- | | |
| TRINITY_DN26205_c0_g1_i1_len_2141_230675.p4_type_complete_len_108 | ---NA--- | | |
| TRINITY_DN14087_c0_g1_i1_len_830_64323.p3_type_3prime_partial_len_109 | ---NA--- | | |
| TRINITY_DN21991_c0_g1_i3_len_1786_1079.p2_type_5prime_partial_len_108 | ---NA--- | | |
| TRINITY_DN2750_c0_g1_i1_len_328_44642.p1_type_internal_len_109 | ---NA--- | | |
| TRINITY_DN17370_c0_g1_i1_len_818_198403.p2_type_complete_len_108 | ---NA--- | | |
| TRINITY_DN37972_c0_g1_i1_len_327_212272.p1_type_internal_len_109 | ---NA--- | | |
| TRINITY_DN7127_c0_g1_i1_len_326_15216.p1_type_internal_len_109 | ---NA--- | | |

| | | | |
|---|---|---|---|
| TRINITY_DN4968_c0_g1_i1_len_325_212374.p1_type_internal_len_109 | ---NA--- | | |
| TRINITY_DN39338_c0_g1_i1_len_324_212461.p1_type_internal_len_109 | XP_026666782.1peroxisomal targeting signal 1 receptor isoform X2 | | |
| TRINITY_DN34182_c0_g1_i1_len_327_212266.p1_type_internal_len_109 | ---NA--- | | |
| TRINITY_DN21426_c0_g1_i3_len_2476_229840.p2_type_complete_len_108 | ---NA--- | | |
| TRINITY_DN29374_c0_g1_i1_len_327_15152.p1_type_internal_len_109 | ---NA--- | | |
| TRINITY_DN12920_c0_g1_i1_len_495_243155.p1_type_5prime_partial_len_108 | ---NA--- | | |
| TRINITY_DN41900_c0_g1_i1_len_324_212452.p1_type_internal_len_109 | ---NA--- | | |
| TRINITY_DN26659_c0_g1_i1_len_606_240703.p1_type_5prime_partial_len_108 | ---NA--- | | |
| TRINITY_DN101_c0_g1_i1_len_327_107968.p1_type_internal_len_109 | ---NA--- | | |
| TRINITY_DN18405_c0_g1_i1_len_1730_92712.p3_type_complete_len_108 | KHJ83979.1S-adenosyl-L-homocysteine hydrolase | F:GO:0004013 | EC:3.3.1.1 |
| TRINITY_DN28751_c0_g1_i1_len_3949_187616.p6_type_complete_len_108 | ---NA--- | | |
| TRINITY_DN2114_c0_g1_i1_len_326_212293.p1_type_internal_len_109 | ---NA--- | | |
| TRINITY_DN24739_c0_g1_i1_len_703_199945.p1_type_complete_len_108 | ---NA--- | | |
| TRINITY_DN33784_c0_g1_i1_len_325_212391.p1_type_internal_len_109 | ---NA--- | | |
| TRINITY_DN39031_c0_g1_i1_len_351_210700.p1_type_5prime_partial_len_108 | ---NA--- | | |
| TRINITY_DN9230_c0_g1_i1_len_324_212453.p1_type_internal_len_109 | ---NA--- | | |

| | | | |
|---|---|---|---|
| TRINITY_DN26383_c0_g1_i4_len_2308_230220.p4_type_5prime_partial_len_108 | ---NA--- | | |
| TRINITY_DN2653_c0_g1_i1_len_327_212265.p1_type_internal_len_109 | ---NA--- | | |
| TRINITY_DN6263_c0_g1_i1_len_326_212365.p1_type_internal_len_109 | ---NA--- | | |
| TRINITY_DN12112_c0_g1_i1_len_1221_297720.p1_type_5prime_partial_len_108 | ---NA--- | | |
| TRINITY_DN32590_c0_g1_i1_len_325_212425.p1_type_internal_len_109 | ---NA--- | | |
| TRINITY_DN36280_c0_g1_i1_len_327_212286.p1_type_internal_len_109 | KZS09588.1Phosphoenolpyruvate synthase | F:GO:0016772 | |
| TRINITY_DN36527_c0_g1_i1_len_326_212367.p1_type_internal_len_109 | ---NA--- | | |
| TRINITY_DN22800_c0_g1_i2_len_1802_231741.p2_type_complete_len_108 | ---NA--- | | |
| TRINITY_DN10045_c0_g1_i1_len_639_240119.p3_type_5prime_partial_len_108 | ---NA--- | | |
| TRINITY_DN10823_c0_g1_i1_len_577_241226.p1_type_5prime_partial_len_108 | ---NA--- | | |
| TRINITY_DN12541_c0_g1_i1_len_324_310623.p1_type_internal_len_109 | ---NA--- | | |
| TRINITY_DN31713_c0_g1_i1_len_327_212283.p1_type_internal_len_109 | KDR16213.1Huntingtin | C:GO:0005634; C:GO:0005737 | |
| TRINITY_DN3105_c0_g1_i1_len_326_212307.p1_type_internal_len_109 | ---NA--- | | |
| TRINITY_DN4089_c0_g1_i1_len_327_212253.p1_type_internal_len_109 | ---NA--- | | |
| TRINITY_DN32641_c0_g1_i1_len_326_212306.p1_type_internal_len_109 | ---NA--- | | |
| TRINITY_DN19791_c0_g1_i1_len_4885_13.p3_type_3prime_partial_len_109 | ---NA--- | | |

| | | | |
|---|---|---|---|
| TRINITY_DN18099_c0_g1_i1_len_2467_29812.p2_type_5prime_partial_len_108 | ---NA--- | | |
| TRINITY_DN23386_c0_g1_i1_len_2173_230586.p3_type_5prime_partial_len_108 | ---NA--- | | |
| TRINITY_DN11572_c0_g1_i1_len_326_212298.p1_type_internal_len_109 | ---NA--- | | |
| TRINITY_DN22771_c0_g1_i1_len_324_310638.p1_type_internal_len_109 | ---NA--- | | |
| TRINITY_DN39779_c0_g1_i1_len_325_212432.p1_type_internal_len_109 | ---NA--- | | |
| 2802\|_\|c17705_f3p3_2494_isoform_c17705_full_length_coverage_3_non_full_length_coverage_3_isoform_length_2494_22466 | XP_018018787.1 F-box only protein 7-like | | |
| TRINITY_DN25799_c0_g1_i1_len_2627_229556.p1_type_5prime_partial_len_831 | XP_018019629.1  IWS1-like protein | C:GO:0005634 | |
| TRINITY_DN30245_c0_g1_i1_len_2870_188744.p1_type_5prime_partial_len_831 | XP_014345342.1 probable C-mannosyltransferase DPY19L3 isoform X1 | C:GO:0016020; C:GO:0016021 | |
| 2805\|_\|c2134_f2p6_2493_isoform_c2134_full_length_coverage_2_non_full_length_coverage_6_isoform_length_2493_28242 | ---NA--- | | |
| 2806\|_\|c23225_f2p0_2493_isoform_c23225_full_length_coverage_2_non_full_length_coverage_0_isoform_length_2493_18282 | XP_015111532.1 syntaxin-7 | | |
| 2807\|_\|c47067_f3p21_2493_isoform_c47067_full_length_coverage_3_non_full_length_coverage_21_isoform_length_2493_19411 | ---NA--- | | |
| TRINITY_DN30806_c0_g1_i1_len_2827_188815.p1_type_5prime_partial_len_831 | XP_002740361.1 HEAT repeat-containing protein 2-like | | |
| TRINITY_DN30405_c0_g1_i1_len_2655_189118.p1_type_5prime_partial_len_831 | XP_022239433.1serine/threonine-protein kinase 40-like | F:GO:0004672; F:GO:0005524; P:GO:0006468 | |
| TRINITY_DN22575_c1_g1_i1_len_2490_189472.p1_type_internal_len_831 | ---NA--- | | |

| | | | |
|---|---|---|---|
| 2811\|_\|c18277_f2p4_2489_isoform_c18277_full_length_coverage_2_non_full_length_coverage_4_isoform_length_2489_18076 | PNF40199.1PTCD3-like protein, mitochondrial | F:GO:0005488 | |
| TRINITY_DN32142_c0_g1_i1_len_326_212296.p1_type_internal_len_109 | ---NA--- | | |
| TRINITY_DN13517_c0_g1_i1_len_456_39722.p1_type_5prime_partial_len_108 | ---NA--- | | |
| TRINITY_DN2440_c0_g1_i1_len_326_212303.p1_type_internal_len_109 | PNF26149.1hypothetical protein B7P43_G04459 | F:GO:0003676; F:GO:0008270; F:GO:0016787 | |
| TRINITY_DN8417_c0_g1_i1_len_1022_160479.p1_type_5prime_partial_len_108 | ---NA--- | | |
| TRINITY_DN19849_c0_g1_i1_len_685_200229.p1_type_5prime_partial_len_108 | ---NA--- | | |
| TRINITY_DN14722_c0_g1_i1_len_326_310471.p1_type_internal_len_109 | ---NA--- | | |
| TRINITY_DN36420_c0_g1_i1_len_328_212181.p1_type_internal_len_109 | ---NA--- | | |
| TRINITY_DN38823_c0_g1_i1_len_328_212186.p1_type_internal_len_109 | XP_018024103.1 organic solute transporter subunit alpha-like | | |
| TRINITY_DN2547_c0_g1_i1_len_326_44762.p1_type_internal_len_109 | ---NA--- | | |
| TRINITY_DN26626_c0_g1_i1_len_326_44763.p1_type_internal_len_109 | ---NA--- | | |
| TRINITY_DN909_c0_g1_i1_len_522_203584.p1_type_5prime_partial_len_108 | KFM78402.1F-box/LRR-repeat protein 5 | | |
| TRINITY_DN21743_c0_g1_i2_len_626_302016.p1_type_3prime_partial_len_109 | ---NA--- | | |
| TRINITY_DN12582_c0_g1_i1_len_326_44785.p1_type_internal_len_109 | ABB73282.1reverse transcriptase | F:GO:0003964; P:GO:0006278 | EC:2.7.7.49 |
| TRINITY_DN9924_c0_g1_i1_len_326_212350.p1_type_internal_len_109 | ---NA--- | | |
| TRINITY_DN13259_c0_g1_i1_len_328_212169.p1_type_5prime_partial_len_108 | ---NA--- | | |

| | | | |
|---|---|---|---|
| TRINITY_DN22072_c0_g1_i1_len_326_44777.p1_type_internal_len_109 | ---NA--- | | |
| TRINITY_DN5666_c0_g1_i1_len_326_212336.p1_type_internal_len_109 | ---NA--- | | |
| TRINITY_DN5029_c0_g1_i1_len_325_212446.p1_type_internal_len_109 | XP_016925208.1 27 kDa hemolymph protein | C:GO:0016020; C:GO:0016021 | |
| TRINITY_DN8695_c0_g1_i1_len_328_212192.p1_type_internal_len_109 | ---NA--- | | |
| TRINITY_DN16180_c0_g1_i3_len_327_310448.p2_type_internal_len_109 | ---NA--- | | |
| TRINITY_DN35010_c0_g1_i1_len_328_212191.p1_type_internal_len_109 | ---NA--- | | |
| TRINITY_DN19086_c0_g1_i2_len_1761_231900.p3_type_complete_len_108 | ---NA--- | | |
| TRINITY_DN39336_c0_g1_i1_len_328_212189.p1_type_internal_len_109 | ---NA--- | | |
| TRINITY_DN3304_c0_g1_i1_len_327_310462.p1_type_internal_len_109 | ---NA--- | | |
| TRINITY_DN26685_c0_g1_i1_len_327_44751.p1_type_internal_len_109 | XP_007662933.1 polypeptide N-acetylgalactosaminyltransferase 2 | | |
| TRINITY_DN22227_c0_g1_i1_len_710_65548.p1_type_5prime_partial_len_108 | ---NA--- | | |
| TRINITY_DN23002_c0_g1_i1_len_470_276809.p1_type_5prime_partial_len_108 | ---NA--- | | |
| TRINITY_DN6231_c0_g1_i1_len_479_243617.p1_type_5prime_partial_len_108 | ---NA--- | | |
| TRINITY_DN1505_c0_g1_i1_len_450_244566.p2_type_5prime_partial_len_108 | ---NA--- | | |
| TRINITY_DN22164_c0_g1_i1_len_326_310505.p1_type_internal_len_109 | XP_015905073.1uncharacterized protein LOC107437515 | C:GO:0005576; P:GO:0006030; F:GO:0008061 | |
| TRINITY_DN9340_c0_g1_i1_len_326_212348.p2_type_internal_len_109 | ---NA--- | | |

| | | | |
|---|---|---|---|
| TRINITY_DN13214_c0_g1_i2_len_457_39693.p1_type_5prime_partial_len_108 | ---NA--- | | |
| TRINITY_DN21419_c0_g1_i1_len_1696_232148.p2_type_complete_len_108 | ---NA--- | | |
| TRINITY_DN21982_c0_g1_i4_len_1843_156550.p3_type_5prime_partial_len_108 | ---NA--- | | |
| TRINITY_DN30714_c0_g1_i1_len_5112_187098.p5_type_complete_len_108 | ---NA--- | | |
| TRINITY_DN29578_c0_g1_i2_len_5082_187102.p3_type_5prime_partial_len_108 | ---NA--- | | |
| TRINITY_DN19682_c0_g1_i1_len_1359_158436.p2_type_5prime_partial_len_108 | ---NA--- | | |
| TRINITY_DN42109_c0_g1_i1_len_326_212343.p1_type_internal_len_109 | ---NA--- | | |
| TRINITY_DN37379_c0_g1_i1_len_326_212347.p1_type_internal_len_109 | ---NA--- | | |
| TRINITY_DN2403_c0_g1_i1_len_326_44804.p1_type_internal_len_109 | ---NA--- | | |
| TRINITY_DN17537_c0_g1_i1_len_498_204335.p2_type_5prime_partial_len_108 | ---NA--- | | |
| TRINITY_DN20408_c0_g1_i1_len_498_204326.p1_type_5prime_partial_len_108 | ---NA--- | | |
| TRINITY_DN19512_c0_g1_i1_len_3126_29434.p5_type_5prime_partial_len_108 | ---NA--- | | |
| TRINITY_DN21193_c0_g1_i4_len_904_63736.p1_type_complete_len_108 | ---NA--- | | |
| TRINITY_DN135_c0_g1_i1_len_325_108122.p1_type_internal_len_109 | ---NA--- | | |
| TRINITY_DN15699_c0_g1_i1_len_545_241883.p2_type_5prime_partial_len_108 | ---NA--- | | |
| TRINITY_DN7095_c0_g1_i1_len_327_212209.p1_type_internal_len_109 | ---NA--- | | |

| | | | |
|---|---|---|---|
| TRINITY_DN22459_c0_g1_i1_len_5742_186969.p2_type_5prime_partial_len_108 | ---NA--- | | |
| TRINITY_DN1965_c0_g1_i1_len_333_310093.p1_type_5prime_partial_len_108 | ---NA--- | | |
| TRINITY_DN14233_c0_g1_i1_len_325_212443.p1_type_internal_len_109 | ---NA--- | | |
| TRINITY_DN10617_c0_g1_i1_len_328_310401.p1_type_internal_len_109 | ---NA--- | | |
| TRINITY_DN40625_c0_g1_i1_len_325_212403.p1_type_internal_len_109 | KZS04530.1putative Zinc finger homeobox protein 3 | P:GO:0000122; F:GO:0000978; C:GO:0005634; F:GO:0008270; P:GO:0045893 | |
| TRINITY_DN10896_c0_g1_i2_len_494_204436.p1_type_5prime_partial_len_108 | ---NA--- | | |
| TRINITY_DN24648_c0_g1_i1_len_1125_62364.p3_type_complete_len_108 | ---NA--- | | |
| TRINITY_DN2085_c0_g1_i1_len_325_108076.p1_type_internal_len_109 | ---NA--- | | |
| TRINITY_DN27222_c0_g1_i1_len_402_11657.p1_type_5prime_partial_len_108 | ---NA--- | | |
| TRINITY_DN14731_c0_g1_i2_len_643_240068.p1_type_5prime_partial_len_108 | ---NA--- | | |
| TRINITY_DN21673_c0_g1_i3_len_673_66003.p1_type_3prime_partial_len_109 | ---NA--- | | |
| TRINITY_DN17234_c0_g1_i1_len_523_203561.p2_type_5prime_partial_len_108 | ---NA--- | | |
| TRINITY_DN10326_c0_g1_i1_len_460_244238.p1_type_5prime_partial_len_108 | ---NA--- | | |
| TRINITY_DN16866_c0_g1_i1_len_328_15133.p1_type_internal_len_109 | ---NA--- | | |
| TRINITY_DN38386_c0_g1_i1_len_324_212475.p1_type_internal_len_109 | ---NA--- | | |
| TRINITY_DN10488_c0_g1_i1_len_451_277321.p1_type_complete_len_108 | ---NA--- | | |

| | | | |
|---|---|---|---|
| TRINITY_DN719_c0_g1_i1_len_326_212355.p1_type_internal_len_109 | AQT26398.1chitin-binding protein | F:GO:0042302 | |
| TRINITY_DN33983_c0_g1_i1_len_326_212330.p2_type_internal_len_109 | XP_026730758.1protein stoned-B-like | P:GO:0006897 | |
| TRINITY_DN36482_c0_g1_i1_len_326_212329.p1_type_internal_len_109 | ---NA--- | | |
| TRINITY_DN32183_c0_g1_i1_len_324_108169.p1_type_internal_len_109 | XP_018023453.1PREDICTED: uncharacterized protein LOC108679354 isoform X4 | | |
| TRINITY_DN22405_c0_g1_i1_len_700_239202.p1_type_complete_len_108 | ---NA--- | | |
| TRINITY_DN22877_c1_g1_i3_len_944_161136.p1_type_complete_len_108 | ---NA--- | | |
| TRINITY_DN15627_c0_g1_i1_len_329_212063.p1_type_3prime_partial_len_109 | ---NA--- | | |
| TRINITY_DN39418_c0_g1_i1_len_326_212357.p1_type_internal_len_109 | ---NA--- | | |
| TRINITY_DN9137_c0_g1_i1_len_327_310420.p1_type_internal_len_109 | ---NA--- | | |
| TRINITY_DN36021_c0_g1_i1_len_325_212445.p1_type_internal_len_109 | ---NA--- | | |
| TRINITY_DN25127_c0_g1_i1_len_4004_228182.p3_type_complete_len_108 | XP_022196166.1coiled-coil domain-containing protein 186 isoform X2 | | |
| TRINITY_DN36829_c0_g1_i1_len_327_250170.p1_type_internal_len_109 | XP_018021520.1 A disintegrin and metalloproteinase with thrombospondin motifs 2-like | F:GO:0008233 | |
| TRINITY_DN29728_c0_g1_i1_len_327_250175.p1_type_internal_len_109 | ---NA--- | | |
| TRINITY_DN27842_c0_g1_i1_len_327_250177.p1_type_internal_len_109 | ---NA--- | | |
| TRINITY_DN32612_c0_g1_i1_len_541_166155.p2_type_5prime_partial_len_108 | ALP06206.1crustacean cardioactive peptide | | |

| | | | |
|---|---|---|---|
| TRINITY_DN33621_c0_g1_i1_len_327_250156.p1_type_internal_len_109 | XP_018013472.1 N-acetylated-alpha-linked acidic dipeptidase 2-like | | |
| TRINITY_DN27691_c0_g1_i1_len_327_250157.p1_type_internal_len_109 | ---NA--- | | |
| TRINITY_DN33554_c0_g1_i1_len_327_250167.p1_type_internal_len_109 | ---NA--- | | |
| TRINITY_DN34876_c0_g1_i1_len_326_250208.p1_type_internal_len_109 | ---NA--- | | |
| TRINITY_DN9893_c0_g1_i1_len_341_14348.p1_type_5prime_partial_len_108 | ---NA--- | | |
| TRINITY_DN5582_c0_g1_i1_len_326_250212.p1_type_internal_len_109 | ---NA--- | | |
| TRINITY_DN11379_c0_g1_i1_len_326_250227.p1_type_internal_len_109 | ---NA--- | | |
| TRINITY_DN3240_c0_g1_i1_len_327_250193.p1_type_internal_len_109 | ---NA--- | | |
| TRINITY_DN34452_c0_g1_i1_len_326_250206.p1_type_internal_len_109 | ---NA--- | | |
| TRINITY_DN14569_c0_g1_i1_len_326_250207.p1_type_internal_len_109 | ---NA--- | | |
| TRINITY_DN4493_c0_g1_i1_len_436_206491.p1_type_5prime_partial_len_108 | ---NA--- | | |
| TRINITY_DN33343_c0_g1_i1_len_328_250103.p1_type_internal_len_109 | ---NA--- | | |
| TRINITY_DN14124_c1_g1_i1_len_328_250105.p1_type_internal_len_109 | ---NA--- | | |
| TRINITY_DN23790_c0_g1_i1_len_363_137020.p1_type_5prime_partial_len_108 | ABB73282.1reverse transcriptase | F:GO:0003964; P:GO:0006278 | EC:2.7.7.49 |
| TRINITY_DN30839_c0_g1_i1_len_324_15314.p1_type_internal_len_109 | XP_018025794.1 voltage-dependent calcium channel subunit alpha-2/delta-3-like | | |

| | | | |
|---|---|---|---|
| TRINITY_DN26084_c0_g1_i1_len_369_136759.p1_type_5prime_partial_len_108 | XP_019864802.1 sushi, von Willebrand factor type A, EGF and pentraxin domain-containing protein 1-like | | |
| TRINITY_DN31068_c0_g1_i1_len_2346_189830.p1_type_complete_len_108 | ---NA--- | | |
| TRINITY_DN34811_c0_g1_i1_len_325_250332.p1_type_internal_len_109 | KZS03269.1KH domain-containing protein C56G2.1 | F:GO:0005488 | |
| TRINITY_DN17678_c0_g1_i1_len_325_250337.p1_type_internal_len_109 | ---NA--- | | |
| TRINITY_DN7241_c0_g1_i2_len_325_250325.p1_type_internal_len_109 | ---NA--- | | |
| TRINITY_DN6930_c0_g1_i1_len_985_236242.p1_type_5prime_partial_len_108 | ---NA--- | | |
| TRINITY_DN30952_c0_g1_i3_len_1583_192645.p3_type_complete_len_108 | ---NA--- | | |
| 2812\|_\|c2792_f7p1_2489_isoform_c2792_full_length_coverage_7_non_full_length_coverage_1_isoform_length_2489_16705 | XP_018024825.1 U4/U6 small nuclear ribonucleoprotein Prp3-like isoform X2 | | |
| 2813\|_\|c15421_f22p22_2489_isoform_c15421_full_length_coverage_22_non_full_length_coverage_22_isoform_length_2489_17657 | XP_018021038.1 protein arginine N-methyltransferase 1-like | | |
| 2814\|_\|c2566_f2p1_2488_isoform_c2566_full_length_coverage_2_non_full_length_coverage_1_isoform_length_2488_11976 | XP_018579245.1phosphatidylinositol 3-kinase catalytic subunit type 3 isoform X2 | F:GO:0005524; F:GO:0016303; P:GO:0036092; P:GO:0046854; P:GO:0048015 | EC:2.7.1.137 |
| 2815\|_\|c33733_f3p1_2488_isoform_c33733_full_length_coverage_3_non_full_length_coverage_1_isoform_length_2488_23299 | ---NA--- | | |
| 2816\|_\|c5670_f2p2_2487_isoform_c5670_full_length_coverage_2_non_full_length_coverage_2_isoform_length_2487_12490 | PRD20660.1zinc finger protein | F:GO:0003676 | |

| | | | |
|---|---|---|---|
| 2817\|_\|c2358_f8p4_2487_isoform_c2358_full_length_coverage_8_non_full_length_coverage_4_isoform_length_2487_11805 | XP_002742449.2 origin recognition complex subunit 2-like | C:GO:0000808; C:GO:0005634; P:GO:0006260 | |
| TRINITY_DN22691_c0_g1_i1_len_2732_58361.p1_type_5prime_partial_len_829 | XP_015520345.1 disks large 1 tumor suppressor protein isoform X4 | | |
| 2822\|_\|c2149_f4p2_2486_isoform_c2149_full_length_coverage_4_non_full_length_coverage_2_isoform_length_2486_20739 | ---NA--- | | |
| 2823\|_\|c2094_f3p6_2486_isoform_c2094_full_length_coverage_3_non_full_length_coverage_6_isoform_length_2486_11652 | XP_018015767.1 THO complex subunit 5 homolog B-like | | |
| 2826\|_\|c20485_f2p0_2485_isoform_c20485_full_length_coverage_2_non_full_length_coverage_0_isoform_length_2485_18187 | XP_018024429.1 BTB/POZ domain-containing protein 6-like | | |
| TRINITY_DN29449_c0_g1_i1_len_327_282337.p1_type_internal_len_109 | XP_018019423.1PREDICTED: uncharacterized protein LOC108675887 | | |
| TRINITY_DN28442_c0_g1_i1_len_324_250362.p1_type_internal_len_109 | XP_018008658.1  dystrobrevin beta-like | | |
| TRINITY_DN10521_c0_g1_i1_len_324_250392.p1_type_internal_len_109 | ---NA--- | | |
| TRINITY_DN2805_c0_g1_i1_len_326_282397.p1_type_internal_len_109 | XP_018023299.1PREDICTED: uncharacterized protein LOC108679210 | | |
| TRINITY_DN30008_c0_g1_i1_len_324_250357.p1_type_internal_len_109 | ---NA--- | | |
| TRINITY_DN11584_c0_g1_i1_len_326_282406.p1_type_internal_len_109 | ---NA--- | | |
| TRINITY_DN443_c0_g1_i1_len_516_166723.p1_type_5prime_partial_len_108 | ---NA--- | | |
| TRINITY_DN28121_c0_g1_i1_len_326_250237.p1_type_internal_len_109 | ---NA--- | | |
| TRINITY_DN5026_c0_g1_i1_len_326_250241.p1_type_internal_len_109 | XP_018008658.1  dystrobrevin beta-like | | |

| | | | |
|---|---|---|---|
| TRINITY_DN26798_c0_g1_i1_len_326_250247.p1_type_internal_len_109 | ---NA--- | | |
| TRINITY_DN33771_c0_g1_i1_len_326_250248.p1_type_internal_len_109 | ---NA--- | | |
| TRINITY_DN10546_c0_g1_i1_len_326_250252.p1_type_internal_len_109 | ---NA--- | | |
| TRINITY_DN27237_c0_g1_i1_len_326_250258.p1_type_internal_len_109 | ---NA--- | | |
| TRINITY_DN24304_c0_g1_i1_len_2207_190219.p2_type_5prime_partial_len_108 | ---NA--- | | |
| TRINITY_DN3020_c0_g1_i1_len_551_202839.p2_type_3prime_partial_len_109 | ---NA--- | | |
| TRINITY_DN28657_c0_g1_i1_len_2202_190233.p2_type_5prime_partial_len_108 | ---NA--- | | |
| TRINITY_DN33986_c0_g1_i1_len_506_166920.p1_type_5prime_partial_len_108 | ---NA--- | | |
| TRINITY_DN30823_c0_g1_i1_len_328_282257.p1_type_internal_len_109 | ---NA--- | | |
| TRINITY_DN10031_c0_g1_i1_len_325_250299.p1_type_internal_len_109 | ---NA--- | | |
| TRINITY_DN3464_c1_g1_i1_len_325_250300.p1_type_internal_len_109 | ---NA--- | | |
| TRINITY_DN29524_c0_g1_i1_len_325_250303.p1_type_internal_len_109 | AWD73767.1chitinase 5 | F:GO:0004553; C:GO:0005576; P:GO:0005975; P:GO:0006030; F:GO:0008061 | |
| TRINITY_DN4089_c0_g1_i1_len_325_250314.p1_type_internal_len_109 | ---NA--- | | |
| TRINITY_DN37862_c0_g1_i1_len_326_250266.p1_type_internal_len_109 | ---NA--- | | |
| TRINITY_DN21393_c0_g1_i1_len_981_236267.p1_type_5prime_partial_len_108 | ---NA--- | | |
| TRINITY_DN33793_c0_g1_i1_len_326_250275.p1_type_internal_len_109 | ---NA--- | | |

| | | | |
|---|---|---|---|
| TRINITY_DN4297_c0_g1_i1_len_325_250284.p1_type_internal_len_109 | XP_014348736.1 tubulin alpha chain | F:GO:0005200; F:GO:0005525; C:GO:0005874; P:GO:0007010; P:GO:0007017 | |
| TRINITY_DN32226_c0_g1_i1_len_325_250294.p1_type_internal_len_109 | ---NA--- | | |
| TRINITY_DN12381_c0_g1_i1_len_654_164140.p1_type_complete_len_108 | ---NA--- | | |
| TRINITY_DN32061_c0_g1_i1_len_326_76166.p1_type_internal_len_109 | ---NA--- | | |
| TRINITY_DN8680_c0_g1_i1_len_326_76159.p1_type_internal_len_109 | ---NA--- | | |
| TRINITY_DN31134_c0_g1_i1_len_328_76064.p1_type_internal_len_109 | XP_018024896.1 fat-like cadherin-related tumor suppressor homolog | | |
| TRINITY_DN25965_c0_g1_i1_len_324_76266.p1_type_internal_len_109 | XP_018019107.1 14-3-3 protein zeta isoform X2 | F:GO:0019904 | |
| TRINITY_DN34669_c0_g1_i1_len_325_76249.p1_type_internal_len_109 | ---NA--- | | |
| TRINITY_DN10085_c0_g1_i1_len_664_164002.p2_type_5prime_partial_len_108 | ---NA--- | | |
| TRINITY_DN35714_c0_g1_i1_len_325_76226.p1_type_3prime_partial_len_109 | KRT85284.1Immunoglobulin | F:GO:0005007; F:GO:0005524; P:GO:0008284; P:GO:0008543; C:GO:0016021; P:GO:0018108 | EC:2.7.10; EC:2.7.10.1; EC:2.7.11 |
| TRINITY_DN29985_c0_g1_i1_len_325_76196.p2_type_internal_len_109 | ---NA--- | | |
| TRINITY_DN6820_c0_g1_i1_len_325_76194.p1_type_internal_len_109 | XP_018020177.1 syndetin-like | P:GO:0016197; C:GO:0044444 | |
| TRINITY_DN29384_c1_g1_i17_len_2527_189394.p2_type_5prime_partial_len_108 | ---NA--- | | |
| TRINITY_DN18570_c0_g1_i2_len_494_101432.p1_type_complete_len_108 | XP_026733440.1HIG1 domain family member 2A, mitochondrial | C:GO:0016020 | |
| TRINITY_DN23375_c0_g1_i1_len_469_69987.p2_type_complete_len_108 | ---NA--- | | |

| | | | |
|---|---|---|---|
| TRINITY_DN33470_c0_g1_i1_len_577_165401.p1_type_5prime_partial_len_108 | ---NA--- | | |
| TRINITY_DN4313_c1_g1_i1_len_334_249769.p1_type_5prime_partial_len_108 | ---NA--- | | |
| TRINITY_DN22633_c0_g1_i1_len_2219_58914.p4_type_5prime_partial_len_108 | ---NA--- | | |
| TRINITY_DN20556_c0_g2_i1_len_339_281670.p1_type_5prime_partial_len_108 | ---NA--- | | |
| TRINITY_DN9319_c0_g1_i1_len_339_249411.p1_type_5prime_partial_len_108 | ---NA--- | | |
| TRINITY_DN22189_c0_g1_i2_len_400_135399.p1_type_5prime_partial_len_108 | ODM95427.1Trans-1,2-dihydrobenzene-1,2-diol dehydrogenase | F:GO:0016491; P:GO:0055114 | |
| TRINITY_DN14084_c0_g1_i1_len_624_164610.p2_type_complete_len_108 | ---NA--- | | |
| TRINITY_DN17764_c0_g1_i1_len_406_135145.p2_type_5prime_partial_len_108 | ---NA--- | | |
| TRINITY_DN20303_c0_g1_i1_len_1268_234254.p1_type_complete_len_108 | ---NA--- | | |
| TRINITY_DN21875_c0_g1_i1_len_1039_196201.p2_type_5prime_partial_len_108 | ---NA--- | | |
| TRINITY_DN30667_c0_g1_i1_len_1038_196212.p1_type_complete_len_108 | ---NA--- | | |
| TRINITY_DN19990_c0_g1_i1_len_1057_3296.p2_type_5prime_partial_len_108 | ---NA--- | | |
| TRINITY_DN28870_c0_g1_i1_len_1892_191296.p3_type_complete_len_108 | ---NA--- | | |
| TRINITY_DN28019_c1_g1_i1_len_995_196596.p1_type_5prime_partial_len_108 | ---NA--- | | |
| TRINITY_DN9545_c0_g1_i1_len_427_206838.p2_type_5prime_partial_len_108 | ---NA--- | | |
| TRINITY_DN29718_c0_g1_i3_len_2007_190868.p2_type_3prime_partial_len_109 | ---NA--- | | |

| | | | |
|---|---|---|---|
| TRINITY_DN11909_c0_g1_i1_len_412_306603.p1_type_5prime_partial_len_108 | ---NA--- | | |
| TRINITY_DN21738_c0_g1_i3_len_1996_59371.p3_type_complete_len_108 | ---NA--- | | |
| TRINITY_DN21257_c0_g1_i1_len_382_208940.p1_type_complete_len_108 | ---NA--- | | |
| TRINITY_DN16719_c0_g1_i2_len_896_270966.p1_type_5prime_partial_len_108 | ---NA--- | | |
| TRINITY_DN26679_c0_g1_i1_len_939_197106.p1_type_complete_len_108 | ---NA--- | | |
| TRINITY_DN5541_c0_g1_i1_len_374_209312.p1_type_5prime_partial_len_108 | ---NA--- | | |
| TRINITY_DN20_c0_g1_i1_len_373_209344.p1_type_5prime_partial_len_108 | ---NA--- | | |
| TRINITY_DN8951_c0_g1_i2_len_378_209133.p1_type_5prime_partial_len_108 | AUW64506.2serine protease 5 | F:GO:0004252; P:GO:0006508 | EC:3.4.21 |
| TRINITY_DN20342_c0_g1_i1_len_953_196960.p1_type_complete_len_108 | ---NA--- | | |
| TRINITY_DN18452_c0_g1_i1_len_938_270638.p1_type_complete_len_108 | ---NA--- | | |
| TRINITY_DN13181_c0_g1_i3_len_397_307138.p1_type_complete_len_108 | ---NA--- | | |
| TRINITY_DN23981_c0_g1_i1_len_5496_227684.p3_type_5prime_partial_len_108 | KZS18228.1E3 ubiquitin-protein ligase listerin | F:GO:0008270; P:GO:0016567; F:GO:0016874; F:GO:0046872; F:GO:0061630; C:GO:1990112; P:GO:1990116 | |
| TRINITY_DN28368_c0_g1_i1_len_380_73199.p1_type_5prime_partial_len_108 | ---NA--- | | |
| TRINITY_DN24040_c0_g1_i2_len_2246_122721.p1_type_complete_len_108 | ---NA--- | | |
| TRINITY_DN24758_c0_g1_i3_len_1670_192210.p6_type_5prime_partial_len_108 | ---NA--- | | |

| | | | |
|---|---|---|---|
| TRINITY_DN444_c0_g1_i1_len_329_138905.p1_type_5prime_partial_len_108 | ---NA--- | | |
| TRINITY_DN382_c0_g1_i1_len_806_271695.p1_type_5prime_partial_len_108 | ---NA--- | | |
| TRINITY_DN38536_c0_g1_i1_len_394_208302.p1_type_5prime_partial_len_108 | ---NA--- | | |
| TRINITY_DN23101_c0_g2_i1_len_327_139069.p1_type_internal_len_109 | ---NA--- | | |
| TRINITY_DN10703_c0_g1_i1_len_326_139095.p1_type_internal_len_109 | BBC20718.1integrin alpha V | P:GO:0007155; P:GO:0007229; C:GO:0008305 | |
| TRINITY_DN33048_c0_g1_i1_len_328_139014.p1_type_internal_len_109 | ---NA--- | | |
| TRINITY_DN24387_c0_g1_i1_len_327_139051.p1_type_internal_len_109 | ---NA--- | | |
| TRINITY_DN28548_c0_g1_i1_len_327_139053.p1_type_internal_len_109 | XP_024080935.1titin isoform X1 | | |
| TRINITY_DN34145_c0_g1_i1_len_324_173918.p1_type_internal_len_109 | XP_021930396.1uncharacterized protein CG10915-like isoform X7 | F:GO:0005199 | |
| TRINITY_DN166_c0_g1_i1_len_325_282478.p1_type_internal_len_109 | ---NA--- | | |
| TRINITY_DN9586_c0_g1_i1_len_324_282489.p1_type_internal_len_109 | XP_018018463.1 protein EFR3 homolog B-like | | |
| TRINITY_DN6217_c0_g1_i1_len_324_250401.p1_type_internal_len_109 | ---NA--- | | |
| TRINITY_DN6610_c0_g1_i1_len_325_282462.p1_type_internal_len_109 | ---NA--- | | |
| TRINITY_DN22179_c0_g1_i1_len_325_282465.p1_type_internal_len_109 | ---NA--- | | |
| TRINITY_DN23771_c0_g1_i1_len_439_168783.p1_type_complete_len_108 | ---NA--- | | |
| TRINITY_DN10307_c0_g1_i1_len_439_168807.p1_type_5prime_partial_len_108 | ---NA--- | | |

| | | | |
|---|---|---|---|
| TRINITY_DN15036_c0_g1_i1_len_324_139235.p1_type_internal_len_109 | OWF38525.1Stress protein DDR48 | | |
| TRINITY_DN18976_c0_g1_i1_len_324_139237.p1_type_internal_len_109 | ---NA--- | | |
| TRINITY_DN33472_c0_g1_i1_len_324_139245.p1_type_internal_len_109 | ---NA--- | | |
| TRINITY_DN18020_c0_g1_i2_len_324_139249.p1_type_internal_len_109 | ---NA--- | | |
| TRINITY_DN20407_c0_g1_i1_len_2617_265319.p3_type_complete_len_108 | ---NA--- | | |
| TRINITY_DN30680_c0_g1_i1_len_329_173605.p1_type_5prime_partial_len_108 | ---NA--- | | |
| TRINITY_DN11263_c0_g1_i1_len_327_173738.p2_type_3prime_partial_len_109 | ADG86240.1triosephosphate isomerase | F:GO:0004807; P:GO:0006094; P:GO:0006096 | EC:5.3.1.1 |
| TRINITY_DN5786_c0_g1_i1_len_415_306481.p1_type_5prime_partial_len_108 | ---NA--- | | |
| TRINITY_DN10389_c0_g1_i2_len_328_173675.p2_type_internal_len_109 | ---NA--- | | |
| TRINITY_DN8250_c0_g1_i1_len_326_139119.p1_type_internal_len_109 | ---NA--- | | |
| TRINITY_DN4773_c0_g1_i1_len_326_139100.p1_type_internal_len_109 | ---NA--- | | |
| TRINITY_DN27396_c0_g1_i1_len_325_173873.p1_type_internal_len_109 | ---NA--- | | |
| TRINITY_DN31003_c0_g1_i1_len_326_139106.p1_type_internal_len_109 | XP_018016653.1 SCY1-like protein 2 | | |
| TRINITY_DN17488_c0_g1_i1_len_3498_153917.p1_type_complete_len_828 | AKB96224.1heat shock protein | F:GO:0005524 | |
| 2829\|_\|c6735_f3p3_2484_isoform_c6735_full_length_coverage_3_non_full_length_coverage_3_isoform_length_2484_17074 | ALA09304.1cytochrome P450 NADPH reductase | F:GO:0003958; C:GO:0005789; F:GO:0010181; F:GO:0050660; F:GO:0050661; P:GO:0055114 | EC:1.6.2.4 |
| TRINITY_DN25576_c0_g1_i1_len_2526_189395.p1_type_3prime_partial_len_829 | XP_014279929.1eukaryotic translation initiation factor 2-alpha kinase | | |

<table>
<tr><th></th><th></th><th></th><th></th></tr>
<tr><td>TRINITY_DN27452_c1_g1_i2_len_3981_187598.p1_type_5prime_partial_len_828</td><td>XP_018007798.1 guanine deaminase-like</td><td>F:GO:0016810</td><td></td></tr>
<tr><td>2832|_|c20290_f3p7_2484_isoform_c20290_full_length_coverage_3_non_full_length_coverage_7_isoform_length_2484_13772</td><td>AEY84718.1YIPF3-like protein</td><td>C:GO:0016021</td><td></td></tr>
<tr><td>2833|_|c1989_f4p0_2484_isoform_c1989_full_length_coverage_4_non_full_length_coverage_0_isoform_length_2484_16107</td><td>XP_023723418.1O-glucosyltransferase rumi homolog</td><td></td><td></td></tr>
<tr><td>2834|_|c8030_f2p0_2483_isoform_c8030_full_length_coverage_2_non_full_length_coverage_0_isoform_length_2483_21740</td><td>---NA---</td><td></td><td></td></tr>
<tr><td>2835|_|c1217_f31p14_2483_isoform_c1217_full_length_coverage_31_non_full_length_coverage_14_isoform_length_2483_15822</td><td>XP_022100353.1protein SDA1 homolog isoform X1</td><td>P:GO:0042254</td><td></td></tr>
<tr><td>2836|_|c5938_f4p6_2483_isoform_c5938_full_length_coverage_4_non_full_length_coverage_6_isoform_length_2483_12515</td><td>XP_018910664.1 probable N-acetyltransferase CML3</td><td></td><td></td></tr>
<tr><td>2837|_|c2720_f3p26_2483_isoform_c2720_full_length_coverage_3_non_full_length_coverage_26_isoform_length_2483_12103</td><td>XP_026680174.1protein transport protein Sec61 subunit alpha isoform X1</td><td>P:GO:0015031; C:GO:0016021</td><td></td></tr>
<tr><td>TRINITY_DN31259_c0_g4_i1_len_468_205274.p1_type_5prime_partial_len_108</td><td>---NA---</td><td></td><td></td></tr>
<tr><td>TRINITY_DN33334_c0_g1_i1_len_326_173793.p1_type_internal_len_109</td><td>AUW64506.2serine protease 5</td><td>F:GO:0008236</td><td></td></tr>
<tr><td>TRINITY_DN3841_c0_g1_i1_len_325_139163.p1_type_internal_len_109</td><td>---NA---</td><td></td><td></td></tr>
<tr><td>TRINITY_DN33963_c0_g1_i1_len_326_173832.p1_type_internal_len_109</td><td>---NA---</td><td></td><td></td></tr>
<tr><td>TRINITY_DN31744_c0_g1_i1_len_326_173824.p1_type_internal_len_109</td><td>---NA---</td><td></td><td></td></tr>
<tr><td>TRINITY_DN27128_c0_g1_i1_len_326_173821.p1_type_internal_len_109</td><td>---NA---</td><td></td><td></td></tr>
</table>

| | | | |
|---|---|---|---|
| TRINITY_DN30273_c0_g1_i1_len_373_308108.p1_type_5prime_partial_len_108 | ---NA--- | | |
| TRINITY_DN822_c0_g1_i1_len_403_246274.p1_type_5prime_partial_len_108 | ---NA--- | | |
| TRINITY_DN7381_c0_g1_i1_len_594_274235.p1_type_complete_len_108 | ---NA--- | | |
| TRINITY_DN17170_c0_g1_i1_len_2866_154495.p1_type_3prime_partial_len_109 | ---NA--- | | |
| TRINITY_DN0_c0_g1_i1_len_328_15111.p1_type_internal_len_109 | XP_026277723.1transcriptional regulator ovo-like isoform X2 | | |
| TRINITY_DN27940_c0_g1_i1_len_2988_188555.p4_type_5prime_partial_len_108 | ---NA--- | | |
| TRINITY_DN31088_c7_g2_i2_len_1510_193045.p1_type_complete_len_108 | ---NA--- | | |
| TRINITY_DN21114_c0_g1_i1_len_735_199518.p2_type_complete_len_108 | ---NA--- | | |
| TRINITY_DN21035_c0_g1_i1_len_847_4503.p1_type_5prime_partial_len_108 | ---NA--- | | |
| TRINITY_DN23495_c0_g1_i1_len_1468_233179.p2_type_complete_len_108 | ---NA--- | | |
| TRINITY_DN16096_c0_g1_i1_len_354_210469.p1_type_5prime_partial_len_108 | ---NA--- | | |
| TRINITY_DN23911_c0_g1_i1_len_350_106634.p1_type_5prime_partial_len_108 | ---NA--- | | |
| TRINITY_DN20188_c0_g1_i2_len_1866_30627.p2_type_5prime_partial_len_108 | ---NA--- | | |
| TRINITY_DN578_c0_g1_i1_len_537_131538.p1_type_5prime_partial_len_108 | XP_022218957.1leucine carboxyl methyltransferase 1 | P:GO:0006481; F:GO:0018423 | |
| TRINITY_DN6808_c0_g1_i1_len_362_248168.p1_type_5prime_partial_len_108 | ---NA--- | | |
| TRINITY_DN28626_c0_g1_i1_len_376_105377.p1_type_5prime_partial_len_108 | ---NA--- | | |

| | | | |
|---|---|---|---|
| TRINITY_DN9677_c0_g1_i1_len_809_237858.p2_type_complete_len_108 | ---NA--- | | |
| TRINITY_DN14852_c0_g1_i1_len_402_246320.p1_type_5prime_partial_len_108 | ---NA--- | | |
| TRINITY_DN6836_c0_g1_i1_len_398_246484.p1_type_3prime_partial_len_109 | ---NA--- | | |
| TRINITY_DN17069_c0_g1_i1_len_1108_195641.p2_type_complete_len_108 | ---NA--- | | |
| TRINITY_DN26315_c0_g2_i2_len_1510_193041.p1_type_complete_len_108 | KYB29108.1Disks large 1 tumor suppressor protein-like Protein | | |
| TRINITY_DN28820_c0_g1_i1_len_426_134430.p1_type_5prime_partial_len_108 | ---NA--- | | |
| TRINITY_DN28226_c0_g1_i2_len_1404_193628.p1_type_complete_len_108 | ---NA--- | | |
| TRINITY_DN18079_c0_g1_i1_len_1185_2692.p3_type_5prime_partial_len_108 | KZS18483.1Phosphopantothenoylcysteine decarboxylase | F:GO:0003824 | |
| TRINITY_DN38289_c0_g1_i1_len_335_211685.p1_type_5prime_partial_len_108 | ---NA--- | | |
| TRINITY_DN24814_c0_g1_i2_len_557_202728.p1_type_complete_len_108 | ---NA--- | | |
| TRINITY_DN9042_c0_g1_i1_len_566_274700.p1_type_5prime_partial_len_108 | ---NA--- | | |
| TRINITY_DN5301_c0_g1_i1_len_551_67990.p1_type_5prime_partial_len_108 | ---NA--- | | |
| TRINITY_DN1840_c0_g1_i2_len_864_161808.p3_type_complete_len_108 | BBC28099.1xanthine dehydrogenase | F:GO:0004854; F:GO:0004855; F:GO:0005506; F:GO:0009055; P:GO:0022900; F:GO:0051537; F:GO:0071949 | EC:1.17.3; EC:1.17.1; EC:1.17.1.4; EC:1.17.3.2 |
| TRINITY_DN21347_c0_g1_i1_len_2031_156025.p2_type_5prime_partial_len_108 | ---NA--- | | |
| TRINITY_DN11061_c0_g1_i1_len_793_198697.p1_type_complete_len_108 | ---NA--- | | |

| | | | |
|---|---|---|---|
| TRINITY_DN393_c0_g1_i1_len_369_308291.p1_type_5prime_partial_len_108 | ---NA--- | | |
| TRINITY_DN12975_c0_g1_i1_len_325_15288.p1_type_internal_len_109 | XP_018008324.1 cadherin-23-like | | |
| TRINITY_DN19157_c0_g1_i1_len_3035_228953.p3_type_complete_len_108 | ---NA--- | | |
| TRINITY_DN18447_c0_g2_i1_len_938_33843.p3_type_5prime_partial_len_108 | ---NA--- | | |
| TRINITY_DN12963_c0_g1_i1_len_534_203332.p1_type_5prime_partial_len_108 | ---NA--- | | |
| TRINITY_DN14853_c0_g1_i1_len_1473_124376.p2_type_5prime_partial_len_108 | ---NA--- | | |
| TRINITY_DN15459_c0_g1_i1_len_610_201569.p1_type_complete_len_108 | ---NA--- | | |
| TRINITY_DN7306_c0_g1_i1_len_323_139324.p1_type_internal_len_108 | ---NA--- | | |
| TRINITY_DN912_c0_g1_i1_len_321_250575.p1_type_internal_len_108 | ---NA--- | | |
| TRINITY_DN18130_c0_g1_i1_len_324_15329.p2_type_internal_len_108 | ---NA--- | | |
| TRINITY_DN21506_c0_g1_i11_len_2103_230773.p3_type_complete_len_107 | ---NA--- | | |
| TRINITY_DN4899_c0_g1_i1_len_321_250580.p1_type_internal_len_108 | ---NA--- | | |
| TRINITY_DN2937_c0_g1_i1_len_324_212494.p2_type_internal_len_108 | ---NA--- | | |
| TRINITY_DN23441_c0_g1_i1_len_321_250568.p1_type_internal_len_108 | ---NA--- | | |
| TRINITY_DN35249_c0_g1_i1_len_321_250590.p1_type_internal_len_108 | ---NA--- | | |
| TRINITY_DN41899_c0_g1_i1_len_406_207781.p1_type_5prime_partial_len_107 | ---NA--- | | |

| | | | |
|---|---|---|---|
| TRINITY_DN37557_c0_g1_i1_len_321_250592.p1_type_internal_len_108 | XP_023238631.1porphobilinogen deaminase-like | F:GO:0004418; P:GO:0033014 | EC:2.5.1.61 |
| TRINITY_DN1106_c0_g1_i1_len_321_250593.p1_type_internal_len_108 | AXY87730.1carnitine O-palmitoyltransferase 1a | F:GO:0016746 | |
| TRINITY_DN15158_c0_g1_i1_len_321_282672.p1_type_internal_len_108 | BAM76968.1dual oxidase | F:GO:0004601; F:GO:0005509; P:GO:0006979; C:GO:0016021; F:GO:0016174; F:GO:0020037; P:GO:0050665; P:GO:0055114; P:GO:0098869 | EC:1.6.3.1; EC:1.11.1.7 |
| TRINITY_DN27283_c0_g1_i1_len_420_278286.p1_type_5prime_partial_len_107 | ---NA--- | | |
| TRINITY_DN37529_c0_g1_i1_len_322_250541.p1_type_internal_len_108 | ---NA--- | | |
| TRINITY_DN16881_c0_g1_i1_len_1648_267246.p4_type_complete_len_107 | ---NA--- | | |
| TRINITY_DN32507_c0_g1_i1_len_322_139380.p1_type_internal_len_108 | ODM97566.1Protein unc-13 B | F:GO:0005509; F:GO:0005543; P:GO:0007268; F:GO:0019992; P:GO:0035556 | |
| TRINITY_DN12400_c0_g1_i1_len_322_250538.p1_type_internal_len_108 | ---NA--- | | |
| TRINITY_DN17088_c0_g1_i1_len_323_108240.p2_type_internal_len_108 | ---NA--- | | |
| TRINITY_DN7352_c0_g1_i1_len_323_108241.p1_type_internal_len_108 | ---NA--- | | |
| TRINITY_DN31119_c0_g1_i1_len_321_282662.p1_type_internal_len_108 | ---NA--- | | |
| TRINITY_DN29107_c0_g1_i1_len_321_250558.p1_type_internal_len_108 | XP_018027333.1 low-density lipoprotein receptor-related protein 2-like | F:GO:0005509 | |
| TRINITY_DN22575_c0_g1_i2_len_3606_153849.p2_type_complete_len_107 | ---NA--- | | |
| TRINITY_DN8237_c0_g1_i1_len_688_65818.p2_type_5prime_partial_len_107 | XP_016521914.1PREDICTED: uncharacterized protein LOC103131153 | | |

| | | | |
|---|---|---|---|
| TRINITY_DN33392_c0_g1_i1_len_321_250548.p1_type_internal_len_108 | ---NA--- | | |
| TRINITY_DN20626_c0_g1_i3_len_322_108292.p1_type_internal_len_108 | ---NA--- | | |
| TRINITY_DN27382_c0_g1_i1_len_324_139241.p2_type_internal_len_108 | ANH58178.1delta class glutathione S-transferase | F:GO:0016740 | |
| TRINITY_DN30771_c0_g1_i1_len_324_139224.p2_type_internal_len_108 | ---NA--- | | |
| TRINITY_DN13589_c0_g1_i1_len_322_108313.p1_type_internal_len_108 | ---NA--- | | |
| TRINITY_DN29182_c0_g1_i1_len_322_108310.p1_type_internal_len_108 | ---NA--- | | |
| TRINITY_DN12960_c0_g1_i2_len_686_65852.p1_type_5prime_partial_len_107 | ---NA--- | | |
| TRINITY_DN31624_c0_g1_i1_len_322_108325.p1_type_internal_len_108 | ---NA--- | | |
| TRINITY_DN18863_c0_g1_i1_len_362_106009.p1_type_5prime_partial_len_107 | ---NA--- | | |
| TRINITY_DN22471_c0_g1_i1_len_323_108216.p1_type_internal_len_108 | XP_011449309.1 deoxynucleoside triphosphate triphosphohydrolase SAMHD1 | F:GO:0016787 | |
| TRINITY_DN11051_c0_g1_i1_len_672_200435.p2_type_3prime_partial_len_108 | ---NA--- | | |
| TRINITY_DN26765_c0_g1_i1_len_323_139291.p1_type_internal_len_108 | ---NA--- | | |
| TRINITY_DN33108_c0_g1_i1_len_324_212499.p1_type_internal_len_108 | XP_018010243.1 tyrosine-protein kinase Abl-like | | |
| TRINITY_DN37392_c0_g1_i1_len_404_207842.p1_type_3prime_partial_len_108 | PNJ46751.1RPS20 isoform 6 | F:GO:0003723; F:GO:0003735; P:GO:0006412; C:GO:0015935 | |
| TRINITY_DN17786_c0_g1_i1_len_359_106146.p1_type_5prime_partial_len_107 | KRX83908.1hypothetical protein T4E_7988, partial | | |
| TRINITY_DN23362_c0_g1_i1_len_2371_155225.p1_type_complete_len_107 | ---NA--- | | |

| | | | |
|---|---|---|---|
| TRINITY_DN10752_c0_g1_i2_len_321_108351.p1_type_internal_len_108 | ---NA--- | | |
| TRINITY_DN23861_c0_g1_i1_len_322_108297.p1_type_internal_len_108 | XP_014006237.1 histone demethylase UTY-like | F:GO:0008168; P:GO:0032259 | |
| TRINITY_DN38874_c0_g1_i1_len_323_212558.p1_type_internal_len_108 | ---NA--- | | |
| TRINITY_DN5381_c0_g1_i1_len_324_212492.p1_type_internal_len_108 | ---NA--- | | |
| TRINITY_DN4961_c0_g1_i1_len_321_15519.p1_type_internal_len_108 | ---NA--- | | |
| TRINITY_DN1589_c0_g1_i1_len_830_96801.p3_type_5prime_partial_len_107 | ---NA--- | | |
| TRINITY_DN18030_c0_g1_i1_len_1262_158969.p1_type_complete_len_107 | ---NA--- | | |
| TRINITY_DN38447_c0_g1_i1_len_323_212541.p1_type_internal_len_108 | ---NA--- | | |
| TRINITY_DN22968_c0_g1_i1_len_2104_155815.p4_type_3prime_partial_len_108 | ---NA--- | | |
| TRINITY_DN15728_c0_g1_i2_len_632_66606.p1_type_complete_len_107 | ---NA--- | | |
| TRINITY_DN4324_c0_g1_i1_len_740_97648.p2_type_5prime_partial_len_107 | ---NA--- | | |
| TRINITY_DN4278_c0_g1_i1_len_388_208601.p1_type_complete_len_107 | ---NA--- | | |
| TRINITY_DN20556_c0_g1_i3_len_3505_264678.p1_type_complete_len_107 | ---NA--- | | |
| TRINITY_DN850_c0_g1_i1_len_388_208604.p1_type_5prime_partial_len_107 | ---NA--- | | |
| TRINITY_DN13035_c0_g1_i1_len_397_72480.p1_type_5prime_partial_len_107 | ---NA--- | | |
| TRINITY_DN26806_c0_g1_i1_len_1869_191403.p2_type_3prime_partial_len_108 | ---NA--- | | |

| | | | |
|---|---|---|---|
| TRINITY_DN16583_c1_g1_i1_len_554_131190.p1_type_5prime_partial_len_107 | XP_018019750.1PREDICTED: uncharacterized protein LOC108676208 | | |
| 2838\|_\|c5644_f2p13_2482_isoform_c5644_full_length_coverage_2_non_full_length_coverage_13_isoform_length_2482_21544 | XP_018008579.1 carnitine O-acetyltransferase-like | | |
| 2839\|_\|c1728_f5p6_2482_isoform_c1728_full_length_coverage_5_non_full_length_coverage_6_isoform_length_2482_11461 | AIT97133.1innexin 1 | C:GO:0005886; C:GO:0005921; P:GO:0006811; C:GO:0016021 | |
| 2840\|_\|c11707_f2p12_2482_isoform_c11707_full_length_coverage_2_non_full_length_coverage_12_isoform_length_2482_22077 | PIO37004.1Pyruvate kinase PKM | F:GO:0000287; F:GO:0004743; P:GO:0006096; F:GO:0016301; F:GO:0030955 | EC:2.7.1.40 |
| 2841\|_\|c3605_f3p2_2481_isoform_c3605_full_length_coverage_3_non_full_length_coverage_2_isoform_length_2481_21361 | XP_021932813.1zinc finger protein 346-like | F:GO:0046872 | |
| 2842\|_\|c3502_f2p2_2481_isoform_c3502_full_length_coverage_2_non_full_length_coverage_2_isoform_length_2481_12302 | KFM58717.1Kelch domain-containing protein 10 | | |
| 2843\|_\|c2138_f3p1_2481_isoform_c2138_full_length_coverage_3_non_full_length_coverage_1_isoform_length_2481_16211 | ---NA--- | | |
| 2844\|_\|c46915_f39p13_2481_isoform_c46915_full_length_coverage_39_non_full_length_coverage_13_isoform_length_2481_19322 | ATA58410.1mitochondrial citrate synthase | F:GO:0004108; C:GO:0005759; P:GO:0005975; P:GO:0006099; P:GO:0006101 | EC:2.3.3.1 |
| TRINITY_DN17978_c0_g1_i4_len_2764_29592.p1_type_complete_len_827 | XP_023703868.1RNA-binding protein 25-like | F:GO:0003676; F:GO:0003723; P:GO:0006397 | |
| TRINITY_DN24419_c0_g1_i1_len_3089_228894.p1_type_complete_len_827 | XP_780532.4 crossover junction endonuclease MUS81 | F:GO:0003677; F:GO:0004518; F:GO:0004519; P:GO:0090305 | |
| TRINITY_DN22087_c0_g1_i2_len_2595_229614.p1_type_5prime_partial_len_827 | XP_018024947.1 late secretory pathway protein AVL9 homolog | F:GO:0016301; P:GO:0016310 | |
| TRINITY_DN16551_c0_g1_i1_len_561_131043.p2_type_5prime_partial_len_107 | ---NA--- | | |

| | | | |
|---|---|---|---|
| TRINITY_DN29869_c0_g1_i1_len_1864_191420.p2_type_5prime_partial_len_107 | ---NA--- | | |
| TRINITY_DN38088_c0_g1_i1_len_323_212527.p1_type_internal_len_108 | ---NA--- | | |
| TRINITY_DN41049_c0_g1_i1_len_323_212531.p1_type_internal_len_108 | ---NA--- | | |
| TRINITY_DN14109_c0_g1_i1_len_368_73799.p1_type_5prime_partial_len_107 | XP_003748370.1 zinc finger protein 513-like | | |
| TRINITY_DN25696_c0_g1_i1_len_331_14917.p1_type_5prime_partial_len_107 | ---NA--- | | |
| TRINITY_DN5340_c0_g1_i1_len_1745_191888.p1_type_complete_len_107 | ---NA--- | | |
| TRINITY_DN37541_c0_g1_i1_len_323_212523.p1_type_internal_len_108 | ---NA--- | | |
| TRINITY_DN6524_c0_g1_i1_len_373_209359.p1_type_5prime_partial_len_107 | ---NA--- | | |
| TRINITY_DN18529_c0_g1_i1_len_585_130609.p2_type_complete_len_107 | ---NA--- | | |
| TRINITY_DN8707_c0_g1_i1_len_329_107869.p1_type_5prime_partial_len_107 | ---NA--- | | |
| TRINITY_DN23366_c0_g1_i1_len_928_197190.p2_type_3prime_partial_len_108 | XP_018027336.1 THO complex subunit 7 homolog | | |
| TRINITY_DN18354_c0_g1_i1_len_323_212525.p1_type_internal_len_108 | XP_018017486.1 Niemann-Pick C1 protein-like | | |
| TRINITY_DN16391_c0_g1_i1_len_321_15532.p1_type_internal_len_108 | ---NA--- | | |
| TRINITY_DN24459_c0_g1_i7_len_1964_231219.p2_type_complete_len_107 | ---NA--- | | |
| TRINITY_DN35792_c0_g1_i1_len_323_212526.p1_type_internal_len_108 | XP_021924864.1protein unc-79 homolog isoform X7 | | |
| TRINITY_DN14979_c0_g1_i1_len_569_241409.p1_type_5prime_partial_len_107 | ---NA--- | | |

| | | | |
|---|---|---|---|
| TRINITY_DN12482_c0_g1_i1_len_571_7464.p2_type_complete_len_107 | ---NA--- | | |
| TRINITY_DN23681_c0_g1_i5_len_386_208680.p1_type_5prime_partial_len_107 | ---NA--- | | |
| TRINITY_DN41017_c0_g1_i1_len_323_212540.p1_type_internal_len_108 | ---NA--- | | |
| TRINITY_DN9238_c0_g1_i1_len_323_212539.p1_type_internal_len_108 | ---NA--- | | |
| TRINITY_DN32452_c0_g1_i1_len_979_63247.p2_type_complete_len_107 | XP_018007178.1 histidine ammonia-lyase-like | | |
| TRINITY_DN17261_c1_g1_i2_len_423_245532.p1_type_complete_len_107 | ---NA--- | | |
| TRINITY_DN30139_c0_g1_i1_len_369_73735.p1_type_5prime_partial_len_107 | ---NA--- | | |
| TRINITY_DN31379_c0_g1_i1_len_324_15360.p1_type_internal_len_108 | XP_018897810.1 cadherin EGF LAG seven-pass G-type receptor 2 | F:GO:0005509; C:GO:0005886; P:GO:0007155; P:GO:0007156; C:GO:0016020 | |
| TRINITY_DN2337_c0_g1_i1_len_475_205033.p1_type_complete_len_107 | XP_018022904.1 putative glutamate synthase [NADPH] | F:GO:0005506; P:GO:0006537; F:GO:0010181; F:GO:0016040; F:GO:0050660; F:GO:0051538; P:GO:0055114 | EC:1.4.1.14 |
| TRINITY_DN37397_c0_g1_i1_len_323_212552.p3_type_internal_len_108 | XP_025160842.1Down syndrome cell adhesion molecule-like protein Dscam2 | C:GO:0016020; C:GO:0016021 | |
| TRINITY_DN4624_c0_g1_i1_len_348_106702.p1_type_5prime_partial_len_107 | ---NA--- | | |
| TRINITY_DN26693_c0_g1_i2_len_1985_190957.p4_type_5prime_partial_len_107 | ---NA--- | | |
| TRINITY_DN17628_c0_g1_i1_len_442_70818.p1_type_5prime_partial_len_107 | ---NA--- | | |
| TRINITY_DN36923_c0_g1_i1_len_323_212554.p1_type_internal_len_108 | PNF34581.1Transmembrane protein 181 | C:GO:0016020 | |
| TRINITY_DN32287_c0_g1_i1_len_324_212502.p1_type_3prime_partial_len_108 | ---NA--- | | |

| | | | |
|---|---|---|---|
| TRINITY_DN13917_c0_g1_i2_len_878_96419.p1_type_5prime_partial_len_107 | ---NA--- | | |
| TRINITY_DN6849_c0_g1_i1_len_608_201616.p1_type_5prime_partial_len_107 | ---NA--- | | |
| TRINITY_DN17822_c0_g1_i1_len_1614_267356.p3_type_complete_len_107 | ---NA--- | | |
| TRINITY_DN24863_c1_g5_i1_len_933_63540.p1_type_complete_len_107 | ---NA--- | | |
| TRINITY_DN2761_c0_g1_i1_len_321_139467.p1_type_internal_len_108 | ---NA--- | | |
| TRINITY_DN500_c0_g1_i1_len_321_139437.p1_type_internal_len_108 | ---NA--- | | |
| TRINITY_DN28774_c0_g1_i1_len_322_139393.p1_type_internal_len_108 | ---NA--- | | |
| TRINITY_DN19357_c1_g1_i1_len_391_246766.p1_type_5prime_partial_len_107 | ---NA--- | | |
| TRINITY_DN19789_c0_g1_i1_len_351_106580.p1_type_5prime_partial_len_107 | ---NA--- | | |
| TRINITY_DN25038_c0_g1_i1_len_323_15397.p1_type_internal_len_108 | XP_018025785.1 long-chain-fatty-acid--CoA ligase 4-like isoform X3 | | |
| TRINITY_DN2641_c0_g1_i1_len_589_67291.p1_type_5prime_partial_len_107 | ---NA--- | | |
| TRINITY_DN9134_c0_g1_i2_len_606_130273.p2_type_5prime_partial_len_107 | ---NA--- | | |
| TRINITY_DN25440_c0_g1_i2_len_837_198181.p1_type_complete_len_107 | ---NA--- | | |
| TRINITY_DN23375_c0_g1_i1_len_911_197377.p1_type_complete_len_107 | ---NA--- | | |
| TRINITY_DN23950_c0_g1_i1_len_321_15506.p1_type_internal_len_108 | XP_023337716.1apolipoprotein D-like | | |
| TRINITY_DN10780_c0_g1_i1_len_1059_196035.p1_type_complete_len_107 | ---NA--- | | |

| | | | |
|---|---|---|---|
| TRINITY_DN23492_c0_g1_i1_len_325_108125.p1_type_internal_len_108 | ---NA--- | | |
| TRINITY_DN6560_c0_g1_i1_len_418_71638.p1_type_5prime_partial_len_107 | ADK63101.1thrombospondin protein | F:GO:0005509; C:GO:0005576; P:GO:0006030; P:GO:0007155; F:GO:0008061 | |
| TRINITY_DN14764_c0_g1_i3_len_321_15513.p1_type_internal_len_108 | ---NA--- | | |
| TRINITY_DN32402_c0_g1_i1_len_323_212551.p1_type_internal_len_108 | XP_018017556.1 bicaudal D-related protein homolog | | |
| TRINITY_DN31481_c0_g1_i1_len_323_212545.p1_type_internal_len_108 | XP_018016366.1 serine protease 30-like | | |
| TRINITY_DN41281_c0_g1_i1_len_323_212548.p1_type_internal_len_108 | XP_018023552.1 membrane-bound transcription factor site-1 protease-like | | |
| TRINITY_DN16397_c0_g1_i1_len_699_98095.p2_type_complete_len_107 | ---NA--- | | |
| TRINITY_DN18164_c0_g1_i1_len_2800_265150.p3_type_complete_len_107 | ---NA--- | | |
| TRINITY_DN21212_c0_g1_i1_len_2167_155665.p2_type_complete_len_107 | ---NA--- | | |
| TRINITY_DN23885_c0_g1_i1_len_1018_196404.p1_type_5prime_partial_len_107 | ---NA--- | | |
| TRINITY_DN16605_c0_g1_i1_len_1069_195943.p2_type_5prime_partial_len_107 | ---NA--- | | |
| TRINITY_DN25378_c0_g1_i1_len_324_108158.p1_type_internal_len_108 | ---NA--- | | |
| TRINITY_DN40609_c0_g1_i1_len_321_212667.p1_type_internal_len_108 | ---NA--- | | |
| TRINITY_DN22196_c0_g1_i1_len_1528_157684.p3_type_complete_len_107 | ---NA--- | | |
| TRINITY_DN33626_c0_g1_i1_len_325_212419.p1_type_internal_len_108 | ---NA--- | | |
| TRINITY_DN24590_c0_g1_i1_len_446_133741.p2_type_complete_len_107 | ---NA--- | | |

| | | | |
|---|---|---|---|
| TRINITY_DN6168_c0_g1_i1_len_322_15435.p1_type_internal_len_108 | ---NA--- | | |
| TRINITY_DN39407_c0_g1_i1_len_321_212664.p1_type_internal_len_108 | ---NA--- | | |
| TRINITY_DN356_c0_g1_i1_len_324_310609.p1_type_internal_len_108 | XP_026287304.1protein bric-a-brac 2-like | | |
| TRINITY_DN25127_c0_g1_i1_len_322_15433.p1_type_internal_len_108 | ---NA--- | | |
| TRINITY_DN4963_c0_g1_i1_len_322_212645.p1_type_internal_len_108 | ---NA--- | | |
| TRINITY_DN18355_c0_g1_i1_len_322_212646.p1_type_internal_len_108 | ---NA--- | | |
| TRINITY_DN39072_c0_g1_i1_len_322_212639.p1_type_internal_len_108 | ---NA--- | | |
| TRINITY_DN14875_c0_g2_i1_len_347_281252.p1_type_5prime_partial_len_107 | ---NA--- | | |
| TRINITY_DN11394_c0_g1_i1_len_744_128513.p2_type_5prime_partial_len_107 | ---NA--- | | |
| TRINITY_DN20217_c0_g1_i2_len_668_200502.p1_type_complete_len_107 | ---NA--- | | |
| TRINITY_DN29337_c0_g1_i1_len_336_249612.p1_type_5prime_partial_len_107 | ---NA--- | | |
| TRINITY_DN35212_c0_g1_i1_len_322_212611.p1_type_internal_len_108 | ---NA--- | | |
| TRINITY_DN3703_c0_g1_i1_len_336_249625.p1_type_5prime_partial_len_107 | ---NA--- | | |
| TRINITY_DN25851_c0_g1_i2_len_2831_229236.p1_type_complete_len_107 | ---NA--- | | |
| TRINITY_DN24204_c0_g1_i1_len_899_197513.p2_type_5prime_partial_len_107 | ---NA--- | | |
| TRINITY_DN21274_c0_g1_i2_len_1738_156889.p5_type_5prime_partial_len_107 | ---NA--- | | |

| | | | |
|---|---|---|---|
| TRINITY_DN20310_c0_g2_i1_len_405_104199.p1_type_5prime_partial_len_107 | ---NA--- | | |
| TRINITY_DN26025_c0_g1_i1_len_322_15426.p3_type_internal_len_108 | ---NA--- | | |
| TRINITY_DN33175_c0_g1_i1_len_321_212714.p1_type_internal_len_108 | ---NA--- | | |
| TRINITY_DN3891_c0_g1_i1_len_372_136584.p1_type_5prime_partial_len_107 | ---NA--- | | |
| TRINITY_DN19544_c0_g1_i2_len_1710_30953.p2_type_5prime_partial_len_107 | ---NA--- | | |
| TRINITY_DN5066_c0_g1_i1_len_342_211229.p1_type_5prime_partial_len_107 | ---NA--- | | |
| TRINITY_DN25418_c0_g1_i3_len_496_204390.p1_type_3prime_partial_len_108 | ---NA--- | | |
| TRINITY_DN9234_c0_g1_i1_len_324_310613.p1_type_internal_len_108 | ---NA--- | | |
| TRINITY_DN2280_c0_g1_i1_len_408_104095.p1_type_5prime_partial_len_107 | ASC55671.1putative retinol dehydrogenase 14 | C:GO:0016020; C:GO:0016021 | |
| TRINITY_DN9001_c0_g1_i1_len_1515_157746.p3_type_5prime_partial_len_107 | EKC37743.1Zinc finger FYVE domain-containing protein 1 | F:GO:0003924; F:GO:0005525; F:GO:0046872 | EC:3.6.1.15 |
| TRINITY_DN12159_c0_g1_i1_len_322_212599.p1_type_internal_len_108 | ---NA--- | | |
| TRINITY_DN16128_c0_g1_i2_len_321_15542.p1_type_internal_len_108 | ---NA--- | | |
| TRINITY_DN29971_c0_g1_i1_len_324_310628.p1_type_internal_len_108 | XP_018023003.1 transmembrane protein 184C-like | C:GO:0016020 | |
| TRINITY_DN28245_c0_g1_i1_len_322_15468.p1_type_internal_len_108 | ---NA--- | | |
| TRINITY_DN9770_c0_g1_i1_len_503_242970.p1_type_5prime_partial_len_107 | ---NA--- | | |
| TRINITY_DN36866_c0_g1_i1_len_325_212413.p1_type_internal_len_108 | ---NA--- | | |

| | | | |
|---|---|---|---|
| TRINITY_DN32948_c0_g1_i1_len_324_76259.p1_type_internal_len_108 | ---NA--- | | |
| TRINITY_DN33791_c0_g1_i1_len_322_76389.p1_type_internal_len_108 | XP_020711663.1zinc finger protein 808-like | | |
| TRINITY_DN17033_c0_g2_i1_len_322_76395.p1_type_internal_len_108 | ---NA--- | | |
| TRINITY_DN20505_c0_g1_i2_len_322_76402.p1_type_internal_len_108 | ---NA--- | | |
| 2849\|_\|c18050_f2p0_2480_isoform_c18050_full_length_coverage_2_non_full_length_coverage_0_isoform_length_2480_22658 | ---NA--- | | |
| 2850\|_\|c18104_f2p0_2480_isoform_c18104_full_length_coverage_2_non_full_length_coverage_0_isoform_length_2480_22692 | ---NA--- | | |
| 2852\|_\|c1619_f2p25_2479_isoform_c1619_full_length_coverage_2_non_full_length_coverage_25_isoform_length_2479_25448 | AMJ52354.1cullin 1 | P:GO:0006511; C:GO:0031461; F:GO:0031625 | |
| 2853\|_\|c2189_f8p3_2479_isoform_c2189_full_length_coverage_8_non_full_length_coverage_3_isoform_length_2479_11725 | XP_025836841.1mitochondrial carrier homolog 2 | C:GO:0016020 | |
| 2855\|_\|c6297_f5p12_2479_isoform_c6297_full_length_coverage_5_non_full_length_coverage_12_isoform_length_2479_17041 | XP_018006803.1 longitudinals lacking protein, isoforms H/M/V-like isoform X39 | F:GO:0003676 | |
| 2856\|_\|c2522_f2p0_2478_isoform_c2522_full_length_coverage_2_non_full_length_coverage_0_isoform_length_2478_16479 | XP_018024396.1 cyclin-dependent kinase 8-like | F:GO:0004674; F:GO:0005524; P:GO:0006468 | EC:2.7.11 |
| 2857\|_\|c3735_f3p1_2478_isoform_c3735_full_length_coverage_3_non_full_length_coverage_1_isoform_length_2478_21375 | KYB29730.1Solute carrier family 12 member 6-like Protein | C:GO:0005887; P:GO:0006884; P:GO:0007268; F:GO:0015379; P:GO:0055064; P:GO:0055075; P:GO:0071477; P:GO:1902476; P:GO:1990573 | |

| | | | |
|---|---|---|---|
| 2860\|_\|c2474_f4p27_2478_isoform_c2474_full_length_coverage_4_non_full_length_coverage_27_isoform_length_2478_16440 | XP_018007540.1 innexin inx2-like | | |
| TRINITY_DN19862_c0_g1_i2_len_2478_189506.p1_type_internal_len_827 | PNF38743.1putative ATP-dependent RNA helicase YTHDC2 | F:GO:0000166; F:GO:0003676; F:GO:0016787 | |
| TRINITY_DN23016_c0_g1_i1_len_2748_229349.p1_type_complete_len_826 | XP_023717155.1endoribonuclease ZC3H12A-like isoform X3 | F:GO:0046872 | |
| TRINITY_DN30038_c0_g1_i1_len_321_76469.p1_type_internal_len_108 | XP_023719174.1nose resistant to fluoxetine protein 6-like | C:GO:0016020; C:GO:0016021; F:GO:0016747 | |
| TRINITY_DN12383_c0_g1_i2_len_321_76470.p1_type_internal_len_108 | XP_020283093.1alcohol dehydrogenase [NADP(+)] A-like isoform X2 | | |
| TRINITY_DN23663_c0_g1_i1_len_447_102761.p1_type_complete_len_107 | ---NA--- | | |
| TRINITY_DN27155_c0_g1_i1_len_323_76364.p1_type_internal_len_108 | ---NA--- | | |
| TRINITY_DN19937_c0_g2_i2_len_323_76362.p3_type_internal_len_108 | ---NA--- | | |
| TRINITY_DN6367_c0_g1_i1_len_322_212627.p1_type_internal_len_108 | ACU31809.1alpha2 macroglobulin isoform 3 | F:GO:0004866; C:GO:0005615; P:GO:0010951 | |
| TRINITY_DN7015_c0_g1_i1_len_323_76379.p1_type_internal_len_108 | ---NA--- | | |
| TRINITY_DN5035_c0_g1_i2_len_323_76378.p1_type_internal_len_108 | ---NA--- | | |
| TRINITY_DN8290_c0_g1_i1_len_323_76365.p1_type_internal_len_108 | ---NA--- | | |
| TRINITY_DN5799_c0_g1_i1_len_323_76315.p2_type_internal_len_108 | ---NA--- | | |
| TRINITY_DN12129_c0_g1_i1_len_325_212437.p1_type_internal_len_108 | ---NA--- | | |
| TRINITY_DN26310_c0_g1_i1_len_323_76385.p1_type_5prime_partial_len_107 | XP_022523061.1fibrocystin-L | C:GO:0016020 | |
| TRINITY_DN37529_c0_g1_i1_len_325_212439.p1_type_internal_len_108 | ---NA--- | | |

| | | | |
|---|---|---|---|
| TRINITY_DN29923_c0_g1_i2_len_3916_187634.p2_type_complete_len_107 | ---NA--- | | |
| TRINITY_DN32798_c0_g1_i1_len_324_212447.p1_type_internal_len_108 | XP_018027163.1 UDP-glucuronosyltransferase 2B14-like | | |
| TRINITY_DN23506_c0_g1_i2_len_1535_192922.p3_type_complete_len_107 | ---NA--- | | |
| TRINITY_DN11670_c0_g1_i1_len_325_212430.p1_type_internal_len_108 | ---NA--- | | |
| TRINITY_DN6724_c0_g1_i1_len_322_212619.p1_type_internal_len_108 | XP_018006960.1PREDICTED: uncharacterized protein LOC108664786 isoform X3 | | |
| TRINITY_DN25510_c2_g1_i1_len_3204_228768.p4_type_3prime_partial_len_108 | ---NA--- | | |
| TRINITY_DN41842_c0_g1_i1_len_325_212426.p1_type_internal_len_108 | KDR23219.1Vacuolar protein sorting-associated protein 13A | | |
| TRINITY_DN15217_c0_g1_i1_len_322_212632.p1_type_internal_len_108 | ---NA--- | | |
| TRINITY_DN14389_c0_g1_i1_len_2370_230069.p3_type_3prime_partial_len_108 | XP_018018630.1 GMP reductase 2-like | F:GO:0003920; P:GO:0006144; P:GO:0006163; F:GO:0046872; P:GO:0055114; C:GO:1902560 | EC:1.7.1.7 |
| TRINITY_DN35002_c0_g1_i1_len_321_76484.p1_type_internal_len_108 | XP_005098392.1 proprotein convertase subtilisin/kexin type 7-like | | |
| TRINITY_DN25375_c0_g1_i1_len_2400_229998.p3_type_5prime_partial_len_107 | ---NA--- | | |
| TRINITY_DN27079_c0_g1_i1_len_323_250423.p1_type_internal_len_108 | ---NA--- | | |
| TRINITY_DN12749_c0_g1_i1_len_323_250425.p1_type_internal_len_108 | ---NA--- | | |
| TRINITY_DN33423_c0_g1_i1_len_323_250426.p1_type_internal_len_108 | XP_019756257.1 ankyrin repeat and IBR domain-containing protein 1-like isoform X1 | | |
| TRINITY_DN10166_c0_g1_i1_len_323_250427.p1_type_internal_len_108 | ---NA--- | | |

| | | | |
|---|---|---|---|
| TRINITY_DN36478_c0_g1_i1_len_323_250433.p1_type_internal_len_108 | ---NA--- | | |
| TRINITY_DN8052_c0_g1_i1_len_324_282496.p1_type_internal_len_108 | XP_006520819.1 tubulin alpha-1B chain isoform X1 | F:GO:0003924; F:GO:0005200; F:GO:0005525; C:GO:0005737; C:GO:0005874; P:GO:0007010; P:GO:0007017 | EC:3.6.1.15 |
| TRINITY_DN26704_c0_g1_i1_len_323_250422.p1_type_internal_len_108 | XP_018012118.1 kinesin-like protein KIF21B | F:GO:0003777; F:GO:0005524; C:GO:0005871; C:GO:0005874; P:GO:0007018; F:GO:0008017; F:GO:0016887 | EC:3.6.1.3; EC:3.6.1.15 |
| TRINITY_DN7828_c0_g1_i1_len_614_99221.p1_type_5prime_partial_len_107 | ---NA--- | | |
| TRINITY_DN32919_c0_g1_i1_len_323_250450.p1_type_internal_len_108 | ---NA--- | | |
| TRINITY_DN37946_c0_g1_i1_len_323_250437.p1_type_internal_len_108 | XP_021937197.1rab-like protein 6 isoform X2 | F:GO:0003924; F:GO:0005525 | EC:3.6.1.15 |
| TRINITY_DN27390_c0_g1_i1_len_324_250394.p1_type_internal_len_108 | ---NA--- | | |
| TRINITY_DN32263_c0_g1_i1_len_323_212569.p1_type_internal_len_108 | ---NA--- | | |
| TRINITY_DN7574_c0_g1_i1_len_325_212377.p1_type_internal_len_108 | ---NA--- | | |
| TRINITY_DN7465_c0_g1_i1_len_324_250364.p1_type_3prime_partial_len_108 | ---NA--- | | |
| TRINITY_DN36460_c0_g1_i1_len_323_250405.p1_type_internal_len_108 | ---NA--- | | |
| TRINITY_DN36895_c0_g1_i1_len_323_250409.p1_type_internal_len_108 | ---NA--- | | |
| TRINITY_DN31933_c0_g1_i1_len_324_250398.p1_type_internal_len_108 | ---NA--- | | |
| TRINITY_DN2581_c0_g1_i1_len_324_250402.p1_type_internal_len_108 | XP_026280022.1dystrophin, isoforms A/C/F/G/H-like | | |

| | | | |
|---|---|---|---|
| TRINITY_DN17341_c1_g1_i2_len_322_250516.p1_type_internal_len_108 | ---NA--- | | |
| TRINITY_DN37599_c0_g1_i1_len_322_250504.p1_type_internal_len_108 | KDR13847.1E3 ubiquitin-protein ligase UBR2 | F:GO:0008270; P:GO:0016567; F:GO:0016874; F:GO:0061630; P:GO:0071596 | |
| TRINITY_DN15668_c0_g1_i1_len_323_282586.p2_type_internal_len_108 | ---NA--- | | |
| TRINITY_DN30214_c0_g1_i1_len_322_250509.p1_type_internal_len_108 | XP_018006424.1 sorting nexin-13-like | | |
| TRINITY_DN33632_c0_g1_i1_len_323_212563.p1_type_internal_len_108 | ---NA--- | | |
| TRINITY_DN31624_c0_g1_i1_len_322_250510.p1_type_internal_len_108 | KOB72609.1Mixed-lineage leukemia protein, mll | F:GO:0016740 | |
| TRINITY_DN30825_c0_g1_i1_len_322_282592.p1_type_internal_len_108 | ---NA--- | | |
| TRINITY_DN42_c0_g1_i1_len_322_250530.p1_type_internal_len_108 | KXJ23963.1Aminopeptidase O | | |
| TRINITY_DN33309_c0_g1_i1_len_322_250533.p1_type_internal_len_108 | AAT77812.1calpain T | F:GO:0004198; P:GO:0006508 | EC:3.4.22 |
| TRINITY_DN26877_c0_g1_i1_len_322_282619.p1_type_internal_len_108 | ---NA--- | | |
| TRINITY_DN30988_c0_g1_i1_len_322_250517.p1_type_internal_len_108 | ---NA--- | | |
| TRINITY_DN30496_c0_g1_i1_len_323_15405.p1_type_internal_len_108 | ---NA--- | | |
| TRINITY_DN18607_c0_g1_i3_len_2115_230742.p2_type_3prime_partial_len_108 | ---NA--- | | |
| TRINITY_DN13579_c0_g1_i1_len_349_210802.p1_type_5prime_partial_len_107 | ---NA--- | | |
| TRINITY_DN33228_c0_g1_i1_len_323_250459.p1_type_internal_len_108 | XP_018026245.1 solute carrier family 35 member F2-like | | |
| TRINITY_DN4559_c0_g1_i1_len_323_282546.p1_type_internal_len_108 | ---NA--- | | |

| | | | |
|---|---|---|---|
| TRINITY_DN32281_c0_g1_i1_len_322_250474.p1_type_internal_len_108 | ---NA--- | | |
| TRINITY_DN17693_c0_g1_i1_len_525_242395.p1_type_5prime_partial_len_107 | ---NA--- | | |
| TRINITY_DN33201_c0_g1_i1_len_323_250452.p1_type_internal_len_108 | PNF19820.1EFR3-like protein cmp44E | P:GO:0072659 | |
| TRINITY_DN7817_c0_g1_i2_len_323_282566.p1_type_internal_len_108 | ---NA--- | | |
| TRINITY_DN7817_c0_g1_i2_len_323_282566.p2_type_internal_len_108 | ---NA--- | | |
| TRINITY_DN15012_c1_g1_i1_len_322_250499.p1_type_internal_len_108 | ---NA--- | | |
| TRINITY_DN31422_c0_g1_i1_len_322_250500.p1_type_5prime_partial_len_107 | ---NA--- | | |
| TRINITY_DN33787_c0_g1_i1_len_322_250481.p1_type_internal_len_108 | ---NA--- | | |
| TRINITY_DN9925_c0_g1_i1_len_323_282554.p1_type_internal_len_108 | ---NA--- | | |
| TRINITY_DN31484_c0_g1_i1_len_322_250487.p1_type_internal_len_108 | ---NA--- | | |
| TRINITY_DN31323_c0_g1_i1_len_321_212730.p1_type_internal_len_108 | CAP64302.2putative LOC540076 protein | F:GO:0003777; F:GO:0005524; P:GO:0007018; F:GO:0008017 | EC:3.6.1.15 |
| TRINITY_DN13606_c0_g1_i1_len_324_212472.p1_type_3prime_partial_len_108 | ---NA--- | | |
| TRINITY_DN23857_c0_g1_i2_len_2254_190086.p2_type_5prime_partial_len_107 | ---NA--- | | |
| TRINITY_DN34568_c0_g1_i1_len_322_212597.p1_type_internal_len_108 | ---NA--- | | |
| TRINITY_DN23637_c0_g1_i1_len_780_128201.p2_type_5prime_partial_len_107 | ---NA--- | | |
| TRINITY_DN42036_c0_g1_i1_len_321_212725.p1_type_internal_len_108 | ---NA--- | | |

| | | | |
|---|---|---|---|
| TRINITY_DN14934_c0_g1_i1_len_323_310673.p1_type_internal_len_108 | ---NA--- | | |
| TRINITY_DN13073_c0_g1_i1_len_651_129589.p2_type_5prime_partial_len_107 | ---NA--- | | |
| TRINITY_DN27100_c0_g1_i1_len_503_69025.p1_type_5prime_partial_len_107 | ---NA--- | | |
| TRINITY_DN16493_c0_g1_i1_len_649_129616.p1_type_complete_len_107 | ---NA--- | | |
| TRINITY_DN5977_c0_g1_i1_len_503_69043.p1_type_5prime_partial_len_107 | ---NA--- | | |
| TRINITY_DN10793_c0_g1_i1_len_324_15315.p1_type_5prime_partial_len_107 | ---NA--- | | |
| TRINITY_DN19808_c0_g1_i1_len_1350_31952.p2_type_5prime_partial_len_107 | ---NA--- | | |
| TRINITY_DN11804_c0_g1_i1_len_322_212598.p1_type_internal_len_108 | ---NA--- | | |
| TRINITY_DN13688_c0_g1_i1_len_543_100409.p1_type_5prime_partial_len_107 | ---NA--- | | |
| TRINITY_DN16837_c0_g2_i1_len_509_242804.p2_type_complete_len_107 | ABC33915.1kazal-type proteinase inhibitor | | |
| TRINITY_DN6657_c0_g1_i1_len_321_212723.p1_type_internal_len_108 | XP_026685263.1oxysterol-binding protein-related protein 6-like | | |
| TRINITY_DN21090_c0_g1_i1_len_678_5978.p1_type_5prime_partial_len_107 | ---NA--- | | |
| TRINITY_DN29929_c0_g1_i1_len_322_310725.p1_type_internal_len_108 | ---NA--- | | |
| TRINITY_DN22452_c0_g1_i2_len_2160_190364.p4_type_complete_len_107 | XP_018016928.1 gamma-butyrobetaine dioxygenase-like | | |
| TRINITY_DN13904_c0_g1_i1_len_324_212476.p1_type_internal_len_108 | ---NA--- | | |
| TRINITY_DN6805_c0_g1_i1_len_321_15534.p1_type_internal_len_108 | ---NA--- | | |

<table>
<tr><td>TRINITY_DN10476_c0_g1_i1_len_325_250335.p1_type_internal_len_108</td><td>---NA---</td><td></td><td></td></tr>
<tr><td>TRINITY_DN21130_c0_g1_i3_len_2198_230518.p2_type_5prime_partial_len_107</td><td>---NA---</td><td></td><td></td></tr>
<tr><td>TRINITY_DN6449_c0_g1_i1_len_325_250327.p1_type_internal_len_108</td><td>AKK75092.1tet methylcytosine dioxygenase</td><td>P:GO:0006211; P:GO:0055114; F:GO:0070579; P:GO:0080111</td><td>EC:1.14.11</td></tr>
<tr><td>TRINITY_DN37778_c0_g1_i1_len_1106_195655.p2_type_5prime_partial_len_107</td><td>---NA---</td><td></td><td></td></tr>
<tr><td>TRINITY_DN11868_c0_g1_i1_len_323_310692.p1_type_internal_len_108</td><td>---NA---</td><td></td><td></td></tr>
<tr><td>TRINITY_DN2671_c0_g1_i1_len_460_70261.p1_type_5prime_partial_len_107</td><td>---NA---</td><td></td><td></td></tr>
<tr><td>TRINITY_DN36214_c0_g1_i1_len_325_250282.p1_type_internal_len_108</td><td>---NA---</td><td></td><td></td></tr>
<tr><td>TRINITY_DN3809_c0_g1_i1_len_322_212593.p1_type_internal_len_108</td><td>---NA---</td><td></td><td></td></tr>
<tr><td>TRINITY_DN33665_c0_g1_i1_len_375_171280.p1_type_5prime_partial_len_107</td><td>---NA---</td><td></td><td></td></tr>
<tr><td>TRINITY_DN17182_c0_g1_i1_len_626_164573.p1_type_5prime_partial_len_107</td><td>---NA---</td><td></td><td></td></tr>
<tr><td>TRINITY_DN29053_c0_g1_i1_len_2569_189293.p1_type_5prime_partial_len_826</td><td>XP_018024302.1 PR domain zinc finger protein 16-like</td><td></td><td></td></tr>
<tr><td>TRINITY_DN17770_c0_g1_i1_len_2479_189501.p1_type_internal_len_827</td><td>XP_018018437.1PREDICTED: uncharacterized protein LOC108674963</td><td></td><td></td></tr>
<tr><td>2866|_|c1623_f2p17_2477_isoform_c1623_full_length_coverage_2_non_full_length_coverage_17_isoform_length_2477_25452</td><td>---NA---</td><td></td><td></td></tr>
<tr><td>2867|_|c2021_f7p3_2476_isoform_c2021_full_length_coverage_7_non_full_length_coverage_3_isoform_length_2476_20648</td><td>XP_018015603.1 nucleoporin GLE1-like</td><td></td><td></td></tr>
<tr><td>2868|_|c2200_f6p3_2475_isoform_c2200_full_length_coverage_6_non_full_length_coverage_3_isoform_length_2475_20775</td><td>XP_018008337.1 puff-specific protein Bx42-like</td><td></td><td></td></tr>
</table>

| | | | |
|---|---|---|---|
| 2869\|_\|c25690_f3p2_2475_isoform_c25690_full_length_coverage_3_non_full_length_coverage_2_isoform_length_2475_18369 | XP_019629751.1 transmembrane protein 231-like | | |
| 2870\|_\|c2582_f3p6_2475_isoform_c2582_full_length_coverage_3_non_full_length_coverage_6_isoform_length_2475_11988 | XP_018006803.1 longitudinals lacking protein, isoforms H/M/V-like isoform X39 | F:GO:0003676 | |
| TRINITY_DN16538_c0_g1_i1_len_2478_229834.p1_type_internal_len_826 | ---NA--- | | |
| TRINITY_DN17033_c0_g1_i1_len_2962_154370.p1_type_complete_len_825 | XP_013421286.1AP-4 complex subunit epsilon-1-like | C:GO:0005794; P:GO:0006886; P:GO:0015031; C:GO:0016020; P:GO:0016192; C:GO:0030117; C:GO:0030124 | |
| 2873\|_\|c42706_f2p1_2474_isoform_c42706_full_length_coverage_2_non_full_length_coverage_1_isoform_length_2474_14784 | XP_014367166.1 serine protease nudel | F:GO:0004252; F:GO:0005044; P:GO:0006508; P:GO:0006898; P:GO:0007306; P:GO:0007310; P:GO:0007313; F:GO:0008233; F:GO:0008236; C:GO:0016020; F:GO:0016787 | |
| TRINITY_DN9110_c0_g1_i2_len_321_45042.p1_type_internal_len_108 | ---NA--- | | |
| TRINITY_DN23315_c0_g1_i1_len_1094_125975.p1_type_complete_len_107 | ---NA--- | | |
| TRINITY_DN31434_c0_g1_i1_len_323_174019.p2_type_internal_len_108 | ---NA--- | | |
| TRINITY_DN34806_c0_g1_i1_len_323_174024.p1_type_internal_len_108 | ---NA--- | | |
| TRINITY_DN23256_c1_g2_i1_len_322_174064.p2_type_internal_len_108 | ---NA--- | | |
| TRINITY_DN5679_c0_g1_i1_len_322_174060.p3_type_internal_len_108 | ---NA--- | | |
| TRINITY_DN8505_c0_g1_i1_len_322_45038.p1_type_internal_len_108 | ---NA--- | | |

| | | | |
|---|---|---|---|
| TRINITY_DN13738_c0_g1_i1_len_619_240478.p1_type_5prime_partial_len_107 | ---NA--- | | |
| TRINITY_DN25197_c0_g1_i1_len_322_174067.p1_type_internal_len_108 | XP_015927887.1F-box/LRR-repeat protein 2 | | |
| TRINITY_DN33314_c0_g1_i1_len_322_174043.p1_type_internal_len_108 | ---NA--- | | |
| TRINITY_DN27035_c0_g1_i1_len_1354_193930.p2_type_complete_len_107 | ---NA--- | | |
| TRINITY_DN15174_c0_g1_i1_len_580_241155.p1_type_5prime_partial_len_107 | XP_013142182.1 peripheral plasma membrane protein CASK-like | | |
| TRINITY_DN9430_c0_g1_i1_len_712_199808.p1_type_3prime_partial_len_108 | ---NA--- | | |
| TRINITY_DN33828_c0_g1_i1_len_324_173933.p1_type_internal_len_108 | XP_023713630.1sorting nexin-13-like isoform X2 | | |
| TRINITY_DN20405_c0_g1_i1_len_716_199747.p2_type_3prime_partial_len_108 | ---NA--- | | |
| TRINITY_DN4010_c0_g1_i1_len_324_173941.p1_type_internal_len_108 | XP_023727167.1tumor protein p63-regulated gene 1-like protein isoform X1 | | |
| TRINITY_DN631_c0_g1_i1_len_323_173999.p2_type_internal_len_108 | ---NA--- | | |
| TRINITY_DN8592_c0_g1_i1_len_784_162570.p1_type_5prime_partial_len_107 | ---NA--- | | |
| TRINITY_DN5383_c0_g1_i1_len_323_173996.p2_type_internal_len_108 | ---NA--- | | |
| TRINITY_DN31131_c3_g2_i4_len_431_206702.p1_type_5prime_partial_len_107 | ---NA--- | | |
| TRINITY_DN27961_c3_g1_i1_len_713_199801.p2_type_5prime_partial_len_107 | ---NA--- | | |
| TRINITY_DN12867_c0_g1_i1_len_323_174003.p2_type_internal_len_108 | ---NA--- | | |
| TRINITY_DN28955_c0_g1_i1_len_323_174000.p1_type_internal_len_108 | ---NA--- | | |

| | | | |
|---|---|---|---|
| TRINITY_DN30289_c0_g1_i1_len_323_173988.p1_type_internal_len_108 | OXA59962.1Multidrug resistance-associated protein 4 | F:GO:0000166; C:GO:0016020; F:GO:0042626; P:GO:0055085 | EC:3.6.1.3; EC:3.6.1.15 |
| TRINITY_DN9191_c0_g1_i1_len_653_164146.p1_type_complete_len_107 | ---NA--- | | |
| TRINITY_DN18417_c0_g1_i2_len_1097_235391.p2_type_5prime_partial_len_107 | ---NA--- | | |
| TRINITY_DN11261_c0_g1_i1_len_562_202601.p1_type_5prime_partial_len_107 | ---NA--- | | |
| TRINITY_DN34269_c0_g1_i1_len_323_173995.p2_type_internal_len_108 | ---NA--- | | |
| TRINITY_DN14337_c0_g1_i2_len_472_9531.p2_type_5prime_partial_len_107 | ---NA--- | | |
| TRINITY_DN4617_c0_g1_i1_len_453_305323.p1_type_5prime_partial_len_107 | ---NA--- | | |
| TRINITY_DN16345_c0_g1_i2_len_1121_159827.p2_type_complete_len_107 | ---NA--- | | |
| TRINITY_DN17500_c0_g1_i1_len_667_239693.p1_type_5prime_partial_len_107 | ---NA--- | | |
| TRINITY_DN30575_c0_g1_i1_len_325_44814.p1_type_internal_len_108 | ---NA--- | | |
| TRINITY_DN16138_c0_g1_i1_len_833_162084.p2_type_complete_len_107 | ---NA--- | | |
| TRINITY_DN22063_c0_g1_i1_len_468_276837.p1_type_5prime_partial_len_107 | ---NA--- | | |
| TRINITY_DN23682_c0_g1_i1_len_583_165311.p2_type_5prime_partial_len_107 | ---NA--- | | |
| TRINITY_DN476_c0_g1_i1_len_322_44991.p1_type_internal_len_108 | ---NA--- | | |
| TRINITY_DN4553_c0_g1_i1_len_1140_235113.p4_type_complete_len_107 | ---NA--- | | |
| TRINITY_DN22749_c0_g1_i1_len_1723_232059.p2_type_5prime_partial_len_107 | ---NA--- | | |

| | | | |
|---|---|---|---|
| TRINITY_DN9308_c0_g1_i1_len_413_207446.p1_type_5prime_partial_len_107 | ---NA--- | | |
| TRINITY_DN15933_c0_g1_i2_len_437_10504.p1_type_5prime_partial_len_107 | ---NA--- | | |
| TRINITY_DN20306_c1_g1_i2_len_949_33769.p1_type_complete_len_107 | ---NA--- | | |
| TRINITY_DN25623_c0_g1_i1_len_1256_194542.p3_type_complete_len_107 | ---NA--- | | |
| TRINITY_DN22272_c0_g1_i1_len_355_210409.p1_type_5prime_partial_len_107 | ---NA--- | | |
| TRINITY_DN14086_c0_g1_i1_len_803_162387.p1_type_5prime_partial_len_107 | ---NA--- | | |
| TRINITY_DN27275_c0_g1_i1_len_322_45014.p1_type_internal_len_108 | ---NA--- | | |
| TRINITY_DN4510_c0_g1_i1_len_547_166013.p1_type_5prime_partial_len_107 | ---NA--- | | |
| TRINITY_DN15797_c0_g1_i1_len_401_306977.p1_type_5prime_partial_len_107 | ---NA--- | | |
| TRINITY_DN4378_c0_g1_i1_len_321_174131.p1_type_internal_len_108 | PIK48227.1hypothetical protein BSL78_14913, partial | | |
| TRINITY_DN7532_c0_g1_i1_len_322_45012.p1_type_internal_len_108 | ---NA--- | | |
| TRINITY_DN16487_c0_g1_i1_len_528_203462.p1_type_complete_len_107 | ---NA--- | | |
| TRINITY_DN24436_c0_g1_i1_len_324_44892.p1_type_internal_len_108 | ---NA--- | | |
| TRINITY_DN27013_c0_g1_i1_len_6450_186865.p4_type_5prime_partial_len_107 | KRT79554.1HEAT domain-containing protein | P:GO:0006886; P:GO:0016192; C:GO:0030117 | |
| TRINITY_DN2395_c0_g1_i1_len_335_211701.p1_type_5prime_partial_len_107 | ---NA--- | | |
| TRINITY_DN22302_c0_g1_i1_len_323_44943.p1_type_internal_len_108 | XP_023327029.1tyrosine-protein phosphatase 10D-like | | |

| | | | |
|---|---|---|---|
| TRINITY_DN18055_c0_g1_i1_len_412_11318.p2_type_5prime_partial_len_107 | ---NA--- | | |
| TRINITY_DN20908_c0_g1_i1_len_1766_92611.p1_type_complete_len_107 | ---NA--- | | |
| TRINITY_DN12660_c0_g1_i1_len_330_44575.p1_type_5prime_partial_len_107 | ---NA--- | | |
| TRINITY_DN35860_c0_g1_i1_len_428_206805.p1_type_5prime_partial_len_107 | ---NA--- | | |
| TRINITY_DN18601_c0_g1_i9_len_2159_91881.p2_type_5prime_partial_len_107 | ---NA--- | | |
| TRINITY_DN20760_c0_g1_i1_len_324_44897.p1_type_internal_len_108 | ---NA--- | | |
| TRINITY_DN2195_c0_g1_i1_len_324_44899.p1_type_internal_len_108 | ---NA--- | | |
| TRINITY_DN24395_c0_g1_i1_len_1644_60213.p2_type_3prime_partial_len_108 | ---NA--- | | |
| TRINITY_DN21231_c0_g1_i2_len_1173_234875.p1_type_complete_len_107 | ---NA--- | | |
| TRINITY_DN26361_c1_g1_i1_len_1116_235267.p2_type_3prime_partial_len_108 | ---NA--- | | |
| TRINITY_DN10472_c0_g1_i1_len_324_44896.p1_type_internal_len_108 | ---NA--- | | |
| TRINITY_DN19155_c0_g1_i1_len_1844_231588.p1_type_complete_len_107 | ---NA--- | | |
| TRINITY_DN11498_c0_g1_i1_len_332_211865.p1_type_5prime_partial_len_107 | ---NA--- | | |
| TRINITY_DN14510_c0_g1_i2_len_446_168569.p1_type_5prime_partial_len_107 | ---NA--- | | |
| TRINITY_DN20007_c0_g1_i6_len_1334_268433.p2_type_complete_len_107 | XP_012683495.1 ADP-sugar pyrophosphatase | | |
| TRINITY_DN4743_c0_g1_i2_len_323_44929.p1_type_internal_len_108 | ---NA--- | | |

| | | | |
|---|---|---|---|
| TRINITY_DN22296_c0_g1_i2_len_1792_1074.p2_type_complete_len_107 | ---NA--- | | |
| TRINITY_DN26975_c0_g1_i1_len_357_210272.p1_type_5prime_partial_len_107 | ---NA--- | | |
| TRINITY_DN39378_c0_g1_i1_len_542_203101.p2_type_5prime_partial_len_107 | ---NA--- | | |
| TRINITY_DN14550_c0_g1_i1_len_1571_1503.p2_type_5prime_partial_len_107 | ---NA--- | | |
| TRINITY_DN2238_c0_g1_i1_len_324_44886.p1_type_internal_len_108 | ---NA--- | | |
| TRINITY_DN9483_c0_g1_i2_len_323_44945.p1_type_internal_len_108 | ---NA--- | | |
| TRINITY_DN22549_c0_g1_i1_len_321_108383.p1_type_internal_len_107 | ---NA--- | | |
| TRINITY_DN27618_c0_g1_i1_len_321_108362.p2_type_internal_len_107 | XP_018028145.1 fibrillin-1-like isoform X3 | | |
| TRINITY_DN24837_c0_g1_i1_len_319_174219.p1_type_internal_len_107 | ---NA--- | | |
| TRINITY_DN8096_c0_g1_i1_len_321_108357.p1_type_internal_len_107 | XP_023712070.1phosphoinositide 3-kinase regulatory subunit 4 | | |
| TRINITY_DN1447_c0_g1_i1_len_321_310759.p1_type_internal_len_107 | ---NA--- | | |
| TRINITY_DN25074_c0_g1_i1_len_2139_190432.p6_type_complete_len_106 | ---NA--- | | |
| TRINITY_DN13749_c0_g1_i2_len_320_212751.p1_type_internal_len_107 | ---NA--- | | |
| TRINITY_DN24330_c0_g1_i1_len_320_174212.p1_type_internal_len_107 | XP_023715455.1small G protein signaling modulator 2-like isoform X1 | | |
| TRINITY_DN3043_c0_g1_i1_len_322_250492.p1_type_internal_len_107 | XP_019637948.1 zinc finger protein OZF-like | F:GO:0003676 | |
| TRINITY_DN25334_c0_g1_i1_len_319_174217.p1_type_internal_len_107 | XP_018028132.1 protein ECT2-like | | |

| | | | |
|---|---|---|---|
| TRINITY_DN34542_c0_g1_i1_len_319_174218.p1_type_internal_len_107 | ---NA--- | | |
| TRINITY_DN12799_c0_g1_i1_len_319_108491.p3_type_internal_len_107 | ---NA--- | | |
| TRINITY_DN31676_c0_g1_i1_len_319_108511.p1_type_internal_len_107 | ---NA--- | | |
| TRINITY_DN17847_c0_g1_i3_len_1028_33347.p2_type_complete_len_106 | ---NA--- | | |
| TRINITY_DN36366_c0_g1_i1_len_320_212757.p2_type_internal_len_107 | ---NA--- | | |
| TRINITY_DN15413_c0_g1_i1_len_320_108463.p1_type_internal_len_107 | ---NA--- | | |
| TRINITY_DN32477_c0_g1_i1_len_320_108464.p1_type_internal_len_107 | XP_018026020.1PREDICTED: uncharacterized protein C18orf8 homolog isoform X2 | P:GO:0010506; C:GO:0035658 | |
| TRINITY_DN3863_c0_g1_i2_len_486_204675.p1_type_5prime_partial_len_106 | XP_013421780.1protein SON-like | F:GO:0003676; P:GO:0050794 | |
| TRINITY_DN15544_c0_g1_i1_len_564_202553.p1_type_3prime_partial_len_107 | ---NA--- | | |
| TRINITY_DN22564_c0_g1_i1_len_723_199677.p1_type_complete_len_106 | ---NA--- | | |
| TRINITY_DN14612_c0_g1_i1_len_779_34963.p2_type_5prime_partial_len_106 | ---NA--- | | |
| TRINITY_DN23702_c0_g1_i1_len_2105_190537.p3_type_complete_len_106 | ---NA--- | | |
| TRINITY_DN26099_c0_g1_i1_len_319_108536.p1_type_internal_len_107 | ---NA--- | | |
| TRINITY_DN25559_c0_g1_i1_len_5554_227673.p1_type_complete_len_1437 | KZS14260.1VWFA and cache domain-containing protein 1 | C:GO:0016020; C:GO:0016021 | |
| TRINITY_DN31016_c0_g1_i1_len_4360_187381.p1_type_5prime_partial_len_1437 | XP_019627525.1 thyroid adenoma-associated protein homolog isoform X1 | | |
| TRINITY_DN10706_c0_g1_i1_len_6501_186856.p1_type_5prime_partial_len_1436 | XP_015925856.1transmembrane protein 131 isoform X1 | | |

| | | | |
|---|---|---|---|
| TRINITY_DN24748_c0_g1_i2_len_5065_227768.p1_type_5prime_partial_len_1434 | XP_018017948.1 actin-binding protein anillin-like | | |
| TRINITY_DN18034_c0_g1_i1_len_4344_90860.p1_type_5prime_partial_len_1432 | XP_023930625.1Fanconi anemia group A protein homolog | P:GO:0036297; C:GO:0043240 | |
| TRINITY_DN26136_c0_g1_i2_len_6706_227530.p1_type_5prime_partial_len_1430 | PNF40248.1hypothetical protein B7P43_G07072 | P:GO:0007165 | |
| TRINITY_DN21808_c0_g1_i1_len_4691_24.p1_type_5prime_partial_len_1428 | XP_018022442.1 splicing factor, arginine/serine-rich 15-like isoform X2 | | |
| TRINITY_DN26217_c0_g1_i1_len_5400_227711.p1_type_5prime_partial_len_1427 | XP_024868212.1baculoviral IAP repeat-containing protein 6 isoform X3 | | |
| TRINITY_DN30732_c0_g1_i1_len_4502_187313.p1_type_5prime_partial_len_1426 | KDR23845.1Nuclear pore complex protein Nup160-like protein | | |
| TRINITY_DN25642_c0_g1_i1_len_4785_187204.p1_type_complete_len_1418 | XP_018019260.1 dynactin subunit 1-like isoform X2 | | |
| 2875\|_\|c18117_f2p2_2474_isoform_c18117_full_length_coverage_2_non_full_length_coverage_2_isoform_length_2474_17963 | XP_018020908.1 protein N-terminal asparagine amidohydrolase-like isoform X1 | F:GO:0008418 | |
| 2876\|_\|c9494_f2p0_2474_isoform_c9494_full_length_coverage_2_non_full_length_coverage_0_isoform_length_2474_17286 | XP_018026676.1 cytochrome b reductase 1-like isoform X4 | | |
| 2877\|_\|c18021_f2p0_2474_isoform_c18021_full_length_coverage_2_non_full_length_coverage_0_isoform_length_2474_17900 | XP_018009310.1PREDICTED: uncharacterized protein LOC108666881 | | |
| 2879\|_\|c1833_f11p8_2474_isoform_c1833_full_length_coverage_11_non_full_length_coverage_8_isoform_length_2474_16026 | XP_018021907.1 V-type proton ATPase 16 kDa proteolipid subunit | C:GO:0005774; F:GO:0015078; P:GO:0015991; C:GO:0016021; C:GO:0033179 | |
| 2880\|_\|c8115_f2p3_2473_isoform_c8115_full_length_coverage_2_non_full_length_coverage_3_isoform_length_2473_21748 | EEB11118.1Paired AMPhipathic helix protein Sin3A, putative | F:GO:0003714; C:GO:0005634; P:GO:0006355; C:GO:0016580; P:GO:1903507 | |
| 2881\|_\|c1263_f4p18_2473_isoform_c1263_full_length_coverage_4_non_full_length_coverage_18_isoform_length_2473_27715 | ---NA--- | | |

| | | | |
|---|---|---|---|
| TRINITY_DN24927_c0_g1_i2_len_2685_58401.p1_type_5prime_partial_len_824 | ---NA--- | | |
| TRINITY_DN28688_c0_g1_i2_len_2474_189524.p1_type_internal_len_825 | ---NA--- | | |
| TRINITY_DN21441_c0_g1_i2_len_2718_91321.p1_type_5prime_partial_len_824 | XP_016912892.1 dyslexia-associated protein KIAA0319-like protein | | |
| TRINITY_DN30595_c0_g1_i2_len_4282_187430.p1_type_complete_len_824 | XP_010146621.1 probable E3 ubiquitin-protein ligase DTX2 | P:GO:0007219; P:GO:0016567; F:GO:0016874; F:GO:0046872 | |
| TRINITY_DN15922_c0_g1_i1_len_1697_232142.p2_type_5prime_partial_len_106 | ---NA--- | | |
| TRINITY_DN5795_c0_g1_i1_len_526_275460.p1_type_complete_len_106 | ---NA--- | | |
| TRINITY_DN17756_c0_g1_i1_len_1703_232128.p2_type_5prime_partial_len_106 | ---NA--- | | |
| TRINITY_DN25417_c0_g1_i1_len_320_108424.p1_type_internal_len_107 | EDO46894.1predicted protein | C:GO:0005774; F:GO:0015184; P:GO:0015811 | |
| TRINITY_DN3008_c0_g1_i1_len_943_161137.p1_type_5prime_partial_len_106 | ---NA--- | | |
| TRINITY_DN20461_c0_g1_i1_len_565_202542.p2_type_complete_len_106 | ---NA--- | | |
| TRINITY_DN27991_c0_g1_i1_len_319_174260.p1_type_internal_len_107 | ---NA--- | | |
| TRINITY_DN12002_c0_g1_i1_len_728_65346.p2_type_5prime_partial_len_106 | ---NA--- | | |
| TRINITY_DN7291_c0_g1_i1_len_816_4728.p1_type_complete_len_106 | XP_026725262.1putative ATP synthase subunit f, mitochondrial | C:GO:0016021 | |
| TRINITY_DN34617_c0_g1_i1_len_319_174264.p1_type_internal_len_107 | ---NA--- | | |
| TRINITY_DN651_c0_g1_i1_len_320_108434.p1_type_internal_len_107 | ---NA--- | | |
| TRINITY_DN26017_c0_g1_i1_len_320_108440.p1_type_internal_len_107 | ---NA--- | | |

| | | | |
|---|---|---|---|
| TRINITY_DN5050_c0_g1_i1_len_318_250747.p1_type_5prime_partial_len_106 | ---NA--- | | |
| TRINITY_DN18237_c0_g1_i1_len_2180_58983.p3_type_complete_len_106 | ---NA--- | | |
| TRINITY_DN14766_c0_g1_i1_len_318_45262.p1_type_internal_len_107 | ---NA--- | | |
| TRINITY_DN31364_c0_g1_i1_len_321_139458.p1_type_internal_len_107 | ---NA--- | | |
| TRINITY_DN2713_c0_g1_i1_len_318_250760.p1_type_internal_len_107 | ---NA--- | | |
| TRINITY_DN29554_c0_g1_i1_len_318_250755.p1_type_internal_len_107 | ---NA--- | | |
| TRINITY_DN31046_c0_g1_i2_len_3757_187732.p3_type_complete_len_106 | ---NA--- | | |
| TRINITY_DN30595_c0_g1_i1_len_319_250700.p1_type_internal_len_107 | ---NA--- | | |
| TRINITY_DN12443_c0_g1_i1_len_319_282762.p1_type_internal_len_107 | XP_026287900.1heat shock 70 kDa protein cognate 4 | F:GO:0005524 | |
| TRINITY_DN4692_c0_g1_i1_len_319_250707.p1_type_internal_len_107 | ---NA--- | | |
| TRINITY_DN1776_c0_g1_i1_len_319_250708.p1_type_internal_len_107 | ---NA--- | | |
| TRINITY_DN17319_c0_g1_i1_len_3718_153797.p5_type_5prime_partial_len_106 | ---NA--- | | |
| TRINITY_DN26565_c0_g1_i1_len_319_250685.p2_type_internal_len_107 | ---NA--- | | |
| TRINITY_DN17651_c0_g1_i2_len_319_250690.p1_type_internal_len_107 | ---NA--- | | |
| TRINITY_DN5333_c0_g1_i1_len_319_250696.p1_type_internal_len_107 | ---NA--- | | |
| TRINITY_DN28950_c0_g1_i1_len_319_250720.p1_type_internal_len_107 | ---NA--- | | |

| | | | |
|---|---|---|---|
| TRINITY_DN15173_c0_g1_i1_len_1767_92609.p2_type_complete_len_106 | ---NA--- | | |
| TRINITY_DN3076_c0_g1_i1_len_319_250730.p1_type_internal_len_107 | ---NA--- | | |
| TRINITY_DN21633_c0_g1_i1_len_1641_157272.p2_type_5prime_partial_len_106 | ---NA--- | | |
| TRINITY_DN31487_c0_g1_i1_len_319_250715.p1_type_internal_len_107 | ---NA--- | | |
| TRINITY_DN11613_c0_g1_i1_len_1152_235020.p3_type_complete_len_106 | ---NA--- | | |
| TRINITY_DN30401_c0_g1_i1_len_319_282810.p1_type_internal_len_107 | ---NA--- | | |
| TRINITY_DN9593_c0_g1_i2_len_318_139613.p1_type_internal_len_107 | ---NA--- | | |
| TRINITY_DN6129_c0_g1_i1_len_319_139571.p1_type_3prime_partial_len_107 | XP_018022184.1 cytochrome P450 6k1-like | F:GO:0016491; F:GO:0046872 | |
| TRINITY_DN21455_c0_g1_i1_len_319_282809.p1_type_internal_len_107 | ---NA--- | | |
| TRINITY_DN6606_c0_g1_i1_len_360_210107.p1_type_complete_len_106 | ---NA--- | | |
| TRINITY_DN33482_c0_g1_i1_len_318_139664.p1_type_internal_len_107 | ---NA--- | | |
| TRINITY_DN27733_c0_g1_i1_len_318_139642.p1_type_internal_len_107 | ---NA--- | | |
| TRINITY_DN28924_c0_g1_i1_len_318_139659.p1_type_internal_len_107 | XP_018016102.1 protein bric-a-brac 2-like isoform X2 | | |
| TRINITY_DN30700_c0_g1_i1_len_320_139496.p1_type_internal_len_107 | ---NA--- | | |
| TRINITY_DN22500_c0_g1_i2_len_2380_230047.p3_type_3prime_partial_len_107 | ---NA--- | | |
| TRINITY_DN34253_c0_g1_i1_len_320_139498.p1_type_internal_len_107 | ---NA--- | | |

| | | | |
|---|---|---|---|
| TRINITY_DN16832_c0_g1_i1_len_320_139505.p1_type_internal_len_107 | ---NA--- | | |
| TRINITY_DN1911_c0_g1_i1_len_320_139506.p1_type_internal_len_107 | ---NA--- | | |
| TRINITY_DN29353_c0_g1_i1_len_318_250768.p1_type_internal_len_107 | XP_018027327.1PREDICTED: uncharacterized protein LOC108682635 | C:GO:0016020; C:GO:0016021 | |
| TRINITY_DN15257_c0_g1_i1_len_994_160709.p3_type_complete_len_106 | ---NA--- | | |
| TRINITY_DN14154_c0_g1_i1_len_320_139487.p1_type_internal_len_107 | ---NA--- | | |
| TRINITY_DN9023_c0_g1_i1_len_318_250769.p1_type_internal_len_107 | ---NA--- | | |
| TRINITY_DN6824_c0_g1_i1_len_319_139569.p1_type_internal_len_107 | XP_011866021.1 muscle-specific protein 20 | | |
| TRINITY_DN13187_c0_g1_i2_len_320_139515.p1_type_internal_len_107 | ACF28464.1single whey acidic protein domain-containing protein isoform 1 | C:GO:0005576; P:GO:0010466; F:GO:0030414 | |
| TRINITY_DN24271_c0_g1_i1_len_320_139519.p1_type_internal_len_107 | ---NA--- | | |
| TRINITY_DN25601_c0_g1_i1_len_2025_190814.p3_type_5prime_partial_len_106 | ---NA--- | | |
| TRINITY_DN30980_c0_g1_i1_len_320_139538.p1_type_internal_len_107 | XP_014134004.1 tyrosine-protein kinase receptor Tie-1 | F:GO:0004672; P:GO:0016310 | |
| TRINITY_DN29071_c0_g1_i1_len_321_282676.p1_type_internal_len_107 | ---NA--- | | |
| TRINITY_DN3962_c0_g1_i1_len_321_282680.p1_type_internal_len_107 | ---NA--- | | |
| TRINITY_DN39919_c0_g1_i1_len_320_212763.p2_type_internal_len_107 | XP_018016962.1 membrane metallo-endopeptidase-like 1 | | |
| TRINITY_DN12396_c0_g1_i1_len_519_275632.p1_type_3prime_partial_len_107 | ---NA--- | | |
| TRINITY_DN22849_c0_g1_i1_len_3579_153861.p3_type_complete_len_106 | ---NA--- | | |

| | | | |
|---|---|---|---|
| TRINITY_DN6464_c0_g1_i1_len_321_282659.p1_type_internal_len_107 | BAK78916.1mitogen-activated protein kinase 14 | P:GO:0000165; F:GO:0004707; F:GO:0005524 | EC:2.7.11; EC:2.7.11.24 |
| TRINITY_DN12422_c0_g1_i1_len_321_250552.p1_type_internal_len_107 | ---NA--- | | |
| TRINITY_DN28687_c0_g1_i1_len_321_174103.p1_type_internal_len_107 | ---NA--- | | |
| TRINITY_DN3303_c0_g1_i1_len_321_250554.p1_type_internal_len_107 | ---NA--- | | |
| TRINITY_DN16919_c0_g1_i1_len_663_200562.p1_type_5prime_partial_len_106 | ---NA--- | | |
| TRINITY_DN214_c0_g1_i1_len_321_250559.p1_type_internal_len_107 | ---NA--- | | |
| TRINITY_DN12555_c0_g1_i1_len_1922_123268.p4_type_complete_len_106 | ---NA--- | | |
| TRINITY_DN2194_c0_g1_i1_len_321_250550.p1_type_internal_len_107 | XP_013784173.1LIM domain kinase 1-like isoform X2 | | |
| TRINITY_DN21531_c0_g1_i1_len_3663_294724.p6_type_complete_len_106 | ---NA--- | | |
| TRINITY_DN26343_c0_g1_i1_len_320_174192.p1_type_internal_len_107 | ---NA--- | | |
| TRINITY_DN11988_c0_g1_i1_len_320_174205.p2_type_internal_len_107 | ---NA--- | | |
| TRINITY_DN28142_c0_g1_i1_len_320_174198.p2_type_internal_len_107 | ---NA--- | | |
| TRINITY_DN31855_c0_g1_i1_len_320_174197.p1_type_internal_len_107 | ---NA--- | | |
| TRINITY_DN10123_c0_g1_i1_len_476_167700.p1_type_5prime_partial_len_106 | ---NA--- | | |
| TRINITY_DN17973_c0_g1_i1_len_1866_266617.p4_type_complete_len_106 | ---NA--- | | |
| TRINITY_DN7934_c0_g1_i1_len_320_212762.p1_type_internal_len_107 | ---NA--- | | |

| | | | |
|---|---|---|---|
| TRINITY_DN28443_c0_g1_i1_len_321_282649.p1_type_internal_len_107 | XP_018018630.1 GMP reductase 2-like | F:GO:0003920; P:GO:0006144; P:GO:0006163; F:GO:0046872; P:GO:0055114; C:GO:1902560 | EC:1.7.1.7 |
| TRINITY_DN24190_c1_g1_i1_len_320_174178.p1_type_internal_len_107 | ---NA--- | | |
| TRINITY_DN27278_c0_g1_i1_len_320_250652.p1_type_internal_len_107 | ---NA--- | | |
| TRINITY_DN16928_c0_g1_i1_len_320_250654.p1_type_internal_len_107 | ---NA--- | | |
| TRINITY_DN3203_c0_g1_i1_len_320_250661.p1_type_internal_len_107 | ---NA--- | | |
| TRINITY_DN31100_c0_g1_i1_len_320_250630.p1_type_internal_len_107 | ---NA--- | | |
| TRINITY_DN35604_c0_g1_i1_len_320_250631.p1_type_internal_len_107 | ---NA--- | | |
| TRINITY_DN37095_c0_g1_i1_len_320_250645.p1_type_internal_len_107 | ---NA--- | | |
| TRINITY_DN7422_c0_g1_i1_len_320_212766.p2_type_internal_len_107 | ---NA--- | | |
| TRINITY_DN36719_c0_g1_i1_len_319_250677.p1_type_internal_len_107 | ---NA--- | | |
| TRINITY_DN7429_c0_g1_i1_len_356_210350.p1_type_5prime_partial_len_106 | ---NA--- | | |
| TRINITY_DN26621_c0_g1_i1_len_319_250684.p1_type_internal_len_107 | XP_018020972.1 synaptojanin-1-like isoform X4 | | |
| TRINITY_DN31517_c0_g1_i1_len_319_250670.p1_type_internal_len_107 | ---NA--- | | |
| TRINITY_DN33381_c0_g1_i1_len_321_250569.p1_type_internal_len_107 | ---NA--- | | |
| TRINITY_DN32334_c0_g1_i1_len_321_250591.p1_type_internal_len_107 | XP_018027377.1 ubiquitin carboxyl-terminal hydrolase 16-like | P:GO:0006511; F:GO:0008270; P:GO:0016579; F:GO:0036459 | EC:3.4.19.12 |
| TRINITY_DN29538_c0_g1_i1_len_321_250560.p1_type_internal_len_107 | ---NA--- | | |

| | | | |
|---|---|---|---|
| TRINITY_DN20655_c0_g2_i1_len_320_282712.p1_type_internal_len_107 | ---NA--- | | |
| TRINITY_DN28066_c0_g1_i1_len_321_250562.p1_type_internal_len_107 | ---NA--- | | |
| TRINITY_DN27862_c0_g1_i1_len_320_250620.p1_type_internal_len_107 | XP_020296299.1GTPase-activating protein and VPS9 domain-containing protein 1 isoform X3 | P:GO:0007165; P:GO:0043087 | |
| TRINITY_DN11819_c0_g1_i1_len_566_202519.p3_type_5prime_partial_len_106 | APG16029.1broad-complex | F:GO:0003676 | |
| TRINITY_DN30157_c0_g1_i1_len_320_250622.p1_type_internal_len_107 | EDO46894.1predicted protein | C:GO:0005774; F:GO:0015184; P:GO:0015811 | |
| TRINITY_DN26829_c0_g1_i1_len_321_250596.p1_type_internal_len_107 | XP_018024896.1 fat-like cadherin-related tumor suppressor homolog | | |
| TRINITY_DN5212_c0_g1_i1_len_320_250604.p1_type_internal_len_107 | XP_018023988.1 gamma-butyrobetaine dioxygenase-like | | |
| TRINITY_DN6614_c0_g1_i1_len_320_250606.p1_type_internal_len_107 | ---NA--- | | |
| 2886\|_\|c18136_f2p1_2472_isoform_c18136_full_length_coverage_2_non_full_length_coverage_1_isoform_length_2472_17976 | BBC28099.1xanthine dehydrogenase | F:GO:0004854; F:GO:0004855; F:GO:0005506; F:GO:0009055; P:GO:0022900; F:GO:0051537; F:GO:0071949 | EC:1.17.3; EC:1.17.1; EC:1.17.1.4; EC:1.17.3.2 |
| 2887\|_\|c2234_f3p1_2472_isoform_c2234_full_length_coverage_3_non_full_length_coverage_1_isoform_length_2472_11747 | ALM09354.1ubiquitin carboxyl-terminal esterase L3 | F:GO:0004843; P:GO:0006511 | EC:3.4.19.12 |
| 2889\|_\|c2125_f2p6_2471_isoform_c2125_full_length_coverage_2_non_full_length_coverage_6_isoform_length_2471_28237 | ---NA--- | | |
| 2890\|_\|c2581_f3p1_2471_isoform_c2581_full_length_coverage_3_non_full_length_coverage_1_isoform_length_2471_21060 | AVN99064.1uracil phosphoribosyltransferase | F:GO:0004845; P:GO:0009116 | EC:2.4.2.9 |
| 2891\|_\|c2636_f4p2_2471_isoform_c2636_full_length_coverage_4_non_full_length_coverage_2_isoform_length_2471_21107 | XP_014663448.1 DCN1-like protein 3 | | |

| | | | |
|---|---|---|---|
| 2892\|_\|c26298_f2p15_2471_isoform_c26298_full_length_coverage_2_non_full_length_coverage_15_isoform_length_2471_23004 | XP_022239686.1eukaryotic translation initiation factor 4 gamma 3-like | F:GO:0003729; F:GO:0003743; P:GO:0006413; C:GO:0016281 | |
| TRINITY_DN28479_c0_g1_i1_len_2819_188821.p1_type_5prime_partial_len_823 | XP_018012684.1PREDICTED: uncharacterized protein LOC108669786 | | |
| 2895\|_\|c1899_f7p2_2468_isoform_c1899_full_length_coverage_7_non_full_length_coverage_2_isoform_length_2468_20596 | KNC21688.1Cytoplasmic phosphatidylinositol transfer protein 1 | F:GO:0005548; P:GO:0015914 | |
| 2896\|_\|c2628_f5p4_2468_isoform_c2628_full_length_coverage_5_non_full_length_coverage_4_isoform_length_2468_16569 | GBC13699.1retrotransposable element | F:GO:0003676; P:GO:0015074 | |
| 2897\|_\|c3126_f2p1_2468_isoform_c3126_full_length_coverage_2_non_full_length_coverage_1_isoform_length_2468_16742 | XP_016520193.1 zinc finger protein OZF-like | | |
| TRINITY_DN10767_c0_g1_i1_len_613_201513.p1_type_complete_len_106 | ---NA--- | | |
| TRINITY_DN17695_c0_g1_i1_len_687_163711.p1_type_complete_len_106 | ---NA--- | | |
| TRINITY_DN12474_c0_g1_i1_len_372_105563.p1_type_5prime_partial_len_106 | ---NA--- | | |
| TRINITY_DN29552_c0_g1_i1_len_3317_188119.p3_type_complete_len_106 | ---NA--- | | |
| TRINITY_DN8066_c0_g1_i1_len_846_237477.p5_type_5prime_partial_len_106 | ---NA--- | | |
| TRINITY_DN13147_c0_g1_i1_len_697_301154.p1_type_3prime_partial_len_107 | ---NA--- | | |
| TRINITY_DN11040_c0_g1_i1_len_669_163947.p1_type_complete_len_106 | ---NA--- | | |
| TRINITY_DN31806_c0_g1_i1_len_349_281126.p1_type_5prime_partial_len_106 | ---NA--- | | |
| TRINITY_DN21755_c0_g1_i1_len_886_299521.p2_type_5prime_partial_len_106 | ---NA--- | | |

| | | | |
|---|---|---|---|
| TRINITY_DN21088_c0_g1_i1_len_1826_296024.p3_type_5prime_partial_len_106 | ---NA--- | | |
| TRINITY_DN18401_c0_g1_i1_len_775_238243.p1_type_complete_len_106 | ---NA--- | | |
| TRINITY_DN10692_c0_g1_i1_len_320_76509.p1_type_internal_len_107 | ---NA--- | | |
| TRINITY_DN18219_c0_g1_i1_len_372_247636.p1_type_5prime_partial_len_106 | ACF28467.1single whey acidic protein domain-containing protein isoform 2 | C:GO:0005576; P:GO:0010466; F:GO:0030414 | |
| TRINITY_DN6525_c0_g1_i2_len_351_281039.p1_type_5prime_partial_len_106 | ---NA--- | | |
| TRINITY_DN6575_c0_g1_i1_len_322_212640.p1_type_internal_len_107 | ---NA--- | | |
| TRINITY_DN17959_c0_g1_i1_len_2048_266189.p4_type_complete_len_106 | ---NA--- | | |
| TRINITY_DN22613_c0_g1_i1_len_462_205481.p1_type_complete_len_106 | ---NA--- | | |
| TRINITY_DN2758_c0_g1_i1_len_358_106241.p1_type_5prime_partial_len_106 | ---NA--- | | |
| TRINITY_DN14868_c0_g1_i2_len_886_237087.p2_type_5prime_partial_len_106 | XP_018027257.1 UPF0047 protein YjbQ-like | | |
| TRINITY_DN24585_c0_g2_i1_len_339_249471.p1_type_5prime_partial_len_106 | ---NA--- | | |
| TRINITY_DN13683_c0_g1_i1_len_1246_234395.p1_type_complete_len_106 | ---NA--- | | |
| TRINITY_DN16373_c0_g1_i2_len_617_164707.p1_type_5prime_partial_len_106 | ---NA--- | | |
| TRINITY_DN20816_c0_g1_i1_len_1115_195578.p1_type_3prime_partial_len_107 | ---NA--- | | |
| TRINITY_DN26406_c0_g1_i1_len_1359_193908.p1_type_5prime_partial_len_106 | ---NA--- | | |
| TRINITY_DN21171_c0_g2_i1_len_1663_296392.p7_type_5prime_partial_len_106 | ---NA--- | | |

| | | | |
|---|---|---|---|
| TRINITY_DN23293_c0_g1_i1_len_1316_194161.p1_type_complete_len_106 | ---NA--- | | |
| TRINITY_DN16964_c0_g1_i1_len_751_272247.p1_type_5prime_partial_len_106 | ---NA--- | | |
| TRINITY_DN21866_c0_g1_i1_len_5594_121768.p3_type_5prime_partial_len_106 | ---NA--- | | |
| TRINITY_DN13768_c0_g1_i1_len_422_207047.p1_type_5prime_partial_len_106 | ---NA--- | | |
| TRINITY_DN24539_c1_g1_i1_len_3014_188523.p1_type_5prime_partial_len_106 | ---NA--- | | |
| TRINITY_DN34291_c0_g1_i1_len_531_203391.p1_type_3prime_partial_len_107 | ---NA--- | | |
| TRINITY_DN24047_c0_g1_i1_len_1361_233724.p3_type_complete_len_106 | ---NA--- | | |
| TRINITY_DN4837_c0_g1_i1_len_490_69373.p1_type_3prime_partial_len_107 | ---NA--- | | |
| TRINITY_DN7594_c0_g1_i1_len_351_248740.p1_type_complete_len_106 | ---NA--- | | |
| TRINITY_DN8611_c0_g1_i1_len_358_210209.p1_type_5prime_partial_len_106 | ---NA--- | | |
| TRINITY_DN14470_c0_g1_i1_len_675_273082.p1_type_complete_len_106 | ---NA--- | | |
| TRINITY_DN31145_c0_g1_i3_len_3125_188345.p1_type_complete_len_106 | ---NA--- | | |
| TRINITY_DN18209_c0_g1_i2_len_1842_30664.p2_type_complete_len_106 | ---NA--- | | |
| TRINITY_DN30471_c0_g1_i1_len_362_280548.p1_type_3prime_partial_len_107 | PSN45457.1UPF0668 protein | | |
| TRINITY_DN10898_c0_g1_i1_len_400_104382.p1_type_complete_len_106 | ---NA--- | | |
| TRINITY_DN23068_c0_g2_i2_len_883_197672.p1_type_3prime_partial_len_107 | ---NA--- | | |

| | | | |
|---|---|---|---|
| TRINITY_DN25966_c0_g1_i1_len_411_103948.p1_type_5prime_partial_len_106 | XP_025833080.1protein cab-1 | C:GO:0016021 | |
| TRINITY_DN17271_c0_g1_i1_len_7753_227486.p2_type_complete_len_106 | ---NA--- | | |
| TRINITY_DN13223_c0_g1_i1_len_455_133498.p2_type_complete_len_106 | ABB73282.1reverse transcriptase | F:GO:0003964; P:GO:0006278 | EC:2.7.7.49 |
| TRINITY_DN11840_c0_g1_i1_len_319_76594.p1_type_internal_len_107 | ---NA--- | | |
| TRINITY_DN1482_c0_g1_i2_len_319_76585.p3_type_internal_len_107 | ---NA--- | | |
| TRINITY_DN25235_c1_g5_i1_len_371_247717.p1_type_5prime_partial_len_106 | ---NA--- | | |
| TRINITY_DN8514_c0_g1_i1_len_430_103294.p1_type_5prime_partial_len_106 | ---NA--- | | |
| TRINITY_DN28932_c0_g1_i1_len_319_76612.p1_type_internal_len_107 | ---NA--- | | |
| TRINITY_DN27567_c0_g1_i1_len_319_76600.p3_type_internal_len_107 | AYD41582.1macrophage mannose receptor 1-like protein | C:GO:0016020; C:GO:0016021 | |
| TRINITY_DN10736_c0_g1_i2_len_699_163548.p1_type_5prime_partial_len_106 | ---NA--- | | |
| TRINITY_DN30039_c0_g1_i1_len_320_76539.p1_type_internal_len_107 | XP_018028132.1 protein ECT2-like | P:GO:0050794; F:GO:0098772 | |
| TRINITY_DN27085_c0_g1_i1_len_320_76534.p1_type_internal_len_107 | ---NA--- | | |
| TRINITY_DN7230_c0_g1_i1_len_737_199484.p1_type_5prime_partial_len_106 | ---NA--- | | |
| TRINITY_DN3742_c0_g1_i1_len_320_76567.p1_type_internal_len_107 | ---NA--- | | |
| TRINITY_DN12368_c0_g1_i1_len_360_13414.p1_type_3prime_partial_len_107 | ---NA--- | | |
| TRINITY_DN18931_c0_g1_i1_len_655_200721.p1_type_5prime_partial_len_106 | ---NA--- | | |

| | | | |
|---|---|---|---|
| TRINITY_DN1210_c0_g1_i1_len_318_76714.p1_type_internal_len_107 | ---NA--- | | |
| TRINITY_DN20435_c0_g1_i1_len_1135_2892.p3_type_5prime_partial_len_106 | ---NA--- | | |
| TRINITY_DN9036_c0_g1_i1_len_322_212590.p1_type_internal_len_107 | ---NA--- | | |
| TRINITY_DN19103_c0_g1_i1_len_444_10281.p2_type_5prime_partial_len_106 | ---NA--- | | |
| TRINITY_DN5930_c0_g1_i1_len_319_76645.p2_type_internal_len_107 | ---NA--- | | |
| TRINITY_DN25771_c0_g1_i1_len_318_76679.p1_type_internal_len_107 | ---NA--- | | |
| TRINITY_DN14345_c0_g1_i1_len_1352_61256.p3_type_5prime_partial_len_106 | ---NA--- | | |
| TRINITY_DN7779_c0_g1_i1_len_320_310833.p1_type_internal_len_107 | KMQ82071.1enzymatic polyprotein endonuclease reverse | F:GO:0004519; P:GO:0090305 | |
| TRINITY_DN27432_c0_g1_i2_len_2278_190016.p2_type_complete_len_106 | ---NA--- | | |
| TRINITY_DN27166_c0_g1_i1_len_2244_190117.p2_type_3prime_partial_len_107 | ---NA--- | | |
| TRINITY_DN10301_c0_g1_i1_len_395_208275.p1_type_5prime_partial_len_106 | ---NA--- | | |
| TRINITY_DN5824_c0_g1_i1_len_321_212728.p1_type_internal_len_107 | XP_018027756.1PREDICTED: uncharacterized protein C630.12-like | | |
| TRINITY_DN16028_c0_g1_i1_len_782_198840.p2_type_complete_len_106 | ---NA--- | | |
| TRINITY_DN34033_c0_g1_i1_len_321_212715.p2_type_internal_len_107 | XP_018017581.1 RNA-binding protein 45-like | F:GO:0003723 | |
| TRINITY_DN20862_c0_g1_i1_len_953_236497.p1_type_5prime_partial_len_106 | XP_019629822.1 LYR motif-containing protein 4-like isoform X2 | | |
| TRINITY_DN28623_c0_g1_i1_len_427_245384.p1_type_complete_len_106 | ---NA--- | | |

| | | | |
|---|---|---|---|
| TRINITY_DN18873_c0_g1_i2_len_1661_1318.p3_type_complete_len_106 | ---NA--- | | |
| TRINITY_DN20935_c0_g1_i1_len_1741_123623.p3_type_5prime_partial_len_106 | ---NA--- | | |
| TRINITY_DN12379_c1_g1_i1_len_428_245344.p2_type_5prime_partial_len_106 | ---NA--- | | |
| TRINITY_DN9852_c0_g1_i1_len_542_166111.p1_type_5prime_partial_len_106 | ---NA--- | | |
| TRINITY_DN20646_c0_g1_i1_len_2463_265508.p3_type_5prime_partial_len_106 | ---NA--- | | |
| TRINITY_DN7607_c0_g1_i1_len_322_108269.p1_type_internal_len_107 | ---NA--- | | |
| TRINITY_DN16347_c0_g1_i7_len_422_40783.p1_type_5prime_partial_len_106 | ---NA--- | | |
| TRINITY_DN22268_c0_g1_i1_len_321_310787.p1_type_internal_len_107 | ---NA--- | | |
| TRINITY_DN20010_c0_g1_i3_len_417_207271.p1_type_3prime_partial_len_107 | XP_018025670.1 tropomyosin isoform X22 | | |
| TRINITY_DN7299_c0_g1_i1_len_327_282338.p1_type_3prime_partial_len_107 | ---NA--- | | |
| TRINITY_DN12301_c0_g1_i2_len_499_167076.p1_type_complete_len_106 | ---NA--- | | |
| TRINITY_DN20306_c1_g1_i1_len_325_250333.p1_type_5prime_partial_len_106 | ---NA--- | | |
| TRINITY_DN8648_c0_g1_i1_len_320_212744.p1_type_internal_len_107 | ---NA--- | | |
| TRINITY_DN26817_c0_g1_i1_len_322_108302.p1_type_internal_len_107 | ---NA--- | | |
| TRINITY_DN28524_c0_g1_i1_len_321_310769.p3_type_internal_len_107 | ---NA--- | | |
| TRINITY_DN9609_c0_g1_i1_len_320_310803.p1_type_internal_len_107 | ---NA--- | | |

| | | | |
|---|---|---|---|
| TRINITY_DN1264_c0_g1_i1_len_330_282163.p1_type_5prime_partial_len_106 | ---NA--- | | |
| TRINITY_DN13396_c0_g1_i1_len_320_212738.p1_type_internal_len_107 | ---NA--- | | |
| TRINITY_DN2224_c0_g1_i1_len_355_137435.p1_type_5prime_partial_len_106 | ---NA--- | | |
| TRINITY_DN33283_c0_g1_i1_len_320_212739.p1_type_internal_len_107 | XP_018024610.1 galactosylceramide sulfotransferase-like | | |
| TRINITY_DN12635_c0_g1_i2_len_597_165040.p1_type_5prime_partial_len_106 | ---NA--- | | |
| TRINITY_DN6622_c0_g1_i1_len_339_107184.p1_type_5prime_partial_len_106 | ---NA--- | | |
| TRINITY_DN4698_c0_g1_i1_len_959_299032.p2_type_3prime_partial_len_107 | ---NA--- | | |
| TRINITY_DN41522_c0_g1_i1_len_321_212672.p1_type_internal_len_107 | ---NA--- | | |
| TRINITY_DN7509_c0_g1_i1_len_403_246269.p1_type_3prime_partial_len_107 | XP_018009639.1 protein THEM6-like | | |
| TRINITY_DN6730_c0_g1_i1_len_321_212678.p1_type_internal_len_107 | ---NA--- | | |
| TRINITY_DN33794_c0_g1_i1_len_610_201561.p1_type_3prime_partial_len_107 | ---NA--- | | |
| 2898\|_\|c36269_f2p0_2467_isoform_c36269_full_length_coverage_2_non_full_length_coverage_0_isoform_length_2467_18944 | ---NA--- | | |
| TRINITY_DN29720_c0_g1_i2_len_5185_187086.p1_type_complete_len_822 | ATU31742.1armadillo segment polarity protein-like protein | P:GO:0007155; F:GO:0045296 | |
| TRINITY_DN21406_c0_g1_i1_len_3785_153768.p1_type_complete_len_822 | PNF28200.1Autophagy-related protein 9A | C:GO:0016020; C:GO:0043227; C:GO:0043229; C:GO:0044444 | |
| TRINITY_DN29537_c0_g1_i1_len_2656_189116.p1_type_5prime_partial_len_822 | AMO02545.1carboxypeptidase D | F:GO:0004180; F:GO:0004181; P:GO:0006508; F:GO:0008270; C:GO:0016020; C:GO:0016021 | |

| | | | |
|---|---|---|---|
| TRINITY_DN25355_c0_g1_i1_len_3573_228424.p1_type_complete_len_822 | XP_018016913.1PREDICTED: uncharacterized protein LOC108673573 | C:GO:0016020; C:GO:0016021 | |
| TRINITY_DN30503_c0_g1_i1_len_3293_188146.p1_type_5prime_partial_len_822 | XP_026289597.1TBC1 domain family member 1 | | |
| 2904\|_\|c23701_f2p4_2466_isoform_c23701_full_length_coverage_2_non_full_length_coverage_4_isoform_length_2466_13882 | AEB41038.1selenophosphate synthetase | F:GO:0004756; F:GO:0005524; P:GO:0016260; P:GO:0016310 | EC:2.7.9.3 |
| 2905\|_\|c2578_f4p5_2466_isoform_c2578_full_length_coverage_4_non_full_length_coverage_5_isoform_length_2466_16526 | XP_021915891.1CCR4-NOT transcription complex subunit 6-like | | |
| 2907\|_\|c23426_f2p3_2465_isoform_c23426_full_length_coverage_2_non_full_length_coverage_3_isoform_length_2465_18287 | ---NA--- | | |
| 2908\|_\|c2687_f3p1_2465_isoform_c2687_full_length_coverage_3_non_full_length_coverage_1_isoform_length_2465_21152 | ---NA--- | | |
| TRINITY_DN21495_c0_g1_i1_len_2164_155675.p3_type_complete_len_106 | ---NA--- | | |
| TRINITY_DN39554_c0_g1_i1_len_321_212673.p1_type_internal_len_107 | CAX48990.1angiotensin converting enzyme | F:GO:0004180; P:GO:0006508; F:GO:0008237; F:GO:0008241; C:GO:0016020; F:GO:0046872 | EC:3.4.15 |
| TRINITY_DN31858_c0_g1_i1_len_622_201365.p2_type_complete_len_106 | ---NA--- | | |
| TRINITY_DN26424_c0_g1_i1_len_347_106770.p1_type_5prime_partial_len_106 | ---NA--- | | |
| TRINITY_DN32935_c0_g1_i1_len_321_212657.p1_type_internal_len_107 | EFA07004.1Ankyrin repeat and IBR domain-containing protein 1-like Protein | C:GO:0000151; P:GO:0000209; C:GO:0005737; F:GO:0031624; P:GO:0032436; F:GO:0046872; F:GO:0061630 | |
| TRINITY_DN14855_c0_g1_i1_len_322_212650.p1_type_5prime_partial_len_106 | ---NA--- | | |
| TRINITY_DN9295_c0_g1_i1_len_337_249570.p1_type_5prime_partial_len_106 | ---NA--- | | |

| | | | |
|---|---|---|---|
| TRINITY_DN17319_c0_g1_i1_len_420_207127.p1_type_5prime_partial_len_106 | ---NA--- | | |
| TRINITY_DN19544_c0_g1_i1_len_801_198601.p1_type_3prime_partial_len_107 | ---NA--- | | |
| TRINITY_DN15175_c0_g1_i1_len_803_162384.p1_type_5prime_partial_len_106 | ---NA--- | | |
| TRINITY_DN41583_c0_g1_i1_len_321_212659.p1_type_internal_len_107 | AGY46351.1domeless | C:GO:0016021 | |
| TRINITY_DN17126_c0_g1_i1_len_436_206485.p1_type_complete_len_106 | ---NA--- | | |
| TRINITY_DN8590_c0_g1_i1_len_319_310896.p2_type_internal_len_107 | ---NA--- | | |
| TRINITY_DN38560_c0_g1_i1_len_321_212705.p1_type_3prime_partial_len_107 | ---NA--- | | |
| TRINITY_DN9650_c0_g1_i1_len_321_212708.p1_type_internal_len_107 | ---NA--- | | |
| TRINITY_DN19771_c0_g1_i1_len_414_245843.p1_type_5prime_partial_len_106 | ---NA--- | | |
| TRINITY_DN14281_c0_g1_i1_len_644_66423.p2_type_complete_len_106 | ---NA--- | | |
| TRINITY_DN11488_c0_g1_i1_len_329_250038.p1_type_5prime_partial_len_106 | APU54662.1suppressor of cytokine signaling 2 | P:GO:0001558; F:GO:0005159; C:GO:0005737; F:GO:0008269; P:GO:0035556; P:GO:0046426 | |
| TRINITY_DN20633_c0_g1_i1_len_3219_154116.p2_type_5prime_partial_len_106 | ---NA--- | | |
| TRINITY_DN18678_c0_g1_i2_len_1550_232772.p2_type_3prime_partial_len_107 | AGW23856.1nitric oxide synthase adaptor protein | | |
| TRINITY_DN24121_c0_g1_i1_len_3527_228449.p3_type_complete_len_106 | ---NA--- | | |
| TRINITY_DN1905_c0_g1_i1_len_597_274188.p1_type_5prime_partial_len_106 | ---NA--- | | |
| TRINITY_DN30041_c0_g1_i1_len_2482_189488.p2_type_complete_len_106 | ---NA--- | | |

| | | | |
|---|---|---|---|
| TRINITY_DN4498_c0_g1_i1_len_318_212898.p1_type_internal_len_107 | ---NA--- | | |
| TRINITY_DN36414_c0_g1_i1_len_320_212782.p1_type_internal_len_107 | ---NA--- | | |
| TRINITY_DN24750_c0_g1_i1_len_996_196590.p1_type_complete_len_106 | ---NA--- | | |
| TRINITY_DN19994_c1_g1_i4_len_8407_264318.p6_type_complete_len_106 | ---NA--- | | |
| TRINITY_DN18590_c0_g1_i1_len_544_7947.p1_type_5prime_partial_len_106 | ---NA--- | | |
| TRINITY_DN39967_c0_g1_i1_len_319_212824.p1_type_internal_len_107 | ---NA--- | | |
| TRINITY_DN25612_c0_g2_i2_len_505_204086.p1_type_complete_len_106 | ---NA--- | | |
| TRINITY_DN7694_c0_g1_i1_len_319_15656.p2_type_internal_len_107 | ---NA--- | | |
| TRINITY_DN23673_c0_g1_i1_len_460_277043.p2_type_5prime_partial_len_106 | XP_015378495.1 low-density lipoprotein receptor-related protein 2-like | | |
| TRINITY_DN17445_c0_g1_i1_len_548_202934.p1_type_5prime_partial_len_106 | ---NA--- | | |
| TRINITY_DN5544_c0_g1_i1_len_337_173181.p1_type_5prime_partial_len_106 | XP_023708370.1ring canal kelch homolog | | |
| TRINITY_DN8089_c0_g1_i1_len_1364_158406.p1_type_complete_len_106 | ---NA--- | | |
| TRINITY_DN8312_c0_g1_i1_len_339_211444.p1_type_5prime_partial_len_106 | ---NA--- | | |
| TRINITY_DN6373_c0_g1_i1_len_771_97329.p3_type_complete_len_106 | ---NA--- | | |
| TRINITY_DN16383_c0_g1_i2_len_1091_33042.p2_type_5prime_partial_len_106 | AQW41378.1selenium-dependent glutathione peroxidase | F:GO:0004602; P:GO:0006979; P:GO:0055114; P:GO:0098869 | EC:1.11.1.9; EC:1.11.1.7 |
| TRINITY_DN7311_c0_g1_i1_len_369_171519.p1_type_5prime_partial_len_106 | ---NA--- | | |

| | | | |
|---|---|---|---|
| TRINITY_DN17153_c0_g1_i1_len_320_212776.p1_type_internal_len_107 | ---NA--- | | |
| TRINITY_DN4458_c0_g1_i1_len_328_212157.p1_type_5prime_partial_len_106 | ---NA--- | | |
| TRINITY_DN28004_c0_g1_i1_len_355_172243.p1_type_5prime_partial_len_106 | ---NA--- | | |
| TRINITY_DN9748_c0_g1_i1_len_319_212798.p1_type_internal_len_107 | XP_015775355.1 Golgi-associated PDZ and coiled-coil motif-containing protein-like | | |
| TRINITY_DN560_c1_g1_i1_len_319_212828.p1_type_internal_len_107 | KFM58512.1Dual serine/threonine and tyrosine protein kinase | F:GO:0004674; F:GO:0004712; F:GO:0004713; F:GO:0005524; C:GO:0005737; C:GO:0016323; C:GO:0016324; P:GO:0018108; C:GO:0030054; P:GO:0048568 | EC:2.7.10; EC:2.7.11; EC:2.7.12.1 |
| TRINITY_DN8700_c0_g1_i1_len_362_74124.p1_type_3prime_partial_len_107 | ---NA--- | | |
| TRINITY_DN19931_c1_g3_i1_len_692_35815.p1_type_complete_len_106 | ---NA--- | | |
| TRINITY_DN12142_c0_g1_i1_len_318_212915.p1_type_internal_len_107 | ---NA--- | | |
| TRINITY_DN4714_c0_g1_i1_len_1185_159421.p2_type_complete_len_106 | XP_022193978.1GTP-binding protein YPT53 | F:GO:0003924; F:GO:0005525 | EC:3.6.1.15 |
| TRINITY_DN11095_c0_g1_i1_len_384_208824.p1_type_5prime_partial_len_106 | ---NA--- | | |
| TRINITY_DN8182_c0_g1_i1_len_319_212799.p1_type_internal_len_107 | XP_018021444.1 pseudouridine-5'-phosphatase-like isoform X1 | F:GO:0016787 | |
| TRINITY_DN17229_c0_g1_i1_len_682_200280.p1_type_complete_len_106 | ---NA--- | | |
| TRINITY_DN31501_c0_g1_i1_len_319_212847.p1_type_internal_len_107 | ---NA--- | | |
| TRINITY_DN25487_c0_g1_i1_len_320_15605.p1_type_internal_len_107 | CAD45189.1Notch protein | F:GO:0005509; P:GO:0007219; P:GO:0007275; C:GO:0016021; P:GO:0030154; P:GO:0050793 | |

| | | | |
|---|---|---|---|
| TRINITY_DN27490_c0_g1_i1_len_320_15557.p1_type_internal_len_107 | ---NA--- | | |
| TRINITY_DN28946_c0_g1_i1_len_320_15579.p1_type_internal_len_107 | ---NA--- | | |
| TRINITY_DN9121_c0_g1_i1_len_1445_267954.p2_type_complete_len_106 | ---NA--- | | |
| TRINITY_DN8649_c0_g1_i1_len_583_241112.p1_type_5prime_partial_len_106 | AFU61123.1phosphatidylinositol-4,5-bisphosphate 3-kinase catalytic subunit delta isoform | F:GO:0005524; F:GO:0016303; P:GO:0036092; P:GO:0046854; P:GO:0048015 | EC:2.7.1.137 |
| TRINITY_DN4932_c0_g1_i1_len_831_64309.p3_type_complete_len_106 | ---NA--- | | |
| TRINITY_DN5145_c0_g1_i1_len_321_45066.p1_type_internal_len_107 | ---NA--- | | |
| TRINITY_DN40768_c0_g1_i1_len_320_212785.p1_type_internal_len_107 | ---NA--- | | |
| TRINITY_DN238_c0_g1_i1_len_338_173127.p1_type_5prime_partial_len_106 | ---NA--- | | |
| TRINITY_DN10700_c0_g1_i2_len_319_212809.p1_type_internal_len_107 | ---NA--- | | |
| TRINITY_DN10459_c0_g1_i1_len_318_212886.p1_type_internal_len_107 | ---NA--- | | |
| TRINITY_DN21111_c0_g1_i1_len_1198_194979.p4_type_5prime_partial_len_106 | ---NA--- | | |
| TRINITY_DN13538_c0_g1_i1_len_321_45060.p1_type_internal_len_107 | ---NA--- | | |
| TRINITY_DN3649_c0_g1_i1_len_406_278732.p1_type_5prime_partial_len_106 | ---NA--- | | |
| TRINITY_DN12783_c0_g1_i1_len_319_212814.p1_type_internal_len_107 | ---NA--- | | |
| TRINITY_DN564_c0_g1_i1_len_1191_195021.p1_type_5prime_partial_len_106 | ---NA--- | | |
| TRINITY_DN31233_c1_g1_i1_len_318_212911.p1_type_5prime_partial_len_106 | ---NA--- | | |

| | | | |
|---|---|---|---|
| TRINITY_DN16208_c0_g1_i1_len_935_197135.p2_type_5prime_partial_len_106 | ---NA--- | | |
| TRINITY_DN29657_c0_g1_i1_len_413_169729.p1_type_5prime_partial_len_106 | ---NA--- | | |
| TRINITY_DN20289_c0_g1_i1_len_1106_298246.p2_type_5prime_partial_len_106 | ---NA--- | | |
| TRINITY_DN9033_c1_g1_i1_len_473_205122.p1_type_3prime_partial_len_107 | XP_017322027.1 acid sphingomyelinase-like phosphodiesterase 3b isoform X1 | F:GO:0004767; C:GO:0005576; C:GO:0005615; P:GO:0006685; F:GO:0016787; F:GO:0046872 | |
| TRINITY_DN25962_c0_g1_i1_len_395_170433.p1_type_5prime_partial_len_106 | ---NA--- | | |
| TRINITY_DN17775_c0_g1_i1_len_761_199109.p1_type_3prime_partial_len_107 | ---NA--- | | |
| TRINITY_DN15044_c0_g1_i2_len_320_45124.p1_type_internal_len_107 | ---NA--- | | |
| TRINITY_DN36122_c0_g1_i1_len_319_212823.p1_type_internal_len_107 | ---NA--- | | |
| TRINITY_DN19530_c0_g1_i4_len_520_303769.p3_type_5prime_partial_len_106 | ---NA--- | | |
| TRINITY_DN9147_c0_g1_i1_len_606_240688.p1_type_5prime_partial_len_106 | ---NA--- | | |
| TRINITY_DN6580_c0_g1_i1_len_320_212792.p1_type_internal_len_107 | ADZ96218.1JHE-like carboxylesterase 2 | F:GO:0016787 | |
| TRINITY_DN20949_c0_g1_i1_len_322_45029.p1_type_internal_len_107 | ---NA--- | | |
| TRINITY_DN12999_c0_g1_i1_len_1237_159123.p1_type_complete_len_106 | ---NA--- | | |
| TRINITY_DN40678_c0_g1_i1_len_319_212822.p1_type_internal_len_107 | XP_018017231.1 dmX-like protein 2 isoform X2 | | |
| TRINITY_DN26071_c0_g1_i1_len_4969_227791.p2_type_5prime_partial_len_106 | ---NA--- | | |
| TRINITY_DN19219_c0_g1_i1_len_1061_126171.p2_type_3prime_partial_len_107 | ---NA--- | | |

| | | | |
|---|---|---|---|
| TRINITY_DN30469_c0_g1_i1_len_1923_191178.p1_type_5prime_partial_len_106 | ---NA--- | | |
| TRINITY_DN6401_c0_g1_i1_len_418_71637.p1_type_3prime_partial_len_107 | ---NA--- | | |
| TRINITY_DN34443_c0_g1_i1_len_413_169727.p1_type_5prime_partial_len_106 | ---NA--- | | |
| TRINITY_DN9392_c0_g1_i1_len_320_212780.p1_type_internal_len_107 | ---NA--- | | |
| TRINITY_DN39043_c0_g1_i1_len_318_212887.p1_type_internal_len_107 | KOB67854.1Pecanex-like protein 1 | C:GO:0016021 | |
| TRINITY_DN18973_c0_g1_i3_len_1533_31364.p3_type_5prime_partial_len_106 | ---NA--- | | |
| TRINITY_DN14183_c0_g1_i1_len_3980_264522.p3_type_complete_len_106 | ---NA--- | | |
| TRINITY_DN25598_c0_g1_i1_len_1637_192369.p1_type_complete_len_106 | ---NA--- | | |
| TRINITY_DN37271_c0_g1_i1_len_320_212777.p1_type_internal_len_107 | ---NA--- | | |
| TRINITY_DN11764_c0_g1_i1_len_320_212772.p1_type_internal_len_107 | XP_005104705.1 Golgi-associated PDZ and coiled-coil motif-containing protein-like | C:GO:0005794; P:GO:0043004 | |
| TRINITY_DN15601_c0_g1_i1_len_320_15591.p1_type_internal_len_107 | ---NA--- | | |
| TRINITY_DN12438_c0_g1_i1_len_319_212806.p1_type_internal_len_107 | ---NA--- | | |
| TRINITY_DN26535_c0_g1_i1_len_318_282840.p1_type_3prime_partial_len_107 | KPI94349.1H(+)/Cl(-) exchange transporter 3 | | |
| TRINITY_DN5537_c0_g1_i1_len_318_282843.p1_type_internal_len_107 | ---NA--- | | |
| TRINITY_DN17003_c0_g1_i1_len_1852_191476.p3_type_5prime_partial_len_106 | AXY87730.1carnitine O-palmitoyltransferase 1a | C:GO:0016021; F:GO:0016746 | |
| 2909\|_\|c2148_f4p6_2464_isoform_c2148_full_length_coverage_4_non_full_length_coverage_6_isoform_length_2464_11697 | ---NA--- | | |

| | | | |
|---|---|---|---|
| 2910\|_\|c6206_f2p4_2463_isoform_c6206_full_length_coverage_2_non_full_length_coverage_4_isoform_length_2463_17033 | ---NA--- | | |
| 2911\|_\|c7504_f2p0_2463_isoform_c7504_full_length_coverage_2_non_full_length_coverage_0_isoform_length_2463_21697 | ---NA--- | | |
| 2912\|_\|c2882_f2p0_2463_isoform_c2882_full_length_coverage_2_non_full_length_coverage_0_isoform_length_2463_12244 | XP_018019760.1 integrator complex subunit 3-like | | |
| TRINITY_DN17736_c0_g1_i2_len_3028_264939.p1_type_complete_len_821 | ---NA--- | | |
| TRINITY_DN18774_c0_g1_i1_len_2499_265451.p1_type_5prime_partial_len_821 | XP_008420497.1 zinc finger protein OZF-like | | |
| 2915\|_\|c2724_f2p2_2461_isoform_c2724_full_length_coverage_2_non_full_length_coverage_2_isoform_length_2461_21182 | XP_018009713.1 serine/arginine-rich-splicing factor SR34-like isoform X2 | | |
| 2917\|_\|c1650_f3p8_2461_isoform_c1650_full_length_coverage_3_non_full_length_coverage_8_isoform_length_2461_26005 | ---NA--- | | |
| TRINITY_DN21542_c0_g1_i4_len_2516_373.p1_type_3prime_partial_len_821 | ---NA--- | | |
| TRINITY_DN30015_c0_g1_i1_len_2643_189145.p1_type_complete_len_820 | ---NA--- | | |
| TRINITY_DN33923_c0_g1_i1_len_920_197296.p3_type_complete_len_106 | ---NA--- | | |
| TRINITY_DN26415_c0_g1_i1_len_352_309093.p1_type_complete_len_106 | ---NA--- | | |
| TRINITY_DN23237_c0_g1_i1_len_320_15555.p1_type_internal_len_107 | ---NA--- | | |
| TRINITY_DN28822_c0_g1_i1_len_363_171836.p1_type_5prime_partial_len_106 | XP_022195500.1gastrula zinc finger protein XlCGF17.1-like | | |
| TRINITY_DN31309_c0_g1_i1_len_318_310977.p1_type_internal_len_106 | ---NA--- | | |

| | | | |
|---|---|---|---|
| TRINITY_DN10693_c0_g1_i1_len_441_277622.p1_type_complete_len_105 | ---NA--- | | |
| TRINITY_DN16520_c0_g1_i1_len_315_15874.p1_type_internal_len_106 | ---NA--- | | |
| TRINITY_DN19107_c0_g1_i1_len_618_66807.p1_type_complete_len_105 | XP_018909596.1 succinate dehydrogenase assembly factor 4, mitochondrial-like | | |
| TRINITY_DN24717_c0_g1_i3_len_1536_232854.p2_type_complete_len_105 | ---NA--- | | |
| TRINITY_DN19975_c0_g1_i1_len_1380_2028.p4_type_5prime_partial_len_105 | ---NA--- | | |
| TRINITY_DN31775_c0_g1_i1_len_357_210257.p1_type_5prime_partial_len_105 | ---NA--- | | |
| TRINITY_DN11095_c0_g1_i2_len_874_127436.p2_type_complete_len_105 | ---NA--- | | |
| TRINITY_DN27102_c0_g1_i1_len_543_241945.p1_type_5prime_partial_len_105 | ---NA--- | | |
| TRINITY_DN23558_c1_g1_i2_len_2221_155545.p3_type_complete_len_105 | ---NA--- | | |
| TRINITY_DN25712_c0_g1_i1_len_316_15820.p1_type_internal_len_106 | ---NA--- | | |
| TRINITY_DN27880_c0_g1_i1_len_316_311094.p1_type_internal_len_106 | ---NA--- | | |
| TRINITY_DN38524_c0_g1_i1_len_316_213040.p1_type_internal_len_106 | ---NA--- | | |
| TRINITY_DN2163_c0_g1_i1_len_391_208442.p1_type_5prime_partial_len_105 | ---NA--- | | |
| TRINITY_DN23987_c0_g1_i1_len_2086_59160.p2_type_complete_len_105 | ---NA--- | | |
| TRINITY_DN30637_c0_g1_i1_len_399_246424.p1_type_5prime_partial_len_105 | ---NA--- | | |
| TRINITY_DN21017_c0_g1_i1_len_4361_121827.p3_type_complete_len_105 | KDR21878.1TAR DNA-binding protein 43 | F:GO:0003677; F:GO:0003723 | |

| | | | |
|---|---|---|---|
| TRINITY_DN20017_c0_g1_i1_len_578_202245.p1_type_5prime_partial_len_105 | ---NA--- | | |
| TRINITY_DN28710_c0_g1_i1_len_318_310999.p1_type_internal_len_106 | XP_024939571.1arf-GAP with coiled-coil, ANK repeat and PH domain-containing protein 2 isoform X2 | | |
| TRINITY_DN20794_c0_g1_i1_len_811_162308.p1_type_complete_len_105 | ---NA--- | | |
| TRINITY_DN21370_c0_g1_i1_len_1100_3075.p2_type_complete_len_105 | ---NA--- | | |
| TRINITY_DN11879_c0_g1_i1_len_317_311011.p1_type_internal_len_106 | ---NA--- | | |
| TRINITY_DN15623_c0_g1_i1_len_1633_267297.p2_type_5prime_partial_len_105 | ---NA--- | | |
| TRINITY_DN383_c0_g1_i1_len_318_212882.p1_type_internal_len_106 | XP_022196874.1tectonin beta-propeller repeat-containing protein 2-like | | |
| TRINITY_DN9537_c0_g1_i1_len_318_212881.p1_type_internal_len_106 | ---NA--- | | |
| TRINITY_DN27024_c0_g1_i1_len_389_135847.p1_type_5prime_partial_len_105 | ---NA--- | | |
| TRINITY_DN15293_c0_g1_i1_len_953_33744.p2_type_complete_len_105 | ---NA--- | | |
| TRINITY_DN12812_c0_g1_i1_len_317_311047.p1_type_internal_len_106 | ---NA--- | | |
| TRINITY_DN20267_c0_g1_i1_len_1336_158554.p2_type_complete_len_105 | ---NA--- | | |
| TRINITY_DN1774_c0_g1_i1_len_766_5122.p1_type_complete_len_105 | ---NA--- | | |
| TRINITY_DN27416_c0_g1_i1_len_317_311018.p1_type_internal_len_106 | XP_022313894.1 sodium/hydrogen exchanger 9B2-like | | |
| TRINITY_DN30925_c0_g1_i1_len_3470_187967.p4_type_3prime_partial_len_106 | ---NA--- | | |
| TRINITY_DN12339_c0_g1_i1_len_318_212876.p2_type_internal_len_106 | ACP19563.1masquerade-like serine proteinase-like protein 3 | F:GO:0004252; P:GO:0006508 | EC:3.4.21 |

| | | | |
|---|---|---|---|
| TRINITY_DN14634_c0_g1_i1_len_388_135860.p1_type_5prime_partial_len_105 | ---NA--- | | |
| TRINITY_DN30667_c0_g1_i1_len_1038_196212.p2_type_5prime_partial_len_105 | ---NA--- | | |
| TRINITY_DN16150_c0_g1_i1_len_529_8247.p1_type_5prime_partial_len_105 | ---NA--- | | |
| TRINITY_DN23460_c0_g1_i2_len_1020_196393.p2_type_complete_len_105 | KFM73828.1Carboxypeptidase A2 | F:GO:0004181; P:GO:0006508; F:GO:0008270 | EC:3.4.17 |
| TRINITY_DN25675_c0_g1_i6_len_5010_227780.p4_type_complete_len_105 | ---NA--- | | |
| TRINITY_DN15206_c0_g1_i1_len_424_245505.p1_type_5prime_partial_len_105 | ---NA--- | | |
| TRINITY_DN12369_c0_g1_i1_len_317_213001.p2_type_internal_len_106 | ---NA--- | | |
| TRINITY_DN20720_c0_g1_i1_len_1705_123710.p2_type_complete_len_105 | XP_018012526.1 longitudinals lacking protein, isoforms A/B/D/L-like isoform X12 | | |
| TRINITY_DN8003_c0_g1_i1_len_348_74882.p1_type_5prime_partial_len_105 | ---NA--- | | |
| TRINITY_DN17855_c0_g1_i1_len_948_236542.p2_type_complete_len_105 | ---NA--- | | |
| TRINITY_DN2332_c0_g1_i1_len_596_240868.p2_type_5prime_partial_len_105 | ---NA--- | | |
| TRINITY_DN22204_c0_g1_i3_len_1647_1352.p2_type_5prime_partial_len_105 | ---NA--- | | |
| TRINITY_DN20581_c0_g1_i1_len_524_203550.p2_type_5prime_partial_len_105 | ---NA--- | | |
| TRINITY_DN4149_c0_g1_i1_len_317_212992.p1_type_internal_len_106 | ---NA--- | | |
| TRINITY_DN35757_c0_g1_i1_len_317_212995.p1_type_internal_len_106 | ---NA--- | | |
| TRINITY_DN5035_c0_g1_i2_len_316_15834.p1_type_internal_len_106 | ---NA--- | | |

| | | | |
|---|---|---|---|
| TRINITY_DN27404_c0_g1_i1_len_327_250149.p1_type_5prime_partial_len_105 | ---NA--- | | |
| TRINITY_DN25256_c0_g1_i1_len_4008_228177.p4_type_complete_len_105 | ---NA--- | | |
| TRINITY_DN13757_c0_g1_i1_len_317_212991.p1_type_internal_len_106 | ---NA--- | | |
| TRINITY_DN14516_c0_g1_i2_len_554_100228.p2_type_5prime_partial_len_105 | AEU11366.1Broad-complex protein isoform 4 | F:GO:0003676 | |
| TRINITY_DN13436_c0_g1_i1_len_1178_234836.p1_type_5prime_partial_len_105 | ---NA--- | | |
| TRINITY_DN16335_c0_g1_i3_len_1948_92257.p4_type_complete_len_105 | ---NA--- | | |
| TRINITY_DN15146_c0_g1_i1_len_363_137069.p1_type_5prime_partial_len_105 | ALO17555.1Neuroparsin | | |
| TRINITY_DN23697_c0_g1_i1_len_1178_234841.p1_type_5prime_partial_len_105 | ---NA--- | | |
| TRINITY_DN18627_c0_g1_i1_len_419_207193.p1_type_5prime_partial_len_105 | ---NA--- | | |
| TRINITY_DN21170_c0_g1_i1_len_628_66659.p1_type_5prime_partial_len_105 | ---NA--- | | |
| TRINITY_DN31097_c3_g5_i1_len_318_212890.p1_type_internal_len_106 | ---NA--- | | |
| TRINITY_DN9559_c0_g1_i1_len_318_310945.p1_type_internal_len_106 | ---NA--- | | |
| TRINITY_DN3041_c0_g1_i1_len_316_213029.p1_type_internal_len_106 | ---NA--- | | |
| TRINITY_DN18088_c0_g1_i3_len_379_136267.p1_type_5prime_partial_len_105 | ---NA--- | | |
| TRINITY_DN12033_c0_g1_i1_len_344_211108.p1_type_5prime_partial_len_105 | ---NA--- | | |
| TRINITY_DN22330_c0_g1_i1_len_3246_154093.p1_type_complete_len_105 | ---NA--- | | |

| | | | |
|---|---|---|---|
| TRINITY_DN13002_c0_g1_i1_len_820_162210.p1_type_complete_len_105 | XP_023299268.1cytochrome c oxidase protein 20 homolog | C:GO:0016020 | |
| TRINITY_DN8275_c0_g1_i1_len_711_97951.p1_type_complete_len_105 | ABO43764.1Toll protein | P:GO:0007165; C:GO:0016021; P:GO:0045087 | |
| TRINITY_DN18000_c0_g1_i1_len_1080_235514.p2_type_5prime_partial_len_105 | ---NA--- | | |
| TRINITY_DN21352_c0_g1_i3_len_594_201896.p2_type_complete_len_105 | ---NA--- | | |
| TRINITY_DN18239_c0_g1_i1_len_595_240898.p1_type_3prime_partial_len_106 | ---NA--- | | |
| TRINITY_DN30377_c0_g1_i1_len_496_304264.p4_type_5prime_partial_len_105 | AFC76411.1sarco/endoplasmic reticulum Ca2+-ATPase | F:GO:0005388; F:GO:0005524; C:GO:0016021; C:GO:0033017; P:GO:0070588; P:GO:0099132 | EC:3.6.1.3; EC:3.6.3.8; EC:3.6.1.15 |
| TRINITY_DN38013_c0_g1_i1_len_316_213012.p1_type_internal_len_106 | XP_018021037.1 endochitinase A-like | | |
| TRINITY_DN6898_c0_g1_i1_len_406_72100.p1_type_5prime_partial_len_105 | ---NA--- | | |
| TRINITY_DN25226_c0_g1_i1_len_5082_227764.p2_type_3prime_partial_len_106 | ---NA--- | | |
| TRINITY_DN7924_c0_g1_i1_len_332_107640.p1_type_5prime_partial_len_105 | ---NA--- | | |
| TRINITY_DN9640_c0_g1_i1_len_446_277462.p1_type_5prime_partial_len_105 | ---NA--- | | |
| TRINITY_DN10142_c0_g1_i1_len_319_310923.p1_type_internal_len_106 | ---NA--- | | |
| TRINITY_DN14330_c0_g1_i1_len_395_208237.p1_type_complete_len_105 | ---NA--- | | |
| TRINITY_DN25394_c0_g1_i2_len_3603_228402.p2_type_complete_len_105 | ---NA--- | | |
| TRINITY_DN17125_c0_g1_i1_len_315_213148.p1_type_internal_len_106 | CCW43203.1serine proteinase | F:GO:0004252; P:GO:0006508 | |
| TRINITY_DN12824_c0_g1_i1_len_803_97016.p1_type_complete_len_105 | ---NA--- | | |

| | | | |
|---|---|---|---|
| TRINITY_DN4722_c0_g1_i1_len_318_76710.p1_type_internal_len_106 | RFN53836.1nfx1-type zinc finger-containing protein 1 | F:GO:0046872 | |
| TRINITY_DN11785_c0_g1_i1_len_367_247912.p1_type_3prime_partial_len_106 | ---NA--- | | |
| TRINITY_DN28058_c1_g1_i2_len_1108_195634.p2_type_5prime_partial_len_105 | FAA00226.1TPA: zinc finger protein | F:GO:0003676; P:GO:0015074 | |
| TRINITY_DN6867_c0_g1_i1_len_319_212830.p1_type_internal_len_106 | ---NA--- | | |
| TRINITY_DN7910_c0_g1_i1_len_318_76660.p3_type_internal_len_106 | XP_018015601.1 mucin-17-like | P:GO:0005975; F:GO:0016810 | |
| TRINITY_DN17785_c0_g1_i1_len_317_76746.p1_type_internal_len_106 | ---NA--- | | |
| TRINITY_DN35853_c0_g1_i1_len_796_198669.p1_type_complete_len_105 | ---NA--- | | |
| TRINITY_DN4446_c0_g1_i1_len_317_76771.p1_type_internal_len_106 | ---NA--- | | |
| TRINITY_DN25266_c0_g1_i1_len_367_73842.p1_type_5prime_partial_len_105 | ---NA--- | | |
| TRINITY_DN3199_c0_g1_i1_len_317_76745.p2_type_internal_len_106 | XP_018023310.1 protein O-linked-mannose beta-1,2-N-acetylglucosaminyltransferase 1-like | | |
| TRINITY_DN35160_c0_g1_i1_len_317_76737.p1_type_internal_len_106 | ---NA--- | | |
| TRINITY_DN21867_c0_g1_i1_len_880_197704.p1_type_complete_len_105 | ---NA--- | | |
| TRINITY_DN24185_c0_g1_i1_len_1514_193026.p2_type_5prime_partial_len_105 | ---NA--- | | |
| TRINITY_DN9061_c0_g1_i1_len_410_207590.p1_type_5prime_partial_len_105 | ---NA--- | | |
| TRINITY_DN25301_c0_g1_i1_len_498_132404.p2_type_5prime_partial_len_105 | AYF59252.1beta-arrestin 1 | P:GO:0007165 | |

| | | | |
|---|---|---|---|
| TRINITY_DN22815_c0_g1_i4_len_3319_57998.p1_type_complete_len_820 | XP_018027019.1 tyrosine-protein phosphatase non-receptor type 13-like isoform X4 | | |
| TRINITY_DN23515_c0_g1_i1_len_3054_122094.p1_type_complete_len_820 | PNF38939.1RAD50-interacting protein 1 | C:GO:0005783; P:GO:0048193 | |
| TRINITY_DN19607_c0_g1_i1_len_2726_265215.p1_type_complete_len_820 | XP_017784029.1 RNA polymerase II subunit A C-terminal domain phosphatase | | |
| TRINITY_DN27019_c0_g1_i1_len_3328_188097.p1_type_complete_len_820 | XP_022086457.1uncharacterized aarF domain-containing protein kinase 2-like | | |
| 2924\|_\|c36158_f3p8_2460_isoform_c36158_full_length_coverage_3_non_full_length_coverage_8_isoform_length_2460_18910 | XP_019626034.1 mini-chromosome maintenance complex-binding protein-like | | |
| 2925\|_\|c4182_f2p3_2460_isoform_c4182_full_length_coverage_2_non_full_length_coverage_3_isoform_length_2460_16833 | ---NA--- | | |
| 2926\|_\|c31493_f5p2_2460_isoform_c31493_full_length_coverage_5_non_full_length_coverage_2_isoform_length_2460_23201 | XP_018015976.1 zinc finger protein 236-like isoform X4 | | |
| 2927\|_\|c2518_f4p2_2460_isoform_c2518_full_length_coverage_4_non_full_length_coverage_2_isoform_length_2460_21008 | OXA59575.1D-2-hydroxyglutarate dehydrogenase, mitochondrial | F:GO:0016491; P:GO:0055114; F:GO:0071949 | |
| 2928\|_\|c4640_f2p4_2460_isoform_c4640_full_length_coverage_2_non_full_length_coverage_4_isoform_length_2460_16874 | ---NA--- | | |
| 2929\|_\|c5312_f4p4_2459_isoform_c5312_full_length_coverage_4_non_full_length_coverage_4_isoform_length_2459_12465 | XP_019621786.1PREDICTED: uncharacterized protein LOC109468021 | F:GO:0003676; P:GO:0006139; F:GO:0008408; P:GO:0090305 | |
| TRINITY_DN31828_c0_g1_i1_len_319_15626.p1_type_internal_len_106 | ---NA--- | | |
| TRINITY_DN16477_c1_g1_i1_len_717_272616.p1_type_5prime_partial_len_105 | ---NA--- | | |
| TRINITY_DN23823_c0_g1_i1_len_1746_191877.p2_type_5prime_partial_len_105 | ---NA--- | | |

| | | | |
|---|---|---|---|
| TRINITY_DN8522_c0_g1_i1_len_319_76611.p2_type_internal_len_106 | ---NA--- | | |
| TRINITY_DN22576_c0_g3_i1_len_319_76604.p1_type_internal_len_106 | ---NA--- | | |
| TRINITY_DN5196_c0_g1_i1_len_706_163471.p1_type_complete_len_105 | ---NA--- | | |
| TRINITY_DN42011_c0_g1_i1_len_315_213093.p1_type_internal_len_106 | ---NA--- | | |
| TRINITY_DN37279_c0_g1_i1_len_319_212837.p1_type_internal_len_106 | XP_023933033.1dihydropyrimidinase isoform X2 | C:GO:0005737; F:GO:0016810 | |
| TRINITY_DN31343_c0_g1_i1_len_315_213127.p2_type_internal_len_106 | ---NA--- | | |
| TRINITY_DN34396_c0_g1_i1_len_566_241458.p1_type_3prime_partial_len_106 | ---NA--- | | |
| TRINITY_DN30985_c6_g3_i2_len_934_197148.p1_type_5prime_partial_len_105 | ---NA--- | | |
| TRINITY_DN14155_c0_g1_i1_len_480_39170.p2_type_5prime_partial_len_105 | ---NA--- | | |
| TRINITY_DN37952_c0_g1_i1_len_319_212819.p1_type_internal_len_106 | ---NA--- | | |
| TRINITY_DN16139_c0_g1_i1_len_397_208157.p1_type_complete_len_105 | ---NA--- | | |
| TRINITY_DN37935_c0_g1_i1_len_319_212821.p1_type_internal_len_106 | ---NA--- | | |
| TRINITY_DN19338_c0_g1_i2_len_1184_195076.p2_type_5prime_partial_len_105 | ---NA--- | | |
| TRINITY_DN35430_c0_g1_i1_len_315_213115.p1_type_internal_len_106 | ---NA--- | | |
| TRINITY_DN6387_c0_g1_i1_len_315_213114.p1_type_internal_len_106 | ---NA--- | | |
| TRINITY_DN13783_c0_g1_i1_len_461_133354.p2_type_5prime_partial_len_105 | KYB29688.1Epidermal growth factor receptor kinase substrate 8-like protein 2 | F:GO:0016301; P:GO:0016310 | |

| | | | |
|---|---|---|---|
| TRINITY_DN30943_c0_g1_i1_len_356_248460.p1_type_5prime_partial_len_105 | ---NA--- | | |
| TRINITY_DN19310_c0_g1_i1_len_870_4348.p2_type_5prime_partial_len_105 | ---NA--- | | |
| TRINITY_DN17112_c0_g1_i1_len_546_203006.p1_type_3prime_partial_len_106 | ---NA--- | | |
| TRINITY_DN35432_c0_g1_i1_len_693_200113.p1_type_complete_len_105 | ---NA--- | | |
| TRINITY_DN24078_c1_g1_i6_len_437_134053.p1_type_5prime_partial_len_105 | ---NA--- | | |
| TRINITY_DN1505_c0_g1_i1_len_356_172162.p2_type_5prime_partial_len_105 | ---NA--- | | |
| TRINITY_DN18900_c0_g1_i1_len_750_162931.p1_type_5prime_partial_len_105 | ---NA--- | | |
| TRINITY_DN38837_c0_g1_i1_len_315_213139.p1_type_internal_len_106 | PIK38217.1putative aminopeptidase O-like | F:GO:0004177; F:GO:0008237 | EC:3.4.11 |
| TRINITY_DN27489_c0_g1_i1_len_315_76922.p1_type_internal_len_106 | ---NA--- | | |
| TRINITY_DN23756_c0_g1_i1_len_2704_229421.p3_type_5prime_partial_len_105 | ---NA--- | | |
| TRINITY_DN34392_c0_g1_i1_len_322_212614.p1_type_5prime_partial_len_105 | ---NA--- | | |
| TRINITY_DN28920_c0_g1_i1_len_315_76879.p2_type_internal_len_106 | XP_018896580.1 frizzled-4-like | F:GO:0004888; P:GO:0007166; P:GO:0007275; C:GO:0016020; C:GO:0016021; P:GO:0016055; F:GO:0042813 | |
| TRINITY_DN31842_c0_g1_i1_len_315_213143.p1_type_internal_len_106 | ---NA--- | | |
| TRINITY_DN17129_c0_g1_i3_len_316_76863.p2_type_internal_len_106 | ---NA--- | | |
| TRINITY_DN4421_c0_g1_i1_len_331_211986.p1_type_5prime_partial_len_105 | ---NA--- | | |

| | | | |
|---|---|---|---|
| TRINITY_DN1081_c0_g1_i1_len_315_76885.p1_type_internal_len_106 | ---NA--- | | |
| TRINITY_DN32747_c0_g1_i1_len_316_213063.p1_type_internal_len_106 | ---NA--- | | |
| TRINITY_DN25792_c0_g1_i1_len_574_67542.p1_type_5prime_partial_len_105 | ---NA--- | | |
| TRINITY_DN9964_c0_g1_i1_len_316_213064.p1_type_internal_len_106 | ---NA--- | | |
| TRINITY_DN12697_c0_g1_i2_len_483_9244.p1_type_5prime_partial_len_105 | ---NA--- | | |
| TRINITY_DN26483_c0_g1_i1_len_461_305125.p1_type_complete_len_105 | ---NA--- | | |
| TRINITY_DN32019_c0_g1_i1_len_315_311157.p1_type_internal_len_106 | ---NA--- | | |
| TRINITY_DN8171_c0_g1_i1_len_316_15812.p2_type_internal_len_106 | ---NA--- | | |
| TRINITY_DN10106_c0_g1_i1_len_386_246989.p1_type_5prime_partial_len_105 | ---NA--- | | |
| TRINITY_DN3492_c0_g1_i1_len_316_213068.p1_type_internal_len_106 | XP_016987954.1 nicotinate phosphoribosyltransferase | | |
| TRINITY_DN23674_c0_g1_i4_len_1308_194209.p2_type_complete_len_105 | ---NA--- | | |
| TRINITY_DN11684_c0_g1_i1_len_316_213042.p1_type_internal_len_106 | ---NA--- | | |
| TRINITY_DN26857_c0_g1_i1_len_339_249478.p1_type_5prime_partial_len_105 | ---NA--- | | |
| TRINITY_DN24820_c0_g1_i1_len_316_311124.p1_type_internal_len_106 | KYP65095.1Retrovirus-related Pol polyprotein from transposon 17.6 | | |
| TRINITY_DN25332_c0_g1_i1_len_317_15764.p1_type_internal_len_106 | EWM27401.1pol polyprotein | | |
| TRINITY_DN41462_c0_g1_i1_len_316_213052.p1_type_internal_len_106 | ---NA--- | | |

| | | | |
|---|---|---|---|
| TRINITY_DN38248_c0_g1_i1_len_316_213084.p1_type_5prime_partial_len_105 | ---NA--- | | |
| TRINITY_DN24991_c0_g1_i1_len_315_15879.p2_type_internal_len_106 | ---NA--- | | |
| TRINITY_DN24237_c0_g1_i2_len_3092_228891.p4_type_5prime_partial_len_105 | ---NA--- | | |
| TRINITY_DN33285_c0_g1_i1_len_324_76256.p1_type_5prime_partial_len_105 | ---NA--- | | |
| TRINITY_DN9589_c0_g1_i1_len_556_202734.p1_type_3prime_partial_len_106 | ---NA--- | | |
| TRINITY_DN12187_c0_g1_i1_len_319_15615.p1_type_internal_len_106 | ---NA--- | | |
| TRINITY_DN9009_c0_g1_i2_len_388_41926.p1_type_5prime_partial_len_105 | ---NA--- | | |
| TRINITY_DN31095_c0_g1_i1_len_317_15768.p1_type_internal_len_106 | ---NA--- | | |
| TRINITY_DN20170_c0_g1_i1_len_1113_195597.p1_type_complete_len_105 | ---NA--- | | |
| TRINITY_DN12804_c0_g1_i1_len_783_162579.p1_type_5prime_partial_len_105 | ---NA--- | | |
| TRINITY_DN2814_c0_g1_i1_len_357_210302.p1_type_5prime_partial_len_105 | ---NA--- | | |
| TRINITY_DN5173_c0_g1_i1_len_316_213080.p1_type_internal_len_106 | ---NA--- | | |
| TRINITY_DN21409_c0_g1_i1_len_740_199440.p2_type_5prime_partial_len_105 | ---NA--- | | |
| TRINITY_DN23041_c0_g1_i2_len_772_238274.p3_type_complete_len_105 | ---NA--- | | |
| TRINITY_DN18922_c0_g1_i2_len_742_97617.p2_type_5prime_partial_len_105 | ---NA--- | | |
| TRINITY_DN17272_c0_g1_i2_len_316_213081.p1_type_internal_len_106 | ---NA--- | | |

| | | | |
|---|---|---|---|
| TRINITY_DN15537_c0_g1_i1_len_1174_159475.p2_type_3prime_partial_len_106 | ---NA--- | | |
| TRINITY_DN20291_c0_g1_i1_len_2060_266158.p2_type_3prime_partial_len_106 | ---NA--- | | |
| TRINITY_DN2570_c0_g1_i1_len_317_212944.p2_type_internal_len_106 | ---NA--- | | |
| TRINITY_DN10552_c0_g1_i1_len_862_64060.p2_type_5prime_partial_len_105 | ---NA--- | | |
| TRINITY_DN31837_c0_g1_i1_len_317_108633.p1_type_internal_len_106 | ANQ44675.1heat shock protein 70 kDa | F:GO:0005524 | |
| TRINITY_DN2871_c0_g1_i1_len_318_174313.p1_type_internal_len_106 | ---NA--- | | |
| TRINITY_DN42307_c0_g1_i1_len_487_204641.p1_type_5prime_partial_len_105 | ---NA--- | | |
| TRINITY_DN34506_c0_g1_i1_len_319_250721.p2_type_internal_len_106 | ---NA--- | | |
| TRINITY_DN2063_c0_g1_i1_len_316_282980.p2_type_internal_len_106 | ---NA--- | | |
| TRINITY_DN14100_c0_g1_i3_len_340_211347.p1_type_5prime_partial_len_105 | ---NA--- | | |
| TRINITY_DN25306_c0_g1_i1_len_316_282958.p1_type_internal_len_106 | CDW53334.1Succinate-semialdehyde dehydrogenase, mitochondri al | F:GO:0016620; P:GO:0055114 | |
| TRINITY_DN21114_c0_g1_i1_len_1078_160122.p1_type_complete_len_105 | ---NA--- | | |
| TRINITY_DN5850_c0_g1_i1_len_317_212943.p1_type_internal_len_106 | ---NA--- | | |
| TRINITY_DN3566_c0_g1_i1_len_316_108734.p2_type_internal_len_106 | ---NA--- | | |
| TRINITY_DN3566_c0_g1_i1_len_316_108734.p1_type_internal_len_106 | ---NA--- | | |
| TRINITY_DN12778_c0_g1_i1_len_319_282773.p1_type_internal_len_106 | ---NA--- | | |

| | | | |
|---|---|---|---|
| TRINITY_DN32951_c0_g1_i1_len_316_108726.p1_type_internal_len_106 | ---NA--- | | |
| TRINITY_DN34266_c0_g1_i1_len_318_174285.p1_type_internal_len_106 | ---NA--- | | |
| TRINITY_DN23116_c0_g1_i1_len_483_167470.p1_type_5prime_partial_len_105 | XP_013985963.1 zinc finger protein 345-like isoform X2 | F:GO:0003676 | |
| TRINITY_DN1637_c0_g1_i1_len_318_174289.p1_type_internal_len_106 | ---NA--- | | |
| TRINITY_DN236_c0_g1_i1_len_315_15851.p1_type_internal_len_106 | ---NA--- | | |
| TRINITY_DN15105_c0_g1_i1_len_606_201644.p1_type_3prime_partial_len_106 | ---NA--- | | |
| TRINITY_DN7299_c0_g1_i1_len_319_250719.p1_type_internal_len_106 | XP_019411343.1 SEC23-interacting protein isoform X2 | | |
| TRINITY_DN7062_c0_g1_i1_len_316_108710.p1_type_internal_len_106 | ---NA--- | | |
| TRINITY_DN28656_c0_g1_i1_len_316_108707.p1_type_internal_len_106 | ---NA--- | | |
| TRINITY_DN29029_c0_g1_i1_len_318_174274.p1_type_internal_len_106 | ---NA--- | | |
| TRINITY_DN24573_c0_g1_i1_len_318_174278.p1_type_internal_len_106 | ---NA--- | | |
| TRINITY_DN10437_c0_g1_i1_len_318_174282.p1_type_internal_len_106 | ---NA--- | | |
| TRINITY_DN21602_c1_g1_i1_len_316_108701.p2_type_internal_len_106 | ---NA--- | | |
| TRINITY_DN36942_c0_g1_i1_len_316_250867.p2_type_internal_len_106 | XP_011143807.1general transcription factor 3C polypeptide 1 | | |
| TRINITY_DN15821_c0_g1_i1_len_433_206613.p1_type_3prime_partial_len_106 | ---NA--- | | |
| TRINITY_DN26567_c0_g1_i1_len_315_45440.p1_type_internal_len_106 | ---NA--- | | |

<table>
<tr><td>TRINITY_DN38114_c0_g1_i1_len_318_250752.p1_type_internal_len_106</td><td>---NA---</td><td></td><td></td></tr>
<tr><td>TRINITY_DN21856_c0_g1_i1_len_315_283001.p1_type_internal_len_106</td><td>KZS05987.1 WSCD family member</td><td>F:GO:0008146; C:GO:0016020; C:GO:0016021</td><td></td></tr>
<tr><td>2931|_|c1886_f3p0_2458_isoform_c1886_full_length_coverage_3_non_full_length_coverage_0_isoform_length_2458_11537</td><td>XP_018020703.1 ATP-dependent DNA helicase Q1-like</td><td></td><td></td></tr>
<tr><td>2932|_|c2553_f2p7_2458_isoform_c2553_full_length_coverage_2_non_full_length_coverage_7_isoform_length_2458_21036</td><td>XP_018022499.1 EH domain-containing protein 1-like isoform X1</td><td>F:GO:0005509; F:GO:0005525; C:GO:0005623; P:GO:0032456</td><td></td></tr>
<tr><td>TRINITY_DN25340_c0_g1_i1_len_3704_228343.p1_type_complete_len_819</td><td>---NA---</td><td></td><td></td></tr>
<tr><td>2935|_|c2484_f3p0_2457_isoform_c2484_full_length_coverage_3_non_full_length_coverage_0_isoform_length_2457_20976</td><td>---NA---</td><td></td><td></td></tr>
<tr><td>2936|_|c7748_f3p0_2457_isoform_c7748_full_length_coverage_3_non_full_length_coverage_0_isoform_length_2457_21721</td><td>---NA---</td><td></td><td></td></tr>
<tr><td>2937|_|c12545_f3p1_2456_isoform_c12545_full_length_coverage_3_non_full_length_coverage_1_isoform_length_2456_13075</td><td>---NA---</td><td></td><td></td></tr>
<tr><td>2938|_|c1637_f12p8_2456_isoform_c1637_full_length_coverage_12_non_full_length_coverage_8_isoform_length_2456_15931</td><td>XP_026481802.1actin-interacting protein 1</td><td></td><td></td></tr>
<tr><td>2941|_|c22736_f2p2_2456_isoform_c22736_full_length_coverage_2_non_full_length_coverage_2_isoform_length_2456_18270</td><td>XP_018023714.1  RNA-binding protein 25-like</td><td></td><td></td></tr>
<tr><td>2942|_|c973_f27p11_2455_isoform_c973_full_length_coverage_27_non_full_length_coverage_11_isoform_length_2455_15798</td><td>XP_015511362.1PREDICTED: uncharacterized protein LOC107218115</td><td>F:GO:0004601; P:GO:0006979; F:GO:0020037; P:GO:0055114; P:GO:0098869</td><td></td></tr>
<tr><td>2943|_|c13535_f2p4_2454_isoform_c13535_full_length_coverage_2_non_full_length_coverage_4_isoform_length_2454_13145</td><td>---NA---</td><td></td><td></td></tr>
</table>

| | | | |
|---|---|---|---|
| TRINITY_DN21715_c0_g1_i1_len_1629_157317.p2_type_3prime_partial_len_106 | ---NA--- | | |
| TRINITY_DN31640_c0_g1_i1_len_316_250868.p1_type_internal_len_106 | ---NA--- | | |
| TRINITY_DN30421_c0_g1_i1_len_316_174418.p1_type_internal_len_106 | ---NA--- | | |
| TRINITY_DN35208_c0_g1_i1_len_318_250753.p1_type_internal_len_106 | ---NA--- | | |
| TRINITY_DN21573_c0_g1_i2_len_433_206599.p1_type_5prime_partial_len_105 | PIK36482.1endonuclease-reverse transcriptase | F:GO:0003964; F:GO:0004519; P:GO:0006278; P:GO:0090305 | |
| TRINITY_DN28665_c0_g1_i1_len_315_139845.p1_type_internal_len_106 | AJP31314.1broad-complex-like protein | F:GO:0003676 | |
| TRINITY_DN157_c0_g1_i1_len_315_45448.p1_type_internal_len_106 | KRY16906.1Retrovirus-related Pol polyprotein from transposon | | |
| TRINITY_DN27150_c0_g1_i1_len_317_174396.p1_type_internal_len_106 | XP_018091160.1 peroxisomal N(1)-acetyl-spermine/spermidine oxidase-like | F:GO:0016491; P:GO:0055114 | |
| TRINITY_DN23784_c0_g1_i1_len_317_174403.p1_type_internal_len_106 | ---NA--- | | |
| TRINITY_DN4632_c0_g1_i1_len_315_139850.p1_type_internal_len_106 | ---NA--- | | |
| TRINITY_DN21505_c0_g1_i2_len_1532_157667.p2_type_5prime_partial_len_105 | ---NA--- | | |
| TRINITY_DN12603_c0_g2_i1_len_316_174415.p1_type_internal_len_106 | XP_018942668.1 gastrula zinc finger protein XlCGF57.1-like | | |
| TRINITY_DN29597_c0_g1_i2_len_2114_190507.p3_type_complete_len_105 | ---NA--- | | |
| TRINITY_DN34217_c0_g1_i1_len_318_174331.p1_type_internal_len_106 | EEC12352.1hypothetical protein IscW_ISCW020853, partial | C:GO:0005886; P:GO:0007155; C:GO:0016021 | |
| TRINITY_DN11419_c0_g1_i1_len_318_108554.p1_type_internal_len_106 | ---NA--- | | |
| TRINITY_DN30711_c0_g1_i1_len_318_108577.p2_type_5prime_partial_len_105 | ---NA--- | | |

| | | | |
|---|---|---|---|
| TRINITY_DN31246_c0_g1_i1_len_318_250739.p1_type_internal_len_106 | ---NA--- | | |
| TRINITY_DN14016_c0_g1_i1_len_453_244447.p2_type_5prime_partial_len_105 | ---NA--- | | |
| TRINITY_DN605_c0_g1_i1_len_318_174323.p1_type_internal_len_106 | ---NA--- | | |
| TRINITY_DN20807_c0_g1_i1_len_451_244519.p1_type_5prime_partial_len_105 | ---NA--- | | |
| TRINITY_DN23148_c0_g1_i1_len_318_45253.p2_type_5prime_partial_len_105 | ---NA--- | | |
| TRINITY_DN18425_c0_g1_i1_len_542_203093.p1_type_5prime_partial_len_105 | ---NA--- | | |
| TRINITY_DN32442_c0_g1_i1_len_316_250901.p3_type_internal_len_106 | ---NA--- | | |
| TRINITY_DN12023_c0_g1_i1_len_1032_270012.p3_type_complete_len_105 | ---NA--- | | |
| TRINITY_DN20462_c0_g1_i1_len_322_45031.p1_type_5prime_partial_len_105 | ---NA--- | | |
| TRINITY_DN25372_c0_g1_i1_len_317_282893.p1_type_internal_len_106 | ASW35106.1FH1/FH2 domain-containing protein 1 | P:GO:0007015 | |
| TRINITY_DN6006_c0_g1_i2_len_315_250984.p2_type_internal_len_106 | ---NA--- | | |
| TRINITY_DN27765_c0_g1_i1_len_316_45353.p3_type_internal_len_106 | ---NA--- | | |
| TRINITY_DN34759_c0_g1_i1_len_316_250908.p1_type_internal_len_106 | XP_025994356.1chloride channel protein 2 isoform X4 | F:GO:0005247; C:GO:0016021; P:GO:1902476 | |
| TRINITY_DN16245_c0_g1_i1_len_317_15739.p1_type_internal_len_106 | ---NA--- | | |
| TRINITY_DN36337_c0_g1_i1_len_318_212924.p1_type_internal_len_106 | XP_011563152.1 arf-GAP with coiled-coil, ANK repeat and PH domain-containing protein 2-like | | |
| TRINITY_DN22197_c0_g1_i1_len_498_204320.p1_type_complete_len_105 | ---NA--- | | |

| | | | |
|---|---|---|---|
| TRINITY_DN37273_c0_g1_i1_len_319_250672.p1_type_internal_len_106 | ---NA--- | | |
| TRINITY_DN16329_c0_g1_i3_len_998_298821.p3_type_3prime_partial_len_106 | ---NA--- | | |
| TRINITY_DN30836_c0_g1_i1_len_316_250897.p1_type_internal_len_106 | ACC62400.2ribonuclease III | F:GO:0003723; F:GO:0004525; P:GO:0006396; P:GO:0016075; P:GO:0031047; P:GO:0090502 | EC:3.1.30; EC:3.1.26; EC:3.1.26.3 |
| TRINITY_DN21018_c0_g1_i1_len_879_96410.p2_type_complete_len_105 | ---NA--- | | |
| TRINITY_DN5888_c0_g1_i1_len_315_250945.p1_type_internal_len_106 | AUI80370.1cryptochrome 1 | | |
| TRINITY_DN16596_c0_g1_i1_len_316_45344.p1_type_5prime_partial_len_105 | ---NA--- | | |
| TRINITY_DN31399_c0_g1_i1_len_315_250952.p1_type_internal_len_106 | ---NA--- | | |
| TRINITY_DN30212_c0_g1_i1_len_316_250925.p1_type_internal_len_106 | ---NA--- | | |
| TRINITY_DN25441_c0_g1_i1_len_318_282868.p1_type_internal_len_106 | ---NA--- | | |
| TRINITY_DN8472_c0_g1_i1_len_316_45328.p1_type_internal_len_106 | XP_014881147.1 zinc finger protein OZF-like | | |
| TRINITY_DN16865_c1_g1_i1_len_316_250930.p1_type_internal_len_106 | ---NA--- | | |
| TRINITY_DN27109_c0_g1_i1_len_316_250940.p1_type_internal_len_106 | ---NA--- | | |
| TRINITY_DN20000_c0_g1_i1_len_414_207436.p1_type_3prime_partial_len_106 | ---NA--- | | |
| TRINITY_DN23293_c0_g1_i1_len_317_15743.p1_type_internal_len_106 | ---NA--- | | |
| TRINITY_DN3308_c0_g1_i1_len_317_45317.p1_type_internal_len_106 | XP_018006892.1PREDICTED: uncharacterized protein ZK1073.1-like isoform X3 | | |

| | | | |
|---|---|---|---|
| TRINITY_DN31136_c0_g1_i1_len_4187_187485.p1_type_complete_len_105 | ---NA--- | | |
| TRINITY_DN34424_c0_g1_i1_len_318_212928.p1_type_internal_len_106 | ---NA--- | | |
| TRINITY_DN34490_c0_g1_i1_len_316_250920.p1_type_internal_len_106 | ---NA--- | | |
| TRINITY_DN17721_c0_g1_i1_len_670_239646.p1_type_complete_len_105 | ---NA--- | | |
| TRINITY_DN26228_c0_g1_i2_len_316_45385.p1_type_internal_len_106 | ---NA--- | | |
| TRINITY_DN25708_c0_g1_i2_len_1258_194528.p3_type_complete_len_105 | ALL27072.1vascular endothelial growth factor 2 | F:GO:0008083; P:GO:0010469; C:GO:0016020 | |
| TRINITY_DN2893_c0_g1_i1_len_327_173774.p1_type_5prime_partial_len_105 | ---NA--- | | |
| TRINITY_DN3689_c0_g1_i1_len_317_212941.p1_type_internal_len_106 | XP_018018682.1 glutamate--cysteine ligase catalytic subunit-like | | |
| TRINITY_DN39671_c0_g1_i1_len_551_202866.p1_type_complete_len_105 | ---NA--- | | |
| TRINITY_DN15458_c0_g1_i1_len_316_250875.p2_type_internal_len_106 | ---NA--- | | |
| TRINITY_DN21903_c0_g1_i3_len_1999_123130.p4_type_3prime_partial_len_106 | AEU11366.1Broad-complex protein isoform 4 | F:GO:0003676 | |
| TRINITY_DN31820_c0_g1_i1_len_317_282927.p1_type_internal_len_106 | ---NA--- | | |
| TRINITY_DN19548_c0_g1_i1_len_1771_156779.p1_type_5prime_partial_len_105 | ---NA--- | | |
| TRINITY_DN35723_c0_g1_i1_len_317_212940.p3_type_internal_len_106 | APF30034.1ionotropic glutamate receptor NMDA 2A | F:GO:0004970; C:GO:0016021; C:GO:0030054; P:GO:0034220; P:GO:0035235; C:GO:0045211 | |
| TRINITY_DN30777_c0_g1_i1_len_317_282932.p1_type_internal_len_106 | XP_018403002.1 trafficking kinesin-binding protein 2-like | | |
| TRINITY_DN2570_c0_g1_i1_len_646_36347.p1_type_5prime_partial_len_105 | ---NA--- | | |

| | | | |
|---|---|---|---|
| TRINITY_DN13596_c0_g1_i1_len_315_108739.p1_type_internal_len_106 | ---NA--- | | |
| TRINITY_DN121_c0_g1_i1_len_315_108738.p1_type_internal_len_106 | ---NA--- | | |
| TRINITY_DN9518_c0_g1_i1_len_501_204230.p2_type_3prime_partial_len_106 | ---NA--- | | |
| TRINITY_DN31474_c0_g1_i1_len_317_282916.p1_type_internal_len_106 | ---NA--- | | |
| TRINITY_DN34113_c0_g1_i1_len_421_169393.p1_type_5prime_partial_len_105 | ALT10383.1juvenile hormone esterase-like protein 1 | F:GO:0016787 | |
| TRINITY_DN33803_c0_g1_i1_len_316_250885.p1_type_internal_len_106 | ---NA--- | | |
| TRINITY_DN18852_c0_g1_i1_len_341_211282.p1_type_5prime_partial_len_105 | ---NA--- | | |
| TRINITY_DN33960_c0_g1_i1_len_319_250680.p1_type_internal_len_106 | ---NA--- | | |
| TRINITY_DN27290_c0_g1_i1_len_319_250697.p1_type_internal_len_106 | ---NA--- | | |
| TRINITY_DN20723_c0_g1_i1_len_316_45373.p1_type_internal_len_106 | ---NA--- | | |
| TRINITY_DN6969_c0_g1_i1_len_316_250884.p1_type_internal_len_106 | ---NA--- | | |
| TRINITY_DN7452_c0_g1_i1_len_318_212914.p1_type_internal_len_106 | ---NA--- | | |
| TRINITY_DN7630_c0_g1_i1_len_318_250806.p1_type_internal_len_106 | ---NA--- | | |
| TRINITY_DN17924_c0_g1_i2_len_533_38044.p1_type_5prime_partial_len_105 | ---NA--- | | |
| TRINITY_DN30934_c0_g1_i1_len_436_245006.p1_type_3prime_partial_len_106 | XP_021002018.1disks large homolog 5 | F:GO:0005515 | |
| TRINITY_DN10201_c0_g1_i1_len_317_250811.p1_type_internal_len_106 | ---NA--- | | |

| | | | |
|---|---|---|---|
| TRINITY_DN25958_c0_g1_i1_len_2146_230653.p3_type_complete_len_105 | ---NA--- | | |
| TRINITY_DN2817_c0_g1_i1_len_317_139739.p1_type_internal_len_106 | ---NA--- | | |
| TRINITY_DN36501_c0_g1_i1_len_317_250808.p1_type_internal_len_106 | ---NA--- | | |
| TRINITY_DN21757_c0_g1_i1_len_329_173622.p1_type_5prime_partial_len_105 | ---NA--- | | |
| TRINITY_DN35840_c0_g1_i1_len_318_250784.p1_type_internal_len_106 | ---NA--- | | |
| TRINITY_DN25030_c0_g1_i1_len_319_282802.p1_type_internal_len_106 | ---NA--- | | |
| TRINITY_DN9563_c0_g1_i1_len_316_139767.p2_type_internal_len_106 | ---NA--- | | |
| TRINITY_DN23644_c0_g1_i3_len_1817_231682.p2_type_3prime_partial_len_106 | ---NA--- | | |
| TRINITY_DN25812_c0_g1_i1_len_4300_228026.p3_type_complete_len_105 | ---NA--- | | |
| TRINITY_DN39785_c0_g1_i1_len_318_212927.p1_type_3prime_partial_len_106 | XP_018022821.1 ubiquitin carboxyl-terminal hydrolase 25-like | P:GO:0006511; P:GO:0016579; F:GO:0036459 | |
| TRINITY_DN28831_c0_g1_i1_len_3813_187693.p2_type_complete_len_105 | ---NA--- | | |
| TRINITY_DN11848_c0_g1_i1_len_329_212067.p1_type_5prime_partial_len_105 | ---NA--- | | |
| TRINITY_DN32025_c0_g1_i1_len_318_250789.p1_type_internal_len_106 | ---NA--- | | |
| TRINITY_DN11483_c0_g1_i2_len_317_139718.p1_type_internal_len_106 | ---NA--- | | |
| TRINITY_DN24381_c0_g1_i1_len_318_139660.p1_type_internal_len_106 | ---NA--- | | |
| TRINITY_DN29408_c0_g1_i1_len_319_282820.p1_type_internal_len_106 | ---NA--- | | |

<table>
<tr><td>TRINITY_DN24548_c0_g1_i1_len_317_250835.p5_type_internal_len_106</td><td>---NA---</td><td></td><td></td></tr>
<tr><td>TRINITY_DN5432_c0_g1_i1_len_317_139697.p1_type_internal_len_106</td><td>---NA---</td><td></td><td></td></tr>
<tr><td>TRINITY_DN6516_c0_g1_i1_len_429_245290.p1_type_5prime_partial_len_105</td><td>XP_022319564.1RUN and FYVE domain-containing protein 2-like isoform X1</td><td></td><td></td></tr>
<tr><td>TRINITY_DN20607_c0_g1_i1_len_1435_193444.p2_type_complete_len_105</td><td>---NA---</td><td></td><td></td></tr>
<tr><td>TRINITY_DN24545_c0_g1_i1_len_318_139641.p1_type_internal_len_106</td><td>---NA---</td><td></td><td></td></tr>
<tr><td>TRINITY_DN27338_c0_g1_i1_len_3141_188323.p1_type_5prime_partial_len_818</td><td>XP_013777601.2NF-X1-type zinc finger protein NFXL1-like isoform X1</td><td>F:GO:0003700; C:GO:0005634; P:GO:0006355; F:GO:0008270; C:GO:0016020; C:GO:0016021</td><td></td></tr>
<tr><td>TRINITY_DN23340_c0_g1_i1_len_3997_153690.p1_type_complete_len_818</td><td>---NA---</td><td></td><td></td></tr>
<tr><td>TRINITY_DN21916_c0_g1_i1_len_2625_154790.p1_type_5prime_partial_len_818</td><td>XP_015903193.1uncharacterized protein LOC107436127</td><td></td><td></td></tr>
<tr><td>2947|_|c12212_f2p1_2454_isoform_c12212_full_length_coverage_2_non_full_length_coverage_1_isoform_length_2454_17492</td><td>XP_018023071.1PREDICTED: uncharacterized protein LOC108679063 isoform X1</td><td></td><td></td></tr>
<tr><td>TRINITY_DN29084_c0_g1_i1_len_3963_187604.p1_type_5prime_partial_len_818</td><td>XP_012236193.1tudor domain-containing protein 3 isoform X2</td><td>F:GO:0003723; C:GO:0005634; C:GO:0005737; P:GO:0006397</td><td></td></tr>
<tr><td>2949|_|c27042_f2p1_2453_isoform_c27042_full_length_coverage_2_non_full_length_coverage_1_isoform_length_2453_18414</td><td>XP_018023298.1 CSC1-like protein 2</td><td>C:GO:0016020</td><td></td></tr>
<tr><td>2950|_|c10767_f6p0_2453_isoform_c10767_full_length_coverage_6_non_full_length_coverage_0_isoform_length_2453_21994</td><td>XP_018026012.1 E3 ubiquitin-protein ligase CBL-B-like isoform X2</td><td></td><td></td></tr>
<tr><td>2951|_|c1966_f13p6_2453_isoform_c1966_full_length_coverage_13_non_full_length_coverage_6_isoform_length_2453_16088</td><td>XP_017464504.1 zinc finger protein weckle-like</td><td></td><td></td></tr>
</table>

| | | | |
|---|---|---|---|
| 2952\|_\|c5473_f2p2_2453_isoform_c5473_full_length_coverage_2_non_full_length_coverage_2_isoform_length_2453_21528 | AXY87732.1carnitine O-palmitoyltransferase 2 | F:GO:0016746 | |
| 2953\|_\|c2779_f4p2_2453_isoform_c2779_full_length_coverage_4_non_full_length_coverage_2_isoform_length_2453_21228 | XP_022237208.1pescadillo-like isoform X1 | C:GO:0005634; P:GO:0006364 | |
| TRINITY_DN31298_c0_g1_i1_len_317_250820.p1_type_internal_len_106 | ---NA--- | | |
| TRINITY_DN27688_c0_g1_i1_len_317_250836.p1_type_internal_len_106 | PIK40063.1putative exportin-6 | C:GO:0005634; P:GO:0006611; F:GO:0008536; F:GO:0008565 | |
| TRINITY_DN1642_c0_g1_i1_len_317_250821.p1_type_internal_len_106 | XP_018028132.1 protein ECT2-like | | |
| TRINITY_DN9425_c0_g1_i1_len_318_139631.p1_type_internal_len_106 | ---NA--- | | |
| TRINITY_DN37052_c0_g1_i1_len_434_245105.p1_type_5prime_partial_len_105 | XP_018025288.1 DNA damage-regulated autophagy modulator protein 2-like | P:GO:0010506; C:GO:0016020; C:GO:0016021 | |
| TRINITY_DN31061_c0_g2_i1_len_317_212964.p1_type_internal_len_106 | ---NA--- | | |
| TRINITY_DN24910_c0_g1_i1_len_317_139722.p1_type_internal_len_106 | XP_018018682.1 glutamate--cysteine ligase catalytic subunit-like | F:GO:0004357; P:GO:0006750 | EC:6.3.2.2 |
| TRINITY_DN4904_c0_g1_i1_len_317_250828.p1_type_internal_len_106 | ---NA--- | | |
| TRINITY_DN2112_c0_g1_i1_len_889_161590.p2_type_5prime_partial_len_105 | ---NA--- | | |
| TRINITY_DN29593_c0_g1_i1_len_317_250823.p1_type_internal_len_106 | KFM73440.1Diacylglycerol kinase delta | F:GO:0003951; F:GO:0004143; P:GO:0016310; P:GO:0035556 | EC:2.7.1.23; EC:2.7.1.107 |
| TRINITY_DN1101_c0_g1_i1_len_317_212981.p1_type_internal_len_106 | AVI10170.1inhibitor of nuclear factor kappa-B kinase epsilon isoform 2 | F:GO:0004672; F:GO:0005524; P:GO:0006468 | |
| TRINITY_DN26842_c0_g1_i1_len_317_250825.p1_type_internal_len_106 | ---NA--- | | |
| TRINITY_DN28219_c0_g1_i1_len_315_174506.p1_type_internal_len_106 | ---NA--- | | |

| | | | |
|---|---|---|---|
| TRINITY_DN1733_c0_g1_i1_len_334_173333.p1_type_5prime_partial_len_105 | ---NA--- | | |
| TRINITY_DN29090_c0_g1_i3_len_1498_193118.p2_type_complete_len_105 | ---NA--- | | |
| TRINITY_DN24395_c0_g1_i1_len_318_250771.p1_type_5prime_partial_len_105 | ---NA--- | | |
| TRINITY_DN16643_c0_g1_i1_len_689_239381.p1_type_5prime_partial_len_105 | ---NA--- | | |
| TRINITY_DN6977_c0_g1_i1_len_727_35410.p1_type_5prime_partial_len_105 | ---NA--- | | |
| TRINITY_DN28838_c0_g1_i1_len_315_174517.p1_type_internal_len_106 | XP_020779033.1multidrug resistance-associated protein 5 | | |
| TRINITY_DN11273_c0_g1_i1_len_443_244785.p1_type_5prime_partial_len_105 | ---NA--- | | |
| TRINITY_DN12536_c0_g1_i1_len_316_139796.p1_type_internal_len_106 | ---NA--- | | |
| TRINITY_DN13218_c0_g1_i1_len_315_15863.p1_type_internal_len_106 | ---NA--- | | |
| TRINITY_DN3509_c0_g1_i1_len_317_250859.p1_type_internal_len_106 | PNF28630.1Calcium-dependent secretion activator | P:GO:0016079; C:GO:0098793; P:GO:1990504 | |
| TRINITY_DN14034_c0_g1_i1_len_469_205227.p1_type_complete_len_105 | ---NA--- | | |
| TRINITY_DN29552_c0_g1_i1_len_318_250767.p1_type_internal_len_106 | ---NA--- | | |
| TRINITY_DN11352_c0_g1_i1_len_318_250775.p1_type_internal_len_106 | ---NA--- | | |
| TRINITY_DN15084_c0_g1_i2_len_1034_235838.p4_type_5prime_partial_len_105 | ---NA--- | | |
| TRINITY_DN18311_c0_g1_i2_len_543_203066.p1_type_complete_len_105 | ---NA--- | | |
| TRINITY_DN36995_c0_g1_i1_len_317_212952.p1_type_internal_len_106 | XP_018017735.1 blastula protease 10-like | | |

| | | | |
|---|---|---|---|
| TRINITY_DN39409_c0_g1_i1_len_317_212951.p1_type_internal_len_106 | XP_015751981.1 DNA-dependent protein kinase catalytic subunit-like | | |
| TRINITY_DN6836_c0_g1_i1_len_315_139831.p1_type_internal_len_106 | ---NA--- | | |
| TRINITY_DN34280_c0_g1_i1_len_312_140023.p1_type_internal_len_105 | ---NA--- | | |
| TRINITY_DN35236_c0_g1_i1_len_316_250899.p1_type_internal_len_105 | ---NA--- | | |
| TRINITY_DN31493_c0_g1_i1_len_314_15912.p1_type_internal_len_105 | ---NA--- | | |
| TRINITY_DN17977_c0_g1_i2_len_355_248540.p1_type_complete_len_104 | ---NA--- | | |
| TRINITY_DN24439_c0_g1_i1_len_314_139869.p1_type_internal_len_105 | ---NA--- | | |
| TRINITY_DN23840_c0_g1_i5_len_1867_59631.p3_type_complete_len_104 | ---NA--- | | |
| TRINITY_DN32545_c0_g1_i1_len_316_76855.p1_type_internal_len_105 | ODN04273.1Protein c-ets-1-B | F:GO:0003700; C:GO:0005634; P:GO:0006355; F:GO:0043565 | |
| TRINITY_DN15792_c0_g1_i1_len_573_37372.p1_type_complete_len_104 | ---NA--- | | |
| TRINITY_DN33981_c0_g1_i1_len_348_210841.p1_type_5prime_partial_len_104 | ---NA--- | | |
| TRINITY_DN25964_c0_g1_i1_len_365_13180.p1_type_5prime_partial_len_104 | ---NA--- | | |
| TRINITY_DN25090_c0_g1_i1_len_1122_235231.p1_type_complete_len_104 | ---NA--- | | |
| TRINITY_DN22923_c0_g1_i1_len_1524_192970.p3_type_complete_len_104 | ---NA--- | | |
| TRINITY_DN14099_c0_g1_i2_len_703_272774.p1_type_5prime_partial_len_104 | ---NA--- | | |
| TRINITY_DN34264_c0_g1_i1_len_359_171984.p1_type_complete_len_104 | ---NA--- | | |

| | | | |
|---|---|---|---|
| TRINITY_DN29615_c0_g1_i1_len_315_139829.p1_type_internal_len_105 | XP_018016137.1 alpha-(1,3)-fucosyltransferase C-like | | |
| TRINITY_DN33648_c0_g1_i1_len_315_139833.p1_type_internal_len_105 | ---NA--- | | |
| TRINITY_DN10112_c0_g1_i1_len_492_38870.p2_type_complete_len_104 | ---NA--- | | |
| TRINITY_DN40940_c0_g1_i1_len_315_213087.p1_type_internal_len_105 | ---NA--- | | |
| TRINITY_DN22070_c1_g1_i13_len_1243_94293.p3_type_5prime_partial_len_104 | ---NA--- | | |
| TRINITY_DN20136_c0_g1_i1_len_1308_234003.p4_type_complete_len_104 | ---NA--- | | |
| TRINITY_DN19101_c0_g1_i2_len_358_13509.p1_type_3prime_partial_len_105 | RID66984.1hypothetical protein BRARA_D02090 | C:GO:0009522; C:GO:0009523; C:GO:0009535; P:GO:0009765; C:GO:0016021; F:GO:0016168; P:GO:0018298 | |
| TRINITY_DN16728_c0_g1_i1_len_314_15918.p1_type_internal_len_105 | ---NA--- | | |
| TRINITY_DN33359_c0_g1_i1_len_315_213086.p1_type_internal_len_105 | XP_018012859.1 rab GTPase-activating protein 1-like | | |
| TRINITY_DN8280_c0_g1_i1_len_315_213102.p1_type_internal_len_105 | ---NA--- | | |
| TRINITY_DN27843_c0_g1_i1_len_315_139847.p1_type_internal_len_105 | ---NA--- | | |
| TRINITY_DN23647_c0_g1_i1_len_2416_58670.p4_type_complete_len_104 | ---NA--- | | |
| TRINITY_DN4498_c0_g1_i1_len_719_272604.p2_type_complete_len_104 | ---NA--- | | |
| TRINITY_DN11289_c0_g1_i1_len_2926_91194.p3_type_5prime_partial_len_104 | ---NA--- | | |
| TRINITY_DN10199_c0_g1_i1_len_1393_233543.p1_type_complete_len_104 | ---NA--- | | |

| | | | |
|---|---|---|---|
| TRINITY_DN16139_c0_g1_i1_len_397_208157.p2_type_5prime_partial_len_104 | ---NA--- | | |
| TRINITY_DN23766_c0_g1_i1_len_2782_229310.p2_type_complete_len_104 | ---NA--- | | |
| TRINITY_DN13410_c0_g1_i1_len_315_213122.p1_type_internal_len_105 | XP_023301109.1inositol hexakisphosphate and diphosphoinositol-pentakisphosphate kinase isoform X8 | | |
| TRINITY_DN34392_c0_g1_i1_len_324_173948.p1_type_5prime_partial_len_104 | ---NA--- | | |
| TRINITY_DN29430_c0_g1_i1_len_351_248750.p1_type_5prime_partial_len_104 | XP_018021683.1PREDICTED: putative uncharacterized protein DDB_G0277255 | | |
| TRINITY_DN13816_c0_g1_i1_len_1622_192449.p2_type_5prime_partial_len_104 | ---NA--- | | |
| TRINITY_DN4695_c0_g1_i1_len_312_16048.p1_type_internal_len_105 | XP_018013899.1 translocation protein SEC62-like | | |
| TRINITY_DN24808_c0_g1_i2_len_1541_192891.p3_type_complete_len_104 | ---NA--- | | |
| TRINITY_DN29471_c0_g1_i1_len_316_250906.p1_type_internal_len_105 | ---NA--- | | |
| TRINITY_DN22693_c2_g1_i2_len_2771_229319.p4_type_complete_len_104 | ---NA--- | | |
| TRINITY_DN27897_c0_g1_i3_len_948_197017.p1_type_3prime_partial_len_105 | ---NA--- | | |
| TRINITY_DN21574_c0_g1_i1_len_1692_192125.p1_type_complete_len_104 | ---NA--- | | |
| TRINITY_DN3375_c0_g1_i1_len_313_77027.p1_type_internal_len_105 | XP_018026562.1PREDICTED: uncharacterized protein LOC108681986, partial | F:GO:0004857; F:GO:0004867; C:GO:0005576; P:GO:0010466; P:GO:0010951; C:GO:0016020; C:GO:0016021; F:GO:0030414; P:GO:0043086 | |
| TRINITY_DN2699_c0_g1_i1_len_315_213104.p1_type_internal_len_105 | ---NA--- | | |

| | | | |
|---|---|---|---|
| TRINITY_DN14856_c0_g1_i1_len_702_35711.p2_type_3prime_partial_len_105 | ---NA--- | | |
| TRINITY_DN23397_c0_g1_i1_len_376_209222.p1_type_5prime_partial_len_104 | ---NA--- | | |
| TRINITY_DN10840_c0_g1_i1_len_425_103460.p1_type_5prime_partial_len_104 | ---NA--- | | |
| TRINITY_DN7138_c0_g1_i1_len_452_10030.p1_type_complete_len_104 | PNF36066.1hypothetical protein B7P43_G12745 | | |
| TRINITY_DN3837_c0_g1_i1_len_314_139912.p1_type_internal_len_105 | XP_018020685.1 tubulin alpha-3 chain-like | F:GO:0003924; F:GO:0005200; F:GO:0005525; C:GO:0005737; C:GO:0005874; P:GO:0007010; P:GO:0007017 | EC:3.6.1.15 |
| TRINITY_DN35734_c0_g1_i1_len_313_77075.p1_type_internal_len_105 | CRK99649.1CLUMA_CG012960, isoform A | | |
| TRINITY_DN10656_c0_g1_i1_len_312_16046.p1_type_internal_len_105 | ---NA--- | | |
| TRINITY_DN24246_c0_g1_i1_len_441_133903.p1_type_5prime_partial_len_104 | ---NA--- | | |
| TRINITY_DN37492_c0_g1_i1_len_315_213095.p1_type_internal_len_105 | ---NA--- | | |
| TRINITY_DN25342_c0_g1_i1_len_313_77054.p1_type_internal_len_105 | ---NA--- | | |
| TRINITY_DN11022_c0_g1_i1_len_1284_2325.p2_type_complete_len_104 | ---NA--- | | |
| TRINITY_DN31984_c0_g1_i1_len_314_76956.p1_type_3prime_partial_len_105 | ---NA--- | | |
| TRINITY_DN33495_c0_g1_i1_len_314_76963.p1_type_internal_len_105 | ---NA--- | | |
| TRINITY_DN6789_c0_g1_i1_len_314_76964.p1_type_internal_len_105 | XP_022541067.1zinc finger protein OZF-like | F:GO:0003676 | |
| TRINITY_DN8517_c0_g1_i1_len_314_139885.p2_type_internal_len_105 | ---NA--- | | |

| | | | |
|---|---|---|---|
| TRINITY_DN7342_c0_g1_i1_len_313_77010.p1_type_internal_len_105 | XP_018021444.1 pseudouridine-5'-phosphatase-like isoform X1 | F:GO:0000287; F:GO:0016853 | |
| TRINITY_DN29016_c0_g1_i2_len_940_197079.p1_type_5prime_partial_len_104 | ---NA--- | | |
| TRINITY_DN33487_c0_g1_i1_len_314_139897.p1_type_internal_len_105 | KFM79348.1Semaphorin-5A, partial | | |
| TRINITY_DN12074_c0_g1_i1_len_314_76987.p1_type_internal_len_105 | ---NA--- | | |
| TRINITY_DN9627_c0_g1_i1_len_314_76988.p1_type_internal_len_105 | ---NA--- | | |
| TRINITY_DN18728_c0_g1_i1_len_313_139958.p1_type_internal_len_105 | ---NA--- | | |
| TRINITY_DN7613_c0_g1_i1_len_315_76882.p1_type_internal_len_105 | ---NA--- | | |
| TRINITY_DN22163_c0_g1_i10_len_1354_2098.p2_type_5prime_partial_len_104 | ---NA--- | | |
| TRINITY_DN25232_c0_g1_i1_len_326_139137.p1_type_3prime_partial_len_105 | ---NA--- | | |
| TRINITY_DN37721_c0_g1_i1_len_315_213108.p1_type_internal_len_105 | ---NA--- | | |
| TRINITY_DN32118_c0_g1_i1_len_381_208981.p1_type_5prime_partial_len_104 | ---NA--- | | |
| 2954\|_\|c33628_f45p13_2452_isoform_c33628_full_length_coverage_45_non_full_length_coverage_13_isoform_length_2452_23249 | XP_018009730.1 N-acetyltransferase ESCO2-like isoform X2 | | |
| 2955\|_\|c11161_f2p2_2452_isoform_c11161_full_length_coverage_2_non_full_length_coverage_2_isoform_length_2452_22032 | XP_007620765.1 regulator of G-protein signaling 3-like | | |
| 2956\|_\|c7393_f2p0_2452_isoform_c7393_full_length_coverage_2_non_full_length_coverage_0_isoform_length_2452_12622 | ODM95859.1Sterol regulatory element-binding protein cleavage-activating protein | C:GO:0012505; C:GO:0031090; C:GO:0043231; C:GO:0044425; C:GO:0044444; C:GO:0044446 | |

| | | | |
|---|---|---|---|
| 2957\|_\|c1209_f21p9_2451_isoform_c1209_full_length_coverage_21_non_full_length_coverage_9_isoform_length_2451_20396 | XP_018014775.1 DNA replication licensing factor mcm7-B-like | | |
| 2958\|_\|c4020_f2p0_2451_isoform_c4020_full_length_coverage_2_non_full_length_coverage_0_isoform_length_2451_12342 | XP_018023323.1 PAX3- and PAX7-binding protein 1-like | P:GO:0000122; F:GO:0000978; F:GO:0003677; F:GO:0003700; C:GO:0005634; P:GO:0006355 | |
| TRINITY_DN29284_c0_g1_i4_len_3070_188423.p1_type_complete_len_817 | XP_018023122.1 longitudinals lacking protein, isoforms H/M/V-like isoform X3 | | |
| 2960\|_\|c2011_f2p2_2451_isoform_c2011_full_length_coverage_2_non_full_length_coverage_2_isoform_length_2451_28148 | ---NA--- | | |
| TRINITY_DN24923_c0_g1_i1_len_2564_58499.p1_type_5prime_partial_len_817 | XP_013096036.1 vacuolar protein sorting-associated protein 51 homolog | | |
| TRINITY_DN21896_c0_g1_i1_len_3173_228801.p1_type_5prime_partial_len_817 | XP_021940303.1nipped-B-like protein | F:GO:0003682; C:GO:0005634; P:GO:0007049; P:GO:0010468 | |
| 2964\|_\|c1349_f13p8_2450_isoform_c1349_full_length_coverage_13_non_full_length_coverage_8_isoform_length_2450_15836 | XP_019626034.1 mini-chromosome maintenance complex-binding protein-like | | |
| TRINITY_DN17717_c0_g1_i1_len_1231_194743.p1_type_complete_len_104 | ---NA--- | | |
| TRINITY_DN12073_c0_g1_i1_len_1012_270128.p2_type_complete_len_104 | ---NA--- | | |
| TRINITY_DN24093_c0_g1_i4_len_313_139947.p1_type_internal_len_105 | ---NA--- | | |
| TRINITY_DN32809_c0_g1_i1_len_313_139953.p1_type_internal_len_105 | XP_018022393.1 myosin heavy chain, muscle-like isoform X5 | | |
| TRINITY_DN9116_c0_g1_i1_len_423_103513.p1_type_5prime_partial_len_104 | ---NA--- | | |
| TRINITY_DN11666_c0_g1_i1_len_370_73703.p1_type_5prime_partial_len_104 | ---NA--- | | |
| TRINITY_DN36541_c0_g1_i1_len_315_213098.p1_type_internal_len_105 | ---NA--- | | |

| | | | |
|---|---|---|---|
| TRINITY_DN31991_c0_g1_i1_len_313_139979.p1_type_5prime_partial_len_104 | ---NA--- | | |
| TRINITY_DN38030_c0_g1_i1_len_316_250892.p1_type_internal_len_105 | ---NA--- | | |
| TRINITY_DN463_c0_g1_i1_len_313_139969.p1_type_internal_len_105 | XP_017104117.1 mitochondrial intermediate peptidase isoform X2 | F:GO:0004222; C:GO:0005759; P:GO:0006627; F:GO:0046872 | EC:3.4.24 |
| TRINITY_DN30651_c0_g1_i1_len_313_139972.p1_type_internal_len_105 | KFM70839.1Retrovirus-related Pol polyprotein from transposon 17.6 | F:GO:0003676; P:GO:0015074 | |
| TRINITY_DN23193_c0_g1_i2_len_1381_193766.p2_type_5prime_partial_len_104 | ---NA--- | | |
| TRINITY_DN26929_c0_g1_i1_len_314_139921.p1_type_internal_len_105 | AOR07228.1potassium voltage-gated channel subfamily H member 2 | F:GO:0005249; C:GO:0016021; P:GO:0071805 | |
| TRINITY_DN8806_c0_g1_i1_len_315_213106.p1_type_internal_len_105 | ---NA--- | | |
| TRINITY_DN12718_c0_g1_i1_len_347_248923.p1_type_5prime_partial_len_104 | ---NA--- | | |
| TRINITY_DN29499_c0_g1_i1_len_314_139878.p2_type_internal_len_105 | ---NA--- | | |
| TRINITY_DN3118_c0_g1_i2_len_312_77079.p1_type_internal_len_105 | PSN50942.1Host cell factor 1 | C:GO:0048188; C:GO:0071339 | |
| TRINITY_DN23613_c0_g1_i1_len_3854_153738.p5_type_complete_len_104 | ---NA--- | | |
| TRINITY_DN28203_c0_g1_i1_len_312_77139.p1_type_internal_len_105 | ---NA--- | | |
| TRINITY_DN28239_c0_g1_i1_len_315_76888.p1_type_internal_len_105 | XP_018015557.1 palmitoyltransferase Hip14-like | F:GO:0015095; C:GO:0016021; F:GO:0019706; P:GO:1903830 | EC:2.3.1.225 |
| TRINITY_DN32549_c0_g1_i1_len_314_139924.p1_type_internal_len_105 | ---NA--- | | |
| TRINITY_DN17530_c0_g1_i1_len_830_237654.p1_type_5prime_partial_len_104 | ---NA--- | | |
| TRINITY_DN18204_c0_g1_i1_len_4850_153470.p4_type_complete_len_104 | ---NA--- | | |

| | | | |
|---|---|---|---|
| TRINITY_DN25410_c0_g1_i1_len_315_174508.p1_type_internal_len_105 | ---NA--- | | |
| TRINITY_DN24189_c0_g1_i1_len_1244_194642.p2_type_complete_len_104 | ---NA--- | | |
| TRINITY_DN26296_c0_g1_i1_len_347_14052.p1_type_5prime_partial_len_104 | ---NA--- | | |
| TRINITY_DN19312_c0_g1_i1_len_752_199247.p1_type_5prime_partial_len_104 | PIK58476.1hypothetical protein BSL78_04609 | | |
| TRINITY_DN19347_c0_g2_i1_len_450_305401.p1_type_5prime_partial_len_104 | KNC33235.1putative UDP-glucose 4-epimerase | F:GO:0016853 | |
| TRINITY_DN26119_c0_g1_i3_len_321_212700.p1_type_5prime_partial_len_104 | AUB30162.1fast-type skeletal muscle actin 17 | F:GO:0005524 | |
| TRINITY_DN11707_c0_g1_i1_len_315_174507.p1_type_internal_len_105 | ---NA--- | | |
| TRINITY_DN29391_c0_g1_i1_len_313_15986.p1_type_internal_len_105 | ---NA--- | | |
| TRINITY_DN2610_c0_g1_i1_len_331_249908.p1_type_5prime_partial_len_104 | ---NA--- | | |
| TRINITY_DN10524_c0_g1_i1_len_315_174501.p2_type_internal_len_105 | ---NA--- | | |
| TRINITY_DN14002_c0_g1_i1_len_406_169983.p1_type_5prime_partial_len_104 | ---NA--- | | |
| TRINITY_DN32002_c0_g1_i1_len_314_174567.p1_type_internal_len_105 | ---NA--- | | |
| TRINITY_DN8823_c0_g1_i1_len_330_249994.p1_type_5prime_partial_len_104 | ---NA--- | | |
| TRINITY_DN34328_c0_g1_i1_len_314_174566.p1_type_internal_len_105 | ---NA--- | | |
| TRINITY_DN28065_c0_g1_i1_len_315_283058.p1_type_internal_len_105 | ---NA--- | | |
| TRINITY_DN26069_c0_g1_i1_len_4305_228023.p5_type_3prime_partial_len_105 | ---NA--- | | |

| | | | |
|---|---|---|---|
| TRINITY_DN17256_c0_g1_i1_len_970_270400.p1_type_complete_len_104 | ---NA--- | | |
| TRINITY_DN4314_c0_g1_i2_len_994_196603.p1_type_5prime_partial_len_104 | ---NA--- | | |
| TRINITY_DN26428_c0_g1_i1_len_345_75035.p1_type_5prime_partial_len_104 | ---NA--- | | |
| TRINITY_DN21900_c0_g1_i1_len_1244_194638.p2_type_5prime_partial_len_104 | ---NA--- | | |
| TRINITY_DN32179_c0_g1_i1_len_314_174546.p1_type_5prime_partial_len_104 | ---NA--- | | |
| TRINITY_DN11099_c0_g1_i1_len_316_213016.p1_type_internal_len_105 | XP_018568580.1multidrug resistance-associated protein 1-like | F:GO:0005524; C:GO:0016021; F:GO:0042626; P:GO:0055085 | EC:3.6.1.3; EC:3.6.1.15 |
| TRINITY_DN9237_c0_g1_i1_len_313_15980.p1_type_internal_len_105 | ---NA--- | | |
| TRINITY_DN42063_c0_g1_i1_len_316_213030.p1_type_internal_len_105 | ---NA--- | | |
| TRINITY_DN5478_c0_g1_i1_len_346_211023.p1_type_5prime_partial_len_104 | ---NA--- | | |
| TRINITY_DN13330_c0_g1_i1_len_344_138059.p1_type_5prime_partial_len_104 | ---NA--- | | |
| TRINITY_DN18411_c0_g1_i1_len_4691_264413.p5_type_5prime_partial_len_104 | ASW35102.1eukaryotic translation initiation factor 3 subunit A-like protein | F:GO:0003743; C:GO:0005852; P:GO:0006413 | |
| TRINITY_DN3192_c0_g1_i1_len_315_283002.p1_type_internal_len_105 | XP_018014863.1 tyrosine-protein kinase Src64B-like isoform X5 | | |
| TRINITY_DN7542_c0_g1_i1_len_785_198800.p2_type_complete_len_104 | ---NA--- | | |
| TRINITY_DN17761_c0_g1_i2_len_1030_95410.p2_type_3prime_partial_len_105 | ---NA--- | | |
| TRINITY_DN27349_c0_g1_i1_len_315_283027.p3_type_internal_len_105 | BAW35379.1lipoxygenase | F:GO:0004096; F:GO:0016702; F:GO:0020037; F:GO:0046872; P:GO:0055114; P:GO:0098869 | EC:1.13.11; EC:1.11.1.7; EC:1.11.1.6 |
| TRINITY_DN1784_c0_g1_i1_len_333_14780.p1_type_5prime_partial_len_104 | ---NA--- | | |

| | | | |
|---|---|---|---|
| TRINITY_DN2550_c0_g1_i1_len_553_202813.p1_type_5prime_partial_len_104 | ---NA--- | | |
| TRINITY_DN11937_c0_g1_i1_len_384_136052.p1_type_complete_len_104 | AEB54795.1single VWC domain protein 5 | | |
| TRINITY_DN26069_c0_g1_i1_len_348_13980.p1_type_5prime_partial_len_104 | ---NA--- | | |
| TRINITY_DN29758_c0_g1_i1_len_522_166594.p1_type_complete_len_104 | ---NA--- | | |
| TRINITY_DN22211_c0_g1_i2_len_1907_191232.p4_type_5prime_partial_len_104 | ---NA--- | | |
| TRINITY_DN6100_c0_g1_i1_len_549_37752.p1_type_5prime_partial_len_104 | ---NA--- | | |
| TRINITY_DN20492_c0_g1_i1_len_1430_1876.p3_type_5prime_partial_len_104 | ---NA--- | | |
| TRINITY_DN27182_c0_g1_i1_len_312_174695.p1_type_internal_len_105 | ---NA--- | | |
| TRINITY_DN4471_c0_g1_i1_len_551_100279.p2_type_5prime_partial_len_104 | ---NA--- | | |
| TRINITY_DN22602_c0_g1_i1_len_314_283128.p1_type_internal_len_105 | ---NA--- | | |
| TRINITY_DN30048_c0_g1_i2_len_2262_190064.p2_type_complete_len_104 | PIK57841.1putative mucin-19-like | F:GO:0003684; P:GO:0006289; F:GO:0008534 | EC:3.2.2.23 |
| TRINITY_DN22452_c0_g1_i1_len_332_282039.p1_type_5prime_partial_len_104 | ---NA--- | | |
| TRINITY_DN19987_c0_g1_i1_len_960_236448.p1_type_5prime_partial_len_104 | ---NA--- | | |
| TRINITY_DN2010_c0_g1_i1_len_313_283161.p1_type_internal_len_105 | ---NA--- | | |
| TRINITY_DN13780_c0_g1_i1_len_313_283153.p1_type_internal_len_105 | ---NA--- | | |
| TRINITY_DN27573_c0_g1_i1_len_396_170374.p1_type_5prime_partial_len_104 | ---NA--- | | |

| | | | |
|---|---|---|---|
| TRINITY_DN8801_c0_g1_i1_len_312_283238.p1_type_internal_len_105 | ---NA--- | | |
| TRINITY_DN21580_c0_g1_i1_len_315_283070.p1_type_internal_len_105 | ACR24223.1relish small isoform | F:GO:0003677; F:GO:0003700; C:GO:0005634; C:GO:0005737; P:GO:0006355 | |
| TRINITY_DN27094_c0_g1_i1_len_2359_189800.p2_type_5prime_partial_len_104 | ---NA--- | | |
| TRINITY_DN5296_c0_g1_i1_len_314_174570.p1_type_internal_len_105 | XP_012258404.1endoplasmic reticulum-Golgi intermediate compartment protein 2 | C:GO:0016020 | |
| TRINITY_DN6117_c0_g1_i1_len_314_174569.p3_type_internal_len_105 | AJG06864.1laccase-1-like protein | F:GO:0005507; F:GO:0016491; P:GO:0055114 | |
| TRINITY_DN8295_c0_g1_i1_len_314_174582.p1_type_internal_len_105 | ---NA--- | | |
| TRINITY_DN27960_c0_g1_i1_len_314_174578.p1_type_internal_len_105 | XP_018021514.1 heparan sulfate 2-O-sulfotransferase 1-like | F:GO:0008146; C:GO:0016021 | |
| TRINITY_DN2726_c0_g1_i1_len_355_210420.p1_type_5prime_partial_len_104 | ---NA--- | | |
| TRINITY_DN20984_c0_g1_i1_len_1026_196345.p2_type_5prime_partial_len_104 | ---NA--- | | |
| TRINITY_DN10290_c0_g1_i1_len_1107_269592.p2_type_complete_len_104 | ---NA--- | | |
| TRINITY_DN33496_c0_g1_i1_len_313_174652.p1_type_internal_len_105 | XP_018011710.1 venom phosphodiesterase 2-like | F:GO:0003676; F:GO:0003824; C:GO:0016020; C:GO:0016021; F:GO:0016787; F:GO:0046872 | |
| TRINITY_DN20134_c0_g1_i3_len_1649_192319.p2_type_complete_len_104 | ---NA--- | | |
| TRINITY_DN32234_c0_g1_i1_len_340_14394.p1_type_5prime_partial_len_104 | ---NA--- | | |
| TRINITY_DN8568_c0_g1_i1_len_403_306919.p1_type_5prime_partial_len_104 | XP_022192053.1E3 ubiquitin-protein ligase SH3RF1-like | | |
| TRINITY_DN2099_c0_g1_i1_len_317_212962.p1_type_5prime_partial_len_104 | ---NA--- | | |

| | | | |
|---|---|---|---|
| TRINITY_DN17257_c0_g1_i1_len_1283_194377.p2_type_5prime_partial_len_104 | ---NA--- | | |
| TRINITY_DN17493_c0_g1_i2_len_1110_269570.p4_type_3prime_partial_len_105 | ---NA--- | | |
| TRINITY_DN8310_c0_g1_i1_len_313_174632.p2_type_internal_len_105 | ---NA--- | | |
| TRINITY_DN3510_c0_g1_i1_len_314_251055.p1_type_internal_len_105 | ---NA--- | | |
| TRINITY_DN1724_c0_g1_i1_len_314_251049.p1_type_3prime_partial_len_105 | KZS04237.1Collagen type IV alpha-3-binding protein | C:GO:0005581; C:GO:0005794; C:GO:0005829; C:GO:0016020; P:GO:0035621; F:GO:0070273; P:GO:0120009; F:GO:1902387; F:GO:1902388; P:GO:1902389 | |
| TRINITY_DN27022_c0_g1_i1_len_314_251038.p2_type_internal_len_105 | ---NA--- | | |
| TRINITY_DN23753_c0_g1_i1_len_314_15973.p1_type_internal_len_105 | XP_019869459.1 galactosylgalactosylxylosylprotein 3-beta-glucuronosyltransferase P | | |
| TRINITY_DN29030_c0_g1_i1_len_314_251061.p1_type_internal_len_105 | AFQ62792.1inositol-requiring enzyme-1 | F:GO:0004540; F:GO:0004672; F:GO:0005524; P:GO:0006397; P:GO:0006468; C:GO:0016021; P:GO:0090501 | |
| TRINITY_DN6193_c0_g1_i1_len_387_208634.p1_type_5prime_partial_len_104 | ---NA--- | | |
| TRINITY_DN33576_c0_g1_i1_len_314_251060.p1_type_internal_len_105 | ---NA--- | | |
| TRINITY_DN9487_c0_g1_i1_len_314_251036.p2_type_internal_len_105 | ---NA--- | | |
| TRINITY_DN20066_c0_g1_i1_len_624_6641.p2_type_5prime_partial_len_104 | ---NA--- | | |
| TRINITY_DN36294_c0_g1_i1_len_314_251034.p1_type_internal_len_105 | ---NA--- | | |

| | | | |
|---|---|---|---|
| TRINITY_DN1502_c0_g1_i1_len_314_251029.p1_type_internal_len_105 | ---NA--- | | |
| 2966\|_\|c46929_f2p1_2450_isoform_c46929_full_length_coverage_2_non_full_length_coverage_1_isoform_length_2450_19332 | AOD27423.1presenilin 1 | C:GO:0000139; F:GO:0004190; C:GO:0005789; P:GO:0007219; C:GO:0016021; P:GO:0016485 | EC:3.4.23 |
| 2967\|_\|c2123_f7p5_2450_isoform_c2123_full_length_coverage_7_non_full_length_coverage_5_isoform_length_2450_20723 | XP_019881193.1 tether containing UBX domain for GLUT4 | | |
| 2970\|_\|c33233_f2p2_2449_isoform_c33233_full_length_coverage_2_non_full_length_coverage_2_isoform_length_2449_14215 | ---NA--- | | |
| 2971\|_\|c15361_f4p0_2449_isoform_c15361_full_length_coverage_4_non_full_length_coverage_0_isoform_length_2449_22333 | ---NA--- | | |
| TRINITY_DN24645_c0_g1_i2_len_2972_229028.p1_type_complete_len_816 | XP_026286400.1post-GPI attachment to proteins factor 6 | C:GO:0016020; C:GO:0016021 | |
| TRINITY_DN28507_c0_g1_i2_len_2451_189573.p1_type_internal_len_817 | ---NA--- | | |
| TRINITY_DN18791_c0_g1_i3_len_2687_229453.p1_type_complete_len_816 | ---NA--- | | |
| 2975\|_\|c18222_f2p2_2448_isoform_c18222_full_length_coverage_2_non_full_length_coverage_2_isoform_length_2448_13658 | PNF35312.1Ragulator complex protein LAMTOR1 | P:GO:0031323; C:GO:0031902; C:GO:0045121; P:GO:0060255; P:GO:1902533 | |
| 2976\|_\|c5076_f4p1_2448_isoform_c5076_full_length_coverage_4_non_full_length_coverage_1_isoform_length_2448_16921 | ---NA--- | | |
| TRINITY_DN30448_c0_g1_i1_len_2628_189175.p1_type_5prime_partial_len_816 | XP_021913475.1spatacsin isoform X1 | | |
| TRINITY_DN33476_c0_g1_i1_len_313_251102.p1_type_internal_len_105 | ---NA--- | | |
| TRINITY_DN3378_c0_g1_i1_len_313_251099.p1_type_internal_len_105 | ---NA--- | | |

| | | | |
|---|---|---|---|
| TRINITY_DN1660_c0_g1_i1_len_313_251094.p1_type_internal_len_105 | ---NA--- | | |
| TRINITY_DN6186_c0_g1_i1_len_313_251089.p1_type_internal_len_105 | XP_023932336.1rho GTPase-activating protein 7 | P:GO:0007165; F:GO:0008289 | |
| TRINITY_DN19005_c0_g1_i2_len_431_206682.p1_type_5prime_partial_len_104 | ---NA--- | | |
| TRINITY_DN7104_c0_g1_i1_len_314_251072.p2_type_internal_len_105 | EEB16015.1rabconnectin, putative | | |
| TRINITY_DN18748_c0_g1_i1_len_2060_190689.p2_type_complete_len_104 | ---NA--- | | |
| TRINITY_DN28470_c0_g1_i1_len_314_251071.p2_type_internal_len_105 | ---NA--- | | |
| TRINITY_DN11316_c0_g1_i2_len_314_251069.p3_type_internal_len_105 | ---NA--- | | |
| TRINITY_DN6519_c0_g1_i2_len_341_249328.p1_type_5prime_partial_len_104 | ---NA--- | | |
| TRINITY_DN2811_c0_g1_i1_len_314_251084.p1_type_internal_len_105 | XP_018011606.1 UDP-N-acetylglucosamine transporter-like isoform X2 | C:GO:0000139; F:GO:0015165; C:GO:0016021; P:GO:0090481 | |
| TRINITY_DN30710_c0_g1_i1_len_373_171378.p1_type_5prime_partial_len_104 | ---NA--- | | |
| TRINITY_DN1946_c0_g1_i1_len_407_135107.p1_type_5prime_partial_len_104 | ---NA--- | | |
| TRINITY_DN33124_c0_g1_i1_len_314_251076.p1_type_internal_len_105 | XP_018008738.1 guanine nucleotide exchange factor subunit Rich-like | | |
| TRINITY_DN28281_c0_g1_i1_len_314_15966.p1_type_internal_len_105 | ---NA--- | | |
| TRINITY_DN24031_c0_g1_i1_len_675_163876.p1_type_5prime_partial_len_104 | ---NA--- | | |
| TRINITY_DN15010_c0_g1_i1_len_683_163765.p1_type_complete_len_104 | ---NA--- | | |
| TRINITY_DN8307_c0_g1_i1_len_1036_235815.p1_type_complete_len_104 | ---NA--- | | |

| | | | |
|---|---|---|---|
| TRINITY_DN35742_c0_g1_i1_len_618_201415.p1_type_5prime_partial_len_104 | ---NA--- | | |
| TRINITY_DN25186_c0_g1_i1_len_414_134882.p1_type_5prime_partial_len_104 | ---NA--- | | |
| TRINITY_DN23894_c1_g1_i1_len_315_250994.p1_type_internal_len_105 | ---NA--- | | |
| TRINITY_DN32963_c0_g1_i1_len_315_250989.p1_type_internal_len_105 | XP_026807908.1protein crumbs isoform X1 | | |
| TRINITY_DN26647_c0_g1_i1_len_315_251003.p2_type_internal_len_105 | ---NA--- | | |
| TRINITY_DN16335_c1_g1_i1_len_347_172661.p1_type_5prime_partial_len_104 | ---NA--- | | |
| TRINITY_DN24987_c0_g1_i1_len_1819_191602.p2_type_complete_len_104 | ---NA--- | | |
| TRINITY_DN20502_c0_g1_i1_len_682_35932.p1_type_5prime_partial_len_104 | ---NA--- | | |
| TRINITY_DN17428_c0_g1_i1_len_424_169300.p1_type_5prime_partial_len_104 | EFA00831.1Muscle LIM protein Mlp84B-like Protein | F:GO:0046872 | |
| TRINITY_DN25446_c0_g1_i1_len_1813_191616.p5_type_5prime_partial_len_104 | ---NA--- | | |
| TRINITY_DN35670_c0_g1_i1_len_315_250974.p1_type_internal_len_105 | ---NA--- | | |
| TRINITY_DN30157_c0_g1_i5_len_2770_188914.p2_type_complete_len_104 | ---NA--- | | |
| TRINITY_DN34166_c0_g1_i1_len_333_173420.p1_type_3prime_partial_len_105 | ---NA--- | | |
| TRINITY_DN39310_c0_g1_i1_len_316_213041.p1_type_internal_len_105 | ---NA--- | | |
| TRINITY_DN19002_c0_g1_i2_len_396_135566.p1_type_5prime_partial_len_104 | ---NA--- | | |
| TRINITY_DN21244_c0_g1_i2_len_1966_191020.p1_type_5prime_partial_len_104 | ---NA--- | | |

| | | | |
|---|---|---|---|
| TRINITY_DN9665_c0_g1_i1_len_503_38625.p1_type_5prime_partial_len_104 | ---NA--- | | |
| TRINITY_DN10932_c0_g1_i2_len_316_213047.p1_type_internal_len_105 | XP_015905073.1uncharacterized protein LOC107437515 | C:GO:0005576; P:GO:0006030; F:GO:0008061 | |
| TRINITY_DN9186_c0_g1_i1_len_478_204964.p2_type_3prime_partial_len_105 | ---NA--- | | |
| TRINITY_DN31814_c0_g1_i1_len_475_205036.p1_type_complete_len_104 | ---NA--- | | |
| TRINITY_DN21963_c0_g1_i1_len_316_282983.p1_type_internal_len_105 | ---NA--- | | |
| TRINITY_DN36477_c0_g1_i1_len_392_208432.p1_type_3prime_partial_len_105 | ---NA--- | | |
| TRINITY_DN17678_c0_g1_i1_len_944_270600.p1_type_complete_len_104 | ---NA--- | | |
| TRINITY_DN19653_c0_g1_i1_len_1216_234602.p2_type_complete_len_104 | ---NA--- | | |
| TRINITY_DN19653_c0_g1_i1_len_1216_234602.p1_type_complete_len_104 | ---NA--- | | |
| TRINITY_DN11621_c0_g1_i1_len_380_171037.p1_type_5prime_partial_len_104 | ---NA--- | | |
| TRINITY_DN19128_c0_g1_i1_len_1592_31227.p2_type_complete_len_104 | ---NA--- | | |
| TRINITY_DN10997_c0_g1_i1_len_312_251187.p1_type_internal_len_105 | ---NA--- | | |
| TRINITY_DN15220_c0_g1_i1_len_312_251184.p1_type_internal_len_105 | ---NA--- | | |
| TRINITY_DN24028_c0_g1_i2_len_884_237095.p2_type_5prime_partial_len_104 | ---NA--- | | |
| TRINITY_DN10423_c0_g1_i1_len_331_75867.p1_type_5prime_partial_len_104 | ---NA--- | | |
| TRINITY_DN20267_c0_g1_i2_len_740_199428.p1_type_complete_len_104 | ---NA--- | | |

| | | | |
|---|---|---|---|
| TRINITY_DN37384_c0_g1_i1_len_312_251194.p1_type_internal_len_105 | ---NA--- | | |
| TRINITY_DN3444_c0_g1_i1_len_322_282620.p1_type_5prime_partial_len_104 | ---NA--- | | |
| TRINITY_DN35419_c0_g1_i1_len_360_74209.p1_type_5prime_partial_len_104 | ---NA--- | | |
| TRINITY_DN31002_c0_g1_i1_len_313_251113.p1_type_internal_len_105 | ---NA--- | | |
| TRINITY_DN28636_c0_g1_i1_len_313_251110.p1_type_internal_len_105 | ---NA--- | | |
| TRINITY_DN30674_c0_g1_i1_len_314_15919.p1_type_internal_len_105 | ---NA--- | | |
| TRINITY_DN28980_c0_g1_i1_len_313_251145.p1_type_internal_len_105 | XP_018328204.1nuclear hormone receptor FTZ-F1 beta | F:GO:0003700; F:GO:0003707; C:GO:0005634; P:GO:0006355; F:GO:0008270; P:GO:0043401; F:GO:0043565 | |
| TRINITY_DN29726_c0_g1_i1_len_313_251142.p1_type_internal_len_105 | ---NA--- | | |
| TRINITY_DN7372_c0_g1_i1_len_368_209643.p1_type_5prime_partial_len_104 | ---NA--- | | |
| TRINITY_DN1380_c0_g1_i1_len_313_251127.p1_type_internal_len_105 | ---NA--- | | |
| TRINITY_DN23337_c0_g1_i4_len_3253_228710.p6_type_5prime_partial_len_104 | ---NA--- | | |
| TRINITY_DN20879_c0_g1_i1_len_316_213054.p1_type_internal_len_105 | ---NA--- | | |
| TRINITY_DN17662_c0_g1_i1_len_900_236951.p2_type_complete_len_104 | ---NA--- | | |
| TRINITY_DN15255_c0_g1_i1_len_480_167582.p1_type_5prime_partial_len_104 | ---NA--- | | |
| TRINITY_DN27710_c0_g1_i1_len_312_251231.p1_type_internal_len_105 | ---NA--- | | |

| | | | |
|---|---|---|---|
| TRINITY_DN32431_c0_g1_i1_len_312_251208.p1_type_internal_len_105 | ---NA--- | | |
| TRINITY_DN29918_c0_g1_i1_len_626_164581.p1_type_5prime_partial_len_104 | ---NA--- | | |
| TRINITY_DN12184_c1_g1_i1_len_312_251205.p1_type_internal_len_105 | ---NA--- | | |
| TRINITY_DN28080_c0_g1_i1_len_314_45517.p1_type_internal_len_105 | XP_010565822.1 cystatin-B isoform X1 | F:GO:0004866 | |
| TRINITY_DN7575_c0_g1_i1_len_312_311321.p1_type_internal_len_105 | ---NA--- | | |
| TRINITY_DN20655_c0_g2_i1_len_463_205443.p1_type_5prime_partial_len_104 | ---NA--- | | |
| TRINITY_DN41493_c0_g1_i1_len_314_213167.p3_type_internal_len_105 | ---NA--- | | |
| TRINITY_DN22116_c0_g1_i4_len_437_244982.p3_type_5prime_partial_len_104 | ---NA--- | | |
| TRINITY_DN28266_c0_g1_i1_len_313_311311.p1_type_internal_len_105 | ---NA--- | | |
| TRINITY_DN28795_c0_g1_i1_len_314_45496.p3_type_internal_len_105 | ---NA--- | | |
| TRINITY_DN15416_c0_g1_i1_len_903_161469.p1_type_5prime_partial_len_104 | ---NA--- | | |
| TRINITY_DN8704_c0_g1_i1_len_312_311354.p2_type_internal_len_105 | ---NA--- | | |
| TRINITY_DN25012_c0_g1_i1_len_312_311352.p1_type_internal_len_105 | ---NA--- | | |
| TRINITY_DN41094_c0_g1_i1_len_314_213177.p1_type_internal_len_105 | ---NA--- | | |
| TRINITY_DN15324_c0_g1_i1_len_1433_193463.p5_type_complete_len_104 | ---NA--- | | |
| TRINITY_DN37680_c0_g1_i1_len_314_213179.p1_type_internal_len_105 | ---NA--- | | |

| | | | |
|---|---|---|---|
| TRINITY_DN29378_c0_g1_i1_len_313_311289.p1_type_internal_len_105 | ---NA--- | | |
| TRINITY_DN40115_c0_g1_i1_len_314_213174.p1_type_internal_len_105 | ---NA--- | | |
| TRINITY_DN26953_c0_g1_i1_len_313_45534.p1_type_internal_len_105 | ---NA--- | | |
| TRINITY_DN37296_c0_g1_i1_len_312_213348.p1_type_internal_len_105 | ---NA--- | | |
| TRINITY_DN11897_c0_g1_i1_len_575_302754.p1_type_5prime_partial_len_104 | ---NA--- | | |
| TRINITY_DN18843_c0_g1_i2_len_2058_230937.p4_type_5prime_partial_len_104 | ---NA--- | | |
| TRINITY_DN19453_c0_g1_i1_len_828_4644.p3_type_complete_len_104 | ---NA--- | | |
| TRINITY_DN32571_c0_g1_i1_len_370_247758.p1_type_3prime_partial_len_105 | ---NA--- | | |
| TRINITY_DN19735_c0_g1_i1_len_725_65384.p1_type_3prime_partial_len_105 | ---NA--- | | |
| TRINITY_DN11876_c0_g1_i2_len_513_68793.p2_type_5prime_partial_len_104 | ---NA--- | | |
| TRINITY_DN33183_c0_g1_i1_len_315_213156.p1_type_internal_len_105 | XP_018027377.1 ubiquitin carboxyl-terminal hydrolase 16-like | | |
| TRINITY_DN3202_c0_g1_i1_len_315_213150.p1_type_internal_len_105 | AGK34834.1Sushi/von Willebrand factor type A/EGF/pentraxin domain-containing 1 | | |
| TRINITY_DN31226_c0_g1_i1_len_315_108743.p1_type_internal_len_105 | ---NA--- | | |
| TRINITY_DN28737_c0_g1_i1_len_315_108741.p1_type_internal_len_105 | ---NA--- | | |
| TRINITY_DN23320_c0_g1_i1_len_315_108737.p2_type_internal_len_105 | ABP97431.1alpha 2 macroglobulin | F:GO:0004866; C:GO:0005615; P:GO:0010951 | |
| TRINITY_DN8622_c0_g1_i2_len_420_11024.p2_type_5prime_partial_len_104 | ---NA--- | | |

<table>
<tr><td></td><td></td><td></td><td></td></tr>
<tr><td>TRINITY_DN27189_c0_g1_i1_len_316_108730.p1_type_internal_len_105</td><td>---NA---</td><td></td><td></td></tr>
<tr><td>TRINITY_DN9033_c0_g1_i1_len_369_247804.p1_type_5prime_partial_len_104</td><td>---NA---</td><td></td><td></td></tr>
<tr><td>TRINITY_DN3946_c0_g1_i1_len_316_108722.p1_type_internal_len_105</td><td>---NA---</td><td></td><td></td></tr>
<tr><td>2978|_|c8887_f5p4_2448_isoform_c8887_full_length_coverage_5_non_full_length_coverage_4_isoform_length_2448_21814</td><td>XP_023194945.1zinc finger protein OZF-like</td><td>F:GO:0003676</td><td></td></tr>
<tr><td>2979|_|c17899_f2p0_2447_isoform_c17899_full_length_coverage_2_non_full_length_coverage_0_isoform_length_2447_22552</td><td>---NA---</td><td></td><td></td></tr>
<tr><td>2980|_|c7305_f2p20_2446_isoform_c7305_full_length_coverage_2_non_full_length_coverage_20_isoform_length_2446_12616</td><td>XP_018026409.1 coatomer subunit delta-like isoform X2</td><td>P:GO:0016192; C:GO:0030663; C:GO:0044431</td><td></td></tr>
<tr><td>2982|_|c5333_f3p17_2445_isoform_c5333_full_length_coverage_3_non_full_length_coverage_17_isoform_length_2445_25759</td><td>---NA---</td><td></td><td></td></tr>
<tr><td>TRINITY_DN25671_c0_g1_i1_len_3063_228925.p1_type_5prime_partial_len_815</td><td>XP_016919963.1 protein kintoun</td><td></td><td></td></tr>
<tr><td>2985|_|c2003_f5p0_2445_isoform_c2003_full_length_coverage_5_non_full_length_coverage_0_isoform_length_2445_16118</td><td>---NA---</td><td></td><td></td></tr>
<tr><td>2986|_|c35001_f2p9_2445_isoform_c35001_full_length_coverage_2_non_full_length_coverage_9_isoform_length_2445_14420</td><td>XP_018015699.1 signal recognition particle subunit SRP68-like</td><td>F:GO:0005488</td><td></td></tr>
<tr><td>2987|_|c1991_f6p2_2444_isoform_c1991_full_length_coverage_6_non_full_length_coverage_2_isoform_length_2444_11602</td><td>XP_016428473.1 spindle assembly abnormal protein 6 homolog</td><td></td><td></td></tr>
<tr><td>2988|_|c662_f30p1_2444_isoform_c662_full_length_coverage_30_non_full_length_coverage_1_isoform_length_2444_20359</td><td>XP_022101545.1origin recognition complex subunit 3-like</td><td>F:GO:0003677; C:GO:0005664; P:GO:0006260</td><td></td></tr>
</table>

| | | | |
|---|---|---|---|
| 2989\|_\|c18068_f2p1_2444_isoform_c18068_full_length_coverage_2_non_full_length_coverage_1_isoform_length_2444_13549 | ---NA--- | | |
| TRINITY_DN3222_c0_g1_i1_len_312_213354.p1_type_internal_len_105 | XP_022257396.1uncharacterized protein KIAA1109-like, partial | | |
| TRINITY_DN15600_c0_g1_i1_len_312_213351.p1_type_internal_len_105 | ---NA--- | | |
| TRINITY_DN3566_c0_g1_i1_len_316_108734.p3_type_internal_len_105 | ---NA--- | | |
| TRINITY_DN6155_c0_g1_i1_len_369_247824.p1_type_3prime_partial_len_105 | ---NA--- | | |
| TRINITY_DN18382_c0_g1_i1_len_400_208017.p1_type_complete_len_104 | ---NA--- | | |
| TRINITY_DN10495_c0_g1_i1_len_321_108386.p1_type_5prime_partial_len_104 | ---NA--- | | |
| TRINITY_DN29322_c0_g1_i1_len_375_247497.p1_type_5prime_partial_len_104 | ---NA--- | | |
| TRINITY_DN3938_c0_g1_i1_len_537_275228.p1_type_5prime_partial_len_104 | ---NA--- | | |
| TRINITY_DN29006_c0_g1_i1_len_314_45466.p1_type_internal_len_105 | ---NA--- | | |
| TRINITY_DN19343_c0_g1_i1_len_1317_158658.p3_type_complete_len_104 | ---NA--- | | |
| TRINITY_DN17423_c0_g1_i1_len_710_65552.p1_type_5prime_partial_len_104 | ---NA--- | | |
| TRINITY_DN21353_c0_g2_i1_len_1827_156598.p2_type_5prime_partial_len_104 | ---NA--- | | |
| TRINITY_DN27521_c0_g1_i1_len_762_162807.p1_type_complete_len_104 | ---NA--- | | |
| TRINITY_DN7371_c0_g1_i1_len_315_45450.p1_type_internal_len_105 | ---NA--- | | |

| | | | |
|---|---|---|---|
| TRINITY_DN37659_c0_g1_i1_len_411_207563.p1_type_3prime_partial_len_105 | APP91326.1retinoid X receptor | F:GO:0003700; F:GO:0003707; C:GO:0005634; P:GO:0006355; F:GO:0008270; P:GO:0043401; F:GO:0043565 | |
| TRINITY_DN38002_c0_g1_i1_len_314_213163.p1_type_internal_len_105 | ---NA--- | | |
| TRINITY_DN21071_c0_g1_i1_len_883_127365.p1_type_5prime_partial_len_104 | ---NA--- | | |
| TRINITY_DN16654_c0_g1_i1_len_1394_158294.p2_type_complete_len_104 | ---NA--- | | |
| TRINITY_DN28173_c0_g1_i1_len_312_45653.p1_type_internal_len_105 | ---NA--- | | |
| TRINITY_DN5075_c0_g1_i1_len_516_242624.p3_type_5prime_partial_len_104 | ADC42878.1serine proteinase inhibitor 7 | C:GO:0005615; P:GO:0006508; F:GO:0008233 | |
| TRINITY_DN12628_c0_g1_i2_len_464_276943.p1_type_3prime_partial_len_105 | ---NA--- | | |
| TRINITY_DN28693_c0_g1_i4_len_572_202367.p1_type_5prime_partial_len_104 | ---NA--- | | |
| TRINITY_DN35130_c0_g1_i1_len_314_213216.p1_type_internal_len_105 | ---NA--- | | |
| TRINITY_DN5891_c0_g1_i1_len_416_40947.p1_type_5prime_partial_len_104 | ---NA--- | | |
| TRINITY_DN31523_c0_g1_i1_len_433_10650.p1_type_5prime_partial_len_104 | ---NA--- | | |
| TRINITY_DN41037_c0_g1_i1_len_503_204163.p1_type_5prime_partial_len_104 | ---NA--- | | |
| TRINITY_DN19899_c0_g1_i1_len_1553_296651.p2_type_5prime_partial_len_104 | ---NA--- | | |
| TRINITY_DN9370_c1_g1_i2_len_372_42568.p1_type_5prime_partial_len_104 | ---NA--- | | |
| TRINITY_DN3933_c0_g1_i1_len_316_311083.p1_type_internal_len_105 | ---NA--- | | |

| | | | |
|---|---|---|---|
| TRINITY_DN11525_c0_g1_i1_len_314_213205.p1_type_internal_len_105 | ---NA--- | | |
| TRINITY_DN27809_c0_g1_i1_len_312_45647.p1_type_internal_len_105 | ---NA--- | | |
| TRINITY_DN39501_c0_g1_i1_len_314_213214.p1_type_internal_len_105 | KRT84944.1Ankyrin repeat-containing protein | F:GO:0004842; P:GO:0016567; F:GO:0046872 | |
| TRINITY_DN37856_c0_g1_i1_len_314_213215.p1_type_internal_len_105 | AOR07226.1sodium leak channel non-selective | F:GO:0005261; C:GO:0016021; P:GO:0098655 | |
| TRINITY_DN2349_c0_g1_i1_len_312_213317.p1_type_internal_len_105 | ---NA--- | | |
| TRINITY_DN30637_c0_g1_i1_len_399_246424.p2_type_5prime_partial_len_104 | ---NA--- | | |
| TRINITY_DN13639_c0_g1_i1_len_339_309781.p1_type_5prime_partial_len_104 | ---NA--- | | |
| TRINITY_DN39667_c0_g1_i1_len_314_213223.p2_type_internal_len_105 | ---NA--- | | |
| TRINITY_DN13573_c0_g1_i1_len_815_127922.p4_type_5prime_partial_len_104 | ---NA--- | | |
| TRINITY_DN26274_c0_g1_i1_len_318_310948.p1_type_5prime_partial_len_104 | ---NA--- | | |
| TRINITY_DN21269_c0_g1_i2_len_312_213320.p1_type_internal_len_105 | ---NA--- | | |
| TRINITY_DN19283_c0_g1_i1_len_821_162204.p1_type_complete_len_104 | ---NA--- | | |
| TRINITY_DN27292_c0_g1_i1_len_334_107500.p1_type_5prime_partial_len_104 | ---NA--- | | |
| TRINITY_DN39517_c0_g1_i1_len_312_213324.p1_type_internal_len_105 | ---NA--- | | |
| TRINITY_DN21185_c0_g1_i1_len_312_213329.p1_type_internal_len_105 | ---NA--- | | |
| TRINITY_DN28601_c0_g1_i1_len_369_42693.p1_type_5prime_partial_len_104 | XP_018013922.1 E3 ubiquitin-protein ligase rnf146-like | F:GO:0003824; F:GO:0005488; P:GO:0009987 | |

| | | | |
|---|---|---|---|
| TRINITY_DN22004_c0_g1_i2_len_526_8292.p1_type_5prime_partial_len_104 | ---NA--- | | |
| TRINITY_DN21644_c0_g1_i1_len_1430_158132.p2_type_5prime_partial_len_104 | ---NA--- | | |
| TRINITY_DN38471_c0_g1_i1_len_312_213331.p1_type_internal_len_105 | ---NA--- | | |
| TRINITY_DN7274_c0_g1_i1_len_844_198091.p1_type_complete_len_104 | ---NA--- | | |
| TRINITY_DN9781_c0_g1_i1_len_312_213339.p1_type_internal_len_105 | ---NA--- | | |
| TRINITY_DN28372_c0_g1_i2_len_821_198353.p1_type_complete_len_104 | ---NA--- | | |
| TRINITY_DN21734_c0_g1_i2_len_789_162529.p2_type_5prime_partial_len_104 | ---NA--- | | |
| TRINITY_DN3795_c0_g1_i2_len_312_16076.p1_type_internal_len_105 | ---NA--- | | |
| TRINITY_DN32931_c0_g1_i1_len_314_213186.p2_type_internal_len_105 | ---NA--- | | |
| TRINITY_DN3452_c0_g1_i1_len_314_311191.p1_type_internal_len_105 | ---NA--- | | |
| TRINITY_DN31342_c0_g1_i1_len_314_213181.p1_type_internal_len_105 | ---NA--- | | |
| TRINITY_DN3464_c0_g1_i1_len_314_311231.p2_type_internal_len_105 | ---NA--- | | |
| TRINITY_DN19348_c0_g1_i1_len_314_213185.p1_type_internal_len_105 | ---NA--- | | |
| TRINITY_DN41738_c0_g1_i1_len_312_213347.p1_type_internal_len_105 | ---NA--- | | |
| TRINITY_DN9453_c0_g1_i1_len_314_311247.p2_type_internal_len_105 | ---NA--- | | |
| TRINITY_DN27168_c0_g1_i1_len_614_240551.p2_type_complete_len_104 | XP_018011765.1PREDICTED: uncharacterized protein LOC108668992 | | |

| | | | |
|---|---|---|---|
| TRINITY_DN21638_c0_g1_i7_len_707_239095.p1_type_3prime_partial_len_105 | ---NA--- | | |
| TRINITY_DN7465_c0_g1_i2_len_314_311242.p1_type_internal_len_105 | ---NA--- | | |
| TRINITY_DN15496_c0_g1_i1_len_314_311215.p3_type_internal_len_105 | ---NA--- | | |
| TRINITY_DN21990_c0_g1_i1_len_313_45580.p1_type_internal_len_105 | ---NA--- | | |
| TRINITY_DN14193_c0_g1_i1_len_361_210033.p1_type_5prime_partial_len_104 | ---NA--- | | |
| TRINITY_DN12504_c0_g1_i1_len_566_67682.p1_type_5prime_partial_len_104 | ---NA--- | | |
| TRINITY_DN5424_c0_g1_i2_len_312_45614.p1_type_internal_len_105 | ---NA--- | | |
| TRINITY_DN24106_c0_g4_i1_len_536_131562.p1_type_3prime_partial_len_105 | ---NA--- | | |
| TRINITY_DN19401_c0_g1_i2_len_404_207848.p1_type_3prime_partial_len_105 | ---NA--- | | |
| TRINITY_DN32524_c0_g1_i1_len_314_213191.p1_type_internal_len_105 | ---NA--- | | |
| TRINITY_DN36650_c0_g1_i1_len_312_213345.p1_type_internal_len_105 | XP_023716720.1solute carrier family 35 member G1 isoform X2 | C:GO:0016021 | |
| TRINITY_DN6919_c0_g1_i1_len_483_204782.p1_type_5prime_partial_len_104 | ---NA--- | | |
| TRINITY_DN41959_c0_g1_i1_len_314_213192.p1_type_internal_len_105 | ---NA--- | | |
| TRINITY_DN6380_c0_g1_i1_len_312_45617.p1_type_internal_len_105 | ---NA--- | | |
| TRINITY_DN12426_c0_g1_i1_len_569_274644.p2_type_complete_len_104 | ---NA--- | | |
| TRINITY_DN3041_c0_g1_i1_len_315_311162.p1_type_internal_len_105 | ---NA--- | | |

| | | | |
|---|---|---|---|
| TRINITY_DN19383_c0_g1_i2_len_1363_158412.p2_type_complete_len_104 | ---NA--- | | |
| TRINITY_DN40080_c0_g1_i1_len_314_213188.p1_type_internal_len_105 | ---NA--- | | |
| TRINITY_DN25636_c0_g1_i1_len_313_45592.p1_type_internal_len_105 | ---NA--- | | |
| TRINITY_DN17732_c0_g1_i1_len_715_238978.p1_type_5prime_partial_len_104 | ---NA--- | | |
| TRINITY_DN40897_c0_g1_i1_len_313_213277.p1_type_internal_len_105 | XP_018027710.1 probable cation-transporting ATPase 13A3 | P:GO:0006812; C:GO:0016020; C:GO:0016021; F:GO:0016887 | |
| TRINITY_DN21698_c0_g1_i1_len_516_203755.p1_type_5prime_partial_len_104 | ---NA--- | | |
| TRINITY_DN37611_c0_g1_i1_len_313_213275.p1_type_internal_len_105 | ABI20154.146 kDa salivary protein | F:GO:0003824 | |
| TRINITY_DN28656_c0_g1_i1_len_4069_187550.p1_type_complete_len_104 | ---NA--- | | |
| TRINITY_DN19672_c0_g1_i1_len_566_241460.p1_type_complete_len_104 | ---NA--- | | |
| TRINITY_DN9011_c0_g1_i2_len_356_308910.p1_type_5prime_partial_len_104 | ---NA--- | | |
| TRINITY_DN10534_c0_g1_i1_len_829_198273.p2_type_5prime_partial_len_104 | ---NA--- | | |
| TRINITY_DN25618_c0_g1_i1_len_313_108894.p1_type_internal_len_105 | ---NA--- | | |
| TRINITY_DN39601_c0_g1_i1_len_313_213301.p1_type_internal_len_105 | ---NA--- | | |
| TRINITY_DN19128_c0_g1_i1_len_1799_266789.p4_type_5prime_partial_len_104 | ---NA--- | | |
| TRINITY_DN40743_c0_g1_i1_len_440_206323.p1_type_5prime_partial_len_104 | ---NA--- | | |
| TRINITY_DN23962_c0_g1_i1_len_312_108927.p1_type_internal_len_105 | ---NA--- | | |

| | | | |
|---|---|---|---|
| TRINITY_DN37497_c0_g1_i1_len_313_213296.p1_type_internal_len_105 | XP_018007044.1 cytosolic purine 5'-nucleotidase-like isoform X1 | | |
| TRINITY_DN36103_c0_g1_i1_len_315_213138.p1_type_internal_len_105 | XP_023716704.1citron Rho-interacting kinase | F:GO:0005488; P:GO:0009987; F:GO:0016301 | |
| TRINITY_DN24059_c0_g1_i17_len_1129_125795.p2_type_3prime_partial_len_105 | ---NA--- | | |
| TRINITY_DN6925_c0_g1_i2_len_667_200506.p1_type_5prime_partial_len_104 | ---NA--- | | |
| TRINITY_DN31427_c0_g1_i1_len_363_280471.p1_type_5prime_partial_len_104 | ---NA--- | | |
| TRINITY_DN19246_c0_g1_i3_len_361_248223.p1_type_5prime_partial_len_104 | XP_009102801.1 chlorophyll a-b binding protein 1, chloroplastic-like | C:GO:0009522; C:GO:0009523; C:GO:0009535; P:GO:0009765; C:GO:0016021; F:GO:0016168; P:GO:0018298 | |
| TRINITY_DN36749_c0_g1_i1_len_315_213132.p1_type_internal_len_105 | XP_023340186.1alpha-mannosidase 2-like isoform X1 | | |
| TRINITY_DN28939_c0_g1_i1_len_5241_187074.p1_type_complete_len_1417 | PNF22602.1Tectonin beta-propeller repeat-containing protein | C:GO:0016021; F:GO:0030246 | |
| TRINITY_DN31173_c1_g1_i2_len_5438_187022.p1_type_5prime_partial_len_1417 | XP_018019844.1 kinesin-like protein KIF14 | | |
| TRINITY_DN29447_c0_g1_i1_len_4695_187231.p1_type_complete_len_1416 | OXA43762.1DNA-directed RNA polymerase, mitochondrial | F:GO:0016740 | |
| TRINITY_DN27881_c0_g1_i1_len_4495_187319.p1_type_5prime_partial_len_1416 | XP_026709775.1abnormal spindle-like microcephaly-associated protein | P:GO:0007051; F:GO:0019902 | |
| TRINITY_DN24036_c0_g1_i1_len_4487_121815.p1_type_complete_len_1415 | XP_018028199.1 cleavage and polyadenylation specificity factor subunit 1-like | F:GO:0003676; C:GO:0005634 | |
| TRINITY_DN29995_c0_g1_i1_len_4396_187364.p1_type_5prime_partial_len_1414 | XP_018020105.1 transcription termination factor 2-like | | |
| TRINITY_DN25499_c0_g1_i1_len_4601_227909.p1_type_complete_len_1414 | XP_007902718.1 rab3 GTPase-activating protein non-catalytic subunit | | |

| | | | |
|---|---|---|---|
| 321\|_\|c1884_f2p8_4237_isoform_c1884_full_length_coverage_2_non_full_length_coverage_8_isoform_length_4237_25560 | XP_018011297.1 hyaluronan mediated motility receptor-like | F:GO:0005540 | |
| TRINITY_DN30387_c0_g1_i2_len_4537_187298.p1_type_5prime_partial_len_1411 | XP_018007330.1 mucin-19-like | | |
| TRINITY_DN24132_c0_g1_i1_len_4851_57728.p1_type_5prime_partial_len_1410 | PNF27625.1Protein shuttle craft | F:GO:0003676; F:GO:0003700; C:GO:0005634; P:GO:0006355; F:GO:0008270 | |
| 2991\|_\|c6090_f2p3_2442_isoform_c6090_full_length_coverage_2_non_full_length_coverage_3_isoform_length_2442_17020 | XP_018022507.1 mannosyl-oligosaccharide 1,2-alpha-mannosidase IA-like | | |
| TRINITY_DN27646_c0_g2_i12_len_3202_188255.p1_type_complete_len_814 | XP_018023798.1 von Willebrand factor A domain-containing protein 5A-like | | |
| TRINITY_DN23582_c0_g1_i2_len_3234_228734.p1_type_complete_len_814 | XP_018015625.1 ubiquitin thioesterase trabid-like | F:GO:0046872 | |
| 2994\|_\|c1509_f16p7_2441_isoform_c1509_full_length_coverage_16_non_full_length_coverage_7_isoform_length_2441_15887 | OWF56314.1Nucleoporin NDC1 | C:GO:0016020; C:GO:0016021 | |
| 2996\|_\|c17963_f2p12_2440_isoform_c17963_full_length_coverage_2_non_full_length_coverage_12_isoform_length_2440_22593 | ANZ80589.1checkpoint kinase 1 | P:GO:0000077; F:GO:0004674; F:GO:0005524; P:GO:0006468 | EC:2.7.11 |
| 2997\|_\|c17869_f3p0_2440_isoform_c17869_full_length_coverage_3_non_full_length_coverage_0_isoform_length_2440_17828 | XP_018014862.1 tyrosine-protein kinase Src64B-like isoform X4 | | |
| 2998\|_\|c2011_f5p2_2439_isoform_c2011_full_length_coverage_5_non_full_length_coverage_2_isoform_length_2439_20641 | ---NA--- | | |
| 2999\|_\|c1917_f2p4_2439_isoform_c1917_full_length_coverage_2_non_full_length_coverage_4_isoform_length_2439_28073 | XP_018019666.1 structural maintenance of chromosomes protein 3-like isoform X1 | | |
| 3000\|_\|c9716_f5p3_2439_isoform_c9716_full_length_coverage_5_non_full_length_coverage_3_isoform_length_2439_21894 | ---NA--- | | |

| | | | |
|---|---|---|---|
| 3002\|_\|c18238_f2p0_2439_isoform_c18238_full_length_coverage_2_non_full_length_coverage_0_isoform_length_2439_13670 | XP_018023220.1 protein Hook homolog 3-like | | |
| TRINITY_DN13542_c0_g1_i2_len_415_207374.p1_type_5prime_partial_len_104 | ---NA--- | | |
| TRINITY_DN38484_c0_g1_i1_len_313_213293.p1_type_internal_len_105 | ---NA--- | | |
| TRINITY_DN5265_c0_g1_i1_len_312_213367.p1_type_internal_len_105 | ---NA--- | | |
| TRINITY_DN32508_c0_g1_i1_len_314_108812.p1_type_internal_len_105 | ---NA--- | | |
| TRINITY_DN14380_c0_g1_i1_len_314_108813.p1_type_internal_len_105 | ---NA--- | | |
| TRINITY_DN1771_c0_g1_i1_len_314_108852.p1_type_internal_len_105 | ---NA--- | | |
| TRINITY_DN34021_c0_g1_i1_len_315_213147.p1_type_internal_len_105 | XP_018011346.1 diacylglycerol kinase delta-like | F:GO:0016301; P:GO:0016310 | |
| TRINITY_DN29692_c0_g1_i1_len_313_108861.p2_type_internal_len_105 | XP_018019672.1 toll-like receptor Tollo | P:GO:0007165; C:GO:0016021 | |
| TRINITY_DN33502_c0_g1_i1_len_313_213289.p1_type_internal_len_105 | ---NA--- | | |
| TRINITY_DN23076_c0_g1_i1_len_603_302337.p3_type_5prime_partial_len_104 | ---NA--- | | |
| TRINITY_DN29424_c0_g1_i1_len_314_108837.p1_type_internal_len_105 | AFU60544.1transformer-2b | F:GO:0003723 | |
| TRINITY_DN1942_c0_g1_i1_len_313_213258.p1_type_internal_len_105 | ---NA--- | | |
| TRINITY_DN32289_c0_g1_i1_len_314_108823.p1_type_internal_len_105 | ---NA--- | | |
| TRINITY_DN1507_c0_g1_i1_len_676_200374.p1_type_5prime_partial_len_104 | ---NA--- | | |
| TRINITY_DN23324_c0_g1_i3_len_1994_231137.p4_type_5prime_partial_len_104 | ---NA--- | | |

| | | | |
|---|---|---|---|
| TRINITY_DN7230_c0_g1_i1_len_314_108848.p2_type_5prime_partial_len_104 | ---NA--- | | |
| TRINITY_DN21159_c0_g1_i1_len_485_243426.p1_type_5prime_partial_len_104 | ---NA--- | | |
| TRINITY_DN31340_c0_g1_i1_len_410_207593.p1_type_3prime_partial_len_105 | ---NA--- | | |
| TRINITY_DN36442_c0_g1_i1_len_481_243568.p1_type_5prime_partial_len_104 | ---NA--- | | |
| TRINITY_DN23666_c0_g1_i2_len_1003_63105.p1_type_5prime_partial_len_104 | ---NA--- | | |
| TRINITY_DN11872_c0_g1_i2_len_315_108783.p1_type_internal_len_105 | ---NA--- | | |
| TRINITY_DN40991_c0_g1_i1_len_313_213247.p1_type_internal_len_105 | XP_018028173.1PREDICTED: uncharacterized protein LOC108683371 | | |
| TRINITY_DN19312_c0_g1_i1_len_1023_160475.p2_type_5prime_partial_len_104 | ---NA--- | | |
| TRINITY_DN18243_c0_g1_i1_len_946_126909.p1_type_5prime_partial_len_104 | ---NA--- | | |
| TRINITY_DN17049_c0_g1_i1_len_643_240066.p1_type_complete_len_104 | ---NA--- | | |
| TRINITY_DN34681_c0_g1_i1_len_310_251351.p1_type_internal_len_104 | ---NA--- | | |
| TRINITY_DN34792_c0_g1_i1_len_310_251353.p1_type_internal_len_104 | ---NA--- | | |
| TRINITY_DN8179_c0_g1_i1_len_341_211333.p1_type_5prime_partial_len_103 | ---NA--- | | |
| TRINITY_DN3763_c0_g1_i1_len_310_251342.p1_type_internal_len_104 | AMJ52357.1cullin 4 | P:GO:0006511; C:GO:0031461; F:GO:0031625 | |
| TRINITY_DN32397_c0_g1_i1_len_310_251345.p1_type_internal_len_104 | ---NA--- | | |
| TRINITY_DN36011_c0_g1_i1_len_312_251235.p1_type_internal_len_104 | ---NA--- | | |

| | | | |
|---|---|---|---|
| TRINITY_DN29383_c0_g1_i1_len_309_251387.p1_type_internal_len_104 | ---NA--- | | |
| TRINITY_DN28468_c0_g1_i1_len_310_251348.p1_type_internal_len_104 | ---NA--- | | |
| TRINITY_DN31051_c0_g1_i1_len_309_16281.p2_type_internal_len_104 | ---NA--- | | |
| TRINITY_DN9813_c0_g1_i1_len_313_213294.p2_type_internal_len_104 | ---NA--- | | |
| TRINITY_DN28767_c0_g1_i1_len_310_251358.p1_type_internal_len_104 | ---NA--- | | |
| TRINITY_DN8971_c0_g1_i2_len_310_251365.p1_type_internal_len_104 | ---NA--- | | |
| TRINITY_DN12664_c0_g1_i1_len_341_211330.p1_type_3prime_partial_len_104 | ---NA--- | | |
| TRINITY_DN3777_c0_g1_i1_len_310_251367.p1_type_internal_len_104 | BAD16776.1calcification-associated peptide-2 | F:GO:0042302 | |
| TRINITY_DN2927_c0_g1_i1_len_447_168534.p2_type_5prime_partial_len_103 | ---NA--- | | |
| TRINITY_DN10732_c0_g1_i2_len_309_251380.p1_type_internal_len_104 | ---NA--- | | |
| TRINITY_DN61_c0_g1_i1_len_568_274657.p1_type_complete_len_103 | ---NA--- | | |
| TRINITY_DN35498_c0_g1_i1_len_311_251282.p2_type_internal_len_104 | ---NA--- | | |
| TRINITY_DN5514_c0_g1_i1_len_311_251251.p1_type_internal_len_104 | ---NA--- | | |
| TRINITY_DN32733_c0_g1_i1_len_351_210649.p1_type_5prime_partial_len_103 | ---NA--- | | |
| TRINITY_DN35747_c0_g1_i1_len_311_251248.p2_type_internal_len_104 | XP_021935571.1X-linked retinitis pigmentosa GTPase regulator-like isoform X2 | | |
| TRINITY_DN20867_c1_g1_i1_len_383_12403.p2_type_5prime_partial_len_103 | ---NA--- | | |

| | | | |
|---|---|---|---|
| TRINITY_DN34692_c0_g1_i1_len_311_251249.p1_type_internal_len_104 | AXF35707.1CWO | F:GO:0003677; C:GO:0005634; P:GO:0006355; F:GO:0046983 | |
| TRINITY_DN30348_c0_g1_i1_len_813_198457.p2_type_5prime_partial_len_103 | ---NA--- | | |
| TRINITY_DN35219_c0_g1_i1_len_311_251262.p1_type_internal_len_104 | ---NA--- | | |
| TRINITY_DN34101_c0_g1_i1_len_309_174873.p1_type_internal_len_104 | ---NA--- | | |
| TRINITY_DN2833_c0_g1_i1_len_311_251266.p1_type_internal_len_104 | XP_023018200.1rab GTPase-activating protein 1-like isoform X2 | F:GO:0005096; C:GO:0005623; P:GO:0006886; F:GO:0017137; P:GO:0090630 | |
| TRINITY_DN28827_c0_g1_i1_len_312_45603.p1_type_internal_len_104 | XP_015835912.1 nuclear receptor coactivator 2 isoform X6 | | |
| TRINITY_DN1246_c0_g1_i1_len_311_251281.p3_type_internal_len_104 | AOG13000.1serotonin receptor type 1A | F:GO:0004930; P:GO:0007186; C:GO:0016021 | |
| TRINITY_DN14226_c0_g1_i1_len_489_204589.p1_type_5prime_partial_len_103 | ---NA--- | | |
| TRINITY_DN32351_c0_g1_i1_len_311_251253.p1_type_internal_len_104 | ---NA--- | | |
| TRINITY_DN16224_c0_g1_i1_len_487_276332.p1_type_complete_len_103 | ---NA--- | | |
| TRINITY_DN4967_c0_g1_i1_len_312_16095.p1_type_internal_len_104 | XP_018023988.1 gamma-butyrobetaine dioxygenase-like | C:GO:0005739; F:GO:0016702; P:GO:0045329 | EC:1.13.11 |
| TRINITY_DN9723_c1_g1_i1_len_311_251307.p1_type_internal_len_104 | XP_013091249.1 neuroblastoma-amplified sequence-like | P:GO:0006890 | |
| TRINITY_DN866_c0_g1_i1_len_312_251237.p1_type_internal_len_104 | ---NA--- | | |
| TRINITY_DN28301_c0_g1_i1_len_310_251337.p1_type_internal_len_104 | ---NA--- | | |
| TRINITY_DN8214_c0_g1_i1_len_310_174847.p1_type_internal_len_104 | ---NA--- | | |
| TRINITY_DN645_c0_g1_i1_len_311_251240.p1_type_internal_len_104 | ---NA--- | | |

| | | | |
|---|---|---|---|
| TRINITY_DN8034_c0_g1_i1_len_796_4884.p3_type_complete_len_103 | XP_022659078.1ETS domain-containing protein Elk-1-like isoform X1 | | |
| TRINITY_DN32688_c0_g1_i1_len_311_251241.p1_type_internal_len_104 | XP_021696451.1inositol hexakisphosphate and diphosphoinositol-pentakisphosphate kinase 2 isoform X13 | | |
| TRINITY_DN26982_c0_g1_i1_len_310_251334.p1_type_internal_len_104 | ---NA--- | | |
| TRINITY_DN36968_c0_g1_i1_len_310_251333.p1_type_internal_len_104 | XP_017858565.1 patatin-like phospholipase domain-containing protein 3 isoform X2 | P:GO:0006629 | |
| TRINITY_DN18462_c0_g1_i1_len_310_16237.p2_type_internal_len_104 | XP_018027683.1 alpha-tocopherol transfer protein-like isoform X4 | | |
| TRINITY_DN27202_c0_g1_i1_len_311_251294.p1_type_internal_len_104 | XP_013398188.1intraflagellar transport protein 172 homolog | C:GO:0031514 | |
| TRINITY_DN11158_c0_g1_i2_len_364_137001.p1_type_5prime_partial_len_103 | ---NA--- | | |
| TRINITY_DN21778_c0_g1_i1_len_1822_231664.p3_type_complete_len_103 | ---NA--- | | |
| TRINITY_DN5076_c0_g1_i1_len_311_251285.p1_type_internal_len_104 | ---NA--- | | |
| TRINITY_DN33419_c0_g1_i1_len_414_207401.p1_type_5prime_partial_len_103 | ---NA--- | | |
| TRINITY_DN31842_c0_g1_i1_len_311_251298.p1_type_internal_len_104 | XP_021930432.1LIM domain and actin-binding protein 1 isoform X21 | | |
| TRINITY_DN32721_c0_g1_i1_len_311_251297.p1_type_internal_len_104 | XP_018023018.1 GRIP1-associated protein 1-like | | |
| TRINITY_DN35562_c0_g1_i1_len_319_250676.p1_type_5prime_partial_len_103 | ---NA--- | | |
| TRINITY_DN23200_c1_g3_i1_len_431_169073.p1_type_5prime_partial_len_103 | ---NA--- | | |
| TRINITY_DN23026_c0_g1_i1_len_4551_153521.p5_type_complete_len_103 | ---NA--- | | |

| | | | |
|---|---|---|---|
| TRINITY_DN21392_c0_g1_i1_len_940_236612.p1_type_complete_len_103 | ---NA--- | | |
| TRINITY_DN40027_c0_g1_i1_len_313_213225.p1_type_internal_len_104 | ---NA--- | | |
| TRINITY_DN14483_c0_g1_i2_len_2098_155835.p3_type_complete_len_103 | ---NA--- | | |
| TRINITY_DN20553_c0_g1_i1_len_481_243557.p1_type_5prime_partial_len_103 | ---NA--- | | |
| TRINITY_DN6025_c0_g1_i1_len_1069_33137.p2_type_5prime_partial_len_103 | XP_021914357.1ephrin-B1 isoform X1 | | |
| TRINITY_DN18786_c0_g1_i2_len_1435_193450.p2_type_complete_len_103 | ---NA--- | | |
| TRINITY_DN15949_c0_g1_i1_len_582_274408.p1_type_5prime_partial_len_103 | ---NA--- | | |
| TRINITY_DN24652_c0_g1_i1_len_1874_59616.p3_type_complete_len_103 | ---NA--- | | |
| TRINITY_DN22286_c0_g1_i1_len_1026_95436.p3_type_complete_len_103 | XP_026550341.1zinc finger protein 572-like | | |
| TRINITY_DN18332_c0_g1_i1_len_1645_192332.p2_type_3prime_partial_len_104 | ---NA--- | | |
| TRINITY_DN15764_c0_g1_i2_len_311_45717.p2_type_internal_len_104 | ---NA--- | | |
| TRINITY_DN4542_c0_g1_i1_len_391_12080.p1_type_5prime_partial_len_103 | ---NA--- | | |
| TRINITY_DN25306_c0_g1_i5_len_313_213280.p1_type_5prime_partial_len_103 | ---NA--- | | |
| TRINITY_DN16904_c0_g1_i1_len_646_240019.p3_type_5prime_partial_len_103 | ---NA--- | | |
| TRINITY_DN2379_c0_g1_i1_len_517_38367.p1_type_3prime_partial_len_104 | ---NA--- | | |
| TRINITY_DN22238_c0_g1_i2_len_1623_157340.p1_type_complete_len_103 | ---NA--- | | |

<table>
<tr><td>TRINITY_DN8309_c0_g1_i1_len_433_168997.p1_type_5prime_partial_len_103</td><td>XP_011874450.1 acylphosphatase-1-like isoform X5</td><td></td><td></td></tr>
<tr><td>TRINITY_DN22549_c1_g1_i5_len_798_4873.p1_type_5prime_partial_len_103</td><td>---NA---</td><td></td><td></td></tr>
<tr><td>TRINITY_DN2948_c0_g1_i1_len_309_251425.p1_type_internal_len_104</td><td>---NA---</td><td></td><td></td></tr>
<tr><td>TRINITY_DN37918_c0_g1_i1_len_309_251424.p1_type_internal_len_104</td><td>---NA---</td><td></td><td></td></tr>
<tr><td>TRINITY_DN14804_c0_g1_i1_len_552_202822.p1_type_3prime_partial_len_104</td><td>---NA---</td><td></td><td></td></tr>
<tr><td>TRINITY_DN16783_c0_g1_i1_len_428_40586.p1_type_complete_len_103</td><td>---NA---</td><td></td><td></td></tr>
<tr><td>3003|_|c2047_f2p4_2439_isoform_c2047_full_length_coverage_2_non_full_length_coverage_4_isoform_length_2439_28181</td><td>XP_018010693.1 chromodomain-helicase-DNA-binding protein Mi-2 homolog isoform X3</td><td></td><td></td></tr>
<tr><td>TRINITY_DN20291_c0_g1_i1_len_2980_29496.p1_type_complete_len_813</td><td>XP_013787947.1prolyl 3-hydroxylase OGFOD1-like isoform X2</td><td></td><td></td></tr>
<tr><td>3008|_|c2032_f5p10_2438_isoform_c2032_full_length_coverage_5_non_full_length_coverage_10_isoform_length_2438_20655</td><td>XP_018007946.1 neural Wiskott-Aldrich syndrome protein-like</td><td>F:GO:0003779; P:GO:0007015; P:GO:2000601</td><td></td></tr>
<tr><td>3009|_|c2127_f7p4_2438_isoform_c2127_full_length_coverage_7_non_full_length_coverage_4_isoform_length_2438_20726</td><td>---NA---</td><td></td><td></td></tr>
<tr><td>3010|_|c3388_f2p10_2438_isoform_c3388_full_length_coverage_2_non_full_length_coverage_10_isoform_length_2438_21346</td><td>XP_018006803.1 longitudinals lacking protein, isoforms H/M/V-like isoform X39</td><td>F:GO:0003676</td><td></td></tr>
<tr><td>3011|_|c46878_f2p2_2438_isoform_c46878_full_length_coverage_2_non_full_length_coverage_2_isoform_length_2438_19298</td><td>---NA---</td><td></td><td></td></tr>
<tr><td>3012|_|c8253_f3p1_2438_isoform_c8253_full_length_coverage_3_non_full_length_coverage_1_isoform_length_2438_12705</td><td>---NA---</td><td></td><td></td></tr>
</table>

| | | | |
|---|---|---|---|
| 3013\|_\|c2071_f6p4_2437_isoform_c2071_full_length_coverage_6_non_full_length_coverage_4_isoform_length_2437_11635 | ---NA--- | | |
| 3015\|_\|c13659_f6p1_2437_isoform_c13659_full_length_coverage_6_non_full_length_coverage_1_isoform_length_2437_22218 | EDO39006.1predicted protein | F:GO:0005524 | |
| 3017\|_\|c18122_f2p1_2437_isoform_c18122_full_length_coverage_2_non_full_length_coverage_1_isoform_length_2437_17967 | ---NA--- | | |
| TRINITY_DN22704_c0_g1_i1_len_312_45626.p1_type_internal_len_104 | ---NA--- | | |
| TRINITY_DN30992_c0_g1_i1_len_309_251403.p1_type_internal_len_104 | KZS14138.1Neurofibromin | P:GO:0007165; P:GO:0043087 | |
| TRINITY_DN34024_c0_g1_i1_len_309_251401.p1_type_3prime_partial_len_104 | ---NA--- | | |
| TRINITY_DN710_c0_g1_i1_len_309_251399.p1_type_internal_len_104 | ---NA--- | | |
| TRINITY_DN7774_c0_g1_i1_len_722_238881.p1_type_5prime_partial_len_103 | ---NA--- | | |
| TRINITY_DN30155_c0_g1_i1_len_1434_193459.p1_type_5prime_partial_len_103 | ---NA--- | | |
| TRINITY_DN24423_c0_g2_i1_len_309_251393.p1_type_internal_len_104 | ---NA--- | | |
| TRINITY_DN14451_c0_g1_i1_len_416_207343.p1_type_5prime_partial_len_103 | ---NA--- | | |
| TRINITY_DN9384_c0_g1_i2_len_807_64531.p1_type_5prime_partial_len_103 | ---NA--- | | |
| TRINITY_DN14589_c0_g1_i1_len_491_243286.p1_type_5prime_partial_len_103 | ---NA--- | | |
| TRINITY_DN14418_c0_g1_i2_len_943_236588.p2_type_3prime_partial_len_104 | ---NA--- | | |
| TRINITY_DN19213_c0_g1_i3_len_3709_228341.p6_type_complete_len_103 | ---NA--- | | |

| | | | |
|---|---|---|---|
| TRINITY_DN4436_c0_g1_i1_len_319_45180.p1_type_5prime_partial_len_103 | ---NA--- | | |
| TRINITY_DN17176_c0_g1_i1_len_639_240125.p1_type_complete_len_103 | ---NA--- | | |
| TRINITY_DN26879_c0_g1_i1_len_412_169777.p1_type_5prime_partial_len_103 | ---NA--- | | |
| TRINITY_DN20792_c0_g1_i1_len_3315_154039.p5_type_complete_len_103 | ---NA--- | | |
| TRINITY_DN28616_c1_g1_i1_len_1422_193524.p2_type_3prime_partial_len_104 | ---NA--- | | |
| TRINITY_DN17856_c0_g1_i1_len_506_242875.p1_type_5prime_partial_len_103 | ---NA--- | | |
| TRINITY_DN22423_c0_g1_i2_len_3307_121996.p5_type_complete_len_103 | ---NA--- | | |
| TRINITY_DN6118_c0_g1_i1_len_312_108941.p1_type_internal_len_104 | ---NA--- | | |
| TRINITY_DN24711_c0_g1_i1_len_312_140035.p1_type_internal_len_104 | ---NA--- | | |
| TRINITY_DN62_c0_g1_i1_len_662_129470.p2_type_complete_len_103 | ---NA--- | | |
| TRINITY_DN4124_c0_g1_i1_len_322_108287.p1_type_5prime_partial_len_103 | ---NA--- | | |
| TRINITY_DN14245_c0_g1_i1_len_595_99496.p3_type_5prime_partial_len_103 | ---NA--- | | |
| TRINITY_DN32708_c0_g1_i1_len_312_108949.p1_type_internal_len_104 | ---NA--- | | |
| TRINITY_DN12695_c0_g1_i1_len_313_15982.p1_type_internal_len_104 | ---NA--- | | |
| TRINITY_DN18872_c0_g1_i1_len_457_70337.p1_type_3prime_partial_len_104 | ---NA--- | | |
| TRINITY_DN32618_c0_g1_i1_len_320_108422.p1_type_5prime_partial_len_103 | ---NA--- | | |

| | | | |
|---|---|---|---|
| TRINITY_DN38245_c0_g1_i1_len_400_208021.p1_type_3prime_partial_len_104 | ---NA--- | | |
| TRINITY_DN875_c0_g1_i1_len_309_140287.p1_type_internal_len_104 | ---NA--- | | |
| TRINITY_DN33647_c0_g1_i1_len_309_140285.p1_type_internal_len_104 | ---NA--- | | |
| TRINITY_DN15946_c0_g1_i1_len_616_36723.p2_type_3prime_partial_len_104 | ---NA--- | | |
| TRINITY_DN7891_c0_g1_i1_len_315_251000.p1_type_5prime_partial_len_103 | ---NA--- | | |
| TRINITY_DN34473_c0_g1_i1_len_618_240495.p1_type_5prime_partial_len_103 | ---NA--- | | |
| TRINITY_DN27610_c0_g1_i1_len_312_139995.p2_type_internal_len_104 | ---NA--- | | |
| TRINITY_DN21047_c0_g4_i2_len_502_304121.p2_type_5prime_partial_len_103 | ---NA--- | | |
| TRINITY_DN18711_c0_g1_i2_len_1011_33429.p3_type_5prime_partial_len_103 | ---NA--- | | |
| TRINITY_DN38396_c0_g1_i1_len_400_208016.p1_type_3prime_partial_len_104 | ---NA--- | | |
| TRINITY_DN1897_c0_g1_i1_len_791_271853.p1_type_5prime_partial_len_103 | ---NA--- | | |
| TRINITY_DN21318_c0_g1_i4_len_824_4665.p2_type_5prime_partial_len_103 | ---NA--- | | |
| TRINITY_DN19273_c0_g1_i1_len_942_161156.p4_type_complete_len_103 | ---NA--- | | |
| TRINITY_DN28841_c0_g1_i1_len_524_275508.p3_type_3prime_partial_len_104 | ---NA--- | | |
| TRINITY_DN16340_c0_g1_i1_len_1797_156694.p2_type_5prime_partial_len_103 | ---NA--- | | |
| TRINITY_DN10186_c0_g1_i1_len_311_140088.p1_type_internal_len_104 | ---NA--- | | |

| | | | |
|---|---|---|---|
| TRINITY_DN29180_c0_g1_i1_len_461_244191.p1_type_5prime_partial_len_103 | ---NA--- | | |
| TRINITY_DN14580_c0_g1_i1_len_311_140079.p2_type_internal_len_104 | ---NA--- | | |
| TRINITY_DN16376_c0_g1_i2_len_311_140099.p1_type_internal_len_104 | ---NA--- | | |
| TRINITY_DN24461_c0_g1_i1_len_311_140102.p1_type_internal_len_104 | XP_018025653.1 protein ELYS-like | | |
| TRINITY_DN3056_c0_g1_i1_len_339_138331.p1_type_5prime_partial_len_103 | ---NA--- | | |
| TRINITY_DN1795_c0_g1_i1_len_392_279280.p1_type_5prime_partial_len_103 | ---NA--- | | |
| TRINITY_DN26823_c0_g1_i1_len_309_140236.p1_type_internal_len_104 | ---NA--- | | |
| TRINITY_DN11167_c0_g1_i2_len_610_99281.p1_type_5prime_partial_len_103 | ---NA--- | | |
| TRINITY_DN34399_c0_g1_i1_len_361_210072.p1_type_5prime_partial_len_103 | ---NA--- | | |
| TRINITY_DN27277_c0_g1_i1_len_310_140210.p1_type_internal_len_104 | ---NA--- | | |
| TRINITY_DN26303_c0_g1_i1_len_311_140060.p1_type_internal_len_104 | ---NA--- | | |
| TRINITY_DN34355_c0_g1_i1_len_309_140243.p1_type_internal_len_104 | XP_018015438.1 multidrug resistance-associated protein 1-like | | |
| TRINITY_DN2908_c0_g1_i1_len_309_16308.p1_type_internal_len_104 | ---NA--- | | |
| TRINITY_DN12507_c0_g1_i1_len_310_140183.p2_type_internal_len_104 | ---NA--- | | |
| TRINITY_DN4438_c0_g1_i1_len_310_140199.p1_type_internal_len_104 | XP_019329526.1 mismatch repair endonuclease PMS2 | P:GO:0006298; F:GO:0016887; C:GO:0032300 | EC:3.6.1.3; EC:3.6.1.15 |
| TRINITY_DN14452_c1_g1_i1_len_311_140072.p1_type_internal_len_104 | ---NA--- | | |

| | | | |
|---|---|---|---|
| TRINITY_DN33864_c0_g1_i1_len_313_213235.p1_type_internal_len_104 | ---NA--- | | |
| TRINITY_DN30733_c0_g1_i1_len_310_109101.p1_type_internal_len_104 | ---NA--- | | |
| TRINITY_DN29626_c0_g1_i1_len_311_174766.p1_type_internal_len_104 | ---NA--- | | |
| TRINITY_DN933_c0_g1_i1_len_310_109100.p1_type_internal_len_104 | ---NA--- | | |
| TRINITY_DN27879_c0_g1_i1_len_2254_190090.p3_type_5prime_partial_len_103 | ---NA--- | | |
| TRINITY_DN7082_c0_g1_i1_len_311_174761.p1_type_internal_len_104 | ---NA--- | | |
| TRINITY_DN24518_c0_g1_i1_len_310_109098.p2_type_internal_len_104 | XP_014667791.1 xaa-Pro aminopeptidase 1-like isoform X3 | | |
| TRINITY_DN2085_c0_g1_i1_len_330_138845.p1_type_5prime_partial_len_103 | ---NA--- | | |
| TRINITY_DN28360_c0_g1_i1_len_311_174757.p1_type_internal_len_104 | XP_018014905.1PREDICTED: uncharacterized protein LOC108671825 | C:GO:0016020; C:GO:0016021 | |
| TRINITY_DN2501_c0_g1_i1_len_313_251107.p1_type_internal_len_104 | ---NA--- | | |
| TRINITY_DN24526_c0_g1_i1_len_310_109077.p1_type_internal_len_104 | ---NA--- | | |
| TRINITY_DN16361_c0_g2_i1_len_513_275742.p1_type_5prime_partial_len_103 | ---NA--- | | |
| TRINITY_DN11700_c0_g1_i1_len_313_139926.p1_type_internal_len_104 | ---NA--- | | |
| TRINITY_DN32171_c0_g1_i1_len_310_109097.p1_type_internal_len_104 | ---NA--- | | |
| TRINITY_DN3627_c0_g1_i2_len_311_174758.p2_type_internal_len_104 | ---NA--- | | |
| TRINITY_DN27067_c0_g1_i1_len_313_251121.p1_type_internal_len_104 | XP_018027333.1 low-density lipoprotein receptor-related protein 2-like | | |

| | | | |
|---|---|---|---|
| TRINITY_DN12282_c0_g1_i1_len_313_213259.p1_type_internal_len_104 | ---NA--- | | |
| TRINITY_DN22452_c0_g1_i1_len_309_109144.p1_type_internal_len_104 | ---NA--- | | |
| TRINITY_DN4767_c0_g1_i1_len_312_251219.p1_type_internal_len_104 | ---NA--- | | |
| TRINITY_DN23149_c0_g1_i2_len_1963_123199.p4_type_complete_len_103 | ---NA--- | | |
| TRINITY_DN9459_c0_g1_i1_len_309_109153.p2_type_internal_len_104 | ---NA--- | | |
| TRINITY_DN13190_c0_g1_i2_len_311_174794.p1_type_internal_len_104 | ---NA--- | | |
| TRINITY_DN5713_c0_g1_i1_len_310_109115.p1_type_internal_len_104 | ---NA--- | | |
| TRINITY_DN22627_c0_g1_i1_len_310_109112.p1_type_internal_len_104 | ---NA--- | | |
| TRINITY_DN898_c0_g1_i1_len_310_109111.p1_type_internal_len_104 | ---NA--- | | |
| TRINITY_DN28873_c0_g1_i1_len_311_174786.p1_type_internal_len_104 | ---NA--- | | |
| TRINITY_DN29217_c0_g1_i1_len_312_251203.p2_type_internal_len_104 | EFX78838.1hypothetical protein DAPPUDRAFT_320144 | F:GO:0004866; C:GO:0005576; C:GO:0005615; P:GO:0010951; C:GO:0016020; C:GO:0016021 | |
| TRINITY_DN14838_c0_g1_i1_len_1480_157893.p2_type_5prime_partial_len_103 | ---NA--- | | |
| TRINITY_DN4929_c0_g1_i1_len_518_166684.p1_type_5prime_partial_len_103 | ---NA--- | | |
| TRINITY_DN13877_c0_g1_i1_len_892_96316.p4_type_5prime_partial_len_103 | ---NA--- | | |
| TRINITY_DN23870_c0_g1_i1_len_312_174669.p2_type_internal_len_104 | XP_023723845.1cytoplasmic dynein 2 light intermediate chain 1 isoform X1 | C:GO:0005868; C:GO:0005929; P:GO:0035721; P:GO:0035735 | |
| TRINITY_DN22769_c0_g1_i1_len_311_109013.p1_type_internal_len_104 | XP_009490169.1 bile salt-activated lipase | F:GO:0016787 | |

| | | | |
|---|---|---|---|
| TRINITY_DN25435_c0_g1_i1_len_379_42290.p1_type_5prime_partial_len_103 | ---NA--- | | |
| TRINITY_DN30472_c0_g1_i1_len_313_174637.p1_type_internal_len_104 | ---NA--- | | |
| TRINITY_DN22017_c0_g2_i2_len_699_239213.p2_type_5prime_partial_len_103 | ---NA--- | | |
| TRINITY_DN10216_c0_g1_i1_len_356_43300.p1_type_3prime_partial_len_104 | ---NA--- | | |
| TRINITY_DN20586_c0_g1_i1_len_1733_156914.p2_type_5prime_partial_len_103 | ---NA--- | | |
| TRINITY_DN13666_c0_g1_i1_len_459_70274.p2_type_5prime_partial_len_103 | ---NA--- | | |
| TRINITY_DN32358_c0_g1_i1_len_311_108994.p1_type_internal_len_104 | ---NA--- | | |
| TRINITY_DN25715_c0_g1_i1_len_313_174627.p1_type_internal_len_104 | ---NA--- | | |
| TRINITY_DN25931_c0_g1_i1_len_3997_228187.p1_type_complete_len_812 | XP_018010803.1 85/88 kDa calcium-independent phospholipase A2-like | | |
| TRINITY_DN25199_c0_g1_i3_len_2982_229009.p1_type_5prime_partial_len_812 | PNF16894.1RING finger protein unkempt-like protein | F:GO:0046872 | |
| TRINITY_DN29898_c0_g4_i1_len_2436_189601.p1_type_internal_len_813 | XP_024002309.1gastrula zinc finger protein XlCGF17.1-like | F:GO:0003676 | |
| TRINITY_DN21031_c0_g1_i1_len_3206_154130.p1_type_5prime_partial_len_812 | XP_017792338.1 inner nuclear membrane protein Man1 | | |
| 3022\|_\|c2009_f5p19_2436_isoform_c2009_full_length_coverage_5_non_full_length_coverage_19_isoform_length_2436_11611 | ALR99738.1nuclear autoantigenic sperm protein | | |
| 3023\|_\|c9662_f3p0_2436_isoform_c9662_full_length_coverage_3_non_full_length_coverage_0_isoform_length_2436_21889 | XP_018012434.1 gamma-glutamyltransferase ARB_02921-like | P:GO:0006508; P:GO:0006751; C:GO:0016020; C:GO:0016021; F:GO:0016740; F:GO:0036374 | |
| 3024\|_\|c33705_f8p0_2435_isoform_c33705_full_length_coverage_8_non_full_length_coverage_0_isoform_length_2435_23282 | XP_018021642.1 exocyst complex component 8-like isoform X1 | C:GO:0000145; P:GO:0006887 | |

| | | | |
|---|---|---|---|
| 3025\|_\|c2557_f8p2_2435_isoform_c2557_full_length_coverage_8_non_full_length_coverage_2_isoform_length_2435_21039 | XP_018017641.1 heat shock protein 75 kDa, mitochondrial-like | F:GO:0005524; P:GO:0006457; F:GO:0051082 | |
| 3026\|_\|c7960_f2p1_2434_isoform_c7960_full_length_coverage_2_non_full_length_coverage_1_isoform_length_2434_12680 | XP_018011713.1 mediator of RNA polymerase II transcription subunit 21-like | | |
| 3027\|_\|c25887_f2p2_2434_isoform_c25887_full_length_coverage_2_non_full_length_coverage_2_isoform_length_2434_13942 | XP_018017434.1 pre-mRNA 3' end processing protein WDR33-like | C:GO:0016020 | |
| TRINITY_DN6321_c0_g1_i1_len_311_109012.p1_type_internal_len_104 | XP_005110842.1 ankyrin repeat domain-containing protein 13C-like | | |
| TRINITY_DN8301_c0_g1_i1_len_312_139990.p2_type_internal_len_104 | ---NA--- | | |
| TRINITY_DN19097_c0_g1_i1_len_790_4926.p2_type_5prime_partial_len_103 | ---NA--- | | |
| TRINITY_DN14363_c0_g1_i2_len_313_139939.p1_type_internal_len_104 | ---NA--- | | |
| TRINITY_DN16777_c0_g1_i1_len_312_213313.p2_type_internal_len_104 | ---NA--- | | |
| TRINITY_DN34781_c0_g1_i1_len_313_251093.p1_type_internal_len_104 | ---NA--- | | |
| TRINITY_DN29702_c0_g1_i1_len_331_282125.p1_type_5prime_partial_len_103 | ---NA--- | | |
| TRINITY_DN26599_c0_g1_i1_len_322_174047.p1_type_5prime_partial_len_103 | ---NA--- | | |
| TRINITY_DN21638_c0_g1_i1_len_712_5607.p1_type_complete_len_103 | ---NA--- | | |
| TRINITY_DN26143_c0_g1_i1_len_311_109062.p2_type_internal_len_104 | XP_023327811.1long-chain-fatty-acid--CoA ligase 4-like | | |
| TRINITY_DN14940_c0_g1_i1_len_317_212967.p1_type_5prime_partial_len_103 | XP_013394242.1gamma-butyrobetaine dioxygenase-like | P:GO:0008152; F:GO:0051213 | |
| TRINITY_DN29918_c0_g1_i2_len_2216_190190.p2_type_3prime_partial_len_104 | ---NA--- | | |

| | | | |
|---|---|---|---|
| TRINITY_DN19339_c0_g1_i1_len_1195_61967.p4_type_5prime_partial_len_103 | ---NA--- | | |
| TRINITY_DN16166_c0_g1_i1_len_499_204289.p1_type_5prime_partial_len_103 | AFP23394.1neural precursor cell expressed developmentally down-regulated 8 | | |
| TRINITY_DN10184_c0_g2_i1_len_312_174700.p1_type_internal_len_104 | PRD19284.1zinc finger protein | F:GO:0003677 | |
| TRINITY_DN15552_c0_g1_i1_len_541_203121.p1_type_5prime_partial_len_103 | ---NA--- | | |
| TRINITY_DN10454_c0_g1_i1_len_312_174698.p1_type_internal_len_104 | ---NA--- | | |
| TRINITY_DN34690_c0_g1_i1_len_310_213490.p1_type_internal_len_104 | XP_012153062.1 laminin subunit alpha | F:GO:0005102; P:GO:0030155; P:GO:0030334; P:GO:0045995 | |
| TRINITY_DN13526_c0_g1_i1_len_543_241954.p1_type_5prime_partial_len_103 | ---NA--- | | |
| TRINITY_DN10087_c0_g1_i1_len_310_283380.p3_type_internal_len_104 | ---NA--- | | |
| TRINITY_DN31054_c0_g1_i2_len_6235_186901.p2_type_complete_len_103 | ---NA--- | | |
| TRINITY_DN4610_c0_g1_i1_len_419_134721.p1_type_5prime_partial_len_103 | ---NA--- | | |
| TRINITY_DN21837_c0_g1_i2_len_545_68090.p3_type_5prime_partial_len_103 | ---NA--- | | |
| TRINITY_DN17372_c0_g1_i1_len_688_200183.p2_type_5prime_partial_len_103 | ---NA--- | | |
| TRINITY_DN26410_c0_g1_i3_len_343_249186.p1_type_5prime_partial_len_103 | ---NA--- | | |
| TRINITY_DN4752_c0_g1_i1_len_417_134764.p1_type_5prime_partial_len_103 | ---NA--- | | |
| TRINITY_DN13321_c0_g1_i1_len_312_77124.p1_type_internal_len_104 | ---NA--- | | |
| TRINITY_DN9405_c1_g1_i1_len_313_77056.p1_type_internal_len_104 | ---NA--- | | |

| | | | |
|---|---|---|---|
| TRINITY_DN9104_c0_g1_i1_len_423_207029.p1_type_complete_len_103 | ---NA--- | | |
| TRINITY_DN22364_c0_g1_i1_len_1323_158627.p4_type_complete_len_103 | ---NA--- | | |
| TRINITY_DN31552_c0_g1_i1_len_310_213483.p1_type_internal_len_104 | ---NA--- | | |
| TRINITY_DN4675_c0_g1_i1_len_2184_91846.p3_type_5prime_partial_len_103 | ---NA--- | | |
| TRINITY_DN2131_c0_g1_i1_len_312_16041.p1_type_internal_len_104 | ---NA--- | | |
| TRINITY_DN38274_c0_g1_i1_len_310_213482.p1_type_internal_len_104 | ---NA--- | | |
| TRINITY_DN2126_c0_g1_i1_len_382_208936.p1_type_5prime_partial_len_103 | ---NA--- | | |
| TRINITY_DN24179_c0_g1_i1_len_309_283409.p1_type_internal_len_104 | XP_018008324.1 cadherin-23-like | F:GO:0005509; C:GO:0005886; P:GO:0007155; P:GO:0007156; C:GO:0016020; C:GO:0016021 | |
| TRINITY_DN29863_c0_g1_i1_len_379_171101.p1_type_5prime_partial_len_103 | ---NA--- | | |
| TRINITY_DN23622_c0_g1_i2_len_720_163274.p1_type_complete_len_103 | ---NA--- | | |
| TRINITY_DN9840_c0_g1_i1_len_312_77080.p1_type_internal_len_104 | ---NA--- | | |
| TRINITY_DN28373_c0_g1_i1_len_469_39424.p1_type_5prime_partial_len_103 | ---NA--- | | |
| TRINITY_DN38974_c0_g1_i1_len_310_213486.p2_type_internal_len_104 | ---NA--- | | |
| TRINITY_DN28601_c0_g1_i1_len_310_16216.p1_type_internal_len_104 | KFM59872.1Microtubule-associated proteins 1A/1B light chain 3A | C:GO:0005737; P:GO:0006914 | |
| TRINITY_DN37917_c0_g1_i1_len_310_213525.p1_type_internal_len_104 | XP_022238456.1collagen type IV alpha-3-binding protein-like | | |
| TRINITY_DN8129_c0_g1_i1_len_310_213527.p1_type_internal_len_104 | ASW35089.1carboxypeptidase D-like protein | F:GO:0004181; P:GO:0006508; F:GO:0008270 | |

| | | | |
|---|---|---|---|
| TRINITY_DN21088_c0_g1_i1_len_868_237246.p1_type_complete_len_103 | ---NA--- | | |
| TRINITY_DN6570_c0_g1_i2_len_311_16156.p2_type_internal_len_104 | ACL36280.1Kazal-type serine proteinase inhibitor 1 | | |
| TRINITY_DN22043_c0_g1_i1_len_2391_155195.p3_type_3prime_partial_len_104 | ---NA--- | | |
| TRINITY_DN17699_c0_g1_i3_len_2125_266011.p4_type_5prime_partial_len_103 | ---NA--- | | |
| TRINITY_DN27016_c0_g1_i1_len_311_77221.p3_type_internal_len_104 | XP_018025284.1 F-box/LRR-repeat protein 7-like | | |
| TRINITY_DN32282_c0_g1_i1_len_310_213528.p1_type_internal_len_104 | ---NA--- | | |
| TRINITY_DN14598_c0_g2_i1_len_647_164259.p1_type_5prime_partial_len_103 | ACQ66008.1penaeidin 5 antimicrobial peptide | C:GO:0005737; F:GO:0008061; P:GO:0022404; P:GO:0031640; P:GO:0042742; P:GO:0050832 | |
| TRINITY_DN4761_c0_g1_i1_len_311_283308.p5_type_5prime_partial_len_103 | ---NA--- | | |
| TRINITY_DN21349_c0_g1_i1_len_753_199241.p1_type_5prime_partial_len_103 | ---NA--- | | |
| TRINITY_DN28719_c0_g1_i1_len_1867_191412.p3_type_5prime_partial_len_103 | ---NA--- | | |
| TRINITY_DN10050_c0_g1_i1_len_313_311291.p1_type_internal_len_104 | ---NA--- | | |
| TRINITY_DN36378_c0_g1_i1_len_310_213510.p1_type_internal_len_104 | ---NA--- | | |
| TRINITY_DN7405_c0_g1_i1_len_1236_194706.p2_type_complete_len_103 | ---NA--- | | |
| TRINITY_DN9854_c0_g1_i1_len_310_213514.p1_type_internal_len_104 | ---NA--- | | |
| TRINITY_DN40204_c0_g1_i1_len_310_213516.p1_type_internal_len_104 | ---NA--- | | |
| TRINITY_DN22566_c0_g1_i1_len_310_283354.p1_type_internal_len_104 | ---NA--- | | |

| | | | |
|---|---|---|---|
| TRINITY_DN21075_c0_g1_i1_len_310_283352.p1_type_internal_len_104 | XP_018013891.1 rho guanine nucleotide exchange factor 17-like | F:GO:0005089; P:GO:0030036 | |
| TRINITY_DN35284_c0_g1_i1_len_310_213502.p1_type_internal_len_104 | ---NA--- | | |
| TRINITY_DN10914_c0_g1_i1_len_313_311299.p1_type_internal_len_104 | XP_018021065.1 protein white-like | F:GO:0000166; C:GO:0016020 | |
| TRINITY_DN9649_c0_g1_i1_len_310_283357.p1_type_internal_len_104 | ---NA--- | | |
| TRINITY_DN21408_c0_g1_i2_len_792_198713.p2_type_5prime_partial_len_103 | ---NA--- | | |
| TRINITY_DN3221_c0_g1_i1_len_328_212188.p1_type_3prime_partial_len_104 | KPJ06763.1Transcription factor collier | F:GO:0003677; C:GO:0005634; P:GO:0006355; P:GO:0007275; F:GO:0046872 | |
| TRINITY_DN5859_c0_g1_i1_len_479_204913.p2_type_5prime_partial_len_103 | ---NA--- | | |
| TRINITY_DN27669_c0_g1_i1_len_310_283336.p1_type_internal_len_104 | ---NA--- | | |
| TRINITY_DN25897_c0_g1_i1_len_311_77178.p1_type_3prime_partial_len_104 | ---NA--- | | |
| TRINITY_DN40162_c0_g1_i1_len_310_213517.p2_type_internal_len_104 | ---NA--- | | |
| TRINITY_DN24937_c0_g1_i1_len_310_16210.p1_type_internal_len_104 | ---NA--- | | |
| TRINITY_DN21969_c0_g1_i1_len_311_77168.p1_type_internal_len_104 | ASY04991.1crustacyanin A2 | F:GO:0031409 | |
| TRINITY_DN22184_c0_g1_i2_len_3035_154297.p3_type_5prime_partial_len_103 | ---NA--- | | |
| TRINITY_DN5246_c0_g1_i1_len_310_283338.p1_type_internal_len_104 | ---NA--- | | |
| TRINITY_DN4631_c0_g1_i1_len_369_247821.p2_type_3prime_partial_len_104 | ---NA--- | | |
| TRINITY_DN9593_c0_g1_i1_len_311_213411.p1_type_internal_len_104 | ---NA--- | | |

| | | | |
|---|---|---|---|
| TRINITY_DN10986_c0_g1_i1_len_310_311496.p1_type_internal_len_104 | ---NA--- | | |
| TRINITY_DN14075_c0_g1_i1_len_311_213417.p1_type_internal_len_104 | ---NA--- | | |
| TRINITY_DN18549_c0_g2_i1_len_674_200395.p1_type_complete_len_103 | XP_021001679.1zinc finger protein 525-like isoform X2 | F:GO:0003676; F:GO:0003677 | |
| TRINITY_DN17171_c0_g1_i1_len_311_213408.p1_type_internal_len_104 | ---NA--- | | |
| TRINITY_DN2388_c0_g1_i1_len_373_171381.p1_type_5prime_partial_len_103 | ---NA--- | | |
| TRINITY_DN16126_c0_g1_i1_len_934_299200.p3_type_5prime_partial_len_103 | XP_022253724.1nicotinamide phosphoribosyltransferase-like isoform X2 | | |
| TRINITY_DN828_c0_g1_i1_len_309_311520.p1_type_internal_len_104 | ---NA--- | | |
| TRINITY_DN23611_c0_g2_i2_len_893_127288.p2_type_complete_len_103 | PIK55799.1hypothetical protein BSL78_07287 | | |
| TRINITY_DN18638_c0_g1_i1_len_311_16162.p1_type_internal_len_104 | ---NA--- | | |
| TRINITY_DN12081_c0_g1_i1_len_310_311466.p1_type_internal_len_104 | ---NA--- | | |
| TRINITY_DN5124_c0_g1_i1_len_311_213420.p1_type_internal_len_104 | ---NA--- | | |
| TRINITY_DN11941_c0_g1_i1_len_397_208164.p1_type_5prime_partial_len_103 | ---NA--- | | |
| TRINITY_DN10858_c0_g1_i1_len_310_311491.p1_type_internal_len_104 | PIK37135.1hypothetical protein BSL78_26032 | | |
| TRINITY_DN21529_c1_g1_i10_len_311_213422.p1_type_internal_len_104 | ---NA--- | | |
| TRINITY_DN12385_c0_g1_i1_len_310_311490.p1_type_internal_len_104 | ---NA--- | | |
| TRINITY_DN29014_c0_g1_i1_len_310_311488.p1_type_internal_len_104 | ---NA--- | | |

<table>
<tr><td>TRINITY_DN29589_c0_g1_i1_len_310_311475.p1_type_internal_len_104</td><td>---NA---</td><td></td><td></td></tr>
<tr><td>TRINITY_DN2202_c0_g1_i1_len_311_213379.p1_type_internal_len_104</td><td>---NA---</td><td></td><td></td></tr>
<tr><td>TRINITY_DN33716_c0_g1_i1_len_311_213375.p2_type_internal_len_104</td><td>---NA---</td><td></td><td></td></tr>
<tr><td>TRINITY_DN4675_c0_g1_i1_len_756_199191.p1_type_complete_len_103</td><td>---NA---</td><td></td><td></td></tr>
<tr><td>TRINITY_DN16850_c0_g1_i1_len_835_198198.p3_type_5prime_partial_len_103</td><td>---NA---</td><td></td><td></td></tr>
<tr><td>TRINITY_DN42344_c0_g1_i1_len_311_213391.p1_type_5prime_partial_len_103</td><td>---NA---</td><td></td><td></td></tr>
<tr><td>TRINITY_DN30258_c0_g1_i1_len_309_311555.p1_type_internal_len_104</td><td>---NA---</td><td></td><td></td></tr>
<tr><td>TRINITY_DN3469_c0_g1_i1_len_311_213398.p1_type_internal_len_104</td><td>---NA---</td><td></td><td></td></tr>
<tr><td>3028|_|c3957_f2p10_2434_isoform_c3957_full_length_coverage_2_non_full_length_coverage_10_isoform_length_2434_16812</td><td>---NA---</td><td></td><td></td></tr>
<tr><td>3029|_|c11495_f2p9_2433_isoform_c11495_full_length_coverage_2_non_full_length_coverage_9_isoform_length_2433_26113</td><td>---NA---</td><td></td><td></td></tr>
<tr><td>TRINITY_DN29577_c0_g1_i3_len_2715_189005.p1_type_complete_len_811</td><td>XP_016658319.1 zinc finger protein 271-like</td><td></td><td></td></tr>
<tr><td>TRINITY_DN29022_c0_g1_i1_len_2668_189094.p1_type_5prime_partial_len_811</td><td>XP_018025589.1 dnaJ homolog subfamily C member 10-like isoform X1</td><td>C:GO:0005623; P:GO:0045454</td><td></td></tr>
<tr><td>3032|_|c1738_f4p1_2433_isoform_c1738_full_length_coverage_4_non_full_length_coverage_1_isoform_length_2433_15965</td><td>XP_018012644.1 dynein light chain Tctex-type 1-like</td><td></td><td></td></tr>
<tr><td>3033|_|c1987_f4p1_2432_isoform_c1987_full_length_coverage_4_non_full_length_coverage_1_isoform_length_2432_16105</td><td>AKB96215.1heat shock protein</td><td>F:GO:0005524</td><td></td></tr>
</table>

| | | | |
|---|---|---|---|
| 3035\|_\|c1974_f6p4_2432_isoform_c1974_full_length_coverage_6_non_full_length_coverage_4_isoform_length_2432_11592 | XP_018018997.1 WD repeat-containing protein 55-like | | |
| 3038\|_\|c6865_f3p0_2431_isoform_c6865_full_length_coverage_3_non_full_length_coverage_0_isoform_length_2431_21639 | ---NA--- | | |
| TRINITY_DN23621_c0_g1_i2_len_5273_153425.p1_type_5prime_partial_len_810 | XP_018022170.1 dnaJ homolog subfamily C member 16-like isoform X1 | C:GO:0005623; C:GO:0016020; C:GO:0016021; P:GO:0045454 | |
| 3040\|_\|c8832_f3p1_2430_isoform_c8832_full_length_coverage_3_non_full_length_coverage_1_isoform_length_2430_12752 | XP_023704019.1serine/threonine-protein kinase S6KL isoform X1 | | |
| TRINITY_DN7482_c0_g1_i1_len_711_199821.p1_type_5prime_partial_len_103 | ---NA--- | | |
| TRINITY_DN22572_c0_g1_i2_len_738_5366.p2_type_3prime_partial_len_104 | ---NA--- | | |
| TRINITY_DN20759_c0_g1_i1_len_979_196732.p1_type_5prime_partial_len_103 | ---NA--- | | |
| TRINITY_DN24181_c0_g1_i4_len_1529_192946.p3_type_5prime_partial_len_103 | ---NA--- | | |
| TRINITY_DN19454_c0_g1_i1_len_311_311409.p2_type_internal_len_104 | ---NA--- | | |
| TRINITY_DN37812_c0_g1_i1_len_310_213479.p1_type_internal_len_104 | ---NA--- | | |
| TRINITY_DN10451_c0_g1_i1_len_310_213470.p1_type_internal_len_104 | XP_018025771.1 cytoplasmic dynein 2 heavy chain 1-like | | |
| TRINITY_DN23543_c0_g1_i1_len_1464_233199.p2_type_5prime_partial_len_103 | ---NA--- | | |
| TRINITY_DN32856_c0_g1_i1_len_310_213481.p1_type_internal_len_104 | ---NA--- | | |
| TRINITY_DN18585_c0_g1_i3_len_309_283427.p1_type_internal_len_104 | ---NA--- | | |
| TRINITY_DN13324_c0_g1_i1_len_1372_233659.p1_type_5prime_partial_len_103 | ---NA--- | | |

| | | | |
|---|---|---|---|
| TRINITY_DN3908_c0_g1_i1_len_311_213440.p1_type_internal_len_104 | XP_012237694.1histone-lysine N-methyltransferase 2C isoform X11 | F:GO:0042800; C:GO:0044666; F:GO:0046872; P:GO:0051568 | EC:2.1.1.43 |
| TRINITY_DN29664_c0_g1_i1_len_311_16161.p2_type_internal_len_104 | ---NA--- | | |
| TRINITY_DN39921_c0_g1_i1_len_311_213434.p1_type_internal_len_104 | XP_022909115.1WD repeat and FYVE domain-containing protein 3 | | |
| TRINITY_DN22371_c0_g1_i1_len_1110_195625.p2_type_5prime_partial_len_103 | ---NA--- | | |
| TRINITY_DN33548_c0_g1_i1_len_311_213437.p1_type_internal_len_104 | ---NA--- | | |
| TRINITY_DN3181_c0_g1_i1_len_311_213438.p1_type_internal_len_104 | ---NA--- | | |
| TRINITY_DN1818_c0_g1_i1_len_310_213452.p2_type_internal_len_104 | ---NA--- | | |
| TRINITY_DN19145_c0_g1_i1_len_516_203752.p1_type_5prime_partial_len_103 | ---NA--- | | |
| TRINITY_DN14368_c0_g1_i1_len_392_104703.p1_type_3prime_partial_len_104 | ---NA--- | | |
| TRINITY_DN590_c0_g1_i1_len_310_213460.p1_type_internal_len_104 | ---NA--- | | |
| TRINITY_DN37813_c0_g1_i1_len_310_213467.p1_type_internal_len_104 | ---NA--- | | |
| TRINITY_DN18055_c0_g1_i1_len_311_311433.p2_type_internal_len_104 | ---NA--- | | |
| TRINITY_DN33656_c0_g1_i1_len_311_213444.p1_type_internal_len_104 | ---NA--- | | |
| TRINITY_DN11611_c0_g1_i1_len_311_311426.p1_type_internal_len_104 | ---NA--- | | |
| TRINITY_DN27814_c0_g1_i1_len_309_77367.p1_type_internal_len_104 | ---NA--- | | |
| TRINITY_DN25133_c0_g1_i1_len_695_200085.p1_type_5prime_partial_len_103 | ---NA--- | | |

| | | | |
|---|---|---|---|
| TRINITY_DN14730_c0_g1_i2_len_309_77351.p1_type_internal_len_104 | ---NA--- | | |
| TRINITY_DN23775_c1_g1_i1_len_923_236765.p1_type_complete_len_103 | ---NA--- | | |
| TRINITY_DN4323_c0_g1_i1_len_309_213591.p1_type_internal_len_104 | AGA83299.1glutamine synthetase | F:GO:0004356; F:GO:0005524; P:GO:0006542 | EC:6.3.1.2 |
| TRINITY_DN24582_c0_g1_i1_len_942_126942.p1_type_5prime_partial_len_103 | ---NA--- | | |
| TRINITY_DN38394_c0_g1_i1_len_309_213588.p1_type_internal_len_104 | ---NA--- | | |
| TRINITY_DN8453_c0_g1_i1_len_1242_159092.p3_type_complete_len_103 | ---NA--- | | |
| TRINITY_DN34155_c0_g1_i1_len_312_213340.p2_type_5prime_partial_len_103 | ---NA--- | | |
| TRINITY_DN7391_c0_g1_i1_len_309_45798.p1_type_internal_len_104 | ---NA--- | | |
| TRINITY_DN2522_c0_g1_i1_len_354_210496.p1_type_5prime_partial_len_103 | ---NA--- | | |
| TRINITY_DN9919_c0_g1_i1_len_1083_95080.p2_type_5prime_partial_len_103 | ---NA--- | | |
| TRINITY_DN16542_c0_g1_i2_len_908_236877.p1_type_complete_len_103 | ---NA--- | | |
| TRINITY_DN21717_c0_g1_i1_len_310_45773.p1_type_internal_len_104 | ---NA--- | | |
| TRINITY_DN30189_c0_g1_i1_len_336_249637.p1_type_5prime_partial_len_103 | ---NA--- | | |
| TRINITY_DN32475_c0_g1_i1_len_357_172106.p1_type_5prime_partial_len_103 | XP_018020402.1 transcription factor AP-2-epsilon-like isoform X2 | F:GO:0003700; C:GO:0005634; P:GO:0006355 | |
| TRINITY_DN18296_c0_g1_i1_len_340_75285.p1_type_5prime_partial_len_103 | ---NA--- | | |
| TRINITY_DN2850_c0_g1_i1_len_402_170162.p1_type_5prime_partial_len_103 | ---NA--- | | |

| | | | |
|---|---|---|---|
| TRINITY_DN19720_c0_g1_i3_len_626_66706.p1_type_5prime_partial_len_103 | ---NA--- | | |
| TRINITY_DN12438_c0_g1_i2_len_354_308996.p1_type_5prime_partial_len_103 | KZS16524.1Alcohol dehydrogenase A | F:GO:0004032; C:GO:0005829; P:GO:0055114 | EC:1.1.1.21; EC:1.1.1.2 |
| TRINITY_DN20653_c0_g1_i1_len_1123_195507.p1_type_complete_len_103 | ---NA--- | | |
| TRINITY_DN21500_c0_g1_i3_len_1552_232765.p3_type_3prime_partial_len_104 | ---NA--- | | |
| TRINITY_DN1905_c0_g1_i1_len_597_274188.p2_type_complete_len_103 | ---NA--- | | |
| TRINITY_DN31152_c0_g1_i1_len_4619_187263.p5_type_3prime_partial_len_104 | ---NA--- | | |
| TRINITY_DN14490_c0_g1_i2_len_428_71311.p1_type_3prime_partial_len_104 | ---NA--- | | |
| TRINITY_DN9381_c0_g1_i1_len_401_104341.p1_type_5prime_partial_len_103 | ---NA--- | | |
| TRINITY_DN22100_c0_g1_i1_len_2511_189425.p2_type_5prime_partial_len_103 | ---NA--- | | |
| TRINITY_DN21347_c0_g1_i3_len_350_210769.p1_type_5prime_partial_len_103 | ---NA--- | | |
| TRINITY_DN23829_c0_g1_i2_len_1897_231418.p3_type_complete_len_103 | AWU67119.1putative triacylglycerol lipase | P:GO:0016042; F:GO:0016788 | |
| TRINITY_DN1579_c0_g1_i1_len_792_97102.p1_type_3prime_partial_len_104 | ---NA--- | | |
| TRINITY_DN6264_c0_g1_i1_len_309_213603.p1_type_internal_len_104 | ---NA--- | | |
| TRINITY_DN6736_c0_g1_i1_len_749_238538.p2_type_5prime_partial_len_103 | ---NA--- | | |
| TRINITY_DN15432_c0_g1_i1_len_596_165064.p1_type_3prime_partial_len_104 | ---NA--- | | |
| TRINITY_DN6273_c0_g1_i1_len_412_278560.p1_type_5prime_partial_len_103 | ---NA--- | | |

| | | | |
|---|---|---|---|
| TRINITY_DN16232_c0_g1_i1_len_1064_235618.p2_type_complete_len_103 | ---NA--- | | |
| TRINITY_DN13624_c0_g1_i1_len_498_101338.p1_type_5prime_partial_len_103 | ---NA--- | | |
| TRINITY_DN37971_c0_g1_i1_len_309_213593.p1_type_internal_len_104 | XP_023710572.1histone-lysine N-methyltransferase SETMAR-like | | |
| TRINITY_DN40326_c0_g1_i1_len_309_213601.p1_type_internal_len_104 | AXN72354.1Wnt10 | F:GO:0005102; C:GO:0005576; P:GO:0007275; P:GO:0016055 | |
| TRINITY_DN24167_c0_g1_i1_len_310_45745.p1_type_internal_len_104 | XP_014481887.1 collagen alpha-1(I) chain-like | C:GO:0005576; P:GO:0006030; F:GO:0008061 | |
| TRINITY_DN6042_c0_g1_i2_len_420_207139.p1_type_3prime_partial_len_104 | ---NA--- | | |
| TRINITY_DN12230_c0_g1_i1_len_310_45747.p1_type_3prime_partial_len_104 | ---NA--- | | |
| TRINITY_DN8682_c0_g1_i1_len_451_205912.p1_type_5prime_partial_len_103 | ---NA--- | | |
| TRINITY_DN10197_c0_g1_i1_len_501_101267.p1_type_5prime_partial_len_103 | ---NA--- | | |
| TRINITY_DN32276_c0_g1_i1_len_312_213334.p1_type_internal_len_104 | XP_018026203.1 glucose dehydrogenase [FAD, quinone]-like | | |
| TRINITY_DN26018_c0_g1_i1_len_1015_196428.p3_type_5prime_partial_len_103 | ---NA--- | | |
| TRINITY_DN8242_c0_g1_i1_len_331_75876.p1_type_5prime_partial_len_103 | ---NA--- | | |
| TRINITY_DN20400_c0_g1_i1_len_898_236970.p1_type_3prime_partial_len_104 | ---NA--- | | |
| TRINITY_DN34229_c0_g1_i1_len_309_213555.p2_type_internal_len_104 | XP_018024522.1 reelin-like | | |
| TRINITY_DN19805_c0_g1_i1_len_312_213342.p1_type_internal_len_104 | ---NA--- | | |
| TRINITY_DN33491_c0_g1_i1_len_349_210793.p1_type_5prime_partial_len_103 | ---NA--- | | |

| | | | |
|---|---|---|---|
| TRINITY_DN22629_c0_g1_i2_len_2325_122624.p4_type_3prime_partial_len_104 | ---NA--- | | |
| TRINITY_DN25395_c0_g1_i2_len_1020_196389.p3_type_complete_len_103 | XP_011442459.1PREDICTED: uncharacterized protein LOC105338874 | | |
| TRINITY_DN17077_c0_g2_i1_len_943_33807.p4_type_5prime_partial_len_103 | ---NA--- | | |
| TRINITY_DN1056_c0_g1_i1_len_310_77232.p3_type_internal_len_104 | XP_018018636.1PREDICTED: uncharacterized protein LOC108675156 | C:GO:0016020; C:GO:0016021 | |
| TRINITY_DN25315_c0_g1_i1_len_312_283227.p2_type_5prime_partial_len_103 | ---NA--- | | |
| TRINITY_DN28533_c0_g1_i1_len_311_283265.p1_type_internal_len_104 | ---NA--- | | |
| TRINITY_DN2912_c0_g1_i2_len_311_283250.p1_type_internal_len_104 | ---NA--- | | |
| TRINITY_DN26105_c0_g1_i1_len_311_283260.p1_type_internal_len_104 | ---NA--- | | |
| TRINITY_DN28870_c0_g1_i1_len_1892_191296.p4_type_5prime_partial_len_103 | ---NA--- | | |
| TRINITY_DN16756_c0_g1_i1_len_353_106438.p1_type_5prime_partial_len_103 | ---NA--- | | |
| TRINITY_DN18785_c0_g1_i1_len_849_127628.p1_type_5prime_partial_len_103 | ---NA--- | | |
| TRINITY_DN15030_c0_g2_i3_len_402_135335.p1_type_5prime_partial_len_103 | XP_018007781.1 probable palmitoyltransferase ZDHHC24 | | |
| TRINITY_DN10537_c0_g1_i2_len_312_283232.p1_type_internal_len_104 | ---NA--- | | |
| TRINITY_DN3634_c0_g1_i2_len_310_77290.p1_type_internal_len_104 | ---NA--- | | |
| TRINITY_DN2079_c0_g1_i1_len_311_283288.p1_type_internal_len_104 | ---NA--- | | |
| TRINITY_DN1145_c0_g1_i1_len_1229_234513.p1_type_5prime_partial_len_103 | ---NA--- | | |

<table>
<tr><td>TRINITY_DN10476_c0_g2_i1_len_310_77294.p1_type_internal_len_104</td><td>---NA---</td><td></td><td></td></tr>
<tr><td>TRINITY_DN39453_c0_g1_i1_len_309_213536.p1_type_internal_len_104</td><td>---NA---</td><td></td><td></td></tr>
<tr><td>TRINITY_DN13045_c0_g1_i1_len_658_200655.p1_type_5prime_partial_len_103</td><td>---NA---</td><td></td><td></td></tr>
<tr><td>TRINITY_DN29564_c0_g1_i1_len_413_71849.p1_type_5prime_partial_len_103</td><td>---NA---</td><td></td><td></td></tr>
<tr><td>TRINITY_DN27219_c0_g1_i1_len_903_236922.p3_type_5prime_partial_len_103</td><td>---NA---</td><td></td><td></td></tr>
<tr><td>TRINITY_DN23647_c2_g1_i6_len_1245_159065.p2_type_complete_len_103</td><td>---NA---</td><td></td><td></td></tr>
<tr><td>TRINITY_DN9561_c0_g1_i1_len_309_213532.p1_type_internal_len_103</td><td>---NA---</td><td></td><td></td></tr>
<tr><td>TRINITY_DN41215_c0_g1_i1_len_308_213652.p1_type_internal_len_103</td><td>---NA---</td><td></td><td></td></tr>
<tr><td>TRINITY_DN1713_c0_g1_i1_len_632_240245.p2_type_5prime_partial_len_102</td><td>---NA---</td><td></td><td></td></tr>
<tr><td>TRINITY_DN25194_c0_g1_i2_len_2757_229336.p1_type_5prime_partial_len_810</td><td>XP_018012734.1 uncharacterized threonine-rich GPI-anchored glycoprotein PJ4664.02-like</td><td>F:GO:0016301</td><td></td></tr>
<tr><td>3043|_|c2153_f4p1_2429_isoform_c2153_full_length_coverage_4_non_full_length_coverage_1_isoform_length_2429_16216</td><td>KFM73310.1Hippocampus abundant transcript 1 protein</td><td>F:GO:0005215; C:GO:0016021; P:GO:0055085</td><td></td></tr>
<tr><td>3044|_|c11284_f2p0_2429_isoform_c11284_full_length_coverage_2_non_full_length_coverage_0_isoform_length_2429_17428</td><td>KDR23879.1hypothetical protein L798_09202</td><td></td><td></td></tr>
<tr><td>3045|_|c2124_f5p1_2428_isoform_c2124_full_length_coverage_5_non_full_length_coverage_1_isoform_length_2428_20724</td><td>XP_018017707.1 transcription factor mef2A-like</td><td></td><td></td></tr>
<tr><td>TRINITY_DN21030_c0_g1_i1_len_2975_229021.p1_type_complete_len_809</td><td>XP_018026334.1 probable ATP-dependent RNA helicase DDX23</td><td>F:GO:0003676; F:GO:0004386; F:GO:0005524</td><td>EC:3.6.1.15</td></tr>
</table>

<table>
<tr><td>TRINITY_DN29886_c0_g1_i2_len_2802_188852.p1_type_5prime_partial_len_809</td><td>KDR23731.1RNA-binding protein 28</td><td>F:GO:0003676; F:GO:0003723</td><td></td></tr>
<tr><td>3049|_|c2501_f7p4_2427_isoform_c2501_full_length_coverage_7_non_full_length_coverage_4_isoform_length_2427_11921</td><td>XP_014270957.1jmjC domain-containing histone demethylation protein 1</td><td>F:GO:0005488</td><td></td></tr>
<tr><td>3050|_|c13518_f2p1_2427_isoform_c13518_full_length_coverage_2_non_full_length_coverage_1_isoform_length_2427_13144</td><td>---NA---</td><td></td><td></td></tr>
<tr><td>3051|_|c3588_f2p2_2427_isoform_c3588_full_length_coverage_2_non_full_length_coverage_2_isoform_length_2427_16780</td><td>---NA---</td><td></td><td></td></tr>
<tr><td>3052|_|c4993_f2p3_2426_isoform_c4993_full_length_coverage_2_non_full_length_coverage_3_isoform_length_2426_16913</td><td>XP_008274290.1 spermatogenesis-associated protein 5-like protein 1</td><td>F:GO:0005524</td><td></td></tr>
<tr><td>TRINITY_DN37912_c0_g1_i1_len_308_213630.p2_type_internal_len_103</td><td>---NA---</td><td></td><td></td></tr>
<tr><td>TRINITY_DN11249_c0_g1_i1_len_308_213612.p1_type_internal_len_103</td><td>---NA---</td><td></td><td></td></tr>
<tr><td>TRINITY_DN18718_c0_g1_i1_len_717_199741.p1_type_5prime_partial_len_102</td><td>---NA---</td><td></td><td></td></tr>
<tr><td>TRINITY_DN39799_c0_g1_i1_len_307_213694.p1_type_internal_len_103</td><td>---NA---</td><td></td><td></td></tr>
<tr><td>TRINITY_DN815_c0_g1_i1_len_308_213644.p1_type_internal_len_103</td><td>BBC20717.1integrin alpha ps</td><td>C:GO:0016021</td><td></td></tr>
<tr><td>TRINITY_DN13914_c0_g1_i1_len_308_213653.p2_type_internal_len_103</td><td>---NA---</td><td></td><td></td></tr>
<tr><td>TRINITY_DN22365_c0_g1_i1_len_717_128814.p1_type_complete_len_102</td><td>---NA---</td><td></td><td></td></tr>
<tr><td>TRINITY_DN21979_c0_g1_i1_len_928_197188.p1_type_3prime_partial_len_103</td><td>---NA---</td><td></td><td></td></tr>
<tr><td>TRINITY_DN4641_c0_g1_i1_len_308_213655.p1_type_internal_len_103</td><td>---NA---</td><td></td><td></td></tr>
</table>

| | | | |
|---|---|---|---|
| TRINITY_DN15106_c0_g1_i1_len_348_172606.p1_type_5prime_partial_len_102 | AOD27423.1presenilin 1 | C:GO:0000139; F:GO:0004190; C:GO:0005789; P:GO:0007219; C:GO:0016021; P:GO:0016485 | EC:3.4.23 |
| TRINITY_DN33859_c0_g1_i1_len_307_213746.p2_type_internal_len_103 | XP_022914014.1synaptogenesis protein syg-2 isoform X2 | C:GO:0016020; C:GO:0016021 | |
| TRINITY_DN4469_c0_g1_i1_len_306_213803.p1_type_internal_len_103 | ---NA--- | | |
| TRINITY_DN4855_c0_g1_i1_len_369_171539.p1_type_5prime_partial_len_102 | ---NA--- | | |
| TRINITY_DN5116_c0_g1_i1_len_309_213541.p1_type_internal_len_103 | ---NA--- | | |
| TRINITY_DN41121_c0_g1_i1_len_385_208767.p1_type_complete_len_102 | ---NA--- | | |
| TRINITY_DN17041_c0_g1_i1_len_309_213548.p1_type_internal_len_103 | XP_023327503.1uncharacterized protein LOC111700723 | | |
| TRINITY_DN12843_c0_g1_i1_len_310_213521.p1_type_internal_len_103 | ---NA--- | | |
| TRINITY_DN9726_c0_g1_i2_len_368_73814.p1_type_5prime_partial_len_102 | ---NA--- | | |
| TRINITY_DN6786_c0_g1_i1_len_308_213689.p1_type_internal_len_103 | ---NA--- | | |
| TRINITY_DN19076_c0_g1_i1_len_3353_29367.p2_type_complete_len_102 | ---NA--- | | |
| TRINITY_DN35090_c0_g1_i1_len_308_213616.p1_type_internal_len_103 | XP_018018407.1 dedicator of cytokinesis protein 4-like | F:GO:0005085; P:GO:0007264 | |
| TRINITY_DN13329_c0_g1_i1_len_308_213650.p1_type_5prime_partial_len_102 | ---NA--- | | |
| TRINITY_DN36198_c0_g1_i1_len_308_213627.p1_type_internal_len_103 | ---NA--- | | |
| TRINITY_DN5161_c0_g1_i1_len_308_213651.p1_type_internal_len_103 | ---NA--- | | |
| TRINITY_DN23371_c0_g1_i1_len_1815_231686.p1_type_5prime_partial_len_102 | ---NA--- | | |

| | | | |
|---|---|---|---|
| TRINITY_DN9358_c0_g1_i1_len_747_5284.p1_type_5prime_partial_len_102 | ---NA--- | | |
| TRINITY_DN877_c0_g1_i1_len_309_213535.p1_type_internal_len_103 | ---NA--- | | |
| TRINITY_DN3940_c0_g1_i1_len_308_213641.p1_type_internal_len_103 | XP_018019831.1 membrane-bound alkaline phosphatase-like isoform X2 | C:GO:0016020; F:GO:0016791 | |
| TRINITY_DN20202_c0_g1_i1_len_1533_267619.p4_type_complete_len_102 | ---NA--- | | |
| TRINITY_DN34685_c0_g1_i1_len_307_213744.p1_type_internal_len_103 | ---NA--- | | |
| TRINITY_DN34740_c0_g1_i1_len_307_213742.p1_type_internal_len_103 | ---NA--- | | |
| TRINITY_DN23905_c0_g1_i2_len_692_200128.p1_type_5prime_partial_len_102 | ---NA--- | | |
| TRINITY_DN32130_c0_g1_i1_len_309_213571.p2_type_internal_len_103 | ---NA--- | | |
| TRINITY_DN28621_c0_g1_i3_len_4089_187541.p4_type_5prime_partial_len_102 | ---NA--- | | |
| TRINITY_DN34245_c0_g1_i1_len_307_213703.p1_type_internal_len_103 | ---NA--- | | |
| TRINITY_DN1736_c0_g1_i1_len_369_73749.p1_type_5prime_partial_len_102 | XP_018026563.1 transmembrane 9 superfamily member 1-like | C:GO:0016020 | |
| TRINITY_DN6203_c0_g1_i1_len_308_213680.p1_type_internal_len_103 | ---NA--- | | |
| TRINITY_DN14884_c0_g2_i1_len_308_213672.p1_type_internal_len_103 | ---NA--- | | |
| TRINITY_DN10656_c0_g1_i1_len_410_207587.p1_type_5prime_partial_len_102 | ---NA--- | | |
| TRINITY_DN27192_c0_g1_i1_len_310_16246.p1_type_internal_len_103 | ---NA--- | | |
| TRINITY_DN15141_c0_g1_i1_len_1207_159294.p1_type_complete_len_102 | ---NA--- | | |

| | | | |
|---|---|---|---|
| TRINITY_DN21619_c0_g1_i1_len_1770_191770.p1_type_complete_len_102 | ---NA--- | | |
| TRINITY_DN39062_c0_g1_i1_len_308_213682.p1_type_internal_len_103 | ---NA--- | | |
| TRINITY_DN34431_c0_g1_i1_len_308_213684.p1_type_internal_len_103 | XP_008212995.1 TATA element modulatory factor isoform X2 | | |
| TRINITY_DN15577_c0_g1_i1_len_1838_231607.p2_type_complete_len_102 | ---NA--- | | |
| TRINITY_DN34097_c0_g1_i1_len_309_213561.p1_type_internal_len_103 | ASO76431.1Disabled Protein | P:GO:0032502 | |
| TRINITY_DN6484_c0_g1_i1_len_307_213711.p1_type_internal_len_103 | ---NA--- | | |
| TRINITY_DN10972_c0_g1_i1_len_602_201735.p3_type_3prime_partial_len_103 | ---NA--- | | |
| TRINITY_DN18209_c0_g1_i3_len_363_171810.p2_type_5prime_partial_len_102 | ---NA--- | | |
| TRINITY_DN9050_c0_g1_i1_len_306_213795.p1_type_internal_len_103 | ---NA--- | | |
| TRINITY_DN19052_c0_g1_i1_len_314_139908.p1_type_5prime_partial_len_102 | ABW82154.1crustin-like protein | C:GO:0005576; P:GO:0010466; F:GO:0030414 | |
| TRINITY_DN23222_c0_g1_i2_len_758_199140.p2_type_5prime_partial_len_102 | ---NA--- | | |
| TRINITY_DN35047_c0_g1_i1_len_307_213708.p1_type_internal_len_103 | ---NA--- | | |
| TRINITY_DN25895_c0_g1_i3_len_2296_230256.p2_type_complete_len_102 | ---NA--- | | |
| TRINITY_DN2920_c0_g1_i2_len_415_207375.p1_type_3prime_partial_len_103 | AKG54423.1interferon regulatory factor | F:GO:0044212 | |
| TRINITY_DN38799_c0_g1_i1_len_309_213567.p1_type_internal_len_103 | ---NA--- | | |
| TRINITY_DN2495_c1_g1_i1_len_308_213676.p1_type_internal_len_103 | ---NA--- | | |

| | | | |
|---|---|---|---|
| TRINITY_DN35654_c0_g1_i1_len_307_213713.p1_type_internal_len_103 | ---NA--- | | |
| TRINITY_DN14333_c0_g1_i3_len_635_240190.p1_type_5prime_partial_len_102 | ---NA--- | | |
| TRINITY_DN6053_c0_g1_i1_len_308_213670.p1_type_internal_len_103 | ---NA--- | | |
| TRINITY_DN31130_c0_g1_i1_len_309_213592.p1_type_internal_len_103 | ---NA--- | | |
| TRINITY_DN6897_c0_g1_i1_len_308_213666.p2_type_internal_len_103 | ABP97431.1alpha 2 macroglobulin | F:GO:0004866; C:GO:0005615; P:GO:0010951 | |
| TRINITY_DN37314_c0_g1_i1_len_310_213449.p1_type_internal_len_103 | ---NA--- | | |
| TRINITY_DN5101_c0_g1_i1_len_374_209288.p1_type_3prime_partial_len_103 | ---NA--- | | |
| TRINITY_DN34321_c0_g1_i1_len_318_250785.p1_type_5prime_partial_len_102 | ---NA--- | | |
| TRINITY_DN37109_c0_g1_i1_len_307_213697.p1_type_internal_len_103 | ---NA--- | | |
| TRINITY_DN6741_c0_g1_i1_len_307_213730.p2_type_internal_len_103 | XP_015520569.1 myosin-IIIb-like isoform X1 | | |
| TRINITY_DN16898_c0_g1_i1_len_307_213731.p1_type_internal_len_103 | ---NA--- | | |
| TRINITY_DN7418_c0_g1_i1_len_308_213659.p1_type_internal_len_103 | ---NA--- | | |
| TRINITY_DN37711_c0_g1_i1_len_587_202052.p1_type_complete_len_102 | ---NA--- | | |
| TRINITY_DN23345_c0_g1_i1_len_1827_231646.p4_type_5prime_partial_len_102 | ---NA--- | | |
| TRINITY_DN17361_c0_g1_i1_len_696_200067.p1_type_complete_len_102 | ---NA--- | | |
| TRINITY_DN21218_c0_g1_i1_len_308_213663.p1_type_internal_len_103 | ---NA--- | | |

| | | | |
|---|---|---|---|
| TRINITY_DN9971_c0_g1_i1_len_596_37005.p1_type_complete_len_102 | ---NA--- | | |
| TRINITY_DN40330_c0_g1_i1_len_309_213576.p1_type_internal_len_103 | ---NA--- | | |
| TRINITY_DN25505_c0_g1_i1_len_357_308838.p1_type_5prime_partial_len_102 | ---NA--- | | |
| TRINITY_DN31541_c0_g1_i1_len_310_213499.p1_type_internal_len_103 | ---NA--- | | |
| TRINITY_DN2216_c0_g1_i1_len_309_213578.p1_type_internal_len_103 | BBC20718.1integrin alpha V | C:GO:0016021 | |
| TRINITY_DN7534_c0_g1_i1_len_310_213480.p1_type_internal_len_103 | ---NA--- | | |
| TRINITY_DN960_c0_g1_i1_len_639_240117.p3_type_5prime_partial_len_102 | ---NA--- | | |
| TRINITY_DN876_c0_g1_i1_len_653_98644.p1_type_complete_len_102 | ---NA--- | | |
| TRINITY_DN14810_c0_g1_i1_len_370_209518.p1_type_5prime_partial_len_102 | ---NA--- | | |
| TRINITY_DN35681_c0_g1_i1_len_310_213458.p1_type_internal_len_103 | ---NA--- | | |
| TRINITY_DN1725_c0_g1_i1_len_309_213585.p1_type_internal_len_103 | ---NA--- | | |
| TRINITY_DN29185_c0_g2_i1_len_829_198270.p2_type_complete_len_102 | ---NA--- | | |
| TRINITY_DN38554_c0_g1_i1_len_310_213469.p1_type_internal_len_103 | KXJ10492.1RNA-directed DNA polymerase from mobile element jockey | | |
| TRINITY_DN39473_c0_g1_i1_len_307_213700.p1_type_internal_len_103 | ---NA--- | | |
| TRINITY_DN621_c0_g1_i1_len_309_213580.p1_type_internal_len_103 | AYA73279.1sirtuin 1 | F:GO:0008270; P:GO:0016575; F:GO:0017136; F:GO:0070403 | EC:3.5.1.98 |
| TRINITY_DN13333_c0_g1_i1_len_309_213582.p1_type_internal_len_103 | XP_014254870.1diacylglycerol kinase eta isoform X2 | F:GO:0003951; F:GO:0004143; P:GO:0007165; P:GO:0016310 | EC:2.7.1.23; EC:2.7.1.107 |

| | | | |
|---|---|---|---|
| TRINITY_DN7322_c0_g1_i1_len_310_251340.p1_type_internal_len_103 | XP_018013606.1 WD repeat-containing protein 19-like | | |
| TRINITY_DN36550_c0_g1_i1_len_310_251349.p1_type_internal_len_103 | XP_018012859.1 rab GTPase-activating protein 1-like | F:GO:0005096; P:GO:0043547 | |
| TRINITY_DN14761_c0_g1_i1_len_307_213765.p1_type_internal_len_103 | ---NA--- | | |
| TRINITY_DN34140_c0_g1_i1_len_310_251375.p1_type_internal_len_103 | ---NA--- | | |
| TRINITY_DN14396_c0_g1_i2_len_310_251378.p1_type_internal_len_103 | ---NA--- | | |
| TRINITY_DN23351_c0_g1_i1_len_1659_192264.p2_type_5prime_partial_len_102 | ---NA--- | | |
| TRINITY_DN19506_c0_g1_i1_len_1050_62807.p1_type_complete_len_102 | ---NA--- | | |
| TRINITY_DN21504_c0_g1_i1_len_1267_194467.p2_type_complete_len_102 | ---NA--- | | |
| TRINITY_DN23165_c0_g1_i1_len_1020_196390.p2_type_5prime_partial_len_102 | ---NA--- | | |
| TRINITY_DN22011_c0_g1_i2_len_725_5494.p1_type_5prime_partial_len_102 | ---NA--- | | |
| TRINITY_DN13844_c0_g1_i1_len_531_242226.p1_type_complete_len_102 | ---NA--- | | |
| 3053\|_\|c12902_f2p1_2426_isoform_c12902_full_length_coverage_2_non_full_length_coverage_1_isoform_length_2426_17553 | KDR18932.1Leucine-rich repeat serine/threonine-protein kinase 1 | | |
| 3056\|_\|c24900_f2p1_2426_isoform_c24900_full_length_coverage_2_non_full_length_coverage_1_isoform_length_2426_18340 | XP_021937995.1constitutive coactivator of peroxisome proliferator-activated receptor gamma-like isoform X2 | | |
| 3057\|_\|c4935_f2p0_2425_isoform_c4935_full_length_coverage_2_non_full_length_coverage_0_isoform_length_2425_12427 | XP_015914892.1zinc finger protein 271-like isoform X2 | | |

| | | | |
|---|---|---|---|
| 3058\|_\|c18006_f2p0_2425_isoform_c18006_full_length_coverage_2_non_full_length_coverage_0_isoform_length_2425_22625 | XP_019628150.1  fas-binding factor 1-like | P:GO:0060271; P:GO:0090162; C:GO:0097539 | |
| 3059\|_\|c18246_f2p2_2424_isoform_c18246_full_length_coverage_2_non_full_length_coverage_2_isoform_length_2424_18058 | ---NA--- | | |
| 3060\|_\|c19872_f2p1_2424_isoform_c19872_full_length_coverage_2_non_full_length_coverage_1_isoform_length_2424_26613 | ---NA--- | | |
| TRINITY_DN19481_c0_g1_i1_len_3310_228645.p1_type_complete_len_808 | XP_015925621.1sodium bicarbonate transporter-like protein 11 isoform X1 | F:GO:0005452; C:GO:0005887; P:GO:0015698; P:GO:0051453 | |
| 3062\|_\|c21029_f2p0_2424_isoform_c21029_full_length_coverage_2_non_full_length_coverage_0_isoform_length_2424_18207 | KZS17013.1ATP-dependent RNA Helicase pitchoune | F:GO:0000166; F:GO:0003676; F:GO:0003723; F:GO:0004386; F:GO:0005524; F:GO:0016787 | |
| TRINITY_DN28536_c0_g1_i2_len_3419_188018.p1_type_complete_len_808 | XP_023972069.1zinc finger protein 850 isoform X1 | F:GO:0003676; P:GO:0006355; F:GO:0046872 | |
| TRINITY_DN28099_c0_g1_i1_len_3267_188171.p1_type_complete_len_808 | XP_018008335.1 cell division cycle 5-like protein | F:GO:0003677; C:GO:0005634; P:GO:0051301 | |
| TRINITY_DN8813_c1_g1_i1_len_309_251416.p1_type_internal_len_103 | ---NA--- | | |
| TRINITY_DN29546_c0_g1_i1_len_415_71773.p1_type_5prime_partial_len_102 | ---NA--- | | |
| TRINITY_DN6339_c0_g1_i2_len_309_251431.p1_type_internal_len_103 | ---NA--- | | |
| TRINITY_DN3275_c0_g1_i1_len_309_251435.p1_type_internal_len_103 | ---NA--- | | |
| TRINITY_DN30198_c0_g1_i1_len_309_251383.p1_type_internal_len_103 | ---NA--- | | |
| TRINITY_DN597_c0_g1_i1_len_309_251397.p1_type_internal_len_103 | ---NA--- | | |
| TRINITY_DN13632_c0_g1_i1_len_309_251404.p1_type_3prime_partial_len_103 | XP_018025305.1 sodium/glucose cotransporter 4-like | | |

| | | | |
|---|---|---|---|
| TRINITY_DN1754_c0_g1_i1_len_309_251407.p1_type_internal_len_103 | ---NA--- | | |
| TRINITY_DN2054_c0_g1_i1_len_338_173100.p1_type_5prime_partial_len_102 | ---NA--- | | |
| TRINITY_DN15887_c0_g1_i1_len_401_72312.p1_type_5prime_partial_len_102 | ---NA--- | | |
| TRINITY_DN11850_c0_g1_i1_len_1642_267271.p1_type_complete_len_102 | ---NA--- | | |
| TRINITY_DN25900_c0_g2_i4_len_2069_230898.p1_type_complete_len_102 | ---NA--- | | |
| TRINITY_DN17245_c0_g1_i4_len_1502_267731.p2_type_complete_len_102 | ---NA--- | | |
| TRINITY_DN12473_c0_g1_i1_len_408_72051.p2_type_5prime_partial_len_102 | XP_018014793.1 pro-resilin-like | | |
| TRINITY_DN5145_c0_g1_i1_len_415_278458.p2_type_complete_len_102 | ---NA--- | | |
| TRINITY_DN17852_c0_g1_i1_len_1132_159748.p5_type_5prime_partial_len_102 | ---NA--- | | |
| TRINITY_DN32673_c0_g1_i1_len_310_16219.p1_type_internal_len_103 | ---NA--- | | |
| TRINITY_DN34907_c0_g1_i1_len_306_251635.p1_type_internal_len_103 | ASA69501.1penlectin 5-1 | | |
| TRINITY_DN35519_c0_g1_i1_len_329_212102.p1_type_5prime_partial_len_102 | ---NA--- | | |
| TRINITY_DN9714_c0_g1_i1_len_547_37774.p1_type_complete_len_102 | ---NA--- | | |
| TRINITY_DN23961_c1_g1_i1_len_306_251573.p1_type_internal_len_103 | XP_016420355.1 gastrula zinc finger protein XlCGF49.1-like | F:GO:0003676 | |
| TRINITY_DN35656_c0_g1_i1_len_306_251590.p1_type_internal_len_103 | ---NA--- | | |
| TRINITY_DN29155_c0_g1_i1_len_306_251598.p1_type_internal_len_103 | XP_018323976.2matrix metalloproteinase-2 | F:GO:0004222; C:GO:0005615; P:GO:0030198; P:GO:0030574; F:GO:0046872 | EC:3.4.24 |

| | | | |
|---|---|---|---|
| TRINITY_DN32508_c0_g1_i1_len_306_251612.p1_type_internal_len_103 | XP_018024595.1 polycomb protein Sfmbt-like isoform X1 | C:GO:0005634; P:GO:0006342; P:GO:0006355; F:GO:0008270; F:GO:0035064 | |
| TRINITY_DN24658_c0_g1_i1_len_461_277024.p2_type_complete_len_102 | ---NA--- | | |
| TRINITY_DN20367_c2_g1_i7_len_1679_267149.p3_type_complete_len_102 | ---NA--- | | |
| TRINITY_DN24275_c0_g1_i2_len_1941_191123.p5_type_3prime_partial_len_103 | ---NA--- | | |
| TRINITY_DN21141_c0_g1_i1_len_1068_62702.p4_type_5prime_partial_len_102 | ---NA--- | | |
| TRINITY_DN22482_c1_g1_i5_len_819_127877.p1_type_complete_len_102 | ---NA--- | | |
| TRINITY_DN3313_c0_g1_i2_len_407_306788.p1_type_3prime_partial_len_103 | XP_018007791.1 PHD finger protein 19-like | | |
| TRINITY_DN14474_c0_g1_i1_len_598_201821.p1_type_5prime_partial_len_102 | ---NA--- | | |
| TRINITY_DN18362_c0_g1_i1_len_1889_231446.p3_type_complete_len_102 | ---NA--- | | |
| TRINITY_DN8788_c0_g1_i1_len_307_213771.p2_type_internal_len_103 | ---NA--- | | |
| TRINITY_DN39099_c0_g1_i1_len_367_209720.p1_type_5prime_partial_len_102 | ---NA--- | | |
| TRINITY_DN7241_c0_g1_i1_len_353_210571.p1_type_5prime_partial_len_102 | ---NA--- | | |
| TRINITY_DN17916_c0_g1_i1_len_1419_158182.p2_type_3prime_partial_len_103 | AIX11620.1cyclin-dependent kinase 7 | F:GO:0005524; P:GO:0006468; F:GO:0008353; C:GO:0070985 | EC:2.7.11; EC:2.7.11.23 |
| TRINITY_DN27059_c0_g1_i1_len_308_251463.p1_type_internal_len_103 | XP_021942265.1protein KIAA0100 isoform X2 | | |
| TRINITY_DN28334_c0_g1_i1_len_308_251465.p1_type_internal_len_103 | XP_013923706.1 von Willebrand factor A domain-containing protein 8-like | | |

| | | | |
|---|---|---|---|
| TRINITY_DN30094_c0_g1_i1_len_308_251470.p1_type_internal_len_103 | XP_022574129.1ribulose bisphosphate carboxylase/oxygenase activase, chloroplastic isoform X2 | F:GO:0005524 | |
| TRINITY_DN8143_c0_g1_i1_len_308_251482.p1_type_internal_len_103 | XP_023706739.1nuclear hormone receptor FTZ-F1 beta isoform X2 | F:GO:0003700; F:GO:0003707; C:GO:0005634; P:GO:0006355; F:GO:0008270; P:GO:0043401; F:GO:0043565 | |
| TRINITY_DN37891_c0_g1_i1_len_308_251492.p1_type_internal_len_103 | ---NA--- | | |
| TRINITY_DN32466_c0_g1_i1_len_308_251494.p1_type_internal_len_103 | ---NA--- | | |
| TRINITY_DN7931_c0_g1_i1_len_307_251501.p1_type_internal_len_103 | ---NA--- | | |
| TRINITY_DN17424_c0_g1_i2_len_529_242288.p1_type_5prime_partial_len_102 | ---NA--- | | |
| TRINITY_DN4802_c0_g1_i1_len_307_251512.p1_type_internal_len_103 | XP_008483819.1Ig-like and fibronectin type-III domain-containing protein 1 | C:GO:0016020; C:GO:0016021 | |
| TRINITY_DN13479_c0_g1_i1_len_308_251441.p1_type_internal_len_103 | ---NA--- | | |
| TRINITY_DN33595_c0_g1_i1_len_308_251444.p1_type_internal_len_103 | ---NA--- | | |
| TRINITY_DN3372_c0_g1_i1_len_308_251446.p1_type_internal_len_103 | ---NA--- | | |
| TRINITY_DN36920_c0_g1_i1_len_308_251448.p1_type_internal_len_103 | ---NA--- | | |
| TRINITY_DN22523_c0_g1_i1_len_1910_191225.p4_type_3prime_partial_len_103 | ---NA--- | | |
| TRINITY_DN48_c0_g1_i1_len_308_251450.p1_type_internal_len_103 | ---NA--- | | |
| TRINITY_DN14982_c0_g1_i1_len_308_251461.p1_type_internal_len_103 | ---NA--- | | |

| | | | |
|---|---|---|---|
| TRINITY_DN27518_c0_g1_i1_len_307_251544.p1_type_internal_len_103 | AGO05992.1peroxinectin, partial | F:GO:0004601; P:GO:0006979; F:GO:0020037; P:GO:0055114; P:GO:0098869 | EC:1.11.1.7 |
| TRINITY_DN22041_c0_g1_i1_len_807_127996.p1_type_5prime_partial_len_102 | ---NA--- | | |
| TRINITY_DN33315_c0_g1_i1_len_307_251546.p1_type_internal_len_103 | ---NA--- | | |
| TRINITY_DN16373_c0_g1_i4_len_307_251555.p1_type_internal_len_103 | ---NA--- | | |
| TRINITY_DN32548_c0_g1_i1_len_307_251560.p1_type_internal_len_103 | XP_018026632.1 titin-like isoform X14 | | |
| TRINITY_DN3947_c0_g1_i1_len_306_251569.p1_type_internal_len_103 | ---NA--- | | |
| TRINITY_DN1741_c0_g1_i1_len_307_251514.p1_type_internal_len_103 | XP_023242687.1zinc finger MYND domain-containing protein 19-like isoform X2 | F:GO:0046872 | |
| TRINITY_DN33659_c0_g1_i1_len_307_251520.p1_type_internal_len_103 | ---NA--- | | |
| TRINITY_DN34093_c0_g1_i1_len_307_251524.p1_type_internal_len_103 | XP_018014150.1PREDICTED: uncharacterized protein LOC108671168 | | |
| TRINITY_DN32655_c0_g1_i1_len_307_251526.p1_type_internal_len_103 | ---NA--- | | |
| TRINITY_DN14996_c0_g1_i1_len_348_210893.p1_type_3prime_partial_len_103 | ---NA--- | | |
| TRINITY_DN4827_c0_g1_i1_len_307_251532.p1_type_internal_len_103 | ---NA--- | | |
| TRINITY_DN20082_c0_g1_i2_len_834_127744.p2_type_5prime_partial_len_102 | ETN58999.1dihydropteridine reductase | F:GO:0016491; P:GO:0055114 | |
| TRINITY_DN1366_c0_g1_i1_len_307_251535.p1_type_internal_len_103 | ---NA--- | | |
| TRINITY_DN6767_c0_g1_i1_len_307_251537.p1_type_internal_len_103 | ---NA--- | | |
| TRINITY_DN7909_c0_g1_i1_len_310_140223.p1_type_internal_len_103 | ---NA--- | | |

| | | | |
|---|---|---|---|
| TRINITY_DN20078_c0_g1_i1_len_1150_235039.p1_type_complete_len_102 | ---NA--- | | |
| TRINITY_DN25970_c0_g1_i1_len_307_283514.p2_type_internal_len_103 | ---NA--- | | |
| TRINITY_DN8518_c0_g1_i1_len_310_140192.p1_type_internal_len_103 | ---NA--- | | |
| TRINITY_DN32684_c0_g1_i1_len_308_140310.p1_type_internal_len_103 | ---NA--- | | |
| TRINITY_DN5454_c0_g1_i1_len_308_140314.p1_type_internal_len_103 | ---NA--- | | |
| TRINITY_DN25_c0_g1_i1_len_309_140246.p1_type_internal_len_103 | AFD52981.1glutamine synthetase 2 | F:GO:0004356; F:GO:0005524; P:GO:0006542 | EC:6.3.1.2 |
| TRINITY_DN27985_c0_g1_i1_len_309_140281.p1_type_internal_len_103 | ---NA--- | | |
| TRINITY_DN1071_c0_g1_i1_len_306_213780.p2_type_internal_len_103 | XP_018013475.1 protein sax-3-like | | |
| TRINITY_DN11149_c0_g1_i1_len_307_213755.p1_type_internal_len_103 | ---NA--- | | |
| TRINITY_DN1739_c0_g1_i1_len_306_283576.p1_type_internal_len_103 | ---NA--- | | |
| TRINITY_DN34006_c0_g1_i1_len_307_213747.p1_type_internal_len_103 | ---NA--- | | |
| TRINITY_DN12567_c0_g1_i1_len_568_7515.p3_type_5prime_partial_len_102 | ---NA--- | | |
| TRINITY_DN35620_c0_g1_i1_len_307_213752.p1_type_5prime_partial_len_102 | ---NA--- | | |
| TRINITY_DN35651_c0_g1_i1_len_362_209962.p1_type_5prime_partial_len_102 | ---NA--- | | |
| TRINITY_DN30583_c0_g1_i1_len_306_283595.p1_type_internal_len_103 | ---NA--- | | |
| TRINITY_DN19726_c0_g1_i1_len_1297_158782.p1_type_complete_len_102 | ---NA--- | | |

| | | | |
|---|---|---|---|
| TRINITY_DN19453_c0_g1_i2_len_1602_267391.p2_type_complete_len_102 | ---NA--- | | |
| TRINITY_DN30090_c0_g1_i1_len_307_283534.p1_type_internal_len_103 | KFM65400.1Protein SZT2 | C:GO:0005777 | |
| TRINITY_DN27032_c0_g1_i1_len_307_283520.p1_type_internal_len_103 | ---NA--- | | |
| TRINITY_DN29538_c0_g1_i1_len_310_140166.p1_type_internal_len_103 | ---NA--- | | |
| TRINITY_DN23946_c0_g1_i1_len_348_309295.p1_type_5prime_partial_len_102 | ---NA--- | | |
| TRINITY_DN4674_c0_g1_i1_len_306_283566.p1_type_internal_len_103 | ---NA--- | | |
| TRINITY_DN31741_c0_g1_i1_len_583_241116.p2_type_5prime_partial_len_102 | ---NA--- | | |
| TRINITY_DN7663_c0_g1_i1_len_374_171334.p1_type_5prime_partial_len_102 | ---NA--- | | |
| TRINITY_DN22547_c0_g1_i1_len_1839_191521.p3_type_3prime_partial_len_103 | ---NA--- | | |
| TRINITY_DN26446_c0_g1_i1_len_306_16493.p1_type_internal_len_103 | ---NA--- | | |
| TRINITY_DN37063_c0_g1_i1_len_307_213759.p1_type_internal_len_103 | XP_017196166.1 60S ribosomal protein L7-like | | |
| TRINITY_DN20122_c0_g1_i1_len_307_140441.p1_type_internal_len_103 | ---NA--- | | |
| TRINITY_DN3476_c0_g1_i1_len_334_310048.p1_type_5prime_partial_len_102 | ---NA--- | | |
| TRINITY_DN30353_c0_g1_i4_len_1631_192403.p1_type_complete_len_102 | ---NA--- | | |
| TRINITY_DN9304_c0_g1_i1_len_317_282913.p1_type_5prime_partial_len_102 | ---NA--- | | |
| TRINITY_DN21784_c0_g1_i1_len_310_283348.p1_type_internal_len_103 | ---NA--- | | |

| | | | |
|---|---|---|---|
| 3065\|_\|c9857_f3p1_2424_isoform_c9857_full_length_coverage_3_non_full_length_coverage_1_isoform_length_2424_12838 | XP_018014829.1 phosphoglucomutase-2-like | P:GO:0071704 | |
| TRINITY_DN22897_c0_g1_i1_len_2556_122402.p1_type_5prime_partial_len_808 | XP_018027105.1 aconitate hydratase, mitochondrial-like | F:GO:0003994; C:GO:0005739; P:GO:0006099; F:GO:0046872; F:GO:0051539 | EC:4.2.1.3 |
| TRINITY_DN22602_c0_g1_i2_len_2852_188771.p1_type_5prime_partial_len_808 | XP_018898792.1 protein 4.1 homolog | | |
| 3069\|_\|c10281_f2p0_2423_isoform_c10281_full_length_coverage_2_non_full_length_coverage_0_isoform_length_2423_17343 | XP_018025591.1 uncharacterized aarF domain-containing protein kinase 5-like isoform X1 | | |
| 3070\|_\|c47914_f10p11_2423_isoform_c47914_full_length_coverage_10_non_full_length_coverage_11_isoform_length_2423_24344 | ---NA--- | | |
| 3071\|_\|c18146_f2p3_2423_isoform_c18146_full_length_coverage_2_non_full_length_coverage_3_isoform_length_2423_22720 | ---NA--- | | |
| 3072\|_\|c13026_f2p10_2422_isoform_c13026_full_length_coverage_2_non_full_length_coverage_10_isoform_length_2422_22185 | XP_013780130.1cryptochrome-2-like isoform X2 | | |
| TRINITY_DN31271_c0_g1_i2_len_5245_187072.p1_type_complete_len_807 | XP_022320414.1uncharacterized protein LOC111122777 | | |
| TRINITY_DN22228_c0_g1_i2_len_2484_229823.p1_type_5prime_partial_len_807 | XP_021934893.1E3 ubiquitin-protein ligase Nedd-4 isoform X3 | F:GO:0004842; P:GO:0044267 | |
| 3075\|_\|c9154_f3p15_2421_isoform_c9154_full_length_coverage_3_non_full_length_coverage_15_isoform_length_2421_21840 | PNF38108.1Cytoplasmic dynein 1 light intermediate chain 2 | F:GO:0003777; C:GO:0005868; P:GO:0007018 | |
| TRINITY_DN8744_c0_g1_i1_len_310_283341.p2_type_internal_len_103 | ---NA--- | | |
| TRINITY_DN20268_c0_g1_i2_len_716_199758.p1_type_5prime_partial_len_102 | ---NA--- | | |

| | | | |
|---|---|---|---|
| TRINITY_DN32423_c0_g1_i1_len_306_140501.p1_type_internal_len_103 | ---NA--- | | |
| TRINITY_DN26576_c0_g1_i1_len_306_140509.p1_type_internal_len_103 | ---NA--- | | |
| TRINITY_DN19958_c0_g1_i2_len_753_199229.p3_type_complete_len_102 | ---NA--- | | |
| TRINITY_DN13536_c0_g1_i3_len_506_242882.p1_type_3prime_partial_len_103 | ---NA--- | | |
| TRINITY_DN14840_c0_g1_i1_len_580_202198.p1_type_5prime_partial_len_102 | ELT91856.1hypothetical protein CAPTEDRAFT_195438 | | |
| TRINITY_DN32466_c0_g1_i1_len_308_140363.p1_type_internal_len_103 | ---NA--- | | |
| TRINITY_DN5640_c0_g1_i1_len_309_283410.p1_type_internal_len_103 | XP_018023034.1 protein O-linked-mannose beta-1,2-N-acetylglucosaminyltransferase 1-like | | |
| TRINITY_DN29580_c0_g1_i1_len_2002_190893.p3_type_5prime_partial_len_102 | ---NA--- | | |
| TRINITY_DN27405_c0_g1_i1_len_308_140341.p1_type_internal_len_103 | ---NA--- | | |
| TRINITY_DN18957_c0_g1_i1_len_308_140344.p1_type_internal_len_103 | ---NA--- | | |
| TRINITY_DN29604_c0_g1_i1_len_307_140414.p1_type_internal_len_103 | APF30031.1ionotropic glutamate receptor NMDA 1A | F:GO:0004970; C:GO:0016021; C:GO:0030054; P:GO:0034220; P:GO:0035235; C:GO:0045211 | |
| TRINITY_DN28891_c0_g1_i1_len_309_283391.p2_type_internal_len_103 | ---NA--- | | |
| TRINITY_DN14994_c0_g1_i1_len_307_140426.p1_type_internal_len_103 | ---NA--- | | |
| TRINITY_DN33798_c0_g1_i1_len_307_140429.p1_type_internal_len_103 | ---NA--- | | |
| TRINITY_DN21894_c0_g2_i1_len_398_307118.p1_type_5prime_partial_len_102 | ---NA--- | | |

| | | | |
|---|---|---|---|
| TRINITY_DN16722_c0_g1_i1_len_483_9235.p2_type_5prime_partial_len_102 | XP_026292790.1low-density lipoprotein receptor-related protein 2 | | |
| TRINITY_DN31647_c0_g1_i1_len_307_140383.p1_type_internal_len_103 | ---NA--- | | |
| TRINITY_DN30948_c0_g1_i1_len_307_140397.p1_type_5prime_partial_len_102 | ---NA--- | | |
| TRINITY_DN21991_c0_g1_i1_len_350_106610.p1_type_5prime_partial_len_102 | ---NA--- | | |
| TRINITY_DN10006_c0_g1_i1_len_309_45815.p1_type_internal_len_103 | ---NA--- | | |
| TRINITY_DN30768_c0_g1_i1_len_390_246794.p1_type_3prime_partial_len_103 | ---NA--- | | |
| TRINITY_DN11831_c0_g1_i1_len_607_164876.p1_type_5prime_partial_len_102 | ---NA--- | | |
| TRINITY_DN15329_c1_g1_i1_len_346_106788.p1_type_5prime_partial_len_102 | ---NA--- | | |
| TRINITY_DN18661_c0_g2_i1_len_337_75480.p1_type_5prime_partial_len_102 | ---NA--- | | |
| TRINITY_DN2702_c0_g1_i1_len_1213_234616.p1_type_complete_len_102 | ---NA--- | | |
| TRINITY_DN30708_c0_g2_i1_len_2615_189202.p2_type_complete_len_102 | ---NA--- | | |
| TRINITY_DN18181_c0_g1_i1_len_592_67234.p2_type_complete_len_102 | ---NA--- | | |
| TRINITY_DN25685_c0_g1_i1_len_3302_228654.p3_type_complete_len_102 | ---NA--- | | |
| TRINITY_DN25427_c0_g1_i1_len_308_45864.p1_type_internal_len_103 | XP_018015438.1  multidrug resistance-associated protein 1-like | | |
| TRINITY_DN26874_c0_g1_i1_len_658_164095.p1_type_5prime_partial_len_102 | ---NA--- | | |
| TRINITY_DN13312_c0_g1_i1_len_649_164225.p1_type_3prime_partial_len_103 | ---NA--- | | |

| | | | |
|---|---|---|---|
| TRINITY_DN3093_c0_g1_i1_len_308_45863.p1_type_internal_len_103 | ---NA--- | | |
| TRINITY_DN13512_c0_g1_i1_len_740_199439.p1_type_complete_len_102 | ---NA--- | | |
| TRINITY_DN31154_c0_g1_i3_len_3340_188086.p2_type_3prime_partial_len_103 | ---NA--- | | |
| TRINITY_DN22239_c0_g1_i3_len_413_134905.p1_type_5prime_partial_len_102 | AUB30144.1slow-type skeletal muscle actin 9 | F:GO:0005524 | |
| TRINITY_DN9489_c0_g1_i1_len_860_237337.p1_type_complete_len_102 | ---NA--- | | |
| TRINITY_DN23759_c0_g1_i1_len_308_16363.p1_type_internal_len_103 | ---NA--- | | |
| TRINITY_DN16834_c0_g1_i2_len_1281_234169.p1_type_complete_len_102 | ---NA--- | | |
| TRINITY_DN19651_c0_g4_i1_len_309_45841.p1_type_3prime_partial_len_103 | ---NA--- | | |
| TRINITY_DN17367_c0_g1_i1_len_477_69742.p2_type_complete_len_102 | ---NA--- | | |
| TRINITY_DN30338_c0_g1_i1_len_308_16354.p1_type_internal_len_103 | ---NA--- | | |
| TRINITY_DN2080_c0_g1_i1_len_638_164408.p2_type_5prime_partial_len_102 | ---NA--- | | |
| TRINITY_DN22342_c0_g1_i1_len_1272_234223.p3_type_5prime_partial_len_102 | ---NA--- | | |
| TRINITY_DN17083_c2_g1_i1_len_308_16360.p1_type_internal_len_103 | XP_017918477.1 zinc finger protein 665 | F:GO:0003676 | |
| TRINITY_DN24336_c0_g1_i1_len_307_16413.p1_type_internal_len_103 | XP_018006365.1 ras GTPase-activating protein 3-like | | |
| TRINITY_DN21554_c0_g1_i1_len_1477_1726.p4_type_complete_len_102 | ---NA--- | | |
| TRINITY_DN13725_c0_g1_i2_len_360_106101.p1_type_5prime_partial_len_102 | ---NA--- | | |

| | | | |
|---|---|---|---|
| TRINITY_DN7282_c0_g1_i4_len_1373_124724.p1_type_5prime_partial_len_102 | ---NA--- | | |
| TRINITY_DN22523_c0_g1_i1_len_374_280018.p1_type_3prime_partial_len_103 | ---NA--- | | |
| TRINITY_DN15799_c0_g1_i1_len_386_246974.p1_type_5prime_partial_len_102 | ---NA--- | | |
| TRINITY_DN3682_c0_g1_i1_len_308_16334.p1_type_internal_len_103 | ---NA--- | | |
| TRINITY_DN32731_c0_g1_i1_len_306_175137.p1_type_internal_len_103 | XP_022191607.1GTPase-activating protein and VPS9 domain-containing protein 1 | | |
| TRINITY_DN28835_c0_g1_i1_len_419_245678.p1_type_5prime_partial_len_102 | ---NA--- | | |
| TRINITY_DN30914_c0_g1_i1_len_306_175118.p1_type_internal_len_103 | ---NA--- | | |
| TRINITY_DN12033_c0_g1_i1_len_344_211108.p2_type_5prime_partial_len_102 | ---NA--- | | |
| TRINITY_DN14436_c0_g1_i1_len_637_66528.p1_type_complete_len_102 | ---NA--- | | |
| TRINITY_DN12668_c0_g1_i1_len_518_38328.p1_type_5prime_partial_len_102 | ---NA--- | | |
| TRINITY_DN14516_c0_g1_i1_len_834_162075.p1_type_5prime_partial_len_102 | ---NA--- | | |
| TRINITY_DN33293_c0_g1_i1_len_307_175002.p1_type_internal_len_103 | ---NA--- | | |
| TRINITY_DN7216_c0_g1_i1_len_306_16439.p1_type_internal_len_103 | XP_017302581.1anaphase-promoting complex subunit 11 | F:GO:0004842; C:GO:0005680; P:GO:0016567 | |
| TRINITY_DN30768_c0_g1_i1_len_307_175005.p1_type_internal_len_103 | XP_018021508.1 sialic acid synthase-like | | |
| TRINITY_DN27242_c0_g1_i1_len_307_175010.p1_type_internal_len_103 | ---NA--- | | |
| TRINITY_DN16276_c0_g1_i1_len_578_130728.p1_type_complete_len_102 | ---NA--- | | |

| | | | |
|---|---|---|---|
| TRINITY_DN25560_c0_g1_i1_len_306_175071.p1_type_internal_len_103 | XP_023704757.11-phosphatidylinositol 4,5-bisphosphate phosphodiesterase epsilon-1-like isoform X2 | P:GO:0006629; P:GO:0007165; F:GO:0008081 | |
| TRINITY_DN27261_c0_g1_i1_len_306_175081.p1_type_internal_len_103 | XP_018028241.1 calpain-5-like | F:GO:0008234 | |
| TRINITY_DN26151_c0_g1_i1_len_1403_193632.p1_type_complete_len_102 | ---NA--- | | |
| TRINITY_DN24213_c0_g1_i6_len_609_201575.p1_type_5prime_partial_len_102 | ---NA--- | | |
| TRINITY_DN24906_c0_g1_i1_len_741_199404.p2_type_complete_len_102 | ALO17555.1Neuroparsin | | |
| TRINITY_DN28027_c0_g1_i2_len_2530_189386.p3_type_3prime_partial_len_103 | ---NA--- | | |
| TRINITY_DN4569_c0_g1_i1_len_340_107166.p1_type_5prime_partial_len_102 | ---NA--- | | |
| TRINITY_DN22752_c0_g1_i2_len_1212_234623.p3_type_3prime_partial_len_103 | ---NA--- | | |
| TRINITY_DN5615_c0_g1_i1_len_522_100820.p1_type_5prime_partial_len_102 | ---NA--- | | |
| TRINITY_DN17085_c0_g1_i1_len_459_205582.p1_type_complete_len_102 | ---NA--- | | |
| TRINITY_DN15921_c0_g1_i1_len_818_162231.p1_type_5prime_partial_len_102 | ---NA--- | | |
| TRINITY_DN9537_c0_g1_i1_len_308_16340.p1_type_internal_len_103 | ---NA--- | | |
| TRINITY_DN37309_c0_g1_i1_len_345_211052.p1_type_5prime_partial_len_102 | ---NA--- | | |
| TRINITY_DN8272_c0_g1_i1_len_306_77637.p1_type_internal_len_103 | XP_021957508.1osmotic avoidance abnormal protein 3-like | | |
| TRINITY_DN29343_c0_g1_i1_len_306_77630.p1_type_internal_len_103 | XP_025106706.1nicotinamide phosphoribosyltransferase-like | F:GO:0016757 | |
| TRINITY_DN10585_c0_g1_i1_len_408_41198.p1_type_5prime_partial_len_102 | ---NA--- | | |

| | | | |
|---|---|---|---|
| TRINITY_DN7861_c0_g1_i1_len_306_46046.p1_type_internal_len_103 | ---NA--- | | |
| TRINITY_DN1960_c0_g1_i1_len_306_311767.p1_type_internal_len_103 | ---NA--- | | |
| TRINITY_DN8461_c0_g1_i1_len_306_46003.p1_type_internal_len_103 | ---NA--- | | |
| TRINITY_DN11679_c0_g1_i1_len_345_211069.p2_type_5prime_partial_len_102 | ALP06206.1crustacean cardioactive peptide | | |
| TRINITY_DN13043_c0_g1_i1_len_306_311716.p1_type_internal_len_103 | ---NA--- | | |
| TRINITY_DN8680_c0_g1_i1_len_306_46012.p1_type_5prime_partial_len_102 | ---NA--- | | |
| TRINITY_DN28576_c0_g1_i2_len_315_213142.p1_type_5prime_partial_len_102 | ---NA--- | | |
| TRINITY_DN28879_c0_g1_i1_len_307_311694.p1_type_internal_len_103 | XP_018016474.1 beta-sarcoglycan-like | C:GO:0016020 | |
| TRINITY_DN11684_c0_g1_i2_len_307_311698.p1_type_internal_len_103 | ---NA--- | | |
| TRINITY_DN15373_c0_g1_i1_len_796_237997.p1_type_5prime_partial_len_102 | XP_018010986.1PREDICTED: uncharacterized protein LOC108668307 | | |
| TRINITY_DN25806_c0_g1_i1_len_307_77549.p1_type_internal_len_103 | ---NA--- | | |
| TRINITY_DN23068_c0_g2_i2_len_883_197672.p2_type_complete_len_102 | ---NA--- | | |
| TRINITY_DN27988_c0_g1_i1_len_307_77486.p1_type_internal_len_103 | XP_018018463.1 protein EFR3 homolog B-like | | |
| TRINITY_DN28012_c0_g1_i1_len_307_77509.p1_type_internal_len_103 | XP_023342178.1connector enhancer of kinase suppressor of ras 3-like | | |
| TRINITY_DN30199_c0_g1_i1_len_308_77447.p1_type_internal_len_103 | ---NA--- | | |
| TRINITY_DN4169_c0_g1_i1_len_308_77460.p1_type_internal_len_103 | ---NA--- | | |

| | | | |
|---|---|---|---|
| TRINITY_DN27502_c0_g1_i1_len_307_77474.p1_type_internal_len_103 | XP_008543496.1 protein zer-1 homolog | | |
| TRINITY_DN25711_c0_g1_i1_len_306_77604.p1_type_internal_len_103 | ---NA--- | | |
| TRINITY_DN10439_c0_g1_i1_len_310_77300.p1_type_internal_len_103 | ---NA--- | | |
| 3076\|_\|c33888_f33p11_2421_isoform_c33888_full_length_coverage_33_non_full_length_coverage_11_isoform_length_2421_18624 | XP_018028238.1 peroxidase-like | F:GO:0004601; P:GO:0006979; F:GO:0020037; P:GO:0055114; P:GO:0098869 | |
| 3077\|_\|c2702_f7p3_2420_isoform_c2702_full_length_coverage_7_non_full_length_coverage_3_isoform_length_2420_21162 | ---NA--- | | |
| 3078\|_\|c7661_f2p3_2420_isoform_c7661_full_length_coverage_2_non_full_length_coverage_3_isoform_length_2420_12651 | XP_968231.1 RING finger protein 10 | F:GO:0046872 | |
| 3079\|_\|c1479_f9p9_2420_isoform_c1479_full_length_coverage_9_non_full_length_coverage_9_isoform_length_2420_15876 | XP_018022175.1 long-chain specific acyl-CoA dehydrogenase, mitochondrial-like isoform X1 | | |
| 3080\|_\|c26449_f2p2_2420_isoform_c26449_full_length_coverage_2_non_full_length_coverage_2_isoform_length_2420_18394 | ---NA--- | | |
| 3081\|_\|c2818_f3p7_2420_isoform_c2818_full_length_coverage_3_non_full_length_coverage_7_isoform_length_2420_21260 | ---NA--- | | |
| 3082\|_\|c15503_f8p3_2419_isoform_c15503_full_length_coverage_8_non_full_length_coverage_3_isoform_length_2419_17670 | XP_018022508.1 caseinolytic peptidase B protein homolog | | |
| 3084\|_\|c2785_f2p0_2419_isoform_c2785_full_length_coverage_2_non_full_length_coverage_0_isoform_length_2419_21233 | ABQ41248.1male reproductive-related microfibril-associated protein | | |

| | | | |
|---|---|---|---|
| 3086\|_\|c43228_f12p0_2418_isoform_c43228_full_length_coverage_12_non_full_length_coverage_0_isoform_length_2418_15075 | ACH72070.1cyclin B | C:GO:0005634 | |
| 3087\|_\|c1968_f5p3_2418_isoform_c1968_full_length_coverage_5_non_full_length_coverage_3_isoform_length_2418_16090 | XP_013185936.1 vesicle-associated membrane protein 7 | | |
| TRINITY_DN28499_c0_g1_i1_len_310_77228.p1_type_internal_len_103 | ---NA--- | | |
| TRINITY_DN2431_c0_g1_i1_len_310_77230.p1_type_internal_len_103 | ---NA--- | | |
| TRINITY_DN9276_c0_g1_i1_len_306_77570.p1_type_internal_len_103 | ---NA--- | | |
| TRINITY_DN26850_c0_g1_i1_len_309_77377.p1_type_internal_len_103 | AXC43907.1histone acetyltransferase KAT2A | F:GO:0004402; C:GO:0005634; P:GO:0006355; P:GO:0016573 | EC:2.3.1.5; EC:2.3.1.48 |
| TRINITY_DN18007_c0_g1_i1_len_1397_233516.p2_type_5prime_partial_len_102 | ---NA--- | | |
| TRINITY_DN29408_c0_g1_i1_len_309_77382.p1_type_internal_len_103 | ---NA--- | | |
| TRINITY_DN32047_c0_g1_i1_len_309_77326.p1_type_internal_len_103 | XP_015458757.2 peroxisomal sarcosine oxidase | | |
| TRINITY_DN22893_c0_g1_i1_len_371_12912.p1_type_5prime_partial_len_102 | XP_018026648.1 plasminogen receptor (KT)-like isoform X1 | | |
| TRINITY_DN12094_c0_g1_i1_len_781_238178.p2_type_complete_len_102 | ---NA--- | | |
| TRINITY_DN9719_c0_g1_i1_len_308_45908.p1_type_internal_len_103 | ---NA--- | | |
| TRINITY_DN29441_c0_g1_i1_len_486_69499.p1_type_5prime_partial_len_102 | ---NA--- | | |
| TRINITY_DN18740_c0_g1_i1_len_546_68082.p1_type_5prime_partial_len_102 | ---NA--- | | |
| TRINITY_DN26702_c0_g1_i1_len_344_249164.p1_type_5prime_partial_len_102 | XP_014246401.1zinc finger protein 395 isoform X2 | | |

| | | | |
|---|---|---|---|
| TRINITY_DN12580_c0_g1_i1_len_308_45902.p1_type_internal_len_103 | XP_023716849.1MPN domain-containing protein-like isoform X2 | | |
| TRINITY_DN9853_c0_g1_i1_len_309_311562.p1_type_internal_len_103 | ---NA--- | | |
| TRINITY_DN28720_c0_g1_i1_len_306_45997.p1_type_internal_len_103 | ---NA--- | | |
| TRINITY_DN1999_c0_g1_i1_len_355_280831.p1_type_5prime_partial_len_102 | ---NA--- | | |
| TRINITY_DN19315_c0_g2_i2_len_350_248772.p1_type_5prime_partial_len_102 | ---NA--- | | |
| TRINITY_DN16821_c0_g1_i1_len_371_247697.p1_type_5prime_partial_len_102 | ---NA--- | | |
| TRINITY_DN1772_c0_g1_i2_len_307_45975.p1_type_internal_len_103 | ---NA--- | | |
| TRINITY_DN5704_c0_g1_i1_len_309_311543.p1_type_internal_len_103 | XP_023701956.1galactoside 3(4)-L-fucosyltransferase-like | C:GO:0016020; F:GO:0016740 | |
| TRINITY_DN13072_c0_g1_i1_len_790_238068.p1_type_complete_len_102 | ---NA--- | | |
| TRINITY_DN23837_c0_g1_i4_len_1416_124583.p3_type_3prime_partial_len_103 | ---NA--- | | |
| TRINITY_DN26043_c0_g1_i1_len_307_45970.p1_type_internal_len_103 | ---NA--- | | |
| TRINITY_DN18142_c0_g1_i2_len_1828_266714.p2_type_5prime_partial_len_102 | ---NA--- | | |
| TRINITY_DN11204_c0_g1_i1_len_451_168389.p2_type_5prime_partial_len_102 | ---NA--- | | |
| TRINITY_DN14219_c0_g1_i1_len_2179_190313.p2_type_complete_len_102 | ---NA--- | | |
| TRINITY_DN10400_c0_g1_i1_len_306_213836.p1_type_internal_len_103 | ---NA--- | | |
| TRINITY_DN3030_c0_g1_i1_len_308_109219.p1_type_internal_len_103 | ---NA--- | | |

| | | | |
|---|---|---|---|
| TRINITY_DN11387_c0_g1_i1_len_308_109220.p1_type_internal_len_103 | ---NA--- | | |
| TRINITY_DN7734_c0_g1_i1_len_308_109221.p1_type_internal_len_103 | ---NA--- | | |
| TRINITY_DN34073_c0_g1_i1_len_417_207261.p1_type_complete_len_102 | ---NA--- | | |
| TRINITY_DN4441_c0_g1_i1_len_417_207260.p1_type_5prime_partial_len_102 | APG16029.1broad-complex | F:GO:0003676 | |
| TRINITY_DN10935_c0_g2_i1_len_308_109215.p1_type_internal_len_103 | ---NA--- | | |
| TRINITY_DN32785_c0_g1_i1_len_309_109169.p1_type_internal_len_103 | ---NA--- | | |
| TRINITY_DN1173_c0_g1_i1_len_306_213811.p1_type_internal_len_103 | ---NA--- | | |
| TRINITY_DN14379_c0_g1_i1_len_309_109185.p1_type_internal_len_103 | BAU19444.1serine racemase | P:GO:0006520; F:GO:0030170 | |
| TRINITY_DN29907_c0_g1_i1_len_308_16317.p1_type_internal_len_103 | ---NA--- | | |
| TRINITY_DN35647_c0_g1_i1_len_695_239283.p1_type_complete_len_102 | ---NA--- | | |
| TRINITY_DN26780_c0_g1_i1_len_309_109176.p1_type_internal_len_103 | ---NA--- | | |
| TRINITY_DN1068_c0_g1_i1_len_607_201620.p1_type_complete_len_102 | ---NA--- | | |
| TRINITY_DN10660_c0_g1_i1_len_349_137751.p1_type_5prime_partial_len_102 | ---NA--- | | |
| TRINITY_DN19649_c0_g1_i5_len_641_36410.p1_type_5prime_partial_len_102 | ---NA--- | | |
| TRINITY_DN22891_c0_g1_i1_len_771_198970.p1_type_5prime_partial_len_102 | ---NA--- | | |
| TRINITY_DN18450_c0_g1_i1_len_447_168537.p1_type_5prime_partial_len_102 | ---NA--- | | |

| | | | |
|---|---|---|---|
| TRINITY_DN21711_c0_g1_i1_len_331_282110.p1_type_5prime_partial_len_102 | ---NA--- | | |
| TRINITY_DN12463_c0_g1_i2_len_570_99944.p1_type_5prime_partial_len_102 | ---NA--- | | |
| TRINITY_DN9786_c0_g1_i1_len_307_109298.p1_type_internal_len_103 | ---NA--- | | |
| TRINITY_DN27402_c0_g1_i1_len_308_16325.p1_type_internal_len_103 | XP_018026620.1 titin-like isoform X3 | | |
| TRINITY_DN27951_c0_g1_i1_len_307_109299.p1_type_internal_len_103 | KZS20093.1putative Beta-hexosaminidase fdl | F:GO:0004553; P:GO:0008152 | |
| TRINITY_DN1558_c0_g1_i1_len_317_45308.p1_type_5prime_partial_len_102 | ---NA--- | | |
| TRINITY_DN28158_c0_g1_i1_len_308_109257.p2_type_internal_len_103 | ---NA--- | | |
| TRINITY_DN15247_c0_g1_i1_len_306_213845.p1_type_internal_len_103 | ---NA--- | | |
| TRINITY_DN11624_c0_g1_i1_len_972_3765.p2_type_5prime_partial_len_102 | ---NA--- | | |
| TRINITY_DN30492_c0_g1_i1_len_325_173882.p1_type_5prime_partial_len_102 | ---NA--- | | |
| TRINITY_DN2048_c0_g1_i2_len_359_210134.p1_type_5prime_partial_len_102 | ---NA--- | | |
| TRINITY_DN13758_c0_g1_i2_len_612_99247.p1_type_5prime_partial_len_102 | ---NA--- | | |
| TRINITY_DN5251_c0_g1_i2_len_306_213827.p1_type_internal_len_103 | ---NA--- | | |
| TRINITY_DN23411_c0_g1_i1_len_804_271721.p1_type_5prime_partial_len_102 | XP_018024766.1 sorting nexin-18-like | | |
| TRINITY_DN13636_c0_g1_i2_len_952_161067.p4_type_5prime_partial_len_102 | ---NA--- | | |
| TRINITY_DN13218_c0_g1_i1_len_431_206667.p1_type_5prime_partial_len_102 | ---NA--- | | |

| | | | |
|---|---|---|---|
| TRINITY_DN29866_c0_g1_i1_len_2091_190580.p1_type_complete_len_102 | ---NA--- | | |
| TRINITY_DN21753_c0_g1_i1_len_1782_156747.p2_type_5prime_partial_len_102 | ---NA--- | | |
| TRINITY_DN37167_c0_g1_i1_len_442_244832.p1_type_5prime_partial_len_102 | ---NA--- | | |
| TRINITY_DN14689_c0_g1_i3_len_694_65740.p2_type_5prime_partial_len_102 | ---NA--- | | |
| TRINITY_DN37601_c0_g1_i1_len_469_243944.p1_type_3prime_partial_len_103 | ---NA--- | | |
| TRINITY_DN3274_c0_g1_i2_len_530_275371.p1_type_5prime_partial_len_102 | ---NA--- | | |
| TRINITY_DN20807_c0_g1_i1_len_451_244519.p2_type_5prime_partial_len_102 | ---NA--- | | |
| TRINITY_DN25251_c0_g1_i1_len_310_109084.p1_type_internal_len_103 | ---NA--- | | |
| TRINITY_DN9306_c0_g1_i1_len_642_200956.p1_type_5prime_partial_len_102 | ---NA--- | | |
| TRINITY_DN15052_c0_g1_i3_len_313_213270.p1_type_5prime_partial_len_102 | ---NA--- | | |
| TRINITY_DN26551_c0_g1_i1_len_1424_193515.p2_type_5prime_partial_len_102 | ---NA--- | | |
| TRINITY_DN23130_c0_g1_i4_len_720_199710.p1_type_complete_len_102 | ---NA--- | | |
| TRINITY_DN28920_c0_g1_i1_len_327_173709.p1_type_5prime_partial_len_102 | ---NA--- | | |
| TRINITY_DN31604_c0_g1_i1_len_307_109327.p1_type_internal_len_103 | ---NA--- | | |
| TRINITY_DN23378_c0_g1_i1_len_306_109376.p1_type_internal_len_103 | XP_018006407.1 histone-lysine N-methyltransferase 2D-like | | |
| TRINITY_DN7383_c0_g1_i2_len_440_168762.p1_type_5prime_partial_len_102 | XP_013383226.1sushi, von Willebrand factor type A, EGF and pentraxin domain-containing protein 1 | F:GO:0005488 | |

| | | | |
|---|---|---|---|
| TRINITY_DN15835_c0_g1_i1_len_559_100119.p1_type_5prime_partial_len_102 | ---NA--- | | |
| TRINITY_DN25896_c0_g1_i1_len_306_16450.p1_type_internal_len_103 | ---NA--- | | |
| TRINITY_DN35122_c0_g1_i1_len_306_213808.p1_type_internal_len_103 | AET34914.1prophenoloxide activating enzyme III | F:GO:0016787 | |
| TRINITY_DN27461_c0_g1_i1_len_308_174940.p2_type_internal_len_103 | XP_014271960.1lysophospholipase D GDPD1-like isoform X3 | C:GO:0016020 | |
| TRINITY_DN11676_c0_g1_i1_len_334_211780.p1_type_5prime_partial_len_102 | ---NA--- | | |
| TRINITY_DN17281_c0_g1_i3_len_1613_157369.p4_type_5prime_partial_len_102 | ---NA--- | | |
| TRINITY_DN3247_c0_g1_i1_len_306_213865.p1_type_internal_len_103 | ---NA--- | | |
| TRINITY_DN27686_c0_g1_i1_len_308_174981.p2_type_internal_len_103 | ---NA--- | | |
| TRINITY_DN12334_c0_g1_i1_len_308_174977.p1_type_internal_len_103 | ---NA--- | | |
| TRINITY_DN10850_c0_g1_i1_len_309_174901.p1_type_internal_len_103 | ---NA--- | | |
| TRINITY_DN14993_c0_g1_i2_len_485_204705.p1_type_3prime_partial_len_103 | ---NA--- | | |
| TRINITY_DN2491_c0_g1_i1_len_309_174889.p1_type_internal_len_103 | ---NA--- | | |
| TRINITY_DN14911_c0_g1_i1_len_351_43502.p1_type_5prime_partial_len_102 | ---NA--- | | |
| TRINITY_DN3276_c0_g1_i1_len_308_174935.p1_type_internal_len_103 | XP_023716129.1Golgi reassembly-stacking protein 2 | | |
| TRINITY_DN10667_c0_g1_i1_len_307_109306.p1_type_internal_len_103 | ---NA--- | | |
| TRINITY_DN22855_c0_g1_i1_len_1835_59705.p5_type_5prime_partial_len_102 | ---NA--- | | |

<table>
<tr><td></td><td></td><td></td><td></td></tr>
<tr><td>TRINITY_DN31288_c0_g1_i1_len_332_282046.p1_type_5prime_partial_len_102</td><td>---NA---</td><td></td><td></td></tr>
<tr><td>TRINITY_DN20176_c0_g1_i2_len_1943_231289.p4_type_complete_len_101</td><td>---NA---</td><td></td><td></td></tr>
<tr><td>TRINITY_DN16168_c0_g1_i1_len_548_303223.p2_type_complete_len_101</td><td>---NA---</td><td></td><td></td></tr>
<tr><td>TRINITY_DN7403_c0_g1_i2_len_375_171270.p1_type_5prime_partial_len_101</td><td>BBC20610.1hypothetical protein</td><td></td><td></td></tr>
<tr><td>TRINITY_DN20895_c0_g1_i2_len_1030_235879.p1_type_complete_len_101</td><td>---NA---</td><td></td><td></td></tr>
<tr><td>TRINITY_DN2300_c0_g1_i1_len_303_16681.p1_type_internal_len_102</td><td>---NA---</td><td></td><td></td></tr>
<tr><td>TRINITY_DN15443_c0_g1_i1_len_370_247776.p1_type_5prime_partial_len_101</td><td>---NA---</td><td></td><td></td></tr>
<tr><td>TRINITY_DN16713_c0_g1_i1_len_2702_229427.p1_type_5prime_partial_len_806</td><td>OPL33833.1hypothetical protein AM593_07708, partial</td><td></td><td></td></tr>
<tr><td>3089|_|c47265_f6p13_2418_isoform_c47265_full_length_coverage_6_non_full_length_coverage_13_isoform_length_2418_19515</td><td>XP_018007946.1 neural Wiskott-Aldrich syndrome protein-like</td><td></td><td></td></tr>
<tr><td>3090|_|c2543_f7p1_2417_isoform_c2543_full_length_coverage_7_non_full_length_coverage_1_isoform_length_2417_21028</td><td>---NA---</td><td></td><td></td></tr>
<tr><td>3092|_|c17963_f2p0_2417_isoform_c17963_full_length_coverage_2_non_full_length_coverage_0_isoform_length_2417_13474</td><td>PNF16894.1RING finger protein unkempt-like protein</td><td>F:GO:0046872</td><td></td></tr>
<tr><td>3093|_|c18009_f2p3_2416_isoform_c18009_full_length_coverage_2_non_full_length_coverage_3_isoform_length_2416_17892</td><td>XP_023222711.1bromodomain-containing protein 3-like</td><td></td><td></td></tr>
<tr><td>3095|_|c2749_f6p3_2416_isoform_c2749_full_length_coverage_6_non_full_length_coverage_3_isoform_length_2416_21204</td><td>---NA---</td><td></td><td></td></tr>
</table>

| | | | |
|---|---|---|---|
| 3096\|_\|c2321_f2p1_2416_isoform_c2321_full_length_coverage_2_non_full_length_coverage_1_isoform_length_2416_16322 | ---NA--- | | |
| TRINITY_DN30955_c0_g1_i1_len_2856_188763.p1_type_5prime_partial_len_805 | KXJ06669.1Retrovirus-related Pol polyprotein from transposon 17.6 | F:GO:0003676; P:GO:0015074 | |
| TRINITY_DN22346_c1_g1_i4_len_2564_154895.p1_type_5prime_partial_len_805 | XP_018021193.1 SWI/SNF-related matrix-associated actin-dependent regulator of chromatin subfamily E member 1-like isoform X3 | | |
| 3099\|_\|c34968_f3p14_2414_isoform_c34968_full_length_coverage_3_non_full_length_coverage_14_isoform_length_2414_14416 | ---NA--- | | |
| TRINITY_DN17929_c0_g1_i2_len_1008_236052.p1_type_complete_len_101 | ---NA--- | | |
| TRINITY_DN21943_c0_g1_i1_len_1117_235266.p1_type_complete_len_101 | XP_022245499.1receptor-type tyrosine-protein phosphatase R-like | | |
| TRINITY_DN26788_c0_g1_i1_len_305_16552.p1_type_internal_len_102 | ---NA--- | | |
| TRINITY_DN31841_c0_g1_i1_len_1034_160403.p2_type_5prime_partial_len_101 | ---NA--- | | |
| TRINITY_DN22095_c0_g1_i1_len_1097_62520.p2_type_complete_len_101 | XP_018012068.1 charged multivesicular body protein 1b-2-like | P:GO:0007034 | |
| TRINITY_DN6593_c0_g1_i1_len_788_238086.p1_type_complete_len_101 | ---NA--- | | |
| TRINITY_DN16241_c0_g1_i1_len_468_243983.p1_type_3prime_partial_len_102 | XP_018015763.1PREDICTED: uncharacterized protein LOC108672575 isoform X3 | | |
| TRINITY_DN22080_c0_g1_i1_len_304_311912.p1_type_internal_len_102 | ---NA--- | | |
| TRINITY_DN23498_c0_g1_i1_len_304_46174.p1_type_internal_len_102 | ---NA--- | | |
| TRINITY_DN19418_c0_g1_i1_len_368_247840.p1_type_5prime_partial_len_101 | ---NA--- | | |

| | | | |
|---|---|---|---|
| TRINITY_DN24535_c0_g1_i3_len_991_63173.p1_type_complete_len_101 | ---NA--- | | |
| TRINITY_DN21015_c0_g1_i1_len_1782_59837.p2_type_complete_len_101 | ---NA--- | | |
| TRINITY_DN1461_c0_g1_i1_len_346_249034.p1_type_5prime_partial_len_101 | ---NA--- | | |
| TRINITY_DN21186_c0_g1_i2_len_2520_154957.p3_type_complete_len_101 | ---NA--- | | |
| TRINITY_DN32508_c0_g1_i1_len_327_15145.p2_type_5prime_partial_len_101 | ---NA--- | | |
| TRINITY_DN31717_c0_g1_i1_len_516_303834.p1_type_complete_len_101 | ---NA--- | | |
| TRINITY_DN16567_c0_g1_i1_len_1219_159215.p2_type_5prime_partial_len_101 | ---NA--- | | |
| TRINITY_DN25297_c0_g1_i3_len_2380_230048.p1_type_complete_len_101 | ---NA--- | | |
| TRINITY_DN22581_c0_g1_i1_len_303_46204.p2_type_internal_len_102 | ---NA--- | | |
| TRINITY_DN15799_c0_g1_i1_len_1601_157412.p4_type_5prime_partial_len_101 | ---NA--- | | |
| TRINITY_DN20013_c0_g1_i1_len_380_209045.p1_type_5prime_partial_len_101 | ---NA--- | | |
| TRINITY_DN12589_c0_g1_i1_len_793_64658.p1_type_complete_len_101 | ---NA--- | | |
| TRINITY_DN19831_c0_g1_i1_len_1103_235346.p3_type_5prime_partial_len_101 | ---NA--- | | |
| TRINITY_DN7248_c0_g1_i1_len_305_311825.p2_type_internal_len_102 | XP_023335301.1RNA-binding protein Musashi homolog Rbp6-like | | |
| TRINITY_DN18815_c0_g1_i1_len_1733_232013.p3_type_complete_len_101 | XP_017772215.1 protein NDRG3 isoform X2 | | |
| TRINITY_DN28494_c0_g1_i1_len_305_46068.p1_type_internal_len_102 | ---NA--- | | |

| | | | |
|---|---|---|---|
| TRINITY_DN27159_c0_g1_i1_len_305_46099.p1_type_internal_len_102 | ---NA--- | | |
| TRINITY_DN17690_c0_g1_i1_len_1253_159025.p1_type_complete_len_101 | ---NA--- | | |
| TRINITY_DN15597_c0_g1_i1_len_397_307158.p1_type_5prime_partial_len_101 | ---NA--- | | |
| TRINITY_DN14302_c0_g1_i1_len_448_206023.p1_type_5prime_partial_len_101 | ---NA--- | | |
| TRINITY_DN25140_c0_g1_i3_len_1117_235265.p1_type_5prime_partial_len_101 | ---NA--- | | |
| TRINITY_DN6059_c0_g1_i1_len_383_73080.p1_type_complete_len_101 | ---NA--- | | |
| TRINITY_DN22246_c0_g1_i2_len_1626_157333.p2_type_complete_len_101 | ---NA--- | | |
| TRINITY_DN25218_c0_g1_i2_len_3765_187728.p4_type_complete_len_101 | ---NA--- | | |
| TRINITY_DN24419_c0_g1_i2_len_2898_58240.p3_type_complete_len_101 | ---NA--- | | |
| TRINITY_DN31938_c0_g1_i1_len_303_16704.p1_type_internal_len_102 | EDW57721.1uncharacterized protein Dvir_GJ18247, isoform A | C:GO:0016020 | |
| TRINITY_DN9939_c0_g1_i1_len_455_205749.p1_type_5prime_partial_len_101 | ---NA--- | | |
| TRINITY_DN10735_c0_g1_i1_len_455_205745.p1_type_5prime_partial_len_101 | ---NA--- | | |
| TRINITY_DN18241_c0_g1_i1_len_1006_236066.p2_type_complete_len_101 | ---NA--- | | |
| TRINITY_DN29832_c0_g1_i1_len_2052_190717.p3_type_5prime_partial_len_101 | ---NA--- | | |
| TRINITY_DN20410_c0_g1_i1_len_1741_156881.p1_type_complete_len_101 | ---NA--- | | |
| TRINITY_DN23108_c0_g1_i1_len_1402_233486.p1_type_complete_len_101 | ---NA--- | | |

| | | | |
|---|---|---|---|
| TRINITY_DN20975_c0_g1_i1_len_1310_61445.p2_type_complete_len_101 | ---NA--- | | |
| TRINITY_DN4434_c0_g1_i1_len_356_248486.p1_type_5prime_partial_len_101 | ---NA--- | | |
| TRINITY_DN24345_c0_g1_i1_len_306_46007.p1_type_internal_len_102 | ---NA--- | | |
| TRINITY_DN16385_c0_g1_i1_len_305_46119.p2_type_internal_len_102 | ---NA--- | | |
| TRINITY_DN20551_c0_g1_i3_len_946_197029.p1_type_complete_len_101 | ---NA--- | | |
| TRINITY_DN6494_c1_g1_i1_len_304_16653.p2_type_internal_len_102 | ---NA--- | | |
| TRINITY_DN16419_c0_g1_i2_len_958_196916.p2_type_complete_len_101 | ---NA--- | | |
| TRINITY_DN11397_c0_g1_i1_len_304_16637.p1_type_internal_len_102 | ---NA--- | | |
| TRINITY_DN22989_c0_g3_i1_len_356_172171.p1_type_5prime_partial_len_101 | ---NA--- | | |
| TRINITY_DN27819_c0_g1_i1_len_307_311699.p1_type_internal_len_102 | ---NA--- | | |
| TRINITY_DN7626_c0_g1_i1_len_354_43365.p1_type_5prime_partial_len_101 | ---NA--- | | |
| TRINITY_DN17994_c0_g1_i1_len_1158_195246.p2_type_5prime_partial_len_101 | ---NA--- | | |
| TRINITY_DN15207_c0_g1_i2_len_961_196888.p2_type_complete_len_101 | ---NA--- | | |
| TRINITY_DN3111_c0_g1_i1_len_304_311908.p1_type_internal_len_102 | ---NA--- | | |
| TRINITY_DN5157_c0_g1_i1_len_303_16675.p1_type_internal_len_102 | XP_018011656.1 laminin subunit alpha-2-like | F:GO:0005488 | |
| TRINITY_DN15984_c0_g1_i1_len_2922_229103.p4_type_3prime_partial_len_102 | ---NA--- | | |

| | | | |
|---|---|---|---|
| TRINITY_DN17111_c0_g1_i1_len_1293_158805.p2_type_complete_len_101 | ---NA--- | | |
| TRINITY_DN16719_c0_g1_i2_len_303_311980.p1_type_internal_len_102 | ---NA--- | | |
| TRINITY_DN4518_c0_g1_i1_len_304_46144.p1_type_internal_len_102 | ---NA--- | | |
| TRINITY_DN32541_c0_g1_i1_len_372_209396.p1_type_5prime_partial_len_101 | ---NA--- | | |
| TRINITY_DN6610_c0_g1_i1_len_304_311901.p1_type_internal_len_102 | ---NA--- | | |
| TRINITY_DN14530_c1_g1_i1_len_306_45993.p1_type_internal_len_102 | ---NA--- | | |
| TRINITY_DN11816_c0_g1_i1_len_306_45996.p1_type_internal_len_102 | ---NA--- | | |
| TRINITY_DN12636_c0_g1_i1_len_386_208730.p1_type_5prime_partial_len_101 | ---NA--- | | |
| TRINITY_DN34782_c0_g1_i1_len_303_251827.p1_type_internal_len_102 | ---NA--- | | |
| TRINITY_DN2274_c0_g1_i1_len_303_251838.p1_type_internal_len_102 | ---NA--- | | |
| TRINITY_DN29090_c0_g1_i1_len_303_251852.p1_type_internal_len_102 | ASK05861.1chitin-binding protein | C:GO:0031012; F:GO:0042302 | |
| TRINITY_DN36681_c0_g1_i1_len_304_251792.p1_type_internal_len_102 | ---NA--- | | |
| TRINITY_DN32752_c0_g1_i1_len_303_251799.p1_type_internal_len_102 | ---NA--- | | |
| TRINITY_DN37147_c0_g1_i1_len_303_251810.p1_type_internal_len_102 | AUC65357.1serine protease 4 | F:GO:0016787 | |
| TRINITY_DN533_c0_g1_i1_len_312_251213.p1_type_5prime_partial_len_101 | ---NA--- | | |
| TRINITY_DN30889_c0_g1_i1_len_303_175285.p1_type_internal_len_102 | ---NA--- | | |

| | | | |
|---|---|---|---|
| TRINITY_DN15330_c0_g1_i1_len_582_165330.p1_type_5prime_partial_len_101 | ---NA--- | | |
| TRINITY_DN30697_c0_g1_i1_len_303_251864.p1_type_internal_len_102 | ---NA--- | | |
| TRINITY_DN10757_c0_g1_i1_len_304_175261.p1_type_internal_len_102 | ---NA--- | | |
| TRINITY_DN18879_c0_g1_i1_len_1098_159996.p1_type_complete_len_101 | ---NA--- | | |
| TRINITY_DN29877_c0_g1_i1_len_304_175262.p1_type_internal_len_102 | ---NA--- | | |
| TRINITY_DN4116_c0_g1_i2_len_304_175266.p1_type_internal_len_102 | ---NA--- | | |
| TRINITY_DN2751_c0_g1_i1_len_304_251742.p1_type_internal_len_102 | ---NA--- | | |
| TRINITY_DN13390_c0_g1_i2_len_368_42720.p1_type_5prime_partial_len_101 | ---NA--- | | |
| TRINITY_DN32094_c0_g1_i1_len_305_175196.p2_type_internal_len_102 | XP_018008763.1 zinc finger protein castor homolog 1-like isoform X5 | | |
| TRINITY_DN7815_c0_g1_i1_len_304_251746.p1_type_internal_len_102 | ---NA--- | | |
| TRINITY_DN2644_c0_g1_i1_len_305_175199.p1_type_internal_len_102 | ---NA--- | | |
| TRINITY_DN33357_c0_g1_i1_len_304_251755.p1_type_3prime_partial_len_102 | XP_018561161.1putative inorganic phosphate cotransporter | C:GO:0016020 | |
| TRINITY_DN34684_c0_g1_i1_len_305_251707.p2_type_internal_len_102 | ---NA--- | | |
| TRINITY_DN28909_c0_g1_i1_len_305_251713.p1_type_internal_len_102 | XP_012136680.1 cubilin-like isoform X2 | | |
| TRINITY_DN15635_c1_g1_i1_len_441_70862.p1_type_3prime_partial_len_102 | ---NA--- | | |
| TRINITY_DN28197_c0_g1_i1_len_304_251728.p1_type_internal_len_102 | ---NA--- | | |

<table>
<tr><th></th><th></th><th></th><th></th></tr>
<tr><td>TRINITY_DN4779_c0_g1_i1_len_304_251729.p1_type_internal_len_102</td><td>XP_018024062.1 kinase D-interacting substrate of 220 kDa-like isoform X2</td><td></td><td></td></tr>
<tr><td>TRINITY_DN34117_c0_g1_i1_len_304_251738.p1_type_internal_len_102</td><td>---NA---</td><td></td><td></td></tr>
<tr><td>TRINITY_DN33103_c0_g1_i1_len_304_251768.p1_type_internal_len_102</td><td>---NA---</td><td></td><td></td></tr>
<tr><td>TRINITY_DN36840_c0_g1_i1_len_304_251779.p1_type_internal_len_102</td><td>---NA---</td><td></td><td></td></tr>
<tr><td>TRINITY_DN36570_c0_g1_i1_len_304_251781.p1_type_internal_len_102</td><td>---NA---</td><td></td><td></td></tr>
<tr><td>TRINITY_DN31531_c0_g1_i1_len_304_251783.p1_type_internal_len_102</td><td>---NA---</td><td></td><td></td></tr>
<tr><td>TRINITY_DN19843_c0_g1_i1_len_1057_235661.p1_type_complete_len_101</td><td>---NA---</td><td></td><td></td></tr>
<tr><td>TRINITY_DN9017_c0_g1_i1_len_304_251758.p1_type_internal_len_102</td><td>AAW78893.1testis beta tubulin</td><td>F:GO:0003924; F:GO:0005200; F:GO:0005525; C:GO:0005874; P:GO:0007010; P:GO:0007017</td><td>EC:3.6.1.15</td></tr>
<tr><td>TRINITY_DN14124_c0_g1_i1_len_304_251759.p1_type_3prime_partial_len_102</td><td>---NA---</td><td></td><td></td></tr>
<tr><td>TRINITY_DN2923_c0_g1_i1_len_304_251764.p1_type_internal_len_102</td><td>---NA---</td><td></td><td></td></tr>
<tr><td>3100|_|c2628_f3p0_2414_isoform_c2628_full_length_coverage_3_non_full_length_coverage_0_isoform_length_2414_12025</td><td>---NA---</td><td></td><td></td></tr>
<tr><td>3102|_|c11982_f2p5_2413_isoform_c11982_full_length_coverage_2_non_full_length_coverage_5_isoform_length_2413_22097</td><td>---NA---</td><td></td><td></td></tr>
<tr><td>3104|_|c995_f26p16_2412_isoform_c995_full_length_coverage_26_non_full_length_coverage_16_isoform_length_2412_15799</td><td>---NA---</td><td></td><td></td></tr>
<tr><td>TRINITY_DN30178_c0_g1_i1_len_3912_187637.p1_type_5prime_partial_len_804</td><td>XP_018015832.1 ubiquitin carboxyl-terminal hydrolase CYLD-like isoform X2</td><td></td><td></td></tr>
</table>

| | | | |
|---|---|---|---|
| 3106\|_\|c17830_f3p3_2412_isoform_c17830_full_length_coverage_3_non_full_length_coverage_3_isoform_length_2412_13431 | XP_018027040.1 selenocysteine-specific elongation factor-like | P:GO:0001514; F:GO:0003746 | |
| 3107\|_\|c11380_f3p3_2411_isoform_c11380_full_length_coverage_3_non_full_length_coverage_3_isoform_length_2411_22054 | XP_018015859.1 transcriptional regulator ATRX-like | F:GO:0005524 | |
| 3108\|_\|c2788_f4p0_2410_isoform_c2788_full_length_coverage_4_non_full_length_coverage_0_isoform_length_2410_21236 | XP_013791026.1ankyrin repeat and SOCS box protein 1-like | | |
| TRINITY_DN24291_c0_g1_i1_len_2591_189237.p1_type_5prime_partial_len_803 | EKC35155.1Sentrin-specific protease 2 | P:GO:0006508; F:GO:0008233; F:GO:0008234 | |
| TRINITY_DN25488_c0_g1_i1_len_3067_228917.p1_type_5prime_partial_len_803 | ---NA--- | | |
| TRINITY_DN18868_c0_g1_i2_len_2746_58352.p1_type_3prime_partial_len_804 | KFM82580.1Eukaryotic translation initiation factor 4E transporter | F:GO:0003743; P:GO:0006413 | |
| TRINITY_DN10333_c0_g1_i1_len_357_210319.p1_type_5prime_partial_len_101 | ---NA--- | | |
| TRINITY_DN8024_c0_g1_i1_len_602_240758.p1_type_complete_len_101 | ---NA--- | | |
| TRINITY_DN21538_c0_g1_i3_len_3203_154134.p2_type_complete_len_101 | ---NA--- | | |
| TRINITY_DN22980_c0_g1_i1_len_2162_155681.p2_type_5prime_partial_len_101 | ---NA--- | | |
| TRINITY_DN23637_c0_g3_i8_len_335_173276.p1_type_5prime_partial_len_101 | ---NA--- | | |
| TRINITY_DN14793_c0_g1_i1_len_1411_193592.p1_type_5prime_partial_len_101 | ---NA--- | | |
| TRINITY_DN15932_c0_g1_i2_len_703_239159.p1_type_complete_len_101 | ---NA--- | | |
| TRINITY_DN28862_c0_g1_i1_len_432_245171.p1_type_3prime_partial_len_102 | XP_018024053.1 tyrosine-protein phosphatase 10D-like | | |
| TRINITY_DN34894_c0_g1_i1_len_303_175335.p1_type_internal_len_102 | ---NA--- | | |

| | | | |
|---|---|---|---|
| TRINITY_DN20621_c0_g1_i2_len_772_300388.p2_type_complete_len_101 | ---NA--- | | |
| TRINITY_DN21130_c0_g1_i1_len_851_64136.p1_type_5prime_partial_len_101 | ---NA--- | | |
| TRINITY_DN37834_c0_g1_i1_len_327_250199.p1_type_5prime_partial_len_101 | XP_018023046.1 ras-related protein Rab-32-like isoform X2 | F:GO:0003924; F:GO:0005525; C:GO:0005802; P:GO:0016192; C:GO:0031982 | EC:3.6.1.15 |
| TRINITY_DN33177_c0_g1_i1_len_1557_192778.p1_type_complete_len_101 | ---NA--- | | |
| TRINITY_DN11611_c0_g1_i1_len_442_70839.p1_type_5prime_partial_len_101 | ---NA--- | | |
| TRINITY_DN8198_c0_g1_i1_len_327_250180.p1_type_5prime_partial_len_101 | ---NA--- | | |
| TRINITY_DN36853_c0_g1_i1_len_327_250186.p1_type_3prime_partial_len_102 | ACU31809.1alpha2 macroglobulin isoform 3 | C:GO:0005576 | |
| TRINITY_DN31141_c0_g1_i1_len_8414_186761.p8_type_complete_len_101 | ---NA--- | | |
| TRINITY_DN31307_c0_g1_i1_len_307_251530.p1_type_internal_len_102 | XP_014674344.1 N-acetylated-alpha-linked acidic dipeptidase 2-like isoform X2 | | |
| TRINITY_DN15095_c0_g1_i1_len_306_175094.p1_type_internal_len_102 | ---NA--- | | |
| TRINITY_DN18659_c0_g1_i1_len_2064_155931.p5_type_5prime_partial_len_101 | ---NA--- | | |
| TRINITY_DN16977_c0_g1_i1_len_945_236567.p1_type_complete_len_101 | ---NA--- | | |
| TRINITY_DN1129_c0_g1_i1_len_307_251509.p1_type_internal_len_102 | XP_018022944.1 carboxypeptidase D-like | F:GO:0004180; P:GO:0006508 | |
| TRINITY_DN7369_c0_g1_i1_len_307_251545.p1_type_internal_len_102 | ---NA--- | | |
| TRINITY_DN29575_c0_g1_i1_len_2291_189975.p4_type_complete_len_101 | ---NA--- | | |
| TRINITY_DN7240_c0_g1_i1_len_309_174872.p1_type_5prime_partial_len_101 | ---NA--- | | |

| | | | |
|---|---|---|---|
| TRINITY_DN3185_c0_g1_i1_len_306_175117.p1_type_internal_len_102 | ---NA--- | | |
| TRINITY_DN33504_c0_g1_i1_len_426_245415.p1_type_complete_len_101 | ---NA--- | | |
| TRINITY_DN6759_c0_g1_i1_len_307_175031.p1_type_internal_len_102 | ---NA--- | | |
| TRINITY_DN1124_c0_g1_i1_len_307_175004.p1_type_internal_len_102 | ---NA--- | | |
| TRINITY_DN29742_c0_g1_i1_len_344_43840.p1_type_5prime_partial_len_101 | ---NA--- | | |
| TRINITY_DN3493_c0_g1_i1_len_306_175088.p1_type_internal_len_102 | ---NA--- | | |
| TRINITY_DN15775_c0_g1_i1_len_849_161953.p2_type_5prime_partial_len_101 | ---NA--- | | |
| TRINITY_DN29981_c0_g1_i1_len_332_173460.p1_type_5prime_partial_len_101 | ---NA--- | | |
| TRINITY_DN2974_c0_g1_i1_len_305_175180.p1_type_internal_len_102 | ---NA--- | | |
| TRINITY_DN7050_c0_g1_i1_len_305_251663.p2_type_internal_len_102 | ---NA--- | | |
| TRINITY_DN36174_c0_g1_i1_len_305_251671.p1_type_internal_len_102 | ---NA--- | | |
| TRINITY_DN34236_c0_g1_i1_len_305_251672.p1_type_internal_len_102 | ---NA--- | | |
| TRINITY_DN26109_c0_g1_i5_len_1562_192750.p3_type_complete_len_101 | ---NA--- | | |
| TRINITY_DN4850_c0_g1_i1_len_305_251657.p1_type_internal_len_102 | XP_018015378.1 GTP-binding protein 2-like | F:GO:0003924; F:GO:0005525 | |
| TRINITY_DN35007_c0_g1_i1_len_305_251696.p1_type_internal_len_102 | ---NA--- | | |
| TRINITY_DN10900_c0_g1_i1_len_305_251700.p1_type_internal_len_102 | ---NA--- | | |

| | | | |
|---|---|---|---|
| TRINITY_DN28570_c0_g1_i1_len_305_251706.p1_type_internal_len_102 | XP_011695685.1 H(+)/Cl(-) exchange transporter 5 isoform X4 | | |
| TRINITY_DN33845_c0_g1_i1_len_305_251677.p1_type_internal_len_102 | XP_018011656.1 laminin subunit alpha-2-like | | |
| TRINITY_DN14896_c0_g1_i1_len_538_166218.p1_type_complete_len_101 | ---NA--- | | |
| TRINITY_DN7633_c0_g1_i1_len_305_251681.p1_type_internal_len_102 | ---NA--- | | |
| TRINITY_DN26518_c0_g1_i1_len_305_175184.p1_type_internal_len_102 | XP_018010015.1 4-coumarate--CoA ligase-like 4 | F:GO:0003824 | |
| TRINITY_DN33099_c0_g1_i1_len_306_251583.p1_type_internal_len_102 | ---NA--- | | |
| TRINITY_DN15732_c0_g1_i1_len_375_12726.p1_type_5prime_partial_len_101 | ---NA--- | | |
| TRINITY_DN33327_c0_g1_i1_len_306_251584.p1_type_internal_len_102 | ---NA--- | | |
| TRINITY_DN10356_c0_g1_i2_len_442_305607.p1_type_5prime_partial_len_101 | XP_022241908.1kyphoscoliosis peptidase-like | | |
| TRINITY_DN31787_c0_g1_i1_len_306_251601.p1_type_internal_len_102 | ---NA--- | | |
| TRINITY_DN27320_c0_g1_i1_len_306_251603.p1_type_internal_len_102 | AHY82567.1transcription factor ATF-b | F:GO:0003700; P:GO:0006355 | |
| TRINITY_DN15210_c0_g1_i3_len_305_175141.p1_type_internal_len_102 | ---NA--- | | |
| TRINITY_DN3008_c0_g1_i1_len_306_251566.p1_type_internal_len_102 | ---NA--- | | |
| TRINITY_DN18875_c0_g1_i1_len_1403_193638.p1_type_5prime_partial_len_101 | ---NA--- | | |
| TRINITY_DN9517_c0_g1_i1_len_449_305438.p2_type_complete_len_101 | ---NA--- | | |
| TRINITY_DN12631_c0_g1_i3_len_306_251632.p1_type_internal_len_102 | ---NA--- | | |

| | | | |
|---|---|---|---|
| TRINITY_DN27933_c0_g1_i1_len_305_251643.p1_type_internal_len_102 | ---NA--- | | |
| TRINITY_DN21167_c0_g1_i1_len_1595_192592.p2_type_5prime_partial_len_101 | ---NA--- | | |
| TRINITY_DN27036_c0_g1_i1_len_452_244495.p1_type_5prime_partial_len_101 | XP_013148572.1 retinol dehydrogenase 12-like | | |
| TRINITY_DN32603_c0_g1_i1_len_381_247190.p1_type_5prime_partial_len_101 | ---NA--- | | |
| TRINITY_DN11709_c0_g1_i2_len_423_206990.p1_type_3prime_partial_len_102 | ---NA--- | | |
| TRINITY_DN17286_c0_g1_i1_len_1083_235496.p2_type_complete_len_101 | ---NA--- | | |
| TRINITY_DN14817_c0_g1_i1_len_625_302037.p1_type_5prime_partial_len_101 | ---NA--- | | |
| TRINITY_DN36797_c0_g1_i1_len_316_250929.p1_type_5prime_partial_len_101 | ---NA--- | | |
| TRINITY_DN19767_c0_g1_i3_len_1593_192603.p3_type_complete_len_101 | ---NA--- | | |
| TRINITY_DN28685_c0_g1_i2_len_5535_187005.p5_type_complete_len_101 | ---NA--- | | |
| TRINITY_DN30985_c6_g1_i6_len_986_196676.p1_type_5prime_partial_len_101 | ---NA--- | | |
| TRINITY_DN33379_c0_g1_i1_len_320_174170.p1_type_5prime_partial_len_101 | ---NA--- | | |
| TRINITY_DN22673_c0_g1_i2_len_532_68362.p1_type_3prime_partial_len_102 | ---NA--- | | |
| TRINITY_DN30954_c0_g1_i4_len_2079_190625.p1_type_complete_len_101 | ---NA--- | | |
| TRINITY_DN23120_c0_g1_i1_len_1165_234928.p1_type_complete_len_101 | ---NA--- | | |
| TRINITY_DN2585_c0_g1_i1_len_379_247306.p1_type_5prime_partial_len_101 | XP_018011797.1 E3 ubiquitin-protein ligase HUWE1-like | P:GO:0000209; C:GO:0005634; C:GO:0005737; P:GO:0006511; F:GO:0016874; F:GO:0061630 | |

| | | | |
|---|---|---|---|
| TRINITY_DN5713_c0_g1_i1_len_487_304466.p1_type_5prime_partial_len_101 | ---NA--- | | |
| TRINITY_DN22741_c0_g1_i2_len_854_237386.p1_type_complete_len_101 | ---NA--- | | |
| TRINITY_DN25295_c0_g1_i1_len_3324_188103.p3_type_3prime_partial_len_102 | ---NA--- | | |
| TRINITY_DN32846_c0_g1_i1_len_458_244291.p1_type_3prime_partial_len_102 | ---NA--- | | |
| TRINITY_DN3556_c0_g1_i1_len_1518_232940.p2_type_complete_len_101 | ---NA--- | | |
| TRINITY_DN7153_c0_g1_i1_len_341_44021.p1_type_3prime_partial_len_102 | ---NA--- | | |
| TRINITY_DN2192_c0_g1_i1_len_474_69845.p1_type_5prime_partial_len_101 | ---NA--- | | |
| TRINITY_DN10049_c0_g1_i1_len_315_251010.p1_type_5prime_partial_len_101 | ---NA--- | | |
| TRINITY_DN31069_c0_g1_i10_len_3572_187869.p5_type_complete_len_101 | ---NA--- | | |
| TRINITY_DN20041_c0_g1_i1_len_394_208320.p1_type_3prime_partial_len_102 | ---NA--- | | |
| TRINITY_DN20346_c0_g1_i3_len_1745_30873.p3_type_5prime_partial_len_101 | ---NA--- | | |
| TRINITY_DN28233_c0_g1_i1_len_1057_196053.p1_type_3prime_partial_len_102 | ---NA--- | | |
| TRINITY_DN17109_c0_g1_i2_len_1252_61689.p2_type_5prime_partial_len_101 | ---NA--- | | |
| TRINITY_DN19998_c0_g1_i1_len_347_210926.p1_type_5prime_partial_len_101 | ---NA--- | | |
| TRINITY_DN24529_c0_g1_i1_len_1887_191325.p2_type_3prime_partial_len_102 | ---NA--- | | |
| TRINITY_DN4872_c0_g1_i2_len_387_246916.p1_type_5prime_partial_len_101 | ---NA--- | | |

| | | | |
|---|---|---|---|
| TRINITY_DN19855_c0_g1_i1_len_1270_61619.p2_type_complete_len_101 | ---NA--- | | |
| TRINITY_DN13536_c0_g1_i3_len_506_242882.p2_type_complete_len_101 | ---NA--- | | |
| TRINITY_DN9267_c0_g1_i2_len_637_164420.p1_type_3prime_partial_len_102 | ---NA--- | | |
| TRINITY_DN2278_c0_g1_i1_len_536_242102.p1_type_complete_len_101 | ---NA--- | | |
| TRINITY_DN16587_c0_g1_i3_len_640_164374.p1_type_5prime_partial_len_101 | ---NA--- | | |
| TRINITY_DN18946_c1_g1_i2_len_2344_29927.p3_type_5prime_partial_len_101 | ---NA--- | | |
| TRINITY_DN494_c0_g1_i1_len_461_9782.p1_type_5prime_partial_len_101 | XP_018008461.1 ubiquitin carboxyl-terminal hydrolase 46-like | F:GO:0004197; F:GO:0004843; P:GO:0006511; P:GO:0016579 | EC:3.4.22; EC:3.4.19.12 |
| TRINITY_DN22863_c1_g1_i1_len_1928_156327.p2_type_5prime_partial_len_101 | ---NA--- | | |
| TRINITY_DN17381_c0_g1_i1_len_533_242179.p1_type_complete_len_101 | ---NA--- | | |
| TRINITY_DN7909_c0_g1_i1_len_391_246784.p1_type_3prime_partial_len_102 | ---NA--- | | |
| TRINITY_DN15388_c0_g1_i1_len_997_196574.p1_type_5prime_partial_len_101 | ---NA--- | | |
| TRINITY_DN23443_c0_g2_i1_len_4831_153473.p1_type_5prime_partial_len_1409 | ---NA--- | | |
| TRINITY_DN24538_c0_g1_i1_len_4444_57766.p1_type_5prime_partial_len_1408 | KDR13941.1Cohesin subunit SA-1 | | |
| TRINITY_DN30217_c0_g1_i1_len_5451_187021.p1_type_complete_len_1408 | XP_012252103.1sterol regulatory element-binding protein cleavage-activating protein isoform X1 | | |
| 327\|_\|c12377_f2p193_4222_isoform_c12377_full_length_coverage_2_non_full_length_coverage_193_isoform_length_4222_26183 | AGI56293.2thrombospondin II | F:GO:0005509; C:GO:0005576; P:GO:0006030; P:GO:0007155; F:GO:0008061 | |

<table>
<tr><td></td><td></td><td></td><td></td></tr>
<tr><td>TRINITY_DN30866_c0_g1_i1_len_4691_187233.p1_type_complete_len_1407</td><td>PNF14178.1Tonsoku-like protein</td><td></td><td></td></tr>
<tr><td>TRINITY_DN30422_c0_g1_i1_len_4606_187269.p1_type_complete_len_1407</td><td>XP_018017849.1 intron-binding protein aquarius-like</td><td></td><td></td></tr>
<tr><td>332|_|c30085_f23p17_4216_isoform_c30085_full_length_coverage_23_non_full_length_coverage_17_isoform_length_4216_27140</td><td>XP_018019971.1PREDICTED: uncharacterized protein LOC108676407</td><td></td><td></td></tr>
<tr><td>333|_|c11658_f2p95_4214_isoform_c11658_full_length_coverage_2_non_full_length_coverage_95_isoform_length_4214_26118</td><td>AMJ52354.1cullin 1</td><td>P:GO:0006511; C:GO:0031461; F:GO:0031625</td><td></td></tr>
<tr><td>TRINITY_DN11582_c0_g1_i1_len_4414_227977.p1_type_complete_len_1404</td><td>XP_015116996.1 calpain-D-like</td><td></td><td></td></tr>
<tr><td>TRINITY_DN23912_c0_g1_i2_len_4284_228038.p1_type_5prime_partial_len_1403</td><td>XP_023704668.1uncharacterized protein LOC111862993 isoform X2</td><td></td><td></td></tr>
<tr><td>3113|_|c5419_f3p2_2409_isoform_c5419_full_length_coverage_3_non_full_length_coverage_2_isoform_length_2409_16965</td><td>---NA---</td><td></td><td></td></tr>
<tr><td>3115|_|c10082_f2p1_2409_isoform_c10082_full_length_coverage_2_non_full_length_coverage_1_isoform_length_2409_1113</td><td>XP_023715783.1probable elongator complex protein 3 isoform X2</td><td>F:GO:0004402; P:GO:0016573; F:GO:0046872; F:GO:0051536</td><td>EC:2.3.1.5; EC:2.3.1.48</td></tr>
<tr><td>TRINITY_DN22817_c0_g1_i1_len_2655_154745.p1_type_5prime_partial_len_803</td><td>XP_018024966.1 polycystic kidney disease protein 1-like 2</td><td>F:GO:0005509; C:GO:0016020; C:GO:0016021</td><td></td></tr>
<tr><td>TRINITY_DN16928_c0_g1_i1_len_3233_154104.p1_type_5prime_partial_len_803</td><td>XP_023223688.1rho GTPase-activating protein 190-like isoform X6</td><td></td><td></td></tr>
<tr><td>3120|_|c28167_f2p1_2407_isoform_c28167_full_length_coverage_2_non_full_length_coverage_1_isoform_length_2407_14016</td><td>KDR21294.1Protein preli-like</td><td>C:GO:0005758</td><td></td></tr>
<tr><td>3121|_|c27021_f2p2_2407_isoform_c27021_full_length_coverage_2_non_full_length_coverage_2_isoform_length_2407_18413</td><td>XP_023230969.1crooked neck-like protein 1</td><td></td><td></td></tr>
</table>

| | | | |
|---|---|---|---|
| 3122\|_\|c6800_f2p0_2407_isoform_c6800_full_length_coverage_2_non_full_length_coverage_0_isoform_length_2407_17083 | XP_022818514.1uncharacterized protein LOC111350991 isoform X8 | | |
| TRINITY_DN24522_c0_g1_i3_len_3385_57971.p1_type_5prime_partial_len_802 | XP_023323894.1MICOS complex subunit MIC60-1-like | C:GO:0016020 | |
| TRINITY_DN23617_c0_g1_i4_len_2601_154831.p1_type_5prime_partial_len_802 | XP_001186509.2PREDICTED: uncharacterized protein LOC754648 | F:GO:0003676; F:GO:0003700; P:GO:0006355; F:GO:0008270; P:GO:0015074 | |
| 3126\|_\|c22166_f2p1_2406_isoform_c22166_full_length_coverage_2_non_full_length_coverage_1_isoform_length_2406_18254 | ---NA--- | | |
| TRINITY_DN18981_c0_g1_i1_len_434_305858.p2_type_5prime_partial_len_101 | ---NA--- | | |
| TRINITY_DN23871_c0_g1_i1_len_1859_231531.p2_type_3prime_partial_len_102 | ---NA--- | | |
| TRINITY_DN22752_c0_g1_i1_len_1298_194273.p3_type_5prime_partial_len_101 | ---NA--- | | |
| TRINITY_DN11136_c0_g1_i1_len_581_67389.p2_type_complete_len_101 | APG16029.1broad-complex | F:GO:0003676 | |
| TRINITY_DN13474_c0_g1_i1_len_336_211635.p1_type_5prime_partial_len_101 | ---NA--- | | |
| TRINITY_DN20142_c0_g1_i1_len_960_126812.p3_type_5prime_partial_len_101 | ---NA--- | | |
| TRINITY_DN8199_c0_g1_i1_len_305_213876.p2_type_internal_len_102 | KQK77373.1MAU2 chromatid cohesion factor like protein | P:GO:0007064 | |
| TRINITY_DN18690_c0_g1_i1_len_354_74528.p1_type_3prime_partial_len_102 | ---NA--- | | |
| TRINITY_DN29649_c0_g1_i1_len_387_279443.p1_type_5prime_partial_len_101 | ---NA--- | | |
| TRINITY_DN41028_c0_g1_i1_len_303_214071.p1_type_internal_len_102 | APP91326.1retinoid X receptor | F:GO:0003700; F:GO:0003707; C:GO:0005634; P:GO:0006355; F:GO:0008270; P:GO:0043401; F:GO:0043565 | |

| | | | |
|---|---|---|---|
| TRINITY_DN8525_c0_g1_i1_len_305_213908.p4_type_internal_len_102 | XP_018011656.1 laminin subunit alpha-2-like | | |
| TRINITY_DN11443_c0_g1_i2_len_463_39564.p1_type_5prime_partial_len_101 | ---NA--- | | |
| TRINITY_DN10278_c0_g1_i1_len_307_213758.p1_type_internal_len_102 | ---NA--- | | |
| TRINITY_DN15430_c0_g1_i1_len_645_6353.p2_type_5prime_partial_len_101 | XP_018027471.1 ADP-ribosylation factor-like protein 16 | | |
| TRINITY_DN36518_c0_g1_i1_len_305_213885.p2_type_internal_len_102 | ---NA--- | | |
| TRINITY_DN28626_c0_g1_i1_len_305_77698.p3_type_internal_len_102 | ---NA--- | | |
| TRINITY_DN11442_c0_g1_i2_len_329_107877.p1_type_5prime_partial_len_101 | ---NA--- | | |
| TRINITY_DN11213_c0_g1_i1_len_444_277536.p2_type_5prime_partial_len_101 | ---NA--- | | |
| TRINITY_DN13982_c0_g1_i1_len_315_15909.p1_type_5prime_partial_len_101 | ---NA--- | | |
| TRINITY_DN21020_c0_g1_i1_len_332_211911.p1_type_5prime_partial_len_101 | ---NA--- | | |
| TRINITY_DN11270_c0_g1_i1_len_304_213976.p1_type_internal_len_102 | ---NA--- | | |
| TRINITY_DN11431_c0_g1_i1_len_316_108687.p1_type_5prime_partial_len_101 | ---NA--- | | |
| TRINITY_DN68_c0_g1_i1_len_305_140540.p1_type_internal_len_102 | XP_018024151.1 exonuclease 3'-5' domain-containing protein 2-like | | |
| TRINITY_DN5654_c0_g1_i1_len_305_140544.p1_type_internal_len_102 | ---NA--- | | |
| TRINITY_DN2084_c0_g1_i1_len_305_140548.p1_type_internal_len_102 | ---NA--- | | |
| TRINITY_DN31987_c0_g1_i1_len_518_131944.p1_type_5prime_partial_len_101 | ---NA--- | | |

| | | | |
|---|---|---|---|
| TRINITY_DN15745_c0_g1_i1_len_305_140560.p1_type_internal_len_102 | ---NA--- | | |
| TRINITY_DN2403_c0_g1_i1_len_307_213767.p1_type_internal_len_102 | XP_021923286.1cytoplasmic dynein 2 light intermediate chain 1 isoform X4 | | |
| TRINITY_DN26581_c0_g1_i1_len_306_140498.p1_type_internal_len_102 | ---NA--- | | |
| TRINITY_DN36297_c0_g1_i1_len_304_213993.p1_type_internal_len_102 | ---NA--- | | |
| TRINITY_DN26338_c0_g1_i1_len_305_140597.p1_type_3prime_partial_len_102 | ACR23315.1chitinase 5 | F:GO:0004553; C:GO:0005576; P:GO:0005975; P:GO:0006030; F:GO:0008061 | |
| TRINITY_DN3616_c0_g1_i1_len_305_140599.p1_type_internal_len_102 | ---NA--- | | |
| TRINITY_DN38638_c0_g1_i1_len_303_214043.p1_type_internal_len_102 | ---NA--- | | |
| TRINITY_DN26257_c0_g1_i1_len_305_140567.p1_type_internal_len_102 | ---NA--- | | |
| TRINITY_DN19244_c0_g1_i2_len_451_102637.p2_type_5prime_partial_len_101 | ACD74843.1trehalose-6-phosphate synthase | F:GO:0003825; P:GO:0005992 | EC:2.4.1.15 |
| TRINITY_DN30683_c0_g1_i1_len_399_135433.p1_type_5prime_partial_len_101 | AAS57714.1peroxinectin, partial | F:GO:0004601; P:GO:0006979; F:GO:0020037; P:GO:0055114; P:GO:0098869 | EC:1.11.1.7 |
| TRINITY_DN13541_c0_g1_i1_len_332_75790.p1_type_5prime_partial_len_101 | ---NA--- | | |
| TRINITY_DN16807_c0_g1_i1_len_320_108437.p1_type_5prime_partial_len_101 | ---NA--- | | |
| TRINITY_DN30283_c0_g1_i1_len_304_77717.p1_type_internal_len_102 | XP_018020290.1PREDICTED: uncharacterized protein LOC108676682 | | |
| TRINITY_DN14130_c0_g1_i1_len_310_140143.p1_type_5prime_partial_len_101 | ---NA--- | | |
| TRINITY_DN1963_c0_g1_i1_len_303_214053.p1_type_internal_len_102 | ---NA--- | | |

| | | | |
|---|---|---|---|
| TRINITY_DN13034_c0_g1_i1_len_693_98168.p2_type_3prime_partial_len_102 | ---NA--- | | |
| TRINITY_DN6839_c0_g1_i1_len_306_140465.p1_type_internal_len_102 | ---NA--- | | |
| TRINITY_DN10389_c0_g1_i1_len_304_213987.p1_type_internal_len_102 | ---NA--- | | |
| TRINITY_DN4456_c0_g1_i1_len_304_213989.p1_type_internal_len_102 | ---NA--- | | |
| TRINITY_DN31547_c0_g1_i1_len_306_140488.p1_type_internal_len_102 | ---NA--- | | |
| TRINITY_DN27968_c0_g1_i1_len_307_140423.p1_type_internal_len_102 | ---NA--- | | |
| TRINITY_DN10534_c0_g1_i1_len_829_198273.p3_type_complete_len_101 | ---NA--- | | |
| TRINITY_DN34047_c0_g1_i1_len_304_213948.p1_type_internal_len_102 | ---NA--- | | |
| TRINITY_DN25664_c0_g1_i1_len_342_107055.p1_type_5prime_partial_len_101 | ---NA--- | | |
| TRINITY_DN31811_c0_g1_i1_len_305_213892.p1_type_internal_len_102 | ---NA--- | | |
| TRINITY_DN10643_c0_g1_i1_len_331_211981.p1_type_5prime_partial_len_101 | AVP74312.1leucine rich repeat only protein 2 | | |
| TRINITY_DN17094_c0_g1_i1_len_881_34209.p1_type_5prime_partial_len_101 | ---NA--- | | |
| TRINITY_DN1311_c0_g1_i1_len_309_213564.p1_type_5prime_partial_len_101 | ---NA--- | | |
| TRINITY_DN14072_c0_g1_i1_len_473_133034.p1_type_3prime_partial_len_102 | ---NA--- | | |
| TRINITY_DN11113_c0_g1_i3_len_344_106928.p1_type_5prime_partial_len_101 | ---NA--- | | |
| TRINITY_DN35445_c0_g1_i1_len_304_213947.p1_type_internal_len_102 | ---NA--- | | |

| | | | |
|---|---|---|---|
| TRINITY_DN15412_c0_g1_i1_len_540_100459.p1_type_complete_len_101 | ---NA--- | | |
| TRINITY_DN30369_c0_g1_i1_len_711_272679.p2_type_5prime_partial_len_101 | ---NA--- | | |
| TRINITY_DN29770_c0_g1_i1_len_306_16457.p2_type_internal_len_102 | XP_018010987.1 pro-resilin-like | | |
| TRINITY_DN18591_c0_g1_i1_len_1526_124179.p3_type_complete_len_101 | ---NA--- | | |
| TRINITY_DN18888_c0_g1_i1_len_1526_124178.p4_type_3prime_partial_len_102 | ---NA--- | | |
| TRINITY_DN15048_c0_g1_i1_len_305_77641.p1_type_internal_len_102 | ---NA--- | | |
| TRINITY_DN18417_c0_g1_i1_len_678_200337.p1_type_5prime_partial_len_101 | ---NA--- | | |
| TRINITY_DN38775_c0_g1_i1_len_305_213904.p1_type_internal_len_102 | ---NA--- | | |
| TRINITY_DN34379_c0_g1_i1_len_305_213900.p1_type_internal_len_102 | ---NA--- | | |
| TRINITY_DN25934_c0_g1_i1_len_365_105884.p1_type_5prime_partial_len_101 | ---NA--- | | |
| TRINITY_DN16406_c0_g1_i1_len_367_105762.p3_type_5prime_partial_len_101 | ---NA--- | | |
| TRINITY_DN40981_c0_g1_i1_len_304_213945.p2_type_internal_len_102 | ---NA--- | | |
| TRINITY_DN30759_c2_g2_i2_len_879_197711.p1_type_5prime_partial_len_101 | ---NA--- | | |
| TRINITY_DN14666_c0_g1_i2_len_689_200171.p1_type_5prime_partial_len_101 | ---NA--- | | |
| TRINITY_DN15169_c0_g1_i3_len_305_77664.p1_type_internal_len_102 | ---NA--- | | |
| TRINITY_DN4995_c0_g1_i1_len_304_213944.p1_type_internal_len_102 | ---NA--- | | |

| | | | |
|---|---|---|---|
| TRINITY_DN12902_c0_g1_i1_len_730_199581.p1_type_complete_len_101 | ---NA--- | | |
| TRINITY_DN11981_c0_g1_i1_len_685_200237.p2_type_3prime_partial_len_102 | ---NA--- | | |
| TRINITY_DN28530_c0_g1_i1_len_306_77599.p1_type_internal_len_102 | ---NA--- | | |
| TRINITY_DN341_c0_g1_i2_len_308_77443.p1_type_5prime_partial_len_101 | ---NA--- | | |
| TRINITY_DN28058_c0_g1_i1_len_305_213941.p1_type_internal_len_102 | ---NA--- | | |
| TRINITY_DN35947_c0_g1_i1_len_307_213734.p2_type_internal_len_102 | XP_018012069.1 zinc finger protein 2 homolog | | |
| TRINITY_DN6351_c0_g1_i1_len_307_77471.p2_type_5prime_partial_len_101 | ---NA--- | | |
| TRINITY_DN21355_c0_g1_i2_len_2193_91832.p3_type_5prime_partial_len_101 | ---NA--- | | |
| TRINITY_DN16513_c0_g1_i1_len_307_213749.p1_type_internal_len_102 | ---NA--- | | |
| TRINITY_DN18425_c0_g1_i1_len_542_203093.p2_type_3prime_partial_len_102 | ---NA--- | | |
| TRINITY_DN7040_c0_g1_i1_len_303_214091.p1_type_internal_len_102 | ANQ44702.1heat shock protein 70 kDa | F:GO:0005524 | |
| TRINITY_DN11847_c0_g1_i1_len_305_77695.p1_type_internal_len_102 | ---NA--- | | |
| TRINITY_DN23926_c0_g1_i2_len_671_129326.p1_type_5prime_partial_len_101 | ---NA--- | | |
| TRINITY_DN35788_c0_g1_i1_len_305_77688.p1_type_internal_len_102 | ---NA--- | | |
| TRINITY_DN18554_c0_g1_i2_len_304_213949.p1_type_internal_len_102 | ---NA--- | | |
| TRINITY_DN20352_c0_g1_i3_len_1244_125235.p2_type_5prime_partial_len_101 | ---NA--- | | |

| | | | |
|---|---|---|---|
| TRINITY_DN25027_c0_g3_i1_len_350_74743.p1_type_5prime_partial_len_101 | ---NA--- | | |
| TRINITY_DN23443_c0_g1_i1_len_303_214092.p1_type_internal_len_102 | ---NA--- | | |
| TRINITY_DN41098_c0_g1_i1_len_304_213967.p3_type_internal_len_102 | ---NA--- | | |
| TRINITY_DN5577_c0_g1_i1_len_307_213727.p1_type_internal_len_102 | ---NA--- | | |
| TRINITY_DN5230_c0_g1_i1_len_306_16461.p2_type_internal_len_102 | XP_018023003.1 transmembrane protein 184C-like | | |
| TRINITY_DN1106_c0_g1_i1_len_303_214100.p1_type_internal_len_102 | ---NA--- | | |
| TRINITY_DN21086_c0_g1_i1_len_305_213929.p1_type_internal_len_102 | XP_018016658.1 protein DENND6A-like | F:GO:0017112 | |
| TRINITY_DN14668_c0_g1_i1_len_305_16510.p2_type_internal_len_102 | XP_013782432.2UPF0462 protein C4orf33 homolog | | |
| TRINITY_DN19281_c0_g1_i1_len_303_77780.p1_type_internal_len_102 | ---NA--- | | |
| TRINITY_DN29060_c0_g1_i1_len_304_283773.p1_type_3prime_partial_len_102 | XP_018015531.1PREDICTED: uncharacterized protein LOC108672397 | | |
| TRINITY_DN35089_c0_g1_i1_len_306_213792.p2_type_internal_len_102 | ---NA--- | | |
| 3127\|_\|c7970_f3p0_2406_isoform_c7970_full_length_coverage_3_non_full_length_coverage_0_isoform_length_2406_21735 | ---NA--- | | |
| 3128\|_\|c2718_f2p3_2406_isoform_c2718_full_length_coverage_2_non_full_length_coverage_3_isoform_length_2406_12101 | XP_012531249.1 actin-related protein 5 | | |
| 3129\|_\|c2229_f2p0_2406_isoform_c2229_full_length_coverage_2_non_full_length_coverage_0_isoform_length_2406_16249 | ---NA--- | | |

| | | | |
|---|---|---|---|
| 3130\|_\|c1219_f17p5_2405_isoform_c1219_full_length_coverage_17_non_full_length_coverage_5_isoform_length_2405_15823 | ASW35103.1Beta-amyloid-like protein | F:GO:0008201; C:GO:0016021; F:GO:0046914 | |
| 3131\|_\|c5746_f4p1_2404_isoform_c5746_full_length_coverage_4_non_full_length_coverage_1_isoform_length_2404_12500 | AAI16551.1Zgc:136471 protein | C:GO:0005634; P:GO:0006260 | |
| 3132\|_\|c2405_f7p3_2404_isoform_c2405_full_length_coverage_7_non_full_length_coverage_3_isoform_length_2404_16385 | XP_022306545.1DNA mismatch repair protein Mlh1-like | | |
| 3133\|_\|c40364_f2p1_2403_isoform_c40364_full_length_coverage_2_non_full_length_coverage_1_isoform_length_2403_19073 | XP_018008679.1 U5 small nuclear ribonucleoprotein 200 kDa helicase-like | F:GO:0003676; F:GO:0004386; F:GO:0005524; C:GO:0016021 | EC:3.6.1.15 |
| TRINITY_DN24548_c1_g1_i5_len_3712_228339.p1_type_complete_len_801 | ATY51984.1cAMP-responsive element binding protein-3 | F:GO:0003700; P:GO:0030968; F:GO:0035497; P:GO:0045944 | |
| 3136\|_\|c13081_f2p1_2403_isoform_c13081_full_length_coverage_2_non_full_length_coverage_1_isoform_length_2403_22189 | ---NA--- | | |
| 3138\|_\|c43089_f2p3_2403_isoform_c43089_full_length_coverage_2_non_full_length_coverage_3_isoform_length_2403_15017 | XP_022343652.125S rRNA (cytosine-C(5))-methyltransferase nop2-like | | |
| TRINITY_DN1402_c0_g1_i1_len_306_213793.p1_type_internal_len_102 | ---NA--- | | |
| TRINITY_DN29566_c0_g2_i1_len_634_201126.p1_type_5prime_partial_len_101 | ---NA--- | | |
| TRINITY_DN13612_c0_g1_i1_len_305_109434.p1_type_internal_len_102 | ---NA--- | | |
| TRINITY_DN10497_c0_g1_i2_len_303_283802.p1_type_internal_len_102 | ---NA--- | | |
| TRINITY_DN22185_c0_g1_i1_len_303_283817.p1_type_internal_len_102 | ---NA--- | | |
| TRINITY_DN319_c0_g1_i1_len_303_283819.p1_type_internal_len_102 | ---NA--- | | |

| | | | |
|---|---|---|---|
| TRINITY_DN29715_c0_g1_i1_len_418_134751.p1_type_5prime_partial_len_101 | ---NA--- | | |
| TRINITY_DN31649_c0_g1_i1_len_306_109391.p1_type_internal_len_102 | ---NA--- | | |
| TRINITY_DN26712_c0_g1_i1_len_306_109404.p1_type_internal_len_102 | ---NA--- | | |
| TRINITY_DN29703_c0_g1_i1_len_304_77775.p1_type_internal_len_102 | GBC18203.1retrotransposable element | | |
| TRINITY_DN30603_c0_g1_i1_len_306_109369.p1_type_internal_len_102 | ---NA--- | | |
| TRINITY_DN7606_c0_g1_i1_len_619_201396.p2_type_3prime_partial_len_102 | XP_018011033.1 putative polypeptide N-acetylgalactosaminyltransferase 9 isoform X1 | | |
| TRINITY_DN29690_c0_g1_i1_len_306_109380.p1_type_internal_len_102 | XP_015605113.1non-lysosomal glucosylceramidase | F:GO:0004553 | |
| TRINITY_DN2323_c0_g1_i1_len_304_77767.p1_type_internal_len_102 | ---NA--- | | |
| TRINITY_DN9257_c0_g1_i1_len_306_109382.p1_type_internal_len_102 | ---NA--- | | |
| TRINITY_DN33029_c0_g1_i1_len_305_109422.p1_type_internal_len_102 | ---NA--- | | |
| TRINITY_DN14969_c0_g1_i2_len_325_76200.p1_type_5prime_partial_len_101 | ---NA--- | | |
| TRINITY_DN28078_c0_g1_i1_len_307_16412.p1_type_internal_len_102 | ---NA--- | | |
| TRINITY_DN23354_c0_g1_i1_len_304_283740.p1_type_internal_len_102 | XP_018011984.1 delta-like protein B | F:GO:0005509; P:GO:0007219; P:GO:0007275; C:GO:0016021 | |
| TRINITY_DN6283_c0_g1_i1_len_305_283692.p1_type_internal_len_102 | ---NA--- | | |
| TRINITY_DN21205_c0_g1_i1_len_305_283706.p1_type_internal_len_102 | ---NA--- | | |
| TRINITY_DN6353_c0_g1_i1_len_305_283710.p1_type_internal_len_102 | ---NA--- | | |

| | | | |
|---|---|---|---|
| TRINITY_DN3270_c0_g1_i1_len_304_283720.p1_type_internal_len_102 | XP_023327503.1uncharacterized protein LOC111700723 | | |
| TRINITY_DN34530_c0_g1_i1_len_305_213926.p2_type_internal_len_102 | ---NA--- | | |
| TRINITY_DN22925_c0_g1_i2_len_306_213809.p1_type_internal_len_102 | ---NA--- | | |
| TRINITY_DN19677_c1_g1_i3_len_661_273259.p1_type_complete_len_101 | ---NA--- | | |
| TRINITY_DN26967_c0_g1_i1_len_303_77836.p1_type_3prime_partial_len_102 | ---NA--- | | |
| TRINITY_DN12383_c0_g1_i1_len_306_213815.p1_type_internal_len_102 | ---NA--- | | |
| TRINITY_DN15375_c0_g1_i1_len_652_6275.p2_type_5prime_partial_len_101 | ADG86240.1triosephosphate isomerase | F:GO:0004807; P:GO:0006094; P:GO:0006096 | EC:5.3.1.1 |
| TRINITY_DN10225_c0_g1_i1_len_304_109541.p1_type_internal_len_102 | ---NA--- | | |
| TRINITY_DN30491_c0_g1_i1_len_304_109509.p1_type_internal_len_102 | ---NA--- | | |
| TRINITY_DN827_c0_g1_i1_len_303_283840.p1_type_internal_len_102 | AET34915.1heat shock protein 21 | | |
| TRINITY_DN26271_c0_g1_i1_len_304_109525.p1_type_internal_len_102 | ---NA--- | | |
| TRINITY_DN19666_c0_g1_i1_len_1240_297642.p3_type_5prime_partial_len_101 | ---NA--- | | |
| TRINITY_DN31860_c0_g1_i1_len_306_213822.p1_type_internal_len_102 | ---NA--- | | |
| TRINITY_DN21181_c0_g2_i1_len_517_203737.p2_type_5prime_partial_len_101 | ---NA--- | | |
| TRINITY_DN316_c0_g1_i1_len_678_5974.p1_type_5prime_partial_len_101 | ---NA--- | | |
| TRINITY_DN13401_c0_g1_i1_len_303_77796.p1_type_internal_len_102 | XP_023718620.1post-GPI attachment to proteins factor 6-like | C:GO:0016020 | |

| | | | |
|---|---|---|---|
| TRINITY_DN34839_c0_g1_i1_len_306_213824.p1_type_internal_len_102 | ---NA--- | | |
| TRINITY_DN34111_c0_g1_i1_len_306_213779.p1_type_internal_len_102 | ---NA--- | | |
| TRINITY_DN18003_c0_g1_i1_len_841_34480.p2_type_5prime_partial_len_101 | ---NA--- | | |
| TRINITY_DN2758_c0_g1_i1_len_304_214017.p1_type_internal_len_102 | XP_013079692.1 hemicentin-1-like | F:GO:0005509 | |
| TRINITY_DN32106_c0_g1_i1_len_443_133835.p1_type_5prime_partial_len_101 | ---NA--- | | |
| TRINITY_DN36153_c0_g1_i1_len_479_204920.p1_type_3prime_partial_len_102 | ---NA--- | | |
| TRINITY_DN30818_c0_g1_i1_len_303_140740.p1_type_internal_len_102 | ---NA--- | | |
| TRINITY_DN19741_c0_g1_i1_len_2339_91643.p3_type_5prime_partial_len_101 | ---NA--- | | |
| TRINITY_DN8903_c0_g1_i1_len_306_213850.p1_type_internal_len_102 | ---NA--- | | |
| TRINITY_DN11886_c0_g1_i1_len_304_214014.p2_type_internal_len_102 | ---NA--- | | |
| TRINITY_DN19287_c0_g1_i1_len_496_101395.p1_type_complete_len_101 | ---NA--- | | |
| TRINITY_DN29529_c0_g1_i3_len_563_202595.p1_type_5prime_partial_len_101 | ---NA--- | | |
| TRINITY_DN9314_c0_g1_i1_len_774_128255.p1_type_complete_len_101 | ---NA--- | | |
| TRINITY_DN5748_c0_g1_i1_len_304_213996.p1_type_internal_len_102 | ACC62400.2ribonuclease III | F:GO:0003723; F:GO:0004525; P:GO:0006396; P:GO:0016075; P:GO:0031047; P:GO:0090502 | EC:3.1.30; EC:3.1.26; EC:3.1.26.3 |
| TRINITY_DN32400_c0_g1_i1_len_306_213777.p1_type_internal_len_102 | EFA02368.1Nidogen-2-like Protein | F:GO:0005509; P:GO:0007160; C:GO:0016020 | |
| TRINITY_DN23356_c0_g1_i1_len_340_281591.p1_type_5prime_partial_len_101 | ---NA--- | | |

| | | | |
|---|---|---|---|
| TRINITY_DN6006_c0_g1_i1_len_305_213912.p1_type_internal_len_102 | ---NA--- | | |
| TRINITY_DN17133_c0_g1_i1_len_821_198355.p1_type_5prime_partial_len_101 | ---NA--- | | |
| TRINITY_DN9389_c0_g1_i1_len_304_140627.p1_type_internal_len_102 | ---NA--- | | |
| TRINITY_DN5865_c0_g1_i1_len_303_214036.p1_type_internal_len_102 | ---NA--- | | |
| TRINITY_DN29997_c0_g1_i1_len_304_140633.p1_type_internal_len_102 | ---NA--- | | |
| TRINITY_DN2512_c0_g1_i1_len_306_213858.p1_type_internal_len_102 | ---NA--- | | |
| TRINITY_DN791_c0_g1_i1_len_303_140694.p1_type_internal_len_102 | ---NA--- | | |
| TRINITY_DN19750_c0_g1_i1_len_580_37265.p1_type_5prime_partial_len_101 | ---NA--- | | |
| TRINITY_DN14960_c0_g1_i1_len_2879_294934.p3_type_complete_len_101 | ---NA--- | | |
| TRINITY_DN11570_c0_g1_i1_len_304_140684.p1_type_internal_len_102 | BAH03696.1immunoglobulin light chain | | |
| TRINITY_DN40481_c0_g1_i1_len_303_214033.p1_type_internal_len_102 | ---NA--- | | |
| TRINITY_DN32905_c0_g1_i1_len_306_213838.p1_type_3prime_partial_len_102 | ---NA--- | | |
| TRINITY_DN11113_c0_g1_i1_len_306_283604.p1_type_3prime_partial_len_102 | ---NA--- | | |
| TRINITY_DN22588_c0_g1_i1_len_306_283586.p1_type_internal_len_102 | ---NA--- | | |
| TRINITY_DN29877_c0_g1_i1_len_362_280530.p1_type_complete_len_101 | ---NA--- | | |
| TRINITY_DN35827_c0_g1_i1_len_304_77747.p1_type_internal_len_102 | ---NA--- | | |

| | | | |
|---|---|---|---|
| TRINITY_DN11460_c0_g1_i1_len_306_109360.p1_type_internal_len_102 | XP_026281139.1smoothened homolog | | |
| TRINITY_DN28793_c0_g1_i1_len_305_283638.p1_type_internal_len_102 | ---NA--- | | |
| TRINITY_DN30957_c0_g1_i1_len_305_283641.p1_type_internal_len_102 | ---NA--- | | |
| TRINITY_DN30990_c0_g2_i1_len_306_213841.p1_type_internal_len_102 | XP_019879397.1 multidrug resistance-associated protein 1-like | F:GO:0005524; C:GO:0016021; F:GO:0042626; P:GO:0055085 | EC:3.6.1.3; EC:3.6.1.15 |
| TRINITY_DN26700_c0_g1_i1_len_306_283562.p1_type_internal_len_102 | ---NA--- | | |
| TRINITY_DN34204_c0_g1_i1_len_306_213843.p2_type_internal_len_102 | PSN53731.1Cytoplasmic dynein 2 light intermediate chain 1 | P:GO:0042073; C:GO:0043226; C:GO:0044464 | |
| TRINITY_DN13031_c0_g1_i1_len_304_77739.p1_type_5prime_partial_len_101 | ---NA--- | | |
| TRINITY_DN17045_c0_g1_i1_len_423_278197.p1_type_5prime_partial_len_100 | ---NA--- | | |
| TRINITY_DN8881_c0_g1_i1_len_301_312079.p1_type_internal_len_101 | PNJ69128.1AP2M1 isoform 10 | P:GO:0006886; P:GO:0016192; C:GO:0030131 | |
| TRINITY_DN9119_c0_g1_i1_len_302_312038.p1_type_internal_len_101 | ---NA--- | | |
| TRINITY_DN32608_c0_g1_i1_len_423_103518.p3_type_5prime_partial_len_100 | ---NA--- | | |
| TRINITY_DN16210_c0_g1_i1_len_327_15181.p1_type_5prime_partial_len_100 | ---NA--- | | |
| TRINITY_DN20500_c0_g1_i1_len_3280_154075.p2_type_5prime_partial_len_100 | ---NA--- | | |
| TRINITY_DN31041_c0_g1_i3_len_5630_186988.p3_type_complete_len_100 | EEC16454.1bromodomain and PHD finger-containing protein 1, brpf1, putative | F:GO:0003676; F:GO:0004402; P:GO:0016573; F:GO:0018024; P:GO:0034968; F:GO:0046872 | EC:2.1.1.43; EC:2.3.1.5; EC:2.3.1.48 |
| TRINITY_DN21731_c0_g2_i1_len_459_168172.p1_type_5prime_partial_len_100 | ---NA--- | | |
| TRINITY_DN5354_c0_g1_i1_len_300_214325.p2_type_internal_len_101 | ---NA--- | | |

<table>
<tr><td>TRINITY_DN18707_c0_g1_i1_len_1534_192925.p4_type_complete_len_100</td><td>---NA---</td><td></td><td></td></tr>
<tr><td>TRINITY_DN12268_c0_g1_i1_len_372_136628.p1_type_5prime_partial_len_100</td><td>---NA---</td><td></td><td></td></tr>
<tr><td>TRINITY_DN7289_c0_g1_i1_len_375_136452.p1_type_5prime_partial_len_100</td><td>---NA---</td><td></td><td></td></tr>
<tr><td>TRINITY_DN9611_c0_g1_i1_len_422_278224.p1_type_5prime_partial_len_100</td><td>---NA---</td><td></td><td></td></tr>
<tr><td>TRINITY_DN29368_c0_g1_i1_len_301_312082.p1_type_internal_len_101</td><td>EEB13671.1proline-serine-threonine phosphatase interacting protein, putative</td><td></td><td></td></tr>
<tr><td>TRINITY_DN15077_c0_g1_i1_len_373_136539.p1_type_complete_len_100</td><td>---NA---</td><td></td><td></td></tr>
<tr><td>TRINITY_DN10374_c0_g1_i1_len_300_312205.p1_type_internal_len_101</td><td>---NA---</td><td></td><td></td></tr>
<tr><td>TRINITY_DN25698_c1_g1_i4_len_1013_236006.p1_type_complete_len_100</td><td>---NA---</td><td></td><td></td></tr>
<tr><td>TRINITY_DN8924_c0_g1_i1_len_418_103694.p2_type_5prime_partial_len_100</td><td>XP_018023308.1 sodium channel protein 60E-like</td><td></td><td></td></tr>
<tr><td>TRINITY_DN14064_c0_g1_i1_len_1835_156571.p2_type_complete_len_100</td><td>---NA---</td><td></td><td></td></tr>
<tr><td>TRINITY_DN30771_c0_g1_i1_len_2668_189092.p4_type_complete_len_100</td><td>---NA---</td><td></td><td></td></tr>
<tr><td>TRINITY_DN15835_c0_g1_i1_len_522_242462.p1_type_5prime_partial_len_100</td><td>---NA---</td><td></td><td></td></tr>
<tr><td>TRINITY_DN38743_c0_g1_i1_len_301_214248.p1_type_internal_len_101</td><td>AVN99049.1ionotropic glutamate receptor NMDA 1A</td><td>F:GO:0004970; C:GO:0016021; C:GO:0030054; P:GO:0034220; P:GO:0035235; C:GO:0045211</td><td></td></tr>
<tr><td>TRINITY_DN25617_c0_g1_i1_len_1831_231637.p2_type_complete_len_100</td><td>---NA---</td><td></td><td></td></tr>
<tr><td>3139|_|c1952_f4p2_2402_isoform_c1952_full_length_coverage_4_non_full_length_coverage_2_isoform_length_2402_20626</td><td>XP_018009396.1 disco-interacting protein 2 homolog C-like</td><td>F:GO:0003824</td><td></td></tr>
</table>

| | | | |
|---|---|---|---|
| 3140\|_\|c2785_f2p2_2402_isoform_c2785_full_length_coverage_2_non_full_length_coverage_2_isoform_length_2402_16698 | AEU11366.1Broad-complex protein isoform 4 | F:GO:0003676 | |
| 3142\|_\|c2405_f3p0_2402_isoform_c2405_full_length_coverage_3_non_full_length_coverage_0_isoform_length_2402_11842 | XP_021916003.1trafficking protein particle complex subunit 11 isoform X3 | | |
| 3143\|_\|c13608_f2p3_2402_isoform_c13608_full_length_coverage_2_non_full_length_coverage_3_isoform_length_2402_17609 | KDR09385.1RING finger protein 10 | F:GO:0046872 | |
| 3144\|_\|c28177_f2p0_2401_isoform_c28177_full_length_coverage_2_non_full_length_coverage_0_isoform_length_2401_14018 | XP_017792338.1 inner nuclear membrane protein Man1 | | |
| 3145\|_\|c2798_f5p7_2401_isoform_c2798_full_length_coverage_5_non_full_length_coverage_7_isoform_length_2401_12171 | ---NA--- | | |
| 3146\|_\|c3283_f2p2_2401_isoform_c3283_full_length_coverage_2_non_full_length_coverage_2_isoform_length_2401_16755 | XP_021919426.1replication factor C subunit 1 isoform X3 | F:GO:0003689; F:GO:0005524; C:GO:0005634; C:GO:0005663; P:GO:0006260; P:GO:0006281 | EC:3.6.1.3; EC:3.6.1.15 |
| 3147\|_\|c26370_f2p14_2401_isoform_c26370_full_length_coverage_2_non_full_length_coverage_14_isoform_length_2401_18391 | XP_018006803.1 longitudinals lacking protein, isoforms H/M/V-like isoform X39 | F:GO:0003676 | |
| 3148\|_\|c9274_f2p3_2401_isoform_c9274_full_length_coverage_2_non_full_length_coverage_3_isoform_length_2401_12789 | XP_015512144.1 dnaJ homolog subfamily C member 11 isoform X2 | | |
| 3149\|_\|c2384_f6p26_2400_isoform_c2384_full_length_coverage_6_non_full_length_coverage_26_isoform_length_2400_20897 | AFC36878.114-3-3 epsilon-like transcript variant 2 | F:GO:0019904 | |
| TRINITY_DN16716_c0_g1_i2_len_1309_32082.p2_type_complete_len_100 | ---NA--- | | |
| TRINITY_DN11094_c0_g1_i1_len_491_276255.p1_type_5prime_partial_len_100 | ---NA--- | | |

| | | | |
|---|---|---|---|
| TRINITY_DN22603_c0_g1_i2_len_2167_594.p5_type_complete_len_100 | XP_008507880.1 poly [ADP-ribose] polymerase 1-like | F:GO:0003677; F:GO:0003950; C:GO:0005634; P:GO:0006471; F:GO:0008270; F:GO:0051287 | EC:2.4.2.30 |
| TRINITY_DN13443_c0_g1_i1_len_1113_94935.p2_type_complete_len_100 | ---NA--- | | |
| TRINITY_DN13198_c0_g1_i2_len_342_249218.p1_type_3prime_partial_len_101 | ---NA--- | | |
| TRINITY_DN24643_c0_g1_i1_len_1867_231507.p1_type_complete_len_100 | ---NA--- | | |
| TRINITY_DN12922_c0_g1_i2_len_471_276781.p1_type_5prime_partial_len_100 | ---NA--- | | |
| TRINITY_DN16174_c0_g1_i2_len_457_102434.p1_type_5prime_partial_len_100 | ---NA--- | | |
| TRINITY_DN11519_c0_g1_i1_len_381_42177.p1_type_3prime_partial_len_101 | ---NA--- | | |
| TRINITY_DN23893_c0_g1_i2_len_1298_61492.p2_type_complete_len_100 | ---NA--- | | |
| TRINITY_DN22920_c0_g1_i1_len_1912_156372.p2_type_complete_len_100 | ---NA--- | | |
| TRINITY_DN4447_c0_g1_i1_len_328_76073.p1_type_5prime_partial_len_100 | ---NA--- | | |
| TRINITY_DN7580_c0_g1_i1_len_395_170421.p1_type_5prime_partial_len_100 | ---NA--- | | |
| TRINITY_DN11406_c0_g1_i2_len_395_170409.p1_type_complete_len_100 | ---NA--- | | |
| TRINITY_DN31397_c0_g1_i1_len_301_214226.p1_type_internal_len_101 | ---NA--- | | |
| TRINITY_DN358_c0_g1_i1_len_423_134569.p1_type_5prime_partial_len_100 | ---NA--- | | |
| TRINITY_DN350_c0_g1_i1_len_428_206812.p1_type_5prime_partial_len_100 | ---NA--- | | |
| TRINITY_DN36464_c0_g1_i1_len_764_238376.p1_type_5prime_partial_len_100 | PNF26992.1Rho GTPase-activating protein 1 | P:GO:0007165 | |

| | | | |
|---|---|---|---|
| TRINITY_DN24698_c0_g1_i1_len_441_206280.p1_type_5prime_partial_len_100 | ---NA--- | | |
| TRINITY_DN29737_c0_g1_i1_len_404_306894.p1_type_complete_len_100 | ---NA--- | | |
| TRINITY_DN24518_c0_g1_i1_len_300_16912.p1_type_internal_len_101 | ---NA--- | | |
| TRINITY_DN28125_c0_g1_i1_len_424_134507.p1_type_3prime_partial_len_101 | ---NA--- | | |
| TRINITY_DN6443_c0_g1_i1_len_495_276171.p2_type_complete_len_100 | ---NA--- | | |
| TRINITY_DN22012_c0_g1_i1_len_406_306826.p1_type_5prime_partial_len_100 | ---NA--- | | |
| TRINITY_DN34457_c0_g1_i1_len_398_170295.p1_type_5prime_partial_len_100 | ---NA--- | | |
| TRINITY_DN30063_c1_g1_i4_len_301_214230.p1_type_internal_len_101 | ---NA--- | | |
| TRINITY_DN25093_c0_g1_i1_len_1787_231797.p1_type_complete_len_100 | ---NA--- | | |
| TRINITY_DN25044_c0_g1_i2_len_1949_231265.p3_type_complete_len_100 | ---NA--- | | |
| TRINITY_DN21980_c0_g1_i1_len_1953_231257.p4_type_complete_len_100 | ---NA--- | | |
| TRINITY_DN3899_c0_g1_i1_len_477_204978.p1_type_complete_len_100 | ---NA--- | | |
| TRINITY_DN24591_c0_g1_i1_len_7335_227500.p3_type_complete_len_100 | ---NA--- | | |
| TRINITY_DN12004_c0_g1_i1_len_857_64087.p2_type_5prime_partial_len_100 | ---NA--- | | |
| TRINITY_DN35112_c0_g1_i1_len_308_213686.p1_type_3prime_partial_len_101 | ---NA--- | | |
| TRINITY_DN7812_c0_g1_i1_len_429_206748.p1_type_5prime_partial_len_100 | ---NA--- | | |

| | | | |
|---|---|---|---|
| TRINITY_DN2821_c0_g1_i1_len_300_214288.p1_type_internal_len_101 | ADD63783.1cytochrome P450 | F:GO:0004497; F:GO:0005506; C:GO:0016021; F:GO:0016705; F:GO:0020037; P:GO:0055114 | |
| TRINITY_DN19157_c0_g1_i1_len_3035_228953.p4_type_complete_len_100 | ---NA--- | | |
| TRINITY_DN39457_c0_g1_i1_len_327_212212.p1_type_5prime_partial_len_100 | XP_018006424.1 sorting nexin-13-like | | |
| TRINITY_DN40039_c0_g1_i1_len_310_213446.p1_type_5prime_partial_len_100 | ---NA--- | | |
| TRINITY_DN18978_c0_g1_i1_len_755_35166.p1_type_5prime_partial_len_100 | ---NA--- | | |
| TRINITY_DN25167_c0_g1_i1_len_680_200315.p1_type_complete_len_100 | ---NA--- | | |
| TRINITY_DN6430_c0_g1_i1_len_340_75339.p1_type_5prime_partial_len_100 | ---NA--- | | |
| TRINITY_DN8338_c0_g1_i1_len_300_214309.p1_type_internal_len_101 | AHH32890.1integrin beta 1 | P:GO:0007155; P:GO:0007229; C:GO:0016021 | |
| TRINITY_DN39606_c0_g1_i1_len_301_214267.p1_type_internal_len_101 | XP_018011644.1 irregular chiasm C-roughest protein-like | | |
| TRINITY_DN31750_c0_g1_i1_len_301_214270.p1_type_5prime_partial_len_100 | ---NA--- | | |
| TRINITY_DN15701_c0_g1_i1_len_429_206779.p1_type_complete_len_100 | ---NA--- | | |
| TRINITY_DN1045_c0_g1_i1_len_417_169570.p1_type_5prime_partial_len_100 | ---NA--- | | |
| TRINITY_DN16229_c0_g1_i1_len_569_202432.p1_type_complete_len_100 | ---NA--- | | |
| TRINITY_DN29921_c0_g1_i1_len_443_102874.p2_type_3prime_partial_len_101 | ---NA--- | | |
| TRINITY_DN18502_c0_g1_i1_len_1927_231334.p2_type_complete_len_100 | ---NA--- | | |
| TRINITY_DN23551_c0_g1_i1_len_1032_235852.p2_type_complete_len_100 | ---NA--- | | |

| | | | |
|---|---|---|---|
| TRINITY_DN10158_c0_g1_i1_len_429_206754.p1_type_complete_len_100 | ---NA--- | | |
| TRINITY_DN12350_c0_g1_i1_len_301_214276.p1_type_internal_len_101 | ---NA--- | | |
| TRINITY_DN30531_c0_g1_i1_len_516_242630.p3_type_complete_len_100 | XP_018023256.1 motile sperm domain-containing protein 2-like | | |
| TRINITY_DN27067_c0_g1_i1_len_424_169288.p1_type_3prime_partial_len_101 | ---NA--- | | |
| TRINITY_DN16865_c0_g1_i3_len_400_278963.p1_type_5prime_partial_len_100 | ---NA--- | | |
| TRINITY_DN26733_c0_g1_i1_len_300_252118.p1_type_internal_len_101 | ---NA--- | | |
| TRINITY_DN31648_c0_g1_i1_len_303_140725.p1_type_internal_len_101 | ---NA--- | | |
| TRINITY_DN31605_c0_g1_i1_len_302_140773.p2_type_internal_len_101 | PFX23784.1Transmembrane and TPR repeat-containing protein 4 | C:GO:0016020 | |
| TRINITY_DN30365_c0_g1_i1_len_302_140770.p1_type_internal_len_101 | ---NA--- | | |
| TRINITY_DN10224_c0_g1_i1_len_302_140767.p3_type_internal_len_101 | ---NA--- | | |
| TRINITY_DN28433_c0_g1_i1_len_3638_187820.p2_type_5prime_partial_len_100 | ---NA--- | | |
| TRINITY_DN24212_c0_g2_i2_len_422_71484.p1_type_5prime_partial_len_100 | ---NA--- | | |
| TRINITY_DN4761_c0_g1_i1_len_302_140818.p1_type_internal_len_101 | ---NA--- | | |
| TRINITY_DN24223_c0_g1_i1_len_307_109317.p1_type_5prime_partial_len_100 | ---NA--- | | |
| TRINITY_DN15658_c0_g1_i2_len_302_140815.p2_type_internal_len_101 | ---NA--- | | |
| TRINITY_DN16220_c0_g1_i2_len_325_310542.p1_type_5prime_partial_len_100 | ---NA--- | | |

| | | | |
|---|---|---|---|
| TRINITY_DN23909_c0_g1_i1_len_301_251983.p1_type_internal_len_101 | ---NA--- | | |
| TRINITY_DN28401_c0_g1_i1_len_302_251942.p1_type_internal_len_101 | XP_021920441.1tigger transposable element-derived protein 1-like | | |
| TRINITY_DN36587_c0_g1_i1_len_302_251944.p1_type_internal_len_101 | ---NA--- | | |
| TRINITY_DN11584_c0_g1_i1_len_470_205215.p1_type_3prime_partial_len_101 | ---NA--- | | |
| TRINITY_DN26408_c3_g3_i1_len_302_251953.p1_type_5prime_partial_len_100 | ---NA--- | | |
| TRINITY_DN3359_c0_g1_i1_len_302_251956.p1_type_internal_len_101 | ---NA--- | | |
| TRINITY_DN18819_c0_g1_i1_len_302_251957.p1_type_internal_len_101 | ---NA--- | | |
| TRINITY_DN36008_c0_g1_i1_len_301_251961.p1_type_internal_len_101 | ---NA--- | | |
| TRINITY_DN6872_c0_g1_i1_len_301_252018.p4_type_internal_len_101 | ---NA--- | | |
| TRINITY_DN37875_c0_g1_i1_len_301_252022.p1_type_internal_len_101 | ---NA--- | | |
| TRINITY_DN34410_c0_g1_i1_len_301_252027.p1_type_internal_len_101 | ---NA--- | | |
| TRINITY_DN32180_c0_g1_i1_len_300_252047.p1_type_internal_len_101 | XP_026277761.1protein KIAA0100 | | |
| TRINITY_DN15975_c0_g1_i2_len_302_140778.p1_type_internal_len_101 | ---NA--- | | |
| TRINITY_DN10857_c0_g1_i1_len_300_252052.p1_type_internal_len_101 | XP_026819426.1zinc finger MYM-type protein 1-like | | |
| TRINITY_DN30423_c0_g1_i1_len_301_251989.p1_type_internal_len_101 | ---NA--- | | |
| TRINITY_DN31123_c0_g1_i1_len_301_252008.p1_type_internal_len_101 | XP_018025197.1 polypeptide N-acetylgalactosaminyltransferase 2-like | F:GO:0016740 | |

| | | | |
|---|---|---|---|
| TRINITY_DN8317_c0_g1_i1_len_302_140793.p1_type_internal_len_101 | ---NA--- | | |
| TRINITY_DN17773_c0_g1_i1_len_449_70586.p1_type_5prime_partial_len_100 | EEC15736.1growth hormone-inducible soluble protein, putative | | |
| TRINITY_DN21060_c0_g1_i1_len_910_197383.p1_type_complete_len_100 | ---NA--- | | |
| TRINITY_DN23004_c0_g1_i1_len_1487_193169.p4_type_complete_len_100 | ---NA--- | | |
| TRINITY_DN16707_c0_g1_i1_len_1151_269328.p2_type_complete_len_100 | ---NA--- | | |
| TRINITY_DN23800_c0_g1_i1_len_601_201747.p1_type_5prime_partial_len_100 | ---NA--- | | |
| TRINITY_DN26053_c0_g1_i1_len_324_108184.p1_type_3prime_partial_len_101 | XP_018009335.1 hemicentin-1-like | | |
| TRINITY_DN22018_c0_g1_i2_len_609_201585.p1_type_3prime_partial_len_101 | ---NA--- | | |
| TRINITY_DN19038_c0_g1_i1_len_543_166097.p1_type_5prime_partial_len_100 | ---NA--- | | |
| TRINITY_DN10356_c0_g1_i1_len_726_128705.p1_type_5prime_partial_len_100 | ---NA--- | | |
| TRINITY_DN23286_c0_g1_i4_len_3970_153699.p2_type_complete_len_100 | ---NA--- | | |
| TRINITY_DN33206_c0_g1_i1_len_616_164718.p1_type_complete_len_100 | ---NA--- | | |
| TRINITY_DN11539_c0_g1_i1_len_459_305183.p1_type_complete_len_100 | XP_010565822.1 cystatin-B isoform X1 | F:GO:0004866; F:GO:0004869; P:GO:0010951 | |
| TRINITY_DN25990_c0_g1_i2_len_607_201636.p2_type_5prime_partial_len_100 | ---NA--- | | |
| TRINITY_DN11557_c0_g1_i1_len_389_279361.p1_type_5prime_partial_len_100 | ACF93414.1single IB domain protein | F:GO:0005520; C:GO:0005576 | |
| TRINITY_DN19149_c0_g1_i1_len_775_64824.p3_type_5prime_partial_len_100 | ---NA--- | | |

| | | | |
|---|---|---|---|
| TRINITY_DN33089_c0_g1_i1_len_303_109596.p3_type_internal_len_101 | ---NA--- | | |
| TRINITY_DN32003_c0_g1_i1_len_300_140958.p1_type_internal_len_101 | ---NA--- | | |
| 3150\|_\|c18152_f3p0_2400_isoform_c18152_full_length_coverage_3_non_full_length_coverage_0_isoform_length_2400_17987 | XP_018020172.1PREDICTED: uncharacterized protein LOC108676584 | | |
| TRINITY_DN23266_c0_g1_i3_len_2416_189652.p1_type_5prime_partial_len_800 | PNF14351.1hypothetical protein B7P43_G05566 | | |
| 3152\|_\|c1729_f2p4_2400_isoform_c1729_full_length_coverage_2_non_full_length_coverage_4_isoform_length_2400_26053 | ---NA--- | | |
| 3153\|_\|c11851_f3p3_2400_isoform_c11851_full_length_coverage_3_non_full_length_coverage_3_isoform_length_2400_13019 | XP_018022968.1PREDICTED: uncharacterized protein LOC108678981 isoform X2 | | |
| 3155\|_\|c9106_f2p6_2400_isoform_c9106_full_length_coverage_2_non_full_length_coverage_6_isoform_length_2400_17259 | XP_018006612.1 protein bric-a-brac 1-like isoform X4 | | |
| TRINITY_DN25318_c0_g1_i1_len_2445_189582.p1_type_5prime_partial_len_800 | ABP97431.1alpha 2 macroglobulin | F:GO:0004866; C:GO:0005615; P:GO:0010951 | |
| 3157\|_\|c47217_f44p10_2398_isoform_c47217_full_length_coverage_44_non_full_length_coverage_10_isoform_length_2398_19477 | ---NA--- | | |
| 3158\|_\|c17980_f2p0_2398_isoform_c17980_full_length_coverage_2_non_full_length_coverage_0_isoform_length_2398_22606 | ---NA--- | | |
| 3159\|_\|c3989_f3p41_2398_isoform_c3989_full_length_coverage_3_non_full_length_coverage_41_isoform_length_2398_12340 | AVN99057.1isocitrate dehydrogenase | F:GO:0000287; F:GO:0004450; P:GO:0006099; P:GO:0006102; F:GO:0051287 | EC:1.1.1.42 |
| 3160\|_\|c2406_f2p0_2397_isoform_c2406_full_length_coverage_2_non_full_length_coverage_0_isoform_length_2397_20915 | ---NA--- | | |

| | | | |
|---|---|---|---|
| TRINITY_DN30172_c0_g1_i1_len_348_210896.p1_type_5prime_partial_len_100 | ---NA--- | | |
| TRINITY_DN31796_c0_g1_i1_len_300_109784.p1_type_internal_len_101 | ---NA--- | | |
| TRINITY_DN7542_c0_g1_i1_len_785_198800.p3_type_complete_len_100 | ---NA--- | | |
| TRINITY_DN27110_c0_g1_i1_len_304_251757.p1_type_internal_len_101 | XP_015514196.1 alpha-1,6-mannosyl-glycoprotein 2-beta-N-acetylglucosaminyltransferase isoform X1 | | |
| TRINITY_DN32357_c0_g1_i1_len_304_251762.p1_type_internal_len_101 | ---NA--- | | |
| TRINITY_DN2748_c0_g1_i1_len_303_109588.p1_type_internal_len_101 | ---NA--- | | |
| TRINITY_DN36943_c0_g1_i1_len_304_251737.p1_type_internal_len_101 | ---NA--- | | |
| TRINITY_DN15096_c0_g1_i1_len_357_74400.p1_type_5prime_partial_len_100 | ---NA--- | | |
| TRINITY_DN31601_c0_g1_i1_len_303_109578.p1_type_internal_len_101 | ---NA--- | | |
| TRINITY_DN28386_c0_g1_i1_len_304_251744.p1_type_internal_len_101 | ---NA--- | | |
| TRINITY_DN4875_c0_g1_i1_len_304_251749.p1_type_internal_len_101 | ---NA--- | | |
| TRINITY_DN30684_c0_g1_i1_len_300_109824.p1_type_internal_len_101 | ---NA--- | | |
| TRINITY_DN23265_c0_g1_i1_len_302_109662.p2_type_internal_len_101 | ---NA--- | | |
| TRINITY_DN27412_c0_g1_i1_len_301_109734.p1_type_internal_len_101 | ---NA--- | | |
| TRINITY_DN31205_c0_g1_i1_len_301_109735.p1_type_internal_len_101 | XP_023690726.1tubulin alpha-3 chain-like | | |
| TRINITY_DN30139_c0_g1_i1_len_302_109660.p2_type_internal_len_101 | ---NA--- | | |

| | | | |
|---|---|---|---|
| TRINITY_DN11024_c0_g1_i2_len_301_109706.p1_type_internal_len_101 | XP_023345384.1glucuronokinase 1-like | | |
| TRINITY_DN30004_c0_g1_i1_len_301_109726.p1_type_internal_len_101 | ---NA--- | | |
| TRINITY_DN11892_c0_g1_i1_len_302_109692.p1_type_internal_len_101 | ---NA--- | | |
| TRINITY_DN32069_c0_g1_i1_len_302_109691.p1_type_internal_len_101 | AKS25864.1Fem-1A | | |
| TRINITY_DN19397_c0_g1_i1_len_4474_153538.p4_type_5prime_partial_len_100 | ---NA--- | | |
| TRINITY_DN24979_c0_g1_i1_len_302_109633.p2_type_internal_len_101 | ---NA--- | | |
| TRINITY_DN29799_c0_g1_i1_len_301_109754.p1_type_internal_len_101 | ---NA--- | | |
| TRINITY_DN24505_c0_g1_i1_len_301_109765.p1_type_5prime_partial_len_100 | ---NA--- | | |
| TRINITY_DN35750_c0_g1_i1_len_302_251883.p1_type_internal_len_101 | ---NA--- | | |
| TRINITY_DN33340_c0_g1_i1_len_302_251888.p1_type_internal_len_101 | AKS04548.1cysteine sulfinic acid decarboxylase | F:GO:0016831; P:GO:0019752; F:GO:0030170 | |
| TRINITY_DN11738_c1_g1_i1_len_302_251892.p1_type_internal_len_101 | ---NA--- | | |
| TRINITY_DN10746_c0_g1_i1_len_303_251869.p1_type_internal_len_101 | ---NA--- | | |
| TRINITY_DN6330_c0_g1_i1_len_302_251921.p1_type_internal_len_101 | ---NA--- | | |
| TRINITY_DN1619_c0_g1_i1_len_302_251924.p1_type_internal_len_101 | ---NA--- | | |
| TRINITY_DN30830_c0_g1_i1_len_302_251927.p1_type_internal_len_101 | ---NA--- | | |
| TRINITY_DN36578_c0_g1_i1_len_302_251929.p1_type_internal_len_101 | ---NA--- | | |

| | | | |
|---|---|---|---|
| TRINITY_DN204_c0_g1_i1_len_302_251930.p1_type_internal_len_101 | ---NA--- | | |
| TRINITY_DN12409_c0_g1_i1_len_302_251939.p1_type_internal_len_101 | ---NA--- | | |
| TRINITY_DN38054_c0_g1_i1_len_302_251894.p1_type_internal_len_101 | ---NA--- | | |
| TRINITY_DN34796_c0_g1_i1_len_302_251895.p1_type_internal_len_101 | XP_023716704.1citron Rho-interacting kinase | | |
| TRINITY_DN38066_c0_g1_i1_len_302_251897.p1_type_internal_len_101 | ---NA--- | | |
| TRINITY_DN30207_c0_g1_i1_len_302_251901.p1_type_internal_len_101 | ---NA--- | | |
| TRINITY_DN6026_c0_g1_i1_len_302_251911.p1_type_internal_len_101 | ---NA--- | | |
| TRINITY_DN25708_c3_g1_i2_len_302_251914.p1_type_internal_len_101 | ---NA--- | | |
[truncated: 401,969 more chars]
